# Transcriptome landscape of perennial wild *Cicer microphyllum* uncovers functionally relevant molecular tags regulating agronomic traits in chickpea

**DOI:** 10.1038/srep33616

**Published:** 2016-09-29

**Authors:** Rishi Srivastava, Deepak Bajaj, Ayushi Malik, Mohar Singh, Swarup K. Parida

**Affiliations:** 1 National Institute of Plant Genome Research (NIPGR), Aruna Asaf Ali Marg, New Delhi 110067, India; 2 Faculty of Science, Jamia Hamdard University, Hamdard Nagar, New Delhi 110062, India; 3 National Bureau of Plant Genetic Resources Regional Station, Shimla, Himachal Pradesh 171004, India

## Abstract

The RNA-sequencing followed by *de-novo* transcriptome assembly identified 11621 genes differentially xpressed in roots vs. shoots of a wild perennial *Cicer microphyllum*. Comparative analysis of transcriptomes between *microphyllum* and cultivated *desi* cv. ICC4958 detected 12772 including 3242 root- and 1639 shoot-specific *microphyllum* genes with 85% expression validation success rate. Transcriptional reprogramming of *microphyllum* root-specific genes implicates their possible role in regulating differential natural adaptive characteristics between wild and cultivated chickpea. The transcript-derived 5698 including 282 *in-silico* polymorphic SSR and 127038 SNP markers annotated at a genome-wide scale exhibited high amplification and polymorphic potential among cultivated (*desi* and *kabuli*) and wild accessions suggesting their utility in chickpea genomics-assisted breeding applications. The functional significance of markers was assessed based on their localization in non-synonymous coding and regulatory regions of *microphyllum* root-specific genes differentially expressed predominantly in ICC 4958 roots under drought stress. A high-density 490 genic SSR- and SNP markers-anchored genetic linkage map identified six major QTLs regulating drought tolerance-related traits, yield per plant and harvest-index in chickpea. The integration of high-resolution QTL mapping with comparative transcriptome profiling delineated five *microphyllum* root-specific genes with non-synonymous and regulatory SNPs governing drought-responsive yield traits. Multiple potential key regulators and functionally relevant molecular tags delineated can drive translational research and drought tolerance-mediated chickpea genetic enhancement.

Chickpea belongs to the genus *Cicer* that includes 44 annual and perennial species, grouped into primary, secondary and tertiary gene pools based on their crossability with cultivated chickpea^1^,^2^. These cultivated/wild annual and perennial wild *Cicer* species reportedly originated from the South Eastern Turkey and Syria (Fertile Crescent)^3^,^4^,^5^,^6^,^7^. The wild accessions representing diverse *Cicer* gene pools are rich reservoir of potential genes and alleles for improving abiotic/biotic stress tolerance and yield component traits in cultivated chickpea (*C. arietinum*)^1^,^2^,^8^,^9^,^10^,^11^. The development of high-yielding durable stress tolerant (climate resilient) chickpea cultivars thus requires identification and exploitation of genetically diverse wild relatives with beneficial traits to broaden the genetic base of modern cultivars and enhance yield-component and stress tolerance traits in the cultivated gene pool. This process of genetic enhancement can be complemented and accelerated through marker-based introgression of trait-associated novel genes, QTLs (quantitative trait loci) and natural allelic variants scanned from diverse wild gene pools into cultivated accessions. Significant efforts concerning inter-specific hybridization and marker-aided introduction of desirable traits specifically from annual wild species (*C. reticulatum*) of primary gene pool (contrasting characteristics of high crossability with cultivated gene pool) have been made for enhancing the seed and pod yield and stress tolerance in cultivated *desi* and *kabuli* chickpea^1^,^2^,^8^,^9^,^10^,^11^,^12^,^13^,^14^,^15^,^16^,^17^.

*C. microphyllum*, a member of tertiary gene pool is presumed to have originated from India and Turkey. The recent marker-based genetic diversity and population genetic structure studies documented a higher admixture and close phylogenetic relationship of one contrasting *C. microphyllum* accession domesticated primarily in the natural habitat alongside glaciers of the Hindu Kush Himalayas with cultivated *desi* chickpea of Indian origin^2^,^11^. The agro-ecological distribution of this *C. microphyllum* accession characterized by a peculiar feature of deep tap root system in the diverse rocky soil topography of Himalayan region, infers its strong adaptation towards drought and cold climatic conditions. Furthermore, this perennial wild accession exhibiting a vital characteristic of dense pod dehiscence is believed to serve as a potential source of pod borer resistance in chickpea^18^. The existing diversity among the wild *C. microphyllum* accessions for pod borer resistance can be thus exploited to broaden the genetic basis of resistance with an objective of driving marker-assisted disease resistance breeding and crop improvement in cultivated chickpea^18^. Collectively, a wild relative *C. microphyllum* accession revealing close evolutionary relationship with cultivated *desi* accessions is a potential natural source of novel genes and alleles especially related to drought, salinity and cold tolerance as well as pod borer resistance in chickpea.

The draft whole genome and transcriptome sequences available for diverse growth/development and stress-imposed tissues/stages of multiple cultivated (*desi* and *kabuli*) and wild (including *C. reticulatum*) chickpea accessions provide opportunity to characterize their global transcriptomes rapidly with use of limited resources^19^,^20^,^21^,^22^,^23^,^24^,^25^,^26^,^27^,^28^,^29^,^30^,^31^,^32^,^33^,^34^,^35^. The implications of transcriptome sequencing/RNA-seq (RNA-sequencing) in precise quantitation of differential expression profiles of gene-encoding transcripts and for discovering novel transcripts/isoforms is well-documented in chickpea^21^,^22^,^27^,^36^,^37^,^38^. Consequently, the assay is expedient to mine genic SSR (simple sequence repeat) and SNP (single nucleotide polymorphism) markers from expressed sequence components of the genome including differentially expressed genes/transcripts of chickpea^21^,^22^,^27^,^36^,^37^,^38^. The differentially expressed genes including tissue and stage-specific novel transcripts/genes generated at a genome-wide scale by transcriptome profiling are found proficient enough to scan key regulators (transcription factors) and metabolic pathways governing manifold stress and developmental responses in chickpea accessions with contrasting agronomic traits^21^,^22^,^27^,^36^,^37^,^38^. In addition, the genic markers discovered by RNA-seq representing diverse coding and regulatory sequence components of genes/transcripts (especially differentially expressed genes) have functional significance in quickly establishing marker-trait linkages as well as identification and targeted mapping of potential genes/QTLs regulating vital agronomic traits including drought-responsive yield traits on diverse intra- and inter-specific mapping populations-derived high-density genetic linkage maps of chickpea^22^,^23^,^25^,^26^,^32^,^39^,^40^. These informative markers thus can be extensively deployed in various genomics-assisted breeding applications for genetic improvement of chickpea^19^,^20^,^21^,^22^,^23^,^24^,^25^,^26^,^27^,^28^,^29^,^30^,^31^,^32^,^33^,^34^. In this context, transcriptome profiling in a wild *C. microphyllum* accession contrasting for several a/biotic stress tolerance traits (including drought, salinity, cold and pod borer resistance) can be an attractive strategy to understand its transcriptomic constitution aside scanning of functionally relevant molecular tags (markers, QTLs and genes) and natural allelic variants at a genome-wide scale for expediting marker-assisted genetic enhancement in chickpea.

In view of aforesaid possibilities, the present study employed a RNA-seq strategy to optimize *de novo* high-quality transcriptome assembly and generate comprehensive transcript sequences of an Indian origin perennial wild *C. microphyllum* accession for the first time at a high-resolution scale. A comparative analysis of root and shoot transcriptomes between *C. microphyllum* and a widely studied drought tolerant cultivated *desi* chickpea accession ICC 4958^39^,^40^ was performed to identify molecular tags like candidate genes, transcription factors (TFs) and key regulators/metabolic pathways especially governing inherent root-mediated natural adaptation characteristics of *C. microphyllum* in adverse agro-climatic condition. The informative transcript-derived genic SSR and SNP markers developed at a genome-wide scale were further utilized for molecular mapping of major QTLs governing two drought-responsive important yield traits (yield per plant and harvest-index) on a constructed high-density inter-specific genetic linkage map (transcript map) of chickpea. The high-resolution QTL mapping was further integrated with comparative transcriptome profiling to delineate candidate genes underlying major drought yield QTLs in chickpea.

## Results and Discussion

### Transcriptome sequencing of *C. microphyllum*

To generate the global transcriptome of a wild perennial *C. microphyllum* accession, the transcriptome sequencing of root and shoot tissue samples-derived RNA-seq libraries was performed using an Illumina NGS (next-generation sequencing) platform. These libraries were constructed by using the high-quality RNA isolated from three independent biological replicates of root and shoot tissue samples, each comprising pooled tissues from three randomly selected seedlings of a *C. microphyllum* accession. The natural adaptation of *C. microphyllum* accession towards abiotic stress (drought, salinity and cold) tolerance in adverse agro-climatic conditions is primarily because of its inherent characteristic feature of deep tap root system required for survival of this perennial wild chickpea in the diverse rocky soil topography of Himalayan region^18^. Considering the above facts, the root and shoot tissues of *C. microphyllum* were utilized specifically for transcriptome profiling to obtain the maximum representation of genes/transcripts differentially expressed/regulated in this hardy wild perennial accession as compared to a cultivated *desi* (ICC 4958) chickpea during natural adaptive climatic conditions. The RNA sequencing of both root (mean 54.7 million reads) and shoot (64.1) tissues of *C. microphyllum* accession generated on an average of ~118.7 million paired-end sequence reads across all three replicates of studied samples (Table 1). Of these, on an average ~105.9 million high-quality filtered sequence reads (root: 48.6 and shoot: 57.3 mean million reads) with a mean Phred quality score ≥30 at an individual base position were obtained (Table 1). The sequencing data generated from three independent biological replicates of each root and shoot tissue sample was highly correlated (91%) based on average Pearson’s correlation coefficient estimated among samples. These observations assured high reproducibility among biological replicates despite generating variable number of high-quality transcript sequence reads by transcriptome sequencing of *C. microphyllum* roots and shoots. All the *C. microphyllum* transcriptome sequence resource generated in the present study was submitted to NCBI-SRA (sequence read archive) database under BioProject ID PRJNA31486 with accession IDs SRR3340324–3340327 for unrestricted use.

### Assembly and mapping of *C. microphyllum* transcriptome

With an effort to constitute a reference-guided assembly of the *C. microphyllum* transcriptome, the filtered high-quality transcript sequence reads were mapped primarily on the reference draft genome sequences of *desi* (ICC 4958) chickpea. On an average 55% (ranging 49–61% in shoots and roots) of transcript sequence reads derived from root and shoot samples of *C. microphyllum* were mapped uniquely across the chickpea genome. Subsequently, to increase the mapping efficiency of uniquely mapped sequence-reads and generate the optimal assembly of *C. microphyllum* transcriptome, the *de novo* assembly using all high-quality non-redundant sequence reads (by removing the duplicate reads) obtained from both root and shoot samples was performed. The summary statistics defining the best *de novo* transcriptome assembly outputs of *C. microphyllum* optimized in our study, are mentioned in the Table S1. The mapping of on an average about 94 million *de novo* assembled high-quality transcript sequences uniquely suggests the use of at least 89% of transcript sequence reads in *de novo* assembly of *C. microphyllum*. The optimized assembly resulted in generation of 154352 unique transcript sequences with the average transcript and N50 length of ~896 and ~1641 bp, respectively (Table S1). The mapping efficiency of *de novo* assembled high-quality (longer average transcript and N50 length) transcript sequence reads constituted by us on the *C. microphyllum* transcriptome is higher/comparable to that documented earlier in reference-, *de novo*- and hybrid (reference and *de novo*) assembly-based transcriptome studies in crop plants including chickpea^19^,^20^,^21^,^22^,^23^,^24^,^25^,^26^,^27^,^28^,^32^,^33^,^34^,^35^,^38^,^41^. Interestingly, about 85% of these improved *de novo* assembled transcripts of *C. microphyllum* were mapped to reference *desi* chickpea genome. The use of 154352 unique high-quality transcript sequences in Cufflink followed by Cuffmerge assembly generated 38149 putative gene loci. The comparison of these candidate gene loci with the genes annotated from *desi* chickpea genome based on BLAST homology searches (with 90% sequence identity and 70% query coverage) exhibited orthology of 11219 (29.4%) unique *C. microphyllum* genes with 8465 (27.8%) candidate protein-coding genes (total 30257) annotated from *desi* genome.

### Differential transcriptome responses of *C. microphyllum*

To determine the transcriptome responses based on differential gene expression profiling, the high-quality transcript sequence reads generated from each root and shoot tissue sample were mapped on our optimized *de novo* assembled *C. microphyllum* transcriptome. This led to the mapping of ~89% transcript sequence reads on the *C. microphyllum* transcriptome. The abundance of transcripts (referred as genes) from each sample was estimated in accordance with FPKM (fragments per kilobase of exon per million fragments mapped) followed by differential expression profiling through Cuffdiff. This analysis identified 11621 genes exhibiting significant (≥two-fold at P ≤ 0.05) differential up (7592, 65.3%)- and down (4029, 37%)-regulated expression specifically in roots as compared to shoots of *C. microphyllum*. Remarkably, 2514 (21.6%) including 1597 (63.5%) and 917 (36.5%) genes showed a pronounced higher (≥10-fold at P ≤ 0.05) differential up- and down-regulation, respectively in roots than that of *C. microphyllum* shoots. Higher accumulation of differentially regulated transcripts especially in the roots of *C. microphyllum* may underlie its deep tap root system-mediated inherent characteristics of natural adaptation towards drought, salinity and cold tolerance in adverse agro-climatic condition. Therefore, these differentially regulated molecular tags especially expressed in the roots of *C. microphyllum* have functional relevance to decipher the regulatory gene (TFs) networks and/or metabolic pathways controlling abiotic stress tolerance traits in wild as well as cultivated chickpea.

**Figure 1:**
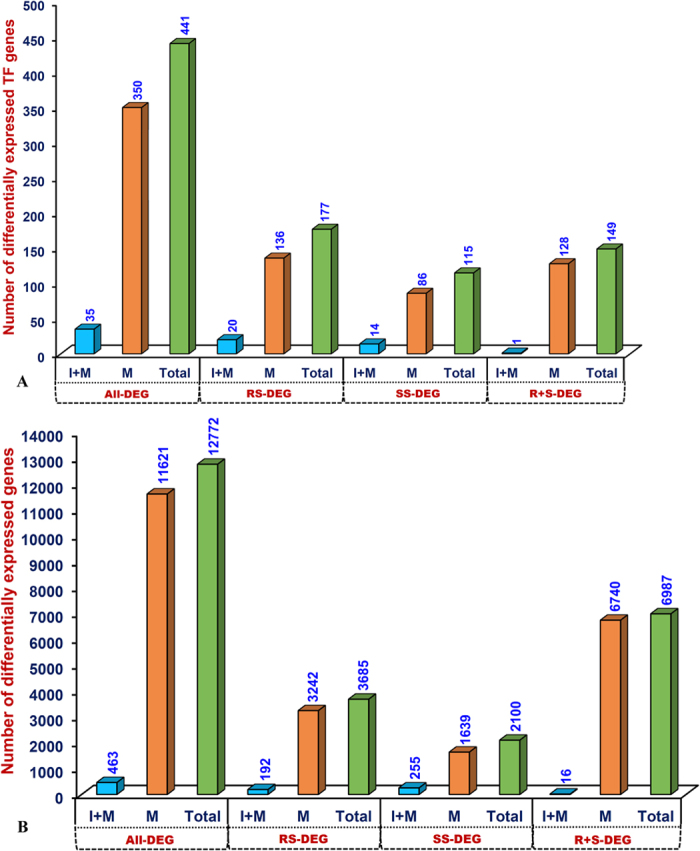
Characteristics of total genes (**A**) including TF-encoding genes (**B**) differentially expressed in roots and shoots of *C. microphyllum* and ICC 4958. DEG: differentially expressed genes, I: ICC 4958, M: *microphyllum*, (I + M): genes differentially expressed commonly between ICC 4958 and *C. microphyllum*. RS: root-specific, SS: shoot-specific, (R + S): genes differentially expressed commonly between roots and shoots of ICC 4958 and *C. microphyllum*. Total: genes differentially expressed in both ICC 4958 and *C. microphyllum*.

The comparative analysis of transcriptomes between *C. microphyllum* and ICC 4958 by correlating the differential expression profiles of genes in the roots and shoots of these accessions was performed. The detail statistics regarding mapping of transcript sequence reads obtained from control unstressed root and shoot tissues of ICC 4958 on the *C. microphyllum* transcriptome, are mentioned in the Table 1. This identified a total of 12772 genes differentially up-and down-regulated (≥two-fold at P ≤ 0.05) under at least one root and shoot tissue sample of *C. microphyllum* and ICC 4958 (Fig. 1A, Table S2). This included 463 genes differentially expressed commonly between *C. microphyllum* and ICC 4958. Higher number of root-specific genes (3685) as compared to shoot-specific genes (2100) were differentially expressed in *C. microphyllum* and ICC 4958. A total of 3242 root- and 1639 shoot-specific genes exhibiting pronounced higher differential expression specifically in *C. microphyllum* were identified (Fig. 1A, Table S2). Notably, 6987 genes were differentially expressed commonly between roots and shoots of both *C. microphyllum* and ICC 4958. This included 16 genes commonly expressed between *C. microphyllum* and ICC 4958, whereas 6740 genes specific to *C. microphyllum* (Fig. 1A, Table S2). Interestingly, we observed about 7.3 times higher differential up-and down-regulated expression of genes in the roots of *C. microphyllum* (3242 genes, 88%) as compared to that of ICC 4958 (443 genes, 12%). Therefore, these abundant root-specific differentially expressed genes especially scanned from the *C. microphyllum* could be vital to decipher the possible key regulatory pathway/mechanism governing superior root-responsive natural adaptive characteristic of this hardy wild chickpea accession in adverse climatic condition (diverse rocky soil topography of Himalayan region) as compared to a known cultivated drought tolerant *desi* accession ICC 4958.

### Expression dynamics of genes and transcription factors

**Figure 2:**
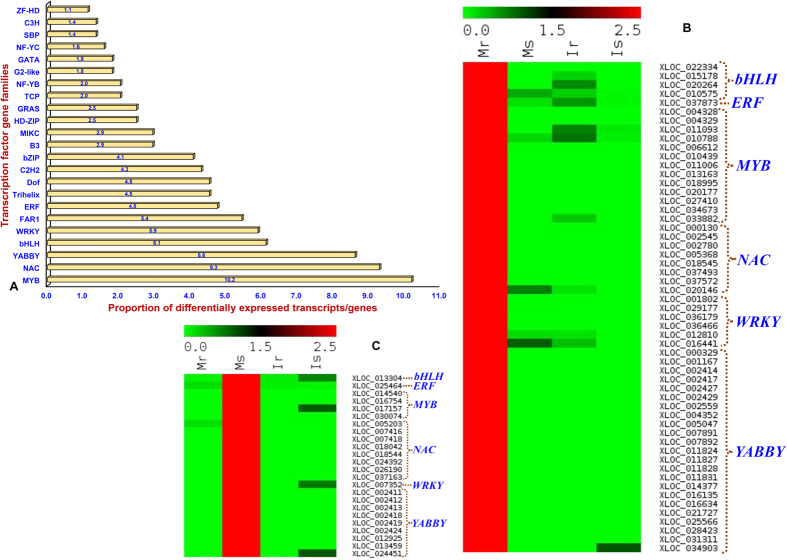
(**A**) Frequency (%) distribution of diverse TF-encoding genes differentially expressed in roots and shoots of *C. microphyllum* and ICC 4958. Heat-maps exhibiting the differential expression profiles of root (**B**)- and shoot (**C**)-specific three most abundant class of TF-encoding genes in *C. microphyllum*. These abiotic stress-responsive differentially expressed genes were scanned from the comparative transcriptome profiles of roots and shoots between *C. microphyllum* and ICC 4958. The average log signal expression value of genes is represented at the top with a colour scale; in which green, black and red color denote low, medium and high level of expression, respectively. The tissues/stages and genes selected for expression profiling are mentioned on the upper and right side of expression map, respectively. The structural and functional annotation of genes are mentioned in the Table S2. Mr: *Microphyllum* root, Ms: *Microphyllum* shoot, Ir: ICC 4958 root and Is: ICC 4958 shoot.

The KOG-based determination of putative function for 12772 differentially regulated genes identified from comparative transcriptome profiles of roots and shoots between *C. microphyllum* and ICC 4958 indicated primary roles of 3229 (25.3%) genes in multiple cellular, biological and molecular processes in crop plants. This revealed enrichment of differentially expressed genes (especially root- and shoot-specific genes) involved in translation, ribosomal structure and biogenesis (J, 709 genes, 22%), post-translational modification, protein turnover and chaperones (O, 411, 12.7%), and energy production and conversion (C, 275, 8.5%) (Figure S1). Among 441 (3.4%) differentially expressed TF-encoding genes (representing 39 TF gene family), the genes belonging to *MYB* (45 genes, 10.2%), *NAC* (41, 9.3%), *YABBY* (38, 8.6%), *bHLH* (27, 6.1%) and *WRKY* (26, 5.9%) TF families were predominately altered/regulated in roots and shoots of *C. microphyllum* and ICC 4958 (Fig. 2). Of these, 149 TFs were differentially expressed commonly between roots and shoots whereas 177 and 115 TFs differentially regulated specifically in roots and shoots, respectively of both *C. microphyllum* and ICC 4958. Notably, 350 (79.4%) of 441 TF-encoding genes were differentially regulated in both roots and shoots while 128 TFs commonly between roots and shoots, and 136 (30.8%) and 86 (19.5%) TFs differentially expressed particularly in roots and shoots of *C. microphyllum*, respectively (Fig. 1B, Table S2).

Despite higher root-specific gene expression, more than three times differential expression of TF-encoding genes specifically belonging to abundant class of TF gene families like *MYB, bHLH, NAC* and *WRKY* in *C. microphyllum* (136, 76.8%) than that of ICC 4958 (41, 23.2%) was observed. In contrast, we observed more than 2.5 times higher expression of root-specific genes especially belonging to genetic information processing (transcription, translation and post-translational modifications) in *C. microphyllum* than that of ICC 4958. In this context, remarkable differential regulation/alteration of diverse class of gene transcripts especially in *C. microphyllum* roots might impart enhanced adaptation to this hardy wild chickpea in adverse climatic condition (Himalayan region) than that of a known cultivated drought tolerant *desi* accession ICC 4958. The differential transcript accumulation and regulation of TF-encoding genes (especially *MYB, NAC* and *WRKY* TFs in this study) representing diverse TF families have implicated their common and/or specific role in multiple adaptive trait mechanism functioning in many crop plants (including chickpea) during acute climatic adversities^38^,^42^,^43^,^44^. Molecular characterization and functional validation of these TFs and dissection of their regulatory networks would further assist us to understand the global transcriptional reprogramming and thereby useful in defining the precise role of TF genes underlying adaptive trait mechanism in chickpea.

### Regulation of molecular pathways

To determine the possible gene regulatory pathways/interactions involved in natural adaptation responses, the pathway analysis of 12772 genes/TFs differentially expressed in roots and shoots of *C. microphyllum* and ICC 4958 was performed using KEGG database. Of these, 2212 (17.3%) genes were found to be significantly enriched with diverse pathways related to metabolism [primary (carbohydrate, lipid, nucleotide, amino acid, fatty acid, photosynthesis and abscisic acid) and secondary (flavonoids and terpenoids) metabolism 656, 29.7%], genetic information processing (transcription, translation and post-translational modifications, 595, 26.9%) and environmental information processing (signal transduction and membrane transport, 183, 8.3%) (Figure S4). Remarkably, more than two times higher altered expression of above-mentioned multiple pathway-related genes in the roots of *C. microphyllum* than that of ICC 4958 was observed suggesting potential involvement of these genes in imparting root-mediated natural adaptation to this wild chickpea. The pronounced differential regulation of genes involved in various primary and secondary metabolic pathways and post-translational modifications (phosphorylation and ubiquitination) are majorly known to elicit diverse cell signalling and transcriptional events, and thereby play crucial role in various plant adaptive abiotic stress responses^38^,^42^,^45^,^46^,^47^,^48^,^49^. Collectively, the differentially expressed root- and shoot-specific genes and TFs scanned from the comparative transcriptome profiles between known stress tolerant wild *C. microphyllum* and cultivated *desi* ICC 4958 accessions could serve as an immense transcriptomic resource for elucidating the key gene regulatory mechanism involved in differential abiotic stress tolerance adaptation traits especially between cultivated and wild chickpea. This will assist us to select best suitable differentially induced/regulated potential genes (especially root-specific genes) from *C. microphyllum* for developing climate resilient cultivars and thereby accelerate translational genomics in chickpea.

### Experimental validation of gene expression

**Figure 3:**
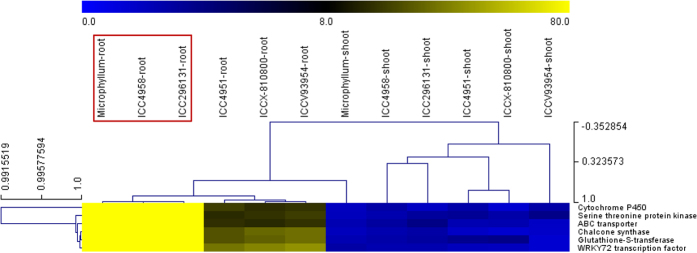
Hierarchical cluster display depicting the differential expression profiles of six candidate genes underlying the major drought yield QTLs in the roots and shoots of three known drought tolerant (*C. microphyllum*, ICC 4958 and ICC 296131) and sensitive (ICC 4951, ICCX- 810800 and ICCV 93954) chickpea accessions. The average log signal expression value of genes is represented at the top with a colour scale; in which blue, black and yellow color denote low, medium and high level of expression, respectively. The tissues/stages and genes selected for expression profiling are mentioned on the upper and right side of expression map, respectively. Digits indicated in the vertical and horizontal bars illustrate the range (minimum, optimum and maximum) of correlation coefficient varying among tissues of drought tolerant and sensitive chickpea accessions, and across genes, respectively. The drought tolerant chickpea accessions exhibiting root-specific expression including pronounced differential up-regulation are highlighted with red boxes. The structural and functional annotation of genes are mentioned in the Tables 4, S2 and S5.

For experimental validation of differentially up- and down-regulated genes identified though transcriptome profiling, the genes exhibiting high root-specific expression in *C. microphyllum* and ICC 4958 were used for differential expression profiling. For this, the RNA isolated from the root and shoot tissues of six drought tolerant (*C. microphyllum*, ICC 4958 and ICC 296131) and sensitive (ICC 4951, ICCX-810800 and ICCV 93954) *desi* and *kabuli* chickpea accessions including a *C. microphyllum* accession (used for transcriptome sequencing) was amplified with the gene-specific and endogenous internal control (*EF1α*) primers using the semi-quantitative and quantitative RT-PCR assays. The up- and down-regulated differential expression profiles of genes observed in roots and shoots of both *C. microphyllum* and ICC 4958 by RT-PCR assay was strongly correlated (85%) with that of our RNA seq-led transcriptome profiling study (Fig. 3, Table S2).

### Development and characterization of genic SSR and SNP markers

**Figure 4:**
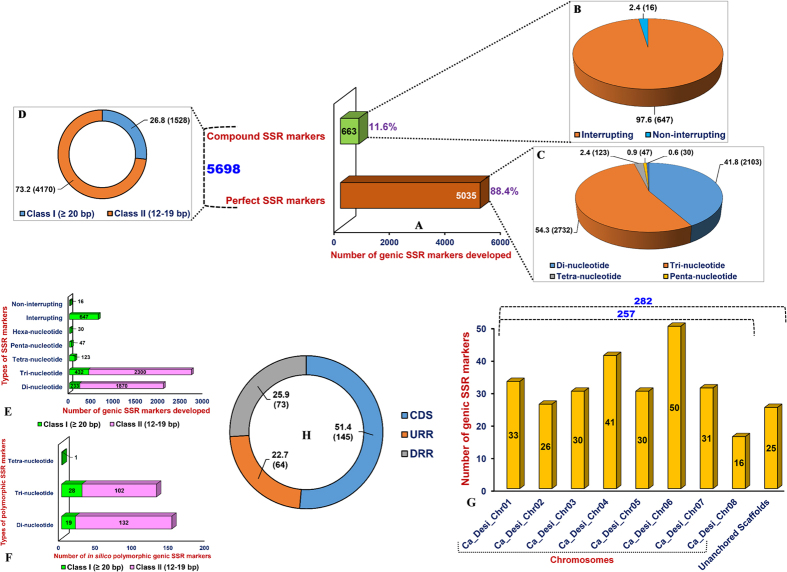
Genomic constitution and relative distribution of genic SSR markers, including *in silico* polymorphic SSR markers (between *C. microphyllum* and ICC 4958) developed from the transcriptome sequences of *C. microphyllum* in the *desi* chickpea genome. (**A**) Frequency (number and %) of compound (**B**) and perfect (**C**) genic SSR markers belonging to different repeat-motif classes. (**D**) Proportion of hypervariable class I and variable class II genic SSR markers. (**E**) Number of class I and class II genic SSR markers belonging to different repeat-motif classes. (**F**) Number of *in silico* polymorphic class I and class II SSR markers representing various repeat-motif classes. (**G**) Number of genic SSR markers physically mapped on eight chromosomes and unanchored scaffolds of *desi* chickpea genome. Digits within/above the bars represent total number of markers mapped. (**H**) Relative distribution and frequency of SSR markers in different coding and non-coding sequence components of genes annotated from *desi* chickpea genome. URR and DRR: upstream/downstream regulatory region and CDS: coding DNA sequence. Digits within the Parentheses indicate number of SSR markers developed.

The *de novo* assembled high-quality transcriptome sequences of *C. microphyllum* were utilized to discover and design genic SSR markers. This led to development of 5698 including 5035 (88.4%) perfect and 663 (11.6%) compound genic SSR markers from the transcript sequences of *C. microphyllum* (Fig. 4A, Table S3). The compound SSR markers included a higher proportion of non-interrupting marker types (97.6%, 647 markers) as compared to that of interrupting markers (2.4%, 16) (Fig. 4B). Among the designed perfect SSR markers, the abundance of tri-nucleotide (54.3%, 2732 markers) than that of di (41.8%, 2103)- and tetra (2.4%, 123)-nucleotide repeat motifs-containing markers was observed (Fig. 4C). The potential variable (12–19 bp) class II (73.2%, 4170 markers) perfect genic SSR markers were found most frequent than the hypervariable (≥20 bp) class I (26.8%, 1528) perfect and compound SSR markers (Fig. 4D,E).

To enrich the informativeness of designed genic SSR markers in *C. microphyllum*, these markers were compared with that derived from a *desi* chickpea accession ICC 4958 based on variation of the repeat-units, which led to develop 282 *in silico* polymorphic genic SSR markers. The fragment length polymorphism detected by *in silico* polymorphic genic SSR markers between *C. microphyllum* and ICC 4958 varied from 2 to 106 bp with an average of 6.6 bp. The di-nucleotide class I (132 markers, 87.4%) *in silico* polymorphic genic SSR markers were found most abundant than that of tri-nucleotide class I (102, 78.5%) and class II (28, 21.5%) SSR markers (Fig. 4F). The *in silico* polymorphic 257 (91.1%) and 25 (8.9%) SSR markers were physically mapped on the eight chromosomes and unanchored scaffolds of the *desi* chickpea genome, respectively (Fig. 4G). Highest and lowest number of markers were mapped on the chromosomes 6 (50 markers) and 8 (16 markers), respectively. The physically mapped 282 polymorphic SSR markers were structurally and functionally annotated in different coding and non-coding regulatory sequence components of 282 *desi* genes. Maximum proportion of polymorphic SSR markers were derived from the CDS (coding DNA sequence) (51.4%, 145 markers) followed by DRRs (downstream regulatory regions) (25.9%, 73) and URRs (upstream regulatory regions) (22.7%, 64) of genes (Fig. 4H, Table S2).

**Figure 5:**
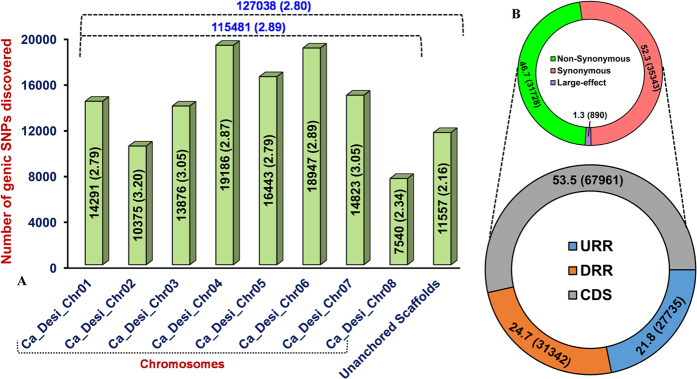
(**A**) Number of genic SNPs physically mapped on eight chromosomes and unanchored scaffolds of *desi* chickpea genome. Digits above/within the bars represent total number of markers mapped. Digits within the Parentheses indicate the SNP frequency which is defined as one SNP mapped per kb. (**B**) Relative distribution and frequency of genic SNPs in different coding (including synonymous, non-synonymous and large-effect substitutions) and non-coding regulatory sequence components of genes annotated from *desi* chickpea genome. Digits within the Parentheses indicate the number of SNPs identified from each sequence regions of genes. URR and DRR: upstream/downstream regulatory region and CDS: coding DNA sequence.

The *de novo* assembled high-quality transcriptome sequences of *C. microphyllum* were compared with the genomic sequences of *desi* (ICC 4958) chickpea to discover genic SNPs at a genome-wide scale. Accordingly, 127038 transcript-derived genic SNPs differentiating the *C. microphyllum* and ICC 4958 were discovered with an average density of one SNP per 2.80 kb (Fig. 5A, Table S4). Of these, 115481 (mean density: 2.80 kb) and 11557 (2.16 kb) SNPs were physically mapped on the eight chromosomes and unanchored scaffolds of the *desi* chickpea genome, respectively. Chromosomes 2 and 8 exhibited highest (3.20 kb) and lowest (2.34 kb) SNP map-density, respectively (Fig. 5A, Table S4). The A/G (50%, 37629 SNPs) and C/T (50%, 37587) genic SNPs classified under transitions (59.2%, 75213) were most abundant than the A/T (32.4%, 16808)-type genic SNPs representing the transversions (40.8%, 51822). The structural annotation of 127038 SNPs mined in diverse sequence components of 9858 genes exhibited the presence of a greater proportion of SNPs in the CDS (53.5%, 67961 SNPs) followed by DRRs (24.7%, 31342 SNPs), and a minimum percentage in the URRs (21.8%, 27735 SNPs) (Fig. 5B, Table S4). The coding SNPs included 35343 (52.3%), 31728 (46.7%) and 890 (1.3%) SNPs in 8219, 6973 and 574 genes revealing synonymous, missense/nonsense non-synonymous and large-effect substitutions, respectively. Notably, the presence of 59077 regulatory SNPs in 8599 genes was observed (Fig. 5B, Table S4).

### Validation and polymorphic potential of genic SSR and SNP markers

To experimentally validate the genic SSR markers, 192 *C. microphyllum* transcript-derived SSR markers and 96 *in silico* polymorphic (between *C. microphyllum* and ICC 4958) SSR markers physically mapped on chromosomes were genotyped in 96 wild and cultivated chickpea accessions using agarose gel-based assay (Table S3). This included six known drought tolerant and sensitive accessions selected in our study for differential expression profiling. Two hundred sixty-three of 288 markers (used for experimental validation) produced single reproducible PCR amplicons with an average amplification success rate of 91.3%. One hundred eighty seven of 263 amplified SSR markers exhibited polymorphism among 96 chickpea accessions with a mean polymorphic potential of 71.1%. The number of alleles detected by the SSR markers varied from 2 to 3 with an average of 2.25 alleles per marker. Notably, 90 (93.7%, mean PIC: 0.62) of 94 amplified SSR markers (physically mapped on chromosomes) showing *in silico* fragment length polymorphism between *C. microphyllum* and ICC 4958 based on repeat-unit variation were experimentally validated successfully using gel-based assay. One hundred eighty-seven polymorphic SSR markers validated in our study were localized in diverse coding and regulatory sequence components of differentially expressed root-specific genes/TFs of *C. microphyllum* and ICC 4958 (Tables S2 and S3). This implicates the functional relevance of genic SSR markers (including *in silico* polymorphic markers) developed from the *C. microphyllum* transcriptome at a genome-wide scale for large-scale genotyping applications including QTL/eQTL (expression QTL) mapping and association analysis to identify potential genes/QTLs controlling important agronomic traits in chickpea.

The validation of 127038 SNPs mined *in silico* between *C. microphyllum* and ICC 4958 exhibited the presence of 1124 (0.9%) SNPs common between our present and past studies^11^,^25^,^26^,^50^,^51^ based on their nature/types of SNP alleles and physical positions (bp) on the *desi* (ICC 4958) genome (Table S4). Interestingly, 30 SNPs in the 26 genes of these were differentially expressed in roots of *C. microphyllum* and ICC 4958. The SNPs discovered from the genes as well as differentially expressed genes exhibiting differentiation between *C. microphyllum* and ICC 4958 could serve as an informative marker resource to be deployed in genomics-assisted breeding applications of chickpea. To experimentally validate the *C. microphyllum* transcriptome-derived genic SNPs, 192 genome-wide well-distributed (physically mapped on chromosomes) SNPs were genotyped in 96 wild and cultivated chickpea accessions using the MALDI-TOF MassARRAY genotyping assay. This included six known drought tolerant and sensitive accessions selected in our study for differential expression profiling. One hundred eighty-three (95.3%, mean PIC: 0.41) of 192 non-synonymous and regulatory SNP loci were successfully validated (polymorphic between *C. microphyllum* and ICC 4958) and exhibited polymorphism among 96 wild and cultivated (*desi* and *kabuli*) chickpea accessions with 95.3% validation success rate. Notably, these experimentally validated informative SNPs were derived from diverse coding and regulatory sequence components of differentially expressed root-specific genes/TFs of *C. microphyllum* and ICC 4958 (Tables S2 and S4). This suggests the functional significance of genic SNPs discovered from *C. microphyllum* transcriptome at a genome-wide scale for high-throughput genetic analysis in chickpea.

### Functional significance of genic SNPs

To determine the functional significance of genic SNPs more elaborately, comparative analysis of transcriptomes by correlating the genes differentially expressed in the roots and shoots of *C. microphyllum* with the control and stress (drought, salinity and cold)-imposed root and shoot tissue samples of ICC 4958 was performed. The detail statistics regarding mapping of transcript sequence reads obtained from ICC 4958 on the *C. microphyllum* transcriptome are mentioned in the Table 1. This identified a total of 13077 including 3580, 3532 and 3524 *C. microphyllum* genes that were differentially up- and down-regulated (≥two-fold at P ≤ 0.05) under at least one root and shoot tissue sample/stress condition of ICC 4958 during drought, salinity and cold stress, respectively (Table S5). The *C. microphyllum* genes with coding and regulatory SNPs differentially regulated in control and stress-imposed root and shoot tissues of ICC 4958 were correlated with the TFs, KOG and KEGG-based functional annotation information of these genes (Table 2). The TFs-encoding *C. microphyllum* genes belonging to different abundant class of TF families such as *MYB, NAC* and *bHLH* that were differentially expressed in roots and shoots of ICC 4958 during drought, cold and salinity stress responses contained more than 1.5 times higher frequency of regulatory SNPs than non-synonymous SNPs. Likewise, higher frequency (2.8 to 4-times) of regulatory SNPs as compared to non-synonymous SNPs in the abiotic stress-responsive differentially expressed genes representing abundant class of KOG and KEGG-based functional categories was observed (Table 3). Henceforth, it will be interesting to correlate the differential expression profiles of genes with the SNPs mined from their gene regulatory regions for understanding the molecular basis of altered gene transcriptional regulation underlying abiotic stress responses in wild and cultivated chickpea.

Our global transcriptome sequencing-based comparative differential gene expression profiles in shoots and roots of *C. microphyllum* and/or ICC 4958 accessions under drought, salinity and cold stress responses was correlated with the occurrence of diverse non-synonymous/large-effect and regulatory SSR and SNP markers in the coding and non-coding sequence components of these genes (Table S6). This analysis identified the presence of 457 SSR and 5687 SNP (including 706 non-synonymous, 9 large-effect and 1948 regulatory SNPs) markers in the 381 and 579 *C. microphyllum* genes, respectively that were differentially expressed in shoots and roots of ICC 4958 during drought, salinity and cold stress responses. Especially, 67 and 141 *C. microphyllum* genes with 86 and 1388 SSR and SNP markers, respectively were differentially expressed in roots of ICC 4958 during drought, salinity and cold stress responses. Of these, 21 and 22 genes with 27 SSR and 295 SNP markers, respectively were differentially regulated especially in roots of *C. microphyllum* (Table S5). Notably, 86 and 217 *C. microphyllum* genes with 125 SSR and 1930 SNP markers, respectively were differentially expressed in roots and shoots of ICC 4958 commonly across drought, salinity and cold stress responses (Table S6). Of these, 25 and 53 *C. microphyllum* genes with 31 SSR and 479 SNP markers, respectively were differentially expressed in roots of ICC 4958 commonly across drought, salinity and cold stress responses whereas 10 genes with markers differentially regulated in roots of *C. microphyllum* (Table S6). The details regarding SSR and SNP markers-containing *C. microphyllum* genes responsive to at least one specific stress condition (drought, cold and salinity) in both the roots and shoots of ICC 4958 and especially in the roots of ICC 4958, are provided in the Table S6.

Collectively, these analyses delineated a diverse set of root- and shoot-specific differentially up- and down-regulated abiotic stress-responsive genes and TFs with regulatory and non-synonymous SNPs exhibiting differentiation between *C. microphyllum* and ICC 4958 (Table S6). Henceforth, these gene-based informative SNP markers can be utilized in rapid targeted mapping of drought, cold and salinity stress-responsive differentially expressed root- and shoot-specific genes on the chromosomes by high-throughput marker genotyping and high-resolution QTL mapping in the advanced generation mapping populations of chickpea. Consequently, this will accelerate the delineation of functionally relevant molecular tags like QTLs, eQTLs (expressed QTLs) and potential genes especially regulating abiotic stress tolerance traits in chickpea. The SNPs being derived from the diverse coding (non-synonymous and large-effect) and regulatory sequence components of drought, salinity and cold stress-responsive differentially expressed genes can also have potential in rapidly establishing marker-trait linkages for identification of potential genes/QTLs and natural allelic variants governing abiotic stress tolerance by high-throughput marker genotyping and high-resolution genetic association mapping in natural germplasm lines of chickpea.

### Construction of a high-resolution inter-specific chickpea genetic linkage (transcript) map

**Figure 6:**
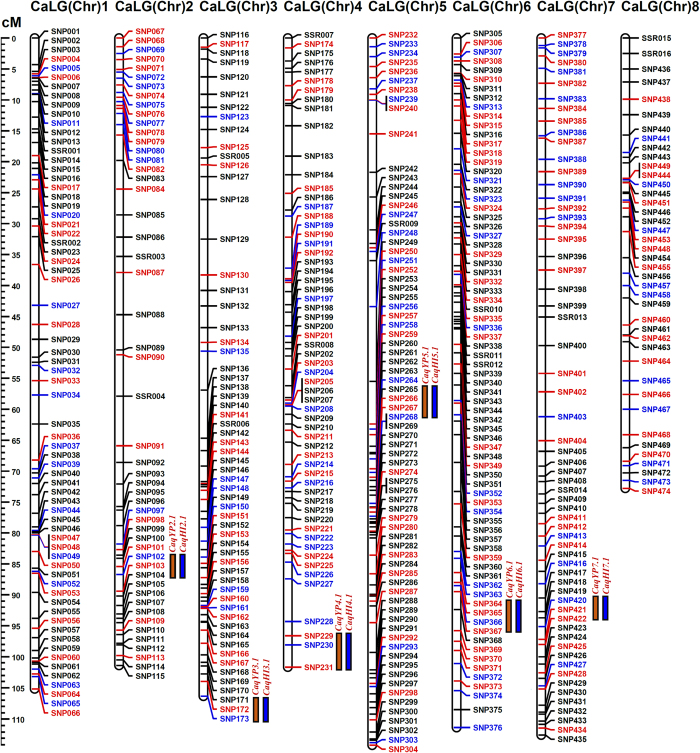
Six major genomic regions harbouring six robust QTLs associated with two drought-responsive yield traits (yield per plant and harvest-index) were identified and mapped on six chromosomes of a high-density integrated inter-specific genetic linkage/trancript map (ICC 4958 × ICC 17163) of chickpea. A left-side scale bar spanning 5 cM uniform genetic distance interval represents the genetic distance (cM) of SSR and SNP marker loci mapped on the eight LGs/chromosomes. The identity of the marker loci integrated on the chromosomes are represented on the right side of the chromosomes. The details of markers flanking and tightly linked to the major drought yield QTLs are mentioned in the Table 4. Orange and blue boxes indicate the QTLs governing yield per plant and harvest-index, respectively mapped on the chromosomes of a high-density transcript map. The SNPs used as anchor markers to construct a transcript map are illustrated with black colour lines. The non-synonymous/regulatory and synonymous SNPs derived from the differentially expressed genes are indicated with red and blue colour lines, respectively.

To construct a saturated inter-specific genetic linkage (transcript) map, 500 SSR and SNP markers derived from the drought-responsive differentially expressed root-specific genes of *C. microphyllum* and ICC 4958 exhibiting polymorphism between parental accessions (ICC 4958 and ICC 17163) were genotyped among 190 individuals of a RIL mapping population (ICC 4958 × ICC 17163). The linkage analysis utilizing 500 marker genotyping information mapped 490 genic marker loci onto eight LGs (linkage groups) of an inter-specific transcript map of chickpea (Table 3, Fig. 6). This eight LGs-based transcript map covered a total map length of 825.4 cM with an average inter-marker distance of 1.68 cM (Table 3, Fig. 6). Longest and shortest transcript map length spanning 112.5 and 72.8 cM were observed in LG5 and LG8, respectively. Maximum (75 markers) and minimum (41) numbers of genic markers were mapped on LG6 and LG8, respectively (Table 3). LG6 contained a most saturated transcript map (an average inter-marker distance of 1.48 cM) whereas LG2 had the least saturated map (1.99 cM) (Table 3). Collectively, an inter-specific transcript map constructed in the present study revealed a comparable/higher mean map-density (an average inter-marker distance of 1.68 cM) than that previously reported in multiple SSR and SNP markers-anchored intra- and inter-specific integrated genetic linkage maps of chickpea^11^,^17^,^25^,^26^,^32^,^40^,^51^,^52^,^53^,^54^. Therefore, the integrated SSR and SNP markers-based transcript map constructed in our study has adequate map-density to be utilized as a reference genetic linkage map for high-resolution molecular mapping of QTLs governing diverse agronomic traits including abiotic stress tolerance traits in chickpea.

### Molecular mapping of drought yield QTLs in chickpea

A significant difference and normal frequency distribution of two drought tolerance-related traits, yield per plant (YP: 18.2–62.8 with 8.7–9.8% CV and 80–82% H^2^) and harvest-index (HI: 12.9–41.7 with 9.3–10.6% CV and 80–81% H^2^) in 190 individuals and two parental accessions of an inter-specific mapping population (ICC 4958 × ICC 17163) under rainfed and irrigated environments across two geographical locations was observed (Table S7). A highly significant positive correlation between YP and HI traits (r: 98% at a P < 10^−4^) based on estimated Pearson’s correlation coefficient (r) was observed. The QTL mapping using the genotyping information of 490 gene-derived SSR and SNP markers mapped on an inter-specific transcript map and field phenotyping data of 190 RIL mapping population identified six major genomic regions harbouring six significant (6.5–11.5 LOD) QTLs associated with YP and HI traits which were mapped on six chromosomes (except chromosomes 1 and 8) of chickpea (Table 4, Fig. 6). These identified QTLs exhibited consistent and stable phenotypic expression with more than 10% PVE (phenotypic variation explained) across rainfed and irrigated environments of two geographical locations and thus were considered as robust QTLs governing YP and HI traits in chickpea. The six major genomic regions underlying robust QTLs covered (1.5 cM on chromosome 5 to 5.0 cM on chromosome 4) with 20 SNP and SSR markers were genetically mapped on LGs/chromosomes and showed positive additive gene effect for increasing yield and harvest-index with large effective allelic contribution from ICC 4958. The proportion of PVE by individual robust QTL varied from 11.2–23.7%. The combined PVE estimated for all six robust QTLs was 39.8% (Table 4, Fig. 6). Six QTLs associated with multiple traits (YP and HI) were mapped on six different genomic regions at the similar marker intervals of chromosomes. The high-resolution QTL mapping identified SSR and SNP markers in the diverse drought-responsive differentially expressed root-specific genes tightly linked to the major drought yield QTLs (YP and HI) which are mentioned in the Table 4 with corresponding marker genetic positions (cM) on the chromosomes. To ascertain the validity of identified robust QTLs, the major genomic regions harbouring six YP and HI QTLs were compared with that documented by previous inter/intra-specific mapping population-led QTL mapping studies^39^,^40^. None of the YP and HI QTLs identified by us exhibited correspondence with previously reported known drought-responsive yield QTLs based on their congruent physical positions on chickpea chromosomes. This implicates the novelty and population-specific characteristics of six major QTLs identified in our study for dissecting the drought tolerance-related yield trait regulation in chickpea. Therefore, six markers-containing drought-responsive root-specific genes tightly linked with the major YP and HI QTLs mapped on chromosomes could have functional relevance to be deployed in marker-assisted genetic enhancement for developing high-yielding drought tolerant chickpea cultivars.

### An integrated genomic approach to delineate candidate genes underlying drought yield QTLs in chickpea

Six robust YP (*CaqYP2.1, CaqYP3.1, CaqYP4.1, CaqYP5.1, CaqYP6.1, CaqYP7.1* and *CaqHI7.1*) and HI (*CaqHI2.1, CaqHI3.1, CaqHI4.1, CaqHI5.1, CaqHI6.1* and *CaqHI7.1*)-associated major QTLs genetically mapped on six chromosomes identified by high-resolution QTL mapping were selected to delineate candidate gene(s) regulating drought tolerance-related yield traits in chickpea (Table 4, Fig. 6). The integration of genetic linkage map information of markers flanking the six major QTLs with that of physical maps of *desi* chickpea genome defined genomic regions (spanning 518931–4888587 bp) harbouring such robust QTLs on six chromosomes (Table 4). The structural and functional annotation of these physical QTL intervals on six chromosomes identified multiple (19 to 230 genes) candidate protein-coding *desi* chickpea genes (Table 4). The differential expression profiles of genes annotated in the major genomic regions harbouring six robust QTLs were determined using the aforementioned comparative transcriptome profiling data generated by us in the roots and shoots of *C. microphyllum* as compared to that of control and drought imposed root and shoot tissues of ICC 4958. Interestingly, non-synonymous and URR SNPs-containing six *C. microphyllum* genes/TFs revealing tight linkage with six major YP and HI QTLs (based on single marker analysis-led high-resolution QTL mapping) exhibited drought-responsive root-specific expression as well as pronounced up-regulated expression (6.4 to 46.8-fold) especially in ICC 4958 based on our comparative transcriptome profiling study. Our quantitative RT PCR-based gene expression profile validation data also revealed up-regulated (>10-fold) differential expression of these six SNPs-carrying genes especially in the roots of known drought tolerant (*C. microphyllum*, ICC 4958 and ICC 296131) chickpea accessions as compared to that of sensitive (ICC 4951, ICCX-810800 and ICCV 93954) accessions (Fig. 3). Interestingly, more than three-fold up-regulation of six genes particularly in the roots of a known drought tolerant hardy wild *C. microphyllum* accession in contrast to other two known drought tolerant cultivated accessions (ICC 4958 and ICC 296131) was observed implicating the efficacy of delineated genes in regulating drought tolerance of chickpea.

The five *desi* genes (Serine threonine protein kinase, ABC transporter G-family, Cytochrome P450, Chalcone synthase and Glutathione-S-transferase) and one TF (*WRKY72*) with non-synonymous and URR SNPs regulating drought tolerance-related yield traits scaled-down in this study, by integrating high-resolution transcript-derived SSR and SNP markers-based QTL mapping with comparative transcriptome profiling are functionally relevant. Based on previous documentation, these six genes/TFs are known to play crucial role in various abiotic (drought) stress responses by regulating diverse DNA binding and transcriptional activity, hormonal cell signalling, transport, metabolism and accumulation of transcripts/proteins in crop plants^55^,^56^,^57^,^58^,^59^,^60^,^61^,^62^,^63^,^64^,^65^,^66^,^67^,^68^,^69^. One of these gene coding for serine threonine protein kinase governing root-mediated drought tolerance-related yield trait has been validated by combining high-density QTL mapping with marker-trait association and gene enrichment analysis in chickpea^70^. The informative non-synonymous/large-effect and regulatory SSR and SNP markers as well as potential differentially expressed root-specific genes underlying major QTLs regulating two most vital drought-responsive yield traits delineated in this study from the comparative *C. microphyllum* and ICC 4958 transcriptome profiles can be deployed for various functional and translational genomic (marker-assisted breeding and transgenics) research. This will provide multiple essential leads, which could accelerate genomics-assisted crop improvement to develop drought tolerant superior high-yielding cultivars of chickpea.

The current study utilized a RNA-seq strategy to sequence transcriptome of an Indian origin perennial wild *C. microphyllum* accession for the first time at a high-resolution scale. The optimized *de novo* high-quality transcriptome assembly and comprehensive transcript sequence data for root and shoot tissues of this wild accession were generated to provide a global view of its gene content. The gene-encoding transcripts differentially expressed between roots and shoots of *C. microphyllum* accession were correlated with that of an available whole genome transcript sequence data of a *desi* chickpea accession (ICC 4958) based on comparative transcriptome profiling to identify candidate genes, transcription factors (TFs) and key regulators/metabolic pathways especially governing inherent root-mediated natural adaptation characteristics of *C. microphyllum* in adverse agro-climatic condition. Based on these, numerous experimentally well-validated *C. microphyllum* transcript/gene-based SSR and SNP markers derived from the coding and regulatory sequence components of genome and/or root- and shoot-specific abiotic (drought, cold and salinity) stress-responsive differentially expressed genes of ICC 4958 were discovered to accelerate high-throughput genetic analysis in chickpea. These informative genic SSR and SNP markers were genotyped successfully in an advanced generation mapping population to construct a high-density inter-specific genetic linkage map (transcript map) and for molecular mapping of major QTLs governing two drought-responsive important yield traits (yield per plant and harvest-index) in chickpea. An integrated genomic approach by combining high-resolution drought yield QTL mapping with comparative transcriptome profiling was employed to delineate candidate gene(s) harbouring major QTLs regulating yield per plant and harvest-index in chickpea. Overall, this study provides a deep transcriptomic insight into the gene regulatory networks governing differential root-mediated natural adaptation responses in a hardy wild perennial *C. microphyllum* accession as compared to that of an already existing cultivated high-yielding drought stress tolerant *desi* chickpea accession (ICC 4958). The comprehensive transcriptome, informative genic SSR and SNP markers, differentially expressed genes, a high-resolution inter-specific genetic linkage/transcript map and major drought yield QTLs and potential genes/natural allelic variants especially generated in our study at a global scale can serve as a useful resource to drive translational genomics for genetic enhancement in chickpea.

## Methods

### Plant materials used for RNA isolation

The seeds of one selected perennial wild (*C. microphyllum*) accession were grown in the pots filled with soil between the temperature range of minimum 17 ± 1.5 °C to maximum 20 ± 1.2 °C at Palampur, Himachal Pradesh (latitude: 32.1°N and longitude: 76.5°E). At least three independent biological replicates of each root and shoot tissue sample were collected from the 10–15 days old seedlings (vegetative stage) of *C. microphyllum* accession and frozen instantly with liquid nitrogen. The total RNA was isolated from the tissues using an RNeasy Plant Mini Kit (QIAGEN, USA) following manufacturer’s instruction. The quality and quantity of RNA samples was assessed by denaturing agarose gel-based assay, NANODROP 2000 Spectrophotometer (Thermo Scientific, NanoDrop products, USA) and Qubit® 3.0 Fluorometer (Thermo Fisher Scientific, USA) following the methods of Garg *et al*.^71^ and Kujur *et al*.^52^.

### NGS-based Illumina transcriptome sequencing

The total high-quality RNA isolated from three independent biological replicates of each root and shoot tissue sample of a *C. microphyllum* accession was used individually to constitute the cDNA libraries. These libraries were sequenced by Illumina HiSeq2000 platform to generate 100 base long paired-end (PE) sequence reads for each sample. The raw Fastq sequences were filtered through the recommended Illumina pipeline and NGS QC Toolkit v2.3^72^ to remove the low-quality including primer/adaptor contaminated sequence reads.

### High-quality transcriptome assembly

For *de novo* transcriptome assembly, the high-quality filtered sequence reads obtained from three independent biological replicates of root and shoot tissue samples of a *C. microphyllum* accession were analysed with Trinity (vr2012-05-18) and CLC Genomics Workbench (v4.7.2) tools. To obtain best transcriptome assembly, most vital parameters including *k*-mer and insert length, and expected coverage were optimized as per Garg *et al*.^19^,^42^. Reference-based transcriptome assembly of *C. microphyllum* using the available draft genome sequence of its evolutionary close *desi* chickpea accession (ICC 4958) (CGAP v2.0^50^) as a reference was performed following the criteria as defined by Garg *et al*.^42^. The diverse quality parameters of developed transcriptome assemblies were evaluated using the perl scripts employed in NGS QC Toolkit. The high-quality transcripts generated for *C. microphyllum* were functionally annotated by their BLASTX search against *desi* chickpea proteome^50^, NCBI non-redundant (nr) protein sequence database (http://www.ncbi.nlm.nih.gov/refseq/about/nonredundantproteins) and PFAM database v27.0 (http://pfam.sanger.ac.uk). The KOG (eukaryotic orthologous groups of proteins, ftp://ftp.ncbi.nih.gov/pub/COG/KOG)-based functional annotation of the *C. microphyllum* transcripts and identification of TF-encoding genes from these transcripts were performed following the methods of Kujur *et al*.^51^ with ≥50 and 70% query coverage and percent identity, respectively.

### Differential gene expression analysis

For gene-encoding transcript expression analysis, all the high-quality sequence reads obtained from each of the root and shoot tissue samples of *C. microphyllum* accession were mapped/aligned individually on the constituted *de novo* transcriptome assembly using Tophat (v2.0.0)^73^ and CLC Genomics Workbench tools (http://www.clcbio.com) with default parameters. The Cufflinks (v2.0.2)^74^, Cuffmerge and Cuffdiff were used to estimate the differential expression of genes/transcripts in roots and shoots of *C. microphyllum* accession. The genes/transcripts revealing at least two-fold expression change with P value ≤ 0.05, were considered significant to be differentially expressed.

For comparative transcriptome profiling, the raw transcriptome sequence data of control (unstressed) as well as drought, cold and salinity stress-imposed root and shoot tissue samples of ICC 4958 that are freely accessible at NCBI Gene Expression Omnibus (GEO) database (GSM1299261-GSM1299268) were retrieved^38^. The processing of these raw sequence reads to obtain high-quality reads, reads mapping to *C. microphyllum* transcript assembly and differential expression profiling of root and shoot (control/unstressed)-derived transcripts/genes between *C. microphyllum* and ICC 4958 were performed following the above-mentioned strategy. The heatmaps depicting the differential expression profiles of genes/transcripts (log2 fold-change) in unstressed control roots and shoots of *C. microphyllum* and ICC 4958 were constructed based on hierarchical clustering employing MultiExperiment Viewer (Mev v4.9). The KOG-based functional annotation of the *C. microphyllum* and ICC 4958 transcripts and TF-encoding gene identification from the differentially expressed transcripts were performed following the aforesaid strategy. The molecular interactions based on biochemical pathway mapping of differentially expressed genes/transcripts were performed using KEGG pathway database (http://www.genome.jp/kegg/pathway.html).

### Experimental validation of gene expression

For experimental validation of gene expression through semi-quantitative and quantitative real-time (RT) PCR assays, the genes/transcripts exhibiting high root-specific differential expression especially in *C. microphyllum* and ICC 4958 were selected to design primers using Primer Express (v3.0) (Applied Biosystems, USA). The RNA isolated from the root and shoot tissue samples of six drought tolerant and sensitive *desi* and *kabuli* chickpea accessions including a *C. microphyllum* accession (used for transcriptome sequencing) following aforesaid methods. In the RT-PCR assay, RNA isolated from three independent biological replicates of each root and shoot sample and two technical replicates of each biological replicate with no template and primer as control was used for expression study. For gene/transcript expression profiling, the isolated high-quality RNA was amplified with gene-specific primers using ABI 7500 Real-Time PCR System (Applied Biosystems, USA), following the methods as previously described by Kujur *et al*.^52^ and Bajaj *et al*.^75^. An internal control gene elongation factor 1-alpha (*EF1α*) was utilized in RT-PCR assay for normalization of gene/transcript expression value. The correlation between transcriptome sequencing and RT-PCR profiles assayed in roots and shoots of *C. microphyllum* and ICC 4958 was analysed through R program. The differential expression (fold change) profiles of genes/transcripts assayed in roots and shoots of drought tolerant and sensitive chickpea accessions was measured. Significant difference in gene/transcript expression among diverse tissues and/or accessions was determined by LSD-ANOVA significance test using SPSS 17.0 (http://www.spss.com/statistics). The significant difference in gene/transcript expression profile was visualized by a heat map using MeV.

### Development and annotation of genic SSR and SNP markers

To discover genic SSRs, the *C. microphyllum* transcripts were analysed with MISA (MIcroSAtellite; http://pgrc.ipk-gatersleben.de/misa/) as per Kujur *et al*.^52^. The *in silico* polymorphic genic SSRs based on variation of repeat-units length in the transcript/gene sequences between *C. microphyllum* and ICC 4958 (*desi* reference genome) were identified following the methods as described previously^27^,^76^,^77^. To develop genic SSR markers, the primer-pairs were designed from the sequences flanking the SSR repeat-motifs including the *in silico* polymorphic SSR repeats using the Primer3 interface module of MISA as per Kujur *et al*.^52^.

The genic SNPs in the transcript/gene sequences between *C. microphyllum* and ICC 4958 were mined using Tophat and samtool. The high-quality genic SNPs differentiating the *C. microphyllum* and ICC 4958 accessions were screened following the criteria as defined by Jain *et al*.^78^ and Kujur *et al*.^51^. These mined SSR (*in silico* polymorphic SSR) and SNP markers were structurally and functionally annotated in the diverse coding and non-coding regulatory sequence components of *desi* chickpea (ICC 4958) genome as per Jain *et al*.^78^ and Kujur *et al*.^42^,^51^. The functional significance of SSRs and SNP markers was determined based on their presence particularly in the coding (including non-synonymous and large-effect substitutions) and regulatory regions of genes/TF-encoding genes of *C. microphyllum* that were differentially expressed in the roots and shoots of ICC 4958 under drought, cold and salinity stresses.

### Validation of genic SSR markers

For experimental validation of genic SSR markers, 192 *C. microphyllum* transcript-derived SSR markers and 96 *in silico* polymorphic SSR markers (physically mapped on chromosomes) were selected based on their localization especially in the diverse coding and regulatory sequence components of root-specific genes (TFs)/transcripts of *C. microphyllum* that were differentially expressed especially in the roots of ICC 4958 under drought stress. These SSR markers were genotyped in 96 wild and cultivated chickpea accessions^2^,^11^ (including known drought tolerant and sensitive accessions selected for differential expression profiling) following the methods as described previously^27^,^76^,^77^.

### Validation of genic SNPs

To validate genic SNPs identified between *C. microphyllum* and ICC 4958 *in silico*, the genotyping information of SNPs differentiating these two chickpea accessions was compared/correlated with the available *in silico* SNP database according to their nature/types of SNP alleles and physical positions (bp) on the *desi* (ICC 4958) genome^11^,^25^,^26^,^50^,^51^. For experimental validation and understanding the functional significance of mined SNPs, 192 coding (including non-synonymous and large-effect substitutions) and regulatory SNPs (physically mapped on chromosomes) were selected from root-specific genes/TF-encoding genes of *C. microphyllum* based on their drought-responsive differential expression especially in the roots of ICC 4958. These SNPs were further genotyped in 96 wild and cultivated chickpea accessions (including known drought tolerant and sensitive accessions selected for differential expression profiling) using Sequenom MALDI-TOF (matrix-assisted laser desorption ionization-time of flight) MassARRAY as per Saxena *et al*.^2^ and Bajaj *et al*.^11^.

### Construction of an inter-specific genetic linkage map and QTL mapping

A 190 F_8_ RIL (recombinant inbred line) mapping population derived from the inter-specific crosses between two parental chickpea accessions (*C. arietinum desi* accession ICC 4958 × *C. reticulatum* wild accession ICC 17163) was developed to construct a genetic linkage map (transcript map) and for identification/mapping of major drought-responsive yield QTLs in chickpea. The genotyping data of drought-responsive root-specific differentially expressed gene-derived SSR (genotyped by gel-based assay) and SNP (Sequenom MALDI-TOF MassARRAY) markers revealing polymorphism between mapping parental accessions (ICC 4958 and ICC 17163) was utilized to generate an inter-specific genetic linkage map (transcript map) at a higher LOD (logarithm of odds) threshold (>4.0) with Kosambi mapping function following Saxena *et al*.^17^ and Bajaj *et al*.^79^. Accordingly, the informative polymorphic genic markers were integrated into eight LGs of a transcript map based on their centiMorgan (cM) genetic distance. The LGs were designated (LG1 to LG8) as per their corresponding marker physical positions (bp) on the chromosomes of *desi* chickpea genome. The previously documented genic SNP markers mapped on eight LGs exhibiting polymorphism between two mapping parental accessions (ICC 4958 and ICC 17163) were used as anchor markers to define the identity of LGs/chromosomes of an inter-specific genetic map constructed in the present study. The identity of LGs was further ascertained by positions (cM) and groupings shared by anchor markers among eight LGs/chromosomes between current and past studies^25^,^26^.

The RIL mapping individuals and two parental accessions (ICC 4958 and ICC 17163) were grown in the field [as per randomized block design (RBD) with at least two replications] during crop growing season (minimum 13 ± 2.2 °C and maximum 21 ± 2.5 °C) for two consecutive years (2012 and 2013) under rainfed and irrigated environments at two diverse geographical locations (New Delhi: latitude 28.6 °C and longitude 77.2 °C, Palampur: 32.1 °C and 76.5 °C) of India. Accordingly, these were phenotyped for two vital drought tolerance-related traits, namely yield (g) per plant (YP) and harvest-index (HI%) especially under rainfed and irrigated environments following Varshney *et al*.^40^. The mean, standard deviation, coefficient of variation (CV), frequency distribution, Pearson’s correlation coefficient and broad-sense heritability (H^2^) of YP and HI traits phenotyped in a RIL mapping population were measured as per Saxena *et al*.^17^ and Bajaj *et al*.^79^. The QTL mapping was performed by integrating the genotyping data of genic SSR and SNP markers genetically mapped on eight LGs/chromosomes with the field phenotypic data (YP and HI) of 190 RIL mapping individuals and parental accessions based on composite interval mapping function of MapQTL v6.0. The LOD threshold score of more than 4.0 at 1000 permutation was considered significant (p < 0.05) to identify and map the major QTLs on LGs/chromosomes governing YP and HI traits in chickpea. The positional genetic effect and PVE% defined by QTLs were evaluated at a significant LOD as per Saxena *et al*.^17^ and Bajaj *et al*.^79^.

## Additional Information

**How to cite this article**: Srivastava, R. *et al*. Transcriptome landscape of perennial wild *Cicer microphyllum* uncovers functionally relevant molecular tags regulating agronomic traits in chickpea. *Sci. Rep.* **6**, 33616; doi: 10.1038/srep33616 (2016).

## Supplementary Material

Supplementary Information

## Figures and Tables

**Table 1:** Summary statistics defining the sequencing data generated and mapped to *C. microphyllum* transcriptome.

*Cicer* accessions	Tissue samples/ conditions		Average sequence-reads (bp) generated	Average high-quality filtered sequence-reads (bp)	Average bp-sequence-reads (%) mapped
*C. microphyllum* (wild perennial)	Root	Control	54710508	48564742	42691898 (87.9)
	Shoot	Control	64043612	57336824	51420350 (89.7)
Total			**118754120**	**105901566**	**94112248 (88.8)**
*ICC 4958 (cultivated *desi* annual)	Root	Control	31028774	24735291	17919127 (72.4)
		Drought	41375822	31379229	22487640 (71.7)
		Salinity	41131359	32957042	24671371 (74.9)
		Cold	46076764	33055693	24872415 (75.2)
	Shoot	Control	38025508	31510717	24739582 (78.5)
		Drought	40312475	32791372	25641449 (78.2)
		Salinity	39821172	32402177	25672827 (79.2)
		Cold	42284442	33386737	26255946 (78.6)
Total			**320056316**	**252218258**	**192260357 (78.6)**

*Raw sequence data downloaded from NCBI Gene Expression Omnibus (GEO) database with accession IDs GSM1299261–GSM1299268 (Garg *et al*.^38^). Average sequence reads were obtained by estimating the mean of reads generated from three independent biological replicates of each sample.

**Table 2:** Characteristics of *C. microphyllum* gene-derived SNPs mined from the abiotic stress-responsive genes of ICC 4958 representing different abundant functional classes.

Functional classification		Total SNPs mined	Non-Synonymous SNPs	Regulatory SNPs
TFs	*MYB* (myeloblastosis)	67 (17)	14 (6)	17 (5)
	*bHLH* (basic helix-loop-helix)	209 (23)	17 (10)	50 (9)
	*NAC* (No apical meristem Arabidopsis transcription activation factor-cup shaped cotyledon)	216 (13)	47 (10)	45 (6)
KOG	J (translation, ribosomal structure and biogenesis)	93 (13)	2 (2)	54 (7)
	O (post-translational modification, protein turnover and chaperones)	138 (19)	10 (6)	65 (10)
	C (Energy production and conversion)	126 (12)	10 (5)	22 (6)
KEGG	Genetic information processing	180 (13)	13 (3)	91 (9)
	Metabolism	538 (65)	82 (30)	169 (32)
	Environmental information processing	61 (7)	8 (3)	21 (2)

Parentheses indicate the number of genes with SNPs.

**Table 3:** Characteristics of an inter-specific chickpea genetic linkage (transcript) map comprising eight chromosomes constructed using a RIL mapping population (ICC 4958 × ICC 17163).

Linkage groups (LGs)/chromosomes (Chr)	Transcript-derived genic SSR and SNP markers	Map length covered (cM)	Mean inter-marker distance (cM)
LG(Chr)01	68	105.2	1.55
LG(Chr)02	51	101.4	1.99
LG(Chr)03	60	106.4	1.77
LG(Chr)04	60	101.7	1.69
LG(Chr)05	74	114.1	1.54
LG(Chr)06	75	111.3	1.48
LG(Chr)07	61	112.5	1.84
LG(Chr)08	41	72.8	1.77
Total	**490**	**825.4**	**1.68**

**Table 4:** Candidate genes underlying six major QTLs regulating YP and HI traits identified/mapped on the chromosomes by integrating high-resolution QTL mapping with comparative global transcriptome profiling.

*QTLs	**LGs/chromosomes**	**SSR and SNP markers mapped at QTL intervals with genetic positions** (**cM**)	**SSR and SNP markers mapped at QTL intervals with physical positions** (**bp**)	**Candidate** ***desi*** **genes annotated at physical QTL intervals**	**SNPs tightly linked to major QTLs mapped genetically** (**cM**) **and physically** (**bp**) **on chromosomes**	***C. microphyllum*** **genes with SNPs tightly linked to major QTLs exhibiting drought-responsive root-specific differential expression in ICC 4958 based on comparative transcriptome profiling**	**2012**			**2013**		
							**LOD**	**PVE** (**%**)	**A**	**LOD**	**PVE** (**%**)	**A**
*CaqYP2.1 CaqHI2.1*	CaLG(Chr)02	SNP102 (86.7) to SNP104 (90.2): 3.5 cM	SNP102 (19217598) to SNP104 (19858454): 640856 bp	19	SNP103 (89.4 cM) (19217771 bp) Non-synonymous SNP	Serine threonine protein kinase (29-fold upregulated)	8.0	13.4	2.7	8.5	12.8	2.5
*CaqYP3.1 CaqHI3.1*	CaLG(Chr)03	SNP171 (102.8) to SNP173 (106.4): 4.4 cM	SNP171 (39595395) to SNP173 (41312124): 1716729 bp	187	SNP172 (104.0 cM) (41309731 bp) Non-synonymous SNP	ABC transporter G-family (16-fold upregulated)	7.5	12.8	3.0	7.1	11.7	3.2
*CaqYP4.1 CaqHI4.1*	CaLG(Chr)04	SNP229 (96.6) to SNP231 (101.6): 5.0 cM	SNP229 (42305468) to SNP231 (47194055): 4888587 bp	230	SNP231 (101.6 cM) (47194055 bp) Non-synonymous SNP	Cytochrome P450 (6.4-fold upregulated)	6.7	11.7	4.1	6.5	11.2	4.0
*CaqYP5.1 CaqHI5.1*	CaLG(Chr)05	SNP265 (75.8) to SNP268 (77.3): 1.5 cM	SNP265 (30096600) to SNP268 (30668019): 571419 bp	59	SNP266 (75.9 cM) (30165766 bp) URR-SNP	Chalcone synthase (31.6-fold upregulated)	8.2	16.5	2.8	8.0	17.1	2.5
*CaqYP6.1 CaqHI6.1*	CaLG(Chr)06	SNP364 (86.4) to SNP367 (89.5): 3.1 cM	SNP364 (45260071) to SNP367 (45779002): 518931 bp	24	SNP365 (87.9 cM) (45774802 bp) URR-SNP	Glutathione-S-transferase (19.8-fold upregulated)	9.5	19.6	3.7	10.0	18.5	3.2
*CaqYP7.1 CaqHI7.1*	CaLG(Chr)07	SNP420 (95.1) to SNP422 (98.0): 2.9 cM	SNP420 (36856864) to SNP422 (38002080): 1145216 bp	116	SNP421 (97.2 cM) (36857151 bp) URR-SNP	WRKY72 transcription factor (46.8-fold upregulated)	10.8	22.5	4.5	11.5	23.7	4.0

*CaqYP2.1 (QTL for yield per plant on chromosome 2 number 1) and *CaqHI2.1* (QTL for harvest-index on chromosome 2 number 1). LOD: logarithm of odds, PVE: phenotypic variation explained. A: additive effect of alleles from ICC 4958 with high YP and HI. Details regarding the genes and markers are provided in the Tables S4 and S5. URR: upstream regulatory region.
