# Supplementary material for: Transcriptome landscape of perennial wild Cicer microphyllum uncovers functionally relevant molecular tags regulating agronomic traits in chickpea
Source: Sci Rep. 2016 Sep 29;6:33616. doi: 10.1038/srep33616 (PMC5041113; doi:10.1038/srep33616)
Supplement: Supplementary Information [file srep33616-s1.pdf]

Transcriptome landscape of perennial wild *Cicer microphyllum* uncovers functionally relevant molecular tags regulating agronomic traits in chickpea

Rishi Srivastava[1], Deepak Bajaj[1], Ayushi Malik[2], Mohar Singh[3], Swarup K. Parida[1]*

[1]National Institute of Plant Genome Research (NIPGR), Aruna Asaf Ali Marg, New Delhi 110067, India

[2]Faculty of Science, Jamia Hamdard University, Hamdard Nagar, New Delhi 110062, India

[3]National Bureau of Plant Genetic Resources Regional Station, Shimla, Himachal Pradesh 171004, India

*Corresponding authors

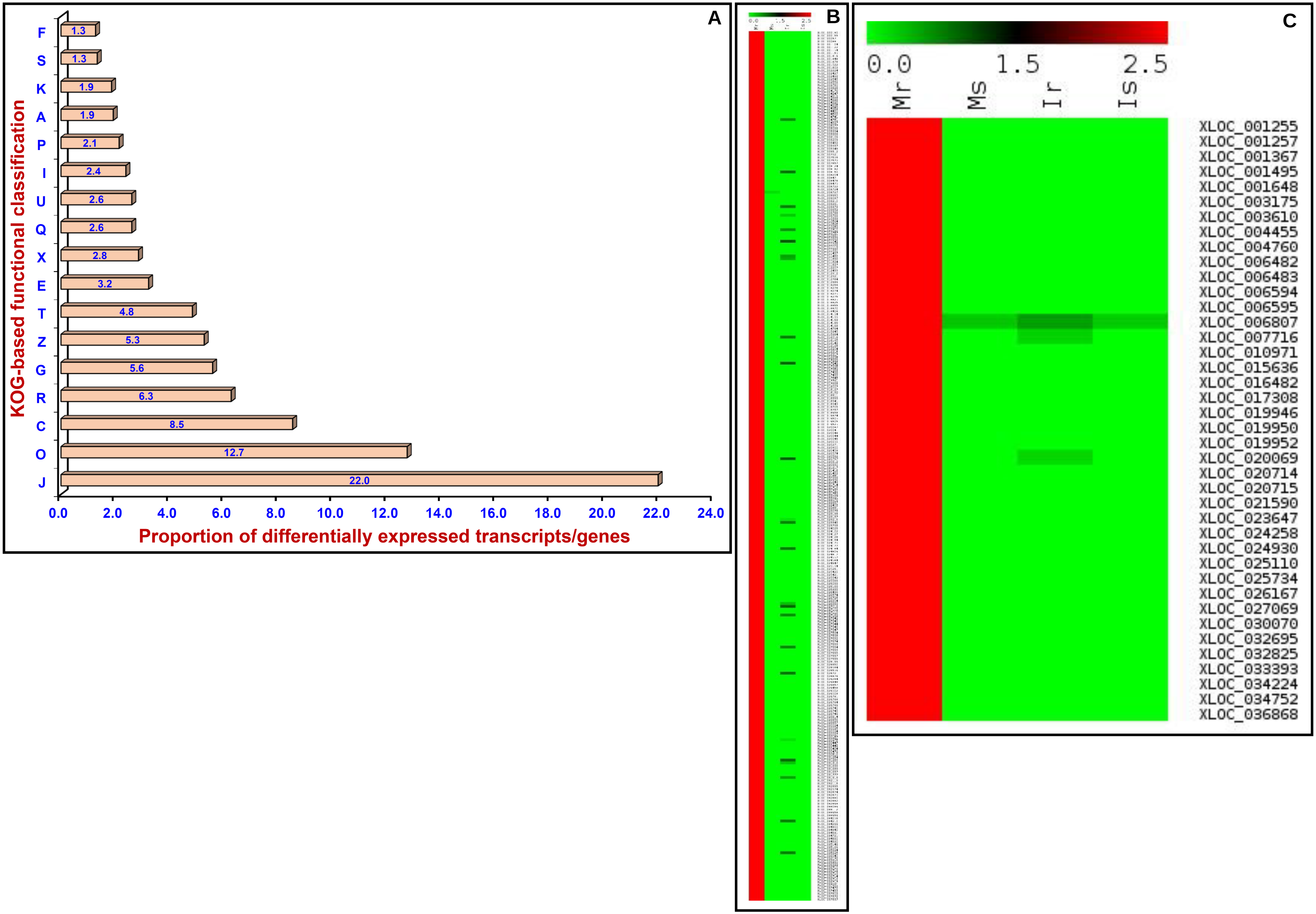
A
KOG-based functional classification
F
S
K
A
P
I
U
Q
X
E
T
Z
G
R
C
O
J
1.3
1.3
1.9
1.9
2.1
2.4
2.6
2.6
2.8
3.2
4.8
5.3
5.6
6.3
8.5
12.7
22.0
0.0
2.0
4.0
6.0
8.0
10.0
12.0
14.0
16.0
18.0
20.0
22.0
24.0
Proportion of differentially expressed transcripts/genes
B
C
0.0
1.5
2.5
Mr
Ms
Ir
Is
XLOC_001255
XLOC_001257
XLOC_001367
XLOC_001495
XLOC_001648
XLOC_003175
XLOC_003610
XLOC_004455
XLOC_004760
XLOC_006482
XLOC_006483
XLOC_006594
XLOC_006595
XLOC_006807
XLOC_007716
XLOC_010971
XLOC_015636
XLOC_016482
XLOC_017308
XLOC_019946
XLOC_019950
XLOC_019952
XLOC_020069
XLOC_020714
XLOC_020715
XLOC_021590
XLOC_023647
XLOC_024258
XLOC_024930
XLOC_025110
XLOC_025734
XLOC_026167
XLOC_027069
XLOC_030070
XLOC_032695
XLOC_032825
XLOC_033393
XLOC_034224
XLOC_034752
XLOC_036868

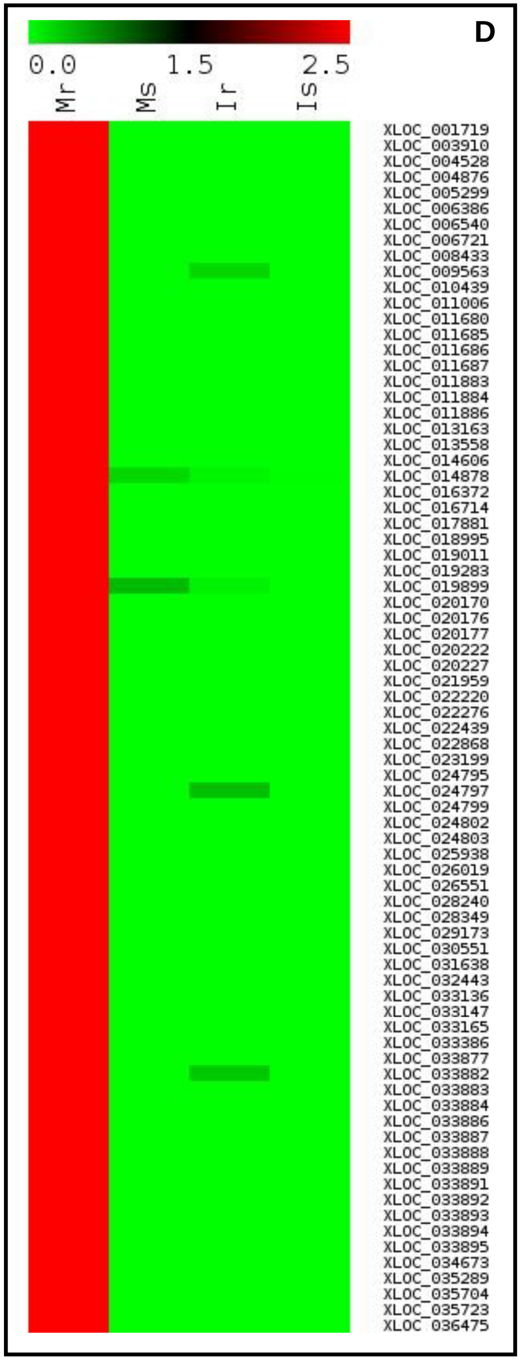

Figure S1. (A) Frequency (%) distribution of genes representing diverse KOG-based functional classes differentially expressed in roots and shoots of *C. microphyllum* and ICC 4958. Heat-maps exhibiting the differential expression profiles of root-specific genes enriched with three most abundant KOG-based functional classes [J (B), C (C) and O (D)] in *C. microphyllum*. These differentially expressed genes were scanned from the comparative transcriptome profiles of roots and shoots between *C. microphyllum* and ICC 4958. The average log signal expression value of genes is represented at the top with a colour scale; in which green, black and red color denote low, medium and high level of expression, respectively. The tissues/stages and genes selected for expression profiling are mentioned on the upper and right side of expression map, respectively. Digits indicated in the vertical and horizontal bars illustrate the range (minimum, optimum and maximum) of correlation coefficient varying among tissues of *C. microphyllum* and/or ICC 4958, and across genes, respectively. The structural and functional annotation of genes are mentioned in the Tables S2 and S5. Mrc: *Microphyllum* root control, Msc: *Microphyllum* shoot control, Irc: ICC 4958 root control, Isc: ICC 4958 shoot control. The diverse KOG classes are defined as J: Translation, ribosomal structure and biogenesis, A: RNA processing and modification, K: Transcription, T: Signal transduction mechanisms, Z: Cytoskeleton, O: Post-translational modification, protein turnover and chaperones, C: Energy production and conversion, F: Nucleotide transport and metabolism, G: Carbohydrate transport and metabolism, E: Amino acid transport and metabolism, I: Lipid transport and metabolism, P: Inorganic ion transport and metabolism, Q: Secondary metabolites biosynthesis, transport and catabolism, R: General function prediction only, S: Function unknown, U: Intracellular trafficking, secretion, and vesicular transport, and X: Unnamed proteins.

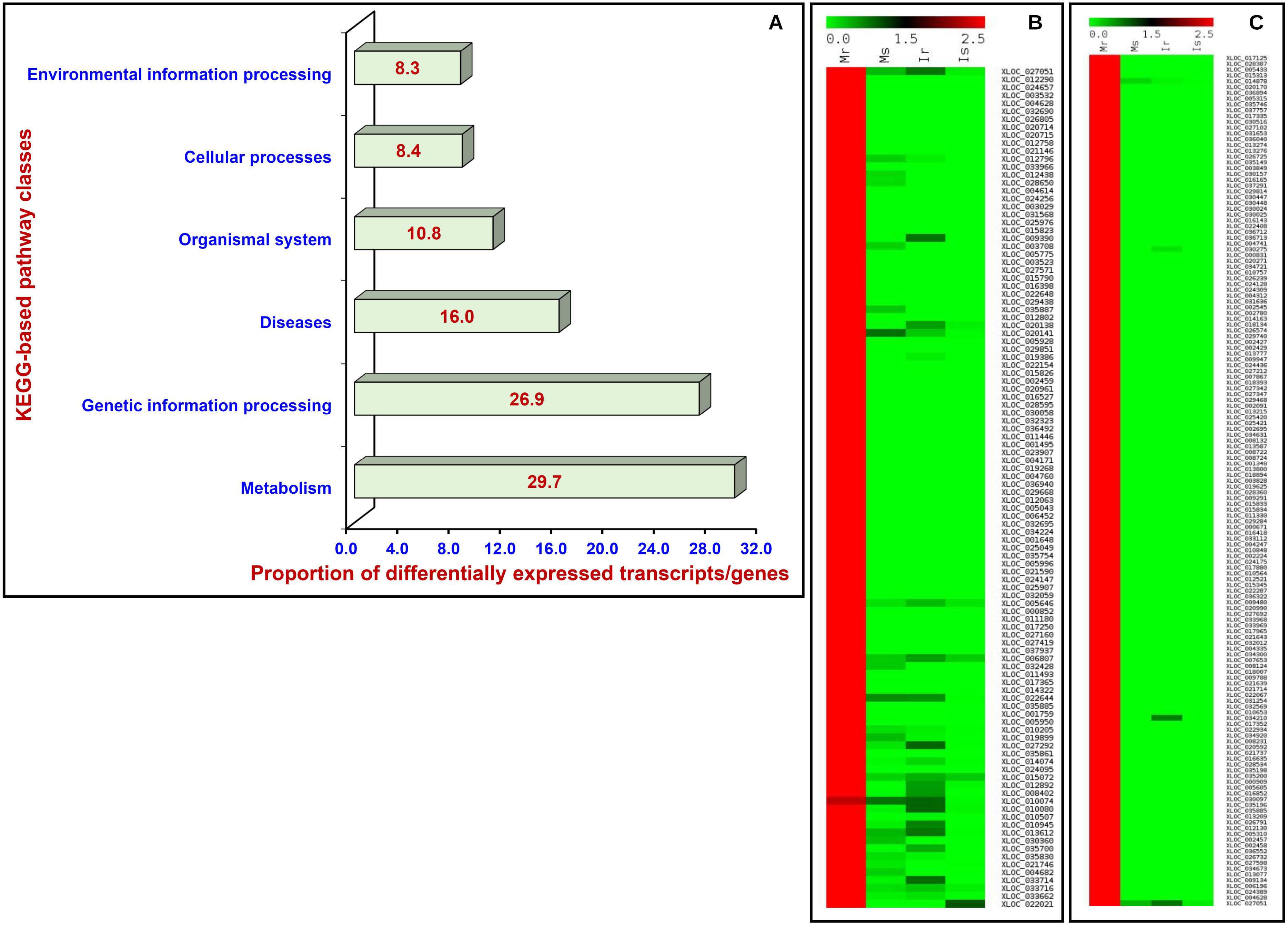
A
KEGG-based pathway classes
Environmental information processing
8.3
Cellular processes
8.4
Organismal system
10.8
Diseases
16.0
Genetic information processing
26.9
Metabolism
29.7
0.0
4.0
8.0
12.0
16.0
20.0
24.0
28.0
32.0
Proportion of differentially expressed transcripts/genes
B
0.0
1.5
2.5
Mr
Ms
Ir
Is
C
0.0
1.5
2.5
Mr
Ms
Ir
Is

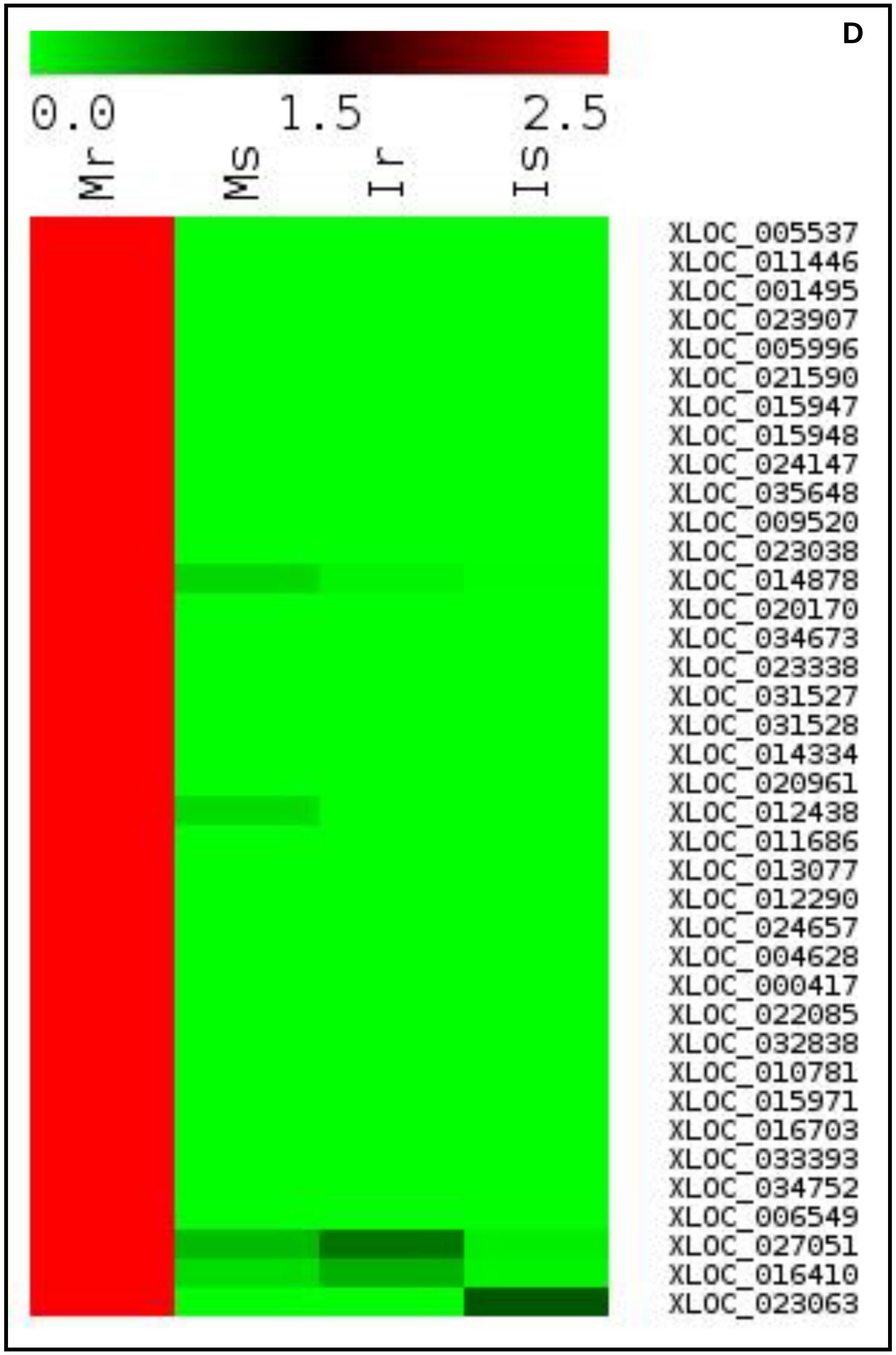

Figure S2. (A) Frequency (%) distribution of genes representing diverse KEGG-based pathway classes differentially expressed in roots and shoots of *C. microphyllum* and ICC 4958. Heat-maps exhibiting the differentially expression profiles of root-specific genes enriched with three most abundant classes of KEGG-based biochemical pathways [metabolism **(B)**, genetic information processing **(C)** and environmental information processing **(D)**] in *C. microphyllum*. These differentially expressed genes were scanned from the comparative transcriptome profiles of roots and shoots between *C. microphyllum* and ICC 4958. The average log signal expression value of genes is represented at the top with a colour scale; in which green, black and red color denote low, medium and high level of expression, respectively. The tissues/stages and genes selected for expression profiling are mentioned on the upper and right side of expression map, respectively. Digits indicated in the vertical and horizontal bars illustrate the range (minimum, optimum and maximum) of correlation coefficient varying among tissues of *C. microphyllum* and/or ICC 4958, and across genes, respectively. The structural and functional annotation of genes are mentioned in the Tables S2 and S5. Mrc: *Microphyllum* root control, Msc: *Microphyllum* shoot control, Irc: ICC 4958 root control, Isc: ICC 4958 shoot control.

Table S1. Assembly statistics of *C. microphyllum* transcriptome

Total high-quality sequence-reads used	105901566
Total transcripts generated	154352
Size (Mb) covered	167
Average transcript length (bp) obtained	896.57
N50 length (bp) estimated	1641
GC%	40.1

Table S2. Differentially expressed genes-encoding transcripts identified from the comparative transcriptome profiles of roots and shoots between *microphyllum* (M) and ICC 4958 (I)

Differentially expressed gene (DEG) IDs	*C. microphyllum* root and shoot-specific (>2 fold)	*C. microphyllum* root and shoot-specific (>10 fold)	ICC4958 and *C. microphyllum* root-specific (>2 fold)	*C. microphyllum* root-specific (>2 fold)	Functional Annotation			
					NCBI nr IDs	TFs	KOG	KEGG
XLOC_000011	No	No	No	No	--	--	--	--
XLOC_000040	No	No	No	No	XP_004509869.1	--	--	--
XLOC_000164	No	No	No	No	--	--	--	--
XLOC_000190	No	No	No	No	XP_004512050.1	ZF-HD	--	--
XLOC_000292	No	No	No	No	--	--	--	--
XLOC_000321	No	No	No	No	--	--	--	--
XLOC_000455	No	No	No	No	XP_004488734.1	--	--	--
XLOC_000500	No	No	No	No	--	--	--	--
XLOC_000506	No	No	No	No	XP_004511808.1	bZIP	--	--
XLOC_000510	No	No	No	No	XP_004510943.1	LBD	--	--
XLOC_000532	No	No	No	No	XP_004488076.1	--	--	--
XLOC_000545	No	No	No	No	XP_012573137.1	--	--	--
XLOC_000562	No	No	No	No	--	--	--	--
XLOC_000597	No	No	No	No	KEH37267.1	--	--	--
XLOC_000629	No	No	No	No	XP_004517017.1	--	R	K19788
XLOC_000714	No	No	No	No	--	--	--	--
XLOC_000765	No	No	No	No	XP_004508413.1	--	--	--
XLOC_000771	No	No	No	No	XP_004502269.1	--	J	--
XLOC_000791	No	No	No	No	XP_004508401.1	--	--	--
XLOC_000823	No	No	No	No	XP_004502147.1	--	P	K08176
XLOC_000838	No	No	No	No	XP_004504956.1	--	--	--
XLOC_000947	No	No	No	No	XP_004516837.1	--	--	--
XLOC_001055	No	No	No	No	XP_004491329.1	WRKY	--	--
XLOC_001078	No	No	No	No	XP_004514799.1	--	E	--
XLOC_001177	No	No	No	No	XP_004498184.1	--	I	K01897
XLOC_001259	No	No	No	No	XP_004510906.1	--	--	--
XLOC_001292	No	No	No	No	XP_012571897.1	--	--	K18213
XLOC_001321	No	No	No	No	XP_004488612.1	--	--	--
XLOC_001322	No	No	No	No	--	--	--	--
XLOC_001332	No	No	No	No	XP_004506738.1	--	O	--
XLOC_001340	No	No	No	No	--	--	--	--
XLOC_001361	No	No	No	No	--	--	--	--
XLOC_001447	No	No	No	No	XP_004509465.1	--	R	--
XLOC_001553	No	No	No	No	--	--	--	--
XLOC_001574	No	No	No	No	XP_004503606.1	--	O	--
XLOC_001595	No	No	No	No	KJZ78175.1	NAC	J	K02976
XLOC_001633	No	No	No	No	--	--	--	--

Differentially expressed gene (DEG) IDs	*C. microphyllum* root and shoot-specific (>2 fold)	*C. microphyllum* root and shoot-specific (>10 fold)	ICC4958 and *C. microphyllum* root-specific (>2 fold)	*C. microphyllum* root-specific (>2 fold)	Functional Annotation			
					NCBI nr IDs	TFs	KOG	KEGG
XLOC_001744	No	No	No	No	--	--	--	--
XLOC_001751	No	No	No	No	XP_004496654.1	--	--	--
XLOC_001753	No	No	No	No	--	--	--	--
XLOC_001779	No	No	No	No	--	--	--	--
XLOC_001860	No	No	No	No	XP_012568436.1	--	--	--
XLOC_001924	No	No	No	No	XP_004489636.2	--	--	--
XLOC_001948	No	No	No	No	XP_004493718.1	--	--	--
XLOC_002050	No	No	No	No	XP_004504125.1	--	S	--
XLOC_002053	No	No	No	No	--	--	--	--
XLOC_002092	No	No	No	No	--	--	--	--
XLOC_002139	No	No	No	No	--	--	--	--
XLOC_002205	No	No	No	No	XP_004486177.1	WRKY	--	--
XLOC_002210	No	No	No	No	XP_004508411.1	--	P	--
XLOC_002214	No	No	No	No	XP_004498397.1	--	--	K15382
XLOC_002297	No	No	No	No	XP_004499315.1	--	G	K01176
XLOC_002343	No	No	No	No	XP_004493069.1	bHLH	--	--
XLOC_002443	No	No	No	No	XP_004515597.1	--	T	--
XLOC_002494	No	No	No	No	XP_004503743.1	SBP	--	--
XLOC_002520	No	No	No	No	XP_004494144.1	--	--	--
XLOC_002528	No	No	No	No	--	--	--	--
XLOC_002531	No	No	No	No	KEH18593.1	--	--	--
XLOC_002536	No	No	No	No	--	--	--	--
XLOC_002586	No	No	No	No	XP_004494691.1	--	--	--
XLOC_002639	No	No	No	No	XP_012573103.1	--	--	--
XLOC_002660	No	No	No	No	--	--	--	--
XLOC_002781	No	No	No	No	XP_004500509.1	--	--	--
XLOC_002785	No	No	No	No	XP_004504123.1	--	--	--
XLOC_002827	No	No	No	No	XP_003593663.1	--	--	K03676
XLOC_002908	No	No	No	No	XP_012574444.1	--	O	--
XLOC_003099	No	No	No	No	--	--	--	--
XLOC_003108	No	No	No	No	XP_004497112.1	--	--	--
XLOC_003217	No	No	No	No	XP_004487348.1	--	--	K18812
XLOC_003287	No	No	No	No	XP_012574159.1	--	--	--
XLOC_003303	No	No	No	No	XP_012571719.1	--	OR	--
XLOC_003349	No	No	No	No	XP_004487947.1	--	R	K17710
XLOC_003470	No	No	No	No	XP_012572562.1	--	--	K10400
XLOC_003477	No	No	No	No	XP_012575045.1	--	X	K06617
XLOC_003569	No	No	No	No	XP_004496427.1	--	--	K09874
XLOC_003583	No	No	No	No	XP_004509079.1	--	--	--
XLOC_003772	No	No	No	No	--	--	--	--

Differentially expressed gene (DEG) IDs	*C. microphyllum* root and shoot-specific (>2 fold)	*C. microphyllum* root and shoot-specific (>10 fold)	ICC4958 and *C. microphyllum* root-specific (>2 fold)	*C. microphyllum* root-specific (>2 fold)	Functional Annotation			
					NCBI nr IDs	TFs	KOG	KEGG
XLOC_003817	No	No	No	No	XP_004486257.2	--	--	--
XLOC_003833	No	No	No	No	XP_004499349.1	--	--	--
XLOC_003839	No	No	No	No	XP_012571730.1	--	--	--
XLOC_003865	No	No	No	No	XP_004492382.1	--	--	--
XLOC_003889	No	No	No	No	XP_004510198.1	--	--	--
XLOC_003900	No	No	No	No	--	--	--	--
XLOC_004017	No	No	No	No	XP_004511809.1	--	--	--
XLOC_004033	No	No	No	No	XP_004497868.1	--	--	--
XLOC_004117	No	No	No	No	--	--	--	--
XLOC_004203	No	No	No	No	--	--	--	--
XLOC_004356	No	No	No	No	--	--	--	--
XLOC_004411	No	No	No	No	XP_004495644.1	--	--	--
XLOC_004473	No	No	No	No	--	--	--	--
XLOC_004534	No	No	No	No	XP_004497904.1	G2-like	--	--
XLOC_004676	No	No	No	No	--	--	--	--
XLOC_004687	No	No	No	No	--	--	--	--
XLOC_004819	No	No	No	No	XP_004489790.1	--	--	--
XLOC_004904	No	No	No	No	--	--	--	--
XLOC_004908	No	No	No	No	XP_004507747.1	--	--	--
XLOC_005004	No	No	No	No	XP_003627601.1	--	--	--
XLOC_005021	No	No	No	No	XP_012568083.1	--	--	K08900
XLOC_005025	No	No	No	No	--	--	--	--
XLOC_005031	No	No	No	No	--	--	--	--
XLOC_005036	No	No	No	No	XP_004494634.1	--	--	K10664
XLOC_005037	No	No	No	No	XP_004501681.1	--	--	K10664
XLOC_005038	No	No	No	No	XP_012569854.1	--	--	K10664
XLOC_005198	No	No	No	No	XP_004502947.1	--	--	--
XLOC_005220	No	No	No	No	--	--	--	--
XLOC_005221	No	No	No	No	--	--	--	--
XLOC_005250	No	No	No	No	XP_012569466.1	--	O	K01358
XLOC_005305	No	No	No	No	--	--	--	--
XLOC_005356	No	No	No	No	XP_004498796.1	--	R	--
XLOC_005582	No	No	No	No	XP_004487762.1	GRAS	--	K14494
XLOC_005783	No	No	No	No	--	--	--	--
XLOC_005789	No	No	No	No	--	--	--	--
XLOC_005817	No	No	No	No	--	--	--	--
XLOC_005837	No	No	No	No	XP_003591185.1	--	T	--
XLOC_005868	No	No	No	No	XP_004493643.1	--	--	K10523
XLOC_006174	No	No	No	No	XP_004509914.1	--	--	--
XLOC_006392	No	No	No	No	--	--	--	--

Differentially expressed gene (DEG) IDs	*C. microphyllum* root and shoot-specific (>2 fold)	*C. microphyllum* root and shoot-specific (>10 fold)	ICC4958 and *C. microphyllum* root-specific (>2 fold)	*C. microphyllum* root-specific (>2 fold)	Functional Annotation			
					NCBI nr IDs	TFs	KOG	KEGG
XLOC_006436	No	No	No	No	XP_004498688.1	--	O	--
XLOC_006604	No	No	No	No	XP_004501209.1	--	X	--
XLOC_006627	No	No	No	No	XP_004507338.1	FAR1	T	K13448
XLOC_006640	No	No	No	No	--	--	--	--
XLOC_006653	No	No	No	No	--	--	--	--
XLOC_006656	No	No	No	No	XP_004508564.1	--	MVG	--
XLOC_006690	No	No	No	No	--	--	--	--
XLOC_006691	No	No	No	No	XP_004497313.1	--	H	--
XLOC_006773	No	No	No	No	XP_004503163.1	--	R	K08081
XLOC_006779	No	No	No	No	--	--	--	--
XLOC_006813	No	No	No	No	--	--	--	--
XLOC_006850	No	No	No	No	XP_004500526.1	FAR1	--	--
XLOC_006878	No	No	No	No	CDW75723.1	--	--	--
XLOC_006879	No	No	No	No	CDW75723.1	--	--	--
XLOC_006895	No	No	No	No	XP_003637074.1	--	--	--
XLOC_006925	No	No	No	No	XP_004504562.1	C3H	C	K11517
XLOC_006940	No	No	No	No	XP_004486779.1	--	Q	--
XLOC_006959	No	No	No	No	XP_012575518.1	--	Q	K15095
XLOC_006960	No	No	No	No	--	--	--	--
XLOC_007100	No	No	No	No	--	--	--	--
XLOC_007121	No	No	No	No	XP_004512802.1	--	X	--
XLOC_007185	No	No	No	No	ABM53545.1	--	--	--
XLOC_007221	No	No	No	No	GAO47202.1	--	--	--
XLOC_007232	No	No	No	No	XP_013238305.1	--	--	--
XLOC_007307	No	No	No	No	--	--	--	--
XLOC_007440	No	No	No	No	XP_004515442.1	--	--	--
XLOC_007441	No	No	No	No	XP_004515442.1	--	--	--
XLOC_007444	No	No	No	No	XP_004515442.1	--	--	--
XLOC_007445	No	No	No	No	XP_004515442.1	--	--	--
XLOC_007464	No	No	No	No	XP_004495797.1	--	GM	--
XLOC_007497	No	No	No	No	XP_004503951.1	--	--	--
XLOC_007500	No	No	No	No	XP_004514831.1	--	--	--
XLOC_007516	No	No	No	No	XP_004506201.1	--	--	--
XLOC_007694	No	No	No	No	XP_004497441.1	--	T	K01102
XLOC_007712	No	No	No	No	XP_004489142.1	--	--	--
XLOC_007731	No	No	No	No	XP_004494863.1	--	V	--
XLOC_007742	No	No	No	No	--	--	--	--
XLOC_007748	No	No	No	No	XP_004489117.2	--	--	K19476
XLOC_007794	No	No	No	No	--	--	--	--
XLOC_007819	No	No	No	No	--	--	--	--

Differentially expressed gene (DEG) IDs	***C. microphyllum* root and shoot-specific (>2 fold)**	***C. microphyllum* root and shoot-specific (>10 fold)**	**ICC4958 and *C. microphyllum* root-specific (>2 fold)**	***C. microphyllum* root-specific (>2 fold)**	**Functional Annotation**			
					**NCBI nr IDs**	**TFs**	**KOG**	**KEGG**
XLOC_007861	No	No	No	No	XP_004501558.1	--	--	--
XLOC_007872	No	No	No	No	XP_004498912.1	TALE	--	--
XLOC_008136	No	No	No	No	XP_003618953.1	--	--	--
XLOC_008145	No	No	No	No	XP_004494704.1	--	R	--
XLOC_008189	No	No	No	No	XP_004516409.1	--	--	--
XLOC_008206	No	No	No	No	XP_004487697.1	--	--	--
XLOC_008311	No	No	No	No	XP_004498582.1	GRAS	P	--
XLOC_008313	No	No	No	No	ACC62036.1	GRAS	P	--
XLOC_008401	No	No	No	No	XP_004495876.1	--	--	--
XLOC_008402	No	No	No	No	XP_004495876.1	--	--	K00430
XLOC_008511	No	No	No	No	XP_012569077.1	--	--	--
XLOC_008583	No	No	No	No	XP_006597158.1	--	G	--
XLOC_008617	No	No	No	No	--	--	--	--
XLOC_008695	No	No	No	No	XP_004489241.1	bZIP	--	--
XLOC_008698	No	No	No	No	XP_004489240.1	--	V	--
XLOC_008705	No	No	No	No	--	--	--	--
XLOC_008712	No	No	No	No	--	--	--	K03116
XLOC_008755	No	No	No	No	YP_002149772.1	--	J	K02886
XLOC_008850	No	No	No	No	XP_004502348.1	--	--	K10251
XLOC_008959	No	No	No	No	--	--	--	--
XLOC_008967	No	No	No	No	XP_004505558.1	--	--	--
XLOC_009117	No	No	No	No	--	--	--	--
XLOC_009195	No	No	No	No	XP_004498409.1	--	--	--
XLOC_009205	No	No	No	No	XP_004493138.1	NAC	--	--
XLOC_009206	No	No	No	No	XP_004489720.1	NAC	S	--
XLOC_009424	No	No	No	No	XP_012569756.1	--	--	--
XLOC_009465	No	No	No	No	XP_004516865.1	--	--	--
XLOC_009527	No	No	No	No	--	--	--	--
XLOC_009566	No	No	No	No	--	--	--	--
XLOC_009582	No	No	No	No	XP_004504648.1	--	--	--
XLOC_009625	No	No	No	No	--	--	--	--
XLOC_009644	No	No	No	No	XP_004497331.1	Nin-like	Q	--
XLOC_009702	No	No	No	No	--	--	--	--
XLOC_009704	No	No	No	No	XP_012574657.1	--	--	--
XLOC_009783	No	No	No	No	--	--	--	--
XLOC_009939	No	No	No	No	--	--	--	--
XLOC_009973	No	No	No	No	XP_004489938.1	--	--	--
XLOC_009977	No	No	No	No	XP_004489938.1	--	--	--
XLOC_009994	No	No	No	No	XP_004513891.1	--	--	--
XLOC_010006	No	No	No	No	XP_004492060.1	--	--	--

**Differentially expressed gene (DEG) IDs**	***C. microphyllum* root and shoot-specific (>2 fold)**	***C. microphyllum* root and shoot-specific (>10 fold)**	**ICC4958 and *C. microphyllum* root-specific (>2 fold)**	***C. microphyllum* root-specific (>2 fold)**	**Functional Annotation**			
					**NCBI nr IDs**	**TFs**	**KOG**	**KEGG**
XLOC_010032	No	No	No	No	GAO47198.1	--	--	--
XLOC_010070	No	No	No	No	XP_004500734.1	--	GM	--
XLOC_010074	No	No	No	No	XP_012569970.1	--	--	K00430
XLOC_010083	No	No	No	No	XP_012569976.1	--	--	K00430
XLOC_010208	No	No	No	No	CBA31922.1	--	--	--
XLOC_010226	No	No	No	No	XP_004490623.1	--	--	--
XLOC_010257	No	No	No	No	--	--	--	K13993
XLOC_010293	No	No	No	No	XP_004485609.1	--	--	K01673
XLOC_010327	No	No	No	No	XP_004500969.1	--	--	--
XLOC_010378	No	No	No	No	--	--	--	--
XLOC_010390	No	No	No	No	XP_004503662.1	--	--	--
XLOC_010416	No	No	No	No	XP_004511008.1	--	--	--
XLOC_010434	No	No	No	No	XP_004495322.1	--	--	--
XLOC_010435	No	No	No	No	XP_004495322.1	--	P	--
XLOC_010472	No	No	No	No	XP_004489250.1	--	GE	K15283
XLOC_010473	No	No	No	No	XP_004504803.1	--	--	--
XLOC_010534	No	No	No	No	XP_004495205.1	--	--	--
XLOC_010545	No	No	No	No	XP_012575034.1	--	--	--
XLOC_010578	No	No	No	No	XP_004498005.1	--	T	--
XLOC_010690	No	No	No	No	--	--	--	--
XLOC_010759	No	No	No	No	XP_004490092.1	--	S	--
XLOC_010764	No	No	No	No	--	--	--	--
XLOC_010815	No	No	No	No	--	--	--	--
XLOC_010832	No	No	No	No	XP_004515645.1	--	P	--
XLOC_010934	No	No	No	No	XP_004504935.1	--	--	--
XLOC_010949	No	No	No	No	--	--	--	--
XLOC_010996	No	No	No	No	--	--	--	--
XLOC_011108	No	No	No	No	CAN79196.1	--	--	--
XLOC_011121	No	No	No	No	XP_004501462.1	--	Q	--
XLOC_011123	No	No	No	No	XP_004495587.1	--	--	--
XLOC_011295	No	No	No	No	--	--	--	--
XLOC_011347	No	No	No	No	--	--	--	--
XLOC_011354	No	No	No	No	XP_004501679.1	--	R	K03327
XLOC_011405	No	No	No	No	XP_004501013.1	--	--	--
XLOC_011455	No	No	No	No	--	--	--	--
XLOC_011499	No	No	No	No	--	--	--	--
XLOC_011523	No	No	No	No	XP_004488942.1	--	--	--
XLOC_011564	No	No	No	No	XP_004505951.1	--	--	--
XLOC_011588	No	No	No	No	XP_003608123.1	--	--	--
XLOC_011598	No	No	No	No	XP_004507178.1	GRAS	--	--

**Differentially expressed gene (DEG) IDs**	***C. microphyllum* root and shoot-specific (>2 fold)**	***C. microphyllum* root and shoot-specific (>10 fold)**	**ICC4958 and *C. microphyllum* root-specific (>2 fold)**	***C. microphyllum* root-specific (>2 fold)**	**Functional Annotation**			
					**NCBI nr IDs**	**TFs**	**KOG**	**KEGG**
XLOC_011619	No	No	No	No	--	--	--	--
XLOC_011620	No	No	No	No	XP_012574860.1	--	T	--
XLOC_011626	No	No	No	No	XP_003628047.1	--	--	--
XLOC_011776	No	No	No	No	XP_004510440.1	--	--	--
XLOC_012036	No	No	No	No	XP_004503953.1	Trihelix	F	K09903
XLOC_012067	No	No	No	No	XP_004510293.1	--	--	--
XLOC_012121	No	No	No	No	--	--	--	--
XLOC_012125	No	No	No	No	--	--	--	--
XLOC_012128	No	No	No	No	XP_004507880.1	--	Z	K05768
XLOC_012171	No	No	No	No	XP_004511534.1	bZIP	G	--
XLOC_012191	No	No	No	No	XP_004493898.1	--	R	--
XLOC_012228	No	No	No	No	XP_004507478.1	--	T	K00924
XLOC_012251	No	No	No	No	XP_004502939.1	--	--	--
XLOC_012280	No	No	No	No	--	--	--	--
XLOC_012305	No	No	No	No	XP_004499155.1	--	F	--
XLOC_012340	No	No	No	No	XP_004512942.1	--	--	--
XLOC_012384	No	No	No	No	XP_004496194.1	--	--	--
XLOC_012401	No	No	No	No	--	--	--	--
XLOC_012406	No	No	No	No	XP_004491022.1	LBD	--	--
XLOC_012427	No	No	No	No	XP_012571385.1	--	I	--
XLOC_012431	No	No	No	No	XP_012572390.1	--	R	--
XLOC_012432	No	No	No	No	XP_012572390.1	--	R	--
XLOC_012499	No	No	No	No	XP_004485615.1	--	--	--
XLOC_012522	No	No	No	No	XP_004496328.1	--	--	--
XLOC_012532	No	No	No	No	--	--	--	--
XLOC_012541	No	No	No	No	XP_012567536.1	--	--	--
XLOC_012553	No	No	No	No	XP_004502597.1	--	--	--
XLOC_012571	No	No	No	No	XP_004500800.1	--	--	K08241
XLOC_012673	No	No	No	No	--	--	--	--
XLOC_012748	No	No	No	No	XP_004493972.1	--	G	K09872
XLOC_012794	No	No	No	No	XP_012571142.1	--	R	--
XLOC_012964	No	No	No	No	--	--	--	--
XLOC_013097	No	No	No	No	--	--	--	--
XLOC_013188	No	No	No	No	--	--	--	--
XLOC_013213	No	No	No	No	XP_004489344.1	--	E	K14638
XLOC_013226	No	No	No	No	XP_004503738.1	--	--	--
XLOC_013245	No	No	No	No	XP_004485745.1	--	--	--
XLOC_013299	No	No	No	No	XP_006965400.1	--	--	--
XLOC_013329	No	No	No	No	--	--	--	--
XLOC_013441	No	No	No	No	XP_004494858.1	--	I	--

Differentially expressed gene (DEG) IDs	*C. microphyllum* root and shoot-specific (>2 fold)	*C. microphyllum* root and shoot-specific (>10 fold)	ICC4958 and *C. microphyllum* root-specific (>2 fold)	*C. microphyllum* root-specific (>2 fold)	Functional Annotation			
					NCBI nr IDs	TFs	KOG	KEGG
XLOC_013577	No	No	No	No	XP_004497247.1	--	--	--
XLOC_013676	No	No	No	No	XP_004516199.1	--	--	--
XLOC_013725	No	No	No	No	XP_004486779.1	--	Q	--
XLOC_013756	No	No	No	No	XP_004497411.1	--	--	--
XLOC_013793	No	No	No	No	--	--	--	K03809
XLOC_013930	No	No	No	No	--	--	--	--
XLOC_013985	No	No	No	No	XP_004507994.1	--	--	K15082
XLOC_013986	No	No	No	No	XP_004507994.1	--	--	K15082
XLOC_014034	No	No	No	No	--	--	--	--
XLOC_014083	No	No	No	No	XP_004511004.1	--	--	--
XLOC_014091	No	No	No	No	--	--	--	--
XLOC_014214	No	No	No	No	XP_004486138.1	--	S	--
XLOC_014220	No	No	No	No	XP_004497022.1	--	--	--
XLOC_014429	No	No	No	No	XP_004492093.1	C2H2	--	--
XLOC_014431	No	No	No	No	--	--	--	--
XLOC_014464	No	No	No	No	XP_004495900.1	--	--	--
XLOC_014550	No	No	No	No	--	--	--	--
XLOC_014566	No	No	No	No	XP_004507302.1	--	--	--
XLOC_014715	No	No	No	No	--	--	--	--
XLOC_014743	No	No	No	No	XP_012574125.1	B3	--	--
XLOC_014890	No	No	No	No	XP_004492759.1	--	--	--
XLOC_014898	No	No	No	No	XP_004504219.1	--	--	--
XLOC_014922	No	No	No	No	--	--	--	--
XLOC_014934	No	No	No	No	XP_012567618.1	--	--	--
XLOC_015119	No	No	No	No	--	--	--	--
XLOC_015134	No	No	No	No	XP_004516636.1	--	KR	K15032
XLOC_015153	No	No	No	No	--	--	--	--
XLOC_015189	No	No	No	No	XP_012569543.1	WRKY	--	--
XLOC_015249	No	No	No	No	XP_004504503.1	--	--	--
XLOC_015304	No	No	No	No	XP_004499461.1	--	--	--
XLOC_015331	No	No	No	No	--	--	--	--
XLOC_015336	No	No	No	No	--	--	--	--
XLOC_015374	No	No	No	No	XP_012571597.1	--	--	K00430
XLOC_015397	No	No	No	No	XP_012570815.1	--	R	K03327
XLOC_015451	No	No	No	No	XP_004514405.1	--	--	--
XLOC_015452	No	No	No	No	XP_004494321.1	--	--	K11816
XLOC_015463	No	No	No	No	XP_004501430.1	--	--	K02221
XLOC_015473	No	No	No	No	--	--	--	--
XLOC_015483	No	No	No	No	XP_004487568.1	G2-like	--	--
XLOC_015591	No	No	No	No	XP_004487335.1	--	X	--

**Differentially expressed gene (DEG) IDs**	***C. microphyllum* root and shoot-specific (>2 fold)**	***C. microphyllum* root and shoot-specific (>10 fold)**	**ICC4958 and *C. microphyllum* root-specific (>2 fold)**	***C. microphyllum* root-specific (>2 fold)**	**Functional Annotation**			
					**NCBI nr IDs**	**TFs**	**KOG**	**KEGG**
XLOC_015615	No	No	No	No	XP_012571346.1	--	--	--
XLOC_015671	No	No	No	No	XP_004515986.1	--	--	--
XLOC_015697	No	No	No	No	XP_004492252.1	--	Z	K17275
XLOC_015738	No	No	No	No	XP_004494108.1	--	--	--
XLOC_015739	No	No	No	No	XP_004512144.1	--	--	--
XLOC_015831	No	No	No	No	--	--	--	--
XLOC_015996	No	No	No	No	XP_012569979.1	--	--	--
XLOC_016016	No	No	No	No	--	--	--	--
XLOC_016042	No	No	No	No	XP_004499718.1	--	G	--
XLOC_016056	No	No	No	No	XP_004501377.1	--	S	--
XLOC_016148	No	No	No	No	XP_012573720.1	--	G	--
XLOC_016192	No	No	No	No	XP_004496625.1	--	--	--
XLOC_016239	No	No	No	No	XP_004495537.1	C3H	--	--
XLOC_016274	No	No	No	No	ADI72890.1	--	--	--
XLOC_016276	No	No	No	No	ADI72890.1	--	--	--
XLOC_016281	No	No	No	No	XP_004493628.1	--	V	K14493
XLOC_016373	No	No	No	No	XP_004491415.1	--	--	--
XLOC_016441	No	No	No	No	XP_012573992.1	WRKY	--	--
XLOC_016535	No	No	No	No	KEH33664.1	G2-like	--	--
XLOC_016580	No	No	No	No	XP_004507406.1	--	I	K13513
XLOC_016653	No	No	No	No	XP_004502038.1	--	G	--
XLOC_016700	No	No	No	No	XP_004498200.1	--	--	--
XLOC_016705	No	No	No	No	--	--	--	--
XLOC_016793	No	No	No	No	XP_004494859.1	--	--	--
XLOC_016798	No	No	No	No	XP_004490927.1	--	--	--
XLOC_016881	No	No	No	No	XP_004514723.1	bZIP	--	--
XLOC_016984	No	No	No	No	XP_004498594.1	--	--	K00783
XLOC_017005	No	No	No	No	XP_004500806.1	--	RQ	--
XLOC_017048	No	No	No	No	XP_004491894.1	--	X	--
XLOC_017164	No	No	No	No	--	--	--	--
XLOC_017455	No	No	No	No	XP_004516301.1	--	--	--
XLOC_017476	No	No	No	No	XP_004511300.1	--	--	--
XLOC_017506	No	No	No	No	XP_004490894.1	--	--	--
XLOC_017509	No	No	No	No	XP_004501327.1	ERF	--	--
XLOC_017554	No	No	No	No	--	--	--	K01807
XLOC_017581	No	No	No	No	--	--	--	--
XLOC_017587	No	No	No	No	--	--	--	--
XLOC_017620	No	No	No	No	XP_004516029.1	--	--	--
XLOC_017658	No	No	No	No	--	--	--	--
XLOC_017840	No	No	No	No	XP_004511937.1	--	--	K13448

**Differentially expressed gene (DEG) IDs**	***C. microphyllum* root and shoot-specific (>2 fold)**	***C. microphyllum* root and shoot-specific (>10 fold)**	**ICC4958 and *C. microphyllum* root-specific (>2 fold)**	***C. microphyllum* root-specific (>2 fold)**	**Functional Annotation**			
					**NCBI nr IDs**	**TFs**	**KOG**	**KEGG**
XLOC_017844	No	No	No	No	--	--	--	--
XLOC_017928	No	No	No	No	XP_004502947.1	--	Q	--
XLOC_017930	No	No	No	No	XP_012571312.1	--	T	--
XLOC_017931	No	No	No	No	--	--	--	--
XLOC_017992	No	No	No	No	XP_004497332.1	--	--	--
XLOC_017999	No	No	No	No	--	--	--	--
XLOC_018064	No	No	No	No	XP_004515713.1	--	R	--
XLOC_018153	No	No	No	No	--	--	--	K03043
XLOC_018154	No	No	No	No	XP_004501838.1	--	--	--
XLOC_018192	No	No	No	No	--	--	--	--
XLOC_018195	No	No	No	No	XP_004508628.1	ERF	--	--
XLOC_018210	No	No	No	No	XP_004501594.1	--	S	--
XLOC_018232	No	No	No	No	--	--	--	--
XLOC_018244	No	No	No	No	XP_004488786.1	--	IR	--
XLOC_018337	No	No	No	No	--	--	--	--
XLOC_018344	No	No	No	No	--	--	--	--
XLOC_018401	No	No	No	No	--	--	--	--
XLOC_018555	No	No	No	No	XP_004485853.1	--	--	--
XLOC_018575	No	No	No	No	XP_004492195.1	--	--	K08237
XLOC_018610	No	No	No	No	XP_004504140.1	E2F/DP	T	--
XLOC_018612	No	No	No	No	XP_004504141.1	--	--	--
XLOC_018631	No	No	No	No	--	--	--	--
XLOC_018685	No	No	No	No	XP_004504243.1	--	--	--
XLOC_018873	No	No	No	No	XP_004485861.1	--	--	--
XLOC_018895	No	No	No	No	--	--	--	--
XLOC_018933	No	No	No	No	XP_004487949.1	--	--	--
XLOC_018971	No	No	No	No	NP_001266157.1	--	--	--
XLOC_019055	No	No	No	No	XP_012570399.1	--	--	--
XLOC_019069	No	No	No	No	XP_004509535.1	--	G	--
XLOC_019098	No	No	No	No	XP_004500543.1	--	E	K14638
XLOC_019144	No	No	No	No	--	--	--	--
XLOC_019191	No	No	No	No	AES69823.2	--	C	--
XLOC_019271	No	No	No	No	XP_004489691.1	ERF	--	K09286
XLOC_019311	No	No	No	No	XP_004513904.1	--	X	K13508
XLOC_019362	No	No	No	No	--	--	--	--
XLOC_019459	No	No	No	No	XP_004506726.1	--	EH	K01568
XLOC_019489	No	No	No	No	--	--	--	--
XLOC_019554	No	No	No	No	XP_004513824.1	--	--	K15095
XLOC_019561	No	No	No	No	XP_004496174.1	--	G	--
XLOC_019618	No	No	No	No	--	--	--	--

Differentially expressed gene (DEG) IDs	*C. microphyllum* root and shoot-specific (>2 fold)	*C. microphyllum* root and shoot-specific (>10 fold)	ICC4958 and *C. microphyllum* root-specific (>2 fold)	*C. microphyllum* root-specific (>2 fold)	Functional Annotation			
					NCBI nr IDs	TFs	KOG	KEGG
XLOC_019757	No	No	No	No	XP_004514688.1	--	X	K00891
XLOC_020024	No	No	No	No	AES98640.2	--	--	--
XLOC_020075	No	No	No	No	XP_004490711.1	--	R	K08867
XLOC_020086	No	No	No	No	--	--	--	K02996
XLOC_020124	No	No	No	No	XP_004504562.1	--	C	--
XLOC_020146	No	No	No	No	XP_012573142.1	NAC	G	--
XLOC_020283	No	No	No	No	XP_004499610.1	--	--	--
XLOC_020299	No	No	No	No	XP_004493247.1	--	--	K01738
XLOC_020314	No	No	No	No	XP_004501574.1	--	--	K14499
XLOC_020385	No	No	No	No	XP_004487428.1	--	--	--
XLOC_020486	No	No	No	No	--	--	--	--
XLOC_020529	No	No	No	No	XP_012572063.1	--	--	--
XLOC_020604	No	No	No	No	XP_004501179.1	--	--	--
XLOC_020699	No	No	No	No	XP_004485562.1	--	--	--
XLOC_020735	No	No	No	No	--	--	--	--
XLOC_020828	No	No	No	No	XP_004490507.1	--	--	--
XLOC_020845	No	No	No	No	XP_004498345.1	--	--	K13600
XLOC_020858	No	No	No	No	--	--	--	K14492
XLOC_020883	No	No	No	No	XP_004496540.1	--	--	--
XLOC_021123	No	No	No	No	--	--	--	--
XLOC_021138	No	No	No	No	--	--	--	--
XLOC_021197	No	No	No	No	XP_004509745.1	--	--	--
XLOC_021211	No	No	No	No	XP_004504632.1	--	T	--
XLOC_021335	No	No	No	No	XP_004487192.2	C2H2	--	--
XLOC_021345	No	No	No	No	XP_004493043.1	--	--	K15032
XLOC_021377	No	No	No	No	XP_004492380.2	--	--	--
XLOC_021400	No	No	No	No	XP_004510819.1	--	P	--
XLOC_021401	No	No	No	No	XP_004510819.1	--	--	--
XLOC_021450	No	No	No	No	XP_012570069.1	--	G	--
XLOC_021460	No	No	No	No	XP_004494320.1	--	--	--
XLOC_021542	No	No	No	No	XP_004490661.1	--	Q	K05909
XLOC_021647	No	No	No	No	XP_004501141.1	--	--	--
XLOC_021664	No	No	No	No	XP_004488124.1	--	--	--
XLOC_021667	No	No	No	No	XP_004513629.1	--	C	K00387
XLOC_021778	No	No	No	No	--	--	--	--
XLOC_021851	No	No	No	No	XP_004495957.1	--	--	--
XLOC_021890	No	No	No	No	XP_004489463.1	--	--	--
XLOC_021917	No	No	No	No	--	--	--	--
XLOC_021956	No	No	No	No	XP_004504756.1	--	--	--
XLOC_022199	No	No	No	No	XP_004510788.1	--	QR	--

Differentially expressed gene (DEG) IDs	*C. microphyllum* root and shoot-specific (>2 fold)	*C. microphyllum* root and shoot-specific (>10 fold)	ICC4958 and *C. microphyllum* root-specific (>2 fold)	*C. microphyllum* root-specific (>2 fold)	Functional Annotation			
					NCBI nr IDs	TFs	KOG	KEGG
XLOC_022291	No	No	No	No	XP_004508669.1	--	--	--
XLOC_022309	No	No	No	No	XP_004487677.1	--	--	--
XLOC_022313	No	No	No	No	XP_004489319.1	--	R	K10529
XLOC_022411	No	No	No	No	--	--	--	--
XLOC_022487	No	No	No	No	--	--	--	--
XLOC_022493	No	No	No	No	XP_004503338.1	--	S	--
XLOC_022510	No	No	No	No	--	--	--	--
XLOC_022535	No	No	No	No	XP_004491028.1	--	--	--
XLOC_022600	No	No	No	No	XP_004492492.1	--	P	K00522
XLOC_022630	No	No	No	No	--	--	--	--
XLOC_022634	No	No	No	No	--	--	--	--
XLOC_022719	No	No	No	No	XP_004514326.1	RAV	O	K01265
XLOC_022732	No	No	No	No	--	--	--	--
XLOC_022745	No	No	No	No	--	--	--	--
XLOC_022782	No	No	No	No	XP_004486939.1	WRKY	--	--
XLOC_022815	No	No	No	No	--	--	--	K02639
XLOC_022894	No	No	No	No	XP_012568432.1	--	P	K07300
XLOC_023016	No	No	No	No	XP_004515876.1	--	X	--
XLOC_023039	No	No	No	No	XP_004486529.1	--	--	--
XLOC_023065	No	No	No	No	XP_004492729.1	--	--	--
XLOC_023067	No	No	No	No	XP_012569365.1	MYB	--	K09422
XLOC_023138	No	No	No	No	XP_004511690.1	--	--	--
XLOC_023176	No	No	No	No	XP_004512650.1	--	--	--
XLOC_023178	No	No	No	No	XP_004487977.1	--	--	--
XLOC_023184	No	No	No	No	--	--	--	--
XLOC_023220	No	No	No	No	XP_012570400.1	--	--	--
XLOC_023265	No	No	No	No	XP_004506125.1	--	E	--
XLOC_023336	No	No	No	No	XP_004500365.1	--	--	K07437
XLOC_023361	No	No	No	No	--	--	--	--
XLOC_023368	No	No	No	No	XP_004487449.1	--	--	--
XLOC_023458	No	No	No	No	XP_004488736.1	SBP	--	--
XLOC_023597	No	No	No	No	XP_003610971.1	--	X	K03404
XLOC_023605	No	No	No	No	XP_004504827.1	--	--	--
XLOC_023606	No	No	No	No	XP_004504827.1	--	--	--
XLOC_023682	No	No	No	No	XP_004497360.1	--	--	--
XLOC_023696	No	No	No	No	XP_004500613.1	--	--	--
XLOC_023729	No	No	No	No	XP_004512013.1	HD-ZIP	--	K09338
XLOC_023875	No	No	No	No	XP_012573641.1	--	--	--
XLOC_023920	No	No	No	No	--	--	--	--
XLOC_023922	No	No	No	No	XP_004510689.1	--	T	K01762

Differentially expressed gene (DEG) IDs	*C. microphyllum* root and shoot-specific (>2 fold)	*C. microphyllum* root and shoot-specific (>10 fold)	ICC4958 and *C. microphyllum* root-specific (>2 fold)	*C. microphyllum* root-specific (>2 fold)	Functional Annotation			
					NCBI nr IDs	TFs	KOG	KEGG
XLOC_023949	No	No	No	No	XP_004516616.1	--	--	--
XLOC_023977	No	No	No	No	XP_004488829.1	--	--	--
XLOC_024054	No	No	No	No	KEH40040.1	--	--	--
XLOC_024068	No	No	No	No	XP_004489330.1	--	--	--
XLOC_024072	No	No	No	No	XP_004501854.1	--	--	--
XLOC_024099	No	No	No	No	XP_004512519.1	--	--	--
XLOC_024152	No	No	No	No	--	--	--	--
XLOC_024288	No	No	No	No	AJA74416.1	--	O	--
XLOC_024293	No	No	No	No	EGZ74191.1	--	--	--
XLOC_024346	No	No	No	No	KIM19341.1	--	--	--
XLOC_024381	No	No	No	No	XP_004493694.1	--	U	--
XLOC_024382	No	No	No	No	XP_004511566.1	--	--	--
XLOC_024474	No	No	No	No	XP_004493454.1	--	C	--
XLOC_024526	No	No	No	No	--	--	--	--
XLOC_024579	No	No	No	No	XP_004509358.1	--	QR	--
XLOC_024580	No	No	No	No	XP_004509358.1	--	QR	K00475
XLOC_024596	No	No	No	No	--	--	--	--
XLOC_024716	No	No	No	No	XP_004495104.1	ERF	--	K14516
XLOC_024746	No	No	No	No	XP_012570176.1	HD-ZIP	K	--
XLOC_024747	No	No	No	No	XP_004510857.1	HD-ZIP	K	--
XLOC_024772	No	No	No	No	--	--	--	--
XLOC_024818	No	No	No	No	XP_012574456.1	--	TZ	--
XLOC_024839	No	No	No	No	XP_004487366.1	--	TZ	--
XLOC_024921	No	No	No	No	XP_004495876.1	--	--	--
XLOC_024966	No	No	No	No	--	--	--	--
XLOC_025128	No	No	No	No	--	--	--	--
XLOC_025133	No	No	No	No	XP_004497839.1	ERF	K	K14516
XLOC_025235	No	No	No	No	--	--	--	K14496
XLOC_025251	No	No	No	No	XP_004506641.1	--	G	--
XLOC_025360	No	No	No	No	--	--	--	--
XLOC_025375	No	No	No	No	XP_004506281.1	--	--	--
XLOC_025403	No	No	No	No	--	--	--	--
XLOC_025407	No	No	No	No	--	--	--	--
XLOC_025440	No	No	No	No	--	--	--	--
XLOC_025451	No	No	No	No	--	--	--	--
XLOC_025459	No	No	No	No	--	--	--	--
XLOC_025470	No	No	No	No	XP_004496370.1	FAR1	T	K13412
XLOC_025472	No	No	No	No	XP_004491961.1	--	--	--
XLOC_025512	No	No	No	No	YP_002149755.1	--	--	K02963
XLOC_025515	No	No	No	No	XP_004504032.1	--	R	--

**Differentially expressed gene (DEG) IDs**	***C. microphyllum* root and shoot-specific (>2 fold)**	***C. microphyllum* root and shoot-specific (>10 fold)**	**ICC4958 and *C. microphyllum* root-specific (>2 fold)**	***C. microphyllum* root-specific (>2 fold)**	**Functional Annotation**			
					**NCBI nr IDs**	**TFs**	**KOG**	**KEGG**
XLOC_025532	No	No	No	No	XP_004489494.1	ERF	--	--
XLOC_025535	No	No	No	No	XP_004501930.1	--	--	--
XLOC_025565	No	No	No	No	--	--	--	--
XLOC_025577	No	No	No	No	XP_004487168.1	--	--	--
XLOC_025717	No	No	No	No	XP_004496291.1	--	S	--
XLOC_025768	No	No	No	No	XP_004495744.1	--	T	--
XLOC_025913	No	No	No	No	XP_004489801.1	TALE	H	K03183
XLOC_025922	No	No	No	No	XP_004486794.1	--	RI	--
XLOC_026007	No	No	No	No	XP_004504475.1	--	--	--
XLOC_026081	No	No	No	No	XP_004510634.1	--	O	K08900
XLOC_026083	No	No	No	No	--	--	--	--
XLOC_026118	No	No	No	No	--	--	--	--
XLOC_026160	No	No	No	No	XP_004490093.1	NAC	S	--
XLOC_026161	No	No	No	No	--	--	--	--
XLOC_026164	No	No	No	No	WP_016831114.1	--	--	--
XLOC_026178	No	No	No	No	--	--	--	--
XLOC_026217	No	No	No	No	XP_004495431.1	--	T	--
XLOC_026307	No	No	No	No	XP_004499889.1	--	--	--
XLOC_026349	No	No	No	No	XP_004485681.1	--	--	--
XLOC_026350	No	No	No	No	XP_004504260.1	--	--	--
XLOC_026529	No	No	No	No	XP_004514578.1	--	T	K04733
XLOC_026580	No	No	No	No	--	--	--	--
XLOC_026638	No	No	No	No	--	--	--	--
XLOC_026748	No	No	No	No	XP_004495509.1	--	--	--
XLOC_026824	No	No	No	No	--	--	--	--
XLOC_026830	No	No	No	No	--	--	--	--
XLOC_026906	No	No	No	No	XP_004498046.1	--	C	K00006
XLOC_026907	No	No	No	No	XP_004493461.1	--	C	K00006
XLOC_026930	No	No	No	No	AIQ78384.1	--	--	--
XLOC_026946	No	No	No	No	XP_003549550.1	bHLH	C	K00131
XLOC_026967	No	No	No	No	--	--	--	--
XLOC_026991	No	No	No	No	XP_004492963.1	--	--	--
XLOC_027110	No	No	No	No	XP_004485865.1	--	R	--
XLOC_027145	No	No	No	No	--	--	--	--
XLOC_027171	No	No	No	No	XP_004492137.1	--	--	--
XLOC_027194	No	No	No	No	XP_004494100.1	--	FH	K03457
XLOC_027284	No	No	No	No	XP_004513770.1	--	T	K13412
XLOC_027302	No	No	No	No	--	--	--	--
XLOC_027328	No	No	No	No	--	--	--	--
XLOC_027398	No	No	No	No	--	--	--	--

**Differentially expressed gene (DEG) IDs**	***C. microphyllum* root and shoot-specific (>2 fold)**	***C. microphyllum* root and shoot-specific (>10 fold)**	**ICC4958 and *C. microphyllum* root-specific (>2 fold)**	***C. microphyllum* root-specific (>2 fold)**	**Functional Annotation**			
					**NCBI nr IDs**	**TFs**	**KOG**	**KEGG**
XLOC_027433	No	No	No	No	XP_004503443.1	--	--	K03100
XLOC_027611	No	No	No	No	XP_004495614.1	--	E	--
XLOC_027626	No	No	No	No	XP_012567416.1	--	--	--
XLOC_027630	No	No	No	No	--	--	--	--
XLOC_027631	No	No	No	No	XP_004506588.1	--	U	K08511
XLOC_027700	No	No	No	No	XP_004503320.1	--	--	--
XLOC_027712	No	No	No	No	XP_004514689.1	--	--	--
XLOC_027864	No	No	No	No	XP_004502982.1	--	--	--
XLOC_027867	No	No	No	No	--	--	--	--
XLOC_027899	No	No	No	No	XP_004507453.1	bHLH	--	--
XLOC_027944	No	No	No	No	--	--	--	--
XLOC_027959	No	No	No	No	XP_004517160.1	--	A	K12581
XLOC_028030	No	No	No	No	ACJ85012.1	--	--	--
XLOC_028083	No	No	No	No	XP_004488850.1	--	T	--
XLOC_028140	No	No	No	No	XP_004490028.1	--	--	--
XLOC_028221	No	No	No	No	XP_012570621.1	--	--	--
XLOC_028361	No	No	No	No	XP_004503625.1	--	I	--
XLOC_028411	No	No	No	No	XP_003612829.1	--	--	--
XLOC_028449	No	No	No	No	--	--	--	--
XLOC_028450	No	No	No	No	XP_004494259.1	NF-YA	--	K08064
XLOC_028451	No	No	No	No	XP_004502972.1	--	--	--
XLOC_028480	No	No	No	No	XP_004506047.1	--	O	--
XLOC_028583	No	No	No	No	--	--	--	--
XLOC_028586	No	No	No	No	XP_004501676.1	--	--	--
XLOC_028587	No	No	No	No	--	--	--	--
XLOC_028699	No	No	No	No	XP_004513802.1	M-type	--	K03216
XLOC_028768	No	No	No	No	XP_004505268.1	--	OE	K09645
XLOC_028851	No	No	No	No	--	--	--	--
XLOC_028852	No	No	No	No	XP_004514695.1	--	V	--
XLOC_028868	No	No	No	No	--	--	--	--
XLOC_029009	No	No	No	No	KEH31612.1	--	--	--
XLOC_029027	No	No	No	No	--	--	--	K02986
XLOC_029042	No	No	No	No	KEH40722.1	--	--	--
XLOC_029067	No	No	No	No	--	--	--	--
XLOC_029107	No	No	No	No	--	--	--	--
XLOC_029169	No	No	No	No	--	--	--	K14488
XLOC_029213	No	No	No	No	--	--	--	--
XLOC_029265	No	No	No	No	--	--	--	--
XLOC_029302	No	No	No	No	--	--	--	--
XLOC_029317	No	No	No	No	--	--	--	K19306

Differentially expressed gene (DEG) IDs	*C. microphyllum* root and shoot-specific (>2 fold)	*C. microphyllum* root and shoot-specific (>10 fold)	ICC4958 and *C. microphyllum* root-specific (>2 fold)	*C. microphyllum* root-specific (>2 fold)	Functional Annotation			
					NCBI nr IDs	TFs	KOG	KEGG
XLOC_029446	No	No	No	No	XP_004512062.1	--	--	--
XLOC_029448	No	No	No	No	XP_004487895.1	--	--	--
XLOC_029527	No	No	No	No	--	--	--	--
XLOC_029538	No	No	No	No	XP_004488592.1	--	--	--
XLOC_029617	No	No	No	No	XP_004490618.1	--	--	--
XLOC_029685	No	No	No	No	--	--	--	--
XLOC_029720	No	No	No	No	--	--	--	--
XLOC_029757	No	No	No	No	XP_004489859.1	--	--	--
XLOC_029781	No	No	No	No	XP_004497538.1	--	--	K01904
XLOC_029828	No	No	No	No	XP_004487582.1	WRKY	--	--
XLOC_029852	No	No	No	No	XP_004511863.1	--	--	--
XLOC_029891	No	No	No	No	XP_003611848.1	--	--	--
XLOC_029902	No	No	No	No	--	--	--	K04078
XLOC_029989	No	No	No	No	WP_042854931.1	--	C	K02689
XLOC_030060	No	No	No	No	XP_004508379.1	--	--	--
XLOC_030131	No	No	No	No	--	--	--	--
XLOC_030142	No	No	No	No	XP_004499303.1	--	M	--
XLOC_030185	No	No	No	No	--	--	--	--
XLOC_030517	No	No	No	No	--	--	--	--
XLOC_030602	No	No	No	No	--	--	--	--
XLOC_030689	No	No	No	No	--	--	--	--
XLOC_030705	No	No	No	No	XP_012568472.1	--	--	--
XLOC_030737	No	No	No	No	XP_004485917.1	bZIP	MG	K10999
XLOC_030795	No	No	No	No	XP_004496557.1	--	--	K01280
XLOC_030796	No	No	No	No	XP_004496557.1	--	O	K01280
XLOC_030826	No	No	No	No	XP_004485681.1	--	--	--
XLOC_030931	No	No	No	No	XP_004501558.1	--	--	--
XLOC_030939	No	No	No	No	XP_004485623.1	MIKC	K	K09264
XLOC_031067	No	No	No	No	--	--	--	--
XLOC_031087	No	No	No	No	XP_004513876.1	--	--	--
XLOC_031092	No	No	No	No	--	--	--	--
XLOC_031135	No	No	No	No	XP_012571135.1	MYB	R	--
XLOC_031214	No	No	No	No	--	--	--	--
XLOC_031234	No	No	No	No	XP_004491439.1	--	--	--
XLOC_031243	No	No	No	No	XP_012568549.1	--	--	--
XLOC_031355	No	No	No	No	--	--	--	--
XLOC_031356	No	No	No	No	XP_012574030.1	--	MVG	--
XLOC_031428	No	No	No	No	XP_004498466.1	ERF	--	K09286
XLOC_031511	No	No	No	No	XP_004493756.1	--	--	K13407
XLOC_031522	No	No	No	No	XP_004486518.1	--	--	--

Differentially expressed gene (DEG) IDs	*C. microphyllum* root and shoot-specific (>2 fold)	*C. microphyllum* root and shoot-specific (>10 fold)	ICC4958 and *C. microphyllum* root-specific (>2 fold)	*C. microphyllum* root-specific (>2 fold)	Functional Annotation			
					NCBI nr IDs	TFs	KOG	KEGG
XLOC_031623	No	No	No	No	XP_004495401.1	--	--	--
XLOC_031647	No	No	No	No	XP_004504393.1	--	--	K12462
XLOC_031684	No	No	No	No	XP_004503625.1	--	I	K01897
XLOC_031786	No	No	No	No	--	--	--	--
XLOC_031836	No	No	No	No	--	--	--	--
XLOC_031841	No	No	No	No	XP_004504592.1	--	O	--
XLOC_031847	No	No	No	No	ABY91221.1	--	--	--
XLOC_031901	No	No	No	No	XP_004489337.1	--	--	--
XLOC_031912	No	No	No	No	--	--	--	--
XLOC_031925	No	No	No	No	XP_012573917.1	--	--	--
XLOC_031955	No	No	No	No	--	--	--	--
XLOC_031962	No	No	No	No	XP_004511392.1	--	--	--
XLOC_032057	No	No	No	No	XP_004516682.1	--	--	--
XLOC_032105	No	No	No	No	--	--	--	--
XLOC_032156	No	No	No	No	--	--	--	--
XLOC_032227	No	No	No	No	--	--	--	--
XLOC_032237	No	No	No	No	XP_004497425.1	--	--	K03768
XLOC_032273	No	No	No	No	XP_004513852.1	--	--	--
XLOC_032327	No	No	No	No	--	--	--	--
XLOC_032335	No	No	No	No	--	--	--	--
XLOC_032366	No	No	No	No	--	--	--	--
XLOC_032589	No	No	No	No	XP_004510997.1	--	T	--
XLOC_032615	No	No	No	No	--	--	--	--
XLOC_032667	No	No	No	No	--	--	--	--
XLOC_032734	No	No	No	No	XP_004515791.1	--	--	--
XLOC_032738	No	No	No	No	XP_004489889.1	--	--	--
XLOC_032810	No	No	No	No	--	--	--	--
XLOC_032826	No	No	No	No	XP_004496212.1	--	--	--
XLOC_032942	No	No	No	No	XP_004504739.1	WRKY	--	--
XLOC_032970	No	No	No	No	XP_004491627.1	--	Q	K10775
XLOC_033075	No	No	No	No	--	--	--	--
XLOC_033120	No	No	No	No	XP_004486079.1	SBP	R	K11801
XLOC_033157	No	No	No	No	XP_004509087.1	--	--	--
XLOC_033159	No	No	No	No	XP_012573684.1	--	X	--
XLOC_033281	No	No	No	No	XP_004506407.1	--	--	--
XLOC_033499	No	No	No	No	XP_012572486.1	--	--	--
XLOC_033559	No	No	No	No	XP_004494245.1	--	--	--
XLOC_033612	No	No	No	No	KEH33163.1	--	--	--
XLOC_033649	No	No	No	No	XP_012569277.1	--	--	--
XLOC_033718	No	No	No	No	--	--	--	--

Differentially expressed gene (DEG) IDs	*C. microphyllum* root and shoot-specific (>2 fold)	*C. microphyllum* root and shoot-specific (>10 fold)	ICC4958 and *C. microphyllum* root-specific (>2 fold)	*C. microphyllum* root-specific (>2 fold)	Functional Annotation			
					NCBI nr IDs	TFs	KOG	KEGG
XLOC_033730	No	No	No	No	ABM53543.1	--	--	--
XLOC_033731	No	No	No	No	BAO87435.1	--	--	--
XLOC_033846	No	No	No	No	--	--	--	--
XLOC_033868	No	No	No	No	XP_004489030.1	--	C	--
XLOC_033907	No	No	No	No	XP_012575093.1	--	--	--
XLOC_033933	No	No	No	No	XP_004509420.1	--	--	--
XLOC_033938	No	No	No	No	XP_004490978.1	--	X	--
XLOC_034017	No	No	No	No	XP_004487372.1	--	--	--
XLOC_034057	No	No	No	No	--	--	--	--
XLOC_034059	No	No	No	No	EGU84837.1	--	J	--
XLOC_034138	No	No	No	No	YP_002149745.1	--	--	--
XLOC_034154	No	No	No	No	XP_004488314.1	--	T	K12123
XLOC_034202	No	No	No	No	XP_004485547.1	NAC	--	--
XLOC_034395	No	No	No	No	XP_004504167.1	NF-YB	OR	--
XLOC_034494	No	No	No	No	XP_004506434.1	--	--	--
XLOC_034496	No	No	No	No	XP_004501691.1	--	J	K01875
XLOC_034508	No	No	No	No	--	--	--	--
XLOC_034514	No	No	No	No	XP_004512062.1	--	--	--
XLOC_034531	No	No	No	No	XP_012567523.1	--	I	--
XLOC_034656	No	No	No	No	--	--	--	--
XLOC_034657	No	No	No	No	XP_004486283.1	--	--	--
XLOC_034658	No	No	No	No	--	--	--	--
XLOC_034718	No	No	No	No	XP_004494707.1	--	X	--
XLOC_034784	No	No	No	No	XP_004495442.1	--	--	K15777
XLOC_034798	No	No	No	No	XP_004500717.1	--	A	K13179
XLOC_034837	No	No	No	No	XP_004500735.1	--	T	--
XLOC_034894	No	No	No	No	KEH21548.1	--	--	--
XLOC_034930	No	No	No	No	XP_004503646.1	ERF	T	--
XLOC_034987	No	No	No	No	XP_004500623.1	--	--	--
XLOC_035013	No	No	No	No	XP_004514044.1	--	--	--
XLOC_035156	No	No	No	No	XP_004493726.1	--	P	K01537
XLOC_035305	No	No	No	No	--	--	--	--
XLOC_035382	No	No	No	No	XP_004509968.1	ZF-HD	--	--
XLOC_035422	No	No	No	No	--	--	--	--
XLOC_035439	No	No	No	No	XP_004491539.1	--	G	K01623
XLOC_035637	No	No	No	No	--	--	--	--
XLOC_035700	No	No	No	No	XP_004511773.1	--	--	K00430
XLOC_035729	No	No	No	No	XP_004512015.1	--	--	K17508
XLOC_035811	No	No	No	No	KEH16934.1	--	X	--
XLOC_035814	No	No	No	No	XP_004498601.1	--	--	--

Differentially expressed gene (DEG) IDs	*C. microphyllum* root and shoot-specific (>2 fold)	*C. microphyllum* root and shoot-specific (>10 fold)	ICC4958 and *C. microphyllum* root-specific (>2 fold)	*C. microphyllum* root-specific (>2 fold)	Functional Annotation			
					NCBI nr IDs	TFs	KOG	KEGG
XLOC_035953	No	No	No	No	XP_004510728.1	--	--	--
XLOC_035996	No	No	No	No	XP_012569452.1	--	--	--
XLOC_036074	No	No	No	No	--	--	--	--
XLOC_036099	No	No	No	No	XP_004485880.1	--	--	--
XLOC_036193	No	No	No	No	XP_004515262.1	--	--	--
XLOC_036194	No	No	No	No	XP_004492677.1	HD-ZIP	--	--
XLOC_036287	No	No	No	No	--	--	--	--
XLOC_036301	No	No	No	No	XP_004504303.1	--	--	--
XLOC_036362	No	No	No	No	--	--	--	--
XLOC_036396	No	No	No	No	XP_004490516.1	--	--	--
XLOC_036397	No	No	No	No	XP_004505255.1	--	--	--
XLOC_036576	No	No	No	No	--	--	--	--
XLOC_036581	No	No	No	No	XP_004495508.1	--	E	K01761
XLOC_036604	No	No	No	No	XP_003621687.1	--	C	K05580
XLOC_036637	No	No	No	No	--	--	--	--
XLOC_036646	No	No	No	No	XP_004500476.1	--	--	--
XLOC_036660	No	No	No	No	XP_004497888.1	--	--	--
XLOC_036723	No	No	No	No	WP_050552042.1	--	--	--
XLOC_036742	No	No	No	No	--	--	--	--
XLOC_036744	No	No	No	No	--	--	--	K10046
XLOC_036766	No	No	No	No	XP_004493408.1	--	R	K14508
XLOC_036838	No	No	No	No	--	--	--	--
XLOC_036924	No	No	No	No	--	--	--	--
XLOC_036972	No	No	No	No	XP_004512137.1	--	--	--
XLOC_037007	No	No	No	No	XP_004507270.1	--	P	K13811
XLOC_037017	No	No	No	No	--	--	--	--
XLOC_037037	No	No	No	No	XP_004493554.1	--	--	--
XLOC_037041	No	No	No	No	--	--	--	--
XLOC_037066	No	No	No	No	--	--	--	--
XLOC_037099	No	No	No	No	--	--	--	--
XLOC_037116	No	No	No	No	--	--	--	--
XLOC_037135	No	No	No	No	XP_004491047.1	--	--	--
XLOC_037205	No	No	No	No	--	--	--	--
XLOC_037296	No	No	No	No	XP_004491368.1	--	--	--
XLOC_037385	No	No	No	No	--	--	--	--
XLOC_037403	No	No	No	No	--	--	--	--
XLOC_037446	No	No	No	No	XP_004491139.1	--	--	--
XLOC_037595	No	No	No	No	XP_004495749.1	--	--	--
XLOC_037663	No	No	No	No	XP_004509701.1	--	--	--
XLOC_037702	No	No	No	No	--	--	--	--

Differentially expressed gene (DEG) IDs	*C. microphyllum* root and shoot-specific (>2 fold)	*C. microphyllum* root and shoot-specific (>10 fold)	ICC4958 and *C. microphyllum* root-specific (>2 fold)	*C. microphyllum* root-specific (>2 fold)	Functional Annotation			
					NCBI nr IDs	TFs	KOG	KEGG
XLOC_037703	No	No	No	No	--	--	--	--
XLOC_037722	No	No	No	No	XP_004502403.2	--	--	--
XLOC_037868	No	No	No	No	XP_004495880.1	--	--	--
XLOC_037881	No	No	No	No	--	--	--	--
XLOC_037980	No	No	No	No	--	--	--	--
XLOC_038006	No	No	No	No	--	--	--	--
XLOC_038009	No	No	No	No	--	--	--	--
XLOC_038040	No	No	No	No	--	--	--	--
XLOC_000001	No	No	No	No	--	--	--	--
XLOC_000002	No	No	No	No	XP_008896547.1	--	--	--
XLOC_000004	DEG(M)	No	No	No	--	--	--	--
XLOC_000006	No	No	No	No	--	--	--	--
XLOC_000012	No	No	No	No	--	--	--	K13993
XLOC_000013	No	No	No	No	ETL43467.1	--	--	K03953
XLOC_000019	No	No	No	No	--	--	--	--
XLOC_000022	No	No	No	No	--	--	--	--
XLOC_000024	No	No	No	No	--	--	--	--
XLOC_000025	DEG(M)	DEG(M)	No	No	--	--	--	--
XLOC_000029	No	No	No	No	--	--	--	--
XLOC_000030	No	No	No	No	EGU83720.1	--	--	--
XLOC_000032	No	No	No	No	CEL53921.1	--	--	--
XLOC_000036	No	No	No	No	--	--	B	--
XLOC_000038	No	No	No	No	EUC64149.1	--	--	K10950
XLOC_000041	DEG(M)	No	No	No	--	--	--	--
XLOC_000043	No	No	DEG(I+M)	No	--	--	--	--
XLOC_000045	DEG(M)	No	No	No	EUC65363.1	--	--	--
XLOC_000046	No	No	No	No	--	--	--	--
XLOC_000049	No	No	No	No	ETL78984.1	--	E	--
XLOC_000077	No	No	No	No	XP_004506994.1	--	RT	K14488
XLOC_000078	DEG(M)	No	No	No	--	--	--	--
XLOC_000081	No	No	No	No	--	--	G	K01100
XLOC_000083	DEG(M)	No	No	No	EUC59099.1	--	--	--
XLOC_000085	No	No	No	No	EUC59099.1	--	--	--
XLOC_000086	No	No	No	No	KEP49966.1	--	--	--
XLOC_000093	DEG(M)	No	No	No	--	--	--	--
XLOC_000094	DEG(M)	No	No	No	--	--	--	--
XLOC_000098	No	No	No	No	--	--	O	--
XLOC_000104	DEG(M)	DEG(M)	No	No	XP_003050503.1	--	--	--
XLOC_000107	DEG(M)	No	No	No	--	--	--	--
XLOC_000116	DEG(M)	DEG(M)	No	No	--	--	--	--

Differentially expressed gene (DEG) IDs	*C. microphyllum* root and shoot-specific (>2 fold)	*C. microphyllum* root and shoot-specific (>10 fold)	ICC4958 and *C. microphyllum* root-specific (>2 fold)	*C. microphyllum* root-specific (>2 fold)	Functional Annotation			
					NCBI nr IDs	TFs	KOG	KEGG
XLOC_000120	DEG(M)	DEG(M)	DEG(I+M)	DEG(M)	CCO32502.1	--	--	--
XLOC_000124	No	No	No	No	--	--	--	--
XLOC_000130	DEG(M)	No	No	No	XP_008891756.1	NAC	J	--
XLOC_000131	DEG(M)	DEG(M)	DEG(I+M)	DEG(M)	--	--	--	--
XLOC_000137	No	No	No	No	--	--	--	--
XLOC_000141	No	No	No	No	EUC55641.1	--	J	--
XLOC_000142	No	No	No	No	EUC55641.1	--	J	--
XLOC_000143	No	No	No	No	--	--	--	--
XLOC_000144	No	No	No	No	--	--	--	--
XLOC_000145	No	No	No	No	EUC54937.1	--	--	--
XLOC_000146	No	No	No	No	EUC54937.1	--	--	--
XLOC_000147	No	No	No	No	EUC54937.1	--	--	--
XLOC_000148	DEG(M)	No	No	No	--	--	--	--
XLOC_000150	No	No	No	No	KDN34978.1	--	R	--
XLOC_000151	No	No	No	No	KDN34978.1	--	R	K03146
XLOC_000156	No	No	No	No	--	--	--	--
XLOC_000157	DEG(M)	No	No	No	--	--	--	--
XLOC_000158	DEG(M)	No	No	No	--	--	--	--
XLOC_000165	No	No	No	No	--	--	--	--
XLOC_000166	DEG(M)	No	No	No	EUC66279.1	--	J	--
XLOC_000169	No	No	No	No	EUC66279.1	--	--	--
XLOC_000171	DEG(M)	No	No	No	--	--	--	--
XLOC_000172	DEG(M)	No	No	No	--	--	--	--
XLOC_000173	No	No	DEG(I+M)	No	--	--	--	--
XLOC_000176	No	No	No	No	--	--	--	--
XLOC_000185	DEG(M)	No	No	No	EUC53664.1	--	--	--
XLOC_000189	No	No	No	No	--	--	--	--
XLOC_000193	No	No	No	No	--	--	O	K01363
XLOC_000194	No	No	No	No	--	--	O	K01363
XLOC_000195	No	No	No	No	CEL51610.1	--	H	--
XLOC_000199	No	No	No	No	--	--	--	--
XLOC_000200	No	No	No	No	--	--	--	--
XLOC_000201	No	No	No	No	--	--	--	--
XLOC_000203	No	No	No	No	ACY66807.1	--	--	--
XLOC_000204	DEG(M)	DEG(M)	No	No	--	--	--	--
XLOC_000208	No	No	No	No	XP_002514129.1	--	X	--
XLOC_000210	DEG(M)	No	DEG(I+M)	DEG(M)	--	--	--	--
XLOC_000211	No	No	No	No	CEL57451.1	--	--	--
XLOC_000212	No	No	No	No	--	--	--	--
XLOC_000213	No	No	No	No	--	--	--	--

Differentially expressed gene (DEG) IDs	*C. microphyllum* root and shoot-specific (>2 fold)	*C. microphyllum* root and shoot-specific (>10 fold)	ICC4958 and *C. microphyllum* root-specific (>2 fold)	*C. microphyllum* root-specific (>2 fold)	Functional Annotation			
					NCBI nr IDs	TFs	KOG	KEGG
XLOC_000214	DEG(M)	No	No	No	AHZ64889.2	--	--	--
XLOC_000215	No	No	No	No	--	--	--	--
XLOC_000216	No	No	No	No	--	--	--	--
XLOC_000217	No	No	No	No	--	--	--	--
XLOC_000218	DEG(M)	No	No	No	--	--	--	--
XLOC_000220	No	No	No	No	--	--	--	--
XLOC_000222	No	No	No	No	ELU45622.1	--	J	--
XLOC_000225	No	No	No	No	--	--	--	--
XLOC_000226	No	No	No	No	--	--	--	--
XLOC_000230	No	No	No	No	--	--	--	--
XLOC_000235	DEG(M)	No	No	No	--	--	--	--
XLOC_000238	No	No	No	No	XP_001943874.1	--	--	K03247
XLOC_000241	No	No	No	No	XP_001948556.2	--	C	K00162
XLOC_000242	DEG(M)	No	No	No	XP_008893447.1	--	--	--
XLOC_000246	No	No	No	No	--	--	--	--
XLOC_000250	No	No	No	No	--	--	--	--
XLOC_000253	DEG(M)	No	No	No	--	--	--	--
XLOC_000257	No	No	DEG(I+M)	No	XP_004487066.1	MYB	--	K09422
XLOC_000259	DEG(M)	No	No	No	--	--	--	--
XLOC_000260	No	No	No	No	--	--	--	--
XLOC_000262	No	No	No	No	KHN84204.1	--	J	--
XLOC_000263	No	No	No	No	--	--	--	--
XLOC_000264	DEG(M)	DEG(M)	DEG(I+M)	DEG(M)	--	--	--	--
XLOC_000269	No	No	No	No	--	--	--	--
XLOC_000279	DEG(M)	No	DEG(I+M)	DEG(M)	--	--	--	--
XLOC_000281	DEG(M)	No	No	No	EUC63162.1	--	--	--
XLOC_000285	No	No	No	No	--	--	--	--
XLOC_000291	No	No	No	No	--	--	--	--
XLOC_000293	No	No	No	No	XP_003048460.1	--	--	--
XLOC_000299	DEG(M)	No	No	No	--	--	--	--
XLOC_000300	No	No	No	No	--	--	--	--
XLOC_000308	No	No	No	No	--	--	--	--
XLOC_000313	No	No	No	No	--	--	--	--
XLOC_000314	DEG(M)	DEG(M)	No	No	ENH67687.1	--	--	--
XLOC_000316	No	No	No	No	--	--	--	--
XLOC_000318	No	No	No	No	--	--	--	--
XLOC_000320	DEG(M)	No	No	No	EUC58674.1	--	--	--
XLOC_000322	DEG(M)	No	DEG(I+M)	DEG(M)	KEP50011.1	NF-YC	B	--
XLOC_000324	DEG(M)	No	No	No	CEL64056.1	--	--	--
XLOC_000325	DEG(M)	No	No	No	EUC60526.1	--	--	--

Differentially expressed gene (DEG) IDs	*C. microphyllum* root and shoot-specific (>2 fold)	*C. microphyllum* root and shoot-specific (>10 fold)	ICC4958 and *C. microphyllum* root-specific (>2 fold)	*C. microphyllum* root-specific (>2 fold)	Functional Annotation			
					NCBI nr IDs	TFs	KOG	KEGG
XLOC_000329	DEG(M)	No	No	No	AAM51199.1	YABBY	OR	--
XLOC_000330	No	No	No	No	ABC33921.1	--	O	--
XLOC_000336	No	No	No	No	XP_003045611.1	--	--	--
XLOC_000337	No	No	No	No	--	--	--	--
XLOC_000340	DEG(M)	No	No	No	--	--	--	--
XLOC_000353	No	No	No	No	--	--	O	--
XLOC_000359	No	No	No	No	CCO32241.1	--	--	--
XLOC_000361	No	No	No	No	--	--	--	--
XLOC_000362	DEG(M)	No	No	No	XP_008890825.1	--	--	--
XLOC_000363	No	No	No	No	XP_004508678.1	--	--	--
XLOC_000367	No	No	No	No	CCO36236.1	--	--	--
XLOC_000369	No	No	No	No	--	--	--	--
XLOC_000371	No	No	DEG(I+M)	No	--	--	--	K06901
XLOC_000375	No	No	No	No	--	--	--	--
XLOC_000377	No	No	No	No	ETI32806.1	--	--	--
XLOC_000378	No	No	No	No	ETI32805.1	--	--	--
XLOC_000381	No	No	No	No	CCO27851.1	--	--	--
XLOC_000383	DEG(M)	No	No	No	--	--	--	--
XLOC_000384	No	No	No	No	--	--	--	--
XLOC_000387	No	No	No	No	--	--	--	--
XLOC_000388	No	No	No	No	--	--	--	--
XLOC_000389	No	No	DEG(I+M)	No	--	--	--	--
XLOC_000392	No	No	No	No	CCO33210.1	--	--	--
XLOC_000394	No	No	No	No	--	HD-ZIP	M	K01728
XLOC_000398	No	No	No	No	--	--	--	--
XLOC_000402	No	No	No	No	--	--	--	--
XLOC_000403	No	No	No	No	--	--	--	--
XLOC_000406	No	No	No	No	--	--	--	--
XLOC_000410	DEG(M)	No	DEG(I+M)	DEG(M)	--	--	--	--
XLOC_000411	No	No	No	No	--	--	--	--
XLOC_000417	DEG(M)	No	No	No	XP_007863642.1	--	Z	K08341
XLOC_000421	DEG(M)	No	No	No	XP_003052666.1	--	--	--
XLOC_000423	No	No	No	No	--	--	--	--
XLOC_000424	No	No	No	No	--	--	J	--
XLOC_000427	DEG(M)	No	No	No	--	--	--	--
XLOC_000428	DEG(M)	No	No	No	--	--	--	--
XLOC_000430	No	No	No	No	ETP54420.1	--	--	K00654
XLOC_000432	DEG(M)	No	No	No	--	--	--	--
XLOC_000433	No	No	No	No	XP_002529394.1	--	U	K13431
XLOC_000435	DEG(M)	DEG(M)	DEG(I+M)	DEG(M)	--	--	--	--

Differentially expressed gene (DEG) IDs	*C. microphyllum* root and shoot-specific (>2 fold)	*C. microphyllum* root and shoot-specific (>10 fold)	ICC4958 and *C. microphyllum* root-specific (>2 fold)	*C. microphyllum* root-specific (>2 fold)	Functional Annotation			
					NCBI nr IDs	TFs	KOG	KEGG
XLOC_000437	No	No	No	No	--	--	--	--
XLOC_000438	No	No	No	No	EUC62371.1	--	T	--
XLOC_000439	No	No	No	No	--	--	--	--
XLOC_000440	No	No	No	No	EUC63263.1	--	--	--
XLOC_000441	No	No	No	No	XP_003053570.1	--	--	--
XLOC_000443	No	No	No	No	--	--	--	--
XLOC_000445	No	No	No	No	CCO26635.1	--	I	--
XLOC_000446	No	No	No	No	XP_003047702.1	--	--	--
XLOC_000453	No	No	No	No	--	--	--	--
XLOC_000454	No	No	No	No	NP_001165435.1	--	Z	--
XLOC_000458	No	No	No	No	NP_001119691.1	--	--	--
XLOC_000465	No	No	No	No	--	--	--	--
XLOC_000469	No	No	No	No	XP_012065894.1	--	A	K14409
XLOC_000471	No	No	No	No	--	--	--	K03007
XLOC_000472	No	No	No	No	--	--	--	--
XLOC_000475	No	No	No	No	NP_001156232.1	--	--	--
XLOC_000478	DEG(M)	No	No	No	--	--	--	--
XLOC_000480	No	No	No	No	--	--	TZ	--
XLOC_000482	No	No	No	No	--	--	--	--
XLOC_000483	No	No	No	No	XP_003302037.1	--	--	--
XLOC_000488	No	No	No	No	BAH72354.1	--	C	--
XLOC_000490	No	No	No	No	EUC67375.1	--	--	--
XLOC_000491	DEG(M)	DEG(M)	No	No	XP_003302315.1	--	--	--
XLOC_000494	No	No	No	No	--	--	--	--
XLOC_000495	DEG(M)	No	No	No	--	--	--	--
XLOC_000496	No	No	No	No	--	--	--	--
XLOC_000497	No	No	No	No	--	--	--	K12876
XLOC_000498	No	No	No	No	--	--	--	--
XLOC_000501	No	No	No	No	ENH63714.1	--	--	--
XLOC_000502	No	No	No	No	CCO31206.1	--	--	--
XLOC_000503	No	No	No	No	EMT67922.1	--	--	--
XLOC_000505	No	No	No	No	--	--	--	K13237
XLOC_000517	No	No	No	No	XP_004510968.1	--	--	--
XLOC_000518	No	No	No	No	CEL57981.1	--	--	--
XLOC_000519	No	No	No	No	NP_001156169.1	--	--	--
XLOC_000521	No	No	No	No	XP_007011538.1	NAC	S	--
XLOC_000522	No	No	No	No	--	--	--	--
XLOC_000527	No	No	No	No	XP_008909588.1	--	R	--
XLOC_000528	DEG(M)	No	No	No	ETP45818.1	--	--	--
XLOC_000531	No	No	No	No	YP_002323941.1	--	--	--

**Differentially expressed gene (DEG) IDs**	***C. microphyllum*** **root and shoot-specific (>2 fold)**	***C. microphyllum*** **root and shoot-specific (>10 fold)**	**ICC4958 and** ***C. microphyllum*** **root-specific (>2 fold)**	***C. microphyllum*** **root-specific (>2 fold)**	**Functional Annotation**			
					**NCBI nr IDs**	**TFs**	**KOG**	**KEGG**
XLOC_000536	DEG(M)	No	No	No	XP_009516025.1	--	--	--
XLOC_000539	No	No	No	No	--	--	A	K12896
XLOC_000540	No	No	No	No	--	--	--	--
XLOC_000541	No	No	No	No	--	--	--	--
XLOC_000542	No	No	No	No	--	--	--	--
XLOC_000552	No	No	No	No	CEL53899.1	--	--	--
XLOC_000553	DEG(M)	No	No	No	--	--	--	--
XLOC_000554	No	No	No	No	--	--	--	--
XLOC_000556	No	No	No	No	--	--	--	--
XLOC_000557	No	No	No	No	--	--	--	K02109
XLOC_000558	DEG(M)	No	No	No	--	--	--	--
XLOC_000560	No	No	No	No	XP_008902960.1	--	EG	--
XLOC_000561	DEG(M)	No	No	No	XP_003052364.1	--	--	--
XLOC_000563	DEG(M)	No	No	No	--	--	--	--
XLOC_000564	No	No	No	No	--	--	--	--
XLOC_000565	No	No	No	No	--	--	--	--
XLOC_000566	DEG(M)	No	No	No	XP_008911170.1	--	--	--
XLOC_000567	No	No	No	No	XP_004499200.1	--	--	--
XLOC_000570	No	No	No	No	--	--	--	--
XLOC_000571	DEG(M)	DEG(M)	No	No	--	--	--	--
XLOC_000573	DEG(M)	No	No	No	--	--	O	--
XLOC_000574	DEG(M)	DEG(M)	DEG(I+M)	DEG(M)	EWG47635.1	--	--	--
XLOC_000575	DEG(M)	No	No	No	--	--	--	--
XLOC_000576	DEG(M)	No	No	No	--	--	--	--
XLOC_000577	No	No	No	No	CEL63179.1	--	--	--
XLOC_000579	No	No	No	No	CCO27676.1	--	--	--
XLOC_000582	DEG(M)	No	No	No	XP_008906437.1	--	--	--
XLOC_000583	DEG(M)	DEG(M)	DEG(I+M)	DEG(M)	--	--	--	--
XLOC_000584	DEG(M)	No	No	No	--	--	--	--
XLOC_000586	DEG(M)	No	No	No	--	--	J	--
XLOC_000588	No	No	No	No	--	--	--	--
XLOC_000589	DEG(M)	No	No	No	EUC67308.1	--	--	--
XLOC_000595	No	No	No	No	XP_001949624.2	--	I	--
XLOC_000598	No	No	No	No	XP_008184138.1	--	--	K10258
XLOC_000599	No	No	No	No	--	--	--	--
XLOC_000600	DEG(M)	DEG(M)	DEG(I+M)	DEG(M)	--	--	--	--
XLOC_000601	No	No	No	No	XP_004493636.1	--	--	--
XLOC_000607	DEG(M)	No	No	No	--	--	--	--
XLOC_000608	No	No	No	No	--	--	--	--
XLOC_000613	No	No	No	No	KHN80715.1	NAC	J	--

Differentially expressed gene (DEG) IDs	*C. microphyllum* root and shoot-specific (>2 fold)	*C. microphyllum* root and shoot-specific (>10 fold)	ICC4958 and *C. microphyllum* root-specific (>2 fold)	*C. microphyllum* root-specific (>2 fold)	Functional Annotation			
					NCBI nr IDs	TFs	KOG	KEGG
XLOC_000614	No	No	No	No	--	--	--	--
XLOC_000617	DEG(M)	No	No	No	AAH53790.1	--	T	--
XLOC_000618	No	No	No	No	--	--	--	--
XLOC_000620	No	No	No	No	EUC63854.1	--	--	--
XLOC_000625	No	No	No	No	KDN49713.1	--	--	--
XLOC_000634	No	No	No	No	--	--	--	--
XLOC_000636	No	No	No	No	--	--	--	--
XLOC_000638	No	No	No	No	--	--	--	K03260
XLOC_000639	No	No	No	No	XP_008179981.1	--	--	--
XLOC_000640	No	No	No	No	--	--	C	K01647
XLOC_000641	No	No	DEG(I+M)	No	--	--	QI	--
XLOC_000644	No	No	No	No	XP_001655557.1	--	--	--
XLOC_000645	DEG(M)	DEG(M)	No	No	--	--	--	--
XLOC_000647	No	No	No	No	--	--	--	--
XLOC_000650	DEG(M)	No	DEG(I+M)	DEG(M)	KIM87401.1	--	B	--
XLOC_000651	No	No	No	No	--	--	--	--
XLOC_000652	No	No	No	No	--	--	--	--
XLOC_000653	No	No	No	No	--	--	--	--
XLOC_000654	No	No	No	No	--	--	--	--
XLOC_000656	No	No	No	No	--	MYB_related	--	K04650
XLOC_000660	DEG(M)	No	No	No	--	--	--	--
XLOC_000662	No	No	No	No	ELU40990.1	--	--	--
XLOC_000666	No	No	No	No	--	--	--	K01190
XLOC_000667	No	No	No	No	--	--	--	--
XLOC_000670	No	No	No	No	XP_013063739.1	--	C	--
XLOC_000671	DEG(M)	No	No	No	AFK44593.1	--	J	K02889
XLOC_000673	DEG(M)	No	No	No	--	--	--	--
XLOC_000674	No	No	No	No	XP_003047792.1	--	--	--
XLOC_000675	No	No	No	No	--	--	--	--
XLOC_000679	No	No	DEG(I+M)	No	XP_004497292.1	--	--	--
XLOC_000680	No	No	No	No	XP_001952818.2	--	--	--
XLOC_000681	No	No	No	No	--	--	--	--
XLOC_000682	No	No	No	No	--	--	--	--
XLOC_000688	No	No	No	No	XP_003043211.1	--	--	--
XLOC_000689	No	No	No	No	--	--	--	--
XLOC_000690	No	No	No	No	--	--	--	--
XLOC_000692	DEG(M)	No	No	No	KDN43127.1	--	--	--
XLOC_000693	No	No	No	No	--	--	--	--
XLOC_000697	No	No	No	No	EUC55478.1	--	--	--
XLOC_000698	DEG(M)	No	No	No	KEH39093.1	--	R	--

Differentially expressed gene (DEG) IDs	*C. microphyllum* root and shoot-specific (>2 fold)	*C. microphyllum* root and shoot-specific (>10 fold)	ICC4958 and *C. microphyllum* root-specific (>2 fold)	*C. microphyllum* root-specific (>2 fold)	Functional Annotation			
					NCBI nr IDs	TFs	KOG	KEGG
XLOC_000699	No	No	No	No	KDN50517.1	--	--	K09510
XLOC_000700	No	No	No	No	--	--	--	--
XLOC_000701	DEG(M)	DEG(M)	No	No	NP_001156256.1	--	J	--
XLOC_000702	No	No	No	No	--	--	QI	K09837
XLOC_000703	No	No	No	No	--	--	--	--
XLOC_000705	No	No	No	No	--	--	--	--
XLOC_000713	No	No	No	No	--	--	--	--
XLOC_000715	No	No	No	No	XP_003050747.1	--	--	--
XLOC_000717	No	No	No	No	ETL77578.1	--	--	--
XLOC_000718	No	No	No	No	XP_011161394.1	--	TZR	--
XLOC_000720	DEG(M)	No	No	No	--	--	--	--
XLOC_000725	No	No	No	No	--	--	F	--
XLOC_000726	No	No	No	No	--	--	--	--
XLOC_000727	No	No	No	No	--	--	--	--
XLOC_000728	No	No	No	No	KEP49572.1	--	--	--
XLOC_000731	No	No	No	No	EUC61103.1	--	--	--
XLOC_000735	No	No	No	No	--	--	--	K08762
XLOC_000738	DEG(M)	No	No	No	XP_008893312.1	--	--	--
XLOC_000739	No	No	No	No	--	--	--	--
XLOC_000742	No	No	No	No	--	--	--	--
XLOC_000744	No	No	DEG(I+M)	No	--	--	--	--
XLOC_000746	DEG(M)	DEG(M)	No	No	KLO96367.1	--	C	--
XLOC_000751	No	No	No	No	AAL85609.1	--	--	--
XLOC_000753	No	No	No	No	--	--	--	--
XLOC_000754	DEG(M)	No	No	No	--	--	G	--
XLOC_000758	DEG(M)	No	No	No	--	--	--	--
XLOC_000759	No	No	No	No	EUC56534.1	--	--	--
XLOC_000767	No	No	No	No	--	--	--	--
XLOC_000768	No	No	No	No	--	--	--	--
XLOC_000769	DEG(M)	No	No	No	--	--	--	--
XLOC_000774	No	No	No	No	--	--	--	--
XLOC_000775	No	No	No	No	XP_001945478.1	--	O	K03035
XLOC_000776	No	No	No	No	--	--	--	K14484
XLOC_000778	No	No	No	No	CEL59138.1	--	--	--
XLOC_000781	DEG(M)	DEG(M)	DEG(I+M)	DEG(M)	CCO37453.1	--	--	--
XLOC_000787	No	No	No	No	XP_001651231.2	--	--	--
XLOC_000788	No	No	No	No	EUC56858.1	--	--	--
XLOC_000789	DEG(M)	No	DEG(I+M)	DEG(M)	--	--	--	--
XLOC_000790	DEG(M)	DEG(M)	No	No	--	--	--	--
XLOC_000792	No	No	No	No	XP_001650852.1	--	U	--

Differentially expressed gene (DEG) IDs	*C. microphyllum* root and shoot-specific (>2 fold)	*C. microphyllum* root and shoot-specific (>10 fold)	ICC4958 and *C. microphyllum* root-specific (>2 fold)	*C. microphyllum* root-specific (>2 fold)	Functional Annotation			
					NCBI nr IDs	TFs	KOG	KEGG
XLOC_000794	No	No	No	No	NP_001119637.1	--	C	--
XLOC_000795	DEG(M)	No	No	No	NP_001129370.1	--	J	--
XLOC_000796	No	No	No	No	--	--	--	--
XLOC_000797	No	No	No	No	--	--	--	--
XLOC_000798	No	No	No	No	--	--	--	--
XLOC_000802	No	No	No	No	KFM63085.1	--	O	--
XLOC_000804	No	No	No	No	XP_003050154.1	--	--	--
XLOC_000805	DEG(M)	No	No	No	--	--	T	--
XLOC_000810	No	No	No	No	--	--	--	--
XLOC_000812	No	No	No	No	XP_003045654.1	--	--	--
XLOC_000818	No	No	No	No	--	--	--	K03253
XLOC_000819	DEG(M)	No	No	No	XP_009534754.1	--	--	--
XLOC_000825	No	No	No	No	--	--	--	--
XLOC_000826	DEG(M)	DEG(M)	No	No	--	--	--	--
XLOC_000827	No	No	No	No	--	--	--	--
XLOC_000828	No	No	No	No	--	--	--	--
XLOC_000831	DEG(M)	DEG(M)	DEG(I+M)	DEG(M)	XP_002903085.1	--	J	K02966
XLOC_000832	No	No	No	No	XP_004487306.1	--	--	K08902
XLOC_000834	No	No	No	No	CCO30537.1	--	U	--
XLOC_000835	DEG(M)	No	DEG(I+M)	DEG(M)	CCO30537.1	--	--	--
XLOC_000836	No	No	No	No	--	--	O	--
XLOC_000837	DEG(M)	No	No	No	--	--	--	--
XLOC_000840	No	No	No	No	--	--	--	--
XLOC_000845	No	No	No	No	XP_003050859.1	--	--	--
XLOC_000846	No	No	No	No	NP_001156707.1	--	O	--
XLOC_000852	DEG(M)	No	No	No	--	--	--	K05894
XLOC_000853	No	No	No	No	XP_003040992.1	--	--	--
XLOC_000856	DEG(M)	DEG(M)	No	No	EGU80493.1	--	P	--
XLOC_000857	No	No	No	No	XP_003052643.1	--	P	K04565
XLOC_000865	No	No	DEG(I+M)	No	--	--	R	--
XLOC_000872	DEG(M)	No	No	No	--	--	--	--
XLOC_000874	DEG(M)	DEG(M)	DEG(I+M)	DEG(M)	--	--	--	--
XLOC_000875	DEG(M)	No	No	No	XP_003054277.1	--	--	--
XLOC_000876	DEG(M)	No	No	No	--	--	--	--
XLOC_000879	No	No	No	No	XP_003047535.1	--	J	--
XLOC_000881	No	No	No	No	ABC67965.1	--	O	--
XLOC_000882	DEG(M)	No	No	No	AAU84935.1	--	--	K03233
XLOC_000891	No	No	No	No	XP_009523844.1	--	G	K01810
XLOC_000892	No	No	No	No	--	--	--	--
XLOC_000896	No	No	No	No	--	--	--	--

**Differentially expressed gene (DEG) IDs**	***C. microphyllum* root and shoot-specific (>2 fold)**	***C. microphyllum* root and shoot-specific (>10 fold)**	**ICC4958 and *C. microphyllum* root-specific (>2 fold)**	***C. microphyllum* root-specific (>2 fold)**	**Functional Annotation**			
					**NCBI nr IDs**	**TFs**	**KOG**	**KEGG**
XLOC_000897	No	No	No	No	--	--	--	K00670
XLOC_000898	No	No	No	No	--	--	--	--
XLOC_000899	No	No	No	No	--	--	--	--
XLOC_000901	DEG(M)	No	No	No	NP_001127759.1	--	X	--
XLOC_000902	No	No	No	No	XP_009521646.1	--	--	K02863
XLOC_000905	No	No	No	No	--	--	--	--
XLOC_000907	No	No	No	No	--	--	--	--
XLOC_000909	DEG(M)	No	No	No	--	--	--	K02942
XLOC_000913	No	No	No	No	--	--	--	--
XLOC_000914	No	No	No	No	CEL62963.1	--	--	--
XLOC_000918	No	No	No	No	XP_012266498.1	--	J	--
XLOC_000919	No	No	No	No	--	--	--	--
XLOC_000923	No	No	No	No	--	--	--	--
XLOC_000924	No	No	No	No	XP_008894985.1	--	--	--
XLOC_000926	No	No	No	No	--	--	--	--
XLOC_000927	No	No	No	No	XP_001654749.1	--	F	K10808
XLOC_000946	DEG(M)	No	No	No	--	--	--	--
XLOC_000948	No	No	No	No	--	--	--	--
XLOC_000951	DEG(M)	No	No	No	BAJ89401.1	--	--	K02974
XLOC_000963	DEG(M)	No	No	No	KNZ53904.1	--	Z	--
XLOC_000974	DEG(M)	No	No	No	KDN44622.1	--	R	--
XLOC_000977	No	No	No	No	--	--	--	--
XLOC_000979	No	No	DEG(I+M)	No	XP_003617399.1	--	O	--
XLOC_000981	DEG(M)	DEG(M)	DEG(I+M)	DEG(M)	CCO33299.1	--	--	--
XLOC_000984	No	No	No	No	EUC67244.1	--	--	--
XLOC_000987	DEG(M)	DEG(M)	No	No	CEF73082.1	--	--	--
XLOC_000989	DEG(M)	DEG(M)	DEG(I+M)	DEG(M)	--	--	--	--
XLOC_000990	No	No	No	No	XP_003048220.1	--	--	--
XLOC_000991	DEG(M)	DEG(M)	No	No	--	--	--	--
XLOC_000992	DEG(M)	DEG(M)	No	No	--	--	--	--
XLOC_000993	DEG(M)	No	No	No	--	--	--	--
XLOC_000994	No	No	No	No	--	--	--	--
XLOC_000996	No	No	No	No	CCO28284.1	--	--	--
XLOC_000997	No	No	No	No	--	--	--	--
XLOC_000998	DEG(M)	DEG(M)	DEG(I+M)	DEG(M)	EMT62038.1	--	EJ	--
XLOC_000999	DEG(M)	No	DEG(I+M)	DEG(M)	EMT62038.1	--	EJ	--
XLOC_001000	No	No	No	No	--	--	--	--
XLOC_001004	No	No	No	No	--	--	--	--
XLOC_001006	No	No	No	No	KEP50802.1	--	--	--
XLOC_001008	DEG(M)	No	No	No	--	--	--	--

Differentially expressed gene (DEG) IDs	*C. microphyllum* root and shoot-specific (>2 fold)	*C. microphyllum* root and shoot-specific (>10 fold)	ICC4958 and *C. microphyllum* root-specific (>2 fold)	*C. microphyllum* root-specific (>2 fold)	Functional Annotation			
					NCBI nr IDs	TFs	KOG	KEGG
XLOC_001013	No	No	No	No	--	--	--	--
XLOC_001015	No	No	No	No	--	--	--	--
XLOC_001016	No	No	No	No	--	--	--	--
XLOC_001019	DEG(M)	No	No	No	ETI41807.1	--	--	--
XLOC_001021	DEG(M)	No	No	No	--	--	--	--
XLOC_001022	No	No	No	No	--	--	--	--
XLOC_001025	DEG(M)	No	No	No	--	--	--	--
XLOC_001029	No	No	No	No	XP_002519066.1	--	--	K10581
XLOC_001030	DEG(M)	No	No	No	--	--	--	--
XLOC_001031	No	No	No	No	--	--	--	--
XLOC_001032	No	No	No	No	XP_003043405.1	--	--	--
XLOC_001037	No	No	No	No	ETK93128.1	--	--	--
XLOC_001042	No	No	No	No	--	--	--	K13207
XLOC_001043	No	No	No	No	--	--	--	--
XLOC_001045	DEG(M)	No	No	No	CEL55905.1	--	--	--
XLOC_001047	No	No	No	No	XP_003243749.1	--	TZ	--
XLOC_001048	DEG(M)	No	No	No	XP_003047792.1	--	--	--
XLOC_001049	No	No	No	No	XP_003047792.1	--	--	--
XLOC_001050	No	No	No	No	AAU84932.1	--	--	--
XLOC_001058	No	No	No	No	--	--	--	--
XLOC_001059	No	No	DEG(I+M)	No	XP_004516741.1	--	--	--
XLOC_001066	No	No	No	No	--	--	--	--
XLOC_001067	No	No	No	No	KNG50001.1	--	J	K03237
XLOC_001068	No	No	No	No	XP_002901967.1	--	U	--
XLOC_001073	No	No	No	No	XP_001946818.1	--	O	--
XLOC_001074	DEG(M)	No	No	No	--	--	--	K03671
XLOC_001076	DEG(M)	No	DEG(I+M)	DEG(M)	KLP10195.1	--	J	--
XLOC_001077	No	No	No	No	EUC53680.1	--	--	--
XLOC_001079	No	No	No	No	CCO30024.1	--	--	--
XLOC_001080	No	No	No	No	--	--	--	--
XLOC_001081	No	No	No	No	--	--	--	--
XLOC_001082	DEG(M)	No	No	No	--	--	--	--
XLOC_001083	No	No	No	No	--	--	--	--
XLOC_001087	No	No	No	No	--	--	--	K02639
XLOC_001099	DEG(M)	No	No	No	KEP49879.1	--	--	--
XLOC_001102	No	No	No	No	--	--	--	--
XLOC_001103	No	No	No	No	XP_008894642.1	--	G	--
XLOC_001104	DEG(M)	DEG(M)	DEG(I+M)	DEG(M)	XP_002908804.1	GATA	J	--
XLOC_001106	DEG(M)	No	No	No	NP_001155568.1	bZIP	J	K02865
XLOC_001107	No	No	No	No	ELU42619.1	--	--	--

Differentially expressed gene (DEG) IDs	*C. microphyllum* root and shoot-specific (>2 fold)	*C. microphyllum* root and shoot-specific (>10 fold)	ICC4958 and *C. microphyllum* root-specific (>2 fold)	*C. microphyllum* root-specific (>2 fold)	Functional Annotation			
					NCBI nr IDs	TFs	KOG	KEGG
XLOC_001112	No	No	No	No	--	--	--	--
XLOC_001116	No	No	No	No	--	--	G	K01689
XLOC_001117	No	No	No	No	EUC57346.1	--	G	--
XLOC_001119	No	No	No	No	EUC58677.1	--	J	--
XLOC_001121	DEG(M)	No	No	No	ABE68882.1	--	J	--
XLOC_001122	DEG(M)	No	No	No	XP_007419339.1	--	J	--
XLOC_001125	No	No	No	No	XP_003043878.1	--	--	--
XLOC_001127	DEG(M)	No	No	No	CCO31137.1	--	--	--
XLOC_001128	DEG(M)	No	No	No	CCO30639.1	--	--	--
XLOC_001129	No	No	No	No	CCO30639.1	--	--	--
XLOC_001131	DEG(M)	DEG(M)	DEG(I+M)	DEG(M)	ELU45956.1	--	--	--
XLOC_001133	No	No	DEG(I+M)	No	--	--	--	--
XLOC_001135	No	No	No	No	XP_008892103.1	--	O	--
XLOC_001137	DEG(M)	No	No	No	XP_007303331.1	--	--	--
XLOC_001138	DEG(M)	No	No	No	CCO31970.1	--	--	--
XLOC_001142	No	No	No	No	KDN44594.1	--	--	K00026
XLOC_001143	No	No	No	No	--	--	J	--
XLOC_001146	DEG(M)	DEG(M)	DEG(I+M)	DEG(M)	XP_008911988.1	--	--	--
XLOC_001148	No	No	No	No	NP_001155834.1	--	--	--
XLOC_001154	DEG(M)	No	No	No	CEL54367.1	--	J	--
XLOC_001156	No	No	No	No	--	--	--	--
XLOC_001157	No	No	No	No	--	--	--	--
XLOC_001165	DEG(M)	No	No	No	ABX44786.1	--	J	--
XLOC_001167	DEG(M)	No	No	No	KIM78989.1	YABBY	OR	--
XLOC_001179	No	No	No	No	--	--	--	--
XLOC_001186	No	No	No	No	EUC64154.1	--	--	--
XLOC_001190	No	No	No	No	ELU40293.1	--	--	--
XLOC_001191	No	No	No	No	ELU40293.1	--	--	--
XLOC_001203	DEG(M)	No	DEG(I+M)	DEG(M)	--	--	--	--
XLOC_001204	DEG(M)	No	DEG(I+M)	DEG(M)	--	--	--	--
XLOC_001213	DEG(M)	No	No	No	--	--	--	--
XLOC_001214	No	No	No	No	--	--	--	--
XLOC_001215	No	No	No	No	--	--	O	--
XLOC_001216	DEG(M)	No	No	No	--	--	--	--
XLOC_001218	No	No	No	No	--	--	--	--
XLOC_001219	No	No	No	No	--	--	T	--
XLOC_001221	No	No	No	No	XP_001656268.1	--	--	K05609
XLOC_001229	No	No	No	No	--	--	--	--
XLOC_001231	No	No	No	No	KEP47164.1	--	--	--
XLOC_001235	No	No	No	No	--	--	--	--

**Differentially expressed gene (DEG) IDs**	***C. microphyllum* root and shoot-specific (>2 fold)**	***C. microphyllum* root and shoot-specific (>10 fold)**	**ICC4958 and *C. microphyllum* root-specific (>2 fold)**	***C. microphyllum* root-specific (>2 fold)**	**Functional Annotation**			
					**NCBI nr IDs**	**TFs**	**KOG**	**KEGG**
XLOC_001237	No	No	No	No	XP_002511100.1	ARF	TK	--
XLOC_001239	No	No	No	No	KDN43343.1	--	O	--
XLOC_001240	No	No	No	No	--	--	RI	--
XLOC_001241	No	No	No	No	--	--	--	K14026
XLOC_001242	DEG(M)	No	No	No	--	--	--	--
XLOC_001244	No	No	No	No	--	--	--	--
XLOC_001249	No	No	No	No	--	--	--	--
XLOC_001252	No	No	No	No	--	--	R	--
XLOC_001253	No	No	No	No	--	--	--	--
XLOC_001255	DEG(M)	No	No	No	EUC62095.1	--	C	--
XLOC_001256	No	No	No	No	EUC62095.1	--	C	--
XLOC_001257	DEG(M)	DEG(M)	DEG(I+M)	DEG(M)	CEL51952.1	--	C	--
XLOC_001264	No	No	DEG(I+M)	No	--	--	--	--
XLOC_001267	No	No	DEG(I+M)	No	XP_004503972.1	--	--	K19040
XLOC_001268	No	No	DEG(I+M)	No	--	--	--	K19040
XLOC_001277	No	No	No	No	KHN70773.1	--	J	K02925
XLOC_001286	DEG(M)	DEG(M)	No	No	--	--	--	--
XLOC_001299	DEG(M)	DEG(M)	DEG(I+M)	DEG(M)	--	--	--	--
XLOC_001301	No	No	No	No	--	--	--	--
XLOC_001312	DEG(M)	DEG(M)	DEG(I+M)	DEG(M)	--	--	--	--
XLOC_001319	DEG(M)	DEG(M)	DEG(I+M)	DEG(M)	CEL54131.1	--	J	--
XLOC_001320	No	No	No	No	CEL54131.1	--	J	--
XLOC_001336	No	No	No	No	XP_003245192.1	--	--	--
XLOC_001337	No	No	No	No	XP_003245192.1	--	--	K15191
XLOC_001348	DEG(M)	DEG(M)	DEG(I+M)	DEG(M)	XP_008912007.1	--	J	K02868
XLOC_001351	DEG(M)	No	No	No	--	--	--	--
XLOC_001362	No	No	No	No	CEL57818.1	--	C	--
XLOC_001363	No	No	No	No	--	--	--	--
XLOC_001364	No	No	No	No	--	--	--	--
XLOC_001365	No	No	No	No	--	--	--	--
XLOC_001366	No	No	No	No	CCO29598.1	--	A	--
XLOC_001367	DEG(M)	No	No	No	--	--	C	--
XLOC_001373	No	No	No	No	--	--	--	--
XLOC_001374	No	No	No	No	--	--	--	--
XLOC_001375	No	No	No	No	CCO29515.1	--	--	--
XLOC_001376	No	No	No	No	--	--	--	--
XLOC_001377	No	No	No	No	--	--	--	--
XLOC_001378	No	No	No	No	XP_003048582.1	--	J	--
XLOC_001383	DEG(M)	No	No	No	XP_007264972.1	Trihelix	G	--
XLOC_001387	No	No	No	No	--	--	--	--

**Differentially expressed gene (DEG) IDs**	***C. microphyllum*** **root and shoot-specific (>2 fold)**	***C. microphyllum*** **root and shoot-specific (>10 fold)**	**ICC4958 and** ***C. microphyllum*** **root-specific (>2 fold)**	***C. microphyllum*** **root-specific (>2 fold)**	**Functional Annotation**			
					**NCBI nr IDs**	**TFs**	**KOG**	**KEGG**
XLOC_001388	No	No	No	No	NP_001156153.1	--	--	--
XLOC_001389	No	No	No	No	XP_002946634.1	--	J	K02989
XLOC_001391	No	No	No	No	--	--	--	--
XLOC_001393	No	No	No	No	--	--	--	--
XLOC_001394	No	No	No	No	--	--	--	--
XLOC_001396	No	No	No	No	--	--	--	--
XLOC_001397	No	No	No	No	XP_003044389.1	--	--	--
XLOC_001398	DEG(M)	No	No	No	CCO28404.1	--	--	--
XLOC_001400	No	No	No	No	--	--	A	--
XLOC_001401	No	No	No	No	XP_008904930.1	--	--	K17080
XLOC_001402	DEG(M)	DEG(M)	DEG(I+M)	DEG(M)	--	--	--	--
XLOC_001404	No	No	No	No	ACT10281.1	--	--	K08145
XLOC_001406	DEG(M)	No	No	No	ELU40173.1	--	E	--
XLOC_001417	DEG(M)	No	No	No	KEP52948.1	--	--	--
XLOC_001418	No	No	DEG(I+M)	No	--	--	--	--
XLOC_001419	No	No	No	No	--	--	--	--
XLOC_001421	No	No	No	No	XP_008917255.1	--	H	--
XLOC_001422	No	No	No	No	--	--	--	--
XLOC_001423	No	No	No	No	EUC56198.1	--	--	--
XLOC_001424	No	No	No	No	EWZ46531.1	--	--	--
XLOC_001425	No	No	No	No	--	--	--	--
XLOC_001429	No	No	No	No	--	--	--	--
XLOC_001430	DEG(M)	No	No	No	--	--	--	--
XLOC_001432	No	No	No	No	EUC62911.1	--	--	--
XLOC_001436	No	No	No	No	--	--	--	--
XLOC_001437	No	No	No	No	--	SBP	--	--
XLOC_001439	No	No	No	No	CEL60736.1	--	--	--
XLOC_001442	DEG(M)	No	No	No	CEL61894.1	--	--	--
XLOC_001443	No	No	DEG(I+M)	No	--	--	--	--
XLOC_001446	No	No	No	No	XP_001843221.1	--	Z	--
XLOC_001448	No	No	No	No	XP_003051234.1	--	--	--
XLOC_001449	No	No	No	No	XP_004510115.1	--	--	--
XLOC_001451	No	No	No	No	--	--	--	--
XLOC_001453	No	No	No	No	--	--	--	K02935
XLOC_001454	DEG(M)	No	No	No	--	--	--	--
XLOC_001463	DEG(M)	No	No	No	--	--	--	--
XLOC_001464	No	No	No	No	--	--	J	K02952
XLOC_001468	No	No	No	No	XP_002511389.1	--	P	--
XLOC_001469	No	No	No	No	XP_001654996.1	--	C	--
XLOC_001470	No	No	DEG(I+M)	No	--	--	--	--

**Differentially expressed gene (DEG) IDs**	***C. microphyllum* root and shoot-specific (>2 fold)**	***C. microphyllum* root and shoot-specific (>10 fold)**	**ICC4958 and *C. microphyllum* root-specific (>2 fold)**	***C. microphyllum* root-specific (>2 fold)**	**Functional Annotation**			
					**NCBI nr IDs**	**TFs**	**KOG**	**KEGG**
XLOC_001474	No	No	No	No	--	--	--	--
XLOC_001476	DEG(M)	No	No	No	--	--	--	--
XLOC_001479	No	No	DEG(I+M)	No	XP_004488571.1	--	--	--
XLOC_001480	No	No	No	No	XP_002512298.1	--	--	K13832
XLOC_001481	DEG(M)	No	No	No	--	--	--	--
XLOC_001482	No	No	No	No	XP_003051243.1	--	--	--
XLOC_001485	No	No	No	No	ETL98140.1	--	--	--
XLOC_001489	No	No	DEG(I+M)	No	XP_004512922.1	NAC	--	--
XLOC_001495	No	No	No	No	CEL61872.1	--	C	K00413
XLOC_001501	No	No	No	No	XP_001651423.1	--	G	--
XLOC_001503	No	No	No	No	--	--	K	--
XLOC_001504	No	No	No	No	--	--	--	--
XLOC_001505	No	No	No	No	--	--	--	K14944
XLOC_001506	No	No	No	No	KDN50453.1	--	--	--
XLOC_001507	No	No	DEG(I+M)	No	--	--	G	--
XLOC_001508	No	No	No	No	--	--	--	--
XLOC_001510	No	No	No	No	XP_008905368.1	--	C	--
XLOC_001513	No	No	No	No	XP_003242168.1	--	--	--
XLOC_001522	DEG(M)	No	No	No	XP_003052107.1	--	J	--
XLOC_001523	DEG(M)	No	No	No	--	--	--	--
XLOC_001524	No	No	No	No	--	--	--	--
XLOC_001526	No	No	No	No	--	--	--	--
XLOC_001528	No	No	DEG(I+M)	No	XP_004489476.1	--	--	--
XLOC_001529	No	No	No	No	KDN50734.1	--	--	--
XLOC_001530	No	No	No	No	--	--	--	--
XLOC_001532	No	No	No	No	XP_004516688.1	--	--	--
XLOC_001537	DEG(M)	No	No	No	--	--	--	--
XLOC_001538	DEG(M)	DEG(M)	No	No	XP_003046089.1	MYB	E	--
XLOC_001540	DEG(M)	DEG(M)	DEG(I+M)	DEG(M)	--	--	--	--
XLOC_001541	DEG(M)	No	No	No	XP_003050613.1	--	--	--
XLOC_001542	DEG(M)	No	No	No	CCT74483.1	--	--	--
XLOC_001543	No	No	No	No	--	--	--	--
XLOC_001552	DEG(M)	No	No	No	--	--	--	--
XLOC_001554	No	No	No	No	XP_002529002.1	--	J	K14824
XLOC_001555	No	No	No	No	--	--	--	--
XLOC_001556	No	No	No	No	--	--	--	--
XLOC_001559	No	No	No	No	--	--	J	K03231
XLOC_001561	No	No	No	No	--	--	--	--
XLOC_001566	No	No	No	No	--	--	--	--
XLOC_001568	No	No	No	No	--	--	--	--

Differentially expressed gene (DEG) IDs	*C. microphyllum* root and shoot-specific (>2 fold)	*C. microphyllum* root and shoot-specific (>10 fold)	ICC4958 and *C. microphyllum* root-specific (>2 fold)	*C. microphyllum* root-specific (>2 fold)	Functional Annotation			
					NCBI nr IDs	TFs	KOG	KEGG
XLOC_001576	No	No	No	No	CCO28312.1	--	--	--
XLOC_001584	DEG(M)	No	No	No	--	--	--	--
XLOC_001594	No	No	No	No	KDN39552.1	--	J	--
XLOC_001614	No	No	No	No	--	--	--	--
XLOC_001625	No	No	No	No	--	--	--	--
XLOC_001637	No	No	No	No	--	--	--	K01920
XLOC_001638	DEG(M)	No	No	No	--	--	--	--
XLOC_001640	DEG(M)	No	No	No	--	--	--	--
XLOC_001641	DEG(M)	No	No	No	--	--	--	--
XLOC_001648	DEG(M)	No	No	No	KDN44809.1	--	C	K01507
XLOC_001649	DEG(M)	No	No	No	CEL54255.1	--	C	--
XLOC_001657	No	No	No	No	XP_003053447.1	--	--	K14404
XLOC_001658	No	No	No	No	--	--	--	--
XLOC_001659	DEG(M)	No	No	No	--	--	--	--
XLOC_001660	No	No	No	No	--	--	--	--
XLOC_001661	DEG(M)	DEG(M)	DEG(I+M)	DEG(M)	XP_002484510.1	--	--	--
XLOC_001662	No	No	No	No	--	--	--	--
XLOC_001663	No	No	No	No	--	--	--	--
XLOC_001665	DEG(M)	No	No	No	--	--	--	--
XLOC_001666	No	No	No	No	--	--	--	--
XLOC_001670	No	No	No	No	KEP51491.1	--	--	--
XLOC_001675	No	No	No	No	--	--	--	--
XLOC_001681	No	No	No	No	XP_008912890.1	--	--	--
XLOC_001683	No	No	No	No	--	--	--	--
XLOC_001684	No	No	No	No	XP_003045654.1	--	--	--
XLOC_001688	No	No	No	No	--	--	O	K06630
XLOC_001690	No	No	No	No	AFY99037.1	--	--	--
XLOC_001693	No	No	No	No	KEP52552.1	--	--	--
XLOC_001694	DEG(M)	No	No	No	CCO32342.1	--	--	--
XLOC_001699	No	No	No	No	--	--	--	--
XLOC_001700	No	No	No	No	EWZ33365.1	--	--	--
XLOC_001701	No	No	No	No	ENH66489.1	--	--	--
XLOC_001702	No	No	No	No	--	--	--	--
XLOC_001703	DEG(M)	No	No	No	XP_008906957.1	--	--	--
XLOC_001705	DEG(M)	DEG(M)	No	No	ACJ84004.1	--	O	K00799
XLOC_001710	DEG(M)	DEG(M)	No	No	--	--	--	--
XLOC_001712	No	No	No	No	--	--	--	--
XLOC_001713	No	No	No	No	KEP49393.1	--	--	--
XLOC_001714	No	No	No	No	--	--	--	--
XLOC_001719	DEG(M)	No	No	No	XP_004333640.1	--	O	--

**Differentially expressed gene (DEG) IDs**	***C. microphyllum* root and shoot-specific (>2 fold)**	***C. microphyllum* root and shoot-specific (>10 fold)**	**ICC4958 and *C. microphyllum* root-specific (>2 fold)**	***C. microphyllum* root-specific (>2 fold)**	**Functional Annotation**			
					**NCBI nr IDs**	**TFs**	**KOG**	**KEGG**
XLOC_001720	No	No	No	No	--	--	--	--
XLOC_001722	No	No	No	No	XP_003246360.1	--	Q	--
XLOC_001724	No	No	No	No	--	--	--	--
XLOC_001728	No	No	No	No	--	--	--	--
XLOC_001729	DEG(M)	No	No	No	EGU87943.1	--	--	--
XLOC_001734	No	No	No	No	CEL55739.1	--	DO	--
XLOC_001736	No	No	No	No	--	--	--	--
XLOC_001738	No	No	No	No	--	--	--	--
XLOC_001743	No	No	No	No	--	--	--	--
XLOC_001749	No	No	No	No	XP_003052684.1	--	--	--
XLOC_001750	No	No	No	No	ETM48417.1	--	--	--
XLOC_001754	DEG(M)	No	No	No	--	--	J	--
XLOC_001755	No	No	No	No	--	--	--	--
XLOC_001756	No	No	No	No	--	SAP	--	--
XLOC_001759	DEG(M)	No	No	No	--	--	--	K00799
XLOC_001760	DEG(M)	No	No	No	KDN38664.1	--	--	--
XLOC_001763	No	No	No	No	CCO36707.1	--	O	--
XLOC_001767	No	No	No	No	--	--	--	--
XLOC_001768	No	No	No	No	--	--	--	--
XLOC_001771	No	No	No	No	BAJ91724.1	--	--	--
XLOC_001772	No	No	No	No	--	--	--	--
XLOC_001775	No	No	No	No	--	--	--	K16365
XLOC_001776	No	No	No	No	ABP35661.1	--	--	--
XLOC_001777	No	No	No	No	KDN34509.1	--	--	--
XLOC_001780	DEG(M)	DEG(M)	No	No	--	--	--	--
XLOC_001781	DEG(M)	No	No	No	--	--	--	--
XLOC_001782	DEG(M)	No	DEG(I+M)	DEG(M)	--	--	--	--
XLOC_001784	No	No	No	No	--	--	--	--
XLOC_001785	No	No	No	No	ETL81617.1	--	--	--
XLOC_001786	No	No	No	No	XP_003043296.1	--	--	--
XLOC_001789	No	No	No	No	--	--	--	--
XLOC_001794	No	No	No	No	XP_008896078.1	--	--	--
XLOC_001795	No	No	No	No	--	--	--	--
XLOC_001796	No	No	No	No	--	--	O	K02727
XLOC_001797	No	No	No	No	--	--	--	--
XLOC_001798	DEG(M)	DEG(M)	DEG(I+M)	DEG(M)	KDN38350.1	--	--	--
XLOC_001802	DEG(M)	No	No	No	CDY59177.1	WRKY	J	--
XLOC_001803	No	No	No	No	CEL52070.1	--	O	K11884
XLOC_001807	No	No	No	No	CCO26193.1	--	--	--
XLOC_001808	No	No	No	No	XP_001652269.1	--	J	K03248

Differentially expressed gene (DEG) IDs	*C. microphyllum* root and shoot-specific (>2 fold)	*C. microphyllum* root and shoot-specific (>10 fold)	ICC4958 and *C. microphyllum* root-specific (>2 fold)	*C. microphyllum* root-specific (>2 fold)	Functional Annotation			
					NCBI nr IDs	TFs	KOG	KEGG
XLOC_001809	No	No	No	No	--	--	--	--
XLOC_001812	No	No	No	No	--	--	--	--
XLOC_001814	No	No	No	No	--	--	--	--
XLOC_001816	No	No	No	No	EUC67349.1	--	--	--
XLOC_001832	No	No	No	No	NP_001155679.1	--	C	K03661
XLOC_001834	No	No	No	No	XP_001951599.1	--	J	--
XLOC_001838	DEG(M)	DEG(M)	No	No	--	--	--	--
XLOC_001839	No	No	No	No	--	--	--	--
XLOC_001844	No	No	No	No	XP_002899864.1	--	C	--
XLOC_001845	No	No	No	No	EMT65664.1	--	--	--
XLOC_001846	No	No	No	No	--	--	R	--
XLOC_001848	DEG(M)	DEG(M)	No	No	--	--	--	--
XLOC_001849	DEG(M)	No	No	No	--	--	--	--
XLOC_001850	No	No	No	No	XP_008895547.1	--	--	--
XLOC_001852	No	No	No	No	AHW46052.1	--	X	--
XLOC_001853	No	No	No	No	--	--	--	--
XLOC_001855	No	No	No	No	--	--	--	--
XLOC_001857	No	No	No	No	--	--	--	--
XLOC_001858	No	No	No	No	XP_003043017.1	--	--	--
XLOC_001859	DEG(M)	DEG(M)	No	No	EGU80099.1	--	--	--
XLOC_001863	No	No	No	No	--	--	--	--
XLOC_001864	No	No	No	No	--	--	--	--
XLOC_001867	DEG(M)	DEG(M)	DEG(I+M)	DEG(M)	--	--	--	--
XLOC_001869	No	No	No	No	KDN49143.1	--	--	--
XLOC_001870	No	No	No	No	--	--	--	--
XLOC_001871	DEG(M)	DEG(M)	No	No	--	--	--	--
XLOC_001874	No	No	No	No	XP_001949253.1	--	--	--
XLOC_001876	No	No	No	No	--	--	--	--
XLOC_001878	No	No	No	No	XP_002513266.1	--	--	--
XLOC_001880	DEG(M)	DEG(M)	No	No	XP_003053731.1	--	C	--
XLOC_001885	No	No	No	No	XP_002533247.1	--	O	K06688
XLOC_001887	DEG(M)	No	No	No	ETP51083.1	--	--	--
XLOC_001888	DEG(M)	DEG(M)	DEG(I+M)	DEG(M)	--	--	--	--
XLOC_001893	No	No	No	No	AHX71991.1	--	--	--
XLOC_001894	No	No	No	No	CCO32752.1	--	--	--
XLOC_001897	No	No	No	No	XP_008893577.1	--	--	K02150
XLOC_001898	No	No	No	No	--	--	--	--
XLOC_001899	No	No	No	No	--	--	--	--
XLOC_001902	No	No	No	No	--	--	R	--
XLOC_001903	No	No	No	No	--	--	--	--

Differentially expressed gene (DEG) IDs	***C. microphyllum* root and shoot-specific (>2 fold)**	***C. microphyllum* root and shoot-specific (>10 fold)**	**ICC4958 and *C. microphyllum* root-specific (>2 fold)**	***C. microphyllum* root-specific (>2 fold)**	**Functional Annotation**			
					**NCBI nr IDs**	**TFs**	**KOG**	**KEGG**
XLOC_001905	No	No	No	No	--	--	--	--
XLOC_001906	DEG(M)	No	No	No	AAU84931.1	--	J	K02941
XLOC_001909	No	No	No	No	XP_008912460.1	--	--	--
XLOC_001910	No	No	No	No	XP_004487295.1	--	M	--
XLOC_001916	No	No	No	No	--	--	--	--
XLOC_001919	No	No	No	No	XP_001659555.1	--	--	--
XLOC_001920	No	No	No	No	XP_003049769.1	--	--	--
XLOC_001922	No	No	No	No	--	--	O	--
XLOC_001923	No	No	No	No	--	--	--	--
XLOC_001925	No	No	No	No	--	--	--	--
XLOC_001928	No	No	No	No	--	--	H	K03428
XLOC_001930	DEG(M)	No	No	No	--	--	--	--
XLOC_001933	No	No	No	No	ETP42198.1	--	--	--
XLOC_001935	No	No	No	No	--	--	--	--
XLOC_001936	No	No	No	No	--	--	--	--
XLOC_001937	No	No	No	No	KFM62271.1	--	YU	K14290
XLOC_001938	No	No	No	No	--	--	--	--
XLOC_001940	No	No	No	No	XP_002898325.1	--	C	--
XLOC_001942	No	No	No	No	--	--	--	--
XLOC_001943	No	No	No	No	--	--	--	--
XLOC_001945	No	No	No	No	--	--	--	--
XLOC_001946	No	No	No	No	--	--	--	--
XLOC_001947	No	No	No	No	--	--	--	--
XLOC_001951	DEG(M)	No	No	No	--	--	--	--
XLOC_001952	No	No	No	No	--	--	--	--
XLOC_001954	DEG(M)	No	No	No	AHZ64883.1	--	--	--
XLOC_001955	No	No	No	No	--	--	--	--
XLOC_001956	No	No	No	No	--	--	--	--
XLOC_001959	No	No	No	No	XP_002531589.1	--	J	K03252
XLOC_001960	No	No	No	No	KEP51604.1	--	--	K14001
XLOC_001964	No	No	No	No	--	--	S	--
XLOC_001966	No	No	No	No	--	--	--	--
XLOC_001969	No	No	No	No	--	--	--	--
XLOC_001970	No	No	No	No	--	--	--	--
XLOC_001971	DEG(M)	No	No	No	AAV31409.1	--	--	--
XLOC_001972	No	No	No	No	--	--	--	--
XLOC_001974	No	No	No	No	KDN49439.1	--	--	K08776
XLOC_001978	No	No	No	No	--	--	--	--
XLOC_001980	No	No	No	No	EUC56864.1	--	O	K02739
XLOC_001981	No	No	No	No	XP_012492357.1	--	O	K09569

Differentially expressed gene (DEG) IDs	*C. microphyllum* root and shoot-specific (>2 fold)	*C. microphyllum* root and shoot-specific (>10 fold)	ICC4958 and *C. microphyllum* root-specific (>2 fold)	*C. microphyllum* root-specific (>2 fold)	Functional Annotation			
					NCBI nr IDs	TFs	KOG	KEGG
XLOC_001989	No	No	No	No	AGZ87461.1	--	--	--
XLOC_001990	No	No	No	No	NP_001232994.1	--	--	--
XLOC_001996	No	No	No	No	--	--	--	--
XLOC_001997	No	No	No	No	XP_001949775.1	--	--	--
XLOC_002002	No	No	No	No	NP_001155556.1	--	U	K07936
XLOC_002003	DEG(M)	No	No	No	--	--	--	--
XLOC_002004	No	No	No	No	ETM49507.1	--	Q	--
XLOC_002008	No	No	No	No	--	--	--	--
XLOC_002014	DEG(M)	No	No	No	--	--	--	--
XLOC_002017	No	No	No	No	NP_001156047.1	--	--	--
XLOC_002018	No	No	No	No	ETP38962.1	--	P	--
XLOC_002019	DEG(M)	No	No	No	--	--	--	K15378
XLOC_002023	No	No	No	No	XP_003041092.1	--	--	--
XLOC_002024	No	No	No	No	ETL81917.1	--	--	K01885
XLOC_002025	No	No	No	No	--	--	O	K10583
XLOC_002026	No	No	No	No	EUC67117.1	--	--	--
XLOC_002027	No	No	No	No	--	--	L	K03163
XLOC_002029	No	No	No	No	XP_009529905.1	--	O	K02728
XLOC_002031	No	No	DEG(I+M)	No	--	--	--	--
XLOC_002035	No	No	No	No	--	--	--	--
XLOC_002036	No	No	No	No	NP_001155754.1	--	O	K10573
XLOC_002037	DEG(M)	No	No	No	--	--	--	--
XLOC_002038	No	No	No	No	--	--	--	--
XLOC_002040	No	No	No	No	--	--	--	--
XLOC_002041	DEG(M)	No	DEG(I+M)	DEG(M)	--	--	--	--
XLOC_002042	No	No	No	No	--	--	--	--
XLOC_002043	DEG(M)	No	No	No	KDN43642.1	--	--	--
XLOC_002044	DEG(M)	No	No	No	KDN35740.1	--	--	--
XLOC_002045	DEG(M)	No	No	No	--	--	--	--
XLOC_002046	No	No	No	No	--	--	OR	--
XLOC_002069	No	No	DEG(I+M)	No	--	--	--	K05953
XLOC_002075	DEG(M)	No	No	No	EUC56995.1	--	--	--
XLOC_002077	DEG(M)	No	No	No	--	--	--	--
XLOC_002078	DEG(M)	No	No	No	--	--	--	--
XLOC_002089	No	No	No	No	KEP55737.1	--	--	--
XLOC_002090	No	No	No	No	EUC67500.1	--	--	K02934
XLOC_002091	DEG(M)	No	No	No	CEL59326.1	--	--	K02934
XLOC_002094	No	No	No	No	--	--	--	K14484
XLOC_002104	No	No	No	No	KIO28679.1	--	--	--
XLOC_002105	No	No	No	No	--	--	--	--

Differentially expressed gene (DEG) IDs	*C. microphyllum* root and shoot-specific (>2 fold)	*C. microphyllum* root and shoot-specific (>10 fold)	ICC4958 and *C. microphyllum* root-specific (>2 fold)	*C. microphyllum* root-specific (>2 fold)	Functional Annotation			
					NCBI nr IDs	TFs	KOG	KEGG
XLOC_002113	DEG(M)	DEG(M)	DEG(I+M)	DEG(M)	--	--	--	--
XLOC_002118	No	No	No	No	--	--	--	--
XLOC_002120	No	No	DEG(I+M)	No	--	--	--	K14802
XLOC_002126	No	No	No	No	--	--	--	--
XLOC_002127	DEG(M)	DEG(M)	DEG(I+M)	DEG(M)	--	--	--	--
XLOC_002141	No	No	DEG(I+M)	No	XP_004487804.1	--	--	--
XLOC_002142	DEG(M)	No	No	No	--	--	--	--
XLOC_002143	DEG(M)	No	DEG(I+M)	DEG(M)	--	--	--	--
XLOC_002144	DEG(M)	No	DEG(I+M)	DEG(M)	XP_004508175.1	--	--	--
XLOC_002145	DEG(M)	DEG(M)	DEG(I+M)	DEG(M)	--	--	--	--
XLOC_002153	DEG(M)	No	No	No	EUC56860.1	--	--	--
XLOC_002156	No	No	No	No	--	--	E	--
XLOC_002157	No	No	No	No	--	bHLH	--	--
XLOC_002159	No	No	DEG(I+M)	No	XP_004505065.1	--	--	--
XLOC_002172	DEG(M)	No	DEG(I+M)	DEG(M)	--	--	--	--
XLOC_002187	No	No	No	No	--	--	--	--
XLOC_002190	No	No	No	No	--	--	--	--
XLOC_002191	No	No	No	No	--	--	--	--
XLOC_002199	DEG(M)	No	No	No	--	--	--	--
XLOC_002201	No	No	No	No	--	--	--	K02437
XLOC_002209	No	No	DEG(I+M)	No	XP_004486171.1	--	--	--
XLOC_002212	No	No	No	No	XP_003045241.1	--	--	--
XLOC_002213	No	No	No	No	ETI39978.1	--	--	--
XLOC_002216	DEG(M)	No	No	No	--	--	--	--
XLOC_002222	No	No	No	No	--	--	--	--
XLOC_002224	DEG(M)	DEG(M)	DEG(I+M)	DEG(M)	XP_008890949.1	--	J	K02894
XLOC_002227	No	No	No	No	KEP55034.1	--	--	--
XLOC_002228	No	No	No	No	--	--	--	--
XLOC_002229	No	No	DEG(I+M)	No	--	--	--	--
XLOC_002230	No	No	No	No	EXA39773.1	--	--	--
XLOC_002233	No	No	No	No	CCO33921.1	--	--	--
XLOC_002236	No	No	No	No	--	--	--	--
XLOC_002239	No	No	No	No	XP_001663513.1	--	--	--
XLOC_002240	DEG(M)	DEG(M)	DEG(I+M)	DEG(M)	ACA28683.1	--	Z	--
XLOC_002241	DEG(M)	No	DEG(I+M)	DEG(M)	ETM37694.1	--	--	--
XLOC_002247	No	No	No	No	--	--	--	--
XLOC_002248	No	No	No	No	ETL39071.1	--	--	--
XLOC_002249	No	No	No	No	--	--	--	--
XLOC_002250	No	No	No	No	XP_009530443.1	--	C	K03522
XLOC_002253	No	No	No	No	CCO28201.1	--	--	--

Differentially expressed gene (DEG) IDs	*C. microphyllum* root and shoot-specific (>2 fold)	*C. microphyllum* root and shoot-specific (>10 fold)	ICC4958 and *C. microphyllum* root-specific (>2 fold)	*C. microphyllum* root-specific (>2 fold)	Functional Annotation			
					NCBI nr IDs	TFs	KOG	KEGG
XLOC_002258	DEG(M)	No	No	No	--	--	--	--
XLOC_002273	No	No	No	No	XP_002896957.1	--	--	K01939
XLOC_002277	No	No	No	No	KDN47635.1	--	--	--
XLOC_002279	DEG(M)	No	No	No	--	--	--	--
XLOC_002281	No	No	No	No	--	--	--	--
XLOC_002282	DEG(M)	No	No	No	--	--	--	--
XLOC_002310	DEG(M)	No	No	No	KDN33568.1	--	--	--
XLOC_002313	No	No	No	No	--	--	--	--
XLOC_002314	DEG(M)	DEG(M)	DEG(I+M)	DEG(M)	--	--	--	--
XLOC_002326	No	No	No	No	--	--	--	K10268
XLOC_002327	DEG(M)	No	DEG(I+M)	DEG(M)	--	--	--	--
XLOC_002333	DEG(M)	No	No	No	--	--	--	--
XLOC_002348	No	No	No	No	KDN40906.1	--	--	--
XLOC_002352	DEG(M)	No	No	No	XP_002999240.1	--	--	--
XLOC_002353	DEG(M)	No	No	No	AAN31470.1	--	P	--
XLOC_002354	DEG(M)	No	No	No	XP_008892299.1	--	P	--
XLOC_002355	DEG(M)	No	No	No	ETL95939.1	--	--	--
XLOC_002356	DEG(M)	No	No	No	--	--	--	--
XLOC_002368	DEG(M)	No	No	No	--	--	--	--
XLOC_002373	No	No	DEG(I+M)	No	XP_004502599.1	--	--	--
XLOC_002379	DEG(M)	DEG(M)	No	No	--	--	--	--
XLOC_002380	DEG(M)	No	No	No	--	--	--	--
XLOC_002389	DEG(M)	No	DEG(I+M)	DEG(M)	--	--	--	--
XLOC_002391	No	No	No	No	XP_012571416.1	--	--	--
XLOC_002393	DEG(M)	DEG(M)	No	No	--	--	--	--
XLOC_002394	DEG(M)	DEG(M)	No	No	--	--	R	--
XLOC_002405	No	No	No	No	CEL52530.1	--	--	--
XLOC_002406	No	No	No	No	EUC57457.1	--	--	--
XLOC_002407	DEG(M)	No	No	No	XP_004513333.1	--	--	--
XLOC_002411	DEG(M)	No	No	No	BAH70616.1	YABBY	OR	--
XLOC_002412	DEG(M)	No	No	No	BAH71115.1	YABBY	OR	K08770
XLOC_002413	DEG(M)	DEG(M)	No	No	BAH71115.1	YABBY	OR	--
XLOC_002414	DEG(M)	No	No	No	AAC39466.1	YABBY	OR	--
XLOC_002417	DEG(M)	DEG(M)	DEG(I+M)	DEG(M)	CAI77899.1	YABBY	OR	--
XLOC_002418	DEG(M)	No	No	No	KFQ96542.1	YABBY	J	--
XLOC_002419	DEG(M)	No	No	No	BAE48510.1	YABBY	OR	--
XLOC_002424	DEG(M)	No	No	No	XP_011278194.1	YABBY	OR	--
XLOC_002427	DEG(M)	No	No	No	ELU44102.1	YABBY	J	K02977
XLOC_002429	DEG(M)	No	DEG(I+M)	DEG(M)	EJU03816.1	YABBY	J	K02977
XLOC_002438	DEG(M)	No	No	No	XP_002903189.1	--	T	K14753

Differentially expressed gene (DEG) IDs	*C. microphyllum* root and shoot-specific (>2 fold)	*C. microphyllum* root and shoot-specific (>10 fold)	ICC4958 and *C. microphyllum* root-specific (>2 fold)	*C. microphyllum* root-specific (>2 fold)	Functional Annotation			
					NCBI nr IDs	TFs	KOG	KEGG
XLOC_002441	No	No	DEG(I+M)	No	--	--	T	--
XLOC_002442	DEG(M)	No	DEG(I+M)	DEG(M)	XP_004515597.1	--	--	--
XLOC_002456	DEG(M)	No	No	No	CEL54337.1	--	--	--
XLOC_002457	No	No	No	No	EUC59209.1	--	--	K08054
XLOC_002458	DEG(M)	No	No	No	EUC59209.1	--	--	K08054
XLOC_002459	DEG(M)	No	No	No	KEP50300.1	--	--	K01209
XLOC_002468	No	No	No	No	--	--	--	--
XLOC_002480	DEG(M)	No	No	No	--	--	--	--
XLOC_002481	DEG(M)	No	No	No	--	--	--	--
XLOC_002488	DEG(M)	No	No	No	EUC54176.1	--	--	--
XLOC_002489	DEG(M)	No	No	No	EUC54176.1	--	--	--
XLOC_002497	No	No	No	No	CEL52460.1	--	U	K07953
XLOC_002498	No	No	No	No	--	--	O	--
XLOC_002499	No	No	No	No	--	--	O	K09493
XLOC_002504	No	No	No	No	ETO72884.1	--	--	K03943
XLOC_002505	No	No	No	No	EUC58918.1	--	--	--
XLOC_002508	No	No	No	No	--	--	--	--
XLOC_002509	No	No	No	No	XP_002899896.1	--	--	--
XLOC_002513	No	No	No	No	--	--	--	--
XLOC_002516	No	No	No	No	--	--	--	--
XLOC_002517	No	No	No	No	--	--	--	--
XLOC_002519	No	No	No	No	--	--	--	--
XLOC_002522	No	No	No	No	--	--	--	--
XLOC_002523	No	No	No	No	--	--	--	--
XLOC_002526	No	No	No	No	--	--	O	--
XLOC_002538	No	No	DEG(I+M)	No	--	--	--	--
XLOC_002542	DEG(M)	No	No	No	XP_003048445.1	--	--	--
XLOC_002545	DEG(M)	No	No	No	EFO27996.2	NAC	J	K02976
XLOC_002550	No	No	No	No	XP_002527127.1	--	--	--
XLOC_002552	No	No	DEG(I+M)	No	XP_004516834.1	--	--	--
XLOC_002553	DEG(M)	No	No	No	CCO38172.1	--	--	--
XLOC_002555	No	No	No	No	CCO33734.1	--	--	--
XLOC_002556	No	No	No	No	--	--	--	--
XLOC_002557	No	No	No	No	--	--	--	--
XLOC_002559	DEG(M)	No	No	No	XP_004348414.1	YABBY	J	--
XLOC_002563	No	No	No	No	KIO27812.1	--	O	--
XLOC_002568	DEG(M)	No	No	No	XP_008894032.1	--	--	--
XLOC_002576	No	No	No	No	--	--	--	--
XLOC_002580	No	No	No	No	NP_001156136.1	--	--	K02732
XLOC_002583	No	No	No	No	--	--	--	K01183

Differentially expressed gene (DEG) IDs	*C. microphyllum* root and shoot-specific (>2 fold)	*C. microphyllum* root and shoot-specific (>10 fold)	ICC4958 and *C. microphyllum* root-specific (>2 fold)	*C. microphyllum* root-specific (>2 fold)	Functional Annotation			
					NCBI nr IDs	TFs	KOG	KEGG
XLOC_002585	No	No	No	No	KDN47495.1	--	--	--
XLOC_002588	No	No	No	No	--	--	--	K00008
XLOC_002589	No	No	No	No	--	--	--	--
XLOC_002592	No	No	No	No	--	--	--	--
XLOC_002593	No	No	No	No	EUC59564.1	--	--	--
XLOC_002594	No	No	No	No	--	--	--	--
XLOC_002595	DEG(M)	No	No	No	--	--	--	--
XLOC_002597	No	No	No	No	--	--	--	K01166
XLOC_002598	No	No	No	No	KDN37178.1	--	--	--
XLOC_002599	No	No	No	No	--	--	E	--
XLOC_002600	No	No	No	No	CCO27611.1	--	O	K03178
XLOC_002601	No	No	No	No	--	--	--	--
XLOC_002604	No	No	No	No	XP_001651207.1	--	--	--
XLOC_002606	No	No	No	No	NP_001280351.1	M-type	O	--
XLOC_002611	No	No	No	No	CEL60799.1	--	--	--
XLOC_002612	No	No	No	No	KDN49479.1	--	A	K11097
XLOC_002614	No	No	No	No	--	--	--	--
XLOC_002615	No	No	No	No	CEL55303.1	--	--	--
XLOC_002617	No	No	No	No	--	--	--	--
XLOC_002619	No	No	No	No	--	--	C	--
XLOC_002624	No	No	No	No	EUC62861.1	--	--	--
XLOC_002625	No	No	No	No	CEL53083.1	--	--	--
XLOC_002628	No	No	No	No	--	--	K	--
XLOC_002629	No	No	No	No	--	--	--	--
XLOC_002630	No	No	No	No	CEL55543.1	--	--	--
XLOC_002631	No	No	DEG(I+M)	No	--	--	--	--
XLOC_002634	No	No	No	No	--	--	E	K16871
XLOC_002635	No	No	No	No	--	--	--	K07399
XLOC_002637	No	No	No	No	--	--	--	--
XLOC_002638	No	No	No	No	XP_012573103.1	--	RT	--
XLOC_002641	No	No	No	No	AAT09066.1	--	U	K07936
XLOC_002643	No	No	No	No	XP_008895053.1	--	--	--
XLOC_002646	No	No	No	No	--	--	--	K11147
XLOC_002649	DEG(M)	No	No	No	--	--	--	--
XLOC_002651	No	No	No	No	NP_001155596.1	--	Z	--
XLOC_002652	No	No	No	No	--	--	--	K05868
XLOC_002656	No	No	DEG(I+M)	No	--	--	P	--
XLOC_002658	No	No	No	No	XP_008184035.1	--	--	--
XLOC_002659	No	No	No	No	XP_001950215.1	--	--	--
XLOC_002666	DEG(M)	DEG(M)	No	No	XP_012571226.1	--	--	--

Differentially expressed gene (DEG) IDs	*C. microphyllum* root and shoot-specific (>2 fold)	*C. microphyllum* root and shoot-specific (>10 fold)	ICC4958 and *C. microphyllum* root-specific (>2 fold)	*C. microphyllum* root-specific (>2 fold)	Functional Annotation			
					NCBI nr IDs	TFs	KOG	KEGG
XLOC_002667	DEG(M)	DEG(M)	No	No	XP_012571226.1	--	--	--
XLOC_002668	No	No	No	No	--	--	--	--
XLOC_002670	No	No	No	No	CCO28456.1	--	--	K14652
XLOC_002672	DEG(M)	DEG(M)	DEG(I+M)	DEG(M)	--	--	--	--
XLOC_002673	DEG(M)	DEG(M)	No	No	EUC54118.1	--	--	--
XLOC_002675	DEG(M)	No	No	No	XP_003043397.1	--	--	--
XLOC_002676	No	No	DEG(I+M)	No	XP_004516675.1	--	QI	--
XLOC_002677	DEG(M)	DEG(M)	No	No	--	--	--	--
XLOC_002679	No	No	No	No	--	--	--	--
XLOC_002681	No	No	No	No	AAY56793.1	--	P	K01507
XLOC_002682	No	No	No	No	--	--	--	--
XLOC_002684	No	No	No	No	--	--	--	--
XLOC_002687	No	No	No	No	--	--	J	K03242
XLOC_002689	DEG(M)	No	No	No	XP_001663282.1	--	T	K14753
XLOC_002690	No	No	No	No	XP_001652843.1	--	--	--
XLOC_002692	No	No	No	No	--	--	--	K12666
XLOC_002694	No	No	No	No	CEL59337.1	--	--	--
XLOC_002695	DEG(M)	No	No	No	XP_008909489.1	--	--	K02936
XLOC_002697	No	No	No	No	--	--	--	--
XLOC_002698	No	No	No	No	--	--	--	--
XLOC_002699	No	No	No	No	CCO29373.1	--	--	--
XLOC_002701	DEG(M)	No	No	No	--	--	--	--
XLOC_002702	No	No	No	No	EUC59098.1	--	--	--
XLOC_002704	No	No	No	No	--	--	--	--
XLOC_002706	No	No	No	No	--	--	--	--
XLOC_002707	No	No	No	No	XP_008909259.1	--	--	--
XLOC_002712	No	No	No	No	--	--	--	--
XLOC_002713	No	No	No	No	--	--	--	--
XLOC_002714	No	No	No	No	CCO31166.1	--	UR	--
XLOC_002716	No	No	No	No	XP_008899801.1	--	--	K10527
XLOC_002718	No	No	No	No	--	--	--	--
XLOC_002719	No	No	No	No	--	--	J	K02951
XLOC_002722	No	No	No	No	--	--	--	--
XLOC_002724	No	No	No	No	--	--	--	--
XLOC_002728	No	No	No	No	ENH71064.1	--	--	--
XLOC_002731	DEG(M)	DEG(M)	No	No	XP_003590760.1	--	--	--
XLOC_002735	DEG(M)	No	DEG(I+M)	DEG(M)	--	--	--	--
XLOC_002736	No	No	No	No	EUC63145.1	--	--	--
XLOC_002737	No	No	No	No	XP_001647823.1	--	--	--
XLOC_002740	No	No	No	No	KEP47124.1	--	G	--

Differentially expressed gene (DEG) IDs	*C. microphyllum* root and shoot-specific (>2 fold)	*C. microphyllum* root and shoot-specific (>10 fold)	ICC4958 and *C. microphyllum* root-specific (>2 fold)	*C. microphyllum* root-specific (>2 fold)	Functional Annotation			
					NCBI nr IDs	TFs	KOG	KEGG
XLOC_002742	No	No	No	No	CCO27059.1	--	--	--
XLOC_002748	No	No	No	No	--	--	--	--
XLOC_002752	DEG(M)	No	No	No	--	--	--	--
XLOC_002753	DEG(M)	No	No	No	--	--	--	--
XLOC_002756	DEG(M)	DEG(M)	No	No	--	--	--	--
XLOC_002768	DEG(M)	No	DEG(I+M)	DEG(M)	--	--	--	--
XLOC_002780	DEG(M)	No	No	No	KEP46004.1	NAC	J	K02976
XLOC_002787	No	No	DEG(I+M)	No	XP_004493869.2	ERF	--	K09286
XLOC_002788	DEG(M)	No	DEG(I+M)	DEG(M)	KKA73802.1	--	Z	--
XLOC_002789	DEG(M)	DEG(M)	DEG(I+M)	DEG(M)	KKA73802.1	--	Z	--
XLOC_002790	DEG(M)	DEG(M)	DEG(I+M)	DEG(M)	AAO92429.1	--	Z	--
XLOC_002794	DEG(M)	No	No	No	--	--	--	--
XLOC_002797	No	No	No	No	--	--	I	--
XLOC_002800	DEG(M)	DEG(M)	No	No	--	--	--	--
XLOC_002802	No	No	No	No	XP_003053087.1	--	--	--
XLOC_002805	No	No	No	No	--	--	--	--
XLOC_002811	No	No	No	No	--	--	--	--
XLOC_002815	No	No	No	No	CCO30010.1	--	--	--
XLOC_002825	No	No	No	No	ETL80335.1	--	--	--
XLOC_002829	DEG(M)	No	No	No	--	--	U	--
XLOC_002831	No	No	No	No	--	--	--	--
XLOC_002832	DEG(M)	No	No	No	--	MIKC	J	--
XLOC_002833	No	No	No	No	--	--	J	--
XLOC_002844	No	No	DEG(I+M)	No	--	--	--	--
XLOC_002845	No	No	No	No	XP_008906113.1	--	J	K02358
XLOC_002846	DEG(M)	No	No	No	--	--	--	--
XLOC_002849	No	No	No	No	KDN48087.1	--	--	--
XLOC_002851	DEG(M)	No	No	No	XP_003612068.1	--	--	K08241
XLOC_002853	DEG(M)	DEG(M)	DEG(I+M)	DEG(M)	XP_002670548.1	Dof	Z	--
XLOC_002857	No	No	No	No	KEH37065.1	--	--	--
XLOC_002859	No	No	No	No	XP_008183336.1	--	D	K03347
XLOC_002860	No	No	No	No	--	--	--	--
XLOC_002861	No	No	No	No	--	--	--	--
XLOC_002867	No	No	No	No	--	--	--	--
XLOC_002868	No	No	No	No	--	--	--	--
XLOC_002878	No	No	No	No	EUC56249.1	--	--	--
XLOC_002879	No	No	No	No	--	--	--	--
XLOC_002883	No	No	No	No	--	--	--	--
XLOC_002884	No	No	No	No	XP_008908484.1	--	--	--
XLOC_002886	No	No	No	No	XP_003050736.1	--	--	--

Differentially expressed gene (DEG) IDs	*C. microphyllum* root and shoot-specific (>2 fold)	*C. microphyllum* root and shoot-specific (>10 fold)	ICC4958 and *C. microphyllum* root-specific (>2 fold)	*C. microphyllum* root-specific (>2 fold)	Functional Annotation			
					NCBI nr IDs	TFs	KOG	KEGG
XLOC_002887	No	No	No	No	--	--	O	--
XLOC_002889	No	No	DEG(I+M)	No	XP_004498566.1	WRKY	--	--
XLOC_002890	No	No	No	No	--	--	--	--
XLOC_002891	DEG(M)	DEG(M)	DEG(I+M)	DEG(M)	--	--	--	--
XLOC_002894	No	No	No	No	XP_003240088.1	--	O	K08057
XLOC_002895	DEG(M)	No	No	No	--	--	O	K08057
XLOC_002898	No	No	No	No	--	--	--	K14484
XLOC_002904	No	No	No	No	--	--	--	K17261
XLOC_002906	DEG(M)	No	No	No	--	--	--	--
XLOC_002910	DEG(M)	No	No	No	--	--	--	--
XLOC_002916	No	No	No	No	XP_008188310.1	--	TZR	--
XLOC_002917	No	No	DEG(I+M)	No	XP_004505893.1	--	--	--
XLOC_002918	No	No	No	No	--	--	--	--
XLOC_002919	DEG(M)	No	No	No	--	--	--	--
XLOC_002920	DEG(M)	No	No	No	--	--	--	--
XLOC_002923	DEG(M)	No	No	No	--	--	--	--
XLOC_002929	No	No	No	No	--	--	--	--
XLOC_002933	No	No	No	No	--	--	--	--
XLOC_002939	No	No	DEG(I+M)	No	--	--	--	K14496
XLOC_002940	No	No	No	No	--	--	--	--
XLOC_002941	No	No	No	No	KIM41705.1	--	J	K03263
XLOC_002942	No	No	No	No	KIM41705.1	--	J	K03263
XLOC_002943	No	No	No	No	XP_002901169.1	--	O	K02738
XLOC_002945	No	No	No	No	XP_003048983.1	--	--	--
XLOC_002946	No	No	DEG(I+M)	No	XP_004513441.1	--	--	K00430
XLOC_002947	No	No	No	No	CEL51584.1	--	--	K13800
XLOC_002949	DEG(M)	No	No	No	KFH49050.1	--	U	--
XLOC_002951	No	No	No	No	--	--	--	--
XLOC_002959	No	No	No	No	--	--	--	--
XLOC_002963	No	No	No	No	CEL54733.1	--	--	--
XLOC_002964	No	No	No	No	--	--	--	--
XLOC_002965	No	No	No	No	--	--	--	--
XLOC_002966	DEG(M)	No	No	No	--	--	--	--
XLOC_002968	No	No	No	No	CCO31942.1	--	--	--
XLOC_002977	No	No	No	No	KFM79411.1	--	R	--
XLOC_002985	DEG(M)	No	No	No	--	--	--	--
XLOC_002986	No	No	No	No	CEL59490.1	--	--	--
XLOC_002987	No	No	No	No	XP_004512856.1	--	M	--
XLOC_002989	No	No	No	No	--	--	--	--
XLOC_002991	DEG(M)	No	No	No	--	--	--	--

**Differentially expressed gene (DEG) IDs**	***C. microphyllum*** **root and shoot-specific (>2 fold)**	***C. microphyllum*** **root and shoot-specific (>10 fold)**	**ICC4958 and** ***C. microphyllum*** **root-specific (>2 fold)**	***C. microphyllum*** **root-specific (>2 fold)**	**Functional Annotation**			
					**NCBI nr IDs**	**TFs**	**KOG**	**KEGG**
XLOC_002992	No	No	No	No	CEL51876.1	--	--	--
XLOC_002993	No	No	DEG(I+M)	No	--	--	--	--
XLOC_002999	DEG(M)	No	DEG(I+M)	DEG(M)	XP_002510890.1	--	A	K16911
XLOC_003002	DEG(M)	No	No	No	CEL57935.1	--	--	--
XLOC_003003	No	No	No	No	XP_008188458.1	--	Z	--
XLOC_003004	No	No	No	No	XP_008188458.1	--	Z	--
XLOC_003006	No	No	No	No	--	--	O	K16292
XLOC_003013	No	No	No	No	XP_008905800.1	--	--	--
XLOC_003014	No	No	No	No	--	--	--	K00365
XLOC_003016	No	No	No	No	CEL55583.1	--	--	--
XLOC_003018	DEG(M)	No	No	No	EUC61475.1	--	--	--
XLOC_003021	DEG(M)	No	No	No	XP_003051897.1	--	--	--
XLOC_003022	No	No	No	No	XP_003051897.1	--	--	--
XLOC_003023	DEG(M)	No	No	No	--	--	--	--
XLOC_003026	No	No	No	No	--	--	--	--
XLOC_003027	No	No	No	No	--	--	--	--
XLOC_003029	DEG(M)	No	No	No	EUC65596.1	--	--	K00059
XLOC_003030	No	No	No	No	KEP52135.1	--	--	K00059
XLOC_003031	No	No	DEG(I+M)	No	--	--	QI	--
XLOC_003032	No	No	No	No	XP_004507874.1	--	--	--
XLOC_003033	No	No	No	No	--	--	--	--
XLOC_003040	DEG(M)	No	No	No	--	--	--	--
XLOC_003046	No	No	No	No	--	--	--	--
XLOC_003049	No	No	No	No	--	--	--	--
XLOC_003055	No	No	No	No	AKM28425.1	--	--	--
XLOC_003056	DEG(M)	DEG(M)	No	No	BAD21357.1	--	--	--
XLOC_003057	No	No	No	No	EUC67025.1	--	--	--
XLOC_003062	No	No	No	No	EWG52261.1	--	--	--
XLOC_003065	No	No	No	No	--	--	--	--
XLOC_003067	No	No	No	No	--	--	--	--
XLOC_003069	No	No	No	No	--	--	--	--
XLOC_003073	No	No	No	No	XP_008889631.1	--	--	--
XLOC_003075	No	No	No	No	--	--	--	--
XLOC_003078	DEG(M)	No	No	No	--	--	--	--
XLOC_003083	DEG(M)	No	No	No	--	--	--	--
XLOC_003084	DEG(M)	No	No	No	--	--	--	--
XLOC_003085	No	No	No	No	--	--	--	--
XLOC_003086	No	No	No	No	--	--	--	--
XLOC_003091	DEG(M)	No	No	No	CCO31476.1	--	--	--
XLOC_003101	DEG(M)	DEG(M)	DEG(I+M)	DEG(M)	EGU86327.1	--	--	--

Differentially expressed gene (DEG) IDs	*C. microphyllum* root and shoot-specific (>2 fold)	*C. microphyllum* root and shoot-specific (>10 fold)	ICC4958 and *C. microphyllum* root-specific (>2 fold)	*C. microphyllum* root-specific (>2 fold)	Functional Annotation			
					NCBI nr IDs	TFs	KOG	KEGG
XLOC_003107	DEG(M)	No	No	No	--	--	--	--
XLOC_003113	No	No	No	No	XP_008909584.1	--	--	--
XLOC_003114	No	No	No	No	--	--	--	--
XLOC_003116	No	No	No	No	--	--	--	--
XLOC_003121	No	No	No	No	--	--	--	--
XLOC_003122	No	No	No	No	KEP52750.1	--	--	--
XLOC_003127	DEG(M)	No	No	No	NP_001155753.1	--	J	K02868
XLOC_003129	DEG(M)	No	No	No	--	--	--	--
XLOC_003131	No	No	No	No	--	--	--	--
XLOC_003151	No	No	No	No	NP_001280344.1	--	--	--
XLOC_003152	No	No	No	No	--	--	--	--
XLOC_003154	DEG(M)	DEG(M)	DEG(I+M)	DEG(M)	--	--	--	--
XLOC_003155	DEG(M)	No	DEG(I+M)	DEG(M)	CEL56687.1	--	--	--
XLOC_003157	No	No	No	No	CEL52376.1	--	C	--
XLOC_003158	No	No	No	No	--	--	--	--
XLOC_003161	No	No	No	No	--	--	--	--
XLOC_003162	No	No	No	No	--	--	--	K18749
XLOC_003166	DEG(M)	No	No	No	--	--	J	--
XLOC_003169	No	No	No	No	XP_001652845.1	--	J	--
XLOC_003170	No	No	No	No	--	--	--	K02155
XLOC_003172	No	No	No	No	XP_003053174.1	--	--	--
XLOC_003173	No	No	No	No	ETO64449.1	--	C	--
XLOC_003174	No	No	No	No	ETO64449.1	--	C	K00031
XLOC_003175	DEG(M)	No	No	No	ETO64449.1	--	C	--
XLOC_003179	DEG(M)	No	No	No	KEP49481.1	--	--	--
XLOC_003181	No	No	No	No	XP_009517336.1	--	--	--
XLOC_003183	DEG(M)	No	No	No	--	--	--	--
XLOC_003184	No	No	No	No	XP_008900885.1	--	--	K02734
XLOC_003185	DEG(M)	No	No	No	EXA46522.1	--	--	--
XLOC_003191	No	No	No	No	XP_003046077.1	--	--	--
XLOC_003193	No	No	No	No	KNG46270.1	--	--	--
XLOC_003198	No	No	No	No	--	--	--	--
XLOC_003199	No	No	No	No	--	--	--	--
XLOC_003200	No	No	No	No	XP_008911076.1	--	--	K03695
XLOC_003201	No	No	No	No	--	--	--	--
XLOC_003204	No	No	No	No	--	--	--	--
XLOC_003205	No	No	No	No	--	--	--	--
XLOC_003206	DEG(M)	No	No	No	--	--	--	--
XLOC_003207	DEG(M)	No	No	No	--	--	--	--
XLOC_003210	No	No	No	No	--	--	--	--

Differentially expressed gene (DEG) IDs	*C. microphyllum* root and shoot-specific (>2 fold)	*C. microphyllum* root and shoot-specific (>10 fold)	ICC4958 and *C. microphyllum* root-specific (>2 fold)	*C. microphyllum* root-specific (>2 fold)	Functional Annotation			
					NCBI nr IDs	TFs	KOG	KEGG
XLOC_003211	No	No	No	No	--	--	--	--
XLOC_003212	DEG(M)	DEG(M)	No	No	--	--	--	--
XLOC_003218	No	No	No	No	CEL55318.1	--	R	--
XLOC_003224	DEG(M)	DEG(M)	No	No	--	--	--	--
XLOC_003229	DEG(M)	DEG(M)	No	No	--	--	--	--
XLOC_003231	DEG(M)	No	No	No	--	--	--	--
XLOC_003233	DEG(M)	No	No	No	YP_001649168.1	--	C	--
XLOC_003235	No	No	No	No	--	--	--	--
XLOC_003240	DEG(M)	DEG(M)	DEG(I+M)	DEG(M)	--	--	--	--
XLOC_003241	DEG(M)	No	DEG(I+M)	DEG(M)	--	--	--	--
XLOC_003242	DEG(M)	No	DEG(I+M)	DEG(M)	--	--	--	--
XLOC_003246	DEG(M)	DEG(M)	No	No	--	--	--	--
XLOC_003250	No	No	No	No	CEL56505.1	--	--	--
XLOC_003260	DEG(M)	No	No	No	--	--	--	--
XLOC_003261	No	No	No	No	--	--	--	--
XLOC_003268	No	No	No	No	KNG46714.1	--	--	--
XLOC_003269	No	No	No	No	XP_001934975.1	--	--	--
XLOC_003272	DEG(M)	No	No	No	CEL56560.1	--	--	--
XLOC_003273	DEG(M)	No	No	No	CEL56560.1	--	--	--
XLOC_003274	No	No	No	No	--	--	--	--
XLOC_003282	DEG(M)	No	No	No	--	--	--	--
XLOC_003283	DEG(M)	No	DEG(I+M)	DEG(M)	--	--	MVG	--
XLOC_003294	DEG(M)	No	No	No	--	--	--	--
XLOC_003295	No	No	No	No	--	--	--	--
XLOC_003296	No	No	No	No	EUC66386.1	--	--	--
XLOC_003298	DEG(M)	DEG(M)	No	No	XP_004509433.1	--	--	--
XLOC_003306	No	No	No	No	--	--	--	--
XLOC_003312	No	No	No	No	--	--	--	--
XLOC_003313	No	No	No	No	--	--	--	--
XLOC_003315	No	No	No	No	--	--	J	K02879
XLOC_003316	No	No	DEG(I+M)	No	--	--	--	--
XLOC_003317	No	No	No	No	--	--	--	--
XLOC_003319	DEG(M)	No	No	No	--	--	--	--
XLOC_003324	DEG(M)	No	No	No	EGU83363.1	--	--	--
XLOC_003325	DEG(M)	No	No	No	--	--	--	--
XLOC_003326	DEG(M)	No	No	No	--	--	--	--
XLOC_003327	DEG(M)	No	No	No	--	--	--	--
XLOC_003332	DEG(M)	No	No	No	--	--	--	--
XLOC_003333	DEG(M)	No	No	No	--	--	Z	--
XLOC_003334	No	No	No	No	--	--	--	--

Differentially expressed gene (DEG) IDs	*C. microphyllum* root and shoot-specific (>2 fold)	*C. microphyllum* root and shoot-specific (>10 fold)	ICC4958 and *C. microphyllum* root-specific (>2 fold)	*C. microphyllum* root-specific (>2 fold)	Functional Annotation			
					NCBI nr IDs	TFs	KOG	KEGG
XLOC_003336	DEG(M)	DEG(M)	DEG(I+M)	DEG(M)	--	--	--	--
XLOC_003340	No	No	No	No	XP_001946482.2	--	T	--
XLOC_003342	No	No	No	No	XP_008916918.1	--	--	K15296
XLOC_003347	No	No	No	No	--	--	--	--
XLOC_003348	No	No	No	No	KEP49369.1	--	--	--
XLOC_003352	No	No	No	No	KEP47048.1	--	--	--
XLOC_003353	No	No	No	No	KEP47048.1	--	--	--
XLOC_003356	No	No	No	No	XP_003045171.1	--	--	--
XLOC_003358	No	No	No	No	NP_001280353.1	--	--	--
XLOC_003359	No	No	No	No	CCO26007.1	--	--	--
XLOC_003363	DEG(M)	No	No	No	--	--	--	--
XLOC_003364	No	No	No	No	XP_001945112.2	--	O	--
XLOC_003365	DEG(M)	No	No	No	--	--	--	--
XLOC_003366	DEG(M)	DEG(M)	DEG(I+M)	DEG(M)	EHK48867.1	--	--	--
XLOC_003375	DEG(M)	No	No	No	--	--	--	--
XLOC_003376	No	No	No	No	--	--	--	--
XLOC_003377	DEG(M)	DEG(M)	DEG(I+M)	DEG(M)	--	--	--	--
XLOC_003378	No	No	No	No	--	--	--	--
XLOC_003379	No	No	No	No	--	--	--	--
XLOC_003380	No	No	No	No	--	--	--	--
XLOC_003391	No	No	No	No	--	--	--	--
XLOC_003392	DEG(M)	No	No	No	EUC64939.1	--	--	--
XLOC_003395	DEG(M)	No	DEG(I+M)	DEG(M)	--	--	--	--
XLOC_003396	No	No	No	No	--	--	--	--
XLOC_003398	No	No	No	No	--	--	--	--
XLOC_003399	No	No	No	No	KEP52176.1	--	--	--
XLOC_003405	DEG(M)	No	No	No	--	--	R	--
XLOC_003406	DEG(M)	No	No	No	--	--	--	--
XLOC_003407	No	No	No	No	--	--	--	--
XLOC_003409	No	No	No	No	--	--	--	--
XLOC_003413	No	No	No	No	--	--	--	--
XLOC_003420	No	No	No	No	--	--	--	--
XLOC_003421	No	No	No	No	--	--	--	K13963
XLOC_003422	No	No	No	No	CCO28644.1	--	--	--
XLOC_003423	No	No	No	No	--	--	--	--
XLOC_003428	No	No	No	No	--	--	--	--
XLOC_003430	DEG(M)	DEG(M)	No	No	--	--	--	--
XLOC_003431	DEG(M)	DEG(M)	No	No	--	--	--	--
XLOC_003435	No	No	No	No	CCO36676.1	--	--	--
XLOC_003439	No	No	No	No	--	--	--	--

Differentially expressed gene (DEG) IDs	*C. microphyllum* root and shoot-specific (>2 fold)	*C. microphyllum* root and shoot-specific (>10 fold)	ICC4958 and *C. microphyllum* root-specific (>2 fold)	*C. microphyllum* root-specific (>2 fold)	Functional Annotation			
					NCBI nr IDs	TFs	KOG	KEGG
XLOC_003443	DEG(M)	No	No	No	--	--	--	--
XLOC_003448	DEG(M)	No	No	No	CEL51596.1	--	--	--
XLOC_003449	DEG(M)	No	No	No	CEL51597.1	--	--	--
XLOC_003450	No	No	No	No	--	--	--	--
XLOC_003451	No	No	DEG(I+M)	No	--	--	--	K00430
XLOC_003453	No	No	No	No	--	--	--	--
XLOC_003455	DEG(M)	No	DEG(I+M)	DEG(M)	XP_008906124.1	--	--	--
XLOC_003462	No	No	DEG(I+M)	No	--	--	--	--
XLOC_003479	No	No	No	No	--	--	--	--
XLOC_003487	No	No	No	No	XP_004495992.1	--	O	K03676
XLOC_003488	No	No	No	No	--	--	A	--
XLOC_003489	No	No	No	No	--	--	--	--
XLOC_003491	DEG(M)	No	No	No	--	--	--	--
XLOC_003495	No	No	No	No	--	--	--	--
XLOC_003507	DEG(M)	DEG(M)	No	No	XP_003240334.1	--	A	K12823
XLOC_003509	No	No	No	No	--	--	F	--
XLOC_003512	No	No	No	No	XP_001944205.2	--	QI	--
XLOC_003513	No	No	No	No	XP_001944205.2	--	QI	--
XLOC_003515	No	No	DEG(I+M)	No	XP_004495754.1	--	--	--
XLOC_003519	DEG(M)	No	No	No	ABZ91671.1	--	Z	--
XLOC_003520	DEG(M)	No	No	No	ABZ91671.1	--	Z	--
XLOC_003523	DEG(M)	No	No	No	CEL53892.1	--	--	K00011
XLOC_003524	DEG(M)	No	DEG(I+M)	DEG(M)	--	--	--	--
XLOC_003525	No	No	No	No	--	--	E	K01592
XLOC_003530	No	No	DEG(I+M)	No	--	--	--	K15095
XLOC_003531	DEG(M)	No	No	No	--	--	--	K15095
XLOC_003532	DEG(M)	No	No	No	ETI37027.1	--	--	K01834
XLOC_003533	No	No	No	No	ETL83920.1	--	--	--
XLOC_003536	No	No	No	No	--	--	--	--
XLOC_003545	DEG(M)	DEG(M)	DEG(I+M)	DEG(M)	--	--	--	--
XLOC_003546	DEG(M)	DEG(M)	DEG(I+M)	DEG(M)	--	--	--	--
XLOC_003549	No	No	No	No	EUC53695.1	--	--	--
XLOC_003562	DEG(M)	DEG(M)	No	No	XP_003053450.1	--	--	--
XLOC_003570	DEG(M)	No	No	No	--	--	--	--
XLOC_003573	DEG(M)	No	No	No	--	--	--	K00430
XLOC_003577	No	No	No	No	--	--	Z	--
XLOC_003578	DEG(M)	No	No	No	--	--	--	--
XLOC_003579	DEG(M)	No	No	No	--	--	--	--
XLOC_003585	No	No	No	No	--	--	--	--
XLOC_003586	No	No	No	No	--	--	--	--

Differentially expressed gene (DEG) IDs	*C. microphyllum* root and shoot-specific (>2 fold)	*C. microphyllum* root and shoot-specific (>10 fold)	ICC4958 and *C. microphyllum* root-specific (>2 fold)	*C. microphyllum* root-specific (>2 fold)	Functional Annotation			
					NCBI nr IDs	TFs	KOG	KEGG
XLOC_003591	No	No	No	No	XP_003044552.1	--	--	--
XLOC_003602	No	No	No	No	CCO29076.1	--	--	--
XLOC_003610	DEG(M)	No	No	No	CCI44251.1	--	C	--
XLOC_003611	DEG(M)	No	No	No	--	--	--	--
XLOC_003613	No	No	No	No	--	--	--	--
XLOC_003616	No	No	No	No	XP_001944842.2	--	--	--
XLOC_003618	No	No	No	No	--	--	--	--
XLOC_003620	DEG(M)	No	No	No	AFM78644.1	--	--	K01800
XLOC_003623	DEG(M)	No	No	No	XP_001649694.1	--	J	K02901
XLOC_003626	No	No	No	No	--	--	--	--
XLOC_003628	No	No	No	No	--	--	--	--
XLOC_003629	DEG(M)	No	No	No	NP_001162142.1	--	--	--
XLOC_003630	No	No	No	No	EUC67029.1	--	--	--
XLOC_003638	DEG(M)	No	No	No	--	--	--	--
XLOC_003644	DEG(M)	DEG(M)	No	No	--	--	--	--
XLOC_003645	DEG(M)	No	No	No	EUC60247.1	--	--	--
XLOC_003648	No	No	No	No	EUC67601.1	--	--	--
XLOC_003650	DEG(M)	No	DEG(I+M)	DEG(M)	--	--	--	--
XLOC_003652	No	No	No	No	--	--	--	--
XLOC_003654	No	No	No	No	XP_001942878.2	--	I	--
XLOC_003655	No	No	No	No	XP_001660185.1	--	--	--
XLOC_003656	No	No	No	No	--	--	--	--
XLOC_003657	DEG(M)	No	DEG(I+M)	DEG(M)	--	--	--	--
XLOC_003662	No	No	No	No	--	--	--	--
XLOC_003663	No	No	No	No	--	--	--	--
XLOC_003668	No	No	No	No	NP_001155423.1	--	--	--
XLOC_003669	No	No	No	No	XP_004510115.1	--	--	--
XLOC_003670	DEG(M)	DEG(M)	No	No	--	--	--	--
XLOC_003671	DEG(M)	No	No	No	--	--	--	--
XLOC_003672	No	No	No	No	CCO29181.1	--	T	--
XLOC_003673	DEG(M)	No	No	No	--	--	--	--
XLOC_003674	No	No	No	No	--	--	--	--
XLOC_003679	DEG(M)	No	No	No	XP_003042432.1	--	--	--
XLOC_003680	No	No	No	No	XP_002514229.1	MYB_related	QR	--
XLOC_003683	No	No	No	No	--	--	--	--
XLOC_003685	DEG(M)	No	No	No	--	--	--	--
XLOC_003688	No	No	No	No	--	--	--	--
XLOC_003691	No	No	No	No	KDN36376.1	--	--	--
XLOC_003692	No	No	No	No	XP_002516123.1	--	J	K02985
XLOC_003693	No	No	No	No	AJL33751.1	--	--	--

Differentially expressed gene (DEG) IDs	*C. microphyllum* root and shoot-specific (>2 fold)	*C. microphyllum* root and shoot-specific (>10 fold)	ICC4958 and *C. microphyllum* root-specific (>2 fold)	*C. microphyllum* root-specific (>2 fold)	Functional Annotation			
					NCBI nr IDs	TFs	KOG	KEGG
XLOC_003694	No	No	No	No	XP_002525322.1	--	--	K02907
XLOC_003696	DEG(M)	No	No	No	--	--	--	--
XLOC_003701	DEG(M)	DEG(M)	DEG(I+M)	DEG(M)	--	--	--	--
XLOC_003702	No	No	No	No	--	--	G	--
XLOC_003703	No	No	No	No	--	--	--	--
XLOC_003704	No	No	No	No	--	--	--	--
XLOC_003708	DEG(M)	DEG(M)	DEG(I+M)	DEG(M)	KEH31997.1	--	GT	K00012
XLOC_003709	No	No	No	No	XP_001944547.1	--	--	--
XLOC_003717	DEG(M)	No	No	No	KIJ44121.1	--	--	--
XLOC_003719	No	No	No	No	EUC57812.1	--	--	--
XLOC_003723	No	No	No	No	NP_001232980.1	--	--	--
XLOC_003724	DEG(M)	No	No	No	ETL90434.1	--	--	--
XLOC_003727	No	No	No	No	--	--	--	--
XLOC_003728	No	No	No	No	KFM72198.1	--	B	K06067
XLOC_003729	No	No	No	No	--	--	--	--
XLOC_003730	No	No	No	No	--	--	--	--
XLOC_003731	No	No	No	No	XP_002896132.1	FAR1	O	--
XLOC_003732	No	No	DEG(I+M)	No	--	--	--	--
XLOC_003733	DEG(M)	No	No	No	--	--	--	--
XLOC_003734	No	No	No	No	XP_001946073.1	--	--	K01581
XLOC_003735	No	No	No	No	--	--	--	--
XLOC_003738	DEG(M)	No	No	No	--	--	--	--
XLOC_003740	No	No	No	No	--	--	--	--
XLOC_003744	No	No	No	No	--	--	--	--
XLOC_003746	No	No	No	No	--	--	--	--
XLOC_003748	No	No	No	No	--	--	--	--
XLOC_003750	DEG(M)	No	No	No	--	--	--	--
XLOC_003752	No	No	No	No	XP_008906440.1	--	--	--
XLOC_003760	No	No	No	No	--	--	--	--
XLOC_003761	No	No	No	No	--	--	--	--
XLOC_003763	No	No	No	No	--	--	--	--
XLOC_003765	No	No	No	No	--	--	--	--
XLOC_003766	No	No	No	No	EUC67115.1	--	--	--
XLOC_003768	No	No	No	No	XP_001651208.1	--	I	K00967
XLOC_003770	No	No	No	No	--	--	--	--
XLOC_003771	No	No	No	No	XP_004513065.1	--	--	K19032
XLOC_003773	DEG(M)	No	No	No	KDN43622.1	--	--	--
XLOC_003778	No	No	No	No	--	--	--	--
XLOC_003779	No	No	No	No	--	--	Q	--
XLOC_003780	No	No	No	No	KKY34763.1	--	--	--

Differentially expressed gene (DEG) IDs	*C. microphyllum* root and shoot-specific (>2 fold)	*C. microphyllum* root and shoot-specific (>10 fold)	ICC4958 and *C. microphyllum* root-specific (>2 fold)	*C. microphyllum* root-specific (>2 fold)	Functional Annotation			
					NCBI nr IDs	TFs	KOG	KEGG
XLOC_003781	No	No	No	No	CEL63900.1	--	--	--
XLOC_003786	No	No	No	No	--	--	--	--
XLOC_003787	No	No	No	No	--	--	--	--
XLOC_003789	No	No	No	No	--	--	--	--
XLOC_003791	No	No	No	No	XP_002511444.1	--	--	--
XLOC_003794	No	No	No	No	--	--	--	--
XLOC_003795	No	No	No	No	--	--	--	--
XLOC_003796	DEG(M)	No	No	No	--	--	--	--
XLOC_003797	DEG(M)	No	No	No	--	--	--	--
XLOC_003799	No	No	No	No	--	--	--	--
XLOC_003800	No	No	No	No	--	--	--	K16297
XLOC_003809	No	No	No	No	--	--	--	--
XLOC_003814	No	No	No	No	KFM73238.1	--	T	--
XLOC_003816	No	No	No	No	XP_001945365.2	--	--	--
XLOC_003819	No	No	No	No	XP_002509886.1	FAR1	T	--
XLOC_003821	DEG(M)	No	No	No	--	--	--	--
XLOC_003825	No	No	No	No	ETK88328.1	--	--	--
XLOC_003828	DEG(M)	No	No	No	--	--	J	K02877
XLOC_003830	No	No	No	No	EFX90077.1	--	H	--
XLOC_003831	No	No	No	No	XP_003399358.1	--	H	--
XLOC_003834	No	No	No	No	XP_008898560.1	--	--	--
XLOC_003835	No	No	No	No	XP_004509757.1	--	I	K01661
XLOC_003840	No	No	No	No	XP_001943357.1	--	--	--
XLOC_003847	No	No	No	No	CCO28914.1	--	--	--
XLOC_003848	No	No	No	No	--	--	--	--
XLOC_003849	DEG(M)	No	DEG(I+M)	DEG(M)	XP_002908887.1	--	--	K02947
XLOC_003852	No	No	No	No	XP_003051606.1	--	--	--
XLOC_003853	No	No	No	No	--	--	--	--
XLOC_003859	No	No	No	No	--	--	--	--
XLOC_003860	No	No	No	No	--	--	--	--
XLOC_003862	No	No	No	No	CDR45007.1	--	J	--
XLOC_003863	No	No	No	No	--	--	--	--
XLOC_003869	No	No	No	No	--	--	--	--
XLOC_003870	No	No	No	No	XP_001943465.2	--	--	K13172
XLOC_003871	No	No	No	No	XP_008906189.1	--	--	--
XLOC_003874	No	No	No	No	--	--	--	--
XLOC_003875	No	No	No	No	KDN45174.1	--	--	--
XLOC_003877	No	No	No	No	--	--	G	K09873
XLOC_003878	No	No	No	No	--	--	--	--
XLOC_003884	No	No	No	No	--	--	O	--

Differentially expressed gene (DEG) IDs	*C. microphyllum* root and shoot-specific (>2 fold)	*C. microphyllum* root and shoot-specific (>10 fold)	ICC4958 and *C. microphyllum* root-specific (>2 fold)	*C. microphyllum* root-specific (>2 fold)	Functional Annotation			
					NCBI nr IDs	TFs	KOG	KEGG
XLOC_003888	No	No	No	No	EUC57026.1	--	--	--
XLOC_003891	No	No	No	No	--	--	--	--
XLOC_003896	DEG(M)	No	No	No	--	--	--	--
XLOC_003897	No	No	No	No	--	--	--	--
XLOC_003902	DEG(M)	No	No	No	--	--	--	--
XLOC_003903	No	No	No	No	ETM45814.1	--	--	K10249
XLOC_003908	DEG(M)	No	No	No	--	--	--	--
XLOC_003910	DEG(M)	No	No	No	CCO26137.1	--	O	--
XLOC_003920	No	No	No	No	--	--	--	--
XLOC_003923	No	No	No	No	XP_008908204.1	--	--	--
XLOC_003924	DEG(M)	No	No	No	--	--	--	--
XLOC_003926	No	No	DEG(I+M)	No	XP_004516471.1	--	--	--
XLOC_003928	No	No	No	No	--	--	E	--
XLOC_003929	No	No	No	No	--	--	--	--
XLOC_003932	DEG(M)	No	No	No	--	--	--	--
XLOC_003933	No	No	No	No	XP_009523442.1	--	--	--
XLOC_003934	No	No	No	No	BAJ91582.1	--	C	--
XLOC_003936	No	No	No	No	KDN34855.1	TCP	J	K03264
XLOC_003937	No	No	DEG(I+M)	No	--	--	X	K16190
XLOC_003941	No	No	No	No	NP_001155549.1	--	--	K09250
XLOC_003943	No	No	No	No	--	--	--	--
XLOC_003945	No	No	No	No	--	--	--	--
XLOC_003946	No	No	No	No	ERG87159.1	--	U	K17086
XLOC_003947	No	No	No	No	ETP13430.1	--	--	--
XLOC_003950	No	No	No	No	ETI52587.1	--	--	--
XLOC_003951	No	No	No	No	XP_008186504.1	--	--	K00826
XLOC_003953	No	No	No	No	XP_004506718.1	--	--	K06910
XLOC_003954	No	No	No	No	--	--	--	--
XLOC_003958	No	No	No	No	--	--	--	K00799
XLOC_003960	No	No	No	No	--	--	--	--
XLOC_003961	No	No	No	No	--	--	--	K14488
XLOC_003962	No	No	No	No	CEL55535.1	--	--	--
XLOC_003963	No	No	No	No	XP_008907368.1	--	--	--
XLOC_003966	No	No	No	No	--	--	--	--
XLOC_003967	DEG(M)	No	No	No	XP_001651545.1	--	--	--
XLOC_003970	DEG(M)	No	DEG(I+M)	DEG(M)	CCO31092.1	--	--	--
XLOC_003972	No	No	No	No	--	--	--	--
XLOC_003973	No	No	No	No	--	--	--	K16276
XLOC_003976	No	No	No	No	CCO26761.1	--	--	--
XLOC_003977	DEG(M)	No	DEG(I+M)	DEG(M)	--	--	--	--

Differentially expressed gene (DEG) IDs	*C. microphyllum* root and shoot-specific (>2 fold)	*C. microphyllum* root and shoot-specific (>10 fold)	ICC4958 and *C. microphyllum* root-specific (>2 fold)	*C. microphyllum* root-specific (>2 fold)	Functional Annotation			
					NCBI nr IDs	TFs	KOG	KEGG
XLOC_003981	DEG(M)	DEG(M)	No	No	--	--	--	--
XLOC_003987	No	No	No	No	XP_008908824.1	--	--	--
XLOC_003988	DEG(M)	No	No	No	XP_008908824.1	--	--	--
XLOC_003989	No	No	No	No	--	--	--	--
XLOC_003990	No	No	No	No	XP_003049108.1	--	--	--
XLOC_003993	DEG(M)	No	No	No	XP_008182999.1	--	E	--
XLOC_003994	No	No	DEG(I+M)	No	XP_004502395.1	--	--	--
XLOC_003995	No	No	No	No	--	--	--	--
XLOC_003996	No	No	No	No	--	B3	O	--
XLOC_003997	No	No	No	No	--	--	PT	--
XLOC_004001	No	No	No	No	ETI45029.1	--	--	--
XLOC_004002	DEG(M)	No	No	No	XP_009531178.1	--	--	--
XLOC_004003	No	No	No	No	XP_001950902.2	--	--	--
XLOC_004004	No	No	No	No	XP_001950902.2	--	--	--
XLOC_004005	No	No	No	No	XP_001948450.1	--	--	--
XLOC_004009	No	No	No	No	--	--	--	--
XLOC_004010	No	No	No	No	--	--	--	--
XLOC_004016	DEG(M)	DEG(M)	DEG(I+M)	DEG(M)	XP_004504297.1	--	--	--
XLOC_004018	No	No	No	No	XP_003048825.1	--	--	--
XLOC_004019	No	No	No	No	--	--	--	--
XLOC_004026	DEG(M)	No	No	No	--	--	--	--
XLOC_004027	No	No	No	No	KDN48673.1	--	--	--
XLOC_004030	DEG(M)	No	No	No	CCO28027.1	--	--	--
XLOC_004032	No	No	No	No	--	--	Z	--
XLOC_004035	No	No	No	No	--	--	--	--
XLOC_004037	No	No	No	No	XP_003043395.1	--	--	--
XLOC_004038	DEG(M)	No	No	No	--	--	--	--
XLOC_004039	DEG(M)	No	No	No	--	--	--	--
XLOC_004047	No	No	No	No	CCO32950.1	--	--	--
XLOC_004051	DEG(M)	No	No	No	EUC56717.1	--	--	--
XLOC_004054	No	No	No	No	NP_001155562.1	--	O	--
XLOC_004055	No	No	No	No	--	--	--	--
XLOC_004056	No	No	No	No	--	--	--	--
XLOC_004057	DEG(M)	No	No	No	--	--	J	--
XLOC_004063	DEG(M)	DEG(M)	No	No	--	--	--	--
XLOC_004064	No	No	No	No	--	--	--	--
XLOC_004067	No	No	No	No	--	--	--	K09522
XLOC_004068	No	No	No	No	EUC57050.1	--	--	--
XLOC_004070	No	No	No	No	AJQ30125.1	--	C	K00025
XLOC_004071	No	No	No	No	--	--	--	K04523

Differentially expressed gene (DEG) IDs	*C. microphyllum* root and shoot-specific (>2 fold)	*C. microphyllum* root and shoot-specific (>10 fold)	ICC4958 and *C. microphyllum* root-specific (>2 fold)	*C. microphyllum* root-specific (>2 fold)	Functional Annotation			
					NCBI nr IDs	TFs	KOG	KEGG
XLOC_004075	No	No	No	No	XP_002634132.1	--	B	K11251
XLOC_004077	No	No	No	No	--	--	--	--
XLOC_004079	No	No	No	No	XP_008187595.1	--	--	--
XLOC_004082	No	No	No	No	ETO61253.1	--	--	K06911
XLOC_004085	DEG(M)	No	No	No	XP_001847462.1	--	O	--
XLOC_004086	No	No	No	No	--	--	--	--
XLOC_004088	No	No	No	No	XP_001653857.1	--	--	--
XLOC_004089	No	No	No	No	XP_008898685.1	--	--	K11599
XLOC_004091	No	No	No	No	--	--	--	--
XLOC_004094	No	No	No	No	XP_002509620.1	--	U	K15292
XLOC_004098	No	No	No	No	XP_008903125.1	--	--	--
XLOC_004099	No	No	No	No	--	--	--	--
XLOC_004101	No	No	No	No	--	--	--	--
XLOC_004106	No	No	No	No	KEP53177.1	--	--	--
XLOC_004108	No	No	No	No	XP_001949209.1	--	--	--
XLOC_004110	No	No	No	No	CCO36949.1	--	--	K00222
XLOC_004112	No	No	No	No	--	--	--	--
XLOC_004113	No	No	No	No	--	--	--	--
XLOC_004115	No	No	No	No	EUC66604.1	--	--	--
XLOC_004118	No	No	No	No	XP_003053352.1	--	--	--
XLOC_004122	No	No	No	No	--	--	--	--
XLOC_004123	No	No	No	No	EJW77744.1	--	W	--
XLOC_004126	DEG(M)	No	No	No	EUC62179.1	--	--	--
XLOC_004128	No	No	No	No	--	--	--	--
XLOC_004131	No	No	No	No	--	--	--	--
XLOC_004133	No	No	No	No	--	--	--	--
XLOC_004135	No	No	No	No	--	--	--	--
XLOC_004138	DEG(M)	No	No	No	--	--	--	--
XLOC_004139	No	No	No	No	KDN36914.1	--	--	--
XLOC_004143	No	No	No	No	XP_004505134.1	--	--	--
XLOC_004144	No	No	No	No	KEP47221.1	--	--	--
XLOC_004145	No	No	No	No	NP_001155784.1	--	A	--
XLOC_004147	No	No	No	No	XP_003044228.1	--	--	--
XLOC_004150	DEG(M)	No	No	No	--	--	--	--
XLOC_004152	DEG(M)	No	No	No	--	--	--	--
XLOC_004156	No	No	No	No	--	--	--	--
XLOC_004157	No	No	No	No	XP_001661879.1	--	J	K03265
XLOC_004158	No	No	No	No	CEL55095.1	--	--	--
XLOC_004160	No	No	No	No	XP_004508574.1	TCP	--	--
XLOC_004162	No	No	No	No	--	--	--	--

Differentially expressed gene (DEG) IDs	*C. microphyllum* root and shoot-specific (>2 fold)	*C. microphyllum* root and shoot-specific (>10 fold)	ICC4958 and *C. microphyllum* root-specific (>2 fold)	*C. microphyllum* root-specific (>2 fold)	Functional Annotation			
					NCBI nr IDs	TFs	KOG	KEGG
XLOC_004163	No	No	No	No	ELU41746.1	--	--	--
XLOC_004170	No	No	No	No	XP_002524403.1	bHLH	X	--
XLOC_004171	DEG(M)	No	No	No	XP_008907054.1	--	--	K00416
XLOC_004173	No	No	No	No	KDR11240.1	--	R	--
XLOC_004176	DEG(M)	No	No	No	XP_008893022.1	--	J	K03263
XLOC_004178	No	No	No	No	XP_003051589.1	--	--	K15849
XLOC_004189	No	No	No	No	ETL80527.1	--	--	--
XLOC_004194	No	No	No	No	--	--	--	--
XLOC_004195	No	No	No	No	--	--	--	--
XLOC_004196	DEG(M)	No	No	No	--	--	--	--
XLOC_004200	No	No	No	No	XP_008183838.1	--	--	--
XLOC_004201	No	No	No	No	--	--	C	--
XLOC_004202	No	No	No	No	--	--	--	K10400
XLOC_004204	DEG(M)	No	No	No	--	--	--	--
XLOC_004205	DEG(M)	No	No	No	ADV40164.1	--	J	K02985
XLOC_004206	DEG(M)	DEG(M)	No	No	EWG42059.1	--	--	K14293
XLOC_004207	No	No	No	No	KJA23676.1	--	--	--
XLOC_004212	No	No	No	No	--	--	--	--
XLOC_004214	No	No	No	No	--	--	O	--
XLOC_004216	No	No	No	No	--	--	--	--
XLOC_004218	DEG(M)	No	DEG(I+M)	DEG(M)	--	--	--	--
XLOC_004219	DEG(M)	No	DEG(I+M)	DEG(M)	AHE74293.1	--	J	--
XLOC_004221	No	No	No	No	XP_002524470.1	--	I	K00894
XLOC_004224	No	No	No	No	--	--	--	--
XLOC_004226	No	No	No	No	--	--	--	--
XLOC_004227	No	No	No	No	NP_001155534.1	--	J	K03236
XLOC_004233	No	No	No	No	EUC65460.1	--	--	--
XLOC_004234	No	No	No	No	--	--	--	K02924
XLOC_004238	DEG(M)	No	No	No	--	--	--	--
XLOC_004239	No	No	No	No	--	--	--	--
XLOC_004240	No	No	No	No	KEP53653.1	--	--	--
XLOC_004244	No	No	No	No	XP_003051186.1	--	--	--
XLOC_004247	DEG(M)	DEG(M)	DEG(I+M)	DEG(M)	XP_008903238.1	--	J	K02891
XLOC_004251	No	No	No	No	XP_003053250.1	--	--	--
XLOC_004252	No	No	No	No	--	--	--	--
XLOC_004254	DEG(M)	No	No	No	--	--	--	--
XLOC_004255	No	No	No	No	KEP47078.1	--	--	--
XLOC_004256	No	No	No	No	NP_001156137.1	--	--	--
XLOC_004260	No	No	No	No	--	--	--	--
XLOC_004261	No	No	No	No	--	--	--	--

Differentially expressed gene (DEG) IDs	*C. microphyllum* root and shoot-specific (>2 fold)	*C. microphyllum* root and shoot-specific (>10 fold)	ICC4958 and *C. microphyllum* root-specific (>2 fold)	*C. microphyllum* root-specific (>2 fold)	Functional Annotation			
					NCBI nr IDs	TFs	KOG	KEGG
XLOC_004262	No	No	No	No	XP_004504908.1	bHLH	--	K15086
XLOC_004265	No	No	No	No	--	--	--	--
XLOC_004266	No	No	No	No	KDN48967.1	--	H	--
XLOC_004271	No	No	No	No	CEL57127.1	--	--	--
XLOC_004273	No	No	No	No	CCO31528.1	--	--	--
XLOC_004274	DEG(M)	No	No	No	EMT72865.1	--	--	--
XLOC_004275	DEG(M)	No	No	No	--	--	--	--
XLOC_004277	No	No	No	No	EUC55092.1	--	--	--
XLOC_004278	No	No	No	No	KEP46745.1	--	C	--
XLOC_004279	No	No	No	No	CEL59173.1	--	--	--
XLOC_004280	No	No	No	No	--	--	--	--
XLOC_004282	DEG(M)	No	No	No	XP_010682849.1	--	R	K08818
XLOC_004285	No	No	No	No	--	--	--	--
XLOC_004286	No	No	No	No	XP_004516682.1	--	--	--
XLOC_004287	DEG(M)	DEG(M)	No	No	XP_008179625.1	--	DZ	--
XLOC_004290	DEG(M)	No	No	No	XP_001648567.1	--	J	--
XLOC_004302	No	No	No	No	XP_003044686.1	--	--	--
XLOC_004303	DEG(M)	No	No	No	EUC65363.1	--	--	--
XLOC_004304	No	No	No	No	--	--	--	--
XLOC_004309	DEG(M)	No	No	No	NP_001155561.1	--	J	K02940
XLOC_004312	DEG(M)	No	No	No	ACR24971.1	--	J	K02975
XLOC_004315	No	No	No	No	--	--	--	--
XLOC_004324	DEG(M)	DEG(M)	No	No	XP_001948685.1	--	Z	K07374
XLOC_004325	No	No	No	No	--	--	--	--
XLOC_004328	DEG(M)	No	No	No	--	MYB	J	--
XLOC_004329	DEG(M)	No	No	No	--	MYB	J	--
XLOC_004332	No	No	No	No	--	--	--	--
XLOC_004333	No	No	No	No	XP_009844134.1	--	R	--
XLOC_004335	DEG(M)	DEG(M)	DEG(I+M)	DEG(M)	XP_004339588.1	--	J	K02908
XLOC_004336	No	No	No	No	KDN47915.1	--	T	--
XLOC_004338	No	No	No	No	--	--	--	--
XLOC_004340	No	No	No	No	XP_001943118.1	--	--	--
XLOC_004341	No	No	No	No	--	--	--	--
XLOC_004342	No	No	No	No	--	--	--	--
XLOC_004346	No	No	No	No	ETI44157.1	--	--	--
XLOC_004352	DEG(M)	DEG(M)	DEG(I+M)	DEG(M)	--	YABBY	J	--
XLOC_004355	No	No	No	No	--	--	--	--
XLOC_004357	No	No	No	No	CEL61057.1	--	--	--
XLOC_004363	No	No	No	No	--	--	R	--
XLOC_004364	No	No	No	No	XP_009525936.1	--	--	--

Differentially expressed gene (DEG) IDs	*C. microphyllum* root and shoot-specific (>2 fold)	*C. microphyllum* root and shoot-specific (>10 fold)	ICC4958 and *C. microphyllum* root-specific (>2 fold)	*C. microphyllum* root-specific (>2 fold)	Functional Annotation			
					NCBI nr IDs	TFs	KOG	KEGG
XLOC_004365	No	No	No	No	ETL93890.1	--	G	--
XLOC_004367	No	No	DEG(I+M)	No	--	--	--	--
XLOC_004372	No	No	No	No	--	--	--	K10258
XLOC_004373	DEG(M)	DEG(M)	No	No	--	--	--	--
XLOC_004374	DEG(M)	DEG(M)	No	No	--	--	I	K17756
XLOC_004376	No	No	No	No	--	--	--	--
XLOC_004377	No	No	No	No	--	--	--	--
XLOC_004379	No	No	No	No	--	--	--	--
XLOC_004384	No	No	No	No	EUC57111.1	--	--	--
XLOC_004386	No	No	No	No	CEL55620.1	--	--	--
XLOC_004392	No	No	No	No	XP_001662512.1	--	F	K00940
XLOC_004394	No	No	No	No	--	--	--	--
XLOC_004396	No	No	No	No	AAA99920.1	--	O	--
XLOC_004400	No	No	No	No	--	--	--	--
XLOC_004401	DEG(M)	No	No	No	--	--	--	--
XLOC_004403	No	No	No	No	CCA23809.1	--	O	--
XLOC_004404	No	No	No	No	KNB14459.1	--	--	--
XLOC_004407	No	No	No	No	--	--	--	--
XLOC_004409	No	No	No	No	--	--	--	--
XLOC_004413	No	No	No	No	CEL61869.1	--	--	K00621
XLOC_004414	No	No	No	No	EUC64373.1	--	--	--
XLOC_004415	No	No	No	No	XP_004367925.1	--	J	--
XLOC_004416	No	No	No	No	CCO36354.1	--	--	--
XLOC_004417	DEG(M)	No	No	No	CCO31096.1	--	--	--
XLOC_004418	DEG(M)	No	No	No	XP_008901486.1	--	--	K08762
XLOC_004422	No	No	No	No	--	--	--	--
XLOC_004423	No	No	No	No	EUC56104.1	--	--	--
XLOC_004426	DEG(M)	No	No	No	NP_001119626.1	--	Z	--
XLOC_004427	No	No	DEG(I+M)	No	XP_004498551.1	--	X	--
XLOC_004430	No	No	No	No	--	--	--	--
XLOC_004431	No	No	No	No	--	--	--	--
XLOC_004433	No	No	No	No	--	--	--	--
XLOC_004435	No	No	No	No	KEP51916.1	--	--	--
XLOC_004444	No	No	No	No	XP_008904391.1	--	--	--
XLOC_004447	No	No	No	No	--	--	--	--
XLOC_004448	No	No	No	No	--	--	T	K04368
XLOC_004449	No	No	No	No	XP_004503796.1	--	C	K15102
XLOC_004452	No	No	No	No	--	--	--	--
XLOC_004454	No	No	No	No	EMT67829.1	--	--	--
XLOC_004455	DEG(M)	No	No	No	KIH42507.1	--	C	--

Differentially expressed gene (DEG) IDs	*C. microphyllum* root and shoot-specific (>2 fold)	*C. microphyllum* root and shoot-specific (>10 fold)	ICC4958 and *C. microphyllum* root-specific (>2 fold)	*C. microphyllum* root-specific (>2 fold)	Functional Annotation			
					NCBI nr IDs	TFs	KOG	KEGG
XLOC_004457	No	No	No	No	XP_001661939.1	--	ZT	K17261
XLOC_004459	No	No	No	No	KEP55275.1	--	--	--
XLOC_004461	No	No	No	No	CEL59221.1	--	--	--
XLOC_004464	No	No	No	No	CCT73222.1	--	--	--
XLOC_004465	No	No	No	No	ETI36691.1	--	--	--
XLOC_004466	No	No	No	No	--	--	--	--
XLOC_004467	No	No	No	No	KDN36621.1	--	--	--
XLOC_004470	DEG(M)	DEG(M)	No	No	--	--	--	--
XLOC_004472	No	No	No	No	--	--	--	--
XLOC_004474	No	No	No	No	XP_001652727.1	--	--	--
XLOC_004477	No	No	No	No	XP_002523450.1	--	J	K01876
XLOC_004479	No	No	No	No	--	--	--	--
XLOC_004481	No	No	No	No	CAM84512.1	--	S	--
XLOC_004482	No	No	No	No	XP_001951891.1	--	--	K14324
XLOC_004488	No	No	No	No	--	--	Q	K00121
XLOC_004489	No	No	No	No	--	--	--	K01890
XLOC_004490	DEG(M)	No	No	No	ABK94700.1	--	J	--
XLOC_004491	No	No	No	No	GAO15331.1	--	O	--
XLOC_004492	No	No	No	No	XP_003242070.1	--	--	--
XLOC_004494	DEG(M)	No	No	No	--	--	--	--
XLOC_004496	No	No	No	No	--	--	--	--
XLOC_004498	No	No	DEG(I+M)	No	XP_004489160.1	WRKY	--	--
XLOC_004499	No	No	No	No	--	--	--	--
XLOC_004500	No	No	No	No	--	--	--	--
XLOC_004503	No	No	No	No	--	--	--	--
XLOC_004507	DEG(M)	No	No	No	EUC55268.1	--	--	--
XLOC_004510	No	No	No	No	--	--	--	--
XLOC_004511	No	No	No	No	XP_009526013.1	--	T	--
XLOC_004514	DEG(M)	No	No	No	--	--	--	--
XLOC_004515	No	No	No	No	--	--	--	K04508
XLOC_004518	No	No	No	No	--	--	--	--
XLOC_004520	DEG(M)	No	No	No	XP_009261766.1	--	J	--
XLOC_004528	DEG(M)	No	No	No	XP_009529394.1	--	O	--
XLOC_004529	No	No	No	No	EUC59783.1	--	--	--
XLOC_004530	No	No	No	No	--	--	CR	--
XLOC_004533	No	No	DEG(I+M)	No	--	--	R	--
XLOC_004539	DEG(M)	No	No	No	--	--	--	--
XLOC_004547	DEG(M)	DEG(M)	DEG(I+M)	DEG(M)	--	--	--	--
XLOC_004550	No	No	No	No	--	--	--	--
XLOC_004551	No	No	No	No	--	--	--	--

Differentially expressed gene (DEG) IDs	*C. microphyllum* root and shoot-specific (>2 fold)	*C. microphyllum* root and shoot-specific (>10 fold)	ICC4958 and *C. microphyllum* root-specific (>2 fold)	*C. microphyllum* root-specific (>2 fold)	Functional Annotation			
					NCBI nr IDs	TFs	KOG	KEGG
XLOC_004552	DEG(M)	No	No	No	--	--	--	--
XLOC_004553	No	No	No	No	CCO30612.1	--	--	--
XLOC_004555	No	No	No	No	--	--	T	--
XLOC_004557	DEG(M)	No	No	No	EUC58750.1	--	--	--
XLOC_004558	No	No	No	No	CEL61076.1	--	--	--
XLOC_004577	No	No	No	No	--	--	--	--
XLOC_004591	DEG(M)	DEG(M)	No	No	--	--	--	--
XLOC_004592	DEG(M)	No	No	No	--	--	--	--
XLOC_004593	No	No	No	No	--	--	--	--
XLOC_004594	No	No	DEG(I+M)	No	--	--	O	--
XLOC_004595	No	No	No	No	--	--	--	--
XLOC_004597	No	No	No	No	--	--	--	--
XLOC_004598	No	No	No	No	BAK07442.1	--	--	--
XLOC_004599	DEG(M)	DEG(M)	DEG(I+M)	DEG(M)	XP_009524206.1	--	J	--
XLOC_004604	No	No	No	No	KFM73638.1	--	C	K02149
XLOC_004605	No	No	No	No	--	--	--	--
XLOC_004607	No	No	No	No	XP_009515820.1	--	G	--
XLOC_004614	DEG(M)	No	No	No	KEP52891.1	--	E	K14454
XLOC_004615	DEG(M)	No	No	No	--	--	--	--
XLOC_004625	DEG(M)	DEG(M)	DEG(I+M)	DEG(M)	--	--	--	--
XLOC_004627	DEG(M)	DEG(M)	No	No	EXK80136.1	--	--	--
XLOC_004628	DEG(M)	No	No	No	XP_008907532.1	--	G	K01689
XLOC_004630	No	No	No	No	--	--	--	--
XLOC_004634	No	No	No	No	XP_004333044.1	--	J	--
XLOC_004635	No	No	No	No	--	--	--	K01052
XLOC_004636	No	No	No	No	XP_012573117.1	--	--	K15281
XLOC_004638	DEG(M)	No	No	No	--	--	--	K02991
XLOC_004646	No	No	No	No	XP_007149747.1	--	--	K00053
XLOC_004648	No	No	DEG(I+M)	No	XP_004498328.1	--	R	--
XLOC_004649	DEG(M)	No	No	No	XP_003041034.1	--	--	--
XLOC_004651	No	No	No	No	--	--	--	--
XLOC_004653	DEG(M)	No	No	No	XP_008914027.1	--	--	--
XLOC_004657	DEG(M)	No	No	No	KEP50281.1	--	--	--
XLOC_004663	No	No	No	No	--	--	J	--
XLOC_004674	DEG(M)	No	No	No	--	--	--	--
XLOC_004675	No	No	No	No	--	--	--	--
XLOC_004677	DEG(M)	No	No	No	--	--	--	--
XLOC_004678	No	No	No	No	--	--	--	--
XLOC_004679	No	No	No	No	--	--	--	--
XLOC_004681	No	No	No	No	--	--	--	K08245

Differentially expressed gene (DEG) IDs	*C. microphyllum* root and shoot-specific (>2 fold)	*C. microphyllum* root and shoot-specific (>10 fold)	ICC4958 and *C. microphyllum* root-specific (>2 fold)	*C. microphyllum* root-specific (>2 fold)	Functional Annotation			
					NCBI nr IDs	TFs	KOG	KEGG
XLOC_004682	DEG(M)	No	DEG(I+M)	DEG(M)	--	--	--	K13066
XLOC_004683	No	No	No	No	ETI49348.1	--	--	K12448
XLOC_004689	DEG(M)	No	No	No	XP_006457455.1	--	--	--
XLOC_004690	DEG(M)	No	No	No	--	--	--	--
XLOC_004691	No	No	No	No	--	--	--	--
XLOC_004696	No	No	No	No	--	--	H	--
XLOC_004700	DEG(M)	No	No	No	KEP48920.1	--	--	--
XLOC_004716	DEG(M)	No	No	No	--	--	--	--
XLOC_004717	DEG(M)	No	No	No	--	--	--	--
XLOC_004718	DEG(M)	No	No	No	KEP53270.1	--	--	--
XLOC_004719	DEG(M)	No	No	No	KEP53270.1	--	--	--
XLOC_004720	DEG(M)	No	No	No	KDN44474.1	--	--	--
XLOC_004721	DEG(M)	No	No	No	CEL61076.1	--	--	--
XLOC_004723	No	No	DEG(I+M)	No	--	--	--	--
XLOC_004730	No	No	No	No	--	MYB_related	F	K15014
XLOC_004732	No	No	No	No	CCO29782.1	--	--	--
XLOC_004741	DEG(M)	No	No	No	XP_008893682.1	--	J	K02960
XLOC_004750	No	No	No	No	--	--	--	--
XLOC_004751	DEG(M)	No	No	No	KJZ76013.1	--	J	--
XLOC_004753	No	No	No	No	CEL59065.1	--	J	--
XLOC_004759	DEG(M)	DEG(M)	No	No	XP_003836531.1	--	--	--
XLOC_004760	DEG(M)	No	No	No	EUC57569.1	--	C	K02267
XLOC_004761	DEG(M)	No	No	No	EUC57569.1	--	C	--
XLOC_004762	No	No	No	No	--	--	--	--
XLOC_004763	No	No	No	No	--	--	--	--
XLOC_004764	No	No	No	No	KEP52552.1	--	--	--
XLOC_004770	DEG(M)	No	DEG(I+M)	DEG(M)	EUC62999.1	--	--	--
XLOC_004788	No	No	No	No	--	--	--	--
XLOC_004794	No	No	No	No	XP_003050321.1	--	--	--
XLOC_004797	DEG(M)	No	No	No	CEL53910.1	--	--	--
XLOC_004798	DEG(M)	No	No	No	CCO28321.1	--	--	--
XLOC_004799	No	No	No	No	CCO28321.1	--	--	--
XLOC_004805	DEG(M)	No	No	No	--	--	--	--
XLOC_004814	DEG(M)	No	No	No	--	--	--	--
XLOC_004820	No	No	No	No	--	--	Z	--
XLOC_004823	No	No	No	No	--	--	--	K14004
XLOC_004825	No	No	No	No	--	--	--	--
XLOC_004827	DEG(M)	No	No	No	--	--	--	--
XLOC_004829	No	No	DEG(I+M)	No	--	--	--	--
XLOC_004830	DEG(M)	DEG(M)	No	No	--	--	--	--

Differentially expressed gene (DEG) IDs	*C. microphyllum* root and shoot-specific (>2 fold)	*C. microphyllum* root and shoot-specific (>10 fold)	ICC4958 and *C. microphyllum* root-specific (>2 fold)	*C. microphyllum* root-specific (>2 fold)	Functional Annotation			
					NCBI nr IDs	TFs	KOG	KEGG
XLOC_004832	No	No	No	No	CCO30399.1	--	--	--
XLOC_004834	No	No	No	No	--	--	--	K07195
XLOC_004835	No	No	No	No	--	--	--	--
XLOC_004836	No	No	No	No	XP_008902974.1	--	--	--
XLOC_004838	No	No	No	No	AFR41401.1	--	E	--
XLOC_004843	No	No	No	No	--	--	--	--
XLOC_004847	No	No	No	No	ELU45494.1	--	--	--
XLOC_004848	No	No	No	No	XP_003046107.1	--	--	--
XLOC_004853	No	No	No	No	--	--	--	--
XLOC_004854	No	No	No	No	XP_001658896.1	--	--	--
XLOC_004856	No	No	No	No	--	--	--	--
XLOC_004857	No	No	No	No	--	--	--	--
XLOC_004858	DEG(M)	No	No	No	KDN51027.1	--	--	--
XLOC_004859	DEG(M)	No	No	No	--	--	--	--
XLOC_004860	No	No	No	No	--	--	--	--
XLOC_004861	DEG(M)	No	No	No	CEL61932.1	--	--	--
XLOC_004862	No	No	No	No	AAU84940.1	--	G	K01834
XLOC_004863	No	No	No	No	--	--	--	--
XLOC_004864	No	No	No	No	CEL52099.1	--	--	--
XLOC_004866	No	No	No	No	--	--	G	K01807
XLOC_004867	No	No	No	No	--	--	--	--
XLOC_004868	No	No	No	No	XP_002516232.1	--	O	K04078
XLOC_004869	No	No	No	No	CEL59890.1	--	--	--
XLOC_004873	No	No	No	No	XP_001654138.1	--	--	--
XLOC_004876	DEG(M)	No	No	No	CCA69074.1	--	O	--
XLOC_004878	No	No	No	No	XP_008187304.1	--	--	--
XLOC_004880	No	No	No	No	--	--	--	--
XLOC_004881	DEG(M)	No	No	No	ETI36365.1	--	--	--
XLOC_004882	No	No	No	No	--	--	--	--
XLOC_004892	DEG(M)	No	No	No	EGU84042.1	--	--	--
XLOC_004894	No	No	DEG(I+M)	No	--	--	--	--
XLOC_004895	No	No	No	No	--	--	--	--
XLOC_004896	DEG(M)	No	No	No	--	--	--	--
XLOC_004901	No	No	DEG(I+M)	No	--	--	--	--
XLOC_004902	DEG(M)	DEG(M)	No	No	--	--	--	--
XLOC_004903	No	No	No	No	--	--	--	--
XLOC_004905	No	No	No	No	--	--	O	--
XLOC_004911	No	No	No	No	--	--	--	--
XLOC_004913	No	No	No	No	--	--	--	--
XLOC_004916	DEG(M)	No	No	No	XP_003041041.1	--	--	--

Differentially expressed gene (DEG) IDs	*C. microphyllum* root and shoot-specific (>2 fold)	*C. microphyllum* root and shoot-specific (>10 fold)	ICC4958 and *C. microphyllum* root-specific (>2 fold)	*C. microphyllum* root-specific (>2 fold)	Functional Annotation			
					NCBI nr IDs	TFs	KOG	KEGG
XLOC_004918	No	No	No	No	CEL52495.1	--	--	--
XLOC_004919	No	No	No	No	KEP50571.1	--	--	--
XLOC_004921	No	No	No	No	--	--	--	--
XLOC_004922	No	No	No	No	XP_004508497.1	ERF	K	--
XLOC_004923	DEG(M)	No	DEG(I+M)	DEG(M)	--	--	--	--
XLOC_004924	DEG(M)	No	No	No	--	--	--	--
XLOC_004927	DEG(M)	No	No	No	--	--	J	--
XLOC_004928	No	No	No	No	EUC59099.1	--	P	--
XLOC_004931	No	No	No	No	XP_002592130.1	bHLH	U	K07342
XLOC_004935	No	No	No	No	NP_001119696.2	--	--	--
XLOC_004936	No	No	No	No	--	--	--	--
XLOC_004939	No	No	No	No	XP_004341446.1	--	--	K11252
XLOC_004942	DEG(M)	No	No	No	NP_001155663.1	--	J	--
XLOC_004945	DEG(M)	No	No	No	ELU38745.1	--	--	--
XLOC_004946	No	No	No	No	KEP51823.1	--	--	--
XLOC_004949	No	No	No	No	KEP51727.1	--	--	--
XLOC_004950	No	No	No	No	XP_009525701.1	--	--	--
XLOC_004952	DEG(M)	No	No	No	XP_003051211.1	--	--	--
XLOC_004955	No	No	No	No	--	--	--	--
XLOC_004956	No	No	No	No	--	--	--	--
XLOC_004958	No	No	No	No	XP_001936808.1	--	--	--
XLOC_004960	No	No	No	No	--	--	--	K07305
XLOC_004964	DEG(M)	No	No	No	--	--	--	--
XLOC_004968	No	No	No	No	--	--	--	--
XLOC_004976	No	No	No	No	XP_004511007.1	--	--	--
XLOC_004977	No	No	No	No	--	--	--	--
XLOC_004980	No	No	No	No	--	--	--	--
XLOC_004991	No	No	No	No	--	--	--	--
XLOC_004992	No	No	No	No	--	--	A	--
XLOC_005000	No	No	No	No	XP_001653901.1	--	--	--
XLOC_005001	No	No	No	No	XP_001653901.1	--	--	--
XLOC_005009	No	No	DEG(I+M)	No	XP_004511088.1	--	R	--
XLOC_005013	No	No	No	No	--	--	--	--
XLOC_005014	DEG(M)	No	No	No	--	--	--	K11253
XLOC_005015	No	No	No	No	--	--	--	--
XLOC_005018	No	No	No	No	--	--	--	--
XLOC_005019	No	No	No	No	KDN48666.1	--	--	--
XLOC_005020	No	No	No	No	CEL55684.1	--	--	--
XLOC_005026	No	No	No	No	--	--	--	--
XLOC_005034	No	No	No	No	--	--	--	K14486

**Differentially expressed gene (DEG) IDs**	***C. microphyllum* root and shoot-specific (>2 fold)**	***C. microphyllum* root and shoot-specific (>10 fold)**	**ICC4958 and *C. microphyllum* root-specific (>2 fold)**	***C. microphyllum* root-specific (>2 fold)**	**Functional Annotation**			
					**NCBI nr IDs**	**TFs**	**KOG**	**KEGG**
XLOC_005035	No	No	No	No	--	--	--	--
XLOC_005041	No	No	No	No	--	--	--	K02864
XLOC_005043	DEG(M)	No	No	No	XP_009527005.1	--	--	K02137
XLOC_005044	No	No	No	No	--	--	--	--
XLOC_005046	DEG(M)	DEG(M)	No	No	--	--	--	--
XLOC_005047	DEG(M)	DEG(M)	DEG(I+M)	DEG(M)	CDY70361.1	YABBY	OR	--
XLOC_005051	DEG(M)	No	No	No	--	--	--	--
XLOC_005052	No	No	No	No	--	--	--	--
XLOC_005053	No	No	No	No	CEL52359.1	--	O	--
XLOC_005054	No	No	No	No	--	--	--	--
XLOC_005055	No	No	No	No	--	--	--	--
XLOC_005060	No	No	No	No	--	--	--	K08867
XLOC_005061	No	No	No	No	EPS62642.1	--	--	K03526
XLOC_005066	No	No	No	No	--	--	--	--
XLOC_005068	No	No	No	No	EUC66569.1	--	--	K03124
XLOC_005069	No	No	DEG(I+M)	No	--	--	--	--
XLOC_005071	No	No	No	No	--	--	--	--
XLOC_005077	DEG(M)	No	No	No	XP_002901172.1	--	J	--
XLOC_005078	No	No	No	No	XP_004498408.1	--	--	K14497
XLOC_005081	No	No	No	No	--	--	--	--
XLOC_005082	No	No	No	No	--	--	--	--
XLOC_005084	No	No	No	No	--	--	--	--
XLOC_005087	No	No	DEG(I+M)	No	--	--	R	--
XLOC_005088	No	No	DEG(I+M)	No	XP_004502439.1	--	--	--
XLOC_005091	DEG(M)	No	No	No	KDN35765.1	--	--	--
XLOC_005094	No	No	No	No	--	--	--	--
XLOC_005097	No	No	No	No	EUC60768.1	--	--	--
XLOC_005098	DEG(M)	No	No	No	--	--	J	--
XLOC_005101	DEG(M)	DEG(M)	No	No	--	--	--	--
XLOC_005107	No	No	No	No	XP_004494312.1	--	--	--
XLOC_005128	No	No	No	No	XP_004495565.1	--	--	--
XLOC_005129	DEG(M)	DEG(M)	No	No	--	--	--	--
XLOC_005133	DEG(M)	DEG(M)	No	No	XP_003537948.1	--	--	K00454
XLOC_005141	No	No	No	No	--	--	--	--
XLOC_005145	DEG(M)	No	No	No	--	--	--	--
XLOC_005160	DEG(M)	No	No	No	--	--	--	--
XLOC_005161	DEG(M)	No	No	No	--	--	--	--
XLOC_005162	No	No	No	No	--	--	--	--
XLOC_005164	No	No	No	No	--	--	--	--
XLOC_005177	No	No	No	No	CCO31672.1	--	Z	--

Differentially expressed gene (DEG) IDs	*C. microphyllum* root and shoot-specific (>2 fold)	*C. microphyllum* root and shoot-specific (>10 fold)	ICC4958 and *C. microphyllum* root-specific (>2 fold)	*C. microphyllum* root-specific (>2 fold)	Functional Annotation			
					NCBI nr IDs	TFs	KOG	KEGG
XLOC_005179	DEG(M)	No	No	No	KIM29562.1	--	Z	--
XLOC_005180	DEG(M)	DEG(M)	DEG(I+M)	DEG(M)	AAD50625.1	--	Z	--
XLOC_005183	No	No	No	No	--	--	--	--
XLOC_005190	DEG(M)	No	No	No	--	--	--	--
XLOC_005191	DEG(M)	No	No	No	--	--	--	--
XLOC_005194	No	No	No	No	--	--	Q	--
XLOC_005203	DEG(M)	No	No	No	XP_001649284.1	NAC	J	K03234
XLOC_005207	No	No	No	No	--	--	--	--
XLOC_005208	No	No	No	No	--	--	--	--
XLOC_005230	DEG(M)	No	No	No	--	--	I	K15397
XLOC_005233	No	No	No	No	XP_004498007.1	--	I	--
XLOC_005234	No	No	No	No	XP_004495403.1	--	I	K15397
XLOC_005235	No	No	No	No	--	--	--	--
XLOC_005236	No	No	No	No	--	--	I	K15397
XLOC_005266	DEG(M)	DEG(M)	DEG(I+M)	DEG(M)	--	--	--	--
XLOC_005267	DEG(M)	DEG(M)	DEG(I+M)	DEG(M)	--	--	--	--
XLOC_005294	No	No	No	No	--	--	E	--
XLOC_005295	No	No	No	No	XP_004490613.1	--	E	K01953
XLOC_005296	No	No	No	No	--	--	E	K01953
XLOC_005297	No	No	No	No	--	--	--	--
XLOC_005299	No	No	No	No	ETO76322.1	--	O	--
XLOC_005302	No	No	No	No	--	--	E	--
XLOC_005303	No	No	No	No	--	--	--	--
XLOC_005304	No	No	No	No	--	--	--	--
XLOC_005306	No	No	No	No	--	--	--	--
XLOC_005307	No	No	No	No	--	--	A	K14396
XLOC_005310	DEG(M)	No	No	No	CEL53227.1	--	UO	K10956
XLOC_005315	DEG(M)	No	No	No	--	--	--	K12741
XLOC_005318	No	No	No	No	--	--	P	K04565
XLOC_005320	No	No	DEG(I+M)	No	KEH25602.1	--	E	--
XLOC_005321	DEG(M)	No	DEG(I+M)	DEG(M)	EUC66575.1	--	--	--
XLOC_005324	No	No	No	No	--	--	--	--
XLOC_005326	No	No	No	No	XP_003050652.1	--	--	--
XLOC_005327	No	No	No	No	XP_008912119.1	--	--	--
XLOC_005331	No	No	No	No	--	--	--	--
XLOC_005334	DEG(M)	No	No	No	--	--	J	--
XLOC_005336	No	No	DEG(I+M)	No	--	--	--	--
XLOC_005339	No	No	No	No	CCO28819.1	--	--	--
XLOC_005341	No	No	No	No	XP_001948954.1	--	--	--
XLOC_005342	No	No	No	No	--	--	--	--

Differentially expressed gene (DEG) IDs	*C. microphyllum* root and shoot-specific (>2 fold)	*C. microphyllum* root and shoot-specific (>10 fold)	ICC4958 and *C. microphyllum* root-specific (>2 fold)	*C. microphyllum* root-specific (>2 fold)	Functional Annotation			
					NCBI nr IDs	TFs	KOG	KEGG
XLOC_005343	No	No	No	No	CCO30631.1	--	--	--
XLOC_005344	No	No	No	No	ELU41493.1	--	--	--
XLOC_005348	No	No	No	No	ETP42768.1	--	--	--
XLOC_005352	No	No	No	No	--	--	O	K09490
XLOC_005357	No	No	No	No	--	--	--	--
XLOC_005362	No	No	No	No	XP_002998420.1	WRKY	J	--
XLOC_005363	No	No	No	No	--	--	T	--
XLOC_005365	No	No	No	No	--	--	--	--
XLOC_005366	No	No	No	No	--	--	--	--
XLOC_005368	DEG(M)	No	No	No	--	NAC	J	--
XLOC_005369	No	No	No	No	XP_001939079.1	--	--	--
XLOC_005370	No	No	No	No	--	--	--	--
XLOC_005372	No	No	No	No	--	--	--	--
XLOC_005373	No	No	No	No	KIJ63737.1	--	H	--
XLOC_005375	No	No	No	No	--	--	--	--
XLOC_005377	No	No	No	No	--	--	K	K12604
XLOC_005380	DEG(M)	No	No	No	ETP22968.1	--	--	--
XLOC_005382	No	No	No	No	--	--	--	--
XLOC_005385	No	No	No	No	--	--	C	K02134
XLOC_005386	No	No	No	No	XP_008910149.1	--	--	--
XLOC_005388	No	No	No	No	--	--	--	--
XLOC_005389	No	No	No	No	--	--	--	--
XLOC_005391	No	No	No	No	--	--	--	--
XLOC_005394	No	No	No	No	--	--	--	--
XLOC_005396	DEG(M)	No	No	No	--	--	--	--
XLOC_005398	DEG(M)	DEG(M)	DEG(I+M)	DEG(M)	--	--	--	--
XLOC_005400	No	No	No	No	--	--	--	--
XLOC_005402	No	No	No	No	KDN45204.1	--	--	--
XLOC_005404	No	No	No	No	--	--	--	--
XLOC_005405	No	No	No	No	--	--	--	--
XLOC_005410	No	No	No	No	XP_004339708.1	--	R	K04392
XLOC_005416	No	No	No	No	BAF98280.1	--	I	K00021
XLOC_005417	No	No	No	No	--	NF-YC	B	K11251
XLOC_005419	DEG(M)	No	No	No	--	--	--	--
XLOC_005420	No	No	No	No	--	--	K	--
XLOC_005422	No	No	No	No	--	--	--	--
XLOC_005423	No	No	No	No	XP_003053821.1	--	--	--
XLOC_005426	No	No	No	No	--	--	--	--
XLOC_005427	No	No	No	No	NP_001155403.1	--	J	--
XLOC_005428	No	No	No	No	--	--	--	--

Differentially expressed gene (DEG) IDs	*C. microphyllum* root and shoot-specific (>2 fold)	*C. microphyllum* root and shoot-specific (>10 fold)	ICC4958 and *C. microphyllum* root-specific (>2 fold)	*C. microphyllum* root-specific (>2 fold)	Functional Annotation			
					NCBI nr IDs	TFs	KOG	KEGG
XLOC_005430	No	No	No	No	XP_001944657.2	--	--	K18753
XLOC_005431	DEG(M)	No	DEG(I+M)	DEG(M)	XP_003297441.1	--	--	--
XLOC_005432	No	No	No	No	--	--	--	K17619
XLOC_005433	DEG(M)	No	No	No	EUC66555.1	--	--	K12833
XLOC_005439	No	No	No	No	XP_003243749.1	--	TZ	--
XLOC_005441	No	No	No	No	CCO31688.1	--	--	--
XLOC_005442	No	No	No	No	XP_003053964.1	--	--	--
XLOC_005444	No	No	No	No	XP_001654164.1	--	--	--
XLOC_005448	DEG(M)	No	No	No	--	--	--	--
XLOC_005450	No	No	No	No	--	--	--	K13366
XLOC_005455	No	No	No	No	XP_002998705.1	--	--	K07955
XLOC_005457	No	No	No	No	ETM56090.1	--	--	K00654
XLOC_005467	DEG(M)	No	No	No	NP_001156420.1	--	O	--
XLOC_005469	No	No	No	No	XP_004497532.1	--	--	--
XLOC_005474	No	No	No	No	--	--	--	--
XLOC_005478	No	No	No	No	--	--	--	--
XLOC_005480	No	No	No	No	--	--	--	--
XLOC_005482	No	No	No	No	--	--	--	--
XLOC_005484	No	No	No	No	XP_009525743.1	--	--	--
XLOC_005486	No	No	No	No	--	--	--	--
XLOC_005489	No	No	No	No	--	--	--	--
XLOC_005495	No	No	No	No	--	--	A	K14563
XLOC_005496	No	No	No	No	ETO64498.1	--	--	--
XLOC_005498	No	No	No	No	XP_001864629.1	--	A	--
XLOC_005501	No	No	No	No	--	--	--	--
XLOC_005505	No	No	No	No	EUC56325.1	--	--	--
XLOC_005507	No	No	No	No	--	--	--	--
XLOC_005510	No	No	No	No	NP_001155431.1	--	--	--
XLOC_005511	No	No	No	No	XP_013294803.1	--	UO	--
XLOC_005512	No	No	No	No	CEL55303.1	--	--	--
XLOC_005513	No	No	No	No	--	--	--	K12741
XLOC_005517	No	No	No	No	--	--	--	--
XLOC_005519	No	No	No	No	XP_002904370.1	--	--	--
XLOC_005521	DEG(M)	DEG(M)	No	No	--	--	--	--
XLOC_005525	No	No	No	No	--	--	--	--
XLOC_005526	No	No	No	No	XP_008893118.1	--	--	--
XLOC_005527	No	No	No	No	--	--	--	--
XLOC_005529	No	No	No	No	--	--	--	K00232
XLOC_005530	No	No	No	No	--	--	--	--
XLOC_005533	DEG(M)	No	No	No	KDN50292.1	--	--	--

Differentially expressed gene (DEG) IDs	*C. microphyllum* root and shoot-specific (>2 fold)	*C. microphyllum* root and shoot-specific (>10 fold)	ICC4958 and *C. microphyllum* root-specific (>2 fold)	*C. microphyllum* root-specific (>2 fold)	Functional Annotation			
					NCBI nr IDs	TFs	KOG	KEGG
XLOC_005537	DEG(M)	No	No	No	KDN34571.1	--	--	K05670
XLOC_005540	No	No	No	No	CCO27985.1	--	--	--
XLOC_005541	DEG(M)	No	No	No	CEL58758.1	--	--	--
XLOC_005542	DEG(M)	No	No	No	ETM45825.1	--	--	--
XLOC_005549	DEG(M)	No	No	No	--	--	G	--
XLOC_005552	DEG(M)	No	No	No	KDN37651.1	--	--	--
XLOC_005553	No	No	No	No	ETK90967.1	--	--	K00793
XLOC_005558	DEG(M)	No	No	No	--	--	--	--
XLOC_005559	No	No	No	No	--	--	G	--
XLOC_005561	No	No	No	No	KDN37063.1	--	--	--
XLOC_005564	DEG(M)	No	No	No	--	--	--	--
XLOC_005569	No	No	No	No	--	--	--	--
XLOC_005572	No	No	No	No	--	--	--	--
XLOC_005576	No	No	No	No	--	--	--	--
XLOC_005577	No	No	No	No	--	--	--	--
XLOC_005583	No	No	No	No	--	--	--	--
XLOC_005585	No	No	No	No	--	--	--	--
XLOC_005588	No	No	No	No	XP_002526727.1	--	F	--
XLOC_005589	No	No	No	No	XP_002532081.1	--	FE	--
XLOC_005600	DEG(M)	No	No	No	--	--	--	--
XLOC_005602	No	No	No	No	--	--	--	--
XLOC_005605	DEG(M)	No	No	No	--	--	--	K02943
XLOC_005608	DEG(M)	No	No	No	CCO28161.1	--	--	--
XLOC_005610	No	No	No	No	--	--	--	--
XLOC_005618	No	No	No	No	--	--	--	--
XLOC_005619	No	No	DEG(I+M)	No	XP_004499033.1	--	--	--
XLOC_005620	DEG(M)	DEG(M)	DEG(I+M)	DEG(M)	--	--	--	--
XLOC_005622	No	No	No	No	--	--	--	--
XLOC_005624	No	No	No	No	--	--	--	K02707
XLOC_005630	DEG(M)	No	No	No	--	--	--	--
XLOC_005633	DEG(M)	DEG(M)	No	No	XP_003051997.1	--	E	--
XLOC_005634	No	No	No	No	--	--	--	--
XLOC_005637	No	No	No	No	--	--	--	--
XLOC_005640	No	No	No	No	KFM79307.1	--	UO	K10956
XLOC_005643	DEG(M)	No	No	No	NP_001156223.1	--	--	--
XLOC_005646	DEG(M)	No	DEG(I+M)	DEG(M)	--	--	--	K01723
XLOC_005647	No	No	No	No	--	--	--	--
XLOC_005649	No	No	No	No	KDN41016.1	--	--	--
XLOC_005651	No	No	No	No	XP_012087370.1	--	V	K01011
XLOC_005655	No	No	No	No	--	--	--	--

Differentially expressed gene (DEG) IDs	*C. microphyllum* root and shoot-specific (>2 fold)	*C. microphyllum* root and shoot-specific (>10 fold)	ICC4958 and *C. microphyllum* root-specific (>2 fold)	*C. microphyllum* root-specific (>2 fold)	Functional Annotation			
					NCBI nr IDs	TFs	KOG	KEGG
XLOC_005676	DEG(M)	No	DEG(I+M)	DEG(M)	--	--	--	--
XLOC_005678	No	No	DEG(I+M)	No	XP_004500392.1	--	T	--
XLOC_005679	No	No	DEG(I+M)	No	--	--	T	--
XLOC_005683	No	No	No	No	ELU42849.1	--	--	K14640
XLOC_005695	No	No	DEG(I+M)	No	XP_004503039.1	--	--	K13448
XLOC_005696	DEG(M)	No	No	No	--	--	--	--
XLOC_005697	DEG(M)	DEG(M)	No	No	--	--	--	--
XLOC_005698	DEG(M)	DEG(M)	No	No	--	--	--	--
XLOC_005699	DEG(M)	DEG(M)	No	No	--	--	--	--
XLOC_005700	DEG(M)	No	No	No	--	--	--	--
XLOC_005701	DEG(M)	DEG(M)	No	No	--	--	--	--
XLOC_005703	DEG(M)	DEG(M)	No	No	--	--	--	--
XLOC_005704	No	No	DEG(I+M)	No	XP_004510691.1	WRKY	--	K13425
XLOC_005709	No	No	No	No	EUC57452.1	--	--	--
XLOC_005710	DEG(M)	No	No	No	EUC57452.1	--	--	--
XLOC_005714	No	No	DEG(I+M)	No	--	--	--	--
XLOC_005715	No	No	No	No	--	--	--	--
XLOC_005716	DEG(M)	No	No	No	--	--	AJ	K13126
XLOC_005718	DEG(M)	No	DEG(I+M)	DEG(M)	KEP49077.1	--	--	--
XLOC_005719	No	No	No	No	CCO36240.1	--	--	--
XLOC_005720	DEG(M)	No	No	No	CCO36240.1	--	--	--
XLOC_005721	DEG(M)	DEG(M)	DEG(I+M)	DEG(M)	CCO36240.1	--	--	--
XLOC_005745	No	No	No	No	XP_001943247.2	--	--	K07359
XLOC_005748	No	No	No	No	--	--	--	--
XLOC_005775	DEG(M)	No	No	No	EUC64368.1	--	G	K00963
XLOC_005780	No	No	DEG(I+M)	No	--	--	--	--
XLOC_005781	No	No	DEG(I+M)	No	--	--	--	--
XLOC_005805	DEG(M)	DEG(M)	No	No	--	--	--	--
XLOC_005813	DEG(M)	DEG(M)	No	No	--	--	Q	--
XLOC_005831	DEG(M)	No	No	No	--	--	--	--
XLOC_005832	DEG(M)	No	DEG(I+M)	DEG(M)	--	--	--	--
XLOC_005851	DEG(M)	No	No	No	EXL94780.1	--	--	--
XLOC_005853	No	No	No	No	--	--	--	--
XLOC_005870	No	No	No	No	--	--	--	--
XLOC_005881	DEG(M)	No	No	No	--	--	--	--
XLOC_005883	No	No	No	No	XP_001653504.1	--	C	--
XLOC_005884	No	No	No	No	EUC67258.1	--	--	--
XLOC_005887	No	No	No	No	CEL62744.1	--	--	K01194
XLOC_005888	No	No	No	No	XP_012091825.1	--	G	K16055
XLOC_005890	No	No	No	No	ETI31944.1	--	E	--

Differentially expressed gene (DEG) IDs	*C. microphyllum* root and shoot-specific (>2 fold)	*C. microphyllum* root and shoot-specific (>10 fold)	ICC4958 and *C. microphyllum* root-specific (>2 fold)	*C. microphyllum* root-specific (>2 fold)	Functional Annotation			
					NCBI nr IDs	TFs	KOG	KEGG
XLOC_005892	No	No	No	No	--	--	--	--
XLOC_005893	No	No	No	No	--	--	--	--
XLOC_005896	DEG(M)	DEG(M)	No	No	--	--	--	--
XLOC_005897	No	No	No	No	--	--	--	--
XLOC_005898	DEG(M)	No	No	No	ETI44035.1	--	--	--
XLOC_005899	No	No	No	No	--	--	--	--
XLOC_005905	No	No	No	No	ETM00251.1	G2-like	E	--
XLOC_005912	DEG(M)	DEG(M)	DEG(I+M)	DEG(M)	KEH32211.1	--	R	--
XLOC_005913	No	No	No	No	--	--	--	--
XLOC_005917	No	No	No	No	--	--	--	--
XLOC_005919	No	No	No	No	XP_008915460.1	--	--	--
XLOC_005924	DEG(M)	No	No	No	--	--	--	--
XLOC_005925	No	No	No	No	--	--	--	--
XLOC_005927	No	No	No	No	XP_011493706.1	--	--	--
XLOC_005928	DEG(M)	No	No	No	XP_007013947.1	--	M	K08679
XLOC_005933	No	No	No	No	--	--	--	--
XLOC_005934	DEG(M)	No	No	No	--	--	--	K04078
XLOC_005938	No	No	No	No	EXK25452.1	--	--	--
XLOC_005939	No	No	No	No	--	--	--	--
XLOC_005940	DEG(M)	No	No	No	XP_003043292.1	--	--	--
XLOC_005942	DEG(M)	No	No	No	--	--	--	--
XLOC_005943	No	No	No	No	XP_003046987.1	--	--	--
XLOC_005944	DEG(M)	No	No	No	--	--	--	--
XLOC_005946	DEG(M)	No	No	No	--	--	--	--
XLOC_005947	No	No	No	No	EUC66126.1	--	--	--
XLOC_005950	DEG(M)	No	No	No	--	--	--	K00799
XLOC_005952	No	No	No	No	--	--	--	--
XLOC_005954	No	No	No	No	--	--	--	--
XLOC_005956	DEG(M)	No	DEG(I+M)	DEG(M)	--	--	--	--
XLOC_005958	No	No	No	No	KDN50472.1	--	--	--
XLOC_005963	No	No	No	No	--	--	X	--
XLOC_005967	No	No	No	No	XP_006814940.1	--	O	K03033
XLOC_005972	No	No	No	No	--	--	--	--
XLOC_005977	No	No	No	No	EUC56630.1	--	--	K00799
XLOC_005981	No	No	No	No	--	--	--	--
XLOC_005982	No	No	No	No	--	--	--	--
XLOC_005983	DEG(M)	No	No	No	--	--	--	--
XLOC_005984	No	No	No	No	EUC64282.1	--	--	K13342
XLOC_005985	No	No	No	No	--	--	--	--
XLOC_005989	DEG(M)	No	No	No	CEL57796.1	--	--	--

Differentially expressed gene (DEG) IDs	*C. microphyllum* root and shoot-specific (>2 fold)	*C. microphyllum* root and shoot-specific (>10 fold)	ICC4958 and *C. microphyllum* root-specific (>2 fold)	*C. microphyllum* root-specific (>2 fold)	Functional Annotation			
					NCBI nr IDs	TFs	KOG	KEGG
XLOC_005996	DEG(M)	No	No	No	XP_008899457.1	--	--	K08738
XLOC_006001	No	No	No	No	EUC61741.1	--	--	--
XLOC_006004	No	No	No	No	--	--	--	--
XLOC_006005	No	No	No	No	--	--	--	--
XLOC_006006	DEG(M)	No	No	No	--	--	--	--
XLOC_006007	No	No	No	No	--	--	--	--
XLOC_006011	DEG(M)	No	No	No	--	--	--	--
XLOC_006013	DEG(M)	No	DEG(I+M)	DEG(M)	--	--	--	--
XLOC_006017	No	No	No	No	--	--	--	--
XLOC_006018	No	No	No	No	XP_008181460.1	--	--	--
XLOC_006023	No	No	No	No	--	--	--	--
XLOC_006024	DEG(M)	No	No	No	XP_001649058.1	--	--	K02943
XLOC_006025	No	No	No	No	XP_008896171.1	--	--	--
XLOC_006031	DEG(M)	No	No	No	ABE68625.1	--	Z	--
XLOC_006035	No	No	No	No	--	--	--	--
XLOC_006036	No	No	No	No	--	--	--	--
XLOC_006037	No	No	No	No	NP_001280296.1	--	U	--
XLOC_006040	No	No	DEG(I+M)	No	--	--	--	--
XLOC_006041	No	No	DEG(I+M)	No	--	--	--	--
XLOC_006043	No	No	No	No	--	--	--	--
XLOC_006044	No	No	No	No	ENH66024.1	--	--	--
XLOC_006046	No	No	No	No	EWY89556.1	--	--	--
XLOC_006047	No	No	No	No	XP_001656392.1	--	JT	K03246
XLOC_006048	No	No	No	No	KDN40070.1	--	--	--
XLOC_006051	No	No	No	No	CEL63741.1	--	--	--
XLOC_006055	No	No	No	No	XP_002523482.1	--	E	K00013
XLOC_006058	No	No	No	No	XP_002529686.1	--	--	K09597
XLOC_006059	No	No	No	No	CEL56035.1	--	--	--
XLOC_006061	No	No	No	No	KDN47854.1	--	--	K15100
XLOC_006062	No	No	No	No	--	--	--	--
XLOC_006067	No	No	No	No	--	--	--	--
XLOC_006069	No	No	No	No	--	--	--	--
XLOC_006070	DEG(M)	No	No	No	--	--	--	--
XLOC_006072	No	No	No	No	XP_003053408.1	--	--	--
XLOC_006073	No	No	No	No	XP_008181998.1	--	--	--
XLOC_006075	No	No	No	No	XP_003046325.1	--	--	--
XLOC_006078	No	No	No	No	--	--	--	--
XLOC_006080	No	No	No	No	XP_001947666.2	--	--	--
XLOC_006082	No	No	No	No	XP_008908083.1	--	O	--
XLOC_006084	No	No	DEG(I+M)	No	EMT61471.1	--	C	--

Differentially expressed gene (DEG) IDs	*C. microphyllum* root and shoot-specific (>2 fold)	*C. microphyllum* root and shoot-specific (>10 fold)	ICC4958 and *C. microphyllum* root-specific (>2 fold)	*C. microphyllum* root-specific (>2 fold)	Functional Annotation			
					NCBI nr IDs	TFs	KOG	KEGG
XLOC_006087	No	No	No	No	CEL52376.1	--	--	--
XLOC_006088	No	No	No	No	CCO27556.1	--	--	--
XLOC_006089	DEG(M)	No	No	No	--	--	--	--
XLOC_006091	No	No	No	No	CEL57973.1	--	M	K00820
XLOC_006095	No	No	No	No	XP_003048258.1	--	--	--
XLOC_006099	No	No	No	No	--	--	--	--
XLOC_006106	DEG(M)	No	No	No	ELU41516.1	--	--	--
XLOC_006107	DEG(M)	No	No	No	--	--	--	--
XLOC_006109	DEG(M)	No	No	No	ELU39252.1	--	J	--
XLOC_006111	DEG(M)	No	No	No	--	--	--	--
XLOC_006113	DEG(M)	No	No	No	ABA97973.2	--	OK	--
XLOC_006116	No	No	DEG(I+M)	No	--	--	--	K00430
XLOC_006117	DEG(M)	No	No	No	--	--	--	--
XLOC_006121	DEG(M)	No	No	No	--	--	--	--
XLOC_006122	No	No	No	No	XP_008914027.1	--	--	--
XLOC_006123	No	No	No	No	--	--	--	--
XLOC_006124	No	No	No	No	--	--	--	--
XLOC_006127	No	No	No	No	NP_001119667.1	--	--	--
XLOC_006128	No	No	No	No	--	--	--	--
XLOC_006129	DEG(M)	No	No	No	CEL61469.1	--	--	--
XLOC_006151	DEG(M)	No	No	No	--	--	--	--
XLOC_006152	No	No	No	No	--	--	--	--
XLOC_006155	No	No	No	No	NP_001191869.1	--	--	--
XLOC_006156	No	No	No	No	CEL63913.1	--	--	--
XLOC_006157	No	No	No	No	--	--	--	--
XLOC_006158	DEG(M)	No	No	No	--	--	--	--
XLOC_006159	No	No	No	No	XP_003245854.2	--	--	--
XLOC_006161	No	No	No	No	--	--	--	--
XLOC_006165	No	No	No	No	--	--	J	--
XLOC_006166	No	No	DEG(I+M)	No	XP_004510320.1	--	--	--
XLOC_006168	DEG(M)	No	No	No	--	--	--	--
XLOC_006173	No	No	No	No	--	--	--	--
XLOC_006181	No	No	No	No	CCO28609.1	--	--	--
XLOC_006185	No	No	No	No	EUC66719.1	--	--	--
XLOC_006188	No	No	No	No	--	--	--	--
XLOC_006192	DEG(M)	No	DEG(I+M)	DEG(M)	XP_008909974.1	--	--	--
XLOC_006193	No	No	No	No	--	--	--	--
XLOC_006196	DEG(M)	No	No	No	--	--	--	K02734
XLOC_006198	No	No	No	No	--	--	--	--
XLOC_006199	No	No	No	No	--	--	--	--

Differentially expressed gene (DEG) IDs	*C. microphyllum* root and shoot-specific (>2 fold)	*C. microphyllum* root and shoot-specific (>10 fold)	ICC4958 and *C. microphyllum* root-specific (>2 fold)	*C. microphyllum* root-specific (>2 fold)	Functional Annotation			
					NCBI nr IDs	TFs	KOG	KEGG
XLOC_006200	DEG(M)	No	No	No	XP_008901188.1	--	--	--
XLOC_006203	DEG(M)	No	No	No	KDN45848.1	--	J	--
XLOC_006204	No	No	No	No	--	--	--	--
XLOC_006205	No	No	No	No	--	--	--	--
XLOC_006209	No	No	No	No	--	--	--	--
XLOC_006210	No	No	No	No	--	--	--	--
XLOC_006211	No	No	No	No	--	--	--	--
XLOC_006213	No	No	No	No	--	--	--	--
XLOC_006214	DEG(M)	No	No	No	--	--	--	--
XLOC_006215	No	No	No	No	XP_002515180.1	--	J	K01881
XLOC_006217	No	No	No	No	--	--	--	--
XLOC_006219	No	No	No	No	--	C2H2	K	K03136
XLOC_006220	No	No	No	No	XP_012575045.1	--	X	--
XLOC_006221	No	No	No	No	--	--	--	--
XLOC_006224	No	No	No	No	XP_003042384.1	--	--	--
XLOC_006229	DEG(M)	DEG(M)	No	No	EWZ91383.1	--	--	--
XLOC_006234	No	No	No	No	XP_003040721.1	--	CE	--
XLOC_006236	No	No	No	No	--	--	--	--
XLOC_006237	No	No	DEG(I+M)	No	EWZ00906.1	--	J	--
XLOC_006239	DEG(M)	DEG(M)	No	No	--	--	--	--
XLOC_006240	No	No	No	No	--	--	--	--
XLOC_006241	No	No	No	No	KDN44650.1	--	--	--
XLOC_006242	No	No	No	No	--	--	--	--
XLOC_006247	No	No	DEG(I+M)	No	NP_001265993.1	C2H2	--	--
XLOC_006248	DEG(M)	No	No	No	--	--	--	--
XLOC_006249	No	No	DEG(I+M)	No	--	--	--	K00430
XLOC_006250	No	No	No	No	--	--	--	K15040
XLOC_006251	No	No	No	No	EUC67782.1	--	--	--
XLOC_006252	No	No	No	No	--	--	--	--
XLOC_006253	No	No	No	No	YP_002149777.1	--	--	--
XLOC_006255	DEG(M)	No	No	No	--	--	--	--
XLOC_006257	No	No	No	No	--	--	--	--
XLOC_006259	No	No	No	No	--	--	--	--
XLOC_006262	No	No	No	No	--	--	--	K01520
XLOC_006263	No	No	No	No	--	--	--	--
XLOC_006264	No	No	No	No	NP_001155671.1	--	E	--
XLOC_006265	No	No	No	No	KEP51003.1	--	--	--
XLOC_006266	No	No	No	No	CEL57813.1	--	--	--
XLOC_006270	No	No	No	No	--	--	--	--
XLOC_006272	No	No	No	No	KFM78577.1	--	G	--

Differentially expressed gene (DEG) IDs	*C. microphyllum* root and shoot-specific (>2 fold)	*C. microphyllum* root and shoot-specific (>10 fold)	ICC4958 and *C. microphyllum* root-specific (>2 fold)	*C. microphyllum* root-specific (>2 fold)	Functional Annotation			
					NCBI nr IDs	TFs	KOG	KEGG
XLOC_006273	No	No	No	No	--	--	G	--
XLOC_006276	DEG(M)	No	No	No	--	--	--	--
XLOC_006277	No	No	No	No	KDP40434.1	--	A	K14811
XLOC_006280	No	No	No	No	--	--	--	--
XLOC_006281	No	No	No	No	--	--	--	--
XLOC_006282	No	No	No	No	--	--	R	--
XLOC_006284	No	No	No	No	XP_003248223.1	--	OW	--
XLOC_006286	No	No	No	No	XP_008179228.1	--	--	K10357
XLOC_006288	No	No	No	No	CEL54552.1	--	--	--
XLOC_006294	No	No	No	No	NP_001280387.1	--	--	--
XLOC_006297	No	No	No	No	--	--	--	--
XLOC_006301	No	No	No	No	XP_012089820.1	--	R	--
XLOC_006302	No	No	No	No	ENI04649.1	--	--	--
XLOC_006304	DEG(M)	No	No	No	--	--	--	--
XLOC_006307	No	No	No	No	--	--	--	--
XLOC_006309	No	No	No	No	XP_003053407.1	--	--	--
XLOC_006311	No	No	No	No	--	--	--	--
XLOC_006312	No	No	No	No	--	--	--	--
XLOC_006313	DEG(M)	DEG(M)	No	No	--	--	--	--
XLOC_006315	No	No	No	No	XP_003052049.1	--	--	--
XLOC_006318	No	No	No	No	--	--	--	--
XLOC_006319	No	No	No	No	KDN49687.1	--	O	--
XLOC_006321	No	No	No	No	--	--	--	K11584
XLOC_006324	No	No	No	No	--	--	--	--
XLOC_006326	DEG(M)	No	No	No	--	--	--	--
XLOC_006329	No	No	No	No	EUC67194.1	--	--	--
XLOC_006332	No	No	No	No	--	--	--	--
XLOC_006333	No	No	No	No	XP_008904930.1	--	--	--
XLOC_006334	No	No	No	No	BAJ93614.1	--	O	--
XLOC_006335	No	No	No	No	XP_003048505.1	--	--	--
XLOC_006337	No	No	No	No	--	--	R	K15403
XLOC_006340	DEG(M)	No	No	No	--	--	--	--
XLOC_006341	DEG(M)	No	No	No	--	--	--	--
XLOC_006342	No	No	No	No	--	--	--	--
XLOC_006343	DEG(M)	No	No	No	--	--	--	--
XLOC_006344	No	No	No	No	--	--	--	--
XLOC_006345	No	No	No	No	--	--	--	--
XLOC_006346	No	No	No	No	CEL60581.1	--	--	--
XLOC_006348	No	No	No	No	--	--	U	K12391
XLOC_006349	DEG(M)	DEG(M)	No	No	--	--	--	--

Differentially expressed gene (DEG) IDs	*C. microphyllum* root and shoot-specific (>2 fold)	*C. microphyllum* root and shoot-specific (>10 fold)	ICC4958 and *C. microphyllum* root-specific (>2 fold)	*C. microphyllum* root-specific (>2 fold)	Functional Annotation			
					NCBI nr IDs	TFs	KOG	KEGG
XLOC_006350	DEG(M)	No	No	No	XP_007336660.1	--	--	--
XLOC_006352	DEG(M)	No	No	No	XP_008898003.1	--	--	--
XLOC_006354	No	No	No	No	XP_001653618.1	--	--	--
XLOC_006357	DEG(M)	No	No	No	--	--	--	--
XLOC_006360	No	No	No	No	EUC63814.1	--	--	--
XLOC_006363	No	No	No	No	--	--	--	K19033
XLOC_006364	DEG(M)	No	DEG(I+M)	DEG(M)	--	--	--	--
XLOC_006365	No	No	No	No	--	--	--	--
XLOC_006366	No	No	No	No	CEL60698.1	--	--	--
XLOC_006367	No	No	No	No	--	--	--	--
XLOC_006368	No	No	No	No	CCO26501.1	--	Q	--
XLOC_006369	No	No	No	No	CCO26746.1	--	--	--
XLOC_006372	No	No	No	No	KEP52316.1	--	--	--
XLOC_006378	DEG(M)	No	No	No	CEL51568.1	--	P	--
XLOC_006379	No	No	No	No	XP_008184922.1	--	E	--
XLOC_006380	No	No	No	No	--	--	--	--
XLOC_006382	DEG(M)	DEG(M)	No	No	--	--	--	--
XLOC_006384	No	No	No	No	EUC66454.1	--	--	--
XLOC_006385	No	No	No	No	--	--	T	--
XLOC_006386	DEG(M)	No	No	No	CAA53368.1	--	O	--
XLOC_006388	No	No	No	No	--	--	--	--
XLOC_006390	DEG(M)	No	No	No	--	--	--	--
XLOC_006399	No	No	No	No	--	--	--	--
XLOC_006400	No	No	No	No	KFM80225.1	--	R	--
XLOC_006405	No	No	No	No	--	--	--	--
XLOC_006406	No	No	No	No	--	--	--	K01674
XLOC_006407	No	No	No	No	CCO26303.1	--	--	--
XLOC_006410	No	No	No	No	--	--	--	--
XLOC_006411	No	No	No	No	CEL62885.1	--	--	--
XLOC_006412	No	No	No	No	EXM10768.1	--	--	K14404
XLOC_006413	No	No	No	No	XP_003050503.1	--	C	--
XLOC_006414	No	No	No	No	CEL59822.1	--	--	--
XLOC_006416	No	No	No	No	--	--	--	--
XLOC_006420	No	No	No	No	XP_004506104.1	YABBY	X	--
XLOC_006421	DEG(M)	DEG(M)	No	No	--	--	--	--
XLOC_006422	No	No	No	No	--	--	--	--
XLOC_006424	DEG(M)	No	No	No	--	--	--	--
XLOC_006425	DEG(M)	No	No	No	--	--	--	--
XLOC_006426	DEG(M)	No	No	No	--	--	--	--
XLOC_006430	DEG(M)	No	No	No	XP_008605167.1	--	H	--

Differentially expressed gene (DEG) IDs	*C. microphyllum* root and shoot-specific (>2 fold)	*C. microphyllum* root and shoot-specific (>10 fold)	ICC4958 and *C. microphyllum* root-specific (>2 fold)	*C. microphyllum* root-specific (>2 fold)	Functional Annotation			
					NCBI nr IDs	TFs	KOG	KEGG
XLOC_006433	No	No	No	No	--	--	--	--
XLOC_006435	DEG(M)	No	No	No	--	--	--	--
XLOC_006438	No	No	No	No	--	--	--	K04712
XLOC_006439	DEG(M)	No	No	No	--	--	--	--
XLOC_006441	No	No	No	No	--	--	--	--
XLOC_006443	No	No	No	No	--	--	--	--
XLOC_006446	No	No	No	No	--	--	--	--
XLOC_006448	No	No	No	No	--	--	--	--
XLOC_006449	DEG(M)	DEG(M)	No	No	--	--	--	--
XLOC_006451	DEG(M)	No	No	No	CCO26591.1	--	--	K11252
XLOC_006452	DEG(M)	No	No	No	XP_008899328.1	--	--	K02137
XLOC_006453	DEG(M)	No	No	No	XP_012572898.1	--	--	--
XLOC_006462	DEG(M)	No	No	No	CEL53647.1	--	J	--
XLOC_006480	DEG(M)	DEG(M)	DEG(I+M)	DEG(M)	--	--	--	--
XLOC_006482	DEG(M)	No	No	No	CCO30752.1	--	C	--
XLOC_006483	DEG(M)	No	No	No	KDN44010.1	--	C	--
XLOC_006484	No	No	No	No	CCO30752.1	--	C	--
XLOC_006489	DEG(M)	DEG(M)	No	No	--	--	--	--
XLOC_006490	DEG(M)	No	No	No	XP_004494533.1	--	--	--
XLOC_006520	DEG(M)	DEG(M)	No	No	--	--	--	--
XLOC_006521	DEG(M)	DEG(M)	DEG(I+M)	DEG(M)	--	--	--	--
XLOC_006529	No	No	DEG(I+M)	No	--	--	--	--
XLOC_006532	DEG(M)	No	No	No	--	--	--	--
XLOC_006536	DEG(M)	DEG(M)	No	No	XP_001933362.1	--	J	--
XLOC_006537	DEG(M)	DEG(M)	DEG(I+M)	DEG(M)	KMP04494.1	--	J	--
XLOC_006540	DEG(M)	No	No	No	KIO27800.1	--	O	--
XLOC_006541	DEG(M)	No	No	No	--	--	--	--
XLOC_006543	DEG(M)	No	No	No	XP_004340430.1	--	J	--
XLOC_006545	DEG(M)	DEG(M)	DEG(I+M)	DEG(M)	ADI78974.1	--	Z	--
XLOC_006546	DEG(M)	DEG(M)	DEG(I+M)	DEG(M)	AAL30373.1	--	Z	--
XLOC_006549	DEG(M)	No	No	No	XP_003036735.1	--	U	K07937
XLOC_006553	DEG(M)	No	No	No	AAI59362.1	--	Z	--
XLOC_006554	DEG(M)	No	No	No	ETM53018.1	--	Z	--
XLOC_006555	No	No	No	No	ETM53037.1	--	Z	--
XLOC_006560	No	No	No	No	--	--	--	--
XLOC_006568	DEG(M)	No	No	No	KDN48746.1	--	--	--
XLOC_006572	No	No	No	No	XP_008897105.1	--	--	--
XLOC_006576	No	No	No	No	--	--	O	--
XLOC_006577	DEG(M)	DEG(M)	No	No	--	--	--	--
XLOC_006587	No	No	No	No	AAM29179.1	--	O	K17081

Differentially expressed gene (DEG) IDs	*C. microphyllum* root and shoot-specific (>2 fold)	*C. microphyllum* root and shoot-specific (>10 fold)	ICC4958 and *C. microphyllum* root-specific (>2 fold)	*C. microphyllum* root-specific (>2 fold)	Functional Annotation			
					NCBI nr IDs	TFs	KOG	KEGG
XLOC_006588	No	No	DEG(I+M)	No	XP_004489308.1	--	R	--
XLOC_006591	No	No	DEG(I+M)	No	--	--	--	--
XLOC_006592	No	No	No	No	--	--	--	K12125
XLOC_006594	DEG(M)	No	No	No	EUC65707.1	--	C	--
XLOC_006595	No	No	No	No	XP_002998081.1	--	C	--
XLOC_006596	DEG(M)	No	No	No	XP_002998081.1	--	--	--
XLOC_006597	No	No	No	No	--	--	--	--
XLOC_006600	DEG(M)	No	No	No	--	--	--	--
XLOC_006601	No	No	No	No	XP_001662259.1	--	S	--
XLOC_006602	No	No	No	No	ACY44807.1	--	U	K04646
XLOC_006603	No	No	No	No	ACY44843.1	--	U	--
XLOC_006607	DEG(M)	No	No	No	EUC63145.1	--	--	--
XLOC_006609	No	No	No	No	--	--	--	--
XLOC_006610	No	No	No	No	--	--	--	--
XLOC_006612	DEG(M)	No	DEG(I+M)	DEG(M)	XP_003040090.1	MYB_related	J	--
XLOC_006613	No	No	No	No	EUC59793.1	--	--	--
XLOC_006620	No	No	No	No	--	--	--	--
XLOC_006624	No	No	No	No	--	--	--	--
XLOC_006625	No	No	No	No	AGT99183.1	--	--	--
XLOC_006630	No	No	No	No	--	--	--	--
XLOC_006631	DEG(M)	No	No	No	--	--	--	--
XLOC_006633	DEG(M)	DEG(M)	No	No	XP_001950683.2	--	--	--
XLOC_006634	No	No	No	No	--	--	--	--
XLOC_006635	No	No	No	No	XP_003044819.1	--	A	--
XLOC_006636	No	No	No	No	--	--	--	--
XLOC_006637	No	No	No	No	XP_001947458.1	--	--	--
XLOC_006638	DEG(M)	No	No	No	--	--	--	--
XLOC_006642	No	No	No	No	EXK87275.1	--	--	--
XLOC_006643	No	No	DEG(I+M)	No	XP_003624413.1	--	--	--
XLOC_006649	No	No	No	No	ETI45962.1	--	--	--
XLOC_006650	DEG(M)	No	No	No	--	--	--	--
XLOC_006651	No	No	No	No	--	--	--	--
XLOC_006655	No	No	No	No	EUC64132.1	--	--	K17278
XLOC_006659	DEG(M)	No	No	No	--	--	--	--
XLOC_006661	DEG(M)	No	No	No	--	--	--	--
XLOC_006663	No	No	No	No	XP_004492905.1	--	--	--
XLOC_006666	No	No	No	No	XP_003041075.1	--	G	--
XLOC_006672	DEG(M)	No	No	No	--	--	--	--
XLOC_006674	No	No	No	No	XP_001655246.1	--	U	--
XLOC_006675	No	No	No	No	--	--	F	K00940

Differentially expressed gene (DEG) IDs	*C. microphyllum* root and shoot-specific (>2 fold)	*C. microphyllum* root and shoot-specific (>10 fold)	ICC4958 and *C. microphyllum* root-specific (>2 fold)	*C. microphyllum* root-specific (>2 fold)	Functional Annotation			
					NCBI nr IDs	TFs	KOG	KEGG
XLOC_006676	No	No	No	No	--	--	--	--
XLOC_006677	No	No	No	No	XP_008185845.1	--	--	--
XLOC_006680	DEG(M)	No	No	No	ETI44773.1	--	--	--
XLOC_006683	DEG(M)	No	No	No	AHZ64890.1	--	C	K02133
XLOC_006684	No	No	No	No	--	--	--	--
XLOC_006686	No	No	No	No	EUC59769.1	--	E	K01940
XLOC_006687	No	No	No	No	ETK79926.1	--	--	--
XLOC_006695	DEG(M)	No	No	No	--	--	--	--
XLOC_006698	No	No	No	No	AHX56890.1	--	--	--
XLOC_006701	No	No	No	No	--	--	--	--
XLOC_006702	DEG(M)	No	No	No	NP_001119643.1	--	J	--
XLOC_006704	No	No	No	No	KEP52917.1	--	--	--
XLOC_006710	No	No	No	No	--	--	--	--
XLOC_006713	DEG(M)	No	No	No	ABI52808.1	NF-YB	J	K02973
XLOC_006714	No	No	No	No	--	--	--	--
XLOC_006716	No	No	No	No	--	--	J	--
XLOC_006717	No	No	No	No	XP_008914604.1	--	--	--
XLOC_006720	No	No	No	No	--	--	R	--
XLOC_006721	DEG(M)	No	No	No	ETM32394.1	--	O	--
XLOC_006723	No	No	No	No	NP_001155402.1	--	--	--
XLOC_006724	No	No	No	No	--	--	--	--
XLOC_006726	DEG(M)	No	DEG(I+M)	DEG(M)	XP_012568568.1	--	--	--
XLOC_006727	No	No	No	No	EUC66505.1	--	--	--
XLOC_006729	No	No	No	No	--	--	--	--
XLOC_006730	No	No	No	No	--	--	--	--
XLOC_006736	No	No	No	No	--	--	--	--
XLOC_006737	DEG(M)	DEG(M)	No	No	--	--	--	--
XLOC_006740	No	No	No	No	--	--	--	--
XLOC_006742	No	No	No	No	XP_002529245.1	--	--	K13249
XLOC_006744	No	No	DEG(I+M)	No	--	--	--	K05909
XLOC_006746	No	No	No	No	--	--	--	--
XLOC_006753	No	No	No	No	--	--	--	--
XLOC_006755	No	No	No	No	--	--	--	--
XLOC_006762	No	No	No	No	--	--	--	--
XLOC_006763	No	No	DEG(I+M)	No	XP_004506687.1	--	--	K00512
XLOC_006767	DEG(M)	No	No	No	--	--	--	--
XLOC_006772	DEG(M)	No	No	No	EUC55847.1	--	--	--
XLOC_006776	No	No	No	No	--	--	--	--
XLOC_006777	No	No	No	No	--	--	--	--
XLOC_006782	No	No	No	No	KDN38071.1	--	--	--

Differentially expressed gene (DEG) IDs	*C. microphyllum* root and shoot-specific (>2 fold)	*C. microphyllum* root and shoot-specific (>10 fold)	ICC4958 and *C. microphyllum* root-specific (>2 fold)	*C. microphyllum* root-specific (>2 fold)	Functional Annotation			
					NCBI nr IDs	TFs	KOG	KEGG
XLOC_006786	No	No	No	No	CCO36017.1	--	J	K03236
XLOC_006787	No	No	No	No	EUC53664.1	--	--	--
XLOC_006792	DEG(M)	No	No	No	--	--	--	K17279
XLOC_006794	DEG(M)	No	No	No	--	--	J	--
XLOC_006795	No	No	No	No	--	--	--	--
XLOC_006796	DEG(M)	DEG(M)	DEG(I+M)	DEG(M)	--	--	--	--
XLOC_006797	No	No	No	No	--	--	--	--
XLOC_006798	DEG(M)	No	No	No	--	--	--	--
XLOC_006799	No	No	DEG(I+M)	No	XP_004489777.1	--	--	--
XLOC_006801	No	No	No	No	CCO30386.1	--	--	--
XLOC_006804	DEG(M)	No	No	No	NP_001155385.1	--	J	K02894
XLOC_006807	No	No	No	No	--	--	C	K00130
XLOC_006849	No	No	No	No	XP_001952817.1	--	--	--
XLOC_006862	DEG(M)	No	DEG(I+M)	DEG(M)	XP_009539724.1	--	--	--
XLOC_006863	DEG(M)	DEG(M)	DEG(I+M)	DEG(M)	XP_009539724.1	--	--	--
XLOC_006867	DEG(M)	No	DEG(I+M)	DEG(M)	EGC42647.1	--	--	--
XLOC_006870	DEG(M)	DEG(M)	DEG(I+M)	DEG(M)	--	--	--	--
XLOC_006873	DEG(M)	DEG(M)	DEG(I+M)	DEG(M)	--	--	--	--
XLOC_006875	DEG(M)	DEG(M)	DEG(I+M)	DEG(M)	--	--	--	--
XLOC_006876	DEG(M)	No	DEG(I+M)	DEG(M)	--	--	--	--
XLOC_006880	DEG(M)	No	No	No	--	--	--	--
XLOC_006882	DEG(M)	No	DEG(I+M)	DEG(M)	--	--	--	--
XLOC_006893	No	No	DEG(I+M)	No	EAW66125.1	--	X	--
XLOC_006894	No	No	No	No	--	--	--	--
XLOC_006896	No	No	No	No	--	--	--	--
XLOC_006898	DEG(M)	DEG(M)	DEG(I+M)	DEG(M)	--	--	--	--
XLOC_006899	DEG(M)	DEG(M)	DEG(I+M)	DEG(M)	CBY13234.1	--	--	--
XLOC_006907	DEG(M)	DEG(M)	No	No	--	--	--	--
XLOC_006908	DEG(M)	DEG(M)	No	No	WP_039860568.1	--	--	--
XLOC_006911	No	No	No	No	--	--	--	--
XLOC_006926	No	No	DEG(I+M)	No	XP_004504561.1	C3H	C	K11517
XLOC_006941	No	No	DEG(I+M)	No	--	--	Q	--
XLOC_007008	DEG(M)	DEG(M)	DEG(I+M)	DEG(M)	AAP46148.1	--	Z	--
XLOC_007009	DEG(M)	No	DEG(I+M)	DEG(M)	AFC88820.1	--	Z	--
XLOC_007010	DEG(M)	No	No	No	EJW72694.1	--	Z	--
XLOC_007012	DEG(M)	DEG(M)	DEG(I+M)	DEG(M)	ADX01224.1	--	Z	--
XLOC_007013	DEG(M)	DEG(M)	DEG(I+M)	DEG(M)	--	--	--	--
XLOC_007014	DEG(M)	DEG(M)	DEG(I+M)	DEG(M)	AAL30373.1	--	Z	--
XLOC_007015	DEG(M)	DEG(M)	DEG(I+M)	DEG(M)	ABF06543.1	--	Z	--
XLOC_007016	DEG(M)	No	No	No	AHF21757.1	--	Z	--

Differentially expressed gene (DEG) IDs	*C. microphyllum* root and shoot-specific (>2 fold)	*C. microphyllum* root and shoot-specific (>10 fold)	ICC4958 and *C. microphyllum* root-specific (>2 fold)	*C. microphyllum* root-specific (>2 fold)	Functional Annotation			
					NCBI nr IDs	TFs	KOG	KEGG
XLOC_007017	DEG(M)	DEG(M)	DEG(I+M)	DEG(M)	AAO19650.1	--	Z	--
XLOC_007019	DEG(M)	DEG(M)	DEG(I+M)	DEG(M)	ABO16472.1	Dof	Z	--
XLOC_007021	DEG(M)	DEG(M)	DEG(I+M)	DEG(M)	AFU72282.1	--	Z	--
XLOC_007022	DEG(M)	DEG(M)	DEG(I+M)	DEG(M)	XP_005315620.1	Dof	Z	--
XLOC_007025	DEG(M)	DEG(M)	No	No	XP_006625208.1	--	Z	--
XLOC_007054	No	No	No	No	KEP48379.1	--	--	K01535
XLOC_007055	DEG(M)	No	DEG(I+M)	DEG(M)	CCO31202.1	--	--	--
XLOC_007057	DEG(M)	No	No	No	CEL57318.1	--	--	--
XLOC_007059	DEG(M)	No	No	No	CEL57318.1	--	--	--
XLOC_007060	No	No	No	No	EUC53553.1	--	--	--
XLOC_007061	DEG(M)	No	No	No	KEP48379.1	--	P	--
XLOC_007085	No	No	No	No	--	--	--	--
XLOC_007167	DEG(M)	No	No	No	--	--	--	--
XLOC_007169	DEG(M)	DEG(M)	No	No	CBW14553.1	--	--	--
XLOC_007177	DEG(M)	DEG(M)	DEG(I+M)	DEG(M)	--	--	--	--
XLOC_007181	DEG(M)	DEG(M)	No	No	--	--	--	--
XLOC_007186	DEG(M)	No	No	No	--	--	--	--
XLOC_007187	DEG(M)	No	No	No	EGW65230.1	--	--	--
XLOC_007220	No	No	No	No	XP_003850793.1	--	--	--
XLOC_007222	DEG(M)	DEG(M)	DEG(I+M)	DEG(M)	--	--	--	--
XLOC_007224	DEG(M)	No	No	No	--	--	--	--
XLOC_007226	DEG(M)	No	DEG(I+M)	DEG(M)	--	--	--	--
XLOC_007227	No	No	DEG(I+M)	No	--	--	--	--
XLOC_007228	DEG(M)	DEG(M)	DEG(I+M)	DEG(M)	--	--	--	--
XLOC_007229	DEG(M)	No	DEG(I+M)	DEG(M)	--	--	--	--
XLOC_007235	DEG(M)	No	DEG(I+M)	DEG(M)	--	--	--	--
XLOC_007262	DEG(M)	DEG(M)	No	No	BAJ91591.1	--	J	--
XLOC_007267	DEG(M)	No	No	No	--	--	--	--
XLOC_007302	No	No	No	No	XP_004506354.1	--	--	--
XLOC_007314	DEG(M)	No	No	No	NP_001165437.1	--	J	K02993
XLOC_007352	No	No	No	No	XP_012571727.1	WRKY	--	--
XLOC_007416	DEG(M)	No	No	No	AAU84933.1	NAC	J	--
XLOC_007417	DEG(M)	No	No	No	--	--	--	--
XLOC_007418	DEG(M)	No	No	No	ADQ64541.1	NAC	J	--
XLOC_007428	No	No	No	No	--	--	--	--
XLOC_007473	DEG(M)	DEG(M)	DEG(I+M)	DEG(M)	--	--	--	--
XLOC_007475	DEG(M)	No	DEG(I+M)	DEG(M)	--	--	--	--
XLOC_007476	No	No	No	No	XP_003148073.1	--	J	--
XLOC_007484	DEG(M)	DEG(M)	DEG(I+M)	DEG(M)	--	--	--	--
XLOC_007488	DEG(M)	DEG(M)	DEG(I+M)	DEG(M)	--	--	--	--

Differentially expressed gene (DEG) IDs	*C. microphyllum* root and shoot-specific (>2 fold)	*C. microphyllum* root and shoot-specific (>10 fold)	ICC4958 and *C. microphyllum* root-specific (>2 fold)	*C. microphyllum* root-specific (>2 fold)	Functional Annotation			
					NCBI nr IDs	TFs	KOG	KEGG
XLOC_007489	No	No	No	No	XP_002903654.1	--	G	--
XLOC_007493	No	No	No	No	--	--	TZ	--
XLOC_007494	DEG(M)	No	No	No	--	--	TZ	--
XLOC_007502	No	No	No	No	AJQ30126.1	--	--	K00026
XLOC_007511	No	No	DEG(I+M)	No	--	--	G	K03841
XLOC_007512	No	No	No	No	--	--	--	--
XLOC_007513	No	No	No	No	XP_001949411.1	--	G	K00844
XLOC_007521	DEG(M)	No	No	No	KDN50738.1	--	J	--
XLOC_007525	No	No	No	No	--	--	--	--
XLOC_007527	No	No	No	No	KEP54223.1	--	--	--
XLOC_007536	No	No	No	No	XP_008025477.1	--	--	--
XLOC_007537	No	No	No	No	--	--	--	--
XLOC_007539	No	No	No	No	--	--	--	--
XLOC_007540	No	No	No	No	--	--	--	--
XLOC_007553	No	No	No	No	--	--	--	K17267
XLOC_007554	No	No	No	No	--	--	--	--
XLOC_007555	No	No	No	No	--	--	--	--
XLOC_007556	No	No	No	No	CCO32567.1	--	--	--
XLOC_007560	No	No	No	No	--	--	--	--
XLOC_007565	DEG(M)	No	No	No	KDN45381.1	--	--	--
XLOC_007567	No	No	No	No	KEP49966.1	--	--	--
XLOC_007568	DEG(M)	No	No	No	AAV90714.1	--	J	K02960
XLOC_007571	No	No	No	No	XP_003041661.1	--	--	--
XLOC_007575	No	No	No	No	--	--	S	K09595
XLOC_007576	DEG(M)	No	No	No	NP_001155777.1	--	J	--
XLOC_007578	No	No	No	No	--	--	--	--
XLOC_007582	No	No	No	No	XP_001936880.1	--	--	--
XLOC_007583	No	No	No	No	KDN50517.1	--	--	K09510
XLOC_007588	No	No	No	No	CCO32297.1	--	--	--
XLOC_007592	DEG(M)	No	No	No	--	--	K	K01527
XLOC_007593	No	No	No	No	--	--	--	--
XLOC_007594	No	No	No	No	KNG45477.1	--	--	--
XLOC_007598	DEG(M)	No	No	No	--	--	--	--
XLOC_007599	DEG(M)	No	No	No	--	--	--	--
XLOC_007606	DEG(M)	No	No	No	XP_003046795.1	--	--	--
XLOC_007609	DEG(M)	No	No	No	EUC53593.1	--	--	--
XLOC_007610	DEG(M)	No	No	No	EGU83851.1	--	O	--
XLOC_007611	No	No	No	No	XP_003053849.1	--	O	--
XLOC_007614	DEG(M)	DEG(M)	DEG(I+M)	DEG(M)	KDN37828.1	--	T	--
XLOC_007615	No	No	DEG(I+M)	No	XP_004488180.1	--	F	--

Differentially expressed gene (DEG) IDs	*C. microphyllum* root and shoot-specific (>2 fold)	*C. microphyllum* root and shoot-specific (>10 fold)	ICC4958 and *C. microphyllum* root-specific (>2 fold)	*C. microphyllum* root-specific (>2 fold)	Functional Annotation			
					NCBI nr IDs	TFs	KOG	KEGG
XLOC_007616	No	No	No	No	XP_004496018.1	--	F	--
XLOC_007621	DEG(M)	DEG(M)	DEG(I+M)	DEG(M)	--	--	--	--
XLOC_007627	No	No	DEG(I+M)	No	XP_004496669.1	--	--	--
XLOC_007635	No	No	No	No	KDN48107.1	--	E	--
XLOC_007636	No	No	No	No	KDN48107.1	--	E	--
XLOC_007637	No	No	No	No	CEL59381.1	--	E	K00549
XLOC_007639	DEG(M)	No	DEG(I+M)	DEG(M)	KEH27441.1	--	--	--
XLOC_007643	DEG(M)	No	No	No	--	--	--	--
XLOC_007644	No	No	No	No	XP_004503442.1	--	--	K17710
XLOC_007653	DEG(M)	DEG(M)	DEG(I+M)	DEG(M)	XP_008890292.1	--	J	K02910
XLOC_007660	No	No	No	No	--	--	--	--
XLOC_007662	No	No	No	No	--	--	--	--
XLOC_007664	No	No	No	No	XP_001938883.1	--	--	--
XLOC_007665	No	No	No	No	XP_001938883.1	--	--	--
XLOC_007675	DEG(M)	No	No	No	KEP55194.1	--	J	--
XLOC_007681	DEG(M)	DEG(M)	No	No	XP_004485960.1	--	--	--
XLOC_007706	DEG(M)	No	No	No	EUC67163.1	--	--	--
XLOC_007707	No	No	No	No	--	--	I	--
XLOC_007709	No	No	No	No	XP_004516319.1	--	I	K02291
XLOC_007711	DEG(M)	No	No	No	--	--	P	K14724
XLOC_007716	DEG(M)	DEG(M)	DEG(I+M)	DEG(M)	KJX98686.1	--	C	--
XLOC_007719	No	No	No	No	--	--	--	--
XLOC_007723	No	No	No	No	--	--	--	--
XLOC_007728	DEG(M)	No	No	No	XP_009530825.1	--	AJ	--
XLOC_007732	No	No	No	No	--	--	--	--
XLOC_007740	No	No	No	No	AEK51003.1	Trihelix	G	--
XLOC_007741	DEG(M)	No	No	No	--	--	--	--
XLOC_007743	DEG(M)	No	No	No	--	--	--	--
XLOC_007745	No	No	No	No	--	--	--	K11188
XLOC_007746	No	No	No	No	CCO28304.1	--	--	--
XLOC_007749	No	No	No	No	--	--	--	--
XLOC_007751	No	No	No	No	--	--	--	--
XLOC_007752	No	No	No	No	--	--	--	--
XLOC_007753	No	No	No	No	--	--	--	--
XLOC_007755	DEG(M)	No	No	No	ABX75439.1	B3	J	K02921
XLOC_007762	No	No	No	No	--	--	--	--
XLOC_007763	No	No	No	No	--	--	--	--
XLOC_007773	No	No	No	No	--	--	J	--
XLOC_007779	No	No	No	No	--	--	--	--
XLOC_007780	No	No	No	No	EXM03436.1	--	--	--

Differentially expressed gene (DEG) IDs	*C. microphyllum* root and shoot-specific (>2 fold)	*C. microphyllum* root and shoot-specific (>10 fold)	ICC4958 and *C. microphyllum* root-specific (>2 fold)	*C. microphyllum* root-specific (>2 fold)	Functional Annotation			
					NCBI nr IDs	TFs	KOG	KEGG
XLOC_007782	No	No	No	No	--	--	--	K08869
XLOC_007783	No	No	No	No	EUC66096.1	--	--	--
XLOC_007786	No	No	No	No	EXL80486.1	--	--	--
XLOC_007796	DEG(M)	No	No	No	--	--	--	--
XLOC_007798	No	No	No	No	XP_001937563.1	--	C	K17761
XLOC_007799	DEG(M)	No	No	No	--	--	--	--
XLOC_007801	No	No	No	No	--	Trihelix	G	--
XLOC_007805	No	No	No	No	EFX69073.1	--	O	K02729
XLOC_007806	No	No	No	No	DAA34760.1	--	O	K10573
XLOC_007809	No	No	No	No	--	--	--	--
XLOC_007810	No	No	No	No	--	--	--	--
XLOC_007818	No	No	No	No	--	--	--	--
XLOC_007823	No	No	No	No	--	--	--	--
XLOC_007824	DEG(M)	No	No	No	--	--	--	--
XLOC_007827	No	No	No	No	CCO27984.1	--	AJ	--
XLOC_007828	No	No	No	No	--	--	--	--
XLOC_007833	No	No	No	No	--	--	--	--
XLOC_007834	No	No	No	No	EUC62396.1	--	--	--
XLOC_007838	No	No	No	No	--	--	--	--
XLOC_007845	No	No	No	No	CCO32777.1	--	--	--
XLOC_007847	No	No	No	No	--	--	--	--
XLOC_007849	No	No	No	No	--	--	--	--
XLOC_007858	No	No	No	No	--	--	--	--
XLOC_007862	DEG(M)	No	No	No	--	--	--	--
XLOC_007863	No	No	No	No	--	--	--	--
XLOC_007866	No	No	DEG(I+M)	No	XP_004513031.1	--	--	--
XLOC_007867	DEG(M)	No	No	No	CEL60785.1	--	J	K02998
XLOC_007869	No	No	No	No	--	--	--	--
XLOC_007870	No	No	No	No	XP_002533145.1	--	G	--
XLOC_007871	No	No	No	No	--	--	--	--
XLOC_007875	No	No	No	No	--	--	YU	K18460
XLOC_007876	No	No	No	No	CEL55395.1	--	--	--
XLOC_007879	No	No	No	No	XP_004510774.1	--	QI	K15398
XLOC_007883	No	No	No	No	--	--	J	--
XLOC_007887	No	No	No	No	--	--	--	--
XLOC_007888	No	No	No	No	XP_006368511.1	WRKY	KT	--
XLOC_007891	DEG(M)	No	No	No	AAM51207.1	YABBY	OR	--
XLOC_007892	DEG(M)	No	No	No	XP_008383094.1	YABBY	OR	--
XLOC_007898	No	No	No	No	CCO31145.1	--	--	--
XLOC_007899	No	No	No	No	XP_007773307.1	--	J	K02951

Differentially expressed gene (DEG) IDs	*C. microphyllum* root and shoot-specific (>2 fold)	*C. microphyllum* root and shoot-specific (>10 fold)	ICC4958 and *C. microphyllum* root-specific (>2 fold)	*C. microphyllum* root-specific (>2 fold)	Functional Annotation			
					NCBI nr IDs	TFs	KOG	KEGG
XLOC_007900	DEG(M)	No	No	No	--	--	--	--
XLOC_007901	DEG(M)	DEG(M)	No	No	XP_003041072.1	--	R	--
XLOC_007905	No	No	No	No	--	--	--	--
XLOC_007913	No	No	No	No	--	--	--	--
XLOC_007917	DEG(M)	DEG(M)	DEG(I+M)	DEG(M)	--	--	--	--
XLOC_007918	No	No	No	No	--	--	--	--
XLOC_007919	DEG(M)	No	No	No	--	--	--	--
XLOC_007920	DEG(M)	No	No	No	--	--	--	--
XLOC_007921	DEG(M)	No	No	No	CCO37793.1	--	--	--
XLOC_007922	No	No	No	No	--	--	E	--
XLOC_007923	No	No	No	No	--	--	--	--
XLOC_007924	No	No	No	No	XP_001947747.2	--	W	--
XLOC_007927	No	No	No	No	XP_001943507.1	--	--	--
XLOC_007930	No	No	No	No	XP_003054238.1	--	--	K11294
XLOC_007935	No	No	No	No	--	--	--	--
XLOC_007936	No	No	No	No	--	--	--	--
XLOC_007942	DEG(M)	DEG(M)	No	No	--	--	--	--
XLOC_007947	No	No	No	No	--	--	--	--
XLOC_007949	No	No	No	No	KEP48765.1	--	R	--
XLOC_007950	No	No	No	No	--	--	--	--
XLOC_007951	DEG(M)	No	No	No	--	--	G	K09872
XLOC_007960	DEG(M)	No	No	No	--	--	--	--
XLOC_007962	No	No	No	No	--	--	--	--
XLOC_007968	No	No	No	No	XP_004506797.1	--	--	K08059
XLOC_007969	No	No	No	No	--	--	--	K12486
XLOC_007971	No	No	No	No	XP_002591311.1	--	U	K13280
XLOC_007973	No	No	No	No	XP_004488231.1	--	F	--
XLOC_007974	No	No	No	No	EMT66964.1	--	--	--
XLOC_007976	No	No	No	No	KDN50965.1	--	--	--
XLOC_007977	No	No	No	No	--	--	--	K05929
XLOC_007982	DEG(M)	No	No	No	AAZ20449.1	--	--	--
XLOC_007983	No	No	No	No	XP_003053639.1	--	--	--
XLOC_007984	No	No	No	No	NP_001233049.1	--	--	--
XLOC_007986	No	No	No	No	--	--	J	--
XLOC_007987	No	No	DEG(I+M)	No	XP_004493979.1	--	--	K14488
XLOC_007989	No	No	No	No	KEP52816.1	--	--	--
XLOC_007992	No	No	No	No	--	--	V	--
XLOC_007993	No	No	DEG(I+M)	No	XP_004506514.1	--	--	--
XLOC_007994	DEG(M)	No	No	No	--	--	--	--
XLOC_008000	No	No	No	No	--	--	--	K05768

Differentially expressed gene (DEG) IDs	*C. microphyllum* root and shoot-specific (>2 fold)	*C. microphyllum* root and shoot-specific (>10 fold)	ICC4958 and *C. microphyllum* root-specific (>2 fold)	*C. microphyllum* root-specific (>2 fold)	Functional Annotation			
					NCBI nr IDs	TFs	KOG	KEGG
XLOC_008005	DEG(M)	No	No	No	--	--	--	--
XLOC_008011	No	No	No	No	--	--	--	K12896
XLOC_008014	DEG(M)	No	No	No	KEP55034.1	--	--	--
XLOC_008015	No	No	No	No	--	--	--	--
XLOC_008021	DEG(M)	No	No	No	--	--	--	--
XLOC_008022	DEG(M)	No	No	No	--	--	--	--
XLOC_008026	No	No	No	No	--	--	--	--
XLOC_008027	No	No	No	No	XP_001654772.1	--	P	K04565
XLOC_008032	No	No	No	No	EUC58906.1	--	--	K09486
XLOC_008038	No	No	No	No	XP_008905106.1	--	--	--
XLOC_008039	No	No	No	No	XP_001649606.1	--	--	--
XLOC_008040	No	No	No	No	NP_001119644.1	--	T	K04382
XLOC_008041	DEG(M)	No	No	No	CCO30076.1	--	--	--
XLOC_008042	No	No	No	No	--	--	--	--
XLOC_008044	No	No	No	No	XP_001664066.1	--	--	--
XLOC_008045	DEG(M)	No	DEG(I+M)	DEG(M)	--	--	--	--
XLOC_008053	No	No	No	No	--	--	--	--
XLOC_008056	DEG(M)	No	No	No	--	--	--	--
XLOC_008059	No	No	No	No	XP_004337289.1	NAC	J	--
XLOC_008067	No	No	No	No	XP_003048555.1	--	--	--
XLOC_008070	No	No	No	No	--	--	--	--
XLOC_008071	DEG(M)	No	No	No	CCO28907.1	--	--	--
XLOC_008072	No	No	No	No	CEL52595.1	--	--	--
XLOC_008074	No	No	No	No	--	--	--	--
XLOC_008078	No	No	No	No	--	--	--	--
XLOC_008079	No	No	No	No	--	--	--	--
XLOC_008080	No	No	No	No	ETL99538.1	--	--	--
XLOC_008083	No	No	No	No	--	--	--	--
XLOC_008084	No	No	No	No	--	--	--	--
XLOC_008087	No	No	DEG(I+M)	No	XP_012575003.1	--	--	--
XLOC_008089	No	No	No	No	--	--	--	--
XLOC_008091	DEG(M)	No	No	No	--	--	--	--
XLOC_008092	No	No	No	No	XP_001650865.1	--	F	K15014
XLOC_008093	No	No	No	No	XP_001653135.1	--	--	K15026
XLOC_008098	No	No	No	No	--	--	--	--
XLOC_008099	No	No	No	No	XP_002407055.1	--	Z	--
XLOC_008101	No	No	No	No	EUC54920.1	--	--	--
XLOC_008102	DEG(M)	No	No	No	ABK59969.1	--	--	--
XLOC_008103	No	No	No	No	XP_004352877.1	--	J	K02974
XLOC_008106	DEG(M)	DEG(M)	DEG(I+M)	DEG(M)	--	--	--	--

Differentially expressed gene (DEG) IDs	*C. microphyllum* root and shoot-specific (>2 fold)	*C. microphyllum* root and shoot-specific (>10 fold)	ICC4958 and *C. microphyllum* root-specific (>2 fold)	*C. microphyllum* root-specific (>2 fold)	Functional Annotation			
					NCBI nr IDs	TFs	KOG	KEGG
XLOC_008107	DEG(M)	No	No	No	--	--	--	--
XLOC_008108	No	No	No	No	--	--	--	K11593
XLOC_008110	No	No	No	No	--	--	--	--
XLOC_008113	No	No	No	No	--	--	--	--
XLOC_008115	No	No	No	No	--	--	--	--
XLOC_008120	DEG(M)	No	No	No	CCO30626.1	--	--	--
XLOC_008122	DEG(M)	No	No	No	--	--	--	--
XLOC_008123	No	No	No	No	CCO37403.1	--	--	--
XLOC_008124	DEG(M)	No	No	No	--	--	J	K02912
XLOC_008125	No	No	No	No	--	--	--	--
XLOC_008131	DEG(M)	No	No	No	KDN49398.1	--	--	--
XLOC_008132	DEG(M)	DEG(M)	DEG(I+M)	DEG(M)	XP_008909337.1	--	J	K02938
XLOC_008137	No	No	No	No	--	--	--	--
XLOC_008154	No	No	No	No	--	--	--	--
XLOC_008157	DEG(M)	No	No	No	KDQ10335.1	--	--	--
XLOC_008159	DEG(M)	No	No	No	NP_001155755.1	--	J	K02981
XLOC_008160	DEG(M)	DEG(M)	No	No	XP_003051263.1	--	J	--
XLOC_008163	DEG(M)	No	No	No	XP_003297921.1	--	--	--
XLOC_008166	DEG(M)	No	No	No	--	--	--	--
XLOC_008167	DEG(M)	No	No	No	--	--	--	--
XLOC_008168	DEG(M)	No	No	No	EUC61730.1	--	--	K06875
XLOC_008170	No	No	DEG(I+M)	No	XP_004491685.1	--	--	--
XLOC_008172	No	No	No	No	--	FAR1	G	K00036
XLOC_008176	DEG(M)	No	No	No	--	--	--	--
XLOC_008177	DEG(M)	No	No	No	--	--	--	--
XLOC_008180	DEG(M)	DEG(M)	No	No	--	--	--	--
XLOC_008186	DEG(M)	No	No	No	--	--	--	--
XLOC_008198	DEG(M)	No	No	No	--	--	--	--
XLOC_008204	DEG(M)	DEG(M)	DEG(I+M)	DEG(M)	XP_008893022.1	--	J	K03263
XLOC_008205	DEG(M)	DEG(M)	No	No	--	--	--	--
XLOC_008207	DEG(M)	DEG(M)	No	No	--	--	--	--
XLOC_008208	No	No	No	No	XP_008912113.1	--	--	--
XLOC_008209	No	No	No	No	ETI37144.1	--	--	--
XLOC_008211	No	No	No	No	--	--	--	--
XLOC_008212	No	No	No	No	--	--	--	--
XLOC_008213	No	No	No	No	--	--	--	--
XLOC_008214	No	No	DEG(I+M)	No	XP_004487376.2	--	--	--
XLOC_008216	No	No	No	No	EYC23926.1	--	A	--
XLOC_008219	No	No	No	No	--	--	--	--
XLOC_008220	No	No	No	No	--	--	--	--

Differentially expressed gene (DEG) IDs	*C. microphyllum* root and shoot-specific (>2 fold)	*C. microphyllum* root and shoot-specific (>10 fold)	ICC4958 and *C. microphyllum* root-specific (>2 fold)	*C. microphyllum* root-specific (>2 fold)	Functional Annotation			
					NCBI nr IDs	TFs	KOG	KEGG
XLOC_008224	No	No	No	No	--	--	--	--
XLOC_008225	No	No	No	No	ELU36439.1	--	--	--
XLOC_008226	No	No	No	No	XP_002902696.1	--	O	K03061
XLOC_008227	No	No	No	No	--	--	--	--
XLOC_008231	DEG(M)	DEG(M)	DEG(I+M)	DEG(M)	--	--	--	K02923
XLOC_008232	No	No	No	No	BAK07442.1	--	O	K09489
XLOC_008233	No	No	No	No	--	--	--	--
XLOC_008234	No	No	No	No	--	--	J	--
XLOC_008236	No	No	No	No	--	--	--	--
XLOC_008237	DEG(M)	No	No	No	--	--	--	--
XLOC_008238	No	No	No	No	--	--	--	--
XLOC_008256	DEG(M)	DEG(M)	No	No	--	--	--	--
XLOC_008264	DEG(M)	DEG(M)	DEG(I+M)	DEG(M)	--	--	--	--
XLOC_008265	No	No	No	No	--	--	--	--
XLOC_008274	DEG(M)	DEG(M)	DEG(I+M)	DEG(M)	CEL52640.1	--	--	--
XLOC_008275	DEG(M)	DEG(M)	DEG(I+M)	DEG(M)	CCO31118.1	--	--	--
XLOC_008276	No	No	No	No	CCO30220.1	--	--	--
XLOC_008277	DEG(M)	No	No	No	CCO31118.1	--	--	--
XLOC_008278	DEG(M)	DEG(M)	DEG(I+M)	DEG(M)	EUC62414.1	--	--	--
XLOC_008279	DEG(M)	No	No	No	EUC62414.1	--	--	--
XLOC_008280	DEG(M)	No	No	No	--	--	--	--
XLOC_008281	DEG(M)	No	No	No	--	--	--	--
XLOC_008306	No	No	DEG(I+M)	No	--	GRAS	P	--
XLOC_008307	No	No	DEG(I+M)	No	--	GRAS	P	--
XLOC_008312	No	No	DEG(I+M)	No	XP_004487948.1	GRAS	P	--
XLOC_008357	No	No	No	No	--	--	T	--
XLOC_008363	No	No	No	No	XP_004493833.1	--	T	--
XLOC_008369	No	No	No	No	--	--	--	--
XLOC_008371	No	No	No	No	--	--	--	--
XLOC_008390	No	No	No	No	XP_004497992.1	--	X	K02902
XLOC_008430	DEG(M)	No	No	No	CCO34014.1	--	--	--
XLOC_008431	DEG(M)	No	No	No	CCO34014.1	--	--	--
XLOC_008433	DEG(M)	No	No	No	XP_009529503.1	--	O	--
XLOC_008434	DEG(M)	No	DEG(I+M)	DEG(M)	CCO34014.1	--	--	--
XLOC_008435	No	No	No	No	CAA53368.1	--	O	--
XLOC_008436	No	No	No	No	XP_008901435.1	--	O	K09490
XLOC_008438	DEG(M)	DEG(M)	No	No	XP_003053763.1	--	O	--
XLOC_008462	No	No	No	No	--	--	G	K01662
XLOC_008464	No	No	No	No	--	--	G	--
XLOC_008465	No	No	No	No	--	--	G	--

Differentially expressed gene (DEG) IDs	*C. microphyllum* root and shoot-specific (>2 fold)	*C. microphyllum* root and shoot-specific (>10 fold)	ICC4958 and *C. microphyllum* root-specific (>2 fold)	*C. microphyllum* root-specific (>2 fold)	Functional Annotation			
					NCBI nr IDs	TFs	KOG	KEGG
XLOC_008467	DEG(M)	No	No	No	ACB70375.1	--	J	--
XLOC_008468	DEG(M)	No	No	No	ACB70375.1	--	J	--
XLOC_008470	No	No	No	No	--	--	--	--
XLOC_008471	DEG(M)	No	DEG(I+M)	DEG(M)	XP_003873869.1	--	J	--
XLOC_008473	DEG(M)	DEG(M)	DEG(I+M)	DEG(M)	CCO27325.1	--	J	--
XLOC_008474	DEG(M)	No	No	No	CCO27325.1	--	J	--
XLOC_008475	DEG(M)	DEG(M)	DEG(I+M)	DEG(M)	CEL58096.1	--	J	--
XLOC_008476	DEG(M)	No	No	No	CEL58096.1	--	J	--
XLOC_008477	DEG(M)	No	No	No	--	--	--	--
XLOC_008481	DEG(M)	DEG(M)	No	No	--	--	--	--
XLOC_008544	DEG(M)	No	DEG(I+M)	DEG(M)	--	--	--	--
XLOC_008545	DEG(M)	DEG(M)	DEG(I+M)	DEG(M)	--	--	--	--
XLOC_008547	No	No	DEG(I+M)	No	XP_004515409.1	--	--	--
XLOC_008551	No	No	No	No	XP_004515413.1	--	--	--
XLOC_008590	DEG(M)	DEG(M)	DEG(I+M)	DEG(M)	XP_003045094.1	--	EJ	--
XLOC_008625	DEG(M)	DEG(M)	No	No	ADN33682.1	--	--	--
XLOC_008627	DEG(M)	DEG(M)	No	No	--	--	--	--
XLOC_008636	No	No	No	No	--	--	--	--
XLOC_008640	DEG(M)	No	No	No	--	--	--	--
XLOC_008643	No	No	DEG(I+M)	No	--	--	--	K13457
XLOC_008645	DEG(M)	No	No	No	KEP48025.1	--	--	--
XLOC_008646	DEG(M)	DEG(M)	DEG(I+M)	DEG(M)	CCO36010.1	--	--	--
XLOC_008654	No	No	No	No	--	--	--	--
XLOC_008658	No	No	No	No	--	--	--	--
XLOC_008665	DEG(M)	No	No	No	KDN37710.1	--	--	--
XLOC_008666	No	No	No	No	KDN37710.1	--	--	--
XLOC_008668	DEG(M)	No	No	No	--	--	--	--
XLOC_008669	DEG(M)	No	No	No	--	--	--	--
XLOC_008670	DEG(M)	DEG(M)	DEG(I+M)	DEG(M)	--	--	--	--
XLOC_008671	DEG(M)	No	No	No	EUC63853.1	--	--	--
XLOC_008674	DEG(M)	No	DEG(I+M)	DEG(M)	--	--	--	--
XLOC_008696	No	No	DEG(I+M)	No	--	--	V	--
XLOC_008697	DEG(M)	No	DEG(I+M)	DEG(M)	XP_004489241.1	--	V	--
XLOC_008703	DEG(M)	No	No	No	--	--	--	--
XLOC_008704	DEG(M)	No	No	No	--	--	--	--
XLOC_008707	DEG(M)	No	No	No	EUC62406.1	--	--	--
XLOC_008719	DEG(M)	No	No	No	--	--	--	--
XLOC_008720	No	No	No	No	--	--	--	--
XLOC_008722	DEG(M)	No	No	No	EUC66717.1	--	J	K02866
XLOC_008723	No	No	No	No	EUC66717.1	--	J	--

Differentially expressed gene (DEG) IDs	*C. microphyllum* root and shoot-specific (>2 fold)	*C. microphyllum* root and shoot-specific (>10 fold)	ICC4958 and *C. microphyllum* root-specific (>2 fold)	*C. microphyllum* root-specific (>2 fold)	Functional Annotation			
					NCBI nr IDs	TFs	KOG	KEGG
XLOC_008724	DEG(M)	No	No	No	CCO26970.1	--	J	K02866
XLOC_008727	DEG(M)	DEG(M)	DEG(I+M)	DEG(M)	AEK26871.1	--	J	--
XLOC_008731	No	No	No	No	--	--	--	--
XLOC_008734	No	No	DEG(I+M)	No	--	--	--	--
XLOC_008748	No	No	DEG(I+M)	No	--	--	--	--
XLOC_008751	No	No	No	No	--	--	--	--
XLOC_008764	No	No	No	No	KEP54688.1	--	--	--
XLOC_008765	No	No	No	No	KEP54688.1	--	--	--
XLOC_008766	No	No	No	No	CCO32708.1	--	--	--
XLOC_008767	DEG(M)	No	No	No	KEP54688.1	--	--	--
XLOC_008775	No	No	DEG(I+M)	No	--	--	--	K01534
XLOC_008786	No	No	No	No	--	--	--	K03255
XLOC_008791	DEG(M)	No	No	No	--	--	--	--
XLOC_008792	No	No	No	No	CEL61957.1	--	--	--
XLOC_008793	No	No	No	No	CEL61957.1	--	--	--
XLOC_008794	No	No	No	No	KEP49981.1	--	--	--
XLOC_008854	No	No	DEG(I+M)	No	--	--	--	K08237
XLOC_008855	DEG(M)	DEG(M)	DEG(I+M)	DEG(M)	XP_004515369.1	--	--	K08237
XLOC_008884	DEG(M)	No	No	No	--	--	--	--
XLOC_008904	No	No	No	No	--	--	--	--
XLOC_008918	No	No	No	No	XP_004507092.1	MYB	K	K09422
XLOC_008944	DEG(M)	No	No	No	--	--	--	--
XLOC_008952	No	No	No	No	XP_003051723.1	--	O	--
XLOC_008954	No	No	No	No	--	--	--	--
XLOC_008955	No	No	No	No	--	--	--	--
XLOC_008957	No	No	No	No	XP_002513516.1	--	--	K00858
XLOC_008960	No	No	No	No	CCO31337.1	--	--	K10592
XLOC_008963	DEG(M)	No	No	No	--	--	--	--
XLOC_008965	DEG(M)	DEG(M)	DEG(I+M)	DEG(M)	XP_002902090.1	B3	J	--
XLOC_008966	No	No	No	No	XP_009522872.1	--	A	K11129
XLOC_008968	No	No	No	No	XP_003054323.1	--	--	--
XLOC_008970	No	No	No	No	--	--	--	--
XLOC_008971	No	No	No	No	XP_008893231.1	--	--	--
XLOC_008973	No	No	No	No	--	--	--	--
XLOC_008974	No	No	No	No	--	--	--	--
XLOC_008975	No	No	No	No	XP_003243789.1	--	--	--
XLOC_008976	No	No	No	No	--	--	DK	--
XLOC_008978	No	No	No	No	--	--	--	--
XLOC_008983	No	No	No	No	--	--	--	--
XLOC_008984	No	No	No	No	CCO27715.1	--	--	--

Differentially expressed gene (DEG) IDs	*C. microphyllum* root and shoot-specific (>2 fold)	*C. microphyllum* root and shoot-specific (>10 fold)	ICC4958 and *C. microphyllum* root-specific (>2 fold)	*C. microphyllum* root-specific (>2 fold)	Functional Annotation			
					NCBI nr IDs	TFs	KOG	KEGG
XLOC_008991	No	No	No	No	--	--	--	--
XLOC_008993	No	No	No	No	XP_002313270.2	--	F	K01464
XLOC_008995	No	No	No	No	EMT65329.1	--	--	--
XLOC_008996	No	No	No	No	XP_008913489.1	--	--	--
XLOC_008999	No	No	No	No	--	--	--	--
XLOC_009000	No	No	No	No	EGY28406.1	--	--	--
XLOC_009001	No	No	No	No	AHZ64888.1	--	--	--
XLOC_009003	No	No	No	No	CDY47176.1	--	K	K11654
XLOC_009007	No	No	No	No	ETI49777.1	--	--	--
XLOC_009010	No	No	No	No	XP_001949124.1	--	--	K12896
XLOC_009013	DEG(M)	No	DEG(I+M)	DEG(M)	CKC05796.1	--	--	--
XLOC_009016	DEG(M)	No	No	No	--	--	--	--
XLOC_009017	DEG(M)	No	No	No	--	--	--	--
XLOC_009021	No	No	No	No	--	--	--	--
XLOC_009022	DEG(M)	No	No	No	CEL60291.1	--	--	--
XLOC_009026	No	No	No	No	--	--	--	--
XLOC_009034	No	No	No	No	XP_003043912.1	--	--	--
XLOC_009035	DEG(M)	No	No	No	--	--	--	--
XLOC_009037	DEG(M)	No	No	No	XP_003718505.1	--	J	--
XLOC_009038	No	No	No	No	XP_008909815.1	--	EI	--
XLOC_009042	No	No	No	No	--	--	--	--
XLOC_009044	No	No	No	No	KIO29346.1	--	--	--
XLOC_009046	DEG(M)	DEG(M)	No	No	EMT64259.1	--	--	--
XLOC_009048	No	No	No	No	KEP46091.1	--	--	--
XLOC_009051	No	No	No	No	ETM54623.1	--	--	--
XLOC_009055	No	No	No	No	--	--	--	--
XLOC_009056	No	No	No	No	ETI52386.1	--	--	--
XLOC_009059	No	No	No	No	--	--	--	--
XLOC_009062	No	No	No	No	--	--	R	--
XLOC_009064	No	No	No	No	--	--	--	--
XLOC_009068	No	No	No	No	--	--	--	--
XLOC_009070	DEG(M)	No	No	No	XP_008903519.1	--	--	--
XLOC_009073	No	No	No	No	KDN49015.1	--	--	--
XLOC_009083	No	No	No	No	CCO33550.1	--	--	--
XLOC_009084	No	No	No	No	XP_001950452.1	--	--	--
XLOC_009085	No	No	No	No	XP_001947745.1	--	--	--
XLOC_009088	No	No	No	No	--	--	--	--
XLOC_009089	No	No	No	No	--	--	--	--
XLOC_009093	No	No	No	No	--	--	--	--
XLOC_009095	No	No	No	No	--	--	--	--

Differentially expressed gene (DEG) IDs	*C. microphyllum* root and shoot-specific (>2 fold)	*C. microphyllum* root and shoot-specific (>10 fold)	ICC4958 and *C. microphyllum* root-specific (>2 fold)	*C. microphyllum* root-specific (>2 fold)	Functional Annotation			
					NCBI nr IDs	TFs	KOG	KEGG
XLOC_009097	DEG(M)	No	DEG(I+M)	DEG(M)	--	--	--	--
XLOC_009099	No	No	No	No	--	--	--	--
XLOC_009105	No	No	No	No	--	--	--	--
XLOC_009109	DEG(M)	No	No	No	--	--	--	--
XLOC_009112	No	No	No	No	--	--	--	--
XLOC_009114	No	No	No	No	XP_001951545.1	--	--	--
XLOC_009116	No	No	No	No	--	--	--	--
XLOC_009119	No	No	No	No	--	--	--	K03671
XLOC_009120	DEG(M)	No	No	No	EUC62627.1	--	--	--
XLOC_009122	No	No	No	No	--	--	--	--
XLOC_009123	DEG(M)	No	No	No	--	--	--	--
XLOC_009128	No	No	No	No	--	--	--	--
XLOC_009129	No	No	No	No	AAT92161.1	--	Z	--
XLOC_009133	No	No	No	No	--	--	--	--
XLOC_009134	DEG(M)	No	No	No	XP_008910035.1	--	--	K02727
XLOC_009135	No	No	No	No	--	--	--	--
XLOC_009136	DEG(M)	No	No	No	EXM03749.1	--	--	--
XLOC_009139	No	No	No	No	ETM31774.1	--	--	--
XLOC_009140	DEG(M)	DEG(M)	No	No	XP_008182369.1	--	J	--
XLOC_009141	DEG(M)	DEG(M)	No	No	BAI52945.1	--	J	--
XLOC_009143	DEG(M)	No	No	No	--	--	--	--
XLOC_009144	No	No	No	No	--	--	--	--
XLOC_009146	No	No	No	No	ELU42849.1	--	--	--
XLOC_009150	No	No	No	No	--	--	--	--
XLOC_009153	No	No	No	No	--	--	J	--
XLOC_009155	DEG(M)	No	No	No	--	--	--	--
XLOC_009156	DEG(M)	No	No	No	--	--	--	--
XLOC_009157	DEG(M)	No	No	No	--	--	--	--
XLOC_009158	DEG(M)	No	No	No	XP_001952275.1	--	--	--
XLOC_009161	DEG(M)	No	No	No	EUC59635.1	--	--	--
XLOC_009162	No	No	No	No	KDN39775.1	--	--	--
XLOC_009164	DEG(M)	No	No	No	--	--	--	--
XLOC_009170	DEG(M)	No	No	No	--	--	--	--
XLOC_009172	No	No	No	No	--	--	--	--
XLOC_009177	No	No	No	No	KDN44966.1	--	--	--
XLOC_009181	No	No	No	No	EGU83316.1	--	--	--
XLOC_009184	No	No	No	No	YP_009027276.1	--	C	K02108
XLOC_009188	DEG(M)	No	No	No	CCO36403.1	--	--	--
XLOC_009190	No	No	No	No	--	--	--	--
XLOC_009191	No	No	No	No	ETI34964.1	--	--	--

Differentially expressed gene (DEG) IDs	*C. microphyllum* root and shoot-specific (>2 fold)	*C. microphyllum* root and shoot-specific (>10 fold)	ICC4958 and *C. microphyllum* root-specific (>2 fold)	*C. microphyllum* root-specific (>2 fold)	Functional Annotation			
					NCBI nr IDs	TFs	KOG	KEGG
XLOC_009192	No	No	No	No	--	--	--	--
XLOC_009197	No	No	No	No	--	--	O	K09498
XLOC_009199	No	No	No	No	CEL51913.1	--	--	--
XLOC_009203	DEG(M)	No	No	No	CEL55574.1	--	--	--
XLOC_009204	No	No	No	No	--	--	--	K13993
XLOC_009210	DEG(M)	No	DEG(I+M)	DEG(M)	XP_002906426.1	--	J	--
XLOC_009212	No	No	DEG(I+M)	No	XP_004487411.1	--	--	--
XLOC_009222	No	No	No	No	XP_004489106.1	--	R	K15382
XLOC_009223	No	No	No	No	--	--	--	--
XLOC_009225	No	No	No	No	--	--	--	--
XLOC_009228	No	No	No	No	--	--	--	K15104
XLOC_009232	No	No	No	No	ETL44384.1	--	EG	K00140
XLOC_009234	No	No	No	No	--	--	--	--
XLOC_009235	No	No	No	No	--	--	--	--
XLOC_009237	DEG(M)	No	No	No	KEP54382.1	--	--	--
XLOC_009247	No	No	No	No	XP_001653970.1	--	--	--
XLOC_009248	No	No	No	No	--	--	--	--
XLOC_009251	No	No	No	No	EMT72510.1	--	--	--
XLOC_009252	DEG(M)	No	No	No	ETI41773.1	--	--	--
XLOC_009254	No	No	No	No	--	--	T	--
XLOC_009256	No	No	No	No	--	--	--	--
XLOC_009259	No	No	No	No	KDN36032.1	--	--	--
XLOC_009262	No	No	No	No	--	--	--	--
XLOC_009263	No	No	No	No	XP_008896359.1	--	I	--
XLOC_009264	No	No	No	No	XP_007014656.1	--	--	--
XLOC_009270	No	No	No	No	AIF75098.1	--	--	--
XLOC_009276	DEG(M)	No	No	No	XP_003047870.1	--	--	--
XLOC_009281	No	No	No	No	--	--	--	--
XLOC_009284	No	No	No	No	--	--	--	--
XLOC_009286	No	No	No	No	EUC66703.1	--	--	--
XLOC_009289	DEG(M)	No	No	No	--	--	--	--
XLOC_009291	DEG(M)	DEG(M)	DEG(I+M)	DEG(M)	XP_008891573.1	--	J	K02883
XLOC_009292	No	No	No	No	CCO27684.1	--	--	--
XLOC_009296	No	No	No	No	--	--	--	--
XLOC_009298	No	No	No	No	--	--	--	--
XLOC_009299	No	No	No	No	--	--	--	--
XLOC_009303	DEG(M)	No	No	No	--	--	--	--
XLOC_009304	No	No	No	No	--	--	--	--
XLOC_009305	No	No	No	No	--	--	--	--
XLOC_009306	No	No	No	No	CEL62975.1	--	CE	--

Differentially expressed gene (DEG) IDs	*C. microphyllum* root and shoot-specific (>2 fold)	*C. microphyllum* root and shoot-specific (>10 fold)	ICC4958 and *C. microphyllum* root-specific (>2 fold)	*C. microphyllum* root-specific (>2 fold)	Functional Annotation			
					NCBI nr IDs	TFs	KOG	KEGG
XLOC_009307	No	No	No	No	CEL61019.1	--	--	--
XLOC_009311	No	No	No	No	--	--	--	--
XLOC_009312	DEG(M)	No	No	No	--	--	--	--
XLOC_009313	No	No	No	No	XP_001930830.1	--	--	--
XLOC_009314	No	No	No	No	--	--	--	--
XLOC_009318	No	No	No	No	--	--	--	--
XLOC_009321	No	No	No	No	--	--	--	--
XLOC_009325	DEG(M)	No	No	No	--	--	--	--
XLOC_009327	No	No	No	No	XP_008890293.1	--	--	--
XLOC_009328	No	No	No	No	CCO28058.1	--	--	K03113
XLOC_009329	No	No	No	No	XP_012570815.1	--	R	--
XLOC_009332	No	No	No	No	XP_001650743.1	--	--	--
XLOC_009334	No	No	No	No	XP_001944099.1	--	--	K01640
XLOC_009335	DEG(M)	No	No	No	--	--	--	--
XLOC_009336	DEG(M)	No	No	No	EUC57125.1	--	--	K13448
XLOC_009337	DEG(M)	No	DEG(I+M)	DEG(M)	XP_004502568.1	--	--	--
XLOC_009338	No	No	No	No	--	--	F	K00088
XLOC_009349	No	No	DEG(I+M)	No	--	--	--	--
XLOC_009351	No	No	No	No	EUC62011.1	--	--	--
XLOC_009354	No	No	No	No	XP_001952514.1	--	--	--
XLOC_009357	No	No	No	No	--	--	--	--
XLOC_009359	No	No	No	No	AFM78642.1	--	--	--
XLOC_009360	No	No	No	No	CCT73568.1	--	--	--
XLOC_009361	DEG(M)	DEG(M)	No	No	EMT65997.1	--	--	--
XLOC_009365	No	No	No	No	--	--	--	--
XLOC_009367	No	No	DEG(I+M)	No	XP_004515105.1	--	R	K03327
XLOC_009369	No	No	No	No	CCO29515.1	--	--	--
XLOC_009373	DEG(M)	DEG(M)	DEG(I+M)	DEG(M)	XP_003044825.1	--	J	--
XLOC_009376	No	No	No	No	--	--	--	--
XLOC_009378	No	No	No	No	KEP52948.1	--	--	--
XLOC_009379	No	No	No	No	--	--	--	--
XLOC_009380	No	No	No	No	KEP49026.1	--	--	--
XLOC_009383	DEG(M)	DEG(M)	No	No	KLO87023.1	--	R	--
XLOC_009388	No	No	No	No	KDN48989.1	--	C	--
XLOC_009389	DEG(M)	No	No	No	EXK29109.1	--	--	--
XLOC_009390	No	No	DEG(I+M)	No	--	--	--	K01213
XLOC_009397	No	No	No	No	--	--	--	--
XLOC_009401	No	No	No	No	--	--	R	--
XLOC_009406	No	No	No	No	CCO31105.1	--	--	--
XLOC_009407	DEG(M)	No	No	No	--	--	--	--

Differentially expressed gene (DEG) IDs	*C. microphyllum* root and shoot-specific (>2 fold)	*C. microphyllum* root and shoot-specific (>10 fold)	ICC4958 and *C. microphyllum* root-specific (>2 fold)	*C. microphyllum* root-specific (>2 fold)	Functional Annotation			
					NCBI nr IDs	TFs	KOG	KEGG
XLOC_009413	No	No	No	No	AKM28423.1	--	I	--
XLOC_009414	No	No	No	No	XP_008906464.1	--	--	--
XLOC_009416	No	No	No	No	CCO30083.1	--	--	--
XLOC_009418	No	No	No	No	--	--	--	--
XLOC_009420	DEG(M)	No	No	No	EUC55036.1	--	--	--
XLOC_009421	No	No	No	No	--	--	--	--
XLOC_009429	No	No	No	No	EMT68070.1	--	--	--
XLOC_009431	No	No	No	No	--	--	--	--
XLOC_009435	DEG(M)	DEG(M)	No	No	NP_001156067.1	NF-YB	J	K02973
XLOC_009436	No	No	DEG(I+M)	No	XP_004507133.1	--	--	--
XLOC_009439	No	No	No	No	XP_008906396.1	--	--	--
XLOC_009440	No	No	No	No	XP_004494242.1	--	--	--
XLOC_009449	No	No	No	No	--	--	--	--
XLOC_009450	No	No	No	No	AJQ30115.1	--	G	K00873
XLOC_009452	No	No	No	No	AAT70232.1	--	X	--
XLOC_009455	No	No	No	No	--	--	--	--
XLOC_009457	DEG(M)	No	No	No	--	--	--	--
XLOC_009458	DEG(M)	DEG(M)	DEG(I+M)	DEG(M)	--	--	--	--
XLOC_009459	DEG(M)	No	No	No	--	--	--	--
XLOC_009460	No	No	No	No	KIH64240.1	--	Z	--
XLOC_009461	No	No	No	No	--	--	--	--
XLOC_009462	DEG(M)	No	No	No	NP_001119657.1	--	--	--
XLOC_009470	No	No	No	No	--	--	--	--
XLOC_009471	No	No	No	No	--	--	--	--
XLOC_009473	No	No	No	No	--	--	--	--
XLOC_009480	DEG(M)	No	DEG(I+M)	DEG(M)	--	--	--	K02901
XLOC_009481	No	No	No	No	--	--	--	--
XLOC_009483	No	No	No	No	--	--	--	--
XLOC_009489	DEG(M)	DEG(M)	DEG(I+M)	DEG(M)	KDN49348.1	--	--	--
XLOC_009490	No	No	No	No	XP_004512336.1	--	--	--
XLOC_009492	No	No	No	No	--	--	--	--
XLOC_009494	DEG(M)	No	No	No	XP_008908636.1	--	--	--
XLOC_009497	No	No	No	No	AIR95625.1	--	--	--
XLOC_009499	DEG(M)	No	No	No	CCO29767.1	--	--	--
XLOC_009500	No	No	No	No	--	--	--	--
XLOC_009501	No	No	No	No	--	--	--	--
XLOC_009502	No	No	DEG(I+M)	No	--	--	--	--
XLOC_009505	No	No	No	No	NP_001119624.1	--	--	--
XLOC_009510	DEG(M)	No	No	No	--	--	J	--
XLOC_009511	DEG(M)	No	No	No	XP_008472763.1	--	J	--

Differentially expressed gene (DEG) IDs	*C. microphyllum* root and shoot-specific (>2 fold)	*C. microphyllum* root and shoot-specific (>10 fold)	ICC4958 and *C. microphyllum* root-specific (>2 fold)	*C. microphyllum* root-specific (>2 fold)	Functional Annotation			
					NCBI nr IDs	TFs	KOG	KEGG
XLOC_009512	No	No	No	No	XP_008183165.1	--	O	--
XLOC_009513	No	No	No	No	--	--	--	--
XLOC_009514	No	No	No	No	--	--	--	--
XLOC_009519	DEG(M)	No	No	No	XP_002516097.1	SBP	L	--
XLOC_009520	DEG(M)	No	No	No	CEL63702.1	--	T	K06268
XLOC_009526	No	No	No	No	--	--	--	--
XLOC_009530	No	No	No	No	--	--	--	--
XLOC_009531	DEG(M)	No	No	No	--	--	--	--
XLOC_009533	No	No	No	No	--	--	--	K15255
XLOC_009536	No	No	No	No	XP_008180676.1	--	--	K12898
XLOC_009537	DEG(M)	No	No	No	--	--	--	--
XLOC_009538	DEG(M)	DEG(M)	No	No	--	--	--	K13449
XLOC_009540	No	No	No	No	ETM34398.1	--	--	--
XLOC_009543	No	No	No	No	--	--	--	--
XLOC_009544	No	No	No	No	--	--	--	--
XLOC_009545	No	No	No	No	XP_003304805.1	--	C	K00658
XLOC_009546	No	No	DEG(I+M)	No	--	--	--	--
XLOC_009548	DEG(M)	No	No	No	--	--	--	--
XLOC_009549	No	No	No	No	XP_008917446.1	--	--	--
XLOC_009557	No	No	No	No	XP_003051819.1	--	--	--
XLOC_009558	No	No	No	No	--	--	--	K07342
XLOC_009563	DEG(M)	DEG(M)	No	No	XP_003053763.1	--	O	--
XLOC_009567	No	No	No	No	--	--	--	--
XLOC_009568	No	No	No	No	--	--	--	--
XLOC_009570	No	No	No	No	KDN48683.1	--	--	--
XLOC_009573	DEG(M)	No	No	No	ACG60422.1	--	O	--
XLOC_009574	DEG(M)	No	No	No	ACG60422.1	--	O	--
XLOC_009575	No	No	No	No	XP_009523702.1	--	--	K01880
XLOC_009576	No	No	No	No	ETP54151.1	--	--	K01880
XLOC_009577	No	No	No	No	--	--	--	--
XLOC_009583	DEG(M)	DEG(M)	DEG(I+M)	DEG(M)	CEL62026.1	--	--	--
XLOC_009587	DEG(M)	DEG(M)	DEG(I+M)	DEG(M)	--	--	--	--
XLOC_009591	No	No	No	No	EUC67258.1	--	--	--
XLOC_009592	DEG(M)	No	No	No	KEP54399.1	--	EH	--
XLOC_009594	No	No	DEG(I+M)	No	--	--	RQ	--
XLOC_009595	DEG(M)	No	No	No	--	--	--	--
XLOC_009602	DEG(M)	No	No	No	EXL75673.1	--	--	--
XLOC_009606	No	No	No	No	--	--	--	--
XLOC_009607	No	No	No	No	ETK87454.1	--	--	--
XLOC_009610	No	No	No	No	--	--	--	--

Differentially expressed gene (DEG) IDs	*C. microphyllum* root and shoot-specific (>2 fold)	*C. microphyllum* root and shoot-specific (>10 fold)	ICC4958 and *C. microphyllum* root-specific (>2 fold)	*C. microphyllum* root-specific (>2 fold)	Functional Annotation			
					NCBI nr IDs	TFs	KOG	KEGG
XLOC_009615	DEG(M)	No	No	No	--	--	--	--
XLOC_009616	No	No	No	No	--	--	J	--
XLOC_009617	No	No	No	No	AIR95627.1	--	--	--
XLOC_009618	No	No	DEG(I+M)	No	XP_004490219.1	--	--	--
XLOC_009619	No	No	No	No	--	--	--	--
XLOC_009623	No	No	No	No	--	--	--	--
XLOC_009626	No	No	No	No	XP_003302069.1	--	--	--
XLOC_009630	No	No	No	No	KEP47352.1	--	--	--
XLOC_009631	DEG(M)	DEG(M)	No	No	--	--	--	--
XLOC_009632	No	No	No	No	--	--	--	--
XLOC_009637	DEG(M)	No	DEG(I+M)	DEG(M)	--	--	--	--
XLOC_009638	DEG(M)	DEG(M)	DEG(I+M)	DEG(M)	--	--	--	--
XLOC_009642	DEG(M)	No	No	No	--	--	--	--
XLOC_009643	DEG(M)	No	No	No	ACH48198.1	--	J	K03263
XLOC_009645	No	No	No	No	CCO27844.1	--	--	--
XLOC_009651	No	No	No	No	CAC51084.1	--	O	--
XLOC_009652	DEG(M)	No	No	No	--	bZIP	J	--
XLOC_009656	No	No	No	No	--	--	--	--
XLOC_009660	No	No	No	No	XP_013300516.1	--	O	--
XLOC_009663	No	No	No	No	--	--	--	--
XLOC_009667	No	No	No	No	XP_012088674.1	--	PQ	--
XLOC_009670	DEG(M)	No	No	No	--	--	--	--
XLOC_009675	DEG(M)	No	No	No	--	--	--	--
XLOC_009676	No	No	No	No	--	--	--	--
XLOC_009677	DEG(M)	DEG(M)	DEG(I+M)	DEG(M)	--	--	--	--
XLOC_009680	No	No	No	No	--	--	--	--
XLOC_009683	No	No	No	No	XP_002527764.1	C3H	J	K11593
XLOC_009686	No	No	No	No	--	--	--	--
XLOC_009690	No	No	No	No	--	--	--	--
XLOC_009693	DEG(M)	DEG(M)	DEG(I+M)	DEG(M)	--	--	--	--
XLOC_009696	No	No	No	No	XP_007156891.1	--	--	--
XLOC_009697	No	No	No	No	XP_003048195.1	--	--	--
XLOC_009703	No	No	No	No	EUC61046.1	--	--	--
XLOC_009706	DEG(M)	No	No	No	--	--	--	--
XLOC_009710	No	No	No	No	XP_003045241.1	--	--	--
XLOC_009711	No	No	No	No	XP_008187830.1	--	--	--
XLOC_009712	DEG(M)	No	No	No	--	--	--	--
XLOC_009716	DEG(M)	No	No	No	--	--	--	--
XLOC_009717	No	No	No	No	--	--	--	--
XLOC_009718	No	No	No	No	--	--	--	--

Differentially expressed gene (DEG) IDs	*C. microphyllum* root and shoot-specific (>2 fold)	*C. microphyllum* root and shoot-specific (>10 fold)	ICC4958 and *C. microphyllum* root-specific (>2 fold)	*C. microphyllum* root-specific (>2 fold)	Functional Annotation			
					NCBI nr IDs	TFs	KOG	KEGG
XLOC_009719	DEG(M)	No	No	No	XP_001653838.1	--	O	--
XLOC_009720	No	No	No	No	--	--	--	--
XLOC_009721	DEG(M)	No	DEG(I+M)	DEG(M)	XP_012071492.1	--	T	--
XLOC_009725	No	No	No	No	ETP03989.1	--	--	--
XLOC_009728	No	No	No	No	--	--	--	--
XLOC_009733	No	No	No	No	--	--	--	--
XLOC_009738	No	No	No	No	--	--	--	--
XLOC_009740	No	No	No	No	ETP53260.1	--	--	--
XLOC_009746	No	No	No	No	XP_008179177.1	--	--	--
XLOC_009749	No	No	No	No	--	--	--	--
XLOC_009750	No	No	No	No	--	--	--	--
XLOC_009752	No	No	No	No	XP_001648359.1	--	T	--
XLOC_009754	DEG(M)	No	No	No	AAU84947.1	--	O	--
XLOC_009755	No	No	No	No	XP_008905415.1	--	--	--
XLOC_009757	No	No	No	No	XP_003049108.1	--	--	--
XLOC_009759	No	No	No	No	--	--	--	--
XLOC_009760	No	No	No	No	XP_002516875.1	--	R	K10144
XLOC_009767	No	No	No	No	CEL61825.1	--	--	K01968
XLOC_009769	No	No	No	No	XP_001950388.1	--	--	--
XLOC_009770	No	No	No	No	--	--	--	--
XLOC_009771	No	No	No	No	--	--	--	--
XLOC_009772	DEG(M)	No	No	No	AAN05584.1	--	J	K02908
XLOC_009776	No	No	No	No	ETI33666.1	--	--	--
XLOC_009777	No	No	No	No	EUC67463.1	--	--	--
XLOC_009779	No	No	No	No	--	--	--	--
XLOC_009780	No	No	No	No	--	--	--	--
XLOC_009785	No	No	No	No	XP_001654061.1	--	R	--
XLOC_009788	DEG(M)	No	No	No	--	--	J	K02915
XLOC_009791	No	No	No	No	KIO21728.1	--	--	--
XLOC_009792	No	No	No	No	--	--	--	--
XLOC_009794	No	No	No	No	XP_004489741.1	--	--	--
XLOC_009795	No	No	No	No	--	--	--	--
XLOC_009796	No	No	No	No	--	C3H	--	--
XLOC_009797	No	No	No	No	--	--	--	--
XLOC_009798	DEG(M)	No	No	No	--	--	--	--
XLOC_009799	DEG(M)	DEG(M)	No	No	XP_003044840.1	--	J	--
XLOC_009800	No	No	No	No	XP_001947250.2	--	--	--
XLOC_009801	No	No	No	No	--	--	--	--
XLOC_009803	No	No	No	No	--	--	--	--
XLOC_009804	No	No	No	No	--	--	--	K11294

Differentially expressed gene (DEG) IDs	*C. microphyllum* root and shoot-specific (>2 fold)	*C. microphyllum* root and shoot-specific (>10 fold)	ICC4958 and *C. microphyllum* root-specific (>2 fold)	*C. microphyllum* root-specific (>2 fold)	Functional Annotation			
					NCBI nr IDs	TFs	KOG	KEGG
XLOC_009807	No	No	No	No	XP_002532951.1	bHLH	T	K00921
XLOC_009813	No	No	DEG(I+M)	No	--	--	--	--
XLOC_009815	DEG(M)	DEG(M)	DEG(I+M)	DEG(M)	--	--	--	--
XLOC_009816	No	No	DEG(I+M)	No	XP_004500912.1	--	--	--
XLOC_009819	No	No	No	No	--	--	--	--
XLOC_009823	No	No	No	No	--	--	--	--
XLOC_009827	No	No	No	No	EUC60294.1	--	--	--
XLOC_009831	No	No	No	No	XP_002514031.1	--	U	K12402
XLOC_009837	No	No	No	No	KJB79157.1	--	E	K01586
XLOC_009838	No	No	No	No	XP_003048555.1	--	--	--
XLOC_009841	DEG(M)	No	No	No	--	--	--	--
XLOC_009842	No	No	No	No	XP_008180318.1	--	--	--
XLOC_009844	No	No	No	No	--	--	--	--
XLOC_009847	No	No	No	No	--	--	--	--
XLOC_009849	No	No	No	No	--	--	O	--
XLOC_009850	No	No	No	No	--	--	--	--
XLOC_009851	No	No	No	No	--	--	--	--
XLOC_009852	No	No	No	No	--	--	--	--
XLOC_009858	DEG(M)	DEG(M)	No	No	EGU79684.1	bZIP	--	--
XLOC_009868	No	No	No	No	--	--	--	--
XLOC_009870	No	No	No	No	XP_003040327.1	--	--	--
XLOC_009872	DEG(M)	No	No	No	--	GRAS	F	K00940
XLOC_009874	No	No	No	No	--	--	--	K01363
XLOC_009875	DEG(M)	No	No	No	--	--	--	--
XLOC_009878	DEG(M)	No	No	No	XP_008915297.1	--	--	--
XLOC_009879	No	No	No	No	--	--	--	--
XLOC_009880	No	No	No	No	--	--	J	--
XLOC_009883	No	No	No	No	CEL58250.1	--	--	--
XLOC_009886	No	No	No	No	--	--	--	--
XLOC_009891	DEG(M)	No	No	No	ETO68108.1	--	--	--
XLOC_009892	DEG(M)	DEG(M)	DEG(I+M)	DEG(M)	--	--	--	--
XLOC_009896	No	No	No	No	XP_001902862.1	--	U	--
XLOC_009897	No	No	No	No	KDN42651.1	--	--	--
XLOC_009901	DEG(M)	No	No	No	--	--	--	--
XLOC_009902	No	No	No	No	--	--	--	--
XLOC_009904	No	No	No	No	XP_009519285.1	--	C	--
XLOC_009909	No	No	No	No	--	--	--	--
XLOC_009910	No	No	No	No	--	--	--	--
XLOC_009912	DEG(M)	No	No	No	XP_003039876.1	--	--	--
XLOC_009913	No	No	No	No	--	--	--	--

Differentially expressed gene (DEG) IDs	*C. microphyllum* root and shoot-specific (>2 fold)	*C. microphyllum* root and shoot-specific (>10 fold)	ICC4958 and *C. microphyllum* root-specific (>2 fold)	*C. microphyllum* root-specific (>2 fold)	Functional Annotation			
					NCBI nr IDs	TFs	KOG	KEGG
XLOC_009915	DEG(M)	No	No	No	KDN44586.1	--	--	--
XLOC_009918	DEG(M)	No	No	No	--	--	--	--
XLOC_009919	No	No	DEG(I+M)	No	XP_004502947.1	--	--	--
XLOC_009920	No	No	No	No	XP_008914176.1	--	--	--
XLOC_009921	No	No	No	No	EUC62463.1	--	--	--
XLOC_009922	No	No	No	No	XP_003628784.1	--	Q	--
XLOC_009923	DEG(M)	DEG(M)	No	No	ACJ64045.1	--	--	--
XLOC_009924	No	No	DEG(I+M)	No	--	--	--	--
XLOC_009926	No	No	No	No	XP_001947747.2	--	--	--
XLOC_009929	No	No	No	No	NP_001155664.1	--	O	--
XLOC_009930	No	No	No	No	--	--	--	--
XLOC_009933	No	No	No	No	--	--	--	--
XLOC_009936	DEG(M)	DEG(M)	DEG(I+M)	DEG(M)	--	--	--	--
XLOC_009938	No	No	No	No	XP_001945190.2	--	--	--
XLOC_009941	No	No	No	No	XP_008904152.1	--	C	--
XLOC_009945	No	No	No	No	XP_012067263.1	--	--	--
XLOC_009947	DEG(M)	DEG(M)	DEG(I+M)	DEG(M)	--	--	--	K02983
XLOC_009950	No	No	No	No	--	--	--	--
XLOC_009954	No	No	No	No	--	--	E	--
XLOC_009955	No	No	No	No	XP_008903595.1	--	A	K12812
XLOC_009956	No	No	No	No	XP_008188167.1	--	--	--
XLOC_009957	No	No	No	No	--	--	--	--
XLOC_009959	DEG(M)	No	No	No	CCO29410.1	--	--	--
XLOC_009960	No	No	No	No	--	--	--	--
XLOC_009963	No	No	No	No	XP_001663853.1	--	--	--
XLOC_009966	No	No	No	No	--	--	QG	--
XLOC_009996	DEG(M)	No	No	No	--	--	--	--
XLOC_009999	DEG(M)	No	No	No	--	--	GC	--
XLOC_010013	No	No	No	No	--	--	--	K02994
XLOC_010022	DEG(M)	DEG(M)	No	No	--	--	--	--
XLOC_010025	DEG(M)	DEG(M)	DEG(I+M)	DEG(M)	--	--	--	--
XLOC_010027	DEG(M)	No	DEG(I+M)	DEG(M)	XP_001785975.1	--	--	--
XLOC_010029	DEG(M)	No	No	No	--	--	--	--
XLOC_010030	DEG(M)	DEG(M)	DEG(I+M)	DEG(M)	--	--	--	--
XLOC_010036	DEG(M)	DEG(M)	No	No	XP_012571187.1	--	--	K13065
XLOC_010075	No	No	No	No	--	--	--	K00430
XLOC_010079	No	No	DEG(I+M)	No	--	--	--	K00430
XLOC_010080	DEG(M)	No	DEG(I+M)	DEG(M)	XP_012569820.1	--	--	K00430
XLOC_010081	No	No	DEG(I+M)	No	XP_012569824.1	--	--	K00430
XLOC_010135	DEG(M)	DEG(M)	DEG(I+M)	DEG(M)	--	--	--	--

Differentially expressed gene (DEG) IDs	*C. microphyllum* root and shoot-specific (>2 fold)	*C. microphyllum* root and shoot-specific (>10 fold)	ICC4958 and *C. microphyllum* root-specific (>2 fold)	*C. microphyllum* root-specific (>2 fold)	Functional Annotation			
					NCBI nr IDs	TFs	KOG	KEGG
XLOC_010146	DEG(M)	No	DEG(I+M)	DEG(M)	--	--	--	--
XLOC_010164	DEG(M)	DEG(M)	DEG(I+M)	DEG(M)	XP_004510422.1	--	--	--
XLOC_010166	DEG(M)	DEG(M)	DEG(I+M)	DEG(M)	XP_004510422.1	--	--	--
XLOC_010182	No	No	No	No	--	--	--	--
XLOC_010205	DEG(M)	DEG(M)	DEG(I+M)	DEG(M)	--	--	--	K00799
XLOC_010211	DEG(M)	DEG(M)	No	No	--	--	--	--
XLOC_010212	No	No	No	No	--	--	--	--
XLOC_010216	No	No	No	No	--	--	--	--
XLOC_010222	No	No	No	No	--	--	--	K11982
XLOC_010227	DEG(M)	DEG(M)	DEG(I+M)	DEG(M)	--	--	--	--
XLOC_010229	DEG(M)	DEG(M)	DEG(I+M)	DEG(M)	--	--	--	--
XLOC_010231	No	No	DEG(I+M)	No	--	--	--	--
XLOC_010233	No	No	No	No	--	--	--	--
XLOC_010234	No	No	No	No	--	--	--	--
XLOC_010236	No	No	No	No	CEL51532.1	--	--	--
XLOC_010241	No	No	No	No	XP_004507508.1	--	G	K01623
XLOC_010242	No	No	No	No	--	--	--	--
XLOC_010254	DEG(M)	DEG(M)	No	No	KEH35116.1	--	--	--
XLOC_010255	No	No	No	No	--	--	--	--
XLOC_010306	DEG(M)	DEG(M)	DEG(I+M)	DEG(M)	--	--	R	--
XLOC_010313	No	No	No	No	--	--	--	--
XLOC_010314	No	No	No	No	--	--	--	--
XLOC_010317	No	No	No	No	CEL52136.1	--	--	K03250
XLOC_010320	No	No	No	No	--	--	--	--
XLOC_010323	No	No	No	No	XP_004494761.1	--	RI	--
XLOC_010324	No	No	No	No	--	--	--	--
XLOC_010325	No	No	No	No	--	--	--	--
XLOC_010326	No	No	No	No	KDN45159.1	--	--	--
XLOC_010328	DEG(M)	DEG(M)	No	No	XP_003051567.1	--	--	--
XLOC_010330	No	No	No	No	--	--	--	--
XLOC_010332	DEG(M)	DEG(M)	DEG(I+M)	DEG(M)	CCO37931.1	--	J	--
XLOC_010333	No	No	No	No	KLO17759.1	--	E	K00819
XLOC_010334	No	No	No	No	--	--	--	--
XLOC_010335	No	No	No	No	--	--	--	--
XLOC_010336	No	No	No	No	--	--	T	--
XLOC_010337	No	No	No	No	--	--	--	--
XLOC_010339	No	No	No	No	AHG94993.1	--	--	--
XLOC_010343	No	No	No	No	--	--	--	--
XLOC_010344	No	No	No	No	EUC66223.1	--	R	--
XLOC_010346	No	No	No	No	EUC56118.1	--	--	--

Differentially expressed gene (DEG) IDs	*C. microphyllum* root and shoot-specific (>2 fold)	*C. microphyllum* root and shoot-specific (>10 fold)	ICC4958 and *C. microphyllum* root-specific (>2 fold)	*C. microphyllum* root-specific (>2 fold)	Functional Annotation			
					NCBI nr IDs	TFs	KOG	KEGG
XLOC_010347	No	No	No	No	XP_001951599.1	--	J	K03242
XLOC_010348	No	No	No	No	--	--	--	--
XLOC_010354	No	No	No	No	--	--	--	--
XLOC_010357	DEG(M)	No	No	No	--	--	--	--
XLOC_010358	No	No	No	No	CEL58779.1	--	E	--
XLOC_010362	No	No	DEG(I+M)	No	--	--	--	--
XLOC_010363	No	No	No	No	--	--	--	--
XLOC_010364	No	No	No	No	--	--	--	--
XLOC_010366	No	No	No	No	--	--	--	--
XLOC_010367	No	No	No	No	XP_004509861.1	--	--	--
XLOC_010369	No	No	No	No	XP_010269878.1	--	O	--
XLOC_010370	No	No	No	No	XP_011114541.1	--	--	--
XLOC_010373	No	No	No	No	--	bHLH	--	K13422
XLOC_010376	No	No	No	No	--	--	--	--
XLOC_010377	No	No	No	No	--	--	--	--
XLOC_010381	No	No	No	No	EGU74500.1	--	--	--
XLOC_010383	No	No	No	No	EUC64914.1	--	--	--
XLOC_010385	No	No	No	No	EUC67208.1	--	--	--
XLOC_010386	No	No	No	No	AIL48319.1	--	J	--
XLOC_010388	No	No	No	No	--	--	--	--
XLOC_010389	No	No	No	No	--	--	--	--
XLOC_010392	No	No	No	No	--	--	--	--
XLOC_010393	DEG(M)	No	No	No	--	--	--	--
XLOC_010394	No	No	No	No	--	--	--	K11290
XLOC_010407	No	No	No	No	XP_008894090.1	--	--	--
XLOC_010408	No	No	No	No	--	--	--	--
XLOC_010414	No	No	No	No	--	--	--	--
XLOC_010415	No	No	No	No	--	--	--	--
XLOC_010419	No	No	No	No	--	--	AJ	K01681
XLOC_010420	No	No	No	No	KDN44663.1	--	--	--
XLOC_010421	No	No	No	No	--	--	CE	--
XLOC_010422	No	No	No	No	--	--	--	--
XLOC_010423	DEG(M)	No	No	No	--	--	--	--
XLOC_010425	No	No	No	No	ETP37312.1	--	--	--
XLOC_010426	No	No	No	No	--	--	--	--
XLOC_010427	No	No	No	No	KEP55826.1	--	--	--
XLOC_010428	No	No	No	No	XP_009833579.1	--	A	--
XLOC_010432	No	No	No	No	--	--	--	--
XLOC_010437	DEG(M)	No	No	No	--	--	--	--
XLOC_010438	DEG(M)	No	No	No	--	--	--	--

**Differentially expressed gene (DEG) IDs**	***C. microphyllum* root and shoot-specific (>2 fold)**	***C. microphyllum* root and shoot-specific (>10 fold)**	**ICC4958 and *C. microphyllum* root-specific (>2 fold)**	***C. microphyllum* root-specific (>2 fold)**	**Functional Annotation**			
					**NCBI nr IDs**	**TFs**	**KOG**	**KEGG**
XLOC_010439	DEG(M)	No	No	No	KIK68638.1	MYB_related	O	--
XLOC_010440	No	No	No	No	XP_008906945.1	--	C	K00382
XLOC_010442	DEG(M)	DEG(M)	DEG(I+M)	DEG(M)	ADC80655.1	--	H	--
XLOC_010444	No	No	No	No	CCO26295.1	--	C	--
XLOC_010447	No	No	No	No	XP_002510855.1	--	Q	--
XLOC_010448	No	No	No	No	--	--	U	--
XLOC_010449	No	No	No	No	--	--	--	--
XLOC_010450	No	No	DEG(I+M)	No	--	--	--	--
XLOC_010451	DEG(M)	No	No	No	--	--	--	--
XLOC_010453	DEG(M)	DEG(M)	No	No	--	--	--	--
XLOC_010455	No	No	No	No	CCI43571.1	--	J	--
XLOC_010459	No	No	No	No	CEL59175.1	--	--	--
XLOC_010461	No	No	No	No	--	--	--	--
XLOC_010464	No	No	No	No	--	--	O	K09497
XLOC_010465	No	No	No	No	--	--	--	--
XLOC_010466	No	No	No	No	KDN47467.1	--	--	--
XLOC_010468	No	No	No	No	EUC59202.1	--	--	K08493
XLOC_010475	DEG(M)	No	No	No	CEL53001.1	--	G	--
XLOC_010478	No	No	No	No	--	--	--	--
XLOC_010479	No	No	DEG(I+M)	No	NP_001296595.1	--	--	--
XLOC_010481	No	No	No	No	--	--	--	--
XLOC_010482	No	No	No	No	--	--	--	--
XLOC_010483	No	No	No	No	EUC67162.1	--	--	--
XLOC_010487	DEG(M)	No	No	No	EMT63630.1	--	--	--
XLOC_010489	No	No	No	No	--	--	--	--
XLOC_010491	DEG(M)	No	No	No	--	--	--	--
XLOC_010494	No	No	No	No	--	--	--	--
XLOC_010495	No	No	No	No	--	--	--	--
XLOC_010499	No	No	No	No	XP_003052164.1	--	--	--
XLOC_010500	No	No	No	No	XP_003052164.1	--	--	--
XLOC_010504	No	No	No	No	CCO35958.1	--	--	--
XLOC_010505	No	No	No	No	CEL61236.1	--	--	--
XLOC_010506	DEG(M)	No	No	No	--	--	O	K01366
XLOC_010507	DEG(M)	DEG(M)	No	No	XP_004490698.1	--	--	K00430
XLOC_010509	DEG(M)	No	No	No	CEL52693.1	--	--	--
XLOC_010510	No	No	No	No	CCO31494.1	--	--	--
XLOC_010515	No	No	No	No	--	--	--	--
XLOC_010516	DEG(M)	No	No	No	XP_008915971.1	--	--	--
XLOC_010517	DEG(M)	No	No	No	XP_008915971.1	--	--	--
XLOC_010519	No	No	No	No	--	--	--	--

Differentially expressed gene (DEG) IDs	*C. microphyllum* root and shoot-specific (>2 fold)	*C. microphyllum* root and shoot-specific (>10 fold)	ICC4958 and *C. microphyllum* root-specific (>2 fold)	*C. microphyllum* root-specific (>2 fold)	Functional Annotation			
					NCBI nr IDs	TFs	KOG	KEGG
XLOC_010520	No	No	No	No	--	--	--	--
XLOC_010527	DEG(M)	No	No	No	--	--	--	--
XLOC_010528	No	No	No	No	--	--	--	--
XLOC_010529	No	No	No	No	--	--	--	--
XLOC_010531	No	No	DEG(I+M)	No	--	--	--	--
XLOC_010532	DEG(M)	No	No	No	XP_004504096.1	--	--	--
XLOC_010538	DEG(M)	No	No	No	ETK77434.1	--	--	--
XLOC_010541	DEG(M)	No	No	No	NP_001155721.1	--	J	K02936
XLOC_010543	No	No	No	No	--	--	I	--
XLOC_010550	No	No	No	No	XP_001653750.1	--	G	--
XLOC_010551	DEG(M)	No	No	No	XP_009834734.1	--	G	--
XLOC_010552	DEG(M)	No	No	No	--	--	--	--
XLOC_010556	No	No	No	No	XP_004499030.1	--	--	--
XLOC_010557	No	No	DEG(I+M)	No	XP_004507879.1	--	--	K00430
XLOC_010559	No	No	No	No	--	--	--	--
XLOC_010560	No	No	No	No	CEL55086.1	--	--	--
XLOC_010564	DEG(M)	No	No	No	EUC67155.1	--	J	K02898
XLOC_010565	DEG(M)	No	No	No	EUC67155.1	--	J	--
XLOC_010569	No	No	No	No	XP_008901054.1	--	--	--
XLOC_010570	DEG(M)	No	No	No	--	--	--	--
XLOC_010572	DEG(M)	DEG(M)	No	No	--	--	--	--
XLOC_010575	No	No	DEG(I+M)	No	XP_012573661.1	bHLH	--	--
XLOC_010582	DEG(M)	No	No	No	CCO29438.1	--	--	--
XLOC_010589	No	No	No	No	CEL63738.1	--	--	--
XLOC_010594	No	No	No	No	KEP49481.1	--	--	--
XLOC_010600	No	No	No	No	--	--	--	--
XLOC_010607	DEG(M)	No	No	No	--	--	--	--
XLOC_010612	No	No	No	No	--	--	--	--
XLOC_010613	No	No	No	No	--	--	--	K02638
XLOC_010615	No	No	No	No	KEP47671.1	--	--	--
XLOC_010616	DEG(M)	No	No	No	CEL63148.1	--	--	--
XLOC_010617	No	No	No	No	--	--	--	--
XLOC_010618	DEG(M)	No	No	No	EUC55870.1	--	--	K04078
XLOC_010619	No	No	No	No	CCO28281.1	--	--	--
XLOC_010621	No	No	No	No	--	--	--	--
XLOC_010622	No	No	No	No	--	--	--	--
XLOC_010626	DEG(M)	No	No	No	--	--	--	--
XLOC_010628	DEG(M)	No	No	No	NP_001156277.1	--	J	--
XLOC_010630	No	No	No	No	XP_001944221.1	--	G	--
XLOC_010631	No	No	No	No	KEP54214.1	--	--	--

Differentially expressed gene (DEG) IDs	***C. microphyllum* root and shoot-specific (>2 fold)**	***C. microphyllum* root and shoot-specific (>10 fold)**	**ICC4958 and *C. microphyllum* root-specific (>2 fold)**	***C. microphyllum* root-specific (>2 fold)**	**Functional Annotation**			
					**NCBI nr IDs**	**TFs**	**KOG**	**KEGG**
XLOC_010633	DEG(M)	No	No	No	--	--	--	--
XLOC_010638	No	No	No	No	EUC62897.1	--	--	--
XLOC_010639	DEG(M)	DEG(M)	DEG(I+M)	DEG(M)	EUC60645.1	--	--	--
XLOC_010646	No	No	No	No	ETL39427.1	--	--	--
XLOC_010648	DEG(M)	DEG(M)	DEG(I+M)	DEG(M)	--	--	--	--
XLOC_010649	No	No	No	No	--	--	--	--
XLOC_010653	No	No	No	No	XP_002513689.1	--	J	K02920
XLOC_010654	No	No	No	No	XP_004506748.2	--	X	--
XLOC_010658	No	No	No	No	CEL53890.1	--	--	--
XLOC_010660	No	No	No	No	--	--	--	--
XLOC_010661	No	No	No	No	--	--	--	--
XLOC_010665	No	No	No	No	EUC62976.1	--	--	--
XLOC_010669	No	No	No	No	EUC64935.1	--	--	--
XLOC_010670	No	No	No	No	--	--	--	--
XLOC_010672	DEG(M)	No	No	No	NP_001119683.1	--	C	--
XLOC_010676	DEG(M)	No	No	No	--	--	--	--
XLOC_010683	DEG(M)	No	No	No	--	--	--	--
XLOC_010684	No	No	No	No	XP_008915127.1	--	--	--
XLOC_010686	No	No	No	No	ETP45330.1	--	--	--
XLOC_010687	DEG(M)	No	No	No	--	--	--	--
XLOC_010692	No	No	No	No	--	--	Q	--
XLOC_010693	No	No	No	No	--	--	--	--
XLOC_010694	No	No	No	No	--	--	--	--
XLOC_010696	No	No	No	No	EWG39712.1	--	--	--
XLOC_010700	No	No	No	No	KDN49503.1	--	DZU	--
XLOC_010703	No	No	No	No	--	--	--	--
XLOC_010707	No	No	No	No	--	--	--	--
XLOC_010708	No	No	DEG(I+M)	No	--	--	RT	--
XLOC_010710	DEG(M)	DEG(M)	No	No	XP_009255266.1	--	J	--
XLOC_010714	No	No	No	No	--	--	--	--
XLOC_010724	No	No	DEG(I+M)	No	XP_004488666.1	--	--	--
XLOC_010746	No	No	DEG(I+M)	No	--	bZIP	K	K14431
XLOC_010748	DEG(M)	DEG(M)	No	No	--	--	--	--
XLOC_010754	No	No	No	No	--	--	--	--
XLOC_010755	No	No	No	No	--	--	--	--
XLOC_010757	DEG(M)	No	DEG(I+M)	DEG(M)	CCO37931.1	--	J	K02971
XLOC_010758	DEG(M)	No	No	No	--	--	--	--
XLOC_010768	No	No	No	No	--	--	Q	K00517
XLOC_010770	DEG(M)	No	No	No	EUC55313.1	--	--	--
XLOC_010779	DEG(M)	No	No	No	--	--	--	--

Differentially expressed gene (DEG) IDs	*C. microphyllum* root and shoot-specific (>2 fold)	*C. microphyllum* root and shoot-specific (>10 fold)	ICC4958 and *C. microphyllum* root-specific (>2 fold)	*C. microphyllum* root-specific (>2 fold)	Functional Annotation			
					NCBI nr IDs	TFs	KOG	KEGG
XLOC_010780	DEG(M)	No	No	No	CEL62550.1	--	--	--
XLOC_010781	DEG(M)	No	No	No	EUC63435.1	--	--	K15040
XLOC_010782	DEG(M)	No	No	No	EUC63435.1	--	--	--
XLOC_010788	No	No	DEG(I+M)	No	XP_004487036.1	MYB	--	K09422
XLOC_010789	No	No	No	No	KEP52359.1	--	--	--
XLOC_010790	DEG(M)	No	No	No	KEP52359.1	--	--	--
XLOC_010791	DEG(M)	No	No	No	--	--	--	--
XLOC_010799	DEG(M)	No	No	No	--	--	--	--
XLOC_010800	DEG(M)	DEG(M)	DEG(I+M)	DEG(M)	--	--	--	--
XLOC_010806	No	No	DEG(I+M)	No	--	--	G	--
XLOC_010807	No	No	No	No	--	--	G	K13680
XLOC_010810	DEG(M)	No	DEG(I+M)	DEG(M)	--	--	--	--
XLOC_010811	DEG(M)	No	DEG(I+M)	DEG(M)	--	--	--	--
XLOC_010812	DEG(M)	No	DEG(I+M)	DEG(M)	--	--	--	--
XLOC_010814	DEG(M)	DEG(M)	No	No	--	--	--	--
XLOC_010817	No	No	No	No	CEL60500.1	--	--	--
XLOC_010821	DEG(M)	DEG(M)	No	No	--	--	--	K11252
XLOC_010822	DEG(M)	No	No	No	--	--	--	--
XLOC_010824	No	No	No	No	--	--	--	--
XLOC_010825	No	No	No	No	ADJ67162.1	--	O	--
XLOC_010830	DEG(M)	No	No	No	KDN42702.1	--	--	--
XLOC_010833	DEG(M)	No	No	No	--	--	--	--
XLOC_010834	No	No	No	No	--	--	--	--
XLOC_010836	No	No	No	No	XP_012184316.1	GATA	K	K03120
XLOC_010838	No	No	No	No	EUC65476.1	--	--	--
XLOC_010841	No	No	No	No	CCO27038.1	--	--	--
XLOC_010845	No	No	No	No	XP_008910729.1	--	--	K07512
XLOC_010848	DEG(M)	No	No	No	--	--	--	K02891
XLOC_010849	No	No	No	No	XP_003047074.1	--	--	--
XLOC_010852	No	No	No	No	--	--	--	K20285
XLOC_010853	DEG(M)	No	No	No	--	--	--	--
XLOC_010855	No	No	No	No	--	--	--	--
XLOC_010857	No	No	No	No	--	--	--	--
XLOC_010860	DEG(M)	No	No	No	--	--	J	--
XLOC_010862	No	No	No	No	CEL56100.1	--	--	--
XLOC_010866	DEG(M)	No	No	No	--	--	--	--
XLOC_010869	DEG(M)	No	No	No	CCO32556.1	--	--	--
XLOC_010878	No	No	No	No	--	--	U	K18211
XLOC_010880	DEG(M)	DEG(M)	DEG(I+M)	DEG(M)	XP_008906950.1	bZIP	J	--
XLOC_010887	No	No	No	No	--	--	--	--

Differentially expressed gene (DEG) IDs	*C. microphyllum* root and shoot-specific (>2 fold)	*C. microphyllum* root and shoot-specific (>10 fold)	ICC4958 and *C. microphyllum* root-specific (>2 fold)	*C. microphyllum* root-specific (>2 fold)	Functional Annotation			
					NCBI nr IDs	TFs	KOG	KEGG
XLOC_010888	No	No	No	No	ETL95261.1	--	--	K00383
XLOC_010890	No	No	No	No	--	--	--	--
XLOC_010893	No	No	DEG(I+M)	No	XP_004494352.1	C2H2	--	--
XLOC_010894	No	No	No	No	EUC64077.1	--	--	--
XLOC_010895	No	No	No	No	ETI38736.1	--	--	--
XLOC_010897	No	No	No	No	--	--	--	--
XLOC_010899	No	No	No	No	--	--	--	K00799
XLOC_010901	No	No	No	No	XP_008905064.1	--	--	--
XLOC_010903	No	No	No	No	--	--	--	--
XLOC_010904	No	No	No	No	ETP08419.1	--	--	--
XLOC_010907	DEG(M)	No	No	No	--	--	--	--
XLOC_010909	No	No	No	No	--	--	--	--
XLOC_010912	No	No	No	No	XP_004509321.1	--	I	--
XLOC_010914	No	No	No	No	--	--	--	--
XLOC_010917	DEG(M)	DEG(M)	No	No	XP_003049840.1	--	--	--
XLOC_010919	No	No	No	No	--	--	--	--
XLOC_010926	No	No	No	No	XP_012196884.1	--	G	--
XLOC_010928	No	No	No	No	XP_008185691.1	--	--	--
XLOC_010930	No	No	No	No	--	--	--	--
XLOC_010931	No	No	No	No	--	--	--	--
XLOC_010932	No	No	No	No	--	--	--	--
XLOC_010935	No	No	No	No	XP_001950262.1	MYB_related	G	--
XLOC_010937	No	No	No	No	XP_001945658.1	--	--	--
XLOC_010939	No	No	No	No	--	--	--	--
XLOC_010943	No	No	No	No	XP_001951495.1	--	--	--
XLOC_010945	DEG(M)	No	DEG(I+M)	DEG(M)	XP_004513315.2	--	--	K00430
XLOC_010951	No	No	No	No	--	--	--	--
XLOC_010953	DEG(M)	No	No	No	KDN43622.1	--	--	--
XLOC_010954	No	No	No	No	CCO26440.1	--	--	--
XLOC_010958	DEG(M)	DEG(M)	No	No	--	--	--	--
XLOC_010962	No	No	No	No	KNG50345.1	--	--	--
XLOC_010963	DEG(M)	DEG(M)	DEG(I+M)	DEG(M)	XP_009515296.1	--	J	--
XLOC_010968	DEG(M)	No	DEG(I+M)	DEG(M)	--	--	--	--
XLOC_010971	DEG(M)	DEG(M)	DEG(I+M)	DEG(M)	ETI31495.1	--	C	--
XLOC_010972	No	No	No	No	XP_008908083.1	--	O	K02726
XLOC_010978	No	No	No	No	--	--	--	--
XLOC_010979	No	No	No	No	CCO26763.1	GATA	U	--
XLOC_010985	No	No	No	No	--	--	--	--
XLOC_010986	No	No	No	No	--	--	--	--
XLOC_010987	No	No	No	No	XP_002514075.1	NF-YA	R	K06889

**Differentially expressed gene (DEG) IDs**	***C. microphyllum* root and shoot-specific (>2 fold)**	***C. microphyllum* root and shoot-specific (>10 fold)**	**ICC4958 and *C. microphyllum* root-specific (>2 fold)**	***C. microphyllum* root-specific (>2 fold)**	**Functional Annotation**			
					**NCBI nr IDs**	**TFs**	**KOG**	**KEGG**
XLOC_010988	No	No	No	No	--	--	--	--
XLOC_010989	DEG(M)	DEG(M)	DEG(I+M)	DEG(M)	EXM32694.1	--	--	--
XLOC_010990	DEG(M)	No	No	No	XP_008895979.1	--	--	--
XLOC_010991	No	No	No	No	--	--	J	K02906
XLOC_010993	DEG(M)	No	No	No	--	--	--	--
XLOC_010995	DEG(M)	No	No	No	XP_004499674.1	--	--	K20131
XLOC_011002	DEG(M)	No	No	No	--	--	--	--
XLOC_011003	No	No	No	No	--	--	--	--
XLOC_011005	No	No	No	No	CCO37080.1	--	--	--
XLOC_011006	DEG(M)	No	No	No	KIJ11196.1	MYB_related	O	--
XLOC_011007	No	No	No	No	XP_003049442.1	--	--	--
XLOC_011009	No	No	No	No	--	--	--	K05759
XLOC_011017	No	No	No	No	--	--	X	--
XLOC_011018	No	No	No	No	XP_008185784.1	--	R	--
XLOC_011022	No	No	No	No	--	--	--	--
XLOC_011025	No	No	No	No	--	bHLH	F	K00759
XLOC_011028	DEG(M)	No	No	No	XP_008179624.1	--	J	K02998
XLOC_011030	No	No	No	No	XP_012070671.1	--	IT	K00901
XLOC_011031	DEG(M)	No	DEG(I+M)	DEG(M)	CCO26990.1	--	--	--
XLOC_011033	DEG(M)	No	No	No	ETM48634.1	--	A	--
XLOC_011037	DEG(M)	No	No	No	CEL57376.1	--	--	--
XLOC_011042	DEG(M)	No	DEG(I+M)	DEG(M)	EQB52632.1	--	J	--
XLOC_011043	DEG(M)	DEG(M)	DEG(I+M)	DEG(M)	--	--	--	--
XLOC_011048	No	No	No	No	--	--	--	--
XLOC_011059	No	No	No	No	CEL54958.1	--	--	--
XLOC_011060	No	No	No	No	EUC61371.1	--	--	--
XLOC_011062	No	No	No	No	--	--	--	K12129
XLOC_011064	DEG(M)	No	No	No	XP_001945548.1	--	--	--
XLOC_011067	DEG(M)	No	No	No	ETK90515.1	--	--	--
XLOC_011068	DEG(M)	No	No	No	XP_003611248.1	--	J	--
XLOC_011069	No	No	No	No	--	--	--	--
XLOC_011071	DEG(M)	No	No	No	--	--	--	--
XLOC_011072	No	No	No	No	CCO35325.1	--	--	--
XLOC_011073	No	No	No	No	--	--	--	--
XLOC_011074	No	No	No	No	YP_002149726.1	--	C	K02690
XLOC_011075	No	No	No	No	ELU45591.1	--	--	K09553
XLOC_011077	No	No	No	No	ETM47429.1	--	--	--
XLOC_011082	DEG(M)	DEG(M)	No	No	--	--	--	--
XLOC_011084	No	No	No	No	--	--	X	--
XLOC_011086	DEG(M)	No	No	No	KEP47284.1	--	--	--

Differentially expressed gene (DEG) IDs	*C. microphyllum* root and shoot-specific (>2 fold)	*C. microphyllum* root and shoot-specific (>10 fold)	ICC4958 and *C. microphyllum* root-specific (>2 fold)	*C. microphyllum* root-specific (>2 fold)	Functional Annotation			
					NCBI nr IDs	TFs	KOG	KEGG
XLOC_011087	No	No	No	No	--	--	--	--
XLOC_011091	No	No	No	No	XP_003240264.2	--	--	--
XLOC_011092	No	No	No	No	XP_003042271.1	--	--	--
XLOC_011093	No	No	DEG(I+M)	No	XP_004498932.1	MYB	--	K09422
XLOC_011094	DEG(M)	No	No	No	CCO33930.1	--	--	--
XLOC_011098	No	No	No	No	XP_012086381.1	--	--	--
XLOC_011100	DEG(M)	No	No	No	NP_001153848.1	--	E	K01915
XLOC_011101	No	No	No	No	--	--	--	--
XLOC_011107	No	No	No	No	KDN45662.1	--	--	--
XLOC_011110	DEG(M)	No	No	No	--	--	--	--
XLOC_011111	DEG(M)	DEG(M)	No	No	XP_003051630.1	--	--	--
XLOC_011113	DEG(M)	DEG(M)	DEG(I+M)	DEG(M)	KDN50332.1	--	J	--
XLOC_011116	No	No	No	No	CCO26277.1	--	A	K12812
XLOC_011118	DEG(M)	No	DEG(I+M)	DEG(M)	--	--	--	--
XLOC_011119	No	No	No	No	XP_003049903.1	--	--	--
XLOC_011122	No	No	No	No	XP_008893280.1	--	--	--
XLOC_011125	No	No	No	No	--	--	--	--
XLOC_011126	DEG(M)	No	No	No	--	--	--	--
XLOC_011127	DEG(M)	No	No	No	XP_008907920.1	--	--	--
XLOC_011129	No	No	No	No	--	--	--	--
XLOC_011130	No	No	No	No	--	--	--	--
XLOC_011131	No	No	No	No	--	--	--	--
XLOC_011132	DEG(M)	No	No	No	--	--	--	--
XLOC_011133	No	No	No	No	--	--	--	K09510
XLOC_011134	No	No	DEG(I+M)	No	XP_012572983.1	--	--	K01166
XLOC_011135	No	No	No	No	--	--	--	K11099
XLOC_011136	No	No	No	No	XP_001948493.1	--	--	--
XLOC_011140	No	No	No	No	--	--	--	--
XLOC_011142	No	No	No	No	--	--	--	--
XLOC_011144	No	No	No	No	NP_001191909.1	--	--	--
XLOC_011146	No	No	No	No	--	--	--	--
XLOC_011148	No	No	No	No	--	--	--	--
XLOC_011155	No	No	No	No	--	--	--	--
XLOC_011162	No	No	No	No	--	--	--	--
XLOC_011163	No	No	No	No	--	--	--	K11251
XLOC_011164	No	No	No	No	--	--	--	--
XLOC_011169	DEG(M)	No	No	No	--	--	--	--
XLOC_011172	No	No	DEG(I+M)	No	--	NAC	--	--
XLOC_011175	No	No	No	No	--	--	--	--
XLOC_011178	No	No	No	No	--	--	--	--

**Differentially expressed gene (DEG) IDs**	***C. microphyllum* root and shoot-specific (>2 fold)**	***C. microphyllum* root and shoot-specific (>10 fold)**	**ICC4958 and *C. microphyllum* root-specific (>2 fold)**	***C. microphyllum* root-specific (>2 fold)**	**Functional Annotation**			
					**NCBI nr IDs**	**TFs**	**KOG**	**KEGG**
XLOC_011180	DEG(M)	No	DEG(I+M)	DEG(M)	KDN51287.1	--	F	K00940
XLOC_011185	No	No	No	No	ETI51930.1	--	--	--
XLOC_011187	DEG(M)	No	No	No	--	--	--	--
XLOC_011190	DEG(M)	No	No	No	CEL54132.1	--	--	--
XLOC_011195	No	No	No	No	--	--	--	--
XLOC_011199	No	No	No	No	--	--	G	K01555
XLOC_011201	No	No	No	No	--	--	--	K08235
XLOC_011203	DEG(M)	No	No	No	XP_003047619.1	--	--	--
XLOC_011204	DEG(M)	DEG(M)	No	No	--	--	--	--
XLOC_011207	No	No	No	No	--	--	--	--
XLOC_011210	No	No	No	No	--	--	--	--
XLOC_011212	No	No	No	No	EUC63445.1	--	--	--
XLOC_011213	No	No	No	No	--	--	--	--
XLOC_011214	DEG(M)	No	No	No	XP_005179845.1	bZIP	J	K02865
XLOC_011215	No	No	No	No	--	--	--	--
XLOC_011220	No	No	No	No	--	--	--	K00417
XLOC_011221	No	No	No	No	XP_009523025.1	--	--	K09273
XLOC_011222	No	No	No	No	--	--	--	--
XLOC_011225	No	No	No	No	NP_001191867.1	--	Z	--
XLOC_011228	DEG(M)	No	DEG(I+M)	DEG(M)	XP_002514108.1	--	G	--
XLOC_011230	No	No	No	No	KEP54849.1	--	--	--
XLOC_011232	No	No	No	No	--	Nin-like	--	--
XLOC_011233	No	No	No	No	XP_008179181.1	--	T	--
XLOC_011236	No	No	No	No	--	--	--	--
XLOC_011239	No	No	No	No	XP_003301469.1	--	--	--
XLOC_011248	No	No	No	No	ETL96747.1	--	--	--
XLOC_011249	No	No	No	No	CCO30290.1	--	--	--
XLOC_011251	No	No	No	No	--	--	--	--
XLOC_011252	No	No	DEG(I+M)	No	--	--	--	--
XLOC_011253	No	No	No	No	XP_003042634.1	--	--	--
XLOC_011260	No	No	No	No	--	--	--	--
XLOC_011262	DEG(M)	No	No	No	--	--	--	--
XLOC_011263	No	No	DEG(I+M)	No	XP_004506817.1	--	--	K07253
XLOC_011265	No	No	No	No	XP_001663323.1	--	R	K11643
XLOC_011266	No	No	No	No	XP_001663323.1	--	R	--
XLOC_011270	DEG(M)	No	No	No	--	--	--	--
XLOC_011271	DEG(M)	No	No	No	CCO32683.1	--	--	--
XLOC_011272	No	No	No	No	--	--	--	--
XLOC_011273	DEG(M)	No	No	No	XP_003047931.1	--	--	--
XLOC_011276	DEG(M)	No	DEG(I+M)	DEG(M)	CCO32800.1	--	--	--

Differentially expressed gene (DEG) IDs	*C. microphyllum* root and shoot-specific (>2 fold)	*C. microphyllum* root and shoot-specific (>10 fold)	ICC4958 and *C. microphyllum* root-specific (>2 fold)	*C. microphyllum* root-specific (>2 fold)	Functional Annotation			
					NCBI nr IDs	TFs	KOG	KEGG
XLOC_011277	No	No	No	No	--	--	--	--
XLOC_011281	No	No	DEG(I+M)	No	--	--	--	K13248
XLOC_011282	No	No	No	No	XP_003053508.1	--	--	--
XLOC_011284	No	No	No	No	XP_003051713.1	--	O	--
XLOC_011285	No	No	No	No	XP_003051713.1	--	--	--
XLOC_011289	DEG(M)	DEG(M)	DEG(I+M)	DEG(M)	--	--	--	--
XLOC_011293	DEG(M)	No	No	No	--	--	--	--
XLOC_011301	No	No	No	No	KEP46871.1	--	--	--
XLOC_011302	DEG(M)	No	No	No	XP_003043299.1	--	--	--
XLOC_011305	DEG(M)	DEG(M)	DEG(I+M)	DEG(M)	--	--	--	--
XLOC_011308	No	No	No	No	CCO28424.1	--	--	K00830
XLOC_011311	No	No	No	No	XP_008901230.1	--	--	--
XLOC_011313	No	No	No	No	--	--	--	--
XLOC_011314	DEG(M)	No	No	No	--	--	--	--
XLOC_011315	No	No	No	No	XP_003046801.1	--	--	--
XLOC_011316	No	No	No	No	ETM33041.1	--	--	K00278
XLOC_011317	No	No	No	No	CEL55661.1	--	--	--
XLOC_011318	No	No	No	No	--	--	O	--
XLOC_011319	No	No	No	No	--	--	O	--
XLOC_011330	DEG(M)	No	No	No	--	--	J	K02885
XLOC_011332	No	No	No	No	--	--	--	--
XLOC_011337	No	No	No	No	--	--	--	K02904
XLOC_011348	No	No	No	No	XP_003302259.1	--	--	--
XLOC_011350	DEG(M)	No	No	No	--	--	--	K12897
XLOC_011355	No	No	No	No	--	--	--	--
XLOC_011358	DEG(M)	No	DEG(I+M)	DEG(M)	--	--	--	--
XLOC_011360	No	No	No	No	--	--	R	K13348
XLOC_011363	No	No	No	No	CCO29027.1	--	--	K08232
XLOC_011364	DEG(M)	No	No	No	--	--	--	--
XLOC_011366	No	No	No	No	--	--	--	K02939
XLOC_011367	No	No	No	No	--	--	--	--
XLOC_011369	DEG(M)	No	No	No	KEP52891.1	--	E	--
XLOC_011371	No	No	No	No	--	--	--	--
XLOC_011376	No	No	DEG(I+M)	No	XP_004511327.2	--	--	--
XLOC_011377	DEG(M)	No	No	No	--	--	--	--
XLOC_011378	DEG(M)	No	No	No	--	--	--	--
XLOC_011381	No	No	No	No	--	--	--	--
XLOC_011384	DEG(M)	No	No	No	--	--	--	--
XLOC_011386	DEG(M)	No	DEG(I+M)	DEG(M)	XP_003052603.1	--	--	--
XLOC_011396	DEG(M)	DEG(M)	DEG(I+M)	DEG(M)	EWZ36057.1	--	--	--

**Differentially expressed gene (DEG) IDs**	***C. microphyllum* root and shoot-specific (>2 fold)**	***C. microphyllum* root and shoot-specific (>10 fold)**	**ICC4958 and *C. microphyllum* root-specific (>2 fold)**	***C. microphyllum* root-specific (>2 fold)**	**Functional Annotation**			
					**NCBI nr IDs**	**TFs**	**KOG**	**KEGG**
XLOC_011401	No	No	No	No	--	--	--	--
XLOC_011404	DEG(M)	No	No	No	--	--	--	--
XLOC_011406	DEG(M)	No	DEG(I+M)	DEG(M)	--	--	--	K14638
XLOC_011407	No	No	No	No	--	--	--	--
XLOC_011409	No	No	No	No	--	--	--	--
XLOC_011412	No	No	No	No	KFM82853.1	--	E	--
XLOC_011413	DEG(M)	No	DEG(I+M)	DEG(M)	--	--	--	--
XLOC_011415	DEG(M)	No	No	No	--	--	--	--
XLOC_011423	No	No	No	No	EUC66874.1	--	--	--
XLOC_011424	No	No	No	No	XP_004486636.1	--	--	--
XLOC_011425	DEG(M)	No	DEG(I+M)	DEG(M)	--	--	--	--
XLOC_011427	No	No	No	No	EXM01719.1	--	--	--
XLOC_011430	No	No	No	No	--	--	--	--
XLOC_011436	No	No	No	No	--	--	--	--
XLOC_011437	DEG(M)	No	No	No	EFA77491.1	--	J	--
XLOC_011438	DEG(M)	No	No	No	--	--	--	--
XLOC_011440	No	No	No	No	--	--	--	--
XLOC_011441	No	No	No	No	ETL78956.1	--	--	--
XLOC_011445	No	No	No	No	XP_008893580.1	--	--	--
XLOC_011446	DEG(M)	No	No	No	--	--	--	K00412
XLOC_011447	DEG(M)	No	No	No	--	--	--	--
XLOC_011448	DEG(M)	No	No	No	--	--	--	--
XLOC_011462	DEG(M)	No	No	No	--	--	--	--
XLOC_011465	No	No	No	No	--	--	--	--
XLOC_011472	DEG(M)	DEG(M)	DEG(I+M)	DEG(M)	--	--	X	--
XLOC_011475	No	No	No	No	XP_003046572.1	--	--	--
XLOC_011476	No	No	No	No	--	--	--	--
XLOC_011481	No	No	DEG(I+M)	No	--	--	--	--
XLOC_011484	No	No	No	No	--	--	--	--
XLOC_011486	DEG(M)	DEG(M)	DEG(I+M)	DEG(M)	EXK42078.1	--	J	--
XLOC_011487	No	No	No	No	--	--	--	--
XLOC_011490	DEG(M)	No	DEG(I+M)	DEG(M)	--	--	--	--
XLOC_011491	DEG(M)	No	No	No	--	--	--	--
XLOC_011493	DEG(M)	No	No	No	ETI49322.1	--	I	K05607
XLOC_011494	No	No	No	No	--	--	--	--
XLOC_011498	DEG(M)	No	No	No	--	--	--	--
XLOC_011515	No	No	No	No	EUC65253.1	--	--	--
XLOC_011519	DEG(M)	DEG(M)	DEG(I+M)	DEG(M)	KEP48892.1	--	--	--
XLOC_011525	No	No	No	No	--	--	--	--
XLOC_011526	No	No	No	No	--	--	--	--

Differentially expressed gene (DEG) IDs	*C. microphyllum* root and shoot-specific (>2 fold)	*C. microphyllum* root and shoot-specific (>10 fold)	ICC4958 and *C. microphyllum* root-specific (>2 fold)	*C. microphyllum* root-specific (>2 fold)	Functional Annotation			
					NCBI nr IDs	TFs	KOG	KEGG
XLOC_011527	DEG(M)	No	No	No	--	--	--	--
XLOC_011535	No	No	No	No	--	--	--	--
XLOC_011536	No	No	No	No	--	--	--	--
XLOC_011538	No	No	No	No	--	--	--	--
XLOC_011543	No	No	No	No	--	--	--	--
XLOC_011545	No	No	No	No	NP_001155692.1	--	--	--
XLOC_011547	No	No	No	No	CEL59170.1	--	--	--
XLOC_011548	No	No	No	No	--	--	--	--
XLOC_011549	No	No	No	No	--	--	Z	--
XLOC_011553	DEG(M)	No	No	No	--	--	--	--
XLOC_011554	No	No	DEG(I+M)	No	XP_004493556.1	--	--	--
XLOC_011555	No	No	No	No	XP_003041026.1	--	--	--
XLOC_011558	No	No	No	No	XP_003053801.1	--	--	--
XLOC_011561	No	No	No	No	--	--	--	--
XLOC_011568	No	No	DEG(I+M)	No	--	--	GM	K12449
XLOC_011570	No	No	No	No	CEL55071.1	--	--	--
XLOC_011572	No	No	No	No	XP_004507604.1	--	--	--
XLOC_011577	DEG(M)	No	No	No	--	--	W	--
XLOC_011578	No	No	No	No	XP_002532315.1	--	R	K20032
XLOC_011579	No	No	No	No	--	--	--	--
XLOC_011582	No	No	No	No	--	--	--	--
XLOC_011583	No	No	No	No	--	--	--	--
XLOC_011592	No	No	No	No	--	--	--	--
XLOC_011595	DEG(M)	No	No	No	ADV40078.1	--	J	--
XLOC_011596	DEG(M)	DEG(M)	No	No	XP_003043509.1	--	J	--
XLOC_011597	No	No	No	No	--	--	--	--
XLOC_011601	No	No	No	No	--	--	--	--
XLOC_011602	No	No	No	No	--	--	--	K02903
XLOC_011603	No	No	No	No	--	--	--	--
XLOC_011604	No	No	No	No	--	--	G	K01792
XLOC_011605	No	No	No	No	--	--	--	--
XLOC_011607	DEG(M)	No	No	No	--	--	--	--
XLOC_011608	No	No	No	No	KEP55436.1	--	--	--
XLOC_011609	No	No	No	No	--	--	AJ	--
XLOC_011611	No	No	No	No	--	--	--	--
XLOC_011614	No	No	DEG(I+M)	No	--	--	S	--
XLOC_011616	No	No	No	No	--	--	--	--
XLOC_011617	DEG(M)	No	No	No	--	--	--	--
XLOC_011618	No	No	No	No	--	--	--	--
XLOC_011621	No	No	No	No	XP_004516643.1	--	G	K03841

Differentially expressed gene (DEG) IDs	*C. microphyllum* root and shoot-specific (>2 fold)	*C. microphyllum* root and shoot-specific (>10 fold)	ICC4958 and *C. microphyllum* root-specific (>2 fold)	*C. microphyllum* root-specific (>2 fold)	Functional Annotation			
					NCBI nr IDs	TFs	KOG	KEGG
XLOC_011622	DEG(M)	DEG(M)	No	No	--	--	--	--
XLOC_011638	No	No	No	No	--	--	X	K03531
XLOC_011670	No	No	DEG(I+M)	No	--	--	--	--
XLOC_011675	No	No	DEG(I+M)	No	XP_004498641.1	--	Q	--
XLOC_011677	No	No	DEG(I+M)	No	--	--	Q	--
XLOC_011679	No	No	No	No	--	--	--	--
XLOC_011680	DEG(M)	No	No	No	XP_004336884.1	--	O	--
XLOC_011681	DEG(M)	DEG(M)	No	No	KDQ15383.1	--	O	--
XLOC_011682	DEG(M)	DEG(M)	DEG(I+M)	DEG(M)	--	--	--	--
XLOC_011685	DEG(M)	No	No	No	--	--	O	--
XLOC_011686	DEG(M)	No	DEG(I+M)	DEG(M)	KDN34974.1	--	O	K06630
XLOC_011687	DEG(M)	No	No	No	KDN34974.1	--	O	--
XLOC_011703	DEG(M)	DEG(M)	No	No	XP_004501726.1	--	--	--
XLOC_011730	No	No	No	No	EUC61690.1	--	--	--
XLOC_011754	DEG(M)	No	No	No	KEP52621.1	--	--	--
XLOC_011756	No	No	No	No	KEP52621.1	--	--	--
XLOC_011778	DEG(M)	No	No	No	ELU44881.1	--	--	--
XLOC_011782	DEG(M)	No	No	No	CCO33298.1	--	--	--
XLOC_011815	DEG(M)	No	DEG(I+M)	DEG(M)	--	--	--	--
XLOC_011819	No	No	No	No	XP_009983603.1	YABBY	OR	--
XLOC_011824	DEG(M)	DEG(M)	DEG(I+M)	DEG(M)	XP_002902716.1	YABBY	J	--
XLOC_011825	DEG(M)	No	No	No	XP_001663729.1	--	J	--
XLOC_011827	DEG(M)	DEG(M)	DEG(I+M)	DEG(M)	XP_002902716.1	YABBY	J	--
XLOC_011828	DEG(M)	DEG(M)	DEG(I+M)	DEG(M)	XP_012209083.1	YABBY	OR	--
XLOC_011831	DEG(M)	DEG(M)	DEG(I+M)	DEG(M)	XP_012209083.1	YABBY	OR	--
XLOC_011832	DEG(M)	No	No	No	KIO29346.1	--	--	--
XLOC_011833	DEG(M)	No	No	No	KIO29346.1	--	--	--
XLOC_011834	DEG(M)	No	DEG(I+M)	DEG(M)	KDN50292.1	--	--	--
XLOC_011835	DEG(M)	No	No	No	EUC53913.1	--	--	--
XLOC_011836	DEG(M)	No	No	No	CEL57610.1	--	--	--
XLOC_011843	No	No	No	No	EUC56065.1	--	--	--
XLOC_011844	No	No	No	No	EUC56065.1	--	--	--
XLOC_011846	No	No	No	No	EUC56065.1	--	--	--
XLOC_011847	No	No	No	No	EUC56065.1	--	--	--
XLOC_011848	DEG(M)	No	No	No	EUC56065.1	--	--	--
XLOC_011849	No	No	No	No	EUC56065.1	--	--	--
XLOC_011881	No	No	No	No	XP_003053706.1	--	O	--
XLOC_011883	DEG(M)	No	No	No	XP_007278159.1	--	O	--
XLOC_011884	DEG(M)	No	DEG(I+M)	DEG(M)	CEL51818.1	--	O	--
XLOC_011886	DEG(M)	No	No	No	CEL58561.1	--	O	--

Differentially expressed gene (DEG) IDs	*C. microphyllum* root and shoot-specific (>2 fold)	*C. microphyllum* root and shoot-specific (>10 fold)	ICC4958 and *C. microphyllum* root-specific (>2 fold)	*C. microphyllum* root-specific (>2 fold)	Functional Annotation			
					NCBI nr IDs	TFs	KOG	KEGG
XLOC_011888	DEG(M)	DEG(M)	No	No	XP_003046172.1	--	O	--
XLOC_011889	DEG(M)	DEG(M)	No	No	--	--	--	--
XLOC_011901	No	No	DEG(I+M)	No	XP_004506004.1	AP2	--	K09284
XLOC_011954	No	No	No	No	--	--	--	--
XLOC_011955	DEG(M)	No	No	No	--	--	--	--
XLOC_011956	DEG(M)	DEG(M)	DEG(I+M)	DEG(M)	CAB88203.1	--	--	--
XLOC_011957	DEG(M)	DEG(M)	DEG(I+M)	DEG(M)	CAB88203.1	--	--	--
XLOC_011958	DEG(M)	No	DEG(I+M)	DEG(M)	CAB88203.1	--	--	--
XLOC_011990	No	No	No	No	--	--	RI	--
XLOC_011999	No	No	No	No	--	--	--	--
XLOC_012000	DEG(M)	DEG(M)	No	No	EXM09116.1	--	G	--
XLOC_012002	No	No	DEG(I+M)	No	--	--	Q	--
XLOC_012003	No	No	No	No	CCO33106.1	--	--	--
XLOC_012008	No	No	No	No	EUC65021.1	--	--	--
XLOC_012010	No	No	No	No	--	--	--	--
XLOC_012016	No	No	No	No	CEL53071.1	--	--	--
XLOC_012018	No	No	No	No	--	--	--	--
XLOC_012020	No	No	No	No	--	--	--	K15803
XLOC_012021	No	No	No	No	--	--	O	--
XLOC_012022	DEG(M)	DEG(M)	No	No	--	--	X	--
XLOC_012026	DEG(M)	No	No	No	EUC66309.1	--	--	--
XLOC_012027	No	No	No	No	--	--	--	--
XLOC_012028	DEG(M)	No	No	No	KEP49879.1	--	--	--
XLOC_012029	DEG(M)	No	No	No	KEP49879.1	--	--	--
XLOC_012031	No	No	No	No	--	--	--	--
XLOC_012032	No	No	No	No	--	--	--	--
XLOC_012035	No	No	No	No	CEL56505.1	--	--	--
XLOC_012040	DEG(M)	No	No	No	ETL98916.1	--	--	--
XLOC_012041	No	No	No	No	XP_004515311.1	--	--	--
XLOC_012046	DEG(M)	DEG(M)	DEG(I+M)	DEG(M)	--	--	--	--
XLOC_012047	No	No	No	No	KDN39727.1	--	--	K02150
XLOC_012049	DEG(M)	DEG(M)	DEG(I+M)	DEG(M)	ELU43412.1	--	--	--
XLOC_012050	No	No	No	No	EUC57304.1	--	--	--
XLOC_012051	No	No	No	No	--	--	--	--
XLOC_012053	No	No	No	No	ELU41444.1	--	--	--
XLOC_012060	No	No	No	No	--	--	--	--
XLOC_012063	DEG(M)	No	No	No	--	--	--	K02135
XLOC_012066	No	No	No	No	--	--	--	--
XLOC_012071	No	No	No	No	AAK15639.1	--	--	--
XLOC_012076	No	No	No	No	--	--	--	--

Differentially expressed gene (DEG) IDs	*C. microphyllum* root and shoot-specific (>2 fold)	*C. microphyllum* root and shoot-specific (>10 fold)	ICC4958 and *C. microphyllum* root-specific (>2 fold)	*C. microphyllum* root-specific (>2 fold)	Functional Annotation			
					NCBI nr IDs	TFs	KOG	KEGG
XLOC_012080	No	No	No	No	EUC65169.1	--	--	K00559
XLOC_012082	No	No	No	No	XP_001945876.1	--	--	--
XLOC_012083	No	No	No	No	--	--	--	--
XLOC_012087	No	No	No	No	--	--	--	--
XLOC_012091	No	No	No	No	EWY89513.1	--	--	--
XLOC_012092	No	No	No	No	XP_003053119.1	--	--	--
XLOC_012093	No	No	No	No	--	--	--	--
XLOC_012095	No	No	No	No	--	NAC	--	--
XLOC_012096	No	No	No	No	--	--	TZ	--
XLOC_012098	No	No	No	No	XP_004512714.1	--	--	K03327
XLOC_012099	No	No	No	No	--	--	--	--
XLOC_012103	No	No	No	No	--	--	--	--
XLOC_012104	No	No	No	No	XP_007275127.1	--	--	--
XLOC_012105	No	No	No	No	--	--	--	--
XLOC_012106	No	No	No	No	--	--	--	--
XLOC_012108	No	No	No	No	KIO23142.1	--	Z	--
XLOC_012110	No	No	No	No	--	--	--	--
XLOC_012111	No	No	No	No	--	--	--	--
XLOC_012113	No	No	No	No	--	--	--	--
XLOC_012115	No	No	No	No	XP_001950452.1	--	--	--
XLOC_012117	No	No	No	No	--	--	--	--
XLOC_012122	DEG(M)	DEG(M)	DEG(I+M)	DEG(M)	--	--	--	--
XLOC_012123	No	No	No	No	--	--	--	--
XLOC_012127	DEG(M)	No	No	No	XP_001944154.1	--	R	K09272
XLOC_012130	DEG(M)	No	No	No	XP_002903084.1	--	AJ	K14565
XLOC_012131	DEG(M)	No	No	No	--	--	--	--
XLOC_012132	No	No	No	No	--	--	--	--
XLOC_012137	DEG(M)	No	No	No	--	--	--	--
XLOC_012139	No	No	No	No	--	--	--	--
XLOC_012144	No	No	No	No	CEL62086.1	--	--	K02137
XLOC_012146	No	No	No	No	--	--	--	--
XLOC_012148	No	No	No	No	--	--	--	--
XLOC_012149	No	No	No	No	--	--	--	--
XLOC_012151	DEG(M)	No	No	No	NP_001280432.1	--	--	K10249
XLOC_012153	No	No	No	No	--	--	--	--
XLOC_012154	No	No	No	No	XP_008178298.1	--	GOT	K09667
XLOC_012155	No	No	No	No	XP_001662665.1	--	O	--
XLOC_012156	DEG(M)	No	No	No	AAU84937.1	--	G	--
XLOC_012157	DEG(M)	No	No	No	NP_001119660.1	--	J	K02910
XLOC_012158	DEG(M)	No	No	No	--	--	--	--

**Differentially expressed gene (DEG) IDs**	***C. microphyllum* root and shoot-specific (>2 fold)**	***C. microphyllum* root and shoot-specific (>10 fold)**	**ICC4958 and *C. microphyllum* root-specific (>2 fold)**	***C. microphyllum* root-specific (>2 fold)**	**Functional Annotation**			
					**NCBI nr IDs**	**TFs**	**KOG**	**KEGG**
XLOC_012161	No	No	No	No	EUC61132.1	--	--	--
XLOC_012168	DEG(M)	No	No	No	--	--	--	--
XLOC_012173	No	No	No	No	--	--	--	K05754
XLOC_012174	No	No	DEG(I+M)	No	XP_003616581.1	WOX	--	--
XLOC_012179	No	No	No	No	XP_002530169.1	--	J	K02962
XLOC_012182	No	No	No	No	--	--	--	--
XLOC_012185	No	No	No	No	XP_001659715.1	--	J	--
XLOC_012187	No	No	No	No	XP_003043912.1	--	--	--
XLOC_012189	No	No	No	No	--	--	--	--
XLOC_012196	DEG(M)	DEG(M)	DEG(I+M)	DEG(M)	--	--	--	--
XLOC_012199	No	No	No	No	XP_008907933.1	--	--	--
XLOC_012201	DEG(M)	No	No	No	--	--	--	--
XLOC_012202	No	No	No	No	--	--	--	--
XLOC_012204	DEG(M)	No	No	No	--	--	--	--
XLOC_012205	No	No	No	No	EXK99674.1	--	--	--
XLOC_012207	DEG(M)	DEG(M)	DEG(I+M)	DEG(M)	--	--	J	--
XLOC_012208	No	No	No	No	NP_001156707.1	--	--	--
XLOC_012211	No	No	No	No	XP_009435328.1	--	X	--
XLOC_012213	DEG(M)	No	No	No	--	--	--	K03113
XLOC_012214	DEG(M)	No	No	No	--	--	--	--
XLOC_012216	No	No	No	No	--	--	--	K09579
XLOC_012218	DEG(M)	No	No	No	KEP47021.1	--	--	--
XLOC_012224	DEG(M)	DEG(M)	DEG(I+M)	DEG(M)	--	--	--	--
XLOC_012225	No	No	No	No	CEL56740.1	--	R	--
XLOC_012230	DEG(M)	No	No	No	--	--	--	--
XLOC_012231	DEG(M)	No	No	No	--	--	--	--
XLOC_012234	DEG(M)	No	No	No	EUC54645.1	--	--	--
XLOC_012235	No	No	No	No	--	--	--	--
XLOC_012237	No	No	No	No	KEP46222.1	--	--	K01183
XLOC_012242	No	No	No	No	--	--	--	--
XLOC_012245	No	No	No	No	--	--	--	--
XLOC_012248	DEG(M)	No	No	No	--	--	--	--
XLOC_012252	No	No	No	No	--	--	--	--
XLOC_012255	DEG(M)	No	No	No	--	--	--	--
XLOC_012256	No	No	No	No	XP_003048876.1	--	--	--
XLOC_012258	No	No	No	No	KDO58905.1	--	G	K01180
XLOC_012260	No	No	No	No	--	--	--	--
XLOC_012261	DEG(M)	No	No	No	--	--	--	--
XLOC_012264	No	No	No	No	--	--	--	--
XLOC_012269	No	No	No	No	--	--	--	--

Differentially expressed gene (DEG) IDs	***C. microphyllum* root and shoot-specific (>2 fold)**	***C. microphyllum* root and shoot-specific (>10 fold)**	**ICC4958 and *C. microphyllum* root-specific (>2 fold)**	***C. microphyllum* root-specific (>2 fold)**	**Functional Annotation**			
					**NCBI nr IDs**	**TFs**	**KOG**	**KEGG**
XLOC_012274	No	No	DEG(I+M)	No	XP_004508056.1	--	--	--
XLOC_012276	No	No	No	No	XP_004514915.1	--	--	--
XLOC_012284	No	No	No	No	KEP46352.1	--	--	--
XLOC_012285	No	No	No	No	KEP46352.1	--	--	--
XLOC_012286	DEG(M)	No	No	No	KEP46352.1	--	--	--
XLOC_012290	DEG(M)	No	No	No	Q9UR38.1	Trihelix	G	K00134
XLOC_012291	DEG(M)	No	DEG(I+M)	DEG(M)	AAR27328.1	Trihelix	G	--
XLOC_012293	No	No	No	No	XP_011493026.1	Trihelix	G	--
XLOC_012311	No	No	No	No	--	--	--	--
XLOC_012312	DEG(M)	No	No	No	--	--	--	--
XLOC_012313	DEG(M)	No	No	No	EUC57052.1	--	--	--
XLOC_012314	DEG(M)	No	No	No	CEL60642.1	--	--	--
XLOC_012315	DEG(M)	No	No	No	CEL60642.1	--	--	--
XLOC_012328	No	No	No	No	--	--	--	--
XLOC_012329	No	No	No	No	--	--	--	--
XLOC_012331	DEG(M)	No	No	No	--	--	--	--
XLOC_012338	DEG(M)	DEG(M)	DEG(I+M)	DEG(M)	XP_004512942.1	--	--	--
XLOC_012339	DEG(M)	No	No	No	--	--	--	--
XLOC_012359	DEG(M)	DEG(M)	DEG(I+M)	DEG(M)	XP_009539701.1	--	--	--
XLOC_012360	DEG(M)	DEG(M)	DEG(I+M)	DEG(M)	XP_009539701.1	--	--	--
XLOC_012377	DEG(M)	No	No	No	AJI44483.1	--	--	--
XLOC_012378	DEG(M)	No	No	No	AJI44483.1	--	--	--
XLOC_012382	DEG(M)	No	No	No	XP_001931554.1	--	--	--
XLOC_012383	DEG(M)	No	No	No	XP_001931554.1	--	--	--
XLOC_012400	No	No	No	No	--	--	--	K06119
XLOC_012402	No	No	DEG(I+M)	No	--	--	--	--
XLOC_012404	No	No	No	No	--	--	--	--
XLOC_012411	DEG(M)	No	No	No	ETI30790.1	--	--	K17279
XLOC_012412	DEG(M)	No	No	No	EUC67164.1	--	--	--
XLOC_012413	No	No	No	No	CCO32154.1	--	--	--
XLOC_012414	No	No	No	No	--	--	--	--
XLOC_012421	No	No	No	No	--	--	J	K02910
XLOC_012422	No	No	No	No	XP_004494779.1	--	X	--
XLOC_012423	No	No	No	No	XP_003039876.1	--	--	--
XLOC_012424	DEG(M)	No	No	No	--	--	A	K02930
XLOC_012425	No	No	No	No	--	--	O	K03036
XLOC_012426	DEG(M)	DEG(M)	No	No	--	--	--	--
XLOC_012429	DEG(M)	No	No	No	CDS82489.1	--	--	--
XLOC_012433	No	No	No	No	XP_004516219.1	--	--	--
XLOC_012437	No	No	No	No	CEL62439.1	--	--	K00327

Differentially expressed gene (DEG) IDs	*C. microphyllum* root and shoot-specific (>2 fold)	*C. microphyllum* root and shoot-specific (>10 fold)	ICC4958 and *C. microphyllum* root-specific (>2 fold)	*C. microphyllum* root-specific (>2 fold)	Functional Annotation			
					NCBI nr IDs	TFs	KOG	KEGG
XLOC_012438	DEG(M)	DEG(M)	DEG(I+M)	DEG(M)	AAD50974.1	--	P	K03781
XLOC_012439	DEG(M)	No	No	No	--	--	--	--
XLOC_012440	No	No	No	No	--	--	--	--
XLOC_012442	No	No	No	No	--	--	--	--
XLOC_012443	No	No	No	No	--	--	--	--
XLOC_012448	No	No	No	No	--	--	--	--
XLOC_012450	No	No	No	No	--	--	--	--
XLOC_012452	No	No	No	No	--	--	J	--
XLOC_012453	No	No	No	No	NP_001156420.1	--	O	--
XLOC_012454	No	No	No	No	--	--	--	--
XLOC_012455	DEG(M)	No	No	No	XP_002901699.1	--	J	--
XLOC_012464	No	No	No	No	--	--	--	--
XLOC_012465	No	No	No	No	EUC62365.1	--	--	--
XLOC_012468	No	No	No	No	--	--	--	--
XLOC_012470	No	No	No	No	XP_001647937.1	--	C	--
XLOC_012472	No	No	No	No	CEL57447.1	--	FE	K00948
XLOC_012474	No	No	No	No	--	--	--	--
XLOC_012476	No	No	No	No	XP_006429758.1	--	TO	--
XLOC_012482	No	No	No	No	NP_001156420.1	--	O	--
XLOC_012483	No	No	No	No	XP_008187830.1	--	--	--
XLOC_012489	No	No	DEG(I+M)	No	--	AP2	--	K09285
XLOC_012490	No	No	No	No	KEP50687.1	--	--	--
XLOC_012496	No	No	No	No	KNG45039.1	--	--	K03320
XLOC_012497	No	No	No	No	--	--	--	--
XLOC_012500	No	No	No	No	--	--	--	--
XLOC_012502	No	No	No	No	--	G2-like	RK	--
XLOC_012503	No	No	No	No	--	--	--	--
XLOC_012504	No	No	No	No	--	--	--	--
XLOC_012508	No	No	No	No	CEL57966.1	--	--	--
XLOC_012509	No	No	No	No	KDN50102.1	--	--	--
XLOC_012512	DEG(M)	DEG(M)	DEG(I+M)	DEG(M)	XP_001829580.1	--	J	--
XLOC_012513	No	No	No	No	KIO27786.1	--	O	--
XLOC_012514	DEG(M)	No	No	No	BAH70706.1	--	J	--
XLOC_012515	DEG(M)	No	No	No	AAY66882.1	--	J	K02975
XLOC_012516	No	No	No	No	XP_008901486.1	--	--	K08762
XLOC_012518	DEG(M)	No	DEG(I+M)	DEG(M)	EUC64523.1	--	--	--
XLOC_012519	No	No	No	No	--	--	--	--
XLOC_012521	DEG(M)	No	No	No	--	--	J	K02898
XLOC_012524	DEG(M)	No	No	No	--	--	--	--
XLOC_012536	No	No	DEG(I+M)	No	--	--	--	--

Differentially expressed gene (DEG) IDs	*C. microphyllum* root and shoot-specific (>2 fold)	*C. microphyllum* root and shoot-specific (>10 fold)	ICC4958 and *C. microphyllum* root-specific (>2 fold)	*C. microphyllum* root-specific (>2 fold)	Functional Annotation			
					NCBI nr IDs	TFs	KOG	KEGG
XLOC_012537	No	No	DEG(I+M)	No	XP_004503763.1	--	--	--
XLOC_012538	No	No	No	No	EMT70484.1	--	--	--
XLOC_012546	No	No	No	No	ETI30056.1	--	--	--
XLOC_012548	DEG(M)	No	No	No	CCO32380.1	--	--	--
XLOC_012551	No	No	No	No	EUC65263.1	--	G	--
XLOC_012552	DEG(M)	DEG(M)	No	No	XP_004504254.1	--	--	--
XLOC_012555	No	No	No	No	CCO37451.1	--	C	--
XLOC_012556	No	No	No	No	CEL58994.1	--	--	--
XLOC_012557	No	No	No	No	--	--	--	--
XLOC_012559	DEG(M)	DEG(M)	No	No	EXM09116.1	--	G	--
XLOC_012561	No	No	No	No	--	--	RT	K13947
XLOC_012563	No	No	No	No	--	--	--	--
XLOC_012565	No	No	No	No	XP_001946940.1	--	--	--
XLOC_012566	No	No	No	No	--	--	--	K01874
XLOC_012573	DEG(M)	No	No	No	--	--	--	--
XLOC_012575	DEG(M)	No	DEG(I+M)	DEG(M)	KEP49081.1	--	--	--
XLOC_012579	No	No	No	No	CEL57981.1	--	--	--
XLOC_012583	No	No	No	No	--	--	--	--
XLOC_012586	No	No	No	No	XP_002533345.1	--	X	--
XLOC_012589	No	No	No	No	XP_003052799.1	--	--	--
XLOC_012591	No	No	No	No	--	--	--	--
XLOC_012592	No	No	No	No	KEP50845.1	--	--	--
XLOC_012593	No	No	No	No	AKM28418.1	--	I	K11262
XLOC_012594	No	No	No	No	--	--	--	--
XLOC_012597	No	No	No	No	CEL52222.1	--	C	--
XLOC_012604	No	No	No	No	ETK79569.1	--	--	--
XLOC_012605	No	No	No	No	ABV82736.1	--	--	--
XLOC_012607	DEG(M)	No	No	No	--	--	--	--
XLOC_012609	DEG(M)	No	No	No	CEL52251.1	--	--	--
XLOC_012615	No	No	No	No	--	--	--	--
XLOC_012616	No	No	No	No	--	--	--	--
XLOC_012619	DEG(M)	No	DEG(I+M)	DEG(M)	XP_004493901.1	C2H2	--	--
XLOC_012621	No	No	No	No	--	--	H	K15746
XLOC_012625	No	No	No	No	EUC65354.1	--	G	--
XLOC_012630	DEG(M)	DEG(M)	DEG(I+M)	DEG(M)	--	--	--	--
XLOC_012636	No	No	No	No	--	--	--	--
XLOC_012637	DEG(M)	DEG(M)	No	No	--	--	--	--
XLOC_012638	No	No	No	No	--	--	--	--
XLOC_012639	DEG(M)	DEG(M)	No	No	EGU88773.1	--	--	--
XLOC_012643	No	No	DEG(I+M)	No	XP_004510579.1	--	R	--

Differentially expressed gene (DEG) IDs	*C. microphyllum* root and shoot-specific (>2 fold)	*C. microphyllum* root and shoot-specific (>10 fold)	ICC4958 and *C. microphyllum* root-specific (>2 fold)	*C. microphyllum* root-specific (>2 fold)	Functional Annotation			
					NCBI nr IDs	TFs	KOG	KEGG
XLOC_012645	No	No	No	No	ETI51423.1	--	--	--
XLOC_012653	No	No	No	No	--	--	--	--
XLOC_012654	DEG(M)	No	No	No	--	--	--	--
XLOC_012663	No	No	No	No	--	--	BL	--
XLOC_012666	No	No	No	No	KEP47392.1	--	--	--
XLOC_012667	DEG(M)	No	No	No	--	--	--	--
XLOC_012668	DEG(M)	No	No	No	--	--	--	--
XLOC_012674	No	No	No	No	--	YABBY	--	--
XLOC_012675	No	No	No	No	XP_012572822.1	YABBY	--	--
XLOC_012680	No	No	No	No	XP_008893578.1	--	--	--
XLOC_012682	No	No	No	No	--	--	--	K15404
XLOC_012683	No	No	No	No	--	--	I	--
XLOC_012689	DEG(M)	DEG(M)	DEG(I+M)	DEG(M)	EUC55575.1	--	--	--
XLOC_012695	No	No	DEG(I+M)	No	--	--	--	--
XLOC_012697	DEG(M)	DEG(M)	No	No	--	--	--	--
XLOC_012698	DEG(M)	No	No	No	--	--	--	--
XLOC_012699	DEG(M)	No	No	No	KEP50571.1	--	--	--
XLOC_012700	DEG(M)	No	No	No	EUC58530.1	--	--	--
XLOC_012714	No	No	No	No	EUC55551.1	--	--	--
XLOC_012721	No	No	DEG(I+M)	No	--	--	--	--
XLOC_012726	No	No	DEG(I+M)	No	--	ERF	--	--
XLOC_012729	DEG(M)	DEG(M)	No	No	--	--	--	--
XLOC_012740	DEG(M)	No	DEG(I+M)	DEG(M)	XP_004506818.1	--	--	--
XLOC_012741	DEG(M)	DEG(M)	DEG(I+M)	DEG(M)	XP_004506819.1	--	--	--
XLOC_012742	No	No	No	No	--	--	--	--
XLOC_012752	No	No	No	No	XP_004514927.1	--	--	--
XLOC_012754	DEG(M)	No	No	No	XP_003053974.1	--	--	--
XLOC_012755	DEG(M)	No	No	No	--	--	--	--
XLOC_012756	No	No	No	No	--	--	--	--
XLOC_012758	DEG(M)	No	DEG(I+M)	DEG(M)	ELU44851.1	--	--	K01637
XLOC_012765	No	No	No	No	XP_004516108.1	--	F	--
XLOC_012776	DEG(M)	DEG(M)	DEG(I+M)	DEG(M)	--	--	--	--
XLOC_012777	No	No	No	No	--	--	--	--
XLOC_012780	No	No	No	No	KDN37710.1	--	--	--
XLOC_012781	DEG(M)	No	No	No	KDN37710.1	--	--	--
XLOC_012782	No	No	No	No	KDN37710.1	--	--	--
XLOC_012783	DEG(M)	No	No	No	KDN37710.1	--	--	--
XLOC_012784	DEG(M)	No	No	No	--	--	J	--
XLOC_012785	DEG(M)	DEG(M)	DEG(I+M)	DEG(M)	EUC65367.1	--	--	--
XLOC_012787	No	No	No	No	CCO27311.1	--	J	K02984

Differentially expressed gene (DEG) IDs	*C. microphyllum* root and shoot-specific (>2 fold)	*C. microphyllum* root and shoot-specific (>10 fold)	ICC4958 and *C. microphyllum* root-specific (>2 fold)	*C. microphyllum* root-specific (>2 fold)	Functional Annotation			
					NCBI nr IDs	TFs	KOG	KEGG
XLOC_012795	No	No	DEG(I+M)	No	--	--	--	--
XLOC_012796	DEG(M)	DEG(M)	DEG(I+M)	DEG(M)	XP_002525427.1	--	E	K00261
XLOC_012798	DEG(M)	No	No	No	--	--	--	--
XLOC_012801	No	No	No	No	XP_004487511.1	--	--	K13356
XLOC_012802	DEG(M)	No	No	No	EUC63332.1	--	--	K01187
XLOC_012807	No	No	No	No	--	--	--	--
XLOC_012810	DEG(M)	No	DEG(I+M)	DEG(M)	XP_004489073.1	WRKY	--	--
XLOC_012827	No	No	No	No	XP_004494482.1	--	--	K03768
XLOC_012828	No	No	No	No	--	--	--	K18551
XLOC_012829	No	No	No	No	KEP51752.1	--	--	--
XLOC_012839	No	No	No	No	--	TCP	--	--
XLOC_012842	DEG(M)	No	No	No	CCO27426.1	--	--	--
XLOC_012843	No	No	No	No	CCO27426.1	--	--	--
XLOC_012848	DEG(M)	No	No	No	--	--	--	--
XLOC_012849	No	No	No	No	XP_001950495.1	--	I	K08762
XLOC_012851	No	No	No	No	--	--	--	--
XLOC_012856	No	No	DEG(I+M)	No	--	--	--	--
XLOC_012857	DEG(M)	No	No	No	CCO30484.1	--	--	--
XLOC_012859	No	No	No	No	--	--	--	--
XLOC_012860	No	No	No	No	KEP54954.1	--	--	--
XLOC_012865	No	No	No	No	AHZ64881.2	--	C	K02136
XLOC_012866	No	No	No	No	XP_002997335.1	--	--	K11583
XLOC_012870	No	No	No	No	XP_008889631.1	--	--	--
XLOC_012871	No	No	No	No	XP_001650309.1	--	C	--
XLOC_012881	No	No	No	No	EUC62213.1	--	--	K00999
XLOC_012883	No	No	No	No	--	--	--	--
XLOC_012887	No	No	No	No	KDN50079.1	--	--	--
XLOC_012891	No	No	No	No	--	--	--	--
XLOC_012892	DEG(M)	No	DEG(I+M)	DEG(M)	XP_004508369.1	--	--	K10760
XLOC_012893	No	No	No	No	--	--	--	--
XLOC_012895	DEG(M)	No	No	No	EUC63230.1	--	--	--
XLOC_012898	No	No	No	No	--	TCP	--	--
XLOC_012899	DEG(M)	No	No	No	NP_001155681.1	--	J	--
XLOC_012900	No	No	No	No	--	--	O	--
XLOC_012901	No	No	No	No	EUC63762.1	--	--	K01869
XLOC_012907	DEG(M)	No	No	No	CCO29620.1	--	--	--
XLOC_012908	No	No	No	No	--	--	--	--
XLOC_012909	No	No	No	No	NP_001156726.1	--	C	--
XLOC_012911	No	No	No	No	--	--	--	--
XLOC_012913	No	No	No	No	--	--	R	--

Differentially expressed gene (DEG) IDs	*C. microphyllum* root and shoot-specific (>2 fold)	*C. microphyllum* root and shoot-specific (>10 fold)	ICC4958 and *C. microphyllum* root-specific (>2 fold)	*C. microphyllum* root-specific (>2 fold)	Functional Annotation			
					NCBI nr IDs	TFs	KOG	KEGG
XLOC_012916	No	No	No	No	XP_008895917.1	--	O	K02729
XLOC_012918	No	No	No	No	EUC64411.1	--	--	--
XLOC_012922	DEG(M)	DEG(M)	DEG(I+M)	DEG(M)	--	--	--	--
XLOC_012925	DEG(M)	No	No	No	CDR41652.1	YABBY	J	--
XLOC_012930	No	No	No	No	EUC66324.1	--	--	--
XLOC_012933	No	No	No	No	NP_001165739.1	--	--	--
XLOC_012934	DEG(M)	No	No	No	XP_012570780.1	--	--	--
XLOC_012939	No	No	No	No	--	--	--	K11097
XLOC_012942	No	No	No	No	--	--	--	--
XLOC_012947	No	No	No	No	--	--	--	--
XLOC_012949	No	No	No	No	--	--	--	--
XLOC_012951	DEG(M)	No	No	No	--	--	--	--
XLOC_012952	DEG(M)	No	No	No	--	--	--	--
XLOC_012953	No	No	No	No	--	--	--	--
XLOC_012954	No	No	No	No	--	--	--	--
XLOC_012956	DEG(M)	DEG(M)	No	No	EXM11528.1	--	--	--
XLOC_012957	No	No	No	No	--	--	--	--
XLOC_012959	No	No	No	No	--	--	--	--
XLOC_012969	No	No	No	No	--	--	--	--
XLOC_012970	No	No	No	No	XP_006492964.1	--	F	K01937
XLOC_012973	No	No	No	No	EMT74528.1	--	--	--
XLOC_012980	No	No	No	No	XP_003050828.1	--	--	--
XLOC_012983	No	No	No	No	XP_003053174.1	--	--	--
XLOC_012986	No	No	No	No	XP_001846672.1	--	--	--
XLOC_012988	No	No	No	No	--	--	--	--
XLOC_012989	No	No	No	No	KDE02973.1	--	J	--
XLOC_012997	DEG(M)	No	No	No	--	--	--	--
XLOC_013002	No	No	No	No	--	--	--	--
XLOC_013003	No	No	No	No	KDN33412.1	--	--	--
XLOC_013004	No	No	No	No	--	--	--	--
XLOC_013008	No	No	No	No	--	--	--	--
XLOC_013010	No	No	No	No	--	--	--	--
XLOC_013011	No	No	No	No	--	--	--	--
XLOC_013014	No	No	No	No	ETL88446.1	--	--	--
XLOC_013015	No	No	No	No	--	--	--	--
XLOC_013017	No	No	No	No	--	--	--	--
XLOC_013019	No	No	No	No	--	--	--	--
XLOC_013023	No	No	No	No	XP_012069970.1	--	U	--
XLOC_013024	No	No	No	No	XP_001654716.1	--	--	--
XLOC_013029	DEG(M)	No	No	No	--	--	--	--

Differentially expressed gene (DEG) IDs	*C. microphyllum* root and shoot-specific (>2 fold)	*C. microphyllum* root and shoot-specific (>10 fold)	ICC4958 and *C. microphyllum* root-specific (>2 fold)	*C. microphyllum* root-specific (>2 fold)	Functional Annotation			
					NCBI nr IDs	TFs	KOG	KEGG
XLOC_013031	No	No	No	No	--	--	--	K12392
XLOC_013034	No	No	No	No	--	--	--	--
XLOC_013036	No	No	No	No	NP_001128376.1	--	--	--
XLOC_013039	No	No	No	No	EUC64476.1	--	--	--
XLOC_013043	No	No	No	No	XP_003048459.1	--	--	--
XLOC_013044	No	No	No	No	XP_001656628.1	--	C	--
XLOC_013046	DEG(M)	No	No	No	--	--	J	--
XLOC_013047	No	No	No	No	ETL50425.1	--	--	K01438
XLOC_013048	DEG(M)	No	No	No	--	--	--	--
XLOC_013049	No	No	No	No	--	--	--	--
XLOC_013051	No	No	No	No	--	--	YU	K18752
XLOC_013053	No	No	No	No	--	--	J	--
XLOC_013054	No	No	No	No	--	--	--	--
XLOC_013057	DEG(M)	No	No	No	--	--	--	--
XLOC_013058	No	No	No	No	--	--	--	--
XLOC_013059	No	No	No	No	XP_004494758.1	--	RI	--
XLOC_013060	No	No	No	No	--	--	--	--
XLOC_013063	No	No	No	No	XP_001654813.1	--	--	--
XLOC_013067	No	No	No	No	EUC54935.1	--	--	--
XLOC_013070	No	No	No	No	XP_001650061.1	--	--	K10863
XLOC_013077	No	No	No	No	--	--	--	K03873
XLOC_013078	DEG(M)	No	DEG(I+M)	DEG(M)	--	--	--	--
XLOC_013079	No	No	No	No	--	--	--	--
XLOC_013080	No	No	No	No	--	--	--	K15040
XLOC_013085	No	No	No	No	CEL60432.1	--	--	--
XLOC_013091	DEG(M)	No	No	No	KDN40640.1	--	--	--
XLOC_013094	No	No	No	No	--	--	--	--
XLOC_013098	No	No	No	No	--	--	J	K02883
XLOC_013099	DEG(M)	No	No	No	--	--	--	--
XLOC_013100	DEG(M)	No	No	No	--	--	--	--
XLOC_013101	No	No	No	No	--	--	--	--
XLOC_013102	No	No	DEG(I+M)	No	XP_004510565.1	--	--	--
XLOC_013103	DEG(M)	No	DEG(I+M)	DEG(M)	--	--	--	--
XLOC_013105	No	No	No	No	KNG46954.1	--	--	--
XLOC_013114	No	No	No	No	KEP50648.1	--	--	--
XLOC_013116	No	No	No	No	--	--	--	--
XLOC_013121	No	No	No	No	--	--	R	--
XLOC_013123	No	No	No	No	--	--	--	--
XLOC_013132	No	No	No	No	--	--	--	--
XLOC_013133	No	No	No	No	--	--	--	K10839

Differentially expressed gene (DEG) IDs	*C. microphyllum* root and shoot-specific (>2 fold)	*C. microphyllum* root and shoot-specific (>10 fold)	ICC4958 and *C. microphyllum* root-specific (>2 fold)	*C. microphyllum* root-specific (>2 fold)	Functional Annotation			
					NCBI nr IDs	TFs	KOG	KEGG
XLOC_013136	No	No	No	No	--	--	--	--
XLOC_013137	No	No	No	No	CCO30886.1	--	--	--
XLOC_013139	No	No	No	No	--	--	--	--
XLOC_013142	DEG(M)	No	No	No	CEL52207.1	--	--	--
XLOC_013147	No	No	No	No	KNB15329.1	--	G	--
XLOC_013148	No	No	No	No	--	--	--	--
XLOC_013149	No	No	No	No	--	--	T	--
XLOC_013151	No	No	No	No	KEP48887.1	--	--	--
XLOC_013155	No	No	No	No	XP_003054080.1	--	--	--
XLOC_013157	No	No	No	No	--	--	--	--
XLOC_013158	No	No	No	No	DAA05871.1	--	J	--
XLOC_013160	No	No	No	No	ETL80552.1	--	--	--
XLOC_013163	DEG(M)	No	DEG(I+M)	DEG(M)	KHG18124.1	MYB_related	O	--
XLOC_013164	No	No	No	No	XP_008179393.1	--	--	--
XLOC_013165	No	No	No	No	XP_003047537.1	--	--	--
XLOC_013166	No	No	No	No	CCO27536.1	--	--	--
XLOC_013168	No	No	No	No	EMD86958.1	--	--	--
XLOC_013169	No	No	No	No	KDN46011.1	--	--	K03938
XLOC_013174	No	No	No	No	--	--	--	--
XLOC_013180	No	No	No	No	--	--	O	--
XLOC_013185	No	No	No	No	--	--	--	--
XLOC_013189	DEG(M)	No	No	No	--	--	--	--
XLOC_013191	DEG(M)	No	DEG(I+M)	DEG(M)	CEL60367.1	--	--	--
XLOC_013192	DEG(M)	DEG(M)	DEG(I+M)	DEG(M)	CEL60367.1	--	--	--
XLOC_013193	No	No	No	No	EUC62178.1	--	--	K08486
XLOC_013194	No	No	No	No	EUC62178.1	--	--	--
XLOC_013197	DEG(M)	No	No	No	--	--	--	--
XLOC_013198	DEG(M)	No	No	No	--	--	--	--
XLOC_013199	No	No	DEG(I+M)	No	CAA66109.3	--	--	--
XLOC_013205	No	No	DEG(I+M)	No	--	WRKY	--	--
XLOC_013206	No	No	No	No	--	--	--	--
XLOC_013209	DEG(M)	No	No	No	--	--	--	K03113
XLOC_013211	No	No	DEG(I+M)	No	--	--	T	--
XLOC_013212	No	No	No	No	EUC54083.1	--	--	--
XLOC_013215	DEG(M)	No	DEG(I+M)	DEG(M)	ETM37694.1	--	--	K02934
XLOC_013223	DEG(M)	DEG(M)	DEG(I+M)	DEG(M)	--	--	--	--
XLOC_013228	No	No	No	No	--	--	--	--
XLOC_013229	No	No	No	No	--	--	--	--
XLOC_013231	No	No	No	No	--	--	--	--
XLOC_013233	No	No	DEG(I+M)	No	--	--	--	--

Differentially expressed gene (DEG) IDs	*C. microphyllum* root and shoot-specific (>2 fold)	*C. microphyllum* root and shoot-specific (>10 fold)	ICC4958 and *C. microphyllum* root-specific (>2 fold)	*C. microphyllum* root-specific (>2 fold)	Functional Annotation			
					NCBI nr IDs	TFs	KOG	KEGG
XLOC_013240	DEG(M)	No	No	No	--	--	--	--
XLOC_013243	No	No	No	No	ETI41741.1	--	--	--
XLOC_013253	No	No	No	No	XP_003043393.1	--	--	--
XLOC_013254	DEG(M)	No	No	No	--	--	--	--
XLOC_013258	No	No	No	No	--	--	--	--
XLOC_013259	No	No	No	No	--	--	--	--
XLOC_013260	No	No	No	No	--	--	--	--
XLOC_013262	No	No	No	No	--	--	--	K15015
XLOC_013263	DEG(M)	No	No	No	EUC64359.1	--	J	--
XLOC_013265	DEG(M)	No	No	No	--	--	DZ	--
XLOC_013266	DEG(M)	No	No	No	--	--	--	--
XLOC_013269	No	No	No	No	--	--	--	--
XLOC_013271	DEG(M)	DEG(M)	No	No	XP_003045590.1	--	--	--
XLOC_013273	DEG(M)	No	No	No	EMT64242.1	--	J	--
XLOC_013274	DEG(M)	No	No	No	KDN50275.1	--	J	K02993
XLOC_013275	DEG(M)	No	No	No	KDN50275.1	--	J	--
XLOC_013276	DEG(M)	No	No	No	KDN50275.1	--	J	K02993
XLOC_013283	No	No	No	No	--	--	--	--
XLOC_013288	No	No	No	No	--	--	--	--
XLOC_013290	No	No	DEG(I+M)	No	--	--	I	--
XLOC_013294	No	No	No	No	--	--	--	--
XLOC_013296	No	No	No	No	--	--	--	--
XLOC_013300	DEG(M)	DEG(M)	DEG(I+M)	DEG(M)	XP_006965400.1	--	--	--
XLOC_013304	DEG(M)	No	No	No	--	bHLH	C	--
XLOC_013305	DEG(M)	No	No	No	--	--	--	--
XLOC_013306	No	No	DEG(I+M)	No	XP_004504507.1	C2H2	--	--
XLOC_013308	No	No	No	No	CCO28084.1	--	U	--
XLOC_013310	DEG(M)	No	No	No	XP_007846113.1	--	U	--
XLOC_013316	DEG(M)	DEG(M)	No	No	--	--	--	--
XLOC_013317	DEG(M)	DEG(M)	DEG(I+M)	DEG(M)	KDN49348.1	--	--	--
XLOC_013320	No	No	No	No	CCO29697.1	--	--	--
XLOC_013321	No	No	No	No	CCO29697.1	--	--	--
XLOC_013325	DEG(M)	DEG(M)	DEG(I+M)	DEG(M)	XP_008905063.1	--	J	--
XLOC_013326	DEG(M)	DEG(M)	DEG(I+M)	DEG(M)	XP_009518381.1	--	J	--
XLOC_013332	DEG(M)	No	No	No	CCO30802.1	--	--	--
XLOC_013333	No	No	No	No	XP_003049898.1	--	--	--
XLOC_013335	No	No	No	No	--	--	--	--
XLOC_013351	No	No	No	No	--	--	--	--
XLOC_013362	DEG(M)	DEG(M)	No	No	CEL57048.1	--	--	--
XLOC_013363	DEG(M)	DEG(M)	DEG(I+M)	DEG(M)	CEL57048.1	--	--	--

Differentially expressed gene (DEG) IDs	*C. microphyllum* root and shoot-specific (>2 fold)	*C. microphyllum* root and shoot-specific (>10 fold)	ICC4958 and *C. microphyllum* root-specific (>2 fold)	*C. microphyllum* root-specific (>2 fold)	Functional Annotation			
					NCBI nr IDs	TFs	KOG	KEGG
XLOC_013365	No	No	No	No	CCO37999.1	--	--	--
XLOC_013366	DEG(M)	DEG(M)	DEG(I+M)	DEG(M)	XP_008898328.1	--	J	--
XLOC_013370	DEG(M)	DEG(M)	DEG(I+M)	DEG(M)	XP_008610312.1	--	J	--
XLOC_013372	DEG(M)	No	No	No	AAW56830.1	--	T	--
XLOC_013373	DEG(M)	No	No	No	AAW56830.1	--	T	--
XLOC_013377	No	No	No	No	--	--	--	--
XLOC_013378	No	No	No	No	EUC65127.1	--	--	--
XLOC_013383	DEG(M)	No	No	No	--	--	--	--
XLOC_013404	DEG(M)	No	No	No	NP_001119620.1	--	O	K09503
XLOC_013408	DEG(M)	No	No	No	--	--	J	--
XLOC_013409	No	No	No	No	--	--	--	--
XLOC_013412	No	No	No	No	CEO97530.1	--	J	K02953
XLOC_013413	No	No	No	No	CEO97530.1	--	J	K02953
XLOC_013414	No	No	No	No	ETI45264.1	--	--	--
XLOC_013416	No	No	No	No	ETP01296.1	--	--	--
XLOC_013418	No	No	No	No	--	--	--	--
XLOC_013419	No	No	No	No	BAN20547.1	--	C	K02145
XLOC_013420	No	No	No	No	EUC65371.1	--	--	--
XLOC_013422	No	No	No	No	--	--	--	K02695
XLOC_013426	No	No	No	No	--	--	I	--
XLOC_013428	No	No	No	No	--	--	--	--
XLOC_013429	No	No	No	No	--	--	--	--
XLOC_013449	No	No	No	No	--	--	--	--
XLOC_013452	DEG(M)	No	No	No	--	--	--	K00477
XLOC_013453	DEG(M)	No	No	No	--	--	--	--
XLOC_013454	No	No	No	No	--	--	--	--
XLOC_013457	No	No	No	No	--	--	--	--
XLOC_013459	DEG(M)	No	No	No	NP_001119694.1	YABBY	J	--
XLOC_013461	No	No	No	No	--	--	M	--
XLOC_013462	No	No	No	No	ETL91065.1	--	--	--
XLOC_013463	No	No	No	No	XP_008908027.1	--	--	--
XLOC_013465	No	No	No	No	ETP17978.1	--	--	K17987
XLOC_013466	No	No	No	No	--	--	--	--
XLOC_013474	No	No	No	No	CCO27996.1	--	--	--
XLOC_013475	No	No	No	No	--	--	--	--
XLOC_013479	No	No	No	No	XP_001946255.2	--	R	--
XLOC_013482	No	No	No	No	--	--	--	--
XLOC_013484	DEG(M)	No	No	No	--	--	--	--
XLOC_013489	No	No	No	No	XP_001652180.1	--	--	--
XLOC_013490	No	No	No	No	CEJ62500.1	--	--	--

Differentially expressed gene (DEG) IDs	*C. microphyllum* root and shoot-specific (>2 fold)	*C. microphyllum* root and shoot-specific (>10 fold)	ICC4958 and *C. microphyllum* root-specific (>2 fold)	*C. microphyllum* root-specific (>2 fold)	Functional Annotation			
					NCBI nr IDs	TFs	KOG	KEGG
XLOC_013497	No	No	No	No	XP_001951777.1	--	B	--
XLOC_013499	DEG(M)	DEG(M)	No	No	EMT73802.1	--	--	--
XLOC_013508	No	No	No	No	--	--	--	--
XLOC_013509	No	No	No	No	NP_001155554.1	--	R	K14842
XLOC_013510	No	No	No	No	--	--	--	--
XLOC_013519	DEG(M)	No	No	No	--	--	--	--
XLOC_013521	No	No	No	No	XP_001949085.1	--	--	--
XLOC_013523	No	No	No	No	XP_004495294.1	bHLH	--	--
XLOC_013529	DEG(M)	No	No	No	--	--	--	--
XLOC_013544	No	No	No	No	--	--	--	--
XLOC_013556	No	No	No	No	--	--	--	--
XLOC_013558	DEG(M)	No	No	No	--	--	O	--
XLOC_013560	No	No	No	No	CEL64056.1	--	--	--
XLOC_013561	DEG(M)	No	No	No	KEP46566.1	--	--	--
XLOC_013567	No	No	No	No	EUC63385.1	--	--	--
XLOC_013568	No	No	No	No	--	--	--	--
XLOC_013569	No	No	No	No	--	--	--	--
XLOC_013587	DEG(M)	DEG(M)	DEG(I+M)	DEG(M)	XP_008870185.1	--	--	K02940
XLOC_013597	DEG(M)	DEG(M)	No	No	--	--	--	--
XLOC_013598	DEG(M)	No	No	No	--	--	--	--
XLOC_013602	DEG(M)	DEG(M)	No	No	--	--	--	--
XLOC_013603	No	No	No	No	--	--	--	--
XLOC_013611	No	No	No	No	--	--	--	--
XLOC_013612	DEG(M)	No	DEG(I+M)	DEG(M)	XP_004515103.1	--	--	K00430
XLOC_013613	DEG(M)	No	No	No	--	--	--	--
XLOC_013614	No	No	No	No	--	--	--	--
XLOC_013617	No	No	No	No	XP_003024736.1	--	Z	K08341
XLOC_013626	DEG(M)	No	No	No	--	--	C	--
XLOC_013627	DEG(M)	DEG(M)	DEG(I+M)	DEG(M)	--	--	--	--
XLOC_013632	No	No	No	No	--	--	--	--
XLOC_013637	No	No	No	No	CEL52299.1	--	--	--
XLOC_013641	DEG(M)	No	No	No	KEP46235.1	--	--	--
XLOC_013642	No	No	No	No	KEP46235.1	--	--	--
XLOC_013643	DEG(M)	No	No	No	KDN38337.1	--	--	--
XLOC_013647	DEG(M)	No	No	No	--	--	--	--
XLOC_013648	No	No	No	No	--	--	--	--
XLOC_013649	No	No	No	No	KEP50675.1	--	--	--
XLOC_013651	No	No	No	No	--	--	--	--
XLOC_013655	DEG(M)	No	No	No	NP_001280441.1	--	--	--
XLOC_013656	No	No	No	No	--	--	--	--

Differentially expressed gene (DEG) IDs	*C. microphyllum* root and shoot-specific (>2 fold)	*C. microphyllum* root and shoot-specific (>10 fold)	ICC4958 and *C. microphyllum* root-specific (>2 fold)	*C. microphyllum* root-specific (>2 fold)	Functional Annotation			
					NCBI nr IDs	TFs	KOG	KEGG
XLOC_013660	DEG(M)	No	No	No	--	--	--	--
XLOC_013661	No	No	No	No	XP_009530605.1	--	--	K03238
XLOC_013669	No	No	DEG(I+M)	No	XP_004495887.1	--	--	K04125
XLOC_013670	No	No	No	No	KEP54214.1	--	--	K00326
XLOC_013675	No	No	No	No	--	--	O	--
XLOC_013687	DEG(M)	No	DEG(I+M)	DEG(M)	--	--	--	--
XLOC_013688	DEG(M)	No	No	No	ETL27348.1	--	--	--
XLOC_013692	No	No	No	No	--	--	--	--
XLOC_013696	No	No	No	No	ETM37883.1	--	--	K11778
XLOC_013700	No	No	No	No	CEL59690.1	--	--	--
XLOC_013717	DEG(M)	No	No	No	--	--	--	K02980
XLOC_013720	DEG(M)	DEG(M)	DEG(I+M)	DEG(M)	EHN78839.1	--	--	--
XLOC_013721	DEG(M)	No	No	No	EHN78839.1	--	--	--
XLOC_013722	DEG(M)	DEG(M)	DEG(I+M)	DEG(M)	GAD81567.1	--	--	--
XLOC_013724	No	No	DEG(I+M)	No	--	--	Q	--
XLOC_013728	No	No	No	No	EMT69059.1	--	--	--
XLOC_013735	No	No	No	No	--	--	--	--
XLOC_013746	DEG(M)	No	No	No	ETI46262.1	--	--	--
XLOC_013747	No	No	No	No	ETO58465.1	--	--	--
XLOC_013748	No	No	No	No	ETP35695.1	--	--	--
XLOC_013764	No	No	No	No	EUC67187.1	--	M	--
XLOC_013774	DEG(M)	No	DEG(I+M)	DEG(M)	--	--	--	--
XLOC_013777	DEG(M)	No	No	No	--	--	--	K02979
XLOC_013794	DEG(M)	DEG(M)	DEG(I+M)	DEG(M)	XP_003042438.1	--	--	--
XLOC_013799	DEG(M)	No	No	No	--	--	--	--
XLOC_013800	DEG(M)	DEG(M)	DEG(I+M)	DEG(M)	XP_008895254.1	--	--	K02873
XLOC_013808	No	No	No	No	--	--	--	--
XLOC_013810	DEG(M)	No	DEG(I+M)	DEG(M)	--	--	--	--
XLOC_013811	No	No	No	No	--	--	--	K09584
XLOC_013812	No	No	No	No	ETP47794.1	--	--	--
XLOC_013813	No	No	No	No	XP_008914616.1	--	--	--
XLOC_013814	No	No	No	No	ETP47794.1	--	O	K09584
XLOC_013816	No	No	No	No	XP_008904756.1	--	--	K17732
XLOC_013817	No	No	No	No	EUC55192.1	--	O	--
XLOC_013823	No	No	No	No	--	--	X	K01246
XLOC_013839	No	No	DEG(I+M)	No	--	--	--	--
XLOC_013851	No	No	No	No	--	--	--	--
XLOC_013852	DEG(M)	No	No	No	--	--	--	K19944
XLOC_013856	No	No	DEG(I+M)	No	--	--	--	--
XLOC_013858	No	No	No	No	XP_003043760.1	--	--	--

Differentially expressed gene (DEG) IDs	*C. microphyllum* root and shoot-specific (>2 fold)	*C. microphyllum* root and shoot-specific (>10 fold)	ICC4958 and *C. microphyllum* root-specific (>2 fold)	*C. microphyllum* root-specific (>2 fold)	Functional Annotation			
					NCBI nr IDs	TFs	KOG	KEGG
XLOC_013862	DEG(M)	No	No	No	--	--	--	K02980
XLOC_013866	No	No	No	No	--	--	--	--
XLOC_013867	DEG(M)	No	No	No	XP_003046751.1	--	--	--
XLOC_013870	DEG(M)	No	No	No	--	--	J	K02883
XLOC_013872	DEG(M)	No	No	No	XP_004497077.1	MYB	--	K09422
XLOC_013884	DEG(M)	DEG(M)	No	No	--	--	--	--
XLOC_013887	No	No	No	No	EUC61422.1	--	--	--
XLOC_013890	No	No	No	No	XP_008915083.1	--	--	--
XLOC_013894	DEG(M)	No	No	No	NP_001155432.1	--	J	--
XLOC_013897	DEG(M)	No	No	No	--	--	--	--
XLOC_013900	DEG(M)	DEG(M)	No	No	--	--	--	--
XLOC_013902	DEG(M)	No	No	No	XP_008563925.1	--	Z	--
XLOC_013903	No	No	No	No	XP_008563925.1	--	Z	--
XLOC_013904	DEG(M)	No	DEG(I+M)	DEG(M)	--	--	Z	--
XLOC_013906	DEG(M)	No	DEG(I+M)	DEG(M)	--	--	Z	--
XLOC_013911	DEG(M)	DEG(M)	No	No	ENH62718.1	--	--	--
XLOC_013912	DEG(M)	No	No	No	XP_003050769.1	--	--	--
XLOC_013917	No	No	No	No	--	--	E	K12524
XLOC_013923	No	No	No	No	EUC65952.1	--	--	--
XLOC_013924	No	No	No	No	EUC65952.1	--	--	--
XLOC_013925	No	No	No	No	--	--	--	--
XLOC_013929	No	No	DEG(I+M)	No	XP_004495688.1	--	--	--
XLOC_013934	No	No	No	No	--	--	--	--
XLOC_013935	No	No	No	No	CEL54137.1	--	--	--
XLOC_013942	DEG(M)	No	No	No	KEP50243.1	--	--	--
XLOC_013943	DEG(M)	No	No	No	--	--	--	--
XLOC_013946	No	No	No	No	--	--	--	--
XLOC_013948	No	No	No	No	ETP48146.1	--	--	--
XLOC_013949	No	No	No	No	--	--	--	--
XLOC_013952	DEG(M)	DEG(M)	No	No	--	--	--	--
XLOC_013954	No	No	No	No	--	--	--	--
XLOC_013957	No	No	No	No	EUC65172.1	--	--	--
XLOC_013970	DEG(M)	DEG(M)	No	No	XP_012574007.1	--	--	K17961
XLOC_013982	No	No	No	No	--	--	--	--
XLOC_013983	No	No	No	No	--	--	--	--
XLOC_013984	No	No	No	No	--	--	--	--
XLOC_013989	No	No	No	No	XP_004498778.1	ERF	K	--
XLOC_014000	DEG(M)	No	No	No	--	--	--	--
XLOC_014001	No	No	No	No	EUC64076.1	--	--	K19729
XLOC_014013	DEG(M)	DEG(M)	DEG(I+M)	DEG(M)	ELU45882.1	--	--	--

Differentially expressed gene (DEG) IDs	*C. microphyllum* root and shoot-specific (>2 fold)	*C. microphyllum* root and shoot-specific (>10 fold)	ICC4958 and *C. microphyllum* root-specific (>2 fold)	*C. microphyllum* root-specific (>2 fold)	Functional Annotation			
					NCBI nr IDs	TFs	KOG	KEGG
XLOC_014014	DEG(M)	No	No	No	ELU45882.1	--	--	--
XLOC_014015	DEG(M)	DEG(M)	DEG(I+M)	DEG(M)	CEL59489.1	--	--	--
XLOC_014016	No	No	DEG(I+M)	No	--	--	--	--
XLOC_014019	No	No	No	No	--	--	--	--
XLOC_014024	No	No	No	No	--	--	M	--
XLOC_014027	DEG(M)	No	No	No	--	--	--	K07213
XLOC_014037	DEG(M)	No	No	No	--	--	--	--
XLOC_014039	DEG(M)	No	No	No	XP_003051431.1	--	--	--
XLOC_014040	DEG(M)	No	No	No	KEP46242.1	--	--	--
XLOC_014041	No	No	No	No	KEP46242.1	--	--	--
XLOC_014042	No	No	No	No	--	--	O	K09580
XLOC_014046	DEG(M)	No	No	No	ELU36030.1	--	--	--
XLOC_014047	DEG(M)	DEG(M)	DEG(I+M)	DEG(M)	ELU36030.1	--	--	--
XLOC_014048	DEG(M)	No	DEG(I+M)	DEG(M)	EUC56251.1	--	--	--
XLOC_014049	DEG(M)	DEG(M)	DEG(I+M)	DEG(M)	CCO30076.1	--	--	--
XLOC_014059	DEG(M)	No	No	No	KDN39184.1	--	--	--
XLOC_014060	DEG(M)	No	No	No	KDN39184.1	--	--	--
XLOC_014064	No	No	DEG(I+M)	No	--	--	--	--
XLOC_014065	DEG(M)	DEG(M)	DEG(I+M)	DEG(M)	XP_004511997.1	--	--	--
XLOC_014066	No	No	DEG(I+M)	No	--	--	--	--
XLOC_014074	DEG(M)	DEG(M)	DEG(I+M)	DEG(M)	XP_004485679.1	--	R	K03809
XLOC_014077	DEG(M)	No	DEG(I+M)	DEG(M)	XP_004485679.1	--	R	--
XLOC_014089	No	No	DEG(I+M)	No	--	bHLH	--	--
XLOC_014092	No	No	No	No	--	--	G	--
XLOC_014102	DEG(M)	No	No	No	--	--	--	--
XLOC_014103	DEG(M)	DEG(M)	DEG(I+M)	DEG(M)	--	--	--	--
XLOC_014104	DEG(M)	DEG(M)	No	No	XP_003612977.1	--	Q	--
XLOC_014118	DEG(M)	No	No	No	--	--	--	--
XLOC_014119	DEG(M)	No	No	No	CCO28518.1	--	--	--
XLOC_014120	DEG(M)	DEG(M)	DEG(I+M)	DEG(M)	CCO36534.1	--	--	--
XLOC_014141	DEG(M)	DEG(M)	DEG(I+M)	DEG(M)	--	--	--	--
XLOC_014144	No	No	No	No	CEL54484.1	--	--	--
XLOC_014145	DEG(M)	No	No	No	EUC65954.1	--	--	--
XLOC_014146	DEG(M)	No	No	No	EUC53682.1	--	--	--
XLOC_014154	DEG(M)	No	No	No	ELU39145.1	--	J	--
XLOC_014155	DEG(M)	No	No	No	CEL52506.1	--	J	--
XLOC_014163	DEG(M)	No	No	No	KEP46004.1	--	J	K02976
XLOC_014166	No	No	No	No	CEF85879.1	--	--	K11254
XLOC_014170	DEG(M)	No	No	No	--	--	--	K11254
XLOC_014171	No	No	No	No	XP_003580388.2	--	--	K11254

Differentially expressed gene (DEG) IDs	*C. microphyllum* root and shoot-specific (>2 fold)	*C. microphyllum* root and shoot-specific (>10 fold)	ICC4958 and *C. microphyllum* root-specific (>2 fold)	*C. microphyllum* root-specific (>2 fold)	Functional Annotation			
					NCBI nr IDs	TFs	KOG	KEGG
XLOC_014172	DEG(M)	No	No	No	EHB12217.1	--	B	K11254
XLOC_014173	No	No	No	No	XP_004336819.1	--	B	K11254
XLOC_014174	DEG(M)	DEG(M)	DEG(I+M)	DEG(M)	KIK54939.1	CO-like	B	--
XLOC_014184	No	No	No	No	EUC60357.1	--	J	--
XLOC_014185	No	No	No	No	EUC60357.1	--	J	--
XLOC_014186	DEG(M)	No	No	No	EUC60357.1	--	J	--
XLOC_014195	DEG(M)	No	No	No	ELU37028.1	--	--	--
XLOC_014196	No	No	No	No	CCO30802.1	--	J	--
XLOC_014197	No	No	No	No	CCO30802.1	--	J	--
XLOC_014198	DEG(M)	DEG(M)	DEG(I+M)	DEG(M)	ELU37028.1	--	J	--
XLOC_014199	DEG(M)	No	No	No	KEP49157.1	--	--	--
XLOC_014200	DEG(M)	No	No	No	KEP49157.1	--	--	--
XLOC_014201	DEG(M)	No	No	No	EUC58347.1	--	--	--
XLOC_014202	No	No	DEG(I+M)	No	XP_004514113.1	--	--	--
XLOC_014205	No	No	No	No	--	--	--	--
XLOC_014211	No	No	No	No	--	--	J	K02912
XLOC_014213	DEG(M)	No	No	No	--	--	--	--
XLOC_014215	No	No	No	No	--	--	--	--
XLOC_014218	No	No	No	No	XP_004509252.1	--	C	K00234
XLOC_014219	No	No	No	No	XP_008188091.1	--	--	--
XLOC_014225	No	No	No	No	--	--	--	--
XLOC_014226	No	No	No	No	EUC61965.1	--	--	--
XLOC_014230	DEG(M)	DEG(M)	No	No	--	--	--	--
XLOC_014243	No	No	No	No	XP_009251971.1	--	--	--
XLOC_014246	No	No	DEG(I+M)	No	XP_004498840.1	WRKY	T	--
XLOC_014258	No	No	No	No	XP_012065212.1	--	R	K01069
XLOC_014259	No	No	No	No	XP_008891931.1	--	--	--
XLOC_014260	No	No	No	No	--	--	--	--
XLOC_014261	DEG(M)	DEG(M)	No	No	--	--	--	--
XLOC_014265	DEG(M)	No	No	No	XP_008895171.1	--	--	K03676
XLOC_014267	No	No	No	No	--	--	--	K00020
XLOC_014269	No	No	No	No	NP_001156258.1	--	--	--
XLOC_014273	No	No	No	No	--	--	--	--
XLOC_014275	No	No	No	No	XP_004507183.1	--	--	K00799
XLOC_014276	No	No	No	No	XP_004491325.1	HD-ZIP	M	K00696
XLOC_014289	No	No	No	No	ACR46937.1	--	Z	--
XLOC_014290	DEG(M)	No	No	No	AEW07366.1	--	Z	--
XLOC_014292	DEG(M)	DEG(M)	DEG(I+M)	DEG(M)	AEN94509.1	--	Z	--
XLOC_014293	DEG(M)	DEG(M)	DEG(I+M)	DEG(M)	AFX84510.1	--	Z	--
XLOC_014295	DEG(M)	No	No	No	ABP73251.1	--	Z	--

Differentially expressed gene (DEG) IDs	*C. microphyllum* root and shoot-specific (>2 fold)	*C. microphyllum* root and shoot-specific (>10 fold)	ICC4958 and *C. microphyllum* root-specific (>2 fold)	*C. microphyllum* root-specific (>2 fold)	Functional Annotation			
					NCBI nr IDs	TFs	KOG	KEGG
XLOC_014296	DEG(M)	DEG(M)	DEG(I+M)	DEG(M)	AHA50435.1	--	Z	--
XLOC_014297	DEG(M)	DEG(M)	DEG(I+M)	DEG(M)	AEM45650.1	--	Z	--
XLOC_014298	DEG(M)	DEG(M)	DEG(I+M)	DEG(M)	AAL30373.1	--	Z	--
XLOC_014299	DEG(M)	DEG(M)	DEG(I+M)	DEG(M)	AFC88820.1	--	Z	--
XLOC_014300	DEG(M)	DEG(M)	DEG(I+M)	DEG(M)	AIC77175.1	--	Z	--
XLOC_014301	DEG(M)	No	No	No	XP_002668382.1	--	Z	--
XLOC_014307	No	No	No	No	XP_004507185.1	--	--	--
XLOC_014313	No	No	DEG(I+M)	No	--	--	X	--
XLOC_014322	DEG(M)	No	No	No	EUC64786.1	--	--	K01581
XLOC_014323	DEG(M)	No	No	No	--	--	--	--
XLOC_014325	No	No	No	No	XP_008912633.1	AP2	C	K00294
XLOC_014331	DEG(M)	No	No	No	NP_001155623.1	--	Z	--
XLOC_014333	No	No	No	No	--	--	--	K14826
XLOC_014334	DEG(M)	No	No	No	XP_009529127.1	--	T	K02218
XLOC_014335	No	No	No	No	--	--	--	--
XLOC_014338	DEG(M)	No	No	No	--	--	--	--
XLOC_014341	No	No	No	No	BAJ89237.1	--	O	K09487
XLOC_014345	No	No	No	No	--	--	--	--
XLOC_014352	No	No	No	No	EUC53695.1	--	--	--
XLOC_014357	DEG(M)	No	No	No	EUC57230.1	--	--	--
XLOC_014359	No	No	No	No	KDN42656.1	--	--	--
XLOC_014362	DEG(M)	No	No	No	DAA06120.1	--	--	--
XLOC_014365	No	No	No	No	--	--	--	--
XLOC_014367	No	No	No	No	XP_001653838.1	--	O	--
XLOC_014368	No	No	No	No	ETM56484.1	--	C	--
XLOC_014369	No	No	No	No	XP_002902119.1	--	C	K03521
XLOC_014373	No	No	No	No	--	--	--	--
XLOC_014375	No	No	No	No	--	--	--	K11982
XLOC_014376	No	No	No	No	KDN46211.1	--	--	--
XLOC_014377	DEG(M)	No	No	No	BAJ61942.1	YABBY	OR	--
XLOC_014384	No	No	No	No	XP_004495320.1	--	P	K08176
XLOC_014385	No	No	DEG(I+M)	No	XP_004497842.1	--	P	K08176
XLOC_014388	No	No	No	No	XP_004341437.1	--	J	--
XLOC_014397	No	No	No	No	ETI45522.1	--	--	K02730
XLOC_014398	No	No	No	No	XP_003048482.1	--	--	--
XLOC_014408	DEG(M)	No	No	No	--	--	--	--
XLOC_014419	No	No	No	No	ETO83706.1	--	--	--
XLOC_014423	No	No	No	No	EUC60653.1	--	--	--
XLOC_014424	No	No	No	No	EUC60653.1	--	C	K00411
XLOC_014432	DEG(M)	No	No	No	--	--	--	--

Differentially expressed gene (DEG) IDs	*C. microphyllum* root and shoot-specific (>2 fold)	*C. microphyllum* root and shoot-specific (>10 fold)	ICC4958 and *C. microphyllum* root-specific (>2 fold)	*C. microphyllum* root-specific (>2 fold)	Functional Annotation			
					NCBI nr IDs	TFs	KOG	KEGG
XLOC_014433	DEG(M)	No	No	No	KEP49546.1	--	--	--
XLOC_014439	No	No	No	No	XP_001663045.1	--	O	--
XLOC_014443	DEG(M)	DEG(M)	DEG(I+M)	DEG(M)	EGU82820.1	--	--	--
XLOC_014447	No	No	DEG(I+M)	No	XP_004513041.1	--	--	K05658
XLOC_014457	No	No	No	No	XP_001943357.1	--	--	K15979
XLOC_014458	No	No	No	No	--	--	C	K08908
XLOC_014459	DEG(M)	No	No	No	XP_003040957.1	--	--	--
XLOC_014466	No	No	No	No	--	Trihelix	G	--
XLOC_014472	No	No	No	No	--	TALE	K	--
XLOC_014481	DEG(M)	No	No	No	--	--	--	--
XLOC_014482	No	No	No	No	--	--	G	--
XLOC_014487	DEG(M)	DEG(M)	No	No	--	--	--	--
XLOC_014488	DEG(M)	DEG(M)	No	No	--	--	--	--
XLOC_014490	No	No	No	No	--	ERF	R	K03327
XLOC_014497	No	No	DEG(I+M)	No	XP_004507637.1	--	T	K12462
XLOC_014498	No	No	No	No	BAN33737.1	--	--	--
XLOC_014500	No	No	No	No	KEP53516.1	--	--	--
XLOC_014502	No	No	DEG(I+M)	No	--	--	U	K17095
XLOC_014503	No	No	No	No	--	--	--	--
XLOC_014505	No	No	DEG(I+M)	No	--	--	--	--
XLOC_014507	No	No	No	No	--	--	--	--
XLOC_014511	No	No	No	No	--	--	--	--
XLOC_014517	No	No	No	No	XP_001950349.1	--	I	K13137
XLOC_014518	No	No	No	No	--	--	--	K17279
XLOC_014522	No	No	No	No	--	--	--	--
XLOC_014524	No	No	No	No	NP_001155868.1	--	--	--
XLOC_014528	No	No	No	No	--	--	EG	--
XLOC_014531	No	No	No	No	--	--	--	--
XLOC_014533	No	No	No	No	EUC65412.1	--	--	--
XLOC_014537	DEG(M)	No	No	No	--	--	--	--
XLOC_014540	DEG(M)	No	No	No	AAU84924.1	MYB	T	K14753
XLOC_014542	No	No	No	No	ETM35079.1	--	--	--
XLOC_014543	No	No	No	No	ETM35079.1	--	--	--
XLOC_014545	No	No	No	No	--	--	--	--
XLOC_014546	No	No	No	No	--	--	--	--
XLOC_014549	No	No	No	No	XP_004511732.1	--	--	K02884
XLOC_014551	No	No	No	No	KEP53528.1	--	--	--
XLOC_014553	No	No	No	No	--	--	--	--
XLOC_014555	No	No	No	No	--	--	--	--
XLOC_014556	DEG(M)	No	No	No	--	--	--	--

Differentially expressed gene (DEG) IDs	*C. microphyllum* root and shoot-specific (>2 fold)	*C. microphyllum* root and shoot-specific (>10 fold)	ICC4958 and *C. microphyllum* root-specific (>2 fold)	*C. microphyllum* root-specific (>2 fold)	Functional Annotation			
					NCBI nr IDs	TFs	KOG	KEGG
XLOC_014557	No	No	No	No	--	--	--	--
XLOC_014558	No	No	No	No	--	--	--	--
XLOC_014560	No	No	No	No	--	--	--	--
XLOC_014561	DEG(M)	No	No	No	NP_001129414.1	--	J	K02947
XLOC_014562	DEG(M)	No	No	No	ETP22964.1	--	--	--
XLOC_014564	DEG(M)	No	No	No	KEP51534.1	--	--	--
XLOC_014565	DEG(M)	DEG(M)	No	No	--	--	--	--
XLOC_014567	No	No	No	No	EUC67655.1	--	C	K00235
XLOC_014570	DEG(M)	No	No	No	--	--	--	--
XLOC_014580	No	No	No	No	--	--	--	--
XLOC_014584	No	No	DEG(I+M)	No	EMT61469.1	--	--	--
XLOC_014585	DEG(M)	No	No	No	--	--	--	--
XLOC_014586	No	No	No	No	--	--	--	--
XLOC_014594	DEG(M)	No	No	No	--	--	--	K02943
XLOC_014595	DEG(M)	No	No	No	--	--	--	--
XLOC_014596	DEG(M)	DEG(M)	No	No	--	--	--	--
XLOC_014598	No	No	No	No	--	--	--	--
XLOC_014606	DEG(M)	No	No	No	--	--	O	--
XLOC_014657	DEG(M)	No	No	No	--	--	--	--
XLOC_014658	DEG(M)	No	No	No	--	--	--	--
XLOC_014659	No	No	No	No	EUC53728.1	--	--	--
XLOC_014660	DEG(M)	No	No	No	--	--	--	--
XLOC_014661	DEG(M)	No	No	No	CEL53157.1	--	--	--
XLOC_014662	DEG(M)	No	No	No	CEL53157.1	--	--	--
XLOC_014667	DEG(M)	DEG(M)	No	No	--	--	--	K02694
XLOC_014671	No	No	No	No	--	--	--	--
XLOC_014696	DEG(M)	DEG(M)	DEG(I+M)	DEG(M)	CCO30270.1	--	Z	--
XLOC_014698	DEG(M)	No	No	No	AFK38702.1	--	Z	--
XLOC_014699	DEG(M)	No	No	No	XP_967267.1	--	Z	K07375
XLOC_014700	No	No	No	No	XP_012423664.1	--	Z	--
XLOC_014701	No	No	No	No	ELU41335.1	--	Z	--
XLOC_014709	No	No	No	No	--	--	T	--
XLOC_014710	DEG(M)	No	No	No	--	--	--	--
XLOC_014733	DEG(M)	DEG(M)	DEG(I+M)	DEG(M)	--	--	--	--
XLOC_014734	DEG(M)	DEG(M)	DEG(I+M)	DEG(M)	BAN33737.1	--	J	--
XLOC_014831	No	No	DEG(I+M)	No	--	--	--	--
XLOC_014832	DEG(M)	No	DEG(I+M)	DEG(M)	--	--	--	--
XLOC_014834	DEG(M)	No	DEG(I+M)	DEG(M)	XP_004497856.1	--	S	--
XLOC_014835	No	No	DEG(I+M)	No	XP_004497856.1	--	--	--
XLOC_014837	No	No	No	No	--	--	--	K02518

Differentially expressed gene (DEG) IDs	*C. microphyllum* root and shoot-specific (>2 fold)	*C. microphyllum* root and shoot-specific (>10 fold)	ICC4958 and *C. microphyllum* root-specific (>2 fold)	*C. microphyllum* root-specific (>2 fold)	Functional Annotation			
					NCBI nr IDs	TFs	KOG	KEGG
XLOC_014878	DEG(M)	No	DEG(I+M)	DEG(M)	XP_012568922.1	--	O	K03283
XLOC_014884	No	No	No	No	XP_004490406.1	ZF-HD	--	--
XLOC_014893	No	No	No	No	--	--	--	--
XLOC_014913	No	No	No	No	--	--	--	--
XLOC_014915	No	No	No	No	--	--	--	--
XLOC_014917	DEG(M)	No	DEG(I+M)	DEG(M)	--	--	--	--
XLOC_014923	DEG(M)	No	No	No	--	--	--	--
XLOC_014931	No	No	No	No	EUC66687.1	--	--	--
XLOC_014947	No	No	No	No	ELU38939.1	--	--	--
XLOC_014948	DEG(M)	DEG(M)	No	No	--	--	--	--
XLOC_014950	DEG(M)	No	No	No	CEL54514.1	--	--	--
XLOC_014958	No	No	No	No	BAN64557.1	--	O	--
XLOC_014965	No	No	No	No	XP_008915460.1	--	--	--
XLOC_014966	No	No	No	No	ETK71084.1	--	O	K09571
XLOC_014967	No	No	No	No	XP_007710108.1	--	--	K01725
XLOC_014970	No	No	No	No	--	--	--	--
XLOC_014971	DEG(M)	DEG(M)	No	No	XP_004502738.1	--	O	K13993
XLOC_014972	No	No	No	No	--	--	--	K00074
XLOC_014973	No	No	No	No	XP_008180865.1	--	--	--
XLOC_014974	DEG(M)	No	No	No	KNB03983.1	--	J	--
XLOC_014977	No	No	No	No	--	--	--	--
XLOC_014981	No	No	No	No	EUC57238.1	--	--	--
XLOC_014985	No	No	No	No	--	--	--	--
XLOC_014986	No	No	No	No	CCO29340.1	--	--	--
XLOC_014987	No	No	DEG(I+M)	No	XP_012569498.1	--	Q	--
XLOC_014988	No	No	No	No	CEL52439.1	--	--	--
XLOC_014989	No	No	No	No	ACO50125.1	--	J	--
XLOC_014990	No	No	No	No	--	--	--	--
XLOC_014991	No	No	No	No	--	--	--	--
XLOC_014992	No	No	No	No	XP_004508730.1	--	--	K10960
XLOC_014994	No	No	No	No	KEP51876.1	--	C	--
XLOC_014995	DEG(M)	No	No	No	--	--	--	--
XLOC_014996	No	No	No	No	--	--	--	--
XLOC_014999	No	No	No	No	--	--	--	--
XLOC_015003	No	No	No	No	--	--	--	--
XLOC_015005	DEG(M)	No	No	No	--	--	--	--
XLOC_015006	No	No	No	No	--	--	--	--
XLOC_015008	No	No	No	No	CEL51784.1	--	--	K03953
XLOC_015009	No	No	No	No	--	--	--	--
XLOC_015014	DEG(M)	No	No	No	--	--	--	--

Differentially expressed gene (DEG) IDs	*C. microphyllum* root and shoot-specific (>2 fold)	*C. microphyllum* root and shoot-specific (>10 fold)	ICC4958 and *C. microphyllum* root-specific (>2 fold)	*C. microphyllum* root-specific (>2 fold)	Functional Annotation			
					NCBI nr IDs	TFs	KOG	KEGG
XLOC_015015	No	No	No	No	--	--	--	--
XLOC_015016	DEG(M)	No	No	No	--	--	--	--
XLOC_015017	DEG(M)	No	No	No	--	--	--	--
XLOC_015018	No	No	No	No	--	--	--	--
XLOC_015019	No	No	No	No	--	--	--	--
XLOC_015020	DEG(M)	No	No	No	XP_012889614.1	--	J	--
XLOC_015021	No	No	No	No	XP_002905419.1	FAR1	TR	--
XLOC_015022	No	No	No	No	--	--	--	--
XLOC_015025	No	No	No	No	--	--	RI	--
XLOC_015027	No	No	No	No	--	--	--	--
XLOC_015032	No	No	No	No	XP_004516742.1	--	--	--
XLOC_015034	No	No	No	No	--	--	--	--
XLOC_015040	No	No	No	No	CEL56302.1	--	--	--
XLOC_015044	DEG(M)	No	No	No	AAQ12016.1	--	Z	--
XLOC_015045	DEG(M)	No	No	No	AAQ12016.1	--	Z	--
XLOC_015046	No	No	No	No	AAQ12016.1	--	Z	--
XLOC_015072	DEG(M)	No	No	No	XP_004487383.1	--	--	K15086
XLOC_015074	DEG(M)	DEG(M)	DEG(I+M)	DEG(M)	--	--	--	--
XLOC_015143	DEG(M)	No	No	No	--	--	--	--
XLOC_015152	DEG(M)	DEG(M)	DEG(I+M)	DEG(M)	--	--	--	--
XLOC_015154	DEG(M)	DEG(M)	DEG(I+M)	DEG(M)	--	--	--	--
XLOC_015158	DEG(M)	No	No	No	--	--	--	--
XLOC_015159	DEG(M)	DEG(M)	No	No	--	--	--	--
XLOC_015176	DEG(M)	No	No	No	ADV77191.1	--	B	--
XLOC_015177	DEG(M)	DEG(M)	DEG(I+M)	DEG(M)	BAD90779.1	--	B	--
XLOC_015178	DEG(M)	DEG(M)	No	No	XP_454338.1	bHLH	B	--
XLOC_015182	DEG(M)	No	No	No	XP_007363844.1	--	B	--
XLOC_015185	DEG(M)	DEG(M)	DEG(I+M)	DEG(M)	--	--	--	--
XLOC_015195	No	No	DEG(I+M)	No	--	--	--	--
XLOC_015197	DEG(M)	No	DEG(I+M)	DEG(M)	XP_004490687.1	--	--	--
XLOC_015199	DEG(M)	No	DEG(I+M)	DEG(M)	XP_004490696.1	--	--	--
XLOC_015220	No	No	DEG(I+M)	No	--	--	X	--
XLOC_015231	DEG(M)	No	No	No	--	--	--	--
XLOC_015243	No	No	DEG(I+M)	No	--	--	Q	--
XLOC_015244	No	No	DEG(I+M)	No	--	--	Q	--
XLOC_015245	DEG(M)	No	DEG(I+M)	DEG(M)	--	--	--	--
XLOC_015246	DEG(M)	DEG(M)	DEG(I+M)	DEG(M)	--	--	--	--
XLOC_015278	DEG(M)	No	No	No	EUC62298.1	--	--	--
XLOC_015279	DEG(M)	DEG(M)	DEG(I+M)	DEG(M)	CEL51747.1	--	--	--
XLOC_015290	DEG(M)	DEG(M)	No	No	--	--	--	--

Differentially expressed gene (DEG) IDs	*C. microphyllum* root and shoot-specific (>2 fold)	*C. microphyllum* root and shoot-specific (>10 fold)	ICC4958 and *C. microphyllum* root-specific (>2 fold)	*C. microphyllum* root-specific (>2 fold)	Functional Annotation			
					NCBI nr IDs	TFs	KOG	KEGG
XLOC_015305	No	No	No	No	--	--	--	--
XLOC_015311	No	No	No	No	--	--	--	--
XLOC_015312	No	No	DEG(I+M)	No	--	--	--	--
XLOC_015313	DEG(M)	No	No	No	--	--	--	K12846
XLOC_015315	No	No	No	No	--	--	--	--
XLOC_015316	No	No	No	No	--	--	--	--
XLOC_015318	No	No	No	No	--	--	--	--
XLOC_015319	No	No	No	No	--	--	J	--
XLOC_015321	DEG(M)	No	No	No	--	--	--	--
XLOC_015322	No	No	No	No	XP_008914020.1	--	--	--
XLOC_015324	No	No	No	No	XP_001951536.1	--	J	K02923
XLOC_015325	DEG(M)	No	No	No	KFM56591.1	--	J	--
XLOC_015326	No	No	DEG(I+M)	No	--	--	--	--
XLOC_015328	DEG(M)	No	No	No	--	--	--	--
XLOC_015332	DEG(M)	DEG(M)	No	No	--	--	--	--
XLOC_015333	No	No	No	No	XP_002510991.1	--	--	--
XLOC_015337	DEG(M)	No	No	No	XP_001951042.1	--	J	K02925
XLOC_015339	No	No	No	No	XP_003053543.1	--	--	--
XLOC_015340	No	No	No	No	NP_001119666.1	--	--	--
XLOC_015342	No	No	No	No	CCO31540.1	--	--	--
XLOC_015343	No	No	No	No	XP_001944825.2	--	--	K12897
XLOC_015345	DEG(M)	DEG(M)	DEG(I+M)	DEG(M)	XP_009529544.1	--	J	K02898
XLOC_015346	No	No	No	No	--	--	E	K01696
XLOC_015349	DEG(M)	No	No	No	--	--	--	--
XLOC_015354	No	No	No	No	--	--	--	--
XLOC_015357	No	No	No	No	KDN48097.1	--	--	--
XLOC_015358	No	No	No	No	EUC63216.1	--	--	--
XLOC_015359	No	No	No	No	XP_002909364.1	--	--	--
XLOC_015360	No	No	No	No	--	--	--	--
XLOC_015362	No	No	No	No	--	--	--	--
XLOC_015365	No	No	No	No	XP_001944681.1	--	--	--
XLOC_015367	No	No	No	No	XP_001949774.2	--	O	K03032
XLOC_015369	No	No	No	No	XP_009521750.1	--	O	K02729
XLOC_015373	No	No	No	No	--	--	--	--
XLOC_015378	DEG(M)	No	No	No	--	--	--	--
XLOC_015379	No	No	No	No	--	--	--	--
XLOC_015382	No	No	No	No	XP_002519742.1	--	--	--
XLOC_015384	DEG(M)	No	No	No	--	--	J	--
XLOC_015386	DEG(M)	No	No	No	Q5XUC7.1	--	J	K02932
XLOC_015392	No	No	No	No	KFM64989.1	--	A	K14298

Differentially expressed gene (DEG) IDs	*C. microphyllum* root and shoot-specific (>2 fold)	*C. microphyllum* root and shoot-specific (>10 fold)	ICC4958 and *C. microphyllum* root-specific (>2 fold)	*C. microphyllum* root-specific (>2 fold)	Functional Annotation			
					NCBI nr IDs	TFs	KOG	KEGG
XLOC_015393	No	No	No	No	KDP45860.1	--	E	K00815
XLOC_015394	No	No	No	No	CCO35361.1	--	--	--
XLOC_015400	No	No	No	No	--	--	--	--
XLOC_015401	No	No	No	No	XP_001658896.1	--	J	--
XLOC_015404	DEG(M)	No	No	No	--	--	--	--
XLOC_015405	No	No	No	No	--	--	--	--
XLOC_015406	No	No	No	No	--	--	--	--
XLOC_015409	No	No	No	No	ETI46039.1	--	--	--
XLOC_015410	No	No	No	No	--	--	--	K02639
XLOC_015411	No	No	No	No	--	--	--	--
XLOC_015412	No	No	No	No	--	--	--	--
XLOC_015413	No	No	No	No	EUC59824.1	--	--	--
XLOC_015416	DEG(M)	No	No	No	--	--	--	--
XLOC_015419	No	No	No	No	--	--	--	--
XLOC_015421	DEG(M)	No	No	No	ELU38122.1	--	--	--
XLOC_015422	No	No	No	No	--	--	--	--
XLOC_015425	No	No	No	No	--	--	--	--
XLOC_015426	DEG(M)	No	No	No	XP_009524103.1	--	--	--
XLOC_015427	DEG(M)	No	No	No	EWZ87609.1	--	--	--
XLOC_015429	No	No	No	No	EUC65158.1	--	--	--
XLOC_015430	No	No	No	No	XP_001949061.2	--	O	--
XLOC_015432	DEG(M)	No	No	No	--	--	--	--
XLOC_015433	No	No	No	No	--	--	--	--
XLOC_015434	No	No	No	No	--	--	--	--
XLOC_015436	No	No	No	No	XP_001653617.1	--	--	--
XLOC_015437	No	No	No	No	CEL63215.1	--	--	--
XLOC_015439	No	No	No	No	--	--	--	--
XLOC_015441	DEG(M)	DEG(M)	No	No	--	--	--	--
XLOC_015442	DEG(M)	No	No	No	EUC65580.1	--	--	--
XLOC_015445	No	No	No	No	--	--	U	--
XLOC_015449	No	No	No	No	AKM28418.1	--	--	--
XLOC_015450	DEG(M)	No	No	No	NP_001119646.1	--	J	--
XLOC_015456	No	No	No	No	AJQ30116.1	--	G	--
XLOC_015457	No	No	No	No	--	--	--	--
XLOC_015458	No	No	No	No	XP_003744731.1	--	J	K03236
XLOC_015460	No	No	No	No	--	--	--	--
XLOC_015461	No	No	No	No	XP_001658105.1	--	J	--
XLOC_015466	No	No	No	No	--	--	--	--
XLOC_015472	No	No	No	No	XP_008880238.1	--	C	--
XLOC_015476	No	No	No	No	ETK84834.1	--	--	--

Differentially expressed gene (DEG) IDs	***C. microphyllum* root and shoot-specific (>2 fold)**	***C. microphyllum* root and shoot-specific (>10 fold)**	**ICC4958 and *C. microphyllum* root-specific (>2 fold)**	***C. microphyllum* root-specific (>2 fold)**	**Functional Annotation**			
					**NCBI nr IDs**	**TFs**	**KOG**	**KEGG**
XLOC_015477	No	No	No	No	ETI44845.1	--	--	--
XLOC_015480	No	No	No	No	EUC66712.1	--	--	--
XLOC_015481	No	No	No	No	--	--	--	--
XLOC_015482	No	No	No	No	--	--	--	--
XLOC_015484	No	No	No	No	XP_002511960.1	--	Z	K11340
XLOC_015486	No	No	No	No	--	--	--	--
XLOC_015487	No	No	No	No	XP_001944294.1	--	--	--
XLOC_015490	No	No	No	No	--	--	G	K00616
XLOC_015491	No	No	No	No	XP_008178869.1	--	O	--
XLOC_015493	No	No	No	No	--	--	--	K12881
XLOC_015494	No	No	No	No	--	--	--	--
XLOC_015495	No	No	No	No	EUC60645.1	--	--	--
XLOC_015496	No	No	No	No	--	--	--	--
XLOC_015498	No	No	No	No	KEP50845.1	--	--	--
XLOC_015502	No	No	No	No	KDN42941.1	--	--	--
XLOC_015505	No	No	No	No	--	--	E	K00457
XLOC_015506	No	No	No	No	--	--	--	--
XLOC_015507	No	No	No	No	XP_008913646.1	--	O	--
XLOC_015511	No	No	No	No	KEP48226.1	--	--	K12309
XLOC_015515	No	No	No	No	ETM45336.1	--	--	K05236
XLOC_015516	No	No	No	No	XP_001949691.2	--	J	--
XLOC_015517	DEG(M)	No	No	No	--	--	--	--
XLOC_015518	No	No	No	No	--	--	--	--
XLOC_015521	No	No	No	No	--	--	J	K02901
XLOC_015526	No	No	No	No	--	MIKC	J	--
XLOC_015527	DEG(M)	No	No	No	--	--	--	--
XLOC_015531	No	No	No	No	--	--	--	--
XLOC_015532	No	No	No	No	--	--	--	--
XLOC_015534	No	No	No	No	--	--	--	--
XLOC_015538	No	No	No	No	--	--	--	--
XLOC_015539	No	No	No	No	XP_008906582.1	--	--	--
XLOC_015541	No	No	No	No	--	--	--	--
XLOC_015542	No	No	No	No	--	--	--	--
XLOC_015543	No	No	No	No	--	--	--	--
XLOC_015544	DEG(M)	No	No	No	ETK84835.1	--	--	--
XLOC_015547	No	No	No	No	--	--	--	--
XLOC_015551	DEG(M)	No	No	No	ELU44878.1	--	--	--
XLOC_015552	DEG(M)	No	No	No	--	--	--	--
XLOC_015556	DEG(M)	DEG(M)	No	No	--	--	--	--
XLOC_015558	DEG(M)	No	No	No	--	--	--	--

Differentially expressed gene (DEG) IDs	***C. microphyllum* root and shoot-specific (>2 fold)**	***C. microphyllum* root and shoot-specific (>10 fold)**	**ICC4958 and *C. microphyllum* root-specific (>2 fold)**	***C. microphyllum* root-specific (>2 fold)**	**Functional Annotation**			
					**NCBI nr IDs**	**TFs**	**KOG**	**KEGG**
XLOC_015559	No	No	No	No	YP_008758398.1	--	--	--
XLOC_015567	No	No	No	No	--	--	--	--
XLOC_015568	No	No	No	No	CCO32385.1	--	--	--
XLOC_015571	No	No	No	No	--	--	I	--
XLOC_015573	No	No	No	No	BAA36503.1	--	--	--
XLOC_015574	DEG(M)	No	No	No	--	--	--	--
XLOC_015575	No	No	No	No	KEP52898.1	--	--	--
XLOC_015579	DEG(M)	No	No	No	--	--	--	--
XLOC_015580	DEG(M)	No	No	No	--	--	--	--
XLOC_015582	No	No	No	No	--	--	--	--
XLOC_015583	No	No	No	No	--	--	--	--
XLOC_015584	No	No	No	No	KIN00968.1	--	B	K11252
XLOC_015587	No	No	No	No	EUC57729.1	--	--	--
XLOC_015590	No	No	No	No	XP_003049592.1	--	--	--
XLOC_015595	No	No	No	No	EWZ50781.1	--	--	--
XLOC_015596	No	No	No	No	--	--	--	--
XLOC_015597	No	No	No	No	--	--	--	--
XLOC_015599	DEG(M)	No	No	No	CCO29659.1	--	--	--
XLOC_015600	No	No	No	No	--	--	--	K03955
XLOC_015601	No	No	No	No	--	--	--	--
XLOC_015603	No	No	No	No	CEL59118.1	--	T	--
XLOC_015604	No	No	No	No	--	--	--	K01641
XLOC_015605	No	No	No	No	--	--	--	--
XLOC_015606	No	No	No	No	CEF70706.1	--	W	--
XLOC_015610	No	No	No	No	--	--	--	--
XLOC_015611	No	No	No	No	KNA19494.1	--	O	--
XLOC_015612	No	No	DEG(I+M)	No	XP_004502903.1	--	--	K19040
XLOC_015613	DEG(M)	No	DEG(I+M)	DEG(M)	--	--	--	--
XLOC_015621	DEG(M)	No	No	No	XP_003243529.1	--	--	--
XLOC_015623	No	No	DEG(I+M)	No	--	--	--	--
XLOC_015624	No	No	No	No	--	--	--	--
XLOC_015625	No	No	No	No	NP_001155581.1	--	Z	--
XLOC_015627	No	No	No	No	--	--	--	--
XLOC_015629	No	No	No	No	--	--	--	--
XLOC_015631	No	No	No	No	KEP47258.1	--	--	--
XLOC_015632	DEG(M)	DEG(M)	DEG(I+M)	DEG(M)	EGU84616.1	--	--	--
XLOC_015633	DEG(M)	DEG(M)	No	No	--	--	--	--
XLOC_015636	DEG(M)	No	No	No	CCO29553.1	--	C	--
XLOC_015638	DEG(M)	No	No	No	--	--	--	--
XLOC_015640	No	No	No	No	CEL55858.1	--	J	K02896

Differentially expressed gene (DEG) IDs	***C. microphyllum* root and shoot-specific (>2 fold)**	***C. microphyllum* root and shoot-specific (>10 fold)**	**ICC4958 and *C. microphyllum* root-specific (>2 fold)**	***C. microphyllum* root-specific (>2 fold)**	**Functional Annotation**			
					**NCBI nr IDs**	**TFs**	**KOG**	**KEGG**
XLOC_015641	No	No	No	No	XP_001652214.2	--	--	--
XLOC_015644	No	No	No	No	XP_001652847.1	--	J	--
XLOC_015646	No	No	No	No	DAA62448.1	--	S	K13648
XLOC_015650	DEG(M)	No	DEG(I+M)	DEG(M)	KIO21459.1	--	--	--
XLOC_015652	No	No	No	No	XP_008182405.1	--	--	--
XLOC_015653	No	No	No	No	--	--	--	--
XLOC_015657	DEG(M)	No	No	No	EUC54637.1	--	--	--
XLOC_015659	No	No	No	No	--	--	E	--
XLOC_015660	No	No	No	No	EUC53652.1	--	--	--
XLOC_015662	DEG(M)	No	No	No	KLO98320.1	--	--	--
XLOC_015669	DEG(M)	No	No	No	--	--	--	--
XLOC_015670	DEG(M)	DEG(M)	DEG(I+M)	DEG(M)	--	--	--	--
XLOC_015673	No	No	No	No	--	--	--	--
XLOC_015674	No	No	No	No	--	--	--	--
XLOC_015675	No	No	No	No	KDQ14482.1	GRAS	F	K00940
XLOC_015676	No	No	No	No	KEP52732.1	B3	O	--
XLOC_015680	No	No	No	No	--	--	--	--
XLOC_015681	No	No	No	No	--	--	--	--
XLOC_015682	No	No	No	No	KDN36371.1	--	--	--
XLOC_015686	No	No	No	No	--	--	J	--
XLOC_015688	No	No	No	No	XP_001949486.2	--	O	--
XLOC_015692	DEG(M)	No	No	No	--	--	--	--
XLOC_015693	No	No	No	No	--	--	--	--
XLOC_015695	DEG(M)	No	No	No	--	--	J	--
XLOC_015696	No	No	No	No	XP_003046131.1	--	--	--
XLOC_015699	No	No	No	No	XP_002840578.1	--	E	K01649
XLOC_015701	DEG(M)	No	No	No	KEP50222.1	--	--	--
XLOC_015703	No	No	No	No	XP_001659089.1	--	--	--
XLOC_015706	No	No	No	No	KDN50943.1	--	DTZ	--
XLOC_015707	No	No	No	No	--	--	--	--
XLOC_015711	DEG(M)	No	No	No	--	--	--	--
XLOC_015713	No	No	No	No	--	--	--	--
XLOC_015721	No	No	No	No	XP_008911755.1	--	--	--
XLOC_015722	No	No	No	No	--	--	--	--
XLOC_015726	No	No	No	No	CEL59691.1	--	--	--
XLOC_015727	No	No	No	No	--	--	--	--
XLOC_015730	No	No	No	No	XP_003043751.1	--	--	--
XLOC_015731	No	No	No	No	--	--	--	--
XLOC_015734	No	No	No	No	XP_008460180.1	--	C	K00028
XLOC_015736	No	No	No	No	--	--	--	--

Differentially expressed gene (DEG) IDs	*C. microphyllum* root and shoot-specific (>2 fold)	*C. microphyllum* root and shoot-specific (>10 fold)	ICC4958 and *C. microphyllum* root-specific (>2 fold)	*C. microphyllum* root-specific (>2 fold)	Functional Annotation			
					NCBI nr IDs	TFs	KOG	KEGG
XLOC_015740	No	No	No	No	KEP49874.1	--	--	--
XLOC_015742	No	No	No	No	--	--	--	--
XLOC_015745	No	No	DEG(I+M)	No	--	--	--	K07195
XLOC_015750	No	No	No	No	--	--	--	--
XLOC_015752	No	No	No	No	--	--	T	--
XLOC_015756	No	No	No	No	EUC65189.1	--	A	--
XLOC_015757	No	No	No	No	CEL63038.1	--	--	--
XLOC_015758	No	No	No	No	--	--	--	--
XLOC_015759	No	No	No	No	CEL60831.1	--	C	K03661
XLOC_015764	DEG(M)	No	DEG(I+M)	DEG(M)	EWY88983.1	--	--	--
XLOC_015765	No	No	No	No	--	--	--	--
XLOC_015768	No	No	No	No	--	--	--	--
XLOC_015772	DEG(M)	No	No	No	--	--	--	--
XLOC_015776	No	No	No	No	--	--	--	--
XLOC_015777	No	No	No	No	--	--	--	--
XLOC_015779	DEG(M)	No	No	No	--	--	--	--
XLOC_015780	No	No	No	No	--	--	--	--
XLOC_015781	DEG(M)	No	No	No	--	--	--	--
XLOC_015783	No	No	No	No	KEP52741.1	--	--	--
XLOC_015785	DEG(M)	No	No	No	--	--	--	--
XLOC_015790	No	No	No	No	CCO32187.1	--	--	K19355
XLOC_015791	DEG(M)	No	No	No	EUC66587.1	--	--	--
XLOC_015796	No	No	No	No	--	--	S	K17987
XLOC_015797	No	No	No	No	--	--	--	--
XLOC_015798	No	No	No	No	--	--	--	--
XLOC_015799	DEG(M)	No	No	No	CEL55684.1	--	--	--
XLOC_015801	No	No	No	No	--	--	--	--
XLOC_015803	DEG(M)	No	No	No	--	--	--	--
XLOC_015804	DEG(M)	DEG(M)	No	No	--	--	--	--
XLOC_015805	No	No	DEG(I+M)	No	XP_004507319.1	--	G	--
XLOC_015807	No	No	No	No	--	--	--	K01785
XLOC_015808	DEG(M)	No	No	No	XP_008901107.1	--	--	--
XLOC_015809	DEG(M)	DEG(M)	DEG(I+M)	DEG(M)	XP_008906950.1	--	--	--
XLOC_015813	No	No	No	No	--	--	--	--
XLOC_015816	DEG(M)	No	No	No	--	--	--	--
XLOC_015820	No	No	No	No	XP_001947772.2	--	O	K03029
XLOC_015822	No	No	No	No	KDN47870.1	--	--	--
XLOC_015823	DEG(M)	No	No	No	--	--	--	K01785
XLOC_015824	No	No	No	No	--	--	--	K05387
XLOC_015825	No	No	No	No	KDN34195.1	--	M	--

Differentially expressed gene (DEG) IDs	*C. microphyllum* root and shoot-specific (>2 fold)	*C. microphyllum* root and shoot-specific (>10 fold)	ICC4958 and *C. microphyllum* root-specific (>2 fold)	*C. microphyllum* root-specific (>2 fold)	Functional Annotation			
					NCBI nr IDs	TFs	KOG	KEGG
XLOC_015826	DEG(M)	No	No	No	EUC65231.1	--	M	K00820
XLOC_015830	No	No	No	No	XP_004506774.2	--	C	K08915
XLOC_015833	DEG(M)	No	No	No	XP_002897608.1	--	--	K02882
XLOC_015834	DEG(M)	DEG(M)	DEG(I+M)	DEG(M)	XP_002897608.1	--	--	K02882
XLOC_015835	DEG(M)	DEG(M)	DEG(I+M)	DEG(M)	XP_002897608.1	--	--	--
XLOC_015836	DEG(M)	DEG(M)	DEG(I+M)	DEG(M)	XP_008897642.1	--	--	--
XLOC_015843	DEG(M)	No	No	No	--	--	--	--
XLOC_015844	DEG(M)	No	No	No	--	--	--	--
XLOC_015852	No	No	No	No	XP_004486405.1	--	--	--
XLOC_015855	No	No	No	No	--	--	--	--
XLOC_015866	DEG(M)	No	No	No	--	--	--	--
XLOC_015867	DEG(M)	No	No	No	--	--	--	--
XLOC_015870	DEG(M)	DEG(M)	DEG(I+M)	DEG(M)	--	--	--	--
XLOC_015874	No	No	DEG(I+M)	No	--	--	--	K13262
XLOC_015879	No	No	No	No	--	--	--	--
XLOC_015883	DEG(M)	No	DEG(I+M)	DEG(M)	ELU36802.1	--	--	--
XLOC_015884	DEG(M)	No	DEG(I+M)	DEG(M)	ELU36802.1	--	--	--
XLOC_015890	No	No	No	No	--	--	--	K02890
XLOC_015923	No	No	No	No	AKM28425.1	--	--	--
XLOC_015926	No	No	No	No	EUC57369.1	--	--	--
XLOC_015927	No	No	No	No	KEP49696.1	--	--	--
XLOC_015928	DEG(M)	No	No	No	XP_004493258.1	--	--	--
XLOC_015937	DEG(M)	No	No	No	--	--	--	--
XLOC_015938	DEG(M)	No	No	No	--	--	--	--
XLOC_015944	DEG(M)	No	No	No	EUC57218.1	--	T	--
XLOC_015946	DEG(M)	DEG(M)	DEG(I+M)	DEG(M)	EUC57218.1	--	T	--
XLOC_015947	DEG(M)	DEG(M)	DEG(I+M)	DEG(M)	ETK82424.1	--	T	K02183
XLOC_015948	DEG(M)	DEG(M)	DEG(I+M)	DEG(M)	ETK82424.1	--	T	K02183
XLOC_015949	DEG(M)	DEG(M)	DEG(I+M)	DEG(M)	AAH53790.1	--	T	--
XLOC_015963	No	No	No	No	--	--	--	--
XLOC_015964	DEG(M)	No	No	No	EUC57244.1	--	--	--
XLOC_015965	DEG(M)	No	No	No	XP_003043730.1	--	--	--
XLOC_015970	No	No	DEG(I+M)	No	--	--	--	--
XLOC_015971	DEG(M)	No	No	No	--	--	--	K15040
XLOC_015977	No	No	No	No	ETK87798.1	--	--	--
XLOC_015981	No	No	No	No	XP_009537046.1	--	Z	--
XLOC_015984	No	No	No	No	--	--	--	--
XLOC_015985	No	No	DEG(I+M)	No	XP_003610532.1	--	P	K14445
XLOC_015991	No	No	No	No	EUC62624.1	--	--	--
XLOC_015992	No	No	No	No	EUC66790.1	FAR1	TR	K06269

Differentially expressed gene (DEG) IDs	*C. microphyllum* root and shoot-specific (>2 fold)	*C. microphyllum* root and shoot-specific (>10 fold)	ICC4958 and *C. microphyllum* root-specific (>2 fold)	*C. microphyllum* root-specific (>2 fold)	Functional Annotation			
					NCBI nr IDs	TFs	KOG	KEGG
XLOC_015993	No	No	No	No	GAA94565.1	FAR1	TR	K06269
XLOC_015995	DEG(M)	No	No	No	--	--	--	--
XLOC_015999	DEG(M)	DEG(M)	No	No	--	--	--	--
XLOC_016005	No	No	No	No	KDN44833.1	--	--	--
XLOC_016011	No	No	No	No	--	--	Q	K05280
XLOC_016018	DEG(M)	DEG(M)	DEG(I+M)	DEG(M)	EWY90435.1	--	J	--
XLOC_016023	No	No	No	No	CCO37403.1	MYB_related	G	--
XLOC_016026	DEG(M)	No	DEG(I+M)	DEG(M)	--	--	--	--
XLOC_016028	No	No	No	No	--	--	--	--
XLOC_016029	No	No	No	No	--	--	G	--
XLOC_016030	No	No	No	No	XP_003242556.1	--	--	K15692
XLOC_016032	No	No	No	No	ETK78779.1	--	--	--
XLOC_016033	No	No	No	No	--	--	--	--
XLOC_016034	No	No	No	No	--	--	--	--
XLOC_016035	No	No	No	No	XP_001657556.1	--	O	--
XLOC_016037	No	No	No	No	--	--	Z	--
XLOC_016038	No	No	No	No	--	--	--	--
XLOC_016041	No	No	No	No	XP_003044686.1	--	--	--
XLOC_016043	No	No	DEG(I+M)	No	XP_004485682.1	--	--	--
XLOC_016047	No	No	No	No	--	--	--	--
XLOC_016052	No	No	No	No	KDN34509.1	--	--	--
XLOC_016053	No	No	No	No	EUC61508.1	--	--	--
XLOC_016062	DEG(M)	DEG(M)	No	No	--	--	--	--
XLOC_016063	DEG(M)	No	No	No	EMT65757.1	--	--	--
XLOC_016064	DEG(M)	No	No	No	--	--	--	--
XLOC_016065	DEG(M)	DEG(M)	No	No	--	--	--	--
XLOC_016069	DEG(M)	No	No	No	--	--	--	--
XLOC_016070	No	No	No	No	--	--	--	--
XLOC_016071	DEG(M)	DEG(M)	DEG(I+M)	DEG(M)	--	--	--	--
XLOC_016076	No	No	No	No	--	--	--	--
XLOC_016077	No	No	No	No	--	--	T	--
XLOC_016094	No	No	No	No	--	--	--	--
XLOC_016095	No	No	No	No	XP_001846040.1	--	O	K04043
XLOC_016097	DEG(M)	No	DEG(I+M)	DEG(M)	--	--	--	--
XLOC_016099	No	No	No	No	CEL51806.1	--	--	--
XLOC_016100	No	No	No	No	--	--	S	--
XLOC_016108	No	No	No	No	EFN86838.1	--	TDK	K03115
XLOC_016112	No	No	No	No	--	--	--	--
XLOC_016115	No	No	No	No	--	--	--	--
XLOC_016126	DEG(M)	No	No	No	--	--	--	--

Differentially expressed gene (DEG) IDs	*C. microphyllum* root and shoot-specific (>2 fold)	*C. microphyllum* root and shoot-specific (>10 fold)	ICC4958 and *C. microphyllum* root-specific (>2 fold)	*C. microphyllum* root-specific (>2 fold)	Functional Annotation			
					NCBI nr IDs	TFs	KOG	KEGG
XLOC_016129	No	No	No	No	ETL42389.1	--	--	--
XLOC_016135	DEG(M)	DEG(M)	DEG(I+M)	DEG(M)	XP_001729044.1	YABBY	J	--
XLOC_016136	No	No	No	No	--	--	--	--
XLOC_016140	DEG(M)	DEG(M)	DEG(I+M)	DEG(M)	--	--	--	--
XLOC_016142	DEG(M)	DEG(M)	DEG(I+M)	DEG(M)	--	--	--	--
XLOC_016143	DEG(M)	No	DEG(I+M)	DEG(M)	XP_008896383.1	--	J	K02958
XLOC_016152	No	No	No	No	--	--	--	--
XLOC_016156	DEG(M)	No	No	No	--	--	--	--
XLOC_016160	No	No	No	No	--	--	--	--
XLOC_016163	No	No	No	No	EUC63051.1	--	--	K04459
XLOC_016164	No	No	No	No	ELU38507.1	--	--	--
XLOC_016165	DEG(M)	DEG(M)	DEG(I+M)	DEG(M)	XP_009529898.1	--	J	K02951
XLOC_016171	No	No	No	No	--	--	J	--
XLOC_016173	No	No	No	No	--	--	--	--
XLOC_016175	DEG(M)	No	No	No	--	--	--	--
XLOC_016180	No	No	No	No	--	--	--	--
XLOC_016187	No	No	DEG(I+M)	No	--	--	--	--
XLOC_016188	DEG(M)	No	No	No	--	--	--	--
XLOC_016189	No	No	No	No	XP_004500603.1	--	P	K17469
XLOC_016190	No	No	No	No	--	--	--	--
XLOC_016193	No	No	No	No	CEL59311.1	--	--	--
XLOC_016194	No	No	No	No	--	--	--	--
XLOC_016195	No	No	No	No	--	--	--	K01836
XLOC_016197	No	No	DEG(I+M)	No	XP_004489732.1	ERF	--	--
XLOC_016198	No	No	No	No	KDN47067.1	--	A	--
XLOC_016199	No	No	No	No	KEP52760.1	--	A	--
XLOC_016200	DEG(M)	No	No	No	XP_001653696.1	--	J	K02957
XLOC_016203	No	No	No	No	EUC61163.1	--	J	--
XLOC_016204	DEG(M)	No	No	No	EUC61163.1	--	J	--
XLOC_016208	No	No	No	No	--	--	R	K08150
XLOC_016216	No	No	No	No	--	--	--	--
XLOC_016217	No	No	No	No	--	--	DKT	--
XLOC_016226	DEG(M)	No	DEG(I+M)	DEG(M)	--	--	--	--
XLOC_016229	DEG(M)	DEG(M)	DEG(I+M)	DEG(M)	--	--	--	--
XLOC_016247	DEG(M)	No	No	No	XP_008912321.1	--	A	--
XLOC_016248	DEG(M)	DEG(M)	DEG(I+M)	DEG(M)	XP_008912321.1	--	A	--
XLOC_016250	DEG(M)	No	No	No	--	--	--	--
XLOC_016257	DEG(M)	DEG(M)	No	No	XP_004491526.1	--	GE	--
XLOC_016259	No	No	No	No	--	--	--	--
XLOC_016260	No	No	No	No	KEP48909.1	--	--	--

Differentially expressed gene (DEG) IDs	*C. microphyllum* root and shoot-specific (>2 fold)	*C. microphyllum* root and shoot-specific (>10 fold)	ICC4958 and *C. microphyllum* root-specific (>2 fold)	*C. microphyllum* root-specific (>2 fold)	Functional Annotation			
					NCBI nr IDs	TFs	KOG	KEGG
XLOC_016261	No	No	No	No	CCO31475.1	--	--	--
XLOC_016266	DEG(M)	DEG(M)	No	No	--	--	--	K00430
XLOC_016270	No	No	No	No	--	--	--	--
XLOC_016273	No	No	DEG(I+M)	No	--	--	--	--
XLOC_016278	DEG(M)	DEG(M)	DEG(I+M)	DEG(M)	CEL61021.1	--	--	--
XLOC_016279	DEG(M)	No	No	No	--	--	--	--
XLOC_016284	DEG(M)	DEG(M)	No	No	--	--	--	--
XLOC_016286	DEG(M)	No	No	No	XP_004491074.1	--	--	--
XLOC_016289	DEG(M)	No	No	No	CEL53407.1	--	--	--
XLOC_016293	DEG(M)	No	DEG(I+M)	DEG(M)	ELU45307.1	--	--	--
XLOC_016300	DEG(M)	No	No	No	--	--	--	--
XLOC_016307	DEG(M)	DEG(M)	No	No	--	--	--	--
XLOC_016308	DEG(M)	DEG(M)	DEG(I+M)	DEG(M)	XP_004489721.1	--	--	K09875
XLOC_016310	No	No	DEG(I+M)	No	--	--	E	--
XLOC_016311	DEG(M)	DEG(M)	DEG(I+M)	DEG(M)	--	--	--	--
XLOC_016312	DEG(M)	No	No	No	--	--	--	--
XLOC_016313	DEG(M)	No	No	No	--	--	--	--
XLOC_016315	DEG(M)	No	No	No	EUC62280.1	--	J	--
XLOC_016316	DEG(M)	No	No	No	XP_007323568.1	--	J	--
XLOC_016317	DEG(M)	No	DEG(I+M)	DEG(M)	CCO33287.1	--	J	--
XLOC_016319	DEG(M)	DEG(M)	No	No	--	--	--	--
XLOC_016338	DEG(M)	No	No	No	--	--	--	--
XLOC_016343	No	No	No	No	--	--	--	--
XLOC_016345	DEG(M)	No	No	No	KDN37362.1	--	--	--
XLOC_016346	No	No	No	No	--	--	--	--
XLOC_016347	DEG(M)	DEG(M)	No	No	--	--	--	--
XLOC_016350	No	No	No	No	CEL53920.1	--	--	--
XLOC_016351	DEG(M)	No	No	No	--	--	--	--
XLOC_016352	No	No	No	No	--	--	--	--
XLOC_016353	DEG(M)	No	DEG(I+M)	DEG(M)	--	--	--	--
XLOC_016354	No	No	No	No	--	--	--	--
XLOC_016355	No	No	No	No	--	--	--	--
XLOC_016356	No	No	No	No	EUC54385.1	--	--	--
XLOC_016357	No	No	No	No	ERG87647.1	--	T	--
XLOC_016358	No	No	No	No	EUC56872.1	--	--	--
XLOC_016360	No	No	No	No	--	--	--	K03147
XLOC_016365	No	No	DEG(I+M)	No	--	--	C	K01507
XLOC_016367	No	No	DEG(I+M)	No	--	--	--	--
XLOC_016369	No	No	DEG(I+M)	No	--	--	--	--
XLOC_016371	DEG(M)	No	No	No	--	--	--	--

Differentially expressed gene (DEG) IDs	*C. microphyllum* root and shoot-specific (>2 fold)	*C. microphyllum* root and shoot-specific (>10 fold)	ICC4958 and *C. microphyllum* root-specific (>2 fold)	*C. microphyllum* root-specific (>2 fold)	Functional Annotation			
					NCBI nr IDs	TFs	KOG	KEGG
XLOC_016372	DEG(M)	No	No	No	KDN35801.1	--	O	K13113
XLOC_016374	No	No	No	No	--	--	--	--
XLOC_016375	DEG(M)	No	DEG(I+M)	DEG(M)	XP_004489677.1	--	--	--
XLOC_016379	DEG(M)	No	DEG(I+M)	DEG(M)	CEL63147.1	--	--	--
XLOC_016380	No	No	No	No	NP_001153860.1	--	O	--
XLOC_016381	No	No	No	No	XP_002509785.1	--	O	--
XLOC_016382	DEG(M)	DEG(M)	No	No	--	--	--	--
XLOC_016383	DEG(M)	No	No	No	--	--	--	--
XLOC_016388	DEG(M)	No	No	No	XP_008901215.1	--	--	--
XLOC_016390	DEG(M)	No	No	No	KDN46211.1	--	--	--
XLOC_016395	DEG(M)	No	No	No	--	--	--	--
XLOC_016397	No	No	No	No	--	--	--	K09584
XLOC_016398	DEG(M)	No	No	No	EUC64250.1	--	--	K19355
XLOC_016401	No	No	No	No	CEL56743.1	--	--	--
XLOC_016403	DEG(M)	No	No	No	--	--	--	--
XLOC_016406	No	No	No	No	KDN46215.1	--	--	--
XLOC_016409	No	No	No	No	XP_004508503.1	--	--	--
XLOC_016410	DEG(M)	No	No	No	XP_004494169.1	bZIP	--	K14432
XLOC_016412	No	No	No	No	CCT67240.1	--	--	--
XLOC_016418	DEG(M)	DEG(M)	DEG(I+M)	DEG(M)	XP_008903366.1	--	--	K02889
XLOC_016420	No	No	No	No	CEL57540.1	--	--	--
XLOC_016421	DEG(M)	No	No	No	CCO27341.1	--	--	--
XLOC_016423	No	No	No	No	--	--	--	--
XLOC_016431	DEG(M)	No	No	No	--	--	--	--
XLOC_016432	DEG(M)	DEG(M)	No	No	XP_003047954.1	--	--	--
XLOC_016433	DEG(M)	No	No	No	CEL61083.1	--	--	--
XLOC_016434	DEG(M)	No	DEG(I+M)	DEG(M)	KDN47500.1	--	--	--
XLOC_016437	No	No	No	No	--	--	--	--
XLOC_016439	No	No	No	No	NP_001153852.1	--	C	K01648
XLOC_016440	No	No	No	No	XP_003040092.1	--	--	--
XLOC_016442	DEG(M)	No	No	No	EUC59871.1	--	--	--
XLOC_016446	No	No	No	No	--	--	--	--
XLOC_016449	No	No	No	No	--	--	--	--
XLOC_016452	No	No	No	No	XP_001948247.2	--	C	K00382
XLOC_016454	No	No	DEG(I+M)	No	--	--	--	--
XLOC_016458	No	No	No	No	--	--	--	--
XLOC_016461	No	No	No	No	EUC62234.1	--	C	--
XLOC_016463	No	No	No	No	--	--	--	--
XLOC_016464	No	No	No	No	CCO37180.1	--	--	--
XLOC_016467	No	No	No	No	XP_008908714.1	--	--	--

Differentially expressed gene (DEG) IDs	*C. microphyllum* root and shoot-specific (>2 fold)	*C. microphyllum* root and shoot-specific (>10 fold)	ICC4958 and *C. microphyllum* root-specific (>2 fold)	*C. microphyllum* root-specific (>2 fold)	Functional Annotation			
					NCBI nr IDs	TFs	KOG	KEGG
XLOC_016469	No	No	No	No	CCO26376.1	--	--	--
XLOC_016470	No	No	No	No	ETP41073.1	--	--	--
XLOC_016471	No	No	No	No	KEP47084.1	--	R	K02206
XLOC_016472	No	No	No	No	--	--	--	--
XLOC_016476	No	No	No	No	CEL58304.1	--	--	--
XLOC_016477	No	No	No	No	--	--	--	--
XLOC_016478	No	No	No	No	CEL59337.1	--	--	--
XLOC_016481	DEG(M)	No	No	No	CEL57336.1	--	B	--
XLOC_016482	DEG(M)	No	No	No	XP_008898418.1	--	C	--
XLOC_016483	No	No	No	No	XP_012069849.1	--	O	--
XLOC_016487	DEG(M)	No	No	No	NP_001155818.1	--	--	K03232
XLOC_016488	DEG(M)	No	No	No	--	--	--	--
XLOC_016489	No	No	No	No	XP_001657499.1	--	--	--
XLOC_016491	No	No	No	No	ETL89373.1	--	--	--
XLOC_016492	No	No	No	No	--	--	--	--
XLOC_016499	No	No	No	No	XP_003053493.1	--	O	K14826
XLOC_016501	No	No	No	No	XP_003043298.1	--	--	--
XLOC_016502	No	No	No	No	--	--	--	K09571
XLOC_016506	No	No	No	No	--	--	--	--
XLOC_016510	No	No	No	No	--	--	--	K12668
XLOC_016511	DEG(M)	No	No	No	--	--	--	--
XLOC_016514	No	No	No	No	--	--	--	K02942
XLOC_016515	DEG(M)	No	No	No	--	--	--	--
XLOC_016516	No	No	No	No	ETL99429.1	--	I	K01895
XLOC_016517	DEG(M)	DEG(M)	No	No	NP_001129421.1	--	J	K02898
XLOC_016518	No	No	No	No	--	--	--	--
XLOC_016520	No	No	No	No	--	--	--	--
XLOC_016522	No	No	No	No	ELU38147.1	--	--	--
XLOC_016524	No	No	No	No	ELU37766.1	--	--	--
XLOC_016525	No	No	No	No	ETM03553.1	--	--	--
XLOC_016527	DEG(M)	No	No	No	XP_008908742.1	--	--	K03938
XLOC_016532	No	No	No	No	XP_008903984.1	--	--	--
XLOC_016534	No	No	No	No	ETK93129.1	--	--	--
XLOC_016536	No	No	No	No	--	--	--	--
XLOC_016538	DEG(M)	No	No	No	--	--	--	--
XLOC_016539	No	No	No	No	--	--	--	K02640
XLOC_016541	No	No	No	No	--	--	--	--
XLOC_016542	No	No	No	No	--	--	--	K01897
XLOC_016543	DEG(M)	No	No	No	--	--	--	--
XLOC_016544	No	No	No	No	--	--	J	--

Differentially expressed gene (DEG) IDs	*C. microphyllum* root and shoot-specific (>2 fold)	*C. microphyllum* root and shoot-specific (>10 fold)	ICC4958 and *C. microphyllum* root-specific (>2 fold)	*C. microphyllum* root-specific (>2 fold)	Functional Annotation			
					NCBI nr IDs	TFs	KOG	KEGG
XLOC_016545	DEG(M)	No	No	No	--	--	--	--
XLOC_016546	DEG(M)	No	No	No	--	--	J	K02891
XLOC_016558	No	No	No	No	CEL59855.1	--	--	--
XLOC_016565	No	No	No	No	XP_001662847.1	--	O	K09500
XLOC_016566	No	No	DEG(I+M)	No	--	--	--	--
XLOC_016575	No	No	No	No	XP_009514793.1	--	--	--
XLOC_016577	No	No	No	No	--	--	T	--
XLOC_016578	No	No	No	No	AAN62919.1	--	J	--
XLOC_016579	DEG(M)	No	No	No	--	--	--	--
XLOC_016587	No	No	No	No	--	--	--	--
XLOC_016588	No	No	No	No	--	--	--	--
XLOC_016591	DEG(M)	No	No	No	--	--	--	--
XLOC_016594	No	No	No	No	--	--	--	--
XLOC_016595	No	No	No	No	--	--	--	--
XLOC_016596	DEG(M)	No	No	No	XP_004510298.1	--	--	--
XLOC_016602	DEG(M)	No	No	No	--	--	--	K02891
XLOC_016607	DEG(M)	No	No	No	XP_002509814.1	--	R	K09597
XLOC_016609	DEG(M)	No	No	No	XP_004510130.1	--	--	--
XLOC_016612	DEG(M)	DEG(M)	No	No	NP_001165735.1	--	--	--
XLOC_016616	DEG(M)	No	DEG(I+M)	DEG(M)	XP_008892912.1	--	--	--
XLOC_016617	DEG(M)	DEG(M)	DEG(I+M)	DEG(M)	--	--	--	--
XLOC_016620	DEG(M)	No	No	No	--	--	--	K08762
XLOC_016621	DEG(M)	No	No	No	--	--	--	--
XLOC_016624	DEG(M)	No	No	No	XP_008183989.1	--	--	--
XLOC_016627	No	No	No	No	--	--	--	--
XLOC_016633	DEG(M)	DEG(M)	DEG(I+M)	DEG(M)	--	--	--	--
XLOC_016634	DEG(M)	No	No	No	AAM51207.1	YABBY	OR	--
XLOC_016635	DEG(M)	No	No	No	AAP34635.1	--	J	K02927
XLOC_016636	No	No	No	No	--	--	--	--
XLOC_016639	DEG(M)	No	No	No	ELU39904.1	--	T	--
XLOC_016640	DEG(M)	No	No	No	ELU39904.1	--	T	--
XLOC_016646	No	No	No	No	--	--	--	--
XLOC_016647	No	No	No	No	KDN50682.1	--	E	K15741
XLOC_016668	DEG(M)	No	No	No	--	--	--	--
XLOC_016672	DEG(M)	No	No	No	KDN41553.1	--	--	--
XLOC_016678	No	No	No	No	XP_002517216.1	--	R	--
XLOC_016679	No	No	No	No	--	--	--	--
XLOC_016680	No	No	No	No	--	--	--	--
XLOC_016683	No	No	No	No	--	--	--	--
XLOC_016684	DEG(M)	No	No	No	--	--	--	--

Differentially expressed gene (DEG) IDs	*C. microphyllum* root and shoot-specific (>2 fold)	*C. microphyllum* root and shoot-specific (>10 fold)	ICC4958 and *C. microphyllum* root-specific (>2 fold)	*C. microphyllum* root-specific (>2 fold)	Functional Annotation			
					NCBI nr IDs	TFs	KOG	KEGG
XLOC_016685	No	No	No	No	AHX56890.1	--	--	--
XLOC_016688	DEG(M)	No	No	No	XP_003049980.1	--	--	--
XLOC_016689	No	No	No	No	XP_012568283.1	--	X	K08901
XLOC_016692	No	No	No	No	--	--	--	--
XLOC_016696	No	No	No	No	--	--	--	--
XLOC_016703	DEG(M)	No	No	No	XP_008911143.1	--	--	K15040
XLOC_016704	DEG(M)	DEG(M)	DEG(I+M)	DEG(M)	--	--	--	--
XLOC_016707	No	No	No	No	--	--	--	K09420
XLOC_016708	No	No	No	No	XP_013139796.1	--	J	K02978
XLOC_016709	No	No	No	No	CCO34829.1	--	C	--
XLOC_016710	No	No	No	No	--	--	--	--
XLOC_016712	No	No	No	No	XP_003054620.1	--	E	K01649
XLOC_016714	DEG(M)	No	No	No	--	--	O	--
XLOC_016715	DEG(M)	DEG(M)	No	No	B0FWC7.2	--	C	K02256
XLOC_016719	No	No	No	No	XP_009537635.1	--	--	--
XLOC_016724	No	No	No	No	--	--	--	K01968
XLOC_016725	DEG(M)	No	No	No	--	--	--	--
XLOC_016728	No	No	No	No	--	--	--	--
XLOC_016735	No	No	No	No	--	--	--	--
XLOC_016736	No	No	No	No	--	--	--	K11290
XLOC_016738	No	No	No	No	XP_002529860.1	--	R	--
XLOC_016740	DEG(M)	DEG(M)	No	No	EXA43907.1	--	--	--
XLOC_016741	No	No	No	No	EUC56981.1	--	--	--
XLOC_016742	No	No	No	No	--	--	--	--
XLOC_016743	DEG(M)	DEG(M)	No	No	XP_003049959.1	--	--	--
XLOC_016744	No	No	No	No	--	--	--	--
XLOC_016745	No	No	No	No	--	--	--	--
XLOC_016747	No	No	DEG(I+M)	No	XP_004495978.1	--	--	K00799
XLOC_016754	DEG(M)	No	No	No	AHX56189.1	MYB	T	K14753
XLOC_016755	No	No	No	No	--	--	--	--
XLOC_016757	No	No	No	No	--	--	--	--
XLOC_016758	No	No	No	No	KFM62399.1	--	W	--
XLOC_016761	No	No	No	No	--	--	--	--
XLOC_016766	No	No	No	No	--	--	--	--
XLOC_016768	DEG(M)	No	No	No	XP_008903775.1	--	--	--
XLOC_016770	No	No	No	No	ABF18368.1	--	--	--
XLOC_016774	No	No	No	No	--	--	O	--
XLOC_016775	No	No	No	No	KIO22274.1	--	--	K02732
XLOC_016777	No	No	No	No	EUC61183.1	--	--	--
XLOC_016779	DEG(M)	DEG(M)	DEG(I+M)	DEG(M)	--	--	--	--

Differentially expressed gene (DEG) IDs	***C. microphyllum* root and shoot-specific (>2 fold)**	***C. microphyllum* root and shoot-specific (>10 fold)**	**ICC4958 and *C. microphyllum* root-specific (>2 fold)**	***C. microphyllum* root-specific (>2 fold)**	**Functional Annotation**			
					**NCBI nr IDs**	**TFs**	**KOG**	**KEGG**
XLOC_016780	No	No	No	No	EUC65108.1	--	--	--
XLOC_016781	No	No	No	No	XP_001655389.1	--	J	--
XLOC_016784	No	No	No	No	EUC67699.1	--	--	--
XLOC_016786	No	No	No	No	--	--	--	--
XLOC_016788	DEG(M)	DEG(M)	No	No	--	--	--	--
XLOC_016791	No	No	No	No	KDN51881.1	--	--	--
XLOC_016792	No	No	No	No	XP_003054324.1	--	RT	--
XLOC_016794	No	No	No	No	--	--	--	--
XLOC_016796	No	No	No	No	--	--	--	--
XLOC_016797	No	No	No	No	--	--	--	--
XLOC_016800	DEG(M)	No	No	No	--	--	--	--
XLOC_016801	DEG(M)	No	DEG(I+M)	DEG(M)	--	--	--	--
XLOC_016805	No	No	No	No	BAH70793.1	--	U	--
XLOC_016807	No	No	No	No	--	--	--	--
XLOC_016811	No	No	No	No	KEP47204.1	--	--	K00249
XLOC_016812	DEG(M)	No	DEG(I+M)	DEG(M)	ETP37648.1	--	--	--
XLOC_016813	No	No	No	No	XP_008902415.1	--	R	--
XLOC_016814	DEG(M)	No	No	No	XP_008902386.1	--	--	--
XLOC_016815	No	No	No	No	--	--	W	--
XLOC_016816	DEG(M)	No	No	No	--	--	--	--
XLOC_016825	No	No	No	No	--	--	--	--
XLOC_016833	No	No	No	No	--	--	--	--
XLOC_016841	No	No	DEG(I+M)	No	XP_012573660.1	--	--	--
XLOC_016844	No	No	DEG(I+M)	No	--	--	--	--
XLOC_016845	No	No	No	No	KEP55374.1	--	--	--
XLOC_016848	DEG(M)	No	No	No	CCO30091.1	--	--	--
XLOC_016851	No	No	No	No	--	--	--	--
XLOC_016852	DEG(M)	No	DEG(I+M)	DEG(M)	--	--	--	K02943
XLOC_016853	DEG(M)	No	No	No	EUC59786.1	--	--	--
XLOC_016854	DEG(M)	No	DEG(I+M)	DEG(M)	EUC59786.1	--	--	--
XLOC_016860	DEG(M)	No	No	No	KEP52000.1	--	--	--
XLOC_016864	No	No	No	No	--	NAC	J	K02991
XLOC_016865	No	No	No	No	AHG94986.1	--	O	--
XLOC_016868	No	No	No	No	--	--	--	--
XLOC_016872	No	No	No	No	XP_004487486.1	--	--	--
XLOC_016882	DEG(M)	No	No	No	--	--	--	--
XLOC_016883	No	No	No	No	KEP46123.1	--	--	--
XLOC_016884	No	No	No	No	KEP46123.1	--	--	--
XLOC_016885	DEG(M)	No	No	No	--	--	--	--
XLOC_016903	DEG(M)	No	No	No	--	--	--	--

Differentially expressed gene (DEG) IDs	*C. microphyllum* root and shoot-specific (>2 fold)	*C. microphyllum* root and shoot-specific (>10 fold)	ICC4958 and *C. microphyllum* root-specific (>2 fold)	*C. microphyllum* root-specific (>2 fold)	Functional Annotation			
					NCBI nr IDs	TFs	KOG	KEGG
XLOC_016904	DEG(M)	No	No	No	--	--	--	--
XLOC_016914	No	No	No	No	--	--	--	--
XLOC_016916	No	No	No	No	--	--	--	--
XLOC_016917	No	No	No	No	--	--	--	--
XLOC_016926	No	No	DEG(I+M)	No	--	--	--	--
XLOC_016945	DEG(M)	No	No	No	--	--	--	--
XLOC_016946	No	No	DEG(I+M)	No	--	--	--	--
XLOC_016950	No	No	No	No	XP_003051258.1	--	--	--
XLOC_016957	No	No	No	No	ELU41192.1	--	--	--
XLOC_016962	DEG(M)	No	No	No	--	--	--	--
XLOC_016978	No	No	No	No	--	--	--	K10257
XLOC_016979	No	No	No	No	--	--	--	K10257
XLOC_016985	No	No	DEG(I+M)	No	--	--	--	--
XLOC_016989	No	No	No	No	ETI50009.1	--	C	--
XLOC_016993	No	No	No	No	CCO26137.1	--	O	--
XLOC_016994	No	No	No	No	EUC67144.1	--	O	--
XLOC_016995	No	No	No	No	CCO35007.1	--	--	--
XLOC_016996	No	No	No	No	EUC60552.1	--	--	--
XLOC_016999	DEG(M)	No	No	No	XP_003045269.1	bHLH	J	K02937
XLOC_017002	No	No	No	No	--	--	--	--
XLOC_017003	No	No	DEG(I+M)	No	EMD91245.1	--	--	--
XLOC_017011	No	No	No	No	XP_002526173.1	--	K	K15172
XLOC_017013	No	No	DEG(I+M)	No	--	--	--	--
XLOC_017015	DEG(M)	DEG(M)	No	No	--	--	--	--
XLOC_017016	No	No	No	No	--	--	--	--
XLOC_017017	No	No	No	No	--	--	--	--
XLOC_017019	No	No	No	No	NP_001155503.1	--	--	--
XLOC_017022	No	No	No	No	--	--	--	--
XLOC_017030	No	No	No	No	EJW74297.1	--	J	--
XLOC_017035	DEG(M)	No	No	No	--	--	--	--
XLOC_017041	No	No	No	No	--	--	--	--
XLOC_017044	No	No	No	No	--	--	--	K02871
XLOC_017047	No	No	No	No	XP_002414970.1	--	C	--
XLOC_017056	No	No	No	No	EUC57729.1	--	--	--
XLOC_017057	No	No	No	No	XP_008907933.1	--	--	--
XLOC_017058	No	No	No	No	XP_008353260.1	MYB_related	K	--
XLOC_017060	No	No	No	No	--	--	J	K03251
XLOC_017061	No	No	No	No	--	--	--	--
XLOC_017064	DEG(M)	No	No	No	XP_004509754.1	MIKC	K	K09264
XLOC_017065	No	No	No	No	--	--	--	--

Differentially expressed gene (DEG) IDs	*C. microphyllum* root and shoot-specific (>2 fold)	*C. microphyllum* root and shoot-specific (>10 fold)	ICC4958 and *C. microphyllum* root-specific (>2 fold)	*C. microphyllum* root-specific (>2 fold)	Functional Annotation			
					NCBI nr IDs	TFs	KOG	KEGG
XLOC_017067	No	No	No	No	--	--	--	--
XLOC_017069	No	No	No	No	XP_008906263.1	MYB	--	K01803
XLOC_017070	No	No	No	No	--	--	--	--
XLOC_017071	No	No	No	No	EUC58678.1	--	--	K00122
XLOC_017072	No	No	No	No	--	--	--	K11308
XLOC_017073	No	No	No	No	--	--	E	--
XLOC_017077	No	No	No	No	--	--	--	--
XLOC_017080	No	No	No	No	KLO84017.1	--	--	--
XLOC_017086	No	No	No	No	--	--	--	--
XLOC_017090	No	No	No	No	--	--	--	--
XLOC_017091	DEG(M)	No	No	No	--	--	--	--
XLOC_017092	No	No	DEG(I+M)	No	XP_004505050.1	HSF	--	K09419
XLOC_017096	DEG(M)	DEG(M)	No	No	NP_001119682.1	--	J	K02912
XLOC_017099	No	No	No	No	XP_012086141.1	--	X	--
XLOC_017100	No	No	No	No	--	--	--	--
XLOC_017101	No	No	No	No	XP_001952329.1	--	G	--
XLOC_017102	No	No	No	No	XP_001952329.1	--	G	--
XLOC_017103	No	No	No	No	EUC57369.1	--	--	--
XLOC_017113	No	No	No	No	AII97801.1	--	C	--
XLOC_017114	No	No	No	No	--	--	--	--
XLOC_017115	DEG(M)	No	DEG(I+M)	DEG(M)	--	--	--	--
XLOC_017116	No	No	No	No	--	--	--	--
XLOC_017118	DEG(M)	DEG(M)	No	No	--	--	--	--
XLOC_017119	No	No	No	No	XP_004510958.1	C2H2	--	--
XLOC_017121	No	No	No	No	--	--	--	--
XLOC_017123	No	No	No	No	NP_001155787.1	--	--	--
XLOC_017125	DEG(M)	No	No	No	EUC66143.1	--	--	K11086
XLOC_017127	DEG(M)	DEG(M)	No	No	--	--	--	--
XLOC_017129	No	No	No	No	--	--	--	--
XLOC_017131	No	No	No	No	--	--	--	--
XLOC_017132	No	No	No	No	--	--	--	--
XLOC_017133	DEG(M)	DEG(M)	No	No	--	--	--	--
XLOC_017134	No	No	No	No	--	--	--	--
XLOC_017135	No	No	No	No	XP_008915960.1	M-type	O	K03030
XLOC_017138	No	No	No	No	EGU83120.1	--	--	--
XLOC_017139	No	No	No	No	--	--	--	--
XLOC_017140	No	No	No	No	--	--	--	--
XLOC_017141	No	No	No	No	--	--	--	--
XLOC_017145	No	No	No	No	ETL84151.1	--	--	--
XLOC_017146	No	No	No	No	ETL84151.1	--	--	--

Differentially expressed gene (DEG) IDs	*C. microphyllum* root and shoot-specific (>2 fold)	*C. microphyllum* root and shoot-specific (>10 fold)	ICC4958 and *C. microphyllum* root-specific (>2 fold)	*C. microphyllum* root-specific (>2 fold)	Functional Annotation			
					NCBI nr IDs	TFs	KOG	KEGG
XLOC_017148	DEG(M)	No	No	No	AAS55947.1	--	J	K02989
XLOC_017149	DEG(M)	No	No	No	--	--	--	--
XLOC_017154	No	No	No	No	ELU41444.1	--	--	--
XLOC_017155	No	No	No	No	XP_009514443.1	--	--	--
XLOC_017156	No	No	No	No	ETP46505.1	--	Q	--
XLOC_017157	DEG(M)	No	No	No	--	MYB_related	F	--
XLOC_017160	No	No	No	No	XP_001944498.1	--	--	K01103
XLOC_017163	No	No	No	No	--	--	J	--
XLOC_017165	No	No	No	No	XP_002523194.1	HD-ZIP	K	K09338
XLOC_017168	No	No	No	No	--	--	--	--
XLOC_017169	No	No	No	No	--	--	--	K03260
XLOC_017172	No	No	No	No	CEL54134.1	--	--	--
XLOC_017173	No	No	No	No	--	--	--	--
XLOC_017175	No	No	No	No	XP_008900470.1	--	--	--
XLOC_017180	No	No	No	No	XP_004491502.1	--	--	--
XLOC_017182	No	No	No	No	XP_001945529.1	--	--	--
XLOC_017187	No	No	No	No	NP_001155502.1	--	C	--
XLOC_017189	No	No	No	No	--	--	--	--
XLOC_017196	No	No	No	No	--	--	CR	K18980
XLOC_017197	No	No	DEG(I+M)	No	XP_004485887.1	--	--	--
XLOC_017198	DEG(M)	No	No	No	--	--	--	--
XLOC_017201	No	No	No	No	--	--	--	K03403
XLOC_017207	No	No	DEG(I+M)	No	--	--	M	K01051
XLOC_017208	No	No	No	No	KIL95500.1	--	--	--
XLOC_017210	No	No	No	No	--	--	--	--
XLOC_017218	No	No	No	No	CEL59691.1	--	--	--
XLOC_017222	DEG(M)	DEG(M)	DEG(I+M)	DEG(M)	XP_002903192.1	--	J	--
XLOC_017226	No	No	DEG(I+M)	No	--	--	--	--
XLOC_017227	No	No	No	No	XP_003052352.1	--	--	--
XLOC_017228	No	No	No	No	--	--	--	--
XLOC_017229	No	No	No	No	--	--	--	--
XLOC_017232	DEG(M)	No	No	No	--	--	--	--
XLOC_017233	No	No	No	No	--	--	--	--
XLOC_017237	No	No	No	No	EUC65378.1	--	--	--
XLOC_017242	DEG(M)	No	DEG(I+M)	DEG(M)	KIE01056.1	--	J	--
XLOC_017245	DEG(M)	DEG(M)	DEG(I+M)	DEG(M)	XP_008904849.1	--	--	--
XLOC_017246	No	No	No	No	--	--	K	K04683
XLOC_017250	DEG(M)	DEG(M)	DEG(I+M)	DEG(M)	--	GRAS	F	K00940
XLOC_017256	DEG(M)	DEG(M)	DEG(I+M)	DEG(M)	--	--	--	--
XLOC_017258	No	No	No	No	--	--	--	--

Differentially expressed gene (DEG) IDs	*C. microphyllum* root and shoot-specific (>2 fold)	*C. microphyllum* root and shoot-specific (>10 fold)	ICC4958 and *C. microphyllum* root-specific (>2 fold)	*C. microphyllum* root-specific (>2 fold)	Functional Annotation			
					NCBI nr IDs	TFs	KOG	KEGG
XLOC_017259	No	No	No	No	--	--	--	--
XLOC_017260	DEG(M)	DEG(M)	No	No	--	--	--	K04078
XLOC_017261	No	No	No	No	XP_008913005.1	--	O	--
XLOC_017262	No	No	No	No	--	--	--	--
XLOC_017263	No	No	No	No	--	--	--	--
XLOC_017265	No	No	No	No	XP_003050029.1	--	A	--
XLOC_017269	No	No	No	No	DAA06091.1	--	--	--
XLOC_017270	No	No	No	No	--	--	--	--
XLOC_017271	No	No	No	No	XP_008024200.1	--	--	--
XLOC_017273	DEG(M)	No	DEG(I+M)	DEG(M)	--	--	--	--
XLOC_017274	No	No	No	No	--	--	--	--
XLOC_017283	No	No	No	No	--	--	--	--
XLOC_017290	No	No	No	No	--	--	--	K14652
XLOC_017298	DEG(M)	No	No	No	--	--	J	K02969
XLOC_017302	No	No	No	No	XP_003043298.1	--	G	--
XLOC_017303	No	No	DEG(I+M)	No	--	--	--	--
XLOC_017306	No	No	No	No	--	--	Q	K11816
XLOC_017308	DEG(M)	No	No	No	KEP54662.1	--	C	--
XLOC_017309	No	No	No	No	--	--	--	--
XLOC_017313	No	No	No	No	--	--	--	--
XLOC_017316	No	No	No	No	--	--	--	K07178
XLOC_017327	No	No	No	No	--	--	--	--
XLOC_017328	No	No	No	No	XP_008179438.1	TCP	J	K03264
XLOC_017331	No	No	No	No	--	--	--	--
XLOC_017332	No	No	No	No	XP_001946617.2	--	--	--
XLOC_017333	No	No	No	No	CEL59662.1	--	--	--
XLOC_017335	DEG(M)	No	No	No	AFK41760.1	--	J	K02985
XLOC_017336	No	No	No	No	NP_149974.2	--	V	--
XLOC_017339	No	No	No	No	--	--	--	--
XLOC_017340	No	No	No	No	AFK09744.1	--	--	--
XLOC_017342	No	No	No	No	--	--	O	K03031
XLOC_017344	No	No	No	No	--	--	--	--
XLOC_017346	No	No	No	No	XP_008893640.1	--	--	--
XLOC_017349	DEG(M)	No	No	No	KEP55638.1	--	--	--
XLOC_017350	No	No	No	No	CCO27996.1	--	--	--
XLOC_017352	DEG(M)	No	DEG(I+M)	DEG(M)	--	B3	J	K02921
XLOC_017353	No	No	No	No	KEP47062.1	--	Q	--
XLOC_017354	No	No	No	No	--	--	C	--
XLOC_017356	No	No	No	No	--	--	--	--
XLOC_017359	No	No	No	No	XP_009537024.1	--	--	--

Differentially expressed gene (DEG) IDs	*C. microphyllum* root and shoot-specific (>2 fold)	*C. microphyllum* root and shoot-specific (>10 fold)	ICC4958 and *C. microphyllum* root-specific (>2 fold)	*C. microphyllum* root-specific (>2 fold)	Functional Annotation			
					NCBI nr IDs	TFs	KOG	KEGG
XLOC_017365	DEG(M)	No	No	No	ETI49322.1	--	--	K05607
XLOC_017366	DEG(M)	No	No	No	--	--	--	--
XLOC_017368	No	No	No	No	--	--	--	--
XLOC_017371	DEG(M)	No	No	No	KDN43479.1	--	--	--
XLOC_017382	No	No	No	No	ERG87338.1	--	J	--
XLOC_017384	No	No	No	No	--	--	--	--
XLOC_017387	DEG(M)	No	No	No	--	--	--	--
XLOC_017389	DEG(M)	No	No	No	ABM55583.1	--	J	K02894
XLOC_017392	No	No	No	No	AAU84932.1	--	--	--
XLOC_017393	No	No	No	No	--	--	R	--
XLOC_017395	No	No	No	No	ETI42493.1	--	--	K06126
XLOC_017398	No	No	No	No	--	--	--	--
XLOC_017399	No	No	No	No	--	--	--	--
XLOC_017400	No	No	No	No	--	--	--	--
XLOC_017401	DEG(M)	No	No	No	NP_001155823.1	--	J	K02949
XLOC_017402	No	No	DEG(I+M)	No	XP_012571730.1	--	--	--
XLOC_017406	No	No	No	No	NP_001280380.1	--	T	--
XLOC_017411	No	No	No	No	KEP50538.1	--	O	--
XLOC_017415	No	No	No	No	KDO68015.1	--	I	--
XLOC_017418	No	No	No	No	--	--	--	--
XLOC_017419	No	No	DEG(I+M)	No	XP_003627676.1	--	--	--
XLOC_017421	No	No	No	No	XP_002415663.1	--	A	K12890
XLOC_017422	No	No	No	No	--	--	--	--
XLOC_017423	DEG(M)	DEG(M)	No	No	--	--	--	--
XLOC_017426	No	No	No	No	XP_002510858.1	--	--	--
XLOC_017427	No	No	No	No	--	--	--	--
XLOC_017430	No	No	No	No	CEL61268.1	--	--	--
XLOC_017431	No	No	No	No	--	--	--	--
XLOC_017432	DEG(M)	No	No	No	--	--	--	--
XLOC_017433	No	No	No	No	--	--	J	--
XLOC_017436	No	No	No	No	KFQ63341.1	--	U	--
XLOC_017438	DEG(M)	DEG(M)	DEG(I+M)	DEG(M)	KDN38350.1	--	J	--
XLOC_017439	DEG(M)	DEG(M)	DEG(I+M)	DEG(M)	XP_008909337.1	--	J	--
XLOC_017440	DEG(M)	No	No	No	--	--	--	--
XLOC_017450	No	No	No	No	XP_008896731.1	--	--	--
XLOC_017451	No	No	No	No	--	--	--	--
XLOC_017457	DEG(M)	No	No	No	ABI52748.1	--	J	K02929
XLOC_017458	DEG(M)	No	No	No	CEL61780.1	--	--	--
XLOC_017460	No	No	No	No	EUC65327.1	--	--	--
XLOC_017461	DEG(M)	No	No	No	NP_001156291.1	--	J	--

Differentially expressed gene (DEG) IDs	*C. microphyllum* root and shoot-specific (>2 fold)	*C. microphyllum* root and shoot-specific (>10 fold)	ICC4958 and *C. microphyllum* root-specific (>2 fold)	*C. microphyllum* root-specific (>2 fold)	Functional Annotation			
					NCBI nr IDs	TFs	KOG	KEGG
XLOC_017468	No	No	No	No	--	--	--	--
XLOC_017475	No	No	No	No	NP_001156170.1	--	--	--
XLOC_017477	No	No	No	No	XP_001654419.1	--	--	--
XLOC_017478	No	No	No	No	--	--	--	--
XLOC_017486	No	No	No	No	--	--	J	K02883
XLOC_017491	No	No	No	No	AGE97632.1	--	--	--
XLOC_017493	No	No	No	No	XP_001649288.1	--	--	--
XLOC_017495	DEG(M)	No	No	No	ETP42624.1	--	--	--
XLOC_017497	No	No	No	No	--	--	--	--
XLOC_017500	No	No	No	No	XP_008898275.1	--	--	--
XLOC_017502	No	No	No	No	KDN39584.1	--	--	--
XLOC_017503	No	No	No	No	ETL95283.1	--	--	K07119
XLOC_017504	No	No	No	No	--	--	--	--
XLOC_017507	No	No	No	No	KDN39218.1	--	--	K00627
XLOC_017508	DEG(M)	DEG(M)	No	No	XP_003051732.1	--	--	--
XLOC_017512	DEG(M)	No	No	No	--	--	--	--
XLOC_017513	No	No	No	No	--	--	--	--
XLOC_017515	No	No	No	No	CEL52319.1	--	--	--
XLOC_017522	No	No	No	No	XP_001949588.1	FAR1	O	--
XLOC_017523	No	No	No	No	--	--	--	--
XLOC_017525	DEG(M)	No	No	No	--	--	--	--
XLOC_017527	No	No	No	No	XP_008891774.1	--	--	K02725
XLOC_017531	DEG(M)	No	No	No	--	--	--	--
XLOC_017532	No	No	No	No	--	--	--	--
XLOC_017533	No	No	No	No	--	--	--	--
XLOC_017535	No	No	No	No	--	--	--	--
XLOC_017541	DEG(M)	DEG(M)	DEG(I+M)	DEG(M)	--	--	--	--
XLOC_017542	No	No	No	No	--	--	--	K01188
XLOC_017543	No	No	No	No	--	--	--	K03260
XLOC_017545	DEG(M)	No	No	No	ETP44255.1	--	J	K02358
XLOC_017547	DEG(M)	No	No	No	CEL53244.1	--	--	--
XLOC_017564	DEG(M)	DEG(M)	DEG(I+M)	DEG(M)	Q9SML4.1	--	Q	--
XLOC_017565	DEG(M)	No	DEG(I+M)	DEG(M)	CAA10131.1	--	Q	--
XLOC_017566	DEG(M)	No	DEG(I+M)	DEG(M)	--	--	Q	K00660
XLOC_017567	DEG(M)	No	DEG(I+M)	DEG(M)	XP_004502226.1	--	Q	--
XLOC_017569	No	No	No	No	--	--	--	--
XLOC_017595	No	No	No	No	--	--	--	--
XLOC_017619	No	No	No	No	--	--	--	--
XLOC_017621	DEG(M)	No	DEG(I+M)	DEG(M)	--	--	--	--
XLOC_017647	No	No	No	No	--	--	--	--

Differentially expressed gene (DEG) IDs	*C. microphyllum* root and shoot-specific (>2 fold)	*C. microphyllum* root and shoot-specific (>10 fold)	ICC4958 and *C. microphyllum* root-specific (>2 fold)	*C. microphyllum* root-specific (>2 fold)	Functional Annotation			
					NCBI nr IDs	TFs	KOG	KEGG
XLOC_017648	No	No	No	No	NP_001156573.1	--	--	--
XLOC_017660	No	No	No	No	--	--	TZ	K00855
XLOC_017669	No	No	DEG(I+M)	No	--	--	--	--
XLOC_017697	No	No	No	No	--	--	--	--
XLOC_017698	No	No	No	No	--	--	--	--
XLOC_017719	No	No	No	No	--	--	--	--
XLOC_017725	No	No	No	No	EUC55551.1	--	--	--
XLOC_017728	DEG(M)	No	No	No	--	--	--	--
XLOC_017733	No	No	No	No	--	--	--	--
XLOC_017734	No	No	No	No	AKM98271.1	--	C	K02637
XLOC_017736	No	No	No	No	YP_002149758.1	--	X	K02704
XLOC_017738	No	No	No	No	--	--	--	--
XLOC_017739	No	No	No	No	--	--	--	K02709
XLOC_017746	No	No	DEG(I+M)	No	--	--	--	--
XLOC_017775	DEG(M)	No	No	No	--	--	--	K01051
XLOC_017777	No	No	No	No	--	--	--	--
XLOC_017780	No	No	No	No	--	--	--	--
XLOC_017783	No	No	No	No	--	--	--	--
XLOC_017785	No	No	No	No	--	--	--	--
XLOC_017791	DEG(M)	No	No	No	--	--	J	--
XLOC_017795	DEG(M)	No	No	No	--	--	--	--
XLOC_017796	No	No	No	No	--	--	--	--
XLOC_017801	No	No	No	No	XP_001650864.1	--	S	K18624
XLOC_017802	No	No	No	No	--	--	--	--
XLOC_017803	No	No	No	No	XP_002998461.1	--	O	--
XLOC_017806	No	No	No	No	--	--	--	--
XLOC_017808	No	No	No	No	--	--	--	--
XLOC_017811	No	No	No	No	--	--	--	--
XLOC_017814	No	No	No	No	CEL52278.1	--	--	--
XLOC_017816	No	No	No	No	--	--	--	--
XLOC_017818	No	No	No	No	--	--	--	--
XLOC_017819	DEG(M)	No	No	No	--	--	--	--
XLOC_017821	No	No	No	No	--	--	--	--
XLOC_017822	DEG(M)	No	No	No	CCO37585.1	--	--	--
XLOC_017823	No	No	No	No	KFM82349.1	--	A	--
XLOC_017827	No	No	No	No	--	--	--	--
XLOC_017829	No	No	No	No	--	--	--	--
XLOC_017830	No	No	No	No	--	--	--	--
XLOC_017831	DEG(M)	No	No	No	--	--	--	--
XLOC_017833	DEG(M)	No	No	No	--	--	--	--

Differentially expressed gene (DEG) IDs	*C. microphyllum* root and shoot-specific (>2 fold)	*C. microphyllum* root and shoot-specific (>10 fold)	ICC4958 and *C. microphyllum* root-specific (>2 fold)	*C. microphyllum* root-specific (>2 fold)	Functional Annotation			
					NCBI nr IDs	TFs	KOG	KEGG
XLOC_017834	No	No	No	No	XP_001942765.1	--	A	--
XLOC_017835	No	No	No	No	--	--	--	--
XLOC_017837	No	No	DEG(I+M)	No	--	--	--	--
XLOC_017838	No	No	No	No	--	--	--	--
XLOC_017848	No	No	No	No	KDN41447.1	--	--	--
XLOC_017854	No	No	No	No	XP_003050943.1	--	--	--
XLOC_017855	No	No	No	No	--	--	--	--
XLOC_017857	No	No	No	No	XP_004339766.1	--	J	--
XLOC_017859	No	No	No	No	--	--	--	--
XLOC_017862	DEG(M)	No	No	No	KFM74024.1	--	J	K02898
XLOC_017867	No	No	No	No	KFM69832.1	--	O	K17255
XLOC_017870	No	No	No	No	XP_008915725.1	--	--	K00413
XLOC_017872	No	No	No	No	DAA34308.1	--	B	--
XLOC_017873	No	No	No	No	--	--	--	--
XLOC_017874	No	No	No	No	--	--	--	--
XLOC_017875	No	No	No	No	XP_001660185.1	--	--	--
XLOC_017876	No	No	No	No	--	--	--	--
XLOC_017880	No	No	No	No	--	--	--	K02896
XLOC_017881	DEG(M)	No	No	No	--	FAR1	O	--
XLOC_017884	No	No	No	No	XP_001952163.2	--	--	--
XLOC_017887	DEG(M)	No	No	No	ELU43663.1	--	--	--
XLOC_017891	No	No	No	No	--	--	--	--
XLOC_017892	No	No	No	No	--	--	--	--
XLOC_017893	DEG(M)	DEG(M)	No	No	--	--	--	--
XLOC_017894	No	No	No	No	--	--	--	--
XLOC_017897	DEG(M)	DEG(M)	No	No	--	--	--	--
XLOC_017899	No	No	No	No	XP_003244734.1	--	--	--
XLOC_017903	DEG(M)	No	No	No	CCO26828.1	--	--	--
XLOC_017904	No	No	No	No	--	--	--	--
XLOC_017905	No	No	No	No	--	--	T	--
XLOC_017908	No	No	No	No	--	--	J	K02883
XLOC_017911	No	No	No	No	XP_004341325.1	--	J	--
XLOC_017913	No	No	No	No	--	--	--	--
XLOC_017916	No	No	No	No	--	--	--	--
XLOC_017919	No	No	No	No	--	--	--	--
XLOC_017922	No	No	No	No	--	--	--	--
XLOC_017923	No	No	No	No	--	--	--	--
XLOC_017924	DEG(M)	No	No	No	--	--	--	--
XLOC_017927	No	No	No	No	XP_002518220.1	--	C	--
XLOC_017932	No	No	No	No	EUC67316.1	--	--	K03869

Differentially expressed gene (DEG) IDs	*C. microphyllum* root and shoot-specific (>2 fold)	*C. microphyllum* root and shoot-specific (>10 fold)	ICC4958 and *C. microphyllum* root-specific (>2 fold)	*C. microphyllum* root-specific (>2 fold)	Functional Annotation			
					NCBI nr IDs	TFs	KOG	KEGG
XLOC_017933	No	No	No	No	--	--	--	--
XLOC_017934	No	No	No	No	XP_004494053.2	--	--	--
XLOC_017935	No	No	No	No	--	--	--	--
XLOC_017937	No	No	No	No	--	--	--	--
XLOC_017938	No	No	No	No	ETK76067.1	--	P	--
XLOC_017940	No	No	No	No	EUC67738.1	--	L	--
XLOC_017945	No	No	No	No	--	--	--	--
XLOC_017948	DEG(M)	No	No	No	ACF75907.1	--	O	K04079
XLOC_017957	No	No	No	No	XP_001942878.2	--	--	--
XLOC_017960	No	No	DEG(I+M)	No	--	--	--	--
XLOC_017961	DEG(M)	No	No	No	ETL32610.1	--	--	--
XLOC_017962	No	No	No	No	--	--	--	--
XLOC_017965	DEG(M)	No	No	No	XP_012079990.1	MIKC	J	K02900
XLOC_017966	No	No	No	No	--	--	--	--
XLOC_017967	No	No	No	No	--	--	--	--
XLOC_017968	DEG(M)	No	No	No	ETK87061.1	--	--	--
XLOC_017969	DEG(M)	No	No	No	CEL60787.1	--	--	--
XLOC_017976	DEG(M)	No	No	No	--	--	--	--
XLOC_017977	No	No	No	No	--	--	--	--
XLOC_017985	No	No	No	No	--	--	--	--
XLOC_017988	DEG(M)	No	No	No	AGL52554.1	--	C	--
XLOC_017996	No	No	No	No	--	--	--	--
XLOC_017997	No	No	No	No	--	--	--	--
XLOC_018003	No	No	No	No	XP_003044754.1	--	--	--
XLOC_018007	DEG(M)	DEG(M)	DEG(I+M)	DEG(M)	XP_002901678.1	--	J	K02912
XLOC_018025	DEG(M)	No	DEG(I+M)	DEG(M)	--	--	--	--
XLOC_018027	DEG(M)	No	No	No	--	--	--	--
XLOC_018028	DEG(M)	No	No	No	EUC66712.1	--	--	--
XLOC_018030	DEG(M)	No	No	No	--	--	--	--
XLOC_018031	No	No	No	No	XP_009529225.1	--	C	--
XLOC_018032	No	No	No	No	XP_009529225.1	--	--	--
XLOC_018036	DEG(M)	DEG(M)	DEG(I+M)	DEG(M)	XP_008902178.1	--	--	--
XLOC_018039	No	No	No	No	--	--	--	K14411
XLOC_018040	DEG(M)	DEG(M)	DEG(I+M)	DEG(M)	--	--	--	--
XLOC_018042	DEG(M)	No	No	No	AGC56217.1	NAC	J	--
XLOC_018044	No	No	No	No	XP_003049713.1	--	--	--
XLOC_018045	No	No	No	No	XP_003049713.1	--	--	--
XLOC_018047	No	No	No	No	XP_003303951.1	--	--	--
XLOC_018048	No	No	No	No	EUC57203.1	--	--	--
XLOC_018049	DEG(M)	No	No	No	--	--	--	--

Differentially expressed gene (DEG) IDs	*C. microphyllum* root and shoot-specific (>2 fold)	*C. microphyllum* root and shoot-specific (>10 fold)	ICC4958 and *C. microphyllum* root-specific (>2 fold)	*C. microphyllum* root-specific (>2 fold)	Functional Annotation			
					NCBI nr IDs	TFs	KOG	KEGG
XLOC_018050	No	No	No	No	EUC64193.1	--	--	K05021
XLOC_018052	No	No	No	No	--	--	--	--
XLOC_018053	No	No	No	No	--	--	C	--
XLOC_018059	DEG(M)	No	No	No	--	--	--	--
XLOC_018061	No	No	No	No	--	--	--	--
XLOC_018065	DEG(M)	No	No	No	--	--	--	--
XLOC_018078	No	No	No	No	CEL58018.1	--	--	--
XLOC_018080	DEG(M)	No	No	No	XP_004504591.1	--	O	--
XLOC_018081	No	No	No	No	--	--	--	--
XLOC_018085	DEG(M)	No	DEG(I+M)	DEG(M)	--	--	--	--
XLOC_018090	No	No	No	No	EFN88778.1	--	T	--
XLOC_018094	DEG(M)	DEG(M)	DEG(I+M)	DEG(M)	--	--	--	--
XLOC_018096	No	No	No	No	--	--	--	--
XLOC_018099	DEG(M)	No	No	No	CCO36240.1	--	--	--
XLOC_018101	No	No	No	No	--	--	--	--
XLOC_018102	DEG(M)	No	No	No	XP_002907678.1	--	--	--
XLOC_018107	DEG(M)	No	No	No	KDN41928.1	--	--	--
XLOC_018108	No	No	No	No	KDN41928.1	--	--	--
XLOC_018112	No	No	No	No	XP_003052665.1	--	--	--
XLOC_018113	DEG(M)	No	No	No	--	--	--	--
XLOC_018118	DEG(M)	No	No	No	XP_008913706.1	--	--	--
XLOC_018121	No	No	No	No	--	--	--	--
XLOC_018122	No	No	No	No	XP_003042980.1	--	--	--
XLOC_018123	DEG(M)	No	No	No	XP_003050991.1	--	--	--
XLOC_018124	DEG(M)	No	No	No	EUC66235.1	--	--	--
XLOC_018125	No	No	No	No	EUC66235.1	--	--	--
XLOC_018126	DEG(M)	No	No	No	--	--	--	--
XLOC_018127	DEG(M)	No	DEG(I+M)	DEG(M)	CEL59065.1	--	J	--
XLOC_018128	No	No	No	No	--	--	--	--
XLOC_018129	DEG(M)	No	No	No	KEP50011.1	NF-YC	B	--
XLOC_018130	No	No	No	No	KEP50011.1	NF-YC	B	--
XLOC_018134	DEG(M)	DEG(M)	DEG(I+M)	DEG(M)	ETL44382.1	--	--	K02978
XLOC_018139	DEG(M)	No	No	No	CEP13548.1	--	A	K11594
XLOC_018140	No	No	No	No	ELU37054.1	--	--	--
XLOC_018142	DEG(M)	No	No	No	--	--	--	--
XLOC_018143	No	No	No	No	--	--	--	--
XLOC_018144	DEG(M)	No	No	No	--	--	--	--
XLOC_018145	DEG(M)	No	No	No	--	--	--	--
XLOC_018147	No	No	No	No	--	--	X	--
XLOC_018152	DEG(M)	No	No	No	ADK90073.1	--	J	--

Differentially expressed gene (DEG) IDs	*C. microphyllum* root and shoot-specific (>2 fold)	*C. microphyllum* root and shoot-specific (>10 fold)	ICC4958 and *C. microphyllum* root-specific (>2 fold)	*C. microphyllum* root-specific (>2 fold)	Functional Annotation			
					NCBI nr IDs	TFs	KOG	KEGG
XLOC_018155	No	No	No	No	CCO30302.1	--	--	--
XLOC_018160	No	No	No	No	--	--	K	K03626
XLOC_018161	No	No	No	No	NP_001156301.1	--	K	K03626
XLOC_018166	No	No	No	No	--	--	--	--
XLOC_018167	No	No	No	No	--	--	--	--
XLOC_018168	No	No	No	No	BAL46049.1	--	C	--
XLOC_018169	DEG(M)	DEG(M)	DEG(I+M)	DEG(M)	--	--	--	--
XLOC_018176	DEG(M)	No	No	No	ELU44240.1	--	B	--
XLOC_018178	No	No	No	No	--	--	--	--
XLOC_018180	No	No	No	No	XP_008889762.1	--	C	--
XLOC_018181	DEG(M)	No	No	No	--	--	--	--
XLOC_018182	No	No	No	No	--	--	--	--
XLOC_018184	No	No	No	No	--	--	--	--
XLOC_018185	DEG(M)	No	No	No	--	--	--	--
XLOC_018186	No	No	No	No	--	--	--	K12824
XLOC_018189	No	No	No	No	XP_008903298.1	--	--	--
XLOC_018190	DEG(M)	No	No	No	--	--	O	--
XLOC_018201	No	No	No	No	--	--	--	--
XLOC_018202	No	No	No	No	--	--	--	--
XLOC_018204	No	No	No	No	CCO32169.1	--	--	--
XLOC_018207	No	No	No	No	--	--	--	--
XLOC_018209	No	No	No	No	--	--	J	--
XLOC_018214	No	No	No	No	--	--	--	--
XLOC_018216	DEG(M)	No	No	No	--	--	J	--
XLOC_018217	No	No	No	No	XP_008911648.1	--	C	--
XLOC_018219	No	No	No	No	--	--	--	--
XLOC_018220	DEG(M)	No	No	No	BAJ85990.1	--	J	--
XLOC_018221	No	No	No	No	XP_001653087.1	--	J	--
XLOC_018222	No	No	No	No	--	--	--	--
XLOC_018224	No	No	No	No	--	--	R	--
XLOC_018228	No	No	No	No	--	--	--	--
XLOC_018230	No	No	No	No	AEV66511.1	--	--	--
XLOC_018233	No	No	No	No	--	--	--	--
XLOC_018240	No	No	No	No	XP_001658105.1	--	J	K03254
XLOC_018241	No	No	No	No	--	--	T	--
XLOC_018242	No	No	No	No	--	--	--	--
XLOC_018245	No	No	No	No	--	--	--	--
XLOC_018248	No	No	No	No	--	--	--	--
XLOC_018252	DEG(M)	No	No	No	--	--	--	--
XLOC_018253	DEG(M)	No	No	No	--	--	--	--

Differentially expressed gene (DEG) IDs	*C. microphyllum* root and shoot-specific (>2 fold)	*C. microphyllum* root and shoot-specific (>10 fold)	ICC4958 and *C. microphyllum* root-specific (>2 fold)	*C. microphyllum* root-specific (>2 fold)	Functional Annotation			
					NCBI nr IDs	TFs	KOG	KEGG
XLOC_018255	DEG(M)	No	No	No	--	--	--	--
XLOC_018256	No	No	No	No	XP_008904936.1	--	--	K16280
XLOC_018257	No	No	No	No	--	--	--	--
XLOC_018258	No	No	No	No	--	--	--	--
XLOC_018260	DEG(M)	No	DEG(I+M)	DEG(M)	--	--	--	--
XLOC_018261	No	No	No	No	--	--	--	--
XLOC_018266	No	No	No	No	CCO27098.1	--	--	--
XLOC_018270	No	No	No	No	ETL43778.1	--	--	K09527
XLOC_018271	No	No	No	No	KEP55727.1	--	--	--
XLOC_018272	No	No	No	No	EUC67487.1	--	--	--
XLOC_018273	No	No	No	No	XP_008181973.1	--	--	--
XLOC_018281	DEG(M)	No	No	No	--	--	--	--
XLOC_018282	No	No	No	No	--	--	--	--
XLOC_018283	No	No	No	No	--	--	--	--
XLOC_018284	No	No	No	No	--	--	--	--
XLOC_018289	No	No	No	No	--	--	--	--
XLOC_018293	No	No	No	No	--	--	--	--
XLOC_018295	DEG(M)	No	No	No	--	--	--	--
XLOC_018296	DEG(M)	DEG(M)	No	No	--	--	--	K02924
XLOC_018298	No	No	No	No	--	--	--	--
XLOC_018299	No	No	DEG(I+M)	No	--	--	--	--
XLOC_018300	No	No	No	No	CEL63479.1	--	--	--
XLOC_018302	No	No	No	No	XP_001951538.1	--	U	K12194
XLOC_018311	DEG(M)	No	No	No	KHJ43032.1	--	J	--
XLOC_018313	No	No	No	No	NP_001155797.1	--	--	--
XLOC_018315	No	No	No	No	--	--	--	--
XLOC_018316	No	No	No	No	CCO27367.1	--	--	--
XLOC_018318	No	No	No	No	CEL51981.1	--	--	--
XLOC_018319	No	No	No	No	--	--	--	--
XLOC_018320	No	No	No	No	XP_003045875.1	--	--	--
XLOC_018322	No	No	No	No	--	--	--	K12349
XLOC_018325	No	No	No	No	--	--	--	--
XLOC_018331	No	No	No	No	--	--	--	--
XLOC_018332	No	No	No	No	--	--	--	--
XLOC_018340	No	No	No	No	--	--	--	--
XLOC_018341	No	No	No	No	--	--	--	--
XLOC_018343	DEG(M)	No	No	No	XP_009539106.1	--	--	K04078
XLOC_018345	No	No	No	No	--	--	--	--
XLOC_018346	DEG(M)	No	DEG(I+M)	DEG(M)	--	--	--	--
XLOC_018347	No	No	No	No	XP_003044960.1	--	--	--

Differentially expressed gene (DEG) IDs	*C. microphyllum* root and shoot-specific (>2 fold)	*C. microphyllum* root and shoot-specific (>10 fold)	ICC4958 and *C. microphyllum* root-specific (>2 fold)	*C. microphyllum* root-specific (>2 fold)	Functional Annotation			
					NCBI nr IDs	TFs	KOG	KEGG
XLOC_018348	No	No	No	No	--	--	--	--
XLOC_018351	No	No	No	No	KEP49039.1	--	--	--
XLOC_018354	DEG(M)	No	No	No	AAL40718.1	--	Z	--
XLOC_018357	DEG(M)	No	No	No	--	--	--	--
XLOC_018358	No	No	No	No	--	--	--	--
XLOC_018360	DEG(M)	No	No	No	AJI44486.1	--	--	--
XLOC_018362	No	No	No	No	--	--	--	--
XLOC_018365	No	No	No	No	--	--	--	K02934
XLOC_018368	No	No	No	No	--	--	--	K09580
XLOC_018375	No	No	No	No	--	--	--	--
XLOC_018379	No	No	No	No	--	--	--	--
XLOC_018383	No	No	No	No	XP_003043293.1	--	--	--
XLOC_018384	No	No	No	No	XP_003043293.1	--	--	--
XLOC_018385	No	No	No	No	XP_003043293.1	--	--	--
XLOC_018386	No	No	No	No	EUC53790.1	--	T	--
XLOC_018388	DEG(M)	No	No	No	XP_008892153.1	--	--	K07393
XLOC_018389	No	No	No	No	XP_001659999.1	--	--	--
XLOC_018390	No	No	No	No	ACZ67203.1	--	O	--
XLOC_018391	No	No	No	No	--	--	O	--
XLOC_018392	DEG(M)	No	No	No	--	--	--	--
XLOC_018393	DEG(M)	No	No	No	XP_009523936.1	--	J	K02998
XLOC_018395	No	No	No	No	XP_001946297.2	--	--	--
XLOC_018404	No	No	No	No	NP_001233020.1	--	A	--
XLOC_018412	No	No	No	No	CEL52621.1	--	--	--
XLOC_018413	No	No	No	No	--	--	--	--
XLOC_018414	No	No	No	No	--	--	--	--
XLOC_018421	No	No	No	No	--	ZF-HD	--	--
XLOC_018422	No	No	No	No	--	--	--	--
XLOC_018424	No	No	No	No	--	--	--	--
XLOC_018430	No	No	No	No	XP_008903309.1	--	--	--
XLOC_018439	DEG(M)	DEG(M)	No	No	AFO70211.1	--	O	--
XLOC_018447	No	No	No	No	--	bHLH	--	--
XLOC_018449	No	No	No	No	--	--	--	--
XLOC_018450	No	No	No	No	--	--	--	--
XLOC_018459	No	No	No	No	--	--	O	K03798
XLOC_018489	DEG(M)	DEG(M)	No	No	--	--	--	--
XLOC_018497	No	No	No	No	--	--	--	--
XLOC_018505	No	No	No	No	--	--	--	--
XLOC_018510	No	No	No	No	XP_012567234.1	--	--	--
XLOC_018511	DEG(M)	DEG(M)	No	No	--	--	--	--

Differentially expressed gene (DEG) IDs	*C. microphyllum* root and shoot-specific (>2 fold)	*C. microphyllum* root and shoot-specific (>10 fold)	ICC4958 and *C. microphyllum* root-specific (>2 fold)	*C. microphyllum* root-specific (>2 fold)	Functional Annotation			
					NCBI nr IDs	TFs	KOG	KEGG
XLOC_018517	DEG(M)	No	No	No	--	--	--	--
XLOC_018523	DEG(M)	DEG(M)	DEG(I+M)	DEG(M)	--	--	--	--
XLOC_018524	DEG(M)	No	No	No	--	--	--	--
XLOC_018525	DEG(M)	DEG(M)	DEG(I+M)	DEG(M)	--	--	--	--
XLOC_018526	DEG(M)	No	No	No	--	--	--	--
XLOC_018541	DEG(M)	No	No	No	EUC63850.1	--	J	--
XLOC_018542	DEG(M)	No	No	No	ELU38673.1	--	J	--
XLOC_018544	DEG(M)	No	No	No	CCO35926.1	NAC	J	--
XLOC_018545	No	No	No	No	CCO35926.1	NAC	J	--
XLOC_018546	No	No	No	No	ELU38673.1	NAC	J	--
XLOC_018561	No	No	No	No	--	--	--	--
XLOC_018571	No	No	No	No	--	--	--	K02516
XLOC_018577	No	No	No	No	XP_012568096.1	--	--	--
XLOC_018583	DEG(M)	DEG(M)	DEG(I+M)	DEG(M)	--	--	--	--
XLOC_018584	DEG(M)	DEG(M)	No	No	ELC06673.1	--	--	--
XLOC_018599	DEG(M)	DEG(M)	No	No	--	--	--	--
XLOC_018620	DEG(M)	No	DEG(I+M)	DEG(M)	--	--	--	--
XLOC_018636	DEG(M)	No	DEG(I+M)	DEG(M)	CCO37497.1	--	--	--
XLOC_018637	DEG(M)	No	No	No	CCO37497.1	--	--	--
XLOC_018638	DEG(M)	DEG(M)	DEG(I+M)	DEG(M)	EUC64469.1	--	--	--
XLOC_018640	DEG(M)	No	No	No	--	--	--	--
XLOC_018641	DEG(M)	No	No	No	--	--	W	--
XLOC_018656	No	No	No	No	--	--	--	--
XLOC_018657	No	No	No	No	--	--	--	--
XLOC_018658	No	No	No	No	ETP35633.1	--	--	--
XLOC_018659	No	No	No	No	XP_002530955.1	--	R	K03327
XLOC_018660	No	No	No	No	ETP12974.1	--	--	--
XLOC_018662	No	No	No	No	XP_008184997.1	--	--	--
XLOC_018663	No	No	No	No	XP_008179401.1	--	--	--
XLOC_018664	No	No	No	No	--	--	--	--
XLOC_018666	No	No	No	No	CEL63203.1	--	--	--
XLOC_018667	No	No	No	No	XP_001649125.1	--	C	--
XLOC_018669	No	No	No	No	KJB38415.1	--	LT	K12119
XLOC_018672	No	No	DEG(I+M)	No	--	--	--	--
XLOC_018675	No	No	No	No	--	--	--	--
XLOC_018678	DEG(M)	No	DEG(I+M)	DEG(M)	--	--	--	--
XLOC_018688	DEG(M)	No	No	No	XP_008184848.1	--	J	K02989
XLOC_018691	No	No	No	No	EUC58591.1	--	R	K04536
XLOC_018693	No	No	No	No	--	--	--	--
XLOC_018694	No	No	No	No	--	--	--	--

Differentially expressed gene (DEG) IDs	*C. microphyllum* root and shoot-specific (>2 fold)	*C. microphyllum* root and shoot-specific (>10 fold)	ICC4958 and *C. microphyllum* root-specific (>2 fold)	*C. microphyllum* root-specific (>2 fold)	Functional Annotation			
					NCBI nr IDs	TFs	KOG	KEGG
XLOC_018698	DEG(M)	No	No	No	--	--	--	--
XLOC_018700	No	No	No	No	XP_002907039.1	--	U	K07953
XLOC_018704	No	No	DEG(I+M)	No	--	--	--	--
XLOC_018706	DEG(M)	No	No	No	KDN39552.1	--	J	--
XLOC_018710	DEG(M)	No	No	No	--	--	--	--
XLOC_018712	DEG(M)	No	No	No	XP_007846109.1	--	--	--
XLOC_018716	No	No	No	No	XP_009515579.1	--	J	--
XLOC_018717	DEG(M)	No	No	No	--	--	--	--
XLOC_018718	No	No	No	No	XP_008914900.1	--	C	--
XLOC_018720	DEG(M)	No	No	No	EUC57148.1	--	--	--
XLOC_018722	No	No	No	No	--	--	--	--
XLOC_018733	DEG(M)	No	DEG(I+M)	DEG(M)	--	--	--	--
XLOC_018737	DEG(M)	No	No	No	--	--	J	--
XLOC_018740	DEG(M)	No	No	No	XP_001656939.1	--	J	K02942
XLOC_018745	No	No	No	No	--	--	--	--
XLOC_018747	No	No	No	No	KEP50118.1	--	--	--
XLOC_018749	No	No	No	No	--	--	--	--
XLOC_018752	DEG(M)	No	No	No	--	--	--	--
XLOC_018753	No	No	No	No	KEP49369.1	--	--	--
XLOC_018759	No	No	No	No	XP_009535964.1	--	--	--
XLOC_018761	No	No	No	No	--	--	--	--
XLOC_018762	No	No	No	No	XP_008890008.1	--	C	--
XLOC_018764	No	No	No	No	--	--	F	--
XLOC_018767	DEG(M)	No	No	No	--	--	--	--
XLOC_018768	No	No	No	No	--	--	--	--
XLOC_018770	No	No	No	No	--	--	--	--
XLOC_018775	DEG(M)	No	No	No	EXK46994.1	--	P	--
XLOC_018776	No	No	No	No	AGI59311.1	--	--	--
XLOC_018777	DEG(M)	No	No	No	XP_001654856.1	--	J	--
XLOC_018778	No	No	No	No	XP_001952564.1	--	--	--
XLOC_018779	No	No	No	No	--	--	--	--
XLOC_018782	No	No	No	No	--	--	--	--
XLOC_018783	DEG(M)	No	No	No	--	--	--	--
XLOC_018785	No	No	No	No	--	--	--	--
XLOC_018786	No	No	No	No	CCO35823.1	--	--	--
XLOC_018787	No	No	No	No	XP_001951763.1	--	TU	--
XLOC_018790	No	No	No	No	XP_003044018.1	--	--	--
XLOC_018791	No	No	No	No	XP_013303820.1	--	C	--
XLOC_018792	No	No	DEG(I+M)	No	XP_004507824.1	MYB	--	K09422
XLOC_018793	DEG(M)	No	No	No	--	--	--	--

Differentially expressed gene (DEG) IDs	*C. microphyllum* root and shoot-specific (>2 fold)	*C. microphyllum* root and shoot-specific (>10 fold)	ICC4958 and *C. microphyllum* root-specific (>2 fold)	*C. microphyllum* root-specific (>2 fold)	Functional Annotation			
					NCBI nr IDs	TFs	KOG	KEGG
XLOC_018798	DEG(M)	No	No	No	YP_001795377.1	--	--	--
XLOC_018800	DEG(M)	No	No	No	--	--	--	--
XLOC_018801	DEG(M)	DEG(M)	DEG(I+M)	DEG(M)	XP_010911507.1	--	UO	--
XLOC_018809	No	No	No	No	XP_002525441.1	--	R	K00059
XLOC_018812	DEG(M)	No	No	No	--	--	--	--
XLOC_018814	No	No	No	No	--	--	--	--
XLOC_018815	No	No	No	No	EUC67115.1	--	C	K05581
XLOC_018817	DEG(M)	No	DEG(I+M)	DEG(M)	KKP05512.1	--	J	--
XLOC_018818	DEG(M)	No	No	No	--	--	--	--
XLOC_018819	No	No	No	No	--	--	E	K15227
XLOC_018820	No	No	No	No	--	--	--	--
XLOC_018821	No	No	No	No	BAJ88019.1	--	U	--
XLOC_018822	No	No	No	No	BAH70799.1	--	--	--
XLOC_018825	DEG(M)	No	No	No	--	--	--	--
XLOC_018830	DEG(M)	No	No	No	EUC53781.1	--	--	--
XLOC_018831	DEG(M)	No	No	No	KFM83326.1	--	J	K02964
XLOC_018833	No	No	No	No	--	--	--	--
XLOC_018834	DEG(M)	DEG(M)	No	No	EGU84611.1	--	--	--
XLOC_018836	No	No	No	No	KEP49547.1	--	--	--
XLOC_018837	No	No	No	No	XP_008902309.1	--	--	--
XLOC_018842	No	No	No	No	--	--	--	--
XLOC_018843	No	No	No	No	--	--	--	--
XLOC_018845	DEG(M)	No	No	No	--	--	--	--
XLOC_018846	No	No	No	No	--	--	E	--
XLOC_018847	No	No	No	No	ETI45427.1	--	--	K03248
XLOC_018851	No	No	No	No	XP_004493218.1	--	P	--
XLOC_018852	No	No	No	No	--	--	--	K02916
XLOC_018854	No	No	No	No	--	--	--	--
XLOC_018855	DEG(M)	No	No	No	--	--	--	--
XLOC_018864	DEG(M)	No	No	No	--	--	--	--
XLOC_018865	No	No	No	No	--	--	--	--
XLOC_018866	No	No	No	No	--	--	--	K08903
XLOC_018870	DEG(M)	No	No	No	--	--	--	--
XLOC_018875	DEG(M)	DEG(M)	DEG(I+M)	DEG(M)	--	--	--	--
XLOC_018879	No	No	No	No	XP_004344078.1	--	H	--
XLOC_018880	No	No	No	No	XP_001950896.2	--	--	--
XLOC_018884	No	No	No	No	--	--	--	--
XLOC_018892	No	No	No	No	EUC64854.1	--	--	--
XLOC_018894	DEG(M)	DEG(M)	DEG(I+M)	DEG(M)	XP_008911987.1	--	--	K02875
XLOC_018902	No	No	No	No	--	--	--	--

Differentially expressed gene (DEG) IDs	*C. microphyllum* root and shoot-specific (>2 fold)	*C. microphyllum* root and shoot-specific (>10 fold)	ICC4958 and *C. microphyllum* root-specific (>2 fold)	*C. microphyllum* root-specific (>2 fold)	Functional Annotation			
					NCBI nr IDs	TFs	KOG	KEGG
XLOC_018903	No	No	No	No	XP_004494761.1	--	RI	--
XLOC_018905	No	No	No	No	--	--	--	--
XLOC_018906	No	No	DEG(I+M)	No	--	--	--	--
XLOC_018915	No	No	No	No	--	--	--	--
XLOC_018917	No	No	No	No	CEL61812.1	--	--	--
XLOC_018918	No	No	No	No	--	MIKC	R	--
XLOC_018920	No	No	No	No	--	--	--	--
XLOC_018922	DEG(M)	No	No	No	XP_001650232.1	--	J	K02894
XLOC_018924	No	No	No	No	CEL56367.1	--	--	--
XLOC_018932	DEG(M)	No	DEG(I+M)	DEG(M)	CCO36789.1	--	--	--
XLOC_018937	No	No	DEG(I+M)	No	XP_004513863.1	--	--	--
XLOC_018940	No	No	No	No	ETK81806.1	--	--	--
XLOC_018941	No	No	No	No	KDN51894.1	--	H	--
XLOC_018950	No	No	No	No	--	--	--	--
XLOC_018953	DEG(M)	No	No	No	EUC57291.1	--	--	--
XLOC_018954	DEG(M)	No	No	No	EUC57291.1	--	--	--
XLOC_018964	No	No	No	No	--	--	--	--
XLOC_018965	No	No	No	No	EGU81053.1	--	--	--
XLOC_018966	No	No	No	No	AAS82856.1	--	--	--
XLOC_018968	DEG(M)	No	DEG(I+M)	DEG(M)	CCO31877.1	--	J	--
XLOC_018969	DEG(M)	No	No	No	--	--	--	--
XLOC_018975	No	No	No	No	--	--	--	--
XLOC_018976	No	No	No	No	--	--	--	--
XLOC_018982	No	No	No	No	--	--	--	--
XLOC_018983	No	No	DEG(I+M)	No	XP_004501532.1	--	OR	--
XLOC_018988	No	No	No	No	EUC64805.1	--	--	--
XLOC_018993	DEG(M)	DEG(M)	No	No	AHW40374.1	--	C	--
XLOC_018994	DEG(M)	No	No	No	NP_001156206.1	--	J	K02873
XLOC_018995	DEG(M)	No	DEG(I+M)	DEG(M)	XP_007384133.1	MYB_related	O	--
XLOC_018999	No	No	No	No	CEL58757.1	--	--	--
XLOC_019000	No	No	No	No	KEP50506.1	FAR1	GM	K08678
XLOC_019003	No	No	No	No	ADV40163.1	--	C	K00235
XLOC_019004	No	No	No	No	XP_003054466.1	--	--	K03006
XLOC_019006	No	No	No	No	EUC58784.1	--	--	--
XLOC_019007	No	No	No	No	CCO27168.1	--	--	K08343
XLOC_019010	No	No	No	No	--	--	--	--
XLOC_019011	DEG(M)	No	No	No	CAK18211.1	--	O	--
XLOC_019012	No	No	No	No	--	--	--	--
XLOC_019013	No	No	No	No	--	--	I	--
XLOC_019015	DEG(M)	No	No	No	--	--	--	--

Differentially expressed gene (DEG) IDs	*C. microphyllum* root and shoot-specific (>2 fold)	*C. microphyllum* root and shoot-specific (>10 fold)	ICC4958 and *C. microphyllum* root-specific (>2 fold)	*C. microphyllum* root-specific (>2 fold)	Functional Annotation			
					NCBI nr IDs	TFs	KOG	KEGG
XLOC_019018	DEG(M)	No	No	No	--	--	--	--
XLOC_019023	No	No	No	No	--	--	--	--
XLOC_019025	DEG(M)	No	No	No	EUC57503.1	--	--	--
XLOC_019026	No	No	No	No	ETP55064.1	--	--	--
XLOC_019028	No	No	No	No	EUC56253.1	--	--	--
XLOC_019030	No	No	No	No	--	--	--	--
XLOC_019031	No	No	No	No	--	--	G	--
XLOC_019033	No	No	No	No	BAG84133.1	--	--	--
XLOC_019040	No	No	No	No	ELU43171.1	--	H	K00652
XLOC_019042	No	No	No	No	EUC67491.1	--	J	--
XLOC_019044	No	No	No	No	--	--	--	--
XLOC_019045	No	No	No	No	--	--	--	--
XLOC_019058	No	No	No	No	XP_003046195.1	--	--	--
XLOC_019059	No	No	No	No	XP_003046195.1	--	--	--
XLOC_019060	No	No	No	No	XP_001647937.1	--	C	--
XLOC_019062	DEG(M)	No	No	No	--	--	--	--
XLOC_019070	DEG(M)	No	No	No	NP_001119684.1	--	J	K02927
XLOC_019071	DEG(M)	No	No	No	CCO32504.1	--	--	--
XLOC_019075	No	No	No	No	CEL58313.1	--	--	--
XLOC_019076	DEG(M)	No	No	No	--	--	--	--
XLOC_019083	DEG(M)	No	No	No	XP_001661827.1	--	J	K02958
XLOC_019086	DEG(M)	No	No	No	CCJ09440.1	--	J	--
XLOC_019087	DEG(M)	No	No	No	--	--	J	--
XLOC_019093	No	No	No	No	--	--	--	--
XLOC_019097	No	No	DEG(I+M)	No	--	--	--	--
XLOC_019105	DEG(M)	No	No	No	XP_001950614.2	--	--	--
XLOC_019106	No	No	No	No	XP_012571424.1	--	R	K18121
XLOC_019115	No	No	No	No	CCO27102.1	--	K	K15731
XLOC_019120	No	No	No	No	--	--	--	--
XLOC_019125	No	No	No	No	XP_003726477.1	--	TU	K07874
XLOC_019130	No	No	No	No	--	--	--	--
XLOC_019131	No	No	No	No	--	--	--	--
XLOC_019132	No	No	No	No	CCO30879.1	--	--	--
XLOC_019134	No	No	No	No	--	--	--	K03247
XLOC_019136	No	No	No	No	XP_001950499.2	--	O	--
XLOC_019138	No	No	No	No	--	--	--	--
XLOC_019139	No	No	No	No	--	--	--	--
XLOC_019140	No	No	No	No	--	--	--	--
XLOC_019142	No	No	No	No	--	--	--	--
XLOC_019148	No	No	No	No	NP_001155506.1	--	A	K12870

Differentially expressed gene (DEG) IDs	*C. microphyllum* root and shoot-specific (>2 fold)	*C. microphyllum* root and shoot-specific (>10 fold)	ICC4958 and *C. microphyllum* root-specific (>2 fold)	*C. microphyllum* root-specific (>2 fold)	Functional Annotation			
					NCBI nr IDs	TFs	KOG	KEGG
XLOC_019150	No	No	No	No	CEL61594.1	--	--	--
XLOC_019151	DEG(M)	No	No	No	XP_008904770.1	--	--	--
XLOC_019153	No	No	No	No	EXL52710.1	--	--	--
XLOC_019158	No	No	No	No	XP_002513024.1	--	X	--
XLOC_019160	No	No	No	No	--	--	--	--
XLOC_019162	No	No	No	No	KEP55364.1	--	--	--
XLOC_019163	No	No	No	No	CEL57252.1	--	--	--
XLOC_019167	No	No	DEG(I+M)	No	--	--	G	--
XLOC_019170	No	No	No	No	XP_002512475.1	--	J	K03254
XLOC_019172	No	No	No	No	--	--	--	--
XLOC_019174	DEG(M)	No	No	No	XP_011139878.1	--	J	--
XLOC_019176	No	No	No	No	XP_003051586.1	--	--	--
XLOC_019177	No	No	No	No	--	--	--	--
XLOC_019178	No	No	No	No	--	--	--	--
XLOC_019181	No	No	No	No	--	--	S	--
XLOC_019182	No	No	No	No	--	--	--	--
XLOC_019183	No	No	No	No	--	--	W	--
XLOC_019185	No	No	No	No	ETI53424.1	--	--	--
XLOC_019187	No	No	No	No	--	--	--	--
XLOC_019192	No	No	No	No	AHE41509.1	--	C	K02689
XLOC_019195	DEG(M)	No	No	No	XP_001950838.2	--	--	--
XLOC_019197	No	No	No	No	--	--	--	--
XLOC_019198	No	No	DEG(I+M)	No	--	--	S	--
XLOC_019204	No	No	No	No	XP_003042636.1	--	--	--
XLOC_019205	No	No	No	No	XP_003042636.1	--	--	--
XLOC_019212	No	No	No	No	AAA29350.1	--	O	--
XLOC_019215	No	No	No	No	--	--	--	--
XLOC_019217	DEG(M)	No	No	No	--	--	R	--
XLOC_019218	No	No	No	No	XP_001949004.1	--	E	K00819
XLOC_019219	No	No	DEG(I+M)	No	XP_004495114.1	LBD	--	--
XLOC_019227	No	No	No	No	--	--	--	--
XLOC_019228	No	No	No	No	KEP52011.1	--	--	--
XLOC_019229	No	No	No	No	XP_009533918.1	--	O	--
XLOC_019231	DEG(M)	No	No	No	--	--	--	--
XLOC_019232	No	No	No	No	XP_012572301.1	--	--	--
XLOC_019233	No	No	No	No	ETO70361.1	--	--	--
XLOC_019237	No	No	No	No	KHN01920.1	--	C	K00413
XLOC_019238	DEG(M)	DEG(M)	No	No	EWY90744.1	--	--	--
XLOC_019239	No	No	No	No	XP_001951488.2	--	--	--
XLOC_019242	No	No	No	No	XP_004488025.1	--	--	K13947

Differentially expressed gene (DEG) IDs	*C. microphyllum* root and shoot-specific (>2 fold)	*C. microphyllum* root and shoot-specific (>10 fold)	ICC4958 and *C. microphyllum* root-specific (>2 fold)	*C. microphyllum* root-specific (>2 fold)	Functional Annotation			
					NCBI nr IDs	TFs	KOG	KEGG
XLOC_019243	No	No	No	No	XP_008179407.1	--	C	--
XLOC_019244	No	No	No	No	--	--	--	--
XLOC_019245	No	No	No	No	XP_003048782.1	--	--	--
XLOC_019246	No	No	DEG(I+M)	No	--	--	--	--
XLOC_019249	No	No	No	No	XP_001950254.2	--	G	--
XLOC_019253	No	No	No	No	--	--	--	--
XLOC_019254	No	No	No	No	--	--	--	--
XLOC_019256	No	No	No	No	EXL99178.1	--	--	--
XLOC_019258	No	No	No	No	--	--	--	--
XLOC_019260	DEG(M)	No	No	No	--	--	--	--
XLOC_019262	DEG(M)	No	No	No	--	--	--	--
XLOC_019267	No	No	No	No	CCO26476.1	--	O	K10576
XLOC_019268	DEG(M)	No	No	No	--	--	--	K02265
XLOC_019276	No	No	No	No	XP_008912872.1	--	--	--
XLOC_019281	No	No	No	No	--	--	--	--
XLOC_019283	DEG(M)	No	No	No	WP_033319250.1	--	O	--
XLOC_019287	No	No	No	No	AIL48440.1	--	J	--
XLOC_019297	No	No	No	No	--	--	--	--
XLOC_019301	No	No	No	No	--	--	--	--
XLOC_019304	DEG(M)	No	DEG(I+M)	DEG(M)	--	--	--	--
XLOC_019305	DEG(M)	No	No	No	--	--	--	K09422
XLOC_019310	No	No	No	No	XP_003044703.1	--	R	--
XLOC_019312	DEG(M)	No	No	No	--	--	--	--
XLOC_019316	No	No	No	No	--	--	--	--
XLOC_019322	DEG(M)	No	DEG(I+M)	DEG(M)	KEP54610.1	--	--	--
XLOC_019324	DEG(M)	No	No	No	ETK91798.1	--	--	--
XLOC_019331	DEG(M)	No	No	No	--	--	--	--
XLOC_019335	DEG(M)	DEG(M)	No	No	EWG43848.1	--	--	--
XLOC_019336	No	No	No	No	XP_003052166.1	--	--	--
XLOC_019339	No	No	No	No	CCO35349.1	--	--	--
XLOC_019342	DEG(M)	No	No	No	--	--	--	--
XLOC_019344	DEG(M)	DEG(M)	No	No	EGU75640.1	--	--	--
XLOC_019345	No	No	No	No	--	--	--	--
XLOC_019348	No	No	No	No	--	--	TU	--
XLOC_019351	No	No	No	No	--	--	--	--
XLOC_019352	DEG(M)	No	No	No	--	--	--	--
XLOC_019355	No	No	No	No	--	--	--	--
XLOC_019358	No	No	No	No	CDJ90032.1	--	C	--
XLOC_019364	No	No	No	No	CEL54220.1	--	--	--
XLOC_019367	No	No	No	No	--	--	Z	K10365

Differentially expressed gene (DEG) IDs	*C. microphyllum* root and shoot-specific (>2 fold)	*C. microphyllum* root and shoot-specific (>10 fold)	ICC4958 and *C. microphyllum* root-specific (>2 fold)	*C. microphyllum* root-specific (>2 fold)	Functional Annotation			
					NCBI nr IDs	TFs	KOG	KEGG
XLOC_019368	No	No	No	No	--	--	--	--
XLOC_019369	No	No	No	No	--	--	--	--
XLOC_019372	No	No	No	No	--	--	--	--
XLOC_019374	DEG(M)	No	No	No	--	--	J	--
XLOC_019375	No	No	No	No	--	--	--	--
XLOC_019376	No	No	No	No	--	--	--	--
XLOC_019377	No	No	No	No	--	--	--	--
XLOC_019381	No	No	No	No	--	--	--	--
XLOC_019383	DEG(M)	No	No	No	--	--	--	--
XLOC_019384	No	No	No	No	--	--	--	--
XLOC_019386	DEG(M)	DEG(M)	DEG(I+M)	DEG(M)	XP_004515450.1	--	--	K01183
XLOC_019389	DEG(M)	No	No	No	--	--	--	--
XLOC_019395	No	No	No	No	CEL53135.1	--	--	--
XLOC_019396	No	No	No	No	--	--	--	--
XLOC_019401	No	No	No	No	ETL94413.1	--	--	--
XLOC_019402	No	No	No	No	ETI47843.1	--	--	--
XLOC_019410	DEG(M)	No	No	No	XP_003050843.1	--	--	--
XLOC_019413	DEG(M)	DEG(M)	No	No	--	--	--	--
XLOC_019417	No	No	No	No	--	--	--	--
XLOC_019424	No	No	No	No	NP_001156708.1	--	--	--
XLOC_019425	DEG(M)	No	No	No	EUC62197.1	--	--	--
XLOC_019427	No	No	No	No	--	--	--	K13065
XLOC_019429	DEG(M)	DEG(M)	DEG(I+M)	DEG(M)	--	--	--	--
XLOC_019431	DEG(M)	No	No	No	--	--	--	--
XLOC_019433	No	No	No	No	--	--	--	--
XLOC_019434	No	No	No	No	--	--	--	--
XLOC_019435	No	No	No	No	XP_003246082.1	--	C	K02147
XLOC_019436	No	No	No	No	--	--	--	--
XLOC_019439	No	No	No	No	CCO26505.1	--	--	--
XLOC_019442	No	No	No	No	--	--	--	--
XLOC_019447	No	No	No	No	KFM58711.1	--	A	K12614
XLOC_019449	DEG(M)	No	No	No	--	--	--	--
XLOC_019450	No	No	No	No	--	--	--	--
XLOC_019451	No	No	DEG(I+M)	No	--	--	--	--
XLOC_019452	No	No	No	No	--	--	--	--
XLOC_019455	No	No	DEG(I+M)	No	CAC08209.2	--	--	--
XLOC_019456	No	No	DEG(I+M)	No	--	--	--	--
XLOC_019457	No	No	No	No	XP_008022801.1	--	--	--
XLOC_019462	DEG(M)	No	No	No	--	--	--	--
XLOC_019463	DEG(M)	DEG(M)	DEG(I+M)	DEG(M)	--	--	--	--

Differentially expressed gene (DEG) IDs	*C. microphyllum* root and shoot-specific (>2 fold)	*C. microphyllum* root and shoot-specific (>10 fold)	ICC4958 and *C. microphyllum* root-specific (>2 fold)	*C. microphyllum* root-specific (>2 fold)	Functional Annotation			
					NCBI nr IDs	TFs	KOG	KEGG
XLOC_019464	No	No	No	No	--	--	--	--
XLOC_019477	No	No	No	No	XP_001948977.1	--	O	K09495
XLOC_019479	No	No	No	No	--	--	--	--
XLOC_019481	No	No	No	No	--	--	--	K09201
XLOC_019482	No	No	No	No	XP_003611284.1	--	E	--
XLOC_019483	No	No	No	No	--	--	--	--
XLOC_019484	No	No	No	No	ETM52941.1	--	--	--
XLOC_019485	DEG(M)	DEG(M)	No	No	EWG42705.1	--	RT	--
XLOC_019487	No	No	No	No	EUC66658.1	--	--	--
XLOC_019493	No	No	No	No	--	--	--	--
XLOC_019494	No	No	DEG(I+M)	No	--	--	--	K01859
XLOC_019498	No	No	No	No	XP_001650898.1	--	O	--
XLOC_019499	No	No	No	No	--	--	RK	--
XLOC_019500	No	No	No	No	KFM73388.1	--	U	K17087
XLOC_019501	No	No	No	No	--	--	--	--
XLOC_019503	No	No	No	No	XP_001949691.2	--	--	--
XLOC_019504	No	No	No	No	AJC97119.1	--	U	--
XLOC_019506	DEG(M)	No	No	No	--	--	--	--
XLOC_019508	No	No	No	No	--	--	--	--
XLOC_019509	No	No	No	No	XP_004488332.1	--	KR	K15032
XLOC_019510	No	No	No	No	ETM46030.1	--	--	--
XLOC_019514	DEG(M)	No	No	No	--	--	--	--
XLOC_019517	DEG(M)	No	No	No	--	--	--	--
XLOC_019518	No	No	No	No	XP_006383916.1	--	O	K05907
XLOC_019520	No	No	No	No	--	--	--	--
XLOC_019530	DEG(M)	No	No	No	ETK94697.1	--	--	--
XLOC_019533	DEG(M)	No	No	No	EUC62578.1	--	--	--
XLOC_019541	No	No	No	No	--	--	--	--
XLOC_019542	No	No	No	No	EDL81548.1	--	C	--
XLOC_019543	No	No	No	No	KFM56835.1	--	C	--
XLOC_019546	No	No	No	No	EUC64899.1	--	--	--
XLOC_019549	No	No	No	No	KDN37051.1	--	--	--
XLOC_019552	No	No	No	No	--	--	--	K12160
XLOC_019555	No	No	No	No	--	--	R	--
XLOC_019556	No	No	No	No	--	--	--	--
XLOC_019557	No	No	No	No	KDN46215.1	--	--	--
XLOC_019558	No	No	No	No	XP_004498593.1	--	--	--
XLOC_019560	No	No	No	No	XP_002529080.1	--	Q	--
XLOC_019562	No	No	DEG(I+M)	No	--	--	--	--
XLOC_019566	No	No	No	No	XP_001650849.1	--	U	--

Differentially expressed gene (DEG) IDs	*C. microphyllum* root and shoot-specific (>2 fold)	*C. microphyllum* root and shoot-specific (>10 fold)	ICC4958 and *C. microphyllum* root-specific (>2 fold)	*C. microphyllum* root-specific (>2 fold)	Functional Annotation			
					NCBI nr IDs	TFs	KOG	KEGG
XLOC_019568	No	No	No	No	--	--	--	--
XLOC_019569	No	No	No	No	XP_004499534.1	--	X	K02717
XLOC_019576	No	No	No	No	--	--	--	--
XLOC_019578	No	No	No	No	--	--	J	K02899
XLOC_019586	No	No	No	No	--	--	--	--
XLOC_019587	No	No	No	No	--	--	--	--
XLOC_019590	No	No	No	No	--	--	--	--
XLOC_019591	No	No	No	No	--	--	--	--
XLOC_019594	DEG(M)	No	No	No	EXM21073.1	--	--	--
XLOC_019597	DEG(M)	No	DEG(I+M)	DEG(M)	--	--	--	--
XLOC_019600	No	No	No	No	--	--	--	--
XLOC_019601	No	No	No	No	XP_002520136.1	--	C	--
XLOC_019602	DEG(M)	DEG(M)	No	No	--	--	--	--
XLOC_019605	DEG(M)	DEG(M)	DEG(I+M)	DEG(M)	XP_008898328.1	--	J	--
XLOC_019606	DEG(M)	No	DEG(I+M)	DEG(M)	XP_008898328.1	--	J	--
XLOC_019614	DEG(M)	No	No	No	--	--	--	--
XLOC_019623	No	No	No	No	--	--	--	--
XLOC_019625	DEG(M)	No	No	No	XP_008897085.1	--	J	K02877
XLOC_019631	No	No	No	No	EMD90380.1	--	--	--
XLOC_019632	DEG(M)	No	No	No	ABF46823.1	--	Z	--
XLOC_019636	DEG(M)	No	No	No	--	--	--	--
XLOC_019637	DEG(M)	No	No	No	--	--	--	--
XLOC_019638	No	No	No	No	--	--	--	--
XLOC_019640	No	No	DEG(I+M)	No	--	--	--	--
XLOC_019641	No	No	No	No	--	--	--	--
XLOC_019679	No	No	No	No	--	--	--	--
XLOC_019718	DEG(M)	No	No	No	XP_003049909.1	--	P	--
XLOC_019719	DEG(M)	DEG(M)	No	No	EXK46994.1	--	P	--
XLOC_019721	DEG(M)	No	No	No	EMT64458.1	--	P	--
XLOC_019774	DEG(M)	No	DEG(I+M)	DEG(M)	--	--	--	--
XLOC_019775	No	No	DEG(I+M)	No	--	--	I	--
XLOC_019800	DEG(M)	No	No	No	EWY84522.1	--	--	--
XLOC_019801	DEG(M)	No	No	No	XP_003048457.1	--	--	--
XLOC_019802	DEG(M)	No	No	No	XP_003048457.1	--	--	--
XLOC_019835	No	No	No	No	--	--	--	--
XLOC_019848	DEG(M)	No	No	No	KEP49443.1	--	--	--
XLOC_019849	No	No	No	No	EUC58409.1	--	--	--
XLOC_019850	DEG(M)	DEG(M)	DEG(I+M)	DEG(M)	KEP49157.1	--	--	--
XLOC_019851	No	No	No	No	KDN38341.1	--	--	--
XLOC_019852	No	No	No	No	EUC58409.1	--	--	--

Differentially expressed gene (DEG) IDs	*C. microphyllum* root and shoot-specific (>2 fold)	*C. microphyllum* root and shoot-specific (>10 fold)	ICC4958 and *C. microphyllum* root-specific (>2 fold)	*C. microphyllum* root-specific (>2 fold)	Functional Annotation			
					NCBI nr IDs	TFs	KOG	KEGG
XLOC_019853	No	No	No	No	KEP46235.1	--	--	--
XLOC_019854	No	No	No	No	KEP46235.1	--	--	--
XLOC_019855	No	No	No	No	KDN38341.1	--	--	--
XLOC_019856	DEG(M)	DEG(M)	DEG(I+M)	DEG(M)	KEP49583.1	--	--	--
XLOC_019857	DEG(M)	No	No	No	--	--	--	--
XLOC_019858	DEG(M)	DEG(M)	DEG(I+M)	DEG(M)	EUC56251.1	--	--	--
XLOC_019859	DEG(M)	No	No	No	--	--	--	--
XLOC_019860	DEG(M)	No	DEG(I+M)	DEG(M)	KEP49583.1	--	--	--
XLOC_019861	DEG(M)	DEG(M)	DEG(I+M)	DEG(M)	EUC59275.1	--	--	--
XLOC_019899	DEG(M)	DEG(M)	DEG(I+M)	DEG(M)	XP_004493928.1	--	O	K00799
XLOC_019946	DEG(M)	No	DEG(I+M)	DEG(M)	XP_008908505.1	--	C	--
XLOC_019949	No	No	No	No	ELU45744.1	--	C	--
XLOC_019950	DEG(M)	No	No	No	XP_008908505.1	--	C	--
XLOC_019952	DEG(M)	No	DEG(I+M)	DEG(M)	KDN51052.1	--	C	--
XLOC_019954	DEG(M)	DEG(M)	DEG(I+M)	DEG(M)	--	--	--	--
XLOC_019955	DEG(M)	No	DEG(I+M)	DEG(M)	ELU45744.1	--	--	--
XLOC_019956	No	No	DEG(I+M)	No	--	--	--	--
XLOC_019965	No	No	No	No	--	--	--	K02721
XLOC_019980	DEG(M)	DEG(M)	DEG(I+M)	DEG(M)	--	--	--	--
XLOC_019981	DEG(M)	DEG(M)	DEG(I+M)	DEG(M)	CCO36393.1	--	EJ	--
XLOC_019982	DEG(M)	No	No	No	EEB91771.1	--	--	--
XLOC_019983	DEG(M)	No	DEG(I+M)	DEG(M)	CCO36393.1	--	--	--
XLOC_019984	DEG(M)	No	No	No	EEB91771.1	--	--	--
XLOC_019986	DEG(M)	DEG(M)	DEG(I+M)	DEG(M)	EEB91771.1	--	--	--
XLOC_019987	DEG(M)	DEG(M)	No	No	CEL54514.1	--	--	--
XLOC_019988	DEG(M)	No	No	No	CCO36393.1	--	--	--
XLOC_019989	DEG(M)	No	DEG(I+M)	DEG(M)	CEL54514.1	--	--	--
XLOC_019990	DEG(M)	No	No	No	EEB91771.1	--	--	--
XLOC_019991	DEG(M)	No	No	No	EEB91771.1	--	--	--
XLOC_019992	DEG(M)	No	No	No	EEB91771.1	--	--	--
XLOC_019993	DEG(M)	No	No	No	CEL54514.1	--	--	--
XLOC_019994	DEG(M)	No	DEG(I+M)	DEG(M)	EUC65989.1	--	EJ	--
XLOC_019995	DEG(M)	No	No	No	CEL54514.1	--	--	--
XLOC_020002	No	No	DEG(I+M)	No	--	bHLH	KT	--
XLOC_020003	No	No	No	No	--	--	--	--
XLOC_020019	DEG(M)	DEG(M)	No	No	XP_012568444.1	--	--	--
XLOC_020021	No	No	No	No	XP_012568444.1	--	--	--
XLOC_020022	No	No	No	No	XP_012568444.1	--	--	K07409
XLOC_020023	No	No	No	No	--	--	--	--
XLOC_020026	No	No	No	No	--	--	--	--

Differentially expressed gene (DEG) IDs	*C. microphyllum* root and shoot-specific (>2 fold)	*C. microphyllum* root and shoot-specific (>10 fold)	ICC4958 and *C. microphyllum* root-specific (>2 fold)	*C. microphyllum* root-specific (>2 fold)	Functional Annotation			
					NCBI nr IDs	TFs	KOG	KEGG
XLOC_020027	No	No	No	No	--	--	--	--
XLOC_020028	DEG(M)	No	No	No	--	--	--	--
XLOC_020029	DEG(M)	No	No	No	--	--	--	--
XLOC_020037	DEG(M)	DEG(M)	DEG(I+M)	DEG(M)	AGX83786.1	--	J	--
XLOC_020039	No	No	DEG(I+M)	No	AJD86488.1	--	J	--
XLOC_020040	No	No	DEG(I+M)	No	AJW67422.1	--	J	--
XLOC_020041	DEG(M)	DEG(M)	No	No	AJD86488.1	--	J	--
XLOC_020043	DEG(M)	No	DEG(I+M)	DEG(M)	GAN00818.1	--	J	--
XLOC_020044	DEG(M)	No	No	No	CEL58096.1	--	J	--
XLOC_020045	DEG(M)	No	No	No	CDH51885.1	--	J	K03231
XLOC_020046	DEG(M)	No	No	No	AFV07287.1	--	J	--
XLOC_020050	DEG(M)	DEG(M)	DEG(I+M)	DEG(M)	ETL93490.1	--	J	--
XLOC_020057	DEG(M)	DEG(M)	No	No	--	--	--	--
XLOC_020058	DEG(M)	DEG(M)	No	No	XP_004502101.1	--	--	--
XLOC_020067	No	No	No	No	CCO29922.1	--	C	K02132
XLOC_020069	DEG(M)	DEG(M)	DEG(I+M)	DEG(M)	XP_003054197.1	--	C	--
XLOC_020071	DEG(M)	No	No	No	ENH60795.1	--	C	--
XLOC_020072	DEG(M)	No	No	No	AHZ64879.2	--	C	K02132
XLOC_020080	No	No	No	No	--	--	--	--
XLOC_020094	No	No	No	No	--	--	--	--
XLOC_020129	DEG(M)	DEG(M)	No	No	--	--	C	--
XLOC_020131	DEG(M)	No	No	No	--	--	--	--
XLOC_020134	DEG(M)	DEG(M)	DEG(I+M)	DEG(M)	XP_004486932.1	--	--	--
XLOC_020135	No	No	DEG(I+M)	No	XP_004507133.1	--	--	--
XLOC_020137	No	No	DEG(I+M)	No	XP_004507133.1	--	--	--
XLOC_020138	DEG(M)	No	DEG(I+M)	DEG(M)	XP_004507318.1	--	--	K01188
XLOC_020139	No	No	DEG(I+M)	No	--	--	--	--
XLOC_020140	No	No	DEG(I+M)	No	--	--	--	--
XLOC_020141	DEG(M)	DEG(M)	DEG(I+M)	DEG(M)	XP_004507123.1	--	--	K01188
XLOC_020144	DEG(M)	DEG(M)	No	No	--	TCP	G	--
XLOC_020148	DEG(M)	No	No	No	XP_003555775.2	--	--	--
XLOC_020170	DEG(M)	No	No	No	XP_008891372.1	--	O	K03283
XLOC_020172	No	No	No	No	ETP20292.1	--	O	--
XLOC_020176	DEG(M)	No	No	No	EUC60696.1	--	O	--
XLOC_020177	DEG(M)	DEG(M)	DEG(I+M)	DEG(M)	ELU38685.1	MYB_related	O	--
XLOC_020200	DEG(M)	DEG(M)	No	No	--	--	--	--
XLOC_020215	DEG(M)	No	DEG(I+M)	DEG(M)	--	--	--	K13457
XLOC_020218	No	No	No	No	--	--	--	--
XLOC_020219	DEG(M)	No	No	No	--	--	--	--
XLOC_020222	No	No	No	No	KDN35801.1	--	O	K13113

Differentially expressed gene (DEG) IDs	*C. microphyllum* root and shoot-specific (>2 fold)	*C. microphyllum* root and shoot-specific (>10 fold)	ICC4958 and *C. microphyllum* root-specific (>2 fold)	*C. microphyllum* root-specific (>2 fold)	Functional Annotation			
					NCBI nr IDs	TFs	KOG	KEGG
XLOC_020225	No	No	No	No	CEL62102.1	--	--	--
XLOC_020226	No	No	No	No	--	--	--	--
XLOC_020227	DEG(M)	No	No	No	AAU94664.1	--	O	--
XLOC_020228	No	No	No	No	EUC63330.1	--	--	--
XLOC_020229	No	No	No	No	EUC62911.1	--	--	--
XLOC_020230	No	No	No	No	--	--	--	--
XLOC_020234	No	No	No	No	--	--	--	--
XLOC_020235	No	No	No	No	--	--	--	--
XLOC_020237	No	No	No	No	--	--	--	--
XLOC_020242	No	No	No	No	KDN45384.1	--	--	--
XLOC_020243	No	No	No	No	--	--	--	--
XLOC_020245	No	No	No	No	--	--	--	--
XLOC_020247	DEG(M)	No	No	No	--	--	--	--
XLOC_020254	No	No	No	No	--	--	--	--
XLOC_020255	No	No	No	No	CEL51569.1	--	--	--
XLOC_020258	No	No	No	No	--	--	GT	--
XLOC_020260	No	No	No	No	CEL62477.1	--	--	--
XLOC_020261	No	No	No	No	--	--	--	--
XLOC_020262	No	No	DEG(I+M)	No	--	bHLH	--	--
XLOC_020263	DEG(M)	DEG(M)	DEG(I+M)	DEG(M)	--	--	--	--
XLOC_020264	DEG(M)	No	DEG(I+M)	DEG(M)	XP_004496638.1	bHLH	--	--
XLOC_020265	No	No	No	No	--	--	--	--
XLOC_020266	DEG(M)	No	No	No	--	--	--	--
XLOC_020271	DEG(M)	No	No	No	XP_002903085.1	--	J	K02966
XLOC_020279	DEG(M)	DEG(M)	No	No	XP_011316840.1	--	C	--
XLOC_020281	DEG(M)	No	No	No	ACL36923.1	--	T	--
XLOC_020282	DEG(M)	No	No	No	NP_001155712.1	--	T	--
XLOC_020292	DEG(M)	No	No	No	ETI53147.1	--	--	--
XLOC_020293	DEG(M)	No	No	No	XP_008901045.1	--	--	--
XLOC_020294	No	No	No	No	EGU86011.1	--	--	--
XLOC_020300	DEG(M)	No	No	No	XP_007766516.1	--	J	--
XLOC_020305	DEG(M)	No	No	No	NP_001155658.1	--	--	--
XLOC_020307	DEG(M)	No	No	No	--	--	--	--
XLOC_020308	DEG(M)	No	No	No	--	--	--	--
XLOC_020309	No	No	No	No	--	--	J	--
XLOC_020310	No	No	No	No	--	--	--	--
XLOC_020311	No	No	No	No	--	--	--	--
XLOC_020313	No	No	No	No	--	--	--	--
XLOC_020315	DEG(M)	No	No	No	--	--	--	--
XLOC_020316	DEG(M)	No	No	No	--	--	--	--

Differentially expressed gene (DEG) IDs	*C. microphyllum* root and shoot-specific (>2 fold)	*C. microphyllum* root and shoot-specific (>10 fold)	ICC4958 and *C. microphyllum* root-specific (>2 fold)	*C. microphyllum* root-specific (>2 fold)	Functional Annotation			
					NCBI nr IDs	TFs	KOG	KEGG
XLOC_020318	No	No	No	No	XP_004497726.1	--	RI	--
XLOC_020322	No	No	No	No	CAU15484.1	--	O	--
XLOC_020324	DEG(M)	No	No	No	KDN38550.1	--	--	--
XLOC_020325	DEG(M)	No	No	No	KEP50067.1	--	--	--
XLOC_020327	DEG(M)	No	No	No	KDN43037.1	--	--	--
XLOC_020329	DEG(M)	No	No	No	EGU82657.1	--	--	--
XLOC_020330	No	No	DEG(I+M)	No	CAA66109.3	--	--	--
XLOC_020332	No	No	No	No	XP_004501999.1	--	--	--
XLOC_020333	DEG(M)	No	No	No	--	--	--	--
XLOC_020342	No	No	No	No	CCO29860.1	--	--	K09578
XLOC_020349	No	No	No	No	ELU42092.1	--	--	--
XLOC_020350	No	No	No	No	XP_003044454.1	--	--	--
XLOC_020351	DEG(M)	No	No	No	--	--	--	--
XLOC_020354	No	No	No	No	CCO33258.1	--	--	--
XLOC_020357	No	No	No	No	KDN49439.1	--	--	--
XLOC_020359	DEG(M)	DEG(M)	DEG(I+M)	DEG(M)	EGU77966.1	--	--	--
XLOC_020360	No	No	No	No	--	--	--	--
XLOC_020361	No	No	No	No	--	--	--	--
XLOC_020363	No	No	No	No	XP_001653914.1	--	--	--
XLOC_020366	DEG(M)	No	No	No	--	--	--	K01527
XLOC_020367	No	No	No	No	--	--	--	--
XLOC_020376	No	No	No	No	--	--	--	--
XLOC_020377	No	No	No	No	--	--	--	K00763
XLOC_020378	No	No	No	No	--	--	--	--
XLOC_020379	DEG(M)	No	No	No	XP_001664050.1	--	--	--
XLOC_020380	No	No	No	No	--	--	--	--
XLOC_020388	No	No	No	No	XP_001659750.1	--	O	--
XLOC_020389	DEG(M)	DEG(M)	DEG(I+M)	DEG(M)	--	--	--	--
XLOC_020390	No	No	No	No	XP_004495839.1	--	--	--
XLOC_020392	DEG(M)	No	No	No	--	--	--	--
XLOC_020396	No	No	DEG(I+M)	No	XP_004507603.1	--	--	--
XLOC_020399	No	No	No	No	--	--	--	--
XLOC_020400	No	No	No	No	--	--	--	--
XLOC_020410	No	No	No	No	XP_003139952.1	--	J	K02866
XLOC_020414	No	No	DEG(I+M)	No	--	--	I	K03921
XLOC_020415	No	No	No	No	--	--	--	--
XLOC_020416	No	No	No	No	--	--	--	--
XLOC_020417	No	No	No	No	--	--	--	--
XLOC_020423	No	No	No	No	CCO28336.1	--	--	--
XLOC_020424	No	No	No	No	--	--	--	--

Differentially expressed gene (DEG) IDs	*C. microphyllum* root and shoot-specific (>2 fold)	*C. microphyllum* root and shoot-specific (>10 fold)	ICC4958 and *C. microphyllum* root-specific (>2 fold)	*C. microphyllum* root-specific (>2 fold)	Functional Annotation			
					NCBI nr IDs	TFs	KOG	KEGG
XLOC_020425	DEG(M)	No	No	No	--	--	--	--
XLOC_020430	No	No	No	No	EUC55094.1	--	--	--
XLOC_020432	DEG(M)	No	No	No	--	--	--	--
XLOC_020433	No	No	No	No	--	--	--	--
XLOC_020438	No	No	No	No	--	--	C	K02150
XLOC_020449	DEG(M)	DEG(M)	DEG(I+M)	DEG(M)	ABC85737.1	--	Z	--
XLOC_020452	DEG(M)	No	No	No	XP_003043586.1	--	--	--
XLOC_020453	DEG(M)	No	No	No	AAU94654.1	--	J	--
XLOC_020454	No	No	No	No	--	--	J	K02865
XLOC_020455	No	No	No	No	--	--	J	--
XLOC_020458	No	No	No	No	--	--	--	K02959
XLOC_020466	DEG(M)	DEG(M)	DEG(I+M)	DEG(M)	EUC56860.1	--	--	--
XLOC_020473	DEG(M)	DEG(M)	No	No	--	--	--	K03098
XLOC_020476	DEG(M)	No	No	No	KEP52133.1	--	--	--
XLOC_020479	No	No	No	No	--	--	--	--
XLOC_020481	No	No	No	No	--	--	--	--
XLOC_020482	DEG(M)	DEG(M)	No	No	--	--	--	--
XLOC_020496	DEG(M)	DEG(M)	DEG(I+M)	DEG(M)	--	--	--	--
XLOC_020502	DEG(M)	No	DEG(I+M)	DEG(M)	NP_001235974.1	--	P	--
XLOC_020504	DEG(M)	DEG(M)	DEG(I+M)	DEG(M)	--	--	--	--
XLOC_020506	No	No	No	No	EUC57148.1	--	--	--
XLOC_020513	DEG(M)	No	DEG(I+M)	DEG(M)	--	--	--	--
XLOC_020516	No	No	No	No	AAU84930.1	--	Z	--
XLOC_020525	DEG(M)	DEG(M)	No	No	--	--	--	--
XLOC_020532	No	No	No	No	BAK03012.1	--	C	--
XLOC_020535	No	No	DEG(I+M)	No	--	bHLH	--	--
XLOC_020541	DEG(M)	No	No	No	--	--	--	--
XLOC_020544	DEG(M)	No	No	No	--	--	--	--
XLOC_020545	No	No	No	No	--	--	--	--
XLOC_020551	No	No	No	No	XP_001950499.2	--	O	--
XLOC_020555	No	No	No	No	EUC67782.1	--	--	--
XLOC_020557	No	No	No	No	--	--	--	K01366
XLOC_020566	No	No	No	No	--	--	--	--
XLOC_020571	No	No	No	No	--	--	--	--
XLOC_020574	DEG(M)	No	No	No	KHN84287.1	--	J	--
XLOC_020580	No	No	No	No	XP_008911306.1	--	--	K17081
XLOC_020587	No	No	DEG(I+M)	No	--	--	--	--
XLOC_020590	DEG(M)	No	No	No	KDN48666.1	--	--	--
XLOC_020591	DEG(M)	No	No	No	ELU40988.1	--	--	--
XLOC_020592	DEG(M)	No	No	No	AGR53502.1	E2F/DP	J	K02923

Differentially expressed gene (DEG) IDs	*C. microphyllum* root and shoot-specific (>2 fold)	*C. microphyllum* root and shoot-specific (>10 fold)	ICC4958 and *C. microphyllum* root-specific (>2 fold)	*C. microphyllum* root-specific (>2 fold)	Functional Annotation			
					NCBI nr IDs	TFs	KOG	KEGG
XLOC_020593	DEG(M)	No	No	No	--	--	--	--
XLOC_020594	DEG(M)	No	No	No	--	--	--	--
XLOC_020602	No	No	No	No	CCO29303.1	--	--	--
XLOC_020609	No	No	No	No	ACA62216.1	--	R	--
XLOC_020612	No	No	No	No	--	--	--	--
XLOC_020616	No	No	No	No	--	--	--	--
XLOC_020618	No	No	No	No	--	--	--	--
XLOC_020619	No	No	No	No	--	--	--	--
XLOC_020620	No	No	No	No	--	--	--	--
XLOC_020632	No	No	No	No	--	--	--	--
XLOC_020643	DEG(M)	No	No	No	--	--	--	--
XLOC_020651	No	No	No	No	ETM35477.1	--	G	K01835
XLOC_020652	DEG(M)	No	No	No	ETM35477.1	--	--	--
XLOC_020656	No	No	No	No	XP_004491943.1	--	--	--
XLOC_020676	No	No	No	No	--	--	C	K02115
XLOC_020686	No	No	No	No	--	--	--	--
XLOC_020689	DEG(M)	DEG(M)	No	No	XP_003053763.1	--	O	--
XLOC_020690	DEG(M)	No	DEG(I+M)	DEG(M)	EMT61507.1	--	O	--
XLOC_020710	DEG(M)	No	No	No	XP_003051714.1	--	--	--
XLOC_020714	DEG(M)	No	No	No	XP_009524111.1	--	C	K01899
XLOC_020715	No	No	No	No	XP_002908607.1	--	C	K01899
XLOC_020716	No	No	No	No	--	--	G	K05298
XLOC_020724	No	No	No	No	--	--	--	--
XLOC_020725	DEG(M)	DEG(M)	No	No	--	--	--	--
XLOC_020727	No	No	DEG(I+M)	No	XP_004497605.1	--	G	K01623
XLOC_020729	DEG(M)	No	No	No	EUC63368.1	--	O	--
XLOC_020730	DEG(M)	No	No	No	EUC63368.1	--	O	K05864
XLOC_020736	DEG(M)	No	No	No	EUC58457.1	--	--	--
XLOC_020738	No	No	No	No	--	--	--	--
XLOC_020740	DEG(M)	No	No	No	--	--	--	--
XLOC_020744	No	No	No	No	--	--	--	--
XLOC_020745	No	No	No	No	--	--	--	--
XLOC_020746	No	No	No	No	--	--	--	--
XLOC_020754	DEG(M)	DEG(M)	No	No	XP_001936044.1	--	J	K03236
XLOC_020760	DEG(M)	No	No	No	--	--	--	--
XLOC_020761	DEG(M)	No	No	No	CCO36676.1	--	--	--
XLOC_020764	No	No	No	No	--	--	--	K00799
XLOC_020767	DEG(M)	No	DEG(I+M)	DEG(M)	XP_003048775.1	--	J	--
XLOC_020768	No	No	No	No	--	--	--	--
XLOC_020770	No	No	No	No	--	--	--	--

Differentially expressed gene (DEG) IDs	*C. microphyllum* root and shoot-specific (>2 fold)	*C. microphyllum* root and shoot-specific (>10 fold)	ICC4958 and *C. microphyllum* root-specific (>2 fold)	*C. microphyllum* root-specific (>2 fold)	Functional Annotation			
					NCBI nr IDs	TFs	KOG	KEGG
XLOC_020771	No	No	No	No	AKJ85499.1	--	IR	--
XLOC_020772	DEG(M)	No	No	No	--	--	--	--
XLOC_020775	No	No	No	No	--	--	--	--
XLOC_020784	No	No	No	No	XP_004503979.1	--	Q	K00083
XLOC_020801	DEG(M)	No	No	No	CCO30393.1	--	--	--
XLOC_020802	No	No	No	No	--	--	--	--
XLOC_020805	DEG(M)	No	No	No	--	--	--	--
XLOC_020806	DEG(M)	DEG(M)	DEG(I+M)	DEG(M)	KKA73802.1	Dof	Z	--
XLOC_020807	No	No	No	No	EUC64077.1	--	--	--
XLOC_020808	DEG(M)	DEG(M)	DEG(I+M)	DEG(M)	--	--	--	--
XLOC_020809	DEG(M)	No	No	No	--	--	--	--
XLOC_020815	DEG(M)	DEG(M)	DEG(I+M)	DEG(M)	--	--	--	--
XLOC_020816	DEG(M)	DEG(M)	DEG(I+M)	DEG(M)	--	--	--	--
XLOC_020821	No	No	No	No	--	--	--	--
XLOC_020824	No	No	No	No	--	--	--	--
XLOC_020825	DEG(M)	No	No	No	--	--	--	--
XLOC_020831	No	No	No	No	--	--	--	K13464
XLOC_020833	No	No	No	No	CEL52042.1	--	--	--
XLOC_020834	No	No	No	No	CCO28799.1	--	--	--
XLOC_020841	No	No	DEG(I+M)	No	XP_004516078.1	--	--	--
XLOC_020851	DEG(M)	DEG(M)	No	No	XP_004515079.1	--	--	--
XLOC_020880	No	No	No	No	--	--	--	--
XLOC_020881	No	No	No	No	XP_003048376.1	--	--	--
XLOC_020892	No	No	No	No	--	--	--	--
XLOC_020893	No	No	No	No	--	--	--	--
XLOC_020899	No	No	No	No	--	--	--	--
XLOC_020900	No	No	No	No	NP_001119632.1	--	O	--
XLOC_020906	DEG(M)	No	No	No	NP_001119631.1	--	C	K08738
XLOC_020911	No	No	No	No	XP_004351625.1	--	Z	K18584
XLOC_020912	DEG(M)	No	No	No	--	--	J	--
XLOC_020913	No	No	No	No	--	--	--	--
XLOC_020914	No	No	No	No	--	--	--	--
XLOC_020916	No	No	No	No	XP_008898946.1	--	--	--
XLOC_020917	No	No	No	No	--	--	--	--
XLOC_020923	DEG(M)	No	No	No	NP_001280333.1	--	O	--
XLOC_020927	No	No	No	No	--	--	--	--
XLOC_020928	No	No	No	No	XP_001622889.1	NF-YC	B	--
XLOC_020931	No	No	No	No	XP_001950124.1	--	H	--
XLOC_020938	DEG(M)	No	DEG(I+M)	DEG(M)	--	--	--	--
XLOC_020939	No	No	No	No	--	--	--	--

**Differentially expressed gene (DEG) IDs**	***C. microphyllum* root and shoot-specific (>2 fold)**	***C. microphyllum* root and shoot-specific (>10 fold)**	**ICC4958 and *C. microphyllum* root-specific (>2 fold)**	***C. microphyllum* root-specific (>2 fold)**	**Functional Annotation**			
					**NCBI nr IDs**	**TFs**	**KOG**	**KEGG**
XLOC_020941	No	No	No	No	--	--	--	--
XLOC_020942	No	No	No	No	--	--	--	--
XLOC_020943	No	No	No	No	XP_008915896.1	--	--	--
XLOC_020944	DEG(M)	No	No	No	EUC66395.1	--	--	--
XLOC_020945	DEG(M)	No	No	No	--	--	--	--
XLOC_020946	No	No	No	No	CEL57452.1	--	--	--
XLOC_020948	No	No	No	No	--	--	--	--
XLOC_020956	No	No	No	No	--	--	--	--
XLOC_020957	No	No	No	No	--	--	--	--
XLOC_020959	No	No	No	No	XP_012199123.1	--	T	--
XLOC_020960	No	No	No	No	--	--	Z	--
XLOC_020961	DEG(M)	No	No	No	XP_002519450.1	--	T	K00889
XLOC_020962	No	No	No	No	--	--	--	--
XLOC_020967	DEG(M)	No	No	No	KDN43479.1	--	--	--
XLOC_020969	No	No	No	No	--	--	--	--
XLOC_020971	No	No	No	No	CCO34427.1	--	--	--
XLOC_020972	No	No	No	No	--	--	--	--
XLOC_020974	No	No	No	No	XP_001943593.2	--	Z	--
XLOC_020975	DEG(M)	No	No	No	--	--	--	--
XLOC_020976	No	No	No	No	--	--	--	--
XLOC_020977	No	No	No	No	CDW60759.1	--	O	--
XLOC_020978	No	No	No	No	--	--	C	--
XLOC_020980	No	No	No	No	--	--	--	--
XLOC_020986	No	No	No	No	KDN34600.1	--	--	--
XLOC_020988	No	No	No	No	--	--	--	--
XLOC_020990	DEG(M)	No	No	No	--	--	J	K02901
XLOC_020992	No	No	No	No	CCO31909.1	--	--	--
XLOC_020993	No	No	No	No	--	--	--	--
XLOC_020996	No	No	No	No	--	--	--	--
XLOC_020999	No	No	No	No	--	--	--	--
XLOC_021000	No	No	No	No	--	--	J	--
XLOC_021001	No	No	No	No	XP_002523672.1	--	X	K01209
XLOC_021002	No	No	No	No	KEP54512.1	--	T	--
XLOC_021003	No	No	No	No	CCO35327.1	--	--	K15923
XLOC_021004	No	No	No	No	--	--	--	K01052
XLOC_021009	No	No	No	No	--	--	J	K02873
XLOC_021011	No	No	No	No	CEL59540.1	--	G	--
XLOC_021015	No	No	No	No	--	--	R	--
XLOC_021016	No	No	No	No	--	--	--	--
XLOC_021017	No	No	DEG(I+M)	No	--	--	--	--

Differentially expressed gene (DEG) IDs	*C. microphyllum* root and shoot-specific (>2 fold)	*C. microphyllum* root and shoot-specific (>10 fold)	ICC4958 and *C. microphyllum* root-specific (>2 fold)	*C. microphyllum* root-specific (>2 fold)	Functional Annotation			
					NCBI nr IDs	TFs	KOG	KEGG
XLOC_021019	No	No	No	No	--	--	--	--
XLOC_021020	No	No	No	No	XP_002901172.1	--	J	K03236
XLOC_021023	DEG(M)	DEG(M)	No	No	XP_005686688.1	--	--	--
XLOC_021024	No	No	No	No	--	--	--	--
XLOC_021025	No	No	No	No	--	--	S	--
XLOC_021027	No	No	No	No	--	--	J	--
XLOC_021030	No	No	No	No	XP_003041166.1	--	--	--
XLOC_021031	No	No	DEG(I+M)	No	--	--	Q	--
XLOC_021038	DEG(M)	No	DEG(I+M)	DEG(M)	CEL58822.1	--	--	--
XLOC_021039	DEG(M)	DEG(M)	DEG(I+M)	DEG(M)	--	--	--	--
XLOC_021041	DEG(M)	DEG(M)	DEG(I+M)	DEG(M)	--	--	--	--
XLOC_021051	DEG(M)	No	No	No	XP_003048469.1	--	--	--
XLOC_021052	No	No	No	No	XP_003048469.1	--	--	--
XLOC_021054	DEG(M)	DEG(M)	No	No	XP_007584396.1	NF-YC	B	K11251
XLOC_021056	No	No	No	No	--	ERF	--	K09286
XLOC_021057	No	No	No	No	--	ERF	--	K09286
XLOC_021061	No	No	No	No	KEP55034.1	--	--	--
XLOC_021062	No	No	No	No	EUC65499.1	--	--	--
XLOC_021063	DEG(M)	No	No	No	XP_009525674.1	--	--	--
XLOC_021064	DEG(M)	DEG(M)	DEG(I+M)	DEG(M)	XP_009525674.1	--	--	--
XLOC_021065	DEG(M)	DEG(M)	DEG(I+M)	DEG(M)	--	--	--	--
XLOC_021066	No	No	DEG(I+M)	No	XP_004488183.1	WRKY	--	--
XLOC_021068	No	No	No	No	--	--	--	--
XLOC_021072	DEG(M)	No	No	No	--	--	--	--
XLOC_021074	No	No	DEG(I+M)	No	--	MYB	--	K09422
XLOC_021085	No	No	No	No	ETK81791.1	--	--	--
XLOC_021108	No	No	No	No	XP_009531371.1	--	--	--
XLOC_021112	No	No	No	No	--	--	--	--
XLOC_021113	DEG(M)	No	No	No	--	--	--	--
XLOC_021125	No	No	No	No	--	--	--	--
XLOC_021126	DEG(M)	DEG(M)	DEG(I+M)	DEG(M)	CEL51915.1	--	--	--
XLOC_021128	DEG(M)	DEG(M)	DEG(I+M)	DEG(M)	CCO34847.1	--	--	--
XLOC_021137	DEG(M)	No	No	No	--	--	--	--
XLOC_021140	DEG(M)	No	No	No	--	--	--	--
XLOC_021142	DEG(M)	No	DEG(I+M)	DEG(M)	CCO37025.1	--	--	--
XLOC_021146	DEG(M)	No	No	No	ELU42372.1	--	--	K01638
XLOC_021159	No	No	No	No	--	--	--	K05768
XLOC_021162	DEG(M)	No	DEG(I+M)	DEG(M)	XP_004513263.1	--	P	--
XLOC_021164	No	No	No	No	--	--	--	--
XLOC_021165	DEG(M)	No	No	No	--	--	--	--

**Differentially expressed gene (DEG) IDs**	***C. microphyllum* root and shoot-specific (>2 fold)**	***C. microphyllum* root and shoot-specific (>10 fold)**	**ICC4958 and *C. microphyllum* root-specific (>2 fold)**	***C. microphyllum* root-specific (>2 fold)**	**Functional Annotation**			
					**NCBI nr IDs**	**TFs**	**KOG**	**KEGG**
XLOC_021166	No	No	No	No	--	--	--	--
XLOC_021169	No	No	No	No	KEP51450.1	--	--	--
XLOC_021172	DEG(M)	No	No	No	--	--	--	--
XLOC_021174	No	No	No	No	--	--	--	--
XLOC_021175	No	No	No	No	--	--	--	K02692
XLOC_021180	No	No	No	No	--	--	R	--
XLOC_021182	DEG(M)	No	No	No	--	--	--	--
XLOC_021186	No	No	No	No	--	--	--	--
XLOC_021190	No	No	No	No	--	--	--	--
XLOC_021191	No	No	DEG(I+M)	No	--	--	--	--
XLOC_021193	No	No	No	No	--	--	--	--
XLOC_021198	No	No	No	No	--	--	--	--
XLOC_021200	No	No	No	No	XP_003051197.1	--	Q	--
XLOC_021202	No	No	No	No	--	--	--	--
XLOC_021204	No	No	No	No	CCO26472.1	--	--	--
XLOC_021205	DEG(M)	No	No	No	--	--	--	--
XLOC_021206	No	No	No	No	--	--	--	--
XLOC_021208	No	No	No	No	XP_002902797.1	--	J	K04567
XLOC_021212	No	No	No	No	--	--	J	K02941
XLOC_021216	No	No	No	No	XP_012079194.1	--	T	K00924
XLOC_021217	DEG(M)	No	No	No	CEL60816.1	--	J	--
XLOC_021221	No	No	No	No	--	--	--	--
XLOC_021223	DEG(M)	No	No	No	--	--	Z	--
XLOC_021224	No	No	No	No	--	--	--	--
XLOC_021226	DEG(M)	No	No	No	--	--	R	K00469
XLOC_021232	No	No	No	No	--	--	--	--
XLOC_021236	No	No	No	No	KDN34510.1	--	--	--
XLOC_021238	No	No	No	No	--	--	--	--
XLOC_021239	DEG(M)	DEG(M)	DEG(I+M)	DEG(M)	XP_008908767.1	--	--	--
XLOC_021241	DEG(M)	No	No	No	AAU84946.1	--	C	K02132
XLOC_021242	DEG(M)	No	No	No	--	--	J	--
XLOC_021246	No	No	No	No	--	--	--	--
XLOC_021247	No	No	No	No	KEP50314.1	--	--	--
XLOC_021248	No	No	No	No	--	--	--	--
XLOC_021249	No	No	DEG(I+M)	No	XP_004504581.1	--	--	--
XLOC_021263	DEG(M)	No	No	No	--	--	--	--
XLOC_021264	DEG(M)	No	No	No	NP_001162108.1	--	--	--
XLOC_021280	No	No	DEG(I+M)	No	--	--	O	--
XLOC_021281	DEG(M)	No	DEG(I+M)	DEG(M)	--	--	O	--
XLOC_021290	DEG(M)	No	No	No	--	--	--	--

Differentially expressed gene (DEG) IDs	*C. microphyllum* root and shoot-specific (>2 fold)	*C. microphyllum* root and shoot-specific (>10 fold)	ICC4958 and *C. microphyllum* root-specific (>2 fold)	*C. microphyllum* root-specific (>2 fold)	Functional Annotation			
					NCBI nr IDs	TFs	KOG	KEGG
XLOC_021291	DEG(M)	No	No	No	--	--	--	--
XLOC_021292	DEG(M)	No	No	No	--	--	--	--
XLOC_021293	No	No	No	No	EUC66845.1	NAC	J	--
XLOC_021294	No	No	No	No	ELU41656.1	--	J	--
XLOC_021333	No	No	DEG(I+M)	No	XP_004487192.2	C2H2	R	--
XLOC_021340	DEG(M)	No	No	No	ELU36626.1	--	--	--
XLOC_021376	No	No	DEG(I+M)	No	--	--	--	--
XLOC_021416	No	No	No	No	KDN40055.1	--	J	--
XLOC_021417	No	No	No	No	KEP54532.1	--	J	K02936
XLOC_021418	DEG(M)	No	No	No	KDN40055.1	--	J	--
XLOC_021437	DEG(M)	No	No	No	--	--	--	--
XLOC_021439	No	No	DEG(I+M)	No	--	--	C	--
XLOC_021446	No	No	No	No	XP_009517402.1	--	J	K03257
XLOC_021449	No	No	No	No	ELU44176.1	--	J	--
XLOC_021451	No	No	No	No	CEL52283.1	--	--	--
XLOC_021458	No	No	No	No	--	--	--	--
XLOC_021459	DEG(M)	No	No	No	--	--	--	--
XLOC_021461	DEG(M)	No	No	No	--	--	--	--
XLOC_021462	No	No	No	No	--	--	--	--
XLOC_021468	DEG(M)	No	No	No	--	--	--	--
XLOC_021469	No	No	No	No	XP_008911519.1	--	--	--
XLOC_021473	No	No	No	No	XP_004504150.1	--	--	K02723
XLOC_021477	No	No	No	No	ENH72675.1	--	--	--
XLOC_021480	No	No	DEG(I+M)	No	XP_004498445.1	--	--	--
XLOC_021483	No	No	No	No	--	--	--	--
XLOC_021484	No	No	No	No	--	--	--	--
XLOC_021487	No	No	No	No	--	--	--	--
XLOC_021488	DEG(M)	DEG(M)	No	No	--	--	--	--
XLOC_021493	DEG(M)	DEG(M)	DEG(I+M)	DEG(M)	XP_008913608.1	--	J	--
XLOC_021495	No	No	No	No	--	--	--	--
XLOC_021496	DEG(M)	No	No	No	EYB88032.1	--	Z	--
XLOC_021498	No	No	No	No	XP_002519849.1	--	S	K17498
XLOC_021499	No	No	No	No	--	--	--	--
XLOC_021505	DEG(M)	No	No	No	EGU75390.1	--	J	--
XLOC_021507	DEG(M)	DEG(M)	No	No	EGU89246.1	--	--	--
XLOC_021508	No	No	No	No	XP_003054108.1	--	--	--
XLOC_021509	DEG(M)	DEG(M)	DEG(I+M)	DEG(M)	XP_004506972.1	--	--	--
XLOC_021514	No	No	No	No	--	--	--	--
XLOC_021515	DEG(M)	No	No	No	--	--	--	--
XLOC_021519	No	No	No	No	XP_001659921.1	--	K	--

**Differentially expressed gene (DEG) IDs**	***C. microphyllum* root and shoot-specific (>2 fold)**	***C. microphyllum* root and shoot-specific (>10 fold)**	**ICC4958 and *C. microphyllum* root-specific (>2 fold)**	***C. microphyllum* root-specific (>2 fold)**	**Functional Annotation**			
					**NCBI nr IDs**	**TFs**	**KOG**	**KEGG**
XLOC_021520	No	No	No	No	--	--	--	--
XLOC_021521	DEG(M)	No	No	No	--	--	--	--
XLOC_021526	No	No	No	No	EUC53763.1	--	--	--
XLOC_021527	No	No	DEG(I+M)	No	XP_004504593.1	--	U	K20279
XLOC_021531	No	No	No	No	ETO69062.1	--	--	K05605
XLOC_021532	No	No	No	No	--	--	--	--
XLOC_021541	No	No	No	No	ETP37243.1	--	--	--
XLOC_021544	No	No	No	No	--	--	--	--
XLOC_021546	DEG(M)	DEG(M)	DEG(I+M)	DEG(M)	CAX83136.1	Dof	Z	--
XLOC_021547	No	No	No	No	--	--	C	K02154
XLOC_021551	DEG(M)	No	No	No	KHN87154.1	--	J	--
XLOC_021552	No	No	No	No	--	--	C	K08914
XLOC_021557	DEG(M)	No	No	No	--	--	--	--
XLOC_021559	DEG(M)	No	DEG(I+M)	DEG(M)	--	--	--	--
XLOC_021577	DEG(M)	No	No	No	--	--	--	--
XLOC_021582	No	No	No	No	--	--	--	--
XLOC_021584	No	No	No	No	XP_008909815.1	--	EI	--
XLOC_021589	DEG(M)	No	No	No	EUC64831.1	--	C	--
XLOC_021590	DEG(M)	No	No	No	EUC64831.1	--	C	K08738
XLOC_021591	No	No	No	No	--	--	--	--
XLOC_021592	No	No	No	No	CEL62731.1	--	--	--
XLOC_021598	DEG(M)	DEG(M)	No	No	--	--	--	--
XLOC_021599	DEG(M)	No	No	No	--	--	--	--
XLOC_021600	DEG(M)	No	DEG(I+M)	DEG(M)	--	--	--	--
XLOC_021601	DEG(M)	No	No	No	--	--	--	--
XLOC_021602	No	No	No	No	EUC67294.1	--	--	--
XLOC_021608	DEG(M)	No	No	No	--	--	--	--
XLOC_021610	DEG(M)	DEG(M)	DEG(I+M)	DEG(M)	KDN46690.1	--	--	--
XLOC_021612	No	No	No	No	EUC63224.1	--	--	--
XLOC_021613	DEG(M)	DEG(M)	DEG(I+M)	DEG(M)	XP_004332908.1	Dof	Z	--
XLOC_021614	DEG(M)	DEG(M)	DEG(I+M)	DEG(M)	XP_004332908.1	Dof	Z	--
XLOC_021617	No	No	No	No	--	--	--	--
XLOC_021618	No	No	No	No	--	--	--	--
XLOC_021621	No	No	No	No	XP_008907567.1	--	EJ	--
XLOC_021622	DEG(M)	No	No	No	--	--	--	--
XLOC_021625	No	No	No	No	--	--	--	--
XLOC_021626	No	No	No	No	KEP55201.1	--	--	--
XLOC_021628	No	No	No	No	--	--	--	--
XLOC_021637	No	No	DEG(I+M)	No	XP_004492974.1	--	--	--
XLOC_021639	DEG(M)	No	No	No	CEL63661.1	--	J	K02918

Differentially expressed gene (DEG) IDs	*C. microphyllum* root and shoot-specific (>2 fold)	*C. microphyllum* root and shoot-specific (>10 fold)	ICC4958 and *C. microphyllum* root-specific (>2 fold)	*C. microphyllum* root-specific (>2 fold)	Functional Annotation			
					NCBI nr IDs	TFs	KOG	KEGG
XLOC_021640	No	No	No	No	--	--	--	--
XLOC_021641	No	No	No	No	--	--	--	--
XLOC_021643	DEG(M)	DEG(M)	DEG(I+M)	DEG(M)	XP_008914876.1	MIKC	J	K02900
XLOC_021652	No	No	No	No	CCO27020.1	--	--	--
XLOC_021653	No	No	No	No	XP_001943200.2	--	--	--
XLOC_021654	DEG(M)	DEG(M)	DEG(I+M)	DEG(M)	--	--	--	--
XLOC_021655	DEG(M)	DEG(M)	DEG(I+M)	DEG(M)	--	--	--	--
XLOC_021656	DEG(M)	DEG(M)	DEG(I+M)	DEG(M)	--	--	--	--
XLOC_021657	DEG(M)	DEG(M)	DEG(I+M)	DEG(M)	--	--	--	--
XLOC_021661	No	No	No	No	XP_002513943.1	--	X	--
XLOC_021666	DEG(M)	No	No	No	KEP49572.1	--	--	--
XLOC_021675	No	No	No	No	--	--	--	--
XLOC_021676	DEG(M)	No	No	No	CCO30904.1	--	--	--
XLOC_021677	DEG(M)	No	No	No	--	--	--	--
XLOC_021685	No	No	No	No	NP_001156347.1	--	--	--
XLOC_021689	DEG(M)	No	No	No	KEP48426.1	--	--	--
XLOC_021696	No	No	No	No	--	--	--	--
XLOC_021709	No	No	No	No	XP_004493454.1	--	C	--
XLOC_021711	No	No	No	No	BAG64751.1	--	T	--
XLOC_021712	No	No	No	No	--	--	--	--
XLOC_021713	No	No	No	No	--	--	--	--
XLOC_021714	DEG(M)	DEG(M)	DEG(I+M)	DEG(M)	XP_009521468.1	--	J	K02918
XLOC_021717	DEG(M)	DEG(M)	No	No	--	--	--	--
XLOC_021719	DEG(M)	No	No	No	CCO36433.1	--	J	--
XLOC_021720	DEG(M)	No	No	No	KDN39728.1	--	J	--
XLOC_021722	DEG(M)	No	No	No	EUC55870.1	--	--	--
XLOC_021725	No	No	No	No	EUC67365.1	--	--	--
XLOC_021727	DEG(M)	No	No	No	AAM51209.1	YABBY	OR	--
XLOC_021728	No	No	No	No	CAA11269.1	YABBY	OR	--
XLOC_021729	No	No	DEG(I+M)	No	--	--	G	K01193
XLOC_021737	DEG(M)	DEG(M)	DEG(I+M)	DEG(M)	--	--	--	K02924
XLOC_021739	No	No	No	No	--	--	--	--
XLOC_021740	DEG(M)	No	No	No	KDN34964.1	--	--	--
XLOC_021743	No	No	No	No	--	--	--	K00854
XLOC_021746	DEG(M)	DEG(M)	DEG(I+M)	DEG(M)	--	--	--	K12356
XLOC_021750	No	No	DEG(I+M)	No	--	bZIP	V	--
XLOC_021751	DEG(M)	DEG(M)	DEG(I+M)	DEG(M)	--	--	--	--
XLOC_021752	No	No	No	No	AAR85527.1	--	O	--
XLOC_021753	DEG(M)	No	No	No	CEL58828.1	--	J	--
XLOC_021754	DEG(M)	No	No	No	XP_002899435.1	--	R	--

Differentially expressed gene (DEG) IDs	*C. microphyllum* root and shoot-specific (>2 fold)	*C. microphyllum* root and shoot-specific (>10 fold)	ICC4958 and *C. microphyllum* root-specific (>2 fold)	*C. microphyllum* root-specific (>2 fold)	Functional Annotation			
					NCBI nr IDs	TFs	KOG	KEGG
XLOC_021756	DEG(M)	No	No	No	--	--	--	--
XLOC_021764	DEG(M)	No	No	No	--	--	--	--
XLOC_021766	No	No	No	No	--	--	--	--
XLOC_021769	No	No	No	No	--	--	--	--
XLOC_021773	No	No	No	No	--	--	--	--
XLOC_021782	No	No	No	No	--	--	W	--
XLOC_021783	No	No	No	No	--	--	--	--
XLOC_021784	DEG(M)	No	No	No	EJW72679.1	--	Z	--
XLOC_021790	No	No	No	No	XP_004499292.1	--	--	--
XLOC_021791	No	No	No	No	XP_008872224.1	--	C	K03941
XLOC_021796	No	No	No	No	--	--	--	--
XLOC_021806	No	No	No	No	--	--	--	--
XLOC_021810	No	No	No	No	EUC65027.1	--	--	--
XLOC_021811	No	No	No	No	--	--	--	--
XLOC_021813	No	No	No	No	EJD75534.1	--	RP	--
XLOC_021814	No	No	No	No	--	--	--	K05758
XLOC_021817	No	No	No	No	--	--	--	--
XLOC_021818	DEG(M)	No	No	No	EUC63304.1	--	--	--
XLOC_021819	No	No	No	No	CEL53227.1	--	UO	--
XLOC_021830	No	No	No	No	XP_004505518.1	TCP	--	--
XLOC_021831	No	No	No	No	--	TCP	--	--
XLOC_021832	No	No	No	No	KEP52665.1	--	E	--
XLOC_021840	DEG(M)	DEG(M)	DEG(I+M)	DEG(M)	CCO32683.1	--	--	--
XLOC_021842	No	No	No	No	--	--	--	--
XLOC_021843	DEG(M)	DEG(M)	DEG(I+M)	DEG(M)	--	--	--	--
XLOC_021846	No	No	No	No	--	--	H	K01599
XLOC_021847	DEG(M)	DEG(M)	No	No	--	--	--	--
XLOC_021848	DEG(M)	DEG(M)	No	No	EUC58715.1	--	--	--
XLOC_021852	No	No	No	No	EUC61659.1	--	H	--
XLOC_021853	No	No	No	No	KEP55664.1	--	--	--
XLOC_021854	No	No	No	No	EUC62495.1	--	--	--
XLOC_021860	DEG(M)	No	No	No	--	--	--	--
XLOC_021862	No	No	No	No	XP_001942751.1	--	--	--
XLOC_021863	No	No	No	No	--	--	--	--
XLOC_021864	No	No	No	No	CCO28665.1	--	R	K19788
XLOC_021870	No	No	No	No	EUC63368.1	--	O	K05864
XLOC_021871	No	No	No	No	--	--	J	K02895
XLOC_021874	No	No	No	No	CEL57824.1	--	--	--
XLOC_021875	No	No	No	No	--	--	--	--
XLOC_021880	DEG(M)	No	No	No	--	--	--	--

Differentially expressed gene (DEG) IDs	***C. microphyllum* root and shoot-specific (>2 fold)**	***C. microphyllum* root and shoot-specific (>10 fold)**	**ICC4958 and *C. microphyllum* root-specific (>2 fold)**	***C. microphyllum* root-specific (>2 fold)**	**Functional Annotation**			
					**NCBI nr IDs**	**TFs**	**KOG**	**KEGG**
XLOC_021881	No	No	No	No	XP_001648645.1	--	G	K01759
XLOC_021882	No	No	No	No	--	--	--	--
XLOC_021885	DEG(M)	No	No	No	ETM35496.1	--	--	--
XLOC_021889	No	No	No	No	KDP30176.1	TALE	K	--
XLOC_021891	No	No	No	No	--	--	--	--
XLOC_021892	No	No	No	No	KNB11030.1	--	--	--
XLOC_021893	DEG(M)	No	No	No	XP_004495836.1	--	M	--
XLOC_021896	No	No	No	No	--	--	--	--
XLOC_021899	DEG(M)	No	No	No	XP_527253.2	--	B	--
XLOC_021900	No	No	DEG(I+M)	No	--	--	--	--
XLOC_021902	No	No	No	No	--	--	J	--
XLOC_021903	No	No	No	No	KEP52552.1	--	--	--
XLOC_021907	No	No	No	No	--	--	--	--
XLOC_021912	No	No	No	No	--	--	--	--
XLOC_021913	No	No	No	No	XP_004514799.1	--	--	--
XLOC_021914	No	No	No	No	--	--	--	--
XLOC_021918	No	No	No	No	--	--	--	--
XLOC_021920	No	No	No	No	--	--	--	K01054
XLOC_021921	DEG(M)	DEG(M)	No	No	--	--	--	--
XLOC_021922	DEG(M)	No	No	No	--	--	--	--
XLOC_021923	No	No	No	No	--	--	--	K10579
XLOC_021926	No	No	No	No	--	--	--	--
XLOC_021929	No	No	No	No	AII18052.1	--	X	K02705
XLOC_021931	DEG(M)	No	DEG(I+M)	DEG(M)	CCO37654.1	--	--	--
XLOC_021935	DEG(M)	DEG(M)	No	No	--	--	--	--
XLOC_021937	No	No	No	No	XP_004337289.1	NAC	J	--
XLOC_021938	No	No	No	No	EUC67512.1	--	--	--
XLOC_021944	DEG(M)	No	No	No	CCO36035.1	--	--	--
XLOC_021945	No	No	No	No	XP_008178869.1	--	O	--
XLOC_021948	No	No	No	No	--	--	--	K00365
XLOC_021949	No	No	No	No	--	--	--	--
XLOC_021950	No	No	No	No	--	--	--	--
XLOC_021951	No	No	No	No	--	--	--	--
XLOC_021953	No	No	No	No	XP_002510332.1	--	TZ	--
XLOC_021957	No	No	No	No	--	--	--	--
XLOC_021959	DEG(M)	DEG(M)	DEG(I+M)	DEG(M)	XP_009520427.1	--	O	--
XLOC_021969	No	No	No	No	NP_001155476.1	--	O	--
XLOC_021970	No	No	No	No	--	--	H	--
XLOC_021971	No	No	No	No	--	--	TU	--
XLOC_021972	No	No	No	No	--	--	--	--

Differentially expressed gene (DEG) IDs	*C. microphyllum* root and shoot-specific (>2 fold)	*C. microphyllum* root and shoot-specific (>10 fold)	ICC4958 and *C. microphyllum* root-specific (>2 fold)	*C. microphyllum* root-specific (>2 fold)	Functional Annotation			
					NCBI nr IDs	TFs	KOG	KEGG
XLOC_021974	No	No	No	No	--	--	--	--
XLOC_021975	No	No	No	No	--	--	--	--
XLOC_021976	No	No	No	No	CEL54874.1	--	--	--
XLOC_021978	No	No	No	No	--	--	--	--
XLOC_021981	No	No	No	No	XP_004487182.1	bZIP	KR	--
XLOC_021986	No	No	DEG(I+M)	No	--	--	--	--
XLOC_021991	No	No	No	No	KEP48093.1	--	--	--
XLOC_021992	No	No	No	No	KEP55118.1	--	--	--
XLOC_021994	No	No	No	No	--	--	C	K00006
XLOC_021995	No	No	No	No	--	--	--	--
XLOC_021996	No	No	No	No	KEP53938.1	--	--	--
XLOC_021998	DEG(M)	No	No	No	--	--	--	--
XLOC_022000	No	No	No	No	--	--	--	--
XLOC_022008	No	No	No	No	--	--	--	--
XLOC_022010	No	No	No	No	EMT69116.1	--	--	--
XLOC_022011	No	No	No	No	--	--	--	--
XLOC_022013	No	No	No	No	--	--	--	--
XLOC_022018	No	No	No	No	--	--	--	--
XLOC_022019	No	No	No	No	--	--	--	--
XLOC_022020	DEG(M)	No	No	No	KKA76157.1	--	K	--
XLOC_022021	DEG(M)	DEG(M)	DEG(I+M)	DEG(M)	--	--	--	K00485
XLOC_022024	No	No	No	No	CCO32912.1	--	--	--
XLOC_022027	No	No	No	No	AEK51003.1	Trihelix	G	--
XLOC_022028	No	No	No	No	--	--	--	--
XLOC_022029	No	No	No	No	--	--	--	--
XLOC_022037	DEG(M)	No	No	No	CEL53244.1	--	--	--
XLOC_022041	DEG(M)	DEG(M)	No	No	XP_003636263.1	--	--	--
XLOC_022044	No	No	No	No	--	--	--	--
XLOC_022045	No	No	No	No	--	--	--	--
XLOC_022051	No	No	No	No	BAS04780.1	--	--	--
XLOC_022056	DEG(M)	No	DEG(I+M)	DEG(M)	CCO32094.1	--	Z	--
XLOC_022057	DEG(M)	DEG(M)	No	No	KIJ06701.1	--	Z	--
XLOC_022058	DEG(M)	No	No	No	AFU72282.1	--	Z	--
XLOC_022059	DEG(M)	No	No	No	XP_002683348.1	--	Z	K10355
XLOC_022060	DEG(M)	No	No	No	KIH60495.1	--	Z	--
XLOC_022062	DEG(M)	No	No	No	AEN94510.1	--	Z	--
XLOC_022067	DEG(M)	DEG(M)	DEG(I+M)	DEG(M)	XP_008896240.1	--	J	K02918
XLOC_022073	No	No	No	No	--	--	--	--
XLOC_022075	DEG(M)	No	No	No	--	--	--	--
XLOC_022076	DEG(M)	No	No	No	XP_001829580.1	--	J	--

Differentially expressed gene (DEG) IDs	*C. microphyllum* root and shoot-specific (>2 fold)	*C. microphyllum* root and shoot-specific (>10 fold)	ICC4958 and *C. microphyllum* root-specific (>2 fold)	*C. microphyllum* root-specific (>2 fold)	Functional Annotation			
					NCBI nr IDs	TFs	KOG	KEGG
XLOC_022078	DEG(M)	DEG(M)	DEG(I+M)	DEG(M)	--	--	--	--
XLOC_022079	No	No	No	No	XP_002529891.1	--	QG	--
XLOC_022085	DEG(M)	No	No	No	KDQ24377.1	--	Z	K08341
XLOC_022086	No	No	No	No	--	--	--	--
XLOC_022087	No	No	No	No	KFP92623.1	--	U	K07877
XLOC_022091	No	No	No	No	EWY88047.1	--	--	--
XLOC_022095	No	No	No	No	--	--	--	--
XLOC_022097	No	No	No	No	XP_008896134.1	--	--	K03963
XLOC_022099	DEG(M)	No	No	No	--	--	--	--
XLOC_022101	No	No	No	No	--	--	--	--
XLOC_022111	No	No	No	No	XP_004498558.1	Trihelix	X	--
XLOC_022113	DEG(M)	No	No	No	--	--	--	--
XLOC_022114	No	No	No	No	--	--	--	--
XLOC_022115	No	No	No	No	YP_002149736.1	--	J	K02967
XLOC_022117	No	No	No	No	--	--	--	--
XLOC_022119	No	No	No	No	--	--	--	K02934
XLOC_022124	No	No	No	No	XP_001939279.1	--	--	--
XLOC_022127	DEG(M)	No	No	No	--	--	--	--
XLOC_022128	DEG(M)	No	No	No	--	--	--	--
XLOC_022129	No	No	No	No	XP_003052983.1	--	--	--
XLOC_022130	No	No	No	No	KEP46871.1	--	--	--
XLOC_022132	No	No	No	No	--	--	--	--
XLOC_022136	No	No	No	No	ETM40649.1	--	--	--
XLOC_022138	No	No	DEG(I+M)	No	--	--	--	--
XLOC_022139	No	No	No	No	--	--	--	--
XLOC_022141	No	No	No	No	ETL39272.1	--	C	--
XLOC_022142	No	No	No	No	--	--	X	--
XLOC_022146	No	No	No	No	NP_001156069.1	--	--	--
XLOC_022149	No	No	DEG(I+M)	No	--	C2H2	--	--
XLOC_022153	DEG(M)	No	No	No	NP_001119681.1	--	J	--
XLOC_022154	DEG(M)	No	No	No	--	--	--	K01183
XLOC_022156	No	No	No	No	--	--	--	--
XLOC_022158	No	No	No	No	--	--	--	--
XLOC_022160	No	No	No	No	EUC62195.1	--	--	--
XLOC_022162	No	No	No	No	--	--	--	--
XLOC_022164	No	No	No	No	NP_001155665.2	--	--	--
XLOC_022167	DEG(M)	DEG(M)	No	No	--	--	--	--
XLOC_022170	No	No	No	No	--	--	--	--
XLOC_022171	No	No	No	No	EUC66304.1	--	--	--
XLOC_022172	No	No	DEG(I+M)	No	XP_012569190.1	--	--	--

**Differentially expressed gene (DEG) IDs**	***C. microphyllum* root and shoot-specific (>2 fold)**	***C. microphyllum* root and shoot-specific (>10 fold)**	**ICC4958 and *C. microphyllum* root-specific (>2 fold)**	***C. microphyllum* root-specific (>2 fold)**	**Functional Annotation**			
					**NCBI nr IDs**	**TFs**	**KOG**	**KEGG**
XLOC_022174	No	No	No	No	--	--	R	--
XLOC_022175	No	No	No	No	NP_001155710.1	--	R	K12876
XLOC_022177	No	No	No	No	ETI41760.1	--	--	--
XLOC_022178	DEG(M)	No	No	No	--	--	DZ	--
XLOC_022181	No	No	No	No	--	--	--	--
XLOC_022183	No	No	No	No	--	--	--	K01265
XLOC_022187	DEG(M)	No	No	No	--	--	--	--
XLOC_022192	No	No	No	No	DAA34970.1	--	--	--
XLOC_022194	No	No	No	No	CCO33584.1	--	--	--
XLOC_022202	No	No	No	No	XP_004489825.1	YABBY	--	--
XLOC_022205	No	No	No	No	--	--	--	K01802
XLOC_022206	No	No	No	No	--	--	--	--
XLOC_022207	DEG(M)	No	No	No	--	--	--	--
XLOC_022211	No	No	No	No	--	--	--	--
XLOC_022212	DEG(M)	No	No	No	CAC28353.1	--	X	--
XLOC_022213	DEG(M)	No	No	No	CAC28353.1	--	X	--
XLOC_022215	No	No	No	No	--	--	--	--
XLOC_022217	No	No	No	No	--	--	--	--
XLOC_022219	No	No	No	No	--	--	--	--
XLOC_022220	DEG(M)	No	No	No	KDN44582.1	--	O	--
XLOC_022222	No	No	No	No	--	--	--	--
XLOC_022223	No	No	No	No	EUC53541.1	--	--	--
XLOC_022225	DEG(M)	DEG(M)	No	No	--	--	--	--
XLOC_022229	No	No	No	No	EUC63455.1	--	--	--
XLOC_022232	No	No	No	No	--	--	--	--
XLOC_022233	No	No	No	No	CCO27844.1	--	--	--
XLOC_022239	No	No	No	No	CCO26390.1	--	EH	K01652
XLOC_022242	No	No	No	No	--	--	--	--
XLOC_022243	No	No	No	No	--	--	--	--
XLOC_022244	No	No	No	No	--	--	--	--
XLOC_022245	No	No	DEG(I+M)	No	XP_004498387.1	--	--	--
XLOC_022247	DEG(M)	No	No	No	ABJ81007.1	--	Z	--
XLOC_022249	DEG(M)	No	No	No	--	--	--	--
XLOC_022250	No	No	No	No	--	--	--	--
XLOC_022255	No	No	No	No	--	--	--	--
XLOC_022256	DEG(M)	No	No	No	--	--	--	--
XLOC_022258	DEG(M)	No	No	No	--	--	--	--
XLOC_022259	No	No	No	No	--	--	--	--
XLOC_022268	No	No	No	No	XP_003044960.1	--	--	--
XLOC_022269	No	No	No	No	EXL58125.1	--	--	--

Differentially expressed gene (DEG) IDs	*C. microphyllum* root and shoot-specific (>2 fold)	*C. microphyllum* root and shoot-specific (>10 fold)	ICC4958 and *C. microphyllum* root-specific (>2 fold)	*C. microphyllum* root-specific (>2 fold)	Functional Annotation			
					NCBI nr IDs	TFs	KOG	KEGG
XLOC_022274	No	No	No	No	--	--	--	--
XLOC_022275	No	No	No	No	--	--	--	--
XLOC_022276	DEG(M)	No	No	No	ABM92916.1	--	O	--
XLOC_022280	DEG(M)	No	No	No	--	--	--	--
XLOC_022282	No	No	No	No	XP_001663853.1	--	J	K03237
XLOC_022287	DEG(M)	DEG(M)	DEG(I+M)	DEG(M)	XP_008901658.1	--	J	K02898
XLOC_022290	No	No	No	No	--	--	--	--
XLOC_022292	No	No	No	No	--	--	--	--
XLOC_022293	No	No	No	No	--	--	T	K07198
XLOC_022297	No	No	No	No	--	--	--	--
XLOC_022304	No	No	No	No	ELU43232.1	--	--	--
XLOC_022305	DEG(M)	No	No	No	EUC56620.1	--	--	--
XLOC_022306	No	No	No	No	--	--	--	--
XLOC_022307	No	No	No	No	--	--	--	--
XLOC_022311	No	No	No	No	--	--	--	--
XLOC_022312	No	No	No	No	--	--	--	--
XLOC_022314	No	No	DEG(I+M)	No	--	--	R	--
XLOC_022316	No	No	No	No	--	--	--	--
XLOC_022322	DEG(M)	DEG(M)	DEG(I+M)	DEG(M)	KDN35388.1	--	JO	--
XLOC_022324	DEG(M)	No	No	No	KEP47514.1	--	--	--
XLOC_022326	DEG(M)	No	No	No	--	--	--	--
XLOC_022328	DEG(M)	DEG(M)	DEG(I+M)	DEG(M)	XP_003043019.1	--	--	--
XLOC_022330	No	No	No	No	--	--	--	--
XLOC_022331	No	No	No	No	CEL55189.1	--	--	--
XLOC_022333	No	No	No	No	--	--	--	K02998
XLOC_022334	DEG(M)	No	No	No	CEL57336.1	bHLH	B	--
XLOC_022336	No	No	No	No	CCO30024.1	--	--	K03564
XLOC_022337	No	No	No	No	--	--	--	--
XLOC_022344	DEG(M)	No	No	No	--	--	--	--
XLOC_022345	No	No	No	No	CCO34538.1	--	E	--
XLOC_022354	No	No	No	No	XP_003040903.1	--	--	--
XLOC_022355	No	No	No	No	XP_001948962.1	--	O	K09499
XLOC_022357	No	No	No	No	--	--	--	--
XLOC_022358	No	No	No	No	--	--	--	--
XLOC_022362	No	No	No	No	ETL78306.1	--	--	--
XLOC_022363	No	No	No	No	XP_008898610.1	--	--	--
XLOC_022368	DEG(M)	No	No	No	ETL38542.1	--	--	--
XLOC_022369	No	No	No	No	CCO26446.1	--	--	--
XLOC_022372	No	No	No	No	--	--	--	--
XLOC_022373	No	No	No	No	--	--	--	--

Differentially expressed gene (DEG) IDs	*C. microphyllum* root and shoot-specific (>2 fold)	*C. microphyllum* root and shoot-specific (>10 fold)	ICC4958 and *C. microphyllum* root-specific (>2 fold)	*C. microphyllum* root-specific (>2 fold)	Functional Annotation			
					NCBI nr IDs	TFs	KOG	KEGG
XLOC_022379	DEG(M)	No	No	No	--	--	--	--
XLOC_022380	DEG(M)	DEG(M)	DEG(I+M)	DEG(M)	EUC63853.1	--	--	--
XLOC_022382	No	No	No	No	--	--	--	K12876
XLOC_022383	No	No	No	No	--	--	--	--
XLOC_022387	No	No	No	No	--	--	--	--
XLOC_022388	No	No	No	No	ABM68586.1	--	A	K12812
XLOC_022391	No	No	No	No	--	--	--	--
XLOC_022392	No	No	No	No	--	--	--	--
XLOC_022398	No	No	No	No	XP_001897295.1	--	J	--
XLOC_022399	DEG(M)	No	No	No	--	--	--	--
XLOC_022403	No	No	No	No	AAU84934.1	--	--	--
XLOC_022404	No	No	No	No	AFL91513.1	--	--	--
XLOC_022407	No	No	No	No	XP_003244253.1	--	--	--
XLOC_022408	DEG(M)	No	DEG(I+M)	DEG(M)	XP_008896383.1	--	J	K02958
XLOC_022409	No	No	No	No	--	--	J	--
XLOC_022412	No	No	No	No	--	--	--	K00432
XLOC_022418	No	No	No	No	--	--	--	--
XLOC_022421	DEG(M)	No	No	No	--	--	--	--
XLOC_022422	No	No	No	No	--	--	--	--
XLOC_022423	No	No	No	No	KLO93013.1	--	--	--
XLOC_022427	DEG(M)	No	No	No	ETI54550.1	--	--	--
XLOC_022428	No	No	No	No	--	--	--	K01322
XLOC_022430	No	No	No	No	ETK72303.1	--	--	--
XLOC_022432	No	No	No	No	NP_001155422.1	--	--	--
XLOC_022434	DEG(M)	No	No	No	KDN45127.1	--	--	--
XLOC_022435	No	No	No	No	--	--	--	--
XLOC_022438	No	No	No	No	XP_003053118.1	--	--	--
XLOC_022439	DEG(M)	No	No	No	XP_003054495.1	--	O	--
XLOC_022440	No	No	No	No	ETL42197.1	--	--	--
XLOC_022441	No	No	No	No	--	--	DO	K03363
XLOC_022443	DEG(M)	DEG(M)	DEG(I+M)	DEG(M)	ACL11882.1	--	--	--
XLOC_022445	No	No	No	No	--	Trihelix	--	--
XLOC_022448	No	No	No	No	--	--	--	--
XLOC_022449	DEG(M)	No	No	No	--	--	O	--
XLOC_022450	DEG(M)	No	No	No	--	--	--	--
XLOC_022452	No	No	No	No	EUC67179.1	--	--	K20221
XLOC_022457	No	No	No	No	KDN49488.1	--	G	--
XLOC_022461	No	No	No	No	XP_001870860.1	--	B	K11253
XLOC_022464	No	No	No	No	--	--	--	--
XLOC_022465	No	No	No	No	--	--	--	--

Differentially expressed gene (DEG) IDs	*C. microphyllum* root and shoot-specific (>2 fold)	*C. microphyllum* root and shoot-specific (>10 fold)	ICC4958 and *C. microphyllum* root-specific (>2 fold)	*C. microphyllum* root-specific (>2 fold)	Functional Annotation			
					NCBI nr IDs	TFs	KOG	KEGG
XLOC_022467	No	No	No	No	--	--	--	--
XLOC_022469	No	No	No	No	--	--	--	--
XLOC_022470	No	No	No	No	EUC67362.1	--	--	--
XLOC_022471	No	No	No	No	--	--	--	--
XLOC_022477	No	No	No	No	ETP47957.1	--	--	--
XLOC_022481	No	No	No	No	--	--	C	K13354
XLOC_022483	No	No	No	No	--	--	T	--
XLOC_022485	No	No	No	No	CCO32888.1	--	--	--
XLOC_022489	No	No	No	No	EUC63951.1	--	--	--
XLOC_022492	No	No	No	No	CCO35098.1	--	--	--
XLOC_022496	No	No	No	No	--	--	--	--
XLOC_022497	No	No	No	No	KFM61120.1	FAR1	O	K13525
XLOC_022498	No	No	No	No	--	--	--	--
XLOC_022499	No	No	No	No	--	--	--	--
XLOC_022501	DEG(M)	DEG(M)	DEG(I+M)	DEG(M)	KDN34466.1	--	--	--
XLOC_022502	No	No	No	No	--	--	--	--
XLOC_022503	No	No	No	No	XP_001949000.1	--	--	--
XLOC_022504	No	No	No	No	--	--	--	--
XLOC_022505	DEG(M)	No	No	No	--	--	--	--
XLOC_022508	No	No	No	No	ETO83594.1	--	--	--
XLOC_022511	DEG(M)	No	No	No	XP_008610333.1	--	--	--
XLOC_022513	No	No	No	No	XP_003046107.1	--	--	--
XLOC_022520	No	No	No	No	EUC61508.1	--	--	--
XLOC_022522	No	No	No	No	CEL62605.1	--	--	K00864
XLOC_022525	No	No	No	No	XP_009526174.1	--	--	--
XLOC_022526	No	No	No	No	--	--	--	--
XLOC_022527	No	No	No	No	--	--	--	K03671
XLOC_022528	DEG(M)	DEG(M)	No	No	AGE97643.1	--	--	--
XLOC_022529	No	No	No	No	XP_001949815.2	--	--	--
XLOC_022531	No	No	No	No	--	--	--	--
XLOC_022532	No	No	No	No	--	--	--	--
XLOC_022534	DEG(M)	No	No	No	--	--	--	--
XLOC_022536	DEG(M)	No	No	No	NP_001155817.1	--	J	K02960
XLOC_022537	DEG(M)	No	DEG(I+M)	DEG(M)	--	GRAS	--	--
XLOC_022538	No	No	No	No	KDR21360.1	--	G	K03083
XLOC_022540	No	No	No	No	EUC57674.1	--	--	--
XLOC_022542	DEG(M)	No	No	No	--	--	J	K02940
XLOC_022543	DEG(M)	No	No	No	--	--	--	--
XLOC_022545	No	No	No	No	--	--	--	--
XLOC_022548	No	No	No	No	KEP47352.1	--	--	--

Differentially expressed gene (DEG) IDs	*C. microphyllum* root and shoot-specific (>2 fold)	*C. microphyllum* root and shoot-specific (>10 fold)	ICC4958 and *C. microphyllum* root-specific (>2 fold)	*C. microphyllum* root-specific (>2 fold)	Functional Annotation			
					NCBI nr IDs	TFs	KOG	KEGG
XLOC_022549	DEG(M)	No	No	No	--	--	--	--
XLOC_022550	No	No	No	No	--	--	--	--
XLOC_022556	No	No	No	No	XP_009521651.1	--	O	--
XLOC_022563	No	No	No	No	CDQ61350.1	--	J	--
XLOC_022566	No	No	No	No	XP_003051265.1	--	--	--
XLOC_022568	No	No	No	No	XP_002896373.1	--	O	K03063
XLOC_022571	No	No	No	No	--	--	--	--
XLOC_022575	No	No	No	No	--	--	--	--
XLOC_022576	No	No	No	No	--	--	--	--
XLOC_022579	No	No	No	No	BAH72316.1	--	E	K12657
XLOC_022582	No	No	No	No	XP_004495202.1	--	A	--
XLOC_022584	No	No	No	No	CCO29508.1	--	Z	--
XLOC_022585	No	No	No	No	EUC61023.1	--	--	--
XLOC_022589	No	No	No	No	ETL31656.1	--	--	--
XLOC_022590	No	No	No	No	ETO71611.1	--	--	--
XLOC_022592	No	No	No	No	--	--	U	--
XLOC_022594	No	No	No	No	CEL61724.1	--	--	K08832
XLOC_022595	No	No	No	No	--	--	--	--
XLOC_022596	DEG(M)	No	No	No	--	--	--	--
XLOC_022597	DEG(M)	No	No	No	--	--	--	--
XLOC_022605	DEG(M)	No	No	No	--	--	--	--
XLOC_022606	No	No	No	No	XP_008609300.1	--	EI	K01969
XLOC_022607	No	No	No	No	--	--	--	--
XLOC_022609	No	No	No	No	--	--	--	--
XLOC_022611	No	No	No	No	--	--	--	--
XLOC_022613	No	No	No	No	XP_001653404.1	--	--	--
XLOC_022615	No	No	No	No	--	--	--	--
XLOC_022616	No	No	No	No	--	--	--	--
XLOC_022618	No	No	No	No	--	--	--	--
XLOC_022619	No	No	No	No	--	--	O	--
XLOC_022622	No	No	No	No	KDN36224.1	--	--	K12349
XLOC_022625	No	No	No	No	XP_008896894.1	--	--	K00069
XLOC_022626	No	No	No	No	CEL55438.1	--	G	K16055
XLOC_022627	No	No	No	No	XP_001653838.1	--	O	--
XLOC_022629	DEG(M)	No	DEG(I+M)	DEG(M)	--	--	--	--
XLOC_022635	No	No	No	No	--	--	--	--
XLOC_022640	DEG(M)	No	No	No	--	--	--	--
XLOC_022641	No	No	No	No	CCO28201.1	--	C	K03941
XLOC_022642	DEG(M)	No	No	No	--	--	--	--
XLOC_022644	DEG(M)	No	DEG(I+M)	DEG(M)	XP_012572688.1	--	--	K01426

Differentially expressed gene (DEG) IDs	*C. microphyllum* root and shoot-specific (>2 fold)	*C. microphyllum* root and shoot-specific (>10 fold)	ICC4958 and *C. microphyllum* root-specific (>2 fold)	*C. microphyllum* root-specific (>2 fold)	Functional Annotation			
					NCBI nr IDs	TFs	KOG	KEGG
XLOC_022646	No	No	No	No	XP_002402966.1	--	B	K10752
XLOC_022648	DEG(M)	No	No	No	--	--	--	K19355
XLOC_022658	No	No	No	No	--	--	--	--
XLOC_022662	No	No	No	No	--	--	--	--
XLOC_022663	No	No	No	No	--	--	--	--
XLOC_022670	DEG(M)	DEG(M)	No	No	XP_003051110.1	--	C	--
XLOC_022679	No	No	No	No	--	--	--	K13153
XLOC_022683	No	No	No	No	--	--	--	--
XLOC_022684	No	No	No	No	CCI40103.1	--	--	K04688
XLOC_022687	No	No	No	No	--	--	I	K12663
XLOC_022688	No	No	No	No	--	--	J	--
XLOC_022694	No	No	No	No	XP_004515985.1	--	--	--
XLOC_022695	DEG(M)	No	DEG(I+M)	DEG(M)	--	--	--	--
XLOC_022703	No	No	No	No	--	--	--	--
XLOC_022704	No	No	No	No	--	--	--	--
XLOC_022710	No	No	No	No	--	--	U	--
XLOC_022711	No	No	No	No	--	--	--	K11296
XLOC_022712	No	No	No	No	--	--	--	--
XLOC_022716	No	No	No	No	ELU43798.1	--	--	--
XLOC_022725	DEG(M)	No	No	No	--	--	--	--
XLOC_022735	DEG(M)	DEG(M)	DEG(I+M)	DEG(M)	ABX79279.1	--	Z	--
XLOC_022738	DEG(M)	No	No	No	--	--	--	--
XLOC_022740	DEG(M)	No	DEG(I+M)	DEG(M)	--	--	--	--
XLOC_022741	DEG(M)	DEG(M)	DEG(I+M)	DEG(M)	--	--	--	--
XLOC_022742	No	No	No	No	--	--	--	--
XLOC_022758	No	No	No	No	--	--	--	--
XLOC_022759	No	No	DEG(I+M)	No	--	--	--	--
XLOC_022760	No	No	No	No	--	--	--	K10418
XLOC_022761	No	No	No	No	--	--	--	--
XLOC_022770	No	No	No	No	--	--	--	K06966
XLOC_022785	No	No	No	No	--	--	--	K15040
XLOC_022786	No	No	No	No	--	--	--	K15040
XLOC_022788	DEG(M)	DEG(M)	DEG(I+M)	DEG(M)	CCT70204.1	--	--	--
XLOC_022789	DEG(M)	No	No	No	CCT70204.1	--	--	--
XLOC_022795	DEG(M)	No	No	No	--	--	--	--
XLOC_022804	DEG(M)	No	No	No	--	--	--	--
XLOC_022807	No	No	No	No	KEP54506.1	--	O	K10580
XLOC_022812	DEG(M)	No	No	No	--	--	--	--
XLOC_022816	DEG(M)	No	No	No	--	--	--	--
XLOC_022818	DEG(M)	No	No	No	KDN50235.1	--	--	--

Differentially expressed gene (DEG) IDs	*C. microphyllum* root and shoot-specific (>2 fold)	*C. microphyllum* root and shoot-specific (>10 fold)	ICC4958 and *C. microphyllum* root-specific (>2 fold)	*C. microphyllum* root-specific (>2 fold)	Functional Annotation			
					NCBI nr IDs	TFs	KOG	KEGG
XLOC_022819	No	No	No	No	EUC66863.1	--	--	--
XLOC_022821	No	No	No	No	--	--	--	--
XLOC_022822	No	No	No	No	ETO84053.1	--	--	--
XLOC_022826	No	No	No	No	--	--	--	K02881
XLOC_022828	DEG(M)	No	DEG(I+M)	DEG(M)	--	--	--	--
XLOC_022831	No	No	No	No	--	--	--	--
XLOC_022832	No	No	No	No	--	--	--	--
XLOC_022836	No	No	DEG(I+M)	No	XP_004509365.1	--	--	--
XLOC_022837	DEG(M)	No	No	No	--	--	--	--
XLOC_022838	DEG(M)	No	No	No	--	--	--	--
XLOC_022839	DEG(M)	DEG(M)	DEG(I+M)	DEG(M)	CDO73081.1	--	B	K11254
XLOC_022840	No	No	No	No	KFM64386.1	--	Z	--
XLOC_022843	No	No	No	No	EUC65611.1	--	--	--
XLOC_022845	DEG(M)	No	No	No	--	--	--	--
XLOC_022849	No	No	No	No	--	--	J	K02988
XLOC_022853	No	No	No	No	XP_004504695.1	--	--	--
XLOC_022859	No	No	No	No	KNG51113.1	--	--	--
XLOC_022866	DEG(M)	DEG(M)	DEG(I+M)	DEG(M)	CCO36534.1	--	--	--
XLOC_022868	DEG(M)	No	No	No	KEP46106.1	--	O	--
XLOC_022869	No	No	No	No	XP_003041666.1	--	--	--
XLOC_022870	No	No	No	No	--	--	--	--
XLOC_022871	No	No	No	No	AFI80890.1	--	--	--
XLOC_022872	No	No	No	No	--	--	--	K02737
XLOC_022874	No	No	DEG(I+M)	No	--	--	--	--
XLOC_022878	No	No	No	No	XP_001654790.1	--	--	--
XLOC_022881	DEG(M)	No	No	No	XP_001897147.1	--	J	--
XLOC_022882	DEG(M)	No	No	No	XP_008914081.1	--	--	K04078
XLOC_022883	No	No	No	No	EUC62384.1	--	--	--
XLOC_022887	No	No	No	No	--	--	--	--
XLOC_022889	No	No	No	No	--	--	S	--
XLOC_022890	DEG(M)	No	No	No	KKA73802.1	Dof	Z	--
XLOC_022891	No	No	No	No	--	--	--	--
XLOC_022902	No	No	No	No	XP_003054561.1	--	--	--
XLOC_022903	DEG(M)	No	DEG(I+M)	DEG(M)	--	--	--	--
XLOC_022904	No	No	No	No	--	--	--	--
XLOC_022907	DEG(M)	No	No	No	CEL60939.1	--	--	--
XLOC_022908	DEG(M)	No	DEG(I+M)	DEG(M)	CEL53910.1	--	--	--
XLOC_022912	No	No	No	No	--	--	--	--
XLOC_022913	No	No	No	No	XP_001649228.1	--	--	--
XLOC_022922	No	No	No	No	EUC64037.1	--	--	--

Differentially expressed gene (DEG) IDs	*C. microphyllum* root and shoot-specific (>2 fold)	*C. microphyllum* root and shoot-specific (>10 fold)	ICC4958 and *C. microphyllum* root-specific (>2 fold)	*C. microphyllum* root-specific (>2 fold)	Functional Annotation			
					NCBI nr IDs	TFs	KOG	KEGG
XLOC_022923	No	No	No	No	XP_001655819.1	--	J	--
XLOC_022924	DEG(M)	No	No	No	--	--	--	--
XLOC_022927	No	No	No	No	--	--	--	--
XLOC_022928	No	No	No	No	XP_001659192.1	--	Z	--
XLOC_022929	No	No	No	No	XP_001659192.1	--	Z	--
XLOC_022934	DEG(M)	No	No	No	--	--	--	K02921
XLOC_022950	No	No	No	No	--	--	--	--
XLOC_022951	No	No	No	No	CCO30365.1	--	--	--
XLOC_022954	No	No	No	No	XP_001946901.1	--	C	--
XLOC_022959	No	No	No	No	--	--	--	--
XLOC_022963	No	No	No	No	--	--	--	--
XLOC_022977	DEG(M)	No	No	No	--	--	--	--
XLOC_022986	No	No	No	No	--	--	--	--
XLOC_023001	No	No	No	No	--	--	--	--
XLOC_023006	DEG(M)	No	No	No	--	--	--	--
XLOC_023008	No	No	No	No	--	--	--	--
XLOC_023010	No	No	DEG(I+M)	No	XP_004516070.1	--	R	--
XLOC_023012	No	No	No	No	EUC56908.1	--	--	--
XLOC_023013	DEG(M)	No	No	No	--	--	--	--
XLOC_023014	No	No	No	No	ETO99857.1	--	--	--
XLOC_023025	No	No	No	No	--	--	--	--
XLOC_023026	No	No	No	No	--	--	--	--
XLOC_023028	No	No	No	No	--	--	J	K02945
XLOC_023029	No	No	No	No	KDN36148.1	--	--	--
XLOC_023038	DEG(M)	No	No	No	KDN51023.1	--	T	K06268
XLOC_023048	No	No	No	No	--	--	--	--
XLOC_023051	DEG(M)	DEG(M)	No	No	NP_001119693.1	--	J	K02901
XLOC_023052	No	No	No	No	--	--	--	--
XLOC_023053	No	No	No	No	--	--	--	--
XLOC_023062	No	No	DEG(I+M)	No	XP_004515260.1	--	--	--
XLOC_023063	DEG(M)	No	No	No	--	--	T	K14512
XLOC_023064	No	No	No	No	--	--	T	--
XLOC_023070	No	No	No	No	--	--	--	--
XLOC_023073	No	No	No	No	KDQ13728.1	--	J	K02929
XLOC_023074	No	No	No	No	KDN48374.1	--	J	--
XLOC_023075	DEG(M)	DEG(M)	DEG(I+M)	DEG(M)	XP_008914558.1	--	J	--
XLOC_023079	No	No	No	No	--	--	--	--
XLOC_023084	DEG(M)	No	DEG(I+M)	DEG(M)	XP_004495384.1	--	--	--
XLOC_023086	No	No	No	No	XP_004492392.1	--	G	K03841
XLOC_023101	No	No	No	No	KDN43503.1	--	J	--

**Differentially expressed gene (DEG) IDs**	***C. microphyllum* root and shoot-specific (>2 fold)**	***C. microphyllum* root and shoot-specific (>10 fold)**	**ICC4958 and *C. microphyllum* root-specific (>2 fold)**	***C. microphyllum* root-specific (>2 fold)**	**Functional Annotation**			
					**NCBI nr IDs**	**TFs**	**KOG**	**KEGG**
XLOC_023107	No	No	No	No	--	--	--	--
XLOC_023109	No	No	DEG(I+M)	No	--	--	--	K10712
XLOC_023123	No	No	No	No	CEL61077.1	--	--	--
XLOC_023132	DEG(M)	No	No	No	EUC67185.1	--	--	--
XLOC_023133	DEG(M)	No	No	No	--	--	--	--
XLOC_023134	DEG(M)	No	DEG(I+M)	DEG(M)	--	--	--	--
XLOC_023135	DEG(M)	No	DEG(I+M)	DEG(M)	CCO31970.1	--	J	--
XLOC_023136	DEG(M)	No	DEG(I+M)	DEG(M)	--	--	--	--
XLOC_023148	No	No	No	No	XP_004487950.1	--	--	--
XLOC_023154	DEG(M)	No	No	No	EUC65583.1	--	--	--
XLOC_023155	No	No	No	No	EUC65583.1	--	--	--
XLOC_023156	No	No	No	No	KND88492.1	--	J	--
XLOC_023157	DEG(M)	No	No	No	KND88492.1	--	J	--
XLOC_023170	No	No	No	No	AHG55039.1	--	O	--
XLOC_023172	No	No	No	No	CEL62091.1	--	--	K01262
XLOC_023173	No	No	No	No	--	--	--	--
XLOC_023180	DEG(M)	DEG(M)	No	No	--	--	--	--
XLOC_023186	DEG(M)	No	No	No	ELU40828.1	--	--	--
XLOC_023192	No	No	No	No	XP_001949588.1	FAR1	O	--
XLOC_023199	DEG(M)	DEG(M)	DEG(I+M)	DEG(M)	--	--	O	--
XLOC_023201	DEG(M)	DEG(M)	No	No	--	--	--	--
XLOC_023202	No	No	No	No	--	--	--	--
XLOC_023203	DEG(M)	No	No	No	--	--	--	--
XLOC_023204	No	No	No	No	XP_003054659.1	--	--	--
XLOC_023205	DEG(M)	No	No	No	CEL52081.1	--	--	--
XLOC_023206	No	No	No	No	--	--	--	--
XLOC_023207	DEG(M)	No	DEG(I+M)	DEG(M)	--	--	--	--
XLOC_023209	No	No	No	No	XP_003302758.1	--	--	--
XLOC_023210	No	No	No	No	--	--	--	K02936
XLOC_023216	DEG(M)	DEG(M)	No	No	XP_003052202.1	--	J	--
XLOC_023218	No	No	No	No	XP_003046131.1	--	--	--
XLOC_023221	No	No	No	No	--	--	--	--
XLOC_023227	No	No	No	No	--	--	--	--
XLOC_023229	No	No	No	No	ETI57609.1	--	--	--
XLOC_023231	No	No	No	No	XP_002511345.1	--	U	K12393
XLOC_023234	No	No	No	No	CEL62515.1	--	--	--
XLOC_023236	No	No	No	No	XP_012073937.1	--	--	K12124
XLOC_023238	No	No	DEG(I+M)	No	--	--	--	--
XLOC_023246	No	No	No	No	XP_008900668.1	--	--	--
XLOC_023247	No	No	No	No	--	--	--	--

Differentially expressed gene (DEG) IDs	*C. microphyllum* root and shoot-specific (>2 fold)	*C. microphyllum* root and shoot-specific (>10 fold)	ICC4958 and *C. microphyllum* root-specific (>2 fold)	*C. microphyllum* root-specific (>2 fold)	Functional Annotation			
					NCBI nr IDs	TFs	KOG	KEGG
XLOC_023248	No	No	No	No	--	--	--	--
XLOC_023249	DEG(M)	No	No	No	KDN49425.1	--	--	--
XLOC_023255	No	No	No	No	--	--	--	--
XLOC_023258	No	No	No	No	XP_008185968.1	--	P	K05853
XLOC_023264	No	No	No	No	--	--	--	--
XLOC_023268	No	No	No	No	--	--	--	--
XLOC_023270	No	No	No	No	XP_001649836.1	--	--	--
XLOC_023272	No	No	No	No	KDN35397.1	--	--	--
XLOC_023275	DEG(M)	No	No	No	--	--	--	--
XLOC_023276	No	No	No	No	--	--	--	--
XLOC_023280	DEG(M)	No	No	No	--	--	--	--
XLOC_023281	No	No	No	No	--	--	--	--
XLOC_023284	No	No	No	No	CCO29498.1	--	--	--
XLOC_023287	No	No	No	No	XP_008892367.1	--	--	--
XLOC_023289	No	No	No	No	--	--	--	--
XLOC_023292	DEG(M)	No	DEG(I+M)	DEG(M)	--	--	--	--
XLOC_023295	No	No	No	No	--	--	--	--
XLOC_023298	DEG(M)	No	No	No	--	--	P	K00522
XLOC_023299	DEG(M)	No	No	No	--	--	--	--
XLOC_023304	No	No	No	No	CCO28463.1	--	--	--
XLOC_023305	No	No	No	No	--	--	--	--
XLOC_023306	DEG(M)	No	No	No	--	--	--	--
XLOC_023309	No	No	No	No	XP_004490656.1	--	TR	--
XLOC_023313	No	No	No	No	--	--	--	--
XLOC_023317	No	No	No	No	--	--	--	--
XLOC_023318	No	No	No	No	--	--	--	--
XLOC_023320	No	No	No	No	--	--	--	--
XLOC_023324	No	No	No	No	--	--	--	K12897
XLOC_023325	DEG(M)	No	No	No	CEL57862.1	--	--	--
XLOC_023327	No	No	No	No	XP_007019452.1	--	G	--
XLOC_023331	No	No	No	No	--	--	--	K04125
XLOC_023338	DEG(M)	No	No	No	--	--	P	K04564
XLOC_023342	DEG(M)	No	DEG(I+M)	DEG(M)	--	--	--	--
XLOC_023346	DEG(M)	DEG(M)	No	No	KIL91706.1	--	J	--
XLOC_023348	No	No	No	No	XP_008890663.1	--	I	K01858
XLOC_023351	DEG(M)	No	No	No	CCO35176.1	--	--	--
XLOC_023355	No	No	No	No	--	--	--	--
XLOC_023357	No	No	No	No	XP_008889762.1	--	C	--
XLOC_023359	DEG(M)	No	No	No	ELU44881.1	--	--	--
XLOC_023362	No	No	No	No	--	--	--	--

Differentially expressed gene (DEG) IDs	*C. microphyllum* root and shoot-specific (>2 fold)	*C. microphyllum* root and shoot-specific (>10 fold)	ICC4958 and *C. microphyllum* root-specific (>2 fold)	*C. microphyllum* root-specific (>2 fold)	Functional Annotation			
					NCBI nr IDs	TFs	KOG	KEGG
XLOC_023371	DEG(M)	No	No	No	--	--	--	--
XLOC_023372	No	No	No	No	--	--	--	--
XLOC_023377	DEG(M)	No	No	No	--	--	--	--
XLOC_023379	No	No	No	No	XP_008903194.1	--	--	--
XLOC_023380	DEG(M)	No	No	No	XP_008903194.1	--	--	--
XLOC_023382	No	No	No	No	--	--	--	--
XLOC_023383	No	No	No	No	--	--	--	--
XLOC_023393	No	No	No	No	--	--	--	--
XLOC_023394	No	No	No	No	XP_002523509.1	--	--	--
XLOC_023395	No	No	No	No	--	--	--	K03878
XLOC_023396	No	No	No	No	--	HD-ZIP	--	--
XLOC_023399	DEG(M)	DEG(M)	No	No	--	--	--	--
XLOC_023400	No	No	No	No	--	--	--	--
XLOC_023409	DEG(M)	DEG(M)	DEG(I+M)	DEG(M)	--	--	--	--
XLOC_023411	No	No	No	No	NP_001119639.1	--	UO	--
XLOC_023412	No	No	No	No	--	--	--	--
XLOC_023416	No	No	No	No	--	--	--	--
XLOC_023418	DEG(M)	No	No	No	AIH15715.1	--	--	--
XLOC_023419	No	No	No	No	NP_001155629.1	--	--	--
XLOC_023420	No	No	No	No	NP_001155629.1	--	--	K04710
XLOC_023422	No	No	No	No	EWM21485.1	--	J	--
XLOC_023429	No	No	No	No	EWG47344.1	--	R	K06185
XLOC_023431	No	No	No	No	XP_008895053.1	--	--	--
XLOC_023436	DEG(M)	No	No	No	--	--	--	--
XLOC_023437	No	No	No	No	BAR91090.1	--	Z	--
XLOC_023438	DEG(M)	DEG(M)	DEG(I+M)	DEG(M)	XP_002519975.1	--	G	K15633
XLOC_023439	No	No	No	No	--	--	--	--
XLOC_023440	DEG(M)	No	No	No	--	--	--	--
XLOC_023441	No	No	No	No	XP_008903352.1	--	--	--
XLOC_023442	No	No	No	No	--	--	--	K10418
XLOC_023444	DEG(M)	No	No	No	--	--	--	--
XLOC_023445	DEG(M)	No	No	No	--	--	--	--
XLOC_023446	No	No	No	No	XP_001943068.1	--	O	--
XLOC_023447	DEG(M)	No	No	No	XP_008891756.1	--	--	--
XLOC_023450	No	No	No	No	--	--	--	--
XLOC_023451	No	No	No	No	ELU41560.1	--	--	--
XLOC_023456	No	No	No	No	--	--	--	--
XLOC_023457	No	No	No	No	--	--	--	--
XLOC_023460	No	No	No	No	XP_001932096.1	--	C	--
XLOC_023465	No	No	No	No	--	--	F	K00940

Differentially expressed gene (DEG) IDs	*C. microphyllum* root and shoot-specific (>2 fold)	*C. microphyllum* root and shoot-specific (>10 fold)	ICC4958 and *C. microphyllum* root-specific (>2 fold)	*C. microphyllum* root-specific (>2 fold)	Functional Annotation			
					NCBI nr IDs	TFs	KOG	KEGG
XLOC_023466	No	No	No	No	--	--	--	--
XLOC_023468	No	No	No	No	ETI53504.1	--	--	--
XLOC_023473	No	No	No	No	--	--	--	--
XLOC_023479	No	No	No	No	CCO31088.1	--	--	--
XLOC_023480	No	No	No	No	NP_001155656.1	--	J	K03249
XLOC_023482	No	No	No	No	XP_003039717.1	--	--	--
XLOC_023485	No	No	DEG(I+M)	No	XP_004498289.1	--	--	--
XLOC_023486	No	No	No	No	--	--	--	--
XLOC_023487	No	No	No	No	--	--	--	--
XLOC_023491	DEG(M)	No	No	No	--	--	--	--
XLOC_023495	No	No	No	No	XP_008908909.1	--	--	--
XLOC_023496	No	No	No	No	--	--	--	--
XLOC_023498	No	No	No	No	--	--	--	--
XLOC_023500	No	No	No	No	XP_008188531.1	--	--	--
XLOC_023502	DEG(M)	No	No	No	--	--	--	--
XLOC_023505	DEG(M)	DEG(M)	DEG(I+M)	DEG(M)	--	--	K	--
XLOC_023518	No	No	No	No	KEP51535.1	--	--	--
XLOC_023524	No	No	No	No	--	--	--	--
XLOC_023525	No	No	No	No	--	--	--	K17893
XLOC_023526	No	No	No	No	--	--	--	--
XLOC_023527	No	No	No	No	--	--	--	--
XLOC_023542	DEG(M)	No	No	No	KEP50672.1	--	--	--
XLOC_023543	No	No	No	No	XP_004341583.1	--	J	K02960
XLOC_023552	No	No	No	No	--	--	--	--
XLOC_023557	No	No	DEG(I+M)	No	XP_004507674.1	--	--	--
XLOC_023564	DEG(M)	No	DEG(I+M)	DEG(M)	CCO35501.1	--	--	--
XLOC_023565	No	No	No	No	--	--	--	--
XLOC_023575	No	No	No	No	XP_007705634.1	--	--	--
XLOC_023576	DEG(M)	No	No	No	--	--	--	--
XLOC_023578	No	No	No	No	--	--	--	--
XLOC_023582	No	No	No	No	--	--	--	--
XLOC_023583	No	No	No	No	--	--	--	--
XLOC_023586	DEG(M)	DEG(M)	DEG(I+M)	DEG(M)	--	--	--	--
XLOC_023587	DEG(M)	DEG(M)	DEG(I+M)	DEG(M)	--	--	--	--
XLOC_023588	DEG(M)	No	No	No	EUC62060.1	--	--	--
XLOC_023589	DEG(M)	No	No	No	CEL51991.1	--	--	--
XLOC_023590	DEG(M)	No	No	No	--	--	--	--
XLOC_023600	DEG(M)	No	DEG(I+M)	DEG(M)	XP_004515911.1	--	R	--
XLOC_023602	No	No	No	No	--	--	O	--
XLOC_023610	DEG(M)	DEG(M)	DEG(I+M)	DEG(M)	--	--	--	--

Differentially expressed gene (DEG) IDs	*C. microphyllum* root and shoot-specific (>2 fold)	*C. microphyllum* root and shoot-specific (>10 fold)	ICC4958 and *C. microphyllum* root-specific (>2 fold)	*C. microphyllum* root-specific (>2 fold)	Functional Annotation			
					NCBI nr IDs	TFs	KOG	KEGG
XLOC_023612	No	No	No	No	--	--	--	--
XLOC_023621	No	No	No	No	XP_004500690.1	--	G	--
XLOC_023625	DEG(M)	No	No	No	--	--	C	K03936
XLOC_023627	No	No	No	No	--	--	--	--
XLOC_023629	No	No	No	No	--	--	--	--
XLOC_023630	No	No	No	No	--	--	C	K00128
XLOC_023631	DEG(M)	No	No	No	XP_003053143.1	--	K	--
XLOC_023633	No	No	No	No	XP_008913871.1	--	--	--
XLOC_023639	No	No	No	No	XP_003046739.1	--	--	--
XLOC_023640	No	No	No	No	ETM54825.1	--	--	--
XLOC_023641	No	No	No	No	NP_001119609.1	--	R	K13095
XLOC_023642	No	No	No	No	XP_002528397.1	--	--	--
XLOC_023643	No	No	No	No	--	--	--	--
XLOC_023644	No	No	No	No	CCO26181.1	--	--	K11000
XLOC_023646	No	No	No	No	NP_001155908.1	--	C	K00235
XLOC_023647	DEG(M)	No	No	No	ETP53063.1	--	C	--
XLOC_023650	No	No	No	No	--	--	--	--
XLOC_023659	No	No	No	No	XP_001944790.2	--	G	--
XLOC_023662	No	No	No	No	AJQ30120.1	--	C	--
XLOC_023663	No	No	No	No	XP_001948008.1	--	O	--
XLOC_023664	No	No	No	No	--	--	--	--
XLOC_023666	No	No	No	No	--	--	--	--
XLOC_023667	No	No	No	No	KDN33554.1	--	--	--
XLOC_023668	No	No	No	No	XP_002532723.1	--	O	--
XLOC_023670	No	No	No	No	--	--	--	--
XLOC_023675	No	No	No	No	ETM50020.1	--	--	--
XLOC_023676	No	No	No	No	XP_002520494.1	bHLH	U	K07904
XLOC_023684	No	No	No	No	KEP50140.1	--	--	--
XLOC_023685	No	No	No	No	CCO28402.1	--	--	--
XLOC_023686	No	No	No	No	XP_001652705.1	--	--	--
XLOC_023688	No	No	No	No	CEL55573.1	--	--	--
XLOC_023690	No	No	No	No	--	--	--	--
XLOC_023697	DEG(M)	No	No	No	--	--	--	--
XLOC_023699	No	No	No	No	--	--	--	--
XLOC_023700	DEG(M)	No	No	No	--	--	--	--
XLOC_023701	DEG(M)	DEG(M)	No	No	--	--	--	--
XLOC_023702	DEG(M)	DEG(M)	No	No	--	--	--	--
XLOC_023704	DEG(M)	DEG(M)	No	No	NP_001155698.1	--	J	--
XLOC_023705	No	No	No	No	--	--	--	K17987
XLOC_023707	DEG(M)	No	No	No	EUC54083.1	--	--	--

Differentially expressed gene (DEG) IDs	*C. microphyllum* root and shoot-specific (>2 fold)	*C. microphyllum* root and shoot-specific (>10 fold)	ICC4958 and *C. microphyllum* root-specific (>2 fold)	*C. microphyllum* root-specific (>2 fold)	Functional Annotation			
					NCBI nr IDs	TFs	KOG	KEGG
XLOC_023709	No	No	No	No	--	--	--	--
XLOC_023711	No	No	No	No	--	--	--	--
XLOC_023712	No	No	No	No	--	--	--	--
XLOC_023717	No	No	No	No	--	--	--	--
XLOC_023719	No	No	No	No	XP_001943983.1	--	I	--
XLOC_023723	No	No	No	No	XP_003053117.1	--	--	--
XLOC_023724	DEG(M)	DEG(M)	No	No	CCT61861.1	--	--	--
XLOC_023726	No	No	No	No	XP_008896359.1	--	--	--
XLOC_023727	No	No	No	No	--	--	--	--
XLOC_023730	No	No	No	No	--	--	--	--
XLOC_023732	No	No	No	No	--	--	R	--
XLOC_023733	No	No	DEG(I+M)	No	--	--	--	--
XLOC_023734	No	No	DEG(I+M)	No	XP_012571439.1	--	--	--
XLOC_023739	DEG(M)	No	No	No	--	--	--	--
XLOC_023741	No	No	No	No	EUC62188.1	--	--	--
XLOC_023743	No	No	No	No	--	--	--	--
XLOC_023745	No	No	No	No	--	--	--	--
XLOC_023746	No	No	No	No	--	--	--	--
XLOC_023750	No	No	DEG(I+M)	No	--	--	--	--
XLOC_023751	No	No	No	No	--	--	--	--
XLOC_023752	No	No	No	No	KDN46576.1	--	--	--
XLOC_023753	No	No	No	No	--	--	--	--
XLOC_023758	DEG(M)	DEG(M)	DEG(I+M)	DEG(M)	XP_002901699.1	--	J	--
XLOC_023761	No	No	No	No	XP_003052026.1	--	--	--
XLOC_023763	No	No	No	No	XP_002909233.1	--	--	--
XLOC_023764	No	No	No	No	XP_001935655.1	--	Z	K05755
XLOC_023770	No	No	No	No	--	--	--	--
XLOC_023772	DEG(M)	No	No	No	CCT67093.1	--	--	--
XLOC_023775	DEG(M)	No	No	No	AIH15714.1	--	--	--
XLOC_023782	No	No	No	No	CEL61733.1	--	--	--
XLOC_023784	DEG(M)	DEG(M)	No	No	EXL96857.1	--	G	--
XLOC_023785	No	No	No	No	--	--	--	--
XLOC_023787	No	No	DEG(I+M)	No	--	--	--	--
XLOC_023800	No	No	No	No	--	--	--	--
XLOC_023802	No	No	No	No	KEP50502.1	--	--	--
XLOC_023803	No	No	No	No	--	--	--	--
XLOC_023805	DEG(M)	DEG(M)	No	No	--	--	--	--
XLOC_023807	No	No	DEG(I+M)	No	--	--	--	--
XLOC_023815	DEG(M)	No	DEG(I+M)	DEG(M)	--	--	--	--
XLOC_023819	No	No	No	No	--	--	--	--

Differentially expressed gene (DEG) IDs	*C. microphyllum* root and shoot-specific (>2 fold)	*C. microphyllum* root and shoot-specific (>10 fold)	ICC4958 and *C. microphyllum* root-specific (>2 fold)	*C. microphyllum* root-specific (>2 fold)	Functional Annotation			
					NCBI nr IDs	TFs	KOG	KEGG
XLOC_023823	No	No	No	No	XP_003054115.1	--	--	--
XLOC_023824	DEG(M)	DEG(M)	DEG(I+M)	DEG(M)	EXM01674.1	--	--	--
XLOC_023826	DEG(M)	No	No	No	NP_001156316.1	--	J	K02993
XLOC_023830	No	No	No	No	ELU37506.1	--	--	--
XLOC_023832	DEG(M)	No	No	No	--	--	--	--
XLOC_023833	No	No	No	No	--	--	--	--
XLOC_023835	No	No	No	No	--	--	--	--
XLOC_023837	No	No	No	No	--	--	--	--
XLOC_023838	No	No	No	No	CEL56402.1	--	--	--
XLOC_023840	No	No	No	No	NP_001155452.1	--	--	--
XLOC_023841	DEG(M)	DEG(M)	DEG(I+M)	DEG(M)	--	--	R	--
XLOC_023843	No	No	No	No	CEL55930.1	--	--	--
XLOC_023851	DEG(M)	DEG(M)	No	No	--	--	--	--
XLOC_023853	No	No	No	No	--	--	U	--
XLOC_023854	DEG(M)	DEG(M)	DEG(I+M)	DEG(M)	XP_010258877.1	--	G	K09873
XLOC_023856	DEG(M)	No	DEG(I+M)	DEG(M)	--	--	--	--
XLOC_023862	No	No	No	No	--	--	--	--
XLOC_023869	No	No	No	No	ETM33105.1	--	--	--
XLOC_023870	No	No	No	No	--	--	--	--
XLOC_023871	DEG(M)	No	No	No	--	--	--	--
XLOC_023872	No	No	No	No	--	--	--	--
XLOC_023874	No	No	No	No	--	--	--	--
XLOC_023876	No	No	No	No	--	--	--	--
XLOC_023877	No	No	No	No	AGN70891.1	--	--	--
XLOC_023879	No	No	No	No	ETI53952.1	--	--	--
XLOC_023882	No	No	No	No	--	--	--	--
XLOC_023885	DEG(M)	No	No	No	KDN46944.1	--	--	--
XLOC_023886	No	No	No	No	XP_001767282.1	--	J	K02908
XLOC_023888	No	No	No	No	--	--	--	--
XLOC_023892	No	No	No	No	--	--	--	K02723
XLOC_023893	DEG(M)	No	No	No	CCO33259.1	--	--	--
XLOC_023895	DEG(M)	DEG(M)	DEG(I+M)	DEG(M)	CEL53910.1	--	--	--
XLOC_023898	No	No	No	No	--	--	--	--
XLOC_023899	No	No	No	No	--	--	--	--
XLOC_023903	DEG(M)	No	No	No	KDN46200.1	--	--	--
XLOC_023905	DEG(M)	No	No	No	EUC64076.1	--	--	--
XLOC_023906	No	No	No	No	--	--	--	K12125
XLOC_023907	DEG(M)	No	No	No	XP_009539528.1	--	--	K00413
XLOC_023908	No	No	No	No	--	--	--	--
XLOC_023910	No	No	No	No	--	--	--	--

**Differentially expressed gene (DEG) IDs**	***C. microphyllum* root and shoot-specific (>2 fold)**	***C. microphyllum* root and shoot-specific (>10 fold)**	**ICC4958 and *C. microphyllum* root-specific (>2 fold)**	***C. microphyllum* root-specific (>2 fold)**	**Functional Annotation**			
					**NCBI nr IDs**	**TFs**	**KOG**	**KEGG**
XLOC_023911	No	No	No	No	--	--	--	--
XLOC_023912	No	No	No	No	ETP52312.1	--	--	--
XLOC_023914	No	No	No	No	--	--	Z	K05757
XLOC_023915	No	No	No	No	--	--	K	--
XLOC_023918	No	No	No	No	XP_003046524.1	--	--	--
XLOC_023919	DEG(M)	No	DEG(I+M)	DEG(M)	ETI33395.1	--	--	--
XLOC_023927	No	No	No	No	--	--	--	--
XLOC_023928	DEG(M)	No	DEG(I+M)	DEG(M)	--	--	--	--
XLOC_023931	No	No	No	No	XP_004505802.2	--	--	--
XLOC_023935	No	No	No	No	--	--	--	--
XLOC_023936	No	No	No	No	--	--	--	--
XLOC_023937	No	No	No	No	XP_003299976.1	--	E	--
XLOC_023942	No	No	No	No	--	--	--	K04354
XLOC_023943	No	No	No	No	--	--	--	--
XLOC_023944	No	No	No	No	--	--	--	--
XLOC_023946	No	No	No	No	XP_003045219.1	--	--	--
XLOC_023951	No	No	No	No	NP_001191854.1	--	C	K02146
XLOC_023954	No	No	No	No	XP_008183837.1	--	--	--
XLOC_023955	No	No	No	No	--	--	--	--
XLOC_023956	No	No	No	No	--	--	--	--
XLOC_023959	DEG(M)	No	No	No	--	--	--	--
XLOC_023962	No	No	No	No	XP_001659427.1	--	C	--
XLOC_023963	No	No	No	No	--	--	--	--
XLOC_023969	No	No	No	No	XP_004508863.1	--	T	--
XLOC_023970	No	No	No	No	--	--	K	K03627
XLOC_023971	No	No	No	No	--	--	--	--
XLOC_023972	DEG(M)	No	No	No	--	--	--	--
XLOC_023974	No	No	No	No	--	--	--	--
XLOC_023976	No	No	No	No	--	--	--	--
XLOC_023978	No	No	No	No	CCO26181.1	--	--	--
XLOC_023979	No	No	No	No	--	--	--	K03671
XLOC_023980	No	No	No	No	--	--	--	K02890
XLOC_023981	No	No	No	No	--	--	--	--
XLOC_023982	No	No	No	No	--	--	--	--
XLOC_023991	No	No	No	No	KEP53726.1	--	--	--
XLOC_023996	No	No	No	No	--	--	--	--
XLOC_023998	No	No	No	No	--	--	--	--
XLOC_024002	No	No	DEG(I+M)	No	XP_004501530.1	C2H2	--	--
XLOC_024003	No	No	No	No	--	--	--	--
XLOC_024007	No	No	No	No	--	--	--	--

Differentially expressed gene (DEG) IDs	*C. microphyllum* root and shoot-specific (>2 fold)	*C. microphyllum* root and shoot-specific (>10 fold)	ICC4958 and *C. microphyllum* root-specific (>2 fold)	*C. microphyllum* root-specific (>2 fold)	Functional Annotation			
					NCBI nr IDs	TFs	KOG	KEGG
XLOC_024008	DEG(M)	No	No	No	NP_001129418.1	--	J	K02978
XLOC_024009	DEG(M)	No	No	No	--	--	--	K12741
XLOC_024010	DEG(M)	DEG(M)	DEG(I+M)	DEG(M)	--	--	--	--
XLOC_024011	DEG(M)	No	DEG(I+M)	DEG(M)	--	--	--	--
XLOC_024012	No	No	No	No	CCO31750.1	--	--	--
XLOC_024014	No	No	No	No	XP_001656266.1	--	I	K04712
XLOC_024015	No	No	No	No	--	--	--	--
XLOC_024021	No	No	No	No	EUC57147.1	--	--	--
XLOC_024022	No	No	No	No	EUC62809.1	--	--	--
XLOC_024023	No	No	No	No	CEL63900.1	--	--	--
XLOC_024024	No	No	No	No	CEL51487.1	--	--	--
XLOC_024028	DEG(M)	No	No	No	--	--	--	K11251
XLOC_024029	DEG(M)	No	No	No	KHN88676.1	--	J	--
XLOC_024030	No	No	No	No	CCO28053.1	--	--	--
XLOC_024033	No	No	No	No	--	--	--	--
XLOC_024034	DEG(M)	No	No	No	--	--	--	--
XLOC_024036	DEG(M)	No	DEG(I+M)	DEG(M)	BAH57273.1	--	U	--
XLOC_024037	No	No	DEG(I+M)	No	--	--	R	--
XLOC_024038	No	No	No	No	--	--	--	K13126
XLOC_024040	DEG(M)	No	No	No	XP_004500748.1	--	--	--
XLOC_024046	DEG(M)	No	No	No	--	--	--	--
XLOC_024049	No	No	No	No	--	--	--	--
XLOC_024055	No	No	No	No	AJQ30109.1	--	G	K01689
XLOC_024056	No	No	No	No	--	--	--	--
XLOC_024062	No	No	No	No	--	--	--	--
XLOC_024063	No	No	No	No	--	--	--	--
XLOC_024067	No	No	No	No	XP_004510958.1	C2H2	R	--
XLOC_024076	No	No	No	No	--	--	--	--
XLOC_024077	No	No	DEG(I+M)	No	XP_012570749.1	--	--	--
XLOC_024078	No	No	No	No	XP_001948145.1	--	--	--
XLOC_024080	No	No	No	No	--	--	--	--
XLOC_024084	No	No	No	No	--	--	--	--
XLOC_024088	No	No	No	No	EUC65200.1	--	--	--
XLOC_024089	DEG(M)	No	DEG(I+M)	DEG(M)	--	--	--	--
XLOC_024090	No	No	No	No	--	--	--	--
XLOC_024095	DEG(M)	No	No	No	BAF98295.1	--	--	K01770
XLOC_024097	No	No	No	No	--	--	--	--
XLOC_024100	No	No	DEG(I+M)	No	--	--	--	--
XLOC_024101	No	No	No	No	--	--	J	K03263
XLOC_024102	DEG(M)	No	No	No	CAB45530.1	--	J	--

Differentially expressed gene (DEG) IDs	*C. microphyllum* root and shoot-specific (>2 fold)	*C. microphyllum* root and shoot-specific (>10 fold)	ICC4958 and *C. microphyllum* root-specific (>2 fold)	*C. microphyllum* root-specific (>2 fold)	Functional Annotation			
					NCBI nr IDs	TFs	KOG	KEGG
XLOC_024105	No	No	No	No	--	--	O	--
XLOC_024106	DEG(M)	No	No	No	--	--	O	--
XLOC_024111	No	No	No	No	XP_008902912.1	Trihelix	G	K00134
XLOC_024118	No	No	No	No	CCO31896.1	--	--	--
XLOC_024122	No	No	No	No	XP_003044029.1	--	--	--
XLOC_024123	No	No	No	No	ETP45476.1	--	--	--
XLOC_024124	No	No	DEG(I+M)	No	--	WRKY	--	--
XLOC_024127	DEG(M)	DEG(M)	DEG(I+M)	DEG(M)	KEP47630.1	--	J	--
XLOC_024128	DEG(M)	No	No	No	KEP47630.1	--	J	K02974
XLOC_024134	DEG(M)	No	No	No	AGJ03605.1	bZIP	J	--
XLOC_024147	DEG(M)	No	No	No	ETI50626.1	--	--	K01115
XLOC_024148	DEG(M)	No	No	No	XP_009519168.1	--	Q	--
XLOC_024151	No	No	No	No	--	--	--	K13356
XLOC_024156	No	No	No	No	EUC64361.1	--	--	--
XLOC_024157	DEG(M)	No	No	No	XP_001647834.1	--	J	--
XLOC_024162	No	No	No	No	XP_004514411.1	--	--	--
XLOC_024164	No	No	No	No	XP_008911755.1	--	O	--
XLOC_024165	DEG(M)	No	No	No	--	--	--	--
XLOC_024166	No	No	No	No	XP_008911755.1	--	O	--
XLOC_024167	DEG(M)	No	No	No	--	--	--	--
XLOC_024170	DEG(M)	No	No	No	--	--	--	--
XLOC_024173	No	No	No	No	XP_001893693.1	--	J	K02995
XLOC_024174	DEG(M)	No	No	No	--	--	--	--
XLOC_024175	DEG(M)	No	No	No	XP_009517215.1	--	J	K02893
XLOC_024177	DEG(M)	DEG(M)	DEG(I+M)	DEG(M)	XP_009517215.1	--	J	--
XLOC_024178	DEG(M)	No	DEG(I+M)	DEG(M)	--	--	--	--
XLOC_024182	DEG(M)	DEG(M)	No	No	--	--	--	--
XLOC_024183	DEG(M)	No	No	No	EUC66298.1	--	--	--
XLOC_024184	DEG(M)	DEG(M)	No	No	EMT61608.1	--	J	--
XLOC_024186	DEG(M)	No	No	No	EXL86692.1	--	J	--
XLOC_024193	No	No	No	No	--	--	--	K00074
XLOC_024197	DEG(M)	No	No	No	--	--	--	--
XLOC_024201	DEG(M)	No	DEG(I+M)	DEG(M)	--	--	--	--
XLOC_024208	No	No	No	No	XP_008891344.1	--	O	K01802
XLOC_024214	No	No	No	No	--	--	--	--
XLOC_024220	No	No	No	No	--	--	--	--
XLOC_024221	No	No	No	No	XP_002999028.1	--	Q	--
XLOC_024224	No	No	No	No	--	--	--	--
XLOC_024225	No	No	No	No	--	--	G	K00688
XLOC_024226	No	No	No	No	--	--	--	--

Differentially expressed gene (DEG) IDs	*C. microphyllum* root and shoot-specific (>2 fold)	*C. microphyllum* root and shoot-specific (>10 fold)	ICC4958 and *C. microphyllum* root-specific (>2 fold)	*C. microphyllum* root-specific (>2 fold)	Functional Annotation			
					NCBI nr IDs	TFs	KOG	KEGG
XLOC_024228	No	No	No	No	XP_003048236.1	--	--	--
XLOC_024230	No	No	No	No	--	--	--	--
XLOC_024232	No	No	No	No	--	--	--	--
XLOC_024233	No	No	No	No	--	--	--	K02724
XLOC_024234	No	No	No	No	--	--	--	--
XLOC_024239	No	No	No	No	--	--	--	--
XLOC_024247	No	No	No	No	--	--	--	--
XLOC_024249	DEG(M)	DEG(M)	No	No	--	--	--	--
XLOC_024251	DEG(M)	No	No	No	NP_001129593.1	--	J	K02984
XLOC_024255	No	No	No	No	CEL62468.1	--	--	--
XLOC_024256	DEG(M)	No	No	No	--	--	--	K00640
XLOC_024257	No	No	No	No	--	--	--	--
XLOC_024258	DEG(M)	No	No	No	XP_008889910.1	--	C	--
XLOC_024260	No	No	No	No	CCG34081.1	--	O	K03094
XLOC_024262	No	No	No	No	ETL96682.1	--	--	--
XLOC_024264	DEG(M)	No	No	No	--	--	--	--
XLOC_024265	No	No	No	No	--	--	--	--
XLOC_024266	No	No	No	No	XP_001951963.1	--	--	--
XLOC_024267	DEG(M)	No	No	No	--	--	--	--
XLOC_024269	DEG(M)	No	No	No	--	--	--	--
XLOC_024272	DEG(M)	No	No	No	EUC55036.1	--	--	--
XLOC_024273	DEG(M)	No	No	No	EUC55036.1	--	--	--
XLOC_024274	No	No	No	No	--	--	--	--
XLOC_024275	DEG(M)	No	No	No	CEL55059.1	--	--	--
XLOC_024283	DEG(M)	DEG(M)	DEG(I+M)	DEG(M)	--	--	--	--
XLOC_024291	DEG(M)	No	DEG(I+M)	DEG(M)	--	--	--	--
XLOC_024292	DEG(M)	DEG(M)	DEG(I+M)	DEG(M)	--	--	--	--
XLOC_024296	No	No	No	No	ELU44100.1	--	--	K07513
XLOC_024297	No	No	No	No	EUC60876.1	--	--	--
XLOC_024300	No	No	DEG(I+M)	No	--	--	G	K09873
XLOC_024309	DEG(M)	DEG(M)	DEG(I+M)	DEG(M)	XP_008905488.1	--	J	K02974
XLOC_024314	DEG(M)	No	No	No	KEP46754.1	--	--	--
XLOC_024317	DEG(M)	No	No	No	EUC57682.1	--	J	--
XLOC_024323	No	No	No	No	KIH67448.1	--	O	--
XLOC_024328	DEG(M)	No	No	No	--	--	--	--
XLOC_024335	DEG(M)	No	No	No	--	--	--	--
XLOC_024336	DEG(M)	DEG(M)	DEG(I+M)	DEG(M)	--	--	--	--
XLOC_024338	No	No	No	No	XP_003052522.1	--	J	--
XLOC_024344	DEG(M)	No	DEG(I+M)	DEG(M)	EGZ67539.1	--	--	--
XLOC_024345	DEG(M)	DEG(M)	DEG(I+M)	DEG(M)	KIM19341.1	--	--	--

**Differentially expressed gene (DEG) IDs**	***C. microphyllum*** **root and shoot-specific (>2 fold)**	***C. microphyllum*** **root and shoot-specific (>10 fold)**	**ICC4958 and** ***C. microphyllum*** **root-specific (>2 fold)**	***C. microphyllum*** **root-specific (>2 fold)**	**Functional Annotation**			
					**NCBI nr IDs**	**TFs**	**KOG**	**KEGG**
XLOC_024347	No	No	No	No	--	--	--	--
XLOC_024371	DEG(M)	No	No	No	--	--	--	--
XLOC_024388	DEG(M)	No	No	No	--	--	--	--
XLOC_024389	DEG(M)	No	No	No	EUC54477.1	--	--	K02732
XLOC_024390	No	No	No	No	EUC54477.1	--	--	--
XLOC_024391	DEG(M)	No	No	No	EUC54477.1	--	--	--
XLOC_024392	DEG(M)	No	No	No	AAU84933.1	NAC	J	--
XLOC_024393	No	No	No	No	--	--	--	--
XLOC_024398	No	No	No	No	--	--	--	--
XLOC_024403	No	No	No	No	CEL54484.1	--	--	--
XLOC_024412	DEG(M)	DEG(M)	No	No	EXM09033.1	--	--	--
XLOC_024413	No	No	No	No	--	--	--	--
XLOC_024416	No	No	No	No	ELU38904.1	--	--	--
XLOC_024418	No	No	No	No	XP_001948267.2	--	O	--
XLOC_024419	DEG(M)	No	No	No	ETI43869.1	--	CE	--
XLOC_024421	No	No	No	No	--	--	--	--
XLOC_024423	No	No	No	No	--	--	--	--
XLOC_024426	DEG(M)	No	No	No	EGU89062.1	--	--	--
XLOC_024427	No	No	No	No	XP_008901318.1	--	O	--
XLOC_024431	No	No	No	No	--	--	--	--
XLOC_024432	No	No	No	No	KEP54610.1	--	--	--
XLOC_024436	DEG(M)	No	No	No	GAA98206.1	--	JO	K02983
XLOC_024437	No	No	No	No	--	--	--	--
XLOC_024439	DEG(M)	No	No	No	--	--	--	--
XLOC_024441	DEG(M)	No	No	No	--	--	--	--
XLOC_024442	No	No	No	No	ETI32119.1	--	--	--
XLOC_024447	No	No	No	No	--	--	--	--
XLOC_024449	DEG(M)	No	No	No	KEP55564.1	--	--	--
XLOC_024451	DEG(M)	No	No	No	NP_001155610.1	YABBY	J	K02977
XLOC_024453	No	No	No	No	XP_003301555.1	--	O	--
XLOC_024454	No	No	No	No	EWY90468.1	--	E	--
XLOC_024456	DEG(M)	No	No	No	CCO35176.1	--	--	--
XLOC_024457	No	No	No	No	ETP47333.1	--	--	--
XLOC_024458	No	No	No	No	--	--	--	--
XLOC_024459	No	No	No	No	--	--	--	--
XLOC_024461	No	No	No	No	--	--	--	--
XLOC_024465	DEG(M)	No	No	No	--	--	--	--
XLOC_024471	DEG(M)	No	No	No	--	--	--	--
XLOC_024472	DEG(M)	No	No	No	CEL53135.1	--	--	--
XLOC_024475	No	No	No	No	XP_002898848.1	--	O	K08675

Differentially expressed gene (DEG) IDs	*C. microphyllum* root and shoot-specific (>2 fold)	*C. microphyllum* root and shoot-specific (>10 fold)	ICC4958 and *C. microphyllum* root-specific (>2 fold)	*C. microphyllum* root-specific (>2 fold)	Functional Annotation			
					NCBI nr IDs	TFs	KOG	KEGG
XLOC_024477	No	No	No	No	XP_009520308.1	B3	O	K09494
XLOC_024478	DEG(M)	No	No	No	KLO05683.1	--	O	--
XLOC_024482	DEG(M)	No	No	No	--	--	J	--
XLOC_024490	DEG(M)	No	No	No	CEL55422.1	--	--	--
XLOC_024491	DEG(M)	No	No	No	--	--	--	--
XLOC_024492	No	No	No	No	XP_012066344.1	--	--	K09528
XLOC_024493	No	No	No	No	--	--	--	--
XLOC_024496	No	No	No	No	--	--	--	--
XLOC_024497	No	No	No	No	--	--	--	--
XLOC_024498	DEG(M)	DEG(M)	No	No	--	--	--	--
XLOC_024503	DEG(M)	No	No	No	--	--	--	--
XLOC_024509	DEG(M)	No	No	No	--	--	--	--
XLOC_024510	DEG(M)	No	No	No	KDN35077.1	--	--	--
XLOC_024511	DEG(M)	No	DEG(I+M)	DEG(M)	--	--	--	--
XLOC_024512	No	No	No	No	XP_001651255.1	--	J	--
XLOC_024515	DEG(M)	No	No	No	EUC57134.1	--	--	--
XLOC_024519	No	No	No	No	--	--	--	--
XLOC_024520	No	No	No	No	XP_001950406.1	--	O	--
XLOC_024532	DEG(M)	No	No	No	XP_002521891.1	GeBP	E	--
XLOC_024533	No	No	No	No	AKC01511.1	--	--	--
XLOC_024537	No	No	No	No	AIL48319.1	--	J	--
XLOC_024539	No	No	No	No	--	--	--	--
XLOC_024546	DEG(M)	No	No	No	--	--	--	--
XLOC_024549	No	No	No	No	AHG55039.1	--	O	--
XLOC_024552	DEG(M)	No	No	No	EUC58632.1	--	--	--
XLOC_024555	No	No	No	No	--	--	--	K03236
XLOC_024557	DEG(M)	No	No	No	--	--	J	--
XLOC_024567	No	No	No	No	EWG47344.1	--	R	K06185
XLOC_024583	DEG(M)	DEG(M)	DEG(I+M)	DEG(M)	--	--	--	--
XLOC_024584	DEG(M)	DEG(M)	DEG(I+M)	DEG(M)	XP_008915203.1	NF-YB	J	--
XLOC_024586	No	No	No	No	EUC64183.1	NF-YB	J	K02973
XLOC_024618	DEG(M)	DEG(M)	No	No	XP_004505028.1	--	--	--
XLOC_024623	DEG(M)	No	DEG(I+M)	DEG(M)	--	NAC	M	K01051
XLOC_024625	No	No	DEG(I+M)	No	--	--	--	--
XLOC_024626	DEG(M)	No	No	No	EUC67110.1	--	--	--
XLOC_024628	DEG(M)	No	DEG(I+M)	DEG(M)	--	--	--	--
XLOC_024630	DEG(M)	DEG(M)	No	No	--	--	--	--
XLOC_024631	DEG(M)	DEG(M)	DEG(I+M)	DEG(M)	--	--	--	--
XLOC_024633	DEG(M)	No	DEG(I+M)	DEG(M)	--	--	--	--
XLOC_024634	DEG(M)	No	No	No	WP_045788053.1	--	--	--

Differentially expressed gene (DEG) IDs	*C. microphyllum* root and shoot-specific (>2 fold)	*C. microphyllum* root and shoot-specific (>10 fold)	ICC4958 and *C. microphyllum* root-specific (>2 fold)	*C. microphyllum* root-specific (>2 fold)	Functional Annotation			
					NCBI nr IDs	TFs	KOG	KEGG
XLOC_024641	DEG(M)	No	No	No	CEL53407.1	--	--	--
XLOC_024657	DEG(M)	No	No	No	XP_008891295.1	Trihelix	G	K00134
XLOC_024658	DEG(M)	No	DEG(I+M)	DEG(M)	XP_004502429.1	--	--	--
XLOC_024667	DEG(M)	No	No	No	EGU76657.1	--	--	--
XLOC_024668	No	No	No	No	EMT64753.1	--	--	--
XLOC_024669	DEG(M)	No	No	No	EXM09745.1	--	--	--
XLOC_024670	No	No	No	No	EGU73493.1	--	--	--
XLOC_024683	DEG(M)	No	No	No	KDN35515.1	--	CE	--
XLOC_024687	DEG(M)	DEG(M)	No	No	XP_012534721.1	Dof	Z	--
XLOC_024689	DEG(M)	DEG(M)	No	No	KKA73802.1	Dof	Z	--
XLOC_024690	DEG(M)	No	No	No	KKA73802.1	Dof	Z	--
XLOC_024691	DEG(M)	DEG(M)	DEG(I+M)	DEG(M)	ACV13203.1	Dof	Z	--
XLOC_024692	DEG(M)	DEG(M)	DEG(I+M)	DEG(M)	AAI13037.1	--	Z	--
XLOC_024693	No	No	No	No	XP_004335590.1	Dof	Z	--
XLOC_024694	DEG(M)	No	DEG(I+M)	DEG(M)	XP_004336648.1	--	Z	--
XLOC_024695	DEG(M)	No	No	No	XP_004335590.1	Dof	Z	--
XLOC_024697	DEG(M)	No	No	No	XP_004335590.1	Dof	Z	--
XLOC_024698	DEG(M)	No	DEG(I+M)	DEG(M)	XP_004335590.1	Dof	Z	--
XLOC_024701	DEG(M)	DEG(M)	DEG(I+M)	DEG(M)	AAP34633.1	Dof	Z	--
XLOC_024702	DEG(M)	DEG(M)	DEG(I+M)	DEG(M)	AAW70169.1	--	Z	--
XLOC_024703	DEG(M)	No	No	No	XP_002408110.1	--	Z	--
XLOC_024704	DEG(M)	DEG(M)	No	No	EJW72694.1	--	Z	--
XLOC_024722	No	No	DEG(I+M)	No	XP_004489961.1	--	--	--
XLOC_024723	DEG(M)	DEG(M)	No	No	--	--	--	--
XLOC_024727	No	No	No	No	XP_004512576.2	--	--	--
XLOC_024739	DEG(M)	No	No	No	--	--	--	--
XLOC_024740	DEG(M)	No	No	No	--	--	--	--
XLOC_024748	No	No	No	No	XP_004510857.1	HD-ZIP	K	--
XLOC_024749	No	No	No	No	XP_004492677.1	HD-ZIP	K	K09338
XLOC_024757	DEG(M)	DEG(M)	No	No	--	--	--	--
XLOC_024787	No	No	No	No	--	--	--	--
XLOC_024791	No	No	DEG(I+M)	No	--	--	--	--
XLOC_024792	DEG(M)	DEG(M)	No	No	XP_001943172.1	--	O	K04079
XLOC_024793	No	No	No	No	XP_001649752.1	--	O	--
XLOC_024795	DEG(M)	DEG(M)	DEG(I+M)	DEG(M)	XP_003047618.1	--	O	--
XLOC_024797	DEG(M)	DEG(M)	DEG(I+M)	DEG(M)	EXM33467.1	--	O	--
XLOC_024798	No	No	No	No	XP_007698721.1	--	O	--
XLOC_024799	DEG(M)	No	No	No	XP_007289409.1	--	O	--
XLOC_024800	No	No	No	No	XP_001649752.1	--	O	--
XLOC_024802	DEG(M)	DEG(M)	DEG(I+M)	DEG(M)	ETK95017.1	--	O	--

Differentially expressed gene (DEG) IDs	*C. microphyllum* root and shoot-specific (>2 fold)	*C. microphyllum* root and shoot-specific (>10 fold)	ICC4958 and *C. microphyllum* root-specific (>2 fold)	*C. microphyllum* root-specific (>2 fold)	Functional Annotation			
					NCBI nr IDs	TFs	KOG	KEGG
XLOC_024803	DEG(M)	No	No	No	XP_009534751.1	--	O	--
XLOC_024804	No	No	No	No	--	--	--	--
XLOC_024805	DEG(M)	No	DEG(I+M)	DEG(M)	--	--	--	--
XLOC_024830	No	No	No	No	KEP47904.1	--	--	--
XLOC_024831	No	No	No	No	XP_011493760.1	--	--	--
XLOC_024833	No	No	No	No	--	--	--	--
XLOC_024837	DEG(M)	No	No	No	EUC59019.1	--	--	--
XLOC_024840	No	No	No	No	--	--	--	--
XLOC_024846	No	No	No	No	--	--	--	--
XLOC_024847	No	No	No	No	XP_001949483.1	--	T	--
XLOC_024848	No	No	No	No	EWG49661.1	--	--	K15029
XLOC_024849	No	No	No	No	--	--	--	--
XLOC_024850	No	No	No	No	XP_001949530.3	--	C	--
XLOC_024851	No	No	No	No	KFQ02889.1	--	C	--
XLOC_024857	No	No	DEG(I+M)	No	--	--	--	K01183
XLOC_024858	DEG(M)	No	No	No	--	--	--	--
XLOC_024861	No	No	No	No	--	--	--	--
XLOC_024866	DEG(M)	No	No	No	--	--	--	--
XLOC_024867	No	No	No	No	--	--	--	--
XLOC_024869	No	No	No	No	--	--	--	--
XLOC_024873	No	No	No	No	--	--	U	--
XLOC_024874	No	No	No	No	XP_003044779.1	--	--	--
XLOC_024877	No	No	No	No	--	--	--	--
XLOC_024878	No	No	No	No	--	--	--	--
XLOC_024879	No	No	No	No	XP_008906269.1	--	--	--
XLOC_024883	No	No	No	No	--	--	--	--
XLOC_024885	No	No	No	No	ETK87052.1	--	--	K04646
XLOC_024889	No	No	No	No	--	--	--	--
XLOC_024894	No	No	No	No	CCO26619.1	--	--	--
XLOC_024900	No	No	No	No	CEL56886.1	--	--	--
XLOC_024901	No	No	No	No	--	--	--	--
XLOC_024914	No	No	No	No	--	--	--	--
XLOC_024916	No	No	No	No	--	--	--	--
XLOC_024923	No	No	No	No	XP_008914578.1	--	O	--
XLOC_024928	DEG(M)	No	No	No	ELU44847.1	--	--	--
XLOC_024930	DEG(M)	No	No	No	CEL51787.1	--	C	--
XLOC_024931	No	No	No	No	KEP48842.1	--	--	K00898
XLOC_024932	No	No	No	No	KEP48842.1	--	--	--
XLOC_024938	DEG(M)	No	No	No	--	--	--	--
XLOC_024940	No	No	No	No	--	--	--	--

**Differentially expressed gene (DEG) IDs**	***C. microphyllum* root and shoot-specific (>2 fold)**	***C. microphyllum* root and shoot-specific (>10 fold)**	**ICC4958 and *C. microphyllum* root-specific (>2 fold)**	***C. microphyllum* root-specific (>2 fold)**	**Functional Annotation**			
					**NCBI nr IDs**	**TFs**	**KOG**	**KEGG**
XLOC_024943	No	No	No	No	CCO29212.1	--	--	--
XLOC_024944	No	No	No	No	CEL57317.1	--	--	--
XLOC_024945	No	No	No	No	CEL57317.1	--	--	--
XLOC_024947	DEG(M)	No	No	No	--	--	J	--
XLOC_024954	No	No	No	No	XP_004490373.1	--	--	--
XLOC_024955	DEG(M)	No	No	No	--	--	--	--
XLOC_024958	DEG(M)	No	No	No	EUC58778.1	--	--	--
XLOC_024961	No	No	No	No	--	--	--	--
XLOC_024972	No	No	No	No	--	--	--	--
XLOC_024973	DEG(M)	No	No	No	--	--	--	--
XLOC_024974	DEG(M)	No	No	No	--	--	--	--
XLOC_024977	No	No	No	No	--	--	--	--
XLOC_024979	No	No	No	No	--	--	--	--
XLOC_024989	No	No	No	No	XP_003245594.2	--	C	--
XLOC_024990	No	No	No	No	--	--	--	K16860
XLOC_024994	No	No	No	No	--	--	--	--
XLOC_025004	No	No	No	No	--	--	--	K06620
XLOC_025007	No	No	No	No	AAL37254.1	--	O	--
XLOC_025009	No	No	No	No	CCO28549.1	--	C	--
XLOC_025015	No	No	No	No	XP_008914381.1	--	--	--
XLOC_025016	No	No	No	No	EGU72095.1	--	--	--
XLOC_025018	No	No	DEG(I+M)	No	XP_004492635.1	--	--	K14423
XLOC_025019	No	No	No	No	--	--	--	--
XLOC_025031	DEG(M)	No	No	No	--	--	--	--
XLOC_025032	No	No	No	No	--	--	H	--
XLOC_025033	DEG(M)	No	No	No	--	--	--	--
XLOC_025034	No	No	No	No	XP_001947176.2	--	I	--
XLOC_025035	No	No	No	No	BAM99026.1	FAR1	O	--
XLOC_025037	No	No	No	No	--	--	--	--
XLOC_025040	No	No	No	No	EUC66715.1	--	C	--
XLOC_025041	DEG(M)	No	DEG(I+M)	DEG(M)	XP_004487809.1	--	--	--
XLOC_025042	No	No	No	No	XP_003305742.1	--	--	--
XLOC_025046	DEG(M)	No	No	No	EUC66587.1	--	--	--
XLOC_025049	DEG(M)	No	No	No	KDN44809.1	--	--	K01507
XLOC_025054	No	No	No	No	--	--	--	--
XLOC_025055	No	No	No	No	--	--	--	--
XLOC_025056	No	No	No	No	--	--	--	--
XLOC_025057	No	No	No	No	--	--	--	--
XLOC_025063	No	No	No	No	XP_003244411.2	--	E	--
XLOC_025065	No	No	No	No	--	--	--	--

Differentially expressed gene (DEG) IDs	*C. microphyllum* root and shoot-specific (>2 fold)	*C. microphyllum* root and shoot-specific (>10 fold)	ICC4958 and *C. microphyllum* root-specific (>2 fold)	*C. microphyllum* root-specific (>2 fold)	Functional Annotation			
					NCBI nr IDs	TFs	KOG	KEGG
XLOC_025066	No	No	No	No	EUC63945.1	--	--	--
XLOC_025067	No	No	No	No	--	--	--	--
XLOC_025072	DEG(M)	No	No	No	--	--	--	--
XLOC_025074	DEG(M)	No	DEG(I+M)	DEG(M)	KEP54685.1	--	--	--
XLOC_025078	DEG(M)	DEG(M)	DEG(I+M)	DEG(M)	KDN49425.1	--	--	--
XLOC_025080	DEG(M)	DEG(M)	DEG(I+M)	DEG(M)	--	--	--	--
XLOC_025085	No	No	No	No	--	--	--	--
XLOC_025088	No	No	No	No	EXA29802.1	--	--	--
XLOC_025091	DEG(M)	No	No	No	--	--	--	--
XLOC_025095	DEG(M)	No	No	No	--	--	--	--
XLOC_025097	No	No	No	No	CCO30617.1	--	E	--
XLOC_025101	No	No	No	No	--	--	--	--
XLOC_025102	No	No	No	No	--	--	--	--
XLOC_025103	No	No	No	No	--	--	--	--
XLOC_025107	No	No	No	No	XP_003048836.1	--	--	--
XLOC_025110	DEG(M)	No	No	No	CEL60300.1	--	C	--
XLOC_025115	DEG(M)	No	No	No	--	--	--	--
XLOC_025116	No	No	No	No	KEP50396.1	--	--	--
XLOC_025119	No	No	No	No	--	--	--	--
XLOC_025120	DEG(M)	DEG(M)	No	No	--	--	--	--
XLOC_025121	DEG(M)	No	No	No	--	--	--	--
XLOC_025122	No	No	No	No	--	--	--	--
XLOC_025123	No	No	No	No	KNA07005.1	--	TDK	--
XLOC_025124	No	No	No	No	--	--	--	--
XLOC_025125	No	No	No	No	AJQ30112.1	--	C	--
XLOC_025126	No	No	No	No	XP_003048210.1	--	--	--
XLOC_025131	No	No	No	No	AEO44494.1	--	J	K01868
XLOC_025135	No	No	No	No	XP_008917043.1	--	--	--
XLOC_025139	DEG(M)	DEG(M)	No	No	XP_004512353.1	--	--	--
XLOC_025140	No	No	No	No	--	--	--	--
XLOC_025146	No	No	No	No	--	--	--	--
XLOC_025148	No	No	No	No	--	TCP	--	--
XLOC_025149	No	No	No	No	--	--	--	--
XLOC_025150	No	No	No	No	XP_008899296.1	--	--	K01507
XLOC_025154	DEG(M)	No	No	No	--	--	J	--
XLOC_025156	No	No	No	No	--	--	--	--
XLOC_025157	No	No	No	No	--	--	--	--
XLOC_025158	No	No	No	No	--	--	--	--
XLOC_025160	No	No	No	No	--	--	--	--
XLOC_025165	DEG(M)	No	No	No	--	--	--	--

Differentially expressed gene (DEG) IDs	*C. microphyllum* root and shoot-specific (>2 fold)	*C. microphyllum* root and shoot-specific (>10 fold)	ICC4958 and *C. microphyllum* root-specific (>2 fold)	*C. microphyllum* root-specific (>2 fold)	Functional Annotation			
					NCBI nr IDs	TFs	KOG	KEGG
XLOC_025179	No	No	No	No	--	--	--	K11279
XLOC_025186	No	No	No	No	CEL56406.1	--	--	--
XLOC_025190	No	No	No	No	ETL49525.1	--	--	K00615
XLOC_025191	No	No	No	No	EUC64220.1	--	--	--
XLOC_025192	No	No	No	No	XP_001648417.1	--	--	--
XLOC_025193	No	No	No	No	--	--	--	--
XLOC_025201	No	No	No	No	EUC54533.1	--	--	--
XLOC_025202	DEG(M)	No	No	No	EUC54533.1	--	--	--
XLOC_025206	No	No	No	No	--	--	--	--
XLOC_025208	No	No	DEG(I+M)	No	--	AP2	--	--
XLOC_025211	No	No	No	No	EUC56031.1	--	--	--
XLOC_025212	DEG(M)	No	No	No	--	--	--	--
XLOC_025214	DEG(M)	DEG(M)	No	No	KDN38602.1	--	--	--
XLOC_025216	No	No	DEG(I+M)	No	--	--	--	--
XLOC_025218	No	No	No	No	XP_009521651.1	--	O	--
XLOC_025223	No	No	No	No	--	--	--	--
XLOC_025225	No	No	No	No	--	--	--	K00856
XLOC_025226	DEG(M)	No	No	No	--	--	--	--
XLOC_025230	No	No	No	No	XP_012149651.1	--	P	--
XLOC_025231	No	No	No	No	KFM58607.1	--	P	--
XLOC_025240	DEG(M)	No	DEG(I+M)	DEG(M)	--	--	--	--
XLOC_025241	No	No	No	No	--	--	--	--
XLOC_025243	No	No	No	No	--	--	--	--
XLOC_025244	No	No	No	No	--	--	--	K09272
XLOC_025248	DEG(M)	No	No	No	CCO34569.1	--	--	--
XLOC_025249	DEG(M)	No	No	No	--	--	--	--
XLOC_025252	No	No	No	No	CEL51972.1	--	--	--
XLOC_025253	No	No	No	No	--	--	--	--
XLOC_025254	No	No	No	No	--	--	--	--
XLOC_025255	No	No	No	No	--	--	--	--
XLOC_025261	DEG(M)	No	No	No	--	--	J	--
XLOC_025262	DEG(M)	No	No	No	EUC61423.1	--	A	--
XLOC_025264	No	No	No	No	--	--	--	--
XLOC_025265	DEG(M)	No	No	No	CCO31112.1	--	--	--
XLOC_025267	DEG(M)	No	DEG(I+M)	DEG(M)	XP_004508639.1	--	--	--
XLOC_025269	No	No	DEG(I+M)	No	XP_004497326.1	--	--	K01859
XLOC_025270	No	No	No	No	XP_004509160.1	--	I	--
XLOC_025272	No	No	No	No	EUC65028.1	--	--	--
XLOC_025274	No	No	No	No	--	--	--	--
XLOC_025275	DEG(M)	No	No	No	--	--	--	--

Differentially expressed gene (DEG) IDs	*C. microphyllum* root and shoot-specific (>2 fold)	*C. microphyllum* root and shoot-specific (>10 fold)	ICC4958 and *C. microphyllum* root-specific (>2 fold)	*C. microphyllum* root-specific (>2 fold)	Functional Annotation			
					NCBI nr IDs	TFs	KOG	KEGG
XLOC_025279	No	No	No	No	EFX77060.1	--	O	--
XLOC_025280	No	No	No	No	XP_008911965.1	--	--	--
XLOC_025285	No	No	No	No	--	--	J	--
XLOC_025292	No	No	No	No	XP_002963234.1	--	J	--
XLOC_025293	No	No	No	No	XP_004345840.1	--	J	--
XLOC_025298	No	No	No	No	EGU78736.1	--	--	--
XLOC_025302	No	No	No	No	CEL59317.1	--	--	--
XLOC_025305	No	No	No	No	--	--	--	--
XLOC_025307	DEG(M)	DEG(M)	DEG(I+M)	DEG(M)	--	--	--	--
XLOC_025311	DEG(M)	No	No	No	XP_008897240.1	--	--	--
XLOC_025312	No	No	No	No	--	--	--	--
XLOC_025320	No	No	No	No	--	--	--	--
XLOC_025322	DEG(M)	No	No	No	--	--	--	--
XLOC_025323	No	No	No	No	XP_003247118.1	--	--	--
XLOC_025324	No	No	No	No	--	--	--	--
XLOC_025327	DEG(M)	No	No	No	--	--	--	--
XLOC_025328	DEG(M)	DEG(M)	No	No	--	--	--	--
XLOC_025329	No	No	No	No	--	--	--	--
XLOC_025332	No	No	No	No	XP_012089066.1	--	V	K07870
XLOC_025334	No	No	No	No	--	--	--	--
XLOC_025335	No	No	No	No	XP_001948281.1	--	--	K01106
XLOC_025338	No	No	DEG(I+M)	No	XP_004493869.2	ERF	--	--
XLOC_025340	No	No	DEG(I+M)	No	--	--	R	--
XLOC_025341	No	No	No	No	--	--	--	--
XLOC_025342	DEG(M)	No	No	No	XP_003042654.1	--	--	--
XLOC_025343	DEG(M)	DEG(M)	DEG(I+M)	DEG(M)	--	--	--	--
XLOC_025344	DEG(M)	No	No	No	XP_003042432.1	--	--	--
XLOC_025348	No	No	No	No	CCO32198.1	--	--	--
XLOC_025351	No	No	No	No	--	--	--	--
XLOC_025352	No	No	No	No	EUC57052.1	--	--	--
XLOC_025354	No	No	No	No	--	--	--	--
XLOC_025355	No	No	No	No	--	--	--	--
XLOC_025356	No	No	No	No	XP_004498605.1	--	--	K02516
XLOC_025357	DEG(M)	No	No	No	KEP47308.1	--	--	--
XLOC_025362	No	No	No	No	--	--	--	--
XLOC_025366	No	No	No	No	--	--	--	--
XLOC_025368	No	No	No	No	AHX56895.1	--	--	--
XLOC_025369	No	No	No	No	--	--	--	K03527
XLOC_025380	No	No	No	No	XP_002525362.1	--	I	--
XLOC_025385	No	No	No	No	--	--	J	K02951

**Differentially expressed gene (DEG) IDs**	***C. microphyllum* root and shoot-specific (>2 fold)**	***C. microphyllum* root and shoot-specific (>10 fold)**	**ICC4958 and *C. microphyllum* root-specific (>2 fold)**	***C. microphyllum* root-specific (>2 fold)**	**Functional Annotation**			
					**NCBI nr IDs**	**TFs**	**KOG**	**KEGG**
XLOC_025386	No	No	No	No	--	--	--	--
XLOC_025389	No	No	No	No	--	--	--	--
XLOC_025391	DEG(M)	No	No	No	--	--	--	--
XLOC_025393	No	No	No	No	KDN45495.1	--	--	--
XLOC_025396	No	No	No	No	BAJ87032.1	--	--	--
XLOC_025401	No	No	No	No	--	--	--	--
XLOC_025405	No	No	No	No	XP_004508270.1	--	--	K01724
XLOC_025415	No	No	No	No	XP_002521851.1	--	--	--
XLOC_025416	No	No	No	No	--	--	--	--
XLOC_025417	DEG(M)	No	No	No	--	--	--	--
XLOC_025418	DEG(M)	No	No	No	XP_002521866.1	--	--	--
XLOC_025419	DEG(M)	No	No	No	ETI36036.1	--	--	--
XLOC_025420	DEG(M)	No	No	No	XP_009521416.1	--	J	K02937
XLOC_025421	DEG(M)	No	No	No	XP_008896295.1	--	J	K02937
XLOC_025422	No	No	No	No	--	--	--	--
XLOC_025435	DEG(M)	No	No	No	KDN48280.1	--	--	--
XLOC_025437	DEG(M)	No	No	No	KFM75314.1	--	O	K03094
XLOC_025439	DEG(M)	No	No	No	--	--	--	--
XLOC_025441	No	No	No	No	--	--	--	--
XLOC_025443	DEG(M)	No	No	No	--	--	--	--
XLOC_025449	No	No	DEG(I+M)	No	--	--	--	--
XLOC_025450	No	No	DEG(I+M)	No	--	--	--	--
XLOC_025452	No	No	DEG(I+M)	No	--	--	--	--
XLOC_025456	No	No	No	No	--	--	--	--
XLOC_025457	No	No	No	No	--	--	--	--
XLOC_025461	No	No	DEG(I+M)	No	XP_004485877.1	--	EH	K01568
XLOC_025464	No	No	No	No	XP_004515794.1	ERF	K	K09286
XLOC_025467	DEG(M)	No	No	No	--	--	--	--
XLOC_025468	No	No	No	No	--	--	--	--
XLOC_025469	No	No	No	No	--	--	--	--
XLOC_025474	DEG(M)	DEG(M)	No	No	--	--	--	--
XLOC_025484	No	No	No	No	CEL56423.1	--	--	--
XLOC_025485	No	No	No	No	CEL56423.1	--	--	--
XLOC_025488	No	No	No	No	--	--	--	--
XLOC_025489	No	No	No	No	EUC61657.1	--	--	--
XLOC_025490	DEG(M)	No	No	No	--	--	--	--
XLOC_025496	DEG(M)	No	No	No	--	--	--	--
XLOC_025497	DEG(M)	No	No	No	KDN35346.1	--	--	--
XLOC_025508	No	No	No	No	--	--	--	--
XLOC_025511	DEG(M)	No	No	No	--	--	--	--

Differentially expressed gene (DEG) IDs	*C. microphyllum* root and shoot-specific (>2 fold)	*C. microphyllum* root and shoot-specific (>10 fold)	ICC4958 and *C. microphyllum* root-specific (>2 fold)	*C. microphyllum* root-specific (>2 fold)	Functional Annotation			
					NCBI nr IDs	TFs	KOG	KEGG
XLOC_025517	DEG(M)	No	DEG(I+M)	DEG(M)	--	--	--	--
XLOC_025518	DEG(M)	No	No	No	XP_004491082.1	--	--	--
XLOC_025520	DEG(M)	No	No	No	--	--	--	--
XLOC_025522	No	No	No	No	KDN44831.1	--	--	--
XLOC_025524	DEG(M)	No	DEG(I+M)	DEG(M)	--	--	--	--
XLOC_025527	No	No	No	No	KDN35057.1	--	C	K00413
XLOC_025533	No	No	No	No	--	ERF	--	--
XLOC_025539	No	No	No	No	--	--	--	K07936
XLOC_025541	No	No	No	No	XP_001655016.1	--	J	K02941
XLOC_025542	DEG(M)	No	DEG(I+M)	DEG(M)	XP_003051714.1	--	J	--
XLOC_025544	No	No	No	No	--	--	--	--
XLOC_025546	No	No	No	No	--	--	--	--
XLOC_025551	No	No	DEG(I+M)	No	--	--	--	--
XLOC_025552	No	No	No	No	CCI47858.1	--	A	--
XLOC_025556	DEG(M)	No	No	No	ADV40160.1	--	J	K02941
XLOC_025558	No	No	No	No	--	--	R	K03327
XLOC_025559	No	No	No	No	--	--	--	--
XLOC_025560	No	No	No	No	--	--	--	--
XLOC_025562	No	No	No	No	--	--	--	--
XLOC_025566	DEG(M)	DEG(M)	DEG(I+M)	DEG(M)	XP_005850414.1	YABBY	J	--
XLOC_025569	No	No	No	No	--	--	--	K14332
XLOC_025573	DEG(M)	No	No	No	--	--	--	--
XLOC_025580	DEG(M)	No	No	No	--	--	--	--
XLOC_025581	No	No	No	No	--	--	--	--
XLOC_025582	No	No	No	No	--	--	R	K11294
XLOC_025584	No	No	DEG(I+M)	No	XP_004516058.1	--	--	--
XLOC_025588	DEG(M)	No	No	No	--	--	--	--
XLOC_025590	No	No	No	No	--	--	--	--
XLOC_025607	No	No	No	No	CCO33617.1	--	--	--
XLOC_025608	No	No	No	No	KDN49473.1	--	--	--
XLOC_025610	No	No	No	No	--	--	--	--
XLOC_025613	No	No	No	No	XP_003049769.1	--	--	--
XLOC_025614	DEG(M)	No	No	No	CEL62931.1	--	CE	--
XLOC_025615	No	No	No	No	--	--	--	--
XLOC_025617	No	No	No	No	CEL59719.1	--	--	--
XLOC_025618	No	No	No	No	--	--	--	K18081
XLOC_025619	No	No	No	No	--	--	--	--
XLOC_025622	No	No	No	No	--	--	--	--
XLOC_025625	No	No	No	No	EUC65467.1	--	--	--
XLOC_025628	DEG(M)	No	No	No	--	--	--	--

Differentially expressed gene (DEG) IDs	***C. microphyllum* root and shoot-specific (>2 fold)**	***C. microphyllum* root and shoot-specific (>10 fold)**	**ICC4958 and *C. microphyllum* root-specific (>2 fold)**	***C. microphyllum* root-specific (>2 fold)**	**Functional Annotation**			
					**NCBI nr IDs**	**TFs**	**KOG**	**KEGG**
XLOC_025630	No	No	No	No	CEL59003.1	--	--	--
XLOC_025635	DEG(M)	No	No	No	--	--	--	--
XLOC_025636	DEG(M)	DEG(M)	DEG(I+M)	DEG(M)	--	--	--	--
XLOC_025642	No	No	No	No	XP_001658440.1	--	Z	--
XLOC_025644	No	No	No	No	--	--	--	--
XLOC_025647	No	No	No	No	--	--	--	--
XLOC_025648	No	No	No	No	--	--	--	--
XLOC_025652	DEG(M)	No	No	No	KDN33249.1	--	--	--
XLOC_025653	No	No	No	No	--	--	--	--
XLOC_025654	No	No	No	No	--	--	--	--
XLOC_025655	No	No	No	No	--	--	--	--
XLOC_025664	No	No	No	No	ETI32119.1	--	--	--
XLOC_025665	No	No	No	No	--	--	--	--
XLOC_025670	No	No	No	No	XP_003054354.1	--	--	--
XLOC_025672	No	No	DEG(I+M)	No	--	--	--	--
XLOC_025677	DEG(M)	No	No	No	--	--	J	--
XLOC_025678	No	No	No	No	CCO26568.1	--	--	--
XLOC_025679	No	No	No	No	--	--	--	--
XLOC_025682	No	No	No	No	KEP51917.1	--	--	--
XLOC_025685	DEG(M)	No	No	No	--	--	--	--
XLOC_025687	No	No	No	No	AJQ30114.1	--	G	--
XLOC_025691	No	No	No	No	XP_008890951.1	--	--	--
XLOC_025692	No	No	No	No	XP_008890951.1	--	--	--
XLOC_025693	No	No	No	No	AAP48704.1	--	U	--
XLOC_025694	No	No	No	No	ETL80358.1	--	--	--
XLOC_025705	DEG(M)	DEG(M)	DEG(I+M)	DEG(M)	--	--	--	--
XLOC_025706	DEG(M)	DEG(M)	DEG(I+M)	DEG(M)	--	--	--	--
XLOC_025707	DEG(M)	DEG(M)	No	No	KDN46590.1	--	--	--
XLOC_025711	No	No	No	No	EUC64821.1	--	--	--
XLOC_025712	DEG(M)	No	No	No	EUC64821.1	--	--	--
XLOC_025716	No	No	No	No	--	--	--	--
XLOC_025720	No	No	No	No	--	--	--	--
XLOC_025721	No	No	No	No	EUC65335.1	--	--	--
XLOC_025729	No	No	No	No	--	--	X	K13508
XLOC_025734	DEG(M)	No	No	No	ETM43550.1	--	C	--
XLOC_025735	No	No	No	No	ETK83664.1	--	--	--
XLOC_025745	DEG(M)	No	No	No	--	--	--	--
XLOC_025747	No	No	No	No	CEL55095.1	--	--	--
XLOC_025748	DEG(M)	DEG(M)	DEG(I+M)	DEG(M)	CEL57163.1	--	--	--
XLOC_025758	No	No	No	No	--	--	--	--

Differentially expressed gene (DEG) IDs	*C. microphyllum* root and shoot-specific (>2 fold)	*C. microphyllum* root and shoot-specific (>10 fold)	ICC4958 and *C. microphyllum* root-specific (>2 fold)	*C. microphyllum* root-specific (>2 fold)	Functional Annotation			
					NCBI nr IDs	TFs	KOG	KEGG
XLOC_025761	No	No	No	No	CCO30562.1	--	--	--
XLOC_025765	DEG(M)	DEG(M)	DEG(I+M)	DEG(M)	EGT55352.1	--	Z	--
XLOC_025774	DEG(M)	No	No	No	--	--	--	--
XLOC_025775	No	No	No	No	--	--	--	--
XLOC_025783	No	No	No	No	--	--	--	--
XLOC_025786	DEG(M)	No	DEG(I+M)	DEG(M)	XP_012574303.1	--	--	--
XLOC_025787	No	No	DEG(I+M)	No	--	--	--	--
XLOC_025798	No	No	No	No	ETI47380.1	--	--	K00654
XLOC_025800	No	No	No	No	--	--	--	--
XLOC_025802	DEG(M)	No	DEG(I+M)	DEG(M)	--	--	--	--
XLOC_025806	No	No	No	No	XP_003054322.1	--	--	--
XLOC_025808	No	No	No	No	--	--	--	--
XLOC_025809	No	No	No	No	--	--	--	--
XLOC_025810	DEG(M)	No	No	No	--	--	--	--
XLOC_025811	DEG(M)	No	No	No	XP_001659807.1	--	--	--
XLOC_025817	No	No	No	No	CCO28662.1	WRKY	J	K11131
XLOC_025822	No	No	No	No	--	--	--	--
XLOC_025823	No	No	No	No	EUC65329.1	--	--	--
XLOC_025824	No	No	No	No	CCO30451.1	--	--	--
XLOC_025828	No	No	No	No	--	--	--	--
XLOC_025829	No	No	No	No	--	--	--	--
XLOC_025831	No	No	No	No	CEL63041.1	--	--	--
XLOC_025832	No	No	No	No	--	--	--	--
XLOC_025835	No	No	No	No	XP_001951884.1	--	--	--
XLOC_025841	DEG(M)	No	DEG(I+M)	DEG(M)	EUC54098.1	--	R	--
XLOC_025842	No	No	No	No	ETP03408.1	--	--	--
XLOC_025844	No	No	No	No	KEP51441.1	--	--	--
XLOC_025846	No	No	No	No	KDN42645.1	--	--	--
XLOC_025847	No	No	No	No	--	--	--	--
XLOC_025848	No	No	No	No	--	--	--	--
XLOC_025850	No	No	No	No	--	--	--	--
XLOC_025851	No	No	No	No	--	--	--	--
XLOC_025855	No	No	No	No	--	--	--	--
XLOC_025856	No	No	No	No	XP_003299142.1	--	--	--
XLOC_025858	No	No	No	No	XP_002901676.1	NF-YB	K	K08065
XLOC_025859	No	No	No	No	XP_003042016.1	--	--	--
XLOC_025860	DEG(M)	No	No	No	--	--	--	--
XLOC_025861	DEG(M)	No	No	No	CEL59886.1	--	--	--
XLOC_025862	No	No	No	No	--	--	--	--
XLOC_025863	No	No	No	No	--	--	J	--

Differentially expressed gene (DEG) IDs	*C. microphyllum* root and shoot-specific (>2 fold)	*C. microphyllum* root and shoot-specific (>10 fold)	ICC4958 and *C. microphyllum* root-specific (>2 fold)	*C. microphyllum* root-specific (>2 fold)	Functional Annotation			
					NCBI nr IDs	TFs	KOG	KEGG
XLOC_025866	No	No	No	No	--	--	--	--
XLOC_025869	No	No	No	No	--	--	--	--
XLOC_025873	No	No	DEG(I+M)	No	XP_004501510.1	--	--	--
XLOC_025876	No	No	No	No	CEL51752.1	--	--	--
XLOC_025877	DEG(M)	No	No	No	XP_003043887.1	--	--	--
XLOC_025878	DEG(M)	No	No	No	P26182.1	--	Z	--
XLOC_025879	DEG(M)	No	No	No	EWZ39305.1	--	--	--
XLOC_025881	No	No	No	No	--	--	--	--
XLOC_025885	No	No	No	No	XP_004494233.1	--	--	--
XLOC_025886	No	No	No	No	--	--	--	--
XLOC_025888	DEG(M)	DEG(M)	DEG(I+M)	DEG(M)	--	--	--	--
XLOC_025889	No	No	No	No	XP_001946991.1	--	C	K03522
XLOC_025891	No	No	No	No	EUC57846.1	--	--	K17871
XLOC_025896	DEG(M)	No	No	No	EUC62994.1	--	--	--
XLOC_025899	No	No	No	No	--	--	--	--
XLOC_025904	No	No	No	No	--	--	--	K07023
XLOC_025905	No	No	No	No	--	--	--	--
XLOC_025907	DEG(M)	No	No	No	XP_002526445.1	--	I	K01613
XLOC_025909	No	No	No	No	--	--	--	--
XLOC_025911	No	No	No	No	XP_002311128.1	--	S	K18624
XLOC_025915	No	No	No	No	XP_001947071.2	--	--	--
XLOC_025923	No	No	No	No	--	--	--	--
XLOC_025926	DEG(M)	DEG(M)	DEG(I+M)	DEG(M)	--	--	--	--
XLOC_025927	No	No	No	No	--	--	--	--
XLOC_025928	No	No	No	No	--	--	--	--
XLOC_025932	No	No	DEG(I+M)	No	--	--	X	--
XLOC_025937	No	No	No	No	XP_003050868.1	--	J	--
XLOC_025938	DEG(M)	No	DEG(I+M)	DEG(M)	--	--	O	--
XLOC_025939	No	No	No	No	--	--	--	--
XLOC_025941	No	No	No	No	--	--	--	K14648
XLOC_025942	No	No	DEG(I+M)	No	XP_004499949.1	--	--	--
XLOC_025945	DEG(M)	No	DEG(I+M)	DEG(M)	--	--	--	--
XLOC_025948	No	No	No	No	XP_001634219.1	--	U	K07937
XLOC_025951	DEG(M)	No	DEG(I+M)	DEG(M)	EUC60768.1	--	--	--
XLOC_025957	No	No	No	No	--	--	--	--
XLOC_025958	No	No	No	No	XP_003054630.1	--	--	--
XLOC_025959	No	No	No	No	XP_003054630.1	--	--	--
XLOC_025960	DEG(M)	DEG(M)	No	No	KNG48404.1	--	--	--
XLOC_025964	No	No	No	No	--	--	U	K07889
XLOC_025966	No	No	No	No	--	--	--	--

Differentially expressed gene (DEG) IDs	*C. microphyllum* root and shoot-specific (>2 fold)	*C. microphyllum* root and shoot-specific (>10 fold)	ICC4958 and *C. microphyllum* root-specific (>2 fold)	*C. microphyllum* root-specific (>2 fold)	Functional Annotation			
					NCBI nr IDs	TFs	KOG	KEGG
XLOC_025970	DEG(M)	No	No	No	--	--	--	K03263
XLOC_025971	DEG(M)	No	No	No	--	--	--	--
XLOC_025973	No	No	No	No	--	WRKY	--	--
XLOC_025976	DEG(M)	No	No	No	ELU41481.1	--	--	K01736
XLOC_025981	No	No	No	No	--	--	--	--
XLOC_025984	No	No	No	No	EUC60484.1	--	--	--
XLOC_025985	No	No	No	No	EUC60484.1	--	--	--
XLOC_025992	No	No	No	No	--	--	--	--
XLOC_025993	DEG(M)	No	No	No	KDN47899.1	--	--	--
XLOC_025994	No	No	No	No	EUC62384.1	--	--	--
XLOC_025995	DEG(M)	No	No	No	XP_008872507.1	--	--	--
XLOC_025996	No	No	No	No	--	--	--	--
XLOC_025997	No	No	No	No	--	--	--	--
XLOC_025999	No	No	No	No	XP_004349662.1	--	J	K02872
XLOC_026000	DEG(M)	No	No	No	--	--	--	--
XLOC_026001	DEG(M)	No	No	No	KEP50294.1	--	--	--
XLOC_026003	No	No	No	No	--	--	--	--
XLOC_026005	DEG(M)	No	No	No	--	--	--	--
XLOC_026006	No	No	No	No	XP_008891746.1	--	C	K00235
XLOC_026008	No	No	No	No	--	--	--	--
XLOC_026009	No	No	No	No	--	--	--	--
XLOC_026010	DEG(M)	DEG(M)	DEG(I+M)	DEG(M)	--	--	--	--
XLOC_026013	No	No	No	No	XP_002510544.1	--	--	--
XLOC_026017	No	No	No	No	XP_008180506.1	--	--	K06694
XLOC_026019	DEG(M)	No	DEG(I+M)	DEG(M)	--	--	O	--
XLOC_026024	No	No	No	No	KEP52037.1	--	--	--
XLOC_026027	No	No	No	No	--	--	--	--
XLOC_026030	No	No	No	No	--	--	--	--
XLOC_026031	No	No	No	No	XP_003052768.1	--	--	--
XLOC_026032	No	No	No	No	--	--	--	--
XLOC_026034	No	No	No	No	CEL54483.1	--	--	--
XLOC_026035	No	No	No	No	--	--	--	--
XLOC_026036	No	No	No	No	--	--	--	--
XLOC_026038	No	No	No	No	--	--	--	--
XLOC_026043	No	No	No	No	XP_003049860.1	--	J	--
XLOC_026047	No	No	No	No	--	--	--	--
XLOC_026056	No	No	No	No	AJI44491.1	--	--	K02261
XLOC_026060	No	No	No	No	--	--	--	--
XLOC_026090	No	No	No	No	--	--	--	--
XLOC_026098	No	No	No	No	--	--	J	K02519

Differentially expressed gene (DEG) IDs	*C. microphyllum* root and shoot-specific (>2 fold)	*C. microphyllum* root and shoot-specific (>10 fold)	ICC4958 and *C. microphyllum* root-specific (>2 fold)	*C. microphyllum* root-specific (>2 fold)	Functional Annotation			
					NCBI nr IDs	TFs	KOG	KEGG
XLOC_026099	DEG(M)	No	No	No	XP_002900135.1	--	J	--
XLOC_026111	No	No	No	No	ELU41243.1	--	--	--
XLOC_026113	DEG(M)	No	No	No	EUC54109.1	--	--	--
XLOC_026115	No	No	No	No	ELU38316.1	--	--	--
XLOC_026116	No	No	No	No	EUC56524.1	--	I	--
XLOC_026117	DEG(M)	No	No	No	--	--	--	--
XLOC_026131	DEG(M)	No	No	No	CEL51752.1	--	--	--
XLOC_026132	No	No	No	No	--	--	--	--
XLOC_026133	No	No	DEG(I+M)	No	--	--	--	--
XLOC_026134	No	No	DEG(I+M)	No	--	--	--	--
XLOC_026152	DEG(M)	No	No	No	--	--	--	--
XLOC_026153	DEG(M)	No	No	No	--	--	--	--
XLOC_026154	No	No	No	No	--	--	--	--
XLOC_026156	DEG(M)	No	No	No	--	--	--	--
XLOC_026167	DEG(M)	No	No	No	XP_003049688.1	--	C	--
XLOC_026170	DEG(M)	DEG(M)	No	No	AGM32536.1	--	Z	--
XLOC_026171	DEG(M)	No	No	No	--	--	--	--
XLOC_026172	DEG(M)	DEG(M)	DEG(I+M)	DEG(M)	--	--	--	--
XLOC_026175	DEG(M)	No	No	No	--	--	--	--
XLOC_026177	No	No	No	No	KDN35563.1	--	--	--
XLOC_026179	No	No	No	No	EUC57379.1	--	E	--
XLOC_026180	No	No	No	No	--	--	--	--
XLOC_026183	No	No	No	No	--	--	--	--
XLOC_026185	No	No	No	No	--	--	--	--
XLOC_026189	No	No	No	No	XP_008186946.1	--	R	--
XLOC_026190	DEG(M)	No	No	No	NP_001119699.1	NAC	J	K02976
XLOC_026191	No	No	No	No	ETL92721.1	--	O	K01366
XLOC_026193	No	No	No	No	EUC53696.1	--	--	--
XLOC_026197	No	No	No	No	BAJ99169.1	--	J	K02981
XLOC_026198	No	No	No	No	XP_008185714.1	--	--	--
XLOC_026199	DEG(M)	No	No	No	KDN41703.1	--	J	--
XLOC_026200	No	No	No	No	--	--	--	--
XLOC_026202	No	No	No	No	CEL60424.1	--	--	--
XLOC_026203	No	No	No	No	--	--	--	--
XLOC_026207	No	No	No	No	XP_002611030.1	M-type	O	--
XLOC_026210	DEG(M)	DEG(M)	No	No	YP_001649167.1	--	C	K02262
XLOC_026211	No	No	No	No	KDR20480.1	--	C	--
XLOC_026216	No	No	No	No	XP_002399544.1	--	U	K07953
XLOC_026218	DEG(M)	DEG(M)	No	No	--	--	--	--
XLOC_026219	No	No	No	No	--	--	--	--

Differentially expressed gene (DEG) IDs	*C. microphyllum* root and shoot-specific (>2 fold)	*C. microphyllum* root and shoot-specific (>10 fold)	ICC4958 and *C. microphyllum* root-specific (>2 fold)	*C. microphyllum* root-specific (>2 fold)	Functional Annotation			
					NCBI nr IDs	TFs	KOG	KEGG
XLOC_026222	No	No	No	No	ETK76711.1	--	--	--
XLOC_026223	No	No	No	No	ETI56774.1	GATA	U	--
XLOC_026226	No	No	No	No	AAU84936.1	--	--	--
XLOC_026227	No	No	No	No	AAB87414.1	--	--	--
XLOC_026229	No	No	No	No	--	--	--	--
XLOC_026233	No	No	No	No	--	--	J	--
XLOC_026234	No	No	No	No	XP_003299225.1	--	--	--
XLOC_026237	No	No	No	No	--	--	--	--
XLOC_026239	DEG(M)	No	No	No	--	--	J	K02971
XLOC_026241	DEG(M)	No	No	No	--	--	--	--
XLOC_026246	No	No	No	No	--	--	--	--
XLOC_026249	No	No	No	No	XP_004501217.1	--	--	K14488
XLOC_026250	DEG(M)	No	No	No	--	--	--	--
XLOC_026251	DEG(M)	No	No	No	--	--	--	--
XLOC_026252	No	No	No	No	--	--	--	--
XLOC_026253	No	No	No	No	KEP54792.1	--	--	--
XLOC_026254	DEG(M)	No	No	No	--	--	J	K02949
XLOC_026255	No	No	No	No	--	--	--	--
XLOC_026259	No	No	No	No	--	--	--	--
XLOC_026263	DEG(M)	No	No	No	--	--	--	--
XLOC_026264	No	No	No	No	XP_001950697.1	--	--	--
XLOC_026265	No	No	No	No	XP_001950697.1	--	--	--
XLOC_026267	No	No	DEG(I+M)	No	XP_004485616.1	--	--	--
XLOC_026271	No	No	No	No	--	--	--	--
XLOC_026275	No	No	No	No	EUC54804.1	--	UR	K17065
XLOC_026276	No	No	No	No	--	--	--	--
XLOC_026279	No	No	No	No	--	--	--	--
XLOC_026286	DEG(M)	No	No	No	ELU39546.1	--	--	--
XLOC_026288	No	No	No	No	EUC66706.1	--	--	--
XLOC_026289	No	No	No	No	ELU39451.1	--	--	K01265
XLOC_026292	No	No	No	No	XP_004516400.1	--	--	--
XLOC_026294	No	No	No	No	--	--	--	--
XLOC_026295	DEG(M)	No	No	No	EWZ33365.1	--	--	--
XLOC_026300	No	No	No	No	--	--	--	--
XLOC_026301	No	No	No	No	KDN42337.1	--	--	--
XLOC_026303	No	No	No	No	--	--	--	--
XLOC_026305	No	No	No	No	XP_003053565.1	--	C	--
XLOC_026309	No	No	No	No	XP_003050443.1	--	C	--
XLOC_026310	No	No	No	No	--	--	--	--
XLOC_026311	No	No	No	No	--	--	--	--

Differentially expressed gene (DEG) IDs	*C. microphyllum* root and shoot-specific (>2 fold)	*C. microphyllum* root and shoot-specific (>10 fold)	ICC4958 and *C. microphyllum* root-specific (>2 fold)	*C. microphyllum* root-specific (>2 fold)	Functional Annotation			
					NCBI nr IDs	TFs	KOG	KEGG
XLOC_026317	No	No	No	No	--	--	--	--
XLOC_026318	No	No	No	No	--	--	--	--
XLOC_026326	No	No	No	No	--	--	--	--
XLOC_026327	DEG(M)	No	No	No	ELU43596.1	--	--	--
XLOC_026328	No	No	No	No	--	--	--	--
XLOC_026331	No	No	No	No	--	--	--	--
XLOC_026335	No	No	No	No	--	--	--	--
XLOC_026336	DEG(M)	No	No	No	--	--	--	--
XLOC_026337	No	No	No	No	ETO63043.1	--	--	--
XLOC_026339	No	No	No	No	EUC63377.1	--	--	K12373
XLOC_026342	No	No	No	No	EHJ72875.1	--	O	K03062
XLOC_026345	No	No	No	No	--	--	--	--
XLOC_026346	No	No	No	No	ETM01350.1	--	--	--
XLOC_026353	No	No	DEG(I+M)	No	--	--	--	--
XLOC_026355	No	No	No	No	XP_003053118.1	--	--	--
XLOC_026357	DEG(M)	No	No	No	--	--	--	--
XLOC_026363	No	No	No	No	--	--	--	--
XLOC_026368	No	No	No	No	--	--	I	K17756
XLOC_026370	No	No	No	No	--	--	T	K01114
XLOC_026372	No	No	No	No	XP_012075699.1	--	I	K08730
XLOC_026374	No	No	No	No	CCO29710.1	--	--	--
XLOC_026376	No	No	No	No	--	--	--	K00058
XLOC_026378	DEG(M)	No	No	No	--	--	--	--
XLOC_026379	No	No	No	No	KIM29844.1	--	--	--
XLOC_026380	No	No	No	No	EUC66843.1	--	--	--
XLOC_026386	No	No	No	No	--	--	--	--
XLOC_026387	No	No	No	No	--	--	O	K03029
XLOC_026391	No	No	No	No	XP_008911015.1	--	--	--
XLOC_026393	No	No	No	No	XP_008187269.1	--	--	--
XLOC_026395	No	No	No	No	--	--	--	K02148
XLOC_026396	No	No	No	No	XP_001951873.2	--	U	--
XLOC_026397	DEG(M)	No	No	No	CCO35603.1	--	--	--
XLOC_026398	No	No	No	No	AFK09744.1	--	--	--
XLOC_026400	No	No	No	No	--	--	--	--
XLOC_026402	No	No	No	No	--	--	E	--
XLOC_026403	No	No	No	No	--	--	--	--
XLOC_026404	No	No	No	No	--	--	--	--
XLOC_026408	No	No	No	No	--	--	--	--
XLOC_026409	No	No	No	No	CEL55554.1	--	O	--
XLOC_026417	No	No	No	No	CCO27020.1	--	--	--

Differentially expressed gene (DEG) IDs	*C. microphyllum* root and shoot-specific (>2 fold)	*C. microphyllum* root and shoot-specific (>10 fold)	ICC4958 and *C. microphyllum* root-specific (>2 fold)	*C. microphyllum* root-specific (>2 fold)	Functional Annotation			
					NCBI nr IDs	TFs	KOG	KEGG
XLOC_026420	No	No	DEG(I+M)	No	--	--	--	--
XLOC_026421	No	No	No	No	--	--	--	--
XLOC_026426	No	No	No	No	ETK77978.1	--	--	--
XLOC_026428	No	No	No	No	EUC62495.1	--	--	--
XLOC_026430	No	No	No	No	--	--	--	--
XLOC_026432	DEG(M)	No	No	No	--	--	--	--
XLOC_026433	No	No	No	No	CCA16391.1	--	U	--
XLOC_026435	No	No	No	No	XP_003053773.1	--	--	--
XLOC_026439	DEG(M)	No	DEG(I+M)	DEG(M)	CEL58056.1	--	J	--
XLOC_026440	No	No	No	No	--	--	--	K08762
XLOC_026443	No	No	No	No	--	--	--	K11593
XLOC_026444	No	No	No	No	--	--	--	--
XLOC_026447	No	No	No	No	--	--	--	--
XLOC_026448	No	No	No	No	--	--	--	--
XLOC_026449	DEG(M)	No	No	No	CEL59824.1	--	F	--
XLOC_026450	No	No	No	No	XP_002514626.1	--	X	K13379
XLOC_026452	DEG(M)	No	No	No	--	--	--	--
XLOC_026473	No	No	DEG(I+M)	No	--	--	G	--
XLOC_026476	DEG(M)	No	No	No	--	--	--	--
XLOC_026498	No	No	No	No	XP_007716173.1	--	--	K01057
XLOC_026499	No	No	No	No	--	--	--	--
XLOC_026500	No	No	No	No	--	--	--	--
XLOC_026501	No	No	No	No	--	--	--	--
XLOC_026505	No	No	No	No	--	--	--	--
XLOC_026508	DEG(M)	DEG(M)	No	No	XP_001940792.1	--	OE	K16297
XLOC_026512	DEG(M)	DEG(M)	DEG(I+M)	DEG(M)	XP_004500113.1	--	QV	K18848
XLOC_026517	No	No	No	No	--	--	--	--
XLOC_026520	No	No	DEG(I+M)	No	--	--	--	--
XLOC_026530	DEG(M)	No	DEG(I+M)	DEG(M)	--	--	--	--
XLOC_026531	DEG(M)	DEG(M)	DEG(I+M)	DEG(M)	--	--	--	--
XLOC_026532	DEG(M)	DEG(M)	No	No	--	--	--	--
XLOC_026539	No	No	No	No	--	--	--	--
XLOC_026543	DEG(M)	No	No	No	CEL61857.1	--	--	--
XLOC_026547	No	No	No	No	CEL63847.1	--	E	--
XLOC_026548	DEG(M)	No	No	No	--	--	--	--
XLOC_026551	DEG(M)	No	No	No	ETP20291.1	--	O	--
XLOC_026552	No	No	No	No	XP_001661529.1	--	--	K14538
XLOC_026553	No	No	No	No	XP_004940673.1	--	FE	K00948
XLOC_026554	No	No	No	No	XP_001950956.1	--	Q	--
XLOC_026555	No	No	No	No	--	--	Z	--

Differentially expressed gene (DEG) IDs	*C. microphyllum* root and shoot-specific (>2 fold)	*C. microphyllum* root and shoot-specific (>10 fold)	ICC4958 and *C. microphyllum* root-specific (>2 fold)	*C. microphyllum* root-specific (>2 fold)	Functional Annotation			
					NCBI nr IDs	TFs	KOG	KEGG
XLOC_026559	No	No	No	No	--	--	--	--
XLOC_026561	No	No	No	No	KDN46556.1	--	--	--
XLOC_026562	No	No	No	No	--	--	--	--
XLOC_026563	No	No	No	No	XP_009525871.1	--	C	--
XLOC_026564	No	No	No	No	XP_008908914.1	--	--	--
XLOC_026565	No	No	No	No	XP_009516788.1	--	--	K02736
XLOC_026567	No	No	No	No	--	--	--	K02691
XLOC_026569	No	No	No	No	--	--	O	K02731
XLOC_026574	DEG(M)	No	No	No	XP_009541432.1	--	J	K02978
XLOC_026576	No	No	No	No	KDN34912.1	--	--	--
XLOC_026579	DEG(M)	No	No	No	--	--	--	--
XLOC_026585	No	No	No	No	--	--	--	--
XLOC_026586	DEG(M)	No	No	No	KDN45630.1	--	--	--
XLOC_026587	DEG(M)	DEG(M)	No	No	--	--	--	--
XLOC_026592	No	No	No	No	EWZ34308.1	--	--	--
XLOC_026593	DEG(M)	DEG(M)	No	No	NP_001129424.1	--	J	--
XLOC_026594	DEG(M)	No	No	No	--	--	--	--
XLOC_026596	No	No	No	No	CCO31229.1	--	C	--
XLOC_026597	No	No	No	No	ELU41204.1	--	C	K03942
XLOC_026600	DEG(M)	No	No	No	--	--	--	--
XLOC_026602	DEG(M)	No	No	No	--	--	--	--
XLOC_026603	DEG(M)	No	No	No	--	--	--	--
XLOC_026606	No	No	No	No	XP_004353426.1	--	Z	K17260
XLOC_026607	DEG(M)	No	No	No	NP_001156203.1	--	Z	--
XLOC_026608	DEG(M)	DEG(M)	No	No	EXK47841.1	--	--	--
XLOC_026615	No	No	No	No	ELU40118.1	--	--	--
XLOC_026616	No	No	No	No	--	--	--	--
XLOC_026617	No	No	No	No	--	--	--	--
XLOC_026619	No	No	No	No	--	--	--	--
XLOC_026628	No	No	No	No	--	--	--	--
XLOC_026632	No	No	No	No	--	--	--	--
XLOC_026633	No	No	No	No	--	--	--	--
XLOC_026636	No	No	No	No	--	--	--	--
XLOC_026639	No	No	No	No	--	--	--	--
XLOC_026641	DEG(M)	No	DEG(I+M)	DEG(M)	--	--	--	--
XLOC_026647	No	No	No	No	--	--	--	K10590
XLOC_026649	DEG(M)	No	No	No	--	--	--	--
XLOC_026650	No	No	No	No	--	--	--	--
XLOC_026651	No	No	No	No	--	--	--	--
XLOC_026653	No	No	No	No	--	--	--	--

Differentially expressed gene (DEG) IDs	*C. microphyllum* root and shoot-specific (>2 fold)	*C. microphyllum* root and shoot-specific (>10 fold)	ICC4958 and *C. microphyllum* root-specific (>2 fold)	*C. microphyllum* root-specific (>2 fold)	Functional Annotation			
					NCBI nr IDs	TFs	KOG	KEGG
XLOC_026657	No	No	No	No	ETP44092.1	--	--	--
XLOC_026659	No	No	No	No	XP_001652214.2	--	--	--
XLOC_026660	No	No	No	No	XP_003040938.1	--	--	--
XLOC_026661	No	No	No	No	--	--	--	--
XLOC_026663	DEG(M)	No	No	No	EHK27357.1	--	--	--
XLOC_026665	No	No	No	No	AKM95032.1	--	--	--
XLOC_026668	No	No	No	No	--	--	--	--
XLOC_026669	No	No	No	No	--	--	G	--
XLOC_026670	No	No	No	No	--	--	--	--
XLOC_026676	DEG(M)	No	DEG(I+M)	DEG(M)	--	--	--	--
XLOC_026677	DEG(M)	No	No	No	--	--	--	--
XLOC_026678	No	No	No	No	--	--	E	--
XLOC_026679	DEG(M)	No	No	No	--	--	--	--
XLOC_026681	No	No	No	No	--	--	--	--
XLOC_026682	DEG(M)	No	No	No	AAA37165.1	Dof	Z	--
XLOC_026687	No	No	No	No	--	--	--	--
XLOC_026691	No	No	No	No	--	MYB	--	--
XLOC_026693	No	No	No	No	--	--	--	--
XLOC_026695	DEG(M)	DEG(M)	No	No	EWZ40196.1	--	--	--
XLOC_026696	No	No	No	No	AFG25455.1	--	--	--
XLOC_026697	No	No	No	No	--	--	--	--
XLOC_026698	DEG(M)	No	No	No	XP_009521350.1	--	--	--
XLOC_026699	No	No	No	No	XP_008894060.1	--	--	--
XLOC_026701	No	No	No	No	--	--	--	--
XLOC_026702	No	No	No	No	ETP38567.1	--	--	--
XLOC_026704	No	No	DEG(I+M)	No	--	--	--	--
XLOC_026706	No	No	No	No	--	--	C	K00026
XLOC_026710	No	No	No	No	CEL63900.1	--	--	--
XLOC_026714	No	No	No	No	--	--	--	--
XLOC_026716	No	No	No	No	--	--	--	--
XLOC_026721	No	No	No	No	CEL56810.1	--	--	--
XLOC_026725	DEG(M)	No	No	No	--	--	J	K02995
XLOC_026727	No	No	No	No	--	--	--	--
XLOC_026728	DEG(M)	DEG(M)	No	No	EXL58319.1	--	--	--
XLOC_026730	DEG(M)	DEG(M)	DEG(I+M)	DEG(M)	XP_002903189.1	--	T	--
XLOC_026731	No	No	No	No	AAL85608.1	--	--	--
XLOC_026732	DEG(M)	No	No	No	--	--	--	K13993
XLOC_026733	DEG(M)	DEG(M)	No	No	--	--	--	--
XLOC_026734	No	No	No	No	XP_001664110.1	--	R	--
XLOC_026736	No	No	No	No	--	--	--	--

Differentially expressed gene (DEG) IDs	*C. microphyllum* root and shoot-specific (>2 fold)	*C. microphyllum* root and shoot-specific (>10 fold)	ICC4958 and *C. microphyllum* root-specific (>2 fold)	*C. microphyllum* root-specific (>2 fold)	Functional Annotation			
					NCBI nr IDs	TFs	KOG	KEGG
XLOC_026739	No	No	No	No	EWZ28850.1	--	--	--
XLOC_026741	No	No	No	No	XP_001653157.1	--	--	--
XLOC_026746	No	No	No	No	--	--	--	--
XLOC_026749	No	No	No	No	--	--	--	--
XLOC_026756	No	No	No	No	--	--	--	--
XLOC_026758	No	No	No	No	--	--	--	--
XLOC_026760	No	No	No	No	XP_011056576.1	--	J	K03113
XLOC_026763	No	No	No	No	--	--	--	K14411
XLOC_026765	No	No	No	No	--	--	--	--
XLOC_026766	DEG(M)	No	No	No	--	--	--	--
XLOC_026769	DEG(M)	DEG(M)	No	No	--	--	--	--
XLOC_026771	DEG(M)	No	No	No	CEL60735.1	--	--	--
XLOC_026776	DEG(M)	No	No	No	--	--	--	--
XLOC_026777	No	No	No	No	--	--	--	--
XLOC_026782	No	No	No	No	--	--	--	--
XLOC_026785	No	No	No	No	--	--	--	--
XLOC_026787	No	No	No	No	--	--	--	--
XLOC_026791	DEG(M)	No	No	No	XP_009519011.1	--	AJ	K14564
XLOC_026792	No	No	No	No	--	--	--	--
XLOC_026793	No	No	No	No	--	--	J	--
XLOC_026794	No	No	No	No	--	--	T	--
XLOC_026796	DEG(M)	No	No	No	XP_004492663.1	--	--	--
XLOC_026797	No	No	No	No	ETM44482.1	--	--	--
XLOC_026799	DEG(M)	No	No	No	XP_003042200.1	--	--	--
XLOC_026804	DEG(M)	No	No	No	XP_008892320.1	--	G	--
XLOC_026805	DEG(M)	No	No	No	XP_009518911.1	--	G	K00616
XLOC_026806	No	No	No	No	XP_008896144.1	--	--	--
XLOC_026823	DEG(M)	No	No	No	CEL55175.1	--	--	--
XLOC_026842	No	No	No	No	--	--	--	--
XLOC_026846	No	No	No	No	XP_004339426.1	--	J	--
XLOC_026848	No	No	No	No	EUC66297.1	--	--	--
XLOC_026849	DEG(M)	No	No	No	KDN37043.1	--	--	--
XLOC_026851	No	No	No	No	--	--	C	--
XLOC_026856	No	No	DEG(I+M)	No	XP_004494244.1	--	--	--
XLOC_026867	DEG(M)	No	No	No	XP_002415781.1	--	J	--
XLOC_026868	DEG(M)	No	No	No	ETL91302.1	--	--	--
XLOC_026881	DEG(M)	No	No	No	--	--	--	--
XLOC_026894	No	No	No	No	--	--	--	--
XLOC_026897	No	No	No	No	--	--	--	K09419
XLOC_026899	DEG(M)	No	No	No	AEK51007.1	--	G	--

Differentially expressed gene (DEG) IDs	*C. microphyllum* root and shoot-specific (>2 fold)	*C. microphyllum* root and shoot-specific (>10 fold)	ICC4958 and *C. microphyllum* root-specific (>2 fold)	*C. microphyllum* root-specific (>2 fold)	Functional Annotation			
					NCBI nr IDs	TFs	KOG	KEGG
XLOC_026900	DEG(M)	No	No	No	AEK51007.1	--	G	--
XLOC_026904	DEG(M)	No	No	No	KIM85231.1	--	J	--
XLOC_026909	No	No	No	No	XP_004500893.1	--	O	K03798
XLOC_026915	DEG(M)	No	No	No	CEL52648.1	--	--	--
XLOC_026916	DEG(M)	DEG(M)	DEG(I+M)	DEG(M)	--	--	--	--
XLOC_026917	No	No	No	No	ETO73995.1	--	--	K13126
XLOC_026918	No	No	No	No	XP_008893142.1	--	--	K13126
XLOC_026927	No	No	No	No	ELU41698.1	--	--	--
XLOC_026935	No	No	No	No	XP_008611237.1	--	H	K01251
XLOC_026945	DEG(M)	No	No	No	--	--	--	--
XLOC_026947	No	No	No	No	XP_004510894.1	MIKC	K	--
XLOC_026949	DEG(M)	No	DEG(I+M)	DEG(M)	EUC63918.1	--	--	--
XLOC_026950	DEG(M)	No	No	No	EUC63918.1	--	--	--
XLOC_026959	No	No	No	No	XP_012573818.1	--	R	K03327
XLOC_026963	DEG(M)	No	No	No	CCO33518.1	--	--	--
XLOC_026964	DEG(M)	No	No	No	--	--	--	--
XLOC_026969	DEG(M)	No	No	No	--	--	--	--
XLOC_026982	No	No	No	No	--	--	--	--
XLOC_026983	DEG(M)	DEG(M)	DEG(I+M)	DEG(M)	XP_003052953.1	--	J	--
XLOC_026999	No	No	No	No	XP_008180168.1	--	R	K06943
XLOC_027001	No	No	No	No	--	--	--	--
XLOC_027002	No	No	No	No	EUC65729.1	--	--	--
XLOC_027004	No	No	No	No	--	--	--	--
XLOC_027005	No	No	No	No	--	--	--	--
XLOC_027011	No	No	No	No	XP_004506989.1	--	RT	K14488
XLOC_027017	No	No	No	No	--	--	--	--
XLOC_027018	DEG(M)	No	No	No	XP_003054230.1	--	J	--
XLOC_027019	DEG(M)	No	No	No	--	--	--	--
XLOC_027020	DEG(M)	No	No	No	CCO33174.1	--	--	--
XLOC_027021	No	No	No	No	AHW98770.1	--	O	--
XLOC_027022	No	No	No	No	ETI30980.1	--	--	--
XLOC_027023	No	No	No	No	XP_009524613.1	--	--	--
XLOC_027024	No	No	No	No	EUC67548.1	--	--	K03934
XLOC_027025	No	No	DEG(I+M)	No	XP_004507254.1	--	--	K00318
XLOC_027027	No	No	No	No	XP_003044889.1	--	J	--
XLOC_027036	No	No	No	No	--	--	--	--
XLOC_027037	DEG(M)	No	No	No	--	--	--	--
XLOC_027040	DEG(M)	No	No	No	--	--	--	--
XLOC_027042	No	No	No	No	ETO64498.1	--	--	--
XLOC_027044	No	No	No	No	ETI50251.1	--	--	--

Differentially expressed gene (DEG) IDs	*C. microphyllum* root and shoot-specific (>2 fold)	*C. microphyllum* root and shoot-specific (>10 fold)	ICC4958 and *C. microphyllum* root-specific (>2 fold)	*C. microphyllum* root-specific (>2 fold)	Functional Annotation			
					NCBI nr IDs	TFs	KOG	KEGG
XLOC_027045	No	No	No	No	ETK90134.1	--	--	--
XLOC_027048	No	No	No	No	--	--	--	--
XLOC_027051	No	No	DEG(I+M)	No	--	--	G	K00850
XLOC_027053	DEG(M)	No	No	No	NP_476776.1	--	J	--
XLOC_027054	DEG(M)	No	No	No	XP_004487911.1	--	--	--
XLOC_027059	No	No	No	No	AHZ64887.1	--	--	--
XLOC_027062	No	No	No	No	NP_001153863.1	--	R	--
XLOC_027064	DEG(M)	No	No	No	EUC62385.1	--	--	K13448
XLOC_027065	No	No	No	No	--	--	--	--
XLOC_027069	No	No	No	No	XP_002314631.1	--	C	--
XLOC_027070	No	No	No	No	--	--	--	--
XLOC_027072	DEG(M)	No	No	No	XP_010067635.1	--	X	--
XLOC_027073	DEG(M)	DEG(M)	No	No	--	--	--	--
XLOC_027075	No	No	No	No	--	--	--	--
XLOC_027077	No	No	No	No	CCO36961.1	--	--	--
XLOC_027080	No	No	No	No	CCO31112.1	--	--	--
XLOC_027082	No	No	No	No	XP_004487301.1	--	--	--
XLOC_027086	No	No	No	No	--	--	--	--
XLOC_027087	No	No	No	No	--	--	--	K03676
XLOC_027090	No	No	No	No	KEP46123.1	--	--	--
XLOC_027094	No	No	No	No	--	--	--	--
XLOC_027097	DEG(M)	No	No	No	--	--	--	--
XLOC_027099	No	No	No	No	XP_009521067.1	--	--	K01507
XLOC_027101	DEG(M)	No	No	No	KLP08057.1	--	A	--
XLOC_027102	DEG(M)	No	No	No	--	--	J	K02987
XLOC_027103	No	No	No	No	CCX34699.1	--	P	--
XLOC_027105	DEG(M)	No	DEG(I+M)	DEG(M)	--	--	--	--
XLOC_027106	No	No	No	No	XP_004503701.1	--	QI	K15398
XLOC_027113	DEG(M)	DEG(M)	No	No	--	--	--	--
XLOC_027114	DEG(M)	No	No	No	--	--	--	--
XLOC_027119	DEG(M)	No	DEG(I+M)	DEG(M)	XP_012450468.1	--	J	--
XLOC_027123	No	No	No	No	--	--	--	K19996
XLOC_027124	No	No	No	No	--	--	--	--
XLOC_027125	DEG(M)	No	No	No	ETP41502.1	--	--	--
XLOC_027136	No	No	No	No	ETO07171.1	--	O	--
XLOC_027137	No	No	No	No	ACG60424.1	--	O	--
XLOC_027138	DEG(M)	DEG(M)	DEG(I+M)	DEG(M)	--	--	--	--
XLOC_027140	DEG(M)	No	No	No	KFH42044.1	--	G	--
XLOC_027143	No	No	No	No	--	--	--	--
XLOC_027144	DEG(M)	No	No	No	--	--	--	--

Differentially expressed gene (DEG) IDs	*C. microphyllum* root and shoot-specific (>2 fold)	*C. microphyllum* root and shoot-specific (>10 fold)	ICC4958 and *C. microphyllum* root-specific (>2 fold)	*C. microphyllum* root-specific (>2 fold)	Functional Annotation			
					NCBI nr IDs	TFs	KOG	KEGG
XLOC_027146	DEG(M)	No	No	No	EXL55587.1	--	--	--
XLOC_027149	No	No	No	No	XP_003049904.1	--	--	--
XLOC_027151	DEG(M)	No	No	No	NP_001119642.1	--	Z	--
XLOC_027153	DEG(M)	No	No	No	--	--	--	--
XLOC_027154	No	No	No	No	XP_008181705.1	--	--	--
XLOC_027155	DEG(M)	No	No	No	--	--	--	--
XLOC_027156	No	No	No	No	--	--	--	--
XLOC_027157	No	No	No	No	CEL59818.1	--	--	K09553
XLOC_027159	No	No	No	No	--	--	--	--
XLOC_027160	DEG(M)	No	No	No	KDN51287.1	--	F	K00940
XLOC_027163	DEG(M)	DEG(M)	DEG(I+M)	DEG(M)	--	--	--	--
XLOC_027165	DEG(M)	DEG(M)	DEG(I+M)	DEG(M)	--	--	--	--
XLOC_027169	DEG(M)	DEG(M)	No	No	--	--	--	--
XLOC_027173	No	No	No	No	--	--	--	K01674
XLOC_027177	DEG(M)	No	No	No	--	--	--	--
XLOC_027178	DEG(M)	No	No	No	--	--	--	--
XLOC_027182	DEG(M)	DEG(M)	No	No	KNB02931.1	--	J	--
XLOC_027184	No	No	No	No	KDN51743.1	--	--	--
XLOC_027185	No	No	No	No	--	--	--	--
XLOC_027188	DEG(M)	No	No	No	XP_003048881.1	--	--	--
XLOC_027193	DEG(M)	No	No	No	CCO29552.1	--	--	--
XLOC_027195	DEG(M)	No	DEG(I+M)	DEG(M)	XP_004494100.1	--	--	--
XLOC_027196	No	No	No	No	XP_004488673.1	STAT	H	K05356
XLOC_027199	No	No	No	No	AJI42791.1	--	G	--
XLOC_027203	No	No	No	No	XP_003043394.1	--	--	--
XLOC_027204	No	No	No	No	--	--	--	--
XLOC_027209	No	No	No	No	--	--	--	--
XLOC_027211	DEG(M)	No	No	No	CAB88203.1	--	--	--
XLOC_027212	DEG(M)	DEG(M)	DEG(I+M)	DEG(M)	XP_009514839.1	--	--	K02983
XLOC_027213	DEG(M)	No	No	No	XP_009539526.1	--	--	--
XLOC_027224	DEG(M)	No	No	No	CEL53211.1	--	--	K13447
XLOC_027225	No	No	No	No	--	--	--	--
XLOC_027231	DEG(M)	No	No	No	KEP51454.1	--	--	--
XLOC_027234	No	No	No	No	--	--	--	--
XLOC_027237	No	No	No	No	ETI50357.1	--	--	--
XLOC_027238	No	No	No	No	--	--	--	--
XLOC_027240	No	No	No	No	EUC55908.1	--	--	--
XLOC_027241	No	No	No	No	KEP50067.1	--	--	--
XLOC_027243	No	No	No	No	--	--	--	--
XLOC_027247	No	No	No	No	--	--	T	K03456

Differentially expressed gene (DEG) IDs	*C. microphyllum* root and shoot-specific (>2 fold)	*C. microphyllum* root and shoot-specific (>10 fold)	ICC4958 and *C. microphyllum* root-specific (>2 fold)	*C. microphyllum* root-specific (>2 fold)	Functional Annotation			
					NCBI nr IDs	TFs	KOG	KEGG
XLOC_027249	No	No	No	No	--	--	--	--
XLOC_027250	No	No	No	No	--	--	--	--
XLOC_027255	No	No	No	No	CCO30083.1	--	--	--
XLOC_027257	No	No	No	No	--	--	--	--
XLOC_027268	DEG(M)	No	No	No	--	--	--	--
XLOC_027272	DEG(M)	No	No	No	CCO28333.1	--	T	--
XLOC_027288	No	No	No	No	NP_001265952.1	--	I	--
XLOC_027292	No	No	DEG(I+M)	No	XP_004492423.1	--	--	K01988
XLOC_027295	No	No	No	No	XP_004516681.1	--	--	--
XLOC_027301	No	No	No	No	--	--	--	--
XLOC_027317	No	No	DEG(I+M)	No	--	Trihelix	--	--
XLOC_027336	No	No	No	No	--	--	J	K02492
XLOC_027339	No	No	No	No	--	--	--	--
XLOC_027341	DEG(M)	No	No	No	CCO26883.1	--	J	--
XLOC_027342	DEG(M)	DEG(M)	No	No	CEL57717.1	--	J	K02925
XLOC_027343	DEG(M)	No	DEG(I+M)	DEG(M)	CCO26884.1	--	J	--
XLOC_027344	DEG(M)	No	No	No	CEL57717.1	--	J	--
XLOC_027345	DEG(M)	No	DEG(I+M)	DEG(M)	XP_003051543.1	--	J	--
XLOC_027346	DEG(M)	No	DEG(I+M)	DEG(M)	ETI43909.1	--	J	--
XLOC_027347	DEG(M)	No	DEG(I+M)	DEG(M)	ETI43909.1	--	J	K02925
XLOC_027352	DEG(M)	No	No	No	--	--	--	--
XLOC_027359	DEG(M)	DEG(M)	DEG(I+M)	DEG(M)	--	--	--	--
XLOC_027361	DEG(M)	DEG(M)	DEG(I+M)	DEG(M)	--	--	--	--
XLOC_027362	DEG(M)	DEG(M)	DEG(I+M)	DEG(M)	--	--	--	--
XLOC_027363	DEG(M)	DEG(M)	DEG(I+M)	DEG(M)	--	--	--	--
XLOC_027364	DEG(M)	DEG(M)	DEG(I+M)	DEG(M)	--	--	--	--
XLOC_027365	DEG(M)	No	DEG(I+M)	DEG(M)	--	--	--	--
XLOC_027366	DEG(M)	DEG(M)	DEG(I+M)	DEG(M)	--	--	--	--
XLOC_027367	DEG(M)	DEG(M)	DEG(I+M)	DEG(M)	--	--	--	--
XLOC_027381	DEG(M)	No	No	No	KEP51052.1	--	--	--
XLOC_027382	DEG(M)	No	No	No	--	--	--	--
XLOC_027383	No	No	No	No	--	--	--	--
XLOC_027384	DEG(M)	No	No	No	XP_001951042.1	--	J	K02925
XLOC_027387	No	No	No	No	NP_001280420.1	--	O	--
XLOC_027388	No	No	No	No	--	--	--	--
XLOC_027389	No	No	No	No	EXL94327.1	--	--	--
XLOC_027391	No	No	No	No	--	--	--	--
XLOC_027394	No	No	No	No	--	--	--	--
XLOC_027395	No	No	No	No	ETM50765.1	--	--	K03695
XLOC_027401	No	No	No	No	CEO95857.1	--	U	--

Differentially expressed gene (DEG) IDs	*C. microphyllum* root and shoot-specific (>2 fold)	*C. microphyllum* root and shoot-specific (>10 fold)	ICC4958 and *C. microphyllum* root-specific (>2 fold)	*C. microphyllum* root-specific (>2 fold)	Functional Annotation			
					NCBI nr IDs	TFs	KOG	KEGG
XLOC_027402	No	No	No	No	--	--	--	--
XLOC_027406	No	No	No	No	XP_003054347.1	--	--	--
XLOC_027407	No	No	No	No	--	--	--	--
XLOC_027408	No	No	No	No	--	--	--	--
XLOC_027410	DEG(M)	No	No	No	XP_002909101.1	MYB_related	--	--
XLOC_027417	No	No	No	No	--	--	--	--
XLOC_027419	DEG(M)	No	No	No	XP_008893396.1	--	F	K00940
XLOC_027423	No	No	No	No	XP_002512840.1	--	G	K01191
XLOC_027429	No	No	No	No	--	--	--	--
XLOC_027430	No	No	No	No	--	--	--	--
XLOC_027431	No	No	No	No	CEL60669.1	--	--	--
XLOC_027435	No	No	No	No	XP_004511988.1	--	--	--
XLOC_027436	No	No	No	No	KJB54978.1	--	PT	--
XLOC_027437	No	No	No	No	XP_008367750.1	--	PT	--
XLOC_027440	DEG(M)	DEG(M)	No	No	XP_003051186.1	--	T	--
XLOC_027441	No	No	No	No	XP_001907345.1	--	T	--
XLOC_027442	No	No	No	No	--	--	--	--
XLOC_027443	No	No	No	No	KEP46093.1	--	--	--
XLOC_027444	No	No	No	No	--	--	--	--
XLOC_027447	No	No	No	No	ETM36369.1	--	--	--
XLOC_027448	No	No	No	No	--	--	--	--
XLOC_027449	No	No	No	No	CEL55057.1	--	--	--
XLOC_027450	No	No	No	No	--	--	--	--
XLOC_027453	No	No	No	No	KEP55719.1	--	--	--
XLOC_027454	DEG(M)	No	DEG(I+M)	DEG(M)	XP_004487816.1	--	--	K13449
XLOC_027457	DEG(M)	No	No	No	XP_008182408.1	--	R	K14411
XLOC_027459	No	No	DEG(I+M)	No	XP_003536895.1	--	--	--
XLOC_027462	No	No	No	No	--	--	--	--
XLOC_027463	No	No	No	No	CCO28352.1	--	--	--
XLOC_027465	DEG(M)	DEG(M)	DEG(I+M)	DEG(M)	--	--	--	--
XLOC_027468	DEG(M)	No	No	No	EUC57832.1	--	--	--
XLOC_027469	No	No	No	No	KDN35656.1	--	--	--
XLOC_027470	No	No	No	No	--	--	--	--
XLOC_027475	No	No	No	No	ELU41444.1	--	--	--
XLOC_027480	No	No	No	No	XP_001659906.1	--	--	--
XLOC_027482	No	No	No	No	--	--	--	--
XLOC_027485	DEG(M)	No	No	No	--	--	--	--
XLOC_027486	DEG(M)	No	DEG(I+M)	DEG(M)	--	--	--	--
XLOC_027487	No	No	No	No	XP_001947745.1	--	--	--
XLOC_027488	No	No	No	No	EXK38789.1	--	--	--

Differentially expressed gene (DEG) IDs	*C. microphyllum* root and shoot-specific (>2 fold)	*C. microphyllum* root and shoot-specific (>10 fold)	ICC4958 and *C. microphyllum* root-specific (>2 fold)	*C. microphyllum* root-specific (>2 fold)	Functional Annotation			
					NCBI nr IDs	TFs	KOG	KEGG
XLOC_027489	No	No	No	No	NP_001119692.1	--	O	K10577
XLOC_027490	No	No	No	No	KDN51555.1	--	--	--
XLOC_027491	DEG(M)	No	No	No	--	--	--	K02937
XLOC_027494	No	No	No	No	CCO35797.1	--	--	--
XLOC_027495	No	No	No	No	--	--	--	--
XLOC_027499	DEG(M)	No	No	No	--	--	R	--
XLOC_027500	No	No	No	No	XP_008183536.1	--	--	--
XLOC_027508	No	No	No	No	--	--	--	--
XLOC_027511	No	No	No	No	ACC59199.1	--	I	--
XLOC_027512	DEG(M)	No	No	No	--	--	--	--
XLOC_027513	No	No	No	No	ETL28020.1	--	O	--
XLOC_027518	No	No	No	No	XP_008907369.1	--	--	K14272
XLOC_027525	DEG(M)	No	DEG(I+M)	DEG(M)	KDN50216.1	--	--	--
XLOC_027526	DEG(M)	No	No	No	XP_001659895.1	--	U	--
XLOC_027529	No	No	No	No	XP_008914025.1	--	--	--
XLOC_027531	No	No	No	No	--	--	--	--
XLOC_027534	DEG(M)	No	DEG(I+M)	DEG(M)	XP_008898524.1	--	J	--
XLOC_027535	DEG(M)	DEG(M)	DEG(I+M)	DEG(M)	XP_008898524.1	--	J	--
XLOC_027537	No	No	No	No	--	--	--	--
XLOC_027538	No	No	No	No	--	--	--	--
XLOC_027539	No	No	No	No	XP_003293307.1	NAC	J	--
XLOC_027540	No	No	No	No	CCO30844.1	--	HR	K01950
XLOC_027541	No	No	No	No	CEL55733.1	--	--	--
XLOC_027543	No	No	No	No	AEV66511.1	--	--	--
XLOC_027544	No	No	No	No	XP_003041794.1	--	--	--
XLOC_027556	DEG(M)	No	No	No	XP_003045998.1	--	--	--
XLOC_027561	DEG(M)	No	No	No	CCO31672.1	--	Z	--
XLOC_027569	No	No	DEG(I+M)	No	--	--	--	--
XLOC_027570	No	No	No	No	--	--	--	K03113
XLOC_027571	DEG(M)	No	DEG(I+M)	DEG(M)	CEL53892.1	--	--	K00011
XLOC_027572	DEG(M)	DEG(M)	No	No	--	--	Q	K00512
XLOC_027579	DEG(M)	DEG(M)	No	No	NP_001265906.1	--	R	K01183
XLOC_027595	DEG(M)	No	No	No	XP_003045277.1	--	--	--
XLOC_027598	DEG(M)	No	No	No	--	--	--	K08653
XLOC_027608	DEG(M)	No	No	No	XP_003047689.1	--	--	--
XLOC_027609	No	No	No	No	CCO27701.1	--	--	--
XLOC_027620	No	No	DEG(I+M)	No	XP_004509414.1	--	--	--
XLOC_027621	DEG(M)	No	No	No	--	--	--	--
XLOC_027633	No	No	DEG(I+M)	No	--	--	--	--
XLOC_027634	No	No	DEG(I+M)	No	NP_001266108.1	--	--	--

**Differentially expressed gene (DEG) IDs**	***C. microphyllum* root and shoot-specific (>2 fold)**	***C. microphyllum* root and shoot-specific (>10 fold)**	**ICC4958 and *C. microphyllum* root-specific (>2 fold)**	***C. microphyllum* root-specific (>2 fold)**	**Functional Annotation**			
					**NCBI nr IDs**	**TFs**	**KOG**	**KEGG**
XLOC_027641	No	No	No	No	--	--	--	--
XLOC_027646	DEG(M)	DEG(M)	DEG(I+M)	DEG(M)	--	--	--	--
XLOC_027648	DEG(M)	No	No	No	--	--	--	--
XLOC_027655	No	No	No	No	--	--	--	--
XLOC_027656	No	No	No	No	--	--	--	--
XLOC_027658	No	No	No	No	--	--	--	--
XLOC_027660	DEG(M)	No	No	No	--	--	--	--
XLOC_027665	No	No	No	No	--	--	UR	--
XLOC_027668	No	No	No	No	--	--	--	--
XLOC_027670	No	No	No	No	XP_008896972.1	--	--	--
XLOC_027672	No	No	No	No	--	--	--	--
XLOC_027673	No	No	No	No	--	--	--	--
XLOC_027682	No	No	No	No	--	--	--	--
XLOC_027684	No	No	No	No	--	--	--	--
XLOC_027686	No	No	No	No	ETL39607.1	--	--	--
XLOC_027688	No	No	No	No	XP_012369293.1	FAR1	TR	--
XLOC_027690	No	No	No	No	NP_001119629.1	--	--	--
XLOC_027691	No	No	No	No	--	--	--	--
XLOC_027692	DEG(M)	No	No	No	--	--	J	K02901
XLOC_027693	No	No	No	No	KEP54399.1	--	--	--
XLOC_027697	No	No	No	No	--	--	E	K01657
XLOC_027704	No	No	No	No	--	--	--	--
XLOC_027714	No	No	No	No	XP_001948117.1	--	--	--
XLOC_027718	DEG(M)	DEG(M)	No	No	XP_001948026.1	--	J	--
XLOC_027722	No	No	No	No	NP_001156243.1	--	P	K04565
XLOC_027726	No	No	No	No	--	--	--	--
XLOC_027727	No	No	No	No	--	--	--	--
XLOC_027729	DEG(M)	No	No	No	EMT60876.1	--	--	--
XLOC_027730	No	No	No	No	NP_502365.1	NF-YB	J	K02973
XLOC_027731	No	No	No	No	--	--	--	--
XLOC_027732	No	No	No	No	--	--	--	--
XLOC_027733	No	No	No	No	EXA38330.1	--	--	--
XLOC_027734	No	No	No	No	--	--	--	K02135
XLOC_027740	DEG(M)	No	No	No	AJQ30119.1	--	C	K01647
XLOC_027746	DEG(M)	No	No	No	XP_008890941.1	--	--	--
XLOC_027747	No	No	No	No	--	--	--	--
XLOC_027748	No	No	No	No	--	--	--	K02976
XLOC_027750	No	No	No	No	CCO37886.1	--	--	--
XLOC_027752	No	No	No	No	--	--	--	--
XLOC_027756	No	No	No	No	EUC54970.1	--	T	K14753

**Differentially expressed gene (DEG) IDs**	***C. microphyllum* root and shoot-specific (>2 fold)**	***C. microphyllum* root and shoot-specific (>10 fold)**	**ICC4958 and *C. microphyllum* root-specific (>2 fold)**	***C. microphyllum* root-specific (>2 fold)**	**Functional Annotation**			
					**NCBI nr IDs**	**TFs**	**KOG**	**KEGG**
XLOC_027758	DEG(M)	No	No	No	CCO34704.1	--	T	--
XLOC_027775	No	No	No	No	ETK80736.1	--	--	K11000
XLOC_027790	No	No	No	No	ACU45029.1	--	J	--
XLOC_027791	No	No	No	No	EXK76811.1	--	--	--
XLOC_027797	DEG(M)	No	DEG(I+M)	DEG(M)	--	--	--	--
XLOC_027801	No	No	No	No	--	--	--	--
XLOC_027802	No	No	No	No	--	--	--	--
XLOC_027804	No	No	No	No	--	--	Q	--
XLOC_027805	DEG(M)	DEG(M)	No	No	CRK21303.1	--	O	--
XLOC_027806	DEG(M)	DEG(M)	No	No	XP_003046530.1	--	O	--
XLOC_027807	No	No	No	No	XP_008914578.1	--	O	--
XLOC_027816	No	No	DEG(I+M)	No	XP_012568012.1	LBD	--	--
XLOC_027824	No	No	No	No	EUC65473.1	--	G	--
XLOC_027825	No	No	No	No	CEL58219.1	--	G	--
XLOC_027854	No	No	DEG(I+M)	No	--	--	--	--
XLOC_027868	No	No	No	No	--	--	--	--
XLOC_027878	No	No	No	No	XP_004495805.1	--	--	K08241
XLOC_027879	DEG(M)	No	No	No	--	--	--	--
XLOC_027903	No	No	No	No	--	--	--	--
XLOC_027904	DEG(M)	No	No	No	--	--	--	--
XLOC_027906	No	No	No	No	--	--	--	--
XLOC_027941	DEG(M)	No	DEG(I+M)	DEG(M)	--	--	--	--
XLOC_027951	DEG(M)	No	No	No	--	--	--	--
XLOC_027952	DEG(M)	No	DEG(I+M)	DEG(M)	--	--	--	--
XLOC_027955	No	No	No	No	--	--	--	--
XLOC_027972	DEG(M)	DEG(M)	No	No	EUC66219.1	--	J	--
XLOC_027973	No	No	No	No	--	--	--	--
XLOC_027974	DEG(M)	No	DEG(I+M)	DEG(M)	CEL63896.1	bZIP	J	--
XLOC_027979	No	No	DEG(I+M)	No	--	NAC	M	--
XLOC_027987	DEG(M)	DEG(M)	DEG(I+M)	DEG(M)	EUC58347.1	--	--	--
XLOC_027988	DEG(M)	DEG(M)	DEG(I+M)	DEG(M)	EUC58347.1	--	--	--
XLOC_027989	DEG(M)	DEG(M)	DEG(I+M)	DEG(M)	CCO34556.1	--	--	--
XLOC_027990	DEG(M)	DEG(M)	DEG(I+M)	DEG(M)	CCO34556.1	--	--	--
XLOC_027991	DEG(M)	DEG(M)	DEG(I+M)	DEG(M)	KEP46235.1	--	--	--
XLOC_027992	DEG(M)	No	No	No	KDN38337.1	--	--	--
XLOC_027993	DEG(M)	No	No	No	ACO50132.1	--	J	--
XLOC_027994	DEG(M)	DEG(M)	No	No	AII77176.1	--	J	--
XLOC_027995	DEG(M)	DEG(M)	DEG(I+M)	DEG(M)	AGC59647.1	--	J	--
XLOC_027996	DEG(M)	No	No	No	ACA63752.1	--	J	--
XLOC_027997	DEG(M)	No	No	No	ADG23121.1	--	J	--

Differentially expressed gene (DEG) IDs	*C. microphyllum* root and shoot-specific (>2 fold)	*C. microphyllum* root and shoot-specific (>10 fold)	ICC4958 and *C. microphyllum* root-specific (>2 fold)	*C. microphyllum* root-specific (>2 fold)	Functional Annotation			
					NCBI nr IDs	TFs	KOG	KEGG
XLOC_027999	DEG(M)	DEG(M)	DEG(I+M)	DEG(M)	ETL93330.1	--	J	--
XLOC_028006	No	No	No	No	--	--	--	--
XLOC_028040	DEG(M)	DEG(M)	No	No	NP_001156072.1	--	--	--
XLOC_028041	DEG(M)	DEG(M)	No	No	XP_001950829.1	--	--	--
XLOC_028053	No	No	No	No	--	--	--	--
XLOC_028060	No	No	No	No	EUC54947.1	--	--	--
XLOC_028061	DEG(M)	DEG(M)	DEG(I+M)	DEG(M)	CEL54174.1	--	--	--
XLOC_028062	No	No	No	No	EUC54947.1	--	--	--
XLOC_028067	No	No	No	No	--	--	--	K11294
XLOC_028068	DEG(M)	No	DEG(I+M)	DEG(M)	--	--	--	--
XLOC_028094	DEG(M)	DEG(M)	No	No	--	--	--	--
XLOC_028097	No	No	No	No	--	--	--	--
XLOC_028098	DEG(M)	No	No	No	--	--	--	--
XLOC_028133	No	No	No	No	XP_004501717.1	--	P	--
XLOC_028135	DEG(M)	No	DEG(I+M)	DEG(M)	--	--	--	--
XLOC_028136	DEG(M)	DEG(M)	DEG(I+M)	DEG(M)	KDN42656.1	--	--	--
XLOC_028137	DEG(M)	No	DEG(I+M)	DEG(M)	EUC56853.1	--	--	--
XLOC_028138	DEG(M)	DEG(M)	DEG(I+M)	DEG(M)	EUC56853.1	--	--	--
XLOC_028158	DEG(M)	DEG(M)	No	No	--	--	--	--
XLOC_028169	No	No	No	No	--	--	--	--
XLOC_028177	No	No	No	No	--	--	--	--
XLOC_028183	DEG(M)	No	No	No	--	--	--	--
XLOC_028187	No	No	No	No	--	--	--	--
XLOC_028188	No	No	No	No	--	--	--	--
XLOC_028192	No	No	No	No	--	--	--	--
XLOC_028193	No	No	No	No	--	--	--	--
XLOC_028198	No	No	No	No	XP_003043913.1	--	--	--
XLOC_028199	DEG(M)	No	DEG(I+M)	DEG(M)	--	--	J	--
XLOC_028200	No	No	No	No	--	--	--	--
XLOC_028201	DEG(M)	No	No	No	--	--	--	--
XLOC_028202	No	No	No	No	NP_001155462.1	--	O	--
XLOC_028205	No	No	No	No	XP_004515900.1	--	--	--
XLOC_028206	No	No	No	No	--	--	--	--
XLOC_028209	No	No	No	No	EUC57457.1	--	--	--
XLOC_028210	No	No	No	No	XP_008903595.1	--	A	--
XLOC_028212	DEG(M)	No	No	No	XP_008443726.1	--	MVG	--
XLOC_028215	No	No	No	No	--	--	V	--
XLOC_028224	DEG(M)	DEG(M)	DEG(I+M)	DEG(M)	EEP52471.1	--	--	--
XLOC_028229	No	No	No	No	--	--	--	--
XLOC_028230	DEG(M)	No	No	No	--	--	--	--

Differentially expressed gene (DEG) IDs	***C. microphyllum* root and shoot-specific (>2 fold)**	***C. microphyllum* root and shoot-specific (>10 fold)**	**ICC4958 and *C. microphyllum* root-specific (>2 fold)**	***C. microphyllum* root-specific (>2 fold)**	**Functional Annotation**			
					**NCBI nr IDs**	**TFs**	**KOG**	**KEGG**
XLOC_028238	DEG(M)	No	No	No	--	--	--	--
XLOC_028240	DEG(M)	DEG(M)	DEG(I+M)	DEG(M)	ETI50516.1	--	O	--
XLOC_028241	DEG(M)	DEG(M)	DEG(I+M)	DEG(M)	--	--	--	--
XLOC_028255	DEG(M)	DEG(M)	No	No	--	--	Q	K09840
XLOC_028259	No	No	No	No	--	--	--	--
XLOC_028267	DEG(M)	No	No	No	ABC85751.1	--	Z	--
XLOC_028269	No	No	No	No	--	--	--	--
XLOC_028280	DEG(M)	No	No	No	--	--	--	--
XLOC_028283	DEG(M)	No	No	No	--	--	--	--
XLOC_028284	DEG(M)	DEG(M)	DEG(I+M)	DEG(M)	--	--	--	--
XLOC_028285	DEG(M)	No	No	No	--	--	--	--
XLOC_028286	No	No	No	No	--	--	--	--
XLOC_028287	No	No	No	No	EXL89957.1	--	--	--
XLOC_028288	No	No	No	No	ENH74236.1	--	--	--
XLOC_028294	No	No	No	No	--	--	--	--
XLOC_028306	DEG(M)	DEG(M)	DEG(I+M)	DEG(M)	--	--	--	--
XLOC_028307	DEG(M)	No	DEG(I+M)	DEG(M)	--	--	--	--
XLOC_028310	DEG(M)	No	No	No	KEP51263.1	--	--	--
XLOC_028311	No	No	No	No	KDN38622.1	--	--	--
XLOC_028312	DEG(M)	DEG(M)	DEG(I+M)	DEG(M)	EUC58221.1	--	--	--
XLOC_028313	No	No	No	No	XP_008899209.1	--	--	--
XLOC_028316	No	No	No	No	--	--	--	--
XLOC_028317	No	No	No	No	--	--	--	K03952
XLOC_028319	No	No	No	No	--	--	--	--
XLOC_028321	DEG(M)	No	No	No	--	--	--	--
XLOC_028331	No	No	No	No	--	--	--	--
XLOC_028333	No	No	No	No	EUC64819.1	--	--	--
XLOC_028337	No	No	No	No	--	--	--	--
XLOC_028338	No	No	No	No	--	--	--	--
XLOC_028341	DEG(M)	DEG(M)	DEG(I+M)	DEG(M)	EMT66245.1	--	--	--
XLOC_028343	No	No	No	No	--	--	--	--
XLOC_028344	DEG(M)	No	No	No	KEP49308.1	--	--	--
XLOC_028345	No	No	No	No	NP_001155532.1	--	--	--
XLOC_028346	No	No	No	No	--	--	--	--
XLOC_028349	DEG(M)	No	No	No	XP_009519261.1	--	O	--
XLOC_028350	No	No	No	No	XP_002901731.1	--	--	--
XLOC_028356	No	No	No	No	--	--	--	--
XLOC_028360	DEG(M)	No	No	No	--	--	J	K02880
XLOC_028369	No	No	No	No	EUC60240.1	--	--	--
XLOC_028370	No	No	No	No	BAH70523.1	--	--	--

Differentially expressed gene (DEG) IDs	*C. microphyllum* root and shoot-specific (>2 fold)	*C. microphyllum* root and shoot-specific (>10 fold)	ICC4958 and *C. microphyllum* root-specific (>2 fold)	*C. microphyllum* root-specific (>2 fold)	Functional Annotation			
					NCBI nr IDs	TFs	KOG	KEGG
XLOC_028371	DEG(M)	No	No	No	ABR23354.1	--	J	K02981
XLOC_028373	No	No	No	No	--	--	--	--
XLOC_028374	No	No	No	No	EXM09465.1	--	--	--
XLOC_028380	DEG(M)	No	No	No	CCI43479.1	--	--	--
XLOC_028383	DEG(M)	No	DEG(I+M)	DEG(M)	KEP54950.1	--	--	--
XLOC_028384	No	No	No	No	XP_003243379.1	--	TDK	K03097
XLOC_028386	DEG(M)	No	No	No	--	--	--	--
XLOC_028387	No	No	No	No	KDN49678.1	--	A	K11088
XLOC_028391	No	No	No	No	--	--	--	K01805
XLOC_028393	No	No	No	No	--	--	J	K02885
XLOC_028396	No	No	No	No	XP_011871111.1	--	R	--
XLOC_028397	No	No	No	No	ETL83389.1	--	--	--
XLOC_028399	No	No	No	No	--	--	--	--
XLOC_028402	No	No	No	No	EGU79132.1	--	J	--
XLOC_028404	No	No	No	No	--	--	--	--
XLOC_028407	No	No	No	No	KEP51885.1	--	--	--
XLOC_028408	No	No	No	No	XP_004496551.1	--	--	--
XLOC_028410	No	No	No	No	--	--	--	K02903
XLOC_028412	No	No	No	No	XP_003054475.1	--	--	--
XLOC_028415	No	No	DEG(I+M)	No	--	--	--	--
XLOC_028419	No	No	No	No	--	--	--	--
XLOC_028420	No	No	No	No	AIA25514.1	--	J	--
XLOC_028422	No	No	No	No	CCO37844.1	--	--	--
XLOC_028423	DEG(M)	DEG(M)	DEG(I+M)	DEG(M)	AFJ04519.1	YABBY	OR	--
XLOC_028430	DEG(M)	No	No	No	--	--	--	--
XLOC_028432	DEG(M)	No	DEG(I+M)	DEG(M)	CCO26451.1	--	--	--
XLOC_028436	No	No	No	No	--	--	E	--
XLOC_028438	DEG(M)	No	No	No	--	--	--	--
XLOC_028443	No	No	No	No	--	--	P	K14640
XLOC_028444	No	No	No	No	--	--	G	K01193
XLOC_028453	No	No	No	No	--	--	C	K06444
XLOC_028466	DEG(M)	DEG(M)	DEG(I+M)	DEG(M)	XP_004487728.1	--	--	--
XLOC_028495	No	No	No	No	--	--	LT	K01669
XLOC_028497	No	No	No	No	--	--	--	--
XLOC_028502	No	No	No	No	XP_004498807.1	--	--	--
XLOC_028507	DEG(M)	No	No	No	--	--	--	--
XLOC_028518	No	No	No	No	--	--	--	--
XLOC_028519	DEG(M)	No	No	No	--	--	--	--
XLOC_028525	DEG(M)	No	DEG(I+M)	DEG(M)	AIE16180.1	MIKC	G	--
XLOC_028526	No	No	No	No	XP_008903478.1	--	--	--

Differentially expressed gene (DEG) IDs	*C. microphyllum* root and shoot-specific (>2 fold)	*C. microphyllum* root and shoot-specific (>10 fold)	ICC4958 and *C. microphyllum* root-specific (>2 fold)	*C. microphyllum* root-specific (>2 fold)	Functional Annotation			
					NCBI nr IDs	TFs	KOG	KEGG
XLOC_028527	No	No	No	No	--	--	--	--
XLOC_028528	No	No	No	No	EUC59240.1	--	--	--
XLOC_028529	No	No	No	No	--	--	--	--
XLOC_028530	No	No	No	No	ETL30424.1	--	--	--
XLOC_028531	DEG(M)	No	No	No	BAH72914.1	--	Z	--
XLOC_028532	No	No	No	No	--	--	--	--
XLOC_028534	DEG(M)	No	No	No	XP_002161180.1	--	J	K02929
XLOC_028535	No	No	No	No	--	--	--	--
XLOC_028537	No	No	No	No	--	--	--	--
XLOC_028538	DEG(M)	No	DEG(I+M)	DEG(M)	--	--	--	--
XLOC_028540	No	No	No	No	XP_001663284.1	--	ZT	--
XLOC_028542	No	No	No	No	XP_003051472.1	--	--	--
XLOC_028544	No	No	No	No	--	--	--	--
XLOC_028545	No	No	No	No	--	--	--	--
XLOC_028554	DEG(M)	No	No	No	EUC57132.1	--	--	--
XLOC_028557	DEG(M)	No	No	No	ELU42413.1	--	--	--
XLOC_028562	DEG(M)	No	No	No	--	--	--	--
XLOC_028577	DEG(M)	No	No	No	--	--	--	--
XLOC_028582	No	No	No	No	XP_004505756.1	--	--	--
XLOC_028585	No	No	No	No	--	--	X	--
XLOC_028590	No	No	No	No	--	--	--	--
XLOC_028594	No	No	No	No	--	--	--	--
XLOC_028595	DEG(M)	No	No	No	--	--	--	K03949
XLOC_028596	No	No	No	No	EUC64203.1	--	--	--
XLOC_028599	No	No	No	No	CCO27643.1	--	--	K02940
XLOC_028600	No	No	No	No	CCO27643.1	--	--	--
XLOC_028606	No	No	No	No	--	--	--	--
XLOC_028607	No	No	No	No	--	--	--	K01938
XLOC_028610	DEG(M)	No	No	No	EUC62328.1	--	--	--
XLOC_028614	DEG(M)	No	No	No	--	--	--	--
XLOC_028616	No	No	No	No	XP_004493244.1	--	--	--
XLOC_028617	DEG(M)	No	No	No	CCO34697.1	--	--	--
XLOC_028618	No	No	No	No	--	MIKC	R	--
XLOC_028622	No	No	No	No	--	--	--	--
XLOC_028624	DEG(M)	No	No	No	KDN41553.1	--	--	--
XLOC_028626	DEG(M)	No	No	No	--	--	--	--
XLOC_028628	DEG(M)	DEG(M)	DEG(I+M)	DEG(M)	AFK45939.1	--	--	--
XLOC_028629	DEG(M)	No	No	No	XP_011516977.1	--	R	--
XLOC_028634	No	No	No	No	--	--	--	--
XLOC_028635	No	No	No	No	EUC53682.1	--	--	--

Differentially expressed gene (DEG) IDs	*C. microphyllum* root and shoot-specific (>2 fold)	*C. microphyllum* root and shoot-specific (>10 fold)	ICC4958 and *C. microphyllum* root-specific (>2 fold)	*C. microphyllum* root-specific (>2 fold)	Functional Annotation			
					NCBI nr IDs	TFs	KOG	KEGG
XLOC_028636	No	No	No	No	EUC65371.1	--	--	--
XLOC_028637	DEG(M)	No	DEG(I+M)	DEG(M)	--	--	--	--
XLOC_028638	DEG(M)	No	DEG(I+M)	DEG(M)	--	--	--	--
XLOC_028643	No	No	No	No	CEL55279.1	--	--	K01897
XLOC_028644	No	No	No	No	XP_004511391.1	--	--	--
XLOC_028646	No	No	No	No	--	--	--	--
XLOC_028650	DEG(M)	DEG(M)	DEG(I+M)	DEG(M)	XP_003610095.1	--	--	K01602
XLOC_028651	No	No	No	No	--	--	O	K10590
XLOC_028652	DEG(M)	DEG(M)	DEG(I+M)	DEG(M)	--	--	--	--
XLOC_028653	No	No	No	No	XP_001952321.2	--	--	K04708
XLOC_028658	No	No	No	No	EUC65229.1	--	--	--
XLOC_028659	DEG(M)	No	No	No	--	--	J	--
XLOC_028661	DEG(M)	No	No	No	--	--	PT	--
XLOC_028662	DEG(M)	No	No	No	EUC62243.1	--	AJ	--
XLOC_028663	DEG(M)	DEG(M)	No	No	EGU77457.1	--	--	--
XLOC_028664	DEG(M)	No	No	No	ABI52727.1	--	U	K07937
XLOC_028670	No	No	No	No	--	--	--	--
XLOC_028672	No	No	No	No	EUC57750.1	--	--	--
XLOC_028674	No	No	No	No	CCO26184.1	--	--	--
XLOC_028675	No	No	No	No	XP_008180721.1	--	--	--
XLOC_028677	DEG(M)	No	No	No	--	--	--	--
XLOC_028679	No	No	No	No	--	--	--	--
XLOC_028680	No	No	No	No	--	--	--	--
XLOC_028684	No	No	No	No	--	--	--	K14568
XLOC_028688	No	No	No	No	XP_012092930.1	--	R	--
XLOC_028689	No	No	No	No	--	--	--	--
XLOC_028692	No	No	No	No	XP_006451747.1	--	X	--
XLOC_028700	No	No	No	No	--	--	--	--
XLOC_028702	No	No	No	No	--	--	--	--
XLOC_028703	DEG(M)	No	No	No	--	--	--	--
XLOC_028705	No	No	No	No	--	--	--	--
XLOC_028706	No	No	No	No	XP_003043730.1	--	--	--
XLOC_028712	No	No	No	No	--	--	--	--
XLOC_028713	No	No	No	No	XP_003052026.1	--	--	--
XLOC_028714	No	No	No	No	XP_001659022.1	--	J	--
XLOC_028715	No	No	No	No	--	--	--	--
XLOC_028716	No	No	No	No	KNG48448.1	--	--	--
XLOC_028717	No	No	No	No	CEL54495.1	WOX	U	K07904
XLOC_028718	No	No	No	No	--	--	--	--
XLOC_028721	DEG(M)	DEG(M)	No	No	XP_003051763.1	--	J	--

Differentially expressed gene (DEG) IDs	*C. microphyllum* root and shoot-specific (>2 fold)	*C. microphyllum* root and shoot-specific (>10 fold)	ICC4958 and *C. microphyllum* root-specific (>2 fold)	*C. microphyllum* root-specific (>2 fold)	Functional Annotation			
					NCBI nr IDs	TFs	KOG	KEGG
XLOC_028725	DEG(M)	No	No	No	XP_008900606.1	--	--	--
XLOC_028727	No	No	No	No	--	--	--	--
XLOC_028729	No	No	No	No	--	--	--	--
XLOC_028730	No	No	No	No	--	--	--	K05579
XLOC_028731	No	No	No	No	--	--	--	--
XLOC_028733	No	No	No	No	ABB77566.1	--	--	--
XLOC_028736	No	No	No	No	XP_001661472.1	--	--	--
XLOC_028738	DEG(M)	No	No	No	CCO26701.1	--	C	--
XLOC_028744	No	No	No	No	XP_001945902.2	FAR1	TR	--
XLOC_028746	DEG(M)	No	No	No	NP_001119689.1	--	J	K02964
XLOC_028747	DEG(M)	No	No	No	--	--	--	--
XLOC_028748	No	No	DEG(I+M)	No	XP_004511510.1	--	--	--
XLOC_028749	No	No	No	No	--	--	--	--
XLOC_028750	No	No	No	No	--	--	--	--
XLOC_028751	No	No	No	No	--	--	--	--
XLOC_028759	DEG(M)	No	No	No	EXA31217.1	--	--	--
XLOC_028762	No	No	No	No	--	--	--	K15223
XLOC_028763	DEG(M)	No	No	No	XP_004513719.1	MIKC	K	K09264
XLOC_028766	No	No	No	No	XP_001950847.1	--	E	--
XLOC_028769	DEG(M)	No	No	No	EUC66513.1	--	--	--
XLOC_028771	DEG(M)	No	No	No	EUC58691.1	--	--	--
XLOC_028775	DEG(M)	DEG(M)	No	No	XP_003050052.1	--	--	--
XLOC_028776	No	No	No	No	--	--	--	--
XLOC_028779	No	No	No	No	CCO31965.1	--	UO	--
XLOC_028794	DEG(M)	No	No	No	--	--	--	--
XLOC_028798	No	No	No	No	CEL59513.1	--	--	--
XLOC_028800	No	No	No	No	--	--	O	--
XLOC_028801	No	No	No	No	--	--	--	--
XLOC_028808	DEG(M)	No	No	No	--	--	--	K02942
XLOC_028810	No	No	No	No	XP_001653796.1	--	J	--
XLOC_028811	No	No	No	No	EMD95951.1	--	--	--
XLOC_028814	No	No	No	No	--	--	--	--
XLOC_028816	No	No	No	No	--	--	--	--
XLOC_028818	No	No	No	No	--	--	--	--
XLOC_028822	No	No	No	No	XP_001951714.2	--	--	K07297
XLOC_028832	No	No	No	No	--	--	C	--
XLOC_028835	No	No	No	No	KHN81681.1	--	C	--
XLOC_028836	No	No	No	No	--	--	--	--
XLOC_028837	No	No	No	No	XP_001648461.1	--	--	--
XLOC_028838	No	No	No	No	--	--	--	--

Differentially expressed gene (DEG) IDs	*C. microphyllum* root and shoot-specific (>2 fold)	*C. microphyllum* root and shoot-specific (>10 fold)	ICC4958 and *C. microphyllum* root-specific (>2 fold)	*C. microphyllum* root-specific (>2 fold)	Functional Annotation			
					NCBI nr IDs	TFs	KOG	KEGG
XLOC_028846	DEG(M)	No	No	No	--	--	--	--
XLOC_028847	No	No	No	No	NP_001155637.1	--	K	K03627
XLOC_028856	DEG(M)	No	No	No	--	--	--	--
XLOC_028857	DEG(M)	No	No	No	--	--	--	--
XLOC_028859	DEG(M)	DEG(M)	No	No	XP_004500341.1	--	--	K00430
XLOC_028866	No	No	DEG(I+M)	No	--	--	--	--
XLOC_028871	DEG(M)	No	DEG(I+M)	DEG(M)	--	--	--	--
XLOC_028873	DEG(M)	No	DEG(I+M)	DEG(M)	KIK09034.1	--	J	--
XLOC_028875	No	No	DEG(I+M)	No	XP_009251452.1	--	J	--
XLOC_028879	No	No	No	No	--	--	--	--
XLOC_028882	DEG(M)	No	DEG(I+M)	DEG(M)	XP_009518629.1	--	K	K03626
XLOC_028884	No	No	No	No	--	--	J	--
XLOC_028887	DEG(M)	DEG(M)	No	No	XP_003045094.1	--	--	--
XLOC_028890	No	No	No	No	KDN38622.1	--	--	--
XLOC_028891	No	No	No	No	--	--	--	--
XLOC_028894	No	No	DEG(I+M)	No	--	--	--	--
XLOC_028899	No	No	No	No	--	--	--	--
XLOC_028907	DEG(M)	No	No	No	--	--	--	--
XLOC_028908	DEG(M)	No	No	No	--	--	--	--
XLOC_028909	DEG(M)	DEG(M)	DEG(I+M)	DEG(M)	--	--	--	--
XLOC_028913	No	No	No	No	--	--	--	--
XLOC_028918	DEG(M)	No	No	No	--	--	--	--
XLOC_028920	No	No	No	No	XP_007700356.1	--	--	--
XLOC_028922	DEG(M)	No	No	No	--	--	--	--
XLOC_028924	DEG(M)	No	No	No	XP_004515866.1	--	--	--
XLOC_028925	DEG(M)	No	No	No	--	--	--	--
XLOC_028927	DEG(M)	No	No	No	--	--	--	--
XLOC_028930	No	No	No	No	--	--	Q	K00218
XLOC_028938	No	No	No	No	--	--	S	--
XLOC_028939	No	No	No	No	--	MYB	--	--
XLOC_028943	No	No	No	No	XP_004512411.1	--	--	--
XLOC_028947	DEG(M)	No	No	No	--	--	--	--
XLOC_028954	No	No	No	No	XP_002520129.1	--	--	--
XLOC_028955	DEG(M)	No	No	No	--	--	--	--
XLOC_028958	No	No	No	No	XP_002521168.1	G2-like	E	--
XLOC_028964	No	No	No	No	--	--	--	--
XLOC_028968	No	No	No	No	ELU38658.1	--	--	--
XLOC_028970	DEG(M)	No	No	No	--	--	--	--
XLOC_028971	No	No	No	No	KEP49211.1	--	--	--
XLOC_028975	No	No	No	No	--	--	--	--

Differentially expressed gene (DEG) IDs	*C. microphyllum* root and shoot-specific (>2 fold)	*C. microphyllum* root and shoot-specific (>10 fold)	ICC4958 and *C. microphyllum* root-specific (>2 fold)	*C. microphyllum* root-specific (>2 fold)	Functional Annotation			
					NCBI nr IDs	TFs	KOG	KEGG
XLOC_028977	No	No	No	No	XP_001951125.1	--	--	--
XLOC_028978	No	No	No	No	EMT63668.1	--	--	--
XLOC_028981	No	No	DEG(I+M)	No	--	--	--	--
XLOC_028984	DEG(M)	No	No	No	--	--	--	K02155
XLOC_028986	No	No	No	No	--	--	--	K13464
XLOC_028988	DEG(M)	No	No	No	--	--	J	K02951
XLOC_028989	No	No	No	No	--	--	--	--
XLOC_028994	No	No	No	No	--	--	--	--
XLOC_028995	No	No	No	No	--	--	--	--
XLOC_029000	No	No	No	No	NP_001156218.1	--	--	--
XLOC_029001	No	No	No	No	KDN33621.1	--	--	--
XLOC_029002	No	No	No	No	ETO75055.1	--	--	--
XLOC_029007	No	No	No	No	KEP54602.1	--	--	--
XLOC_029008	DEG(M)	No	No	No	CCO28245.1	--	--	--
XLOC_029010	No	No	No	No	CEL55579.1	--	--	--
XLOC_029012	DEG(M)	No	No	No	--	--	--	--
XLOC_029013	DEG(M)	DEG(M)	DEG(I+M)	DEG(M)	CCO33134.1	--	U	--
XLOC_029014	No	No	No	No	--	--	--	--
XLOC_029017	No	No	No	No	ELU36700.1	--	--	--
XLOC_029020	No	No	No	No	XP_002525632.1	--	T	--
XLOC_029022	No	No	No	No	--	--	--	--
XLOC_029024	No	No	No	No	KDN34543.1	--	--	--
XLOC_029025	No	No	No	No	--	--	--	--
XLOC_029029	No	No	No	No	--	--	--	--
XLOC_029034	DEG(M)	No	No	No	--	--	--	--
XLOC_029035	DEG(M)	No	No	No	XP_002899876.1	--	--	K17279
XLOC_029036	No	No	No	No	--	--	--	--
XLOC_029037	No	No	No	No	--	--	--	--
XLOC_029038	No	No	DEG(I+M)	No	--	--	X	--
XLOC_029043	No	No	No	No	XP_001663284.1	--	--	--
XLOC_029047	No	No	No	No	ELU38673.1	NAC	J	--
XLOC_029050	No	No	No	No	XP_003041085.1	--	--	--
XLOC_029052	No	No	No	No	--	--	--	--
XLOC_029055	No	No	No	No	XP_010096538.1	NAC	M	--
XLOC_029060	DEG(M)	DEG(M)	DEG(I+M)	DEG(M)	--	--	--	--
XLOC_029061	No	No	No	No	--	--	--	--
XLOC_029065	No	No	No	No	--	--	--	--
XLOC_029066	No	No	No	No	KDN47592.1	--	--	--
XLOC_029068	No	No	No	No	--	--	--	K12382
XLOC_029069	DEG(M)	No	No	No	--	--	--	--

**Differentially expressed gene (DEG) IDs**	***C. microphyllum* root and shoot-specific (>2 fold)**	***C. microphyllum* root and shoot-specific (>10 fold)**	**ICC4958 and *C. microphyllum* root-specific (>2 fold)**	***C. microphyllum* root-specific (>2 fold)**	**Functional Annotation**			
					**NCBI nr IDs**	**TFs**	**KOG**	**KEGG**
XLOC_029071	No	No	No	No	--	--	--	--
XLOC_029073	No	No	No	No	XP_001654171.1	--	F	--
XLOC_029075	DEG(M)	No	No	No	--	--	--	--
XLOC_029080	No	No	No	No	--	--	--	K03676
XLOC_029086	No	No	DEG(I+M)	No	XP_004487557.1	C2H2	--	--
XLOC_029087	No	No	No	No	KEP48388.1	--	--	--
XLOC_029088	No	No	No	No	--	--	--	--
XLOC_029090	No	No	No	No	XP_009524464.1	--	--	K14006
XLOC_029091	No	No	No	No	--	--	--	--
XLOC_029093	No	No	No	No	--	--	--	--
XLOC_029096	No	No	DEG(I+M)	No	XP_004498980.1	--	--	--
XLOC_029101	DEG(M)	No	No	No	XP_003398066.1	--	J	--
XLOC_029102	No	No	No	No	XP_008911145.1	--	--	K15040
XLOC_029104	No	No	No	No	--	--	--	--
XLOC_029105	No	No	No	No	--	--	--	--
XLOC_029106	No	No	No	No	--	--	--	--
XLOC_029108	No	No	No	No	--	--	--	--
XLOC_029117	No	No	No	No	--	--	--	--
XLOC_029121	No	No	No	No	CEL53921.1	--	--	--
XLOC_029123	No	No	No	No	--	--	--	--
XLOC_029127	No	No	No	No	NP_001191944.1	--	--	--
XLOC_029128	No	No	No	No	--	--	--	--
XLOC_029132	No	No	No	No	CEL57595.1	--	--	--
XLOC_029133	No	No	No	No	CEL58298.1	--	--	--
XLOC_029137	No	No	No	No	KDN46034.1	--	--	--
XLOC_029138	No	No	DEG(I+M)	No	XP_004490683.1	--	--	--
XLOC_029140	No	No	No	No	EUC62271.1	--	--	K05906
XLOC_029145	DEG(M)	DEG(M)	DEG(I+M)	DEG(M)	EGU83680.1	--	--	--
XLOC_029149	DEG(M)	DEG(M)	No	No	KFM75839.1	--	O	--
XLOC_029161	DEG(M)	No	No	No	KDN43860.1	--	--	--
XLOC_029162	No	No	No	No	XP_003045058.1	--	--	--
XLOC_029164	DEG(M)	No	No	No	EUC64368.1	--	--	--
XLOC_029165	No	No	No	No	XP_012067275.1	--	--	--
XLOC_029166	No	No	No	No	--	--	--	--
XLOC_029167	No	No	No	No	XP_004496545.1	C2H2	--	--
XLOC_029168	DEG(M)	No	No	No	XP_004511511.1	--	--	--
XLOC_029170	DEG(M)	No	DEG(I+M)	DEG(M)	--	--	--	--
XLOC_029171	No	No	No	No	EUC55784.1	--	--	--
XLOC_029173	DEG(M)	No	No	No	--	--	O	--
XLOC_029175	No	No	No	No	ELU44569.1	--	--	--

Differentially expressed gene (DEG) IDs	***C. microphyllum* root and shoot-specific (>2 fold)**	***C. microphyllum* root and shoot-specific (>10 fold)**	**ICC4958 and *C. microphyllum* root-specific (>2 fold)**	***C. microphyllum* root-specific (>2 fold)**	**Functional Annotation**			
					**NCBI nr IDs**	**TFs**	**KOG**	**KEGG**
XLOC_029176	No	No	No	No	--	--	--	--
XLOC_029177	DEG(M)	No	No	No	XP_002526700.1	WRKY	KT	K13424
XLOC_029179	No	No	No	No	KEP53660.1	--	G	--
XLOC_029182	DEG(M)	No	No	No	BAJ87274.1	--	--	--
XLOC_029186	No	No	DEG(I+M)	No	--	--	--	--
XLOC_029190	No	No	No	No	--	--	--	--
XLOC_029198	No	No	No	No	KEP48870.1	--	--	--
XLOC_029214	DEG(M)	No	No	No	XP_002449484.1	--	--	--
XLOC_029215	DEG(M)	No	DEG(I+M)	DEG(M)	XP_002449484.1	--	--	--
XLOC_029222	No	No	No	No	--	--	C	K15109
XLOC_029224	DEG(M)	DEG(M)	No	No	--	--	--	--
XLOC_029225	DEG(M)	DEG(M)	DEG(I+M)	DEG(M)	--	--	--	--
XLOC_029226	DEG(M)	DEG(M)	No	No	--	--	--	--
XLOC_029227	DEG(M)	DEG(M)	DEG(I+M)	DEG(M)	--	--	--	--
XLOC_029234	No	No	DEG(I+M)	No	--	--	--	--
XLOC_029235	DEG(M)	No	No	No	EUC66575.1	--	--	--
XLOC_029246	No	No	No	No	CEL53284.1	--	--	--
XLOC_029247	No	No	No	No	CEL53284.1	--	--	K02910
XLOC_029248	DEG(M)	No	No	No	--	--	--	--
XLOC_029249	No	No	No	No	--	--	--	--
XLOC_029252	DEG(M)	No	DEG(I+M)	DEG(M)	CCO28490.1	--	--	--
XLOC_029262	DEG(M)	DEG(M)	DEG(I+M)	DEG(M)	EUC58715.1	--	--	--
XLOC_029263	DEG(M)	DEG(M)	No	No	CCO29809.1	--	--	--
XLOC_029264	No	No	No	No	--	--	--	K03541
XLOC_029269	DEG(M)	No	No	No	--	--	--	--
XLOC_029278	No	No	No	No	CEL52234.1	--	--	--
XLOC_029279	DEG(M)	No	No	No	ETM42895.1	--	--	--
XLOC_029281	No	No	No	No	--	--	--	--
XLOC_029284	DEG(M)	DEG(M)	DEG(I+M)	DEG(M)	XP_002903176.1	--	J	K02885
XLOC_029286	No	No	No	No	CEL55601.1	--	--	--
XLOC_029299	DEG(M)	No	No	No	--	--	--	--
XLOC_029300	DEG(M)	No	No	No	--	--	--	--
XLOC_029301	No	No	No	No	KDN50665.1	--	--	--
XLOC_029304	No	No	No	No	--	--	--	--
XLOC_029308	DEG(M)	DEG(M)	DEG(I+M)	DEG(M)	XP_004488020.1	--	R	--
XLOC_029312	No	No	No	No	ETP41184.1	--	--	--
XLOC_029320	No	No	No	No	--	--	--	K02699
XLOC_029322	No	No	No	No	--	--	--	--
XLOC_029334	DEG(M)	No	No	No	--	--	--	--
XLOC_029335	No	No	No	No	AFL91512.1	--	T	K03456

Differentially expressed gene (DEG) IDs	*C. microphyllum* root and shoot-specific (>2 fold)	*C. microphyllum* root and shoot-specific (>10 fold)	ICC4958 and *C. microphyllum* root-specific (>2 fold)	*C. microphyllum* root-specific (>2 fold)	Functional Annotation			
					NCBI nr IDs	TFs	KOG	KEGG
XLOC_029342	No	No	No	No	--	--	--	--
XLOC_029343	DEG(M)	No	No	No	KDN50332.1	--	J	--
XLOC_029345	No	No	No	No	--	--	--	--
XLOC_029347	No	No	No	No	--	--	--	--
XLOC_029348	No	No	No	No	XP_004493851.1	--	--	K02693
XLOC_029352	No	No	No	No	KCW58094.1	--	O	--
XLOC_029353	No	No	DEG(I+M)	No	--	--	--	--
XLOC_029354	No	No	No	No	--	--	--	--
XLOC_029358	No	No	No	No	--	--	--	--
XLOC_029359	No	No	No	No	--	--	--	--
XLOC_029361	No	No	No	No	--	--	--	--
XLOC_029363	DEG(M)	No	No	No	--	--	--	--
XLOC_029367	DEG(M)	DEG(M)	DEG(I+M)	DEG(M)	XP_009524206.1	--	J	--
XLOC_029369	No	No	No	No	--	--	--	--
XLOC_029370	No	No	No	No	--	--	--	--
XLOC_029373	No	No	No	No	--	--	--	--
XLOC_029375	DEG(M)	No	No	No	--	--	--	--
XLOC_029381	No	No	No	No	ETK74657.1	--	--	--
XLOC_029383	No	No	No	No	ADD24298.1	--	A	--
XLOC_029386	No	No	No	No	--	--	--	--
XLOC_029387	No	No	No	No	--	--	--	--
XLOC_029390	No	No	DEG(I+M)	No	--	--	--	--
XLOC_029391	No	No	No	No	--	--	--	--
XLOC_029394	No	No	No	No	--	--	--	--
XLOC_029396	DEG(M)	No	DEG(I+M)	DEG(M)	--	--	--	--
XLOC_029407	No	No	No	No	--	--	--	--
XLOC_029408	DEG(M)	DEG(M)	No	No	--	--	--	--
XLOC_029411	No	No	No	No	--	--	--	--
XLOC_029417	No	No	No	No	--	--	--	--
XLOC_029420	No	No	No	No	--	--	--	--
XLOC_029423	No	No	No	No	--	--	--	--
XLOC_029424	No	No	No	No	--	--	--	--
XLOC_029428	DEG(M)	No	No	No	XP_008913097.1	--	--	--
XLOC_029433	No	No	No	No	XP_001661570.1	--	--	--
XLOC_029435	No	No	No	No	--	--	--	--
XLOC_029438	DEG(M)	No	No	No	--	--	--	K19355
XLOC_029439	No	No	No	No	XP_001663768.1	--	--	--
XLOC_029441	No	No	No	No	--	--	--	--
XLOC_029442	No	No	No	No	CCO28865.1	--	--	--
XLOC_029444	No	No	No	No	CEL57953.1	--	--	--

Differentially expressed gene (DEG) IDs	*C. microphyllum* root and shoot-specific (>2 fold)	*C. microphyllum* root and shoot-specific (>10 fold)	ICC4958 and *C. microphyllum* root-specific (>2 fold)	*C. microphyllum* root-specific (>2 fold)	Functional Annotation			
					NCBI nr IDs	TFs	KOG	KEGG
XLOC_029445	No	No	No	No	--	--	--	--
XLOC_029447	No	No	No	No	--	--	--	--
XLOC_029449	No	No	No	No	--	--	--	--
XLOC_029458	No	No	No	No	--	--	--	--
XLOC_029460	No	No	No	No	ELU38722.1	--	--	--
XLOC_029468	DEG(M)	No	No	No	--	--	J	K02925
XLOC_029469	No	No	No	No	--	--	--	K00574
XLOC_029475	No	No	No	No	XP_003042115.1	--	--	--
XLOC_029476	No	No	No	No	ENH71614.1	--	--	--
XLOC_029487	No	No	No	No	CEL52868.1	--	--	--
XLOC_029494	No	No	No	No	--	--	EI	K00253
XLOC_029502	DEG(M)	No	No	No	CEL53255.1	--	J	--
XLOC_029503	DEG(M)	No	No	No	CEL53255.1	--	J	--
XLOC_029504	DEG(M)	DEG(M)	No	No	XP_003306712.1	--	--	--
XLOC_029506	No	No	No	No	--	ARF	--	K09919
XLOC_029509	DEG(M)	No	DEG(I+M)	DEG(M)	--	--	--	--
XLOC_029514	No	No	No	No	--	--	--	K08070
XLOC_029518	DEG(M)	DEG(M)	DEG(I+M)	DEG(M)	--	--	--	--
XLOC_029528	DEG(M)	No	No	No	--	--	--	--
XLOC_029530	No	No	No	No	--	--	--	--
XLOC_029544	No	No	No	No	EGU81016.1	--	--	--
XLOC_029550	No	No	No	No	--	--	--	--
XLOC_029552	No	No	No	No	CEL60495.1	--	S	--
XLOC_029557	No	No	No	No	--	--	--	--
XLOC_029563	No	No	No	No	XP_003053796.1	--	--	--
XLOC_029564	No	No	No	No	--	--	--	--
XLOC_029565	No	No	No	No	--	--	--	--
XLOC_029567	DEG(M)	DEG(M)	No	No	XP_003049840.1	--	--	--
XLOC_029568	No	No	No	No	EUC54042.1	--	--	--
XLOC_029573	No	No	No	No	--	--	--	--
XLOC_029574	No	No	No	No	XP_009515545.1	--	--	--
XLOC_029575	No	No	No	No	EWZ78107.1	--	--	--
XLOC_029576	No	No	No	No	XP_002524891.1	--	--	--
XLOC_029580	No	No	No	No	--	--	--	--
XLOC_029585	No	No	No	No	--	--	--	--
XLOC_029586	No	No	DEG(I+M)	No	--	--	--	--
XLOC_029587	No	No	No	No	--	--	--	--
XLOC_029588	No	No	No	No	EWZ84125.1	--	--	--
XLOC_029589	No	No	No	No	--	--	--	--
XLOC_029594	No	No	No	No	KDN45640.1	--	--	--

Differentially expressed gene (DEG) IDs	*C. microphyllum* root and shoot-specific (>2 fold)	*C. microphyllum* root and shoot-specific (>10 fold)	ICC4958 and *C. microphyllum* root-specific (>2 fold)	*C. microphyllum* root-specific (>2 fold)	Functional Annotation			
					NCBI nr IDs	TFs	KOG	KEGG
XLOC_029597	No	No	No	No	XP_004501619.1	ZF-HD	--	--
XLOC_029601	DEG(M)	No	No	No	--	--	--	--
XLOC_029606	No	No	No	No	--	--	--	--
XLOC_029612	DEG(M)	No	DEG(I+M)	DEG(M)	EUC63224.1	--	--	--
XLOC_029613	No	No	No	No	--	--	--	--
XLOC_029620	DEG(M)	DEG(M)	DEG(I+M)	DEG(M)	XP_008895518.1	--	--	--
XLOC_029628	No	No	No	No	--	--	--	--
XLOC_029649	DEG(M)	DEG(M)	No	No	--	--	--	--
XLOC_029651	DEG(M)	No	No	No	XP_009252480.1	--	J	--
XLOC_029654	DEG(M)	No	No	No	--	--	--	--
XLOC_029655	DEG(M)	No	DEG(I+M)	DEG(M)	--	--	--	--
XLOC_029656	DEG(M)	No	No	No	EUC63230.1	--	--	--
XLOC_029664	No	No	No	No	--	--	--	--
XLOC_029666	No	No	No	No	--	--	--	--
XLOC_029667	No	No	No	No	--	--	--	--
XLOC_029668	No	No	No	No	--	--	--	K02136
XLOC_029669	No	No	No	No	XP_008893637.1	--	--	K02136
XLOC_029684	No	No	DEG(I+M)	No	XP_004496690.1	--	M	--
XLOC_029708	No	No	No	No	--	--	--	--
XLOC_029712	No	No	No	No	--	--	--	--
XLOC_029713	No	No	No	No	--	--	--	--
XLOC_029714	DEG(M)	No	No	No	--	--	--	--
XLOC_029715	No	No	No	No	NP_001156433.1	--	--	K11279
XLOC_029716	No	No	No	No	XP_008889759.1	--	--	--
XLOC_029721	No	No	No	No	ETI43732.1	--	--	--
XLOC_029725	No	No	No	No	XP_008187020.1	--	--	K00432
XLOC_029726	No	No	No	No	XP_008556044.1	--	CE	--
XLOC_029727	DEG(M)	No	No	No	CCO33810.1	--	--	--
XLOC_029729	DEG(M)	No	No	No	--	--	--	--
XLOC_029730	DEG(M)	No	No	No	CEL57711.1	--	J	--
XLOC_029731	DEG(M)	DEG(M)	DEG(I+M)	DEG(M)	CEL57711.1	--	J	--
XLOC_029733	DEG(M)	No	No	No	ELU37344.1	--	J	--
XLOC_029734	DEG(M)	No	No	No	EUC56301.1	--	J	--
XLOC_029739	DEG(M)	DEG(M)	DEG(I+M)	DEG(M)	CCO26109.1	--	J	--
XLOC_029740	DEG(M)	No	No	No	ELU44646.1	--	J	K02978
XLOC_029742	DEG(M)	No	No	No	--	--	--	--
XLOC_029745	DEG(M)	No	No	No	EUC63296.1	--	J	--
XLOC_029746	DEG(M)	No	No	No	EUC63296.1	--	J	--
XLOC_029774	DEG(M)	No	DEG(I+M)	DEG(M)	KEP47514.1	--	--	--
XLOC_029775	DEG(M)	DEG(M)	DEG(I+M)	DEG(M)	KEP47514.1	--	--	--

Differentially expressed gene (DEG) IDs	*C. microphyllum* root and shoot-specific (>2 fold)	*C. microphyllum* root and shoot-specific (>10 fold)	ICC4958 and *C. microphyllum* root-specific (>2 fold)	*C. microphyllum* root-specific (>2 fold)	Functional Annotation			
					NCBI nr IDs	TFs	KOG	KEGG
XLOC_029785	No	No	No	No	--	--	--	--
XLOC_029808	No	No	DEG(I+M)	No	--	--	U	--
XLOC_029814	DEG(M)	No	No	No	EUC66846.1	--	J	K02953
XLOC_029815	No	No	No	No	EUC66846.1	--	J	--
XLOC_029834	No	No	No	No	XP_003048386.1	--	--	--
XLOC_029841	No	No	No	No	--	--	--	K03386
XLOC_029842	No	No	No	No	--	--	--	--
XLOC_029851	DEG(M)	No	No	No	KEP55133.1	--	G	K00700
XLOC_029853	No	No	No	No	--	--	O	--
XLOC_029856	DEG(M)	No	No	No	ABV00338.1	--	J	--
XLOC_029857	DEG(M)	No	DEG(I+M)	DEG(M)	XP_009517393.1	--	J	--
XLOC_029858	DEG(M)	DEG(M)	No	No	--	--	R	K01183
XLOC_029860	No	No	No	No	--	--	S	--
XLOC_029869	No	No	No	No	--	--	--	--
XLOC_029871	No	No	No	No	XP_008896265.1	--	--	--
XLOC_029875	No	No	No	No	--	--	--	--
XLOC_029876	DEG(M)	No	No	No	--	--	--	--
XLOC_029877	No	No	No	No	--	--	--	--
XLOC_029878	No	No	No	No	--	--	--	K03671
XLOC_029883	No	No	No	No	--	--	--	--
XLOC_029888	No	No	No	No	--	--	--	--
XLOC_029889	No	No	No	No	ETL33540.1	--	--	--
XLOC_029893	No	No	No	No	XP_008185729.1	--	--	--
XLOC_029894	No	No	No	No	--	--	--	--
XLOC_029896	No	No	No	No	--	--	--	--
XLOC_029897	No	No	No	No	KDN50634.1	--	--	--
XLOC_029899	No	No	No	No	XP_008904719.1	--	O	--
XLOC_029904	No	No	No	No	--	--	--	--
XLOC_029910	No	No	No	No	EUC61670.1	--	--	--
XLOC_029913	No	No	No	No	--	--	--	--
XLOC_029917	No	No	No	No	--	--	--	--
XLOC_029926	No	No	No	No	KFM82180.1	--	U	K11826
XLOC_029929	No	No	No	No	--	--	--	K05578
XLOC_029930	No	No	No	No	--	--	--	--
XLOC_029931	DEG(M)	No	No	No	--	--	--	--
XLOC_029935	No	No	No	No	XP_004504097.1	--	--	--
XLOC_029937	No	No	No	No	--	--	--	--
XLOC_029938	No	No	No	No	--	--	--	--
XLOC_029939	No	No	No	No	XP_001944106.1	--	--	--
XLOC_029940	DEG(M)	No	No	No	EWY93906.1	--	--	--

Differentially expressed gene (DEG) IDs	*C. microphyllum* root and shoot-specific (>2 fold)	*C. microphyllum* root and shoot-specific (>10 fold)	ICC4958 and *C. microphyllum* root-specific (>2 fold)	*C. microphyllum* root-specific (>2 fold)	Functional Annotation			
					NCBI nr IDs	TFs	KOG	KEGG
XLOC_029941	No	No	No	No	--	--	--	--
XLOC_029948	No	No	No	No	XP_012574490.1	--	--	--
XLOC_029949	No	No	No	No	--	--	--	--
XLOC_029951	No	No	No	No	--	--	--	--
XLOC_029952	No	No	No	No	--	--	--	--
XLOC_029953	No	No	No	No	--	--	--	--
XLOC_029956	No	No	No	No	ETI42221.1	--	--	--
XLOC_029959	No	No	No	No	--	--	--	--
XLOC_029963	DEG(M)	No	No	No	--	--	--	--
XLOC_029966	No	No	No	No	--	--	--	--
XLOC_029967	No	No	No	No	--	--	--	--
XLOC_029968	DEG(M)	DEG(M)	DEG(I+M)	DEG(M)	XP_012574440.1	--	--	--
XLOC_029970	No	No	No	No	CCO29438.1	--	--	--
XLOC_029972	No	No	No	No	KFM56943.1	--	O	--
XLOC_029975	No	No	No	No	--	--	--	--
XLOC_029976	No	No	No	No	EUC63768.1	--	--	--
XLOC_029978	No	No	No	No	--	--	F	--
XLOC_029981	No	No	No	No	--	--	--	--
XLOC_029982	No	No	No	No	XP_008309434.1	--	G	--
XLOC_029986	No	No	No	No	XP_001958373.1	--	K	--
XLOC_029987	DEG(M)	No	No	No	--	--	--	--
XLOC_029988	No	No	No	No	--	--	--	--
XLOC_029992	No	No	No	No	--	--	T	--
XLOC_029994	No	No	No	No	--	--	--	--
XLOC_029997	No	No	No	No	CCO30585.1	--	--	--
XLOC_030004	DEG(M)	No	No	No	--	--	--	K09377
XLOC_030007	DEG(M)	DEG(M)	DEG(I+M)	DEG(M)	--	--	--	K14686
XLOC_030015	DEG(M)	DEG(M)	DEG(I+M)	DEG(M)	KIL83635.1	--	--	--
XLOC_030017	DEG(M)	DEG(M)	No	No	--	--	--	--
XLOC_030018	DEG(M)	No	No	No	--	--	--	--
XLOC_030019	DEG(M)	DEG(M)	No	No	--	--	--	--
XLOC_030023	DEG(M)	DEG(M)	DEG(I+M)	DEG(M)	XP_003041182.1	--	--	--
XLOC_030024	DEG(M)	No	DEG(I+M)	DEG(M)	KDN46923.1	--	J	K02955
XLOC_030025	DEG(M)	No	DEG(I+M)	DEG(M)	KDN46923.1	--	J	K02955
XLOC_030031	No	No	No	No	XP_004486055.1	--	--	--
XLOC_030033	No	No	No	No	CEL53647.1	--	J	--
XLOC_030034	DEG(M)	No	No	No	CEL53647.1	--	J	--
XLOC_030038	DEG(M)	DEG(M)	DEG(I+M)	DEG(M)	ADV02787.1	--	--	--
XLOC_030045	DEG(M)	No	No	No	XP_001790884.1	--	--	--
XLOC_030046	No	No	No	No	CEL58831.1	--	--	--

Differentially expressed gene (DEG) IDs	***C. microphyllum* root and shoot-specific (>2 fold)**	***C. microphyllum* root and shoot-specific (>10 fold)**	**ICC4958 and *C. microphyllum* root-specific (>2 fold)**	***C. microphyllum* root-specific (>2 fold)**	**Functional Annotation**			
					**NCBI nr IDs**	**TFs**	**KOG**	**KEGG**
XLOC_030048	DEG(M)	No	No	No	--	--	--	--
XLOC_030055	No	No	No	No	--	--	--	--
XLOC_030057	No	No	No	No	ELU37808.1	--	--	--
XLOC_030058	DEG(M)	No	No	No	XP_008911228.1	--	CIQ	K03955
XLOC_030063	No	No	No	No	--	--	--	K18980
XLOC_030064	DEG(M)	No	No	No	--	--	--	--
XLOC_030066	No	No	No	No	--	B3	--	--
XLOC_030070	DEG(M)	No	No	No	XP_002641670.1	--	C	--
XLOC_030073	DEG(M)	No	No	No	--	--	--	--
XLOC_030074	DEG(M)	No	No	No	XP_007310721.1	MYB_related	J	K03263
XLOC_030086	DEG(M)	No	No	No	--	--	--	--
XLOC_030088	DEG(M)	No	No	No	--	--	--	--
XLOC_030090	No	No	No	No	--	--	--	--
XLOC_030091	No	No	No	No	--	--	--	--
XLOC_030096	DEG(M)	No	No	No	ETK81808.1	--	--	--
XLOC_030097	DEG(M)	No	No	No	--	--	J	K02943
XLOC_030102	No	No	No	No	KDN45200.1	--	K	K17616
XLOC_030103	No	No	No	No	--	--	--	--
XLOC_030104	No	No	No	No	--	--	--	--
XLOC_030106	No	No	No	No	--	--	Z	--
XLOC_030108	No	No	No	No	XP_008178627.1	--	A	K12840
XLOC_030109	No	No	No	No	--	--	--	--
XLOC_030113	No	No	No	No	--	--	--	--
XLOC_030116	No	No	DEG(I+M)	No	--	--	--	--
XLOC_030117	No	No	No	No	KDN50756.1	--	--	--
XLOC_030119	No	No	No	No	--	--	--	--
XLOC_030120	No	No	No	No	--	--	--	--
XLOC_030123	No	No	No	No	XP_008891393.1	--	--	--
XLOC_030125	No	No	No	No	XP_001945243.1	--	--	--
XLOC_030128	No	No	No	No	XP_002532104.1	--	F	K01490
XLOC_030129	No	No	No	No	XP_002510434.1	--	T	--
XLOC_030136	No	No	No	No	--	--	U	--
XLOC_030138	No	No	No	No	XP_004486746.1	--	--	--
XLOC_030139	No	No	No	No	--	--	--	--
XLOC_030140	No	No	No	No	--	--	--	--
XLOC_030143	No	No	No	No	--	--	--	K12623
XLOC_030148	No	No	No	No	--	--	--	--
XLOC_030150	No	No	No	No	--	--	--	--
XLOC_030154	No	No	No	No	--	--	--	--
XLOC_030157	DEG(M)	No	No	No	--	--	J	K02949

Differentially expressed gene (DEG) IDs	*C. microphyllum* root and shoot-specific (>2 fold)	*C. microphyllum* root and shoot-specific (>10 fold)	ICC4958 and *C. microphyllum* root-specific (>2 fold)	*C. microphyllum* root-specific (>2 fold)	Functional Annotation			
					NCBI nr IDs	TFs	KOG	KEGG
XLOC_030161	No	No	No	No	--	--	--	--
XLOC_030164	No	No	No	No	--	--	--	--
XLOC_030168	No	No	No	No	--	--	--	K03246
XLOC_030170	No	No	No	No	--	--	--	--
XLOC_030171	No	No	No	No	AHZ64886.2	--	C	--
XLOC_030173	No	No	No	No	--	--	--	--
XLOC_030176	No	No	No	No	XP_001946158.1	--	--	--
XLOC_030177	No	No	No	No	EUC61314.1	--	--	--
XLOC_030184	No	No	No	No	--	--	--	--
XLOC_030187	No	No	No	No	--	--	I	--
XLOC_030192	DEG(M)	No	No	No	--	--	--	K03676
XLOC_030193	No	No	No	No	--	--	--	--
XLOC_030195	DEG(M)	No	No	No	KIM34612.1	--	--	--
XLOC_030196	No	No	No	No	--	--	J	K02931
XLOC_030197	No	No	No	No	--	--	--	--
XLOC_030198	No	No	No	No	XP_003246129.1	--	--	--
XLOC_030203	No	No	No	No	AGE97645.1	--	--	--
XLOC_030205	No	No	No	No	--	--	--	--
XLOC_030206	DEG(M)	No	No	No	--	--	--	--
XLOC_030207	No	No	No	No	EGU88527.1	--	--	--
XLOC_030211	DEG(M)	No	No	No	AHZ64884.1	--	C	--
XLOC_030212	DEG(M)	No	No	No	AHZ64884.1	--	C	--
XLOC_030213	DEG(M)	No	DEG(I+M)	DEG(M)	--	--	--	--
XLOC_030214	DEG(M)	No	No	No	ELU41120.1	--	--	--
XLOC_030215	DEG(M)	No	No	No	KEP47124.1	--	G	--
XLOC_030218	No	No	No	No	CEL62026.1	--	--	--
XLOC_030220	No	No	No	No	--	--	--	--
XLOC_030221	No	No	No	No	--	--	--	--
XLOC_030224	DEG(M)	No	No	No	--	--	--	--
XLOC_030226	No	No	DEG(I+M)	No	--	--	R	--
XLOC_030231	No	No	No	No	--	Trihelix	--	--
XLOC_030237	DEG(M)	No	No	No	EUC60246.1	--	--	--
XLOC_030241	No	No	No	No	--	--	X	--
XLOC_030245	DEG(M)	DEG(M)	No	No	--	--	--	--
XLOC_030251	No	No	No	No	--	--	C	K02128
XLOC_030271	No	No	No	No	--	--	--	--
XLOC_030272	DEG(M)	No	No	No	--	--	--	--
XLOC_030275	DEG(M)	DEG(M)	No	No	XP_007777709.1	--	J	K02964
XLOC_030288	No	No	No	No	--	--	--	--
XLOC_030291	DEG(M)	No	No	No	--	--	--	--

Differentially expressed gene (DEG) IDs	*C. microphyllum* root and shoot-specific (>2 fold)	*C. microphyllum* root and shoot-specific (>10 fold)	ICC4958 and *C. microphyllum* root-specific (>2 fold)	*C. microphyllum* root-specific (>2 fold)	Functional Annotation			
					NCBI nr IDs	TFs	KOG	KEGG
XLOC_030295	DEG(M)	No	No	No	--	--	--	K02983
XLOC_030296	No	No	No	No	--	--	--	K02983
XLOC_030298	DEG(M)	DEG(M)	DEG(I+M)	DEG(M)	--	--	--	--
XLOC_030299	No	No	No	No	XP_002521892.1	--	E	--
XLOC_030300	No	No	No	No	XP_011024937.1	GeBP	E	--
XLOC_030307	No	No	No	No	--	--	--	--
XLOC_030316	DEG(M)	No	No	No	EUC55511.1	--	--	--
XLOC_030318	DEG(M)	No	No	No	--	--	J	K02984
XLOC_030326	DEG(M)	No	No	No	--	--	--	--
XLOC_030339	No	No	No	No	EUC54647.1	--	O	--
XLOC_030355	DEG(M)	No	No	No	--	--	--	--
XLOC_030356	No	No	No	No	XP_006219889.1	--	C	--
XLOC_030357	No	No	No	No	CCO36380.1	--	--	--
XLOC_030359	No	No	No	No	CCO36354.1	--	--	--
XLOC_030360	DEG(M)	DEG(M)	DEG(I+M)	DEG(M)	XP_003625275.1	--	--	K00430
XLOC_030361	No	No	No	No	XP_009515136.1	--	--	K03028
XLOC_030362	No	No	No	No	--	--	--	--
XLOC_030363	DEG(M)	No	No	No	--	--	--	--
XLOC_030365	DEG(M)	No	No	No	--	--	--	--
XLOC_030366	No	No	No	No	--	--	--	--
XLOC_030367	No	No	No	No	--	--	--	K01698
XLOC_030369	No	No	No	No	--	--	--	K11252
XLOC_030371	No	No	No	No	XP_001650993.1	--	O	K03178
XLOC_030377	DEG(M)	DEG(M)	DEG(I+M)	DEG(M)	--	--	--	--
XLOC_030378	DEG(M)	No	DEG(I+M)	DEG(M)	ACN31557.1	--	--	--
XLOC_030380	No	No	No	No	--	--	--	K00876
XLOC_030386	No	No	No	No	XP_003045371.1	--	--	--
XLOC_030389	No	No	No	No	--	--	--	--
XLOC_030390	DEG(M)	No	No	No	--	--	--	--
XLOC_030392	No	No	No	No	--	--	--	K02979
XLOC_030393	No	No	No	No	--	--	--	K02979
XLOC_030395	DEG(M)	No	No	No	--	--	--	--
XLOC_030397	DEG(M)	No	No	No	CEL55417.1	--	--	--
XLOC_030399	No	No	No	No	XP_001654344.1	--	J	K03259
XLOC_030402	DEG(M)	DEG(M)	DEG(I+M)	DEG(M)	--	--	--	--
XLOC_030403	DEG(M)	No	DEG(I+M)	DEG(M)	XP_002519194.1	--	--	--
XLOC_030410	DEG(M)	DEG(M)	No	No	KLO84941.1	--	A	--
XLOC_030415	No	No	No	No	--	--	--	--
XLOC_030430	DEG(M)	No	No	No	KEP51857.1	--	--	--
XLOC_030439	DEG(M)	DEG(M)	DEG(I+M)	DEG(M)	ABO16472.1	Dof	Z	--

Differentially expressed gene (DEG) IDs	*C. microphyllum* root and shoot-specific (>2 fold)	*C. microphyllum* root and shoot-specific (>10 fold)	ICC4958 and *C. microphyllum* root-specific (>2 fold)	*C. microphyllum* root-specific (>2 fold)	Functional Annotation			
					NCBI nr IDs	TFs	KOG	KEGG
XLOC_030447	DEG(M)	DEG(M)	DEG(I+M)	DEG(M)	XP_008873812.1	--	J	K02953
XLOC_030448	DEG(M)	No	DEG(I+M)	DEG(M)	XP_008902251.1	--	J	K02953
XLOC_030451	DEG(M)	No	No	No	CEL58507.1	--	--	--
XLOC_030452	DEG(M)	No	No	No	KDN37303.1	--	--	--
XLOC_030453	No	No	DEG(I+M)	No	--	--	--	--
XLOC_030454	No	No	No	No	ELU42295.1	--	--	--
XLOC_030458	DEG(M)	DEG(M)	DEG(I+M)	DEG(M)	AES81965.2	--	--	--
XLOC_030461	No	No	No	No	--	--	--	--
XLOC_030464	No	No	No	No	--	--	J	--
XLOC_030466	No	No	DEG(I+M)	No	--	--	U	--
XLOC_030470	No	No	No	No	--	--	G	K00927
XLOC_030472	DEG(M)	No	DEG(I+M)	DEG(M)	AHY22512.1	--	J	--
XLOC_030477	No	No	No	No	XP_009520039.1	--	--	K03456
XLOC_030483	No	No	No	No	ABQ41406.1	--	J	--
XLOC_030484	No	No	No	No	--	--	J	--
XLOC_030485	No	No	No	No	--	--	--	--
XLOC_030489	No	No	No	No	AKM28421.1	--	C	K01648
XLOC_030492	No	No	No	No	XP_001945243.1	--	I	--
XLOC_030493	No	No	No	No	--	--	--	--
XLOC_030495	No	No	DEG(I+M)	No	--	--	--	--
XLOC_030496	No	No	No	No	EUC64186.1	--	--	K08343
XLOC_030498	No	No	No	No	--	--	--	--
XLOC_030500	No	No	No	No	--	--	--	--
XLOC_030503	No	No	No	No	--	--	--	--
XLOC_030506	No	No	No	No	CCO33549.1	--	--	--
XLOC_030507	No	No	No	No	--	--	--	--
XLOC_030510	No	No	No	No	XP_001658309.1	--	J	K01868
XLOC_030511	No	No	No	No	XP_001951748.2	--	--	--
XLOC_030512	DEG(M)	DEG(M)	No	No	KLP08035.1	--	--	--
XLOC_030513	No	No	No	No	EUC54552.1	--	--	--
XLOC_030514	DEG(M)	No	No	No	CCO30076.1	--	--	--
XLOC_030515	DEG(M)	DEG(M)	No	No	EWY92288.1	--	--	--
XLOC_030516	DEG(M)	No	No	No	--	--	J	K02984
XLOC_030518	No	No	No	No	--	--	--	--
XLOC_030522	DEG(M)	No	DEG(I+M)	DEG(M)	XP_008896172.1	--	--	--
XLOC_030524	No	No	No	No	--	--	--	--
XLOC_030527	No	No	No	No	--	--	--	--
XLOC_030532	No	No	No	No	ELU44881.1	--	--	--
XLOC_030536	No	No	No	No	CEL62978.1	--	--	--
XLOC_030540	DEG(M)	No	No	No	--	--	--	--

Differentially expressed gene (DEG) IDs	*C. microphyllum* root and shoot-specific (>2 fold)	*C. microphyllum* root and shoot-specific (>10 fold)	ICC4958 and *C. microphyllum* root-specific (>2 fold)	*C. microphyllum* root-specific (>2 fold)	Functional Annotation			
					NCBI nr IDs	TFs	KOG	KEGG
XLOC_030541	No	No	No	No	XP_009527269.1	--	--	--
XLOC_030542	No	No	No	No	--	--	--	--
XLOC_030543	No	No	No	No	EUC55649.1	--	--	--
XLOC_030544	DEG(M)	No	DEG(I+M)	DEG(M)	--	--	--	--
XLOC_030547	No	No	No	No	XP_002903928.1	--	--	K03946
XLOC_030551	DEG(M)	No	No	No	KDN40122.1	--	O	--
XLOC_030555	No	No	No	No	CEL54072.1	--	--	--
XLOC_030557	No	No	No	No	--	--	J	K02966
XLOC_030559	No	No	No	No	--	--	--	--
XLOC_030560	No	No	No	No	KFM57219.1	--	O	--
XLOC_030562	No	No	No	No	CEL55739.1	--	--	--
XLOC_030566	DEG(M)	No	No	No	--	--	--	--
XLOC_030568	No	No	No	No	--	--	--	--
XLOC_030569	DEG(M)	No	No	No	--	--	--	--
XLOC_030570	No	No	No	No	XP_003243533.1	--	--	--
XLOC_030572	DEG(M)	No	No	No	EUC67357.1	--	--	--
XLOC_030573	No	No	No	No	--	--	--	--
XLOC_030576	No	No	DEG(I+M)	No	XP_004486266.1	--	VT	--
XLOC_030577	No	No	No	No	--	--	R	K11446
XLOC_030584	DEG(M)	No	DEG(I+M)	DEG(M)	--	--	--	--
XLOC_030594	No	No	No	No	XP_002898134.1	--	Q	--
XLOC_030595	DEG(M)	DEG(M)	No	No	EMT66773.1	--	--	--
XLOC_030654	No	No	DEG(I+M)	No	XP_004516353.1	--	--	--
XLOC_030655	No	No	No	No	XP_004516336.1	--	R	--
XLOC_030658	No	No	No	No	--	--	--	--
XLOC_030659	DEG(M)	No	No	No	--	--	--	--
XLOC_030660	DEG(M)	No	No	No	--	--	--	--
XLOC_030661	DEG(M)	No	No	No	--	--	--	--
XLOC_030662	No	No	No	No	--	--	--	--
XLOC_030704	No	No	DEG(I+M)	No	XP_004496081.1	ERF	--	--
XLOC_030710	DEG(M)	No	No	No	XP_012572286.1	--	--	--
XLOC_030765	No	No	No	No	XP_012568413.1	--	--	--
XLOC_030776	No	No	No	No	--	--	--	--
XLOC_030778	DEG(M)	No	DEG(I+M)	DEG(M)	XP_004516745.1	--	--	--
XLOC_030811	DEG(M)	No	No	No	--	--	--	K02183
XLOC_030814	No	No	DEG(I+M)	No	XP_004491817.1	--	--	K02183
XLOC_030828	DEG(M)	No	DEG(I+M)	DEG(M)	--	--	--	--
XLOC_030850	DEG(M)	DEG(M)	No	No	--	--	--	--
XLOC_030856	No	No	DEG(I+M)	No	--	C2H2	Q	--
XLOC_030864	No	No	DEG(I+M)	No	XP_004496544.1	--	Q	K19329

Differentially expressed gene (DEG) IDs	*C. microphyllum* root and shoot-specific (>2 fold)	*C. microphyllum* root and shoot-specific (>10 fold)	ICC4958 and *C. microphyllum* root-specific (>2 fold)	*C. microphyllum* root-specific (>2 fold)	Functional Annotation			
					NCBI nr IDs	TFs	KOG	KEGG
XLOC_030877	DEG(M)	No	No	No	--	--	--	--
XLOC_030892	DEG(M)	DEG(M)	DEG(I+M)	DEG(M)	--	--	R	--
XLOC_031024	No	No	No	No	CEL63147.1	--	--	--
XLOC_031026	DEG(M)	DEG(M)	No	No	KDN45713.1	--	--	--
XLOC_031027	DEG(M)	No	No	No	EUC61114.1	--	--	--
XLOC_031081	No	No	DEG(I+M)	No	--	--	--	--
XLOC_031107	DEG(M)	No	No	No	--	--	--	--
XLOC_031114	No	No	No	No	--	--	Q	--
XLOC_031195	No	No	No	No	XP_004505201.1	--	--	--
XLOC_031220	No	No	No	No	--	--	--	K01194
XLOC_031222	No	No	No	No	--	--	--	--
XLOC_031225	DEG(M)	No	No	No	XP_004488754.1	--	--	--
XLOC_031227	DEG(M)	DEG(M)	DEG(I+M)	DEG(M)	XP_004488753.1	--	QV	--
XLOC_031230	DEG(M)	DEG(M)	No	No	KNB02228.1	--	--	--
XLOC_031231	DEG(M)	No	No	No	--	--	--	--
XLOC_031235	DEG(M)	No	No	No	ELU41243.1	--	--	--
XLOC_031236	No	No	No	No	--	--	--	--
XLOC_031237	No	No	No	No	XP_001652914.1	--	--	--
XLOC_031238	DEG(M)	No	No	No	--	--	--	--
XLOC_031245	DEG(M)	No	No	No	EUC54856.1	--	--	--
XLOC_031247	No	No	No	No	XP_004356664.1	--	--	--
XLOC_031248	No	No	No	No	CEL62377.1	--	--	--
XLOC_031254	DEG(M)	No	No	No	EUC55718.1	--	J	K02917
XLOC_031258	DEG(M)	No	No	No	KDN35765.1	--	--	--
XLOC_031259	No	No	No	No	EUC65071.1	--	--	--
XLOC_031263	DEG(M)	No	No	No	KEP50528.1	--	--	--
XLOC_031271	DEG(M)	No	No	No	--	--	O	K08900
XLOC_031279	DEG(M)	No	No	No	ETL79743.1	--	--	--
XLOC_031284	No	No	No	No	--	--	--	K12338
XLOC_031286	DEG(M)	DEG(M)	No	No	XP_003054439.1	--	J	--
XLOC_031287	No	No	No	No	--	--	--	--
XLOC_031291	No	No	No	No	--	--	--	--
XLOC_031297	DEG(M)	DEG(M)	DEG(I+M)	DEG(M)	--	--	--	--
XLOC_031298	DEG(M)	DEG(M)	DEG(I+M)	DEG(M)	--	--	--	--
XLOC_031299	No	No	No	No	--	--	--	--
XLOC_031300	No	No	No	No	--	--	--	--
XLOC_031301	No	No	No	No	--	--	--	--
XLOC_031302	No	No	No	No	--	--	--	--
XLOC_031303	DEG(M)	No	No	No	--	--	--	--
XLOC_031304	DEG(M)	No	No	No	--	--	--	--

Differentially expressed gene (DEG) IDs	*C. microphyllum* root and shoot-specific (>2 fold)	*C. microphyllum* root and shoot-specific (>10 fold)	ICC4958 and *C. microphyllum* root-specific (>2 fold)	*C. microphyllum* root-specific (>2 fold)	Functional Annotation			
					NCBI nr IDs	TFs	KOG	KEGG
XLOC_031306	DEG(M)	No	DEG(I+M)	DEG(M)	--	--	--	--
XLOC_031308	DEG(M)	No	No	No	--	--	--	--
XLOC_031309	DEG(M)	No	No	No	--	--	--	--
XLOC_031311	DEG(M)	No	No	No	ADD59803.1	YABBY	OR	--
XLOC_031316	DEG(M)	DEG(M)	No	No	XP_003049657.1	--	J	--
XLOC_031324	No	No	No	No	AFM93772.1	--	Z	--
XLOC_031325	No	No	No	No	AFM93772.1	--	Z	--
XLOC_031330	No	No	No	No	--	--	--	--
XLOC_031338	DEG(M)	No	No	No	CEL52278.1	--	--	--
XLOC_031345	DEG(M)	DEG(M)	DEG(I+M)	DEG(M)	--	--	--	--
XLOC_031347	No	No	No	No	XP_008902768.1	--	U	K07936
XLOC_031354	No	No	DEG(I+M)	No	XP_004489380.1	--	--	K10712
XLOC_031358	DEG(M)	No	No	No	--	--	--	--
XLOC_031361	DEG(M)	DEG(M)	DEG(I+M)	DEG(M)	AEM64622.1	--	Z	--
XLOC_031362	DEG(M)	DEG(M)	DEG(I+M)	DEG(M)	XP_012747850.1	--	Z	--
XLOC_031365	DEG(M)	No	No	No	--	--	--	--
XLOC_031366	No	No	No	No	--	--	--	K03937
XLOC_031367	No	No	No	No	--	--	--	--
XLOC_031370	No	No	No	No	--	--	--	--
XLOC_031371	No	No	No	No	XP_002520502.1	--	--	--
XLOC_031372	No	No	No	No	--	B3	O	--
XLOC_031377	No	No	DEG(I+M)	No	--	--	--	K04124
XLOC_031379	No	No	No	No	--	--	--	--
XLOC_031380	No	No	No	No	CEL52730.1	--	--	--
XLOC_031381	No	No	No	No	--	--	--	--
XLOC_031384	No	No	No	No	--	--	--	--
XLOC_031385	No	No	No	No	XP_009529532.1	--	--	--
XLOC_031387	DEG(M)	DEG(M)	No	No	--	--	--	--
XLOC_031391	No	No	No	No	XP_001834214.1	MYB_related	J	--
XLOC_031393	No	No	No	No	XP_012069783.1	--	R	--
XLOC_031394	No	No	No	No	XP_006631131.1	--	R	K07897
XLOC_031398	No	No	No	No	--	--	--	--
XLOC_031399	No	No	No	No	--	--	--	--
XLOC_031404	No	No	No	No	XP_003046204.1	--	--	--
XLOC_031405	No	No	No	No	--	--	--	K03687
XLOC_031411	No	No	No	No	--	--	--	--
XLOC_031412	No	No	No	No	--	--	--	--
XLOC_031414	No	No	No	No	AAV31415.1	--	O	--
XLOC_031416	No	No	No	No	KDN41088.1	--	--	--
XLOC_031419	No	No	No	No	--	--	--	--

Differentially expressed gene (DEG) IDs	*C. microphyllum* root and shoot-specific (>2 fold)	*C. microphyllum* root and shoot-specific (>10 fold)	ICC4958 and *C. microphyllum* root-specific (>2 fold)	*C. microphyllum* root-specific (>2 fold)	Functional Annotation			
					NCBI nr IDs	TFs	KOG	KEGG
XLOC_031421	No	No	No	No	--	--	--	--
XLOC_031424	No	No	No	No	EUC62638.1	--	--	--
XLOC_031430	No	No	No	No	XP_008185729.1	--	--	--
XLOC_031431	No	No	No	No	ETI45522.1	--	O	--
XLOC_031436	DEG(M)	No	No	No	--	--	--	--
XLOC_031440	No	No	No	No	--	--	--	--
XLOC_031441	No	No	No	No	--	--	--	--
XLOC_031445	No	No	No	No	--	--	--	--
XLOC_031448	DEG(M)	DEG(M)	No	No	EXM03021.1	--	--	--
XLOC_031455	DEG(M)	No	No	No	KEP54279.1	--	--	--
XLOC_031457	No	No	No	No	--	--	RT	K14488
XLOC_031468	DEG(M)	DEG(M)	DEG(I+M)	DEG(M)	--	--	--	--
XLOC_031469	DEG(M)	DEG(M)	DEG(I+M)	DEG(M)	--	--	--	--
XLOC_031473	DEG(M)	No	No	No	--	--	--	--
XLOC_031477	DEG(M)	No	No	No	ETI33881.1	--	--	--
XLOC_031481	No	No	No	No	KNG50966.1	--	--	--
XLOC_031483	No	No	No	No	--	Trihelix	--	--
XLOC_031485	No	No	No	No	--	--	--	--
XLOC_031486	No	No	No	No	--	--	--	--
XLOC_031488	No	No	No	No	EUC62012.1	--	--	--
XLOC_031489	No	No	No	No	CCO28795.1	--	--	K05349
XLOC_031492	DEG(M)	No	No	No	--	--	--	--
XLOC_031493	DEG(M)	No	No	No	--	--	--	--
XLOC_031499	No	No	No	No	--	--	--	--
XLOC_031501	No	No	No	No	--	--	--	--
XLOC_031506	DEG(M)	No	No	No	--	--	--	--
XLOC_031509	DEG(M)	No	No	No	CCO33240.1	--	--	K03233
XLOC_031527	DEG(M)	No	No	No	XP_008896187.1	--	--	K04564
XLOC_031528	DEG(M)	No	No	No	XP_008896187.1	--	--	K04564
XLOC_031531	No	No	No	No	XP_008914205.1	--	C	K00162
XLOC_031532	DEG(M)	No	No	No	--	--	--	--
XLOC_031533	No	No	No	No	--	--	--	--
XLOC_031535	DEG(M)	No	No	No	CEL54684.1	--	--	--
XLOC_031537	No	No	No	No	--	--	--	--
XLOC_031551	DEG(M)	DEG(M)	No	No	--	--	--	--
XLOC_031552	DEG(M)	DEG(M)	No	No	AAO63551.1	--	--	--
XLOC_031557	DEG(M)	No	No	No	EUC59093.1	--	--	--
XLOC_031558	DEG(M)	No	No	No	XP_003049980.1	--	--	--
XLOC_031562	DEG(M)	No	No	No	--	--	--	--
XLOC_031565	No	No	No	No	--	--	--	--

Differentially expressed gene (DEG) IDs	*C. microphyllum* root and shoot-specific (>2 fold)	*C. microphyllum* root and shoot-specific (>10 fold)	ICC4958 and *C. microphyllum* root-specific (>2 fold)	*C. microphyllum* root-specific (>2 fold)	Functional Annotation			
					NCBI nr IDs	TFs	KOG	KEGG
XLOC_031566	No	No	No	No	--	--	--	--
XLOC_031567	DEG(M)	No	No	No	XP_008917255.1	--	H	--
XLOC_031568	DEG(M)	No	No	No	XP_009526921.1	--	H	K00789
XLOC_031571	DEG(M)	No	No	No	NP_001119645.2	--	C	K02133
XLOC_031572	No	No	No	No	--	--	--	--
XLOC_031574	No	No	No	No	--	--	--	--
XLOC_031577	No	No	No	No	--	--	--	K03671
XLOC_031579	No	No	No	No	EUC57362.1	--	O	K10579
XLOC_031580	No	No	No	No	--	--	--	--
XLOC_031583	No	No	No	No	EUC57306.1	--	--	--
XLOC_031589	No	No	No	No	XP_008889726.1	--	--	--
XLOC_031600	No	No	DEG(I+M)	No	XP_004500649.1	--	--	K13448
XLOC_031601	No	No	No	No	XP_006372416.1	GeBP	E	K01580
XLOC_031602	No	No	No	No	--	--	--	--
XLOC_031605	No	No	No	No	CEL60722.1	--	--	--
XLOC_031610	No	No	No	No	--	--	--	--
XLOC_031613	No	No	No	No	--	--	--	--
XLOC_031615	No	No	No	No	KFM77036.1	--	R	--
XLOC_031616	No	No	No	No	KFM67634.1	--	O	K03038
XLOC_031617	No	No	No	No	XP_008899358.1	--	--	--
XLOC_031618	No	No	No	No	--	--	--	--
XLOC_031619	No	No	No	No	CCO28795.1	--	--	--
XLOC_031621	No	No	No	No	--	--	--	--
XLOC_031622	No	No	No	No	ELU39506.1	--	--	--
XLOC_031625	No	No	No	No	--	--	--	--
XLOC_031630	No	No	No	No	--	--	--	--
XLOC_031633	No	No	No	No	--	--	--	--
XLOC_031636	DEG(M)	No	No	No	--	--	J	K02975
XLOC_031637	No	No	No	No	XP_003043332.1	--	--	--
XLOC_031638	DEG(M)	No	No	No	XP_004336884.1	--	O	--
XLOC_031642	No	No	No	No	--	--	--	--
XLOC_031651	DEG(M)	No	No	No	--	--	--	--
XLOC_031652	DEG(M)	No	No	No	--	--	--	--
XLOC_031653	DEG(M)	No	DEG(I+M)	DEG(M)	XP_008904283.1	--	J	K02987
XLOC_031657	DEG(M)	No	No	No	CCO36228.1	--	J	--
XLOC_031661	No	No	No	No	--	--	I	--
XLOC_031663	No	No	No	No	--	--	I	--
XLOC_031671	No	No	No	No	XP_004507246.1	--	--	--
XLOC_031676	No	No	No	No	EUC54809.1	--	--	--
XLOC_031677	DEG(M)	No	No	No	ELU40684.1	--	--	--

Differentially expressed gene (DEG) IDs	*C. microphyllum* root and shoot-specific (>2 fold)	*C. microphyllum* root and shoot-specific (>10 fold)	ICC4958 and *C. microphyllum* root-specific (>2 fold)	*C. microphyllum* root-specific (>2 fold)	Functional Annotation			
					NCBI nr IDs	TFs	KOG	KEGG
XLOC_031686	No	No	No	No	XP_004494327.1	--	--	--
XLOC_031694	No	No	No	No	--	--	--	--
XLOC_031695	No	No	No	No	--	--	--	--
XLOC_031697	No	No	DEG(I+M)	No	--	--	--	--
XLOC_031700	No	No	No	No	--	--	--	K02942
XLOC_031701	No	No	No	No	XP_008913891.1	--	--	--
XLOC_031706	DEG(M)	DEG(M)	No	No	XP_004492175.1	--	--	K08081
XLOC_031707	DEG(M)	DEG(M)	No	No	--	--	--	--
XLOC_031743	DEG(M)	No	DEG(I+M)	DEG(M)	--	--	--	--
XLOC_031746	No	No	No	No	--	--	--	--
XLOC_031747	No	No	No	No	NP_001280464.1	--	--	--
XLOC_031748	No	No	No	No	XP_009251888.1	--	--	--
XLOC_031749	No	No	DEG(I+M)	No	--	--	--	--
XLOC_031750	No	No	No	No	KDQ20675.1	--	E	--
XLOC_031751	DEG(M)	DEG(M)	No	No	XP_004510294.1	--	--	--
XLOC_031753	No	No	No	No	XP_011554291.1	--	O	K10577
XLOC_031754	No	No	No	No	--	--	--	--
XLOC_031755	DEG(M)	DEG(M)	DEG(I+M)	DEG(M)	--	--	--	--
XLOC_031756	DEG(M)	DEG(M)	DEG(I+M)	DEG(M)	--	--	--	--
XLOC_031757	DEG(M)	No	No	No	ETO62886.1	--	--	--
XLOC_031761	No	No	No	No	--	--	--	--
XLOC_031762	No	No	No	No	--	--	--	--
XLOC_031764	No	No	No	No	--	--	--	--
XLOC_031765	No	No	No	No	XP_008906442.1	--	--	--
XLOC_031766	No	No	No	No	--	--	--	--
XLOC_031768	No	No	No	No	--	--	--	K03671
XLOC_031769	No	No	No	No	--	--	--	--
XLOC_031770	No	No	No	No	--	--	--	K10703
XLOC_031771	No	No	No	No	EUC64475.1	--	--	--
XLOC_031772	No	No	No	No	CCO30956.1	--	--	--
XLOC_031777	DEG(M)	No	No	No	XP_002533343.1	--	J	--
XLOC_031784	No	No	DEG(I+M)	No	--	--	--	--
XLOC_031787	No	No	No	No	--	--	--	--
XLOC_031788	No	No	No	No	--	--	--	--
XLOC_031790	No	No	No	No	XP_003048431.1	--	--	--
XLOC_031791	DEG(M)	DEG(M)	No	No	--	--	--	--
XLOC_031794	No	No	No	No	--	--	--	--
XLOC_031795	No	No	No	No	--	--	--	--
XLOC_031800	No	No	No	No	--	--	--	--
XLOC_031806	No	No	No	No	--	--	--	--

Differentially expressed gene (DEG) IDs	*C. microphyllum* root and shoot-specific (>2 fold)	*C. microphyllum* root and shoot-specific (>10 fold)	ICC4958 and *C. microphyllum* root-specific (>2 fold)	*C. microphyllum* root-specific (>2 fold)	Functional Annotation			
					NCBI nr IDs	TFs	KOG	KEGG
XLOC_031808	No	No	No	No	--	--	--	--
XLOC_031809	No	No	No	No	CEL59403.1	--	--	--
XLOC_031812	No	No	No	No	EUC66894.1	--	--	--
XLOC_031814	No	No	No	No	XP_009530416.1	--	--	K01893
XLOC_031818	No	No	No	No	--	--	--	--
XLOC_031821	No	No	No	No	--	--	--	--
XLOC_031824	DEG(M)	No	DEG(I+M)	DEG(M)	--	--	--	--
XLOC_031826	No	No	No	No	CCO31830.1	--	P	--
XLOC_031830	No	No	No	No	XP_002511399.1	--	--	K00099
XLOC_031833	No	No	No	No	KDN51031.1	--	--	--
XLOC_031837	DEG(M)	No	No	No	--	--	--	--
XLOC_031842	No	No	No	No	--	--	--	--
XLOC_031844	No	No	No	No	--	--	--	--
XLOC_031845	No	No	No	No	--	--	--	--
XLOC_031846	No	No	No	No	XP_003054509.1	--	--	--
XLOC_031848	DEG(M)	No	No	No	--	--	--	--
XLOC_031850	No	No	No	No	--	--	--	--
XLOC_031851	No	No	No	No	XP_003039978.1	--	--	K17800
XLOC_031852	No	No	No	No	--	--	--	--
XLOC_031853	No	No	No	No	BAJ92973.1	--	--	--
XLOC_031854	No	No	No	No	ELU39298.1	--	--	--
XLOC_031855	DEG(M)	DEG(M)	DEG(I+M)	DEG(M)	--	--	--	--
XLOC_031857	No	No	No	No	--	NAC	--	--
XLOC_031858	No	No	No	No	--	--	O	--
XLOC_031859	No	No	No	No	XP_008912119.1	MYB_related	G	K00164
XLOC_031862	No	No	No	No	--	--	--	--
XLOC_031863	DEG(M)	No	No	No	XP_001647949.1	--	J	--
XLOC_031864	No	No	No	No	--	--	O	--
XLOC_031865	No	No	No	No	EUC61103.1	--	--	--
XLOC_031867	No	No	No	No	--	--	C	K01900
XLOC_031868	No	No	No	No	--	--	--	--
XLOC_031869	No	No	No	No	XP_012201891.1	--	GT	--
XLOC_031870	No	No	No	No	--	--	--	--
XLOC_031871	No	No	No	No	--	--	--	--
XLOC_031872	No	No	No	No	ETI42464.1	--	--	--
XLOC_031873	No	No	No	No	--	--	--	--
XLOC_031874	No	No	No	No	CEL57191.1	--	AD	K12859
XLOC_031877	No	No	No	No	CEL61820.1	--	--	--
XLOC_031884	No	No	No	No	--	--	--	--
XLOC_031886	No	No	No	No	--	--	--	--

Differentially expressed gene (DEG) IDs	*C. microphyllum* root and shoot-specific (>2 fold)	*C. microphyllum* root and shoot-specific (>10 fold)	ICC4958 and *C. microphyllum* root-specific (>2 fold)	*C. microphyllum* root-specific (>2 fold)	Functional Annotation			
					NCBI nr IDs	TFs	KOG	KEGG
XLOC_031887	No	No	No	No	--	--	--	--
XLOC_031889	No	No	No	No	KDO71374.1	--	T	--
XLOC_031891	No	No	No	No	--	--	--	--
XLOC_031892	DEG(M)	No	No	No	--	--	--	--
XLOC_031896	DEG(M)	No	No	No	EUC61047.1	--	--	--
XLOC_031897	DEG(M)	No	No	No	--	--	--	--
XLOC_031900	No	No	No	No	ABO26691.1	--	T	--
XLOC_031903	No	No	No	No	--	--	--	--
XLOC_031906	No	No	No	No	--	--	--	--
XLOC_031913	DEG(M)	DEG(M)	DEG(I+M)	DEG(M)	XP_003050314.1	--	J	--
XLOC_031915	No	No	No	No	--	--	--	--
XLOC_031918	No	No	No	No	--	--	--	--
XLOC_031919	No	No	No	No	EUC57335.1	--	--	K03671
XLOC_031920	No	No	No	No	--	--	--	--
XLOC_031923	No	No	No	No	XP_009537239.1	--	--	K03037
XLOC_031926	DEG(M)	No	No	No	AAS82856.1	--	--	--
XLOC_031927	No	No	No	No	--	--	--	--
XLOC_031928	No	No	No	No	XP_001944230.1	--	--	--
XLOC_031935	DEG(M)	No	No	No	--	--	--	--
XLOC_031936	No	No	No	No	--	--	U	--
XLOC_031938	No	No	No	No	--	--	--	--
XLOC_031939	No	No	No	No	--	--	--	--
XLOC_031941	DEG(M)	No	No	No	XP_009251442.1	--	--	K09272
XLOC_031942	DEG(M)	DEG(M)	No	No	XP_003051568.1	--	--	--
XLOC_031945	No	No	DEG(I+M)	No	--	--	--	--
XLOC_031946	No	No	No	No	KEP53685.1	--	--	--
XLOC_031947	No	No	No	No	--	--	--	--
XLOC_031949	No	No	No	No	--	--	--	--
XLOC_031950	No	No	No	No	ETL98155.1	--	--	--
XLOC_031953	No	No	No	No	--	--	--	--
XLOC_031954	No	No	No	No	--	--	--	--
XLOC_031957	No	No	No	No	XP_001944064.2	--	--	--
XLOC_031961	No	No	No	No	--	--	--	--
XLOC_031967	No	No	No	No	NP_001155372.1	--	C	--
XLOC_031968	DEG(M)	No	No	No	EGU72435.1	--	--	--
XLOC_031973	No	No	No	No	--	--	--	--
XLOC_031974	DEG(M)	DEG(M)	No	No	--	--	--	--
XLOC_031975	No	No	No	No	XP_001650791.1	--	--	K05643
XLOC_031977	DEG(M)	No	No	No	ETI57600.1	--	--	--
XLOC_031979	No	No	No	No	XP_004506308.1	--	--	K09875

Differentially expressed gene (DEG) IDs	*C. microphyllum* root and shoot-specific (>2 fold)	*C. microphyllum* root and shoot-specific (>10 fold)	ICC4958 and *C. microphyllum* root-specific (>2 fold)	*C. microphyllum* root-specific (>2 fold)	Functional Annotation			
					NCBI nr IDs	TFs	KOG	KEGG
XLOC_031981	No	No	No	No	--	--	--	K02878
XLOC_031983	DEG(M)	No	No	No	--	--	--	--
XLOC_031985	No	No	No	No	XP_001946593.1	--	--	--
XLOC_031987	No	No	No	No	XP_012085386.1	--	T	K11866
XLOC_031990	No	No	No	No	--	--	--	--
XLOC_031994	No	No	No	No	XP_003047840.1	--	--	--
XLOC_031996	DEG(M)	No	No	No	XP_004507021.1	--	--	--
XLOC_032000	DEG(M)	No	No	No	NP_001155786.1	--	--	--
XLOC_032001	No	No	No	No	--	--	--	--
XLOC_032002	DEG(M)	No	No	No	--	--	--	--
XLOC_032003	No	No	No	No	--	--	--	K09272
XLOC_032007	No	No	No	No	--	--	--	--
XLOC_032008	DEG(M)	No	No	No	ETO60868.1	--	G	--
XLOC_032009	No	No	No	No	KFM76437.1	--	O	K02726
XLOC_032010	DEG(M)	No	No	No	XP_002528222.1	--	M	--
XLOC_032011	No	No	No	No	--	--	--	--
XLOC_032012	DEG(M)	No	No	No	--	--	--	K02905
XLOC_032013	DEG(M)	No	No	No	--	--	J	--
XLOC_032014	No	No	No	No	CEL57784.1	--	--	--
XLOC_032020	No	No	No	No	KDN36527.1	--	--	--
XLOC_032021	No	No	No	No	--	--	--	--
XLOC_032025	No	No	No	No	CCO34780.1	--	O	--
XLOC_032026	No	No	No	No	KDN49426.1	--	--	--
XLOC_032031	No	No	No	No	--	--	--	--
XLOC_032056	No	No	No	No	CCO30288.1	--	G	--
XLOC_032059	DEG(M)	No	No	No	XP_008902145.1	--	--	K15730
XLOC_032061	No	No	No	No	EUC57025.1	--	--	--
XLOC_032062	DEG(M)	No	No	No	KEP47630.1	--	J	--
XLOC_032066	No	No	No	No	--	--	--	--
XLOC_032068	DEG(M)	No	No	No	--	--	--	--
XLOC_032069	DEG(M)	No	No	No	--	--	--	--
XLOC_032075	No	No	No	No	CEL60246.1	--	--	--
XLOC_032076	No	No	No	No	CEL60246.1	--	--	--
XLOC_032078	No	No	No	No	EUC62664.1	--	--	--
XLOC_032081	No	No	No	No	YP_002149764.1	--	--	K03040
XLOC_032084	No	No	No	No	--	--	--	--
XLOC_032086	No	No	No	No	--	--	--	K14950
XLOC_032087	No	No	No	No	--	--	--	--
XLOC_032089	No	No	No	No	ETP40548.1	--	C	K01679
XLOC_032094	DEG(M)	No	No	No	--	--	--	--

Differentially expressed gene (DEG) IDs	*C. microphyllum* root and shoot-specific (>2 fold)	*C. microphyllum* root and shoot-specific (>10 fold)	ICC4958 and *C. microphyllum* root-specific (>2 fold)	*C. microphyllum* root-specific (>2 fold)	Functional Annotation			
					NCBI nr IDs	TFs	KOG	KEGG
XLOC_032096	No	No	No	No	--	--	--	--
XLOC_032100	No	No	No	No	--	--	--	--
XLOC_032103	DEG(M)	No	No	No	CEL57830.1	--	--	--
XLOC_032106	No	No	No	No	EUC64043.1	--	--	K00434
XLOC_032111	No	No	No	No	CEL55231.1	--	--	K03320
XLOC_032115	DEG(M)	No	No	No	--	--	J	--
XLOC_032118	DEG(M)	DEG(M)	DEG(I+M)	DEG(M)	EUC58676.1	--	J	--
XLOC_032120	No	No	No	No	--	--	--	--
XLOC_032122	DEG(M)	No	No	No	--	--	--	--
XLOC_032125	No	No	No	No	--	--	J	K02933
XLOC_032129	No	No	No	No	KCW54765.1	--	TDK	K03097
XLOC_032132	No	No	No	No	XP_008910366.1	--	--	--
XLOC_032133	DEG(M)	No	DEG(I+M)	DEG(M)	--	--	--	--
XLOC_032134	No	No	No	No	NP_001153813.1	--	--	--
XLOC_032135	No	No	No	No	--	--	--	--
XLOC_032136	No	No	No	No	XP_004356682.1	--	J	--
XLOC_032137	DEG(M)	DEG(M)	No	No	--	--	--	--
XLOC_032139	No	No	No	No	--	--	--	--
XLOC_032140	DEG(M)	DEG(M)	DEG(I+M)	DEG(M)	--	--	--	--
XLOC_032141	No	No	No	No	AGC93286.1	MYB_related	O	--
XLOC_032142	No	No	No	No	--	--	--	K13379
XLOC_032144	No	No	No	No	CCO33850.1	--	--	--
XLOC_032147	No	No	No	No	--	--	--	--
XLOC_032148	No	No	No	No	--	--	--	--
XLOC_032149	No	No	No	No	--	--	--	--
XLOC_032150	DEG(M)	No	No	No	--	--	--	--
XLOC_032152	No	No	No	No	AAM93256.1	--	O	--
XLOC_032153	No	No	No	No	--	--	Q	--
XLOC_032154	DEG(M)	No	No	No	--	--	--	--
XLOC_032159	No	No	No	No	XP_002532324.1	--	I	K20174
XLOC_032160	No	No	No	No	--	--	--	--
XLOC_032161	No	No	No	No	XP_008898610.1	--	--	--
XLOC_032165	No	No	No	No	--	--	--	--
XLOC_032167	DEG(M)	No	No	No	--	--	--	--
XLOC_032168	No	No	No	No	EXA00436.1	--	--	--
XLOC_032170	DEG(M)	No	No	No	XP_002909676.1	--	--	--
XLOC_032172	No	No	No	No	--	--	--	--
XLOC_032175	DEG(M)	No	DEG(I+M)	DEG(M)	--	--	--	--
XLOC_032178	No	No	No	No	CCO30212.1	--	--	--
XLOC_032187	No	No	No	No	--	--	--	--

Differentially expressed gene (DEG) IDs	*C. microphyllum* root and shoot-specific (>2 fold)	*C. microphyllum* root and shoot-specific (>10 fold)	ICC4958 and *C. microphyllum* root-specific (>2 fold)	*C. microphyllum* root-specific (>2 fold)	Functional Annotation			
					NCBI nr IDs	TFs	KOG	KEGG
XLOC_032188	No	No	No	No	--	--	--	--
XLOC_032190	DEG(M)	No	No	No	ETP16350.1	--	--	--
XLOC_032192	No	No	No	No	--	--	--	K15188
XLOC_032194	No	No	No	No	XP_008904110.1	--	--	--
XLOC_032195	No	No	No	No	--	--	--	--
XLOC_032198	No	No	No	No	EUC53882.1	GATA	U	--
XLOC_032200	No	No	No	No	CEL59734.1	--	--	--
XLOC_032202	No	No	No	No	--	--	--	--
XLOC_032203	No	No	No	No	--	--	--	--
XLOC_032204	No	No	No	No	--	--	--	--
XLOC_032205	No	No	No	No	AAU84928.1	--	--	K02137
XLOC_032206	DEG(M)	DEG(M)	DEG(I+M)	DEG(M)	EGU80653.1	--	--	--
XLOC_032209	No	No	No	No	--	--	--	--
XLOC_032210	No	No	No	No	--	--	--	--
XLOC_032211	No	No	No	No	XP_009822641.1	--	--	--
XLOC_032212	No	No	No	No	--	--	--	--
XLOC_032218	No	No	No	No	ETO73517.1	--	--	--
XLOC_032219	No	No	No	No	--	--	--	--
XLOC_032222	No	No	No	No	--	--	--	--
XLOC_032224	No	No	No	No	--	--	--	--
XLOC_032226	No	No	No	No	XP_004494393.1	--	--	--
XLOC_032228	No	No	No	No	CEL62028.1	FAR1	O	--
XLOC_032229	No	No	No	No	CCO30452.1	FAR1	O	--
XLOC_032240	No	No	No	No	XP_001658058.1	--	--	--
XLOC_032241	DEG(M)	No	No	No	XP_003049765.1	--	--	--
XLOC_032242	No	No	No	No	--	--	--	K07428
XLOC_032247	DEG(M)	No	No	No	--	--	--	--
XLOC_032251	No	No	No	No	--	--	--	--
XLOC_032266	DEG(M)	No	No	No	--	--	--	--
XLOC_032267	DEG(M)	No	No	No	--	--	--	--
XLOC_032268	DEG(M)	No	No	No	--	--	--	--
XLOC_032269	DEG(M)	No	No	No	--	--	--	--
XLOC_032274	DEG(M)	No	No	No	XP_008914125.1	--	--	--
XLOC_032275	DEG(M)	DEG(M)	DEG(I+M)	DEG(M)	XP_008914125.1	--	--	--
XLOC_032280	No	No	No	No	--	--	--	--
XLOC_032281	No	No	No	No	ETL41729.1	--	--	--
XLOC_032282	No	No	DEG(I+M)	No	XP_004486117.1	--	--	--
XLOC_032284	No	No	DEG(I+M)	No	--	--	--	--
XLOC_032285	No	No	No	No	CEL57816.1	--	C	--
XLOC_032290	No	No	No	No	XP_003242717.1	--	--	--

Differentially expressed gene (DEG) IDs	*C. microphyllum* root and shoot-specific (>2 fold)	*C. microphyllum* root and shoot-specific (>10 fold)	ICC4958 and *C. microphyllum* root-specific (>2 fold)	*C. microphyllum* root-specific (>2 fold)	Functional Annotation			
					NCBI nr IDs	TFs	KOG	KEGG
XLOC_032292	No	No	No	No	XP_003053435.1	--	--	--
XLOC_032301	No	No	No	No	--	--	--	K02946
XLOC_032305	DEG(M)	DEG(M)	No	No	EWY94272.1	--	G	--
XLOC_032306	No	No	No	No	--	--	R	--
XLOC_032309	No	No	No	No	--	--	--	--
XLOC_032310	No	No	No	No	--	--	--	--
XLOC_032314	No	No	DEG(I+M)	No	--	--	--	--
XLOC_032316	No	No	No	No	XP_003045981.1	--	--	--
XLOC_032320	No	No	No	No	ELU40040.1	--	--	--
XLOC_032321	No	No	No	No	ELU40040.1	--	--	--
XLOC_032323	No	No	No	No	XP_008896134.1	--	--	K03963
XLOC_032325	No	No	No	No	--	--	--	--
XLOC_032329	DEG(M)	No	No	No	--	--	--	--
XLOC_032331	No	No	No	No	CEL61000.1	--	--	K09489
XLOC_032334	DEG(M)	No	No	No	--	--	--	--
XLOC_032336	No	No	No	No	--	--	--	--
XLOC_032337	DEG(M)	No	No	No	--	--	--	--
XLOC_032340	No	No	No	No	ABF85690.1	--	--	--
XLOC_032341	No	No	No	No	KDN37798.1	--	--	--
XLOC_032352	No	No	No	No	--	--	--	--
XLOC_032355	No	No	No	No	CCO28029.1	--	--	K15272
XLOC_032356	No	No	No	No	NP_001156280.1	--	--	--
XLOC_032357	No	No	No	No	--	--	--	--
XLOC_032358	No	No	No	No	KDN46048.1	--	J	K03262
XLOC_032359	No	No	No	No	XP_008179248.1	--	--	--
XLOC_032362	No	No	No	No	ETP00035.1	--	--	--
XLOC_032367	No	No	No	No	--	--	--	K01079
XLOC_032368	No	No	No	No	--	--	R	--
XLOC_032371	No	No	No	No	--	--	P	--
XLOC_032373	DEG(M)	No	No	No	--	--	--	--
XLOC_032380	No	No	No	No	--	--	--	--
XLOC_032382	No	No	No	No	--	--	--	--
XLOC_032385	No	No	No	No	--	--	--	--
XLOC_032387	No	No	No	No	--	--	--	--
XLOC_032388	No	No	No	No	--	--	--	--
XLOC_032394	DEG(M)	DEG(M)	DEG(I+M)	DEG(M)	--	--	--	--
XLOC_032396	DEG(M)	No	No	No	XP_009534555.1	--	J	--
XLOC_032399	DEG(M)	No	No	No	--	--	--	--
XLOC_032403	No	No	No	No	XP_012204880.1	--	G	--
XLOC_032404	No	No	No	No	XP_001951753.1	--	--	--

Differentially expressed gene (DEG) IDs	*C. microphyllum* root and shoot-specific (>2 fold)	*C. microphyllum* root and shoot-specific (>10 fold)	ICC4958 and *C. microphyllum* root-specific (>2 fold)	*C. microphyllum* root-specific (>2 fold)	Functional Annotation			
					NCBI nr IDs	TFs	KOG	KEGG
XLOC_032407	No	No	No	No	--	--	--	--
XLOC_032410	No	No	No	No	AGE97642.1	--	--	--
XLOC_032411	No	No	No	No	--	--	--	K05759
XLOC_032412	No	No	No	No	--	--	--	--
XLOC_032413	No	No	No	No	EUC64266.1	--	--	K06890
XLOC_032415	No	No	No	No	--	--	--	--
XLOC_032416	No	No	No	No	--	--	--	--
XLOC_032417	No	No	No	No	ETL30017.1	B3	E	K12657
XLOC_032418	No	No	No	No	KDN42147.1	--	--	--
XLOC_032420	No	No	DEG(I+M)	No	XP_004512446.1	G2-like	--	--
XLOC_032422	No	No	No	No	--	--	--	--
XLOC_032426	DEG(M)	No	No	No	--	--	--	--
XLOC_032427	No	No	No	No	--	--	--	--
XLOC_032428	DEG(M)	No	DEG(I+M)	DEG(M)	XP_002525956.1	--	T	K01611
XLOC_032431	No	No	No	No	--	--	--	--
XLOC_032433	No	No	No	No	--	FAR1	T	--
XLOC_032437	No	No	No	No	--	--	--	--
XLOC_032440	No	No	No	No	--	--	--	--
XLOC_032442	No	No	DEG(I+M)	No	XP_004485667.1	--	--	--
XLOC_032443	DEG(M)	No	No	No	ELU36254.1	--	O	--
XLOC_032444	No	No	No	No	XP_002900875.1	--	--	--
XLOC_032451	No	No	No	No	CCO27808.1	--	--	--
XLOC_032455	No	No	No	No	--	--	--	--
XLOC_032456	No	No	No	No	CCO33441.1	--	--	--
XLOC_032457	No	No	No	No	XP_003048635.1	--	--	--
XLOC_032458	No	No	No	No	--	--	--	--
XLOC_032459	No	No	No	No	XP_008180461.1	--	Z	K05759
XLOC_032462	DEG(M)	No	DEG(I+M)	DEG(M)	XP_008900945.1	--	--	--
XLOC_032463	No	No	No	No	--	--	--	--
XLOC_032464	No	No	No	No	--	--	--	--
XLOC_032466	No	No	No	No	--	--	--	K05756
XLOC_032467	DEG(M)	No	No	No	--	--	--	K11275
XLOC_032471	No	No	No	No	CCO32800.1	--	--	--
XLOC_032472	No	No	No	No	--	--	--	--
XLOC_032475	DEG(M)	DEG(M)	No	No	XP_007701233.1	--	--	--
XLOC_032476	No	No	No	No	CEL61895.1	--	--	--
XLOC_032481	DEG(M)	No	No	No	--	--	--	--
XLOC_032482	No	No	No	No	--	--	O	--
XLOC_032484	DEG(M)	No	No	No	XP_004499623.2	--	R	--
XLOC_032485	No	No	DEG(I+M)	No	--	--	--	--

Differentially expressed gene (DEG) IDs	*C. microphyllum* root and shoot-specific (>2 fold)	*C. microphyllum* root and shoot-specific (>10 fold)	ICC4958 and *C. microphyllum* root-specific (>2 fold)	*C. microphyllum* root-specific (>2 fold)	Functional Annotation			
					NCBI nr IDs	TFs	KOG	KEGG
XLOC_032487	No	No	No	No	--	--	--	--
XLOC_032488	No	No	No	No	XP_001952270.1	--	--	K11093
XLOC_032490	No	No	No	No	CCO26953.1	--	--	--
XLOC_032496	No	No	No	No	AAB04132.1	--	--	--
XLOC_032498	No	No	No	No	--	--	--	--
XLOC_032503	DEG(M)	No	No	No	XP_003051079.1	--	T	--
XLOC_032504	No	No	DEG(I+M)	No	--	--	--	--
XLOC_032507	No	No	No	No	--	--	--	--
XLOC_032509	DEG(M)	No	No	No	--	--	--	--
XLOC_032512	No	No	No	No	--	--	--	--
XLOC_032514	No	No	No	No	--	--	--	--
XLOC_032518	No	No	No	No	--	--	--	--
XLOC_032520	No	No	No	No	XP_001951034.2	--	--	--
XLOC_032521	DEG(M)	DEG(M)	No	No	--	--	--	--
XLOC_032524	No	No	No	No	--	--	R	--
XLOC_032529	No	No	No	No	ETP34548.1	FAR1	O	--
XLOC_032533	No	No	No	No	--	--	--	K03883
XLOC_032536	No	No	DEG(I+M)	No	XP_004508965.1	--	--	--
XLOC_032537	No	No	DEG(I+M)	No	XP_004511012.1	--	--	--
XLOC_032538	DEG(M)	No	No	No	--	--	--	--
XLOC_032540	No	No	No	No	CEL63589.1	--	--	--
XLOC_032541	No	No	No	No	--	--	--	--
XLOC_032542	No	No	No	No	--	--	--	--
XLOC_032546	DEG(M)	DEG(M)	No	No	--	--	--	--
XLOC_032548	DEG(M)	No	No	No	--	--	--	--
XLOC_032549	DEG(M)	No	No	No	ETP13620.1	--	--	--
XLOC_032552	No	No	No	No	EUC57720.1	--	--	--
XLOC_032553	No	No	No	No	CEL62619.1	--	--	--
XLOC_032554	No	No	No	No	--	--	--	--
XLOC_032555	No	No	No	No	--	--	--	--
XLOC_032556	No	No	No	No	--	--	--	--
XLOC_032558	No	No	No	No	XP_009525521.1	--	--	K09503
XLOC_032560	No	No	No	No	XP_008868227.1	--	E	--
XLOC_032564	DEG(M)	No	No	No	--	--	--	--
XLOC_032569	DEG(M)	DEG(M)	DEG(I+M)	DEG(M)	XP_002906315.1	--	--	K02917
XLOC_032572	DEG(M)	No	No	No	--	--	--	--
XLOC_032573	No	No	No	No	--	--	--	--
XLOC_032574	DEG(M)	DEG(M)	DEG(I+M)	DEG(M)	XP_004340976.1	NF-YB	J	--
XLOC_032575	No	No	No	No	--	--	--	--
XLOC_032576	No	No	No	No	XP_004494411.1	MYB	--	K09422

Differentially expressed gene (DEG) IDs	*C. microphyllum* root and shoot-specific (>2 fold)	*C. microphyllum* root and shoot-specific (>10 fold)	ICC4958 and *C. microphyllum* root-specific (>2 fold)	*C. microphyllum* root-specific (>2 fold)	Functional Annotation			
					NCBI nr IDs	TFs	KOG	KEGG
XLOC_032579	No	No	No	No	KFM61555.1	--	K	K13091
XLOC_032580	No	No	No	No	EUC64835.1	--	--	--
XLOC_032586	No	No	No	No	--	--	--	--
XLOC_032590	No	No	No	No	--	--	--	--
XLOC_032595	No	No	No	No	--	--	--	K12663
XLOC_032597	No	No	No	No	--	--	--	--
XLOC_032599	No	No	No	No	XP_003048814.1	--	--	--
XLOC_032602	DEG(M)	No	No	No	--	--	--	--
XLOC_032606	No	No	No	No	--	--	--	--
XLOC_032610	No	No	No	No	CEL51615.1	--	--	--
XLOC_032612	No	No	No	No	ELU40360.1	--	--	--
XLOC_032616	DEG(M)	DEG(M)	DEG(I+M)	DEG(M)	--	--	--	--
XLOC_032619	DEG(M)	No	No	No	--	--	--	--
XLOC_032620	DEG(M)	No	No	No	--	--	--	--
XLOC_032622	No	No	No	No	--	--	--	--
XLOC_032627	No	No	No	No	XP_005105940.1	NF-YC	B	K11251
XLOC_032628	No	No	No	No	AHN53412.1	--	--	K11252
XLOC_032629	DEG(M)	No	No	No	AHN53412.1	--	--	K11252
XLOC_032634	No	No	No	No	--	--	--	--
XLOC_032638	No	No	DEG(I+M)	No	EGU88259.1	--	J	K02910
XLOC_032642	DEG(M)	DEG(M)	DEG(I+M)	DEG(M)	--	--	--	--
XLOC_032646	DEG(M)	No	No	No	--	--	--	--
XLOC_032656	DEG(M)	No	No	No	XP_012570934.1	GATA	GE	K15285
XLOC_032657	No	No	No	No	XP_012570934.1	GATA	--	K15285
XLOC_032661	DEG(M)	DEG(M)	No	No	--	--	--	--
XLOC_032673	DEG(M)	No	No	No	KEP47084.1	--	R	--
XLOC_032679	No	No	No	No	--	--	--	--
XLOC_032680	No	No	No	No	XP_004512553.1	--	E	K00600
XLOC_032682	DEG(M)	DEG(M)	No	No	XP_003047736.1	--	J	--
XLOC_032687	DEG(M)	DEG(M)	DEG(I+M)	DEG(M)	--	--	--	--
XLOC_032690	DEG(M)	No	No	No	ETL93890.1	--	G	K00033
XLOC_032691	No	No	No	No	ETL93890.1	--	G	K00033
XLOC_032695	DEG(M)	No	No	No	XP_008905373.1	--	C	K02155
XLOC_032696	DEG(M)	No	No	No	--	--	--	--
XLOC_032697	DEG(M)	No	No	No	KEP51407.1	--	--	--
XLOC_032698	No	No	No	No	KDN47034.1	--	--	--
XLOC_032699	No	No	DEG(I+M)	No	--	--	O	K00799
XLOC_032701	DEG(M)	No	No	No	EUC64866.1	--	A	--
XLOC_032702	DEG(M)	No	No	No	--	--	--	--
XLOC_032707	No	No	No	No	XP_007266595.1	--	O	--

Differentially expressed gene (DEG) IDs	*C. microphyllum* root and shoot-specific (>2 fold)	*C. microphyllum* root and shoot-specific (>10 fold)	ICC4958 and *C. microphyllum* root-specific (>2 fold)	*C. microphyllum* root-specific (>2 fold)	Functional Annotation			
					NCBI nr IDs	TFs	KOG	KEGG
XLOC_032708	No	No	No	No	EUC64143.1	--	O	--
XLOC_032709	DEG(M)	No	No	No	KEP48438.1	--	--	--
XLOC_032710	No	No	No	No	--	--	--	K02113
XLOC_032711	No	No	No	No	XP_004499538.1	bHLH	--	K16189
XLOC_032712	No	No	No	No	--	--	--	--
XLOC_032718	No	No	No	No	--	--	--	K02968
XLOC_032721	DEG(M)	No	DEG(I+M)	DEG(M)	XP_004515994.1	--	R	K08472
XLOC_032727	No	No	DEG(I+M)	No	--	--	P	K17686
XLOC_032736	No	No	No	No	--	--	--	--
XLOC_032739	No	No	No	No	--	--	--	--
XLOC_032745	DEG(M)	No	No	No	--	--	--	--
XLOC_032749	No	No	No	No	--	--	--	--
XLOC_032754	No	No	No	No	XP_001950501.2	--	E	--
XLOC_032764	No	No	No	No	--	--	--	--
XLOC_032765	DEG(M)	No	No	No	--	--	--	--
XLOC_032767	DEG(M)	No	No	No	--	--	--	--
XLOC_032769	No	No	No	No	XP_001663442.1	--	O	--
XLOC_032780	No	No	No	No	ELU37220.1	--	--	--
XLOC_032782	No	No	No	No	--	--	--	--
XLOC_032783	No	No	No	No	--	--	--	--
XLOC_032784	No	No	No	No	XP_002999173.1	--	--	--
XLOC_032786	No	No	No	No	--	--	--	--
XLOC_032787	No	No	No	No	--	--	--	--
XLOC_032789	No	No	No	No	XP_001654322.1	--	P	K17285
XLOC_032791	No	No	No	No	ETI34554.1	--	--	--
XLOC_032792	DEG(M)	DEG(M)	DEG(I+M)	DEG(M)	AAH53790.1	--	T	--
XLOC_032793	DEG(M)	No	DEG(I+M)	DEG(M)	XP_006125555.1	--	T	--
XLOC_032801	No	No	No	No	--	--	--	--
XLOC_032811	No	No	No	No	--	--	--	--
XLOC_032814	No	No	No	No	YP_001649162.1	--	C	--
XLOC_032819	No	No	No	No	--	--	--	--
XLOC_032820	No	No	No	No	CEL55336.1	--	--	--
XLOC_032821	No	No	No	No	--	--	--	--
XLOC_032825	DEG(M)	No	No	No	KNC95909.1	--	C	--
XLOC_032832	No	No	No	No	KEP50281.1	--	--	--
XLOC_032836	DEG(M)	DEG(M)	No	No	--	--	--	--
XLOC_032837	No	No	No	No	--	--	Z	--
XLOC_032838	DEG(M)	No	No	No	XP_002906463.1	--	Z	K08341
XLOC_032839	No	No	No	No	--	--	X	--
XLOC_032841	No	No	No	No	CEL56041.1	--	--	--

Differentially expressed gene (DEG) IDs	*C. microphyllum* root and shoot-specific (>2 fold)	*C. microphyllum* root and shoot-specific (>10 fold)	ICC4958 and *C. microphyllum* root-specific (>2 fold)	*C. microphyllum* root-specific (>2 fold)	Functional Annotation			
					NCBI nr IDs	TFs	KOG	KEGG
XLOC_032842	No	No	No	No	CCO26659.1	--	--	--
XLOC_032844	DEG(M)	No	No	No	--	--	--	--
XLOC_032849	No	No	No	No	--	--	--	--
XLOC_032850	DEG(M)	DEG(M)	No	No	--	--	--	--
XLOC_032851	DEG(M)	DEG(M)	No	No	--	--	--	--
XLOC_032854	No	No	No	No	--	--	--	K05765
XLOC_032859	No	No	No	No	--	--	--	--
XLOC_032860	DEG(M)	No	No	No	BAH70592.1	--	C	--
XLOC_032862	No	No	No	No	EUC63304.1	--	--	--
XLOC_032874	DEG(M)	DEG(M)	DEG(I+M)	DEG(M)	XP_002901276.1	--	J	--
XLOC_032875	DEG(M)	DEG(M)	DEG(I+M)	DEG(M)	XP_008900376.1	--	J	--
XLOC_032880	DEG(M)	No	No	No	EUC53774.1	--	J	--
XLOC_032882	DEG(M)	No	DEG(I+M)	DEG(M)	EUC53774.1	--	J	--
XLOC_032885	No	No	No	No	--	--	R	K00059
XLOC_032888	No	No	No	No	ETP55064.1	--	--	--
XLOC_032891	No	No	DEG(I+M)	No	XP_012569167.1	--	--	--
XLOC_032893	DEG(M)	No	No	No	EUC59663.1	--	J	--
XLOC_032894	No	No	No	No	EUC59663.1	--	J	--
XLOC_032897	No	No	DEG(I+M)	No	XP_004505624.1	NAC	V	--
XLOC_032900	DEG(M)	No	No	No	--	--	M	--
XLOC_032901	DEG(M)	No	No	No	--	--	--	--
XLOC_032908	No	No	No	No	--	--	--	K01051
XLOC_032912	DEG(M)	No	No	No	CEL53831.1	--	--	--
XLOC_032917	No	No	No	No	--	--	--	--
XLOC_032918	DEG(M)	No	No	No	--	--	--	--
XLOC_032921	No	No	No	No	KEP54040.1	--	--	K18195
XLOC_032923	No	No	No	No	--	--	--	--
XLOC_032925	No	No	No	No	XP_004500683.1	--	C	K08915
XLOC_032926	No	No	DEG(I+M)	No	--	--	R	--
XLOC_032931	DEG(M)	No	No	No	--	--	--	--
XLOC_032932	DEG(M)	No	No	No	--	--	--	--
XLOC_032933	DEG(M)	No	No	No	--	--	--	--
XLOC_032938	DEG(M)	DEG(M)	DEG(I+M)	DEG(M)	--	--	--	K13691
XLOC_032941	No	No	No	No	XP_002513771.1	--	--	--
XLOC_032943	No	No	DEG(I+M)	No	--	WRKY	--	--
XLOC_032947	DEG(M)	No	No	No	--	--	--	--
XLOC_032953	No	No	No	No	--	--	--	--
XLOC_032954	DEG(M)	DEG(M)	No	No	EXL95594.1	--	--	--
XLOC_032955	No	No	No	No	EGU78232.1	--	--	--
XLOC_032961	No	No	No	No	--	--	--	--

Differentially expressed gene (DEG) IDs	*C. microphyllum* root and shoot-specific (>2 fold)	*C. microphyllum* root and shoot-specific (>10 fold)	ICC4958 and *C. microphyllum* root-specific (>2 fold)	*C. microphyllum* root-specific (>2 fold)	Functional Annotation			
					NCBI nr IDs	TFs	KOG	KEGG
XLOC_032969	No	No	No	No	XP_003051386.1	--	--	--
XLOC_032973	DEG(M)	No	DEG(I+M)	DEG(M)	--	--	--	--
XLOC_032975	No	No	No	No	--	--	--	--
XLOC_032976	DEG(M)	No	No	No	--	--	--	--
XLOC_032977	DEG(M)	No	No	No	EWZ49356.1	--	--	--
XLOC_032978	DEG(M)	No	No	No	KDN49398.1	--	--	--
XLOC_032979	No	No	No	No	--	--	--	--
XLOC_032981	DEG(M)	No	No	No	CCO28937.1	--	--	--
XLOC_032983	No	No	No	No	KDN48790.1	--	--	--
XLOC_032984	No	No	No	No	--	--	E	--
XLOC_032986	No	No	No	No	--	--	--	--
XLOC_032988	No	No	No	No	--	--	--	--
XLOC_032989	No	No	No	No	--	--	--	--
XLOC_032993	DEG(M)	No	No	No	CCO32204.1	--	I	--
XLOC_032996	No	No	No	No	--	--	--	--
XLOC_033000	No	No	No	No	--	--	--	--
XLOC_033005	No	No	No	No	--	--	--	--
XLOC_033007	No	No	No	No	--	--	--	K01363
XLOC_033009	No	No	No	No	XP_012570165.1	MYB	K	K09422
XLOC_033017	No	No	No	No	AEZ51040.1	--	--	--
XLOC_033018	No	No	No	No	CCO30188.1	--	O	K10575
XLOC_033020	No	No	No	No	EUC62171.1	--	--	--
XLOC_033021	DEG(M)	No	DEG(I+M)	DEG(M)	AAO39434.1	--	Z	--
XLOC_033023	No	No	No	No	--	--	--	--
XLOC_033024	No	No	No	No	--	--	--	--
XLOC_033029	DEG(M)	No	No	No	--	--	--	--
XLOC_033030	No	No	No	No	XP_001660605.1	--	A	K06174
XLOC_033031	No	No	No	No	--	--	--	--
XLOC_033034	No	No	No	No	NP_001156302.1	--	--	--
XLOC_033035	No	No	No	No	--	--	--	--
XLOC_033037	No	No	No	No	--	--	O	--
XLOC_033038	DEG(M)	No	No	No	CEL57830.1	--	--	--
XLOC_033039	DEG(M)	No	No	No	CEL58729.1	--	--	--
XLOC_033043	No	No	DEG(I+M)	No	--	MYB	--	K09422
XLOC_033045	No	No	No	No	--	--	--	--
XLOC_033046	No	No	No	No	CCO26472.1	--	--	--
XLOC_033049	No	No	No	No	XP_004513659.1	MIKC	K	K09264
XLOC_033050	No	No	No	No	XP_001650465.1	--	Z	--
XLOC_033052	No	No	DEG(I+M)	No	--	--	--	--
XLOC_033053	No	No	No	No	XP_001653469.1	--	T	--

Differentially expressed gene (DEG) IDs	*C. microphyllum* root and shoot-specific (>2 fold)	*C. microphyllum* root and shoot-specific (>10 fold)	ICC4958 and *C. microphyllum* root-specific (>2 fold)	*C. microphyllum* root-specific (>2 fold)	Functional Annotation			
					NCBI nr IDs	TFs	KOG	KEGG
XLOC_033054	DEG(M)	No	No	No	EUC60659.1	--	--	--
XLOC_033055	DEG(M)	No	No	No	NP_001156510.1	--	O	--
XLOC_033058	No	No	No	No	XP_002510104.1	--	Z	--
XLOC_033062	No	No	No	No	NP_001119690.1	--	C	K02151
XLOC_033064	DEG(M)	No	No	No	--	--	--	--
XLOC_033066	No	No	No	No	--	--	--	--
XLOC_033067	No	No	No	No	--	--	--	K06617
XLOC_033068	No	No	No	No	--	--	--	K19035
XLOC_033069	No	No	No	No	--	--	X	--
XLOC_033080	No	No	DEG(I+M)	No	--	--	G	K09873
XLOC_033084	DEG(M)	No	No	No	--	--	--	--
XLOC_033085	No	No	No	No	ACG60423.1	--	O	--
XLOC_033086	DEG(M)	No	No	No	--	--	--	--
XLOC_033088	DEG(M)	DEG(M)	No	No	--	--	--	--
XLOC_033089	DEG(M)	No	No	No	XP_003053396.1	--	J	--
XLOC_033091	No	No	No	No	--	--	--	--
XLOC_033093	No	No	No	No	--	--	--	K13066
XLOC_033104	DEG(M)	No	DEG(I+M)	DEG(M)	XP_004486954.1	C2H2	--	--
XLOC_033105	DEG(M)	No	No	No	XP_001949785.1	--	J	K02987
XLOC_033112	DEG(M)	DEG(M)	DEG(I+M)	DEG(M)	XP_008903366.1	--	J	K02889
XLOC_033114	No	No	No	No	XP_008916458.1	--	--	--
XLOC_033121	No	No	No	No	--	--	--	--
XLOC_033124	No	No	No	No	CCO28146.1	--	--	--
XLOC_033127	No	No	No	No	KNG49993.1	--	--	--
XLOC_033136	DEG(M)	No	No	No	ETI41741.1	--	O	--
XLOC_033137	DEG(M)	No	No	No	ETM41681.1	--	--	--
XLOC_033143	No	No	No	No	XP_004504023.1	--	--	--
XLOC_033146	No	No	No	No	XP_008894090.1	--	O	--
XLOC_033147	No	No	No	No	XP_008862081.1	--	O	--
XLOC_033153	DEG(M)	No	No	No	XP_001952053.1	--	J	K03257
XLOC_033162	DEG(M)	No	No	No	--	--	--	--
XLOC_033163	No	No	No	No	EUC67185.1	--	--	--
XLOC_033165	DEG(M)	DEG(M)	DEG(I+M)	DEG(M)	XP_002997507.1	--	O	--
XLOC_033166	DEG(M)	No	No	No	EUC66088.1	--	--	--
XLOC_033167	DEG(M)	No	No	No	--	--	--	--
XLOC_033171	No	No	No	No	--	--	--	--
XLOC_033172	No	No	No	No	--	--	--	--
XLOC_033175	No	No	No	No	XP_002529366.1	--	I	--
XLOC_033176	No	No	No	No	CEL63774.1	--	--	K05765
XLOC_033179	No	No	No	No	XP_001657500.1	--	K	K03232

Differentially expressed gene (DEG) IDs	***C. microphyllum* root and shoot-specific (>2 fold)**	***C. microphyllum* root and shoot-specific (>10 fold)**	**ICC4958 and *C. microphyllum* root-specific (>2 fold)**	***C. microphyllum* root-specific (>2 fold)**	**Functional Annotation**			
					**NCBI nr IDs**	**TFs**	**KOG**	**KEGG**
XLOC_033181	DEG(M)	No	No	No	--	--	--	--
XLOC_033182	No	No	No	No	--	--	--	--
XLOC_033186	No	No	No	No	KDN43391.1	--	--	--
XLOC_033188	DEG(M)	No	No	No	EUC56119.1	--	--	--
XLOC_033189	DEG(M)	No	No	No	EUC56119.1	--	--	--
XLOC_033190	DEG(M)	No	No	No	KDN43391.1	--	--	--
XLOC_033197	No	No	No	No	--	--	--	--
XLOC_033198	DEG(M)	No	No	No	--	--	--	--
XLOC_033199	DEG(M)	No	No	No	--	--	--	--
XLOC_033207	DEG(M)	No	DEG(I+M)	DEG(M)	XP_004507474.1	--	H	K13998
XLOC_033235	DEG(M)	No	DEG(I+M)	DEG(M)	KEP49546.1	--	--	--
XLOC_033236	DEG(M)	No	DEG(I+M)	DEG(M)	KEP49546.1	--	--	--
XLOC_033239	DEG(M)	DEG(M)	DEG(I+M)	DEG(M)	--	--	--	--
XLOC_033251	DEG(M)	No	No	No	--	--	--	--
XLOC_033252	DEG(M)	No	No	No	--	--	--	--
XLOC_033253	DEG(M)	No	No	No	--	--	--	--
XLOC_033257	No	No	No	No	KEP55133.1	--	G	--
XLOC_033258	No	No	No	No	CEL58105.1	--	--	--
XLOC_033278	No	No	No	No	ELU40828.1	--	--	--
XLOC_033289	DEG(M)	DEG(M)	DEG(I+M)	DEG(M)	ETK76456.1	C2H2	U	--
XLOC_033290	DEG(M)	No	No	No	XP_002900891.1	--	U	--
XLOC_033291	DEG(M)	No	No	No	XP_008610697.1	--	U	--
XLOC_033292	DEG(M)	No	No	No	XP_008610697.1	--	U	--
XLOC_033295	DEG(M)	DEG(M)	DEG(I+M)	DEG(M)	ADX01224.1	--	Z	--
XLOC_033296	DEG(M)	DEG(M)	DEG(I+M)	DEG(M)	XP_008910655.1	--	Z	K10355
XLOC_033298	DEG(M)	DEG(M)	DEG(I+M)	DEG(M)	AHA46382.1	--	Z	--
XLOC_033301	DEG(M)	No	No	No	--	--	--	--
XLOC_033315	No	No	No	No	--	--	--	K02983
XLOC_033319	No	No	No	No	CCO26952.1	--	--	--
XLOC_033325	No	No	No	No	XP_002514195.1	MYB	R	--
XLOC_033327	No	No	No	No	CEL53798.1	--	--	--
XLOC_033328	No	No	No	No	XP_008911982.1	--	--	--
XLOC_033329	No	No	No	No	--	--	--	--
XLOC_033331	No	No	No	No	--	--	--	--
XLOC_033333	No	No	No	No	--	--	--	--
XLOC_033334	No	No	No	No	--	--	--	--
XLOC_033337	No	No	No	No	--	--	--	--
XLOC_033344	No	No	No	No	--	--	--	--
XLOC_033345	No	No	No	No	XP_001946123.1	--	--	--
XLOC_033348	No	No	No	No	CEL57620.1	--	--	--

Differentially expressed gene (DEG) IDs	*C. microphyllum* root and shoot-specific (>2 fold)	*C. microphyllum* root and shoot-specific (>10 fold)	ICC4958 and *C. microphyllum* root-specific (>2 fold)	*C. microphyllum* root-specific (>2 fold)	Functional Annotation			
					NCBI nr IDs	TFs	KOG	KEGG
XLOC_033350	No	No	No	No	--	--	--	--
XLOC_033351	No	No	No	No	EWZ81923.1	--	--	--
XLOC_033354	No	No	No	No	--	bHLH	J	--
XLOC_033356	No	No	No	No	--	--	--	--
XLOC_033359	No	No	No	No	XP_008903595.1	--	A	--
XLOC_033361	No	No	No	No	--	--	--	--
XLOC_033366	No	No	No	No	ETP35633.1	--	--	--
XLOC_033369	No	No	No	No	XP_001951748.2	GATA	U	--
XLOC_033371	No	No	No	No	EUC65573.1	--	--	K10599
XLOC_033374	No	No	No	No	--	NAC	J	--
XLOC_033385	No	No	No	No	CCO26395.1	--	O	--
XLOC_033386	DEG(M)	DEG(M)	DEG(I+M)	DEG(M)	CCO26395.1	--	O	--
XLOC_033387	DEG(M)	DEG(M)	DEG(I+M)	DEG(M)	--	--	--	--
XLOC_033388	DEG(M)	No	DEG(I+M)	DEG(M)	--	--	--	--
XLOC_033391	DEG(M)	No	DEG(I+M)	DEG(M)	KEP54249.1	--	C	--
XLOC_033392	DEG(M)	No	DEG(I+M)	DEG(M)	EWZ02370.1	--	C	--
XLOC_033393	DEG(M)	No	No	No	KDN42973.1	--	C	K05863
XLOC_033394	DEG(M)	No	No	No	KDN42973.1	--	C	--
XLOC_033395	No	No	No	No	KNC95909.1	--	C	--
XLOC_033406	DEG(M)	No	No	No	--	--	--	--
XLOC_033407	DEG(M)	No	No	No	--	--	--	--
XLOC_033408	DEG(M)	DEG(M)	No	No	--	--	--	--
XLOC_033409	DEG(M)	No	No	No	--	--	--	--
XLOC_033410	DEG(M)	No	No	No	--	--	--	--
XLOC_033455	No	No	No	No	--	--	--	--
XLOC_033471	DEG(M)	No	No	No	--	--	--	--
XLOC_033497	DEG(M)	DEG(M)	No	No	--	--	--	--
XLOC_033507	No	No	No	No	--	--	--	--
XLOC_033514	DEG(M)	No	No	No	--	--	--	--
XLOC_033515	DEG(M)	No	DEG(I+M)	DEG(M)	--	--	--	--
XLOC_033516	DEG(M)	No	No	No	--	--	--	--
XLOC_033517	DEG(M)	No	DEG(I+M)	DEG(M)	--	--	--	--
XLOC_033529	No	No	No	No	--	--	--	--
XLOC_033533	No	No	No	No	XP_004487584.1	--	--	--
XLOC_033535	DEG(M)	DEG(M)	DEG(I+M)	DEG(M)	XP_004487589.1	--	--	--
XLOC_033536	DEG(M)	No	DEG(I+M)	DEG(M)	--	--	--	--
XLOC_033538	DEG(M)	No	DEG(I+M)	DEG(M)	XP_004487585.1	--	--	--
XLOC_033548	No	No	No	No	--	--	T	--
XLOC_033560	No	No	DEG(I+M)	No	--	--	--	--
XLOC_033561	No	No	No	No	XP_012567813.1	--	--	--

**Differentially expressed gene (DEG) IDs**	***C. microphyllum* root and shoot-specific (>2 fold)**	***C. microphyllum* root and shoot-specific (>10 fold)**	**ICC4958 and *C. microphyllum* root-specific (>2 fold)**	***C. microphyllum* root-specific (>2 fold)**	**Functional Annotation**			
					**NCBI nr IDs**	**TFs**	**KOG**	**KEGG**
XLOC_033565	DEG(M)	DEG(M)	No	No	--	--	--	--
XLOC_033571	No	No	DEG(I+M)	No	--	--	--	--
XLOC_033588	DEG(M)	No	No	No	--	--	--	--
XLOC_033602	DEG(M)	No	DEG(I+M)	DEG(M)	XP_004504278.1	--	--	K00799
XLOC_033661	DEG(M)	DEG(M)	DEG(I+M)	DEG(M)	--	--	--	--
XLOC_033662	DEG(M)	DEG(M)	DEG(I+M)	DEG(M)	--	--	--	K13260
XLOC_033663	DEG(M)	DEG(M)	DEG(I+M)	DEG(M)	--	--	--	--
XLOC_033665	No	No	DEG(I+M)	No	XP_004499689.1	--	--	--
XLOC_033668	No	No	No	No	--	--	--	--
XLOC_033671	No	No	No	No	--	--	--	--
XLOC_033672	No	No	No	No	--	--	--	--
XLOC_033673	No	No	No	No	--	--	--	--
XLOC_033674	No	No	DEG(I+M)	No	XP_004499689.1	--	--	--
XLOC_033675	DEG(M)	DEG(M)	DEG(I+M)	DEG(M)	--	--	--	--
XLOC_033714	No	No	DEG(I+M)	No	--	--	--	K13066
XLOC_033716	DEG(M)	No	DEG(I+M)	DEG(M)	--	--	--	K13066
XLOC_033717	No	No	DEG(I+M)	No	XP_004513588.1	--	--	--
XLOC_033719	No	No	DEG(I+M)	No	XP_004516751.1	--	--	--
XLOC_033736	DEG(M)	No	No	No	--	--	--	--
XLOC_033737	DEG(M)	DEG(M)	No	No	ABJ03470.1	--	--	--
XLOC_033739	DEG(M)	No	No	No	EDM70194.1	--	--	--
XLOC_033740	DEG(M)	DEG(M)	DEG(I+M)	DEG(M)	--	--	--	--
XLOC_033761	DEG(M)	No	No	No	--	--	T	--
XLOC_033768	No	No	No	No	XP_004496784.1	--	--	--
XLOC_033790	No	No	No	No	--	--	--	--
XLOC_033799	DEG(M)	No	No	No	--	--	--	--
XLOC_033800	DEG(M)	No	No	No	--	--	--	--
XLOC_033801	DEG(M)	No	No	No	--	--	--	--
XLOC_033802	DEG(M)	No	No	No	--	--	--	--
XLOC_033863	No	No	DEG(I+M)	No	--	--	--	--
XLOC_033864	No	No	DEG(I+M)	No	--	--	C	--
XLOC_033867	No	No	DEG(I+M)	No	XP_004489030.1	--	C	K01595
XLOC_033876	No	No	No	No	EUC67144.1	--	O	--
XLOC_033877	DEG(M)	No	No	No	CCO26137.1	--	O	--
XLOC_033878	DEG(M)	No	DEG(I+M)	DEG(M)	--	--	--	--
XLOC_033882	DEG(M)	DEG(M)	DEG(I+M)	DEG(M)	XP_011316555.1	MYB_related	O	--
XLOC_033883	DEG(M)	No	DEG(I+M)	DEG(M)	XP_003053706.1	--	O	--
XLOC_033884	DEG(M)	DEG(M)	DEG(I+M)	DEG(M)	CEL51818.1	--	O	--
XLOC_033886	DEG(M)	DEG(M)	DEG(I+M)	DEG(M)	XP_012181355.1	--	O	--
XLOC_033887	DEG(M)	DEG(M)	DEG(I+M)	DEG(M)	EUC62231.1	--	O	--

Differentially expressed gene (DEG) IDs	*C. microphyllum* root and shoot-specific (>2 fold)	*C. microphyllum* root and shoot-specific (>10 fold)	ICC4958 and *C. microphyllum* root-specific (>2 fold)	*C. microphyllum* root-specific (>2 fold)	Functional Annotation			
					NCBI nr IDs	TFs	KOG	KEGG
XLOC_033888	DEG(M)	DEG(M)	DEG(I+M)	DEG(M)	CEL51818.1	--	O	--
XLOC_033889	DEG(M)	DEG(M)	DEG(I+M)	DEG(M)	CRJ95477.1	--	O	--
XLOC_033891	DEG(M)	No	DEG(I+M)	DEG(M)	ABW06851.1	--	O	--
XLOC_033892	DEG(M)	DEG(M)	DEG(I+M)	DEG(M)	CCO27998.1	--	O	--
XLOC_033893	DEG(M)	DEG(M)	DEG(I+M)	DEG(M)	CCO27998.1	--	O	--
XLOC_033894	DEG(M)	DEG(M)	DEG(I+M)	DEG(M)	CEL51818.1	--	O	--
XLOC_033895	DEG(M)	No	No	No	CCO26137.1	--	O	--
XLOC_033911	No	No	No	No	--	--	--	--
XLOC_033914	No	No	No	No	--	--	--	--
XLOC_033916	No	No	No	No	XP_003045929.1	--	J	--
XLOC_033917	No	No	No	No	XP_012081442.1	NAC	--	--
XLOC_033918	No	No	No	No	--	--	--	--
XLOC_033919	No	No	No	No	XP_001951061.1	--	--	K14826
XLOC_033921	DEG(M)	No	No	No	--	--	--	--
XLOC_033939	No	No	No	No	--	--	--	--
XLOC_033954	No	No	DEG(I+M)	No	--	--	--	--
XLOC_033955	No	No	DEG(I+M)	No	XP_004498941.1	--	I	K00511
XLOC_033961	No	No	No	No	--	--	--	--
XLOC_033966	DEG(M)	No	No	No	CCO30947.1	--	--	K11517
XLOC_033967	No	No	No	No	CEL61011.1	--	J	K02901
XLOC_033968	DEG(M)	No	No	No	CEL61011.1	--	J	K02901
XLOC_033969	DEG(M)	No	No	No	CEL61011.1	--	J	K02901
XLOC_033971	No	No	No	No	--	--	--	--
XLOC_033973	No	No	No	No	--	--	--	--
XLOC_033974	DEG(M)	No	No	No	ACJ74466.1	--	J	--
XLOC_033976	DEG(M)	No	No	No	XP_001650616.1	--	J	--
XLOC_033978	DEG(M)	No	No	No	KIJ20603.1	--	B	K11254
XLOC_033989	No	No	No	No	ERG80132.1	--	O	K01802
XLOC_033991	No	No	No	No	--	--	--	--
XLOC_033992	No	No	No	No	--	--	--	--
XLOC_033994	No	No	DEG(I+M)	No	--	--	--	--
XLOC_034001	No	No	No	No	--	--	--	--
XLOC_034005	No	No	DEG(I+M)	No	--	--	--	--
XLOC_034008	No	No	No	No	CCO27406.1	--	--	--
XLOC_034009	No	No	No	No	--	--	--	--
XLOC_034023	DEG(M)	No	No	No	--	--	--	--
XLOC_034024	DEG(M)	No	No	No	--	--	--	--
XLOC_034029	DEG(M)	No	No	No	XP_003048021.1	--	--	--
XLOC_034031	DEG(M)	DEG(M)	DEG(I+M)	DEG(M)	--	--	--	--
XLOC_034035	DEG(M)	DEG(M)	No	No	--	--	--	--

Differentially expressed gene (DEG) IDs	*C. microphyllum* root and shoot-specific (>2 fold)	*C. microphyllum* root and shoot-specific (>10 fold)	ICC4958 and *C. microphyllum* root-specific (>2 fold)	*C. microphyllum* root-specific (>2 fold)	Functional Annotation			
					NCBI nr IDs	TFs	KOG	KEGG
XLOC_034036	DEG(M)	DEG(M)	No	No	--	--	--	--
XLOC_034037	No	No	No	No	EUC64226.1	--	--	--
XLOC_034039	No	No	No	No	KEP52889.1	--	--	--
XLOC_034040	No	No	No	No	KEP52889.1	--	--	--
XLOC_034041	No	No	No	No	ELU37207.1	--	F	K00939
XLOC_034042	DEG(M)	DEG(M)	DEG(I+M)	DEG(M)	--	--	--	--
XLOC_034043	DEG(M)	No	No	No	--	--	--	--
XLOC_034044	No	No	No	No	--	--	--	--
XLOC_034053	No	No	No	No	--	--	--	--
XLOC_034056	DEG(M)	No	No	No	--	--	--	--
XLOC_034058	DEG(M)	No	No	No	XP_003044867.1	--	J	--
XLOC_034061	No	No	No	No	XP_008891931.1	--	E	--
XLOC_034066	No	No	No	No	XP_004337289.1	NAC	J	--
XLOC_034069	DEG(M)	No	No	No	XP_004505321.1	--	P	K08176
XLOC_034072	DEG(M)	No	DEG(I+M)	DEG(M)	--	--	--	--
XLOC_034073	DEG(M)	No	No	No	--	--	--	--
XLOC_034076	No	No	No	No	KDN48773.1	--	C	K00161
XLOC_034077	No	No	No	No	CEL55600.1	--	--	--
XLOC_034080	No	No	No	No	KDN38622.1	--	--	--
XLOC_034081	DEG(M)	No	No	No	--	--	--	--
XLOC_034091	DEG(M)	DEG(M)	No	No	--	--	--	K06689
XLOC_034094	DEG(M)	No	No	No	EUC66859.1	--	--	--
XLOC_034095	No	No	No	No	--	--	--	--
XLOC_034099	No	No	No	No	XP_003047870.1	--	--	--
XLOC_034104	No	No	No	No	XP_006666312.1	--	C	--
XLOC_034106	No	No	No	No	--	--	--	--
XLOC_034108	No	No	No	No	--	--	--	--
XLOC_034113	No	No	No	No	CEL58309.1	--	--	--
XLOC_034115	DEG(M)	No	No	No	--	--	--	--
XLOC_034116	No	No	No	No	--	--	--	--
XLOC_034117	No	No	No	No	CCO29128.1	--	--	--
XLOC_034121	DEG(M)	No	No	No	XP_003053142.1	--	--	--
XLOC_034122	DEG(M)	No	No	No	ETL28732.1	--	--	--
XLOC_034123	No	No	No	No	ETO63793.1	--	--	--
XLOC_034125	No	No	No	No	--	--	--	--
XLOC_034129	DEG(M)	No	No	No	EUC55388.1	--	--	--
XLOC_034140	No	No	No	No	XP_003043968.1	--	--	--
XLOC_034141	DEG(M)	No	No	No	--	--	--	--
XLOC_034144	No	No	No	No	CEL56996.1	--	--	--
XLOC_034145	DEG(M)	No	No	No	ABX75427.1	--	J	K02880

Differentially expressed gene (DEG) IDs	*C. microphyllum* root and shoot-specific (>2 fold)	*C. microphyllum* root and shoot-specific (>10 fold)	ICC4958 and *C. microphyllum* root-specific (>2 fold)	*C. microphyllum* root-specific (>2 fold)	Functional Annotation			
					NCBI nr IDs	TFs	KOG	KEGG
XLOC_034146	DEG(M)	No	No	No	NP_001165434.1	--	--	--
XLOC_034147	No	No	No	No	EUC66298.1	--	--	--
XLOC_034149	No	No	No	No	--	--	--	--
XLOC_034151	DEG(M)	No	No	No	--	--	--	--
XLOC_034153	No	No	No	No	--	--	--	--
XLOC_034155	DEG(M)	DEG(M)	No	No	XP_001948464.1	--	J	K02942
XLOC_034162	No	No	No	No	--	--	--	--
XLOC_034164	No	No	No	No	--	--	--	--
XLOC_034165	No	No	No	No	XP_004509476.1	--	--	K14457
XLOC_034169	No	No	No	No	--	--	--	--
XLOC_034172	No	No	No	No	--	--	--	--
XLOC_034173	No	No	No	No	--	--	--	--
XLOC_034175	No	No	No	No	XP_003040084.1	--	--	--
XLOC_034176	No	No	No	No	XP_003040084.1	--	--	--
XLOC_034177	No	No	No	No	--	--	--	--
XLOC_034178	DEG(M)	No	No	No	--	--	--	--
XLOC_034179	No	No	No	No	XP_004336000.1	--	J	--
XLOC_034189	No	No	No	No	--	--	--	--
XLOC_034191	No	No	No	No	EUC62348.1	--	--	--
XLOC_034192	No	No	No	No	CEL59736.1	--	--	--
XLOC_034193	DEG(M)	DEG(M)	No	No	EWY93762.1	--	--	--
XLOC_034197	No	No	No	No	XP_008911262.1	--	--	--
XLOC_034200	No	No	No	No	XP_008181434.1	--	--	--
XLOC_034203	No	No	No	No	--	--	--	--
XLOC_034207	No	No	No	No	CEL60888.1	--	--	--
XLOC_034208	DEG(M)	DEG(M)	No	No	--	--	--	--
XLOC_034209	No	No	No	No	--	--	--	--
XLOC_034210	DEG(M)	DEG(M)	No	No	XP_012077456.1	--	J	K02922
XLOC_034212	No	No	No	No	XP_008895323.1	--	--	--
XLOC_034217	DEG(M)	No	No	No	--	--	--	--
XLOC_034218	No	No	No	No	NP_001155685.1	--	A	K12891
XLOC_034220	No	No	No	No	AJQ30120.1	--	C	--
XLOC_034222	No	No	No	No	--	--	--	--
XLOC_034223	No	No	No	No	--	--	--	--
XLOC_034224	DEG(M)	No	No	No	XP_004344009.1	--	C	K02155
XLOC_034226	DEG(M)	No	No	No	--	--	--	--
XLOC_034228	No	No	No	No	CEL58874.1	--	--	--
XLOC_034234	DEG(M)	No	DEG(I+M)	DEG(M)	XP_005862709.1	--	--	--
XLOC_034236	No	No	No	No	--	--	--	--
XLOC_034238	No	No	No	No	--	--	--	--

Differentially expressed gene (DEG) IDs	*C. microphyllum* root and shoot-specific (>2 fold)	*C. microphyllum* root and shoot-specific (>10 fold)	ICC4958 and *C. microphyllum* root-specific (>2 fold)	*C. microphyllum* root-specific (>2 fold)	Functional Annotation			
					NCBI nr IDs	TFs	KOG	KEGG
XLOC_034240	DEG(M)	No	No	No	--	--	--	--
XLOC_034243	No	No	No	No	--	--	I	--
XLOC_034248	No	No	No	No	--	--	--	--
XLOC_034249	DEG(M)	DEG(M)	No	No	--	--	--	--
XLOC_034250	No	No	No	No	--	--	--	--
XLOC_034253	No	No	No	No	--	--	--	--
XLOC_034255	No	No	No	No	ELU43519.1	--	--	--
XLOC_034258	No	No	No	No	--	--	J	--
XLOC_034260	No	No	No	No	--	--	--	--
XLOC_034262	No	No	No	No	--	--	--	--
XLOC_034270	No	No	No	No	--	--	--	--
XLOC_034274	No	No	No	No	XP_004508177.1	--	--	--
XLOC_034275	No	No	No	No	--	--	--	--
XLOC_034280	DEG(M)	No	No	No	CCO33211.1	--	--	--
XLOC_034283	No	No	No	No	ETL49525.1	MYB_related	G	--
XLOC_034284	No	No	No	No	--	--	--	--
XLOC_034285	No	No	No	No	EUC59104.1	--	--	--
XLOC_034288	No	No	No	No	--	--	--	--
XLOC_034289	No	No	No	No	--	--	CE	--
XLOC_034291	No	No	No	No	EUC57178.1	--	--	K18880
XLOC_034293	DEG(M)	No	No	No	--	--	--	--
XLOC_034296	No	No	No	No	--	--	--	--
XLOC_034298	No	No	No	No	NP_001119630.1	--	J	K02908
XLOC_034299	DEG(M)	No	No	No	CEL61764.1	--	J	--
XLOC_034300	DEG(M)	No	No	No	XP_004339588.1	--	J	K02908
XLOC_034302	DEG(M)	No	No	No	--	--	--	--
XLOC_034305	DEG(M)	No	No	No	CEL58840.1	--	--	--
XLOC_034311	DEG(M)	DEG(M)	DEG(I+M)	DEG(M)	CKT03548.1	--	--	--
XLOC_034322	No	No	No	No	EUC67119.1	--	H	K01012
XLOC_034325	DEG(M)	No	No	No	XP_003050970.1	--	--	--
XLOC_034326	DEG(M)	No	No	No	--	--	--	--
XLOC_034331	No	No	No	No	--	--	G	K19073
XLOC_034332	DEG(M)	No	No	No	KDN47670.1	--	--	--
XLOC_034333	DEG(M)	No	No	No	CEL53910.1	--	--	--
XLOC_034334	DEG(M)	No	No	No	EUC63129.1	--	--	--
XLOC_034335	DEG(M)	DEG(M)	DEG(I+M)	DEG(M)	EUC63994.1	--	--	--
XLOC_034340	DEG(M)	No	No	No	AGT17884.1	--	J	--
XLOC_034341	DEG(M)	No	No	No	ELU38673.1	--	J	--
XLOC_034344	No	No	No	No	--	--	O	K04077
XLOC_034347	No	No	No	No	--	--	--	K05765

Differentially expressed gene (DEG) IDs	*C. microphyllum* root and shoot-specific (>2 fold)	*C. microphyllum* root and shoot-specific (>10 fold)	ICC4958 and *C. microphyllum* root-specific (>2 fold)	*C. microphyllum* root-specific (>2 fold)	Functional Annotation			
					NCBI nr IDs	TFs	KOG	KEGG
XLOC_034350	No	No	No	No	CEL51865.1	--	--	--
XLOC_034352	DEG(M)	No	No	No	XP_003052526.1	--	--	--
XLOC_034354	No	No	No	No	--	--	X	--
XLOC_034355	DEG(M)	No	No	No	--	--	--	--
XLOC_034364	No	No	DEG(I+M)	No	XP_004509055.1	bZIP	--	--
XLOC_034374	DEG(M)	No	No	No	--	--	--	--
XLOC_034375	No	No	No	No	--	--	--	--
XLOC_034377	DEG(M)	No	No	No	EUC66338.1	--	U	K15306
XLOC_034381	DEG(M)	No	No	No	KEP52898.1	--	--	--
XLOC_034382	No	No	No	No	XP_008916022.1	--	--	--
XLOC_034383	No	No	No	No	--	--	--	--
XLOC_034384	No	No	No	No	XP_008916022.1	--	--	--
XLOC_034385	DEG(M)	DEG(M)	No	No	--	--	--	--
XLOC_034386	No	No	No	No	CEL59118.1	--	T	--
XLOC_034388	No	No	No	No	EUC59074.1	--	--	--
XLOC_034389	DEG(M)	DEG(M)	No	No	EMT66838.1	--	--	--
XLOC_034390	DEG(M)	No	No	No	NP_001119659.1	--	--	--
XLOC_034391	No	No	No	No	--	--	--	K11000
XLOC_034394	No	No	No	No	CCO32632.1	--	--	--
XLOC_034399	No	No	No	No	--	--	--	--
XLOC_034404	No	No	No	No	--	--	--	--
XLOC_034406	No	No	No	No	--	--	--	--
XLOC_034410	No	No	No	No	ETP39391.1	--	--	--
XLOC_034414	No	No	No	No	XP_001657198.1	--	U	--
XLOC_034415	No	No	No	No	NP_001155435.1	--	O	--
XLOC_034419	No	No	No	No	--	--	--	--
XLOC_034420	No	No	No	No	--	--	--	--
XLOC_034421	DEG(M)	No	No	No	--	--	--	--
XLOC_034422	No	No	No	No	--	--	--	--
XLOC_034425	No	No	No	No	XP_011134856.1	--	J	--
XLOC_034426	No	No	No	No	--	--	--	--
XLOC_034427	No	No	No	No	--	--	--	--
XLOC_034432	DEG(M)	No	No	No	--	--	--	--
XLOC_034435	No	No	No	No	XP_001951061.1	--	--	--
XLOC_034436	No	No	No	No	CCO27120.1	--	--	K01230
XLOC_034439	DEG(M)	No	No	No	--	--	--	--
XLOC_034440	No	No	No	No	--	--	--	--
XLOC_034443	No	No	No	No	XP_012079095.1	--	O	K10666
XLOC_034444	DEG(M)	DEG(M)	No	No	--	--	--	--
XLOC_034448	No	No	No	No	ETO72763.1	--	--	--

Differentially expressed gene (DEG) IDs	*C. microphyllum* root and shoot-specific (>2 fold)	*C. microphyllum* root and shoot-specific (>10 fold)	ICC4958 and *C. microphyllum* root-specific (>2 fold)	*C. microphyllum* root-specific (>2 fold)	Functional Annotation			
					NCBI nr IDs	TFs	KOG	KEGG
XLOC_034451	No	No	No	No	--	--	--	--
XLOC_034454	No	No	No	No	--	--	--	--
XLOC_034459	No	No	No	No	--	--	--	--
XLOC_034461	No	No	No	No	XP_003040685.1	--	--	--
XLOC_034464	No	No	No	No	--	--	--	K04794
XLOC_034467	No	No	No	No	--	--	--	--
XLOC_034469	No	No	No	No	ELU39531.1	--	--	--
XLOC_034474	No	No	No	No	EUC53891.1	--	--	--
XLOC_034475	No	No	No	No	EUC53891.1	--	--	--
XLOC_034476	No	No	No	No	KDN50316.1	--	--	--
XLOC_034481	No	No	DEG(I+M)	No	XP_004500375.1	--	--	K00799
XLOC_034482	No	No	DEG(I+M)	No	--	--	--	--
XLOC_034484	DEG(M)	No	No	No	--	--	--	--
XLOC_034485	No	No	No	No	BAK00381.1	--	--	--
XLOC_034495	DEG(M)	No	No	No	--	--	--	--
XLOC_034497	DEG(M)	No	DEG(I+M)	DEG(M)	--	--	--	--
XLOC_034498	DEG(M)	DEG(M)	DEG(I+M)	DEG(M)	--	--	--	--
XLOC_034499	No	No	DEG(I+M)	No	XP_004497410.1	--	--	--
XLOC_034502	DEG(M)	No	No	No	--	--	--	--
XLOC_034503	DEG(M)	No	No	No	CEL57846.1	--	--	--
XLOC_034519	DEG(M)	DEG(M)	No	No	--	--	--	--
XLOC_034521	No	No	No	No	CEL53252.1	--	--	--
XLOC_034522	No	No	No	No	--	--	--	--
XLOC_034527	DEG(M)	No	No	No	EUC66196.1	--	--	--
XLOC_034530	DEG(M)	No	No	No	--	--	W	--
XLOC_034533	DEG(M)	No	No	No	--	--	--	--
XLOC_034538	No	No	No	No	ETI39155.1	--	--	--
XLOC_034539	No	No	No	No	ETI39155.1	--	--	--
XLOC_034541	DEG(M)	DEG(M)	DEG(I+M)	DEG(M)	WP_000203229.1	--	--	--
XLOC_034553	DEG(M)	No	No	No	--	--	--	--
XLOC_034561	No	No	No	No	XP_004500757.1	--	--	--
XLOC_034572	No	No	No	No	XP_008895985.1	--	--	--
XLOC_034577	No	No	No	No	--	--	--	--
XLOC_034580	No	No	No	No	--	--	--	--
XLOC_034581	No	No	No	No	--	--	--	--
XLOC_034582	No	No	No	No	--	--	--	--
XLOC_034583	No	No	No	No	KEP55372.1	--	--	--
XLOC_034585	No	No	No	No	--	--	--	--
XLOC_034586	No	No	No	No	--	--	--	--
XLOC_034587	No	No	DEG(I+M)	No	--	--	--	--

Differentially expressed gene (DEG) IDs	*C. microphyllum* root and shoot-specific (>2 fold)	*C. microphyllum* root and shoot-specific (>10 fold)	ICC4958 and *C. microphyllum* root-specific (>2 fold)	*C. microphyllum* root-specific (>2 fold)	Functional Annotation			
					NCBI nr IDs	TFs	KOG	KEGG
XLOC_034588	DEG(M)	No	No	No	--	--	--	--
XLOC_034595	No	No	No	No	--	--	--	--
XLOC_034596	No	No	No	No	XP_003048193.1	--	--	--
XLOC_034598	No	No	No	No	KFM70119.1	--	K	--
XLOC_034601	No	No	No	No	KEP53360.1	--	--	K11644
XLOC_034603	No	No	No	No	KNG50248.1	--	--	--
XLOC_034605	No	No	No	No	XP_002532220.1	--	R	K12200
XLOC_034606	DEG(M)	No	No	No	XP_008184469.1	--	--	--
XLOC_034608	DEG(M)	No	No	No	--	--	--	--
XLOC_034609	DEG(M)	No	No	No	XP_004503899.1	--	C	K00128
XLOC_034610	No	No	No	No	XP_002322875.1	--	C	K01647
XLOC_034611	No	No	No	No	XP_003244023.1	--	TZ	--
XLOC_034613	No	No	No	No	EUC66451.1	--	--	--
XLOC_034614	No	No	No	No	--	--	--	--
XLOC_034616	DEG(M)	No	No	No	EUC62197.1	--	--	--
XLOC_034620	No	No	No	No	XP_001653723.1	--	C	--
XLOC_034622	No	No	No	No	--	--	--	--
XLOC_034623	No	No	No	No	--	--	--	--
XLOC_034626	No	No	No	No	--	--	--	--
XLOC_034629	No	No	No	No	XP_012072842.1	--	O	K04797
XLOC_034631	DEG(M)	No	No	No	--	--	J	K02936
XLOC_034632	No	No	No	No	XP_008897749.1	--	I	--
XLOC_034636	No	No	No	No	--	--	S	--
XLOC_034638	No	No	No	No	--	--	--	--
XLOC_034640	No	No	No	No	ETI32189.1	--	--	--
XLOC_034642	No	No	No	No	--	--	--	--
XLOC_034647	No	No	No	No	--	--	--	--
XLOC_034649	No	No	No	No	XP_012068525.1	--	DKT	--
XLOC_034651	No	No	No	No	ENH73726.1	--	--	--
XLOC_034665	DEG(M)	No	No	No	--	--	--	--
XLOC_034669	No	No	No	No	--	--	O	--
XLOC_034673	DEG(M)	No	No	No	XP_002909101.1	MYB_related	O	K06689
XLOC_034674	No	No	No	No	XP_002909101.1	MYB_related	O	K06689
XLOC_034678	No	No	No	No	--	--	X	--
XLOC_034680	No	No	No	No	CEL61194.1	--	O	K04077
XLOC_034695	DEG(M)	No	No	No	--	--	--	--
XLOC_034697	DEG(M)	No	No	No	EUC65580.1	--	--	--
XLOC_034698	No	No	No	No	KCW50322.1	--	J	--
XLOC_034700	No	No	No	No	XP_003044820.1	--	--	--
XLOC_034706	No	No	No	No	--	--	--	--

Differentially expressed gene (DEG) IDs	*C. microphyllum* root and shoot-specific (>2 fold)	*C. microphyllum* root and shoot-specific (>10 fold)	ICC4958 and *C. microphyllum* root-specific (>2 fold)	*C. microphyllum* root-specific (>2 fold)	Functional Annotation			
					NCBI nr IDs	TFs	KOG	KEGG
XLOC_034713	No	No	No	No	--	--	--	--
XLOC_034721	DEG(M)	No	DEG(I+M)	DEG(M)	XP_008896121.1	--	J	K02969
XLOC_034733	No	No	No	No	--	--	--	--
XLOC_034737	DEG(M)	DEG(M)	No	No	XP_003045094.1	--	--	--
XLOC_034738	DEG(M)	No	No	No	EGU88589.1	--	--	--
XLOC_034744	DEG(M)	No	No	No	--	--	--	K05765
XLOC_034748	DEG(M)	No	No	No	XP_004486665.1	--	--	--
XLOC_034751	No	No	DEG(I+M)	No	XP_004486524.1	MYB	--	K09422
XLOC_034752	DEG(M)	No	No	No	XP_008895218.1	--	C	K05863
XLOC_034756	No	No	No	No	--	--	--	--
XLOC_034757	No	No	No	No	--	--	--	K08488
XLOC_034763	No	No	No	No	--	--	R	--
XLOC_034765	No	No	No	No	--	--	--	--
XLOC_034766	No	No	No	No	--	--	--	--
XLOC_034769	No	No	No	No	--	--	--	--
XLOC_034772	No	No	No	No	XP_003246082.1	--	C	--
XLOC_034773	No	No	No	No	XP_008916965.1	--	--	--
XLOC_034774	No	No	No	No	--	--	--	--
XLOC_034776	No	No	No	No	DAA34120.1	FAR1	O	--
XLOC_034777	No	No	No	No	XP_003046119.1	--	--	K19658
XLOC_034778	DEG(M)	No	No	No	XP_003040086.1	--	--	--
XLOC_034781	No	No	No	No	--	--	--	--
XLOC_034789	No	No	No	No	XP_004515311.1	--	--	--
XLOC_034790	DEG(M)	DEG(M)	DEG(I+M)	DEG(M)	KDN39584.1	--	--	--
XLOC_034801	No	No	No	No	KDN51648.1	--	--	--
XLOC_034818	DEG(M)	No	No	No	--	--	--	--
XLOC_034819	DEG(M)	No	No	No	KEP50237.1	--	--	--
XLOC_034820	No	No	No	No	KEP50237.1	--	--	--
XLOC_034844	No	No	No	No	--	--	--	--
XLOC_034849	No	No	No	No	--	--	V	--
XLOC_034850	No	No	No	No	--	--	--	--
XLOC_034852	DEG(M)	DEG(M)	No	No	--	--	--	--
XLOC_034856	No	No	No	No	EUC54431.1	--	--	--
XLOC_034857	No	No	No	No	EUC54431.1	--	--	--
XLOC_034859	No	No	DEG(I+M)	No	--	MIKC	G	K00847
XLOC_034868	No	No	DEG(I+M)	No	--	--	--	--
XLOC_034882	DEG(M)	No	No	No	--	--	--	--
XLOC_034884	DEG(M)	No	No	No	ETK96812.1	--	--	--
XLOC_034888	DEG(M)	DEG(M)	No	No	--	--	--	--
XLOC_034889	DEG(M)	No	No	No	AAV69396.1	--	J	--

Differentially expressed gene (DEG) IDs	*C. microphyllum* root and shoot-specific (>2 fold)	*C. microphyllum* root and shoot-specific (>10 fold)	ICC4958 and *C. microphyllum* root-specific (>2 fold)	*C. microphyllum* root-specific (>2 fold)	Functional Annotation			
					NCBI nr IDs	TFs	KOG	KEGG
XLOC_034890	DEG(M)	DEG(M)	DEG(I+M)	DEG(M)	XP_009524206.1	--	J	--
XLOC_034892	DEG(M)	No	No	No	KDN49362.1	--	--	K11252
XLOC_034903	DEG(M)	No	DEG(I+M)	DEG(M)	CDY70361.1	YABBY	OR	--
XLOC_034905	DEG(M)	No	No	No	--	--	V	--
XLOC_034907	DEG(M)	DEG(M)	No	No	XP_004512636.1	--	--	--
XLOC_034912	DEG(M)	No	No	No	--	--	--	--
XLOC_034913	DEG(M)	No	No	No	--	--	--	--
XLOC_034919	DEG(M)	No	No	No	CEL56213.1	B3	J	K02921
XLOC_034920	DEG(M)	No	No	No	CEL56213.1	B3	J	K02921
XLOC_034931	No	No	No	No	--	bZIP	G	K01792
XLOC_034932	No	No	No	No	--	--	O	--
XLOC_034933	DEG(M)	No	No	No	--	--	--	--
XLOC_034941	No	No	No	No	--	--	--	--
XLOC_034945	No	No	No	No	--	--	--	--
XLOC_034949	DEG(M)	No	No	No	KEP54662.1	--	--	--
XLOC_034951	No	No	No	No	XP_003626512.1	--	--	K03046
XLOC_034952	No	No	No	No	XP_003739759.1	--	E	--
XLOC_034953	DEG(M)	No	No	No	XP_001947860.1	--	--	--
XLOC_034954	DEG(M)	No	No	No	--	--	GC	--
XLOC_034957	No	No	No	No	XP_008180390.1	--	C	--
XLOC_034958	No	No	DEG(I+M)	No	--	--	--	--
XLOC_034960	No	No	No	No	--	--	--	--
XLOC_034961	DEG(M)	No	No	No	--	--	--	--
XLOC_034966	DEG(M)	No	No	No	NP_001129413.1	--	J	K02985
XLOC_034967	No	No	No	No	--	--	--	--
XLOC_034968	No	No	No	No	--	--	--	--
XLOC_034970	No	No	No	No	--	--	K	K11086
XLOC_034971	No	No	No	No	KDN38664.1	--	--	--
XLOC_034973	No	No	No	No	CCO33951.1	--	--	K01267
XLOC_034974	No	No	No	No	--	--	--	--
XLOC_034976	No	No	No	No	EUC67179.1	--	--	K20221
XLOC_034981	DEG(M)	No	No	No	--	--	--	--
XLOC_034983	No	No	No	No	XP_001944025.1	--	EG	--
XLOC_034986	No	No	No	No	--	--	--	--
XLOC_034992	No	No	No	No	--	--	F	K01951
XLOC_034993	No	No	No	No	NP_001155477.1	--	--	--
XLOC_034997	DEG(M)	DEG(M)	No	No	XP_004487722.1	--	--	--
XLOC_035000	No	No	No	No	--	--	--	--
XLOC_035011	No	No	No	No	--	--	--	--
XLOC_035012	DEG(M)	DEG(M)	No	No	--	--	--	--

Differentially expressed gene (DEG) IDs	*C. microphyllum* root and shoot-specific (>2 fold)	*C. microphyllum* root and shoot-specific (>10 fold)	ICC4958 and *C. microphyllum* root-specific (>2 fold)	*C. microphyllum* root-specific (>2 fold)	Functional Annotation			
					NCBI nr IDs	TFs	KOG	KEGG
XLOC_035015	No	No	No	No	XP_008900434.1	--	--	--
XLOC_035019	No	No	No	No	EYC15691.1	--	F	K01490
XLOC_035021	No	No	No	No	--	--	--	--
XLOC_035024	No	No	No	No	--	--	--	K00025
XLOC_035025	No	No	No	No	KDN46053.1	--	--	--
XLOC_035028	No	No	No	No	--	--	--	--
XLOC_035029	No	No	No	No	--	--	P	--
XLOC_035030	No	No	No	No	--	--	--	--
XLOC_035031	No	No	No	No	--	--	O	--
XLOC_035032	No	No	No	No	XP_004503977.1	--	R	--
XLOC_035039	No	No	No	No	KFM66198.1	--	E	K00030
XLOC_035044	No	No	No	No	--	--	--	--
XLOC_035046	DEG(M)	No	No	No	--	--	--	--
XLOC_035049	No	No	No	No	--	--	--	K00844
XLOC_035053	No	No	No	No	XP_003045066.1	--	--	--
XLOC_035055	No	No	No	No	--	--	--	--
XLOC_035058	No	No	No	No	NP_001155655.1	--	--	K12881
XLOC_035060	No	No	No	No	--	--	A	--
XLOC_035066	No	No	No	No	--	--	--	--
XLOC_035067	No	No	No	No	XP_004355015.1	--	Z	K10365
XLOC_035070	No	No	No	No	--	--	--	--
XLOC_035071	No	No	No	No	--	--	--	K01369
XLOC_035072	No	No	No	No	--	--	--	--
XLOC_035073	No	No	No	No	--	--	--	--
XLOC_035075	DEG(M)	DEG(M)	No	No	NP_001119663.1	--	--	--
XLOC_035093	DEG(M)	No	No	No	CEL51878.1	--	--	--
XLOC_035096	DEG(M)	No	No	No	AAL40718.1	--	--	--
XLOC_035097	No	No	DEG(I+M)	No	--	--	T	--
XLOC_035098	DEG(M)	DEG(M)	DEG(I+M)	DEG(M)	--	--	--	--
XLOC_035106	DEG(M)	No	No	No	--	--	--	--
XLOC_035113	DEG(M)	No	DEG(I+M)	DEG(M)	XP_004511941.1	--	--	--
XLOC_035114	DEG(M)	No	DEG(I+M)	DEG(M)	XP_004511941.1	--	--	--
XLOC_035115	DEG(M)	DEG(M)	DEG(I+M)	DEG(M)	XP_004511942.1	--	--	--
XLOC_035130	DEG(M)	No	No	No	XP_004486692.1	--	--	--
XLOC_035134	No	No	DEG(I+M)	No	XP_012572015.1	--	--	--
XLOC_035139	DEG(M)	DEG(M)	No	No	--	--	--	--
XLOC_035140	DEG(M)	DEG(M)	No	No	--	--	--	--
XLOC_035141	No	No	No	No	EUC65543.1	--	--	--
XLOC_035142	No	No	No	No	KDN46053.1	--	--	--
XLOC_035143	DEG(M)	No	No	No	KEP54991.1	--	--	--

Differentially expressed gene (DEG) IDs	*C. microphyllum* root and shoot-specific (>2 fold)	*C. microphyllum* root and shoot-specific (>10 fold)	ICC4958 and *C. microphyllum* root-specific (>2 fold)	*C. microphyllum* root-specific (>2 fold)	Functional Annotation			
					NCBI nr IDs	TFs	KOG	KEGG
XLOC_035149	DEG(M)	DEG(M)	DEG(I+M)	DEG(M)	XP_008905363.1	--	J	K02995
XLOC_035155	No	No	No	No	--	--	--	--
XLOC_035170	No	No	No	No	--	--	--	--
XLOC_035188	DEG(M)	No	No	No	KDN41087.1	--	G	--
XLOC_035196	DEG(M)	DEG(M)	DEG(I+M)	DEG(M)	--	--	J	K02943
XLOC_035197	DEG(M)	DEG(M)	DEG(I+M)	DEG(M)	ETO79552.1	--	--	--
XLOC_035198	DEG(M)	DEG(M)	DEG(I+M)	DEG(M)	XP_009513921.1	--	--	K02941
XLOC_035199	DEG(M)	DEG(M)	DEG(I+M)	DEG(M)	--	--	--	--
XLOC_035200	DEG(M)	No	No	No	XP_008904900.1	--	--	K02941
XLOC_035212	DEG(M)	No	No	No	NP_001155749.1	--	J	K02938
XLOC_035226	No	No	No	No	CEF68345.1	--	C	--
XLOC_035233	DEG(M)	No	No	No	--	--	--	--
XLOC_035237	No	No	No	No	KDN36337.1	--	--	--
XLOC_035238	DEG(M)	No	No	No	--	--	--	--
XLOC_035244	DEG(M)	No	DEG(I+M)	DEG(M)	WP_029550487.1	--	--	--
XLOC_035245	No	No	No	No	--	--	--	--
XLOC_035246	DEG(M)	No	No	No	KEP47406.1	--	--	--
XLOC_035249	DEG(M)	No	No	No	--	--	--	--
XLOC_035256	DEG(M)	No	No	No	--	--	--	--
XLOC_035257	DEG(M)	No	No	No	--	--	--	--
XLOC_035259	DEG(M)	No	No	No	EUC62348.1	--	--	--
XLOC_035260	No	No	No	No	--	--	--	--
XLOC_035266	No	No	No	No	--	--	--	--
XLOC_035267	No	No	No	No	XP_002414702.1	--	C	--
XLOC_035268	DEG(M)	No	No	No	EUC56856.1	--	--	--
XLOC_035269	No	No	No	No	XP_008911228.1	--	--	K03955
XLOC_035270	DEG(M)	No	No	No	--	--	--	--
XLOC_035272	No	No	No	No	XP_004485884.1	--	--	K02914
XLOC_035273	No	No	No	No	--	--	--	--
XLOC_035274	No	No	No	No	EUC65670.1	--	--	--
XLOC_035275	No	No	No	No	--	--	--	--
XLOC_035277	DEG(M)	No	No	No	KEP51106.1	--	--	--
XLOC_035280	No	No	No	No	CEL57784.1	--	--	--
XLOC_035288	DEG(M)	No	No	No	--	--	--	--
XLOC_035289	DEG(M)	No	No	No	CCO26137.1	--	O	--
XLOC_035292	No	No	No	No	ETI39765.1	--	--	--
XLOC_035294	No	No	No	No	--	--	--	--
XLOC_035297	No	No	No	No	EUC62287.1	--	--	--
XLOC_035302	DEG(M)	No	No	No	--	--	--	--
XLOC_035303	No	No	DEG(I+M)	No	XP_004515633.1	C2H2	--	--

Differentially expressed gene (DEG) IDs	***C. microphyllum* root and shoot-specific (>2 fold)**	***C. microphyllum* root and shoot-specific (>10 fold)**	**ICC4958 and *C. microphyllum* root-specific (>2 fold)**	***C. microphyllum* root-specific (>2 fold)**	**Functional Annotation**			
					**NCBI nr IDs**	**TFs**	**KOG**	**KEGG**
XLOC_035307	No	No	DEG(I+M)	No	--	--	--	--
XLOC_035308	No	No	No	No	XP_008903378.1	--	--	--
XLOC_035310	No	No	No	No	XP_004511340.1	HD-ZIP	K	--
XLOC_035314	No	No	No	No	EUC62125.1	--	--	--
XLOC_035319	No	No	No	No	--	--	--	--
XLOC_035320	No	No	No	No	KDN40214.1	--	--	--
XLOC_035321	No	No	No	No	XP_004514371.1	--	--	--
XLOC_035324	No	No	No	No	--	--	--	--
XLOC_035325	No	No	No	No	ABF18415.1	--	--	--
XLOC_035328	No	No	No	No	--	--	--	K05759
XLOC_035330	No	No	No	No	--	--	O	--
XLOC_035331	No	No	No	No	--	--	--	--
XLOC_035333	No	No	No	No	--	--	--	--
XLOC_035336	DEG(M)	No	No	No	NP_001153821.1	--	T	--
XLOC_035337	No	No	No	No	CEL54221.1	--	--	--
XLOC_035338	No	No	No	No	CEL54221.1	--	--	--
XLOC_035339	No	No	No	No	ETK93128.1	--	--	--
XLOC_035340	No	No	No	No	--	--	--	--
XLOC_035343	No	No	No	No	--	--	--	--
XLOC_035349	No	No	No	No	ETP36514.1	--	--	--
XLOC_035350	No	No	No	No	--	--	R	K03255
XLOC_035351	DEG(M)	No	No	No	--	--	--	--
XLOC_035353	No	No	No	No	--	--	--	--
XLOC_035358	DEG(M)	No	No	No	--	--	--	--
XLOC_035359	DEG(M)	No	No	No	--	--	--	--
XLOC_035371	DEG(M)	No	DEG(I+M)	DEG(M)	--	Nin-like	Q	--
XLOC_035373	No	No	No	No	--	--	--	--
XLOC_035374	DEG(M)	No	No	No	CCO32912.1	--	--	--
XLOC_035376	No	No	No	No	EUC59769.1	--	E	--
XLOC_035377	No	No	No	No	--	--	--	--
XLOC_035378	DEG(M)	No	No	No	--	--	--	--
XLOC_035379	DEG(M)	No	DEG(I+M)	DEG(M)	--	--	--	--
XLOC_035383	DEG(M)	DEG(M)	No	No	XP_003050400.1	--	E	K00262
XLOC_035384	No	No	No	No	XP_001655563.1	--	TZ	--
XLOC_035385	DEG(M)	No	No	No	--	--	--	--
XLOC_035386	No	No	DEG(I+M)	No	--	--	P	K17470
XLOC_035387	No	No	No	No	--	--	P	--
XLOC_035390	No	No	No	No	--	--	--	--
XLOC_035391	No	No	No	No	XP_003047113.1	--	--	--
XLOC_035392	No	No	No	No	--	--	--	--

Differentially expressed gene (DEG) IDs	*C. microphyllum* root and shoot-specific (>2 fold)	*C. microphyllum* root and shoot-specific (>10 fold)	ICC4958 and *C. microphyllum* root-specific (>2 fold)	*C. microphyllum* root-specific (>2 fold)	Functional Annotation			
					NCBI nr IDs	TFs	KOG	KEGG
XLOC_035398	No	No	No	No	--	--	--	--
XLOC_035403	No	No	No	No	KEP54436.1	--	J	K02998
XLOC_035405	No	No	No	No	XP_001662644.1	--	L	K04802
XLOC_035406	No	No	No	No	--	--	J	--
XLOC_035407	DEG(M)	DEG(M)	No	No	--	--	--	--
XLOC_035416	No	No	No	No	KEP52580.1	--	--	--
XLOC_035421	No	No	No	No	XP_001941546.1	--	--	--
XLOC_035423	No	No	No	No	--	--	--	--
XLOC_035426	No	No	No	No	EUC54127.1	--	--	K15111
XLOC_035428	No	No	No	No	--	--	T	--
XLOC_035430	No	No	No	No	XP_001952229.3	--	--	--
XLOC_035431	No	No	No	No	XP_001952229.3	--	--	--
XLOC_035432	No	No	No	No	--	--	--	--
XLOC_035434	No	No	No	No	--	--	--	--
XLOC_035435	No	No	No	No	--	--	X	--
XLOC_035438	DEG(M)	No	No	No	CCO33518.1	--	--	--
XLOC_035441	No	No	No	No	--	--	--	--
XLOC_035442	No	No	No	No	XP_009525002.1	--	E	--
XLOC_035444	No	No	No	No	XP_009535973.1	--	--	--
XLOC_035445	No	No	No	No	XP_003050750.1	--	--	--
XLOC_035446	No	No	No	No	EXL96360.1	--	--	--
XLOC_035447	DEG(M)	DEG(M)	DEG(I+M)	DEG(M)	--	--	--	--
XLOC_035448	No	No	No	No	--	--	O	K01802
XLOC_035450	DEG(M)	DEG(M)	No	No	EMT73096.1	--	--	--
XLOC_035451	No	No	No	No	--	--	--	--
XLOC_035463	DEG(M)	DEG(M)	DEG(I+M)	DEG(M)	XP_012747850.1	--	Z	--
XLOC_035464	DEG(M)	No	No	No	EXL56994.1	--	--	--
XLOC_035473	No	No	No	No	CCO27823.1	MYB_related	O	K06689
XLOC_035482	No	No	No	No	--	--	--	--
XLOC_035486	No	No	No	No	EUC53781.1	--	--	--
XLOC_035490	No	No	No	No	KLO84325.1	--	--	K01610
XLOC_035497	No	No	No	No	XP_003048929.1	--	--	--
XLOC_035498	DEG(M)	DEG(M)	No	No	EGU75709.1	--	--	--
XLOC_035499	No	No	No	No	XP_003048929.1	--	--	--
XLOC_035507	No	No	DEG(I+M)	No	--	--	--	--
XLOC_035509	DEG(M)	DEG(M)	No	No	--	--	--	--
XLOC_035511	No	No	No	No	--	--	--	--
XLOC_035513	DEG(M)	No	No	No	--	--	--	--
XLOC_035514	DEG(M)	DEG(M)	No	No	--	--	--	--
XLOC_035525	No	No	No	No	XP_002400999.1	--	K	--

Differentially expressed gene (DEG) IDs	*C. microphyllum* root and shoot-specific (>2 fold)	*C. microphyllum* root and shoot-specific (>10 fold)	ICC4958 and *C. microphyllum* root-specific (>2 fold)	*C. microphyllum* root-specific (>2 fold)	Functional Annotation			
					NCBI nr IDs	TFs	KOG	KEGG
XLOC_035532	No	No	No	No	CCO35687.1	--	G	K00688
XLOC_035533	No	No	No	No	CCO35687.1	--	G	--
XLOC_035535	No	No	No	No	CCO35687.1	--	--	--
XLOC_035537	DEG(M)	No	No	No	KDN36371.1	--	--	--
XLOC_035540	No	No	No	No	XP_004506950.1	--	--	K00507
XLOC_035542	No	No	No	No	--	--	--	--
XLOC_035549	No	No	No	No	XP_004339218.1	--	O	K03094
XLOC_035554	DEG(M)	No	No	No	--	--	--	--
XLOC_035575	DEG(M)	No	No	No	CEF69749.1	--	W	--
XLOC_035576	No	No	No	No	--	--	--	--
XLOC_035585	DEG(M)	No	No	No	--	--	--	K03671
XLOC_035586	No	No	DEG(I+M)	No	XP_004514814.1	--	C	--
XLOC_035588	No	No	No	No	KDN44213.1	--	--	--
XLOC_035589	No	No	No	No	XP_001656786.1	--	DY	--
XLOC_035590	No	No	No	No	ETL39746.1	--	--	--
XLOC_035596	DEG(M)	No	DEG(I+M)	DEG(M)	EWY92425.1	--	--	--
XLOC_035597	No	No	No	No	--	--	--	--
XLOC_035598	No	No	No	No	CCO37774.1	--	--	K01711
XLOC_035601	No	No	No	No	CCO27741.1	--	YU	--
XLOC_035604	DEG(M)	DEG(M)	DEG(I+M)	DEG(M)	--	--	--	--
XLOC_035609	No	No	No	No	--	--	--	--
XLOC_035611	No	No	No	No	--	--	--	--
XLOC_035613	No	No	No	No	ERG81045.1	--	J	--
XLOC_035614	No	No	No	No	--	--	--	K02882
XLOC_035617	No	No	No	No	XP_010535074.1	--	--	K09286
XLOC_035619	No	No	No	No	--	--	--	--
XLOC_035626	No	No	No	No	KIO30285.1	--	--	K15104
XLOC_035628	DEG(M)	DEG(M)	No	No	--	--	--	--
XLOC_035629	DEG(M)	No	No	No	KDN43883.1	--	--	--
XLOC_035630	DEG(M)	No	No	No	CCO28402.1	--	--	--
XLOC_035638	No	No	No	No	--	--	--	K03881
XLOC_035639	No	No	No	No	XP_003044505.1	--	--	--
XLOC_035641	DEG(M)	No	No	No	--	--	--	--
XLOC_035644	DEG(M)	No	No	No	--	--	--	--
XLOC_035646	No	No	No	No	CCO34474.1	--	--	--
XLOC_035648	DEG(M)	No	No	No	--	--	--	K05759
XLOC_035649	No	No	No	No	XP_001650059.1	--	--	--
XLOC_035652	No	No	No	No	XP_012087495.1	--	A	K10661
XLOC_035654	No	No	No	No	NP_001135966.1	--	AJ	K14564
XLOC_035655	No	No	DEG(I+M)	No	XP_004496979.2	--	--	--

Differentially expressed gene (DEG) IDs	*C. microphyllum* root and shoot-specific (>2 fold)	*C. microphyllum* root and shoot-specific (>10 fold)	ICC4958 and *C. microphyllum* root-specific (>2 fold)	*C. microphyllum* root-specific (>2 fold)	Functional Annotation			
					NCBI nr IDs	TFs	KOG	KEGG
XLOC_035660	No	No	No	No	CCO27047.1	--	C	K03940
XLOC_035663	No	No	No	No	--	--	--	--
XLOC_035665	No	No	No	No	EUC67244.1	--	DZU	--
XLOC_035672	No	No	No	No	--	--	--	--
XLOC_035674	No	No	No	No	XP_001648534.1	--	--	--
XLOC_035677	DEG(M)	No	No	No	--	--	--	--
XLOC_035678	No	No	No	No	--	--	--	--
XLOC_035679	No	No	No	No	XP_001640755.1	--	O	--
XLOC_035680	No	No	No	No	--	--	--	--
XLOC_035682	DEG(M)	No	No	No	--	--	--	--
XLOC_035684	DEG(M)	No	No	No	--	--	--	--
XLOC_035687	No	No	No	No	--	--	--	--
XLOC_035693	No	No	No	No	CEL59439.1	--	--	--
XLOC_035694	No	No	No	No	--	--	--	--
XLOC_035696	No	No	No	No	XP_001952123.1	--	I	--
XLOC_035698	DEG(M)	No	No	No	--	--	--	--
XLOC_035701	No	No	No	No	YP_002149719.1	--	C	K02114
XLOC_035704	DEG(M)	No	No	No	XP_008915854.1	--	O	--
XLOC_035706	No	No	No	No	ETP46452.1	--	--	--
XLOC_035712	No	No	No	No	AGE97646.1	--	--	--
XLOC_035714	No	No	No	No	--	--	--	--
XLOC_035717	No	No	No	No	--	--	--	--
XLOC_035719	No	No	No	No	--	--	--	--
XLOC_035720	No	No	No	No	EUC66649.1	--	--	--
XLOC_035722	DEG(M)	No	No	No	XP_008891718.1	--	--	K03233
XLOC_035723	DEG(M)	No	No	No	XP_008878745.1	--	O	K01802
XLOC_035726	No	No	No	No	KFM70178.1	FAR1	TR	--
XLOC_035732	No	No	No	No	XP_009528678.1	--	--	K07512
XLOC_035733	DEG(M)	No	No	No	--	--	V	K13082
XLOC_035735	No	No	No	No	--	--	S	--
XLOC_035736	No	No	No	No	EMD94804.1	--	--	--
XLOC_035737	DEG(M)	No	No	No	--	--	--	--
XLOC_035738	No	No	No	No	--	--	--	--
XLOC_035739	DEG(M)	No	No	No	CEL56886.1	--	--	--
XLOC_035740	No	No	No	No	NP_001156725.1	--	I	--
XLOC_035741	No	No	No	No	EUC62349.1	--	--	--
XLOC_035742	No	No	No	No	KEP53800.1	--	--	--
XLOC_035745	No	No	No	No	--	--	--	--
XLOC_035746	DEG(M)	No	No	No	--	--	--	K12741
XLOC_035751	DEG(M)	DEG(M)	No	No	NP_001155405.1	--	J	K02957

Differentially expressed gene (DEG) IDs	*C. microphyllum* root and shoot-specific (>2 fold)	*C. microphyllum* root and shoot-specific (>10 fold)	ICC4958 and *C. microphyllum* root-specific (>2 fold)	*C. microphyllum* root-specific (>2 fold)	Functional Annotation			
					NCBI nr IDs	TFs	KOG	KEGG
XLOC_035754	No	No	No	No	--	--	--	K01674
XLOC_035755	No	No	No	No	KEP49216.1	--	--	--
XLOC_035756	No	No	No	No	XP_012570426.1	--	--	--
XLOC_035757	DEG(M)	No	No	No	KEP49171.1	--	--	--
XLOC_035762	DEG(M)	No	No	No	--	--	--	K17609
XLOC_035763	No	No	No	No	--	--	O	K00799
XLOC_035766	No	No	No	No	CEL55267.1	--	--	--
XLOC_035769	No	No	No	No	XP_001943996.1	--	C	K00029
XLOC_035770	No	No	No	No	--	--	--	--
XLOC_035771	No	No	DEG(I+M)	No	--	--	--	--
XLOC_035772	No	No	No	No	ETO76250.1	--	--	--
XLOC_035773	DEG(M)	No	No	No	--	--	--	--
XLOC_035775	No	No	No	No	KDN46891.1	--	--	--
XLOC_035776	No	No	No	No	--	--	--	--
XLOC_035778	No	No	No	No	--	--	J	K02937
XLOC_035784	No	No	No	No	--	--	--	--
XLOC_035785	No	No	No	No	EUC57269.1	--	--	--
XLOC_035786	No	No	No	No	EUC59099.1	--	--	--
XLOC_035788	DEG(M)	No	No	No	KDN44849.1	--	--	--
XLOC_035789	No	No	No	No	--	--	--	--
XLOC_035791	No	No	No	No	XP_001950287.1	--	--	--
XLOC_035793	DEG(M)	No	No	No	--	--	--	--
XLOC_035794	DEG(M)	No	No	No	NP_001156049.1	--	--	K09250
XLOC_035796	DEG(M)	No	DEG(I+M)	DEG(M)	--	--	--	--
XLOC_035797	No	No	No	No	XP_003044942.1	--	--	--
XLOC_035800	No	No	No	No	XP_003051586.1	--	I	K19706
XLOC_035801	No	No	DEG(I+M)	No	--	--	R	--
XLOC_035810	No	No	No	No	XP_008908834.1	--	--	--
XLOC_035812	No	No	No	No	--	--	--	--
XLOC_035815	No	No	No	No	--	--	--	--
XLOC_035816	No	No	No	No	XP_009514619.1	--	--	--
XLOC_035820	DEG(M)	DEG(M)	No	No	--	--	--	--
XLOC_035825	No	No	No	No	--	--	--	--
XLOC_035829	DEG(M)	DEG(M)	No	No	--	--	--	K00430
XLOC_035830	DEG(M)	DEG(M)	DEG(I+M)	DEG(M)	XP_004491048.1	--	--	K00430
XLOC_035833	No	No	No	No	--	--	--	--
XLOC_035836	DEG(M)	DEG(M)	No	No	KNG44950.1	--	--	--
XLOC_035840	No	No	No	No	--	--	--	--
XLOC_035843	DEG(M)	No	No	No	--	--	--	--
XLOC_035844	No	No	No	No	--	--	--	K18753

Differentially expressed gene (DEG) IDs	*C. microphyllum* root and shoot-specific (>2 fold)	*C. microphyllum* root and shoot-specific (>10 fold)	ICC4958 and *C. microphyllum* root-specific (>2 fold)	*C. microphyllum* root-specific (>2 fold)	Functional Annotation			
					NCBI nr IDs	TFs	KOG	KEGG
XLOC_035846	No	No	No	No	--	--	DZU	--
XLOC_035847	No	No	No	No	KDN37182.1	--	--	--
XLOC_035848	No	No	No	No	--	--	R	--
XLOC_035851	No	No	No	No	XP_008893560.1	--	--	--
XLOC_035852	No	No	No	No	--	--	--	K01113
XLOC_035853	No	No	No	No	NP_001233064.1	--	--	--
XLOC_035856	DEG(M)	DEG(M)	DEG(I+M)	DEG(M)	EUC55601.1	--	--	--
XLOC_035857	No	No	No	No	AAR85527.1	--	O	--
XLOC_035861	DEG(M)	No	No	No	--	--	--	K00228
XLOC_035865	DEG(M)	No	No	No	KEP49713.1	--	--	--
XLOC_035866	No	No	No	No	KDN50541.1	--	--	--
XLOC_035867	No	No	No	No	--	--	--	K00851
XLOC_035877	No	No	No	No	--	--	--	K07407
XLOC_035878	No	No	No	No	--	--	--	--
XLOC_035879	No	No	DEG(I+M)	No	XP_004489605.1	--	--	--
XLOC_035882	No	No	No	No	XP_003044567.1	--	--	--
XLOC_035884	No	No	No	No	--	--	--	--
XLOC_035885	DEG(M)	No	No	No	ETI33541.1	--	--	K01874
XLOC_035887	DEG(M)	No	DEG(I+M)	DEG(M)	XP_002530623.1	--	X	K06617
XLOC_035890	DEG(M)	No	No	No	KDN49290.1	--	--	--
XLOC_035891	DEG(M)	No	No	No	--	--	--	--
XLOC_035893	No	No	No	No	--	--	--	--
XLOC_035894	No	No	No	No	--	--	--	--
XLOC_035895	No	No	No	No	--	--	--	--
XLOC_035900	DEG(M)	No	No	No	XP_001657955.1	--	J	--
XLOC_035902	No	No	DEG(I+M)	No	XP_004514892.1	--	--	--
XLOC_035905	No	No	No	No	--	--	--	--
XLOC_035908	No	No	DEG(I+M)	No	--	--	--	--
XLOC_035909	No	No	No	No	XP_012264348.1	--	O	--
XLOC_035921	No	No	No	No	--	--	--	--
XLOC_035923	No	No	No	No	XP_004490027.1	--	--	--
XLOC_035924	DEG(M)	No	No	No	XP_001371286.2	--	J	--
XLOC_035925	DEG(M)	No	No	No	XP_003052057.1	--	J	--
XLOC_035930	DEG(M)	No	No	No	--	--	--	--
XLOC_035932	DEG(M)	No	No	No	--	--	--	--
XLOC_035933	DEG(M)	No	No	No	KEP46877.1	--	B	--
XLOC_035936	No	No	No	No	XP_001950339.2	--	--	--
XLOC_035940	DEG(M)	No	No	No	--	--	--	--
XLOC_035947	No	No	No	No	CEL57402.1	--	--	--
XLOC_035948	DEG(M)	No	No	No	--	--	--	--

Differentially expressed gene (DEG) IDs	*C. microphyllum* root and shoot-specific (>2 fold)	*C. microphyllum* root and shoot-specific (>10 fold)	ICC4958 and *C. microphyllum* root-specific (>2 fold)	*C. microphyllum* root-specific (>2 fold)	Functional Annotation			
					NCBI nr IDs	TFs	KOG	KEGG
XLOC_035949	DEG(M)	No	No	No	--	--	--	--
XLOC_035952	DEG(M)	No	DEG(I+M)	DEG(M)	--	--	--	--
XLOC_035960	DEG(M)	DEG(M)	DEG(I+M)	DEG(M)	--	--	--	--
XLOC_035961	DEG(M)	DEG(M)	DEG(I+M)	DEG(M)	--	--	--	--
XLOC_035966	No	No	No	No	--	--	--	K13754
XLOC_035967	No	No	No	No	XP_004493218.1	--	--	--
XLOC_035972	No	No	No	No	--	--	E	K00605
XLOC_035977	DEG(M)	No	No	No	--	--	--	--
XLOC_035989	No	No	No	No	KEP51264.1	--	--	--
XLOC_035990	No	No	No	No	CCO30526.1	--	--	--
XLOC_035992	No	No	DEG(I+M)	No	XP_004515856.1	--	--	--
XLOC_035995	No	No	No	No	--	--	--	--
XLOC_036016	No	No	No	No	--	--	--	--
XLOC_036017	DEG(M)	No	No	No	ETL90554.1	--	--	--
XLOC_036021	DEG(M)	No	No	No	ELU36233.1	--	--	--
XLOC_036022	No	No	No	No	XP_008901482.1	--	--	K00434
XLOC_036032	DEG(M)	No	No	No	--	--	--	--
XLOC_036033	No	No	DEG(I+M)	No	--	--	G	K01087
XLOC_036036	No	No	DEG(I+M)	No	XP_004513754.1	--	--	--
XLOC_036037	DEG(M)	No	No	No	--	--	--	--
XLOC_036040	DEG(M)	DEG(M)	DEG(I+M)	DEG(M)	XP_002903617.1	--	J	K02989
XLOC_036041	DEG(M)	DEG(M)	DEG(I+M)	DEG(M)	--	--	--	--
XLOC_036042	DEG(M)	No	DEG(I+M)	DEG(M)	--	--	--	--
XLOC_036045	No	No	No	No	--	--	--	--
XLOC_036047	DEG(M)	No	No	No	KEP49771.1	--	--	--
XLOC_036050	No	No	No	No	--	--	--	--
XLOC_036053	No	No	No	No	EUC65388.1	--	K	--
XLOC_036055	No	No	No	No	XP_002321182.2	--	X	--
XLOC_036056	No	No	No	No	KHN88363.1	--	J	--
XLOC_036057	No	No	No	No	--	--	--	--
XLOC_036058	No	No	No	No	KFM74612.1	--	E	--
XLOC_036060	No	No	No	No	NP_001156833.1	--	--	--
XLOC_036063	No	No	No	No	--	--	--	--
XLOC_036066	No	No	No	No	--	--	--	--
XLOC_036069	No	No	No	No	ETK83815.1	--	--	K14272
XLOC_036075	No	No	No	No	--	--	--	--
XLOC_036077	DEG(M)	No	No	No	--	--	--	--
XLOC_036078	DEG(M)	No	No	No	XP_003977419.2	--	--	K11253
XLOC_036079	No	No	No	No	--	--	--	--
XLOC_036084	No	No	No	No	EMT74514.1	--	--	--

Differentially expressed gene (DEG) IDs	*C. microphyllum* root and shoot-specific (>2 fold)	*C. microphyllum* root and shoot-specific (>10 fold)	ICC4958 and *C. microphyllum* root-specific (>2 fold)	*C. microphyllum* root-specific (>2 fold)	Functional Annotation			
					NCBI nr IDs	TFs	KOG	KEGG
XLOC_036088	No	No	No	No	--	--	O	--
XLOC_036094	No	No	No	No	--	--	--	--
XLOC_036101	DEG(M)	No	No	No	--	--	--	K01184
XLOC_036102	DEG(M)	No	No	No	KDN42702.1	--	--	--
XLOC_036104	No	No	No	No	--	--	Q	--
XLOC_036108	DEG(M)	DEG(M)	DEG(I+M)	DEG(M)	CEL61021.1	--	--	--
XLOC_036112	DEG(M)	No	No	No	--	--	--	--
XLOC_036114	DEG(M)	No	No	No	--	--	--	--
XLOC_036115	No	No	No	No	--	--	--	--
XLOC_036116	No	No	No	No	--	--	--	--
XLOC_036117	DEG(M)	No	No	No	--	--	--	--
XLOC_036118	No	No	No	No	XP_002532375.1	bHLH	RK	--
XLOC_036122	No	No	No	No	EUC61023.1	--	--	--
XLOC_036123	No	No	No	No	--	--	--	--
XLOC_036124	No	No	No	No	--	--	--	--
XLOC_036125	No	No	No	No	--	--	--	K03872
XLOC_036126	DEG(M)	No	No	No	--	--	--	--
XLOC_036128	No	No	No	No	--	--	--	--
XLOC_036129	No	No	No	No	--	--	--	--
XLOC_036134	No	No	No	No	--	--	--	--
XLOC_036135	No	No	No	No	XP_007155169.1	NF-YB	K	--
XLOC_036136	No	No	No	No	--	--	--	--
XLOC_036137	No	No	No	No	--	--	--	--
XLOC_036139	DEG(M)	No	No	No	--	--	--	--
XLOC_036144	No	No	No	No	--	--	--	--
XLOC_036146	DEG(M)	DEG(M)	No	No	EGU85094.1	--	--	--
XLOC_036148	DEG(M)	No	No	No	XP_001943756.1	--	--	--
XLOC_036150	No	No	No	No	--	--	--	--
XLOC_036152	No	No	No	No	--	--	--	--
XLOC_036153	No	No	No	No	--	--	--	--
XLOC_036155	No	No	No	No	--	--	--	--
XLOC_036159	DEG(M)	No	No	No	--	--	J	K02932
XLOC_036162	No	No	No	No	--	--	--	--
XLOC_036163	No	No	No	No	--	--	--	--
XLOC_036165	DEG(M)	No	No	No	--	--	--	--
XLOC_036167	No	No	No	No	EUC67164.1	--	--	K09832
XLOC_036170	No	No	No	No	--	--	--	--
XLOC_036171	No	No	No	No	XP_001648541.2	--	--	--
XLOC_036172	No	No	No	No	--	--	--	--
XLOC_036174	No	No	No	No	XP_001950745.1	--	O	--

Differentially expressed gene (DEG) IDs	*C. microphyllum* root and shoot-specific (>2 fold)	*C. microphyllum* root and shoot-specific (>10 fold)	ICC4958 and *C. microphyllum* root-specific (>2 fold)	*C. microphyllum* root-specific (>2 fold)	Functional Annotation			
					NCBI nr IDs	TFs	KOG	KEGG
XLOC_036175	No	No	No	No	CEJ61876.1	--	--	--
XLOC_036178	No	No	No	No	--	--	--	--
XLOC_036179	DEG(M)	No	No	No	AIL48397.1	WRKY	J	--
XLOC_036180	No	No	No	No	--	--	--	--
XLOC_036182	DEG(M)	No	No	No	XP_008915727.1	--	--	--
XLOC_036184	No	No	No	No	KEP53596.1	--	S	--
XLOC_036187	No	No	No	No	XP_011492994.1	--	--	--
XLOC_036188	No	No	No	No	XP_003054158.1	--	--	--
XLOC_036191	No	No	No	No	--	--	--	--
XLOC_036195	No	No	No	No	KFM73660.1	--	Z	--
XLOC_036199	No	No	No	No	XP_002520174.1	--	S	--
XLOC_036203	DEG(M)	No	No	No	--	--	--	--
XLOC_036204	DEG(M)	No	No	No	ETO77234.1	--	--	--
XLOC_036207	DEG(M)	DEG(M)	DEG(I+M)	DEG(M)	KKA73802.1	--	Z	--
XLOC_036210	No	No	No	No	--	--	--	--
XLOC_036216	No	No	No	No	--	--	--	--
XLOC_036223	No	No	No	No	ELU43332.1	--	--	--
XLOC_036224	No	No	No	No	KEP52466.1	--	--	K19355
XLOC_036226	No	No	No	No	--	--	T	--
XLOC_036229	No	No	No	No	--	--	PT	K05391
XLOC_036230	No	No	No	No	XP_011493123.1	--	--	--
XLOC_036231	No	No	No	No	--	--	--	--
XLOC_036233	No	No	No	No	ETI44060.1	--	--	--
XLOC_036234	No	No	No	No	ETI44060.1	--	--	--
XLOC_036236	No	No	No	No	--	--	--	--
XLOC_036237	No	No	No	No	--	--	--	--
XLOC_036240	DEG(M)	DEG(M)	No	No	NP_001119668.1	B3	J	--
XLOC_036241	DEG(M)	No	No	No	--	--	--	--
XLOC_036248	DEG(M)	No	No	No	--	--	--	--
XLOC_036252	No	No	No	No	--	--	--	--
XLOC_036253	No	No	No	No	--	--	--	--
XLOC_036257	DEG(M)	DEG(M)	DEG(I+M)	DEG(M)	--	--	--	--
XLOC_036258	No	No	No	No	--	--	--	--
XLOC_036259	DEG(M)	No	DEG(I+M)	DEG(M)	--	--	--	--
XLOC_036260	DEG(M)	No	No	No	EUC58696.1	--	J	--
XLOC_036261	No	No	No	No	--	--	--	--
XLOC_036262	No	No	No	No	--	--	--	--
XLOC_036269	DEG(M)	No	No	No	KDN51464.1	--	--	--
XLOC_036270	DEG(M)	DEG(M)	DEG(I+M)	DEG(M)	XP_008911720.1	--	--	--
XLOC_036271	No	No	No	No	AFY99036.1	--	--	--

Differentially expressed gene (DEG) IDs	*C. microphyllum* root and shoot-specific (>2 fold)	*C. microphyllum* root and shoot-specific (>10 fold)	ICC4958 and *C. microphyllum* root-specific (>2 fold)	*C. microphyllum* root-specific (>2 fold)	Functional Annotation			
					NCBI nr IDs	TFs	KOG	KEGG
XLOC_036272	No	No	No	No	EUC64264.1	--	I	--
XLOC_036275	DEG(M)	No	No	No	--	--	--	--
XLOC_036276	No	No	No	No	EUC64950.1	--	--	--
XLOC_036278	DEG(M)	No	No	No	--	--	--	--
XLOC_036279	No	No	No	No	AAG47838.2	--	G	--
XLOC_036281	No	No	DEG(I+M)	No	XP_012571239.1	--	--	--
XLOC_036285	DEG(M)	DEG(M)	No	No	XP_003047970.1	--	C	--
XLOC_036289	No	No	No	No	XP_002512328.1	--	--	--
XLOC_036291	DEG(M)	No	No	No	CCO29922.1	--	--	--
XLOC_036293	No	No	No	No	--	--	--	--
XLOC_036294	No	No	No	No	--	--	--	--
XLOC_036296	No	No	No	No	--	--	--	--
XLOC_036299	No	No	No	No	XP_003052636.1	--	--	--
XLOC_036300	DEG(M)	No	No	No	--	--	--	--
XLOC_036303	No	No	No	No	XP_001660977.1	--	F	K10807
XLOC_036306	No	No	No	No	--	--	--	--
XLOC_036308	No	No	No	No	XP_001652179.1	--	--	--
XLOC_036309	No	No	No	No	XP_002410299.1	WRKY	O	--
XLOC_036310	No	No	No	No	--	--	--	K01802
XLOC_036319	No	No	No	No	--	--	--	--
XLOC_036322	DEG(M)	No	No	No	--	--	J	K02898
XLOC_036324	No	No	No	No	--	--	--	--
XLOC_036327	No	No	No	No	EUC57043.1	--	--	--
XLOC_036331	DEG(M)	No	No	No	--	--	--	--
XLOC_036333	No	No	No	No	--	--	--	--
XLOC_036335	No	No	DEG(I+M)	No	XP_004496520.1	--	--	--
XLOC_036338	DEG(M)	No	No	No	--	--	--	--
XLOC_036343	No	No	No	No	--	--	--	--
XLOC_036344	No	No	No	No	--	--	--	K16297
XLOC_036345	No	No	No	No	--	--	--	--
XLOC_036346	No	No	No	No	KEP49152.1	--	--	--
XLOC_036348	No	No	No	No	--	--	--	--
XLOC_036351	DEG(M)	No	No	No	--	--	--	--
XLOC_036353	No	No	No	No	--	--	--	--
XLOC_036354	DEG(M)	No	No	No	--	--	--	--
XLOC_036355	No	No	No	No	--	--	--	--
XLOC_036358	DEG(M)	No	No	No	--	--	--	--
XLOC_036361	DEG(M)	No	No	No	XP_001433506.1	--	Z	--
XLOC_036363	No	No	No	No	ACZ13339.1	--	C	--
XLOC_036368	No	No	No	No	--	--	--	--

**Differentially expressed gene (DEG) IDs**	***C. microphyllum* root and shoot-specific (>2 fold)**	***C. microphyllum* root and shoot-specific (>10 fold)**	**ICC4958 and *C. microphyllum* root-specific (>2 fold)**	***C. microphyllum* root-specific (>2 fold)**	**Functional Annotation**			
					**NCBI nr IDs**	**TFs**	**KOG**	**KEGG**
XLOC_036370	No	No	No	No	--	--	--	--
XLOC_036372	No	No	No	No	KEP45992.1	--	T	--
XLOC_036373	No	No	No	No	--	--	--	--
XLOC_036374	DEG(M)	DEG(M)	DEG(I+M)	DEG(M)	XP_008911708.1	--	--	--
XLOC_036376	No	No	DEG(I+M)	No	--	--	--	--
XLOC_036378	No	No	No	No	--	--	U	--
XLOC_036379	No	No	DEG(I+M)	No	XP_004487588.1	--	--	--
XLOC_036380	No	No	No	No	--	--	--	--
XLOC_036381	DEG(M)	DEG(M)	No	No	KFY06957.1	--	A	--
XLOC_036383	No	No	DEG(I+M)	No	--	--	--	--
XLOC_036385	DEG(M)	No	No	No	--	--	--	--
XLOC_036391	DEG(M)	No	No	No	--	--	J	--
XLOC_036392	No	No	No	No	ETI34345.1	--	O	--
XLOC_036393	DEG(M)	No	No	No	--	--	--	--
XLOC_036394	No	No	No	No	--	--	--	--
XLOC_036401	DEG(M)	No	No	No	XP_002904178.1	--	S	--
XLOC_036403	No	No	No	No	KEP51408.1	FAR1	O	--
XLOC_036407	No	No	No	No	KEP50062.1	CPP	O	K10704
XLOC_036415	No	No	No	No	--	--	--	--
XLOC_036417	No	No	No	No	XP_002306624.1	--	R	--
XLOC_036420	DEG(M)	No	No	No	EUC54575.1	--	--	--
XLOC_036424	No	No	No	No	XP_001951379.2	--	--	--
XLOC_036425	No	No	No	No	ELU40986.1	--	--	--
XLOC_036426	No	No	No	No	EUC58219.1	--	--	--
XLOC_036427	No	No	No	No	--	--	--	--
XLOC_036431	No	No	No	No	--	--	--	--
XLOC_036433	No	No	No	No	KDN48821.1	--	GO	K02377
XLOC_036434	No	No	No	No	XP_008188006.1	--	Z	--
XLOC_036436	No	No	No	No	--	--	--	--
XLOC_036437	No	No	No	No	--	--	--	--
XLOC_036438	No	No	DEG(I+M)	No	XP_004491486.1	--	--	--
XLOC_036443	No	No	No	No	CEL57281.1	--	--	--
XLOC_036450	DEG(M)	DEG(M)	No	No	XP_008026603.1	--	--	--
XLOC_036451	No	No	No	No	NP_001156073.1	--	--	--
XLOC_036452	DEG(M)	No	No	No	KDN35708.1	--	--	--
XLOC_036454	DEG(M)	DEG(M)	DEG(I+M)	DEG(M)	--	--	--	--
XLOC_036456	DEG(M)	No	No	No	EGU86992.1	--	--	--
XLOC_036459	No	No	No	No	XP_008891947.1	--	--	--
XLOC_036462	DEG(M)	No	No	No	--	--	--	--
XLOC_036463	No	No	No	No	KDN46041.1	--	--	--

Differentially expressed gene (DEG) IDs	*C. microphyllum* root and shoot-specific (>2 fold)	*C. microphyllum* root and shoot-specific (>10 fold)	ICC4958 and *C. microphyllum* root-specific (>2 fold)	*C. microphyllum* root-specific (>2 fold)	Functional Annotation			
					NCBI nr IDs	TFs	KOG	KEGG
XLOC_036464	No	No	No	No	XP_008190023.1	--	--	--
XLOC_036466	DEG(M)	No	No	No	XP_006339860.1	WRKY	KT	--
XLOC_036469	No	No	No	No	--	--	--	K12670
XLOC_036472	No	No	No	No	--	--	--	--
XLOC_036475	DEG(M)	No	No	No	CRJ95477.1	--	O	--
XLOC_036479	No	No	No	No	--	--	--	--
XLOC_036481	No	No	No	No	NP_001172038.1	--	--	--
XLOC_036482	No	No	No	No	XP_002897246.1	--	--	--
XLOC_036484	No	No	No	No	KFM63302.1	--	A	K14326
XLOC_036486	DEG(M)	No	No	No	ABV82744.1	--	--	--
XLOC_036487	No	No	No	No	--	--	--	K01873
XLOC_036490	DEG(M)	No	No	No	--	--	--	--
XLOC_036491	No	No	No	No	--	--	--	K12897
XLOC_036492	DEG(M)	No	No	No	EUC66431.1	--	--	K03965
XLOC_036493	No	No	No	No	--	--	--	--
XLOC_036494	No	No	No	No	--	--	--	--
XLOC_036495	No	No	No	No	--	--	--	--
XLOC_036496	No	No	No	No	--	--	--	--
XLOC_036497	No	No	No	No	--	--	--	--
XLOC_036498	DEG(M)	No	No	No	--	--	--	--
XLOC_036499	No	No	No	No	--	--	--	--
XLOC_036501	No	No	No	No	--	--	--	K00826
XLOC_036502	No	No	No	No	--	--	--	--
XLOC_036504	No	No	No	No	--	--	--	--
XLOC_036506	No	No	No	No	XP_002900461.1	--	--	--
XLOC_036507	DEG(M)	No	No	No	XP_003611284.1	C3H	E	--
XLOC_036508	No	No	No	No	XP_003244253.1	--	--	--
XLOC_036510	DEG(M)	No	No	No	--	--	--	--
XLOC_036512	No	No	No	No	ETL86586.1	--	--	--
XLOC_036515	DEG(M)	No	No	No	--	--	--	--
XLOC_036517	No	No	No	No	XP_001950225.2	--	--	--
XLOC_036521	No	No	No	No	--	--	--	K13091
XLOC_036522	No	No	No	No	--	--	--	--
XLOC_036529	No	No	No	No	XP_008909677.1	--	U	K17086
XLOC_036532	No	No	No	No	XP_009517555.1	--	U	K17301
XLOC_036533	DEG(M)	DEG(M)	DEG(I+M)	DEG(M)	--	--	--	--
XLOC_036534	No	No	No	No	--	--	--	--
XLOC_036535	No	No	No	No	--	--	--	--
XLOC_036539	No	No	No	No	AAS84454.1	--	O	--
XLOC_036540	No	No	No	No	--	--	O	K09580

Differentially expressed gene (DEG) IDs	*C. microphyllum* root and shoot-specific (>2 fold)	*C. microphyllum* root and shoot-specific (>10 fold)	ICC4958 and *C. microphyllum* root-specific (>2 fold)	*C. microphyllum* root-specific (>2 fold)	Functional Annotation			
					NCBI nr IDs	TFs	KOG	KEGG
XLOC_036541	No	No	No	No	--	--	--	--
XLOC_036542	No	No	No	No	--	--	--	--
XLOC_036545	No	No	No	No	--	--	--	--
XLOC_036549	No	No	No	No	XP_012573350.1	--	--	--
XLOC_036550	DEG(M)	No	No	No	--	--	--	--
XLOC_036551	DEG(M)	No	No	No	--	--	--	--
XLOC_036552	DEG(M)	No	No	No	--	--	--	K09580
XLOC_036554	No	No	No	No	--	--	J	--
XLOC_036555	DEG(M)	No	No	No	--	--	--	--
XLOC_036556	DEG(M)	DEG(M)	DEG(I+M)	DEG(M)	--	--	--	--
XLOC_036557	DEG(M)	DEG(M)	DEG(I+M)	DEG(M)	--	--	--	--
XLOC_036564	DEG(M)	No	No	No	--	--	--	--
XLOC_036569	DEG(M)	No	No	No	--	--	--	--
XLOC_036570	No	No	No	No	--	--	--	--
XLOC_036572	No	No	No	No	EUC63026.1	--	--	K05754
XLOC_036573	No	No	No	No	--	G2-like	--	--
XLOC_036575	DEG(M)	No	No	No	--	--	--	--
XLOC_036577	No	No	No	No	--	--	--	K17794
XLOC_036582	DEG(M)	No	No	No	XP_008908714.1	--	--	--
XLOC_036586	No	No	No	No	XP_001949116.3	--	C	K02144
XLOC_036596	DEG(M)	No	No	No	--	--	--	--
XLOC_036597	No	No	No	No	--	--	--	--
XLOC_036598	DEG(M)	No	No	No	--	--	--	--
XLOC_036599	No	No	No	No	--	--	--	--
XLOC_036600	No	No	DEG(I+M)	No	--	--	--	--
XLOC_036610	DEG(M)	No	No	No	--	--	--	--
XLOC_036611	DEG(M)	DEG(M)	No	No	--	--	--	--
XLOC_036612	DEG(M)	No	No	No	--	--	--	--
XLOC_036613	No	No	No	No	--	--	--	--
XLOC_036617	No	No	No	No	EUC66951.1	--	--	--
XLOC_036619	No	No	No	No	--	--	--	--
XLOC_036620	No	No	No	No	--	--	--	--
XLOC_036623	No	No	No	No	--	--	U	K17302
XLOC_036624	DEG(M)	DEG(M)	DEG(I+M)	DEG(M)	--	--	--	--
XLOC_036627	DEG(M)	No	No	No	--	--	--	--
XLOC_036628	No	No	No	No	--	--	P	--
XLOC_036629	DEG(M)	DEG(M)	No	No	--	--	--	--
XLOC_036630	No	No	No	No	CCO36548.1	--	--	--
XLOC_036635	No	No	No	No	--	--	--	--
XLOC_036640	No	No	No	No	--	--	--	--

Differentially expressed gene (DEG) IDs	*C. microphyllum* root and shoot-specific (>2 fold)	*C. microphyllum* root and shoot-specific (>10 fold)	ICC4958 and *C. microphyllum* root-specific (>2 fold)	*C. microphyllum* root-specific (>2 fold)	Functional Annotation			
					NCBI nr IDs	TFs	KOG	KEGG
XLOC_036643	DEG(M)	DEG(M)	DEG(I+M)	DEG(M)	KEP48266.1	--	--	--
XLOC_036644	DEG(M)	No	No	No	KEP48266.1	--	--	--
XLOC_036651	No	No	DEG(I+M)	No	--	--	--	--
XLOC_036654	DEG(M)	No	DEG(I+M)	DEG(M)	--	--	M	--
XLOC_036656	No	No	No	No	--	--	--	--
XLOC_036661	DEG(M)	No	No	No	EUC55601.1	--	--	--
XLOC_036662	DEG(M)	No	No	No	--	--	--	--
XLOC_036674	No	No	No	No	XP_004485829.1	--	--	--
XLOC_036677	No	No	No	No	--	--	--	--
XLOC_036679	No	No	No	No	--	--	--	--
XLOC_036680	No	No	No	No	--	--	--	--
XLOC_036681	No	No	No	No	--	--	O	--
XLOC_036693	No	No	No	No	--	--	T	--
XLOC_036698	No	No	No	No	EUC57313.1	--	--	--
XLOC_036707	DEG(M)	No	No	No	--	--	--	--
XLOC_036709	No	No	No	No	CEL58988.1	--	J	K02957
XLOC_036710	DEG(M)	DEG(M)	No	No	EGU85761.1	--	J	--
XLOC_036711	DEG(M)	No	No	No	XP_012184215.1	--	J	--
XLOC_036712	DEG(M)	DEG(M)	DEG(I+M)	DEG(M)	XP_002897096.1	--	J	K02957
XLOC_036713	DEG(M)	No	DEG(I+M)	DEG(M)	XP_002897096.1	--	J	K02957
XLOC_036714	DEG(M)	No	No	No	CCO30589.1	--	J	--
XLOC_036716	DEG(M)	No	DEG(I+M)	DEG(M)	EUC61215.1	--	J	--
XLOC_036717	DEG(M)	DEG(M)	DEG(I+M)	DEG(M)	EUC61215.1	--	J	--
XLOC_036718	No	No	No	No	XP_010046645.1	--	J	K02870
XLOC_036727	No	No	No	No	EWZ43623.1	--	--	--
XLOC_036732	No	No	No	No	--	--	--	--
XLOC_036748	No	No	No	No	EXA47805.1	--	--	--
XLOC_036750	No	No	No	No	EUC65480.1	--	R	--
XLOC_036751	No	No	No	No	EUC65480.1	--	R	K07897
XLOC_036752	DEG(M)	No	No	No	CEL55272.1	--	TU	K07874
XLOC_036754	DEG(M)	No	No	No	XP_002906462.1	--	TU	--
XLOC_036755	DEG(M)	No	No	No	CEL55272.1	--	TU	K07874
XLOC_036756	DEG(M)	No	No	No	XP_009832367.1	--	TU	K07874
XLOC_036757	No	No	No	No	NP_001155484.1	--	R	--
XLOC_036761	No	No	No	No	--	--	--	--
XLOC_036763	No	No	No	No	--	--	--	--
XLOC_036764	No	No	No	No	--	--	--	--
XLOC_036767	No	No	No	No	--	--	--	--
XLOC_036768	DEG(M)	No	No	No	--	--	--	--
XLOC_036769	DEG(M)	No	DEG(I+M)	DEG(M)	CEL56850.1	--	R	--

Differentially expressed gene (DEG) IDs	*C. microphyllum* root and shoot-specific (>2 fold)	*C. microphyllum* root and shoot-specific (>10 fold)	ICC4958 and *C. microphyllum* root-specific (>2 fold)	*C. microphyllum* root-specific (>2 fold)	Functional Annotation			
					NCBI nr IDs	TFs	KOG	KEGG
XLOC_036772	DEG(M)	No	No	No	XP_008440195.1	--	S	--
XLOC_036776	DEG(M)	No	DEG(I+M)	DEG(M)	EUC61247.1	--	--	--
XLOC_036781	No	No	No	No	--	--	--	K13464
XLOC_036782	No	No	No	No	--	--	--	--
XLOC_036787	No	No	No	No	--	--	--	--
XLOC_036793	No	No	DEG(I+M)	No	XP_004515408.1	--	--	--
XLOC_036794	No	No	No	No	--	--	--	--
XLOC_036795	No	No	No	No	--	--	--	--
XLOC_036796	DEG(M)	No	No	No	XP_001658905.1	--	--	--
XLOC_036798	No	No	No	No	--	--	T	--
XLOC_036799	DEG(M)	No	No	No	XP_003047550.1	--	--	--
XLOC_036800	No	No	DEG(I+M)	No	XP_004494387.1	--	--	--
XLOC_036803	No	No	No	No	CCO30585.1	--	--	--
XLOC_036805	No	No	No	No	--	--	--	K02979
XLOC_036806	No	No	No	No	XP_001943328.1	--	T	K17499
XLOC_036811	DEG(M)	No	No	No	--	--	--	--
XLOC_036812	No	No	No	No	--	--	K	K03127
XLOC_036813	No	No	No	No	XP_001944506.1	--	--	--
XLOC_036820	No	No	No	No	--	--	--	--
XLOC_036821	No	No	No	No	--	--	--	--
XLOC_036822	No	No	No	No	XP_004607860.1	--	T	--
XLOC_036823	DEG(M)	DEG(M)	DEG(I+M)	DEG(M)	--	--	--	--
XLOC_036825	DEG(M)	No	No	No	--	--	--	--
XLOC_036829	No	No	No	No	KDN50965.1	--	--	K11129
XLOC_036831	No	No	No	No	NP_001155789.1	--	--	--
XLOC_036833	DEG(M)	DEG(M)	No	No	--	--	--	--
XLOC_036835	DEG(M)	No	No	No	--	--	--	--
XLOC_036836	DEG(M)	No	No	No	XP_008186078.1	--	C	--
XLOC_036837	No	No	No	No	--	--	--	--
XLOC_036839	No	No	No	No	XP_001944880.1	--	--	--
XLOC_036840	DEG(M)	No	No	No	XP_008894126.1	--	--	--
XLOC_036846	No	No	DEG(I+M)	No	--	--	--	--
XLOC_036847	No	No	No	No	--	--	--	--
XLOC_036849	No	No	No	No	--	--	--	--
XLOC_036852	No	No	No	No	--	--	--	--
XLOC_036854	No	No	No	No	--	--	--	--
XLOC_036858	DEG(M)	No	No	No	--	--	--	--
XLOC_036859	No	No	No	No	--	--	--	--
XLOC_036861	DEG(M)	No	No	No	XP_001951254.1	--	--	--
XLOC_036866	No	No	No	No	XP_012573097.1	--	--	--

Differentially expressed gene (DEG) IDs	*C. microphyllum* root and shoot-specific (>2 fold)	*C. microphyllum* root and shoot-specific (>10 fold)	ICC4958 and *C. microphyllum* root-specific (>2 fold)	*C. microphyllum* root-specific (>2 fold)	Functional Annotation			
					NCBI nr IDs	TFs	KOG	KEGG
XLOC_036868	DEG(M)	No	No	No	XP_007339593.1	--	C	--
XLOC_036869	No	No	No	No	AJQ30118.1	--	CE	--
XLOC_036870	No	No	No	No	EWY99318.1	--	G	--
XLOC_036878	No	No	No	No	DAA06112.1	--	O	--
XLOC_036880	No	No	No	No	--	--	--	K01760
XLOC_036883	No	No	No	No	--	--	--	--
XLOC_036884	No	No	No	No	--	--	--	--
XLOC_036889	DEG(M)	No	No	No	XP_011493351.1	--	O	--
XLOC_036892	No	No	No	No	XP_001942978.2	--	R	--
XLOC_036894	DEG(M)	No	No	No	CEL63654.1	--	K	K12873
XLOC_036896	No	No	No	No	EUC56818.1	--	--	--
XLOC_036909	No	No	No	No	--	--	--	--
XLOC_036914	No	No	No	No	XP_003042220.1	--	--	K13348
XLOC_036917	No	No	No	No	--	--	EI	--
XLOC_036918	No	No	No	No	ETP26929.1	--	C	--
XLOC_036919	No	No	No	No	--	--	--	--
XLOC_036921	No	No	No	No	--	bHLH	J	--
XLOC_036922	DEG(M)	No	No	No	--	--	--	--
XLOC_036927	DEG(M)	No	No	No	--	--	--	--
XLOC_036928	No	No	No	No	--	--	--	--
XLOC_036930	No	No	No	No	XP_004508601.1	--	--	--
XLOC_036931	No	No	No	No	NP_001156824.1	--	--	--
XLOC_036934	DEG(M)	DEG(M)	No	No	--	--	--	--
XLOC_036935	No	No	No	No	--	--	--	--
XLOC_036937	No	No	No	No	--	--	--	--
XLOC_036938	DEG(M)	No	No	No	--	--	--	--
XLOC_036940	DEG(M)	No	DEG(I+M)	DEG(M)	EUC57569.1	--	--	K02267
XLOC_036946	No	No	No	No	--	--	--	--
XLOC_036947	No	No	No	No	CEL57981.1	--	--	--
XLOC_036948	No	No	No	No	--	--	--	--
XLOC_036959	No	No	No	No	--	--	--	--
XLOC_036963	No	No	No	No	XP_001943335.2	--	--	--
XLOC_036965	DEG(M)	No	No	No	XP_003052980.1	--	--	--
XLOC_036967	DEG(M)	No	No	No	--	--	--	--
XLOC_036971	DEG(M)	DEG(M)	No	No	--	--	--	--
XLOC_036973	No	No	No	No	XP_009538582.1	--	R	--
XLOC_036974	No	No	No	No	CCO30809.1	--	Z	K18584
XLOC_036975	No	No	No	No	--	--	--	--
XLOC_036976	DEG(M)	No	No	No	--	--	--	--
XLOC_036977	No	No	No	No	--	--	--	--

Differentially expressed gene (DEG) IDs	*C. microphyllum* root and shoot-specific (>2 fold)	*C. microphyllum* root and shoot-specific (>10 fold)	ICC4958 and *C. microphyllum* root-specific (>2 fold)	*C. microphyllum* root-specific (>2 fold)	Functional Annotation			
					NCBI nr IDs	TFs	KOG	KEGG
XLOC_036978	No	No	No	No	KDN42898.1	--	--	--
XLOC_036979	No	No	No	No	--	--	A	K14821
XLOC_036980	No	No	No	No	--	--	--	--
XLOC_036981	No	No	No	No	KDN45824.1	--	--	--
XLOC_036984	DEG(M)	No	No	No	--	--	--	--
XLOC_036985	No	No	No	No	EUC55778.1	--	--	--
XLOC_036986	No	No	No	No	KDN42461.1	--	--	--
XLOC_036992	No	No	No	No	--	--	--	--
XLOC_036998	No	No	No	No	KCW55525.1	--	R	K04392
XLOC_037000	No	No	No	No	--	--	--	--
XLOC_037002	No	No	No	No	--	--	--	--
XLOC_037003	No	No	No	No	--	--	--	--
XLOC_037005	No	No	No	No	--	--	--	--
XLOC_037006	No	No	No	No	CCO32574.1	--	--	--
XLOC_037011	No	No	No	No	--	--	--	--
XLOC_037012	No	No	No	No	NP_001155617.1	--	AJ	K12845
XLOC_037014	No	No	No	No	--	--	--	--
XLOC_037015	No	No	No	No	--	--	--	--
XLOC_037020	No	No	No	No	NP_001155486.1	--	--	K06130
XLOC_037021	DEG(M)	No	No	No	YP_009048923.1	--	C	--
XLOC_037022	No	No	No	No	--	--	--	--
XLOC_037024	No	No	No	No	--	--	--	--
XLOC_037027	No	No	No	No	--	--	--	--
XLOC_037028	No	No	No	No	--	--	--	--
XLOC_037031	No	No	No	No	ETK89632.1	--	--	--
XLOC_037032	No	No	No	No	--	--	--	--
XLOC_037039	DEG(M)	No	No	No	XP_008912522.1	--	--	--
XLOC_037040	No	No	No	No	--	--	--	--
XLOC_037043	No	No	No	No	--	--	--	--
XLOC_037045	DEG(M)	No	No	No	--	--	--	--
XLOC_037052	DEG(M)	No	No	No	NP_001129592.1	--	J	K02958
XLOC_037055	No	No	No	No	XP_004491111.1	--	--	--
XLOC_037058	No	No	No	No	--	--	--	--
XLOC_037059	No	No	No	No	ETK75459.1	--	--	--
XLOC_037061	DEG(M)	No	DEG(I+M)	DEG(M)	XP_002903189.1	--	T	--
XLOC_037062	No	No	No	No	XP_003045325.1	--	--	--
XLOC_037064	No	No	No	No	--	--	--	--
XLOC_037069	DEG(M)	No	No	No	--	--	--	--
XLOC_037070	No	No	No	No	--	--	--	--
XLOC_037072	DEG(M)	No	No	No	EUC53751.1	--	--	--

Differentially expressed gene (DEG) IDs	*C. microphyllum* root and shoot-specific (>2 fold)	*C. microphyllum* root and shoot-specific (>10 fold)	ICC4958 and *C. microphyllum* root-specific (>2 fold)	*C. microphyllum* root-specific (>2 fold)	Functional Annotation			
					NCBI nr IDs	TFs	KOG	KEGG
XLOC_037075	No	No	No	No	--	--	--	--
XLOC_037080	No	No	No	No	XP_010781843.1	--	O	K07151
XLOC_037083	No	No	No	No	ETK74249.1	--	--	--
XLOC_037087	DEG(M)	DEG(M)	No	No	XP_003050029.1	--	A	--
XLOC_037090	No	No	No	No	XP_008894610.1	--	--	--
XLOC_037093	No	No	No	No	--	--	--	--
XLOC_037094	No	No	No	No	NP_001119625.1	--	F	--
XLOC_037095	No	No	No	No	--	--	--	--
XLOC_037096	DEG(M)	No	No	No	--	--	--	--
XLOC_037097	DEG(M)	No	No	No	--	--	--	--
XLOC_037101	No	No	No	No	--	--	--	--
XLOC_037102	No	No	No	No	--	--	--	--
XLOC_037103	DEG(M)	DEG(M)	No	No	XP_003048814.1	--	J	K03262
XLOC_037104	DEG(M)	No	No	No	CCO36146.1	--	--	--
XLOC_037107	No	No	No	No	--	--	--	--
XLOC_037110	No	No	No	No	--	--	--	--
XLOC_037111	DEG(M)	No	No	No	ABR27976.1	--	J	K02868
XLOC_037113	No	No	No	No	--	--	--	--
XLOC_037114	No	No	No	No	XP_008897914.1	--	C	K02154
XLOC_037115	No	No	No	No	--	--	--	--
XLOC_037117	No	No	No	No	--	--	--	--
XLOC_037121	DEG(M)	No	No	No	--	--	--	--
XLOC_037125	No	No	No	No	--	--	J	--
XLOC_037126	DEG(M)	No	No	No	--	--	--	--
XLOC_037133	No	No	No	No	--	--	--	--
XLOC_037134	No	No	No	No	EXA34352.1	--	--	--
XLOC_037137	DEG(M)	No	No	No	CEL63831.1	--	--	--
XLOC_037139	No	No	No	No	--	--	--	--
XLOC_037141	No	No	No	No	--	--	--	K09602
XLOC_037142	No	No	No	No	--	--	--	--
XLOC_037145	DEG(M)	No	No	No	--	--	--	--
XLOC_037146	DEG(M)	No	No	No	--	--	--	--
XLOC_037150	No	No	No	No	--	--	--	--
XLOC_037152	DEG(M)	No	DEG(I+M)	DEG(M)	--	--	--	--
XLOC_037153	No	No	No	No	--	--	--	--
XLOC_037155	No	No	No	No	--	--	--	--
XLOC_037156	No	No	No	No	--	--	--	--
XLOC_037161	No	No	No	No	--	--	--	--
XLOC_037163	DEG(M)	No	No	No	XP_002401133.1	NAC	J	K02976
XLOC_037165	DEG(M)	DEG(M)	DEG(I+M)	DEG(M)	--	--	--	--

Differentially expressed gene (DEG) IDs	*C. microphyllum* root and shoot-specific (>2 fold)	*C. microphyllum* root and shoot-specific (>10 fold)	ICC4958 and *C. microphyllum* root-specific (>2 fold)	*C. microphyllum* root-specific (>2 fold)	Functional Annotation			
					NCBI nr IDs	TFs	KOG	KEGG
XLOC_037166	No	No	No	No	XP_003050943.1	--	--	--
XLOC_037167	DEG(M)	No	DEG(I+M)	DEG(M)	--	--	--	--
XLOC_037169	No	No	No	No	--	--	--	--
XLOC_037171	No	No	No	No	--	--	--	--
XLOC_037172	No	No	No	No	--	--	--	--
XLOC_037173	DEG(M)	No	No	No	--	--	--	--
XLOC_037176	No	No	No	No	CCO29038.1	--	--	--
XLOC_037196	No	No	No	No	CEL56743.1	--	--	--
XLOC_037197	No	No	No	No	ETO17530.1	--	--	--
XLOC_037198	DEG(M)	No	No	No	CEL58879.1	--	--	--
XLOC_037201	No	No	No	No	--	--	--	K20222
XLOC_037204	DEG(M)	No	No	No	--	--	--	--
XLOC_037206	DEG(M)	No	No	No	--	--	--	--
XLOC_037208	No	No	No	No	KEH18323.1	--	H	K01495
XLOC_037209	DEG(M)	No	No	No	--	--	--	--
XLOC_037210	No	No	No	No	--	--	--	--
XLOC_037214	DEG(M)	No	No	No	--	--	--	--
XLOC_037215	No	No	No	No	--	--	--	--
XLOC_037218	No	No	No	No	ADO95352.1	--	--	--
XLOC_037220	No	No	No	No	XP_003051573.1	--	--	--
XLOC_037222	No	No	No	No	--	--	--	--
XLOC_037224	No	No	No	No	EGU84681.1	--	--	--
XLOC_037230	No	No	No	No	--	--	--	--
XLOC_037233	No	No	No	No	--	--	X	K03405
XLOC_037234	DEG(M)	No	No	No	--	--	--	--
XLOC_037235	DEG(M)	No	No	No	--	--	--	--
XLOC_037238	DEG(M)	No	No	No	--	--	--	--
XLOC_037240	DEG(M)	DEG(M)	DEG(I+M)	DEG(M)	--	--	--	--
XLOC_037242	DEG(M)	No	No	No	--	--	P	--
XLOC_037243	DEG(M)	DEG(M)	No	No	--	--	--	--
XLOC_037245	No	No	No	No	--	--	--	--
XLOC_037246	No	No	No	No	EUC56947.1	--	Q	--
XLOC_037248	No	No	No	No	XP_001652847.1	--	J	--
XLOC_037257	No	No	No	No	--	--	--	--
XLOC_037258	No	No	No	No	CEL62055.1	--	--	--
XLOC_037262	No	No	No	No	--	--	--	--
XLOC_037264	No	No	No	No	--	--	--	--
XLOC_037265	No	No	No	No	CCO31099.1	--	C	--
XLOC_037267	No	No	No	No	--	--	--	--
XLOC_037268	No	No	No	No	--	--	O	--

Differentially expressed gene (DEG) IDs	*C. microphyllum* root and shoot-specific (>2 fold)	*C. microphyllum* root and shoot-specific (>10 fold)	ICC4958 and *C. microphyllum* root-specific (>2 fold)	*C. microphyllum* root-specific (>2 fold)	Functional Annotation			
					NCBI nr IDs	TFs	KOG	KEGG
XLOC_037275	DEG(M)	No	No	No	--	--	--	--
XLOC_037276	DEG(M)	No	DEG(I+M)	DEG(M)	CCO34937.1	--	--	--
XLOC_037282	No	No	No	No	KDN39067.1	--	--	--
XLOC_037285	No	No	No	No	EUC53701.1	--	--	--
XLOC_037286	No	No	No	No	--	--	--	--
XLOC_037288	No	No	No	No	ELU42446.1	--	--	--
XLOC_037289	No	No	No	No	CEL58938.1	--	E	--
XLOC_037291	DEG(M)	No	DEG(I+M)	DEG(M)	ELU45696.1	--	J	K02951
XLOC_037293	No	No	No	No	--	--	--	--
XLOC_037295	No	No	No	No	KFM74962.1	Trihelix	U	--
XLOC_037299	No	No	No	No	--	--	--	--
XLOC_037300	No	No	No	No	CEL55186.1	--	--	--
XLOC_037302	No	No	No	No	KNG44080.1	--	--	--
XLOC_037306	No	No	No	No	NP_001155695.1	--	O	--
XLOC_037310	No	No	No	No	XP_003044960.1	--	--	--
XLOC_037311	No	No	No	No	--	--	--	--
XLOC_037312	No	No	No	No	--	--	--	--
XLOC_037319	No	No	No	No	XP_001663499.1	--	O	--
XLOC_037321	No	No	No	No	EUC66658.1	--	--	--
XLOC_037323	No	No	No	No	XP_009529666.1	--	--	--
XLOC_037324	No	No	No	No	--	--	--	K01634
XLOC_037332	DEG(M)	No	No	No	--	--	--	--
XLOC_037335	DEG(M)	No	No	No	--	--	--	--
XLOC_037343	No	No	No	No	--	--	--	--
XLOC_037347	No	No	No	No	--	--	--	--
XLOC_037350	DEG(M)	No	No	No	--	--	--	--
XLOC_037352	DEG(M)	DEG(M)	No	No	XP_003047987.1	--	J	--
XLOC_037354	No	No	No	No	--	--	--	--
XLOC_037355	DEG(M)	No	No	No	--	--	--	K02893
XLOC_037356	No	No	No	No	--	--	--	--
XLOC_037359	No	No	No	No	ETK83293.1	--	--	--
XLOC_037364	No	No	No	No	--	--	--	--
XLOC_037365	No	No	No	No	KEP53604.1	--	--	--
XLOC_037366	No	No	No	No	--	--	--	--
XLOC_037372	No	No	No	No	KDP43787.1	--	R	--
XLOC_037373	DEG(M)	No	No	No	KDN34618.1	--	--	--
XLOC_037377	No	No	No	No	CEL56441.1	--	--	--
XLOC_037381	No	No	No	No	--	--	T	--
XLOC_037382	No	No	No	No	XP_002529989.1	--	J	--
XLOC_037384	DEG(M)	No	No	No	--	--	--	K00737

Differentially expressed gene (DEG) IDs	*C. microphyllum* root and shoot-specific (>2 fold)	*C. microphyllum* root and shoot-specific (>10 fold)	ICC4958 and *C. microphyllum* root-specific (>2 fold)	*C. microphyllum* root-specific (>2 fold)	Functional Annotation			
					NCBI nr IDs	TFs	KOG	KEGG
XLOC_037386	DEG(M)	No	No	No	ETL41989.1	--	--	--
XLOC_037391	No	No	No	No	EUC61022.1	--	--	--
XLOC_037392	No	No	No	No	--	--	--	--
XLOC_037394	No	No	No	No	--	--	--	--
XLOC_037399	No	No	No	No	--	--	--	--
XLOC_037400	No	No	No	No	ETI39600.1	--	--	--
XLOC_037401	No	No	No	No	ACR57215.1	--	O	--
XLOC_037404	No	No	No	No	XP_009536762.1	--	--	K02735
XLOC_037411	DEG(M)	No	No	No	CEL61857.1	--	--	--
XLOC_037412	No	No	No	No	--	--	--	--
XLOC_037416	No	No	No	No	KDN49676.1	--	U	K17917
XLOC_037417	DEG(M)	No	DEG(I+M)	DEG(M)	ETL90047.1	--	--	--
XLOC_037424	No	No	No	No	XP_008896957.1	--	--	--
XLOC_037428	No	No	No	No	XP_003053597.1	--	--	--
XLOC_037430	DEG(M)	DEG(M)	DEG(I+M)	DEG(M)	KEP49157.1	--	--	--
XLOC_037432	No	No	No	No	--	--	--	--
XLOC_037433	DEG(M)	No	No	No	--	--	--	--
XLOC_037434	No	No	No	No	--	--	--	--
XLOC_037435	No	No	No	No	--	--	--	--
XLOC_037438	No	No	No	No	--	--	--	--
XLOC_037440	No	No	DEG(I+M)	No	XP_003611453.1	--	E	K14638
XLOC_037451	No	No	No	No	XP_012266892.1	--	C	--
XLOC_037452	DEG(M)	No	No	No	--	--	--	--
XLOC_037455	DEG(M)	No	No	No	--	--	--	--
XLOC_037461	DEG(M)	No	No	No	--	--	--	--
XLOC_037463	DEG(M)	No	No	No	NP_001155672.1	--	J	K02889
XLOC_037469	No	No	No	No	--	--	--	K02930
XLOC_037472	No	No	No	No	--	--	--	--
XLOC_037477	No	No	No	No	--	--	--	--
XLOC_037479	DEG(M)	No	No	No	--	--	J	--
XLOC_037486	No	No	No	No	--	--	--	--
XLOC_037487	DEG(M)	No	DEG(I+M)	DEG(M)	--	--	--	--
XLOC_037491	No	No	No	No	--	--	C	--
XLOC_037493	DEG(M)	No	No	No	CCO35926.1	NAC	J	--
XLOC_037494	No	No	No	No	--	--	--	--
XLOC_037498	DEG(M)	DEG(M)	DEG(I+M)	DEG(M)	KEH29169.1	--	--	--
XLOC_037499	No	No	No	No	ETM50255.1	--	--	K01115
XLOC_037502	No	No	No	No	--	--	OKT	K11434
XLOC_037503	DEG(M)	No	No	No	--	--	J	--
XLOC_037504	No	No	No	No	--	--	--	--

Differentially expressed gene (DEG) IDs	*C. microphyllum* root and shoot-specific (>2 fold)	*C. microphyllum* root and shoot-specific (>10 fold)	ICC4958 and *C. microphyllum* root-specific (>2 fold)	*C. microphyllum* root-specific (>2 fold)	Functional Annotation			
					NCBI nr IDs	TFs	KOG	KEGG
XLOC_037521	No	No	No	No	--	--	--	--
XLOC_037528	DEG(M)	DEG(M)	No	No	--	--	--	--
XLOC_037529	No	No	DEG(I+M)	No	--	--	--	--
XLOC_037530	No	No	DEG(I+M)	No	--	--	--	--
XLOC_037531	No	No	No	No	--	--	C	--
XLOC_037535	No	No	No	No	--	--	--	--
XLOC_037537	DEG(M)	No	No	No	--	--	--	--
XLOC_037538	DEG(M)	DEG(M)	DEG(I+M)	DEG(M)	--	--	--	--
XLOC_037540	No	No	No	No	--	--	--	--
XLOC_037544	DEG(M)	No	No	No	--	--	--	--
XLOC_037545	No	No	No	No	XP_002533474.1	--	--	--
XLOC_037547	DEG(M)	No	No	No	--	--	--	--
XLOC_037548	DEG(M)	No	DEG(I+M)	DEG(M)	--	--	--	--
XLOC_037550	No	No	No	No	EUC65301.1	--	--	--
XLOC_037551	DEG(M)	No	No	No	EUC63787.1	--	--	--
XLOC_037552	No	No	No	No	--	--	J	K09250
XLOC_037553	No	No	No	No	XP_009537600.1	--	--	--
XLOC_037554	No	No	No	No	--	--	--	--
XLOC_037560	DEG(M)	DEG(M)	No	No	--	--	--	--
XLOC_037562	No	No	No	No	XP_004488706.1	--	O	--
XLOC_037564	No	No	No	No	--	--	--	--
XLOC_037565	DEG(M)	No	No	No	--	--	--	--
XLOC_037566	No	No	No	No	--	--	--	--
XLOC_037572	DEG(M)	No	No	No	CCO26849.1	NAC	J	--
XLOC_037574	DEG(M)	No	No	No	--	--	--	--
XLOC_037577	DEG(M)	No	DEG(I+M)	DEG(M)	CEL51841.1	--	--	--
XLOC_037579	DEG(M)	No	No	No	--	--	--	--
XLOC_037581	No	No	No	No	--	--	--	--
XLOC_037583	DEG(M)	No	No	No	XP_002510790.1	--	--	--
XLOC_037585	DEG(M)	DEG(M)	No	No	--	--	--	--
XLOC_037587	No	No	No	No	--	--	--	--
XLOC_037589	No	No	DEG(I+M)	No	--	--	--	--
XLOC_037590	No	No	No	No	EGU79704.1	--	--	--
XLOC_037591	No	No	No	No	--	--	--	--
XLOC_037592	No	No	No	No	--	--	--	--
XLOC_037593	DEG(M)	No	No	No	--	--	J	K02872
XLOC_037594	DEG(M)	No	No	No	XP_001949629.1	--	--	--
XLOC_037597	No	No	DEG(I+M)	No	XP_004496498.1	--	M	K01183
XLOC_037599	No	No	No	No	--	--	--	K15814
XLOC_037607	No	No	DEG(I+M)	No	XP_004514814.1	--	C	K00279

Differentially expressed gene (DEG) IDs	*C. microphyllum* root and shoot-specific (>2 fold)	*C. microphyllum* root and shoot-specific (>10 fold)	ICC4958 and *C. microphyllum* root-specific (>2 fold)	*C. microphyllum* root-specific (>2 fold)	Functional Annotation			
					NCBI nr IDs	TFs	KOG	KEGG
XLOC_037610	DEG(M)	No	No	No	KDN50181.1	--	--	--
XLOC_037612	No	No	No	No	--	--	--	--
XLOC_037617	No	No	No	No	XP_004367478.1	--	O	--
XLOC_037620	No	No	No	No	--	--	H	--
XLOC_037622	DEG(M)	DEG(M)	No	No	EMP42703.1	--	--	--
XLOC_037630	No	No	No	No	KEP47732.1	--	--	--
XLOC_037631	DEG(M)	DEG(M)	No	No	--	--	--	--
XLOC_037632	No	No	No	No	--	--	--	--
XLOC_037634	No	No	No	No	--	--	--	--
XLOC_037635	No	No	No	No	CCA23190.1	--	P	K05016
XLOC_037642	No	No	No	No	XP_009514008.1	--	--	--
XLOC_037643	No	No	No	No	XP_002514855.1	SBP	V	K18045
XLOC_037644	No	No	DEG(I+M)	No	--	--	--	--
XLOC_037647	DEG(M)	No	No	No	XP_003051386.1	--	--	--
XLOC_037648	No	No	No	No	--	--	T	--
XLOC_037658	DEG(M)	No	No	No	AIH15719.1	--	--	--
XLOC_037660	No	No	No	No	--	--	--	--
XLOC_037662	No	No	No	No	EUC59711.1	--	Z	--
XLOC_037664	No	No	No	No	--	--	Z	--
XLOC_037669	DEG(M)	No	No	No	CEL59513.1	--	--	--
XLOC_037670	No	No	No	No	KDN50926.1	--	--	--
XLOC_037672	DEG(M)	No	No	No	--	--	--	--
XLOC_037673	No	No	No	No	BAJ88214.1	--	--	--
XLOC_037675	No	No	No	No	--	--	--	--
XLOC_037677	DEG(M)	No	No	No	XP_009519860.1	--	U	--
XLOC_037679	No	No	No	No	--	--	--	--
XLOC_037682	No	No	No	No	--	--	--	--
XLOC_037686	No	No	No	No	CEL60945.1	--	--	--
XLOC_037689	No	No	No	No	--	--	--	--
XLOC_037690	No	No	No	No	--	--	--	--
XLOC_037691	DEG(M)	No	No	No	EUC57218.1	--	T	--
XLOC_037692	No	No	No	No	--	--	--	--
XLOC_037694	No	No	No	No	--	--	I	--
XLOC_037697	No	No	No	No	--	--	--	--
XLOC_037699	No	No	No	No	--	--	--	--
XLOC_037705	No	No	No	No	CEL63766.1	--	--	--
XLOC_037706	DEG(M)	No	No	No	--	--	--	--
XLOC_037708	No	No	No	No	--	--	--	--
XLOC_037709	No	No	No	No	--	--	--	--
XLOC_037710	DEG(M)	No	No	No	--	--	--	--

Differentially expressed gene (DEG) IDs	*C. microphyllum* root and shoot-specific (>2 fold)	*C. microphyllum* root and shoot-specific (>10 fold)	ICC4958 and *C. microphyllum* root-specific (>2 fold)	*C. microphyllum* root-specific (>2 fold)	Functional Annotation			
					NCBI nr IDs	TFs	KOG	KEGG
XLOC_037711	DEG(M)	No	No	No	XP_003051192.1	--	--	--
XLOC_037712	No	No	No	No	ETO77933.1	--	--	--
XLOC_037713	No	No	No	No	--	--	--	--
XLOC_037716	DEG(M)	No	No	No	--	--	--	K02979
XLOC_037719	No	No	No	No	NP_001162140.1	--	--	--
XLOC_037720	No	No	No	No	EUC62406.1	--	--	--
XLOC_037723	No	No	No	No	--	--	--	--
XLOC_037728	No	No	No	No	XP_012088339.1	--	T	--
XLOC_037730	DEG(M)	DEG(M)	No	No	XP_004485617.1	--	--	--
XLOC_037733	DEG(M)	No	No	No	ETK83919.1	--	CE	--
XLOC_037737	No	No	No	No	--	--	--	--
XLOC_037739	No	No	DEG(I+M)	No	--	--	--	--
XLOC_037752	No	No	No	No	--	--	--	--
XLOC_037755	No	No	No	No	--	--	--	--
XLOC_037757	DEG(M)	No	No	No	--	--	--	K12741
XLOC_037758	No	No	No	No	XP_008895595.1	--	--	--
XLOC_037760	No	No	No	No	--	--	--	--
XLOC_037761	No	No	No	No	--	--	--	--
XLOC_037764	No	No	No	No	--	--	J	K02953
XLOC_037765	No	No	No	No	AHZ64880.2	--	--	--
XLOC_037771	No	No	No	No	--	--	Z	K10364
XLOC_037773	No	No	No	No	--	--	--	--
XLOC_037774	No	No	No	No	XP_001663323.1	--	R	--
XLOC_037777	No	No	No	No	--	--	--	--
XLOC_037778	No	No	No	No	--	--	X	--
XLOC_037779	No	No	No	No	XP_008906457.1	--	--	--
XLOC_037780	DEG(M)	DEG(M)	No	No	ACV31371.1	--	--	--
XLOC_037783	No	No	No	No	--	--	--	--
XLOC_037785	DEG(M)	No	No	No	--	--	--	--
XLOC_037787	No	No	No	No	ETL78984.1	--	--	--
XLOC_037789	DEG(M)	No	No	No	--	--	--	--
XLOC_037790	No	No	No	No	--	--	--	--
XLOC_037795	DEG(M)	No	No	No	--	--	--	--
XLOC_037796	No	No	DEG(I+M)	No	--	C2H2	--	--
XLOC_037797	No	No	No	No	--	--	--	--
XLOC_037798	No	No	No	No	--	--	--	--
XLOC_037799	No	No	No	No	--	--	--	--
XLOC_037800	No	No	No	No	--	--	--	--
XLOC_037806	No	No	No	No	--	--	--	--
XLOC_037808	No	No	No	No	--	--	--	--

Differentially expressed gene (DEG) IDs	*C. microphyllum* root and shoot-specific (>2 fold)	*C. microphyllum* root and shoot-specific (>10 fold)	ICC4958 and *C. microphyllum* root-specific (>2 fold)	*C. microphyllum* root-specific (>2 fold)	**Functional Annotation**			
					**NCBI nr IDs**	**TFs**	**KOG**	**KEGG**
XLOC_037811	No	No	No	No	--	--	--	--
XLOC_037812	No	No	No	No	--	--	--	--
XLOC_037813	No	No	No	No	--	--	--	--
XLOC_037817	No	No	No	No	--	Trihelix	--	--
XLOC_037819	DEG(M)	DEG(M)	No	No	--	--	--	--
XLOC_037821	No	No	No	No	--	--	--	--
XLOC_037825	No	No	No	No	--	--	--	K00876
XLOC_037826	No	No	No	No	--	--	--	--
XLOC_037830	No	No	No	No	EMT63556.1	--	--	--
XLOC_037831	No	No	No	No	EXM03335.1	--	--	--
XLOC_037836	No	No	No	No	--	--	--	--
XLOC_037837	DEG(M)	No	No	No	--	--	J	--
XLOC_037842	DEG(M)	No	No	No	CCO29410.1	--	--	--
XLOC_037843	No	No	No	No	ELU41324.1	--	--	--
XLOC_037844	No	No	No	No	XP_012080241.1	--	J	K02997
XLOC_037845	No	No	No	No	--	--	C	K08916
XLOC_037852	DEG(M)	No	DEG(I+M)	DEG(M)	--	--	--	--
XLOC_037856	DEG(M)	No	No	No	XP_004487944.1	--	--	--
XLOC_037857	DEG(M)	No	No	No	KDN41088.1	--	--	--
XLOC_037862	No	No	No	No	--	--	--	--
XLOC_037867	No	No	No	No	NP_001155626.1	--	--	--
XLOC_037873	DEG(M)	No	DEG(I+M)	DEG(M)	XP_004495105.1	ERF	--	--
XLOC_037879	No	No	No	No	ETL25784.1	--	--	--
XLOC_037880	No	No	No	No	--	--	--	--
XLOC_037888	DEG(M)	DEG(M)	DEG(I+M)	DEG(M)	XP_009833848.1	--	--	K01527
XLOC_037889	DEG(M)	No	No	No	XP_008893332.1	--	--	K01527
XLOC_037894	No	No	No	No	--	--	--	--
XLOC_037898	DEG(M)	No	No	No	--	--	--	--
XLOC_037900	No	No	No	No	--	--	O	--
XLOC_037905	No	No	No	No	--	--	--	--
XLOC_037909	No	No	No	No	CEL51539.1	--	--	K03232
XLOC_037910	DEG(M)	No	No	No	CEL51539.1	--	--	K03232
XLOC_037911	DEG(M)	No	No	No	CEL51539.1	Dof	K	--
XLOC_037915	No	No	No	No	EUC63374.1	--	--	K01850
XLOC_037924	DEG(M)	DEG(M)	DEG(I+M)	DEG(M)	--	--	--	--
XLOC_037927	No	No	No	No	XP_004486286.1	--	--	--
XLOC_037932	DEG(M)	No	No	No	--	--	--	--
XLOC_037933	No	No	No	No	--	--	--	--
XLOC_037937	DEG(M)	No	No	No	XP_009533669.1	--	F	K00940
XLOC_037940	No	No	No	No	XP_008901335.1	--	O	--

Differentially expressed gene (DEG) IDs	*C. microphyllum* root and shoot-specific (>2 fold)	*C. microphyllum* root and shoot-specific (>10 fold)	ICC4958 and *C. microphyllum* root-specific (>2 fold)	*C. microphyllum* root-specific (>2 fold)	Functional Annotation			
					NCBI nr IDs	TFs	KOG	KEGG
XLOC_037943	No	No	No	No	--	--	G	--
XLOC_037945	No	No	No	No	--	--	--	--
XLOC_037965	DEG(M)	No	No	No	--	--	--	--
XLOC_037974	DEG(M)	No	No	No	CCO27676.1	--	--	--
XLOC_037975	DEG(M)	No	No	No	CEL56035.1	--	--	--
XLOC_037981	DEG(M)	No	No	No	AEK51007.1	Trihelix	G	--
XLOC_037982	DEG(M)	No	No	No	AEK51007.1	Trihelix	G	--
XLOC_037991	No	No	No	No	--	--	--	--
XLOC_037997	DEG(M)	No	No	No	AKS48202.1	--	--	K02261
XLOC_037998	No	No	No	No	--	--	--	--
XLOC_037999	No	No	No	No	EUC62463.1	--	--	--
XLOC_038000	No	No	No	No	NP_001232947.1	--	--	--
XLOC_038001	DEG(M)	DEG(M)	No	No	XP_003045287.1	--	--	K02903
XLOC_038007	DEG(M)	No	No	No	XP_003273579.1	--	X	--
XLOC_038011	No	No	No	No	AGN70891.1	--	--	--
XLOC_038016	No	No	No	No	--	--	C	--
XLOC_038020	No	No	No	No	--	--	--	--
XLOC_038023	No	No	No	No	--	--	--	--
XLOC_038031	No	No	No	No	ETP47261.1	--	--	K00102
XLOC_038035	No	No	No	No	XP_004516682.1	--	--	--
XLOC_038038	No	No	No	No	XP_008898912.1	--	--	--
XLOC_038039	No	No	No	No	XP_004339226.1	--	O	--
XLOC_038043	No	No	No	No	--	--	--	--
XLOC_038048	DEG(M)	No	No	No	--	--	J	K02910
XLOC_038049	No	No	No	No	EUC65729.1	--	--	--
XLOC_038060	No	No	No	No	--	--	--	--
XLOC_038063	No	No	No	No	--	--	--	--
XLOC_038064	No	No	No	No	--	--	--	K01285
XLOC_038066	No	No	DEG(I+M)	No	XP_004502894.1	--	--	--
XLOC_038071	No	No	No	No	XP_011493714.1	--	--	--
XLOC_038072	DEG(M)	No	No	No	CEL62223.1	--	--	--
XLOC_038073	No	No	No	No	CCO27535.1	--	--	--
XLOC_038075	No	No	No	No	XP_008905548.1	--	--	K00111
XLOC_038076	No	No	No	No	--	--	--	--
XLOC_038079	No	No	No	No	--	--	--	--
XLOC_038081	No	No	No	No	--	--	--	--
XLOC_038087	DEG(M)	No	No	No	--	--	--	--
XLOC_038097	DEG(M)	No	No	No	EUC65683.1	--	--	--
XLOC_038106	No	No	DEG(I+M)	No	XP_004515396.1	--	--	--
XLOC_038107	No	No	No	No	--	--	--	--

**Differentially expressed gene (DEG) IDs**	***C. microphyllum* root and shoot-specific (>2 fold)**	***C. microphyllum* root and shoot-specific (>10 fold)**	**ICC4958 and *C. microphyllum* root-specific (>2 fold)**	***C. microphyllum* root-specific (>2 fold)**	**Functional Annotation**			
					**NCBI nr IDs**	**TFs**	**KOG**	**KEGG**
XLOC_038108	DEG(M)	No	No	No	--	--	--	--
XLOC_038111	No	No	No	No	KDN37964.1	--	--	--
XLOC_038115	No	No	No	No	CEL54318.1	--	M	--
XLOC_038117	No	No	No	No	KEP50239.1	--	--	--
XLOC_038120	No	No	No	No	--	--	F	K10808
XLOC_038124	No	No	No	No	--	--	--	--
XLOC_038126	No	No	No	No	--	--	--	--
XLOC_038129	DEG(M)	No	No	No	CAC21570.1	--	TU	--
XLOC_038130	No	No	No	No	XP_001951050.1	--	--	--
XLOC_038134	DEG(M)	No	No	No	--	--	--	K06995
XLOC_038137	No	No	DEG(I+M)	No	--	--	--	--
XLOC_038141	No	No	No	No	KEP54601.1	--	--	--
XLOC_038144	No	No	No	No	--	--	--	--
XLOC_038147	No	No	No	No	--	--	--	--
XLOC_038148	DEG(M)	DEG(M)	No	No	EXK40964.1	--	--	--

(I+M): genes differentially expressed in both ICC 4958 and *C. microphyllum* .

Table S3. Genic SSR markers and *in silico* polymorphic SSR markers differentiating *C. microphyllum* and ICC 4958 developed from the comparative transcriptome profiles of roots and shoots between *C. microphyllum* and ICC 4958

Transcript IDs	Differentially expressed gene (DEG) IDs	SSR repeat-motifs	SSR repeat-length (bp)	*In silico* polymorphic SSRs						Forward primers (5'-3')	Annealing tempearture(0C)	Reverse primers (5'-3')	Annealing tempearture(0C)	Amplified product size (bp)	Structural annotation		Functional Annotation			
				C. microphyllum		ICC4958									Gene IDs	Sequence component of genes	NCBI nr IDs	TFs	KOG	KEGG
				Transcript	SSR repeat-motifs	Chromosomes	SSR repeat-motifs	Start physical positions (bp)	End physical positions (bp)											
TR10072|c0_g1_i1	XLOC_000019	(AG)6	12	--	--	--	--	--	--	AATGCGGAGAAACCATAACG	60	CCGCCTTCATGTACAACCTT	60	256	--	--	--	--	--	--
TR10095|c0_g1_i1	No	(AT)7aa(AG)9	34	--	--	--	--	--	--	ATGCAAGACGGCATTCCTAC	60	CGCATGCATCAGACGAAATA	61	233	--	--	--	--	--	--
TR10161|c1_g1_i1	No	(TTG)6	18	--	--	--	--	--	--	GGCAAACGAACCTTGTGAAT	60	AAATTCACTCTCTTCCCTCTTCA	59	205	--	--	--	--	--	--
TR10167|c1_g2_i1	XLOC_000089	(CT)7	14	--	--	--	--	--	--	GGGCCACCAAACAGATGTA	59	TGAAGGAAAGAGGTTGTTGGA	60	182	--	--	--	--	--	--
TR10167|c1_g2_i1	XLOC_000089	(ATT)5	15	--	--	--	--	--	--	TCCAACAACCTCTTTCCTTCA	60	TTGGCTGAACCTTTTCCATC	60	225	--	--	--	--	--	--
TR10171|c1_g2_i1	XLOC_000097	(TC)11	22	TR10171|c1_g2_i1	(TC)11	Ca_LG_1	(TC)6	29691001	29691012	CAAGAGGGACAACAGACAGAGA	59	CAGAGACCAAGGGAAGCAAG	60	243		URR	--	--	--	--
TR10174|c0_g2_i1	XLOC_000098	(ACA)6tgtattagtggttgaagcaaagggtaaaaaagga atgatgtctcgtcaatttcagcgc(CCT)5	91	--	--	--	--	--	--	AGGTTCAAGGTTCCATGACG	60	GGTGCCACCCGATACAATAC	60	277	--	--	XM_004502278.1	--	[O]	--
TR10186|c0_g1_i1	No	(ATT)7	21	--	--	--	--	--	--	TTCATAGCATGCCCATTGAC	60	AAAACCATGGCACCTCTTTG	60	232	--	--	--	--	--	--
TR10186|c0_g1_i2	No	(ATT)7	21	--	--	--	--	--	--	TTCATAGCATGCCCATTGAC	60	AAAACCATGGCACCTCTTTG	60	232	--	--	--	--	--	--
TR10186|c0_g1_i4	No	(ATT)7	21	--	--	--	--	--	--	TTCATAGCATGCCCATTGAC	60	AAAACCATGGCACCTCTTTG	60	232	--	--	--	--	--	--
TR10201|c0_g1_i1	XLOC_000131	(GA)7	14	--	--	--	--	--	--	ACGACCAGTACCTCACCGAG	60	CCGAGTAGTTTCCCCTCTCC	60	138	--	--	EUC66211.1	--	--	--
TR10217|c0_g1_i1	No	(TCC)5	15	--	--	--	--	--	--	CAGGAAAAGCAACAGCAACA	60	TTATCGATGGCGGATCTAGG	60	172	--	--	--	--	--	--
TR10217|c0_g1_i2	No	(TCC)5	15	--	--	--	--	--	--	CAGGAAAAGCAACAGCAACA	60	TTATCGATGGCGGATCTAGG	60	172	--	--	--	--	--	--

<table>
<tr><th rowspan="3">Transcript IDs</th><th rowspan="3">Differentially expressed gene (DEG) IDs</th><th rowspan="3">SSR repeat-motifs</th><th rowspan="3">SSR repeat-length (bp)</th><th colspan="6">In silico polymorphic SSRs</th><th rowspan="3">Forward primers (5'-3')</th><th rowspan="3">Annealing tempearture(0C)</th><th rowspan="3">Reverse primers (5'-3')</th><th rowspan="3">Annealing tempearture(0C)</th><th rowspan="3">Amplified product size (bp)</th><th colspan="2">Structural annotation</th><th colspan="4">Functional Annotation</th></tr>
<tr><th colspan="2">C. microphyllum</th><th colspan="4">ICC4958</th><th rowspan="2">Gene IDs</th><th rowspan="2">Sequence component of genes</th><th rowspan="2">NCBI nr IDs</th><th rowspan="2">TFs</th><th rowspan="2">KOG</th><th rowspan="2">KEGG</th></tr>
<tr><th>Transcript</th><th>SSR repeat-motifs</th><th>Chromosomes</th><th>SSR repeat-motifs</th><th>Start physical positions (bp)</th><th>End physical positions (bp)</th></tr>
<tr><td>TR10217|c0_g1_i3</td><td>No</td><td>(TCC)5</td><td>15</td><td>--</td><td>--</td><td>--</td><td>--</td><td>--</td><td>--</td><td>CAGGAAAAGCAACAGCAACA</td><td>60</td><td>TTATCGATGGCGGATCTAGG</td><td>60</td><td>172</td><td>--</td><td>--</td><td>--</td><td>--</td><td>--</td><td>--</td></tr>
<tr><td>TR10217|c0_g1_i4</td><td>No</td><td>(TCC)5</td><td>15</td><td>--</td><td>--</td><td>--</td><td>--</td><td>--</td><td>--</td><td>CAGGAAAAGCAACAGCAACA</td><td>60</td><td>TTATCGATGGCGGATCTAGG</td><td>60</td><td>172</td><td>--</td><td>--</td><td>--</td><td>--</td><td>--</td><td>--</td></tr>
<tr><td>TR10217|c0_g1_i5</td><td>No</td><td>(TCC)5</td><td>15</td><td>--</td><td>--</td><td>--</td><td>--</td><td>--</td><td>--</td><td>CAGGAAAAGCAACAGCAACA</td><td>60</td><td>TTATCGATGGCGGATCTAGG</td><td>60</td><td>172</td><td>--</td><td>--</td><td>--</td><td>--</td><td>--</td><td>--</td></tr>
<tr><td>TR10218|c0_g1_i1</td><td>No</td><td>(AG)8</td><td>16</td><td>TR10218|c0_g1_i1</td><td>(AG)8</td><td>Ca_LG_2</td><td>(AG)11</td><td>25955367</td><td>25955388</td><td>CACACATCATCATCGACTTTACA</td><td>59</td><td>TCATTCCATCTTTCATGGCA</td><td>60</td><td>218</td><td></td><td>URR</td><td>--</td><td>--</td><td>--</td><td>--</td></tr>
<tr><td>TR10218|c0_g1_i2</td><td>No</td><td>(AG)8</td><td>16</td><td>--</td><td>--</td><td>--</td><td>--</td><td>--</td><td>--</td><td>CACACATCATCATCGACTTTACA</td><td>59</td><td>TCATTCCATCTTTCATGGCA</td><td>60</td><td>218</td><td>--</td><td>--</td><td>--</td><td>--</td><td>--</td><td>--</td></tr>
<tr><td>TR10218|c0_g1_i4</td><td>No</td><td>(AG)8</td><td>16</td><td>--</td><td>--</td><td>--</td><td>--</td><td>--</td><td>--</td><td>CACACATCATCATCGACTTTACA</td><td>59</td><td>TCATTCCATCTTTCATGGCA</td><td>60</td><td>218</td><td>--</td><td>--</td><td>--</td><td>--</td><td>--</td><td>--</td></tr>
<tr><td>TR10218|c0_g1_i5</td><td>No</td><td>(AG)8</td><td>16</td><td>--</td><td>--</td><td>--</td><td>--</td><td>--</td><td>--</td><td>CACACATCATCATCGACTTTACA</td><td>59</td><td>TCATTCCATCTTTCATGGCA</td><td>60</td><td>218</td><td>--</td><td>--</td><td>--</td><td>--</td><td>--</td><td>--</td></tr>
<tr><td>TR10218|c0_g1_i6</td><td>No</td><td>(AG)8</td><td>16</td><td>--</td><td>--</td><td>--</td><td>--</td><td>--</td><td>--</td><td>CACACATCATCATCGACTTTACA</td><td>59</td><td>TCATTCCATCTTTCATGGCA</td><td>60</td><td>218</td><td>--</td><td>--</td><td>--</td><td>--</td><td>--</td><td>--</td></tr>
<tr><td>TR10222|c5_g1_i1</td><td>No</td><td>(A)10ggtttttgttttacatgataagtactatgat(A)10</td><td>51</td><td>--</td><td>--</td><td>--</td><td>--</td><td>--</td><td>--</td><td>CAATTGCAGAGAGGCACAAA</td><td>60</td><td>TCAAACCTCGTCGTTTTCCT</td><td>60</td><td>135</td><td>--</td><td>--</td><td>--</td><td>--</td><td>--</td><td>--</td></tr>
<tr><td>TR10229|c0_g2_i2</td><td>XLOC_000175</td><td>(TAA)5aataaaaacaaaattgagttg(T)10</td><td>46</td><td>--</td><td>--</td><td>--</td><td>--</td><td>--</td><td>--</td><td>AACTTGCTTGCTTCCTTCCA</td><td>60</td><td>CAACCTTTTCACGTGTCTCTCA</td><td>60</td><td>252</td><td>--</td><td>--</td><td>--</td><td>--</td><td>--</td><td>--</td></tr>
<tr><td>TR10232|c1_g1_i1</td><td>No</td><td>(CT)8</td><td>16</td><td>--</td><td>--</td><td>--</td><td>--</td><td>--</td><td>--</td><td>CTCTTTTCATAAATTCGCTTCAA</td><td>58</td><td>CCATTTGTTCCTTCCTCGAA</td><td>60</td><td>101</td><td>--</td><td>--</td><td>--</td><td>--</td><td>--</td><td>--</td></tr>
<tr><td>TR10232|c1_g1_i2</td><td>No</td><td>(CT)8</td><td>16</td><td>--</td><td>--</td><td>--</td><td>--</td><td>--</td><td>--</td><td>CTCTTTTCATAAATTCGCTTCAA</td><td>58</td><td>CCATTTGTTCCTTCCTCGAA</td><td>60</td><td>101</td><td>--</td><td>--</td><td>--</td><td>--</td><td>--</td><td>--</td></tr>
<tr><td>TR10232|c1_g1_i3</td><td>No</td><td>(CT)8</td><td>16</td><td>--</td><td>--</td><td>--</td><td>--</td><td>--</td><td>--</td><td>CTCTTTTCATAAATTCGCTTCAA</td><td>58</td><td>CCATTTGTTCCTTCCTCGAA</td><td>60</td><td>101</td><td>--</td><td>--</td><td>--</td><td>--</td><td>--</td><td>--</td></tr>
<tr><td>TR10235|c0_g1_i1</td><td>No</td><td>(AG)11</td><td>22</td><td>--</td><td>--</td><td>--</td><td>--</td><td>--</td><td>--</td><td>CGTTGTCTAGGGTGCATACTG</td><td>58</td><td>ACCATTTCCAACCACTCCAA</td><td>60</td><td>280</td><td>--</td><td>--</td><td>--</td><td>--</td><td>--</td><td>--</td></tr>
</table>

Transcript IDs	Differentially expressed gene (DEG) IDs	SSR repeat-motifs	SSR repeat-length (bp)	*In silico* polymorphic SSRs						Forward primers (5'-3')	Annealing tempearture(0C)	Reverse primers (5'-3')	Annealing tempearture(0C)	Amplified product size (bp)	Structural annotation		Functional Annotation			
				C. microphyllum		ICC4958														
				Transcript	SSR repeat-motifs	Chromosomes	SSR repeat-motifs	Start physical positions (bp)	End physical positions (bp)						Gene IDs	Sequence component of genes	NCBI nr IDs	TFs	KOG	KEGG
TR10246|c0_g1_i1	No	(TA)7	14	--	--	--	--	--	--	ATTGCGCTTCACCCATAAAC	60	CCAAAATATAGGACATTGGCA	58	245	--	--	--	--	--	--
TR10282|c0_g1_i1	XLOC_000199	(CT)9	18	--	--	--	--	--	--	GCCTCTTTTCCTGCTCCTTT	60	CCGGTTCCTGCTAGTCATCT	59	217	--	--	--	--	--	--
TR10293|c0_g1_i1	XLOC_000204	(AG)6	12	--	--	--	--	--	--	GCAAGTTTCAACAACAAAAACAA	59	TGTCATGATGGTGATTGCCT	60	148	--	--	--	--	--	--
TR10361|c0_g1_i1	XLOC_000231	(TCC)5	15	--	--	--	--	--	--	TCCAAAAGCTTTGGTTCCAT	60	TGATTGCAATGACAAGAGGTG	60	167	--	--	--	--	--	--
TR10379|c0_g1_i1	XLOC_000239	(TC)6	12	--	--	--	--	--	--	GGCCACATACCAAACAGACA	59	GGAAATGACCAAAAGGGGAT	60	106	--	--	--	--	--	--
TR10402|c0_g1_i1	XLOC_000245	(AT)6	12	--	--	--	--	--	--	TTCCTTTCAAATCATCACTCTAACA	59	GCTTGAGGAGAAACCATTGC	60	184	--	--	--	--	--	--
TR10426|c0_g2_i1	XLOC_000257	(CAA)5	15	TR10426|c0_g2_i1	(CAA)5	Ca_LG_1	(CAA)6	5193860	5193877	TCATGTTTGGAAGTGAGGGA	59	TTCCCATTGGTTAAAATCAACA	59	152	Ca_17428	CDS	XP_004487066.1	MYB	[K]	--
TR10436|c0_g2_i1	XLOC_000271	(GAA)7	21	--	--	--	--	--	--	ACTGCAAGAGCCACTGTCCT	60	CCTAACGGTGTTCCATCCAT	60	101	--	--	--	--	--	--
TR10438|c2_g1_i1	XLOC_000275	(CCA)7	21	--	--	--	--	--	--	AAAAACCCTCTTTTGACCCTC	59	GTGCAGGTGAACCCGTTAGT	60	210	--	--	--	--	--	--
TR10438|c2_g1_i1	XLOC_000275	(CAG)5	15	--	--	--	--	--	--	GTTTTTCTCCTTTGCCTCCC	60	ATGTTGATGTTGCTGTTGCG	61	113	--	--	--	--	--	--
TR10440|c1_g1_i1	XLOC_000279	(AT)9	18	--	--	--	--	--	--	TGAATTAGTAAGGTTGGTAGGTGTT	57	ATTTGATGGGCTTTTGTTGC	60	149	--	--	--	--	--	--
TR10461|c0_g1_i1	XLOC_000297	(ACA)5acgttttctgtaacaactaacaacaaatt(A)15	59	--	--	--	--	--	--	CGCATACCACAAACAAACACA	60	AACCCATTTCACTCTGCTGC	60	203	--	--	--	--	--	--
TR10461|c0_g2_i1	No	(ACA)5acgttttctgtaacaactaacaacaaatt(A)15	59	--	--	--	--	--	--	CGCATACCACAAACAAACACA	60	AACCCATTTCACTCTGCTGC	60	203	--	--	--	--	--	--
TR10469|c0_g1_i1	XLOC_000306	(AGA)6	18	--	--	--	--	--	--	CATGAGAAGGCAATAGTTTTGG	59	TCAACTGAAAACTCATCACAACC	59	106	--	--	--	--	--	--

Transcript IDs	Differentially expressed gene (DEG) IDs	SSR repeat-motifs	SSR repeat-length (bp)	*In silico* polymorphic SSRs						Forward primers (5'-3')	Annealing tempearture(0C)	Reverse primers (5'-3')	Annealing tempearture(0C)	Amplified product size (bp)	Structural annotation		Functional Annotation			
				C. microphyllum		ICC4958									Gene IDs	Sequence component of genes	NCBI nr IDs	TFs	KOG	KEGG
				Transcript	SSR repeat-motifs	Chromosomes	SSR repeat-motifs	Start physical positions (bp)	End physical positions (bp)											
TR10470|c0_g1_i2	No	(TC)8	16	--	--	--	--	--	--	ATCTGCTCCTCCATTTCCCT	60	GTGGTTGCGCCATTAAAAAT	60	261	--	--	--	--	--	--
TR10470|c0_g1_i4	No	(TC)8	16	--	--	--	--	--	--	ATCTGCTCCTCCATTTCCCT	60	GTGGTTGCGCCATTAAAAAT	60	261	--	--	--	--	--	--
TR10501|c0_g1_i1	XLOC_000331	(A)10gaaacaagaacacacaccctatgtcaaaagcctacgaatacaaaggtgtaagtacatactactact(ACA)5	91	--	--	--	--	--	--	TCGAAATGGAGAGGAAATGC	60	TGGTGTGTTTGGATAAGAACAA	58	238	--	--	--	--	--	--
TR10501|c0_g1_i2	XLOC_000332	(A)10gaaacaagaacacacaccctatgtcaaaagcctacgaatacaaaggtgtaagtacatactactact(ACA)5gaaatacctc(T)10	111	--	--	--	--	--	--	AATCGATCTCAACTCAAACAAGT	57	TTGGGCTCCTTTACTTTCCA	60	254	--	--	XP_004508838.1	--	[R]	--
TR10541|c0_g1_i1	No	(AG)7	14	--	--	--	--	--	--	TTGTGGGGAAATGGGTAATG	60	TAATTCACATCCCACCCGTT	60	162	--	--	--	--	--	--
TR10558|c0_g1_i1	XLOC_000380	(GGA)5	15	--	--	--	--	--	--	GAAGAGTACGATCCGGCAAG	60	TTCTCTCTCCTTTTCCCTCTCTC	60	150	--	--	--	--	--	--
TR10561|c0_g1_i1	XLOC_000383	(GCA)5	15	--	--	--	--	--	--	GTCGAAGAGACCAAAACCCA	60	TCTGCTGCTGTGTCATACCC	60	276	--	--	CEO97280.1	--	[J]	--
TR10567|c0_g1_i1	XLOC_000388	(ATG)6	18	--	--	--	--	--	--	GAACAAGGCAATGATGGTGA	60	ATTTCACCCGAAATCATGGA	60	127	--	--	--	--	--	--
TR10599|c0_g2_i1	No	(TC)8	16	--	--	--	--	--	--	GGGAAAACGAACGTTGAAGA	60	TCTGGGAATGGTAATGGGAA	60	271	--	--	--	--	--	--
TR10654|c0_g1_i1	No	(AT)6	12	--	--	--	--	--	--	AATGGAAGACACGTCGGAAG	60	TGGTTCTTGAATCTTGTCCTTG	59	137	--	--	--	--	--	--
TR10835|c0_g1_i1	No	(AG)7	14	--	--	--	--	--	--	TTTACAGGGTCCACAGGAGC	60	ACAATCTTCTCCCTGCCAAA	60	103	--	--	--	--	--	--
TR10873|c0_g1_i1	No	(CT)8	16	--	--	--	--	--	--	TGCCAATTGCTACTGGTCAC	60	AAGAGAATTTTCAATTTTTGCCC	60	109	--	--	--	--	--	--
TR1090|c0_g1_i1	No	(GCT)5	15	--	--	--	--	--	--	AATCCGTCATGGGTCACATT	60	GCTACTCTTGTCGGTGGAGG	60	231	--	--	--	--	--	--
TR10914|c0_g1_i1	No	(AT)7	14	--	--	--	--	--	--	CACTGCACTGCATTTTCAAG	58	CCACTTAGTCCAACCTGCAT	58	180	--	--	--	--	--	--

Transcript IDs	Differentially expressed gene (DEG) IDs	SSR repeat-motifs	SSR repeat-length (bp)	*In silico* polymorphic SSRs						Forward primers (5'-3')	Annealing tempearture(0C)	Reverse primers (5'-3')	Annealing tempearture(0C)	Amplified product size (bp)	Structural annotation		Functional Annotation			
				C. microphyllum		ICC4958									Gene IDs	Sequence component of genes	NCBI nr IDs	TFs	KOG	KEGG
				Transcript	SSR repeat-motifs	Chromosomes	SSR repeat-motifs	Start physical positions (bp)	End physical positions (bp)											
TR10927|c0_g1_i1	XLOC_000465	(AT)6	12	--	--	--	--	--	--	AGGGATGACGATCAAGATGC	60	GGTATGGTAATTGCGGTTGG	60	157	--	--	XP_003737509.1	MYB_related	[J]	K03113
TR10949|c0_g1_i1	XLOC_000470	(TCA)7	21	--	--	--	--	--	--	GCTCCCCCTATTGTTCCTTT	59	AGGTCATCGTCTTTGGCAAG	60	162	--	--	--	--	--	--
TR10976|c0_g1_i1	XLOC_000476	(TTA)6	18	--	--	--	--	--	--	AATTGTAGGCACCAACACCC	60	ACAAATGGCTCAATTCCCAG	60	128	--	--	--	--	--	--
TR11053|c0_g1_i1	No	(CTT)5	15	--	--	--	--	--	--	TGGAACCGTTTTGACAATGA	60	AACAGAGCTGGCTTCTTGGA	60	164	--	--	--	--	--	--
TR11108|c0_g1_i1	No	(CAC)5	15	--	--	--	--	--	--	ACGACGACAACCAGCAGAGT	61	CAACACACAATCGGTCCAAG	60	185	--	--	--	--	--	--
TR11186|c0_g1_i1	No	(TCT)5	15	--	--	--	--	--	--	CAATCTTGTCATCCGATCCC	60	CACCTGAAGCCAAGAAGCTC	60	249	--	--	--	--	--	--
TR1124|c0_g1_i1	No	(AG)6	12	--	--	--	--	--	--	TGCACATCAAATACACAATTTCA	59	GCCGTTGGAGTTTCAGGTTA	60	129	--	--	--	--	--	--
TR11287|c0_g1_i1	No	(GGA)5	15	--	--	--	--	--	--	GACATTCCTTGCTTTGGCTT	59	ATGGGGTGTTGCTGGTAAAG	60	147	--	--	--	--	--	--
TR11424|c1_g1_i1	No	(GA)9aatggtcgtgtggtgatccgcaaaacagagttattcaactacaaatatgg(GA)9	86	--	--	--	--	--	--	CCAAGATCGAAGGCAAGAAG	60	CAAGAGTGACCTTCACGATGAC	60	145	--	--	--	--	--	--
TR11443|c0_g1_i1	XLOC_000585	(TAA)5	15	--	--	--	--	--	--	CCTCAATCCTAATACCCCCA	59	TGTGAACTTTCAGGCCAATG	60	146	--	--	--	--	--	--
TR11448|c0_g2_i1	XLOC_000587	(TCA)6	18	--	--	--	--	--	--	CCAACAACACATGCTGGTTC	60	GCATCAACAACAGAAGCATGA	60	119	--	--	--	--	--	--
TR11456|c0_g1_i1	XLOC_000590	(TA)11	22	TR11456|c0_g1_i1	(TA)11	Ca_LG_1	(TA)10	29233917	29233936	TGCTACAAAAGTTAGCATGGG	58	TTGGTTGGACAGTGATGATGA	60	214		URR	--	--	--	--
TR11456|c0_g2_i1	No	(TA)11	22	--	--	--	--	--	--	TGCTACAAAAGTTAGCATGGG	58	TTGGTTGGACAGTGATGATGA	60	214	--	--	--	--	--	--
TR11458|c0_g1_i1	No	(TTC)5	15	--	--	--	--	--	--	TCGCCCTGTACACCATGTAA	60	AGAACTTTGTGCCTCGGCTA	60	136	--	--	--	--	--	--

Transcript IDs	Differentially expressed gene (DEG) IDs	SSR repeat-motifs	SSR repeat-length (bp)	*In silico* polymorphic SSRs						Forward primers (5'-3')	Annealing tempearture(0C)	Reverse primers (5'-3')	Annealing tempearture(0C)	Amplified product size (bp)	Structural annotation		Functional Annotation			
				C. microphyllum		ICC4958														
				Transcript	SSR repeat-motifs	Chromosomes	SSR repeat-motifs	Start physical positions (bp)	End physical positions (bp)						Gene IDs	Sequence component of genes	NCBI nr IDs	TFs	KOG	KEGG
TR11498|c0_g1_i1	No	(GAA)10	30	--	--	--	--	--	--	TCCTCTAGGGAAACACCTCA	57	CAACCAGAAGCAGCATTTGA	60	176	--	--	--	--	--	--
TR11498|c0_g2_i1	No	(GAA)13	39	--	--	--	--	--	--	TCCTCTAGGGAAACACCTCA	57	CAACCAGAAGCAGCATTTGA	60	185	--	--	--	--	--	--
TR1157|c0_g1_i1	No	(GAA)5	15	--	--	--	--	--	--	TGAAAGTGACGAAGACGACG	60	ATGCAAACATGGCATACGAA	60	226	--	--	--	--	--	--
TR1157|c0_g2_i1	No	(GAA)5	15	--	--	--	--	--	--	TGAAAGTGACGAAGACGACG	60	CCTACCTCCAACCAATCACG	60	267	--	--	--	--	--	--
TR11621|c0_g1_i1	XLOC_000640	(AG)7	14	--	--	--	--	--	--	ATGCGTGAGTGTGTGTGTGA	60	AAATCAATGACGAGCGAAGG	60	277	--	--	KFM79448.1	MYB_related	[C]	K01647
TR11646|c1_g1_i1	XLOC_000654	(AG)6	12	--	--	--	--	--	--	TGAAACAAATGTGCTTGGGA	60	TGTTCCGGTAAACCACCAAT	60	239	--	--	--	--	--	--
TR11655|c0_g1_i1	No	(AC)8	16	--	--	--	--	--	--	TTGCTTTCTCGGAGCATTTT	60	TGGTTCTAAGGGGAACACAAA	59	150	--	--	--	--	--	--
TR11761|c0_g1_i1	XLOC_000704	(T)17gtattttaatggaaaatcatt(AGA)5	53	--	--	--	--	--	--	GAAGGCGTGTTGCTCAAAGT	60	AGAAAGTGGGTTTGTGTGGC	60	274	--	--	--	--	--	--
TR11823|c0_g1_i1	No	(T)10aaaaaaacaatgaagggaaatcggctctgcccctaccaacttctcaatgttcca(T)14	78	--	--	--	--	--	--	GAGAAATCGGTCATGCATCC	60	TATTATGAAAGATGTTAACGAGAAAAA	57	180	--	--	--	--	--	--
TR11849|c0_g1_i1	XLOC_000727	(CGA)5	15	--	--	--	--	--	--	AATTACGACCACCTGCCATC	60	TGATTTTGGATCTTCGGAGG	60	200	--	--	--	--	--	--
TR1185|c0_g1_i1	No	(TG)8	16	--	--	--	--	--	--	TGGAACACCTCGTATGTGGA	60	TGATCAACATCGAGAAACACAA	59	238	--	--	--	--	--	--
TR11920|c0_g1_i1	No	(ATT)7	21	--	--	--	--	--	--	TCATGATTGAGCACTCACCAC	60	CCAAAATTAACGTGACCCCA	61	209	--	--	--	--	--	--
TR11968|c0_g1_i1	No	(TA)10	20	--	--	--	--	--	--	CCATCTTAACTAAGCATGACGA	57	TGCAGGTTTAGCAGTAGCCA	60	102	--	--	--	--	--	--
TR11968|c0_g2_i1	No	(TA)10	20	--	--	--	--	--	--	CCATCTTAACTAAGCATGACGA	57	TGCAGGTTTAGCAGTAGCCA	60	102	--	--	--	--	--	--

Transcript IDs	Differentially expressed gene (DEG) IDs	SSR repeat-motifs	SSR repeat-length (bp)	*In silico* polymorphic SSRs						Forward primers (5'-3')	Annealing tempearture(0C)	Reverse primers (5'-3')	Annealing tempearture(0C)	Amplified product size (bp)	Structural annotation		Functional Annotation			
				C. microphyllum		ICC4958									Gene IDs	Sequence component of genes	NCBI nr IDs	TFs	KOG	KEGG
				Transcript	SSR repeat-motifs	Chromosomes	SSR repeat-motifs	Start physical positions (bp)	End physical positions (bp)											
TR11971|c0_g1_i1	No	(AAC)5	15	--	--	--	--	--	--	CCGTTGGAGTTTCGTCAAAT	60	CGGATGATTCCGTTACTGCT	60	207	--	--	--	--	--	--
TR11971|c0_g1_i1	No	(TC)10	20	--	--	--	--	--	--	TCACTCCAAAGACCCATCCT	60	ATTTGACGAAACTCCAACGG	60	216	--	--	--	--	--	--
TR11971|c0_g1_i2	No	(AAC)5	15	--	--	--	--	--	--	CCGTTGGAGTTTCGTCAAAT	60	CGGATGATTCCGTTACTGCT	60	207	--	--	--	--	--	--
TR11971|c0_g1_i2	No	(TC)10	20	--	--	--	--	--	--	TCACTCCAAAGACCCATCCT	60	ATTTGACGAAACTCCAACGG	60	216	--	--	--	--	--	--
TR11973|c0_g1_i1	XLOC_000776	(T)11(TA)6*	22	--	--	--	--	--	--	TGAAATAAGCATGCAGCCAC	60	GGCTTCTCGATGAAAAACCA	60	242	--	--	XM_004495335.1	ARF	[TR]	K14484
TR12015|c0_g1_i2	XLOC_000800	(GAT)7	21	--	--	--	--	--	--	ACGCTTTCGTCGTTGTTTCT	60	CAACAATAAATCAACAACTTCGATCT	60	137	--	--	--	--	--	--
TR12022|c0_g1_i1	No	(CCA)5	15	--	--	--	--	--	--	TCATCTACCACAGTTATTCAGTCCA	60	AAGAGGCAGGAGTTGTGAGC	60	125	--	--	--	--	--	--
TR12040|c0_g1_i1	XLOC_000811	(TC)9	18	--	--	--	--	--	--	CAGCCAAAAGGAGAAGGAAA	59	GCAGAGTCTCTCTCTTCTGTGC	59	212	--	--	--	--	--	--
TR12079|c0_g1_i1	No	(TGT)5	15	--	--	--	--	--	--	TGAGGATCCGGTCAAATACC	60	AGCTTCTCCAGGGGAAAAAG	60	253	--	--	--	--	--	--
TR12079|c0_g2_i4	No	(TGT)5	15	--	--	--	--	--	--	TGAGGATCCGGTCAAATACC	60	AGCTTCTCCAGGGGAAAAAG	60	253	--	--	--	--	--	--
TR12094|c0_g2_i2	No	(TC)7	14	--	--	--	--	--	--	CAATTCTTTTCCTTCAGGCG	60	GAGGTGGACTCGTTGCATCT	60	112	--	--	--	--	--	--
TR12095|c0_g1_i2	No	(TC)8	16	--	--	--	--	--	--	CCACCACCATCTCATCTCAA	59	TGTTGCACCTCCAACAACAA	61	267	--	--	--	--	--	--
TR12095|c0_g1_i3	No	(TC)8	16	--	--	--	--	--	--	CCACCACCATCTCATCTCAA	59	CCAAGATGCAAATCAACACG	60	278	--	--	--	--	--	--
TR12095|c0_g1_i4	No	(TC)8	16	--	--	--	--	--	--	CCACCACCATCTCATCTCAA	59	TGTTGCACCTCCAACAACAA	61	267	--	--	--	--	--	--

Transcript IDs	Differentially expressed gene (DEG) IDs	SSR repeat-motifs	SSR repeat-length (bp)	*In silico* polymorphic SSRs						Forward primers (5'-3')	Annealing tempearture(0C)	Reverse primers (5'-3')	Annealing tempearture(0C)	Amplified product size (bp)	Structural annotation		Functional Annotation			
				C. microphyllum		ICC4958									Gene IDs	Sequence component of genes	NCBI nr IDs	TFs	KOG	KEGG
				Transcript	SSR repeat-motifs	Chromosomes	SSR repeat-motifs	Start physical positions (bp)	End physical positions (bp)											
TR12105|c0_g1_i1	XLOC_000841	(TCT)5	15	TR12105|c0_g1_i1	(TCT)5	Ca_LG_1	(TCT)6	1230561	1230578	TATTCGAGTTGAGTGCGGTG	60	AAGGAGGGAGAATTCCCAGA	60	280	Ca_11263	CDS	--	--	--	--
TR12123|c0_g1_i1	XLOC_000858	(A)13gatcggaggagaaatttcaag(TC)10	54	--	--	--	--	--	--	TCATGAGGGATGATGATGACA	60	CACGATAGACACGTGGAACG	60	201	--	--	--	--	--	--
TR12137|c0_g1_i1	No	(TTC)6cgcctcgaggcaccaaccacaaacatcaacaattacaggtaattcagaa(AAC)5	82	--	--	--	--	--	--	CAAGAACTTGCTCGTCCTCC	60	GCTTGAAATTTGGCGTTGTT	60	240	--	--	--	--	--	--
TR12137|c0_g1_i1	No	(GT)9ggaaggaagctttcaatagcagagaagagataacgatgtgtgaaagtttta(CAT)5	84	--	--	--	--	--	--	CGCGAAGAGAAAGAGACGAG	60	TCGAATCCATTGGAGAATCC	60	268	--	--	--	--	--	--
TR12137|c0_g2_i1	No	(GT)9ggaaggaagctttcaatagcagagaagagataacgatgtgtgaaagtttta(CAT)5	84	--	--	--	--	--	--	CGCGAAGAGAAAGAGACGAG	60	TCGAATCCATTGGAGAATCC	60	268	--	--	--	--	--	--
TR12146|c0_g1_i1	No	(ATC)5	15	--	--	--	--	--	--	ATCCATCAACCCCAAAACAA	60	GGTGAATGGAAACCAAAGGA	60	274	--	--	--	--	--	--
TR12146|c0_g1_i2	No	(ATC)5	15	--	--	--	--	--	--	ATCCATCAACCCCAAAACAA	60	GGTGAATGGAAACCAAAGGA	60	274	--	--	--	--	--	--
TR12157|c0_g1_i1	XLOC_000889	(CAA)5	15	--	--	--	--	--	--	TAGCCCAGCCCAAATATCAG	60	CTAGTTTGTCGGAACGGGAG	60	275	--	--	--	--	--	--
TR12163|c0_g1_i1	No	(TGT)5	15	--	--	--	--	--	--	ATCTTTTCATTTCCTGCCCC	60	AACAACAACCCCAACGGTAA	60	129	--	--	--	--	--	--
TR12163|c0_g2_i1	XLOC_000892	(TGT)5	15	--	--	--	--	--	--	CCAGTGTTGCTGTTGCTGTT	60	AATGGTTGCTTCTTTGGCAC	60	221	--	--	--	--	--	--
TR12165|c0_g1_i1	XLOC_000894	(ATC)5	15	--	--	--	--	--	--	CACCACCTCTCTTCCATTCC	60	CCCCACAAGTTCCTCTTCTG	60	151	--	--	--	--	--	--
TR12193|c0_g1_i1	XLOC_000905	(TTG)5tta(GTG)6	36	--	--	--	--	--	--	GCAATCTGCCTATTTTGCGT	60	GACGAGCAGTCGTGCAAATA	60	266	--	--	BAH71195.1	--	--	--
TR12370|c0_g2_i1	No	(TA)6	12	TR12370|c0_g2_i1	(TA)6	Ca_LG_5	(TA)16	36419870	36419901	TGTTCAATTCCCCACCTAGC	60	CTTCTAGAGGCACCCCATGA	60	277	Ca_06623	CDS	--	--	--	--
TR12370|c0_g2_i2	No	(TA)6	12	--	--	--	--	--	--	TGTTCAATTCCCCACCTAGC	60	CTTCTAGAGGCACCCCATGA	60	277	--	--	--	--	--	--

Transcript IDs	Differentially expressed gene (DEG) IDs	SSR repeat-motifs	SSR repeat-length (bp)	*In silico* polymorphic SSRs						Forward primers (5'-3')	Annealing tempearture(0C)	Reverse primers (5'-3')	Annealing tempearture(0C)	Amplified product size (bp)	Structural annotation		Functional Annotation			
				C. microphyllum		ICC4958														
				Transcript	SSR repeat-motifs	Chromosomes	SSR repeat-motifs	Start physical positions (bp)	End physical positions (bp)						Gene IDs	Sequence component of genes	NCBI nr IDs	TFs	KOG	KEGG
TR12396|c0_g2_i1	XLOC_000953	(TG)6	12	--	--	--	--	--	--	TCGATAGTGGTACCCTTGCC	60	TTCCATCAAATCTAGCGATCC	59	172	--	--	--	--	--	--
TR12396|c0_g2_i2	XLOC_000954	(TG)6	12	--	--	--	--	--	--	TCGATAGTGGTACCCTTGCC	60	TTCCATCAAATCTAGCGATCC	59	172	--	--	--	--	--	--
TR12557|c0_g1_i1	No	(TG)6	12	--	--	--	--	--	--	AGCCCACACTGGATCAATTC	60	TCAAAGGATGGAGAAGGGTG	60	253	--	--	--	--	--	--
TR12593|c0_g1_i1	No	(TCT)6	18	--	--	--	--	--	--	GCTGGGCGAATTATGTTCTC	60	CAAGAACCACACAAAAGGGAA	60	155	--	--	--	--	--	--
TR12627|c1_g1_i1	XLOC_001061	(CT)8	16	--	--	--	--	--	--	TTCCCACACCAACCCTTCTA	60	CAAGCACAGAATGACCCAAA	60	273	--	--	--	--	--	--
TR12664|c1_g1_i1	No	(TC)9(TA)8	34	--	--	--	--	--	--	TTTGCAAGTGCAGGTTTGAC	60	GCTGGAATATTAATGACACACCC	60	268	--	--	--	--	--	--
TR12671|c2_g1_i1	XLOC_001101	(T)10aaaaaaaattttgacaattttc(T)12	44	--	--	--	--	--	--	AAATTTTGTGGGCTTAGGGG	60	TTCTCAAATCAAAAAGGATTTCG	60	181	--	--	--	--	--	--
TR12691|c2_g2_i3	No	(CTA)5	15	--	--	--	--	--	--	CCATCATAGTTTGCTTTTGCC	60	TAGGGTTTCAAAGTCGGTGG	60	250	--	--	--	--	--	--
TR12691|c2_g2_i4	No	(CTA)5	15	--	--	--	--	--	--	CCATCATAGTTTGCTTTTGCC	60	TAGGGTTTCAAAGTCGGTGG	60	250	--	--	--	--	--	--
TR12697|c0_g1_i1	No	(TTC)6	18	--	--	--	--	--	--	GCGATGAACAAATCAAGGGT	60	TGTGAAGAGCAATCGCAGAG	60	182	--	--	--	--	--	--
TR12697|c0_g2_i2	No	(TTC)6	18	--	--	--	--	--	--	GCGATGAACAAATCAAGGGT	60	AATGAAATGTTGCTCAGCCC	60	224	--	--	--	--	--	--
TR12705|c0_g2_i1	No	(CAC)6	18	--	--	--	--	--	--	ACCAGAATTTGTGCCCACTC	60	ATGAGACCGGTAGTGAACCG	60	223	--	--	--	--	--	--
TR12705|c0_g2_i2	No	(CAC)6	18	--	--	--	--	--	--	ACCAGAATTTGTGCCCACTC	60	ATGAGACCGGTAGTGAACCG	60	223	--	--	--	--	--	--
TR12705|c0_g2_i3	No	(CAC)6	18	--	--	--	--	--	--	ACCAGAATTTGTGCCCACTC	60	ATGAGACCGGTAGTGAACCG	60	223	--	--	--	--	--	--

**Transcript IDs**	**Differentially expressed gene (DEG) IDs**	**SSR repeat-motifs**	**SSR repeat-length (bp)**	***In silico* polymorphic SSRs**						**Forward primers (5'-3')**	**Annealing tempearture(0C)**	**Reverse primers (5'-3')**	**Annealing tempearture(0C)**	**Amplified product size (bp)**	**Structural annotation**		**Functional Annotation**			
				C. microphyllum		**ICC4958**									**Gene IDs**	**Sequence component of genes**	**NCBI nr IDs**	**TFs**	**KOG**	**KEGG**
				**Transcript**	**SSR repeat-motifs**	**Chromosomes**	**SSR repeat-motifs**	**Start physical positions (bp)**	**End physical positions (bp)**											
TR12705|c0_g2_i4	No	(CAC)6	18	--	--	--	--	--	--	ACCAGAATTTGTGCCCACTC	60	ATGAGACCGGTAGTGAACCG	60	223	--	--	--	--	--	--
TR12705|c0_g2_i5	No	(CAC)6	18	--	--	--	--	--	--	ACCAGAATTTGTGCCCACTC	60	ATGAGACCGGTAGTGAACCG	60	223	--	--	--	--	--	--
TR12705|c0_g2_i6	No	(CAC)6	18	--	--	--	--	--	--	ACCAGAATTTGTGCCCACTC	60	ATGAGACCGGTAGTGAACCG	60	223	--	--	--	--	--	--
TR12720|c0_g1_i4	No	(CTC)5	15	--	--	--	--	--	--	CACTTCTCGCTTTCGCTCTT	60	GATTGGGATCAGAGTTTGGG	59	139	--	--	--	--	--	--
TR12734|c0_g1_i2	No	(GAA)5	15	--	--	--	--	--	--	TTGGAGGTGTTTGTTTGGGT	60	TCTTCTGTTTGCGTTCATCG	60	230	--	--	--	--	--	--
TR12740|c0_g1_i1	XLOC_001155	(ACG)5	15	--	--	--	--	--	--	CGATGACGACAGCGAAACTA	60	GGAGAGGAGGGAGAGAGGAA	60	143	--	--	--	--	--	--
TR12743|c0_g1_i1	XLOC_001159	(CAC)6	18	--	--	--	--	--	--	TTGGCAACAGCATCTCAAAG	60	TCTCCCCAAACATCTCAACC	60	175	--	--	--	--	--	--
TR12744|c0_g2_i1	XLOC_001160	(AT)7	14	--	--	--	--	--	--	TGGTCAACCCTTACCACCAT	60	CAAGAGACGGCTCAAGGAGT	60	250	--	--	--	--	--	--
TR12746|c0_g1_i2	No	(A)10gcaagataaggtatcacaattatttagcaagttgag agcatctaattatctgtccaaatgaaaccaacgcc(T)10	91	--	--	--	--	--	--	CCTGGCAAGTGGCAACTATT	60	ACTCATCACCATCTCTCTTTTCA	58	274	--	--	--	--	--	--
TR12746|c0_g2_i1	No	(TA)6(A)17*	28	--	--	--	--	--	--	CCCCCGAAAACTATTGATGA	60	TTGGCTGCAACGTTAGTCAA	60	251	--	--	--	--	--	--
TR12746|c1_g1_i1	XLOC_001162	(TTC)5	15	TR12746|c1_g1_i1	(TTC)5	Ca_LG_6	(TTC)6	6191221	6191238	CTGTTTCATCGTCTTCGTCG	59	CCCCTTCTCAAACAAACCAA	60	217	Ca_13288	DRR	--	--	--	--
TR12746|c1_g1_i1	XLOC_001162	(TGT)7tagcagggaagagaaagggt(GAA)5	56	TR12746|c1_g1_i1	(TTC)5	Ca_LG_6	(TTC)6	6191221	6191238	TTGGTTTGTTTGAGAAGGGG	60	CCATACCATCCACCACCATT	60	153	Ca_13288	DRR	--	--	--	--
TR12749|c0_g1_i1	No	(A)10tcataagggaaaataaaagaatacaactgctcatt ggcccaag(A)10	63	--	--	--	--	--	--	TGGTTTACCACACCAACCAA	60	AGGGCTAGCTGCTTCACAAG	60	209	--	--	--	--	--	--
TR12749|c0_g1_i2	No	(A)10tcataagggaaaataaaagaatacaactgctcatt ggcccaag(A)10	63	--	--	--	--	--	--	TGGTTTACCACACCAACCAA	60	AGGGCTAGCTGCTTCACAAG	60	209	--	--	--	--	--	--

Transcript IDs	Differentially expressed gene (DEG) IDs	SSR repeat-motifs	SSR repeat-length (bp)	*In silico* polymorphic SSRs						Forward primers (5'-3')	Annealing tempearture(0C)	Reverse primers (5'-3')	Annealing tempearture(0C)	Amplified product size (bp)	Structural annotation		Functional Annotation			
				C. microphyllum		ICC4958														
				Transcript	SSR repeat-motifs	Chromosomes	SSR repeat-motifs	Start physical positions (bp)	End physical positions (bp)						Gene IDs	Sequence component of genes	NCBI nr IDs	TFs	KOG	KEGG
TR12749|c0_g1_i3	No	(A)10tcataagggaaaataaaagaatacaactgctcattggcccaag(A)10	63	--	--	--	--	--	--	TGGTTTACCACACCAACCAA	60	AGGGCTAGCTGCTTCACAAG	60	209	--	--	--	--	--	--
TR12749|c0_g1_i4	No	(A)10tcataagggaaaataaaagaatacaactgctcattggcccaag(A)10	63	--	--	--	--	--	--	TGGTTTACCACACCAACCAA	60	AGGGCTAGCTGCTTCACAAG	60	209	--	--	--	--	--	--
TR12749|c0_g1_i5	No	(A)10tcataagggaaaataaaagaatacaactgctcattggcccaag(A)10	63	--	--	--	--	--	--	TGGTTTACCACACCAACCAA	60	AGGGCTAGCTGCTTCACAAG	60	209	--	--	--	--	--	--
TR12749|c0_g1_i6	No	(A)10tcataagggaaaataaaagaatacaactgctcattggcccaag(A)10	63	--	--	--	--	--	--	TGGTTTACCACACCAACCAA	60	AGGGCTAGCTGCTTCACAAG	60	209	--	--	--	--	--	--
TR12749|c0_g1_i7	No	(A)10tcataagggaaaataaaagaatacaactgctcattggcccaag(A)10	63	--	--	--	--	--	--	TGGTTTACCACACCAACCAA	60	AGGGCTAGCTGCTTCACAAG	60	209	--	--	--	--	--	--
TR12749|c0_g1_i8	No	(A)10tcataagggaaaataaaagaatacaactgctcattggcccaag(A)10	63	--	--	--	--	--	--	TGGTTTACCACACCAACCAA	60	AGGGCTAGCTGCTTCACAAG	60	209	--	--	--	--	--	--
TR12749|c0_g1_i9	No	(A)10tcataagggaaaataaaagaatacaactgctcattggcccaag(A)10	63	--	--	--	--	--	--	TGGTTTACCACACCAACCAA	60	AGGGCTAGCTGCTTCACAAG	60	209	--	--	--	--	--	--
TR12756|c0_g2_i1	XLOC_001172	(TTCT)6ttgtttctgatctcacgtctcaac(CA)7	62	--	--	--	--	--	--	GCTCTTCAAATTTAGCCATTCC	59	CTTCCTAGGCGGCTGTACC	60	251	--	--	--	--	--	--
TR12766|c0_g1_i1	No	(TCT)5	15	--	--	--	--	--	--	ACACGAAACCAACTCCTTCG	60	AACAGCAACAACCTCCCATC	60	185	--	--	--	--	--	--
TR12766|c0_g1_i2	No	(TCT)5	15	--	--	--	--	--	--	ACACGAAACCAACTCCTTCG	60	AACAGCAACAACCTCCCATC	60	159	--	--	--	--	--	--
TR12766|c0_g1_i3	No	(TCT)5	15	--	--	--	--	--	--	ACACGAAACCAACTCCTTCG	60	AACAGCAACAACCTCCCATC	60	244	--	--	--	--	--	--
TR12766|c0_g1_i4	XLOC_001181	(TCT)5	15	TR12766|c0_g1_i4	(TCT)5	Ca_LG_6	(TCT)11	4244376	4244408	ACACGAAACCAACTCCTTCG	60	GGAACTCGAGCTGCAAGAAA	61	234	Ca_13163	DRR	--	--	--	--
TR12766|c0_g1_i5	No	(TCT)5	15	--	--	--	--	--	--	ACACGAAACCAACTCCTTCG	60	ATTGGACGCATCTTTTCACC	60	214	--	--	--	--	--	--
TR12769|c1_g1_i1	XLOC_001185	(CA)6	12	--	--	--	--	--	--	CAACCAATTTCGTCCTTGAAA	60	GTCGGCTTTCGGTGTGTAAT	60	189	--	--	--	--	--	--

Transcript IDs	Differentially expressed gene (DEG) IDs	SSR repeat-motifs	SSR repeat-length (bp)	*In silico* polymorphic SSRs						Forward primers (5'-3')	Annealing tempearture(0C)	Reverse primers (5'-3')	Annealing tempearture(0C)	Amplified product size (bp)	Structural annotation		Functional Annotation			
				C. microphyllum		ICC4958									Gene IDs	Sequence component of genes	NCBI nr IDs	TFs	KOG	KEGG
				Transcript	SSR repeat-motifs	Chromosomes	SSR repeat-motifs	Start physical positions (bp)	End physical positions (bp)											
TR12771|c0_g1_i1	XLOC_001187	(GAT)5	15	--	--	--	--	--	--	TCTCGTTGATGGGTTGTTGA	60	TTAAGGTGCATTGAGGACCC	60	239	--	--	--	--	--	--
TR12771|c1_g1_i1	XLOC_001188	(CAT)5	15	--	--	--	--	--	--	TGAGAGGACGAAGGAAGAGG	60	TTGTTAACCGTCCAACGACA	60	216	--	--	--	--	--	--
TR12779|c1_g1_i6	No	(TA)6	12	--	--	--	--	--	--	CAGCGTGGATGTTAATGCAG	60	CCTCATTAGGATTCGGTCCA	60	187	--	--	--	--	--	--
TR12780|c1_g1_i2	No	(CT)11	22	--	--	--	--	--	--	CATCAACACTCACCAATGGC	60	TTTGGTGGGAATGGATGAAT	60	107	--	--	--	--	--	--
TR12785|c0_g1_i2	No	(TAA)5	15	--	--	--	--	--	--	CGTTTTCTGCGTACCCATTT	60	CGGTTTGAACCCTTCATTTT	59	149	--	--	--	--	--	--
TR12875|c0_g1_i1	No	(CAG)5cgg(CAA)5	33	--	--	--	--	--	--	CTCGCGGTTATGATTTCGAT	60	GTTGTTGCTGCTGTTGTTGC	61	255	--	--	--	--	--	--
TR12885|c0_g1_i1	XLOC_001232	(CTG)5	15	--	--	--	--	--	--	CATCCAACATCATGTCATCCA	60	GCACGTTTGAAGATGAGACG	59	247	--	--	--	--	--	--
TR12890|c0_g1_i1	No	(TG)7	14	--	--	--	--	--	--	AATTTGTGTCAACATCCGCA	60	TCTTTCTGTGGCAGCAAATG	60	205	--	--	--	--	--	--
TR12932|c0_g1_i1	No	(TC)6	12	--	--	--	--	--	--	CTTTTCATCCTTCACTCACTCG	59	CTTGCGGAAAGTCAAAGGAC	60	208	--	--	--	--	--	--
TR12966|c0_g2_i1	No	(CTA)8	24	--	--	--	--	--	--	ATTAGCTTCCTCCACACCCC	60	TGCAAACCACTTTTCCTTCTG	60	203	--	--	--	--	--	--
TR12970|c0_g1_i1	XLOC_001261	(CT)7	14	--	--	--	--	--	--	CCCAGTAACCGCTCTCTCTG	60	AGCTGGTGAAGCAATCCAAC	60	266	--	--	--	--	--	--
TR12987|c0_g1_i1	No	(TC)10	20	--	--	--	--	--	--	TTCGCACTTTGTCTTCGTCTT	60	AAACTCGCCAGCCACATAAT	60	185	--	--	--	--	--	--
TR12987|c0_g2_i1	No	(TC)10	20	--	--	--	--	--	--	TTCGCACTTTGTCTTCGTCTT	60	AAACTCGCCAGCCACATAAT	60	167	--	--	--	--	--	--
TR12987|c0_g3_i1	No	(TC)10	20	--	--	--	--	--	--	TTCGCACTTTGTCTTCGTCTT	60	AAACTCGCCAGCCACATAAT	60	184	--	--	--	--	--	--

Transcript IDs	Differentially expressed gene (DEG) IDs	SSR repeat-motifs	SSR repeat-length (bp)	*In silico* polymorphic SSRs						Forward primers (5'-3')	Annealing tempearture(0C)	Reverse primers (5'-3')	Annealing tempearture(0C)	Amplified product size (bp)	Structural annotation		Functional Annotation			
				C. microphyllum		ICC4958									Gene IDs	Sequence component of genes	NCBI nr IDs	TFs	KOG	KEGG
				Transcript	SSR repeat-motifs	Chromosomes	SSR repeat-motifs	Start physical positions (bp)	End physical positions (bp)											
TR12987|c0_g4_i1	XLOC_001274	(CT)8	16	--	--	--	--	--	--	ACGGTGGTGCAGTACTTGAG	59	ACCTTGCATCCATTGAGGTC	60	277	--	--	--	--	--	--
TR13000|c0_g1_i1	No	(TC)11	22	--	--	--	--	--	--	TCTTCTTCCATCCATAACCCA	59	TGGACTTTGTGTGAGGCTTG	60	183	--	--	--	--	--	--
TR13000|c0_g2_i1	XLOC_001285	(TC)11	22	--	--	--	--	--	--	TCTTCTTCCATCCATAACCCA	59	TGGACTTTGTGTGAGGCTTG	60	183	--	--	--	--	--	--
TR13013|c0_g1_i1	XLOC_001300	(ACA)5	15	--	--	--	--	--	--	TCCATTCCATTCCATTCTCC	60	GGCGAAGAAGCTGGAAACTA	60	278	--	--	--	--	--	--
TR13015|c0_g1_i1	No	(ACC)5	15	--	--	--	--	--	--	TCTTCTCCACCCGAATCAAC	60	GCACCTTGCTTAAGCGTTTC	60	200	--	--	--	--	--	--
TR13015|c0_g1_i2	No	(ACC)5	15	--	--	--	--	--	--	TCTTCTCCACCCGAATCAAC	60	GCACCTTGCTTAAGCGTTTC	60	200	--	--	--	--	--	--
TR13015|c0_g1_i3	No	(ACC)5	15	--	--	--	--	--	--	TCTTCTCCACCCGAATCAAC	60	GCACCTTGCTTAAGCGTTTC	60	200	--	--	--	--	--	--
TR13015|c0_g1_i4	No	(ACC)5	15	--	--	--	--	--	--	TCTTCTCCACCCGAATCAAC	60	GCACCTTGCTTAAGCGTTTC	60	200	--	--	--	--	--	--
TR13018|c1_g1_i1	No	(A)10catgaatagcagtggtggcagtaacggagggatg atgatgggtttcagtagtagagtagtgtcacaaagaaatca aacaaaacaaccaaaaccc(A)14	118	--	--	--	--	--	--	TGTTCGTTCGTTGCCTACAC	60	ATCGAACTTGTCGGAACCAT	59	188	--	--	--	--	--	--
TR13044|c0_g1_i3	No	(TA)6cac(GT)7	29	--	--	--	--	--	--	AAATAGGCGGTTGTGCATTT	59	ATGCCACATCATTTTCAGCA	60	267	--	--	--	--	--	--
TR13047|c0_g1_i1	XLOC_001336	(GCT)5	15	--	--	--	--	--	--	CATGGGCTGTTGCTGCTG	63	TTTCTCTTTGGCCAACAACC	60	103	--	--	XP_003245192.1	--	--	--
TR13050|c0_g2_i1	No	(CAA)5	15	--	--	--	--	--	--	ATTCCAGGTGTTGGAGTTGC	60	GCAAAGGACCCATCTGTTGT	60	229	--	--	--	--	--	--
TR13055|c0_g1_i1	XLOC_001346	(ATT)6aagttgaaaaacatgggtgaggtattaaggaga agagagaaggaagaggagaagttgagggaagaggaag agagtggaactgagagtaga(GGT)5	123	--	--	--	--	--	--	TCTGAGGTTTTGGTTTTTACGAA	60	CCACCTCATAAGCCCTTTCA	60	197	--	--	XM_004502703.1	ARR-B	[K]	K1213 0
TR13061|c0_g1_i1	No	(TCT)6	18	--	--	--	--	--	--	TTTGCAGCCATATCCAATCA	60	ATTCACTTCATCCGCACTCC	60	252	--	--	--	--	--	--

Transcript IDs	Differentially expressed gene (DEG) IDs	SSR repeat-motifs	SSR repeat-length (bp)	*In silico* polymorphic SSRs						Forward primers (5'-3')	Annealing tempearture(0C)	Reverse primers (5'-3')	Annealing tempearture(0C)	Amplified product size (bp)	Structural annotation		Functional Annotation			
				C. microphyllum		ICC4958														
				Transcript	SSR repeat-motifs	Chromosomes	SSR repeat-motifs	Start physical positions (bp)	End physical positions (bp)						Gene IDs	Sequence component of genes	NCBI nr IDs	TFs	KOG	KEGG
TR13061|c0_g2_i1	No	(TCT)6	18	--	--	--	--	--	--	TTTGCAGCCATATCCAATCA	60	ATTCACTTCATCCGCACTCC	60	252	--	--	--	--	--	--
TR13061|c0_g2_i2	No	(TCT)6	18	--	--	--	--	--	--	TTTGCAGCCATATCCAATCA	60	ATTCACTTCATCCGCACTCC	60	252	--	--	--	--	--	--
TR13061|c0_g2_i3	No	(TCT)6	18	--	--	--	--	--	--	TTTGCAGCCATATCCAATCA	60	ATTCACTTCATCCGCACTCC	60	252	--	--	--	--	--	--
TR13064|c0_g2_i1	XLOC_001355	(TA)7	14	--	--	--	--	--	--	TCTCAAGTTCTTTTGCCGGT	60	TGGGGTAGGGTGTGTGATTT	60	195	--	--	--	--	--	--
TR13085|c0_g1_i1	XLOC_001363	(AT)6	12	--	--	--	--	--	--	CGGGACAATGTGGATTACCT	60	CGCTGGACATTCCTATCACC	60	198	--	--	--	--	--	--
TR13101|c0_g1_i1	No	(TGG)5	15	--	--	--	--	--	--	GACGGAGACGAAGGAGTGTC	60	GCACCGTCTTGAAGAACCCT	62	176	--	--	--	--	--	--
TR13101|c0_g2_i1	No	(TGG)5	15	--	--	--	--	--	--	GACGGAGACGAAGGAGTGTC	60	GCACCGTCTTGAAGAACCCT	62	176	--	--	--	--	--	--
TR13125|c0_g1_i1	No	(TG)6	12	--	--	--	--	--	--	CATGGTAAACTGCAAAACATCTG	60	TGACTTTGGGAGAATGCAAA	59	116	--	--	--	--	--	--
TR13169|c0_g1_i1	No	(GA)6	12	--	--	--	--	--	--	CACCCACACTTTGATGAAGC	59	TGAAATGGGGCAAAGAAAAA	60	143	--	--	--	--	--	--
TR13169|c0_g2_i1	No	(GA)6	12	--	--	--	--	--	--	CGTTCTGTGTTTCTCTTTTGTCA	59	TTTGAAATGGGGCAAAGAAA	60	147	--	--	--	--	--	--
TR13190|c0_g1_i1	XLOC_001395	(ATA)6	18	--	--	--	--	--	--	AAAAAGCACGTTGGTGGAAT	59	CCATCATTTGTTGTGGTCGT	59	211	--	--	--	--	--	--
TR13279|c0_g1_i1	No	(TG)6	12	--	--	--	--	--	--	GATTTCACTATCTAAGGTTTGTTTGTT	58	ACCAAATGCCAAGATGCAA	60	139	--	--	--	--	--	--
TR13301|c0_g1_i1	No	(A)11tcatgtgcaattgcatcatgaaacaaaccgtgactacatgaacaggaaattttgcagacacgctaccatagcaaaccagttgtacatctaaaccaac(A)10	118	--	--	--	--	--	--	CACTGAATCATCGTAGAAAATGAAA	60	GCTATCGAACGGAAGCAATC	60	247	--	--	--	--	--	--
TR13302|c0_g1_i1	XLOC_001441	(CAG)5	15	--	--	--	--	--	--	TGAGACGACATTGCTTCCAG	60	ATTGGTACTCACAGCAGGCG	61	103	--	--	--	--	--	--

Transcript IDs	Differentially expressed gene (DEG) IDs	SSR repeat-motifs	SSR repeat-length (bp)	*In silico* polymorphic SSRs						Forward primers (5'-3')	Annealing tempearture(0C)	Reverse primers (5'-3')	Annealing tempearture(0C)	Amplified product size (bp)	Structural annotation		Functional Annotation			
				C. microphyllum		ICC4958									Gene IDs	Sequence component of genes	NCBI nr IDs	TFs	KOG	KEGG
				Transcript	SSR repeat-motifs	Chromosomes	SSR repeat-motifs	Start physical positions (bp)	End physical positions (bp)											
TR1338|c0_g1_i1	No	(AT)6	12	--	--	--	--	--	--	GCACGTGTCGGACATCAG	60	GGGGACCACATGGGTAAACT	61	113	--	--	--	--	--	--
TR13390|c0_g1_i1	XLOC_001455	(TAA)5	15	--	--	--	--	--	--	GCAATGACAATGGTAAAAATCAA	59	TGATAAAGCCCAATGTAAGCC	59	109	--	--	--	--	--	--
TR13537|c0_g1_i1	No	(CT)6	12	--	--	--	--	--	--	ATTTACAACCGAAAGGGGCT	60	ATTAACTTCCAATCGCACGC	60	214	--	--	--	--	--	--
TR1365|c0_g1_i1	No	(GCA)5	15	--	--	--	--	--	--	GATGCGTTGTTGTAGTGCAAA	60	TATGTTTGTCCGGCCTCTTC	60	148	--	--	--	--	--	--
TR13668|c0_g1_i1	No	(TGG)6	18	--	--	--	--	--	--	GTTGTACCGCTTCAGCACC	59	ACCACACGTACACACAACCC	59	229	--	--	--	--	--	--
TR13682|c0_g1_i1	No	(TG)6	12	--	--	--	--	--	--	TACCACCAAATTCGCATGAC	59	TGCCATAGGAAATAGAATAAATCA	57	107	--	--	--	--	--	--
TR13744|c0_g1_i1	No	(CAA)5	15	--	--	--	--	--	--	ACTCTTCATCATAAGCACCCA	57	GTAAACCCACCTAGAAGGAAAAG	58	219	--	--	--	--	--	--
TR13755|c0_g1_i1	XLOC_001550	(TA)7	14	--	--	--	--	--	--	CCAATCCTCCATTTCACACC	60	TTGATGTCATGAGAGAATGTTCAA	60	260	--	--	--	--	--	--
TR13773|c0_g2_i1	XLOC_001561	(TGG)5	15	--	--	--	--	--	--	TGGTCAGCTTGTTGAGCATC	60	GAGTTCTCCGACAGATTCGC	60	235	--	--	--	--	--	--
TR13780|c1_g1_i1	No	(TGA)5	15	--	--	--	--	--	--	CCATTGATTGCTGTTGCTTG	60	TCTCCAAAAGCTCCTTCCAA	60	240	--	--	--	--	--	--
TR13791|c0_g1_i1	No	(A)10caatctgtcacacaaacacacacaaaaaaacgtcaaaatctatgcaacattgtaacctctca(CT)7	86	--	--	--	--	--	--	TTGCACCCAATATCCAGACA	60	CACATGGAAATGCAAGATGG	60	232	--	--	--	--	--	--
TR13791|c0_g1_i2	No	(A)10caatctgtcacacaaacacacacaaaaaaacgtcaaaatctatgcaacattgtaacctctca(CT)7	86	--	--	--	--	--	--	TTGCACCCAATATCCAGACA	60	CACATGGAAATGCAAGATGG	60	232	--	--	--	--	--	--
TR13799|c0_g1_i1	XLOC_001580	(ACC)6	18	--	--	--	--	--	--	ATGGATCGTGCTCAGCTTCT	60	ATTCTTCATTGTTACCGCCG	60	276	--	--	--	--	--	--
TR13806|c0_g1_i1	XLOC_001587	(AGA)5	15	TR13806|c0_g1_i1	(AGA)5	Ca_LG_7	(AGA)7	41894793	41894813	CCGAAAGAAAACGATTCCAA	60	GGCTCACCTTCAAATCGAAA	60	208	Ca_18617	CDS	--	--	--	--

Transcript IDs	Differentially expressed gene (DEG) IDs	SSR repeat-motifs	SSR repeat-length (bp)	*In silico* polymorphic SSRs						Forward primers (5'-3')	Annealing tempearture(0C)	Reverse primers (5'-3')	Annealing tempearture(0C)	Amplified product size (bp)	Structural annotation		Functional Annotation			
				C. microphyllum		ICC4958									Gene IDs	Sequence component of genes	NCBI nr IDs	TFs	KOG	KEGG
				Transcript	SSR repeat-motifs	Chromosomes	SSR repeat-motifs	Start physical positions (bp)	End physical positions (bp)											
TR13808|c1_g2_i2	No	(TC)16	32	--	--	--	--	--	--	ATTTCAGATCAAACCCACCG	60	GAGCGTTGACTCCGCTAATC	60	255	--	--	--	--	--	--
TR13808|c2_g1_i1	XLOC_001590	(CT)9	18	--	--	--	--	--	--	CCTCACGCAACTCAACTCAA	60	CCGTCTCTCTCCGTCATGTT	60	245	--	--	--	--	--	--
TR13809|c1_g2_i1	XLOC_001592	(AT)7	14	--	--	--	--	--	--	AATTCGGACGCAATCAAATC	60	TTGGAGTAAGCTTGTTGGCA	59	139	--	--	--	--	--	--
TR13812|c0_g1_i1	XLOC_001596	(GA)6	12	--	--	--	--	--	--	AAGCAAAAACACAACAGCGA	60	CAAAGAGAAAAATGGGGCAA	60	250	--	--	--	--	--	--
TR13814|c1_g1_i1	XLOC_001600	(GAA)7	21	--	--	--	--	--	--	CGGCGTAGACTTGGAAGAAG	60	TTTCTCTGCGCATTCTCATT	59	223	--	--	--	--	--	--
TR13826|c0_g1_i1	XLOC_001610	(A)10caactaaataatgcagaagagttaag(A)10	46	--	--	--	--	--	--	CGGATGGAACAAAGAAGGAA	60	GGTCCTCAATGGTTGCAGAT	60	222	--	--	XP_004487298.1	ERF	[K]	--
TR13829|c0_g1_i1	XLOC_001612	(CT)6	12	--	--	--	--	--	--	CTCCACTCCAACCTGTGACC	61	TAAGCAGTGACCCAAAAGGG	60	210	--	--	--	--	--	--
TR13856|c0_g1_i1	No	(AATT)6	24	--	--	--	--	--	--	TATTGCTGTTGTTGCAAGGG	60	TATTGCTGTTGTTGCAAGGG	60	172	--	--	--	--	--	--
TR13856|c0_g2_i1	No	(AATT)6	24	--	--	--	--	--	--	TATTGCTGTTGTTGCAAGGG	60	GGCCATACCAAAGCACAAGT	60	188	--	--	--	--	--	--
TR13856|c1_g1_i1	No	(AATT)5	20	--	--	--	--	--	--	AAAAGCACAGGAGGACGAAA	60	CACCTTGCTAACTACGAGGAGG	60	100	--	--	--	--	--	--
TR13867|c0_g1_i1	XLOC_001650	(CT)7	14	--	--	--	--	--	--	GCAAAAAGCCATCATCCATT	60	AAGCTGAGTAGACGCGAAGC	60	261	--	--	--	--	--	--
TR13869|c0_g1_i1	XLOC_001652	(TC)12	24	TR13869|c0_g1_i1	(TC)12	Ca_LG_4	(TC)7	13785757	13785770	TTTGTGGTGAGCTTCTTCTTCA	60	TCTGATGCTTGTCGGTTCTG	60	102	Ca_03393	DRR	--	--	--	--
TR13967|c0_g1_i1	XLOC_001681	(TTC)5	15	--	--	--	--	--	--	GAAGTGACGACCTTAAGCGG	60	GCACTCGGAGAAGACCGTAG	60	109	--	--	XP_008912890.1	--	[R]	--
TR13982|c0_g1_i1	XLOC_001692	(AGAA)6	24	--	--	--	--	--	--	TTTGTGTAGCCACCATCCAA	60	GTTTTGAATTCCAGGGCAAG	60	212	--	--	--	--	--	--

Transcript IDs	Differentially expressed gene (DEG) IDs	SSR repeat-motifs	SSR repeat-length (bp)	*In silico* polymorphic SSRs						Forward primers (5'-3')	Annealing tempearture(0C)	Reverse primers (5'-3')	Annealing tempearture(0C)	Amplified product size (bp)	Structural annotation		Functional Annotation			
				C. microphyllum		ICC4958									Gene IDs	Sequence component of genes	NCBI nr IDs	TFs	KOG	KEGG
				Transcript	SSR repeat-motifs	Chromosomes	SSR repeat-motifs	Start physical positions (bp)	End physical positions (bp)											
TR13982|c0_g2_i1	No	(AGAA)6	24	--	--	--	--	--	--	TTTGTGTAGCCACCATCCAA	60	GTTTTGAATTCCAGGGCAAG	60	213	--	--	--	--	--	--
TR14142|c0_g1_i1	XLOC_001731	(AAG)5	15	--	--	--	--	--	--	GTTTGACCCAACATCCATCC	60	TGCCAAGAATAGAACCCCAG	60	249	--	--	--	--	--	--
TR14162|c0_g1_i1	XLOC_001737	(ATT)5	15	--	--	--	--	--	--	ACCGGCTGAAAAGGAAATCT	60	TCGTGAATTTCGTGGTGCTA	60	279	--	--	--	--	--	--
TR14216|c0_g1_i1	No	(TA)9	18	--	--	--	--	--	--	GGAATTTTTATGACGAAAATGGA	59	CAAATTCCTTAAACCACAGCAA	59	246	--	--	--	--	--	--
TR14339|c0_g1_i1	No	(ACA)6	18	--	--	--	--	--	--	CCACAAGCAATCAAATCTGG	59	GTTGGCTGCTGATGAAGGAT	60	207	--	--	--	--	--	--
TR14373|c0_g1_i1	XLOC_001769	(ATA)11	33	--	--	--	--	--	--	GGTTTGGTTGGGAAAGTGAA	60	ATTTGTGCCACCAACTGCTT	61	243	--	--	--	--	--	--
TR14400|c0_g1_i1	No	(CT)6	12	--	--	--	--	--	--	TCCGTCCTCAATCAAGTTCC	60	TGAATCGGTCACCTCTTTGA	59	187	--	--	--	--	--	--
TR14412|c0_g1_i1	No	(ACA)5	15	--	--	--	--	--	--	CCAAGTGTTGCAGGTTCTTG	59	AACGGTGTCGTTTCGTTCTC	60	196	--	--	--	--	--	--
TR14413|c0_g1_i1	No	(AT)6	12	--	--	--	--	--	--	TTATTTCGAGCAACTAGGGC	57	GCGGACTGTAATTTCCAACA	59	167	--	--	--	--	--	--
TR14538|c0_g1_i1	XLOC_001814	(AAG)6	18	--	--	--	--	--	--	CTCGAGTTCGAAACGAAAGG	60	GTCCTTCAAGGTCGGACAAC	60	206	--	--	EUC66603.1	--	--	--
TR14584|c0_g1_i1	XLOC_001837	(TCC)5	15	--	--	--	--	--	--	AAGAGCTCCGGTCAAGCAT	60	TGTTGGGAGGTTTCCTTTTG	60	264	--	--	--	--	--	--
TR14692|c0_g1_i1	No	(AG)6	12	--	--	--	--	--	--	GCTTGTCTGTGATTGCTTGC	60	GGATCTTTCCACGAGCCATA	60	146	--	--	--	--	--	--
TR1480|c0_g1_i1	No	(TCA)6	18	--	--	--	--	--	--	ATACTTGGCTGGGATCGTTG	60	GGAGCATCAGAGGACGAAGA	60	168	--	--	--	--	--	--
TR14800|c0_g1_i1	No	(CAA)6	18	--	--	--	--	--	--	AACGCCAACAACATCAACAA	60	CGGAATAACCATCTTCACGG	60	143	--	--	--	--	--	--

Transcript IDs	Differentially expressed gene (DEG) IDs	SSR repeat-motifs	SSR repeat-length (bp)	*In silico* polymorphic SSRs						Forward primers (5'-3')	Annealing tempearture(0C)	Reverse primers (5'-3')	Annealing tempearture(0C)	Amplified product size (bp)	Structural annotation		Functional Annotation			
				C. microphyllum		ICC4958														
				Transcript	SSR repeat-motifs	Chromosomes	SSR repeat-motifs	Start physical positions (bp)	End physical positions (bp)						Gene IDs	Sequence component of genes	NCBI nr IDs	TFs	KOG	KEGG
TR14828|c0_g1_i1	No	(CAG)5	15	--	--	--	--	--	--	ATGCTCATCGAGCTGCCT	60	GGCATGAACCTTGGAACACT	60	106	--	--	--	--	--	--
TR14856|c0_g1_i1	No	(TA)6	12	--	--	--	--	--	--	TGAAGGACGACAACAAATGG	60	GTGAACATCGATCCCAAGGT	60	238	--	--	--	--	--	--
TR14869|c0_g1_i1	No	(TC)7	14	--	--	--	--	--	--	ATCTCTAACCCGTGGGCTTT	60	ATCCTCCAGACACCTCCTTG	59	156	--	--	--	--	--	--
TR14890|c0_g1_i1	XLOC_001915	(CACT)5	20	--	--	--	--	--	--	GGCTTTGTGCTCCTCAAAAA	60	GTGCCGTTTGATTTCCAGTT	60	255	--	--	--	--	--	--
TR15155|c0_g1_i1	XLOC_001965	(AG)7	14	TR15155|c0_g1_i1	(AG)7	Ca_LG_8	(AG)12	9110147	9110170	AAAAGGGAATGAAAAGGAGGA	59	AAGAATCGACGAATGCCATC	60	170	Ca_19925	CDS	--	--	--	--
TR15262|c0_g1_i1	No	(GA)8	16	--	--	--	--	--	--	ACACACACTGGGAGAGGGAG	60	CACTCCCATTTTTGCCTTTC	60	100	--	--	--	--	--	--
TR15290|c0_g1_i1	XLOC_002002	(TA)6	12	--	--	--	--	--	--	TTGGAAAAATACCGGCAAAG	60	GCCTGCTCTTGTACCTCCTG	60	250	--	--	NP_001155556.1	WOX	[U]	K07936
TR15313|c0_g1_i1	No	(TTG)5	15	--	--	--	--	--	--	GCGCTGTTGTCGTCGTAGTA	60	GGTGGACAGCAGTACCATCAA	61	155	--	--	--	--	--	--
TR15314|c0_g1_i1	XLOC_002015	(TC)6	12	--	--	--	--	--	--	TTGGCTCGTCTCTCCAACTT	60	ACGACAGCGAGTCAGATCCT	60	264	--	--	--	--	--	--
TR15325|c0_g1_i1	XLOC_002021	(AAC)7	21	--	--	--	--	--	--	GTTGGTGGGAGAAACTGGTG	60	AAGAAAAAGGCCCCGTTAGA	60	179	--	--	--	--	--	--
TR1536|c0_g1_i1	No	(GTT)5	15	--	--	--	--	--	--	AAATGGTGATGGTGGTGGTT	60	CGAATGCCGAATAGCAAAAT	60	154	--	--	--	--	--	--
TR15395|c0_g1_i1	No	(AAC)5	15	--	--	--	--	--	--	AGCGAAAAGGAGAACGAACA	60	TTGTTGGAAAGGAAAGTGTGG	60	177	--	--	--	--	--	--
TR15417|c0_g1_i1	No	(TCTCA)5	25	--	--	--	--	--	--	GTGTCCCTCCCTCTTTTTCC	60	GCAATGAACGAATGCAACAC	60	167	--	--	--	--	--	--
TR15429|c0_g1_i1	No	(TAT)5	15	--	--	--	--	--	--	ACCAGGACGAAAATCGACTG	60	CATACATCACGATCCCCCAT	60	138	--	--	--	--	--	--

Transcript IDs	Differentially expressed gene (DEG) IDs	SSR repeat-motifs	SSR repeat-length (bp)	*In silico* polymorphic SSRs						Forward primers (5'-3')	Annealing tempearture(0C)	Reverse primers (5'-3')	Annealing tempearture(0C)	Amplified product size (bp)	Structural annotation		Functional Annotation			
				C. microphyllum		ICC4958									Gene IDs	Sequence component of genes	NCBI nr IDs	TFs	KOG	KEGG
				Transcript	SSR repeat-motifs	Chromosomes	SSR repeat-motifs	Start physical positions (bp)	End physical positions (bp)											
TR15467|c2_g1_i1	No	(TC)15	30	--	--	--	--	--	--	GGATCGATGAAAAGGGTTGA	60	CTTTTCCAATCCCTTCACCA	60	252	--	--	--	--	--	--
TR15469|c0_g1_i1	No	(TA)14	28	--	--	--	--	--	--	CCATCAATCTGGCTGCTACA	60	TGTGGAACTCCATACCCAAAG	60	253	--	--	--	--	--	--
TR15469|c1_g2_i2	XLOC_002072	(TTC)5	15	--	--	--	--	--	--	CAGTTCAAGTTATTATTTTCTTGATGA	57	AAGGTTGGAGCAGAAGATGC	59	101	--	--	--	--	--	--
TR15477|c0_g2_i1	XLOC_002081	(TA)11tcacacaaaacacatacagtttacctttattttaagaat(A)11	72	--	--	--	--	--	--	AACTGGTCCATCACAAACTGC	60	TTGATGGCATTGATGTTTGC	60	243	--	--	--	--	--	--
TR15477|c0_g2_i2	No	(TA)11tcacacaaaacacatacagtttacctttattttaagaat(A)11	72	--	--	--	--	--	--	AACTGGTCCATCACAAACTGC	60	TTGATGGCATTGATGTTTGC	60	243	--	--	--	--	--	--
TR15477|c1_g2_i3	No	(CTT)5	15	--	--	--	--	--	--	TCGAGTGTAGTTTATAGGGTTCAA	57	CTTGACGGGCTTTATTGGAG	60	212	--	--	--	--	--	--
TR15483|c2_g1_i1	No	(AAT)5	15	TR15483|c2_g1_i1	(AAT)5	Ca_LG_4	(AAT)6	6648370	6648387	AAGCACACGCTGGCAATAG	60	GCTCGAGTAAAGTCGAGGCT	59	244		URR	--	--	--	--
TR15483|c2_g1_i2	No	(AAT)5	15	--	--	--	--	--	--	AAGCACACGCTGGCAATAG	60	GCTCGAGTAAAGTCGAGGCT	59	244	--	--	--	--	--	--
TR15483|c3_g2_i1	No	(TAT)6	18	--	--	--	--	--	--	CACGTCGTCAACTCCAGAGA	60	CCATAGCCGCGTGGTATAGTA	60	224	--	--	--	--	--	--
TR15483|c3_g2_i11	No	(TAT)6	18	--	--	--	--	--	--	CACGTCGTCAACTCCAGAGA	60	CCATAGCCGCGTGGTATAGTA	60	224	--	--	--	--	--	--
TR15483|c3_g2_i5	No	(TAT)6	18	--	--	--	--	--	--	CACGTCGTCAACTCCAGAGA	60	CCATAGCCGCGTGGTATAGTA	60	224	--	--	--	--	--	--
TR15483|c3_g2_i6	No	(TAT)6	18	--	--	--	--	--	--	CACGTCGTCAACTCCAGAGA	60	CCATAGCCGCGTGGTATAGTA	60	224	--	--	--	--	--	--
TR15483|c3_g2_i8	No	(TAT)6	18	--	--	--	--	--	--	CACGTCGTCAACTCCAGAGA	60	CCATAGCCGCGTGGTATAGTA	60	224	--	--	--	--	--	--
TR15483|c3_g2_i9	No	(TAT)6	18	--	--	--	--	--	--	CACGTCGTCAACTCCAGAGA	60	CCATAGCCGCGTGGTATAGTA	60	224	--	--	--	--	--	--

Transcript IDs	Differentially expressed gene (DEG) IDs	SSR repeat-motifs	SSR repeat-length (bp)	*In silico* polymorphic SSRs						Forward primers (5'-3')	Annealing tempearture(0C)	Reverse primers (5'-3')	Annealing tempearture(0C)	Amplified product size (bp)	Structural annotation		Functional Annotation			
				C. microphyllum		ICC4958														
				Transcript	SSR repeat-motifs	Chromosomes	SSR repeat-motifs	Start physical positions (bp)	End physical positions (bp)						Gene IDs	Sequence component of genes	NCBI nr IDs	TFs	KOG	KEGG
TR15499|c3_g1_i1	XLOC_002121	(GGT)5	15	--	--	--	--	--	--	CTGCTGCTGTTGTTGTGGTT	60	TGAATCTCCTCCAAACTCCG	60	117	--	--	--	--	--	--
TR15500|c0_g1_i3	No	(A)10catttttttgcatgagtatagaccaaatataatattaacatcctagctctgtcagc(T)12	78	--	--	--	--	--	--	GGCTAAAAATCAATTCTTGAGGT	58	AAATTGCTTCCACATTTGCAT	59	187	--	--	--	--	--	--
TR15503|c0_g1_i1	No	(T)11ctttaaacctttcattttcaccttcccagttagagagacaaaataaagcttgtaatataatttttctttc(T)11	92	--	--	--	--	--	--	TGATTTTGTCTTAACAACCCAGAA	60	GCTTAATCCAGAAGCTCTCAGC	60	243	--	--	--	--	--	--
TR15503|c0_g1_i2	No	(T)11ctttaaacctttcattttcaccttcccagttagagagacaaaataaagcttgtaatataatttttctttc(T)11	92	--	--	--	--	--	--	TGATTTTGTCTTAACAACCCAGAA	60	GCTTAATCCAGAAGCTCTCAGC	60	243	--	--	--	--	--	--
TR15509|c0_g1_i1	XLOC_002134	(CT)7	14	--	--	--	--	--	--	CATGGCTGTCACCAATGAAC	60	GGTTTGGTGTTTGGGAAATG	60	201	--	--	--	--	--	--
TR15510|c2_g2_i2	No	(CT)15	30	--	--	--	--	--	--	GCCAGACAAAATCTTTCCTCTCT	60	GACTTGCAGAGACATGCTGG	60	105	--	--	--	--	--	--
TR15510|c2_g2_i4	No	(CT)15	30	--	--	--	--	--	--	GCCAGACAAAATCTTTCCTCTCT	60	GACTTGCAGAGACATGCTGG	60	105	--	--	--	--	--	--
TR15512|c0_g1_i1	XLOC_002138	(TC)8(T)11	27	--	--	--	--	--	--	TCAAGCCTATCTATATTTTGCACAC	59	AGGTCTTGAGGCGAATTCCT	60	252	--	--	--	--	--	--
TR15523|c2_g1_i1	XLOC_002160	(CCT)5	15	--	--	--	--	--	--	GAAATAGTCAAAACTTTGCACCC	59	CTCGCTCACTCATTCACCAA	60	269	--	--	--	--	--	--
TR15523|c2_g2_i1	XLOC_002161	(TTC)6	18	--	--	--	--	--	--	TGACTCAGACGGAGGTTGTG	60	GGTGATTTCAAGAGCCGAAG	60	257	--	--	--	--	--	--
TR15525|c0_g1_i1	XLOC_002171	(TC)7	14	--	--	--	--	--	--	CCTCAACGCTCACAGATTACC	60	GGCAGAGATTCCTGGAACAG	60	116	--	--	XP_004486330.1	GRAS	[TK]	--
TR15530|c0_g1_i1	XLOC_002177	(TAA)5(ATT)5	30	--	--	--	--	--	--	TGCAACAACACCCACTTTGT	60	CCTCAGACCCACATCCTGTT	60	217	--	--	--	--	--	--
TR15538|c2_g1_i1	XLOC_002188	(A)10gtc(T)10	23	--	--	--	--	--	--	TTAACGACGCATTGTTTTGC	60	ACAACTGGGGTTCCTCTGTG	60	244	--	--	XM_004501439.1	--	[G]	--
TR15538|c2_g2_i1	No	(A)10gtc(T)10	23	--	--	--	--	--	--	TTAACGACGCATTGTTTTGC	60	ACAACTGGGGTTCCTCTGTG	60	244	--	--	--	--	--	--

Transcript IDs	Differentially expressed gene (DEG) IDs	SSR repeat-motifs	SSR repeat-length (bp)	*In silico* polymorphic SSRs						Forward primers (5'-3')	Annealing tempearture(0C)	Reverse primers (5'-3')	Annealing tempearture(0C)	Amplified product size (bp)	Structural annotation		Functional Annotation			
				C. microphyllum		ICC4958									Gene IDs	Sequence component of genes	NCBI nr IDs	TFs	KOG	KEGG
				Transcript	SSR repeat-motifs	Chromosomes	SSR repeat-motifs	Start physical positions (bp)	End physical positions (bp)											
TR1554|c1_g1_i1	No	(CTG)5	15	--	--	--	--	--	--	GTGGACACGTAGTAGCCCGT	60	CATTTCGACGACGACGATTA	60	210	--	--	--	--	--	--
TR15542|c0_g1_i1	XLOC_002196	(AG)9	18	--	--	--	--	--	--	GCCTCCTTCGTCTTTCTCCT	60	TTGGCCATCTTTAACCTTCG	60	146	--	--	--	--	--	--
TR15542|c0_g2_i1	No	(AG)9	18	--	--	--	--	--	--	GCCTCCTTCGTCTTTCTCCT	60	TTGGCCATCTTTAACCTTCG	60	146	--	--	--	--	--	--
TR15544|c1_g1_i1	No	(AC)6	12	--	--	--	--	--	--	TTGCTTACTTCTCTCAAAGCTCAA	60	GAGAGCATGTGGTTATGTTGGA	60	108	--	--	--	--	--	--
TR15544|c1_g1_i5	No	(AC)6	12	--	--	--	--	--	--	TTGCTTACTTCTCTCAAAGCTCAA	60	GAGAGCATGTGGTTATGTTGGA	60	108	--	--	--	--	--	--
TR15545|c0_g1_i2	No	(TC)6	12	--	--	--	--	--	--	ACTGGCGGTATCTTCCTCCT	60	TCCATGGTTGATGATGATGC	60	267	--	--	--	--	--	--
TR15545|c0_g2_i1	No	(TC)6	12	--	--	--	--	--	--	ACTGGCGGTATCTTCCTCCT	60	TCCATGGTTGATGATGATGC	60	267	--	--	--	--	--	--
TR15545|c0_g3_i1	XLOC_002202	(TC)6	12	--	--	--	--	--	--	ACTGGCGGTATCTTCCTCCT	60	TCCATGGTTGATGATGATGC	60	267	--	--	--	--	--	--
TR15546|c0_g1_i1	XLOC_002203	(CT)11	22	--	--	--	--	--	--	CTCCATGTTATTTCCATGCG	59	TCTGTGCTGGGAATTTAGCA	59	185	--	--	--	--	--	--
TR15594|c0_g1_i1	No	(GA)6	12	--	--	--	--	--	--	CAGCTCCACCAAAGGAAGAG	60	TAGCCATAGCCACAACCACA	60	273	--	--	--	--	--	--
TR15594|c0_g1_i1	No	(CTT)6	18	--	--	--	--	--	--	CCACTGTGCCAGAGAAGTCA	60	CTCTTCCTTTGGTGGAGCTG	60	215	--	--	--	--	--	--
TR15641|c1_g1_i1	XLOC_002286	(ATC)8	24	--	--	--	--	--	--	AAAAGTGCTGTTTTGCGCTT	60	CAGAGGATGCTGACCCTGAT	60	280	--	--	--	--	--	--
TR15641|c4_g1_i1	XLOC_002288	(TC)14	28	--	--	--	--	--	--	CCATCCAAACCAACCTTCAG	60	CTGAACTCTGAACTTGGGCG	61	101	--	--	--	--	--	--
TR15641|c4_g2_i1	No	(ATA)5	15	--	--	--	--	--	--	GAACTCCAAAACACCCCTCA	60	AATGGACGGGGAGGTTTATC	60	197	--	--	--	--	--	--

Transcript IDs	Differentially expressed gene (DEG) IDs	SSR repeat-motifs	SSR repeat-length (bp)	*In silico* polymorphic SSRs						Forward primers (5'-3')	Annealing tempearture(0C)	Reverse primers (5'-3')	Annealing tempearture(0C)	Amplified product size (bp)	Structural annotation		Functional Annotation			
				C. microphyllum		ICC4958														
				Transcript	SSR repeat-motifs	Chromosomes	SSR repeat-motifs	Start physical positions (bp)	End physical positions (bp)						Gene IDs	Sequence component of genes	NCBI nr IDs	TFs	KOG	KEGG
TR15641|c4_g3_i1	XLOC_002289	(ATA)5	15	--	--	--	--	--	--	GAACTCCAAAACACCCCTCA	60	AATGGACGGGGAGGTTTATC	60	197	--	--	--	--	--	--
TR15661|c0_g1_i1	No	(T)10aggttttgatttcaaatattctttgctcttctttaaagttaaaatatataatttat(A)10	76	--	--	--	--	--	--	CCAAGTCCATCCATCTCCAT	60	GGGCTCACACTCATTGGTCT	60	204	--	--	--	--	--	--
TR15661|c0_g2_i1	No	(T)10aggttttgatttcaaatattctttgctcttctttaaagttaaaatatataatttat(A)10	76	--	--	--	--	--	--	CCAAGTCCATCCATCTCCAT	60	GGGCTCACACTCATTGGTCT	60	204	--	--	--	--	--	--
TR15663|c1_g2_i1	No	(AAT)5	15	--	--	--	--	--	--	AATCAAAAGGCAACCAATGC	60	AGCTTATTGGAGTTGGCAGC	59	208	--	--	--	--	--	--
TR15663|c1_g2_i2	No	(AAT)5	15	--	--	--	--	--	--	AATCAAAAGGCAACCAATGC	60	AGCTTATTGGAGTTGGCAGC	59	208	--	--	--	--	--	--
TR15663|c1_g2_i3	No	(AAT)5	15	--	--	--	--	--	--	AATCAAAAGGCAACCAATGC	60	AGCTTATTGGAGTTGGCAGC	59	208	--	--	--	--	--	--
TR15673|c3_g1_i1	No	(AT)6	12	--	--	--	--	--	--	CTGCGCCTACACGACTAACA	60	TTTTTGTGCTTCCCCAGTTT	60	193	--	--	--	--	--	--
TR15673|c3_g2_i1	No	(AT)6	12	--	--	--	--	--	--	CTGCGCCTACACGACTAACA	60	TTTTTGTGCTTCCCCAGTTT	60	193	--	--	--	--	--	--
TR15673|c3_g3_i1	No	(AT)6	12	--	--	--	--	--	--	CTGCGCCTACACGACTAACA	60	TTTTTGTGCTTCCCCAGTTT	60	193	--	--	--	--	--	--
TR15674|c0_g1_i3	No	(TCT)6ccctcgacccttcaccatcacaacttcagcaatattttctttctccaaatcataaacccattaacaacgtttcttctcttctcaacaat(AAC)5	122	--	--	--	--	--	--	AATCCCTATTTTCGTCCATCA	58	TTTGAGGTAAATGGGGCAAG	60	257	--	--	--	--	--	--
TR15674|c0_g2_i1	XLOC_002343	(TC)8	16	TR15674|c0_g2_i1	(TC)8	Ca_LG_3	(TC)24	29478098	29478145	CATGCAGATTGCAGACACACT	60	TGAACTCCAGGTGGTAAGGG	60	251		URR	--	--	--	--
TR15677|c0_g1_i1	No	(AT)10	20	--	--	--	--	--	--	AACCCTATCCAGCAAGCAAA	60	AACCCTATCCAGCAAGCAAA	60	238	--	--	--	--	--	--
TR15680|c7_g1_i1	XLOC_002373	(TAC)5	15	--	--	--	--	--	--	GCCACTGTTGAAAAAGCCAT	60	TTGTTCTAATTCATGCACATCG	59	138	--	--	XP_004502599.1	--	[X]	--
TR15680|c7_g1_i1	XLOC_002373	(CAA)7ccaccaccacgtcaaccaataataatatacaccgtatcccctaaggtaatccacaccacacccggtgacttcatgaacctcgttcaacgtctaaccggc(TCA)6	138	--	--	--	--	--	--	GGAAGATCGCCAAGAAGAGA	60	GATATGGTTCCGTCACCGTT	60	275	--	--	XP_004502599.1	--	[X]	--

Transcript IDs	Differentially expressed gene (DEG) IDs	SSR repeat-motifs	SSR repeat-length (bp)	*In silico* polymorphic SSRs						Forward primers (5'-3')	Annealing tempearture(0C)	Reverse primers (5'-3')	Annealing tempearture(0C)	Amplified product size (bp)	Structural annotation		Functional Annotation			
				C. microphyllum		ICC4958									Gene IDs	Sequence component of genes	NCBI nr IDs	TFs	KOG	KEGG
				Transcript	SSR repeat-motifs	Chromosomes	SSR repeat-motifs	Start physical positions (bp)	End physical positions (bp)											
TR15710|c2_g1_i1	No	(CTT)7	21	--	--	--	--	--	--	ACATTCCCATTCCCATTGAA	60	ATTTGGAATCCATGGTGCTC	60	224	--	--	--	--	--	--
TR15710|c2_g1_i1	No	(CCA)5	15	--	--	--	--	--	--	GAGCACCATGGATTCCAAAT	60	TTACCGAGGGTTTGTTCGAG	60	225	--	--	--	--	--	--
TR15710|c2_g1_i2	No	(CTT)7	21	--	--	--	--	--	--	ACATTCCCATTCCCATTGAA	60	ATTTGGAATCCATGGTGCTC	60	224	--	--	--	--	--	--
TR15710|c2_g1_i2	No	(CCA)5	15	--	--	--	--	--	--	GAGCACCATGGATTCCAAAT	60	TTACCGAGGGTTTGTTCGAG	60	225	--	--	--	--	--	--
TR15710|c5_g1_i1	XLOC_002444	(TG)6	12	--	--	--	--	--	--	CGAAAGTAAAGGCGATGGAA	60	CGATGAGAGAGAAAGCGAAA	58	100	--	--	--	--	--	--
TR15712|c1_g2_i3	No	(GA)10	20	--	--	--	--	--	--	TTAACCAAGTTTTGGAGTCCG	59	CAGTTGACCAAAACTCAAAAGC	59	246	--	--	--	--	--	--
TR15712|c1_g2_i4	No	(GA)10	20	--	--	--	--	--	--	TTAACCAAGTTTTGGAGTCCG	59	CAGTTGACCAAAACTCAAAAGC	59	246	--	--	--	--	--	--
TR15712|c1_g2_i5	No	(GA)10	20	--	--	--	--	--	--	TTAACCAAGTTTTGGAGTCCG	59	CAGTTGACCAAAACTCAAAAGC	59	246	--	--	--	--	--	--
TR15712|c3_g1_i1	No	(ATT)7	21	--	--	--	--	--	--	GTGCAGTGAATGCGAAATTG	60	GGCTTCTGGCAAATCTTTCA	60	191	--	--	--	--	--	--
TR15722|c0_g1_i1	XLOC_002461	(CTT)5	15	TR15722|c0_g1_i1	(GAA)5	Ca_LG_4	(GAA)6	11460992	11461009	ATAATAACCCAGGTGCTGCG	60	TGCTGGAACGAACATTCAAC	60	272	Ca_00397	URR	--	--	--	--
TR15722|c0_g2_i1	XLOC_002462	(CTT)5	15	--	--	--	--	--	--	ATAATAACCCAGGTGCTGCG	60	TGCTGGAACGAACATTCAAC	60	272	--	--	XM_004494996.1	--	[Q]	--
TR15774|c0_g1_i1	No	(CT)6	12	--	--	--	--	--	--	TCTTTGAAGCAATTCTTGTCCA	60	AAAGGGACGGAGGAGTGAAG	61	159	--	--	--	--	--	--
TR15811|c0_g1_i1	No	(TAA)7	21	--	--	--	--	--	--	GCATTTCACAAATGTTTCGG	59	GGTTGGTTCTTCCTGGTGAA	60	177	--	--	--	--	--	--
TR15811|c0_g2_i1	No	(TAA)7	21	--	--	--	--	--	--	GCATTTCACAAATGTTTCGG	59	GGTTGGTTCTTCCTGGTGAA	60	177	--	--	--	--	--	--

<table>
<tr><th rowspan="3">Transcript IDs</th><th rowspan="3">Differentially expressed gene (DEG) IDs</th><th rowspan="3">SSR repeat-motifs</th><th rowspan="3">SSR repeat-length (bp)</th><th colspan="6">In silico polymorphic SSRs</th><th rowspan="3">Forward primers (5'-3')</th><th rowspan="3">Annealing tempearture(0C)</th><th rowspan="3">Reverse primers (5'-3')</th><th rowspan="3">Annealing tempearture(0C)</th><th rowspan="3">Amplified product size (bp)</th><th colspan="2">Structural annotation</th><th colspan="4">Functional Annotation</th></tr>
<tr><th colspan="2">C. microphyllum</th><th colspan="4">ICC4958</th><th rowspan="2">Gene IDs</th><th rowspan="2">Sequence component of genes</th><th rowspan="2">NCBI nr IDs</th><th rowspan="2">TFs</th><th rowspan="2">KOG</th><th rowspan="2">KEGG</th></tr>
<tr><th>Transcript</th><th>SSR repeat-motifs</th><th>Chromosomes</th><th>SSR repeat-motifs</th><th>Start physical positions (bp)</th><th>End physical positions (bp)</th></tr>
<tr><td>TR15854|c0_g1_i1</td><td>XLOC_002521</td><td>(GAA)10</td><td>30</td><td>--</td><td>--</td><td>--</td><td>--</td><td>--</td><td>--</td><td>GCAGAGTGCAATTGAGATCC</td><td>58</td><td>TTCACCTTCAAGGGTTCGTC</td><td>60</td><td>158</td><td>--</td><td>--</td><td>--</td><td>--</td><td>--</td><td>--</td></tr>
<tr><td>TR15876|c0_g1_i1</td><td>XLOC_002534</td><td>(CT)6</td><td>12</td><td>--</td><td>--</td><td>--</td><td>--</td><td>--</td><td>--</td><td>GCCGCTGAATGTTGTCTTTT</td><td>60</td><td>GAGGAGGGGTAGGGTTTGAG</td><td>60</td><td>235</td><td>--</td><td>--</td><td>--</td><td>--</td><td>--</td><td>--</td></tr>
<tr><td>TR15878|c0_g1_i1</td><td>XLOC_002535</td><td>(TGG)7</td><td>21</td><td>--</td><td>--</td><td>--</td><td>--</td><td>--</td><td>--</td><td>TCTCTGCTTTCATGTCACCG</td><td>60</td><td>AAGCAACTCAAGGGAAAGCA</td><td>60</td><td>103</td><td>--</td><td>--</td><td>--</td><td>--</td><td>--</td><td>--</td></tr>
<tr><td>TR15878|c1_g1_i1</td><td>XLOC_002536</td><td>(CAC)5</td><td>15</td><td>--</td><td>--</td><td>--</td><td>--</td><td>--</td><td>--</td><td>CTCAGGGGTAATATCCACCG</td><td>59</td><td>GTAGTGGTGGAGGAGCTGGT</td><td>59</td><td>165</td><td>--</td><td>--</td><td>--</td><td>--</td><td>--</td><td>--</td></tr>
<tr><td>TR1588|c0_g1_i1</td><td>XLOC_002541</td><td>(GA)6tggtttttt(AG)9</td><td>39</td><td>--</td><td>--</td><td>--</td><td>--</td><td>--</td><td>--</td><td>TTTGACCTCCTTGGCTCTGT</td><td>60</td><td>GAAGAAATTAGGATGGAGGGG</td><td>59</td><td>121</td><td>--</td><td>--</td><td>--</td><td>--</td><td>--</td><td>--</td></tr>
<tr><td>TR1595|c0_g1_i1</td><td>No</td><td>(TGG)5</td><td>15</td><td>--</td><td>--</td><td>--</td><td>--</td><td>--</td><td>--</td><td>AGAATCGGAATTCCCCAGAT</td><td>60</td><td>CAAGGAGCCTGAGAAACCAG</td><td>60</td><td>264</td><td>--</td><td>--</td><td>--</td><td>--</td><td>--</td><td>--</td></tr>
<tr><td>TR15951|c0_g1_i1</td><td>No</td><td>(AT)8</td><td>16</td><td>--</td><td>--</td><td>--</td><td>--</td><td>--</td><td>--</td><td>CAGCCATGGGTTCCACTAAC</td><td>60</td><td>GGGAGAGCATATGAGAAGAGTTTT</td><td>59</td><td>209</td><td>--</td><td>--</td><td>--</td><td>--</td><td>--</td><td>--</td></tr>
<tr><td>TR16017|c0_g1_i1</td><td>XLOC_002573</td><td>(CA)6</td><td>12</td><td>--</td><td>--</td><td>--</td><td>--</td><td>--</td><td>--</td><td>CGCCTCTTTCTGCTTCTCAC</td><td>60</td><td>TCGGTGAGTTGAAGCCTCTT</td><td>60</td><td>217</td><td>--</td><td>--</td><td>--</td><td>--</td><td>--</td><td>--</td></tr>
<tr><td>TR16022|c0_g1_i1</td><td>No</td><td>(CAT)5cacactgatcatcctcaccttcgccatccaggatcaattggttcactgggctcctctggtgtaggaggtggc(GGT)5</td><td>102</td><td>--</td><td>--</td><td>--</td><td>--</td><td>--</td><td>--</td><td>ATCTGATCTTTCGGGACACG</td><td>60</td><td>CGATCCATCACCAGCTGAAC</td><td>62</td><td>169</td><td>--</td><td>--</td><td>--</td><td>--</td><td>--</td><td>--</td></tr>
<tr><td>TR16108|c0_g1_i1</td><td>XLOC_002609</td><td>(ATC)5</td><td>15</td><td>--</td><td>--</td><td>--</td><td>--</td><td>--</td><td>--</td><td>GGAAGAACGTTGACCCAAAA</td><td>60</td><td>AAGCTGCTTCGCTGTCTCTC</td><td>60</td><td>211</td><td>--</td><td>--</td><td>--</td><td>--</td><td>--</td><td>--</td></tr>
<tr><td>TR16148|c0_g1_i1</td><td>XLOC_002628</td><td>(CT)6</td><td>12</td><td>--</td><td>--</td><td>--</td><td>--</td><td>--</td><td>--</td><td>GAACAAAGGGGGAAACTCTG</td><td>59</td><td>TTGGCCATCCATGTTAGTCA</td><td>60</td><td>248</td><td>--</td><td>--</td><td>XM_004511789.1</td><td>C2H2</td><td>[K]</td><td>--</td></tr>
<tr><td>TR16171|c0_g1_i1</td><td>No</td><td>(AC)7</td><td>14</td><td>--</td><td>--</td><td>--</td><td>--</td><td>--</td><td>--</td><td>TCAGCAACAATGGGAATCAA</td><td>60</td><td>CCAAGTCTGTCAACTGTTGGG</td><td>61</td><td>231</td><td>--</td><td>--</td><td>--</td><td>--</td><td>--</td><td>--</td></tr>
<tr><td>TR16182|c1_g1_i1</td><td>XLOC_002644</td><td>(TAA)6</td><td>18</td><td>TR16182|c1_g1_i1</td><td>(TAA)6</td><td>Ca_LG_6</td><td>(TAA)5</td><td>38064923</td><td>38064937</td><td>TCATCAACTAGCATCAGTTAAGCA</td><td>59</td><td>ATTGCAGCCCGTAAATTTTG</td><td>60</td><td>245</td><td>Ca_15328</td><td>URR</td><td>--</td><td>--</td><td>--</td><td>--</td></tr>
<tr><td>TR16188|c0_g1_i1</td><td>XLOC_002648</td><td>(TTA)10</td><td>30</td><td>--</td><td>--</td><td>--</td><td>--</td><td>--</td><td>--</td><td>GGAATGTTGAGGCAAACTTGA</td><td>60</td><td>CAACAAGGTTGTGTTGGTGG</td><td>60</td><td>174</td><td>--</td><td>--</td><td>--</td><td>--</td><td>--</td><td>--</td></tr>
</table>

Transcript IDs	Differentially expressed gene (DEG) IDs	SSR repeat-motifs	SSR repeat-length (bp)	*In silico* polymorphic SSRs						Forward primers (5'-3')	Annealing tempearture(0C)	Reverse primers (5'-3')	Annealing tempearture(0C)	Amplified product size (bp)	Structural annotation		Functional Annotation			
				C. microphyllum		ICC4958									Gene IDs	Sequence component of genes	NCBI nr IDs	TFs	KOG	KEGG
				Transcript	SSR repeat-motifs	Chromosomes	SSR repeat-motifs	Start physical positions (bp)	End physical positions (bp)											
TR16222|c0_g1_i1	XLOC_002666	(TGG)7	21	--	--	--	--	--	--	ATTGATGCAAGGAAGCTTGG	60	CCAAACCACTGCCTAATCC	58	145	--	--	XP_012571226.1	--	--	--
TR16222|c0_g2_i1	XLOC_002667	(TGG)7	21	--	--	--	--	--	--	ATTGATGCAAGGAAGCTTGG	60	CCAAACCACTGCCTAATCC	58	145	--	--	XP_012571226.1	--	--	--
TR16256|c0_g1_i1	XLOC_002679	(CCA)5	15	--	--	--	--	--	--	CCCGTCATTGAAGTTCCAGT	60	GGATCTTGGTCTGAGGGTGA	60	197	--	--	--	--	--	--
TR16300|c0_g1_i1	XLOC_002693	(GA)8	16	--	--	--	--	--	--	TGATTGTGAATTTGGGAGGA	59	GGTAAGTGACGAGGACGAGC	60	159	--	--	--	--	--	--
TR16302|c0_g1_i1	XLOC_002695	(CAC)7	21	--	--	--	--	--	--	CTACGATCACCATGCCCAC	60	CCACAGTGTTGGAGAACTGG	59	279	--	--	XP_008909489.1	--	[J]	K02936
TR16313|c0_g1_i1	XLOC_002700	(CT)6	12	--	--	--	--	--	--	CCGGAAACACCACACTTCTT	60	GAAATGCACTCCCAAAAGGA	60	154	--	--	--	--	--	--
TR1633|c0_g1_i1	No	(AG)7	14	--	--	--	--	--	--	CAAAAAGGCGTTGAGTGTGA	60	GTTACATTTTGGCGCGATTT	60	210	--	--	--	--	--	--
TR1633|c0_g2_i1	No	(AG)7	14	--	--	--	--	--	--	CAAAAAGGCGTTGAGTGTGA	60	GTTACATTTTGGCGCGATTT	60	210	--	--	--	--	--	--
TR16357|c0_g1_i1	XLOC_002721	(CA)6	12	--	--	--	--	--	--	GAAACCAAACGAGAGGACCA	60	CTTTCACCACCAGGGATGAT	60	236	--	--	--	--	--	--
TR16374|c0_g1_i1	No	(TATGC)5	25	--	--	--	--	--	--	AGCAGAATTCGAACAAACCG	60	AGCAGACAGCAGCAGAACAA	60	187	--	--	--	--	--	--
TR1638|c0_g1_i1	XLOC_002730	(T)10gggattgga(T)10	29	--	--	--	--	--	--	ATTGCATGCACGATTGGTAA	60	CCACCAACCGAAGCATTTAT	60	239	--	--	--	--	--	--
TR16384|c0_g1_i1	XLOC_002729	(AT)7	14	--	--	--	--	--	--	CCAATTAGCCGTTTAGGCTG	60	TCGTATTCACCTGCTATATGAACAA	60	169	--	--	--	--	--	--
TR16394|c0_g1_i1	No	(AGA)5	15	--	--	--	--	--	--	ATGTCCAGTTCATCTTCGGC	60	ATGGGACTGGAGGTGAAAGA	60	199	--	--	--	--	--	--
TR16398|c0_g1_i1	No	(TA)8	16	--	--	--	--	--	--	ACGTGTCAGGATTTAACGGC	60	CCAGGATTATATATCAACTCATCCAA	60	232	--	--	--	--	--	--

Transcript IDs	Differentially expressed gene (DEG) IDs	SSR repeat-motifs	SSR repeat-length (bp)	*In silico* polymorphic SSRs						Forward primers (5'-3')	Annealing tempearture(0C)	Reverse primers (5'-3')	Annealing tempearture(0C)	Amplified product size (bp)	Structural annotation		Functional Annotation			
				C. microphyllum		ICC4958									Gene IDs	Sequence component of genes	NCBI nr IDs	TFs	KOG	KEGG
				Transcript	SSR repeat-motifs	Chromosomes	SSR repeat-motifs	Start physical positions (bp)	End physical positions (bp)											
TR16402|c0_g1_i1	No	(CTG)5	15	--	--	--	--	--	--	CAAGGTTCTGGGTGAAGGAG	60	CCAGAACTTTAACCAGGGCA	60	212	--	--	--	--	--	--
TR16417|c0_g1_i1	XLOC_002734	(TCT)6	18	--	--	--	--	--	--	GTCCAAAGTAGCGCTGAAGG	60	AAGAAGAAGTACAGCACGGGA	59	236	--	--	--	--	--	--
TR1656|c0_g1_i2	XLOC_002777	(A)16gttaaaaagaaaaaggtagagaagcataatcacagaatatt(CA)6	69	--	--	--	--	--	--	TGGACCCTTCATGACCTTCT	60	TGTGGAATCATCAATCAAATGAG	60	166	--	--	--	--	--	--
TR16570|c0_g1_i1	No	(TCA)5	15	--	--	--	--	--	--	ATTGCTCGGATACTCGTCCA	61	ATTATGTATGGGGAAGGGGC	60	204	--	--	--	--	--	--
TR1658|c0_g1_i1	XLOC_002780	(CTG)5	15	--	--	--	--	--	--	GTACGGTTCAAGGACGGAAA	60	AGGCCAGATGACATGAATCC	60	113	--	--	KEP46004.1	NAC	[J]	K02976
TR1659|c2_g1_i1	No	(AT)6	12	--	--	--	--	--	--	ATATGCTGGGCCAGTGTTTT	59	CATTTTGGAAGCCAGACCAT	60	167	--	--	--	--	--	--
TR1661|c0_g2_i1	No	(AGA)5	15	--	--	--	--	--	--	ATGGCTCCAAAGTTGGTGAA	60	TTCCTTGGATCAGAACGACC	60	215	--	--	--	--	--	--
TR1661|c0_g2_i2	No	(AGA)5	15	--	--	--	--	--	--	ATGGCTCCAAAGTTGGTGAA	60	TTCCTTGGATCAGAACGACC	60	215	--	--	--	--	--	--
TR1673|c0_g1_i1	No	(TCC)5accaccttcgaattactatccataccctccaccaccacctcctcctccgccggaaaattcatatcagcct(CCA)5	100	--	--	--	--	--	--	GTTACGGTGGTGGTGTTCCT	60	GCGGTTGTTGATGGTTGTATT	60	261	--	--	--	--	--	--
TR1673|c1_g1_i1	XLOC_002847	(GGT)5	15	--	--	--	--	--	--	GACAGGATGGGTATTTCGGA	60	TGTGGATCATGTCCTTGTGG	60	145	--	--	--	--	--	--
TR16732|c1_g1_i1	XLOC_002839	(GGT)5	15	--	--	--	--	--	--	CAGCATCGTAAGAGGCAACA	60	ATGGTTATCCACCTCCACCA	60	249	--	--	--	--	--	--
TR1678|c0_g1_i1	XLOC_002869	(GAA)6	18	--	--	--	--	--	--	CTTCTTTCCGAACGACTTGC	60	ACTCGAACCCCATGTCTCAC	60	202	--	--	--	--	--	--
TR16798|c0_g1_i1	No	(AAT)5	15	--	--	--	--	--	--	TTTGGGAAGAAGAAAGGAAGAA	59	GGAAGCATTGGATGTTCGTT	60	217	--	--	--	--	--	--
TR16800|c0_g1_i1	No	(CG)6	12	--	--	--	--	--	--	AAAGGCCGAGGAGTGAAAAT	60	CGCGCGCCTATATACATCAT	61	189	--	--	--	--	--	--

Transcript IDs	Differentially expressed gene (DEG) IDs	SSR repeat-motifs	SSR repeat-length (bp)	*In silico* polymorphic SSRs						Forward primers (5'-3')	Annealing tempearture(0C)	Reverse primers (5'-3')	Annealing tempearture(0C)	Amplified product size (bp)	Structural annotation		Functional Annotation			
				C. microphyllum		ICC4958									Gene IDs	Sequence component of genes	NCBI nr IDs	TFs	KOG	KEGG
				Transcript	SSR repeat-motifs	Chromosomes	SSR repeat-motifs	Start physical positions (bp)	End physical positions (bp)											
TR16845|c0_g1_i1	No	(GGT)5	15	--	--	--	--	--	--	GTCGTAGTTGCACACGCAGT	60	AGTGGTGGAATATGGGGGTT	60	231	--	--	--	--	--	--
TR16888|c0_g1_i1	XLOC_002910	(AC)7	14	--	--	--	--	--	--	CACAAAACAAGCAAAGCGAA	60	TCGGGACACTCCGAGATAAG	60	198	--	--	--	--	--	--
TR16939|c0_g1_i1	XLOC_002932	(TA)8	16	--	--	--	--	--	--	TCATCAAATGCTTATGTTGCTTG	60	TGCTGATTGTTTGGCATCAT	60	162	--	--	--	--	--	--
TR16973|c0_g1_i1	XLOC_002944	(A)10cttttccaagttatatttgttatttaacttattactttttgaggccca(T)10	68	--	--	--	--	--	--	CACATTGGGTCAGTTTTTGG	59	ACACAAATACGGCGGAGAAC	60	249	--	--	--	--	--	--
TR17002|c0_g1_i1	No	(AG)6	12	--	--	--	--	--	--	GTGGGCTGCAAAGTTATGGT	60	TTGGATTCGCCATTAGTCGT	60	120	--	--	--	--	--	--
TR1704|c1_g1_i1	No	(TAA)5	15	--	--	--	--	--	--	ATGGCCAGTCATGTCACAAA	60	TATCGATTCTTGTGGTGGCA	60	131	--	--	--	--	--	--
TR1704|c1_g1_i2	No	(TAA)5	15	--	--	--	--	--	--	ATGGCCAGTCATGTCACAAA	60	TATCGATTCTTGTGGTGGCA	60	131	--	--	--	--	--	--
TR1704|c1_g1_i3	No	(TAA)5	15	--	--	--	--	--	--	ATGGCCAGTCATGTCACAAA	60	TATCGATTCTTGTGGTGGCA	60	131	--	--	--	--	--	--
TR1704|c1_g1_i4	No	(TAA)5	15	--	--	--	--	--	--	ATGGCCAGTCATGTCACAAA	60	TATCGATTCTTGTGGTGGCA	60	131	--	--	--	--	--	--
TR17057|c0_g1_i1	No	(A)10tcttcaacggtttggggtgtggaaggaaaagtaaaa(T)10	56	--	--	--	--	--	--	TCAGTTCAGTGCGGAATTTG	60	TCCTCACTCGGATCAGCAAT	61	230	--	--	--	--	--	--
TR17094|c0_g1_i1	XLOC_002995	(TTG)6	18	--	--	--	--	--	--	GCAATTAGCATAGTGGTTGGTTT	59	CCATCCCAAGTACACGTTCC	60	200	--	--	--	--	--	--
TR17103|c0_g1_i1	No	(CA)6	12	--	--	--	--	--	--	CCACATCCACATTCACATTCA	60	CAAAGGGCACCTTGAAACAT	60	253	--	--	--	--	--	--
TR17103|c0_g2_i1	No	(CA)6	12	--	--	--	--	--	--	CCACATCCACATTCACATTCA	60	CAAAGGGCACCTTGAAACAT	60	253	--	--	--	--	--	--
TR17116|c0_g1_i1	XLOC_003012	(ATC)5	15	--	--	--	--	--	--	CCCTCTTCAAATTTTCTCACCA	60	CTTTTGAAAGTGCACGAATGA	59	128	--	--	--	--	--	--

Transcript IDs	Differentially expressed gene (DEG) IDs	SSR repeat-motifs	SSR repeat-length (bp)	*In silico* polymorphic SSRs						Forward primers (5'-3')	Annealing tempearture(0C)	Reverse primers (5'-3')	Annealing tempearture(0C)	Amplified product size (bp)	Structural annotation		Functional Annotation			
				C. microphyllum		ICC4958									Gene IDs	Sequence component of genes	NCBI nr IDs	TFs	KOG	KEGG
				Transcript	SSR repeat-motifs	Chromosomes	SSR repeat-motifs	Start physical positions (bp)	End physical positions (bp)											
TR17121|c0_g1_i1	No	(GCA)5	15	--	--	--	--	--	--	ACAATGGAGGAAACGGTCAA	60	CCGTTTCCAGACGATGAACT	60	172	--	--	--	--	--	--
TR1714|c0_g1_i1	XLOC_003034	(TA)7	14	--	--	--	--	--	--	TACCTGGTCCAAGGAGATCG	60	GTTCGATAGTCCGACGCAAT	60	213	--	--	--	--	--	--
TR1714|c0_g1_i2	No	(TA)7	14	--	--	--	--	--	--	TACCTGGTCCAAGGAGATCG	60	GTTCGATAGTCCGACGCAAT	60	213	--	--	--	--	--	--
TR17159|c0_g1_i2	No	(AT)6	12	--	--	--	--	--	--	CCAACAACTTGATGAACGAGAA	60	GGATTTGGATGCTGCTGTTT	60	279	--	--	--	--	--	--
TR17159|c0_g2_i1	No	(AT)6	12	--	--	--	--	--	--	CCAACAACTTGATGAACGAGAA	60	GGATTTGGATGCTGCTGTTT	60	279	--	--	--	--	--	--
TR17167|c0_g1_i1	XLOC_003045	(TCT)6	18	--	--	--	--	--	--	TTCAATCTCTTCACGCCCTT	60	CTGGCAAACATGGTCTTGTG	60	112	--	--	--	--	--	--
TR17186|c0_g1_i1	XLOC_003056	(TCT)5	15	--	--	--	--	--	--	GTGCAGTATGCGCTGACCTA	60	CCAGCCTCTCACAAATGGTT	60	263	--	--	BAD21357.1	--	--	--
TR1720|c0_g1_i1	No	(TTC)6	18	--	--	--	--	--	--	CTACCGGTGATTCTGCAGGT	60	AAACCAAAAGCTTCCCAACA	60	265	--	--	--	--	--	--
TR1720|c0_g1_i2	No	(TTC)6	18	--	--	--	--	--	--	CTACCGGTGATTCTGCAGGT	60	AAACCAAAAGCTTCCCAACA	60	265	--	--	--	--	--	--
TR1722|c0_g1_i1	XLOC_003074	(TC)6	12	--	--	--	--	--	--	CGAAGGGTTTCTGTTGGTGT	60	TGACGAGGAGAAAATATACGGTT	59	265	--	--	--	--	--	--
TR17241|c0_g1_i1	XLOC_003078	(CTG)5	15	--	--	--	--	--	--	GACTGTGTTGCCCAGGTCTC	61	GACCATGTCCAGCGAAGAAG	61	157	--	--	EMT64751.1	--	--	--
TR17241|c0_g1_i1	XLOC_003078	(ATG)5	15	--	--	--	--	--	--	TCTGGTCGTCAGACTCGATG	60	TCAACACTACATTCATCGCCA	60	108	--	--	EMT64751.1	--	--	--
TR17307|c0_g1_i1	No	(TGC)5	15	--	--	--	--	--	--	GGATACGGCAAATTCGGTT	60	AATCCTAGGGGCTTTGAGGA	60	189	--	--	--	--	--	--
TR17331|c0_g1_i1	XLOC_003104	(TC)6	12	--	--	--	--	--	--	ATCGCATCCAAGTCCAAAGA	61	AAATAGCAAGAGCAGGCAGC	60	162	--	--	--	--	--	--

Transcript IDs	Differentially expressed gene (DEG) IDs	SSR repeat-motifs	SSR repeat-length (bp)	*In silico* polymorphic SSRs						Forward primers (5'-3')	Annealing tempearture(0C)	Reverse primers (5'-3')	Annealing tempearture(0C)	Amplified product size (bp)	Structural annotation		Functional Annotation			
				C. microphyllum		ICC4958														
				Transcript	SSR repeat-motifs	Chromosomes	SSR repeat-motifs	Start physical positions (bp)	End physical positions (bp)						Gene IDs	Sequence component of genes	NCBI nr IDs	TFs	KOG	KEGG
TR17333|c0_g1_i1	No	(TAC)6	18	--	--	--	--	--	--	ATCCGCTTCAACACCAAAAC	60	AGGAAGGGAAATTCACACCA	59	232	--	--	--	--	--	--
TR17333|c0_g2_i1	No	(TAC)6	18	--	--	--	--	--	--	ACCGTTGAGCAGGATATTGG	60	GGAAGGGAAATTCACACCAG	59	136	--	--	--	--	--	--
TR1736|c0_g1_i1	No	(AAC)7	21	--	--	--	--	--	--	CACCTGGGGAACCATTACAG	60	GAAACGACGGAGAGAACGAG	60	251	--	--	--	--	--	--
TR1736|c0_g2_i1	No	(AAC)7	21	--	--	--	--	--	--	CACCTGGGGAACCATTACAG	60	GAAACGACGGAGAGAACGAG	60	251	--	--	--	--	--	--
TR1736|c1_g1_i1	No	(GA)6	12	--	--	--	--	--	--	ACAACTAGCCTGCCGCATAC	60	ATCAACCTCTCCTCCACCCT	60	249	--	--	--	--	--	--
TR1736|c1_g1_i2	No	(GA)6	12	--	--	--	--	--	--	ACAACTAGCCTGCCGCATAC	60	ATCAACCTCTCCTCCACCCT	60	249	--	--	--	--	--	--
TR1736|c1_g1_i3	No	(GA)6	12	--	--	--	--	--	--	ACAACTAGCCTGCCGCATAC	60	ATCAACCTCTCCTCCACCCT	60	264	--	--	--	--	--	--
TR1736|c1_g1_i4	No	(GA)6	12	--	--	--	--	--	--	ACAACTAGCCTGCCGCATAC	60	ATCAACCTCTCCTCCACCCT	60	249	--	--	--	--	--	--
TR1736|c2_g1_i2	No	(ACA)6atacatagctgttctttttttgttctttca(T)11	59	--	--	--	--	--	--	TATTGCAGCACCTCAACCAG	60	GCTTCCAATTCTCATGTTGG	58	216	--	--	--	--	--	--
TR1736|c2_g1_i4	No	(ACA)6atacatagctgttctttttttgttctttca(T)11	59	--	--	--	--	--	--	TATTGCAGCACCTCAACCAG	60	GCTTCCAATTCTCATGTTGG	58	216	--	--	--	--	--	--
TR1736|c2_g1_i5	No	(ACA)6atacatagctgttctttttttgttctttca(T)11	59	--	--	--	--	--	--	TATTGCAGCACCTCAACCAG	60	GCTTCCAATTCTCATGTTGG	58	216	--	--	--	--	--	--
TR1736|c3_g1_i1	XLOC_003120	(AAC)6	18	--	--	--	--	--	--	ATTTGCCTTGTGACCAAACC	60	ACTCAAGAACCACACCCCTG	60	255	--	--	--	--	--	--
TR17404|c0_g1_i1	No	(A)10gaacaa(AG)9	34	--	--	--	--	--	--	GATGCTCATGTGGTTTGTGG	60	GGAATTCATGCATGTCTTATTCA	59	180	--	--	--	--	--	--
TR1744|c1_g1_i1	XLOC_003144	(AG)6	12	--	--	--	--	--	--	CACACACCCCACCAATATCA	60	TGCATTTCAGACCATTTCCA	60	253	--	--	--	--	--	--

Transcript IDs	Differentially expressed gene (DEG) IDs	SSR repeat-motifs	SSR repeat-length (bp)	*In silico* polymorphic SSRs						Forward primers (5'-3')	Annealing tempearture(0C)	Reverse primers (5'-3')	Annealing tempearture(0C)	Amplified product size (bp)	Structural annotation		Functional Annotation			
				C. microphyllum		ICC4958									Gene IDs	Sequence component of genes	NCBI nr IDs	TFs	KOG	KEGG
				Transcript	SSR repeat-motifs	Chromosomes	SSR repeat-motifs	Start physical positions (bp)	End physical positions (bp)											
TR1744|c1_g1_i2	XLOC_003145	(AG)6	12	--	--	--	--	--	--	CACACACCCCACCAATATCA	60	TGCATTTCAGACCATTTCCA	60	253	--	--	--	--	--	--
TR17475|c0_g1_i1	XLOC_003158	(ACC)5	15	--	--	--	--	--	--	TCTCGGGAGCAATCTGCTAT	60	CGGTAGTTGTGGGTCTGGTT	60	129	--	--	KEP54602.1	C2H2	--	--
TR17520|c0_g1_i1	XLOC_003189	(GAA)6	18	--	--	--	--	--	--	CGACGAGGATGATGTGAATG	60	AGCCGCCTCTTCTTCATCTT	60	241	--	--	--	--	--	--
TR17527|c0_g1_i1	XLOC_003194	(ACG)5	15	--	--	--	--	--	--	GCATCGCATCAATCAATCAC	60	CTTCGCAGGAAAGACACCTC	60	118	--	--	--	--	--	--
TR1754|c0_g1_i1	No	(GAG)5	15	--	--	--	--	--	--	CAAGGCTCAACAAGGAGAGG	60	CCCTTAAGAGTCCCACTAACTGA	59	213	--	--	--	--	--	--
TR1754|c0_g1_i3	No	(GAG)5	15	--	--	--	--	--	--	CAAGGCTCAACAAGGAGAGG	60	CCCTTAAGAGTCCCACTAACTGA	59	213	--	--	--	--	--	--
TR1756|c0_g1_i1	No	(CT)7	14	--	--	--	--	--	--	GAGATTGTGTTTCGCACCAA	60	CATGAGAAGGAAGGTCATGGA	60	272	--	--	--	--	--	--
TR1756|c0_g1_i1	No	(AG)7	14	--	--	--	--	--	--	TGCAACAATGAGCCCAACTA	60	GGGAATGATAAGGAAAAGGCA	60	220	--	--	--	--	--	--
TR1756|c0_g1_i2	XLOC_003217	(CT)7	14	--	--	--	--	--	--	GAGATTGTGTTTCGCACCAA	60	CATGAGAAGGAAGGTCATGGA	60	272	--	--	--	--	--	--
TR1756|c0_g1_i2	XLOC_003217	(AG)7	14	--	--	--	--	--	--	TGCAACAATGAGCCCAACTA	60	GGGAATGATAAGGAAAAGGCA	60	220	--	--	--	--	--	--
TR17578|c0_g1_i1	No	(ACA)5	15	--	--	--	--	--	--	CTGGTGGTTCTGGTGGTTCT	60	TGAGGTTGACAAATTGGCTG	60	102	--	--	--	--	--	--
TR1762|c1_g1_i5	No	(TA)8	16	--	--	--	--	--	--	AAATCGCGATCTAAGTTGCC	59	AACCGAGCGTACAAACCATC	60	227	--	--	--	--	--	--
TR17645|c0_g1_i1	No	(TTC)5	15	--	--	--	--	--	--	CCAACCCATCTTATTTGTCCA	60	GAACCCAGAAACTGCATCGT	60	270	--	--	--	--	--	--
TR17645|c0_g2_i1	No	(TTC)5	15	--	--	--	--	--	--	CCAACCCATCTTATTTGTCCA	60	GAACCCAGAAACTGCATCGT	60	270	--	--	--	--	--	--

Transcript IDs	Differentially expressed gene (DEG) IDs	SSR repeat-motifs	SSR repeat-length (bp)	*In silico* polymorphic SSRs						Forward primers (5'-3')	Annealing tempearture(0C)	Reverse primers (5'-3')	Annealing tempearture(0C)	Amplified product size (bp)	Structural annotation		Functional Annotation			
				C. microphyllum		ICC4958														
				Transcript	SSR repeat-motifs	Chromosomes	SSR repeat-motifs	Start physical positions (bp)	End physical positions (bp)						Gene IDs	Sequence component of genes	NCBI nr IDs	TFs	KOG	KEGG
TR17667|c1_g1_i1	XLOC_003263	(ACC)5t(GAA)5	31	--	--	--	--	--	--	TGGTTGATGCAGAACCTGAA	60	TCACAACAACAAGCTGCAAG	59	220	--	--	--	--	--	--
TR1769|c0_g1_i1	No	(CAA)6	18	--	--	--	--	--	--	TAGGTCTGCCAAAGGCAATC	60	TGCTTCTTCTACGCTGCTCA	60	197	--	--	--	--	--	--
TR1769|c0_g1_i2	No	(T)10gcgattttatttttctaactttactttttc(T)14	55	--	--	--	--	--	--	GCTGAGCAAAGCAAACATCA	60	TGGCAGACCTAGCCATTTTC	60	260	--	--	--	--	--	--
TR1769|c0_g1_i2	No	(CAA)6	18	--	--	--	--	--	--	TAGGTCTGCCAAAGGCAATC	60	TGCTTCTTCTACGCTGCTCA	60	197	--	--	--	--	--	--
TR1769|c0_g1_i3	No	(CAA)6	18	--	--	--	--	--	--	TAGGTCTGCCAAAGGCAATC	60	TGCTTCTTCTACGCTGCTCA	60	197	--	--	--	--	--	--
TR17697|c0_g1_i1	XLOC_003293	(A)10caagtaatttttttgttactttcttcccacacacgtgtctccttaaa(CT)6	70	--	--	--	--	--	--	CAGTCCTTCGCAACACGTAA	60	ACAAACCCATGATTGCAAGA	59	263	--	--	--	--	--	--
TR17706|c0_g1_i1	No	(GA)7	14	--	--	--	--	--	--	CGCGAGAATAGAGACACCCT	59	AATTTGCTTTACTTGTTTGATTGA	57	271	--	--	--	--	--	--
TR17714|c0_g2_i1	No	(CAA)6	18	--	--	--	--	--	--	AAGAGAAAGAGGGAGCGAGC	60	GCTGCATAGCATATGGGGAT	60	272	--	--	--	--	--	--
TR17714|c0_g2_i2	No	(CAA)6	18	--	--	--	--	--	--	AAGAGAAAGAGGGAGCGAGC	60	GCTGCATAGCATATGGGGAT	60	272	--	--	--	--	--	--
TR1785|c0_g1_i1	No	(GTA)5	15	--	--	--	--	--	--	CTGATACTGCCTGTGCCTCA	60	GATCACACCTCTAAAAACAAACAAA	59	252	--	--	--	--	--	--
TR17864|c0_g1_i1	No	(TA)6	12	--	--	--	--	--	--	AAGGCTTGTTTCATGTTGGC	60	TGCAAATCTACAAATGCTCCA	59	160	--	--	--	--	--	--
TR179|c0_g1_i1	No	(TTCCG)10	50	--	--	--	--	--	--	AATGCCTTGAGTTGAGGGTG	60	GTTTTGACGACGGTTTCCAT	60	185	--	--	--	--	--	--
TR179|c0_g1_i2	No	(TTCCG)6	30	--	--	--	--	--	--	AATGCCTTGAGTTGAGGGTG	60	GTTTTGACGACGGTTTCCAT	60	165	--	--	--	--	--	--
TR17902|c0_g1_i1	No	(AC)8	16	TR17902|c0_g1_i1	(AC)8	Ca_LG_7	(AC)9	23394521	23394538	CTCACGCCCAACACACTCTA	60	GCATTGCGAGAGAGGATTTC	60	278	Ca_16114	DRR	--	--	--	--

Transcript IDs	Differentially expressed gene (DEG) IDs	SSR repeat-motifs	SSR repeat-length (bp)	*In silico* polymorphic SSRs						Forward primers (5'-3')	Annealing tempearture(0C)	Reverse primers (5'-3')	Annealing tempearture(0C)	Amplified product size (bp)	Structural annotation		Functional Annotation			
				C. microphyllum		ICC4958														
				Transcript	SSR repeat-motifs	Chromosomes	SSR repeat-motifs	Start physical positions (bp)	End physical positions (bp)						Gene IDs	Sequence component of genes	NCBI nr IDs	TFs	KOG	KEGG
TR17902|c0_g1_i2	No	(AC)8	16	--	--	--	--	--	--	CTCACGCCCAACACACTCTA	60	GCATTGCGAGAGAGGATTTC	60	278	--	--	--	--	--	--
TR17936|c0_g1_i1	XLOC_003394	(ATA)5	15	--	--	--	--	--	--	AAACACCAAGACATAGCCGC	60	AATGAATGAATGCATTGGGC	61	129	--	--	--	--	--	--
TR1795|c1_g1_i1	XLOC_003416	(AG)6	12	--	--	--	--	--	--	AGACACGATCTTGGGATTGG	60	CAAACAAACAAACTCTGCACG	59	276	--	--	--	--	--	--
TR17978|c0_g1_i1	No	(GT)6	12	--	--	--	--	--	--	CAGGATCCAATATTCCCGAG	59	GGAAGTTCCAGTTCCAACGA	60	208	--	--	--	--	--	--
TR17978|c0_g1_i2	No	(GT)6	12	--	--	--	--	--	--	CAGGATCCAATATTCCCGAG	59	GGAAGTTCCAGTTCCAACGA	60	208	--	--	--	--	--	--
TR1803|c1_g3_i3	XLOC_003452	(AAT)7acataa(T)11	38	--	--	--	--	--	--	GAAACATTGCCTTTGCCACT	60	TGAGTTTATGGGCCTTTTGG	60	161	--	--	XP_012569030.1	M-type	--	--
TR1806|c0_g2_i1	XLOC_003457	(ACA)6	18	--	--	--	--	--	--	GAACGTTAAGAAACAATTCATTTTC	58	CCCTTAAATGTTCTGCAAAGG	59	160	--	--	--	--	--	--
TR1807|c1_g1_i1	XLOC_003461	(AG)10	20	--	--	--	--	--	--	TGCATTCCAAATGAGAGCTG	60	ACGGTGTTGTGTTGGGAACT	60	189	--	--	--	--	--	--
TR18107|c0_g1_i1	No	(TA)7	14	--	--	--	--	--	--	TTATCCAAAGAACCAAGCCG	60	TGGTGTTCCATCCACAGAGA	60	222	--	--	--	--	--	--
TR1812|c4_g1_i1	No	(AGA)5	15	--	--	--	--	--	--	CAACAGCACGAAGAAGACGA	60	ACGCGCTTTTTCTTGTCAAT	60	265	--	--	--	--	--	--
TR1812|c4_g1_i2	XLOC_003474	(AGA)5	15	--	--	--	--	--	--	CAACAGCACGAAGAAGACGA	60	ACGCGCTTTTTCTTGTCAAT	60	265	--	--	--	--	--	--
TR18152|c0_g1_i1	No	(TC)6	12	--	--	--	--	--	--	CCTTCTTTCATGTTTCTTTCCA	58	GAAAATTGTGGTCACAGAGGAA	59	139	--	--	--	--	--	--
TR18179|c0_g2_i1	XLOC_003501	(ACC)6	18	--	--	--	--	--	--	GTTCCGTTCTCCACCTTCAA	60	TTGAGAGAGGGGTGTGTGTG	60	143	--	--	--	--	--	--
TR18179|c1_g1_i1	XLOC_003502	(TTC)5	15	--	--	--	--	--	--	AGCACTCATGGCTCGAACTT	60	CAAGCCGGCTAAAACTCAAG	60	254	--	--	--	--	--	--

<table>
<tr><th rowspan="3">Transcript IDs</th><th rowspan="3">Differentially expressed gene (DEG) IDs</th><th rowspan="3">SSR repeat-motifs</th><th rowspan="3">SSR repeat-length (bp)</th><th colspan="6">In silico polymorphic SSRs</th><th rowspan="3">Forward primers (5'-3')</th><th rowspan="3">Annealing tempearture(0C)</th><th rowspan="3">Reverse primers (5'-3')</th><th rowspan="3">Annealing tempearture(0C)</th><th rowspan="3">Amplified product size (bp)</th><th colspan="2">Structural annotation</th><th colspan="4">Functional Annotation</th></tr>
<tr><th colspan="2">C. microphyllum</th><th colspan="4">ICC4958</th><th rowspan="2">Gene IDs</th><th rowspan="2">Sequence component of genes</th><th rowspan="2">NCBI nr IDs</th><th rowspan="2">TFs</th><th rowspan="2">KOG</th><th rowspan="2">KEGG</th></tr>
<tr><th>Transcript</th><th>SSR repeat-motifs</th><th>Chromosomes</th><th>SSR repeat-motifs</th><th>Start physical positions (bp)</th><th>End physical positions (bp)</th></tr>
<tr><td>TR18181|c0_g1_i1</td><td>XLOC_003506</td><td>(AT)10</td><td>20</td><td>--</td><td>--</td><td>--</td><td>--</td><td>--</td><td>--</td><td>CAATTTCTGTTATAGAGCCTGCAA</td><td>60</td><td>GCAAGAGGTGAGAAAGTCCG</td><td>60</td><td>184</td><td>--</td><td>--</td><td>--</td><td>--</td><td>--</td><td>--</td></tr>
<tr><td>TR18190|c0_g1_i1</td><td>XLOC_003512</td><td>(A)10ttatccactccatagaataac(AAT)5</td><td>46</td><td>--</td><td>--</td><td>--</td><td>--</td><td>--</td><td>--</td><td>TTTAAATCGGCCTTGGTAGC</td><td>59</td><td>TTGGAAACTCCAAGCTGACA</td><td>59</td><td>244</td><td>--</td><td>--</td><td>XP_001944205.2</td><td>B3</td><td>[QI]</td><td>--</td></tr>
<tr><td>TR18196|c0_g1_i1</td><td>XLOC_003517</td><td>(A)10ttataaat(A)10</td><td>28</td><td>--</td><td>--</td><td>--</td><td>--</td><td>--</td><td>--</td><td>CATCCGGTTCAAAGAAACAA</td><td>59</td><td>GGGGGTGATTCTGGAATTTT</td><td>60</td><td>223</td><td>--</td><td>--</td><td>--</td><td>--</td><td>--</td><td>--</td></tr>
<tr><td>TR182|c0_g1_i1</td><td>No</td><td>(GAG)6</td><td>18</td><td>--</td><td>--</td><td>--</td><td>--</td><td>--</td><td>--</td><td>GAGAAGCATTCCACGGGTTA</td><td>60</td><td>CTGTGATCGACACTGCGTTT</td><td>60</td><td>267</td><td>--</td><td>--</td><td>--</td><td>--</td><td>--</td><td>--</td></tr>
<tr><td>TR1820|c0_g1_i1</td><td>No</td><td>(TAG)7</td><td>21</td><td>TR1820|c0_g1_i1</td><td>(TAG)7</td><td>Ca_LG_8</td><td>(TAG)6</td><td>4850665</td><td>4850682</td><td>TCCATCCATCTCCTCCACTC</td><td>60</td><td>GCCCTAGCACACCTTTTTCA</td><td>60</td><td>212</td><td>Ca_20478</td><td>CDS</td><td>--</td><td>--</td><td>--</td><td>--</td></tr>
<tr><td>TR1820|c0_g1_i2</td><td>No</td><td>(TAG)7</td><td>21</td><td>--</td><td>--</td><td>--</td><td>--</td><td>--</td><td>--</td><td>TCCATCCATCTCCTCCACTC</td><td>60</td><td>GCCCTAGCACACCTTTTTCA</td><td>60</td><td>212</td><td>--</td><td>--</td><td>--</td><td>--</td><td>--</td><td>--</td></tr>
<tr><td>TR1821|c0_g3_i1</td><td>No</td><td>(TAAT)5</td><td>20</td><td>--</td><td>--</td><td>--</td><td>--</td><td>--</td><td>--</td><td>TGGTAAGAACCATGCAGCAA</td><td>60</td><td>TTCACCTGTCCAATACATCAAA</td><td>58</td><td>153</td><td>--</td><td>--</td><td>--</td><td>--</td><td>--</td><td>--</td></tr>
<tr><td>TR18210|c0_g1_i2</td><td>No</td><td>(AG)7</td><td>14</td><td>--</td><td>--</td><td>--</td><td>--</td><td>--</td><td>--</td><td>ACGCTGGTTGCACGTTACTC</td><td>61</td><td>ACCGACAGTGGGCATATGAT</td><td>60</td><td>147</td><td>--</td><td>--</td><td>--</td><td>--</td><td>--</td><td>--</td></tr>
<tr><td>TR18210|c0_g1_i3</td><td>No</td><td>(AG)7</td><td>14</td><td>--</td><td>--</td><td>--</td><td>--</td><td>--</td><td>--</td><td>ACGCTGGTTGCACGTTACTC</td><td>61</td><td>ACCGACAGTGGGCATATGAT</td><td>60</td><td>147</td><td>--</td><td>--</td><td>--</td><td>--</td><td>--</td><td>--</td></tr>
<tr><td>TR1823|c0_g1_i1</td><td>No</td><td>(CT)8</td><td>16</td><td>--</td><td>--</td><td>--</td><td>--</td><td>--</td><td>--</td><td>CCTCCACCGATATTTTCACG</td><td>60</td><td>GAACCCTGTGAAGAGGACCA</td><td>60</td><td>181</td><td>--</td><td>--</td><td>--</td><td>--</td><td>--</td><td>--</td></tr>
<tr><td>TR1823|c0_g1_i2</td><td>No</td><td>(CT)8</td><td>16</td><td>--</td><td>--</td><td>--</td><td>--</td><td>--</td><td>--</td><td>CCTCCACCGATATTTTCACG</td><td>60</td><td>GGACCAACCTGTGAAGAGGA</td><td>60</td><td>184</td><td>--</td><td>--</td><td>--</td><td>--</td><td>--</td><td>--</td></tr>
<tr><td>TR1823|c0_g2_i1</td><td>No</td><td>(CT)8</td><td>16</td><td>--</td><td>--</td><td>--</td><td>--</td><td>--</td><td>--</td><td>CCTCCACCGATATTTTCACG</td><td>60</td><td>GAACCCTGTGAAGAGGACCA</td><td>60</td><td>181</td><td>--</td><td>--</td><td>--</td><td>--</td><td>--</td><td>--</td></tr>
<tr><td>TR18232|c0_g3_i1</td><td>XLOC_003544</td><td>(CCA)5</td><td>15</td><td>--</td><td>--</td><td>--</td><td>--</td><td>--</td><td>--</td><td>CATACCCACGCCCAGTCTAC</td><td>60</td><td>GCTTCTTGTACGGTGGTGGT</td><td>60</td><td>141</td><td>--</td><td>--</td><td>--</td><td>--</td><td>--</td><td>--</td></tr>
<tr><td>TR18232|c0_g3_i1</td><td>XLOC_003544</td><td>(CCT)5</td><td>15</td><td>--</td><td>--</td><td>--</td><td>--</td><td>--</td><td>--</td><td>CTTCTCCCCCATACATTCCA</td><td>60</td><td>CAAAACGCGGAAACTTTGTT</td><td>60</td><td>238</td><td>--</td><td>--</td><td>--</td><td>--</td><td>--</td><td>--</td></tr>
</table>

Transcript IDs	Differentially expressed gene (DEG) IDs	SSR repeat-motifs	SSR repeat-length (bp)	*In silico* polymorphic SSRs						Forward primers (5'-3')	Annealing tempearture(0C)	Reverse primers (5'-3')	Annealing tempearture(0C)	Amplified product size (bp)	Structural annotation		Functional Annotation			
				C. microphyllum		ICC4958									Gene IDs	Sequence component of genes	NCBI nr IDs	TFs	KOG	KEGG
				Transcript	SSR repeat-motifs	Chromosomes	SSR repeat-motifs	Start physical positions (bp)	End physical positions (bp)											
TR18235|c0_g3_i1	No	(TA)6	12	--	--	--	--	--	--	ACACGTGGATCCCTTTTGAG	60	CACACATTCCGACGATCAAG	60	258	--	--	--	--	--	--
TR18238|c2_g1_i1	No	(TGA)6	18	--	--	--	--	--	--	GGATGCCGTTACAAGGAAAA	60	TTATGCGGGGAACTACGAAC	60	156	--	--	--	--	--	--
TR18238|c2_g2_i1	No	(TGA)6	18	--	--	--	--	--	--	GGATGCCGTTACAAGGAAAA	60	TTATGCGGGGAACTACGAAC	60	156	--	--	--	--	--	--
TR18238|c2_g3_i1	No	(TGA)6	18	--	--	--	--	--	--	GGATGCCGTTACAAGGAAAA	60	TTATGCGGGGAACTACGAAC	60	156	--	--	--	--	--	--
TR18242|c0_g1_i1	XLOC_003553	(AAG)6	18	--	--	--	--	--	--	GCCATGCTTGGTGAAGAAGT	60	TAGTCCGAAACGAAGAGCGT	60	134	--	--	--	--	--	--
TR18246|c0_g1_i2	XLOC_003558	(TTG)5	15	--	--	--	--	--	--	GTTTTGGGGACAGAGGACAA	60	CACCTTCGTTTTCACTTCCA	59	136	--	--	--	--	--	--
TR18258|c0_g1_i1	No	(CAT)5	15	--	--	--	--	--	--	CAAAGGGTGATCCTTCAAACA	60	TCAATGTTTGCACCATTTTCA	60	190	--	--	--	--	--	--
TR18258|c0_g1_i2	No	(CAT)5	15	--	--	--	--	--	--	TCTTGCCTTCTTTGGTGATTC	59	TGCACCATTTTCATTGTTGG	60	278	--	--	--	--	--	--
TR18258|c0_g3_i1	No	(CAT)5	15	--	--	--	--	--	--	GCCTTCTTTGGTAAAATGGCT	60	TGCACCATTTTCATTGTTGG	60	280	--	--	--	--	--	--
TR18429|c0_g1_i1	No	(AG)6	12	--	--	--	--	--	--	GACAACACCAGCTGTGAGGA	60	CCGACCAAGGAAACAATCTC	60	258	--	--	--	--	--	--
TR18445|c0_g1_i1	XLOC_003666	(TC)6	12	--	--	--	--	--	--	TCCTCTCGTCTTCCCTCAAA	60	TTCATCCTCATCCTTGGGAG	60	247	--	--	--	--	--	--
TR18477|c0_g1_i1	No	(ACT)5	15	--	--	--	--	--	--	CCCATTTTGGAAAACATTGC	60	CAACAGCTTCAGCAGCAAAA	60	232	--	--	--	--	--	--
TR18503|c0_g1_i1	No	(TA)17	34	--	--	--	--	--	--	GAACATGGAGGGTGCAGAAT	60	GAACATGGAGGGTGCAGAAT	60	110	--	--	--	--	--	--
TR18557|c0_g1_i1	XLOC_003683	(TTA)5	15	--	--	--	--	--	--	CATTAGGCAAAGGCACATCA	60	TTTCATTGGCAGATTAGGCA	59	132	--	--	--	--	--	--

Transcript IDs	Differentially expressed gene (DEG) IDs	SSR repeat-motifs	SSR repeat-length (bp)	*In silico* polymorphic SSRs						Forward primers (5'-3')	Annealing tempearture(0C)	Reverse primers (5'-3')	Annealing tempearture(0C)	Amplified product size (bp)	Structural annotation		Functional Annotation			
				C. microphyllum		ICC4958									Gene IDs	Sequence component of genes	NCBI nr IDs	TFs	KOG	KEGG
				Transcript	SSR repeat-motifs	Chromosomes	SSR repeat-motifs	Start physical positions (bp)	End physical positions (bp)											
TR18641|c0_g1_i1	XLOC_003715	(AAC)5	15	--	--	--	--	--	--	GCAAGTCTTGGCAATGATGA	60	TGGAGAGTGGGTTTGATGGT	60	123	--	--	--	--	--	--
TR18641|c0_g1_i1	XLOC_003715	(TTCTC)5	25	--	--	--	--	--	--	TCCTTTTCGCTGCTACATCA	60	TCCATGAGCGTGTTTTTCAG	60	202	--	--	--	--	--	--
TR18708|c0_g1_i1	No	(CAA)6	18	--	--	--	--	--	--	ACAATGTTGCGGAACTAGGG	60	GTTGTAGCTTCTGGAACCGC	60	112	--	--	--	--	--	--
TR18751|c0_g1_i1	No	(A)12g(A)10	23	--	--	--	--	--	--	CCAACATTCAGGTTTCAACTCA	60	GCTTGAACATCCCTGTTGGT	60	177	--	--	--	--	--	--
TR18751|c0_g2_i1	No	(A)12g(A)10	23	--	--	--	--	--	--	CCAACATTCAGGTTTCAACTCA	60	GCTTGAACATCCCTGTTGGT	60	177	--	--	--	--	--	--
TR18752|c0_g1_i1	XLOC_003767	(GA)6	12	--	--	--	--	--	--	ACGGAAAAACCCATCACAGT	59	CAGCAAACTGGTTCAGTCCA	60	138	--	--	--	--	--	--
TR18773|c0_g1_i1	XLOC_003782	(GCA)5tcatagcatttggggaggtgtgaatagtcataatg (CTA)6	68	--	--	--	--	--	--	TTTCTTGCCTTCTCACCTTCA	60	CCCATGTTTTCAACAACACCT	60	207	--	--	--	--	--	--
TR18794|c0_g1_i1	XLOC_003793	(TA)8	16	--	--	--	--	--	--	CCATTCCTGTCCAAAGAACAA	60	TTTGAATTCAGAGATGGGTCTG	59	179	--	--	XM_004491389.1	--	[Z]	--
TR18813|c0_g2_i1	No	(GT)6	12	--	--	--	--	--	--	GAAAAGAAGGGTTGTGGGGT	60	CTTGGCTCTTCGTTGACAAAT	59	138	--	--	--	--	--	--
TR18859|c0_g1_i1	XLOC_003817	(TA)6	12	--	--	--	--	--	--	TCAATTTTGGTCAGATATTTTGGA	60	ACCTGGCAATGTTGAAGGTC	60	164	--	--	--	--	--	--
TR18899|c0_g1_i1	No	(TAT)5	15	--	--	--	--	--	--	CATAGCTAGCCACCACACGA	60	CATAGGAAGTGAAGAGAGGCG	59	130	--	--	--	--	--	--
TR19057|c0_g1_i1	XLOC_003869	(GAT)5	15	--	--	--	--	--	--	CAACGTTTACTTCACGCCAA	60	TGGGTAACAAAGCTACGGGA	60	252	--	--	XP_004489877.1	--	[S]	--
TR19140|c0_g1_i1	XLOC_003887	(AT)8	16	--	--	--	--	--	--	GGATGGTGTTGGCTTGAACT	60	CCAACTTGAGCATGGTTGAA	60	186	--	--	--	--	--	--
TR19151|c0_g1_i1	XLOC_003890	(CAC)5	15	--	--	--	--	--	--	CATGTAATCCAACAGCTCCG	59	AGGGAACGGTTATTATCGGC	60	110	--	--	--	--	--	--

Transcript IDs	Differentially expressed gene (DEG) IDs	SSR repeat-motifs	SSR repeat-length (bp)	*In silico* polymorphic SSRs						Forward primers (5'-3')	Annealing tempearture(0C)	Reverse primers (5'-3')	Annealing tempearture(0C)	Amplified product size (bp)	Structural annotation		Functional Annotation			
				C. microphyllum		ICC4958									Gene IDs	Sequence component of genes	NCBI nr IDs	TFs	KOG	KEGG
				Transcript	SSR repeat-motifs	Chromosomes	SSR repeat-motifs	Start physical positions (bp)	End physical positions (bp)											
TR19208|c0_g1_i1	No	(T)11gtttttta(T)11	30	--	--	--	--	--	--	CCAGCAACTGAATCTGACGA	60	GACGATTCTCGCTTCTTTTGA	60	262	--	--	--	--	--	--
TR19341|c0_g1_i1	XLOC_003913	(AG)9	18	--	--	--	--	--	--	TGTGCTCGCTTAAACCTTCC	60	CATGTGGGTATTCCTTGGTTG	60	208	--	--	--	--	--	--
TR1943|c0_g1_i1	No	(AC)6	12	--	--	--	--	--	--	GGAGGAGATTTGGCTCCTCTA	60	TTGCCTTTTCCATTGCTTTC	60	189	--	--	--	--	--	--
TR19499|c0_g1_i1	XLOC_003969	(TA)8	16	--	--	--	--	--	--	TTTTTAACAACTATCTTCATGGCAA	59	CGGAGTTACTCACGTTGCATT	60	259	--	--	XM_004508141.1	--	[C]	K09699
TR19499|c0_g2_i1	No	(TA)8	16	--	--	--	--	--	--	TTTTTAACAACTATCTTCATGGCAA	59	CGGAGTTACTCACGTTGCATT	60	259	--	--	--	--	--	--
TR19549|c0_g1_i1	XLOC_004015	(GTA)6	18	--	--	--	--	--	--	CCCGATGAGAATTCAGAACC	59	TGCAAGCTTTTAGCACCTCC	61	100	--	--	--	--	--	--
TR19549|c0_g2_i1	No	(GTA)6	18	--	--	--	--	--	--	CCCGATGAGAATTCAGAACC	59	TGCAAGCTTTTAGCACCTCC	61	101	--	--	--	--	--	--
TR19549|c1_g1_i1	XLOC_004016	(AT)7	14	--	--	--	--	--	--	GCTGGCCATTCTTGTGATTT	60	GGCATTTTGGAAGAGCATGT	60	248	--	--	XP_004504297.1	--	[S]	--
TR19554|c0_g1_i1	No	(GTT)5	15	--	--	--	--	--	--	TACGAAACATTCATGGGCAA	60	TCCCAAACCTACCACTTTCG	60	242	--	--	--	--	--	--
TR19570|c0_g2_i1	XLOC_004034	(ACA)5	15	--	--	--	--	--	--	AACAATAGGTGATGCCACCAG	60	TGGTGGCCTTTGATTCCTTA	60	136	--	--	XP_004489028.1	--	[S]	--
TR19682|c0_g1_i1	XLOC_004067	(AAG)5	15	--	--	--	--	--	--	TCCTATCTTCAAGGCCAACG	60	GTTGGCAAGACGACGGTACT	60	256	--	--	XP_002904389.1	MYB	[O]	--
TR19704|c0_g1_i1	No	(GT)6	12	--	--	--	--	--	--	GCCACCGGAAACAAAGTTCT	61	TTTTGCTTCTCTCTCACGCA	60	143	--	--	--	--	--	--
TR19725|c0_g1_i1	XLOC_004083	(TAA)6	18	--	--	--	--	--	--	GCCCATTATACCGTATTTACAGC	59	GATCGAAGAAGAACTGCGCT	60	122	--	--	--	--	--	--
TR19736|c0_g1_i1	XLOC_004088	(CAG)5	15	--	--	--	--	--	--	TGAGGAGTCGTCAGCATTTG	60	AGGATTCGTTACCGACGATG	60	268	--	--	XP_001653857.1	--	--	--

Transcript IDs	Differentially expressed gene (DEG) IDs	SSR repeat-motifs	SSR repeat-length (bp)	*In silico* polymorphic SSRs						Forward primers (5'-3')	Annealing tempearture(0C)	Reverse primers (5'-3')	Annealing tempearture(0C)	Amplified product size (bp)	Structural annotation		Functional Annotation			
				C. microphyllum		ICC4958														
				Transcript	SSR repeat-motifs	Chromosomes	SSR repeat-motifs	Start physical positions (bp)	End physical positions (bp)						Gene IDs	Sequence component of genes	NCBI nr IDs	TFs	KOG	KEGG
TR19744|c0_g1_i1	No	(TG)6	12	--	--	--	--	--	--	CGGTCCATTGCTAATTCCAG	60	GGAAACTGGGCGAATAATGA	60	169	--	--	--	--	--	--
TR19745|c0_g1_i1	No	(GTC)5	15	--	--	--	--	--	--	ACTCGGAATCTCCTGCACAT	60	AACGGTTTCAACACCTACGG	60	157	--	--	--	--	--	--
TR19859|c0_g1_i1	XLOC_004130	(TGT)5	15	--	--	--	--	--	--	GAATGTTGCTGCTGCTGGTA	60	ATGCGGGTTATGGATATGGA	60	244	--	--	--	--	--	--
TR19891|c0_g1_i1	XLOC_004148	(CTT)6	18	--	--	--	--	--	--	GCGGTTGGTGTTTGATTTCT	60	CGGCGTCGAAGAAAGTAGAG	60	255	--	--	--	--	--	--
TR19936|c0_g1_i1	No	(TAT)5	15	--	--	--	--	--	--	GGTTCGATGAAAATGGATGG	60	CCAGACTTCATTGGTTGACA	57	203	--	--	--	--	--	--
TR19957|c0_g1_i1	No	(TGT)7	21	--	--	--	--	--	--	CCCTAACTGGGAAAGCAACA	60	CCACCCCTCAACAACCAC	60	194	--	--	--	--	--	--
TR19998|c0_g1_i1	XLOC_004188	(AAC)5	15	--	--	--	--	--	--	CAATCACAACGAATCATTCTTCA	60	ATGCCGGTGGAGAGTGTAAC	60	245	--	--	XM_004490798.1	C2H2	[R]	--
TR20473|c0_g1_i1	No	(CGA)5	15	--	--	--	--	--	--	TTGTGCGAATCTACAGCGTC	60	GTCGTCGTCGTCATTGTCAT	60	170	--	--	--	--	--	--
TR20539|c0_g1_i1	No	(TGT)5	15	--	--	--	--	--	--	GTTTTCTCTCCTCTGCACGG	60	CAACAGCAACAGTTTGTGCC	60	111	--	--	--	--	--	--
TR20545|c0_g1_i1	XLOC_004370	(AAC)6	18	--	--	--	--	--	--	CAAGGTCACCTATCATTTCAAGA	58	CGACGAAATTTAGACGCAGA	59	205	--	--	--	--	--	--
TR2057|c0_g1_i1	XLOC_004383	(AAAAT)5	25	--	--	--	--	--	--	CCACCATGGCAATCAAAAA	60	GCTCCCGATTTCTAAGTATCATTC	60	255	--	--	--	--	--	--
TR20633|c0_g1_i1	No	(AAG)5	15	--	--	--	--	--	--	TTGTTTCAACAATGAGCACAAA	59	TGACTTGCATTTTCCTTCCA	59	122	--	--	--	--	--	--
TR20655|c1_g1_i1	No	(AAG)6	18	--	--	--	--	--	--	TTCGTAATAGTTATGAATTGCGG	58	ACACTCCACTGATCCATCACA	59	139	--	--	--	--	--	--
TR20725|c0_g1_i1	No	(A)13gagatgtaaaaatatcacatcacaaagaaaatgaattgaatgagaacaaaaatggctatgataaaaggagatt(A)11	97	--	--	--	--	--	--	CTTCAAACATGCAAGCTCCA	60	TGGAGAAGATCGTTCCATGA	59	197	--	--	--	--	--	--

Transcript IDs	Differentially expressed gene (DEG) IDs	SSR repeat-motifs	SSR repeat-length (bp)	*In silico* polymorphic SSRs						Forward primers (5'-3')	Annealing tempearture(0C)	Reverse primers (5'-3')	Annealing tempearture(0C)	Amplified product size (bp)	Structural annotation		Functional Annotation			
				C. microphyllum		ICC4958									Gene IDs	Sequence component of genes	NCBI nr IDs	TFs	KOG	KEGG
				Transcript	SSR repeat-motifs	Chromosomes	SSR repeat-motifs	Start physical positions (bp)	End physical positions (bp)											
TR20725|c0_g2_i1	XLOC_004420	(A)13gagatgtaaaaatatcacatcacaaagaaaatgaattgaatgagaacaaaaatggctatgataaaaggagatt(A)11	97	--	--	--	--	--	--	CTTCAAACATGCAAGCTCCA	60	TGGAGAAGATCGTTCCATGA	59	197	--	--	--	--	--	--
TR20731|c0_g1_i1	XLOC_004424	(ATG)5	15	--	--	--	--	--	--	CCCAAATTTTTGATTCACCG	60	CATCACCATAACCGTCCACA	60	244	--	--	--	--	--	--
TR20821|c0_g1_i1	No	(AGG)6	18	--	--	--	--	--	--	TCGATCTGTAGATCTGTTTGAGGA	60	CTTGGCTTCCACAGGATGTT	60	157	--	--	--	--	--	--
TR2091|c0_g1_i1	No	(AAG)6	18	--	--	--	--	--	--	TTGCTACCACCAGGAACCTC	60	GACAGACCCGGTTACAAGGA	60	174	--	--	--	--	--	--
TR20991|c0_g1_i1	XLOC_004485	(ATC)5	15	--	--	--	--	--	--	GCACCATTGTCCTCGGTACT	60	CCATAGAAGACACAAGCATCG	58	165	--	--	--	--	--	--
TR21137|c0_g2_i1	No	(TCA)5	15	--	--	--	--	--	--	CAATCCACACACATTGTATTGC	59	CGTCGAAGAAGAGGAGGTTG	60	276	--	--	--	--	--	--
TR21137|c0_g2_i2	No	(TCA)5	15	--	--	--	--	--	--	CAATCCACACACATTGTATTGC	59	CGTCGAAGAAGAGGAGGTTG	60	276	--	--	--	--	--	--
TR21137|c0_g2_i3	No	(TCA)5	15	--	--	--	--	--	--	CAATCCACACACATTGTATTGC	59	CGTCGAAGAAGAGGAGGTTG	60	276	--	--	--	--	--	--
TR21186|c1_g1_i1	XLOC_004587	(ATT)6	18	--	--	--	--	--	--	GCACTTGTCATCCAATTTGC	59	GCTTTCAAGCGTGAACTTCC	60	215	--	--	--	--	--	--
TR21199|c1_g1_i1	XLOC_004597	(AAT)5	15	--	--	--	--	--	--	AACTCAACCAGCAACAGCAA	59	AGGTCCATTGGTACCGCTAA	59	162	--	--	--	--	--	--
TR21199|c1_g1_i1	XLOC_004597	(TC)6	12	--	--	--	--	--	--	CTGAAGCAGCAGCATATGGA	60	CATATTTATTCACAATGAGAGTGAGTG	58	223	--	--	--	--	--	--
TR21232|c0_g1_i1	No	(TCA)5	15	--	--	--	--	--	--	TGCAGAATTTGGATCCTTGA	59	CAAATGTCACACACAAGGCA	59	234	--	--	--	--	--	--
TR21240|c0_g1_i1	No	(GA)7	14	--	--	--	--	--	--	GGCACTGATAAGGTGGCAGT	60	TTCTGCAAACACTCAGGCAC	60	102	--	--	--	--	--	--
TR21248|c0_g1_i1	XLOC_004629	(TTC)5	15	--	--	--	--	--	--	CACAGCATGACCTAATCCCA	60	AGGAAAAAGTGAAGCTGCCA	60	106	--	--	--	--	--	--

Transcript IDs	Differentially expressed gene (DEG) IDs	SSR repeat-motifs	SSR repeat-length (bp)	*In silico* polymorphic SSRs						Forward primers (5'-3')	Annealing tempearture(0C)	Reverse primers (5'-3')	Annealing tempearture(0C)	Amplified product size (bp)	Structural annotation		Functional Annotation			
				C. microphyllum		ICC4958									Gene IDs	Sequence component of genes	NCBI nr IDs	TFs	KOG	KEGG
				Transcript	SSR repeat-motifs	Chromosomes	SSR repeat-motifs	Start physical positions (bp)	End physical positions (bp)											
TR21250|c0_g1_i1	No	(ATA)6	18	--	--	--	--	--	--	TAGATTCCGACCATGCCTTC	60	CCACTCGTATTTCCTGAGGC	60	173	--	--	--	--	--	--
TR21250|c0_g1_i2	No	(ATA)6	18	--	--	--	--	--	--	TAGATTCCGACCATGCCTTC	60	CCACTCGTATTTCCTGAGGC	60	173	--	--	--	--	--	--
TR21290|c0_g1_i1	No	(A)10caatataattcacttaaatcaatgc(A)10	45	--	--	--	--	--	--	ATTGTGCTGCAACGTGAAAC	60	AAATCAATCCTGACCTGCAAA	60	173	--	--	--	--	--	--
TR21348|c0_g1_i1	No	(AT)6	12	--	--	--	--	--	--	GGTTCTCAACAACATTGGAGC	60	CGTTGTGAGACCAATTTGACA	60	160	--	--	--	--	--	--
TR21382|c1_g2_i1	No	(ACA)6	18	--	--	--	--	--	--	TCTCACAATTTTGCCCCAAT	60	ATAGCTTGGAAGCCTCACGA	60	162	--	--	--	--	--	--
TR21382|c1_g2_i2	No	(ACA)6	18	--	--	--	--	--	--	TCTCACAATTTTGCCCCAAT	60	ATAGCTTGGAAGCCTCACGA	60	162	--	--	--	--	--	--
TR21382|c1_g2_i3	No	(ACA)6	18	--	--	--	--	--	--	TCTCACAATTTTGCCCCAAT	60	ATAGCTTGGAAGCCTCACGA	60	162	--	--	--	--	--	--
TR21382|c1_g3_i2	No	(ACA)6	18	--	--	--	--	--	--	TCTCACAATTTTGCCCCAAT	60	ATAGCTTGGAAGCCTCACGA	60	162	--	--	--	--	--	--
TR21382|c1_g3_i3	No	(ACA)6	18	--	--	--	--	--	--	TCTCACAATTTTGCCCCAAT	60	ATAGCTTGGAAGCCTCACGA	60	162	--	--	--	--	--	--
TR21382|c2_g1_i1	XLOC_004686	(T)10ctttttgacaagtcatcctcaatggtcagtacaaacaccttaaaaacttcacgtgtaacttttcaaacccgacaaaactaca(AT)7	106	--	--	--	--	--	--	GAAAAAGAGCCGAGAGCAAA	60	TGATGCTCGTGTTTCCTTGA	60	276	--	--	--	--	--	--
TR21395|c0_g1_i1	No	(TGT)5	15	--	--	--	--	--	--	AGCACAGAAGACACGTCCAA	59	TGCCGTTGAGTGACCAACTA	60	202	--	--	--	--	--	--
TR21395|c0_g1_i2	No	(TGT)5	15	--	--	--	--	--	--	AGCACAGAAGACACGTCCAA	59	TGCCGTTGAGTGACCAACTA	60	202	--	--	--	--	--	--
TR21397|c1_g2_i1	No	(AAT)6	18	--	--	--	--	--	--	CAATCAATCGCACTTATGTGAA	59	CTAGTGACGTGGACTGCCAA	60	170	--	--	--	--	--	--
TR21397|c1_g2_i2	No	(AAT)6	18	--	--	--	--	--	--	CAATCAATCGCACTTATGTGAA	59	CTAGTGACGTGGACTGCCAA	60	170	--	--	--	--	--	--

Transcript IDs	Differentially expressed gene (DEG) IDs	SSR repeat-motifs	SSR repeat-length (bp)	*In silico* polymorphic SSRs						Forward primers (5'-3')	Annealing tempearture(0C)	Reverse primers (5'-3')	Annealing tempearture(0C)	Amplified product size (bp)	Structural annotation		Functional Annotation			
				C. microphyllum		ICC4958														
				Transcript	SSR repeat-motifs	Chromosomes	SSR repeat-motifs	Start physical positions (bp)	End physical positions (bp)						Gene IDs	Sequence component of genes	NCBI nr IDs	TFs	KOG	KEGG
TR21398|c0_g1_i1	No	(TGA)5	15	--	--	--	--	--	--	CAAAGTGAGGGTGTTCGTGA	60	ATCCCAAATGAACATTCCCA	60	277	--	--	--	--	--	--
TR21398|c0_g1_i10	No	(TGA)5	15	--	--	--	--	--	--	CAGTGAGGGTGTTCGTGATG	60	CCCAAATGAACATTCCCAAA	61	273	--	--	--	--	--	--
TR21398|c0_g1_i11	No	(TGA)5	15	--	--	--	--	--	--	CAAAGTGAGGGTGTTCGTGA	60	ATCCCAAATGAACATTCCCA	60	277	--	--	--	--	--	--
TR21398|c0_g1_i12	No	(TGA)5	15	--	--	--	--	--	--	CAGTGAGGGTGTTCGTGATG	60	AAATTGCACGTTTCCAGCAT	61	182	--	--	--	--	--	--
TR21398|c0_g1_i13	No	(TGA)5	15	--	--	--	--	--	--	CAGTGAGGGTGTTCGTGATG	60	GAGCATAGAGGCCTTCTCCA	60	214	--	--	--	--	--	--
TR21398|c0_g1_i2	No	(TGA)5	15	--	--	--	--	--	--	TGGGGAAAATGTCAAAGACC	60	AAATTGCACGTTTCCAGCAT	61	256	--	--	--	--	--	--
TR21398|c0_g1_i3	No	(TGA)5	15	--	--	--	--	--	--	CAGTGAGGGTGTTCGTGATG	60	AAAGATCCACTGGGGGAGTT	60	255	--	--	--	--	--	--
TR21398|c0_g1_i4	No	(TGA)5	15	--	--	--	--	--	--	CAGTGAGGGTGTTCGTGATG	60	TACAACATTGACTGGCCTGG	60	217	--	--	--	--	--	--
TR21398|c0_g1_i5	No	(TGA)5	15	--	--	--	--	--	--	CAAAGTGAGGGTGTTCGTGA	60	AAAGATCCACTGGGGGAGTT	60	257	--	--	--	--	--	--
TR21398|c0_g1_i6	No	(TGA)5	15	--	--	--	--	--	--	CAGTGAGGGTGTTCGTGATG	60	TACAACATTGACTGGCCTGG	60	217	--	--	--	--	--	--
TR21398|c0_g1_i7	No	(TGA)5	15	--	--	--	--	--	--	CAGTGAGGGTGTTCGTGATG	60	AAAGATCCACTGGGGGAGTT	60	255	--	--	--	--	--	--
TR21398|c0_g1_i8	No	(TGA)5	15	--	--	--	--	--	--	CAGTGAGGGTGTTCGTGATG	60	TACAACATTGACTGGCCTGG	60	217	--	--	--	--	--	--
TR21398|c0_g1_i9	No	(TGA)5	15	--	--	--	--	--	--	CAAAGTGAGGGTGTTCGTGA	60	ATCCCAAATGAACATTCCCA	60	277	--	--	--	--	--	--
TR21406|c0_g1_i1	No	(GA)6	12	--	--	--	--	--	--	TTCTTCCTACCCTCGCACAC	60	TCCCTTGGTTCCCTTCTCTT	60	173	--	--	--	--	--	--

Transcript IDs	Differentially expressed gene (DEG) IDs	SSR repeat-motifs	SSR repeat-length (bp)	*In silico* polymorphic SSRs						Forward primers (5'-3')	Annealing tempearture(0C)	Reverse primers (5'-3')	Annealing tempearture(0C)	Amplified product size (bp)	Structural annotation		Functional Annotation			
				C. microphyllum		ICC4958									Gene IDs	Sequence component of genes	NCBI nr IDs	TFs	KOG	KEGG
				Transcript	SSR repeat-motifs	Chromosomes	SSR repeat-motifs	Start physical positions (bp)	End physical positions (bp)											
TR21406|c0_g1_i2	No	(GA)6	12	--	--	--	--	--	--	TTCTTCCTACCCTCGCACAC	60	TCCCTTGGTTCCCTTCTCTT	60	173	--	--	--	--	--	--
TR21406|c0_g1_i3	No	(AG)8	16	--	--	--	--	--	--	CTTCTTCCTACCCTCACACACA	59	TCCCTTGGTTCCCTTCTCTT	60	191	--	--	--	--	--	--
TR21406|c0_g1_i4	No	(AG)8	16	--	--	--	--	--	--	CTTCTTCCTACCCTCACACACA	59	TCCCTTGGTTCCCTTCTCTT	60	191	--	--	--	--	--	--
TR21413|c1_g1_i4	XLOC_004728	(TTC)5aa(TTG)5	32	--	--	--	--	--	--	GCAAACAAGGGCAAACAAAT	60	TGGTGGAAGCTCACCAATAA	59	186	--	--	--	--	--	--
TR21418|c0_g1_i1	No	(GAA)5	15	--	--	--	--	--	--	GAGGGCCTCGAAGTAACAGA	59	GCCGCCTATGTGAAGGTAAA	60	231	--	--	--	--	--	--
TR21418|c0_g2_i1	No	(GAA)5	15	--	--	--	--	--	--	GAGGGCCTCGAAGTAACAGA	59	GCCGCCTATGTGAAGGTAAA	60	231	--	--	--	--	--	--
TR21430|c0_g2_i1	No	(TTA)5	15	--	--	--	--	--	--	AACAGCCTTCAACCATCCAG	60	GGTTTAAGCAGTTTCGGCTG	60	220	--	--	--	--	--	--
TR21430|c0_g2_i2	No	(TTA)5	15	--	--	--	--	--	--	AACAGCCTTCAACCATCCAG	60	GGTTTAAGCAGTTTCGGCTG	60	220	--	--	--	--	--	--
TR21430|c0_g3_i1	No	(TTA)5	15	--	--	--	--	--	--	AACAGCCTTCAACCATCCAG	60	GGTTTAAGCAGTTTCGGCTG	60	220	--	--	--	--	--	--
TR21434|c0_g2_i2	XLOC_004755	(TC)10aatcatcaatcttttgtagttcaaggttgttgctttaaggaaagttgaatttggtttctatttttctttttgttatgacact(TG)6	115	--	--	--	--	--	--	TCATTCTTTTCACGTGGGGT	60	CCCCACTTCAATTTTTCAGG	59	277	--	--	--	--	--	--
TR21441|c3_g1_i1	No	(ACA)5	15	--	--	--	--	--	--	AAAAGCATCTCCACCGCTC	60	GCAAAAGCAAGAGGTGAATAGTT	59	135	--	--	--	--	--	--
TR21441|c3_g1_i3	No	(ACA)5	15	--	--	--	--	--	--	AAAAGCATCTCCACCGCTC	60	GCAAAAGCAAGAGGTGAATAGTT	59	135	--	--	--	--	--	--
TR21441|c3_g1_i6	No	(ACA)5	15	--	--	--	--	--	--	AAAAGCATCTCCACCGCTC	60	GCAAAAGCAAGAGGTGAATAGTT	59	135	--	--	--	--	--	--
TR21450|c0_g1_i1	XLOC_004781	(GT)9	18	--	--	--	--	--	--	AAAGATGGTGAATTGGTCGC	60	CACTGCAATCCTCCTTCTCTC	59	170	--	--	--	--	--	--

Transcript IDs	Differentially expressed gene (DEG) IDs	SSR repeat-motifs	SSR repeat-length (bp)	*In silico* polymorphic SSRs						Forward primers (5'-3')	Annealing tempearture(0C)	Reverse primers (5'-3')	Annealing tempearture(0C)	Amplified product size (bp)	Structural annotation		Functional Annotation			
				C. microphyllum		ICC4958									Gene IDs	Sequence component of genes	NCBI nr IDs	TFs	KOG	KEGG
				Transcript	SSR repeat-motifs	Chromosomes	SSR repeat-motifs	Start physical positions (bp)	End physical positions (bp)											
TR21455|c1_g1_i1	XLOC_004783	(A)10taaaaaaagagatattatataaatatgtatacgatccaaaacaagagaggaggggagcgagtgtttctctctctcgaatc(T)10	100	--	--	--	--	--	--	TTCAATCAATTCATGTAAGGAAGA	58	GCAGTGGGGTGTACTCTTGG	61	280	--	--	--	--	--	--
TR21456|c0_g1_i1	No	(TCA)5	15	--	--	--	--	--	--	AGCCCAAAGGACCTTCTCAT	60	GCTGAAGGTGATTGGTTTGG	60	218	--	--	--	--	--	--
TR21456|c0_g1_i2	No	(TCA)5	15	--	--	--	--	--	--	AGCCCAAAGGACCTTCTCAT	60	GCTGAAGGTGATTGGTTTGG	60	218	--	--	--	--	--	--
TR21467|c1_g1_i1	No	(TTC)5tctactacttca(CT)6	39	--	--	--	--	--	--	CAGACCAGATGAAGCAACGA	60	CCGGCAATGAAATAGAAGGA	60	223	--	--	--	--	--	--
TR21467|c1_g1_i3	No	(TTC)5tctactacttca(CT)6	39	--	--	--	--	--	--	CAGACCAGATGAAGCAACGA	60	CCGGCAATGAAATAGAAGGA	60	223	--	--	--	--	--	--
TR21469|c0_g1_i1	No	(AC)6cggaggcggtgg(AC)6cggaggccgtgg(AC)6cggaggccgtgg(AC)6cggaggctttgg(AC)6cggaggcggtgg(AC)6	132	--	--	--	--	--	--	CCACCAGGCCTACCTTCATA	60	CCTCCACCACCAAAAGAAAA	60	233	--	--	--	--	--	--
TR21469|c0_g1_i3	No	(AC)6cggaggcggtgg(AC)6cggaggccgtgg(AC)6cggaggccgtgg(AC)6cggaggctttgg(AC)6cggaggcggtgg(AC)6	132	--	--	--	--	--	--	CCACCAGGCCTACCTTCATA	60	CCTCCACCACCAAAAGAAAA	60	233	--	--	--	--	--	--
TR21469|c0_g1_i6	No	(AC)6cggaggcggtgg(AC)6	36	--	--	--	--	--	--	TGCCACCACCGTAACTATCA	60	CCTCCACCACCAAAAGAAAA	60	226	--	--	--	--	--	--
TR21469|c0_g1_i7	No	(AC)6cggaggcggtgg(AC)6	36	--	--	--	--	--	--	TGCCACCACCGTAACTATCA	60	CCTCCACCACCAAAAGAAAA	60	226	--	--	--	--	--	--
TR21469|c0_g1_i8	No	(AC)6cggaggcggtgg(AC)6cggaggccgtgg(AC)6cggaggccgtgg(AC)6cggaggctttgg(AC)6cggaggcggtgg(AC)6	132	--	--	--	--	--	--	CCACCAGGCCTACCTTCATA	60	CCTCCACCACCAAAAGAAAA	60	233	--	--	--	--	--	--
TR21469|c0_g1_i9	No	(AC)6cggaggcggtgg(AC)6	36	--	--	--	--	--	--	TGCCACCACCGTAACTATCA	60	CCTCCACCACCAAAAGAAAA	60	226	--	--	--	--	--	--
TR21477|c5_g1_i1	XLOC_004805	(GAT)6	18	--	--	--	--	--	--	AGAATCGGCAGATTTGGATG	60	CCTCAGCGTCATCATCTTCA	60	227	--	--	--	--	--	--
TR21477|c8_g1_i1	No	(TGA)6	18	--	--	--	--	--	--	AGCGATGATGCTGATGATGA	60	TCAGTTGCCTGTGCTTGTTC	60	170	--	--	--	--	--	--
TR21496|c0_g1_i1	XLOC_004815	(TTG)5	15	--	--	--	--	--	--	TCCATGTGAAACTGGTTGGA	60	GGCCAAAAGAACAGGTTTTG	60	148	--	--	--	--	--	--

Transcript IDs	Differentially expressed gene (DEG) IDs	SSR repeat-motifs	SSR repeat-length (bp)	*In silico* polymorphic SSRs						Forward primers (5'-3')	Annealing tempearture(0C)	Reverse primers (5'-3')	Annealing tempearture(0C)	Amplified product size (bp)	Structural annotation		Functional Annotation			
				C. microphyllum		ICC4958									Gene IDs	Sequence component of genes	NCBI nr IDs	TFs	KOG	KEGG
				Transcript	SSR repeat-motifs	Chromosomes	SSR repeat-motifs	Start physical positions (bp)	End physical positions (bp)											
TR21524|c0_g1_i1	No	(CA)6	12	--	--	--	--	--	--	GAAACCAAGAAGGGCAACAA	60	TGTTTTAGTTGGGTAGCCCG	60	159	--	--	--	--	--	--
TR21604|c0_g1_i1	XLOC_004843	(CTC)5	15	--	--	--	--	--	--	TGCAATTTACATGCTTCCCA	60	ATTTGAGCCAGTGGATGAGG	60	263	--	--	--	--	--	--
TR21642|c0_g1_i1	XLOC_004851	(CT)9	18	--	--	--	--	--	--	TAAGCATCCCACTGTCACGA	60	TGAGCAAAAAGCAGGGTCTT	60	161	--	--	--	--	--	--
TR21683|c0_g1_i1	No	(GTG)5	15	--	--	--	--	--	--	GATCGTTGGTGTGTGTGGTC	60	TCAAGCTGTCTGATGGCAAG	60	229	--	--	--	--	--	--
TR21708|c0_g1_i1	XLOC_004861	(AGC)5	15	--	--	--	--	--	--	CGTCATTTTACCGGAAGTCA	59	GAGGCATCACCGGATTCTTA	60	267	--	--	CEL61932.1	--	--	--
TR21733|c0_g1_i1	XLOC_004871	(CAGG)5	20	--	--	--	--	--	--	GCTGAGGTGAAGCACAATCA	60	ATACATAAGCCAACCCACGC	60	116	--	--	--	--	--	--
TR21795|c0_g1_i1	XLOC_004894	(ATG)6	18	TR21795|c0_g1_i1	(ATG)6	Ca_LG_4	(ATG)5	25667601	25667615	TCCCTCAATGATGGCTAAAGA	60	GAGTTGATGGGGTGGTGAAT	60	260	Ca_01733	URR	XM_004496754.1	--	--	--
TR21844|c1_g1_i1	No	(CCT)5	15	--	--	--	--	--	--	GCTTGACAATAAACTCCGCC	60	GTCGATGAATACGCCAACCT	60	115	--	--	--	--	--	--
TR21859|c0_g1_i1	XLOC_004922	(ACT)5	15	--	--	--	--	--	--	TTCTGTCGTTGTCGTTGTGG	61	GCAAAGTACCTTCCCCTTCC	60	218	--	--	XP_004508497.1	ERF	[K]	--
TR21860|c0_g1_i1	XLOC_004924	(GTG)5	15	--	--	--	--	--	--	CATCGTAGTGGTGACCAACG	60	ACCGCCCAAACACTCAATAG	60	143	--	--	CCO26440.1	--	--	--
TR2193|c0_g1_i1	No	(AT)6	12	TR2193|c0_g1_i1	(AT)6	Ca_LG_8	(AT)8	7970304	7970319	GGATCCACAAAACTCCCAAC	59	CTCACTATTCCGCAAGCACA	60	181	Ca_19649	CDS	--	--	--	--
TR22013|c0_g1_i1	XLOC_004970	(AGG)5	15	--	--	--	--	--	--	GGTAATGGAGGAGGAGAGCC	60	CCCCCAGTCTTGCTTGAATA	60	250	--	--	--	--	--	--
TR22013|c0_g1_i2	No	(AGG)5	15	--	--	--	--	--	--	GGTAATGGAGGAGGAGAGCC	60	CCCCCAGTCTTGCTTGAATA	60	250	--	--	--	--	--	--
TR22017|c0_g1_i1	XLOC_004973	(ATT)5	15	--	--	--	--	--	--	CATGATGTCAGCATCAACCA	59	GGTTCTGGTGGAAGGAGGAT	60	146	--	--	--	--	--	--

Transcript IDs	Differentially expressed gene (DEG) IDs	SSR repeat-motifs	SSR repeat-length (bp)	*In silico* polymorphic SSRs						Forward primers (5'-3')	Annealing tempearture(0C)	Reverse primers (5'-3')	Annealing tempearture(0C)	Amplified product size (bp)	Structural annotation		Functional Annotation			
				C. microphyllum		ICC4958									Gene IDs	Sequence component of genes	NCBI nr IDs	TFs	KOG	KEGG
				Transcript	SSR repeat-motifs	Chromosomes	SSR repeat-motifs	Start physical positions (bp)	End physical positions (bp)											
TR22017|c0_g1_i2	No	(ATT)5	15	--	--	--	--	--	--	CATGATGTCAGCATCAACCA	59	GGTTCTGGTGGAAGGAGGAT	60	146	--	--	--	--	--	--
TR22061|c0_g1_i1	No	(AG)6	12	--	--	--	--	--	--	CGCAAACGCACTTAATTTGA	60	AACACCGGAACGTAAACGAG	60	160	--	--	--	--	--	--
TR22061|c0_g1_i2	No	(AG)6	12	--	--	--	--	--	--	CGCAAACGCACTTAATTTGA	60	AACACCGGAACGTAAACGAG	60	160	--	--	--	--	--	--
TR22096|c1_g1_i1	No	(CT)10	20	--	--	--	--	--	--	TGGCGGTGTATAACAACGAA	60	TAATTGCCCCTATTCGCAAC	60	159	--	--	--	--	--	--
TR22096|c1_g2_i1	No	(CT)10	20	--	--	--	--	--	--	TGGCGGTGTATAACAACGAA	60	TAATTGCCCCTATTCGCAAC	60	159	--	--	--	--	--	--
TR22100|c1_g1_i1	XLOC_005050	(CAT)5aatcaaaatcaacaaacaaaac(AGA)5	52	--	--	--	--	--	--	GATTGAGCCGTGTAGCCAAT	60	TGGTCTTGTGGCTTTCACAG	60	235	--	--	--	--	--	--
TR22149|c0_g1_i1	XLOC_005073	(CTT)6	18	--	--	--	--	--	--	TCACACCAGAAGGACCAACA	60	ACTTCGCTTCGTCTTTTGGA	60	239	--	--	--	--	--	--
TR22193|c0_g1_i1	No	(CTC)5	15	--	--	--	--	--	--	CCTCTCAATCTCCGAATCCA	60	CTGATGGTGTTCAGGGTGTG	60	140	--	--	--	--	--	--
TR22282|c2_g1_i1	No	(TAA)6	18	--	--	--	--	--	--	TCCTCCCTCATATTCTCCCC	60	ACTGCTGCTTGTGATGATGC	60	263	--	--	--	--	--	--
TR22282|c4_g2_i1	XLOC_005111	(TCA)5	15	--	--	--	--	--	--	TTGAGGCCAGGAATCAAAAG	60	CTTTCCCATTGACCTCTAGGA	58	266	--	--	--	--	--	--
TR22282|c7_g1_i1	No	(ATA)5	15	--	--	--	--	--	--	TCAGAAACAATCCTCCTCCTG	59	GGGATGAATCAGATGGTTGTG	60	213	--	--	--	--	--	--
TR22282|c7_g1_i2	No	(ATA)5	15	--	--	--	--	--	--	TCAGAAACAATCCTCCTCCTG	59	GGGATGAATCAGATGGTTGTG	60	213	--	--	--	--	--	--
TR22284|c0_g1_i1	XLOC_005121	(CAT)5	15	--	--	--	--	--	--	CCGATTCAATTCATTCCCAG	60	GTGAAACGGAACCTCCAGAA	60	152	--	--	--	--	--	--
TR22288|c0_g1_i1	XLOC_005128	(CAA)5	15	--	--	--	--	--	--	CCTTAAACCCATCTCAGCCA	60	AGAGAAGTTGGGGGTGGTCT	60	132	--	--	XP_004495565.1	--	[X]	--

Transcript IDs	Differentially expressed gene (DEG) IDs	SSR repeat-motifs	SSR repeat-length (bp)	*In silico* polymorphic SSRs						Forward primers (5'-3')	Annealing tempearture(0C)	Reverse primers (5'-3')	Annealing tempearture(0C)	Amplified product size (bp)	Structural annotation		Functional Annotation			
				C. microphyllum		ICC4958									Gene IDs	Sequence component of genes	NCBI nr IDs	TFs	KOG	KEGG
				Transcript	SSR repeat-motifs	Chromosomes	SSR repeat-motifs	Start physical positions (bp)	End physical positions (bp)											
TR22296|c0_g1_i1	XLOC_005137	(CGC)5	15	--	--	--	--	--	--	ACTGCCTACGCAAAGGAGC	61	GCGCTTCTCAAGCGATACAT	61	198	--	--	--	--	--	--
TR22300|c0_g3_i1	XLOC_005143	(GA)8	16	--	--	--	--	--	--	ATACACGTGGTGGAGGAAGC	60	TTTGCTTGATCTGCTGCTGT	60	191	--	--	--	--	--	--
TR22302|c0_g1_i1	No	(AT)7	14	--	--	--	--	--	--	TGAGGCTCCACACTCAGAGA	60	CTTTTCTTCCTACCTGCCCC	60	159	--	--	--	--	--	--
TR22306|c3_g2_i2	No	(ATT)6	18	--	--	--	--	--	--	TCTGAATTAACGCCGTCCTC	60	TGACTTCTTGTCATCCCCAA	59	219	--	--	--	--	--	--
TR22306|c3_g2_i6	No	(ATT)6	18	--	--	--	--	--	--	TCTGAATTAACGCCGTCCTC	60	TGACTTCTTGTCATCCCCAA	59	219	--	--	--	--	--	--
TR22306|c3_g2_i7	No	(ATT)6	18	--	--	--	--	--	--	TCTGAATTAACGCCGTCCTC	60	TGACTTCTTGTCATCCCCAA	59	219	--	--	--	--	--	--
TR22317|c1_g2_i2	No	(AG)6	12	--	--	--	--	--	--	GCCCTTACGGACAACATTTC	59	TTTTTGGTGAAGTGATAGCCA	58	165	--	--	--	--	--	--
TR22317|c1_g2_i6	No	(AG)6	12	--	--	--	--	--	--	GCCCTTACGGACAACATTTC	59	TTTTTGGTGAAGTGATAGCCA	58	165	--	--	--	--	--	--
TR22319|c0_g1_i1	No	(GA)14	28	--	--	--	--	--	--	GTCGGTTTGTCTCCCACTGT	60	CTGTTGCTGCTCACACCATT	60	277	--	--	--	--	--	--
TR22319|c0_g1_i2	No	(GA)14	28	--	--	--	--	--	--	GTCGGTTTGTCTCCCACTGT	60	CTGTTGCTGCTCACACCATT	60	277	--	--	--	--	--	--
TR22319|c0_g1_i3	No	(GA)14	28	--	--	--	--	--	--	GTCGGTTTGTCTCCCACTGT	60	CTGTTGCTGCTCACACCATT	60	277	--	--	--	--	--	--
TR22319|c0_g1_i4	No	(GA)14	28	--	--	--	--	--	--	GTCGGTTTGTCTCCCACTGT	60	CTGTTGCTGCTCACACCATT	60	277	--	--	--	--	--	--
TR22323|c1_g1_i1	No	(TCA)7ctgcgattcagcaagaacatctctttctctttc(TCT)6	72	--	--	--	--	--	--	TGAAACAGCCTTTTGTTCCC	60	CACATTTTTGGGGAAGCCTA	60	183	--	--	--	--	--	--
TR22323|c1_g1_i2	No	(TCA)7ctgcgattcagcaagaacatctctttctctttc(TCT)6	72	--	--	--	--	--	--	TGAAACAGCCTTTTGTTCCC	60	CACATTTTTGGGGAAGCCTA	60	183	--	--	--	--	--	--

Transcript IDs	Differentially expressed gene (DEG) IDs	SSR repeat-motifs	SSR repeat-length (bp)	*In silico* polymorphic SSRs						Forward primers (5'-3')	Annealing tempearture(0C)	Reverse primers (5'-3')	Annealing tempearture(0C)	Amplified product size (bp)	Structural annotation		Functional Annotation			
				C. microphyllum		ICC4958									Gene IDs	Sequence component of genes	NCBI nr IDs	TFs	KOG	KEGG
				Transcript	SSR repeat-motifs	Chromosomes	SSR repeat-motifs	Start physical positions (bp)	End physical positions (bp)											
TR22323|c1_g1_i3	No	(TCA)7ctgcgattcagcaagaacatctctttctctttc(TCT)6	72	--	--	--	--	--	--	TGAAACAGCCTTTTGTTCCC	60	CACATTTTTGGGGAAGCCTA	60	183	--	--	--	--	--	--
TR22323|c1_g1_i4	No	(TCA)7ctgcgattcagcaagaacatctctttctctttc(TCT)6	72	--	--	--	--	--	--	TGAAACAGCCTTTTGTTCCC	60	CACATTTTTGGGGAAGCCTA	60	183	--	--	--	--	--	--
TR22323|c1_g1_i5	No	(TCA)7ctgcgattcagcaagaacatctctttctctttc(TCT)6	72	--	--	--	--	--	--	TGAAACAGCCTTTTGTTCCC	60	CACATTTTTGGGGAAGCCTA	60	183	--	--	--	--	--	--
TR22323|c1_g1_i6	No	(TCA)7ctgcgattcagcaagaacatctctttctctttc(TCT)6	72	--	--	--	--	--	--	TGAAACAGCCTTTTGTTCCC	60	CACATTTTTGGGGAAGCCTA	60	183	--	--	--	--	--	--
TR22327|c0_g2_i1	XLOC_005192	(CT)8tagtttcaactttgaaaaattgaaacaattgaagtttaatacttcaatatgattttgtagtggaaacaattattttttt(TC)7	110	--	--	--	--	--	--	GACAGAAACAGCAACGTCAGA	59	TGTTCCCAGCAGATAAAACAAA	60	233	--	--	--	--	--	--
TR22327|c1_g1_i1	XLOC_005193	(AG)10	20	--	--	--	--	--	--	CGAGGAGGTCTGAGAGATGG	60	TCGAAAACTCGAAACCTTCC	59	209	--	--	--	--	--	--
TR22327|c2_g1_i1	No	(AG)11	22	TR22327|c2_g1_i1	(AG)11	Ca_LG_6	(AG)8	7483592	7483607	TGGAGGGATGAACTGAAAGG	60	CCGGTGATGTTTGTGAATTG	60	269	Ca_09779	URR	--	--	--	--
TR22327|c2_g1_i2	XLOC_005194	(AG)11	22	--	--	--	--	--	--	TGGAGGGATGAACTGAAAGG	60	CCGGTGATGTTTGTGAATTG	60	269	--	--	XM_004503871.1	Nin-like	[Q]	--
TR22327|c2_g2_i1	XLOC_005195	(TGT)6	18	--	--	--	--	--	--	TGATGATGTTGTTGGTGTTGC	60	ACACCTACGCAAAGGAAAGC	59	269	--	--	--	--	--	--
TR22330|c0_g1_i1	No	(CCA)5	15	TR22330|c0_g1_i1	(CAC)6	Ca_LG_7	(CAC)5	36882594	36882608	ACACTTTCCCTTTTGCCCTT	60	GTGTGGAGGATTGAGACCGT	60	214	Ca_18518	URR	--	--	--	--
TR22330|c0_g1_i1	No	(CAC)6	18	TR22330|c0_g1_i1	(CAC)6	Ca_LG_7	(CAC)5	36882594	36882608	ACGGTCTCAATCCTCCACAC	60	ACATCATGAACAACGCCAAA	60	187	Ca_18518	URR	--	--	--	--
TR22330|c0_g3_i1	No	(TA)11	22	--	--	--	--	--	--	GCTCTCCCTAGCCGAGTTTT	60	AAATCAAACTTGTGGCTAATTCAA	59	119	--	--	--	--	--	--
TR22330|c0_g4_i1	No	(CCA)5	15	--	--	--	--	--	--	ACACTTTCCCTTTTGCCCTT	60	GTGTGGAGGATTGAGACCGT	60	214	--	--	--	--	--	--
TR22330|c0_g4_i1	No	(CAC)6	18	--	--	--	--	--	--	ACGGTCTCAATCCTCCACAC	60	ACATCATGAACAACGCCAAA	60	187	--	--	--	--	--	--

Transcript IDs	Differentially expressed gene (DEG) IDs	SSR repeat-motifs	SSR repeat-length (bp)	*In silico* polymorphic SSRs						Forward primers (5'-3')	Annealing tempearture(0C)	Reverse primers (5'-3')	Annealing tempearture(0C)	Amplified product size (bp)	Structural annotation		Functional Annotation			
				C. microphyllum		ICC4958														
				Transcript	SSR repeat-motifs	Chromosomes	SSR repeat-motifs	Start physical positions (bp)	End physical positions (bp)						Gene IDs	Sequence component of genes	NCBI nr IDs	TFs	KOG	KEGG
TR22330|c0_g4_i2	No	(CCA)5	15	--	--	--	--	--	--	ACACTTTCCCTTTTGCCCTT	60	GTGTGGAGGATTGAGACCGT	60	214	--	--	--	--	--	--
TR22330|c0_g4_i2	No	(CAC)6	18	--	--	--	--	--	--	ACGGTCTCAATCCTCCACAC	60	ACATCATGAACAACGCCAAA	60	187	--	--	--	--	--	--
TR22330|c0_g4_i3	No	(CCA)5	15	--	--	--	--	--	--	ACACTTTCCCTTTTGCCCTT	60	GTGTGGAGGATTGAGACCGT	60	214	--	--	--	--	--	--
TR22330|c0_g4_i3	No	(CAC)6	18	--	--	--	--	--	--	ACGGTCTCAATCCTCCACAC	60	ACATCATGAACAACGCCAAA	60	187	--	--	--	--	--	--
TR22330|c0_g4_i4	No	(CCA)5	15	--	--	--	--	--	--	ACACTTTCCCTTTTGCCCTT	60	GTGTGGAGGATTGAGACCGT	60	214	--	--	--	--	--	--
TR22330|c0_g4_i4	No	(CAC)6	18	--	--	--	--	--	--	ACGGTCTCAATCCTCCACAC	60	ACATCATGAACAACGCCAAA	60	187	--	--	--	--	--	--
TR22330|c0_g4_i5	No	(CCA)5	15	--	--	--	--	--	--	ACACTTTCCCTTTTGCCCTT	60	GTGTGGAGGATTGAGACCGT	60	214	--	--	--	--	--	--
TR22330|c0_g4_i5	No	(CAC)6	18	--	--	--	--	--	--	ACGGTCTCAATCCTCCACAC	60	ACATCATGAACAACGCCAAA	60	187	--	--	--	--	--	--
TR22333|c0_g1_i17	No	(AG)6atatttagtaacagttactagaagagaaggg(AAT)6	61	--	--	--	--	--	--	CATTGCTAAGTTGCCCCAAT	60	TGCAGTTTTCACTTTCACTCTTC	59	239	--	--	--	--	--	--
TR22342|c0_g3_i1	No	(ATA)5	15	--	--	--	--	--	--	CATCGTTCGTTCGGATATCAA	61	TTTCGCTTTCGCTCTGATTT	60	263	--	--	--	--	--	--
TR22342|c0_g3_i2	No	(ATA)5	15	--	--	--	--	--	--	CATCGTTCGTTCGGATATCAA	61	TTTCGCTTTCGCTCTGATTT	60	263	--	--	--	--	--	--
TR22342|c0_g4_i1	No	(ATA)5	15	--	--	--	--	--	--	CATCGTTCGTTCGGATATCAA	61	TTTCGCTTTCGCTCTGATTT	60	263	--	--	--	--	--	--
TR22342|c0_g4_i2	No	(ATA)5	15	--	--	--	--	--	--	CATCGTTCGTTCGGATATCAA	61	TTTCGCTTTCGCTCTGATTT	60	263	--	--	--	--	--	--
TR22342|c0_g4_i3	XLOC_005222	(ATA)5	15	--	--	--	--	--	--	CATCGTTCGTTCGGATATCAA	61	TTTCGCTTTCGCTCTGATTT	60	263	--	--	--	--	--	--

Transcript IDs	Differentially expressed gene (DEG) IDs	SSR repeat-motifs	SSR repeat-length (bp)	*In silico* polymorphic SSRs						Forward primers (5'-3')	Annealing tempearture(0C)	Reverse primers (5'-3')	Annealing tempearture(0C)	Amplified product size (bp)	Structural annotation		Functional Annotation			
				C. microphyllum		ICC4958									Gene IDs	Sequence component of genes	NCBI nr IDs	TFs	KOG	KEGG
				Transcript	SSR repeat-motifs	Chromosomes	SSR repeat-motifs	Start physical positions (bp)	End physical positions (bp)											
TR22342|c0_g4_i5	No	(ATA)5	15	--	--	--	--	--	--	CATCGTTCGTTCGGATATCAA	61	TTTCGCTTTCGCTCTGATTT	60	263	--	--	--	--	--	--
TR22344|c0_g1_i2	XLOC_005227	(AAT)5	15	--	--	--	--	--	--	TGAAGAAACTTCCTTTTGGTCC	60	AGAAGGGAGCAGTTTCATCG	59	195	--	--	--	--	--	--
TR22344|c1_g1_i1	No	(AAT)5aaaaaattctgctacccttttcca(CT)7tcaccgccactcccttctccctacttctgtaccttccttatttctctccaaacaaat(A)10	120	--	--	--	--	--	--	TGATGGACTCCTAAGAATATCTAACAA	59	CTCCCAATGAAACCATTGCT	60	237	--	--	--	--	--	--
TR22344|c1_g1_i2	No	(AAT)5aaaaaattctgctacccttttcca(CT)7tcaccgccactcccttctccctacttctgtaccttccttatttctctccaaacaaat(A)10	120	--	--	--	--	--	--	TGATGGACTCCTAAGAATATCTAACAA	59	CTCCCAATGAAACCATTGCT	60	237	--	--	--	--	--	--
TR22344|c1_g2_i1	XLOC_005228	(AAT)5aaaaaattctgctacccttttcca(CT)7	53	--	--	--	--	--	--	TGATGGACTCCTAAGAATATCTAACAA	59	CTCCCAATGAAACCATTGCT	60	236	--	--	--	--	--	--
TR22345|c0_g2_i1	No	(T)13gcaactaaaaaaacacattttcatacct(A)10	51	--	--	--	--	--	--	TCCATCAACAAACCAAACCA	60	AGAAAAATCAGGCAACACCG	60	144	--	--	--	--	--	--
TR22345|c0_g2_i2	No	(T)13gcaactaaaaaaacacattttcatacct(A)10	51	--	--	--	--	--	--	TCCATCAACAAACCAAACCA	60	AGAAAAATCAGGCAACACCG	60	144	--	--	--	--	--	--
TR22345|c0_g3_i1	XLOC_005232	(AG)7	14	--	--	--	--	--	--	CACCGATAACAACCATGCAC	60	AAGAACGGTTACGGTGATGTG	60	258	--	--	--	--	--	--
TR22345|c1_g1_i3	XLOC_005234	(A)10ttgccattgagagaag(A)10	36	--	--	--	--	--	--	CATTTCCTCCTTTTCTTTGTTCA	60	TTGATCTGCCATTGGTGTTG	61	270	--	--	XP_004495403.1	--	[I]	K15397
TR22351|c0_g2_i1	No	(TC)10ttcctaga(TCT)5	43	--	--	--	--	--	--	GGAAGTGAAGTGAGCACGC	60	TGCCATTGTTGGAGTTGAAA	60	236	--	--	--	--	--	--
TR22351|c0_g2_i2	No	(TC)10ttcctaga(TCT)5	43	--	--	--	--	--	--	GGAAGTGAAGTGAGCACGC	60	TGCCATTGTTGGAGTTGAAA	60	216	--	--	--	--	--	--
TR22352|c0_g1_i1	XLOC_005261	(T)10aaaaaaaattgaacatgtaaattaccatgttaggttttcaacc(AT)7	68	--	--	--	--	--	--	TTGACTCACATTTCACAAGATCG	60	AAGCATGCATTGATTGTTTTCT	59	277	--	--	--	--	--	--
TR22352|c2_g1_i1	No	(ATA)7	21	--	--	--	--	--	--	CAACTTTTATTGATAGGGTGTCCA	59	CCAACCCCAATGTCTTTGTC	60	146	--	--	--	--	--	--
TR22374|c0_g1_i3	No	(AGA)6	18	--	--	--	--	--	--	ATGCAATCTAAGTGGGCTGG	60	GGCAGGAACAGATCCTTGAA	60	265	--	--	--	--	--	--

Transcript IDs	Differentially expressed gene (DEG) IDs	SSR repeat-motifs	SSR repeat-length (bp)	*In silico* polymorphic SSRs						Forward primers (5'-3')	Annealing tempearture(0C)	Reverse primers (5'-3')	Annealing tempearture(0C)	Amplified product size (bp)	Structural annotation		Functional Annotation			
				C. microphyllum		ICC4958									Gene IDs	Sequence component of genes	NCBI nr IDs	TFs	KOG	KEGG
				Transcript	SSR repeat-motifs	Chromosomes	SSR repeat-motifs	Start physical positions (bp)	End physical positions (bp)											
TR22374|c0_g1_i6	No	(AGA)6	18	--	--	--	--	--	--	ATGCAATCTAAGTGGGCTGG	60	GGCAGGAACAGATCCTTGAA	60	265	--	--	--	--	--	--
TR22379|c0_g1_i1	XLOC_005300	(CA)11	22	--	--	--	--	--	--	GAAAATTGTCCACCTTATATTAATCG	58	CGAAACGAACGGTTGAGATT	60	240	--	--	--	--	--	--
TR22400|c0_g1_i1	No	(AAG)7	21	--	--	--	--	--	--	ATGGCGGTCTCGACCTGA	63	GTCCTCCTTCTCGAGAGCCT	60	104	--	--	--	--	--	--
TR22400|c0_g2_i1	No	(AAG)7	21	--	--	--	--	--	--	ATGGCGGTCTCGACCTGA	63	GTCCTCCTTCTCGAGAGCCT	60	104	--	--	--	--	--	--
TR22418|c0_g1_i1	XLOC_005315	(TCC)5	15	--	--	--	--	--	--	TCAGGACCAGTCACATCGAA	60	ATGCGTTTTCTGAATTTGGC	60	266	--	--	KDN46416.1	G2-like	[R]	K12741
TR22468|c0_g1_i1	No	(TCT)5	15	--	--	--	--	--	--	TGGGTTCTGTTTCACATCCA	60	CCACACACACACACAGACTGA	59	241	--	--	--	--	--	--
TR22468|c0_g1_i1	No	(TG)7	14	--	--	--	--	--	--	TGTGAACTGGGTTCTGTTTCA	59	TCAACATAAAAATCATGTGGAAGTG	60	280	--	--	--	--	--	--
TR22480|c1_g1_i1	XLOC_005342	(TA)6	12	--	--	--	--	--	--	CGTCTATAGTGCCATTCCCC	59	CACTACAAATTGTTTCCATTGTGA	59	131	--	--	XP_001948954.1	--	--	--
TR22487|c0_g1_i1	XLOC_005344	(CT)8	16	--	--	--	--	--	--	CCGTGTAGATGCCAAAGGTT	60	GCGCACAGCAGAGTTGATAA	60	213	--	--	ELU41493.1	--	--	--
TR22533|c0_g1_i1	No	(CA)7	14	--	--	--	--	--	--	GGCATTTGGCTATTGGCTTA	60	GCTTCTTTCGCTCTGCAAAT	60	240	--	--	--	--	--	--
TR22588|c0_g1_i1	XLOC_005372	(GA)9	18	--	--	--	--	--	--	AAAAGGCACAAAGAAAGGCA	60	AACACGCAATATGCCAACAA	60	117	--	--	--	--	--	--
TR22685|c0_g1_i1	No	(CAG)10	30	--	--	--	--	--	--	GGTCCTAGGCATTGAAGCTG	60	CACAGTTGAGAAGGCTGCAA	60	225	--	--	--	--	--	--
TR22797|c0_g1_i1	No	(GAA)5	15	--	--	--	--	--	--	TGCAAGAGAAAGAATTGAAAAGG	60	AACAGAGGAATCGTCATCGG	60	127	--	--	--	--	--	--
TR2288|c0_g1_i1	XLOC_005407	(TTG)5	15	--	--	--	--	--	--	GCCCACCAATCAGCAACTAT	60	GCTCTGTCGAAATCTAAAAACGA	60	248	--	--	--	--	--	--

<table>
<tr><th rowspan="3">Transcript IDs</th><th rowspan="3">Differentially expressed gene (DEG) IDs</th><th rowspan="3">SSR repeat-motifs</th><th rowspan="3">SSR repeat-length (bp)</th><th colspan="6">In silico polymorphic SSRs</th><th rowspan="3">Forward primers (5'-3')</th><th rowspan="3">Annealing tempearture(0C)</th><th rowspan="3">Reverse primers (5'-3')</th><th rowspan="3">Annealing tempearture(0C)</th><th rowspan="3">Amplified product size (bp)</th><th colspan="2">Structural annotation</th><th colspan="4">Functional Annotation</th></tr>
<tr><th colspan="2">C. microphyllum</th><th colspan="4">ICC4958</th><th rowspan="2">Gene IDs</th><th rowspan="2">Sequence component of genes</th><th rowspan="2">NCBI nr IDs</th><th rowspan="2">TFs</th><th rowspan="2">KOG</th><th rowspan="2">KEGG</th></tr>
<tr><th>Transcript</th><th>SSR repeat-motifs</th><th>Chromosomes</th><th>SSR repeat-motifs</th><th>Start physical positions (bp)</th><th>End physical positions (bp)</th></tr>
<tr><td>TR23006|c0_g1_i1</td><td>XLOC_005434</td><td>(CTT)5</td><td>15</td><td>--</td><td>--</td><td>--</td><td>--</td><td>--</td><td>--</td><td>TCCAACAAGACCAACCTTCC</td><td>60</td><td>CCTTTGAAAACTCAGCAGCA</td><td>59</td><td>270</td><td>--</td><td>--</td><td>--</td><td>--</td><td>--</td><td>--</td></tr>
<tr><td>TR23006|c0_g2_i1</td><td>No</td><td>(CTT)5</td><td>15</td><td>--</td><td>--</td><td>--</td><td>--</td><td>--</td><td>--</td><td>TCCAACAAGACCAACCTTCC</td><td>60</td><td>CCTTTGAAAACTCAGCAGCA</td><td>59</td><td>270</td><td>--</td><td>--</td><td>--</td><td>--</td><td>--</td><td>--</td></tr>
<tr><td>TR23037|c0_g1_i1</td><td>No</td><td>(CAT)6</td><td>18</td><td>--</td><td>--</td><td>--</td><td>--</td><td>--</td><td>--</td><td>TCCTTTCTTCCCTTTATTCATTT</td><td>57</td><td>CCTTCACCAGCTGCTAGACC</td><td>60</td><td>145</td><td>--</td><td>--</td><td>--</td><td>--</td><td>--</td><td>--</td></tr>
<tr><td>TR23037|c0_g2_i1</td><td>No</td><td>(CAT)6</td><td>18</td><td>--</td><td>--</td><td>--</td><td>--</td><td>--</td><td>--</td><td>TCCTTTCTTCCCTTTATTCATTT</td><td>57</td><td>CCTTCACCAGCTGCTAGACC</td><td>60</td><td>145</td><td>--</td><td>--</td><td>--</td><td>--</td><td>--</td><td>--</td></tr>
<tr><td>TR23051|c0_g1_i1</td><td>XLOC_005459</td><td>(T)10gtttgcttagaacaaacatacatatagaaaa(AG)6 tt(AG)6</td><td>67</td><td>--</td><td>--</td><td>--</td><td>--</td><td>--</td><td>--</td><td>AAAACCACTTGCTTCATTGATTT</td><td>59</td><td>ACATGGAGCTGAGCCTTGTC</td><td>60</td><td>221</td><td>--</td><td>--</td><td>--</td><td>--</td><td>--</td><td>--</td></tr>
<tr><td>TR23114|c0_g1_i1</td><td>No</td><td>(CAA)5</td><td>15</td><td>--</td><td>--</td><td>--</td><td>--</td><td>--</td><td>--</td><td>ATGAATCTTCTCGCCGTCAC</td><td>60</td><td>GCTGCTGCTGTTGTTGTTGT</td><td>60</td><td>114</td><td>--</td><td>--</td><td>--</td><td>--</td><td>--</td><td>--</td></tr>
<tr><td>TR2313|c0_g1_i1</td><td>XLOC_005491</td><td>(TAC)6</td><td>18</td><td>--</td><td>--</td><td>--</td><td>--</td><td>--</td><td>--</td><td>TGAGTTATGAAGACCATTAAATTTGG</td><td>60</td><td>GTTGCTTCCCATGGTCGTAT</td><td>60</td><td>246</td><td>--</td><td>--</td><td>--</td><td>--</td><td>--</td><td>--</td></tr>
<tr><td>TR2317|c0_g1_i1</td><td>No</td><td>(CA)6</td><td>12</td><td>--</td><td>--</td><td>--</td><td>--</td><td>--</td><td>--</td><td>CACAAATCACCCTCCTCCAC</td><td>60</td><td>GTTCGAACCTCGGATCATGT</td><td>60</td><td>185</td><td>--</td><td>--</td><td>--</td><td>--</td><td>--</td><td>--</td></tr>
<tr><td>TR2324|c0_g1_i1</td><td>XLOC_005518</td><td>(TC)6</td><td>12</td><td>--</td><td>--</td><td>--</td><td>--</td><td>--</td><td>--</td><td>CACCGTACATGCCAGACTACA</td><td>60</td><td>TTGACGGTGGAGCTTAGGTT</td><td>60</td><td>109</td><td>--</td><td>--</td><td>--</td><td>--</td><td>--</td><td>--</td></tr>
<tr><td>TR23260|c0_g1_i1</td><td>No</td><td>(CT)9</td><td>18</td><td>--</td><td>--</td><td>--</td><td>--</td><td>--</td><td>--</td><td>CTGCTCCGCTACTCTTCTTCA</td><td>60</td><td>CAGAGAGAGAAAGGAAGGTGGA</td><td>60</td><td>141</td><td>--</td><td>--</td><td>--</td><td>--</td><td>--</td><td>--</td></tr>
<tr><td>TR23296|c0_g1_i1</td><td>No</td><td>(GGT)5</td><td>15</td><td>--</td><td>--</td><td>--</td><td>--</td><td>--</td><td>--</td><td>TAGGCAGGTGGATTGGTTTC</td><td>60</td><td>CGGTCCAGTGATCAATAGTGTG</td><td>60</td><td>165</td><td>--</td><td>--</td><td>--</td><td>--</td><td>--</td><td>--</td></tr>
<tr><td>TR23355|c0_g1_i1</td><td>XLOC_005545</td><td>(GTT)5</td><td>15</td><td>--</td><td>--</td><td>--</td><td>--</td><td>--</td><td>--</td><td>TGGGTCCACAAGAGAAAACC</td><td>60</td><td>GGTTCATCTCGGAAACCCTT</td><td>60</td><td>211</td><td>--</td><td>--</td><td>--</td><td>--</td><td>--</td><td>--</td></tr>
<tr><td>TR2336|c0_g1_i1</td><td>No</td><td>(CA)10(TA)6</td><td>32</td><td>--</td><td>--</td><td>--</td><td>--</td><td>--</td><td>--</td><td>TCTTTTCGTTTCTAAGCCTTGC</td><td>60</td><td>TGCGAAGAAATTAATACCGGA</td><td>60</td><td>227</td><td>--</td><td>--</td><td>--</td><td>--</td><td>--</td><td>--</td></tr>
<tr><td>TR2336|c0_g2_i1</td><td>XLOC_005556</td><td>(CA)10(TA)6</td><td>32</td><td>--</td><td>--</td><td>--</td><td>--</td><td>--</td><td>--</td><td>TCTTTTCGTTTCTAAGCCTTGC</td><td>60</td><td>TCTCTGAAGTGGTTGCTGAAAA</td><td>60</td><td>189</td><td>--</td><td>--</td><td>--</td><td>--</td><td>--</td><td>--</td></tr>
</table>

Transcript IDs	Differentially expressed gene (DEG) IDs	SSR repeat-motifs	SSR repeat-length (bp)	*In silico* polymorphic SSRs						Forward primers (5'-3')	Annealing tempearture(0C)	Reverse primers (5'-3')	Annealing tempearture(0C)	Amplified product size (bp)	Structural annotation		Functional Annotation			
				C. microphyllum		ICC4958														
				Transcript	SSR repeat-motifs	Chromosomes	SSR repeat-motifs	Start physical positions (bp)	End physical positions (bp)						Gene IDs	Sequence component of genes	NCBI nr IDs	TFs	KOG	KEGG
TR2336|c0_g2_i2	XLOC_005557	(CA)10(TA)6	32	--	--	--	--	--	--	TCTTTTCGTTTCTAAGCCTTGC	60	TCTCTGAAGTGGTTGCTGAAAA	60	189	--	--	--	--	--	--
TR23377|c0_g1_i1	No	(CT)7	14	TR23377|c0_g1_i1	(CT)7	Ca_LG_4	(CT)11	32299674	32299695	TGCATTGGAAATACAGCATGA	60	ATAATTTTGGTTCGCCCACA	60	153		URR	--	--	--	--
TR23377|c0_g2_i1	No	(CT)7	14	--	--	--	--	--	--	TGCATTGGAAATACAGCATGA	60	ATAATTTTGGTTCGCCCACA	60	153	--	--	--	--	--	--
TR23387|c0_g1_i1	No	(TGC)5	15	--	--	--	--	--	--	CCTGATGAGGAAGAAGACGC	60	TAAGCATGCCGAGCTTGTAA	60	145	--	--	--	--	--	--
TR23411|c0_g1_i1	No	(GA)6aagagaggttgtgttgttcttgttgttgttgtttgtttgtgtgt(AG)7	70	--	--	--	--	--	--	AGGGTTCTGTTTGGTTTGGA	59	GTTTGTGAGGCTTTGCTGCT	61	181	--	--	--	--	--	--
TR2348|c0_g2_i1	No	(TTG)5	15	--	--	--	--	--	--	TTGCCTTCTGCATTGAAGTG	60	CCTTCCCAATCTCAACCAAA	60	156	--	--	--	--	--	--
TR2352|c0_g1_i1	No	(TC)11c(CT)10	43	--	--	--	--	--	--	CAATTTGGAAGTTCACTCTTTCA	58	CCATGTTTGCACTTTGACCA	61	276	--	--	--	--	--	--
TR2352|c0_g2_i1	No	(TC)11c(CT)10	43	--	--	--	--	--	--	CAATTTGGAAGTTCACTCTTTCA	58	CCATGTTTGCACTTTGACCA	61	277	--	--	--	--	--	--
TR23548|c0_g1_i1	No	(TCT)5	15	--	--	--	--	--	--	CTGGTCCAAATTGGCTTGTT	60	AAGGTTGAGGAAGTCACCGA	60	110	--	--	--	--	--	--
TR23559|c0_g1_i1	No	(TGC)5	15	--	--	--	--	--	--	GTTGGGATAGTAGGCTGCCA	60	GCACTGCACTGCCTTCATTA	60	217	--	--	--	--	--	--
TR23629|c0_g1_i1	XLOC_005616	(GAT)6	18	--	--	--	--	--	--	AATTGTTGGAGCTGGAGGTG	60	CACAGTACTCCTTAGACACATGCAC	60	199	--	--	--	--	--	--
TR23638|c0_g1_i1	No	(AT)6	12	--	--	--	--	--	--	CTATACAGCGGTGCAGCAAA	60	ATGGGTGTCAAGGGTCTCAC	60	231	--	--	--	--	--	--
TR2364|c0_g1_i1	XLOC_005625	(TC)10	20	--	--	--	--	--	--	AAGACCTTTTCCCCTTCTGC	60	GTGGAGTTTTTGGGGGATTT	60	154	--	--	--	--	--	--
TR23713|c3_g1_i1	No	(AAC)7	21	--	--	--	--	--	--	GCAAAGTTGGGTGGAAACTC	60	TGTGGTGTTAAGAATTGGCG	60	227	--	--	--	--	--	--

Transcript IDs	Differentially expressed gene (DEG) IDs	SSR repeat-motifs	SSR repeat-length (bp)	*In silico* polymorphic SSRs						Forward primers (5'-3')	Annealing tempearture(0C)	Reverse primers (5'-3')	Annealing tempearture(0C)	Amplified product size (bp)	Structural annotation		Functional Annotation			
				C. microphyllum		ICC4958														
				Transcript	SSR repeat-motifs	Chromosomes	SSR repeat-motifs	Start physical positions (bp)	End physical positions (bp)						Gene IDs	Sequence component of genes	NCBI nr IDs	TFs	KOG	KEGG
TR23713|c3_g1_i2	No	(AAC)7	21	--	--	--	--	--	--	GCAAAGTTGGGTGGAAACTC	60	TGTGGTGTTAAGAATTGGCG	60	227	--	--	--	--	--	--
TR23713|c4_g1_i1	XLOC_005667	(GCA)5	15	--	--	--	--	--	--	TCAAATCAACCAATTGCAACA	60	AGTTGGCTCGTCGGTATTTG	60	141	--	--	--	--	--	--
TR23713|c5_g1_i1	No	(ACT)5	15	--	--	--	--	--	--	TGCGTTGACGAATATCCACT	59	TCCACCTGTTTCTGTTAAGGC	59	228	--	--	--	--	--	--
TR23713|c5_g1_i2	No	(ACT)5	15	--	--	--	--	--	--	TGCGTTGACGAATATCCACT	59	TCCACCTGTTTCTGTTAAGGC	59	228	--	--	--	--	--	--
TR23713|c5_g1_i3	No	(ACT)5	15	--	--	--	--	--	--	TGCGTTGACGAATATCCACT	59	TCCACCTGTTTCTGTTAAGGC	59	228	--	--	--	--	--	--
TR23715|c3_g1_i1	XLOC_005670	(TTC)5	15	--	--	--	--	--	--	TATTCAGAAGCTGCGTTTCG	59	GAAACCTCGCCCTTATCCTC	60	266	--	--	XM_004510347.1	--	[R]	--
TR23715|c4_g2_i1	No	(TC)6	12	--	--	--	--	--	--	AGGGATAAAACGTCACACGC	60	GAAGACGAAGCGAGGTTGAC	60	140	--	--	--	--	--	--
TR23715|c4_g2_i2	No	(TC)6	12	--	--	--	--	--	--	AGGGATAAAACGTCACACGC	60	GAAGACGAAGCGAGGTTGAC	60	140	--	--	--	--	--	--
TR23729|c0_g1_i1	XLOC_005694	(AAT)6	18	--	--	--	--	--	--	CAATGATGACACATGATCGG	58	TTTTGAAGTGCAGAGACCCA	59	210	--	--	--	--	--	--
TR23731|c0_g2_i1	No	(TC)10	20	TR23731|c0_g2_i1	(TC)10	Ca_LG_3	(TC)63	27147037	27147162	CAAACGCCCACTTTTCTCTC	60	AACCTCTAACAACCGCATGG	60	207		URR	--	--	--	--
TR23731|c0_g2_i1	No	(CCA)6	18	TR23731|c0_g2_i1	(TC)10	Ca_LG_3	(TC)63	27147037	27147162	CCATGCGGTTGTTAGAGGTT	60	GAAGGTTCTTGAGGGTGCAG	60	111		URR	--	--	--	--
TR23731|c0_g2_i2	No	(TC)10	20	--	--	--	--	--	--	CAAACGCCCACTTTTCTCTC	60	AACCTCTAACAACCGCATGG	60	207	--	--	--	--	--	--
TR23731|c0_g2_i2	No	(CCA)6	18	--	--	--	--	--	--	CCATGCGGTTGTTAGAGGTT	60	GAAGGTTCTTGAGGGTGCAG	60	111	--	--	--	--	--	--
TR23743|c0_g1_i1	XLOC_005722	(AT)10	20	--	--	--	--	--	--	TGGGACAATTTGGTGAAGAAG	60	TCTGTTTCGTCGGTGAAGTG	60	177	--	--	--	--	--	--

Transcript IDs	Differentially expressed gene (DEG) IDs	SSR repeat-motifs	SSR repeat-length (bp)	*In silico* polymorphic SSRs						Forward primers (5'-3')	Annealing tempearture(0C)	Reverse primers (5'-3')	Annealing tempearture(0C)	Amplified product size (bp)	Structural annotation		Functional Annotation			
				C. microphyllum		ICC4958														
				Transcript	SSR repeat-motifs	Chromosomes	SSR repeat-motifs	Start physical positions (bp)	End physical positions (bp)						Gene IDs	Sequence component of genes	NCBI nr IDs	TFs	KOG	KEGG
TR23743|c0_g2_i1	No	(AT)10	20	--	--	--	--	--	--	TGGGACAATTTGGTGAAGAAG	60	TCTGTTTCGTCGGTGAAGTG	60	177	--	--	--	--	--	--
TR23744|c0_g2_i1	XLOC_005725	(TC)10	20	--	--	--	--	--	--	TTTCCATCGACACAATTCCA	60	TGCCATTTCGGATGAATGTA	60	186	--	--	--	--	--	--
TR23747|c0_g1_i1	No	(CA)6	12	--	--	--	--	--	--	CCCCTCATCTCTCGAAACAA	60	GACTGAACGCGCTGTTGTAA	60	242	--	--	--	--	--	--
TR23747|c0_g1_i2	No	(CA)6	12	--	--	--	--	--	--	CCCCTCATCTCTCGAAACAA	60	GACTGAACGCGCTGTTGTAA	60	242	--	--	--	--	--	--
TR23747|c0_g1_i3	No	(CA)6	12	--	--	--	--	--	--	CCCCTCATCTCTCGAAACAA	60	GACTGAACGCGCTGTTGTAA	60	242	--	--	--	--	--	--
TR23747|c0_g1_i8	No	(CA)6	12	--	--	--	--	--	--	CCCCTCATCTCTCGAAACAA	60	GACTGAACGCGCTGTTGTAA	60	242	--	--	--	--	--	--
TR23747|c0_g1_i9	No	(CA)6	12	--	--	--	--	--	--	CCCCTCATCTCTCGAAACAA	60	GACTGAACGCGCTGTTGTAA	60	242	--	--	--	--	--	--
TR23753|c2_g1_i1	XLOC_005740	(TC)6	12	--	--	--	--	--	--	TTATGAAGCGTGGGATAGGC	60	AACCCTAATTTGGTGGAGGG	60	210	--	--	--	--	--	--
TR23753|c2_g2_i1	XLOC_005741	(TC)8	16	--	--	--	--	--	--	CCTTCCCCTCCTTTAACTGC	60	CATGGCGGCTAAGAAGAAAG	60	230	--	--	--	--	--	--
TR23763|c0_g1_i1	XLOC_005748	(AG)7	14	--	--	--	--	--	--	TGATGGCGACAAGGGATTAT	60	ATGGATTTCAACCCCAATGA	60	227	--	--	XP_004501765.1	--	--	--
TR23765|c1_g1_i1	No	(TC)8	16	--	--	--	--	--	--	TTCTCAATTCTCATTCGCCA	59	CTTCTGCTTAGGGCATTTCG	60	237	--	--	--	--	--	--
TR23765|c1_g1_i2	No	(TC)8	16	--	--	--	--	--	--	TTCTCAATTCTCATTCGCCA	59	TCCTTGCAGAATCCATTTCC	60	186	--	--	--	--	--	--
TR23765|c1_g1_i3	No	(TC)8	16	--	--	--	--	--	--	TTCTCAATTCTCATTCGCCA	59	CTTCTGCTTAGGGCATTTCG	60	236	--	--	--	--	--	--
TR23765|c2_g1_i1	XLOC_005754	(A)14ccagctatccaaaaccagaagttttcagggacttgg(T)10ccaaaaccaaactaacaaagaatgtagc(A)12	100	--	--	--	--	--	--	CACAAGAAAATTTCCCAGTCAG	59	TGACCAAATGTTGATGACCG	60	157	--	--	--	--	--	--

Transcript IDs	Differentially expressed gene (DEG) IDs	SSR repeat-motifs	SSR repeat-length (bp)	*In silico* polymorphic SSRs						Forward primers (5'-3')	Annealing tempearture(0C)	Reverse primers (5'-3')	Annealing tempearture(0C)	Amplified product size (bp)	Structural annotation		Functional Annotation			
				C. microphyllum		ICC4958									Gene IDs	Sequence component of genes	NCBI nr IDs	TFs	KOG	KEGG
				Transcript	SSR repeat-motifs	Chromosomes	SSR repeat-motifs	Start physical positions (bp)	End physical positions (bp)											
TR23767|c1_g1_i1	No	(TTG)5	15	--	--	--	--	--	--	TGACCCTTCCATTTCCACTT	59	TACAAACGCGCTTTTCATCA	60	271	--	--	--	--	--	--
TR23767|c1_g3_i2	No	(TA)6	12	TR23767|c1_g3_i2	(TA)6	Ca_LG_1	(TA)8	9705937	9705952	ACTCGCCCTTTCCATAGCTT	60	GAAAACAGTTTGATTTGGCCTT	60	209	Ca_29341	CDS	--	--	--	--
TR23771|c1_g1_i3	No	(TA)8	16	TR23771|c1_g1_i3	(TA)8	Ca_LG_1	(TA)6	32241624	32241635	ATTTCTTCACCTCACGCCAC	60	GGACTTGAGATCTGCCATTGA	60	255	Ca_01277	DRR	--	--	--	--
TR23773|c1_g1_i1	No	(GAG)5	15	--	--	--	--	--	--	CACTGATATTCGGCAACACG	60	AAGCAGCAAAGAGGGCAGTA	60	243	--	--	--	--	--	--
TR23773|c1_g1_i1	No	(AC)6	12	--	--	--	--	--	--	GGAATTACCTCATCAATAGTGCTTT	59	GCTTTCACGATTGGTGGATT	60	105	--	--	--	--	--	--
TR23773|c1_g1_i5	No	(GAG)5	15	--	--	--	--	--	--	CACTGATATTCGGCAACACG	60	AAGCAGCAAAGAGGGCAGTA	60	243	--	--	--	--	--	--
TR23773|c1_g1_i5	No	(AC)6	12	--	--	--	--	--	--	GGAATTACCTCATCAATAGTGCTTT	59	GCTTTCACGATTGGTGGATT	60	105	--	--	--	--	--	--
TR23782|c9_g1_i1	XLOC_005790	(ATA)6	18	--	--	--	--	--	--	ATTTCCGGCTCTTGATTCCT	60	GAGCCACCAAAACCACAGAT	60	204	--	--	--	--	--	--
TR23792|c0_g2_i1	No	(GAT)5	15	--	--	--	--	--	--	ACGGTGATGGAACGATTGAT	60	CGTGAAACTTTAGCATGTTTTCC	60	233	--	--	--	--	--	--
TR23792|c3_g1_i1	No	(A)10g(A)11	22	--	--	--	--	--	--	TTCACTTCCGTGCATGAATC	60	CCGAATCAGAGGTGAACCAT	60	143	--	--	--	--	--	--
TR23792|c3_g1_i2	No	(A)10g(A)11	22	--	--	--	--	--	--	TTCACTTCCGTGCATGAATC	60	CCGAATCAGAGGTGAACCAT	60	143	--	--	--	--	--	--
TR23792|c3_g1_i3	No	(A)10g(A)11	22	--	--	--	--	--	--	TTCACTTCCGTGCATGAATC	60	CCGAATCAGAGGTGAACCAT	60	143	--	--	--	--	--	--
TR23792|c3_g1_i4	No	(A)10g(A)11	22	--	--	--	--	--	--	CCCTTGAGAATCCCTTGTGA	60	CCGAATCAGAGGTGAACCAT	60	228	--	--	--	--	--	--
TR23793|c6_g1_i4	No	(TAGT)6	24	--	--	--	--	--	--	GCGTTCCTAATGAGTCTCCC	59	TGATATCTCAACTAACTACACAATCCC	59	149	--	--	--	--	--	--

Transcript IDs	Differentially expressed gene (DEG) IDs	SSR repeat-motifs	SSR repeat-length (bp)	*In silico* polymorphic SSRs						Forward primers (5'-3')	Annealing tempearture(0C)	Reverse primers (5'-3')	Annealing tempearture(0C)	Amplified product size (bp)	Structural annotation		Functional Annotation			
				C. microphyllum		ICC4958									Gene IDs	Sequence component of genes	NCBI nr IDs	TFs	KOG	KEGG
				Transcript	SSR repeat-motifs	Chromosomes	SSR repeat-motifs	Start physical positions (bp)	End physical positions (bp)											
TR23793|c6_g1_i6	No	(TAGT)6	24	--	--	--	--	--	--	GCGTTCCTAATGAGTCTCCC	59	CTCTCCTCCAAGGGTTTTCC	60	218	--	--	--	--	--	--
TR23797|c0_g1_i1	No	(TC)12	24	--	--	--	--	--	--	CGCGAAAGGAAGGATCAATA	60	AAAATTGGATTGGGAAAGGG	60	161	--	--	--	--	--	--
TR23797|c0_g2_i1	No	(TC)12	24	--	--	--	--	--	--	CGCGAAAGGAAGGATCAATA	60	AAAATTGGATTGGGAAAGGG	60	161	--	--	--	--	--	--
TR23801|c1_g1_i1	No	(GGT)5ta(TGG)5	32	--	--	--	--	--	--	TTGCTGAAGGAGAGTCCGTT	60	TGCTACCACCATAACCACCA	60	255	--	--	--	--	--	--
TR23801|c1_g2_i1	XLOC_005825	(GGT)5ta(TGG)5	32	--	--	--	--	--	--	TTGCTGAAGGAGAGTCCGTT	60	GTATCCACCGCTCCTGCTAC	60	224	--	--	--	--	--	--
TR23802|c2_g2_i7	XLOC_005830	(AT)6	12	--	--	--	--	--	--	AAGAGCATGGAACAAAAGGAA	59	GACATCAAAGTGAGGCCCAT	60	244	--	--	--	--	--	--
TR23802|c2_g5_i1	No	(AT)6	12	--	--	--	--	--	--	AAGAGCATGGAACAAAAGGAA	59	GACATCAAAGTGAGGCCCAT	60	244	--	--	--	--	--	--
TR23804|c1_g1_i1	XLOC_005840	(TC)8	16	--	--	--	--	--	--	ATCGTTCACTCGCTCAACCT	60	CGAACACTTCTTCTCTCGCC	60	181	--	--	--	--	--	--
TR23804|c1_g1_i2	No	(TC)8	16	--	--	--	--	--	--	ATCGTTCACTCGCTCAACCT	60	CGAACACTTCTTCTCTCGCC	60	181	--	--	--	--	--	--
TR23804|c1_g1_i4	No	(TC)8	16	--	--	--	--	--	--	ATCGTTCACTCGCTCAACCT	60	CGAACACTTCTTCTCTCGCC	60	181	--	--	--	--	--	--
TR23807|c0_g1_i1	XLOC_005844	(AG)10	20	--	--	--	--	--	--	TCTGAACCCTTTCGGACAAC	60	CCCCCTTTCATTTCTTCGC	62	135	--	--	--	--	--	--
TR23807|c0_g2_i1	No	(AG)10	20	--	--	--	--	--	--	TCTGAACCCTTTCGGACAAC	60	CCCCCTTTCATTTCTTCGC	62	135	--	--	--	--	--	--
TR23808|c1_g1_i1	XLOC_005848	(TA)7	14	--	--	--	--	--	--	CCACTTTGCCTTGAAACCAG	61	GAGTTTGATGCGAGGGAGTC	60	177	--	--	--	--	--	--
TR23827|c0_g1_i1	XLOC_005855	(GAT)5	15	--	--	--	--	--	--	TCCATTGGAAAAAGAAGGCT	59	TACGTGTTCCAAGGGAGCTT	60	261	--	--	--	--	--	--

Transcript IDs	Differentially expressed gene (DEG) IDs	SSR repeat-motifs	SSR repeat-length (bp)	*In silico* polymorphic SSRs						Forward primers (5'-3')	Annealing tempearture(0C)	Reverse primers (5'-3')	Annealing tempearture(0C)	Amplified product size (bp)	Structural annotation		Functional Annotation			
				C. microphyllum		ICC4958									Gene IDs	Sequence component of genes	NCBI nr IDs	TFs	KOG	KEGG
				Transcript	SSR repeat-motifs	Chromosomes	SSR repeat-motifs	Start physical positions (bp)	End physical positions (bp)											
TR23889|c0_g1_i1	No	(ACC)5gccactccaaccaccatgaaccacctttactccgacacaagcacctcctccaactcaagtccggccacca(CTT)5	100	--	--	--	--	--	--	AAATCATGGCTAGCAGCTTCA	60	TTGAAGAGAAGGAACAGAAGGG	60	189	--	--	--	--	--	--
TR23905|c0_g1_i1	XLOC_005875	(TAG)5	15	--	--	--	--	--	--	GCCAGATTTTTAGGATGCCA	60	CCTTTGGTATTTGCAACAACAT	59	125	--	--	--	--	--	--
TR23991|c0_g1_i1	No	(CAA)5	15	--	--	--	--	--	--	AACCGATTTATCCGGACCTT	60	AGGAACCATGTTCTGATGCC	60	200	--	--	--	--	--	--
TR24018|c0_g1_i1	XLOC_005911	(ATC)5	15	--	--	--	--	--	--	ACCAGAGCGCTTTCACTTGT	60	TTCACCCGAATTTCACCTTC	60	239	--	--	--	--	--	--
TR24042|c0_g1_i1	No	(ATG)5	15	--	--	--	--	--	--	GCGTGAGTAACCAGAGCGAT	60	ACTGAACCTAAAACGCACGC	60	138	--	--	--	--	--	--
TR2406|c0_g1_i1	No	(A)11tggaggactaaatgggtgtttttatttcttatgaattaaatagatcttcata(T)10	73	--	--	--	--	--	--	TGCTTTAGTTTTGAAGAATGTTTTG	59	ATCCCTGTTATTTTTGGGCA	59	252	--	--	--	--	--	--
TR24064|c0_g1_i1	No	(CTG)5	15	--	--	--	--	--	--	ACCAGCTCTCTCAGCTCCTG	60	AGACGCAACAGCAGTCACAC	60	218	--	--	--	--	--	--
TR24161|c0_g1_i1	No	(AT)6	12	--	--	--	--	--	--	CCGAGTAATTAATGGGAGAAATTAG	59	TTGATTACTGCCAAGCCACA	60	127	--	--	--	--	--	--
TR24167|c0_g1_i1	XLOC_005949	(GAA)5	15	--	--	--	--	--	--	AAGCCCTTGATGTTGGTGTC	60	TGTGTTGTTCCTCTGTTTTACCTC	60	264	--	--	--	--	--	--
TR24194|c0_g1_i1	No	(TC)6	12	--	--	--	--	--	--	GTGAAACCACCAATATCGCC	60	AAAGGGGTTGAGGAGAGGAA	60	191	--	--	--	--	--	--
TR24194|c0_g2_i1	No	(TC)6	12	--	--	--	--	--	--	GTGAAACCACCAATATCGCC	60	AAAGGGGTTGAGGAGAGGAA	60	191	--	--	--	--	--	--
TR24223|c0_g1_i1	XLOC_005970	(GAA)7	21	--	--	--	--	--	--	GGCTTTTCGTCATCATCGTT	60	CTCCTACCCTCTCCAAACCC	60	220	--	--	--	--	--	--
TR24224|c0_g1_i1	No	(TGC)6	18	--	--	--	--	--	--	CATTGATGCTGGTGCTGAAT	60	CATCGATATCGCCTCGTTCT	60	278	--	--	--	--	--	--
TR24224|c0_g2_i1	XLOC_005971	(TGC)6	18	TR24224|c0_g2_i1	(TGC)6	Ca_LG_4	(TGC)5	10363679	10363693	CTTCATGCATCACATCAAACCT	60	CAGAAGAAGATGAAAATCTTCCAA	59	252	Ca_00299	DRR	--	--	--	--

Transcript IDs	Differentially expressed gene (DEG) IDs	SSR repeat-motifs	SSR repeat-length (bp)	*In silico* polymorphic SSRs						Forward primers (5'-3')	Annealing tempearture(0C)	Reverse primers (5'-3')	Annealing tempearture(0C)	Amplified product size (bp)	Structural annotation		Functional Annotation			
				C. microphyllum		ICC4958									Gene IDs	Sequence component of genes	NCBI nr IDs	TFs	KOG	KEGG
				Transcript	SSR repeat-motifs	Chromosomes	SSR repeat-motifs	Start physical positions (bp)	End physical positions (bp)											
TR24249|c0_g1_i1	No	(ACC)6	18	TR24249|c0_g1_i1	(ACC)6	Ca_LG_8	(ACC)8	2793317	2793340	GGCTAAATGTCCCAAAACGA	60	GAATTTGGGTGCAAAATTGG	60	146	Ca_20413	CDS	--	--	--	--
TR24249|c0_g2_i1	No	(ACC)6	18	--	--	--	--	--	--	GGCTAAATGTCCCAAAACGA	60	GAATTTGGGTGCAAAATTGG	60	147	--	--	--	--	--	--
TR24263|c0_g1_i1	No	(ACC)5	15	--	--	--	--	--	--	TACAATGACGGTTCGGTTGA	60	TTGGTGTTTTCTCCGGTAGG	60	184	--	--	--	--	--	--
TR24263|c0_g2_i1	No	(ACC)5	15	--	--	--	--	--	--	TACAATGACGGTTCGGTTGA	60	TTGGTGTTTTCTCCGGTAGG	60	184	--	--	--	--	--	--
TR24405|c0_g1_i1	XLOC_006042	(TTA)6	18	--	--	--	--	--	--	AGTTTCCATGGGCTCAGAAG	59	CCTTCCAATTGAAGAGAGCC	59	201	--	--	--	--	--	--
TR24672|c0_g1_i1	XLOC_006098	(TGT)6	18	--	--	--	--	--	--	CACTTGCCATTGCAATTTTC	59	AGTGATCTCACGTCACGCAT	59	213	--	--	--	--	--	--
TR24687|c0_g1_i1	No	(GA)6	12	--	--	--	--	--	--	TCATGAAAAAGGAGTGTTTGGA	60	TGCCCAAAAATGCATATGGT	61	133	--	--	--	--	--	--
TR24851|c0_g1_i2	No	(TC)6	12	TR24851|c0_g1_i2	(TC)6	Ca_LG_6	(TC)8	49482694	49482709	CCGAATTAACCGACCTTCAA	60	GCCGGTCATCATAGTCTTCG	61	246	Ca_12706	DRR	--	--	--	--
TR24851|c0_g1_i3	No	(TC)6	12	--	--	--	--	--	--	CCGAATTAACCGACCTTCAA	60	GCCGGTCATCATAGTCTTCG	61	246	--	--	--	--	--	--
TR24870|c0_g1_i1	No	(GA)6	12	--	--	--	--	--	--	AGGACTTGGCCATGACTTTG	60	TCTGGGAGATAGCACCATCG	61	221	--	--	--	--	--	--
TR24881|c0_g1_i1	XLOC_006138	(AAT)5	15	--	--	--	--	--	--	AATGGTCCTTTGTTTGCTGC	60	CGTCAACAATTCCACCAGAA	60	169	--	--	--	--	--	--
TR24977|c0_g1_i1	No	(AT)9	18	TR24977|c0_g1_i1	(AT)9	Ca_LG_8	(AT)22	4860101	4860144	TGCAATGGACAACACAAACA	60	TAAATGCTCCACTTCGGCTT	60	184	Ca_19249	DRR	--	--	--	--
TR24982|c0_g1_i1	No	(AAC)5	15	--	--	--	--	--	--	GTTCGCTCAATCCGAACAAT	60	GGCATCTACGTTTGGGAAAA	60	257	--	--	--	--	--	--
TR25028|c0_g1_i1	XLOC_006198	(AAG)5	15	--	--	--	--	--	--	ACAATGGCCAAGTCCAAGAC	60	CAGAGGAAGAGTCGGAGTCG	60	268	--	--	--	--	--	--

Transcript IDs	Differentially expressed gene (DEG) IDs	SSR repeat-motifs	SSR repeat-length (bp)	*In silico* polymorphic SSRs						Forward primers (5'-3')	Annealing tempearture(0C)	Reverse primers (5'-3')	Annealing tempearture(0C)	Amplified product size (bp)	Structural annotation		Functional Annotation			
				C. microphyllum		ICC4958									Gene IDs	Sequence component of genes	NCBI nr IDs	TFs	KOG	KEGG
				Transcript	SSR repeat-motifs	Chromosomes	SSR repeat-motifs	Start physical positions (bp)	End physical positions (bp)											
TR25109|c0_g1_i1	XLOC_006206	(TCA)6	18	--	--	--	--	--	--	ACCTGGTTTGCTGTTTCCAC	60	GAGCTTTACGCGATCTCTGC	60	179	--	--	--	--	--	--
TR25201|c0_g1_i1	No	(AAG)6	18	--	--	--	--	--	--	ATACCACAGCCAGACGGAAG	60	CGAATTTCATTAGCTTAACGGTG	60	244	--	--	--	--	--	--
TR25609|c0_g1_i1	XLOC_006352	(CAC)5	15	--	--	--	--	--	--	ATAATGCACTCGTCGCTCCT	60	GTATGAGGCGCTCTGACACA	60	174	--	--	XP_008898003.1	--	--	--
TR25614|c0_g1_i1	No	(CAG)5	15	--	--	--	--	--	--	GTCTTCAGGGAGGAAATCCC	60	TATTCTGCTGGTTCCGGTTC	60	105	--	--	--	--	--	--
TR25650|c0_g1_i1	XLOC_006367	(GCA)5	15	--	--	--	--	--	--	TGCAGCGATATCCTCAGATG	60	TTCCACATTCCGTGTTGAAA	60	184	--	--	XP_005821453.1	--	[K]	--
TR25693|c0_g1_i1	No	(TA)6	12	--	--	--	--	--	--	TCCCACAACCTCTGACAACA	60	ATTAAATATGACAACATCAACATG C	57	200	--	--	--	--	--	--
TR25767|c0_g1_i1	XLOC_006399	(CT)6	12	--	--	--	--	--	--	CCTCTGTTCTTTGCTGCCTC	60	TATCGTGCGTTCAAGGGATT	60	127	--	--	DAA34581.1	--	[R]	--
TR25779|c0_g1_i1	XLOC_006408	(ATT)6	18	--	--	--	--	--	--	GCAAATCAAAGGCACTGTAGG	60	TAAGACACCAAAATGCGACG	60	189	--	--	--	--	--	--
TR25790|c0_g2_i1	XLOC_006415	(ATT)5	15	--	--	--	--	--	--	TATGGCTGTCTCAGTGCCAA	60	CAACGAATGACACCCAAATG	60	278	--	--	XM_004512054.1	--	[OR]	--
TR25798|c0_g1_i1	No	(GA)6	12	--	--	--	--	--	--	CCCCGGAAATATAGTGCTGA	60	ACATGCAAACACACGGACAT	60	187	--	--	--	--	--	--
TR25798|c0_g2_i1	XLOC_006420	(CA)7	14	--	--	--	--	--	--	TGGCATTGATCTCTCTCTCATT	59	TAGTTGTTGTTGCTGGTCCG	60	280	--	--	XP_004506104.1	YABBY	[X]	--
TR25847|c0_g1_i1	No	(TA)8	16	--	--	--	--	--	--	TCTGTGGTTGAATATTGACGG	58	CCCTGATACGCTTTCATTTTG	60	175	--	--	--	--	--	--
TR25847|c0_g1_i1	No	(AT)10	20	--	--	--	--	--	--	TGAAAGCGTATCAGGGAAAGA	60	GAGTCATGCATACCGCTCAA	60	226	--	--	--	--	--	--
TR25847|c0_g1_i5	No	(TA)8	16	--	--	--	--	--	--	TCTGTGGTTGAATATTGACGG	58	CCCTGATACGCTTTCATTTTG	60	175	--	--	--	--	--	--

Transcript IDs	Differentially expressed gene (DEG) IDs	SSR repeat-motifs	SSR repeat-length (bp)	*In silico* polymorphic SSRs						Forward primers (5'-3')	Annealing tempearture(0C)	Reverse primers (5'-3')	Annealing tempearture(0C)	Amplified product size (bp)	Structural annotation		Functional Annotation			
				C. microphyllum		ICC4958									Gene IDs	Sequence component of genes	NCBI nr IDs	TFs	KOG	KEGG
				Transcript	SSR repeat-motifs	Chromosomes	SSR repeat-motifs	Start physical positions (bp)	End physical positions (bp)											
TR25847|c0_g1_i5	No	(AT)10	20	--	--	--	--	--	--	TGAAAGCGTATCAGGGAAAGA	60	GAGTCATGCATACCGCTCAA	60	226	--	--	--	--	--	--
TR25847|c0_g1_i6	XLOC_006448	(TA)8	16	--	--	--	--	--	--	TCTGTGGTTGAATATTGACGG	58	CCCTGATACGCTTTCATTTTG	60	175	--	--	--	--	--	--
TR25847|c0_g1_i6	XLOC_006448	(AT)10	20	--	--	--	--	--	--	TGAAAGCGTATCAGGGAAAGA	60	GAGTCATGCATACCGCTCAA	60	226	--	--	--	--	--	--
TR25854|c1_g1_i1	XLOC_006456	(ATC)5	15	--	--	--	--	--	--	AAGTTGGGGGATTTTAGGGA	60	TCTCATCACCCCATTTTCTG	58	175	--	--	--	--	--	--
TR25855|c0_g1_i1	No	(TCAC)5	20	--	--	--	--	--	--	ACTCACTCACCCCCTTCCTT	60	TAAGGGGTTTGTCGAGGATG	60	200	--	--	--	--	--	--
TR25855|c0_g1_i1	No	(CAC)6	18	--	--	--	--	--	--	CATCCTCGACAAACCCCTTA	60	TTCAAATTCCCTTGTGGAGG	60	246	--	--	--	--	--	--
TR25855|c0_g1_i2	No	(TCAC)5	20	--	--	--	--	--	--	ACTCACTCACCCCCTTCCTT	60	TAAGGGGTTTGTCGAGGATG	60	200	--	--	--	--	--	--
TR25855|c0_g1_i2	No	(CAC)6	18	--	--	--	--	--	--	CATCCTCGACAAACCCCTTA	60	TTCAAATTCCCTTGTGGAGG	60	246	--	--	--	--	--	--
TR25855|c0_g1_i3	No	(TCAC)5	20	--	--	--	--	--	--	ACTCACTCACCCCCTTCCTT	60	TAAGGGGTTTGTCGAGGATG	60	200	--	--	--	--	--	--
TR25855|c0_g1_i3	No	(CAC)6	18	--	--	--	--	--	--	CATCCTCGACAAACCCCTTA	60	TTCAAATTCCCTTGTGGAGG	60	246	--	--	--	--	--	--
TR25855|c0_g1_i4	No	(TCAC)5	20	--	--	--	--	--	--	ACTCACTCACCCCCTTCCTT	60	TAAGGGGTTTGTCGAGGATG	60	200	--	--	--	--	--	--
TR25855|c0_g1_i4	No	(CAC)6	18	--	--	--	--	--	--	CATCCTCGACAAACCCCTTA	60	TTCAAATTCCCTTGTGGAGG	60	246	--	--	--	--	--	--
TR25855|c0_g1_i5	No	(TCAC)5	20	--	--	--	--	--	--	ACTCACTCACCCCCTTCCTT	60	TAAGGGGTTTGTCGAGGATG	60	200	--	--	--	--	--	--
TR25855|c0_g1_i5	No	(CAC)6	18	--	--	--	--	--	--	CATCCTCGACAAACCCCTTA	60	TTCAAATTCCCTTGTGGAGG	60	246	--	--	--	--	--	--

Transcript IDs	Differentially expressed gene (DEG) IDs	SSR repeat-motifs	SSR repeat-length (bp)	*In silico* polymorphic SSRs						Forward primers (5'-3')	Annealing tempearture(0C)	Reverse primers (5'-3')	Annealing tempearture(0C)	Amplified product size (bp)	Structural annotation		Functional Annotation			
				C. microphyllum		ICC4958									Gene IDs	Sequence component of genes	NCBI nr IDs	TFs	KOG	KEGG
				Transcript	SSR repeat-motifs	Chromosomes	SSR repeat-motifs	Start physical positions (bp)	End physical positions (bp)											
TR25855|c0_g1_i6	No	(TCAC)5	20	--	--	--	--	--	--	ACTCACTCACCCCCTTCCTT	60	TAAGGGGTTTGTCGAGGATG	60	200	--	--	--	--	--	--
TR25855|c0_g1_i6	No	(CAC)6	18	--	--	--	--	--	--	CATCCTCGACAAACCCCTTA	60	TTCAAATTCCCTTGTGGAGG	60	246	--	--	--	--	--	--
TR25855|c0_g1_i7	No	(TCAC)5	20	--	--	--	--	--	--	ACTCACTCACCCCCTTCCTT	60	TAAGGGGTTTGTCGAGGATG	60	200	--	--	--	--	--	--
TR25855|c0_g1_i7	No	(CAC)6	18	--	--	--	--	--	--	CATCCTCGACAAACCCCTTA	60	TTCAAATTCCCTTGTGGAGG	60	246	--	--	--	--	--	--
TR25855|c0_g1_i8	No	(TCAC)5	20	--	--	--	--	--	--	ACTCACTCACCCCCTTCCTT	60	TAAGGGGTTTGTCGAGGATG	60	200	--	--	--	--	--	--
TR25855|c0_g1_i8	No	(CAC)6	18	--	--	--	--	--	--	CATCCTCGACAAACCCCTTA	60	TTCAAATTCCCTTGTGGAGG	60	246	--	--	--	--	--	--
TR25860|c0_g1_i1	No	(TGG)5	15	--	--	--	--	--	--	GTGGTTACAGAAGCGGTGGT	60	TCCTCCAGTTCCCATCAGAG	60	195	--	--	--	--	--	--
TR25860|c0_g2_i1	XLOC_006466	(AT)6ttagcccttcaaataaaaaattgccaaaggacccatatcaaaataaatactagtactatatgcaaataattttc(A)15tcacacac(A)12	120	--	--	--	--	--	--	ATATCCATGGGAGCAAGTCG	60	ACACGCTTCTTTCGTTTTGG	60	234	--	--	--	--	--	--
TR25860|c0_g3_i1	No	(AT)6ttagcccttcaaataaaaaattgccaaaggacccatatcaaaataaatactagtactatatgcaaataattttc(A)15tcacacac(A)12	120	--	--	--	--	--	--	ATATCCATGGGAGCAAGTCG	60	ACACGCTTCTTTCGTTTTGG	60	234	--	--	--	--	--	--
TR25864|c0_g1_i1	No	(AG)8	16	--	--	--	--	--	--	TGGGGAATTTATGCTTTGGA	60	AGATCTCCCCTTGTTGGCTT	60	244	--	--	--	--	--	--
TR25864|c0_g1_i2	XLOC_006469	(AG)8	16	--	--	--	--	--	--	TGGGGAATTTATGCTTTGGA	60	AGATCTCCCCTTGTTGGCTT	60	244	--	--	--	--	--	--
TR25882|c0_g1_i1	XLOC_006498	(CT)6	12	--	--	--	--	--	--	CACTCTCACTCACCCATTGC	59	TTGGGTTTTGATGGTTTTGA	59	278	--	--	--	--	--	--
TR25882|c1_g1_i1	XLOC_006499	(ACA)5	15	--	--	--	--	--	--	ACGTGTGGTTGGAAAACACC	61	AGCCAGAAAATCTTTTGCTCA	59	257	--	--	--	--	--	--
TR25903|c0_g1_i1	XLOC_006528	(CT)6	12	--	--	--	--	--	--	TCTCCCTCTCAGCACTACCC	59	TTTTTGGGTCACATGCAAAG	60	272	--	--	XM_004487732.1	C2H2	--	--

Transcript IDs	Differentially expressed gene (DEG) IDs	SSR repeat-motifs	SSR repeat-length (bp)	*In silico* polymorphic SSRs						Forward primers (5'-3')	Annealing tempearture(0C)	Reverse primers (5'-3')	Annealing tempearture(0C)	Amplified product size (bp)	Structural annotation		Functional Annotation			
				C. microphyllum		ICC4958														
				Transcript	SSR repeat-motifs	Chromosomes	SSR repeat-motifs	Start physical positions (bp)	End physical positions (bp)						Gene IDs	Sequence component of genes	NCBI nr IDs	TFs	KOG	KEGG
TR25925|c0_g4_i1	No	(CA)8	16	--	--	--	--	--	--	TGCACAATCTTAAGCTCCCA	59	GATGTGGGACCCGTAGTGAC	60	146	--	--	--	--	--	--
TR25925|c0_g4_i2	No	(CA)8	16	--	--	--	--	--	--	TGCACAATCTTAAGCTCCCA	59	GATGTGGGACCCGTAGTGAC	60	146	--	--	--	--	--	--
TR25934|c0_g2_i1	No	(TC)17	34	--	--	--	--	--	--	TCTCTAACTCCACAAACCCTAGA	57	CGGAGGTGTAGAAGGAGCTG	60	268	--	--	--	--	--	--
TR25934|c0_g2_i2	No	(TC)15	30	--	--	--	--	--	--	TCTCTAACTCCACAAACCCTAGA	57	CGGAGGTGTAGAAGGAGCTG	60	264	--	--	--	--	--	--
TR25949|c0_g1_i1	No	(CTT)5	15	--	--	--	--	--	--	CCACATCAGGTCCTTCTTCTG	60	GGGTAATTGTGGAAACCGAA	60	117	--	--	--	--	--	--
TR25949|c0_g1_i1	No	(TTC)5	15	--	--	--	--	--	--	TTCGGTTTCCACAATTACCC	60	CCACCACCAACAATGTTTCA	60	258	--	--	--	--	--	--
TR25949|c0_g2_i1	No	(CTT)5	15	--	--	--	--	--	--	CCACATCAGGTCCTTCTTCTG	60	GGGTAATTGTGGAAACCGAA	60	117	--	--	--	--	--	--
TR25949|c0_g2_i1	No	(TTC)5	15	--	--	--	--	--	--	TTCGGTTTCCACAATTACCC	60	CCACCACCAACAATGTTTCA	60	258	--	--	--	--	--	--
TR26000|c0_g1_i1	XLOC_006599	(TAAT)5	20	--	--	--	--	--	--	GGGTGTGGAGAGTTGGAAAA	60	AGGGTAGCGTGCCTCACTTA	60	205	--	--	--	--	--	--
TR26013|c0_g1_i1	No	(CA)6	12	--	--	--	--	--	--	CGATACGCAAGCAAAACTCA	60	TGGAATTAACCATGCGTGTG	60	146	--	--	--	--	--	--
TR26041|c0_g1_i1	No	(GAT)5	15	--	--	--	--	--	--	CAAACCAAAAGCAGCAGAAA	59	CTCATCAGCAGAATGACGGT	59	165	--	--	--	--	--	--
TR26101|c0_g1_i1	XLOC_006635	(CCG)5	15	--	--	--	--	--	--	AGACTTCTCGAACTTGGGCA	60	GAAACAACGGATACGGCAAC	60	176	--	--	XP_003044819.1	--	[A]	--
TR26123|c0_g1_i1	No	(TGG)5	15	--	--	--	--	--	--	CCATGTCCAGCATGAACAAC	60	AGGCTGTAAAACATGGGCAC	60	151	--	--	--	--	--	--
TR26172|c0_g1_i1	No	(TC)6	12	TR26172|c0_g1_i1	(TC)6	Ca_LG_1	(TC)12	38904084	38904107	TGTGACCCCAATACTCCAAA	59	GGTTTGATGCTACCCCAAAA	60	215	Ca_08976	DRR	--	--	--	--

Transcript IDs	Differentially expressed gene (DEG) IDs	SSR repeat-motifs	SSR repeat-length (bp)	*In silico* polymorphic SSRs						Forward primers (5'-3')	Annealing tempearture(0C)	Reverse primers (5'-3')	Annealing tempearture(0C)	Amplified product size (bp)	Structural annotation		Functional Annotation			
				C. microphyllum		ICC4958														
				Transcript	SSR repeat-motifs	Chromosomes	SSR repeat-motifs	Start physical positions (bp)	End physical positions (bp)						Gene IDs	Sequence component of genes	NCBI nr IDs	TFs	KOG	KEGG
TR26179|c0_g1_i1	XLOC_006663	(GAA)5	15	--	--	--	--	--	--	TCCATTTCATCAATGCAGGA	60	ACAGCATCACCCCAAAGTTC	60	137	--	--	XP_004492905.1	--	--	--
TR26329|c0_g1_i1	XLOC_006720	(TA)6	12	--	--	--	--	--	--	ATCATTCCCAACCCCAATCT	60	GCAGAACGGATGAGACCAGT	60	273	--	--	XM_004515508.1	--	[R]	--
TR26355|c0_g1_i1	XLOC_006735	(CAA)5	15	--	--	--	--	--	--	GACGACTCCGATCTCTCCAC	60	AATTCGTCGGTGGTGATTGT	60	191	--	--	--	--	--	--
TR26355|c0_g1_i2	No	(CAA)5	15	--	--	--	--	--	--	GACGACTCCGATCTCTCCAC	60	AATTCGTCGGTGGTGATTGT	60	191	--	--	--	--	--	--
TR26361|c0_g1_i1	No	(GTT)7	21	--	--	--	--	--	--	GGTGATGTTGGTGATGGTGA	60	TAAGGGCAGCTTTTGAGGAA	60	255	--	--	--	--	--	--
TR26361|c0_g2_i1	XLOC_006739	(GTT)7	21	--	--	--	--	--	--	GGTGATGTTGGTGATGGTGA	60	TAAGGGCAGCTTTTGAGGAA	60	255	--	--	--	--	--	--
TR26362|c0_g1_i1	XLOC_006740	(GAT)6	18	--	--	--	--	--	--	TCGAACCAGTTAAACCACCA	59	GGTGCACAATATCAATTCCCA	61	229	--	--	--	--	--	--
TR26365|c0_g1_i1	XLOC_006743	(TC)8	16	TR26365|c0_g1_i1	(TC)8	Ca_LG_1	(TC)7	29316800	29316813	TCAACAACTACACACAAATCCTCTC	60	CCGCCATTTCTGTAATTCGT	60	105		URR	--	--	--	--
TR26376|c0_g1_i1	XLOC_006751	(TGA)5	15	--	--	--	--	--	--	GAGTATGGTTATGAGCGTCCG	60	TCGTCATCGTAACTCTTGCG	60	152	--	--	--	--	--	--
TR26404|c0_g1_i1	XLOC_006781	(GGT)6	18	--	--	--	--	--	--	GACACCATTCTCGGCAATTT	60	CCCTCCGATATCATCTCGTC	59	177	--	--	--	--	--	--
TR2643|c0_g1_i1	XLOC_006815	(CTA)5	15	--	--	--	--	--	--	CCGCGAAAGACAACAACAAT	61	GCCGTTTGATATGCGTACCT	60	216	--	--	--	--	--	--
TR26439|c0_g2_i1	XLOC_006811	(AT)6	12	--	--	--	--	--	--	GGGTGCAGCTAGGTTAAAACA	59	ATATGCACCAACTTGAGGGG	60	269	--	--	--	--	--	--
TR26439|c0_g2_i4	No	(AT)6	12	--	--	--	--	--	--	CCATGTGTTTTAGGGTGCAG	59	ATATGCACCAACTTGAGGGG	60	280	--	--	--	--	--	--
TR26439|c0_g2_i5	No	(AT)6	12	--	--	--	--	--	--	GGTTAAAACAGGAGGGAAGGA	59	ATATGCACCAACTTGAGGGG	60	258	--	--	--	--	--	--

Transcript IDs	Differentially expressed gene (DEG) IDs	SSR repeat-motifs	SSR repeat-length (bp)	In silico polymorphic SSRs						Forward primers (5'-3')	Annealing tempearture(0C)	Reverse primers (5'-3')	Annealing tempearture(0C)	Amplified product size (bp)	Structural annotation		Functional Annotation			
				C. microphyllum		ICC4958														
				Transcript	SSR repeat-motifs	Chromosomes	SSR repeat-motifs	Start physical positions (bp)	End physical positions (bp)						Gene IDs	Sequence component of genes	NCBI nr IDs	TFs	KOG	KEGG
TR26439|c0_g2_i6	No	(AT)6	12	--	--	--	--	--	--	GGTTAAAACAGGAGGGAAGGA	59	ATATGCACCAACTTGAGGGG	60	258	--	--	--	--	--	--
TR26439|c0_g2_i7	No	(AT)6	12	--	--	--	--	--	--	CCATGTGTTTTAGGGTGCAG	59	ATATGCACCAACTTGAGGGG	60	280	--	--	--	--	--	--
TR26439|c0_g2_i8	No	(AT)6	12	--	--	--	--	--	--	GGGTGCAGCTAGGTTAAAACA	59	ATATGCACCAACTTGAGGGG	60	269	--	--	--	--	--	--
TR26439|c2_g1_i1	XLOC_006813	(GAA)6	18	--	--	--	--	--	--	CCTTTCGTAACCTTCCCACA	60	TCCTCTTGTTTCTTCTTCTTCCTC	60	269	--	--	--	--	--	--
TR26450|c0_g1_i1	XLOC_006848	(TGC)5	15	--	--	--	--	--	--	AATTCAGGAGCAAAACGGAA	60	TCGAAACAATGGAAGCAGTG	60	267	--	--	--	--	--	--
TR26456|c3_g1_i2	XLOC_006883	(TGA)5	15	--	--	--	--	--	--	TCAAGACATAGAGGCCATACTCA	59	ACACCACCCATGCCAACTAT	60	102	--	--	--	--	--	--
TR26457|c1_g6_i1	No	(TTA)5	15	--	--	--	--	--	--	AGACCCAACTCATGTTCCATT	58	TGACGAGTTAATGGGTGCAG	60	161	--	--	--	--	--	--
TR26457|c1_g8_i2	No	(TTA)5	15	--	--	--	--	--	--	AGACCCAACTCATGTTCCATT	58	AGGGGAACATCACACTCTGG	60	242	--	--	--	--	--	--
TR26461|c0_g3_i3	No	(AG)9	18	--	--	--	--	--	--	TCTGTGGCTCTTTACCGCTT	60	GCAACGGAGGGTTCAGATAC	60	200	--	--	--	--	--	--
TR26461|c0_g3_i4	No	(AG)9	18	--	--	--	--	--	--	TCTGTGGCTCTTTACCGCTT	60	GCAACGGAGGGTTCAGATAC	60	200	--	--	--	--	--	--
TR26480|c4_g4_i1	No	(T)11gaaatgaattaaattcaaaattcataagtgaaataaatacctttggataaaacctttcagtcgtaacaccacgacaaaagtc(AAT)5	108	--	--	--	--	--	--	CCAAAAATTGTAACAGCCCG	60	GCCGAGAATACCTTAGGGGT	59	266	--	--	--	--	--	--
TR26480|c4_g4_i2	No	(AAT)5	15	--	--	--	--	--	--	TCAGTCGTAACACCACGACAA	60	GCCGAGAATACCTTAGGGGT	59	159	--	--	--	--	--	--
TR26492|c0_g10_i1	No	(GAA)5	15	--	--	--	--	--	--	CCTCCTCCGTATCCATCATC	59	GCAGCAGAGAGCAGGAGAAT	60	242	--	--	--	--	--	--
TR26495|c1_g1_i1	No	(AT)7	14	--	--	--	--	--	--	TGTTTCTGCTCAAACACCAGA	59	AGGCAGTCTCCTGCAAGAAT	59	159	--	--	--	--	--	--

Transcript IDs	Differentially expressed gene (DEG) IDs	SSR repeat-motifs	SSR repeat-length (bp)	*In silico* polymorphic SSRs						Forward primers (5'-3')	Annealing tempearture(0C)	Reverse primers (5'-3')	Annealing tempearture(0C)	Amplified product size (bp)	Structural annotation		Functional Annotation			
				C. microphyllum		ICC4958									Gene IDs	Sequence component of genes	NCBI nr IDs	TFs	KOG	KEGG
				Transcript	SSR repeat-motifs	Chromosomes	SSR repeat-motifs	Start physical positions (bp)	End physical positions (bp)											
TR26495|c1_g1_i2	No	(AT)7	14	--	--	--	--	--	--	TGTTTCTGCTCAAACACCAGA	59	AGGCAGTCTCCTGCAAGAAT	59	159	--	--	--	--	--	--
TR26495|c1_g1_i3	No	(AT)8	16	--	--	--	--	--	--	GTTTCTGCCCAAAAACCAGA	60	AGGCAGTCTCCTGCAAGAAT	59	162	--	--	--	--	--	--
TR26495|c1_g1_i4	XLOC_007162	(AT)8	16	--	--	--	--	--	--	ACCCTTATTGTGCATGAGGC	60	AATACTTCTGGTCGGTGTGAA	57	192	--	--	--	--	--	--
TR26495|c1_g1_i5	No	(AT)7	14	--	--	--	--	--	--	TGTTTCTGCTCAAACACCAGA	59	AGGCAGTCTCCTGCAAGAAT	59	159	--	--	--	--	--	--
TR26495|c1_g1_i6	No	(AT)8	16	--	--	--	--	--	--	GTTTCTGCCCAAAAACCAGA	60	AGGCAGTCTCCTGCAAGAAT	59	162	--	--	--	--	--	--
TR26499|c1_g6_i1	XLOC_007216	(GA)10	20	--	--	--	--	--	--	GCAACAGTGCAACGAGGTAA	60	TATGCGAAGATTAGGTGGGG	60	139	--	--	--	--	--	--
TR26503|c4_g7_i1	No	(CTT)7	21	--	--	--	--	--	--	CTCACTCATTCAGATTGCCCT	59	CACTGGAAGAGACTCGGGAC	60	188	--	--	--	--	--	--
TR26503|c4_g7_i2	No	(CTT)7	21	--	--	--	--	--	--	CTCACTCATTCAGATTGCCCT	59	ACCAGATCTGAAATTGCGCT	60	176	--	--	--	--	--	--
TR26503|c4_g7_i4	XLOC_007258	(CTT)7	21	--	--	--	--	--	--	CTCACTCATTCAGATTGCCCT	59	CACTGGAAGAGACTCGGGAC	60	188	--	--	--	--	--	--
TR26506|c2_g1_i1	No	(TGA)8	24	--	--	--	--	--	--	TCATTGAGAGGTATCAGGAGCA	60	CCCCCACCATCTTCTGCTAT	61	163	--	--	--	--	--	--
TR26506|c2_g2_i1	XLOC_007269	(TGA)8	24	--	--	--	--	--	--	CCTTTGAGCATGCACAGCTA	60	CCCCCACCATCTTCTGCTAT	61	280	--	--	--	--	--	--
TR26506|c4_g1_i1	No	(CAA)7	21	--	--	--	--	--	--	CCCAAACAGAAAAATTCCCA	60	ACGGTTTCTCATTTTCGTCG	60	192	--	--	--	--	--	--
TR26506|c4_g2_i10	No	(CAA)7	21	--	--	--	--	--	--	CCCAAACAGAAAAATTCCCA	60	ACGGTTTCTCATTTTCGTCG	60	192	--	--	--	--	--	--
TR26506|c4_g2_i2	No	(CAA)7	21	--	--	--	--	--	--	CCCAAACAGAAAAATTCCCA	60	ACGGTTTCTCATTTTCGTCG	60	192	--	--	--	--	--	--

Transcript IDs	Differentially expressed gene (DEG) IDs	SSR repeat-motifs	SSR repeat-length (bp)	*In silico* polymorphic SSRs						Forward primers (5'-3')	Annealing tempearture(0C)	Reverse primers (5'-3')	Annealing tempearture(0C)	Amplified product size (bp)	Structural annotation		Functional Annotation			
				C. microphyllum		ICC4958														
				Transcript	SSR repeat-motifs	Chromosomes	SSR repeat-motifs	Start physical positions (bp)	End physical positions (bp)						Gene IDs	Sequence component of genes	NCBI nr IDs	TFs	KOG	KEGG
TR26506|c4_g2_i3	No	(CAA)7	21	--	--	--	--	--	--	CCCAAACAGAAAAATTCCCA	60	ACGGTTTCTCATTTTCGTCG	60	192	--	--	--	--	--	--
TR26506|c4_g2_i4	No	(CAA)7	21	--	--	--	--	--	--	CCCAAACAGAAAAATTCCCA	60	ACGGTTTCTCATTTTCGTCG	60	192	--	--	--	--	--	--
TR26506|c4_g2_i5	No	(CAA)7	21	--	--	--	--	--	--	CCCAAACAGAAAAATTCCCA	60	ACGGTTTCTCATTTTCGTCG	60	192	--	--	--	--	--	--
TR26506|c4_g2_i6	No	(CAA)7	21	--	--	--	--	--	--	CCCAAACAGAAAAATTCCCA	60	ACGGTTTCTCATTTTCGTCG	60	192	--	--	--	--	--	--
TR26506|c4_g2_i7	No	(CAA)7	21	--	--	--	--	--	--	CCCAAACAGAAAAATTCCCA	60	ACGGTTTCTCATTTTCGTCG	60	192	--	--	--	--	--	--
TR26506|c4_g2_i9	No	(CAA)7	21	--	--	--	--	--	--	CCCAAACAGAAAAATTCCCA	60	ACGGTTTCTCATTTTCGTCG	60	192	--	--	--	--	--	--
TR26506|c5_g1_i1	No	(TC)6	12	TR26506|c5_g1_i1	(CAC)5	Ca_LG_1	(CAC)7	39050734	39050754	TGGGTTTTCTTTCTCCAATCC	60	CCGCTTCCTTTTCAACTTCA	60	194	Ca_27432	CDS	--	--	--	--
TR26506|c5_g1_i3	XLOC_007270	(TC)6	12	--	--	--	--	--	--	TGGGTTTTCTTTCTCCAATCC	60	CCGCTTCCTTTTCAACTTCA	60	194	--	--	XR_190002.1	--	[OK]	K10592
TR26526|c0_g1_i1	No	(T)10gtagctaatgtcatacttttatctttgattgc(T)10	52	--	--	--	--	--	--	ATCATGTTGCCACTTTCAACT	57	GACAAAAACATAACAGCCTATCCA	59	134	--	--	--	--	--	--
TR26528|c0_g1_i2	XLOC_007429	(AT)7	14	--	--	--	--	--	--	TCAGGAACATCCATACACAACA	59	TCCCTATCCCCATCCATTTT	60	109	--	--	--	--	--	--
TR26528|c0_g1_i4	XLOC_007430	(AT)7	14	--	--	--	--	--	--	TCAGGAACATCCATACACAACA	59	TCCCTATCCCCATCCATTTT	60	109	--	--	--	--	--	--
TR26528|c0_g1_i5	No	(AT)7	14	--	--	--	--	--	--	TCAGGAACATCCATACACAACA	59	TCCCTATCCCCATCCATTTT	60	109	--	--	--	--	--	--
TR26530|c0_g2_i1	No	(TCT)6	18	--	--	--	--	--	--	CAATGGGTTGGTTGCTTCTT	60	GTCACACGTGACACCATTCC	60	230	--	--	--	--	--	--
TR26530|c0_g2_i8	No	(TCT)6	18	--	--	--	--	--	--	CAATGGGTTGGTTGCTTCTT	60	GTCACACGTGACACCATTCC	60	230	--	--	--	--	--	--

Transcript IDs	Differentially expressed gene (DEG) IDs	SSR repeat-motifs	SSR repeat-length (bp)	*In silico* polymorphic SSRs						Forward primers (5'-3')	Annealing tempearture(0C)	Reverse primers (5'-3')	Annealing tempearture(0C)	Amplified product size (bp)	Structural annotation		Functional Annotation			
				C. microphyllum		ICC4958														
				Transcript	SSR repeat-motifs	Chromosomes	SSR repeat-motifs	Start physical positions (bp)	End physical positions (bp)						Gene IDs	Sequence component of genes	NCBI nr IDs	TFs	KOG	KEGG
TR26547|c0_g2_i1	XLOC_007486	(TC)7	14	--	--	--	--	--	--	AGGGGTGAAAATAACTGCACA	59	AAGTAAAGGAGGGGATGGGA	60	160	--	--	--	--	--	--
TR26547|c0_g3_i1	No	(TC)7	14	--	--	--	--	--	--	AGGGGTGAAAATAACTGCACA	59	GGAAAAAGAGGGACACAGCC	61	179	--	--	--	--	--	--
TR26548|c0_g1_i1	XLOC_007487	(A)10caaataaaaaattgggtatttttgttccgttgcggcgtgtcctcctgcaagtggtaacactgacgagaaccca(AAT)7	105	--	--	--	--	--	--	AAGTCGATTTCGTCCTCCATT	60	GACAATGAAACGTGTGGCTG	60	277	--	--	--	--	--	--
TR26558|c0_g1_i1	No	(A)13ttaaggacggcttcaaaaactc(CT)6	47	--	--	--	--	--	--	TTGGAAACGCAAGGAAGATT	60	TGCTAAACTCTTCCATCTCAACA	59	278	--	--	--	--	--	--
TR26558|c0_g2_i1	No	(A)13ttaaggacggcttcaaaaactc(CT)6	47	--	--	--	--	--	--	TTGGAAACGCAAGGAAGATT	60	TGCTAAACTCTTCCATCTCAACA	59	278	--	--	--	--	--	--
TR26561|c0_g1_i1	No	(TTC)6	18	--	--	--	--	--	--	TTGAAAATGAAGGTGGGGTC	60	AATCGGAGAAATGAAGCCCT	60	151	--	--	--	--	--	--
TR26561|c0_g1_i2	No	(TTC)6	18	--	--	--	--	--	--	TTGAAAATGAAGGTGGGGTC	60	AATCGGAGAAATGAAGCCCT	60	151	--	--	--	--	--	--
TR26561|c0_g1_i3	No	(TTC)6	18	--	--	--	--	--	--	TTGAAAATGAAGGTGGGGTC	60	AATCGGAGAAATGAAGCCCT	60	151	--	--	--	--	--	--
TR26562|c0_g1_i1	No	(CT)9	18	--	--	--	--	--	--	CCACACACAGACACTTCTACTTTC	58	GAAGCTTTCATGGCTCTTGC	60	162	--	--	--	--	--	--
TR26562|c0_g1_i2	XLOC_007498	(CT)9	18	--	--	--	--	--	--	CCACACACAGACACTTCTACTTTC	58	GAAGCTTTCATGGCTCTTGC	60	162	--	--	--	--	--	--
TR26577|c0_g1_i1	No	(TAC)6	18	--	--	--	--	--	--	TGAAGGCGTTGTTCATTCAA	60	TCAAAACTGTCACTTGCCGA	60	247	--	--	--	--	--	--
TR26579|c0_g1_i1	XLOC_007508	(CCA)7	21	TR26579|c0_g1_i1	(CCA)7	Ca_LG_4	(CCA)5	12049453	12049467	GTTGCTGAATGCCTCTCCTC	60	TACCCAAAGTGGCAACATCA	60	252	Ca_00451	URR	--	--	--	--
TR26591|c0_g1_i1	No	(AT)6	12	--	--	--	--	--	--	TGCTATGCTCAGAAGAACGC	59	TACAACAATGCCCAAGCAAA	60	234	--	--	--	--	--	--
TR26597|c0_g1_i1	No	(CT)6	12	--	--	--	--	--	--	TTCTAAAATCGCCGCTCTTC	60	CGAAACCCTAGCAGTGAAGG	60	124	--	--	--	--	--	--

Transcript IDs	Differentially expressed gene (DEG) IDs	SSR repeat-motifs	SSR repeat-length (bp)	*In silico* polymorphic SSRs						Forward primers (5'-3')	Annealing tempearture(0C)	Reverse primers (5'-3')	Annealing tempearture(0C)	Amplified product size (bp)	Structural annotation		Functional Annotation			
				C. microphyllum		ICC4958									Gene IDs	Sequence component of genes	NCBI nr IDs	TFs	KOG	KEGG
				Transcript	SSR repeat-motifs	Chromosomes	SSR repeat-motifs	Start physical positions (bp)	End physical positions (bp)											
TR26597|c0_g1_i2	No	(CT)6	12	--	--	--	--	--	--	TTCTAAAATCGCCGCTCTTC	60	CGAAACCCTAGCAGTGAAGG	60	124	--	--	--	--	--	--
TR26605|c0_g1_i1	XLOC_007526	(A)10ctttg(T)10a(T)10	36	--	--	--	--	--	--	CAAAACAAGGGTGAACAAACAA	60	CCAAACCAGCCATAGACACA	60	225	--	--	--	--	--	--
TR26607|c1_g1_i2	XLOC_007529	(CTT)7	21	--	--	--	--	--	--	GCACCTTAGACACCACCCAT	60	TGTTGTTGGGGATAAGGGAA	60	173	--	--	--	--	--	--
TR26633|c0_g1_i2	No	(AC)6agagaatggcattaggatgaatcatttcatgatgaaaactattgatcaaaatagaataaacattgttttcttgctttacttgaaattttaaaa(T)11	116	--	--	--	--	--	--	GCCTGAAAACCACCACAAAT	60	GGGTTCAACACGTCCCTCTA	60	224	--	--	--	--	--	--
TR26716|c0_g1_i1	No	(TTG)5	15	--	--	--	--	--	--	TGCCTTCAGCTGTTGTTGAC	60	CAAGAAGCCATCGAAAAAGC	60	183	--	--	--	--	--	--
TR26735|c0_g1_i1	No	(AT)8	16	--	--	--	--	--	--	AAGCTTGACAAACTCTTTTCCTG	59	TGTTTCTCACTAAAAGATGCAAAA A	59	108	--	--	--	--	--	--
TR26770|c0_g1_i1	No	(CAC)5	15	--	--	--	--	--	--	AAAGTTTGCTTCCAACACCG	60	AATCATAAATGCTCAAGACAAAAA	57	110	--	--	--	--	--	--
TR26810|c0_g2_i1	No	(TG)6	12	--	--	--	--	--	--	AGTCGATGCAGTTGTTGGAA	59	TTTGAGGATGGGAACACACA	60	223	--	--	--	--	--	--
TR26812|c0_g2_i1	No	(T)11cctttacttggttcctaaatttagcataccccatttttctcactcccttctcctccaattattcttatcaacttgga(T)10	98	--	--	--	--	--	--	TCAATCCCCCTTAAAAGTACCA	60	ATGGCACAACCCAATACAAA	59	280	--	--	--	--	--	--
TR26823|c0_g2_i1	No	(TA)7	14	--	--	--	--	--	--	TAGTAATCGTGGTTGGCGCT	61	CGGTTGCCATTTTTGTTTCT	60	249	--	--	--	--	--	--
TR26823|c0_g2_i2	No	(TA)7	14	--	--	--	--	--	--	TAGTAATCGTGGTTGGCGCT	61	CGGTTGCCATTTTTGTTTCT	60	249	--	--	--	--	--	--
TR26826|c0_g1_i1	XLOC_007617	(CT)18	36	--	--	--	--	--	--	AGACAAACAGCCTTCTCTAGTCA	57	CGACGAAGCTGTTGTTGGTA	60	239	--	--	--	--	--	--
TR26830|c0_g1_i1	No	(T)16actcttcaacatctacaaggc(A)12	49	--	--	--	--	--	--	GCAATAATAACTCAATCTTTTGCAC	58	TTCAACATTGTGGCAAGTTTTT	60	280	--	--	--	--	--	--
TR26830|c0_g1_i2	No	(T)16actcttcaacatctacaaggc(A)12	49	--	--	--	--	--	--	GCAATAATAACTCAATCTTTTGCAC	58	TTCAACATTGTGGCAAGTTTTT	60	280	--	--	--	--	--	--

Transcript IDs	Differentially expressed gene (DEG) IDs	SSR repeat-motifs	SSR repeat-length (bp)	*In silico* polymorphic SSRs						Forward primers (5'-3')	Annealing tempearture(0C)	Reverse primers (5'-3')	Annealing tempearture(0C)	Amplified product size (bp)	Structural annotation		Functional Annotation			
				C. microphyllum		ICC4958									Gene IDs	Sequence component of genes	NCBI nr IDs	TFs	KOG	KEGG
				Transcript	SSR repeat-motifs	Chromosomes	SSR repeat-motifs	Start physical positions (bp)	End physical positions (bp)											
TR26836|c0_g1_i1	XLOC_007628	(AT)6	12	--	--	--	--	--	--	CGGGTACCAACTCCCAATTA	60	CCAGTGGCCAATCAGATTTT	60	208	--	--	--	--	--	--
TR26854|c0_g1_i2	No	(CCT)5	15	--	--	--	--	--	--	CAGGGGCACCAAGTTTTAGA	60	AAGGGTGGTGCTGAAAAATG	60	136	--	--	--	--	--	--
TR26854|c0_g1_i3	No	(CCT)5	15	--	--	--	--	--	--	CAGGGGCACCAAGTTTTAGA	60	AAGGGTGGTGCTGAAAAATG	60	136	--	--	--	--	--	--
TR26854|c0_g1_i4	No	(CCT)5	15	--	--	--	--	--	--	CAGGGGCACCAAGTTTTAGA	60	AAGGGTGGTGCTGAAAAATG	60	136	--	--	--	--	--	--
TR26860|c0_g1_i1	No	(CT)6	12	--	--	--	--	--	--	GCAGAGGGAATGGTTGTGAT	60	CGGAAGAAGACATGGTTGGT	60	267	--	--	--	--	--	--
TR26860|c0_g1_i2	No	(CT)6	12	--	--	--	--	--	--	GCAGAGGGAATGGTTGTGAT	60	CGGAAGAAGACATGGTTGGT	60	267	--	--	--	--	--	--
TR26860|c0_g1_i3	No	(CT)6	12	--	--	--	--	--	--	GCAGAGGGAATGGTTGTGAT	60	CGGAAGAAGACATGGTTGGT	60	267	--	--	--	--	--	--
TR26860|c0_g1_i4	No	(CT)6	12	--	--	--	--	--	--	GCAGAGGGAATGGTTGTGAT	60	CGGAAGAAGACATGGTTGGT	60	267	--	--	--	--	--	--
TR26866|c2_g1_i1	XLOC_007670	(AT)9	18	--	--	--	--	--	--	TTTCTGGAAGAGGCAGAAGC	60	CCCTATTTTCATCCACCCCT	60	165	--	--	--	--	--	--
TR26872|c0_g1_i1	No	(AG)6	12	--	--	--	--	--	--	GCTTTTGCTTTGCTTCCATT	59	AAACGTGCACACATGGAAAA	60	187	--	--	--	--	--	--
TR26882|c0_g2_i1	No	(AGA)6	18	--	--	--	--	--	--	TCATCCACCAATCAATTTGC	59	AAAAGGCTCTCCAGGACCAT	60	157	--	--	--	--	--	--
TR26882|c0_g2_i2	No	(AGA)6	18	--	--	--	--	--	--	TCATCCACCAATCAATTTGC	59	AAAAGGCTCTCCAGGACCAT	60	157	--	--	--	--	--	--
TR26882|c0_g2_i3	No	(AGA)6	18	--	--	--	--	--	--	TCATCCACCAATCAATTTGC	59	AAAAGGCTCTCCAGGACCAT	60	157	--	--	--	--	--	--
TR26882|c0_g2_i4	No	(AGA)6	18	--	--	--	--	--	--	TCATCCACCAATCAATTTGC	59	AAAAGGCTCTCCAGGACCAT	60	157	--	--	--	--	--	--

Transcript IDs	Differentially expressed gene (DEG) IDs	SSR repeat-motifs	SSR repeat-length (bp)	*In silico* polymorphic SSRs						Forward primers (5'-3')	Annealing tempearture(0C)	Reverse primers (5'-3')	Annealing tempearture(0C)	Amplified product size (bp)	Structural annotation		Functional Annotation			
				C. microphyllum		ICC4958														
				Transcript	SSR repeat-motifs	Chromosomes	SSR repeat-motifs	Start physical positions (bp)	End physical positions (bp)						Gene IDs	Sequence component of genes	NCBI nr IDs	TFs	KOG	KEGG
TR26893|c3_g1_i1	XLOC_007702	(AG)8	16	--	--	--	--	--	--	GATGCCCCAGTAATCAGGAA	60	CCAAACGGAGGAATCAAGAA	60	218	--	--	--	--	--	--
TR26894|c0_g2_i1	No	(CT)11	22	--	--	--	--	--	--	TCATTCATTCACTCACTCTCTCAA	59	GTGGTGTTGGCAAGATGATG	60	110	--	--	--	--	--	--
TR26894|c0_g2_i2	No	(CT)11	22	--	--	--	--	--	--	TCATTCATTCACTCACTCTCTCAA	59	GTGGTGTTGGCAAGATGATG	60	110	--	--	--	--	--	--
TR26894|c0_g2_i3	No	(CT)11	22	--	--	--	--	--	--	TCATTCATTCACTCACTCTCTCAA	59	GTGGTGTTGGCAAGATGATG	60	110	--	--	--	--	--	--
TR26897|c0_g1_i1	No	(CT)8	16	--	--	--	--	--	--	ATCCACCTTGTTGTCTCCCA	60	CTTCCCATCTTGAAAACCGA	60	280	--	--	--	--	--	--
TR26897|c0_g1_i2	No	(CT)8	16	--	--	--	--	--	--	ATCCACCTTGTTGTCTCCCA	60	CTTCCCATCTTGAAAACCGA	60	280	--	--	--	--	--	--
TR27006|c0_g1_i1	No	(CAT)5	15	--	--	--	--	--	--	CAATCCCCAAACCACAAATC	60	ACCACCGTTTGAAGATACGC	60	193	--	--	--	--	--	--
TR27006|c0_g1_i1	No	(CTT)8	24	--	--	--	--	--	--	TGGCCTGTCCCATTAGACTC	60	GATTTGTGGTTTGGGGATTG	60	205	--	--	--	--	--	--
TR27006|c0_g2_i1	No	(CAT)5	15	--	--	--	--	--	--	CAATCCCCAAACCACAAATC	60	ACCACCGTTTGAAGATACGC	60	193	--	--	--	--	--	--
TR27006|c0_g2_i1	No	(CTT)8	24	--	--	--	--	--	--	TGGCCTGTCCCATTAGACTC	60	GATTTGTGGTTTGGGGATTG	60	205	--	--	--	--	--	--
TR27028|c0_g1_i1	XLOC_007742	(TA)6	12	--	--	--	--	--	--	ATGTTGCTAATCACACCCCC	60	TCTTATTCTCCAACAAGCTCAAA	58	246	--	--	--	--	--	--
TR27045|c0_g1_i1	No	(ACA)5	15	--	--	--	--	--	--	AATTAATCAATGGCACCCGA	60	CTCCGAACACAACGCTGTTA	60	208	--	--	--	--	--	--
TR27051|c0_g1_i1	XLOC_007747	(CTT)5	15	--	--	--	--	--	--	GCTTTTCATTTCCTTTAGGCG	60	TCTCAGAAACTCAATGCCTCA	59	272	--	--	--	--	--	--
TR27087|c0_g1_i1	XLOC_007761	(ATA)5	15	--	--	--	--	--	--	CCAACCAACAAATCAAACCA	59	ACGAGGTAACAAGTTTGCCG	60	205	--	--	--	--	--	--

Transcript IDs	Differentially expressed gene (DEG) IDs	SSR repeat-motifs	SSR repeat-length (bp)	*In silico* polymorphic SSRs						Forward primers (5'-3')	Annealing tempearture(0C)	Reverse primers (5'-3')	Annealing tempearture(0C)	Amplified product size (bp)	Structural annotation		Functional Annotation			
				C. microphyllum		ICC4958									Gene IDs	Sequence component of genes	NCBI nr IDs	TFs	KOG	KEGG
				Transcript	SSR repeat-motifs	Chromosomes	SSR repeat-motifs	Start physical positions (bp)	End physical positions (bp)											
TR27128|c0_g1_i1	XLOC_007775	(CTC)7	21	TR27128|c0_g1_i1	(CTC)7	Ca_LG_3	(CTC)5	7133948	7133962	TCCCACCAAACTCAACATGA	60	TGGTGTGAATGGTTTCGGTA	60	101		URR	XM_004506830.1	MYB_related	[T]	--
TR27166|c0_g1_i1	XLOC_007781	(AT)6	12	--	--	--	--	--	--	TGCACCATCCCAATCATAAA	60	TCCATGTCCCTCCTCATTTT	59	249	--	--	XP_004515822.1	FAR1	[T]	K13448
TR27168|c0_g1_i1	No	(TGC)5	15	--	--	--	--	--	--	TCAACAGAGGAAAACCCCAG	60	TTTGATGAAATAGCAGCCCC	60	155	--	--	--	--	--	--
TR27278|c0_g1_i1	No	(AT)6	12	--	--	--	--	--	--	ACTTGCAAATCATAACCGCC	60	GCCCATAAACATAAACAGATTCAA	59	213	--	--	--	--	--	--
TR27322|c0_g1_i1	No	(AAG)6	18	--	--	--	--	--	--	AGAACGTGAACGCAAGAAGG	60	CACGCTTCAATTGCTGTTGT	60	164	--	--	--	--	--	--
TR27414|c0_g1_i1	XLOC_007828	(ACA)5	15	--	--	--	--	--	--	TGCCAATGGAAGGAAGAAAG	60	TTTCCGAAAAGGCCAGTTTA	60	273	--	--	--	--	--	--
TR27415|c0_g1_i1	No	(CT)7	14	--	--	--	--	--	--	TGCATCCCGCATATTTGATA	60	AAGTTGCGCAGTTGCCTAGT	60	185	--	--	--	--	--	--
TR27463|c0_g1_i1	No	(TTC)5tgtttcttcctcataatcctcataaaagaaaccgt(TTC)5	65	--	--	--	--	--	--	GCATCCACTGAAGCCCTATT	59	GCGGTTTGCTTTTTCTCTTG	60	272	--	--	--	--	--	--
TR27508|c0_g1_i1	XLOC_007843	(AAG)5	15	--	--	--	--	--	--	AATTCCCGCTCAGAGAAACA	60	CCATTGATCTTGGCGTTCTT	60	116	--	--	--	--	--	--
TR2759|c0_g1_i1	No	(TC)6	12	--	--	--	--	--	--	CTCTTTTGCACACTTGGGAA	59	CCCGGTGATGAAGAGAGAAA	60	138	--	--	--	--	--	--
TR276|c0_g1_i1	XLOC_007902	(GTG)5	15	--	--	--	--	--	--	TCGGATACCGGAGATATTGG	60	ATTAACCGCGTCTCAACACC	60	248	--	--	--	--	--	--
TR27651|c0_g1_i1	XLOC_007879	(TTC)5	15	--	--	--	--	--	--	CGGTGTAATGTGAAGAAAACCA	60	TCACCCCAAGAATCAAAAGC	60	255	--	--	XP_004510774.1	B3	[QI]	K15398
TR27680|c0_g1_i1	No	(AC)8agacagagagacagag(AC)8	48	--	--	--	--	--	--	AGGCCAGTTGAGAGCAATTT	59	GCATTTTACCAACAGACAACGA	60	149	--	--	--	--	--	--
TR27680|c0_g2_i1	No	(AC)8agacagagagacagag(AC)8	48	--	--	--	--	--	--	TCCCACAACATGACGAAGAA	60	GCATTTTACCAACAGACAACGA	60	223	--	--	--	--	--	--

<table>
<tr><th rowspan="3">Transcript IDs</th><th rowspan="3">Differentially expressed gene (DEG) IDs</th><th rowspan="3">SSR repeat-motifs</th><th rowspan="3">SSR repeat-length (bp)</th><th colspan="6">In silico polymorphic SSRs</th><th rowspan="3">Forward primers (5'-3')</th><th rowspan="3">Annealing tempearture(0C)</th><th rowspan="3">Reverse primers (5'-3')</th><th rowspan="3">Annealing tempearture(0C)</th><th rowspan="3">Amplified product size (bp)</th><th colspan="2">Structural annotation</th><th colspan="4">Functional Annotation</th></tr>
<tr><th colspan="2">C. microphyllum</th><th colspan="4">ICC4958</th><th rowspan="2">Gene IDs</th><th rowspan="2">Sequence component of genes</th><th rowspan="2">NCBI nr IDs</th><th rowspan="2">TFs</th><th rowspan="2">KOG</th><th rowspan="2">KEGG</th></tr>
<tr><th>Transcript</th><th>SSR repeat-motifs</th><th>Chromosomes</th><th>SSR repeat-motifs</th><th>Start physical positions (bp)</th><th>End physical positions (bp)</th></tr>
<tr><td>TR27708|c0_g1_i1</td><td>XLOC_007905</td><td>(GCA)5</td><td>15</td><td>--</td><td>--</td><td>--</td><td>--</td><td>--</td><td>--</td><td>CACACAGCCATCCAGCTAAA</td><td>60</td><td>GCATTTTCCTCCCCTTCTTC</td><td>60</td><td>265</td><td>--</td><td>--</td><td>--</td><td>--</td><td>--</td><td>--</td></tr>
<tr><td>TR2774|c0_g1_i1</td><td>XLOC_007926</td><td>(CAG)6</td><td>18</td><td>--</td><td>--</td><td>--</td><td>--</td><td>--</td><td>--</td><td>ATGGCAGCTATGCAATACCC</td><td>60</td><td>TCATCCATCCAATGAAGCAA</td><td>60</td><td>239</td><td>--</td><td>--</td><td>--</td><td>--</td><td>--</td><td>--</td></tr>
<tr><td>TR2774|c0_g1_i1</td><td>XLOC_007926</td><td>(ACC)6aactcaaccaccacttcttccgatcacc(CCA)5</td><td>61</td><td>--</td><td>--</td><td>--</td><td>--</td><td>--</td><td>--</td><td>TTTTCACTGCAATGTCCCAA</td><td>60</td><td>ATTGCTGTTGTTGTTGCTGC</td><td>60</td><td>278</td><td>--</td><td>--</td><td>--</td><td>--</td><td>--</td><td>--</td></tr>
<tr><td>TR27790|c0_g1_i1</td><td>XLOC_007937</td><td>(TTG)5</td><td>15</td><td>--</td><td>--</td><td>--</td><td>--</td><td>--</td><td>--</td><td>GCTCTTTGGTATGTTTGGGG</td><td>59</td><td>ATTGCCACTCCACTTTGTCC</td><td>60</td><td>125</td><td>--</td><td>--</td><td>--</td><td>--</td><td>--</td><td>--</td></tr>
<tr><td>TR27970|c0_g1_i1</td><td>XLOC_008001</td><td>(GTG)5</td><td>15</td><td>--</td><td>--</td><td>--</td><td>--</td><td>--</td><td>--</td><td>GGACTTGGTGGTAGTGGCTC</td><td>60</td><td>AGAACCTGCAGAGGAACCAG</td><td>59</td><td>165</td><td>--</td><td>--</td><td>--</td><td>--</td><td>--</td><td>--</td></tr>
<tr><td>TR27972|c0_g1_i1</td><td>XLOC_008003</td><td>(AT)8</td><td>16</td><td>TR27972|c0_g1_i1</td><td>(AT)8</td><td>Ca_LG_5</td><td>(AT)9</td><td>6017195</td><td>6017212</td><td>CATTGTGCGTGTTAGATGGC</td><td>60</td><td>ACCGTTCTACGTTTTCGACG</td><td>60</td><td>116</td><td>Ca_03974</td><td>CDS</td><td>--</td><td>--</td><td>--</td><td>--</td></tr>
<tr><td>TR27973|c0_g1_i1</td><td>No</td><td>(GA)6</td><td>12</td><td>--</td><td>--</td><td>--</td><td>--</td><td>--</td><td>--</td><td>TCAAGAGAGAAAGAAAGAAAGGAA
A</td><td>59</td><td>TTACAACATGGCCCAACTCA</td><td>60</td><td>274</td><td>--</td><td>--</td><td>--</td><td>--</td><td>--</td><td>--</td></tr>
<tr><td>TR27974|c0_g1_i1</td><td>No</td><td>(GAT)7</td><td>21</td><td>--</td><td>--</td><td>--</td><td>--</td><td>--</td><td>--</td><td>AGACGATGATGATGACGACG</td><td>60</td><td>GGGGCGTCCGATTAGTAAAT</td><td>60</td><td>154</td><td>--</td><td>--</td><td>--</td><td>--</td><td>--</td><td>--</td></tr>
<tr><td>TR28036|c0_g1_i1</td><td>XLOC_008030</td><td>(AG)8</td><td>16</td><td>--</td><td>--</td><td>--</td><td>--</td><td>--</td><td>--</td><td>AGAACCACGAAGCCAACAAG</td><td>60</td><td>TCGCTGTTGTCCACGTTATC</td><td>60</td><td>267</td><td>--</td><td>--</td><td>--</td><td>--</td><td>--</td><td>--</td></tr>
<tr><td>TR28106|c0_g1_i1</td><td>XLOC_008063</td><td>(CAA)5</td><td>15</td><td>--</td><td>--</td><td>--</td><td>--</td><td>--</td><td>--</td><td>ACTCCAAGGTGCGAAATCAG</td><td>60</td><td>GCAGAGACCAACGAAGAAGG</td><td>60</td><td>137</td><td>--</td><td>--</td><td>--</td><td>--</td><td>--</td><td>--</td></tr>
<tr><td>TR28110|c0_g1_i1</td><td>No</td><td>(AAC)5</td><td>15</td><td>--</td><td>--</td><td>--</td><td>--</td><td>--</td><td>--</td><td>GGTGATCCACCACAAGTTCC</td><td>60</td><td>GAAGAGGTGGCAGCATTCTC</td><td>60</td><td>244</td><td>--</td><td>--</td><td>--</td><td>--</td><td>--</td><td>--</td></tr>
<tr><td>TR28296|c1_g1_i1</td><td>No</td><td>(GGT)5</td><td>15</td><td>--</td><td>--</td><td>--</td><td>--</td><td>--</td><td>--</td><td>TGCACATGTTTATGGTGGTGT</td><td>60</td><td>ACCACTACCACTGCCACCTC</td><td>60</td><td>187</td><td>--</td><td>--</td><td>--</td><td>--</td><td>--</td><td>--</td></tr>
<tr><td>TR28296|c1_g2_i1</td><td>XLOC_008148</td><td>(GGT)5</td><td>15</td><td>--</td><td>--</td><td>--</td><td>--</td><td>--</td><td>--</td><td>TGCACATGTTTATGGTGGTGT</td><td>60</td><td>CATAACCAGCACCACCTCCT</td><td>60</td><td>272</td><td>--</td><td>--</td><td>--</td><td>--</td><td>--</td><td>--</td></tr>
<tr><td>TR28310|c0_g1_i2</td><td>No</td><td>(AAGA)5</td><td>20</td><td>--</td><td>--</td><td>--</td><td>--</td><td>--</td><td>--</td><td>GACGCTCTTCCGATCTGTTA</td><td>58</td><td>ACGAGATGGCTGGGACATAC</td><td>60</td><td>136</td><td>--</td><td>--</td><td>--</td><td>--</td><td>--</td><td>--</td></tr>
</table>

Transcript IDs	Differentially expressed gene (DEG) IDs	SSR repeat-motifs	SSR repeat-length (bp)	*In silico* polymorphic SSRs						Forward primers (5'-3')	Annealing tempearture(0C)	Reverse primers (5'-3')	Annealing tempearture(0C)	Amplified product size (bp)	Structural annotation		Functional Annotation			
				C. microphyllum		ICC4958														
				Transcript	SSR repeat-motifs	Chromosomes	SSR repeat-motifs	Start physical positions (bp)	End physical positions (bp)						Gene IDs	Sequence component of genes	NCBI nr IDs	TFs	KOG	KEGG
TR28310|c0_g1_i4	No	(AAGA)5	20	--	--	--	--	--	--	GACGCTCTTCCGATCTGTTA	58	ACGAGATGGCTGGGACATAC	60	136	--	--	--	--	--	--
TR28310|c0_g1_i5	No	(AAGA)5	20	--	--	--	--	--	--	GACGCTCTTCCGATCTGTTA	58	ACGAGATGGCTGGGACATAC	60	136	--	--	--	--	--	--
TR28317|c0_g2_i1	XLOC_008164	(TTC)5	15	--	--	--	--	--	--	ATCTCGAGAAGGCGAGAACA	60	AGGGTTTTGTTTGGGGATTC	60	277	--	--	--	--	--	--
TR28343|c0_g1_i1	No	(TA)6	12	--	--	--	--	--	--	TTTCTCCTTTCTTTCCCGGT	60	CTCCTCCTTCACTTTCGCAC	60	266	--	--	--	--	--	--
TR28343|c0_g2_i1	No	(TA)6	12	--	--	--	--	--	--	TTTCTCCTTTCTTTCCCGGT	60	CTCCTCCTTCACTTTCGCAC	60	260	--	--	--	--	--	--
TR28346|c0_g1_i1	XLOC_008187	(CAA)6	18	--	--	--	--	--	--	CCTCAGCTAGCTTTGGCAAT	60	TCTGCTACCCACTTACCCCA	61	243	--	--	--	--	--	--
TR28346|c0_g2_i1	XLOC_008188	(TA)6	12	--	--	--	--	--	--	CAGTTGAAATATGTCTCACGGAA	59	CCACCCATTTACCCCACTTT	61	280	--	--	XP_004499041.1	ERF	[K]	--
TR28365|c0_g1_i1	XLOC_008206	(AT)8	16	TR28365|c0_g1_i1	(AT)8	Ca_LG_1	(AT)6	27701024	27701035	AAATTTTATTTTGCAAAGGCCA	60	GCAAAGGTTTGGTGCTCATT	60	253		URR	--	--	--	--
TR28433|c0_g1_i1	XLOC_008239	(GA)8	16	--	--	--	--	--	--	CTCTCTCTGCCATCCCAAAA	60	CTTCCTCCAAGGTTGTGCAT	60	228	--	--	--	--	--	--
TR28445|c0_g3_i1	No	(TCT)5	15	--	--	--	--	--	--	TCCCTCGTTACCAACACTCA	59	AGAGGTTTGAGATTGCACGC	60	215	--	--	--	--	--	--
TR28445|c0_g3_i3	No	(TCT)5	15	--	--	--	--	--	--	TCCCTCGTTACCAACACTCA	59	AGAGGTTTGAGATTGCACGC	60	215	--	--	--	--	--	--
TR28451|c2_g3_i2	XLOC_008286	(GGA)5	15	--	--	--	--	--	--	ACGGTGGCGAACATAAGAAG	60	AACCCTAGAATCAAAGCGGC	61	206	--	--	--	--	--	--
TR28453|c2_g1_i1	XLOC_008292	(AAAG)6	24	--	--	--	--	--	--	TTGGGACCAAGATCCACAAT	60	AACGCCCCCACACATTACTA	60	209	--	--	--	--	--	--
TR28457|c0_g1_i1	XLOC_008296	(AAG)6	18	TR28457|c0_g1_i1	(AAG)6	Ca_LG_3	(AAG)7	42048971	42048991	TCACTTTCAGGCCTATTTGTCA	60	CGGATCTCGTTGTGCTTGTA	60	171		URR	--	--	--	--

Transcript IDs	Differentially expressed gene (DEG) IDs	SSR repeat-motifs	SSR repeat-length (bp)	*In silico* polymorphic SSRs						Forward primers (5'-3')	Annealing tempearture(0C)	Reverse primers (5'-3')	Annealing tempearture(0C)	Amplified product size (bp)	Structural annotation		Functional Annotation			
				C. microphyllum		ICC4958														
				Transcript	SSR repeat-motifs	Chromosomes	SSR repeat-motifs	Start physical positions (bp)	End physical positions (bp)						Gene IDs	Sequence component of genes	NCBI nr IDs	TFs	KOG	KEGG
TR28457|c0_g1_i2	No	(AAG)6	18	--	--	--	--	--	--	TCACTTTCAGGCCTATTTGTCA	60	CGGATCTCGTTGTGCTTGTA	60	171	--	--	--	--	--	--
TR28457|c1_g3_i1	XLOC_008299	(AGA)5	15	--	--	--	--	--	--	GGAGAAGCTTCAAAACGACG	60	CGCATTCGGATCATCTTTCT	60	238	--	--	--	--	--	--
TR28460|c3_g1_i1	No	(TC)6	12	--	--	--	--	--	--	AAAAGCGCAAAAGCGAATAA	60	CATCGCGAGTGAGAACTTGA	60	248	--	--	--	--	--	--
TR28460|c3_g1_i1	No	(AG)7	14	--	--	--	--	--	--	TAGGGTTTGTTGGATCTCGG	60	GTTCTCCCCCTGCAAGAAAT	60	236	--	--	--	--	--	--
TR28460|c3_g1_i16	No	(TC)6	12	--	--	--	--	--	--	AAAAGCGCAAAAGCGAATAA	60	CATCGCGAGTGAGAACTTGA	60	248	--	--	--	--	--	--
TR28460|c3_g1_i16	No	(AG)7	14	--	--	--	--	--	--	TAGGGTTTGTTGGATCTCGG	60	GTTCTCCCCCTGCAAGAAAT	60	236	--	--	--	--	--	--
TR28460|c3_g1_i17	No	(TC)6	12	--	--	--	--	--	--	AAAAGCGCAAAAGCGAATAA	60	CATCGCGAGTGAGAACTTGA	60	250	--	--	--	--	--	--
TR28460|c3_g1_i17	No	(AG)7	14	--	--	--	--	--	--	TAGGGTTTGTTGGATCTCGG	60	GTTCTCCCCCTGCAAGAAAT	60	236	--	--	--	--	--	--
TR28460|c3_g1_i9	No	(AG)9	18	--	--	--	--	--	--	TAGGGTTTGTTGGATCTCGG	60	GCAAATCACAGGAGCCAAAT	60	275	--	--	--	--	--	--
TR28464|c1_g1_i7	No	(AG)9	18	--	--	--	--	--	--	ACTCCGACGAAAACAACCAA	61	TCCTCATTTTCATCGCATCTT	60	103	--	--	--	--	--	--
TR28464|c3_g1_i1	XLOC_008332	(TCG)5ataacaaaaacgacgttaacccattgaaattgaaagtgaaaattggaaaagtaaagtagttag(TAT)5	93	--	--	--	--	--	--	GGAAGAGAAGAGGATTGGCA	59	GGTTAACGATGAGGCCAGAA	60	187	--	--	--	--	--	--
TR28467|c1_g2_i1	No	(TA)6	12	--	--	--	--	--	--	TGCCTTGTCTCTTTGGTCCT	60	GGTCTATTTGCATGGTTGTGG	60	221	--	--	--	--	--	--
TR28467|c1_g2_i10	No	(TA)6	12	--	--	--	--	--	--	TGCCTTGTCTCTTTGGTCCT	60	GGTCTATTTGCATGGTTGTGG	60	221	--	--	--	--	--	--
TR28467|c1_g2_i12	No	(TA)6	12	--	--	--	--	--	--	TGCCTTGTCTCTTTGGTCCT	60	GGTCTATTTGCATGGTTGTGG	60	221	--	--	--	--	--	--

Transcript IDs	Differentially expressed gene (DEG) IDs	SSR repeat-motifs	SSR repeat-length (bp)	*In silico* polymorphic SSRs						Forward primers (5'-3')	Annealing tempearture(0C)	Reverse primers (5'-3')	Annealing tempearture(0C)	Amplified product size (bp)	Structural annotation		Functional Annotation			
				C. microphyllum		ICC4958									Gene IDs	Sequence component of genes	NCBI nr IDs	TFs	KOG	KEGG
				Transcript	SSR repeat-motifs	Chromosomes	SSR repeat-motifs	Start physical positions (bp)	End physical positions (bp)											
TR28467|c1_g2_i16	No	(TA)6	12	--	--	--	--	--	--	TGCCTTGTCTCTTTGGTCCT	60	GGTCTATTTGCATGGTTGTGG	60	221	--	--	--	--	--	--
TR28467|c1_g2_i9	No	(TA)6	12	--	--	--	--	--	--	TGCCTTGTCTCTTTGGTCCT	60	GGTCTATTTGCATGGTTGTGG	60	221	--	--	--	--	--	--
TR28469|c3_g1_i2	No	(GT)10	20	TR28469|c3_g1_i2	(GT)10	Ca_LG_7	(GT)8	5631903	5631918	GTTCAACTAAAGACTGGCTCGT	58	GACCATCTGCAAAAGTGAACC	60	213	Ca_16084	DRR	--	--	--	--
TR28469|c3_g1_i4	No	(GT)10	20	--	--	--	--	--	--	GTTCAACTAAAGACTGGCTCGT	58	GACCATCTGCAAAAGTGAACC	60	213	--	--	--	--	--	--
TR28473|c0_g3_i1	No	(GTT)5gtgttgagaaattgaagtggggtttgtgcagacgt aataaaaaggatagatctgactttgactttggaggcatctcc gtcattaaaagggtagtata(GTT)5	127	--	--	--	--	--	--	GGGGTTTGTGCAGACGTAAT	60	AAACCCCACTTCAATTTCTCA	59	231	--	--	--	--	--	--
TR28473|c1_g5_i1	XLOC_008360	(ATA)5	15	--	--	--	--	--	--	TTGTTTTCAATGTTTGGAATTG	58	CCCTAATCATAACACAACATACAT CA	59	278	--	--	--	--	--	--
TR28473|c1_g5_i2	No	(ATA)5	15	--	--	--	--	--	--	TTGTTTTCAATGTTTGGAATTG	58	CCCTAATCATAACACAACATACAT CA	59	278	--	--	--	--	--	--
TR28473|c1_g5_i6	No	(ATA)5	15	--	--	--	--	--	--	TTGTTTTCAATGTTTGGAATTG	58	CCCTAATCATAACACAACATACAT CA	59	278	--	--	--	--	--	--
TR28488|c3_g2_i2	No	(ATCGGA)6	36	--	--	--	--	--	--	TGAATTTGAATCCATTGCGA	60	GGTTCCAGTTCTGGTTCCTG	60	248	--	--	--	--	--	--
TR28488|c3_g2_i3	XLOC_008423	(ATCGGA)6	36	--	--	--	--	--	--	TGAATTTGAATCCATTGCGA	60	GGTTCCAGTTCTGGTTCCTG	60	248	--	--	--	--	--	--
TR28488|c6_g1_i1	XLOC_008427	(GAA)5	15	--	--	--	--	--	--	GGTTCTGGGAGAATCGATGA	60	TTCATCAAGTTCCGCTCCTC	60	225	--	--	--	--	--	--
TR28498|c0_g2_i1	XLOC_008456	(A)10tactatata(T)11aatttgggtttttgggg(T)11	58	--	--	--	--	--	--	TCAGGGTTTCGTCTTTGCTT	60	CTTCCCTGAAGTCCAGCATC	60	158	--	--	--	--	--	--
TR28504|c0_g1_i1	No	(TGC)7	21	--	--	--	--	--	--	TATCCTGTCATTGCACCCAA	60	CTATCCATTCGCACCCAAAG	60	199	--	--	--	--	--	--
TR28504|c0_g2_i1	XLOC_008483	(TC)11ttgatttctctcccaaccgcta(TC)8	60	--	--	--	--	--	--	ATGAAGATCCGATTTCGACG	60	GTGTGCCTTTGTCGAAGTGA	60	263	--	--	--	--	--	--

Transcript IDs	Differentially expressed gene (DEG) IDs	SSR repeat-motifs	SSR repeat-length (bp)	*In silico* polymorphic SSRs						Forward primers (5'-3')	Annealing tempearture(0C)	Reverse primers (5'-3')	Annealing tempearture(0C)	Amplified product size (bp)	Structural annotation		Functional Annotation			
				C. microphyllum		ICC4958									Gene IDs	Sequence component of genes	NCBI nr IDs	TFs	KOG	KEGG
				Transcript	SSR repeat-motifs	Chromosomes	SSR repeat-motifs	Start physical positions (bp)	End physical positions (bp)											
TR28504|c1_g2_i5	No	(TC)10	20	--	--	--	--	--	--	TCTTCTTATTTTGCTTCCATTTCA	59	GGGCCATCCACATTTGTTAC	60	191	--	--	--	--	--	--
TR28508|c1_g1_i1	XLOC_008493	(AGA)5	15	--	--	--	--	--	--	ACGTCCGTTGGATGAAACTC	60	AACGAAGATCTTGCGGCTAA	60	112	--	--	--	--	--	--
TR28508|c1_g1_i1	XLOC_008493	(AG)6	12	--	--	--	--	--	--	TTCACAGTTCAAACTCACACCA	59	AGTTCCTGAAGATTCCGGCT	60	244	--	--	--	--	--	--
TR28510|c3_g1_i1	XLOC_008504	(TC)10	20	--	--	--	--	--	--	CACACCACACCCCTTTCTCT	60	TAGCTGGTGATTGCGATGAA	60	273	--	--	--	--	--	--
TR28510|c3_g2_i1	XLOC_008505	(TC)10	20	--	--	--	--	--	--	CACACCACACCCCTTTCTCT	60	TAGCTGGTGATTGCGATGAA	60	273	--	--	--	--	--	--
TR28511|c1_g2_i1	XLOC_008506	(CA)6	12	--	--	--	--	--	--	CACCATTGGACTCTCCAACA	60	GCCGTACCATTGTCGAATCT	60	163	--	--	--	--	--	--
TR28511|c3_g3_i5	No	(AT)6agtca(T)11	28	--	--	--	--	--	--	TTTAAATTCCTTCAAAAATATTGACC	58	ACACCACCGTCATTCAGTCA	60	218	--	--	--	--	--	--
TR28511|c3_g3_i9	XLOC_008510	(AT)6agtca(T)11	28	--	--	--	--	--	--	TTTAAATTCCTTCAAAAATATTGACC	58	ACACCACCGTCATTCAGTCA	60	218	--	--	--	--	--	--
TR28514|c2_g1_i1	XLOC_008529	(TC)8	16	--	--	--	--	--	--	GGAGGTTTTGTCGGAACAAG	60	TGGATTCTGGCCTAATGACC	60	274	--	--	--	--	--	--
TR28520|c2_g1_i1	XLOC_008561	(TGC)5	15	--	--	--	--	--	--	GCTGCAAAGGTTGATGTTGA	60	AAGCTGAATCCCATGTCGTC	60	221	--	--	XM_004515933.1	--	[J]	K02942
TR28520|c2_g5_i5	XLOC_008564	(TA)7	14	--	--	--	--	--	--	CAAAAAGGAGGCTGCAAAAA	60	TCTGATTTTTGGTGTGAATCTCAT	60	225	--	--	--	--	--	--
TR28520|c2_g5_i9	XLOC_008567	(TA)7	14	--	--	--	--	--	--	CAAAAAGGAGGCTGCAAAAA	60	TCTGATTTTTGGTGTGAATCTCAT	60	225	--	--	--	--	--	--
TR28526|c1_g5_i1	XLOC_008594	(TTC)6	18	--	--	--	--	--	--	AACATCATGAACTCAGAAGATGGA	60	GGGGTGTTGAAGAGTGAGGA	60	211	--	--	--	--	--	--
TR28530|c3_g1_i1	No	(TG)6	12	--	--	--	--	--	--	TTGGATCGTCGATGAGTTTG	60	CCCTCCACCACAATATTCCA	61	220	--	--	--	--	--	--

<table>
<tr><th rowspan="3">Transcript IDs</th><th rowspan="3">Differentially expressed gene (DEG) IDs</th><th rowspan="3">SSR repeat-motifs</th><th rowspan="3">SSR repeat-length (bp)</th><th colspan="6">In silico polymorphic SSRs</th><th rowspan="3">Forward primers (5'-3')</th><th rowspan="3">Annealing tempearture(0C)</th><th rowspan="3">Reverse primers (5'-3')</th><th rowspan="3">Annealing tempearture(0C)</th><th rowspan="3">Amplified product size (bp)</th><th colspan="2">Structural annotation</th><th colspan="4">Functional Annotation</th></tr>
<tr><th colspan="2">C. microphyllum</th><th colspan="4">ICC4958</th><th rowspan="2">Gene IDs</th><th rowspan="2">Sequence component of genes</th><th rowspan="2">NCBI nr IDs</th><th rowspan="2">TFs</th><th rowspan="2">KOG</th><th rowspan="2">KEGG</th></tr>
<tr><th>Transcript</th><th>SSR repeat-motifs</th><th>Chromosomes</th><th>SSR repeat-motifs</th><th>Start physical positions (bp)</th><th>End physical positions (bp)</th></tr>
<tr><td>TR28531|c0_g2_i1</td><td>No</td><td>(TAT)5</td><td>15</td><td>TR28531|c0_g2_i1</td><td>(TAT)5</td><td>Ca_LG_6</td><td>(TAT)8</td><td>27139326</td><td>27139349</td><td>GCCACACACAGAACTCAACTTT</td><td>59</td><td>GCATGAATTGGAGCATTTGTT</td><td>60</td><td>280</td><td>Ca_15218</td><td>URR</td><td>--</td><td>--</td><td>--</td><td>--</td></tr>
<tr><td>TR28531|c0_g2_i2</td><td>No</td><td>(TAT)5</td><td>15</td><td>--</td><td>--</td><td>--</td><td>--</td><td>--</td><td>--</td><td>GCCACACACAGAACTCAACTTT</td><td>59</td><td>GCATGAATTGGAGCATTTGTT</td><td>60</td><td>280</td><td>--</td><td>--</td><td>--</td><td>--</td><td>--</td><td>--</td></tr>
<tr><td>TR28531|c0_g2_i3</td><td>No</td><td>(TAT)5</td><td>15</td><td>--</td><td>--</td><td>--</td><td>--</td><td>--</td><td>--</td><td>GCCACACACAGAACTCAACTTT</td><td>59</td><td>GCATGAATTGGAGCATTTGTT</td><td>60</td><td>280</td><td>--</td><td>--</td><td>--</td><td>--</td><td>--</td><td>--</td></tr>
<tr><td>TR28531|c0_g2_i4</td><td>No</td><td>(TAT)5</td><td>15</td><td>--</td><td>--</td><td>--</td><td>--</td><td>--</td><td>--</td><td>GCCACACACAGAACTCAACTTT</td><td>59</td><td>GCATGAATTGGAGCATTTGTT</td><td>60</td><td>280</td><td>--</td><td>--</td><td>--</td><td>--</td><td>--</td><td>--</td></tr>
<tr><td>TR28531|c0_g2_i5</td><td>No</td><td>(TAT)5</td><td>15</td><td>--</td><td>--</td><td>--</td><td>--</td><td>--</td><td>--</td><td>GCCACACACAGAACTCAACTTT</td><td>59</td><td>GCATGAATTGGAGCATTTGTT</td><td>60</td><td>280</td><td>--</td><td>--</td><td>--</td><td>--</td><td>--</td><td>--</td></tr>
<tr><td>TR28531|c0_g2_i9</td><td>No</td><td>(TAT)5</td><td>15</td><td>--</td><td>--</td><td>--</td><td>--</td><td>--</td><td>--</td><td>GCCACACACAGAACTCAACTTT</td><td>59</td><td>GCATGAATTGGAGCATTTGTT</td><td>60</td><td>280</td><td>--</td><td>--</td><td>--</td><td>--</td><td>--</td><td>--</td></tr>
<tr><td>TR28536|c2_g1_i1</td><td>XLOC_008619</td><td>(TTAT)5</td><td>20</td><td>--</td><td>--</td><td>--</td><td>--</td><td>--</td><td>--</td><td>GTTGGGAGTTGGGACATTTG</td><td>60</td><td>GCAAATCAGAAACAAAACCCA</td><td>60</td><td>204</td><td>--</td><td>--</td><td>--</td><td>--</td><td>--</td><td>--</td></tr>
<tr><td>TR28538|c3_g3_i1</td><td>XLOC_008626</td><td>(AAG)6</td><td>18</td><td>TR28538|c3_g3_i1</td><td>(AAG)6</td><td>scaffold00894</td><td>(AAG)5</td><td>9086</td><td>9100</td><td>TGGTTTTGATGAATTTGGTCAC</td><td>60</td><td>AAACCCAAAAGACCAGCAGA</td><td>60</td><td>280</td><td>Ca_21341</td><td>CDS</td><td>--</td><td>--</td><td>--</td><td>--</td></tr>
<tr><td>TR28541|c0_g1_i11</td><td>No</td><td>(TTA)5</td><td>15</td><td>--</td><td>--</td><td>--</td><td>--</td><td>--</td><td>--</td><td>TGGGCCCACATATAGTTTGA</td><td>59</td><td>ATTTTGGGGGCAAAAGAACT</td><td>60</td><td>182</td><td>--</td><td>--</td><td>--</td><td>--</td><td>--</td><td>--</td></tr>
<tr><td>TR28541|c0_g1_i3</td><td>No</td><td>(TTA)5</td><td>15</td><td>--</td><td>--</td><td>--</td><td>--</td><td>--</td><td>--</td><td>TGGGCCCACATATAGTTTGA</td><td>59</td><td>ATTTTGGGGGCAAAAGAACT</td><td>60</td><td>182</td><td>--</td><td>--</td><td>--</td><td>--</td><td>--</td><td>--</td></tr>
<tr><td>TR28541|c0_g1_i4</td><td>No</td><td>(TTA)5</td><td>15</td><td>--</td><td>--</td><td>--</td><td>--</td><td>--</td><td>--</td><td>TGGGCCCACATATAGTTTGA</td><td>59</td><td>ATTTTGGGGGCAAAAGAACT</td><td>60</td><td>182</td><td>--</td><td>--</td><td>--</td><td>--</td><td>--</td><td>--</td></tr>
<tr><td>TR28541|c0_g1_i6</td><td>No</td><td>(TTA)5</td><td>15</td><td>--</td><td>--</td><td>--</td><td>--</td><td>--</td><td>--</td><td>TGGGCCCACATATAGTTTGA</td><td>59</td><td>ATTTTGGGGGCAAAAGAACT</td><td>60</td><td>182</td><td>--</td><td>--</td><td>--</td><td>--</td><td>--</td><td>--</td></tr>
<tr><td>TR28541|c0_g1_i8</td><td>No</td><td>(TTA)5</td><td>15</td><td>--</td><td>--</td><td>--</td><td>--</td><td>--</td><td>--</td><td>TGGGCCCACATATAGTTTGA</td><td>59</td><td>ATTTTGGGGGCAAAAGAACT</td><td>60</td><td>182</td><td>--</td><td>--</td><td>--</td><td>--</td><td>--</td><td>--</td></tr>
<tr><td>TR28541|c0_g1_i9</td><td>No</td><td>(TTA)5</td><td>15</td><td>--</td><td>--</td><td>--</td><td>--</td><td>--</td><td>--</td><td>TGGGCCCACATATAGTTTGA</td><td>59</td><td>ATTTTGGGGGCAAAAGAACT</td><td>60</td><td>182</td><td>--</td><td>--</td><td>--</td><td>--</td><td>--</td><td>--</td></tr>
</table>

Transcript IDs	Differentially expressed gene (DEG) IDs	SSR repeat-motifs	SSR repeat-length (bp)	*In silico* polymorphic SSRs						Forward primers (5'-3')	Annealing tempearture(0C)	Reverse primers (5'-3')	Annealing tempearture(0C)	Amplified product size (bp)	Structural annotation		Functional Annotation			
				C. microphyllum		ICC4958									Gene IDs	Sequence component of genes	NCBI nr IDs	TFs	KOG	KEGG
				Transcript	SSR repeat-motifs	Chromosomes	SSR repeat-motifs	Start physical positions (bp)	End physical positions (bp)											
TR28543|c1_g1_i1	No	(TGT)5	15	--	--	--	--	--	--	GCCTAGTCCATTGCTCTCCA	60	TTTCCATCATTCACCTTTTCA	58	183	--	--	--	--	--	--
TR28543|c1_g1_i2	No	(TGT)5	15	--	--	--	--	--	--	GCCTAGTCCATTGCTCTCCA	60	TTTCCATCATTCACCTTTTCA	58	183	--	--	--	--	--	--
TR28543|c1_g1_i3	No	(TGT)5	15	--	--	--	--	--	--	GCCTAGTCCATTGCTCTCCA	60	TTTCCATCATTCACCTTTTCA	58	183	--	--	--	--	--	--
TR28543|c3_g4_i1	XLOC_008640	(GT)7	14	--	--	--	--	--	--	CGAGTACCACATGAAATGCAG	59	GGAGGCACCATTCTTCTTTG	60	167	--	--	--	--	--	--
TR28543|c4_g2_i2	No	(CAT)8	24	--	--	--	--	--	--	CTGCAAAGGAGGACCTGGTA	60	GCACGTCCTCCAGGAATCTA	60	201	--	--	--	--	--	--
TR28543|c4_g2_i3	No	(CAT)8	24	--	--	--	--	--	--	CTGCAAAGGAGGACCTGGTA	60	GCACGTCCTCCAGGAATCTA	60	201	--	--	--	--	--	--
TR28547|c0_g2_i1	No	(TA)6	12	--	--	--	--	--	--	TGACACAATGAAAAGCATAAAAGA	59	TCATATGGCACGACTCTGGT	59	187	--	--	--	--	--	--
TR28552|c0_g1_i1	No	(AG)9	18	--	--	--	--	--	--	TCATCCGCAGAGGACAATAA	59	AGAAGAGAAGAAGCGGCCAT	60	265	--	--	--	--	--	--
TR28552|c0_g3_i1	XLOC_008656	(AG)9	18	--	--	--	--	--	--	TCATCCGCAGAGGACAATAA	59	AGAAGAGAAGAAGCGGCCAT	60	265	--	--	--	--	--	--
TR28553|c0_g1_i3	No	(TAT)6	18	--	--	--	--	--	--	AACGAGGGTTCGTTCATCAC	60	GCTCCGTAAGATTCGTCAGC	60	232	--	--	--	--	--	--
TR28553|c0_g1_i4	No	(TAT)6	18	--	--	--	--	--	--	AACGAGGGTTCGTTCATCAC	60	GCTCCGTAAGATTCGTCAGC	60	232	--	--	--	--	--	--
TR28553|c0_g1_i5	No	(TAT)6	18	--	--	--	--	--	--	GTCCCTGCAAGAAATGGAAA	60	CCAATGGACCTTGACTCGTT	60	131	--	--	--	--	--	--
TR28553|c0_g1_i7	No	(TAT)6	18	--	--	--	--	--	--	AACGAGGGTTCGTTCATCAC	60	GCTCCGTAAGATTCGTCAGC	60	232	--	--	--	--	--	--
TR28553|c0_g1_i9	No	(TAT)6	18	--	--	--	--	--	--	AACGAGGGTTCGTTCATCAC	60	GCTCCGTAAGATTCGTCAGC	60	232	--	--	--	--	--	--

Transcript IDs	Differentially expressed gene (DEG) IDs	SSR repeat-motifs	SSR repeat-length (bp)	*In silico* polymorphic SSRs						Forward primers (5'-3')	Annealing tempearture(0C)	Reverse primers (5'-3')	Annealing tempearture(0C)	Amplified product size (bp)	Structural annotation		Functional Annotation			
				C. microphyllum		ICC4958									Gene IDs	Sequence component of genes	NCBI nr IDs	TFs	KOG	KEGG
				Transcript	SSR repeat-motifs	Chromosomes	SSR repeat-motifs	Start physical positions (bp)	End physical positions (bp)											
TR28557|c0_g2_i1	No	(A)10cactagtatttgatattctcat(TA)6	44	--	--	--	--	--	--	CGGAAGTTACAAAATTTCCTTCA	60	CAGATGCTCATGGCAAGACT	59	221	--	--	--	--	--	--
TR28558|c1_g1_i1	XLOC_008675	(ATA)6tcacatcacatcacatcgttcacgaacccacatta caattcagaacgaacttgggagacttaagat(TC)6	96	--	--	--	--	--	--	GGATCACACATCCCATCCAT	60	AAGTCTCCCTCTTTGGCGTT	60	184	--	--	--	--	--	--
TR28558|c1_g2_i1	XLOC_008676	(CT)6	12	--	--	--	--	--	--	ACATCATTCACGAACCCACA	60	CAACCTGAAAAACAAGGGGA	60	188	--	--	--	--	--	--
TR28558|c2_g2_i5	No	(TA)8	16	--	--	--	--	--	--	TTTACAGGCTCTTGCTTCTGA	58	ACGCGTGAAGCTAATTCCAA	61	209	--	--	--	--	--	--
TR28562|c0_g1_i1	No	(AT)7	14	--	--	--	--	--	--	CGCAATAGCAAACAAATCTTTC	59	GGGTCGGGATGTTACAAAAA	60	277	--	--	--	--	--	--
TR28563|c0_g1_i1	No	(CT)9ccaaaatgctatcaccga(TC)6	48	--	--	--	--	--	--	TCTCCCAACTCCAACACTCC	60	TGCTTCAGACCACAGAATCG	60	206	--	--	--	--	--	--
TR28563|c0_g1_i10	No	(CT)9ccaaaatgctatcaccga(TC)6	48	--	--	--	--	--	--	TCTCCCAACTCCAACACTCC	60	TGCTTCAGACCACAGAATCG	60	206	--	--	--	--	--	--
TR28563|c0_g1_i12	No	(CT)9ccaaaatgctatcaccga(TC)6	48	--	--	--	--	--	--	TCTCCCAACTCCAACACTCC	60	TGCTTCAGACCACAGAATCG	60	206	--	--	--	--	--	--
TR28563|c0_g1_i13	No	(CT)9ccaaaatgctatcaccga(TC)6	48	--	--	--	--	--	--	TCTCCCAACTCCAACACTCC	60	TGCTTCAGACCACAGAATCG	60	206	--	--	--	--	--	--
TR28563|c0_g1_i14	No	(CT)9ccaaaatgctatcaccga(TC)6	48	--	--	--	--	--	--	TCTCCCAACTCCAACACTCC	60	TGCTTCAGACCACAGAATCG	60	206	--	--	--	--	--	--
TR28563|c0_g1_i15	No	(CT)9ccaaaatgctatcaccga(TC)6	48	--	--	--	--	--	--	TCTCCCAACTCCAACACTCC	60	TGCTTCAGACCACAGAATCG	60	206	--	--	--	--	--	--
TR28563|c0_g1_i16	No	(CT)9ccaaaatgctatcaccga(TC)6	48	--	--	--	--	--	--	TCTCCCAACTCCAACACTCC	60	TGCTTCAGACCACAGAATCG	60	206	--	--	--	--	--	--
TR28563|c0_g1_i2	No	(CT)9ccaaaatgctatcaccga(TC)6	48	--	--	--	--	--	--	TCTCCCAACTCCAACACTCC	60	TGCTTCAGACCACAGAATCG	60	206	--	--	--	--	--	--
TR28563|c0_g1_i3	No	(CT)9ccaaaatgctatcaccga(TC)6	48	--	--	--	--	--	--	TCTCCCAACTCCAACACTCC	60	TGCTTCAGACCACAGAATCG	60	206	--	--	--	--	--	--

Transcript IDs	Differentially expressed gene (DEG) IDs	SSR repeat-motifs	SSR repeat-length (bp)	*In silico* polymorphic SSRs						Forward primers (5'-3')	Annealing tempearture(0C)	Reverse primers (5'-3')	Annealing tempearture(0C)	Amplified product size (bp)	Structural annotation		Functional Annotation			
				C. microphyllum		ICC4958									Gene IDs	Sequence component of genes	NCBI nr IDs	TFs	KOG	KEGG
				Transcript	SSR repeat-motifs	Chromosomes	SSR repeat-motifs	Start physical positions (bp)	End physical positions (bp)											
TR28563|c0_g1_i4	No	(CT)9ccaaaatgctatcaccga(TC)6	48	--	--	--	--	--	--	TCTCCCAACTCCAACACTCC	60	TGCTTCAGACCACAGAATCG	60	206	--	--	--	--	--	--
TR28563|c0_g1_i5	No	(CT)9ccaaaatgctatcaccga(TC)6	48	--	--	--	--	--	--	TCTCCCAACTCCAACACTCC	60	TGCTTCAGACCACAGAATCG	60	206	--	--	--	--	--	--
TR28563|c0_g1_i7	No	(CT)9ccaaaatgctatcaccga(TC)6	48	--	--	--	--	--	--	TCTCCCAACTCCAACACTCC	60	TGCTTCAGACCACAGAATCG	60	206	--	--	--	--	--	--
TR28563|c0_g1_i8	No	(CT)9ccaaaatgctatcaccga(TC)6	48	--	--	--	--	--	--	TCTCCCAACTCCAACACTCC	60	TGCTTCAGACCACAGAATCG	60	206	--	--	--	--	--	--
TR28563|c0_g1_i9	No	(CT)9ccaaaatgctatcaccga(TC)6	48	--	--	--	--	--	--	TCTCCCAACTCCAACACTCC	60	TGCTTCAGACCACAGAATCG	60	206	--	--	--	--	--	--
TR28566|c2_g3_i2	No	(CT)9	18	--	--	--	--	--	--	TATTTGGCGTTGTTGCAAAG	60	TTGTCTTACCAGCAGCATCG	60	194	--	--	--	--	--	--
TR28566|c2_g4_i1	No	(CT)9	18	--	--	--	--	--	--	TATTTGGCGTTGTTGCAAAG	60	TTGTCTTACCAGCAGCATCG	60	194	--	--	--	--	--	--
TR28566|c2_g4_i5	No	(CT)9	18	--	--	--	--	--	--	TATTTGGCGTTGTTGCAAAG	60	TTGTCTTACCAGCAGCATCG	60	194	--	--	--	--	--	--
TR28566|c2_g4_i6	No	(CT)9	18	--	--	--	--	--	--	TATTTGGCGTTGTTGCAAAG	60	TTGTCTTACCAGCAGCATCG	60	194	--	--	--	--	--	--
TR28570|c2_g1_i1	No	(TTC)6	18	--	--	--	--	--	--	AATCACACGAAAATGGAGGC	60	TGTTCTCCGGGTCATCTTTC	60	262	--	--	--	--	--	--
TR28570|c2_g1_i4	No	(TTC)6	18	--	--	--	--	--	--	AATCACACGAAAATGGAGGC	60	TGTTCTCCGGGTCATCTTTC	60	262	--	--	--	--	--	--
TR28570|c2_g1_i5	No	(TTC)6	18	--	--	--	--	--	--	AATCACACGAAAATGGAGGC	60	TGTTCTCCGGGTCATCTTTC	60	262	--	--	--	--	--	--
TR28578|c1_g1_i1	No	(ACA)5	15	--	--	--	--	--	--	TTCAATTTTGTTGTTGTGGGTT	59	TGTTGGATGTTGCCTTGTGT	60	164	--	--	--	--	--	--
TR28578|c1_g2_i1	XLOC_008735	(ACA)5	15	--	--	--	--	--	--	TTCAATTTTGTTGTTGTGGGTT	59	TGTTGGATGTTGCCTTGTGT	60	164	--	--	--	--	--	--

<table>
<tr><th rowspan="3">Transcript IDs</th><th rowspan="3">Differentially expressed gene (DEG) IDs</th><th rowspan="3">SSR repeat-motifs</th><th rowspan="3">SSR repeat-length (bp)</th><th colspan="6">In silico polymorphic SSRs</th><th rowspan="3">Forward primers (5'-3')</th><th rowspan="3">Annealing tempearture(0C)</th><th rowspan="3">Reverse primers (5'-3')</th><th rowspan="3">Annealing tempearture(0C)</th><th rowspan="3">Amplified product size (bp)</th><th colspan="2">Structural annotation</th><th colspan="4">Functional Annotation</th></tr>
<tr><th colspan="2">C. microphyllum</th><th colspan="4">ICC4958</th><th rowspan="2">Gene IDs</th><th rowspan="2">Sequence component of genes</th><th rowspan="2">NCBI nr IDs</th><th rowspan="2">TFs</th><th rowspan="2">KOG</th><th rowspan="2">KEGG</th></tr>
<tr><th>Transcript</th><th>SSR repeat-motifs</th><th>Chromosomes</th><th>SSR repeat-motifs</th><th>Start physical positions (bp)</th><th>End physical positions (bp)</th></tr>
<tr><td>TR28578|c12_g1_i1</td><td>XLOC_008733</td><td>(GCTA)5</td><td>20</td><td>--</td><td>--</td><td>--</td><td>--</td><td>--</td><td>--</td><td>CATATGGCAGGATGGTAGGG</td><td>60</td><td>GATTGCACCAGCTGTTCTGA</td><td>60</td><td>169</td><td>--</td><td>--</td><td>--</td><td>--</td><td>--</td><td>--</td></tr>
<tr><td>TR28578|c15_g1_i1</td><td>XLOC_008734</td><td>(TA)7</td><td>14</td><td>--</td><td>--</td><td>--</td><td>--</td><td>--</td><td>--</td><td>TGTTTCTGGTGTCTTTGGCA</td><td>60</td><td>CAAATTGGCGCTTAATTAAAGTAT</td><td>58</td><td>268</td><td>--</td><td>--</td><td>XM_004504002.1</td><td>--</td><td>--</td><td>--</td></tr>
<tr><td>TR28578|c3_g2_i1</td><td>XLOC_008736</td><td>(TGT)6</td><td>18</td><td>--</td><td>--</td><td>--</td><td>--</td><td>--</td><td>--</td><td>TTTGGAAGGTGCATGAAAGA</td><td>59</td><td>AACACACAACATTGAGGTTTGC</td><td>60</td><td>263</td><td>--</td><td>--</td><td>KFM76745.1</td><td>--</td><td>[Z]</td><td>--</td></tr>
<tr><td>TR28578|c5_g1_i1</td><td>XLOC_008738</td><td>(AG)6</td><td>12</td><td>--</td><td>--</td><td>--</td><td>--</td><td>--</td><td>--</td><td>TGTTATTCTGAAGGTGGGGG</td><td>60</td><td>CAGCAGAAAAACAACAGCAGA</td><td>59</td><td>113</td><td>--</td><td>--</td><td>--</td><td>--</td><td>--</td><td>--</td></tr>
<tr><td>TR28578|c5_g1_i2</td><td>No</td><td>(AG)6</td><td>12</td><td>--</td><td>--</td><td>--</td><td>--</td><td>--</td><td>--</td><td>TGTTATTCTGAAGGTGGGGG</td><td>60</td><td>TCTTCTCTGTGCTGGACCCT</td><td>60</td><td>240</td><td>--</td><td>--</td><td>--</td><td>--</td><td>--</td><td>--</td></tr>
<tr><td>TR28578|c8_g1_i1</td><td>XLOC_008740</td><td>(CA)7</td><td>14</td><td>--</td><td>--</td><td>--</td><td>--</td><td>--</td><td>--</td><td>AAAACCCCACAAATTTTCCC</td><td>60</td><td>AAACGAAACGCAGGAAAGAA</td><td>60</td><td>190</td><td>--</td><td>--</td><td>--</td><td>--</td><td>--</td><td>--</td></tr>
<tr><td>TR28578|c8_g1_i1</td><td>XLOC_008740</td><td>(A)11tacaa(T)12</td><td>28</td><td>--</td><td>--</td><td>--</td><td>--</td><td>--</td><td>--</td><td>CCACACCCGTTCTCAATTTT</td><td>60</td><td>GAGCGCCATAAACAAACACA</td><td>60</td><td>195</td><td>--</td><td>--</td><td>--</td><td>--</td><td>--</td><td>--</td></tr>
<tr><td>TR28578|c8_g2_i10</td><td>No</td><td>(CAT)5caacaacaacatgcacctgctgcttttaataacttgccttctatggaaaacaatgctgctgcaccatcgtcttttt(GGA)6</td><td>108</td><td>--</td><td>--</td><td>--</td><td>--</td><td>--</td><td>--</td><td>TTGGGAGTTGGTGGTCAGAT</td><td>60</td><td>AAAATTCCCTCCTCCACCAG</td><td>60</td><td>204</td><td>--</td><td>--</td><td>--</td><td>--</td><td>--</td><td>--</td></tr>
<tr><td>TR28578|c8_g2_i12</td><td>XLOC_008741</td><td>(CAT)5caacaacaacatgcacctgctgcttttaataacttgccttctatggaaaacaatgctgctgcaccatcgtcttttt(GGA)6</td><td>108</td><td>--</td><td>--</td><td>--</td><td>--</td><td>--</td><td>--</td><td>TTGGGAGTTGGTGGTCAGAT</td><td>60</td><td>AAAATTCCCTCCTCCACCAG</td><td>60</td><td>204</td><td>--</td><td>--</td><td>--</td><td>--</td><td>--</td><td>--</td></tr>
<tr><td>TR28578|c8_g2_i4</td><td>No</td><td>(CAT)5caacaacaacatgcacctgctgcttttaataacttgccttctatggaaaacaatgctgctgcaccatcgtcttttt(GGA)6</td><td>108</td><td>--</td><td>--</td><td>--</td><td>--</td><td>--</td><td>--</td><td>TTGGGAGTTGGTGGTCAGAT</td><td>60</td><td>AAAATTCCCTCCTCCACCAG</td><td>60</td><td>204</td><td>--</td><td>--</td><td>--</td><td>--</td><td>--</td><td>--</td></tr>
<tr><td>TR28578|c8_g2_i6</td><td>No</td><td>(CAT)5caacaacaacatgcacctgctgcttttaataacttgccttctatggaaaacaatgctgctgcaccatcgtcttttt(GGA)6</td><td>108</td><td>--</td><td>--</td><td>--</td><td>--</td><td>--</td><td>--</td><td>TTGGGAGTTGGTGGTCAGAT</td><td>60</td><td>AAAATTCCCTCCTCCACCAG</td><td>60</td><td>204</td><td>--</td><td>--</td><td>--</td><td>--</td><td>--</td><td>--</td></tr>
<tr><td>TR28578|c8_g2_i9</td><td>No</td><td>(CAT)5caacaacaacatgcacctgctgcttttaataacttgccttctatggaaaacaatgctgctgcaccatcgtcttttt(GGA)6</td><td>108</td><td>--</td><td>--</td><td>--</td><td>--</td><td>--</td><td>--</td><td>TTGGGAGTTGGTGGTCAGAT</td><td>60</td><td>AAAATTCCCTCCTCCACCAG</td><td>60</td><td>204</td><td>--</td><td>--</td><td>--</td><td>--</td><td>--</td><td>--</td></tr>
<tr><td>TR28578|c9_g3_i1</td><td>No</td><td>(T)12(A)12</td><td>24</td><td>--</td><td>--</td><td>--</td><td>--</td><td>--</td><td>--</td><td>TTCCAAATCATGAAGACATCAAA</td><td>59</td><td>TTCACACCCCCTTCATTTTC</td><td>60</td><td>254</td><td>--</td><td>--</td><td>--</td><td>--</td><td>--</td><td>--</td></tr>
<tr><td>TR28579|c1_g1_i1</td><td>No</td><td>(GTG)6</td><td>18</td><td>--</td><td>--</td><td>--</td><td>--</td><td>--</td><td>--</td><td>TTCCTCGAGTTCGTCGTCTT</td><td>60</td><td>ACCAGAGCCCTCACCAGAG</td><td>60</td><td>175</td><td>--</td><td>--</td><td>--</td><td>--</td><td>--</td><td>--</td></tr>
</table>

<table>
<tr><th rowspan="3">Transcript IDs</th><th rowspan="3">Differentially expressed gene (DEG) IDs</th><th rowspan="3">SSR repeat-motifs</th><th rowspan="3">SSR repeat-length (bp)</th><th colspan="6">In silico polymorphic SSRs</th><th rowspan="3">Forward primers (5'-3')</th><th rowspan="3">Annealing tempearture(0C)</th><th rowspan="3">Reverse primers (5'-3')</th><th rowspan="3">Annealing tempearture(0C)</th><th rowspan="3">Amplified product size (bp)</th><th colspan="2">Structural annotation</th><th colspan="4">Functional Annotation</th></tr>
<tr><th colspan="2">C. microphyllum</th><th colspan="4">ICC4958</th><th rowspan="2">Gene IDs</th><th rowspan="2">Sequence component of genes</th><th rowspan="2">NCBI nr IDs</th><th rowspan="2">TFs</th><th rowspan="2">KOG</th><th rowspan="2">KEGG</th></tr>
<tr><th>Transcript</th><th>SSR repeat-motifs</th><th>Chromosomes</th><th>SSR repeat-motifs</th><th>Start physical positions (bp)</th><th>End physical positions (bp)</th></tr>
<tr><td>TR28579|c1_g2_i1</td><td>No</td><td>(GTG)6</td><td>18</td><td>--</td><td>--</td><td>--</td><td>--</td><td>--</td><td>--</td><td>TTCCTCGAGTTCGTCGTCTT</td><td>60</td><td>ACCAGAGCCCTCACCAGAG</td><td>60</td><td>175</td><td>--</td><td>--</td><td>--</td><td>--</td><td>--</td><td>--</td></tr>
<tr><td>TR28579|c5_g1_i1</td><td>No</td><td>(TCT)6</td><td>18</td><td>--</td><td>--</td><td>--</td><td>--</td><td>--</td><td>--</td><td>TCCTTAGCAGGAACCGAAGA</td><td>60</td><td>GACGCATCGACTCAGACAGA</td><td>60</td><td>248</td><td>--</td><td>--</td><td>--</td><td>--</td><td>--</td><td>--</td></tr>
<tr><td>TR28579|c5_g2_i1</td><td>No</td><td>(TCT)6</td><td>18</td><td>--</td><td>--</td><td>--</td><td>--</td><td>--</td><td>--</td><td>TCCTTAGCAGGAACCGAAGA</td><td>60</td><td>GACGCATCGACTCAGACAGA</td><td>60</td><td>248</td><td>--</td><td>--</td><td>--</td><td>--</td><td>--</td><td>--</td></tr>
<tr><td>TR28579|c8_g3_i1</td><td>No</td><td>(GAA)5</td><td>15</td><td>--</td><td>--</td><td>--</td><td>--</td><td>--</td><td>--</td><td>ATAACGGTGCTGATCTTGGG</td><td>60</td><td>TGGAAATTTTTGTCTGTGTCATC</td><td>59</td><td>186</td><td>--</td><td>--</td><td>--</td><td>--</td><td>--</td><td>--</td></tr>
<tr><td>TR28580|c1_g7_i1</td><td>XLOC_008759</td><td>(CG)7</td><td>14</td><td>--</td><td>--</td><td>--</td><td>--</td><td>--</td><td>--</td><td>CATGCAAAGTTGACCACCAC</td><td>60</td><td>GTGGAGGGCAGTCACGTAAT</td><td>60</td><td>217</td><td>--</td><td>--</td><td>--</td><td>--</td><td>--</td><td>--</td></tr>
<tr><td>TR28580|c1_g7_i10</td><td>No</td><td>(CG)7ccgccgccgcccgtccgtccgtccggccggctcg ggccttggctttggtcttggtcttggtcttgtggtg(TAT)5</td><td>99</td><td>--</td><td>--</td><td>--</td><td>--</td><td>--</td><td>--</td><td>CATGCAAAGTTGACCACCAC</td><td>60</td><td>TCCATCAGCTCTGAAACGTG</td><td>60</td><td>173</td><td>--</td><td>--</td><td>--</td><td>--</td><td>--</td><td>--</td></tr>
<tr><td>TR28580|c1_g7_i3</td><td>No</td><td>(CG)7</td><td>14</td><td>--</td><td>--</td><td>--</td><td>--</td><td>--</td><td>--</td><td>CATGCAAAGTTGACCACCAC</td><td>60</td><td>GTGGCACCTAGGGTTAGGGT</td><td>60</td><td>280</td><td>--</td><td>--</td><td>--</td><td>--</td><td>--</td><td>--</td></tr>
<tr><td>TR28580|c1_g7_i5</td><td>No</td><td>(CG)7</td><td>14</td><td>--</td><td>--</td><td>--</td><td>--</td><td>--</td><td>--</td><td>CATGCAAAGTTGACCACCAC</td><td>60</td><td>AGGGTTAGGGTTTTTGACCG</td><td>60</td><td>272</td><td>--</td><td>--</td><td>--</td><td>--</td><td>--</td><td>--</td></tr>
<tr><td>TR28580|c1_g7_i7</td><td>XLOC_008760</td><td>(CG)7</td><td>14</td><td>--</td><td>--</td><td>--</td><td>--</td><td>--</td><td>--</td><td>CATGCAAAGTTGACCACCAC</td><td>60</td><td>GTGGAGGGCAGTCACGTAAT</td><td>60</td><td>175</td><td>--</td><td>--</td><td>--</td><td>--</td><td>--</td><td>--</td></tr>
<tr><td>TR28586|c2_g3_i1</td><td>XLOC_008780</td><td>(TAA)5</td><td>15</td><td>--</td><td>--</td><td>--</td><td>--</td><td>--</td><td>--</td><td>TGCTGTACTTGATGTCCAAACA</td><td>59</td><td>TTGCTCGCTCCCTACATTTT</td><td>60</td><td>222</td><td>--</td><td>--</td><td>--</td><td>--</td><td>--</td><td>--</td></tr>
<tr><td>TR28588|c2_g3_i2</td><td>No</td><td>(AT)8</td><td>16</td><td>--</td><td>--</td><td>--</td><td>--</td><td>--</td><td>--</td><td>CTCAACCGTTGGCTCAAATC</td><td>61</td><td>TCAAGAGGAAGAACGTGTCAAA</td><td>60</td><td>244</td><td>--</td><td>--</td><td>--</td><td>--</td><td>--</td><td>--</td></tr>
<tr><td>TR28588|c2_g3_i4</td><td>No</td><td>(AT)8</td><td>16</td><td>--</td><td>--</td><td>--</td><td>--</td><td>--</td><td>--</td><td>CTCAACCGTTGGCTCAAATC</td><td>61</td><td>TCAAGAGGAAGAACGTGTCAAA</td><td>60</td><td>244</td><td>--</td><td>--</td><td>--</td><td>--</td><td>--</td><td>--</td></tr>
<tr><td>TR28588|c2_g3_i6</td><td>No</td><td>(AT)8</td><td>16</td><td>--</td><td>--</td><td>--</td><td>--</td><td>--</td><td>--</td><td>CTCAACCGTTGGCTCAAATC</td><td>61</td><td>TCAAGAGGAAGAACGTGTCAAA</td><td>60</td><td>244</td><td>--</td><td>--</td><td>--</td><td>--</td><td>--</td><td>--</td></tr>
<tr><td>TR28597|c0_g1_i1</td><td>XLOC_008818</td><td>(ATT)5</td><td>15</td><td>--</td><td>--</td><td>--</td><td>--</td><td>--</td><td>--</td><td>TCTTTCTTTTCAAACCGAGTCC</td><td>60</td><td>AGCTTCCGTGCAGATCAAGT</td><td>60</td><td>132</td><td>--</td><td>--</td><td>XP_004485859.1</td><td>ERF</td><td>[K]</td><td>--</td></tr>
</table>

Transcript IDs	Differentially expressed gene (DEG) IDs	SSR repeat-motifs	SSR repeat-length (bp)	*In silico* polymorphic SSRs						Forward primers (5'-3')	Annealing tempearture(0C)	Reverse primers (5'-3')	Annealing tempearture(0C)	Amplified product size (bp)	Structural annotation		Functional Annotation			
				C. microphyllum		ICC4958														
				Transcript	SSR repeat-motifs	Chromosomes	SSR repeat-motifs	Start physical positions (bp)	End physical positions (bp)						Gene IDs	Sequence component of genes	NCBI nr IDs	TFs	KOG	KEGG
TR28602|c0_g1_i1	XLOC_008831	(AG)20	40	TR28602|c0_g1_i1	(AG)20	Ca_LG_4	(AG)7	16493142	16493155	ACCAAAACCCTCACTCTTGC	59	AACCCAAGGCGCTAACACTA	60	272	Ca_03461	CDS	--	--	--	--
TR28602|c3_g1_i5	No	(AGAA)5	20	--	--	--	--	--	--	TGTCACACAGTTTTGGTCTGC	60	TGCGAGAGGGAGAGAGAATG	61	278	--	--	--	--	--	--
TR28603|c1_g1_i1	No	(AG)9	18	--	--	--	--	--	--	GACGAATAGTATAAGCAGCAAGAAA	58	CATCCAAGAAAGGGTGAGGA	60	198	--	--	--	--	--	--
TR28603|c1_g1_i2	XLOC_008837	(AG)9	18	--	--	--	--	--	--	GACGAATAGTATAAGCAGCAAGAAA	58	CATCCAAGAAAGGGTGAGGA	60	198	--	--	--	--	--	--
TR28608|c1_g1_i1	No	(CTGATC)5	30	--	--	--	--	--	--	GGTGCTTTACACGGTGGACT	60	GCAGGAATCGTTCTCTGCTT	60	220	--	--	--	--	--	--
TR28608|c1_g1_i4	No	(CTGATC)5	30	--	--	--	--	--	--	GGTGCTTTACACGGTGGACT	60	GCAGGAATCGTTCTCTGCTT	60	220	--	--	--	--	--	--
TR28609|c4_g1_i4	XLOC_008859	(TA)6ttatttttgttatttgaataaaaattgaa(T)11	52	--	--	--	--	--	--	GAAAACCTCGGCAATGTGAT	60	AGTTGCCATTTGGAATAGCG	60	274	--	--	--	--	--	--
TR28609|c4_g1_i8	No	(AAC)5	15	--	--	--	--	--	--	CCATCGTCGACCTTGAGAAT	60	TCACACGTACGGGATTTTGA	60	255	--	--	--	--	--	--
TR28609|c4_g1_i8	No	(TA)6ttatttttgttatttgaataaaaattgaa(T)11	52	--	--	--	--	--	--	GAAAACCTCGGCAATGTGAT	60	AGTTGCCATTTGGAATAGCG	60	274	--	--	--	--	--	--
TR28610|c1_g1_i1	No	(GA)7	14	--	--	--	--	--	--	ACACGTTGTTGTTCCGTTTT	58	CAATCCGAAGATTGACTGGC	61	277	--	--	--	--	--	--
TR28610|c1_g1_i2	No	(GA)7	14	--	--	--	--	--	--	ACACGTTGTTGTTCCGTTTT	58	CAATCCGAAGATTGACTGGC	61	277	--	--	--	--	--	--
TR28610|c1_g1_i3	XLOC_008862	(GA)7	14	--	--	--	--	--	--	ACACGTTGTTGTTCCGTTTT	58	GATTTTAACCATCCAGGGGG	60	261	--	--	--	--	--	--
TR28610|c1_g1_i4	No	(GA)7	14	--	--	--	--	--	--	ACACGTTGTTGTTCCGTTTT	58	CAATCCGAAGATTGACTGGC	61	277	--	--	--	--	--	--
TR28610|c1_g1_i5	No	(GA)7	14	--	--	--	--	--	--	ACACGTTGTTGTTCCGTTTT	58	CAATCCGAAGATTGACTGGC	61	277	--	--	--	--	--	--

Transcript IDs	Differentially expressed gene (DEG) IDs	SSR repeat-motifs	SSR repeat-length (bp)	*In silico* polymorphic SSRs						Forward primers (5'-3')	Annealing tempearture(0C)	Reverse primers (5'-3')	Annealing tempearture(0C)	Amplified product size (bp)	Structural annotation		Functional Annotation			
				C. microphyllum		ICC4958									Gene IDs	Sequence component of genes	NCBI nr IDs	TFs	KOG	KEGG
				Transcript	SSR repeat-motifs	Chromosomes	SSR repeat-motifs	Start physical positions (bp)	End physical positions (bp)											
TR28610|c1_g1_i6	No	(GA)7	14	--	--	--	--	--	--	ACACGTTGTTGTTCCGTTTT	58	CAATCCGAAGATTGACTGGC	61	277	--	--	--	--	--	--
TR28610|c1_g1_i7	No	(GA)7	14	--	--	--	--	--	--	ACACGTTGTTGTTCCGTTTT	58	CAATCCGAAGATTGACTGGC	61	277	--	--	--	--	--	--
TR28614|c0_g2_i10	No	(AC)6	12	--	--	--	--	--	--	ACATTTGCTGCAAGGTTTCC	60	TGTGTGCACCTGTTGGTTTT	60	252	--	--	--	--	--	--
TR28614|c0_g2_i17	No	(AC)6	12	--	--	--	--	--	--	ACATTTGCTGCAAGGTTTCC	60	TGTGTGCACCTGTTGGTTTT	60	252	--	--	--	--	--	--
TR28625|c2_g1_i1	No	(AAG)5	15	--	--	--	--	--	--	GACCTCACCACACCCTCTGT	60	CCTTCAAATGAGAATCCCGA	60	268	--	--	--	--	--	--
TR28625|c2_g2_i1	No	(AAG)5	15	--	--	--	--	--	--	GACCTCACCACACCCTCTGT	60	CCTTCAAATGAGAATCCCGA	60	268	--	--	--	--	--	--
TR28625|c2_g3_i1	No	(AAG)5	15	--	--	--	--	--	--	GACCTCACCACACCCTCTGT	60	CCTTCAAATGAGAATCCCGA	60	268	--	--	--	--	--	--
TR28625|c2_g4_i1	No	(AAG)5	15	--	--	--	--	--	--	GACCTCACCACACCCTCTGT	60	CCTTCAAATGAGAATCCCGA	60	268	--	--	--	--	--	--
TR28633|c0_g1_i10	No	(AACT)5	20	--	--	--	--	--	--	TCTTGCAGTTGCAGTTGAGG	60	CGCGTGTAGAAGAGGAGAGG	60	163	--	--	--	--	--	--
TR28635|c0_g1_i1	No	(AG)19	38	--	--	--	--	--	--	CCTGAGAAAAGGAAGATAGAAGAAA	59	GCGGTAGCCGATAGATGAGA	60	243	--	--	--	--	--	--
TR28635|c0_g1_i2	No	(AG)19	38	--	--	--	--	--	--	CCTGAGAAAAGGAAGATAGAAGAAA	59	GCGGTAGCCGATAGATGAGA	60	243	--	--	--	--	--	--
TR28635|c10_g1_i1	No	(TC)10	20	--	--	--	--	--	--	ATCCCATACGACCCAATCAA	60	TCGTTCGAGTTCGATTTTCC	60	213	--	--	--	--	--	--
TR28635|c10_g3_i1	No	(ATT)9	27	--	--	--	--	--	--	GAGCCAACGAAAGTTCCAAA	60	ACCTGAATTTCCTGTGGTCC	59	257	--	--	--	--	--	--
TR28635|c10_g3_i2	No	(ATT)9	27	--	--	--	--	--	--	GAGCCAACGAAAGTTCCAAA	60	ACCTGAATTTCCTGTGGTCC	59	257	--	--	--	--	--	--

Transcript IDs	Differentially expressed gene (DEG) IDs	SSR repeat-motifs	SSR repeat-length (bp)	*In silico* polymorphic SSRs						Forward primers (5'-3')	Annealing tempearture(0C)	Reverse primers (5'-3')	Annealing tempearture(0C)	Amplified product size (bp)	Structural annotation		Functional Annotation			
				C. microphyllum		ICC4958														
				Transcript	SSR repeat-motifs	Chromosomes	SSR repeat-motifs	Start physical positions (bp)	End physical positions (bp)						Gene IDs	Sequence component of genes	NCBI nr IDs	TFs	KOG	KEGG
TR28635|c10_g4_i1	No	(TC)10	20	TR28635|c10_g4_i1	(TC)10	Ca_LG_5	(TC)9	31621837	31621854	ATCCCATACGACCCAATCAA	60	ATTTCGAATTCAGCATTGGC	60	279	Ca_05551	CDS	--	--	--	--
TR28635|c10_g4_i2	No	(TC)10	20	--	--	--	--	--	--	ATCCCATACGACCCAATCAA	60	ATTTCGAATTCAGCATTGGC	60	279	--	--	--	--	--	--
TR28635|c10_g4_i3	No	(TC)10	20	--	--	--	--	--	--	ATCCCATACGACCCAATCAA	60	ATTTCGAATTCAGCATTGGC	60	279	--	--	--	--	--	--
TR28635|c4_g3_i1	No	(TC)10	20	--	--	--	--	--	--	TGGAAGAAAATGGGTAACGG	60	AGCATTCCCACCTCACAGTC	60	225	--	--	--	--	--	--
TR28635|c6_g1_i1	XLOC_008920	(TTG)8	24	--	--	--	--	--	--	GAAACAAGCTGCAGGGAGAG	60	GGTGCTCCAAAGGGTAACAA	60	161	--	--	--	--	--	--
TR28635|c7_g2_i1	No	(CT)6	12	TR28635|c7_g2_i1	(CT)8	Ca_LG_5	(CT)15	38462771	38462800	CACTCCTTCCATCACCACCT	60	TGGAAAGGGAAAAGGGATTT	60	194	Ca_07132	DRR	--	--	--	--
TR28635|c7_g2_i1	No	(CT)8	16	TR28635|c7_g2_i1	(CT)8	Ca_LG_5	(CT)15	38462771	38462800	TCCCTTTTCCCTTTCCACTT	60	CACCCACAAACACGACAAAA	60	255	Ca_07132	DRR	--	--	--	--
TR28635|c7_g2_i1	No	(CT)10	20	TR28635|c7_g2_i1	(CT)8	Ca_LG_5	(CT)15	38462771	38462800	TGTTCTCCGTCCGATCTTTC	60	AGGTGGTGATGGAAGGAGTG	60	198	Ca_07132	DRR	--	--	--	--
TR28635|c7_g2_i2	No	(CT)6	12	--	--	--	--	--	--	CACTCCTTCCATCACCACCT	60	TGGAAAGGGAAAAGGGATTT	60	194	--	--	--	--	--	--
TR28635|c7_g2_i2	No	(CT)8	16	--	--	--	--	--	--	TCCCTTTTCCCTTTCCACTT	60	CACCCACAAACACGACAAAA	60	255	--	--	--	--	--	--
TR28635|c7_g2_i2	No	(CT)10	20	--	--	--	--	--	--	TGTTCTCCGTCCGATCTTTC	60	AGGTGGTGATGGAAGGAGTG	60	198	--	--	--	--	--	--
TR28635|c7_g2_i3	No	(CT)6	12	--	--	--	--	--	--	CACTCCTTCCATCACCACCT	60	TGGAAAGGGAAAAGGGATTT	60	194	--	--	--	--	--	--
TR28635|c7_g2_i3	No	(CT)8	16	--	--	--	--	--	--	TCCCTTTTCCCTTTCCACTT	60	CACCCACAAACACGACAAAA	60	255	--	--	--	--	--	--
TR28635|c7_g2_i3	No	(CT)10	20	--	--	--	--	--	--	TGTTCTCCGTCCGATCTTTC	60	AGGTGGTGATGGAAGGAGTG	60	198	--	--	--	--	--	--

Transcript IDs	Differentially expressed gene (DEG) IDs	SSR repeat-motifs	SSR repeat-length (bp)	*In silico* polymorphic SSRs						Forward primers (5'-3')	Annealing tempearture(0C)	Reverse primers (5'-3')	Annealing tempearture(0C)	Amplified product size (bp)	Structural annotation		Functional Annotation			
				C. microphyllum		ICC4958									Gene IDs	Sequence component of genes	NCBI nr IDs	TFs	KOG	KEGG
				Transcript	SSR repeat-motifs	Chromosomes	SSR repeat-motifs	Start physical positions (bp)	End physical positions (bp)											
TR28635|c8_g1_i1	No	(CAA)5	15	--	--	--	--	--	--	GCAGGAGGGAAGCTGTAGTG	60	CATCATTGCACGTTGTTGGT	60	196	--	--	--	--	--	--
TR28635|c8_g1_i3	No	(GAT)6	18	--	--	--	--	--	--	AGCAGCAACAGCAACTAGCA	60	ACATTGAAACGTTTGCCCTC	60	240	--	--	--	--	--	--
TR28638|c1_g3_i1	No	(AT)12	24	TR28638|c1_g3_i1	(AT)12	Ca_LG_7	(AT)14	3954652	3954679	GTAACCTCGAAAACAGCCGA	60	TGTGCTGCTGCAAATAGCTT	60	109	Ca_15971	DRR	--	--	--	--
TR28651|c0_g1_i1	No	(CA)6	12	--	--	--	--	--	--	AAAGCTGCGAGTAGAATGGC	60	CATTTCAGGGGTAATCTTGTAAAC	58	154	--	--	--	--	--	--
TR28665|c0_g1_i1	No	(CTG)5	15	--	--	--	--	--	--	ACGGATGGACGGATATTGAA	60	ACGCTTCTCCCTGATCCTTA	59	115	--	--	--	--	--	--
TR28701|c0_g1_i1	XLOC_008956	(GTA)5	15	--	--	--	--	--	--	AGTGAAGTGCCTGGGATAGC	59	GATTCACCTTTTCTGGCTGG	60	231	--	--	--	--	--	--
TR28737|c0_g1_i1	XLOC_008971	(CTC)5	15	--	--	--	--	--	--	GGAAGGAACCTGCTCTGTTG	60	AACCAAGGCAACTACGTGCT	60	191	--	--	XP_008893231.1	--	--	--
TR28774|c0_g1_i1	No	(GA)7	14	--	--	--	--	--	--	CCATTGTTTCATTTGTTGCG	60	AGATCCCCTTGGAAAGGCTA	60	184	--	--	--	--	--	--
TR28816|c0_g1_i1	XLOC_008994	(TGT)5	15	--	--	--	--	--	--	CAATGGCTTTGTTTGATCCAT	60	TCCAAACAGGGCCTTATAGC	59	226	--	--	--	--	--	--
TR28817|c0_g1_i1	No	(TGA)5	15	--	--	--	--	--	--	TGAGACGATCACCCAATGAA	60	GGCTGTGGATGCTACTGGAT	60	272	--	--	--	--	--	--
TR28853|c0_g1_i1	No	(TA)6	12	--	--	--	--	--	--	ACGAGGCTTCCTTTTATGAAT	57	TTTCACCCATTTTATACCACACA	59	144	--	--	--	--	--	--
TR28883|c0_g1_i1	XLOC_009017	(TCA)6	18	--	--	--	--	--	--	ATCCTCTGAAGCCACCAAAA	60	CAACATGCCACTTCAACCAC	60	185	--	--	--	--	--	--
TR28898|c0_g1_i1	No	(TTG)5	15	--	--	--	--	--	--	TGTTGGATATAGAAGATTGTTGTGG	59	CCACACTAGGTTTTCCCCTC	58	209	--	--	--	--	--	--
TR28948|c0_g1_i1	XLOC_009023	(AG)6	12	--	--	--	--	--	--	CCTAAGGGGAACACCACTCA	60	GGACTTGAGCGGAAATCAAT	59	253	--	--	--	--	--	--

Transcript IDs	Differentially expressed gene (DEG) IDs	SSR repeat-motifs	SSR repeat-length (bp)	*In silico* polymorphic SSRs						Forward primers (5'-3')	Annealing tempearture(0C)	Reverse primers (5'-3')	Annealing tempearture(0C)	Amplified product size (bp)	Structural annotation		Functional Annotation			
				C. microphyllum		ICC4958														
				Transcript	SSR repeat-motifs	Chromosomes	SSR repeat-motifs	Start physical positions (bp)	End physical positions (bp)						Gene IDs	Sequence component of genes	NCBI nr IDs	TFs	KOG	KEGG
TR28974|c0_g1_i1	XLOC_009029	(CCA)5	15	--	--	--	--	--	--	GTTGTAGCTGGTGCTGCTCA	60	GGGTGGTGCTGAGTGAGTTT	60	255	--	--	--	--	--	--
TR28983|c0_g1_i1	XLOC_009032	(TTC)5	15	--	--	--	--	--	--	TTTGGCAACAACTGGTGGTA	60	CGATGCCGATATTTTGGAAG	60	247	--	--	--	--	--	--
TR29027|c0_g1_i1	XLOC_009043	(CT)7	14	--	--	--	--	--	--	TCTCAAACCAATAATAACAAACAACA	59	GCAAATCATTGTCTTCGGGT	60	259	--	--	--	--	--	--
TR29127|c0_g1_i1	No	(TTG)5	15	--	--	--	--	--	--	CTTCTTGGGCTTGCACTTG	60	GGCCCTCCTAAGTACGTTCC	60	204	--	--	--	--	--	--
TR29275|c0_g1_i1	No	(GTC)5	15	--	--	--	--	--	--	TGCTGTGGTGGGTATTGTTG	60	CACAAGCCAAGACTACACGG	59	195	--	--	--	--	--	--
TR29289|c0_g1_i1	XLOC_009119	(GGC)7	21	--	--	--	--	--	--	ACATCAGGGTATTCGTTGGC	60	CGCCGTCCACATACCTAAG	59	261	--	--	NP_001155809.1	--	[O]	--
TR29289|c0_g2_i1	No	(GGC)7	21	--	--	--	--	--	--	ACATCAGGGTATTCGTTGGC	60	CGCCGTCCACATACCTAAG	59	262	--	--	--	--	--	--
TR2929|c0_g3_i1	No	(AC)14	28	--	--	--	--	--	--	TTCTTTGCAAATTCCTAGTTTTCC	60	AGGGACATGTTGACTTGATGG	60	266	--	--	--	--	--	--
TR29334|c1_g1_i1	No	(TC)7	14	--	--	--	--	--	--	AAATGAGCCGTTAGAGGGGT	60	GACAACGCGGGTTTACAGTT	60	116	--	--	--	--	--	--
TR2942|c0_g1_i1	No	(TGA)7	21	--	--	--	--	--	--	TCATTCTCTCCACCACCACA	60	TGACCATGACATGCCTGTTT	60	265	--	--	--	--	--	--
TR2942|c0_g1_i2	No	(TGA)7	21	--	--	--	--	--	--	ACTCCTTCTTCCGCCGTTAT	60	TGACCATGACATGCCTGTTT	60	261	--	--	--	--	--	--
TR2942|c2_g1_i1	XLOC_009202	(TGA)7	21	--	--	--	--	--	--	AAGGCACATCAAACCCTGAC	60	GAACTTTTCCACCACCCTCA	60	228	--	--	--	--	--	--
TR29457|c0_g1_i1	XLOC_009213	(AAT)5	15	--	--	--	--	--	--	TGACCCACAAAGCTCATCAA	60	ATCGTTTTGCCCAAATGAAG	60	271	--	--	--	--	--	--
TR29464|c0_g1_i1	No	(TA)6	12	TR29464|c0_g1_i1	(TA)6	Ca_LG_8	(TA)9	7589091	7589108	TAGAGCTTCGGATGTTGCAG	59	AGAGTCAACAGATCCCCACG	60	113	Ca_19630	DRR	--	--	--	--

Transcript IDs	Differentially expressed gene (DEG) IDs	SSR repeat-motifs	SSR repeat-length (bp)	*In silico* polymorphic SSRs						Forward primers (5'-3')	Annealing tempearture(0C)	Reverse primers (5'-3')	Annealing tempearture(0C)	Amplified product size (bp)	Structural annotation		Functional Annotation			
				C. microphyllum		ICC4958									Gene IDs	Sequence component of genes	NCBI nr IDs	TFs	KOG	KEGG
				Transcript	SSR repeat-motifs	Chromosomes	SSR repeat-motifs	Start physical positions (bp)	End physical positions (bp)											
TR29527|c0_g1_i1	No	(TGT)5	15	--	--	--	--	--	--	TACCCCATAACGGTTCCTCA	60	TCAGACGATACCACCCATGA	60	241	--	--	--	--	--	--
TR29590|c0_g1_i1	No	(CAG)5	15	--	--	--	--	--	--	CTGCTTTGTTCTTTCTGGGC	60	TGCCGTTGTTCAGAAGTTGA	60	221	--	--	--	--	--	--
TR2962|c0_g1_i1	XLOC_009300	(TGTC)5	20	--	--	--	--	--	--	ACGCTCCACAAACACAACAC	60	TTGTGGCAAAATTCAGCAAG	60	226	--	--	--	--	--	--
TR29738|c0_g1_i1	XLOC_009337	(AT)9	18	--	--	--	--	--	--	AGCCACACACATAGTTGCAGA	59	AGGAGCGTATGAGCCTTTGA	60	180	--	--	XP_004502568.1	--	[S]	--
TR29738|c0_g2_i1	No	(AT)9	18	--	--	--	--	--	--	AGCCACACACATAGTTGCAGA	59	AGGAGCGTATGAGCCTTTGA	60	180	--	--	--	--	--	--
TR298|c1_g1_i1	XLOC_009394	(AG)6	12	--	--	--	--	--	--	TGAAACCCTAACCCTAATCCC	59	GATGGTGCTGTGAAATGTGC	60	110	--	--	--	--	--	--
TR29823|c0_g1_i1	No	(CT)9aaatcttcatcccctttctctcactacacccactccactctccctctctcgacgtcaataacgggtctccattaataca(CT)7	111	--	--	--	--	--	--	TGAGCATTCGGGTAATTTGA	59	AGTGGGGATGAGAATGGATG	60	198	--	--	--	--	--	--
TR29823|c0_g2_i1	No	(CT)9aaatcttcatcccctttctctcactacacccactccactctccctctctcgacgtcaataacgggtctccattaataca(CT)7	111	--	--	--	--	--	--	TGAGCATTCGGGTAATTTGA	59	AGTGGGGATGAGAATGGATG	60	198	--	--	--	--	--	--
TR2991|c0_g2_i1	XLOC_009400	(TC)7	14	--	--	--	--	--	--	ACATTTCCTGCCCACAATTT	59	GGCAGCAACAAAGTGAACTG	59	211	--	--	--	--	--	--
TR30036|c0_g1_i1	No	(TA)7	14	TR30036|c0_g1_i1	(TA)7	Ca_LG_5	(TA)8	26116604	26116619	GAAACCAAAGGCACCAATTT	59	CCTGCTTGTTTGTTCCGTTT	60	235	Ca_05005	CDS	--	--	--	--
TR3006|c0_g2_i1	No	(AG)8	16	--	--	--	--	--	--	CAAAAGACAATTTAGCAGCAACA	59	GCTTGTTGATTGATAGGGTGC	60	246	--	--	--	--	--	--
TR3006|c0_g2_i2	No	(AG)8	16	--	--	--	--	--	--	CAAAAGACAATTTAGCAGCAACA	59	GCTTGTTGATTGATAGGGTGC	60	246	--	--	--	--	--	--
TR30078|c1_g1_i1	No	(AC)7	14	--	--	--	--	--	--	TGTTTTGGATACCCATCATGT	58	CCGTGGAGACCGTTACATCT	60	163	--	--	--	--	--	--
TR30106|c0_g1_i1	No	(CA)6	12	--	--	--	--	--	--	CTCGGCCAAGTATGCCTAAG	60	GAGTGAGAACTTTGCACCGA	59	260	--	--	--	--	--	--

Transcript IDs	Differentially expressed gene (DEG) IDs	SSR repeat-motifs	SSR repeat-length (bp)	*In silico* polymorphic SSRs						Forward primers (5'-3')	Annealing tempearture(0C)	Reverse primers (5'-3')	Annealing tempearture(0C)	Amplified product size (bp)	Structural annotation		Functional Annotation			
				C. microphyllum		ICC4958														
				Transcript	SSR repeat-motifs	Chromosomes	SSR repeat-motifs	Start physical positions (bp)	End physical positions (bp)						Gene IDs	Sequence component of genes	NCBI nr IDs	TFs	KOG	KEGG
TR30129|c0_g1_i1	XLOC_009512	(TTC)7	21	--	--	--	--	--	--	ACAATTCATCTTTAGGCTTCTCA	57	AAATCTCCAGTTTCCCCACA	59	201	--	--	XP_008183165.1	MYB_related	[O]	--
TR30145|c0_g1_i1	No	(AGC)5	15	--	--	--	--	--	--	GTGCAGAAGAGTTGCCATCA	60	CGCAACAAACACTGCTCCTA	60	151	--	--	--	--	--	--
TR30145|c0_g1_i1	No	(TGT)5	15	--	--	--	--	--	--	GTGCAGAAGAGTTGCCATCA	60	TCGTTGTTGTTGGGTGGATA	60	251	--	--	--	--	--	--
TR30158|c0_g1_i1	No	(TA)7	14	--	--	--	--	--	--	TGATCATGCAGCACTATTCACA	60	TGCAACAAACAAGAATCCAAA	59	279	--	--	--	--	--	--
TR3018|c0_g1_i1	No	(CCT)6	18	--	--	--	--	--	--	CCAATGAATCACCACTTCCC	60	GATCAGAATTGCACCCATCC	60	165	--	--	--	--	--	--
TR3018|c0_g1_i1	No	(CT)8ccctctccctctcccagtgtgtgtgtacacaaacaaatccacgatttcataatattctttcacaggcaacgagtggtgggtgaaggaagaagtaagcatc(CT)8	132	--	--	--	--	--	--	GCCTCTCTCTCTTCAATACATTATCC	60	CCAAGGAAACTGGAACCTGA	60	270	--	--	--	--	--	--
TR3018|c0_g1_i10	No	(CCT)6	18	--	--	--	--	--	--	CCAATGAATCACCACTTCCC	60	TGTAACCTCAGGGGTCTTGG	60	236	--	--	--	--	--	--
TR3018|c0_g1_i10	No	(CT)8	16	--	--	--	--	--	--	GCCTCTCTCTCTTCAATACATTATCC	60	CCCCCTAAATCCCATGAAGT	60	244	--	--	--	--	--	--
TR3018|c0_g1_i11	No	(CT)8ccctctccctctcccagtgtgtgtgtacacaaacaaatccacgatttcataatattctttcacaggcaacgagtggtgggtgaaggaagaagtaagcatc(CT)8	132	--	--	--	--	--	--	GCCTCTCTCTCTTCAATACATTATCC	60	CCAAGGAAACTGGAACCTGA	60	270	--	--	--	--	--	--
TR3018|c0_g1_i2	No	(CT)8ccctctccctctcccagtgtgtgtgtacacaaacaaatccacgatttcataatattctttcacaggcaacgagtggtgggtgaaggaagaagtaagcatc(CT)8	132	--	--	--	--	--	--	GCCTCTCTCTCTTCAATACATTATCC	60	CCAAGGAAACTGGAACCTGA	60	270	--	--	--	--	--	--
TR3018|c0_g1_i3	No	(CCT)6	18	--	--	--	--	--	--	CCAATGAATCACCACTTCCC	60	GATCAGAATTGCACCCATCC	60	165	--	--	--	--	--	--
TR3018|c0_g1_i3	No	(CT)8ccctctccctctcccagtgtgtgtgtacacaaacaaatccacgatttcataatattctttcacaggcaacgagtggtgggtgaaggaagaagtaagcatc(CT)8	132	--	--	--	--	--	--	GCCTCTCTCTCTTCAATACATTATCC	60	CCAAGGAAACTGGAACCTGA	60	270	--	--	--	--	--	--
TR3018|c0_g1_i4	No	(CCT)6	18	--	--	--	--	--	--	CCAATGAATCACCACTTCCC	60	TGTAACCTCAGGGGTCTTGG	60	236	--	--	--	--	--	--
TR3018|c0_g1_i5	No	(CCT)6	18	--	--	--	--	--	--	CCAATGAATCACCACTTCCC	60	TGTAACCTCAGGGGTCTTGG	60	236	--	--	--	--	--	--

Transcript IDs	Differentially expressed gene (DEG) IDs	SSR repeat-motifs	SSR repeat-length (bp)	*In silico* polymorphic SSRs						Forward primers (5'-3')	Annealing tempearture(0C)	Reverse primers (5'-3')	Annealing tempearture(0C)	Amplified product size (bp)	Structural annotation		Functional Annotation			
				C. microphyllum		ICC4958														
				Transcript	SSR repeat-motifs	Chromosomes	SSR repeat-motifs	Start physical positions (bp)	End physical positions (bp)						Gene IDs	Sequence component of genes	NCBI nr IDs	TFs	KOG	KEGG
TR3018|c0_g1_i5	No	(CT)8	16	--	--	--	--	--	--	GCCTCTCTCTCTTCAATACATTATCC	60	CCCCCTAAATCCCATGAAGT	60	244	--	--	--	--	--	--
TR3018|c0_g1_i6	No	(CT)8	16	--	--	--	--	--	--	GCCTCTCTCTCTTCAATACATTATCC	60	GGTGAAAGGGATTCCCCTAA	60	146	--	--	--	--	--	--
TR3018|c0_g1_i7	No	(CCT)6	18	--	--	--	--	--	--	CCAATGAATCACCACTTCCC	60	GATCAGAATTGCACCCATCC	60	165	--	--	--	--	--	--
TR3018|c0_g1_i7	No	(CT)8	16	--	--	--	--	--	--	GCCTCTCTCTCTTCAATACATTATCC	60	GGTGAAAGGGATTCCCCTAA	60	146	--	--	--	--	--	--
TR3018|c0_g1_i8	No	(CCT)6	18	--	--	--	--	--	--	CCAATGAATCACCACTTCCC	60	GATCAGAATTGCACCCATCC	60	165	--	--	--	--	--	--
TR3018|c0_g1_i8	No	(CT)8	16	--	--	--	--	--	--	GCCTCTCTCTCTTCAATACATTATCC	60	GGTGAAAGGGATTCCCCTAA	60	146	--	--	--	--	--	--
TR3018|c0_g1_i9	No	(CCT)6	18	--	--	--	--	--	--	CCAATGAATCACCACTTCCC	60	TGTAACCTCAGGGGTCTTGG	60	236	--	--	--	--	--	--
TR30245|c0_g1_i1	No	(TGG)5	15	--	--	--	--	--	--	ATGGCGTCACTCTGGGATAG	60	GAACTTATTCCCGCACCAGA	60	272	--	--	--	--	--	--
TR3025|c0_g3_i1	XLOC_009542	(CAA)5	15	--	--	--	--	--	--	ATGCCAAACACACATATCGG	59	CCATTGCCATGCCTAAACTC	60	116	--	--	--	--	--	--
TR30253|c0_g1_i1	No	(GA)6	12	--	--	--	--	--	--	AGGGTATCTCAACGCAATGG	60	ACTCATTCCCACCTGAGCAC	60	171	--	--	--	--	--	--
TR30270|c0_g1_i1	No	(ATG)7	21	--	--	--	--	--	--	CATGATGAAGATGGTCGTGG	60	TATGCGGTGGTCATAGTGGA	60	129	--	--	--	--	--	--
TR30304|c0_g1_i1	No	(GA)6	12	--	--	--	--	--	--	CATGTTGACAATTTTGAGCCA	60	AAAGAGGAACGCAAGAACCA	60	237	--	--	--	--	--	--
TR30310|c0_g1_i1	XLOC_009554	(TA)8	16	--	--	--	--	--	--	GGGTCATCGAAGCTTTAGCA	60	TTTTATGGGCAGTCTCACCC	60	184	--	--	--	--	--	--
TR30325|c0_g1_i1	XLOC_009566	(CTTT)5	20	--	--	--	--	--	--	ATCGGTCCTTCAAAAACACG	60	TGGAGAAGAAGAGCTTGGGA	60	232	--	--	--	--	--	--

Transcript IDs	Differentially expressed gene (DEG) IDs	SSR repeat-motifs	SSR repeat-length (bp)	*In silico* polymorphic SSRs						Forward primers (5'-3')	Annealing tempearture(0C)	Reverse primers (5'-3')	Annealing tempearture(0C)	Amplified product size (bp)	Structural annotation		Functional Annotation			
				C. microphyllum		ICC4958														
				Transcript	SSR repeat-motifs	Chromosomes	SSR repeat-motifs	Start physical positions (bp)	End physical positions (bp)						Gene IDs	Sequence component of genes	NCBI nr IDs	TFs	KOG	KEGG
TR30460|c0_g1_i1	XLOC_009618	(TG)6	12	--	--	--	--	--	--	TGGGTTTGAGTCATTGGGTT	60	GCGCGATTATGGTCAAGATT	60	173	--	--	XP_004490219.1	--	[T]	--
TR30465|c0_g1_i1	XLOC_009619	(CAT)5	15	--	--	--	--	--	--	TCGTTGGTGACGGTAGTCAA	60	CCATTGTCCAGACCTTCGTT	60	181	--	--	--	--	--	--
TR3052|c0_g1_i1	XLOC_009629	(TA)9	18	--	--	--	--	--	--	CTGCATCTGCTGCTTTTGAA	60	AATGTTTTGGTGAAATTCAATTAG ATA	59	269	--	--	--	--	--	--
TR30583|c0_g1_i1	No	(TAT)6	18	--	--	--	--	--	--	GCATCAGGATCATATACATAACTA GC	58	GCCATCTTCAAAGTCAAGGC	60	152	--	--	--	--	--	--
TR30583|c0_g2_i1	No	(TAT)6	18	--	--	--	--	--	--	GCATCAGGATCATATACATAACTA GC	58	GCCATCTTCAAAGTCAAGGC	60	152	--	--	--	--	--	--
TR30615|c0_g1_i1	No	(AAC)6	18	TR30615|c0_g1_i1	(AAC)6	Ca_LG_6	(AAC)5	10477001	10477015	GCAAAATACAGATGCAGAGGG	60	TGAAGCAATTTGAGGCAAGA	60	232	Ca_13914	CDS	--	--	--	--
TR30615|c0_g2_i1	No	(AAC)6	18	--	--	--	--	--	--	GCAAAATACAGATGCAGAGGG	60	TGAAGCAATTTGAGGCAAGA	60	233	--	--	--	--	--	--
TR30645|c0_g1_i1	XLOC_009661	(CAT)6	18	--	--	--	--	--	--	CCTTCCCACCAATCATCATC	60	TCTGATGTTGCCATGGATGT	60	146	--	--	--	--	--	--
TR30648|c0_g1_i1	No	(GGA)5	15	--	--	--	--	--	--	GTTCTGTGTCGGGTTGTTG	57	TATCCCAGTCCAAGTCGAGG	60	131	--	--	--	--	--	--
TR30663|c0_g1_i1	No	(CTC)7	21	--	--	--	--	--	--	AACCACACCGCAACCAGTA	60	ATAGCCTCAACCGCTTTCCT	60	180	--	--	--	--	--	--
TR30728|c0_g1_i1	XLOC_009676	(GCC)7	21	--	--	--	--	--	--	ATCTTCTCTGACCTGCTGGG	59	CGACCTTGAGTAGCACCGAG	61	103	--	--	--	--	--	--
TR30759|c0_g1_i1	No	(GCT)6	18	--	--	--	--	--	--	GCAGAGCCCATAAATTTCCA	60	TCACCACTCCCTTGAAATCC	60	226	--	--	--	--	--	--
TR30763|c0_g1_i1	XLOC_009691	(GGA)5	15	--	--	--	--	--	--	CAACTCCACCTGCAATTCCT	60	GTCTGGCTCTTCACCGTCTC	60	179	--	--	--	--	--	--
TR30809|c0_g1_i1	XLOC_009706	(CGG)7	21	--	--	--	--	--	--	AGGGGCGGAAGAAAATAAAA	60	ACCAGCAGCGGCTTCAAT	62	242	--	--	--	--	--	--

Transcript IDs	Differentially expressed gene (DEG) IDs	SSR repeat-motifs	SSR repeat-length (bp)	*In silico* polymorphic SSRs						Forward primers (5'-3')	Annealing tempearture(0C)	Reverse primers (5'-3')	Annealing tempearture(0C)	Amplified product size (bp)	Structural annotation		Functional Annotation			
				C. microphyllum		ICC4958									Gene IDs	Sequence component of genes	NCBI nr IDs	TFs	KOG	KEGG
				Transcript	SSR repeat-motifs	Chromosomes	SSR repeat-motifs	Start physical positions (bp)	End physical positions (bp)											
TR30815|c0_g1_i1	No	(GTT)6	18	--	--	--	--	--	--	TTTGATTCATGAGGGTGAAGG	60	TCCACCCTTACACAAAACCAC	60	100	--	--	--	--	--	--
TR31051|c0_g1_i1	No	(AAG)5	15	--	--	--	--	--	--	CGTTGCCGTTGATTTCTCTA	59	CGCTACACGTTTTCAGACCA	60	117	--	--	--	--	--	--
TR31064|c0_g1_i1	No	(AGA)6	18	--	--	--	--	--	--	GGAGGCAAAAGAGTGTGTGTG	61	TTAGCCTCGCTACGACGTTT	60	244	--	--	--	--	--	--
TR31064|c0_g2_i1	No	(AGA)6	18	--	--	--	--	--	--	GGAGGCAAAAGAGTGTGTGTG	61	TTAGCCTCGCTACGACGTTT	60	244	--	--	--	--	--	--
TR31088|c0_g1_i1	No	(CT)8	16	--	--	--	--	--	--	TCCCCGTAAATTCTGACAGC	60	TGAGGCAGCTTGTGTTAGGA	60	128	--	--	--	--	--	--
TR31088|c0_g2_i1	XLOC_009782	(CT)8	16	--	--	--	--	--	--	TCCCCGTAAATTCTGACAGC	60	TGAGGCAGCTTGTGTTAGGA	60	128	--	--	--	--	--	--
TR31098|c0_g1_i1	No	(TAA)6	18	--	--	--	--	--	--	TCGCACAGGTGCTAACACAG	61	TGGTCAATGCATAGCATCAAT	59	229	--	--	--	--	--	--
TR311|c0_g1_i1	XLOC_009824	(CAG)7	21	--	--	--	--	--	--	TGATGATGGGTTTTTGGTCA	60	CAAATCAACAGCACCACCAC	60	265	--	--	--	--	--	--
TR31121|c0_g1_i1	XLOC_009798	(AAG)5	15	--	--	--	--	--	--	TTGGCGATGAAACTGATGAA	60	ACATGATGCATTTGCTTGGA	60	132	--	--	--	--	--	--
TR31168|c0_g1_i1	No	(GT)6	12	--	--	--	--	--	--	TGTGTTCAGGCTGTCGTCTC	60	GAAATCAACGGAACGACGAT	60	217	--	--	--	--	--	--
TR31196|c0_g1_i1	No	(AT)16	32	--	--	--	--	--	--	TGAATTAAGTTGTTGATGTGGCA	60	CCTTCTTTTCTTCCCCCAAC	60	171	--	--	--	--	--	--
TR31322|c0_g1_i1	XLOC_009865	(TA)9	18	--	--	--	--	--	--	TGTTAAGCAATCATCCACCG	60	GCTGTGATTCATTATCGACCAA	60	279	--	--	--	--	--	--
TR31402|c0_g1_i1	No	(TG)6	12	--	--	--	--	--	--	TGCAAATCTTGCTTGCGATA	60	CACTACAAGCATGCAAGACCA	60	222	--	--	--	--	--	--
TR31402|c0_g2_i1	No	(TG)6	12	--	--	--	--	--	--	TGCAAATCTTGCTTGCGATA	60	CACTACAAGCATGCAAGACCA	60	222	--	--	--	--	--	--

Transcript IDs	Differentially expressed gene (DEG) IDs	SSR repeat-motifs	SSR repeat-length (bp)	*In silico* polymorphic SSRs						Forward primers (5'-3')	Annealing tempearture(0C)	Reverse primers (5'-3')	Annealing tempearture(0C)	Amplified product size (bp)	Structural annotation		Functional Annotation			
				C. microphyllum		ICC4958									Gene IDs	Sequence component of genes	NCBI nr IDs	TFs	KOG	KEGG
				Transcript	SSR repeat-motifs	Chromosomes	SSR repeat-motifs	Start physical positions (bp)	End physical positions (bp)											
TR31403|c0_g1_i1	No	(TC)10	20	--	--	--	--	--	--	CAAATGAAGATGCCCTCAAA	59	GCTTCACTCACTTGAGCCTTT	59	220	--	--	--	--	--	--
TR31408|c0_g1_i1	XLOC_009898	(ATC)5	15	--	--	--	--	--	--	TTATCAGAAGACGCACGACG	60	ATCTGGTGGCTTTTGCATTC	60	203	--	--	--	--	--	--
TR31411|c0_g1_i1	No	(GA)9	18	--	--	--	--	--	--	GGGTCATCTCGTGCATGTATT	60	ATCCCCCTTGTCATTTTTCC	60	280	--	--	--	--	--	--
TR31411|c0_g2_i1	No	(GA)9	18	--	--	--	--	--	--	GGGTCATCTCGTGCATGTATT	60	ATCCCCCTTGTCATTTTTCC	60	280	--	--	--	--	--	--
TR31413|c0_g1_i1	No	(AC)9	18	TR31413|c0_g1_i1	(AC)9	Ca_LG_8	(AC)11	7196901	7196922	CAATTTCTGCCGTGCAACTA	60	TGTTTGATCCACCTTTGGACT	59	133	Ca_19261	DRR	--	--	--	--
TR31413|c0_g2_i1	No	(AC)9	18	--	--	--	--	--	--	CAATTTCTGCCGTGCAACTA	60	TGTTTGATCCACCTTTGGACT	59	133	--	--	--	--	--	--
TR31423|c0_g1_i1	No	(GTT)5	15	--	--	--	--	--	--	AATTACGCGTAGTGTGTGCG	60	CAAGAGCCAATTCTCTTCCG	60	245	--	--	--	--	--	--
TR31423|c0_g1_i1	No	(GTT)6	18	--	--	--	--	--	--	CGGAAGAGAATTGGCTCTTG	60	CCAGTACAACGCATTACCTCAA	60	122	--	--	--	--	--	--
TR31485|c0_g1_i1	XLOC_009922	(AT)6	12	--	--	--	--	--	--	AGCTAAAGCTACCACCGCCT	60	GGAACAGAGGCACCATGAAT	60	232	--	--	XP_003628784.1	--	[Q]	--
TR31593|c0_g1_i1	No	(GCA)6gcg(GCA)5	36	--	--	--	--	--	--	CGCAATCATTGGTCAACAAC	60	CGGATACCGTTGAGTGAGGT	60	218	--	--	--	--	--	--
TR31636|c0_g1_i1	XLOC_009957	(GTG)5	15	--	--	--	--	--	--	TCCGAAAACGACAAACACAA	60	TCGACAACACTCTTCCAACG	60	279	--	--	ELU38034.1	--	--	--
TR31689|c2_g2_i1	No	(T)10(A)10	20	--	--	--	--	--	--	GGAGTTTGGGGAGATGGATT	60	AGACAAGGGCAAATTTGGAA	60	188	--	--	--	--	--	--
TR31689|c2_g2_i3	No	(T)10(A)10	20	--	--	--	--	--	--	GGAGTTTGGGGAGATGGATT	60	AGACAAGGGCAAATTTGGAA	60	188	--	--	--	--	--	--
TR31693|c1_g2_i2	No	(TAT)7	21	--	--	--	--	--	--	TTTGAGGAACTCCATTTGGG	60	TAAGTGCTATTAAAATGCAGTAAATGT	57	199	--	--	--	--	--	--

Transcript IDs	Differentially expressed gene (DEG) IDs	SSR repeat-motifs	SSR repeat-length (bp)	*In silico* polymorphic SSRs						Forward primers (5'-3')	Annealing tempearture(0C)	Reverse primers (5'-3')	Annealing tempearture(0C)	Amplified product size (bp)	Structural annotation		Functional Annotation			
				C. microphyllum		ICC4958														
				Transcript	SSR repeat-motifs	Chromosomes	SSR repeat-motifs	Start physical positions (bp)	End physical positions (bp)						Gene IDs	Sequence component of genes	NCBI nr IDs	TFs	KOG	KEGG
TR31693|c1_g2_i3	No	(TAT)7	21	--	--	--	--	--	--	TTTGAGGAACTCCATTTGGG	60	TAAGTGCTATTAAAATGCAGTAAATGT	57	199	--	--	--	--	--	--
TR31693|c1_g2_i6	No	(TAT)7	21	--	--	--	--	--	--	TTTGAGGAACTCCATTTGGG	60	TAAGTGCTATTAAAATGCAGTAAATGT	57	199	--	--	--	--	--	--
TR31693|c3_g2_i1	No	(AGA)6	18	--	--	--	--	--	--	TCATCGGAAATTCATGCAAC	59	GTGATGATGGAGGAGGAGGA	60	275	--	--	--	--	--	--
TR31693|c3_g2_i10	No	(AGA)6	18	--	--	--	--	--	--	TCATCGGAAATTCATGCAAC	59	GTGATGATGGAGGAGGAGGA	60	275	--	--	--	--	--	--
TR31693|c3_g2_i11	No	(AGA)6	18	--	--	--	--	--	--	TCATCGGAAATTCATGCAAC	59	GTGATGATGGAGGAGGAGGA	60	275	--	--	--	--	--	--
TR31693|c3_g2_i12	No	(AGA)6	18	--	--	--	--	--	--	TGGTCATTGGAAATTCATGC	59	GTGATGATGGAGGAGGAGGA	60	278	--	--	--	--	--	--
TR31693|c3_g2_i13	No	(AGA)6	18	--	--	--	--	--	--	TCATCGGAAATTCATGCAAC	59	GTGATGATGGAGGAGGAGGA	60	275	--	--	--	--	--	--
TR31693|c3_g2_i14	No	(AGA)6	18	--	--	--	--	--	--	TGGTCATTGGAAATTCATGC	59	GTGATGATGGAGGAGGAGGA	60	278	--	--	--	--	--	--
TR31693|c3_g2_i15	No	(AGA)6	18	--	--	--	--	--	--	TTTTGGCATCAGGAAATTCA	59	GGAGGAGGAGGAGGTTGTTC	60	273	--	--	--	--	--	--
TR31693|c3_g2_i16	No	(AGA)6	18	--	--	--	--	--	--	TTTTGGCATCAGGAAATTCA	59	GGAGGAGGAGGAGGTTGTTC	60	273	--	--	--	--	--	--
TR31693|c3_g2_i17	No	(AGA)6	18	--	--	--	--	--	--	TTTTGGCATCAGGAAATTCA	59	GGAGGAGGAGGAGGTTGTTC	60	273	--	--	--	--	--	--
TR31693|c3_g2_i18	No	(AGA)6	18	--	--	--	--	--	--	TTTTGGCATCAGGAAATTCA	59	GGAGGAGGAGGAGGTTGTTC	60	273	--	--	--	--	--	--
TR31693|c3_g2_i19	No	(AGA)6	18	--	--	--	--	--	--	TGGTCATTGGAAATTCATGC	59	GTGATGATGGAGGAGGAGGA	60	278	--	--	--	--	--	--
TR31693|c3_g2_i2	No	(AGA)6	18	--	--	--	--	--	--	TTTTGGCATCAGGAAATTCA	59	GGAGGAGGAGGAGGTTGTTC	60	273	--	--	--	--	--	--

Transcript IDs	Differentially expressed gene (DEG) IDs	SSR repeat-motifs	SSR repeat-length (bp)	*In silico* polymorphic SSRs						Forward primers (5'-3')	Annealing tempearture(0C)	Reverse primers (5'-3')	Annealing tempearture(0C)	Amplified product size (bp)	Structural annotation		Functional Annotation			
				C. microphyllum		ICC4958									Gene IDs	Sequence component of genes	NCBI nr IDs	TFs	KOG	KEGG
				Transcript	SSR repeat-motifs	Chromosomes	SSR repeat-motifs	Start physical positions (bp)	End physical positions (bp)											
TR31693|c3_g2_i21	No	(TA)7	14	--	--	--	--	--	--	AAAATGGAGATCTTACTCAGGCA	59	AGGCAGCGAAATCAGAGAAA	60	177	--	--	--	--	--	--
TR31693|c3_g2_i22	No	(AGA)6	18	--	--	--	--	--	--	TTTTGGCATCAGGAAATTCA	59	GGAGGAGGAGGAGGTTGTTC	60	273	--	--	--	--	--	--
TR31693|c3_g2_i3	No	(AGA)6	18	--	--	--	--	--	--	TCATCGGAAATTCATGCAAC	59	GTGATGATGGAGGAGGAGGA	60	275	--	--	--	--	--	--
TR31693|c3_g2_i4	No	(AGA)6	18	--	--	--	--	--	--	TCATCGGAAATTCATGCAAC	59	GTGATGATGGAGGAGGAGGA	60	275	--	--	--	--	--	--
TR31693|c3_g2_i5	No	(AGA)6	18	--	--	--	--	--	--	TGGTCATTGGAAATTCATGC	59	GTGATGATGGAGGAGGAGGA	60	278	--	--	--	--	--	--
TR31693|c3_g2_i6	No	(AGA)6	18	--	--	--	--	--	--	TCATCGGAAATTCATGCAAC	59	GTGATGATGGAGGAGGAGGA	60	275	--	--	--	--	--	--
TR31693|c3_g2_i7	No	(AGA)6	18	--	--	--	--	--	--	TCATCGGAAATTCATGCAAC	59	GTGATGATGGAGGAGGAGGA	60	275	--	--	--	--	--	--
TR31693|c3_g2_i8	No	(AGA)6	18	--	--	--	--	--	--	TGGTCATTGGAAATTCATGC	59	GTGATGATGGAGGAGGAGGA	60	278	--	--	--	--	--	--
TR31693|c3_g2_i9	No	(AGA)6	18	--	--	--	--	--	--	TCATCGGAAATTCATGCAAC	59	GTGATGATGGAGGAGGAGGA	60	275	--	--	--	--	--	--
TR31693|c4_g1_i1	XLOC_010008	(AAT)7	21	--	--	--	--	--	--	ATCCAAAGGGATAGGATCGC	60	GATTCTTCTTCTTCGACACCG	59	143	--	--	--	--	--	--
TR31693|c5_g1_i1	XLOC_010009	(TC)6	12	--	--	--	--	--	--	CACGCACCCAGAATTGTATG	60	AGGACAGCAGTGGTTTCTGC	60	200	--	--	--	--	--	--
TR31694|c5_g1_i10	No	(AAT)6	18	--	--	--	--	--	--	CATGAAAGGAAAAACCCTGC	60	CCCCTGCAGTGGAAGATAAA	60	211	--	--	--	--	--	--
TR31694|c5_g1_i2	No	(AAT)6	18	--	--	--	--	--	--	CATGAAAGGAAAAACCCTGC	60	CCCCTGCAGTGGAAGATAAA	60	211	--	--	--	--	--	--
TR31694|c5_g1_i4	No	(AAT)6	18	--	--	--	--	--	--	CATGAAAGGAAAAACCCTGC	60	CCCCTGCAGTGGAAGATAAA	60	211	--	--	--	--	--	--

Transcript IDs	Differentially expressed gene (DEG) IDs	SSR repeat-motifs	SSR repeat-length (bp)	*In silico* polymorphic SSRs						Forward primers (5'-3')	Annealing tempearture(0C)	Reverse primers (5'-3')	Annealing tempearture(0C)	Amplified product size (bp)	Structural annotation		Functional Annotation			
				C. microphyllum		ICC4958									Gene IDs	Sequence component of genes	NCBI nr IDs	TFs	KOG	KEGG
				Transcript	SSR repeat-motifs	Chromosomes	SSR repeat-motifs	Start physical positions (bp)	End physical positions (bp)											
TR31694|c5_g1_i6	No	(AAT)6	18	--	--	--	--	--	--	CATGAAAGGAAAAACCCTGC	60	CCCCTGCAGTGGAAGATAAA	60	211	--	--	--	--	--	--
TR31694|c5_g1_i7	No	(AAT)6	18	--	--	--	--	--	--	CATGAAAGGAAAAACCCTGC	60	CCCCTGCAGTGGAAGATAAA	60	211	--	--	--	--	--	--
TR31694|c5_g1_i8	No	(AAT)6	18	--	--	--	--	--	--	CATGAAAGGAAAAACCCTGC	60	CCCCTGCAGTGGAAGATAAA	60	211	--	--	--	--	--	--
TR31695|c1_g2_i7	No	(ATA)6	18	--	--	--	--	--	--	CCAACTTTTCCTTCCATCTCA	59	GTTTCTGGCAGCATTGGATT	60	102	--	--	--	--	--	--
TR31697|c1_g1_i1	XLOC_010018	(TC)6	12	--	--	--	--	--	--	CGCATCTCAAGCTTTCTCCT	60	CCACCTTCATCCATTATCGG	60	191	--	--	--	--	--	--
TR31705|c2_g2_i2	No	(TAC)6	18	--	--	--	--	--	--	AACCTCACCAATCAAAACGC	60	ACCGGAACATTGAAGTTTGC	60	254	--	--	--	--	--	--
TR31705|c2_g2_i3	No	(TAC)6	18	--	--	--	--	--	--	AACCTCACCAATCAAAACGC	60	ACCGGAACATTGAAGTTTGC	60	254	--	--	--	--	--	--
TR31705|c2_g2_i5	No	(TAC)6	18	--	--	--	--	--	--	AACCTCACCAATCAAAACGC	60	ACCGGAACATTGAAGTTTGC	60	254	--	--	--	--	--	--
TR31708|c0_g3_i1	XLOC_010062	(AG)8	16	--	--	--	--	--	--	GGTCTAACCCGTCCATTCAA	60	GGACTTGCTTTTGCAGGGTA	60	251	--	--	--	--	--	--
TR31720|c0_g1_i1	No	(AT)6	12	--	--	--	--	--	--	TAGTTGGAATGCTCCAATGC	59	TGGACGCAGTGACCTATCAA	60	219	--	--	--	--	--	--
TR31727|c1_g4_i7	XLOC_010119	(TCC)8	24	--	--	--	--	--	--	GAGACATCATTGCCTTGGCT	60	TAGTAATGGGTCTTTGCGCC	60	246	--	--	XP_004495225.1	bHLH	[S]	--
TR31733|c2_g1_i1	XLOC_010138	(TC)8	16	--	--	--	--	--	--	ACAGCATAGACGAGGCCAGT	60	TGACGAGTCATTGAAATTGAAAA	60	131	--	--	--	--	--	--
TR31733|c5_g1_i4	No	(GTA)5	15	--	--	--	--	--	--	CATGAGAGTGTGGAAGCCAA	60	TTTCATGTTTCCAGCAGCAG	60	228	--	--	--	--	--	--
TR31733|c5_g1_i6	No	(GTA)5	15	--	--	--	--	--	--	CATGAGAGTGTGGAAGCCAA	60	TTTCATGTTTCCAGCAGCAG	60	228	--	--	--	--	--	--

Transcript IDs	Differentially expressed gene (DEG) IDs	SSR repeat-motifs	SSR repeat-length (bp)	*In silico* polymorphic SSRs						Forward primers (5'-3')	Annealing tempearture(0C)	Reverse primers (5'-3')	Annealing tempearture(0C)	Amplified product size (bp)	Structural annotation		Functional Annotation			
				C. microphyllum		ICC4958									Gene IDs	Sequence component of genes	NCBI nr IDs	TFs	KOG	KEGG
				Transcript	SSR repeat-motifs	Chromosomes	SSR repeat-motifs	Start physical positions (bp)	End physical positions (bp)											
TR31733|c5_g1_i7	No	(GTA)5	15	--	--	--	--	--	--	CATGAGAGTGTGGAAGCCAA	60	TTTCATGTTTCCAGCAGCAG	60	228	--	--	--	--	--	--
TR31733|c6_g1_i1	XLOC_010140	(TCT)7	21	TR31733|c6_g1_i1	(TCT)7	Ca_LG_7	(TCT)8	40577997	40578020	GCATTCGTCACTCTTCGTGA	60	AGAAGAAGTAGTGGTGGCGG	59	272	Ca_18613	URR	--	--	--	--
TR31735|c0_g2_i1	XLOC_010149	(CT)6	12	--	--	--	--	--	--	CTTGAAGACATTGCGTTGACA	60	CCACATCATGTTCACGGTTC	60	141	--	--	--	--	--	--
TR31735|c0_g2_i2	XLOC_010150	(GGA)10	30	--	--	--	--	--	--	AGTCATGGGCCAAAATGTTC	60	AGGACTACGAACCCAGCAAG	59	234	--	--	--	--	--	--
TR31741|c0_g2_i1	No	(CA)6	12	--	--	--	--	--	--	ACATGGGTTAAGCAAGACCG	60	AACTCCCGAGCATGCTATTC	59	139	--	--	--	--	--	--
TR31741|c5_g1_i1	XLOC_010178	(TC)11ttcctatgttactatctttcttc(TG)8	61	--	--	--	--	--	--	TTGTCCTGCCCAATGATGTA	60	GTTTGTCCAAAGCCCTCAGA	60	262	--	--	XM_004516568.1	--	--	--
TR31741|c6_g1_i1	XLOC_010179	(TA)6	12	--	--	--	--	--	--	TCCTCTTTCAAAACCCCTCA	60	CAACAAGCCTAGCCAAAAGG	60	269	--	--	--	--	--	--
TR31742|c0_g1_i1	No	(A)11cttcttccccaaatatcgttttgaatttctt(A)10	52	--	--	--	--	--	--	TCCTTGAACCCTACTCCGTT	59	CGTGTTTGGTAGTGGTGATGA	59	279	--	--	--	--	--	--
TR31742|c0_g1_i2	No	(A)10cttcttccccaaatatcgttttgaatttctt(A)10	51	--	--	--	--	--	--	TCCCGATATCTTCTTCCTCAA	59	CGTGTTTGGTAGTGGTGATGA	59	119	--	--	--	--	--	--
TR31742|c0_g1_i3	XLOC_010182	(A)11cttcttccccaaatatcgttttgaatttctt(A)10	52	--	--	--	--	--	--	TCCCGATATCTTCTTCCTCAA	59	CGTGTTTGGTAGTGGTGATGA	59	120	--	--	--	--	--	--
TR31747|c0_g2_i1	No	(CAT)5	15	--	--	--	--	--	--	TGGTCCTAGTCCGTCCCATA	60	AAACTTGCAGGCGATCCTAA	60	189	--	--	--	--	--	--
TR31747|c0_g2_i2	No	(CAT)5	15	--	--	--	--	--	--	AAAAATTACTGCCCACACGG	60	GATTGCCCAAGCTGCTAGTC	60	242	--	--	--	--	--	--
TR31747|c0_g2_i3	No	(CAT)5	15	--	--	--	--	--	--	AAAAATTACTGCCCACACGG	60	GATTGCCCAAGCTGCTAATC	60	246	--	--	--	--	--	--
TR31747|c0_g2_i4	No	(CAT)5	15	--	--	--	--	--	--	TGGTCCTAGTCCGTCCCATA	60	AAACTTGCAGGCGATCCTAA	60	189	--	--	--	--	--	--

Transcript IDs	Differentially expressed gene (DEG) IDs	SSR repeat-motifs	SSR repeat-length (bp)	*In silico* polymorphic SSRs						Forward primers (5'-3')	Annealing tempearture(0C)	Reverse primers (5'-3')	Annealing tempearture(0C)	Amplified product size (bp)	Structural annotation		Functional Annotation			
				C. microphyllum		ICC4958														
				Transcript	SSR repeat-motifs	Chromosomes	SSR repeat-motifs	Start physical positions (bp)	End physical positions (bp)						Gene IDs	Sequence component of genes	NCBI nr IDs	TFs	KOG	KEGG
TR31747|c0_g2_i5	XLOC_010195	(CAT)5	15	--	--	--	--	--	--	AAAAATTACTGCCCACACGG	60	GATTGCCCAAGCTGCTAGTC	60	242	--	--	--	--	--	--
TR31747|c0_g2_i6	No	(CAT)5	15	--	--	--	--	--	--	TGGTCCTAGTCCGTCCCATA	60	AAACTTGCAGGCGATCCTAA	60	189	--	--	--	--	--	--
TR31747|c0_g2_i7	XLOC_010196	(CAT)5	15	--	--	--	--	--	--	AAAAATTACTGCCCACACGG	60	GATTGCCCAAGCTGCTAATC	60	246	--	--	--	--	--	--
TR31747|c0_g2_i8	No	(CAT)5	15	--	--	--	--	--	--	AAAAATTACTGCCCACACGG	60	GATTGCCCAAGCTGCTAGTC	60	242	--	--	--	--	--	--
TR31747|c0_g2_i9	No	(CAT)5	15	--	--	--	--	--	--	AAAAATTACTGCCCACACGG	60	GATTGCCCAAGCTGCTAGTC	60	242	--	--	--	--	--	--
TR31748|c0_g1_i1	No	(TC)10	20	--	--	--	--	--	--	CAATCCCTCCTCTCTCACCA	60	CCATTGTCAGGGGAAAAAGA	60	270	--	--	--	--	--	--
TR31748|c0_g1_i2	No	(TC)10	20	--	--	--	--	--	--	CAATCCCTCCTCTCTCACCA	60	CCATTGTCAGGGGAAAAAGA	60	270	--	--	--	--	--	--
TR31757|c0_g1_i1	XLOC_010214	(ATG)5	15	--	--	--	--	--	--	GCAGCACTTAAAGAGGGCAC	60	TAGCCAACCCAACAAAAAGG	60	272	--	--	--	--	--	--
TR31758|c2_g1_i2	No	(AG)7	14	TR31758|c2_g1_i2	(AG)7	scaffold02471	(AG)8	24477	24492	GCAAATTCATTATCATCCAGCA	60	TTCACTTTGCTCACCAACCA	60	261	Ca_21836	CDS	--	--	--	--
TR31758|c2_g1_i3	No	(AG)7	14	--	--	--	--	--	--	GCAAATTCATTATCATCCAGCA	60	TTCACTTTGCTCACCAACCA	60	261	--	--	--	--	--	--
TR31758|c3_g2_i1	No	(CAA)6	18	--	--	--	--	--	--	AACCAAATGCCACCAAGAAG	60	CCGAGAAAGGACCAGTAATCTG	60	171	--	--	--	--	--	--
TR31758|c3_g2_i7	No	(CAA)6	18	--	--	--	--	--	--	AACCAAATGCCACCAAGAAG	60	CCGAGAAAGGACCAGTAATCTG	60	171	--	--	--	--	--	--
TR31761|c0_g1_i1	XLOC_010225	(TTG)6	18	TR31761|c0_g1_i1	(TTG)6	Ca_LG_5	(TTG)5	8596744	8596758	TGGAGGCACGTAATGAGGTA	59	TTGGGGTCTCAAATACTCGG	60	110	Ca_08201	DRR	--	--	--	--
TR31772|c3_g4_i6	No	(CTAA)6	24	--	--	--	--	--	--	GCAAACACTGCCCAAAGATT	60	GTCGATTGCATGCAGCTTT	60	191	--	--	--	--	--	--

Transcript IDs	Differentially expressed gene (DEG) IDs	SSR repeat-motifs	SSR repeat-length (bp)	*In silico* polymorphic SSRs						Forward primers (5'-3')	Annealing tempearture(0C)	Reverse primers (5'-3')	Annealing tempearture(0C)	Amplified product size (bp)	Structural annotation		Functional Annotation			
				C. microphyllum		ICC4958									Gene IDs	Sequence component of genes	NCBI nr IDs	TFs	KOG	KEGG
				Transcript	SSR repeat-motifs	Chromosomes	SSR repeat-motifs	Start physical positions (bp)	End physical positions (bp)											
TR31773|c0_g2_i7	XLOC_010268	(A)10caaaaaaagtcaattgtgacttgtgagtaggatctgctcttttgtagtgcaggaaatt(TTA)5ctattatcttag(T)14	109	--	--	--	--	--	--	CTAAGGTCGTCAAGGGTGGA	60	CACCCACACGCAGAGATAAA	60	222	--	--	--	--	--	--
TR31776|c0_g1_i1	XLOC_010273	(AG)6aaaaaagagagagaagaa(AG)7	44	--	--	--	--	--	--	GATGGTTCAACAAATCAGCAAA	60	AGCTGCTAACTCCCCCTAGC	60	125	--	--	--	--	--	--
TR31780|c0_g2_i3	No	(AT)8	16	--	--	--	--	--	--	CAAGGGTTAAATTCTTCATTCCA	59	AAGTAGGGCAGGGAAAAGGA	60	254	--	--	--	--	--	--
TR31780|c0_g2_i4	No	(AT)8	16	--	--	--	--	--	--	CAAGGGTTAAATTCTTCATTCCA	59	AAGTAGGGCAGGGAAAAGGA	60	254	--	--	--	--	--	--
TR31781|c4_g1_i1	No	(TA)7	14	--	--	--	--	--	--	AAATCCACAACTCGACCACA	59	TGCAGGGTATACCAACATGG	59	257	--	--	--	--	--	--
TR31781|c4_g1_i2	No	(TA)7	14	--	--	--	--	--	--	TTGAAAGCACAAATCTGTTGG	59	GCAGGGTGTAACAACATGGA	59	198	--	--	--	--	--	--
TR31781|c4_g1_i5	XLOC_010301	(TA)7	14	--	--	--	--	--	--	TTGAAAGCACAAATCTGTTGG	59	GCAGGGTATACCAACATGGAA	60	198	--	--	--	--	--	--
TR31784|c1_g1_i1	No	(AG)9	18	--	--	--	--	--	--	GTCACCGCAGATCCAGAAGA	61	GGAGGAGGAGAAGTCTGCAA	60	132	--	--	--	--	--	--
TR31845|c0_g1_i1	XLOC_010334	(CTT)5	15	--	--	--	--	--	--	TAGTCTCCTTAGCGGCAGGA	60	GAGAAGCCAACTGAAAACGC	60	106	--	--	--	--	--	--
TR3187|c0_g1_i1	No	(ATT)5	15	--	--	--	--	--	--	ACGCTGTACTCTGCCAACCT	60	TCCCCTATTTTCTTTTTGCG	59	279	--	--	--	--	--	--
TR31873|c0_g1_i1	XLOC_010340	(GA)7	14	--	--	--	--	--	--	TTTGGCCTGTAGTGTAGAAGTCA	59	CCCAGGTGAAGCATCATTTT	60	271	--	--	--	--	--	--
TR32018|c0_g1_i1	No	(ACC)6	18	--	--	--	--	--	--	AATCCACCATGGAAGACACC	60	TCCTCCACGTCAGAATCTCC	60	103	--	--	--	--	--	--
TR3209|c0_g1_i1	XLOC_010378	(AG)8	16	--	--	--	--	--	--	ATTGGGTTATTGGTGTTGGG	59	GTAGCTCCCCAGTTTTGCAG	60	199	--	--	--	--	--	--
TR3209|c0_g1_i1	XLOC_010378	(TGC)7	21	--	--	--	--	--	--	TGCAAATGGTGAGGATTTTG	60	CATGGAAGCCAATGGAATTT	60	132	--	--	--	--	--	--

Transcript IDs	Differentially expressed gene (DEG) IDs	SSR repeat-motifs	SSR repeat-length (bp)	*In silico* polymorphic SSRs						Forward primers (5'-3')	Annealing tempearture(0C)	Reverse primers (5'-3')	Annealing tempearture(0C)	Amplified product size (bp)	Structural annotation		Functional Annotation			
				C. microphyllum		**ICC4958**									**Gene IDs**	**Sequence component of genes**	**NCBI nr IDs**	**TFs**	**KOG**	**KEGG**
				**Transcript**	**SSR repeat-motifs**	**Chromosomes**	**SSR repeat-motifs**	**Start physical positions (bp)**	**End physical positions (bp)**											
TR32139|c0_g1_i1	No	(A)10caatatcaaaatacaaaatgcttatttagtaaactatt gtcgcaac(A)10	66	--	--	--	--	--	--	CATTACCGGGACATCAGCTT	60	CAGCTCTTCCAATAACCCCA	60	241	--	--	--	--	--	--
TR32155|c0_g1_i1	No	(GT)6	12	--	--	--	--	--	--	ACGAACCACTTCATCTCGCT	60	TATAACGTGTACGTTCCGGG	58	134	--	--	--	--	--	--
TR32169|c0_g1_i1	XLOC_010399	(CAT)5ccactaaatacaagaatctaagggg(A)10	50	--	--	--	--	--	--	TGGATTCTATTCCAAGCCCA	60	GCAACGCCTGCATTTTATTT	60	159	--	--	--	--	--	--
TR32200|c0_g1_i1	XLOC_010411	(CA)7	14	--	--	--	--	--	--	CCATGGACAACAGCTCAAAA	60	TGATGGCTTAGTGGAGCTTG	59	210	--	--	--	--	--	--
TR32273|c1_g1_i1	No	(TG)9	18	--	--	--	--	--	--	CAGCTGCTGCTTCTACATGCT	61	GCAGAATGGGCCTTTCAGTA	60	193	--	--	--	--	--	--
TR32335|c0_g1_i1	XLOC_010453	(GAC)5	15	--	--	--	--	--	--	TGAGCAATGACTGGGTAGCA	60	ACAAATCTATTATGCGGCGG	60	116	--	--	--	--	--	--
TR32356|c0_g1_i1	No	(CGG)5	15	--	--	--	--	--	--	GTACGGGTACGGATACGACG	60	GCACAGCCATACTACTCGCA	60	222	--	--	--	--	--	--
TR32452|c0_g1_i1	No	(ATG)5	15	--	--	--	--	--	--	TGCACGAGGGTTTTCTTCAT	61	TTTCCAAAATTCGGCACTTC	60	213	--	--	--	--	--	--
TR32461|c0_g1_i1	XLOC_010535	(TTC)5	15	--	--	--	--	--	--	ACTTCAACGAATCACCGACC	60	CCACCAGTCTTTTGAACGGT	60	122	--	--	XP_004507965.1	SBP	[L]	--
TR32473|c0_g1_i1	No	(GTT)5	15	--	--	--	--	--	--	CACGTGTGATGGACTATTGGA	59	GGTCCCTTCAACCTCAAACA	60	124	--	--	--	--	--	--
TR32479|c0_g1_i1	XLOC_010555	(ACA)5	15	--	--	--	--	--	--	CTTATTCAACCGTGGTGGCT	60	GTTCGTTCATGTTGACGGTG	60	231	--	--	--	--	--	--
TR32500|c0_g1_i1	XLOC_010577	(AG)6	12	--	--	--	--	--	--	TTGGAGAGGAGGACCAAATG	60	CCCAACGCCACTAACTTCAT	60	186	--	--	--	--	--	--
TR32503|c0_g1_i1	No	(AT)6	12	--	--	--	--	--	--	ATGGTGAAACTTTATGCGCC	60	TTTCTTTCCTTGCAGATGCC	60	211	--	--	--	--	--	--
TR32503|c0_g1_i3	No	(AT)6	12	--	--	--	--	--	--	ATGGTGAAACTTTATGCGCC	60	TTTCTTTCCTTGCAGATGCC	60	211	--	--	--	--	--	--

Transcript IDs	Differentially expressed gene (DEG) IDs	SSR repeat-motifs	SSR repeat-length (bp)	*In silico* polymorphic SSRs						Forward primers (5'-3')	Annealing tempearture(0C)	Reverse primers (5'-3')	Annealing tempearture(0C)	Amplified product size (bp)	Structural annotation		Functional Annotation			
				C. microphyllum		ICC4958									Gene IDs	Sequence component of genes	NCBI nr IDs	TFs	KOG	KEGG
				Transcript	SSR repeat-motifs	Chromosomes	SSR repeat-motifs	Start physical positions (bp)	End physical positions (bp)											
TR32601|c0_g1_i1	XLOC_010657	(AAG)5	15	--	--	--	--	--	--	ACCGCCGTTTATTTCCTTTT	60	TCGATCGCTGTTTGTTGAAG	60	257	--	--	--	--	--	--
TR32629|c0_g1_i1	No	(AAG)6aatagtaccaac(ACA)5	45	--	--	--	--	--	--	TGAGGAATCCTTCGTCAACA	59	CTCCAACGACCTTCACCTTC	60	196	--	--	--	--	--	--
TR32661|c0_g1_i1	No	(CGA)5	15	--	--	--	--	--	--	TTTCGTGGGTTTTAACTCGG	60	AAGAGACCGTGGAACTGCTG	60	176	--	--	--	--	--	--
TR328|c0_g2_i1	XLOC_010749	(TCT)6	18	--	--	--	--	--	--	CATTTGTAAAAGACACCACTCTCA	58	TCACTTCTGTTGCTGCCATC	60	132	--	--	--	--	--	--
TR3280|c0_g1_i1	No	(CTC)5	15	--	--	--	--	--	--	CTCTTATGTCCCCCGCATTA	60	GATGATGATGAAGGGATGGG	60	160	--	--	--	--	--	--
TR32868|c0_g1_i1	No	(CAA)10	30	--	--	--	--	--	--	GACCATTTGTTTGGGCATTT	60	GTTTTGGAAAGGGGAAAGGA	60	162	--	--	--	--	--	--
TR32868|c0_g1_i3	No	(CAA)10	30	--	--	--	--	--	--	GACCATTTGTTTGGGCATTT	60	GTTTTGGAAAGGGGAAAGGA	60	162	--	--	--	--	--	--
TR32868|c0_g1_i4	No	(CAA)10	30	--	--	--	--	--	--	GACCATTTGTTTGGGCATTT	60	GTTTTGGAAAGGGGAAAGGA	60	162	--	--	--	--	--	--
TR32868|c0_g1_i5	No	(CAA)10	30	--	--	--	--	--	--	GACCATTTGTTTGGGCATTT	60	GTTTTGGAAAGGGGAAAGGA	60	162	--	--	--	--	--	--
TR32868|c0_g1_i6	No	(CAA)10	30	--	--	--	--	--	--	GACCATTTGTTTGGGCATTT	60	GTTTTGGAAAGGGGAAAGGA	60	162	--	--	--	--	--	--
TR32869|c1_g1_i1	XLOC_010713	(CAA)6	18	--	--	--	--	--	--	CACCTAATTGAATCCCCCAA	60	ACCACCATAAAGTGAGTGAAGAC	57	147	--	--	--	--	--	--
TR32869|c1_g3_i2	XLOC_010715	(CA)11	22	--	--	--	--	--	--	GGTTGCAGGAATTGGAGAAA	60	CAAATTCACCACCATTAATACATCA	60	218	--	--	--	--	--	--
TR32869|c1_g5_i1	No	(CAA)6	18	--	--	--	--	--	--	CACCTAATTGAATCCCCCAA	60	CATTGCTAGTGCTGCTTCCA	60	166	--	--	--	--	--	--
TR32869|c2_g1_i1	XLOC_010716	(TC)6tatttctctctgtgactctttcttcttcat(CAA)8	66	--	--	--	--	--	--	CCCTTACTAGTCGCGCAAAG	60	ATTGGGAATGGCCAATTGTA	60	263	--	--	--	--	--	--

Transcript IDs	Differentially expressed gene (DEG) IDs	SSR repeat-motifs	SSR repeat-length (bp)	*In silico* polymorphic SSRs						Forward primers (5'-3')	Annealing tempearture(0C)	Reverse primers (5'-3')	Annealing tempearture(0C)	Amplified product size (bp)	Structural annotation		Functional Annotation			
				C. microphyllum		ICC4958									Gene IDs	Sequence component of genes	NCBI nr IDs	TFs	KOG	KEGG
				Transcript	SSR repeat-motifs	Chromosomes	SSR repeat-motifs	Start physical positions (bp)	End physical positions (bp)											
TR32869|c2_g1_i1	XLOC_010716	(CT)6	12	--	--	--	--	--	--	CGCGACGTTTACTTCTCCTC	60	ATGGAGTAAACGGCAACAGG	60	143	--	--	--	--	--	--
TR32888|c0_g1_i1	No	(GTG)6	18	--	--	--	--	--	--	CCTTTACTTCCGTAACCGCA	60	CCTGAACAGCACAGCTTTTG	60	187	--	--	--	--	--	--
TR32888|c0_g1_i1	No	(AAC)6	18	--	--	--	--	--	--	GTCGCTCGTGTTCTTCTTCC	60	TGCGGTTACGGAAGTAAAGG	60	177	--	--	--	--	--	--
TR32888|c0_g1_i2	No	(GTG)6	18	--	--	--	--	--	--	CCTTTACTTCCGTAACCGCA	60	CCTGAACAGCACAGCTTTTG	60	187	--	--	--	--	--	--
TR32888|c0_g1_i2	No	(AAC)6	18	--	--	--	--	--	--	GTCGCTCGTGTTCTTCTTCC	60	TGCGGTTACGGAAGTAAAGG	60	177	--	--	--	--	--	--
TR32888|c0_g1_i3	No	(GTG)6	18	--	--	--	--	--	--	CCTTTACTTCCGTAACCGCA	60	CCTGAACAGCACAGCTTTTG	60	187	--	--	--	--	--	--
TR32888|c0_g1_i3	No	(AAC)6	18	--	--	--	--	--	--	GTCGCTCGTGTTCTTCTTCC	60	TGCGGTTACGGAAGTAAAGG	60	177	--	--	--	--	--	--
TR32893|c0_g2_i1	No	(AG)6	12	--	--	--	--	--	--	GCGGACTAGGGTGAGGAGTT	61	TACTCTCCGTTTCACACCCC	60	197	--	--	--	--	--	--
TR32917|c0_g1_i1	XLOC_010763	(GAT)5	15	--	--	--	--	--	--	AAATGGTCCCTTCTGTTCCA	59	GACAAATCCGCTGGTAGCAT	60	161	--	--	--	--	--	--
TR32917|c0_g1_i2	XLOC_010764	(GAT)5	15	--	--	--	--	--	--	AAATGGTCCCTTCTGTTCCA	59	GACAAATCCGCTGGTAGCAT	60	160	--	--	--	--	--	--
TR32930|c0_g1_i2	No	(AT)6	12	--	--	--	--	--	--	GCAATCGATAACCTGGCAAT	60	ATGCATTGCTGTCACTTTCG	60	274	--	--	--	--	--	--
TR32930|c0_g2_i1	No	(GA)7	14	TR32930|c0_g2_i1	(GA)9	Ca_LG_6	(GA)11	49581910	49581931	GGAGTGGGAGAATTGCATGT	60	TTCACTGCACCGCTCCTTA	61	217	Ca_12715	DRR	--	--	--	--
TR32930|c0_g2_i1	No	(GA)9	18	TR32930|c0_g2_i1	(GA)9	Ca_LG_6	(GA)11	49581910	49581931	TCCACAAAAACAAACACGGA	60	ACATGCAATTCTCCCACTCC	60	128	Ca_12715	DRR	--	--	--	--
TR32931|c0_g2_i1	XLOC_010778	(CA)6	12	--	--	--	--	--	--	AAGATGGAAGAGTGGGAGACA	59	CTGCTAGTGTGCACGTGTGT	59	108	--	--	--	--	--	--

Transcript IDs	Differentially expressed gene (DEG) IDs	SSR repeat-motifs	SSR repeat-length (bp)	*In silico* polymorphic SSRs						Forward primers (5'-3')	Annealing tempearture(0C)	Reverse primers (5'-3')	Annealing tempearture(0C)	Amplified product size (bp)	Structural annotation		Functional Annotation			
				C. microphyllum		ICC4958														
				Transcript	SSR repeat-motifs	Chromosomes	SSR repeat-motifs	Start physical positions (bp)	End physical positions (bp)						Gene IDs	Sequence component of genes	NCBI nr IDs	TFs	KOG	KEGG
TR32934|c0_g1_i1	No	(GT)6	12	--	--	--	--	--	--	TGGTCATCTTTGCTTGCATC	60	ACGACAGCAAAAAGAGCCAG	61	102	--	--	--	--	--	--
TR32934|c0_g1_i4	No	(GT)6	12	--	--	--	--	--	--	TGGTCATCTTTGCTTGCATC	60	ACGACAGCAAAAAGAGCCAG	61	102	--	--	--	--	--	--
TR32939|c0_g1_i1	No	(TA)7	14	--	--	--	--	--	--	GCTTCATCCATCTCTGCCTT	59	AAGCCAAGCATCAGACTTGC	61	229	--	--	--	--	--	--
TR32939|c0_g2_i1	No	(TA)7	14	--	--	--	--	--	--	GCTTCATCCATCTCTGCCTT	59	AAGCCAAGCATCAGACTTGC	61	229	--	--	--	--	--	--
TR32944|c0_g1_i3	No	(AC)10	20	--	--	--	--	--	--	CAAGGGTATCCGTGCTTCAT	60	ATCCTCCATTGTGGGTGTGT	60	109	--	--	--	--	--	--
TR32944|c0_g1_i6	No	(AC)10	20	--	--	--	--	--	--	CAAGGGTATCCGTGCTTCAT	60	ATCCTCCATTGTGGGTGTGT	60	109	--	--	--	--	--	--
TR32945|c0_g3_i3	No	(AT)8	16	TR32945|c0_g3_i3	(AT)8	Ca_LG_5	(AT)7	34826372	34826385	AAGTCCTGGTGCTGGTCACT	60	ATTGGAAATCGTCTTTGGCA	60	126	Ca_05906	CDS	--	--	--	--
TR32946|c1_g1_i1	XLOC_010798	(CTT)5	15	--	--	--	--	--	--	GTGGAGATGGAAGGTGGAGA	60	TTGTCCATCCATGCCAGTAA	60	259	--	--	--	--	--	--
TR32949|c0_g2_i1	No	(CTT)6	18	--	--	--	--	--	--	ACACGTGACTAAGACGCAGC	59	ACTTCTTGAGCGCGTTGTTT	60	271	--	--	--	--	--	--
TR32949|c0_g2_i2	No	(CTT)6	18	--	--	--	--	--	--	ACACGTGACTAAGACGCAGC	59	ACTTCTTGAGCGCGTTGTTT	60	271	--	--	--	--	--	--
TR32949|c0_g2_i3	No	(CTT)6	18	--	--	--	--	--	--	ACACGTGACTAAGACGCAGC	59	ACTTCTTGAGCGCGTTGTTT	60	271	--	--	--	--	--	--
TR32949|c0_g2_i4	No	(CTT)6	18	--	--	--	--	--	--	ACACGTGACTAAGACGCAGC	59	ACTTCTTGAGCGCGTTGTTT	60	271	--	--	--	--	--	--
TR32949|c0_g2_i5	No	(CTT)6	18	--	--	--	--	--	--	ACACGTGACTAAGACGCAGC	59	ACTTCTTGAGCGCGTTGTTT	60	271	--	--	--	--	--	--
TR32949|c0_g2_i6	No	(CTT)6	18	--	--	--	--	--	--	ACACGTGACTAAGACGCAGC	59	ACTTCTTGAGCGCGTTGTTT	60	271	--	--	--	--	--	--

Transcript IDs	Differentially expressed gene (DEG) IDs	SSR repeat-motifs	SSR repeat-length (bp)	*In silico* polymorphic SSRs						Forward primers (5'-3')	Annealing tempearture(0C)	Reverse primers (5'-3')	Annealing tempearture(0C)	Amplified product size (bp)	Structural annotation		Functional Annotation			
				C. microphyllum		ICC4958									Gene IDs	Sequence component of genes	NCBI nr IDs	TFs	KOG	KEGG
				Transcript	SSR repeat-motifs	Chromosomes	SSR repeat-motifs	Start physical positions (bp)	End physical positions (bp)											
TR32950|c0_g1_i1	XLOC_010803	(AAT)11	33	--	--	--	--	--	--	TGTACCGATAAAGAAGAAAACAAGAA	60	GAAGAGAGTCGTTTTTGCCG	60	274	--	--	--	--	--	--
TR32957|c1_g1_i1	XLOC_010811	(CAC)5	15	--	--	--	--	--	--	GTGAGTCCTCCACCACCAGT	60	ATGGTGCATCTGCAATTTGA	60	106	--	--	--	--	--	--
TR32977|c0_g1_i1	XLOC_010831	(ATC)6	18	--	--	--	--	--	--	CCATTGCCTGCATGATTCT	60	GTGATCCAGGATGCCAAGTT	60	212	--	--	--	--	--	--
TR32982|c0_g1_i1	No	(ATTA)5	20	--	--	--	--	--	--	CCTTGCTAACTACGAGGAGGC	60	TGCTTGCAAGTGATTCCATC	60	184	--	--	--	--	--	--
TR32982|c0_g1_i2	No	(ATTA)5	20	--	--	--	--	--	--	CCTTGCTAACTACGAGGAGGC	60	TGCTTGCAAGTGATTCCATC	60	184	--	--	--	--	--	--
TR32987|c0_g1_i1	No	(ACC)5	15	--	--	--	--	--	--	ATCGCCATAAAATCCACGC	61	GGTGGCTTTCTCGGTAAACA	60	102	--	--	--	--	--	--
TR32987|c0_g2_i1	No	(ACC)5	15	--	--	--	--	--	--	ATCGCCATAAAATCCACGC	61	GGTGGCTTTCTCGGTAAACA	60	102	--	--	--	--	--	--
TR32996|c0_g1_i1	XLOC_010846	(AGC)6	18	--	--	--	--	--	--	GAAATGCAACAAGGTGCAGA	60	TAAAGCCACAAGAACCACCC	60	211	--	--	--	--	--	--
TR33003|c0_g1_i1	XLOC_010853	(TGCGTC)5	30	--	--	--	--	--	--	TTCCTCATCCGAGGATGTTC	60	CAATCAACGCACGACAGAGT	60	158	--	--	EUC66730.1	--	--	--
TR33007|c1_g1_i1	No	(TC)8	16	--	--	--	--	--	--	CCTGTGAGTGTGAAGGTTTCTG	60	TGCACATCCCGATCAAATTA	60	129	--	--	--	--	--	--
TR33027|c0_g1_i1	XLOC_010871	(CCA)6	18	--	--	--	--	--	--	CCGAAGAGAGCCAAGTGTTC	60	CTTCAACCTTCTCACGGAGG	60	188	--	--	XP_004501873.1	--	--	--
TR33033|c0_g1_i1	No	(AGC)5	15	--	--	--	--	--	--	TTTCCGTTTCTTTGACTCCG	60	AGTGGCCAAGGAAGATAGCA	60	165	--	--	--	--	--	--
TR33050|c0_g1_i1	XLOC_010886	(AAC)6	18	TR33050|c0_g1_i1	(AAC)6	Ca_LG_6	(AAC)5	26626360	26626374	CCCATACAACCCAAATGGAC	60	ACCCTCAAAACCAACCCAAT	60	277	Ca_15115	CDS	--	--	--	--
TR33080|c0_g1_i1	No	(CAT)5	15	--	--	--	--	--	--	TGATGTGATTTGTGAGCATGAA	60	CAAAGGATTTGGTCCCACTG	60	227	--	--	--	--	--	--

Transcript IDs	Differentially expressed gene (DEG) IDs	SSR repeat-motifs	SSR repeat-length (bp)	*In silico* polymorphic SSRs						Forward primers (5'-3')	Annealing tempearture(0C)	Reverse primers (5'-3')	Annealing tempearture(0C)	Amplified product size (bp)	Structural annotation		Functional Annotation			
				C. microphyllum		ICC4958														
				Transcript	SSR repeat-motifs	Chromosomes	SSR repeat-motifs	Start physical positions (bp)	End physical positions (bp)						Gene IDs	Sequence component of genes	NCBI nr IDs	TFs	KOG	KEGG
TR33095|c0_g1_i1	XLOC_010896	(GTT)6	18	--	--	--	--	--	--	TGACCGAATCCTCCAAAAAG	60	AAAACCAGCAGAAGCAGCAT	60	183	--	--	--	--	--	--
TR33104|c0_g1_i1	No	(CA)6	12	--	--	--	--	--	--	AGACGGTCCTCCTTCCAAGT	60	AGTGCAAGTGCAGCTAATGC	59	265	--	--	--	--	--	--
TR33116|c0_g1_i1	XLOC_010903	(CAT)7	21	--	--	--	--	--	--	GCTTTGTACAACAACAATCATCATC	60	AGGCTGCAATCGATTTGACT	60	221	--	--	--	--	--	--
TR33227|c0_g1_i1	XLOC_010949	(TA)6	12	--	--	--	--	--	--	GCCCATGATGACAATGTCTG	60	TTTGGGAGTGTTTTAGGAACTGA	60	250	--	--	--	--	--	--
TR33230|c0_g1_i1	No	(TCG)5	15	--	--	--	--	--	--	TTGATCTCAATCTTGCGACG	60	CGAATATCGACTCGCAGACA	60	204	--	--	--	--	--	--
TR33326|c0_g1_i1	No	(GTC)5	15	--	--	--	--	--	--	CGTAAACTCTGTTCGCTCCC	60	GACAGAACAACAAGCGCGTA	60	157	--	--	--	--	--	--
TR33333|c0_g1_i1	No	(TC)6	12	--	--	--	--	--	--	TCCATGCTCATTCTCTTCCC	60	GTAGGTAAGGCCAAGGAGGG	60	107	--	--	--	--	--	--
TR33343|c0_g1_i1	No	(TC)7	14	--	--	--	--	--	--	ACGACTGCATTTTCTGACCC	60	AACAGAGCCCTTTTCAGTGC	59	131	--	--	--	--	--	--
TR33343|c0_g2_i1	XLOC_010997	(TC)7	14	--	--	--	--	--	--	ACGACTGCATTTTCTGACCC	60	ATTGTTTCACCAGCAGAGGC	60	205	--	--	--	--	--	--
TR33345|c0_g1_i1	XLOC_010998	(AAG)6	18	--	--	--	--	--	--	CCAAAAATTCCCTTGGAACA	60	CACTCGTTTCCGGTTTCTCT	59	137	--	--	--	--	--	--
TR33354|c0_g1_i1	No	(T)10ctcc(CT)7atagtttgttacatg(TAT)5	58	--	--	--	--	--	--	CAATGGCAGCTCAAGGTACA	60	CAAGACAAAGGAGTGAATGCAA	60	252	--	--	--	--	--	--
TR33354|c0_g2_i1	No	(TCA)5	15	--	--	--	--	--	--	GAGCACACCAATCTTGTGGA	60	CCAGCTGGATACAGCAGCTT	61	226	--	--	--	--	--	--
TR33356|c0_g1_i1	XLOC_011007	(GCA)5gcc(ACA)5	33	--	--	--	--	--	--	TACCGATGACGAAATGGACA	60	TAAGATCGCCATGGATCCTC	60	197	--	--	XP_003049442.1	--	--	--
TR33379|c0_g1_i1	No	(T)10caattatatgtactca(T)13gaattgggatttcaaacaatatatattcataagtttctgcttttaatcttttttgtttggtcttaaaataacgttgttatggccatcataggtattatcc(A)10	149	--	--	--	--	--	--	AAATTCAACTTTTGGGTTCTCC	59	CATAGCTAAAATCAACTACCATGAAA	58	249	--	--	--	--	--	--

Transcript IDs	Differentially expressed gene (DEG) IDs	SSR repeat-motifs	SSR repeat-length (bp)	*In silico* polymorphic SSRs						Forward primers (5'-3')	Annealing tempearture(0C)	Reverse primers (5'-3')	Annealing tempearture(0C)	Amplified product size (bp)	Structural annotation		Functional Annotation			
				C. microphyllum		ICC4958									Gene IDs	Sequence component of genes	NCBI nr IDs	TFs	KOG	KEGG
				Transcript	SSR repeat-motifs	Chromosomes	SSR repeat-motifs	Start physical positions (bp)	End physical positions (bp)											
TR33379|c0_g1_i2	No	(T)10caattatatgtactca(T)13gaattgggatttcaaacaatatatattcataagtttctgcttttaatctttttgtttggtcttaaaataacgttgttatggccatcataggtattatcc(A)10	149	--	--	--	--	--	--	AAATTCAACTTTTGGGTTCTCC	59	CATAGCTAAAATCAACTACCATGAAA	58	249	--	--	--	--	--	--
TR33389|c1_g1_i1	No	(TGA)6	18	--	--	--	--	--	--	TGTGCTGATGATGGTGGTTT	60	CAAACCGGCCAATTCTCTAC	60	101	--	--	--	--	--	--
TR33395|c0_g1_i1	XLOC_011035	(T)12gggtaataactatgtaaaatttactacacctatctcatgctatccacttaaatgataaacatatattgaagctcaata(AT)8	106	--	--	--	--	--	--	TCTCCTGCAAGTCATGAATCC	60	TGGTGTATGGTTTGATTTGTGTG	61	243	--	--	--	--	--	--
TR33400|c0_g1_i1	XLOC_011041	(TC)6	12	--	--	--	--	--	--	CGTTCGGGCACTTTTACTTC	60	TGCTTCCTCCTTTTTCCTTTC	60	207	--	--	--	--	--	--
TR33416|c0_g2_i1	No	(ATTT)5	20	--	--	--	--	--	--	TGAGCTTGTGCATCTGTATCA	58	TTCAGAACGGTCACGAAGTG	60	242	--	--	--	--	--	--
TR33456|c0_g1_i1	No	(GAT)6	18	--	--	--	--	--	--	AACTGTGGAAGGGGAAATGT	58	TCCCTTTGTGAAAATTTTGCTT	60	265	--	--	--	--	--	--
TR33513|c0_g1_i1	No	(CAT)5	15	--	--	--	--	--	--	ATCCTCACCACCTCTCCCTT	60	ATGCAAATGCAAATGCAAGA	60	161	--	--	--	--	--	--
TR33514|c0_g1_i1	No	(TA)6	12	--	--	--	--	--	--	TCACCTCTCTTGTGTATCATTCATT	59	TTGATGTGTCAACGTTCTGTTAGA	60	138	--	--	--	--	--	--
TR33538|c0_g1_i1	No	(TGT)5	15	--	--	--	--	--	--	GTGTTGGGACTGGGATTGTT	60	TCTTCATCATCGGCATTGAA	60	135	--	--	--	--	--	--
TR33546|c0_g1_i1	XLOC_011082	(TC)6	12	--	--	--	--	--	--	GTCGCTAAATACGCTGCTCC	60	CATGAGTTGAGCCTTGGACA	60	215	--	--	--	--	--	--
TR33555|c0_g1_i1	No	(ATC)5	15	--	--	--	--	--	--	CCATTGCCCTTGTTTATGCT	60	TTGACAAAATCACCACCCTG	59	271	--	--	--	--	--	--
TR33562|c0_g1_i1	No	(GA)6	12	--	--	--	--	--	--	AGTCCACCAAGGAATGAGGA	60	ATCTGAATCACTGCCTTCCG	60	165	--	--	--	--	--	--
TR33567|c0_g1_i1	XLOC_011093	(TTG)5	15	--	--	--	--	--	--	TGAGACATTGACGCCATAGG	60	TCTCCACCCTCACCACTACC	60	206	--	--	XP_004498932.1	MYB	[K]	--
TR33691|c0_g1_i1	XLOC_011153	(TC)6	12	--	--	--	--	--	--	TCCCCTCTCACCCTTCTTTT	60	GTGGTTGAGGTTGTGGTTGA	59	180	--	--	--	--	--	--

Transcript IDs	Differentially expressed gene (DEG) IDs	SSR repeat-motifs	SSR repeat-length (bp)	*In silico* polymorphic SSRs						Forward primers (5'-3')	Annealing tempearture(0C)	Reverse primers (5'-3')	Annealing tempearture(0C)	Amplified product size (bp)	Structural annotation		Functional Annotation			
				C. microphyllum		ICC4958														
				Transcript	SSR repeat-motifs	Chromosomes	SSR repeat-motifs	Start physical positions (bp)	End physical positions (bp)						Gene IDs	Sequence component of genes	NCBI nr IDs	TFs	KOG	KEGG
TR33723|c0_g1_i1	No	(AG)6	12	--	--	--	--	--	--	TGACAATGGCTGGATCTACG	60	GGATTCGATCGTCGCTTACA	61	231	--	--	--	--	--	--
TR33756|c0_g1_i1	XLOC_011175	(CGA)5	15	--	--	--	--	--	--	AGCATTCGGAATATCGTTGG	60	GATGCACCGCTAGAAAAGGA	60	192	--	--	KND02974.1	--	--	--
TR33785|c0_g1_i1	XLOC_011189	(AAT)7	21	--	--	--	--	--	--	CCAATTCTAATGGTTTAGGAGGC	60	GGCTGTTGTTGTTGTTGCAT	60	235	--	--	--	--	--	--
TR33795|c0_g1_i1	XLOC_011193	(TA)6	12	--	--	--	--	--	--	AGGATTGCCCACTTCTCCTT	60	TCGTAGCTTCCCTGGGTATG	60	222	--	--	--	--	--	--
TR33812|c0_g1_i1	XLOC_011201	(TTA)6	18	--	--	--	--	--	--	TCCATGGCCATATTTTACCC	59	ATGAGTTGGATTGTCCTGCC	60	185	--	--	XM_004508986.1	MYB_related	--	--
TR33909|c0_g1_i1	XLOC_011242	(TGC)6	18	--	--	--	--	--	--	GCTAAGGATCGTCTTGCTCG	60	ATCCACTGTTTCATCTCGGG	60	198	--	--	--	--	--	--
TR3399|c0_g1_i1	No	(GCA)5	15	--	--	--	--	--	--	CAGTGAGCGGTGTTTCTTCA	60	CCGCTCCATCTACATCTGGT	60	165	--	--	--	--	--	--
TR33992|c0_g1_i1	XLOC_011279	(GTA)6	18	--	--	--	--	--	--	ACCAAAATGCTCGGTGGTAG	60	AAGCCACACTCACAAACAGC	59	184	--	--	--	--	--	--
TR340|c0_g1_i1	No	(AAC)5	15	--	--	--	--	--	--	TTTCTGCAATTACGATGATGC	59	GCCGTTAGTAACACGACGGT	60	232	--	--	--	--	--	--
TR340|c0_g1_i2	No	(AAC)5	15	--	--	--	--	--	--	TTTCTGCAATTACGATGATGC	59	GCCGTTAGTAACACGACGGT	60	232	--	--	--	--	--	--
TR34002|c0_g1_i1	XLOC_011291	(CAA)5	15	--	--	--	--	--	--	GAAACCGAGGTTGATGGAGA	60	TGCAAATTCCAATCCACAAA	60	124	--	--	XP_004497300.1	C2H2	[R]	--
TR34006|c1_g1_i1	XLOC_011295	(ATA)6	18	--	--	--	--	--	--	GGCAAAAGAGCCAGAGAAGA	60	TTACACGCAAACTTTCCTGC	59	259	--	--	--	--	--	--
TR34008|c0_g1_i1	XLOC_011297	(AAG)5	15	--	--	--	--	--	--	CACCATGACCACAAATCTGC	60	TCACTGTCGCTACTGCTGCT	60	209	--	--	--	--	--	--
TR3403|c0_g1_i1	No	(TA)6	12	--	--	--	--	--	--	TTTATAAATTTGCCACTCTAAATGAG	57	AATTAAAAATGGGCTTGTGGG	60	257	--	--	--	--	--	--

Transcript IDs	Differentially expressed gene (DEG) IDs	SSR repeat-motifs	SSR repeat-length (bp)	*In silico* polymorphic SSRs						Forward primers (5'-3')	Annealing tempearture(0C)	Reverse primers (5'-3')	Annealing tempearture(0C)	Amplified product size (bp)	Structural annotation		Functional Annotation			
				C. microphyllum		ICC4958									Gene IDs	Sequence component of genes	NCBI nr IDs	TFs	KOG	KEGG
				Transcript	SSR repeat-motifs	Chromosomes	SSR repeat-motifs	Start physical positions (bp)	End physical positions (bp)											
TR34104|c0_g2_i1	XLOC_011372	(TC)6	12	--	--	--	--	--	--	GTCCTGCTTCCTCAATGCTC	60	TAATATTTGGGCCAGCAAGC	60	120	--	--	--	--	--	--
TR34115|c0_g3_i1	XLOC_011383	(CAC)7	21	--	--	--	--	--	--	TCCCTAATTTCCTGGGGAAC	60	TGGAAGGTTCAGAATTTGGG	60	175	--	--	--	--	--	--
TR34126|c0_g2_i1	XLOC_011393	(CT)11	22	--	--	--	--	--	--	TCTCACTCGAACACGACACC	60	GGGGAAACAAACGAATTTGA	60	150	--	--	--	--	--	--
TR34150|c1_g1_i1	XLOC_011411	(GCC)5	15	--	--	--	--	--	--	CACAACTCATACCCCCAAGG	60	TGGTGGTGACTTCAAAACAGA	59	129	--	--	--	--	--	--
TR34181|c0_g1_i1	XLOC_011424	(AC)6(TC)8	28	--	--	--	--	--	--	GTGCTCAACCGTCTCAACAA	60	ATACGACGACGTTTCGGTTC	60	135	--	--	XP_004486636.1	--	[X]	--
TR34198|c0_g1_i1	XLOC_011429	(AC)7	14	--	--	--	--	--	--	GCAGTTGTAACATTAAGTCATAACA CA	59	TTCACCTCCTTCTCCACCAC	60	116	--	--	--	--	--	--
TR34295|c0_g1_i1	No	(CT)9	18	--	--	--	--	--	--	GCCCCAGTTCTTGTTGGATA	60	GACCGAAGACACCGAAGAAA	60	210	--	--	--	--	--	--
TR34296|c0_g1_i1	No	(TG)8	16	--	--	--	--	--	--	TGGCTTATTTGTTGGCATTG	60	CTCAAGCAACGGCTGACAT	60	103	--	--	--	--	--	--
TR34311|c0_g1_i2	XLOC_011459	(CT)8	16	--	--	--	--	--	--	GCTATTTCTTCGCGCTCTCA	61	ATCCCAACGTAGCCCTTCTT	60	275	--	--	--	--	--	--
TR3435|c0_g1_i1	No	(AAC)5	15	--	--	--	--	--	--	CCACCGGTTGTAACTGAGGT	60	TTTCACTTTCTTCCCATGCC	60	220	--	--	--	--	--	--
TR34362|c0_g2_i1	XLOC_011501	(TC)8	16	--	--	--	--	--	--	GTCAACCTCTCTCTCGCTGG	60	TGGTGTGGCATGGTATAGGA	60	178	--	--	--	--	--	--
TR34398|c1_g1_i1	No	(AT)7	14	--	--	--	--	--	--	CGTGCCCAATACATTTACCC	60	GCAGTACAAATGCCCGTCTT	60	164	--	--	--	--	--	--
TR34637|c0_g1_i1	XLOC_011588	(AT)7	14	--	--	--	--	--	--	GTTCGGTTCAGAATCCGTGT	60	TTCATTTTGGATCGATCGTG	59	137	--	--	--	--	--	--
TR34742|c0_g1_i1	No	(ACAG)6	24	--	--	--	--	--	--	AGCAAACAAGGACAGGAAAGA	59	TCTCGGAACGTTTTTCTGCT	60	213	--	--	--	--	--	--

Transcript IDs	Differentially expressed gene (DEG) IDs	SSR repeat-motifs	SSR repeat-length (bp)	*In silico* polymorphic SSRs						Forward primers (5'-3')	Annealing tempearture(0C)	Reverse primers (5'-3')	Annealing tempearture(0C)	Amplified product size (bp)	Structural annotation		Functional Annotation			
				C. microphyllum		ICC4958														
				Transcript	SSR repeat-motifs	Chromosomes	SSR repeat-motifs	Start physical positions (bp)	End physical positions (bp)						Gene IDs	Sequence component of genes	NCBI nr IDs	TFs	KOG	KEGG
TR34784|c4_g1_i1	No	(TC)8	16	--	--	--	--	--	--	CACTCACTCACCTCATCTTCCA	60	TGAATTCATGGGGTTTGGTT	60	128	--	--	--	--	--	--
TR34784|c4_g1_i4	No	(TC)8	16	--	--	--	--	--	--	CACTCACTCACCTCATCTTCCA	60	TGAATTCATGGGGTTTGGTT	60	128	--	--	--	--	--	--
TR34784|c5_g1_i1	XLOC_011621	(AGTG)6	24	--	--	--	--	--	--	GGTTGCCGGAAAACACTG	60	GAAATCGCCTCGAGATTCAG	60	210	--	--	XP_004516643.1	--	[G]	K03841
TR34784|c5_g2_i1	XLOC_011622	(A)10ttaaagtttttttttcttaa(T)10	39	--	--	--	--	--	--	TGAAACAACAATTTTGGACAATG	60	TGTGTATAACATTACCAATTTGTCAGA	59	279	--	--	XP_004511119.1	--	--	--
TR34789|c0_g1_i1	No	(AT)6ttctctttctctctctccccaatattattaggaaaatatattattact(TA)8	76	--	--	--	--	--	--	CACGTGCTCTCTCCTTCCTC	60	GCATGCAAGTGGCTGTAGAA	60	269	--	--	--	--	--	--
TR34789|c0_g1_i2	No	(AT)6ttctctttctctctctccccaatattattaggaaaatatattattact(TA)8	76	--	--	--	--	--	--	CACGTGCTCTCTCCTTCCTC	60	GCATGCAAGTGGCTGTAGAA	60	269	--	--	--	--	--	--
TR34792|c2_g3_i2	No	(GGT)5	15	--	--	--	--	--	--	TCCGGGACATCGATAGAAAG	60	TATCCGGCATATGCTTCTCC	60	180	--	--	--	--	--	--
TR34794|c1_g1_i1	XLOC_011663	(TCT)6	18	--	--	--	--	--	--	TTGCGGAGACTCCTGAAGAT	60	TGTCGACCTTGTGCTGTTTC	60	231	--	--	--	--	--	--
TR34794|c3_g1_i1	No	(TTC)11	33	--	--	--	--	--	--	CACACCCACCCTAACGTCTT	60	AAAAGCATACCCCAATTCCC	60	255	--	--	--	--	--	--
TR34798|c1_g1_i2	XLOC_011682	(GA)7	14	--	--	--	--	--	--	CCATTTCTTCATAGCGCTCC	60	ACTCAACCTCAACGTCACCC	60	136	--	--	CEL63931.1	--	[O]	--
TR348|c0_g1_i1	No	(TC)20	40	--	--	--	--	--	--	TAATAGCACCACTTTCGCCC	60	AACTGGAAGAAGAGTGCCGA	60	180	--	--	--	--	--	--
TR348|c0_g2_i1	No	(TC)18	36	--	--	--	--	--	--	TAATAGCACCACTTTCGCCC	60	AACTGGAAGAAGAGTGCCGA	60	176	--	--	--	--	--	--
TR348|c0_g3_i1	No	(TC)17	34	--	--	--	--	--	--	TAATAGCACCACTTTCGCCC	60	AACTGGAAGAAGAGTGCCGA	60	174	--	--	--	--	--	--
TR348|c0_g4_i1	No	(TC)21	42	--	--	--	--	--	--	TAATAGCACCACTTTCGCCC	60	AACTGGAAGAAGAGTGCCGA	60	182	--	--	--	--	--	--

Transcript IDs	Differentially expressed gene (DEG) IDs	SSR repeat-motifs	SSR repeat-length (bp)	*In silico* polymorphic SSRs						Forward primers (5'-3')	Annealing tempearture(0C)	Reverse primers (5'-3')	Annealing tempearture(0C)	Amplified product size (bp)	Structural annotation		Functional Annotation			
				C. microphyllum		ICC4958														
				Transcript	SSR repeat-motifs	Chromosomes	SSR repeat-motifs	Start physical positions (bp)	End physical positions (bp)						Gene IDs	Sequence component of genes	NCBI nr IDs	TFs	KOG	KEGG
TR348|c0_g5_i1	No	(TC)19	38	--	--	--	--	--	--	TAATAGCACCACTTTCGCCC	60	AACTGGAAGAAGAGTGCCGA	60	178	--	--	--	--	--	--
TR34803|c2_g1_i1	No	(ATTG)5	20	--	--	--	--	--	--	ACGATCTTGGATTTTCTGCG	60	CGAATCACTTGCATTCTAAACAA	59	223	--	--	--	--	--	--
TR34803|c5_g1_i1	No	(ATA)7	21	--	--	--	--	--	--	GAGAAGTCGTTCACATGAACAATAA	60	ATCTAGTGCCATCTGGCGTC	60	114	--	--	--	--	--	--
TR34803|c5_g1_i2	No	(ATA)7	21	--	--	--	--	--	--	GAGAAGTCGTTCACATGAACAATAA	60	ATCTAGTGCCATCTGGCGTC	60	114	--	--	--	--	--	--
TR34803|c5_g1_i3	No	(ATA)7	21	--	--	--	--	--	--	GAGAAGTCGTTCACATGAACAATAA	60	ATCTAGTGCCATCTGGCGTC	60	114	--	--	--	--	--	--
TR34803|c5_g1_i4	No	(ATA)7	21	--	--	--	--	--	--	GAGAAGTCGTTCACATGAACAATAA	60	ATCTAGTGCCATCTGGCGTC	60	114	--	--	--	--	--	--
TR34804|c0_g6_i1	XLOC_011717	(AAC)5	15	--	--	--	--	--	--	GGGGTTCTGCAATTTCTTTG	60	TTTGGGGGAAGTGAGAGTTG	60	233	--	--	XM_004493836.1	--	[R]	--
TR34805|c1_g4_i2	XLOC_011726	(TGA)5	15	--	--	--	--	--	--	TGCAACTCACCACCACTCTT	59	ACACCACCCATGCCAACTAT	60	177	--	--	--	--	--	--
TR34808|c3_g1_i10	No	(GAA)5	15	--	--	--	--	--	--	TGAAGTTGATGGGGAAGAGG	60	TACCGGGATGACCTCAACAT	60	182	--	--	--	--	--	--
TR34808|c3_g1_i3	No	(GAA)5	15	--	--	--	--	--	--	TGAAGTTGATGGGGAAGAGG	60	CCGGGATGACCTCTACATGA	61	180	--	--	--	--	--	--
TR34808|c3_g1_i8	No	(GAA)5	15	--	--	--	--	--	--	TGAAGTTGATGGGGAAGAGG	60	CCGGGATGACCTCTACATGA	61	180	--	--	--	--	--	--
TR34816|c2_g2_i3	XLOC_011750	(AT)8	16	--	--	--	--	--	--	TGGACCAAATGTTGGGATTT	60	CCGTAATCATTGGGCTTGTC	60	247	--	--	--	--	--	--
TR34818|c6_g1_i1	XLOC_011762	(GT)6	12	--	--	--	--	--	--	AGTTGTGTTCGCATTTCGCT	61	TCATGTTGCACTTGGGAAAA	60	119	--	--	--	--	--	--
TR34822|c0_g1_i1	No	(GGA)5	15	--	--	--	--	--	--	AAGGGTACGGAGGTGGAGAT	60	GAGATTCGTGGCGAGATTTG	61	135	--	--	--	--	--	--

Transcript IDs	Differentially expressed gene (DEG) IDs	SSR repeat-motifs	SSR repeat-length (bp)	*In silico* polymorphic SSRs						Forward primers (5'-3')	Annealing tempearture(0C)	Reverse primers (5'-3')	Annealing tempearture(0C)	Amplified product size (bp)	Structural annotation		Functional Annotation			
				C. microphyllum		ICC4958									Gene IDs	Sequence component of genes	NCBI nr IDs	TFs	KOG	KEGG
				Transcript	SSR repeat-motifs	Chromosomes	SSR repeat-motifs	Start physical positions (bp)	End physical positions (bp)											
TR34822|c0_g1_i2	No	(GGA)5	15	--	--	--	--	--	--	AAGGGTACGGAGGTGGAGAT	60	GAGATTCGTGGCGAGATTTG	61	135	--	--	--	--	--	--
TR34822|c0_g1_i5	No	(GGA)5	15	--	--	--	--	--	--	AAGGGTACGGAGGTGGAGAT	60	GAGATTCGTGGCGAGATTTG	61	135	--	--	--	--	--	--
TR34822|c0_g1_i6	No	(GGA)5	15	--	--	--	--	--	--	AAGGGTACGGAGGTGGAGAT	60	GAGATTCGTGGCGAGATTTG	61	135	--	--	--	--	--	--
TR34822|c0_g1_i7	No	(GGA)5	15	--	--	--	--	--	--	AAGGGTACGGAGGTGGAGAT	60	GAGATTCGTGGCGAGATTTG	61	135	--	--	--	--	--	--
TR34822|c0_g1_i8	No	(GGA)5	15	--	--	--	--	--	--	AAGGGTACGGAGGTGGAGAT	60	GAGATTCGTGGCGAGATTTG	61	135	--	--	--	--	--	--
TR34830|c1_g1_i1	XLOC_011786	(AG)6	12	--	--	--	--	--	--	AGTGCCTTCTGTCCTCTCCA	60	CACCATAAACATAAACCCTCACA	59	256	--	--	XP_004492659.1	--	[S]	--
TR34830|c3_g2_i4	No	(AG)12	24	--	--	--	--	--	--	GTAAGAAAATGGGGGAGGCT	59	GAAGCCGTGGCTAGAGATTG	60	187	--	--	--	--	--	--
TR34830|c3_g2_i5	No	(AG)12	24	--	--	--	--	--	--	GTAAGAAAATGGGGGAGGCT	59	GAAGCCGTGGCTAGAGATTG	60	187	--	--	--	--	--	--
TR34830|c3_g2_i6	No	(AG)12	24	--	--	--	--	--	--	GTAAGAAAATGGGGGAGGCT	59	GAAGCCGTGGCTAGAGATTG	60	187	--	--	--	--	--	--
TR34830|c3_g2_i9	No	(AG)12	24	--	--	--	--	--	--	GTAAGAAAATGGGGGAGGCT	59	GAAGCCGTGGCTAGAGATTG	60	187	--	--	--	--	--	--
TR34831|c0_g1_i2	No	(AGA)5	15	--	--	--	--	--	--	CACCACCTCTCTCTTCTCCG	60	TGTTGTGTTGTTCGTTGGGT	60	167	--	--	--	--	--	--
TR34831|c0_g1_i5	No	(AGA)5	15	--	--	--	--	--	--	CACCACCTCTCTCTTCTCCG	60	TGTTGTGTTGTTCGTTGGGT	60	167	--	--	--	--	--	--
TR34831|c0_g1_i6	No	(AGA)5	15	--	--	--	--	--	--	CACCACCTCTCTCTTCTCCG	60	TGTTGTGTTGTTCGTTGGGT	60	167	--	--	--	--	--	--
TR34831|c0_g1_i8	XLOC_011790	(AGA)5	15	--	--	--	--	--	--	CACCACCTCTCTCTTCTCCG	60	TGTTGTGTTGTTCGTTGGGT	60	167	--	--	--	--	--	--

Transcript IDs	Differentially expressed gene (DEG) IDs	SSR repeat-motifs	SSR repeat-length (bp)	*In silico* polymorphic SSRs						Forward primers (5'-3')	Annealing tempearture(0C)	Reverse primers (5'-3')	Annealing tempearture(0C)	Amplified product size (bp)	Structural annotation		Functional Annotation			
				C. microphyllum		ICC4958									Gene IDs	Sequence component of genes	NCBI nr IDs	TFs	KOG	KEGG
				Transcript	SSR repeat-motifs	Chromosomes	SSR repeat-motifs	Start physical positions (bp)	End physical positions (bp)											
TR34831|c0_g1_i9	No	(AGA)5	15	--	--	--	--	--	--	CACCACCTCTCTCTTCTCCG	60	TGTTGTGTTGTTCGTTGGGT	60	167	--	--	--	--	--	--
TR34833|c1_g2_i1	No	(TC)7	14	--	--	--	--	--	--	ATTTTCGGCCCTCTTTGTTT	60	TCATCCTCACTTACAGCCCC	60	272	--	--	--	--	--	--
TR34833|c1_g2_i10	No	(TC)7	14	--	--	--	--	--	--	ATTTTCGGCCCTCTTTGTTT	60	CTGATTGGTGGATTGCAGTG	60	219	--	--	--	--	--	--
TR34833|c1_g2_i16	No	(TC)7	14	--	--	--	--	--	--	ATTTTCGGCCCTCTTTGTTT	60	CTGATTGGTGGATTGCAGTG	60	219	--	--	--	--	--	--
TR34833|c1_g2_i2	XLOC_011803	(TC)7	14	--	--	--	--	--	--	TTGCACCTCCCTTCAAAATC	60	CTGATTGGTGGATTGCAGTG	60	200	--	--	--	--	--	--
TR34833|c1_g2_i3	No	(TC)7	14	--	--	--	--	--	--	ATTTTCGGCCCTCTTTGTTT	60	TCATCCTCACTTACAGCCCC	60	272	--	--	--	--	--	--
TR34833|c1_g2_i7	No	(TC)7	14	--	--	--	--	--	--	ATTTTCGGCCCTCTTTGTTT	60	CTGATTGGTGGATTGCAGTG	60	219	--	--	--	--	--	--
TR34835|c0_g1_i1	XLOC_011809	(TC)6	12	--	--	--	--	--	--	GGTTTTGGTTTTGGTTTTGC	59	TGGAACAAAGAGCAAAAGGG	60	218	--	--	--	--	--	--
TR34837|c0_g1_i1	No	(TC)10	20	--	--	--	--	--	--	TTTCGCAGCTCTCACAAATG	60	AGAAAGGCTGAGTGTGGGTG	60	170	--	--	--	--	--	--
TR34838|c5_g1_i1	No	(ACA)5	15	--	--	--	--	--	--	AACAAGCACTACACAGCAACTC	57	CGAGGTAGAGGAGAGGAGCA	60	122	--	--	--	--	--	--
TR34846|c3_g8_i5	No	(TA)6	12	--	--	--	--	--	--	TGACCAAAGGCAATGTTGAA	60	CCATTAGACACGGCCTTCAT	60	162	--	--	--	--	--	--
TR34846|c3_g8_i6	No	(TA)6	12	--	--	--	--	--	--	TGACCAAAGGCAATGTTGAA	60	CCATTAGACACGGCCTTCAT	60	162	--	--	--	--	--	--
TR34860|c2_g6_i1	XLOC_011924	(GCC)5	15	--	--	--	--	--	--	ATGAGGGAGGGACTTGAAGG	60	TCGGGTTCGACAATTTCTTC	60	146	--	--	--	--	--	--
TR34860|c2_g8_i8	No	(CT)7attcttttctattccattctattcaataattcacaaattaa tatgctttaa(TAT)5	80	--	--	--	--	--	--	TTTCCACCTCGAATCCAATC	60	TTCGACAGTTTCTTCTCCGAA	60	268	--	--	--	--	--	--

Transcript IDs	Differentially expressed gene (DEG) IDs	SSR repeat-motifs	SSR repeat-length (bp)	*In silico* polymorphic SSRs						Forward primers (5'-3')	Annealing tempearture(0C)	Reverse primers (5'-3')	Annealing tempearture(0C)	Amplified product size (bp)	Structural annotation		Functional Annotation			
				C. microphyllum		ICC4958														
				Transcript	SSR repeat-motifs	Chromosomes	SSR repeat-motifs	Start physical positions (bp)	End physical positions (bp)						Gene IDs	Sequence component of genes	NCBI nr IDs	TFs	KOG	KEGG
TR34870|c1_g1_i1	No	(AG)6	12	--	--	--	--	--	--	TTCAAAGAGGCTGCCAAACT	60	CTGATTCATGCTTTGGGTGA	60	261	--	--	--	--	--	--
TR34871|c1_g1_i1	XLOC_011955	(GGC)6	18	--	--	--	--	--	--	CATGAATTGGTATCCCGACC	60	CTACTGTCCTTGCCCACCTC	60	178	--	--	CAB88204.1	--	[W]	--
TR34874|c2_g1_i1	XLOC_011965	(CT)7	14	--	--	--	--	--	--	TGCGTCCTTCGTCTTCTTCT	60	CACGTGGGTGAGTTATGGTG	60	255	--	--	--	--	--	--
TR34883|c1_g2_i1	No	(A)10ttctcgtcttggtaaagttctaagtttggggttgctcatgggatagcaaccttttaagtttggggcggtggttctc(A)10tcaaaacaaaaaaaaaacacgggggag(A)10	132	--	--	--	--	--	--	CCTTGCCCTTTTATTTCGGT	60	TGATTTCGAATCTTCGGAGC	60	250	--	--	--	--	--	--
TR34883|c1_g2_i2	No	(A)10ttctcgtcttggtaaagttctaagtttggggttgctcatgggatagcaaccttttaagtttggggcggtggttctc(A)10tcaaaacaaaaaaaaaacacgggggag(A)10	132	--	--	--	--	--	--	CCTTGCCCTTTTATTTCGGT	60	TGATTTCGAATCTTCGGAGC	60	250	--	--	--	--	--	--
TR34883|c1_g2_i4	No	(A)10ttctcgtcttggtaaagttctaagtttggggttgctcatgggatagcaaccttttaagtttggggcggtggttctc(A)10tcaaaacaaaaaaaaaacacgggggag(A)10	132	--	--	--	--	--	--	CCTTGCCCTTTTATTTCGGT	60	TGATTTCGAATCTTCGGAGC	60	250	--	--	--	--	--	--
TR3490|c0_g1_i1	XLOC_012009	(GAC)5	15	--	--	--	--	--	--	TCCATTTCTTCCATTTTGGC	60	ACCCACAAAGTACATCCCGA	60	246	--	--	--	--	--	--
TR3496|c0_g1_i1	XLOC_012033	(AAG)5	15	--	--	--	--	--	--	CGAGCGCGAAGAAAGTGATA	62	CTTGTTCATTCTGCAGGCAA	60	173	--	--	--	--	--	--
TR35101|c0_g1_i1	XLOC_012077	(CTG)5	15	--	--	--	--	--	--	AAGGCCAGTATGCACAGCTT	60	TTTTCAACTTGCCTCCCATC	60	167	--	--	--	--	--	--
TR35190|c0_g1_i1	No	(AT)9	18	--	--	--	--	--	--	TCACCCTCTCATTGTAAAACTCC	60	ACTTGCATGTGTCACCCAAA	60	257	--	--	--	--	--	--
TR35202|c0_g1_i1	No	(TGA)5	15	--	--	--	--	--	--	TTGATCCTTCACAAGAGGATG	58	TATCACCTTCACCGTCCCTC	60	195	--	--	--	--	--	--
TR35267|c0_g1_i1	XLOC_012115	(CAC)6	18	--	--	--	--	--	--	CCGATTATTCCTCCTTCGGT	60	TCTGCTGTTGCTCTTGCAGT	60	180	--	--	XP_001950452.1	--	--	--
TR35315|c0_g1_i1	No	(AG)7	14	--	--	--	--	--	--	AAAAGGTGAAACCCAACAGG	59	TGCATCAATGGGAGAAACAA	60	104	--	--	--	--	--	--
TR35337|c0_g1_i1	XLOC_012135	(CT)6	12	--	--	--	--	--	--	AGCTCTGTGAGGACTGGATCA	60	AGCTTGTTGTTTGTGGGTGG	61	102	--	--	--	--	--	--

Transcript IDs	Differentially expressed gene (DEG) IDs	SSR repeat-motifs	SSR repeat-length (bp)	*In silico* polymorphic SSRs						Forward primers (5'-3')	Annealing tempearture(0C)	Reverse primers (5'-3')	Annealing tempearture(0C)	Amplified product size (bp)	Structural annotation		Functional Annotation			
				C. microphyllum		ICC4958									Gene IDs	Sequence component of genes	NCBI nr IDs	TFs	KOG	KEGG
				Transcript	SSR repeat-motifs	Chromosomes	SSR repeat-motifs	Start physical positions (bp)	End physical positions (bp)											
TR35364|c0_g1_i1	XLOC_012143	(GCT)5	15	--	--	--	--	--	--	TCAGGAAATGAATTGAGCCC	60	AACAAAAATTTGAATAACTGAAGCA	58	253	--	--	--	--	--	--
TR35385|c0_g1_i1	No	(TGA)5	15	--	--	--	--	--	--	TGTTGCTTCCCACTTCAACA	60	CATAAAGCAGATGGGGAGGA	60	196	--	--	--	--	--	--
TR35444|c0_g1_i1	No	(AG)8	16	--	--	--	--	--	--	CAGAGTGCAGACAAACTAACGG	60	AAACTCTTCCAAACGTACCCA	59	262	--	--	--	--	--	--
TR35502|c0_g1_i1	No	(GAT)5	15	--	--	--	--	--	--	GTATGGGAGGCCAATGTCTC	59	TTGGTCCATGTTGTTGCACT	60	125	--	--	--	--	--	--
TR35510|c0_g1_i1	XLOC_012179	(AG)6	12	--	--	--	--	--	--	TCCGAAATAAGATTGCTGGG	60	GAACAGCAGCAACAGGATCA	60	243	--	--	XP_002530169.1	--	[J]	K02962
TR35546|c0_g1_i1	XLOC_012194	(AC)6	12	TR35546|c0_g1_i1	(AC)6	Ca_LG_3	(AC)8	30469630	30469645	AACGCCACTGCTCCATCTAT	60	TTGAAGATCGAGGGATTTGG	60	207		URR	--	--	--	--
TR356|c0_g1_i1	No	(A)10catttaactataaaagtttatacttatatactatataattaaac(A)10	64	--	--	--	--	--	--	CCTCCACACTCTGTCCTTCC	60	TTTTGAGTCTTGTGACCCTGAA	60	215	--	--	--	--	--	--
TR356|c0_g1_i2	No	(A)10catttaactataaaagtttatacttatatactatataattaaac(A)10	64	--	--	--	--	--	--	TGTTCGAATTTGAGCTCACTG	59	CATGTTTCCAATGATTTGGTCA	61	280	--	--	--	--	--	--
TR35618|c0_g1_i1	XLOC_012228	(AT)7	14	--	--	--	--	--	--	TCCAATCACTCAACGTCAATG	60	TGCTCACCAATCTTCCTCACT	60	249	--	--	--	--	--	--
TR35644|c0_g1_i1	XLOC_012244	(AG)8(AGA)5*	29	--	--	--	--	--	--	TTGTTCGTATAGTCGACAAAAATCTC	60	CTCCAATTCCTTGGTGGTGT	60	213	--	--	--	--	--	--
TR35658|c0_g1_i1	No	(ACA)5	15	--	--	--	--	--	--	ATGGGAACGTGTCAAGGAAG	60	TCCATCCTCCTGAGTCTTGG	60	276	--	--	--	--	--	--
TR35658|c0_g2_i1	No	(ACA)5	15	--	--	--	--	--	--	ATGGGAACGTGTCAAGGAAG	60	TCCATCCTCCTGAGTCTTGG	60	276	--	--	--	--	--	--
TR35658|c0_g3_i1	No	(ACA)5	15	--	--	--	--	--	--	ATGGGAACGTGTCAAGGAAG	60	TCCATCCTCCTGAGTCTTGG	60	276	--	--	--	--	--	--
TR35658|c0_g4_i1	No	(ACA)5	15	--	--	--	--	--	--	ATGGGAACGTGTCAAGGAAG	60	TCCATCCTCCTGAGTCTTGG	60	276	--	--	--	--	--	--

Transcript IDs	Differentially expressed gene (DEG) IDs	SSR repeat-motifs	SSR repeat-length (bp)	In silico polymorphic SSRs						Forward primers (5'-3')	Annealing tempearture(0C)	Reverse primers (5'-3')	Annealing tempearture(0C)	Amplified product size (bp)	Structural annotation		Functional Annotation			
				C. microphyllum		ICC4958									Gene IDs	Sequence component of genes	NCBI nr IDs	TFs	KOG	KEGG
				Transcript	SSR repeat-motifs	Chromosomes	SSR repeat-motifs	Start physical positions (bp)	End physical positions (bp)											
TR35713|c0_g2_i1	XLOC_012270	(ACA)5	15	--	--	--	--	--	--	ATATGGCCACAACAGCAACA	60	AGAATGAAACGGAAACGACG	60	166	--	--	--	--	--	--
TR35728|c0_g1_i1	XLOC_012276	(GAA)5	15	--	--	--	--	--	--	GAAACTTCTGCTGGTGCCTC	60	CAATGGCCTTAATCTCCTCTTC	59	217	--	--	XP_004514915.1	--	[X]	--
TR35728|c0_g2_i1	No	(TA)6	12	--	--	--	--	--	--	CCCAGGAACTAAAGGAAGCC	60	TGTTGGTAATGTTGGTTGGTG	59	157	--	--	--	--	--	--
TR35728|c0_g2_i3	No	(AAC)5	15	--	--	--	--	--	--	TGCCTCTGTTGTCGTACTCG	60	ATTGATGGCAAGTCTCCCTG	60	226	--	--	--	--	--	--
TR35736|c0_g1_i1	No	(AGA)5	15	--	--	--	--	--	--	ACTCGTGACAATAGACCGGC	60	CGTTATCAATATCACCGCGA	60	238	--	--	--	--	--	--
TR35736|c0_g1_i10	No	(AGA)5	15	--	--	--	--	--	--	ACTCGTGACAATAGACCGGC	60	AGCAGCAGTGACCTGTAGCA	60	195	--	--	--	--	--	--
TR35736|c0_g1_i2	No	(AGA)5	15	--	--	--	--	--	--	ACTCGTGACAATAGACCGGC	60	AGCAGCAGTGACCTGTAGCA	60	154	--	--	--	--	--	--
TR35736|c0_g1_i3	No	(AGA)5	15	--	--	--	--	--	--	ACTCGTGACAATAGACCGGC	60	CGTTATCAATATCACCGCGA	60	238	--	--	--	--	--	--
TR35736|c0_g1_i4	No	(AGA)5	15	--	--	--	--	--	--	ACTCGTGACAATAGACCGGC	60	AGCAGCAGTGACCTGTAGCA	60	195	--	--	--	--	--	--
TR35736|c0_g1_i5	No	(AGA)5	15	--	--	--	--	--	--	ACTCGTGACAATAGACCGGC	60	AGCAGCAGTGACCTGTAGCA	60	195	--	--	--	--	--	--
TR35736|c0_g1_i6	No	(AGA)5	15	--	--	--	--	--	--	ACTCGTGACAATAGACCGGC	60	AGCAGCAGTGACCTGTAGCA	60	154	--	--	--	--	--	--
TR35736|c0_g1_i7	No	(AGA)5	15	--	--	--	--	--	--	ACTCGTGACAATAGACCGGC	60	CAACCATCACCGAAAAGTTG	59	246	--	--	--	--	--	--
TR35736|c0_g1_i8	No	(AGA)5	15	--	--	--	--	--	--	ACTCGTGACAATAGACCGGC	60	CAACCATCACCGAAAAGTTG	59	246	--	--	--	--	--	--
TR35736|c0_g1_i9	No	(AGA)5	15	--	--	--	--	--	--	ACTCGTGACAATAGACCGGC	60	AGCAGCAGTGACCTGTAGCA	60	195	--	--	--	--	--	--

Transcript IDs	Differentially expressed gene (DEG) IDs	SSR repeat-motifs	SSR repeat-length (bp)	*In silico* polymorphic SSRs						Forward primers (5'-3')	Annealing tempearture(0C)	Reverse primers (5'-3')	Annealing tempearture(0C)	Amplified product size (bp)	Structural annotation		Functional Annotation			
				C. microphyllum		ICC4958									Gene IDs	Sequence component of genes	NCBI nr IDs	TFs	KOG	KEGG
				Transcript	SSR repeat-motifs	Chromosomes	SSR repeat-motifs	Start physical positions (bp)	End physical positions (bp)											
TR35761|c0_g2_i1	No	(AAG)5	15	--	--	--	--	--	--	CCCAATCCTAGCTACCACGA	60	CATTTGACAAAGCATGCACC	60	256	--	--	--	--	--	--
TR35761|c0_g2_i3	No	(AAG)5	15	--	--	--	--	--	--	CCCAATCCTAGCTACCACGA	60	CATTTGACAAAGCATGCACC	60	256	--	--	--	--	--	--
TR35761|c0_g2_i4	No	(AAG)5	15	--	--	--	--	--	--	CCCAATCCTAGCTACCACGA	60	CATTTGACAAAGCATGCACC	60	256	--	--	--	--	--	--
TR35761|c0_g2_i5	No	(AAG)5	15	--	--	--	--	--	--	CCCAATCCTAGCTACCACGA	60	CATTTGACAAAGCATGCACC	60	256	--	--	--	--	--	--
TR35761|c0_g3_i1	No	(AAG)5	15	--	--	--	--	--	--	CCCAATCCTAGCTACCACGA	60	CATTTGACAAAGCATGCACC	60	256	--	--	--	--	--	--
TR35761|c0_g4_i1	No	(AAG)5	15	--	--	--	--	--	--	CCCAATCCTAGCTACCACGA	60	CATTTGACAAAGCATGCACC	60	256	--	--	--	--	--	--
TR35764|c0_g2_i1	XLOC_012321	(CT)6ttaacaaac(A)11ca(AG)6	46	--	--	--	--	--	--	CTCACCCAGATGGCAAACTT	60	AACCCATTTTCCCCACTTTC	60	243	--	--	--	--	--	--
TR35767|c0_g1_i1	No	(CT)9	18	--	--	--	--	--	--	CAATTTCAACAAGTGCGTGC	60	AACATGCCGTTGACTTTTCC	60	218	--	--	--	--	--	--
TR35767|c0_g1_i2	No	(CT)9	18	--	--	--	--	--	--	CAATTTCAACAAGTGCGTGC	60	AACATGCCGTTGACTTTTCC	60	218	--	--	--	--	--	--
TR35769|c0_g2_i1	No	(CT)9	18	--	--	--	--	--	--	AAAAACAAGCGCACACACAG	60	ATTTAGCCATGGTGGTGGAG	60	165	--	--	--	--	--	--
TR35769|c0_g2_i2	No	(CT)9	18	--	--	--	--	--	--	ACCACACCTCGTCGTAGCAT	61	ATTTAGCCATGGTGGTGGAG	60	140	--	--	--	--	--	--
TR35769|c0_g2_i3	No	(CT)9	18	--	--	--	--	--	--	ACCACACCTCGTCGTAGCAT	61	ATTTAGCCATGGTGGTGGAG	60	140	--	--	--	--	--	--
TR35769|c0_g2_i4	No	(CT)9	18	--	--	--	--	--	--	AAAAACAAGCGCACACACAG	60	ATTTAGCCATGGTGGTGGAG	60	165	--	--	--	--	--	--
TR35769|c0_g2_i5	No	(CT)9	18	--	--	--	--	--	--	AAAAACAAGCGCACACACAG	60	ATTTAGCCATGGTGGTGGAG	60	165	--	--	--	--	--	--

Transcript IDs	Differentially expressed gene (DEG) IDs	SSR repeat-motifs	SSR repeat-length (bp)	*In silico* polymorphic SSRs						Forward primers (5'-3')	Annealing tempearture(0C)	Reverse primers (5'-3')	Annealing tempearture(0C)	Amplified product size (bp)	Structural annotation		Functional Annotation			
				C. microphyllum		ICC4958									Gene IDs	Sequence component of genes	NCBI nr IDs	TFs	KOG	KEGG
				Transcript	SSR repeat-motifs	Chromosomes	SSR repeat-motifs	Start physical positions (bp)	End physical positions (bp)											
TR35769|c0_g2_i6	No	(CT)9	18	--	--	--	--	--	--	ACCACACCTCGTCGTAGCAT	61	ATTTAGCCATGGTGGTGGAG	60	140	--	--	--	--	--	--
TR35771|c0_g1_i1	No	(TGC)5	15	--	--	--	--	--	--	TCAGTCAGCCCATAGGAACA	59	ACGACGAAGAAGAACGAGGA	60	230	--	--	--	--	--	--
TR35771|c0_g2_i1	No	(TGC)5	15	--	--	--	--	--	--	TCAGTCAGCCCATAGGAACA	59	ACGACGAAGAAGAACGAGGA	60	230	--	--	--	--	--	--
TR35771|c0_g3_i1	No	(TGC)5	15	--	--	--	--	--	--	TCAGTCAGCCCATAGGAACA	59	ACGACGAAGAAGAACGAGGA	60	230	--	--	--	--	--	--
TR35772|c0_g1_i2	No	(CT)8ccttc(A)12	33	--	--	--	--	--	--	GAGATTGCTCTAGCCATATCACTT	58	TAACAATCGAATTTTGGGGG	60	219	--	--	--	--	--	--
TR35772|c0_g1_i2	No	(T)10aaa(T)11ctgatccatc(T)14	48	--	--	--	--	--	--	TCTCACTTTCTCTGCACCCA	60	TCGTTGCTCCTTCAAATCAA	59	235	--	--	--	--	--	--
TR35772|c0_g2_i5	No	(CT)8	16	--	--	--	--	--	--	ATTCATGGCTGTGCAAACAC	60	TTCAATGAATTACAGCCACCA	59	133	--	--	--	--	--	--
TR35777|c0_g1_i1	XLOC_012335	(TC)7	14	--	--	--	--	--	--	ACCAGGCATTGAACACAACA	60	AAGGACGGTGTAGATGTGGC	60	155	--	--	--	--	--	--
TR35784|c0_g2_i1	XLOC_012350	(AG)6	12	--	--	--	--	--	--	ATCTCTCCCAAAATCCCGAC	60	GAGGGAAGAAAGAGTGCGTG	60	180	--	--	--	--	--	--
TR35784|c0_g2_i1	XLOC_012350	(AAC)5	15	--	--	--	--	--	--	GTGCGATGCAATTGAGACAG	60	GTCGGGATTTTGGGAGAGAT	60	211	--	--	--	--	--	--
TR35788|c0_g1_i1	XLOC_012354	(CA)6	12	--	--	--	--	--	--	CTCAAAAACGCTTCACCACA	60	CCTGCAAGATTTTCCAGAGC	60	228	--	--	--	--	--	--
TR35812|c0_g1_i1	XLOC_012390	(GTG)5	15	--	--	--	--	--	--	GAGCAGGCTTTGGAAAATGA	60	CAGAGAAACTGTCCAACGCA	60	166	--	--	--	--	--	--
TR35813|c2_g1_i1	No	(CT)8tgccagctctgcagacttgtcctcccccataacctgtttcttctta(TC)10(AC)7	96	--	--	--	--	--	--	GAATTGCAAATCTCAAAAGGC	59	GTCGCCAAATTCCGAAGTAA	60	264	--	--	--	--	--	--
TR35813|c2_g1_i2	No	(A)10cactcacaagaacagcaattattcactttttttcctctcttttcaacaagtatc(CT)8tgccagctctgcagacttgtcctcccccataacctgtttcttctta(TC)10(AC)7	160	--	--	--	--	--	--	GAATTGCAAATCTCAAAAGGC	59	GTCGCCAAATTCCGAAGTAA	60	264	--	--	--	--	--	--

Transcript IDs	Differentially expressed gene (DEG) IDs	SSR repeat-motifs	SSR repeat-length (bp)	*In silico* polymorphic SSRs						Forward primers (5'-3')	Annealing tempearture(0C)	Reverse primers (5'-3')	Annealing tempearture(0C)	Amplified product size (bp)	Structural annotation		Functional Annotation			
				C. microphyllum		ICC4958									Gene IDs	Sequence component of genes	NCBI nr IDs	TFs	KOG	KEGG
				Transcript	SSR repeat-motifs	Chromosomes	SSR repeat-motifs	Start physical positions (bp)	End physical positions (bp)											
TR35813|c2_g1_i3	No	(A)10cactcacaagaacagcaattattcacttttttcctctcttttcaacaagtatc(CT)8tgccagctctgcagacttgtcctcccccataacctgtttcttctta(TC)10(AC)7	160	--	--	--	--	--	--	GAATTGCAAATCTCAAAAGGC	59	GTCGCCAAATTCCGAAGTAA	60	264	--	--	--	--	--	--
TR35813|c2_g1_i4	No	(CT)8tgccagctctgcagacttgtcctcccccataacctgtttcttctta(TC)10(AC)7	96	--	--	--	--	--	--	GAATTGCAAATCTCAAAAGGC	59	GTCGCCAAATTCCGAAGTAA	60	263	--	--	--	--	--	--
TR35866|c0_g1_i1	No	(AT)9	18	--	--	--	--	--	--	TCTTGCTTACTAATGAACCTGCAA	60	ATGTCTGCACGTCGAACAAT	59	155	--	--	--	--	--	--
TR35866|c0_g2_i1	No	(AT)9	18	--	--	--	--	--	--	TCTTGCTTACTAATGAACCTGCAA	60	ATGTCTGCACGTCGAACAAT	59	155	--	--	--	--	--	--
TR35879|c1_g1_i1	No	(TCAG)5tcggtcgtcggtcgatcagtcggtcggtcgtcttctcg(TTA)5	73	--	--	--	--	--	--	CTTTTCGCGTGAAAGTGTGA	60	ACGGAGGTCATAGTTGCTGC	60	266	--	--	--	--	--	--
TR35889|c0_g2_i1	No	(TA)7	14	--	--	--	--	--	--	AAATATTACAAAAGGATTTAGAAAGGC	58	TTGCAAAAGATAATTCCGGG	60	250	--	--	--	--	--	--
TR35921|c0_g1_i1	No	(TAA)5	15	--	--	--	--	--	--	GTTCGGCCTCTCCAGTAAAA	59	CCCACAAAAATTTGACATTGG	60	271	--	--	--	--	--	--
TR35959|c0_g1_i1	No	(GCA)5	15	--	--	--	--	--	--	AGGCAGTAGTAGCGGCAGAA	60	GGCCACTTCACCTTCAACAT	60	161	--	--	--	--	--	--
TR35959|c0_g2_i1	No	(GCA)5	15	--	--	--	--	--	--	TACTAGCGGCAGCAGTTGTG	60	CGAAGTTGGTAAGTGCCCAT	60	275	--	--	--	--	--	--
TR35977|c0_g1_i1	No	(TTG)5	15	--	--	--	--	--	--	GTGGCTTGTTGGTTTTGGAT	60	TGGAATATTTCCCACCATCAA	60	188	--	--	--	--	--	--
TR35983|c0_g1_i1	No	(AG)7	14	--	--	--	--	--	--	CAATTCCTTTGTTGGCACCT	60	TCAGCTCTCTCTGTCTCTCCG	60	114	--	--	--	--	--	--
TR35991|c0_g1_i1	XLOC_012461	(TA)6	12	--	--	--	--	--	--	AGCACATTTGAACTCTGGCA	59	CGTAGGCTCTCTTCTTCCGTT	60	222	--	--	--	--	--	--
TR360|c2_g1_i1	XLOC_012487	(TTC)6	18	--	--	--	--	--	--	CATCGTTGTCGTCCTGTGTT	60	GCGATGACGAAGACAAATGA	60	179	--	--	--	--	--	--
TR36053|c0_g1_i1	No	(AAT)5	15	--	--	--	--	--	--	TCGATTCACATAATCTGGAGAAGA	60	TGGAATCTTGGGTCTGCATT	60	144	--	--	--	--	--	--

Transcript IDs	Differentially expressed gene (DEG) IDs	SSR repeat-motifs	SSR repeat-length (bp)	*In silico* polymorphic SSRs						Forward primers (5'-3')	Annealing tempearture(0C)	Reverse primers (5'-3')	Annealing tempearture(0C)	Amplified product size (bp)	Structural annotation		Functional Annotation			
				C. microphyllum		ICC4958									Gene IDs	Sequence component of genes	NCBI nr IDs	TFs	KOG	KEGG
				Transcript	SSR repeat-motifs	Chromosomes	SSR repeat-motifs	Start physical positions (bp)	End physical positions (bp)											
TR36064|c0_g1_i1	XLOC_012474	(GAT)6	18	--	--	--	--	--	--	CCCTAATGGGTCGTCTTCAA	60	GCTTCAACGACCCAATCTTC	60	261	--	--	XP_012080836.1	bHLH	[X]	--
TR36109|c0_g1_i1	XLOC_012489	(CCG)5	15	--	--	--	--	--	--	AAATTACTTCGGTGGGGACC	60	CAACACCGAGAGACAAAGCA	60	230	--	--	XM_004509713.1	AP2	[K]	--
TR36119|c0_g1_i1	No	(AGC)5	15	--	--	--	--	--	--	ATACAGAAGGCTCGGAACCA	60	TTGCTTCCCTCGGTACTCAC	60	116	--	--	--	--	--	--
TR36133|c0_g1_i1	XLOC_012493	(GA)6	12	--	--	--	--	--	--	GGAGGCACGTCTCAAAGAAG	60	CTGGATGGCATCAACATCAG	60	259	--	--	--	--	--	--
TR36196|c0_g1_i1	No	(CAC)5	15	--	--	--	--	--	--	CGGCTACGACTACCTCAACC	60	AGTGTAGGAATCGCCGAATG	60	179	--	--	--	--	--	--
TR36228|c0_g1_i1	No	(CAA)6	18	--	--	--	--	--	--	TTCGAACACCAGTCCAACAA	60	TAGTGGGAGTCATTGGTGCC	61	188	--	--	--	--	--	--
TR36243|c0_g1_i1	No	(A)11gatatccaatttaaatttttcctccctcgtatctcaaaacaaaaaaattaaatcattttatagggaagaataagaaagagaactaggcagcat(A)13	117	--	--	--	--	--	--	CAAGAGGAAAGGAATAATTAGCCA	60	ACCCAATTGGTACGGAACCT	60	200	--	--	--	--	--	--
TR36319|c0_g1_i1	No	(ATA)7	21	--	--	--	--	--	--	GACTTTTAATGTTGCCTGCTCA	59	GCCTGTCGTAGGAAGTTAGGAA	60	253	--	--	--	--	--	--
TR36326|c0_g1_i1	No	(AT)8accctttaaaaacagtgaaatataacccaaggacaagtttcataatcaaacaaccaaatattaatgcactatacttttaagggagtattaaagatcat(AG)8	130	--	--	--	--	--	--	GGAAGAGACATCATTCACTCTCG	60	CAACCATGTGAATTAAGCAATCA	60	280	--	--	--	--	--	--
TR36337|c2_g1_i1	No	(ATC)5	15	--	--	--	--	--	--	TACTCATTCGAGACCTGCCA	59	CGATTCCTGGATAAGGTGGA	60	266	--	--	--	--	--	--
TR36365|c0_g1_i1	XLOC_012525	(CTC)8	24	--	--	--	--	--	--	ACGTTTCTTTGGAGGTGGTG	60	CAAGCAAATGGGTCTGGAAT	60	250	--	--	--	--	--	--
TR36369|c0_g1_i1	XLOC_012526	(CAT)7	21	--	--	--	--	--	--	AGGAATGCTCAGCCTCGTAA	60	AATTACAAAGCCAGAGCCCA	60	186	--	--	--	--	--	--
TR36387|c0_g1_i1	No	(TTC)5	15	--	--	--	--	--	--	TCCAACACCTCTTTCTTTCCA	60	ATGCTGGTGGCTCTGAGAAT	60	167	--	--	--	--	--	--
TR364|c0_g1_i1	XLOC_012560	(AC)6	12	--	--	--	--	--	--	CTCCAAACTACTCCCCGAAA	59	ACCAACGGACTGAACCGTAG	60	275	--	--	--	--	--	--

Transcript IDs	Differentially expressed gene (DEG) IDs	SSR repeat-motifs	SSR repeat-length (bp)	*In silico* polymorphic SSRs						Forward primers (5'-3')	Annealing tempearture(0C)	Reverse primers (5'-3')	Annealing tempearture(0C)	Amplified product size (bp)	Structural annotation		Functional Annotation			
				C. microphyllum		ICC4958									Gene IDs	Sequence component of genes	NCBI nr IDs	TFs	KOG	KEGG
				Transcript	SSR repeat-motifs	Chromosomes	SSR repeat-motifs	Start physical positions (bp)	End physical positions (bp)											
TR36502|c0_g1_i1	XLOC_012562	(CAC)5	15	--	--	--	--	--	--	TCAAGAACCCTTCAGCCATC	60	GCAACTCATTCAGCACCAAA	60	118	--	--	--	--	--	--
TR36589|c0_g2_i1	No	(GA)9	18	--	--	--	--	--	--	GCTGAGGAGGGAACATTTGA	60	TCCCTTAAGACAGAGAGAGACCC	60	188	--	--	--	--	--	--
TR36682|c0_g1_i1	XLOC_012602	(TTG)5	15	--	--	--	--	--	--	GCAAAACATAACACACGACGA	60	GGTTTTGGGGTGAAAAGGTT	60	239	--	--	--	--	--	--
TR36702|c0_g1_i1	No	(GAA)5	15	--	--	--	--	--	--	TGCTCGTTTCTTTGGTTGTG	60	TGGATGATGGTTTCATGTCA	58	158	--	--	--	--	--	--
TR36702|c1_g1_i1	No	(GAA)5	15	--	--	--	--	--	--	GCTCTAAGAAAGCCGATTCTGA	60	CGATTATGGTGTTGATCATCGT	60	173	--	--	--	--	--	--
TR36709|c0_g1_i1	XLOC_012627	(AT)6	12	--	--	--	--	--	--	TTGGTACATGTTCAAAATCATACA	57	TGAGGGCTAATTCTGGTGCT	60	274	--	--	--	--	--	--
TR36709|c0_g2_i1	No	(AT)6	12	--	--	--	--	--	--	TTGGTACATGTTCAAAATCATACA	57	TGAGGGCTAATTCTGGTGCT	60	274	--	--	--	--	--	--
TR36711|c0_g1_i1	XLOC_012629	(TAA)5	15	--	--	--	--	--	--	CTCCACAAAATTGGACAGCC	60	CGATACACAATCCATTTGTTGAA	60	175	--	--	--	--	--	--
TR36726|c0_g2_i1	XLOC_012647	(CT)10	20	TR36726|c0_g2_i1	(CT)10	Ca_LG_6	(CT)7	1734949	1734962	AGGAAACTAACCGCAAAGCA	60	CCCCAAGGAGAACCAATTTT	60	233	Ca_09451	URR	--	--	--	--
TR36739|c0_g1_i1	No	(TCTCA)5	25	--	--	--	--	--	--	AGCAGCAACACCAATCTCCT	60	TGTGAAAGCCAAAAGAAGCA	60	236	--	--	--	--	--	--
TR36739|c0_g2_i1	No	(TCTCA)5	25	--	--	--	--	--	--	AGCAGCAACACCAATCTCCT	60	TGTGAAAGCCAAAAGAAGCA	60	237	--	--	--	--	--	--
TR36739|c0_g3_i1	No	(TCTCA)5	25	--	--	--	--	--	--	AGCAGCAACACCAATCTCCT	60	TGTGAAAGCCAAAAGAAGCA	60	236	--	--	--	--	--	--
TR36748|c0_g1_i1	No	(A)11ttacattacttaaaatataaaaataataaaaattcaaaacaaatagttcttttagccgctgattttttttgcagcagaaaagaaccagttttga(T)11	117	--	--	--	--	--	--	TTCCAAATTCCAACTGTAGTACCAT	60	GACATGTCGTGGACAGTTGC	60	235	--	--	--	--	--	--
TR36748|c0_g1_i2	No	(A)11ttacattacttaaaatataaaaataataaaaattcaaaacaaatagttcttttagccgctgattttttttgcagcagaaaagaaccagttttga(T)11	117	--	--	--	--	--	--	TTCCAAATTCCAACTGTAGTACCAT	60	GACATGTCGTGGACAGTTGC	60	235	--	--	--	--	--	--

Transcript IDs	Differentially expressed gene (DEG) IDs	SSR repeat-motifs	SSR repeat-length (bp)	*In silico* polymorphic SSRs						Forward primers (5'-3')	Annealing tempearture(0C)	Reverse primers (5'-3')	Annealing tempearture(0C)	Amplified product size (bp)	Structural annotation		Functional Annotation			
				C. microphyllum		ICC4958									Gene IDs	Sequence component of genes	NCBI nr IDs	TFs	KOG	KEGG
				Transcript	SSR repeat-motifs	Chromosomes	SSR repeat-motifs	Start physical positions (bp)	End physical positions (bp)											
TR36751|c0_g1_i1	No	(TTG)5	15	--	--	--	--	--	--	TCTCTATTCCTTGAAGGGCG	59	GGAAGGTAACAAACCAGCCA	60	264	--	--	--	--	--	--
TR36751|c0_g2_i1	No	(TTG)5	15	--	--	--	--	--	--	TCTCTATTCCTTGAAGGGCG	59	GGAAGGTAACAAACCAGCCA	60	264	--	--	--	--	--	--
TR36764|c0_g1_i1	XLOC_012677	(TC)8	16	--	--	--	--	--	--	CCTTCTCATAATTCCCAACCA	59	TGCAAAGGGGCATCTCTAAT	60	145	--	--	--	--	--	--
TR36768|c0_g1_i1	XLOC_012681	(TA)8	16	--	--	--	--	--	--	TTGAAGCAATCCAATTTCACA	59	CATGCCTTAGAAGGTTGGGA	60	232	--	--	--	--	--	--
TR36779|c7_g2_i2	XLOC_012704	(CTT)5	15	--	--	--	--	--	--	CGGAAAAATGCACTCCAAAT	60	TTGGCAAGCTTTTGAGAGGT	60	246	--	--	--	--	--	--
TR36780|c0_g1_i1	No	(AG)7	14	TR36780|c0_g1_i1	(AG)7	Ca_LG_5	(AG)9	20996531	20996548	AAGTTTCCATTGGCTTTAGGC	60	GAATGGTCGAAAGAATCGGA	60	265	Ca_04548	CDS	--	--	--	--
TR36780|c0_g1_i3	No	(AG)7	14	--	--	--	--	--	--	AAGTTTCCATTGGCTTTAGGC	60	GAATGGTCGAAAGAATCGGA	60	265	--	--	--	--	--	--
TR36780|c0_g1_i4	No	(AG)7	14	--	--	--	--	--	--	AAGTTTCCATTGGCTTTAGGC	60	GAATGGTCGAAAGAATCGGA	60	265	--	--	--	--	--	--
TR36780|c0_g1_i5	No	(AG)7	14	--	--	--	--	--	--	AAGTTTCCATTGGCTTTAGGC	60	GAATGGTCGAAAGAATCGGA	60	265	--	--	--	--	--	--
TR36780|c0_g1_i6	No	(AG)7	14	--	--	--	--	--	--	AAGTTTCCATTGGCTTTAGGC	60	GAATGGTCGAAAGAATCGGA	60	265	--	--	--	--	--	--
TR36781|c0_g1_i1	No	(TCT)5(TCC)8tcttcctcctcttcgtgtttactggaccttgagcggtactttctttttgatcgtctttc(ATT)5	113	--	--	--	--	--	--	GGAGCGTTTGTGATCCCTTA	60	TCACATGTCTGAGGCAAAGC	60	211	--	--	--	--	--	--
TR36781|c0_g1_i2	No	(TCT)5(TCC)8tcttcctcctcttcgtgtttactggaccttgagcggtactttctttttgatcgtctttc(ATT)5	113	--	--	--	--	--	--	GGAGCGTTTGTGATCCCTTA	60	TCACATGTCTGAGGCAAAGC	60	211	--	--	--	--	--	--
TR36781|c0_g1_i3	No	(TCT)5(TCC)8tcttcctcctcttcgtgtttactggaccttgagcggtactttctttttgatcgtctttc(ATT)5	113	--	--	--	--	--	--	GGAGCGTTTGTGATCCCTTA	60	TCACATGTCTGAGGCAAAGC	60	211	--	--	--	--	--	--
TR36781|c0_g1_i4	No	(TCT)5(TCC)8tcttcctcctcttcgtgtttactggaccttgagcggtactttctttttgatcgtctttc(ATT)5	113	--	--	--	--	--	--	GGAGCGTTTGTGATCCCTTA	60	TCACATGTCTGAGGCAAAGC	60	211	--	--	--	--	--	--

Transcript IDs	Differentially expressed gene (DEG) IDs	SSR repeat-motifs	SSR repeat-length (bp)	*In silico* polymorphic SSRs						Forward primers (5'-3')	Annealing tempearture(0C)	Reverse primers (5'-3')	Annealing tempearture(0C)	Amplified product size (bp)	Structural annotation		Functional Annotation			
				C. microphyllum		ICC4958									Gene IDs	Sequence component of genes	NCBI nr IDs	TFs	KOG	KEGG
				Transcript	SSR repeat-motifs	Chromosomes	SSR repeat-motifs	Start physical positions (bp)	End physical positions (bp)											
TR36781|c0_g1_i5	No	(TCT)5(TCC)8tcttcctcctcttcgtgtttactggaccttgagcggtactttctttttgatcgtctttc(ATT)5	113	--	--	--	--	--	--	GGAGCGTTTGTGATCCCTTA	60	TCACATGTCTGAGGCAAAGC	60	211	--	--	--	--	--	--
TR36781|c0_g1_i6	No	(TCT)5(TCC)8tcttcctcctcttcgtgtttactggaccttgagcggtactttctttttgatcgtctttc(ATT)5	113	--	--	--	--	--	--	GGAGCGTTTGTGATCCCTTA	60	TCACATGTCTGAGGCAAAGC	60	211	--	--	--	--	--	--
TR36781|c0_g1_i7	No	(TCT)5(TCC)8tcttcctcctcttcgtgtttactggaccttgagcggtactttctttttgatcgtctttc(ATT)5	113	--	--	--	--	--	--	GGAGCGTTTGTGATCCCTTA	60	TCACATGTCTGAGGCAAAGC	60	211	--	--	--	--	--	--
TR36781|c0_g1_i8	No	(TCT)5(TCC)8tcttcctcctcttcgtgtttactggaccttgagcggtactttctttttgatcgtctttc(ATT)5	113	--	--	--	--	--	--	GGAGCGTTTGTGATCCCTTA	60	TCACATGTCTGAGGCAAAGC	60	211	--	--	--	--	--	--
TR36785|c0_g1_i2	XLOC_012718	(TC)10catttttatattatatcatcaccatcgtcattttcttatattcttctttcttttataagataaacatctttccctattttcaacaatttctctctctaa(T)13	133	--	--	--	--	--	--	TGCAGCGTTAAAAGAACCCT	60	CACAATTTTCAGTGAATCTGGG	59	268	--	--	--	--	--	--
TR36785|c1_g1_i1	XLOC_012720	(AT)6	12	--	--	--	--	--	--	AACTGTCATTGCTTCTGGGG	60	CTGGCACAGCAAGATCAAAA	60	243	--	--	--	--	--	--
TR36785|c2_g1_i1	XLOC_012721	(TC)9	18	--	--	--	--	--	--	TCCTGATGACCCTTGTCTCC	60	CCTTGAAATATTCAACCAACCA	59	102	--	--	XM_004508567.1	--	[X]	--
TR36785|c3_g1_i1	No	(TG)7(T)11	25	--	--	--	--	--	--	TGATTGCATGTGCATGTGTT	60	CTCGCGTGGTAGGTAGTTGA	59	244	--	--	--	--	--	--
TR36817|c0_g1_i1	No	(CTT)6	18	--	--	--	--	--	--	CGACGTCGTATGGAGTTGTG	60	TAGGACGTGGGCCTATGAAA	60	269	--	--	--	--	--	--
TR36817|c0_g1_i2	No	(CTT)6	18	--	--	--	--	--	--	TATATACACCCCCTCCCCCT	59	CCAAAACATGGAAGTGGCTT	60	236	--	--	--	--	--	--
TR36817|c0_g1_i3	No	(CTT)6	18	--	--	--	--	--	--	CGACGTCGTATGGAGTTGTG	60	TAGGACGTGGGCCTATGAAA	60	269	--	--	--	--	--	--
TR36817|c0_g1_i4	No	(CTT)6	18	--	--	--	--	--	--	TATATACACCCCCTCCCCCT	59	CCAAAACATGGAAGTGGCTT	60	236	--	--	--	--	--	--
TR36820|c1_g1_i1	XLOC_012762	(CA)6	12	--	--	--	--	--	--	TTTCTGATCTGTTTCTGCTCCA	60	TCGCAGAACTATATCCGCCT	60	225	--	--	--	--	--	--
TR36824|c0_g1_i2	XLOC_012765	(A)11(AAT)5*	24	--	--	--	--	--	--	TAGGCCAATCATCAGCAACA	60	CAGGGAGGTACAATAGCCCA	60	276	--	--	XP_004516108.1	--	[F]	--

Transcript IDs	Differentially expressed gene (DEG) IDs	SSR repeat-motifs	SSR repeat-length (bp)	*In silico* polymorphic SSRs						Forward primers (5'-3')	Annealing tempearture(0C)	Reverse primers (5'-3')	Annealing tempearture(0C)	Amplified product size (bp)	Structural annotation		Functional Annotation			
				C. microphyllum		ICC4958									Gene IDs	Sequence component of genes	NCBI nr IDs	TFs	KOG	KEGG
				Transcript	SSR repeat-motifs	Chromosomes	SSR repeat-motifs	Start physical positions (bp)	End physical positions (bp)											
TR36825|c0_g1_i2	No	(CTT)7	21	--	--	--	--	--	--	CCTTCTTCCACATTCAACAACA	60	AGCACAATATTACCGGCGTC	60	233	--	--	--	--	--	--
TR36825|c0_g1_i3	XLOC_012766	(CTT)7	21	--	--	--	--	--	--	CCTTCTTCCACATTCAACAACA	60	CCCTGTGGGAGAGAGTTGAG	60	121	--	--	--	--	--	--
TR36827|c0_g2_i1	No	(TA)7	14	TR36827|c0_g2_i1	(TA)7	Ca_LG_8	(TA)6	14175488	14175499	GCCCTTCGTCCCAAAATATAA	60	CAACGCAATGGGGTAAAATC	60	231	Ca_20291	URR	--	--	--	--
TR36827|c0_g2_i10	No	(TA)7	14	--	--	--	--	--	--	GCCCTTCGTCCCAAAATATAA	60	CAACGCAATGGGGTAAAATC	60	231	--	--	--	--	--	--
TR36827|c0_g2_i11	No	(TA)7	14	--	--	--	--	--	--	GCCCTTCGTCCCAAAATATAA	60	CAACGCAATGGGGTAAAATC	60	231	--	--	--	--	--	--
TR36827|c0_g2_i12	No	(TA)7	14	--	--	--	--	--	--	GCCCTTCGTCCCAAAATATAA	60	CAACGCAATGGGGTAAAATC	60	231	--	--	--	--	--	--
TR36827|c0_g2_i13	No	(TA)7	14	--	--	--	--	--	--	GCCCTTCGTCCCAAAATATAA	60	CAACGCAATGGGGTAAAATC	60	231	--	--	--	--	--	--
TR36827|c0_g2_i14	No	(TA)7	14	--	--	--	--	--	--	GCCCTTCGTCCCAAAATATAA	60	CAACGCAATGGGGTAAAATC	60	231	--	--	--	--	--	--
TR36827|c0_g2_i15	No	(TA)7	14	--	--	--	--	--	--	GCCCTTCGTCCCAAAATATAA	60	CAACGCAATGGGGTAAAATC	60	231	--	--	--	--	--	--
TR36827|c0_g2_i16	No	(TA)7	14	--	--	--	--	--	--	GCCCTTCGTCCCAAAATATAA	60	CAACGCAATGGGGTAAAATC	60	231	--	--	--	--	--	--
TR36827|c0_g2_i2	No	(TA)7	14	--	--	--	--	--	--	GCCCTTCGTCCCAAAATATAA	60	CAACGCAATGGGGTAAAATC	60	231	--	--	--	--	--	--
TR36827|c0_g2_i3	No	(TA)7	14	--	--	--	--	--	--	GCCCTTCGTCCCAAAATATAA	60	CAACGCAATGGGGTAAAATC	60	231	--	--	--	--	--	--
TR36827|c0_g2_i4	No	(TA)7	14	--	--	--	--	--	--	GCCCTTCGTCCCAAAATATAA	60	CAACGCAATGGGGTAAAATC	60	231	--	--	--	--	--	--
TR36827|c0_g2_i5	No	(TA)7	14	--	--	--	--	--	--	GCCCTTCGTCCCAAAATATAA	60	CAACGCAATGGGGTAAAATC	60	231	--	--	--	--	--	--

Transcript IDs	Differentially expressed gene (DEG) IDs	SSR repeat-motifs	SSR repeat-length (bp)	*In silico* polymorphic SSRs						Forward primers (5'-3')	Annealing tempearture(0C)	Reverse primers (5'-3')	Annealing tempearture(0C)	Amplified product size (bp)	Structural annotation		Functional Annotation			
				C. microphyllum		ICC4958									Gene IDs	Sequence component of genes	NCBI nr IDs	TFs	KOG	KEGG
				Transcript	SSR repeat-motifs	Chromosomes	SSR repeat-motifs	Start physical positions (bp)	End physical positions (bp)											
TR36827|c0_g2_i6	No	(TA)7	14	--	--	--	--	--	--	GCCCTTCGTCCCAAAATATAA	60	CAACGCAATGGGGTAAAATC	60	231	--	--	--	--	--	--
TR36827|c0_g2_i7	No	(TA)7	14	--	--	--	--	--	--	GCCCTTCGTCCCAAAATATAA	60	CAACGCAATGGGGTAAAATC	60	231	--	--	--	--	--	--
TR36827|c0_g2_i8	No	(TA)7	14	--	--	--	--	--	--	GCCCTTCGTCCCAAAATATAA	60	CAACGCAATGGGGTAAAATC	60	231	--	--	--	--	--	--
TR36827|c0_g2_i9	No	(TA)7	14	--	--	--	--	--	--	GCCCTTCGTCCCAAAATATAA	60	CAACGCAATGGGGTAAAATC	60	231	--	--	--	--	--	--
TR36828|c0_g1_i1	No	(CT)9	18	--	--	--	--	--	--	CAGATGCAAAGCAAAGCAAC	60	TTGAATTGGATTGGAGAGGC	60	136	--	--	--	--	--	--
TR36828|c0_g1_i2	No	(CT)9	18	--	--	--	--	--	--	CAGATGCAAAGCAAAGCAAC	60	TTGAATTGGATTGGAGAGGC	60	136	--	--	--	--	--	--
TR36828|c0_g1_i3	No	(CT)9	18	--	--	--	--	--	--	CAGATGCAAAGCAAAGCAAC	60	TTGAATTGGATTGGAGAGGC	60	136	--	--	--	--	--	--
TR36828|c0_g1_i4	XLOC_012769	(CT)8	16	--	--	--	--	--	--	GCCGAATTTCAAAACTCCAA	60	GCCAGCGTAACCGCTACTAC	60	219	--	--	--	--	--	--
TR36842|c0_g1_i1	XLOC_012790	(ACA)6	18	--	--	--	--	--	--	TCACATTCCACATTGCACCT	60	ATTTGGAGCGAGAAGCTGAA	60	227	--	--	--	--	--	--
TR36842|c0_g1_i3	XLOC_012791	(ACA)6	18	--	--	--	--	--	--	TCACATTCCACATTGCACCT	60	ATTTGGAGCGAGAAGCTGAA	60	227	--	--	--	--	--	--
TR36846|c0_g1_i2	No	(CA)7	14	TR36846|c0_g1_i2	(CA)7	Ca_LG_2	(CA)6	1213104	1213115	CACGTGACTTGTTTCCCCTT	60	TGGAAAAGGATTCAGACCAA	58	252	Ca_29583	DRR	--	--	--	--
TR36846|c0_g1_i3	No	(CA)7	14	--	--	--	--	--	--	CACGTGACTTGTTTCCCCTT	60	TGGAAAAGGATTCAGACCAA	58	252	--	--	--	--	--	--
TR36846|c0_g1_i4	No	(CA)7	14	--	--	--	--	--	--	CACGTGACTTGTTTCCCCTT	60	TGGAAAAGGATTCAGACCAA	58	252	--	--	--	--	--	--
TR36847|c0_g1_i1	No	(TCT)6	18	--	--	--	--	--	--	TTCTAGAAAACGTGGGGGTG	60	TCACCTTTTCCCAAATCAGG	60	259	--	--	--	--	--	--

Transcript IDs	Differentially expressed gene (DEG) IDs	SSR repeat-motifs	SSR repeat-length (bp)	*In silico* polymorphic SSRs						Forward primers (5'-3')	Annealing tempearture(0C)	Reverse primers (5'-3')	Annealing tempearture(0C)	Amplified product size (bp)	Structural annotation		Functional Annotation			
				C. microphyllum		ICC4958									Gene IDs	Sequence component of genes	NCBI nr IDs	TFs	KOG	KEGG
				Transcript	SSR repeat-motifs	Chromosomes	SSR repeat-motifs	Start physical positions (bp)	End physical positions (bp)											
TR36847|c0_g2_i1	No	(TCT)6	18	--	--	--	--	--	--	TTCTAGAAAACGTGGGGGTG	60	TCACCTTTTCCCAAATCAGG	60	259	--	--	--	--	--	--
TR36847|c1_g1_i1	XLOC_012792	(AAG)5	15	--	--	--	--	--	--	GGTGTTGTTGTTGTTGTTGGA	59	AGTGATGCGCTCAGGAGTTT	60	276	--	--	--	--	--	--
TR36847|c1_g1_i1	XLOC_012792	(TGT)5	15	--	--	--	--	--	--	TGATGGTTTTGTTCAATGCAA	60	AACTCCTCTTCATGTTGCTGC	59	264	--	--	--	--	--	--
TR36847|c2_g1_i1	XLOC_012793	(TCA)5	15	--	--	--	--	--	--	TTTCCCCACACAATTTTTCC	60	TGACTTGAACATGGAAGCTGA	59	247	--	--	--	--	--	--
TR36849|c0_g2_i2	No	(GAA)6	18	--	--	--	--	--	--	GTGATTCGCGAGAAGGACAT	60	TTCCTTTGAATTCGGTTTGG	60	249	--	--	--	--	--	--
TR36849|c0_g2_i3	No	(GAA)6	18	--	--	--	--	--	--	GTGATTCGCGAGAAGGACAT	60	TTCCTTTGAATTCGGTTTGG	60	249	--	--	--	--	--	--
TR36849|c0_g2_i4	No	(GAA)6	18	--	--	--	--	--	--	GTGATTCGCGAGAAGGACAT	60	TTCCTTTGAATTCGGTTTGG	60	249	--	--	--	--	--	--
TR36849|c0_g2_i5	No	(GAA)6	18	--	--	--	--	--	--	GTGATTCGCGAGAAGGACAT	60	TTCCTTTGAATTCGGTTTGG	60	249	--	--	--	--	--	--
TR36849|c0_g2_i7	No	(GAA)6	18	--	--	--	--	--	--	GTGATTCGCGAGAAGGACAT	60	TTCCTTTGAATTCGGTTTGG	60	249	--	--	--	--	--	--
TR36849|c0_g2_i9	No	(GAA)6	18	--	--	--	--	--	--	GTGATTCGCGAGAAGGACAT	60	TTCCTTTGAATTCGGTTTGG	60	249	--	--	--	--	--	--
TR36849|c0_g4_i1	No	(GAA)8	24	--	--	--	--	--	--	TAATAAAAGACGAGGCTGCG	58	CTCCATCAATGGAAGGGTGT	60	269	--	--	--	--	--	--
TR36849|c0_g4_i2	No	(GAA)8	24	--	--	--	--	--	--	TAATAAAAGACGAGGCTGCG	58	CTCCATCAATGGAAGGGTGT	60	269	--	--	--	--	--	--
TR36849|c0_g4_i4	No	(GAA)8	24	--	--	--	--	--	--	TAATAAAAGACGAGGCTGCG	58	CTCCATCAATGGAAGGGTGT	60	269	--	--	--	--	--	--
TR36849|c0_g4_i5	No	(GAA)8	24	--	--	--	--	--	--	TAATAAAAGACGAGGCTGCG	58	CTCCATCAATGGAAGGGTGT	60	269	--	--	--	--	--	--

Transcript IDs	Differentially expressed gene (DEG) IDs	SSR repeat-motifs	SSR repeat-length (bp)	*In silico* polymorphic SSRs						Forward primers (5'-3')	Annealing tempearture(0C)	Reverse primers (5'-3')	Annealing tempearture(0C)	Amplified product size (bp)	Structural annotation		Functional Annotation			
				C. microphyllum		ICC4958									Gene IDs	Sequence component of genes	NCBI nr IDs	TFs	KOG	KEGG
				Transcript	SSR repeat-motifs	Chromosomes	SSR repeat-motifs	Start physical positions (bp)	End physical positions (bp)											
TR36864|c6_g2_i1	XLOC_012818	(CT)10	20	--	--	--	--	--	--	ACCAAAGTCACTTGGCCATT	59	AGGAGGCAAATAGAGAGGGG	60	201	--	--	--	--	--	--
TR36864|c6_g2_i1	XLOC_012818	(CT)9	18	--	--	--	--	--	--	CCCCTCTCTATTTGCCTCCT	60	AAAACCGGCGACCTAGAGAT	60	252	--	--	--	--	--	--
TR36864|c6_g3_i1	No	(CT)9cacagtagtttctgaactcactcacaaaattccataa caatttctgggattttctttcattcatatttgaaagaaagaaaa aacaatcacacat(A)10	122	--	--	--	--	--	--	CCCCTCTCTATTTGCCTCCT	60	AACTCTAAAGGGTCATGTCCAAA	59	205	--	--	--	--	--	--
TR36864|c6_g4_i1	No	(CT)9cacagtagtttctgaactcactcacaaaattccataa caatttctgggattttctttcattcatatttgaaagaaagaaaa aacaatcacacat(A)10	122	--	--	--	--	--	--	CCCCTCTCTATTTGCCTCCT	60	AACTCTAAAGGGTCATGTCCAAA	59	205	--	--	--	--	--	--
TR36864|c6_g7_i1	No	(AAG)5	15	--	--	--	--	--	--	ATTGGCCGAGAAGACAGAAA	60	TCCCAAGGATAACGAGGATG	60	198	--	--	--	--	--	--
TR36864|c6_g7_i2	No	(AAG)5	15	--	--	--	--	--	--	ATTGGCCGAGAAGACAGAAA	60	TCCCAAGGATAACGAGGATG	60	198	--	--	--	--	--	--
TR36864|c6_g7_i3	No	(AAG)5	15	--	--	--	--	--	--	CGCAGATGAAAGAACGCATA	60	AGTGCCAAATTGCTTCCATC	60	276	--	--	--	--	--	--
TR36864|c6_g8_i1	No	(AAG)5	15	--	--	--	--	--	--	CAGTACGTTAGCTGGTGCGA	60	TTAAACACCCTGCCCAAAAG	60	207	--	--	--	--	--	--
TR36864|c6_g8_i1	No	(CT)9	18	--	--	--	--	--	--	GACCAGCAGATTTCTCTCGC	60	CAGCCAACTTGTCCACTGAA	60	196	--	--	--	--	--	--
TR36864|c6_g8_i2	No	(AAG)5	15	--	--	--	--	--	--	CAGTACGTTAGCTGGTGCGA	60	GGTAACGAGGTTGCAAGGAA	60	149	--	--	--	--	--	--
TR36864|c7_g1_i1	No	(TC)8	16	--	--	--	--	--	--	TGCTCCAACCGATACAACAA	60	ACGGGAGAACGAGTTAGGGT	60	277	--	--	--	--	--	--
TR36864|c7_g1_i2	No	(CT)7	14	--	--	--	--	--	--	GGCTTCGACGTTTGTTCTCT	59	ACGGGAGAACGAGTTAGGGT	60	223	--	--	--	--	--	--
TR36864|c7_g1_i3	No	(CT)7	14	--	--	--	--	--	--	TCCAGATTGCAACCTTCTCC	60	AGAGCAAACGAGAGAGAGCG	60	194	--	--	--	--	--	--
TR36864|c7_g1_i3	No	(CTT)5	15	--	--	--	--	--	--	TGTTTCTATGGAACCCTAAACCC	60	AGAGCAAACGAGAGAGAGCG	60	243	--	--	--	--	--	--

Transcript IDs	Differentially expressed gene (DEG) IDs	SSR repeat-motifs	SSR repeat-length (bp)	*In silico* polymorphic SSRs						Forward primers (5'-3')	Annealing tempearture(0C)	Reverse primers (5'-3')	Annealing tempearture(0C)	Amplified product size (bp)	Structural annotation		Functional Annotation			
				C. microphyllum		ICC4958									Gene IDs	Sequence component of genes	NCBI nr IDs	TFs	KOG	KEGG
				Transcript	SSR repeat-motifs	Chromosomes	SSR repeat-motifs	Start physical positions (bp)	End physical positions (bp)											
TR36864|c7_g1_i5	No	(CT)7	14	--	--	--	--	--	--	GGCTTCGACGTTTGTTCTCT	59	ACGGGAGAACGAGTTAGGGT	60	223	--	--	--	--	--	--
TR36864|c7_g1_i6	No	(CTT)5	15	--	--	--	--	--	--	TGTTTCTATGGAACCCTAAACCC	60	TGTGAACGAGAGAGAGCGAA	60	144	--	--	--	--	--	--
TR36864|c7_g1_i7	No	(CT)7	14	--	--	--	--	--	--	TTCGCTCTCTCTCGTTCACA	60	CCAAAACAAAACGGGAGAAC	59	221	--	--	--	--	--	--
TR36864|c7_g1_i8	No	(TC)8	16	--	--	--	--	--	--	TGCTCCAACCGATACAACAA	60	ACGGGAGAACGAGTTAGGGT	60	277	--	--	--	--	--	--
TR36864|c7_g2_i1	XLOC_012819	(CT)7	14	--	--	--	--	--	--	CGTCGGTTAGAGCTCTCTGG	60	CGACAACGAACGAGAGTGAA	60	164	--	--	--	--	--	--
TR36866|c0_g1_i1	XLOC_012825	(TTC)5	15	--	--	--	--	--	--	CGACGAATCTAGCTAACGGC	60	AACGTCCTCAAATTCCATCG	60	263	--	--	--	--	--	--
TR36866|c0_g1_i1	XLOC_012825	(CT)6tctgtaacttccgccatagaacacaaaccgaaattc tttcttcgtgtccaataaacaaaaac(A)12	86	--	--	--	--	--	--	CGTGTCTAAATCTCCCGCTC	60	AACCTTGAAATGGTTCAGCG	60	236	--	--	--	--	--	--
TR36868|c0_g3_i1	No	(AC)8	16	TR36868|c0_g3_i1	(AC)8	Ca_LG_4	(AC)7	2382038	2382051	TGATGCAACTAAGCCTTGACA	59	CGAACACAACATGAGCCTTG	60	268	Ca_02743	CDS	--	--	--	--
TR36873|c1_g1_i1	XLOC_012839	(AGC)7	21	--	--	--	--	--	--	AAAGCAAGAGGGTGGTGATG	60	GGTTGGCTTAGACCAAGTCTG	59	240	--	--	XM_004508606.1	TCP	[K]	--
TR36889|c0_g1_i1	No	(AAAAT)5	25	--	--	--	--	--	--	TCCCACTTGAAACCATGAATC	60	CCCTGTTGAAATGGAACGAA	61	139	--	--	--	--	--	--
TR370|c0_g1_i2	XLOC_012906	(CT)7	14	--	--	--	--	--	--	TGCTTCCGTTTCAATCATCA	60	AGCAAAAGGCTTGACTGCAT	60	135	--	--	--	--	--	--
TR37000|c0_g1_i1	XLOC_012874	(AGT)5	15	--	--	--	--	--	--	CAACAAAAAGAAGAAGAGCATGG	60	TTTTCCCGTTCGTTTGAGAC	60	181	--	--	--	--	--	--
TR37057|c0_g1_i1	No	(TAA)5	15	--	--	--	--	--	--	ACGAGGAAACAAATGGCATC	60	GTCATAGGCATCTGCTGCAA	60	221	--	--	--	--	--	--
TR37057|c0_g2_i1	No	(TAA)5	15	--	--	--	--	--	--	ACGAGGAAACAAATGGCATC	60	GTCATAGGCATCTGCTGCAA	60	221	--	--	--	--	--	--

Transcript IDs	Differentially expressed gene (DEG) IDs	SSR repeat-motifs	SSR repeat-length (bp)	*In silico* polymorphic SSRs						Forward primers (5'-3')	Annealing tempearture(0C)	Reverse primers (5'-3')	Annealing tempearture(0C)	Amplified product size (bp)	Structural annotation		Functional Annotation			
				C. microphyllum		ICC4958									Gene IDs	Sequence component of genes	NCBI nr IDs	TFs	KOG	KEGG
				Transcript	SSR repeat-motifs	Chromosomes	SSR repeat-motifs	Start physical positions (bp)	End physical positions (bp)											
TR37089|c0_g1_i1	No	(TCA)5	15	--	--	--	--	--	--	CTCCTGAACTTGCTTTTGGC	60	AGGAAATGCGCAATTTATGG	60	204	--	--	--	--	--	--
TR372|c0_g1_i1	No	(ATA)8	24	TR372|c0_g1_i1	(ATA)8	Ca_LG_1	(ATA)5	5477498	5477512	AACAATTCTCACAGGAGTTTGGA	60	GGTTGACCAAAGAGCCAAAA	60	101	Ca_18606	DRR	--	--	--	--
TR372|c0_g2_i1	XLOC_012946	(ATA)8	24	--	--	--	--	--	--	AACAATTCTCACAGGAGTTTGGA	60	GGTTGACCAAAGAGCCAAAA	60	101	--	--	--	--	--	--
TR37231|c0_g1_i1	No	(GA)6	12	--	--	--	--	--	--	GCTAAGAAGGCTCAAAGGGA	59	GACACTTGGCACAAGATCCA	60	177	--	--	--	--	--	--
TR3726|c0_g1_i1	XLOC_012937	(CTT)5	15	--	--	--	--	--	--	CGTTAGCTGTACATCACCGC	59	GCTGGTTAGGTGGACGGTTA	60	193	--	--	--	--	--	--
TR37263|c0_g1_i1	XLOC_012934	(TAA)5	15	--	--	--	--	--	--	GCAAGACATTTGGGCGTTAT	60	TGGTTTCTCATTGAAGCCAT	58	229	--	--	XP_012570780.1	--	--	--
TR37287|c0_g1_i1	XLOC_012942	(TC)6	12	--	--	--	--	--	--	ACAGCAACCACCAACATGAG	60	GTTCCAACGAACAATGCTGA	60	278	--	--	XP_003740563.1	--	[K]	--
TR37287|c0_g1_i1	XLOC_012942	(AC)7	14	--	--	--	--	--	--	GCTTCACGCAAAACGAAAAT	60	TTGGTGGTTGCTGTCTTTGA	60	161	--	--	XP_003740563.1	--	[K]	--
TR37366|c0_g1_i1	XLOC_012965	(GTT)5	15	--	--	--	--	--	--	CATGGACATAAGCAGCGAAA	60	CTTGCTCCACTACCACCCAT	60	268	--	--	--	--	--	--
TR374|c0_g1_i1	XLOC_013001	(CAA)7	21	--	--	--	--	--	--	GAAACACACGAGTCATCCCC	60	TGTAAGCGATTGCTGAAACG	60	184	--	--	--	--	--	--
TR37417|c0_g1_i1	No	(TC)7gctc(T)10	28	--	--	--	--	--	--	TCCACCACATCGTCTCCATA	60	CGATGATGCCAATTAGGTCC	60	176	--	--	--	--	--	--
TR37425|c0_g1_i1	No	(GTT)5	15	--	--	--	--	--	--	TCTTCTTGTTGGTTGCAAATG	59	TGAATTCAATTTTGGTTGGGA	60	155	--	--	--	--	--	--
TR37510|c0_g1_i1	XLOC_013006	(CTA)6	18	--	--	--	--	--	--	TCCCGTGAAACTGTCACAAA	60	TGGAGAAGCTGAAATTGTTGG	60	278	--	--	--	--	--	--
TR37743|c0_g1_i1	No	(GTC)5	15	--	--	--	--	--	--	AAAATTACCCGAAGCGACG	60	CTTGGGTGATGCATGATACG	60	166	--	--	--	--	--	--

Transcript IDs	Differentially expressed gene (DEG) IDs	SSR repeat-motifs	SSR repeat-length (bp)	*In silico* polymorphic SSRs						Forward primers (5'-3')	Annealing tempearture(0C)	Reverse primers (5'-3')	Annealing tempearture(0C)	Amplified product size (bp)	Structural annotation		Functional Annotation			
				C. microphyllum		ICC4958									Gene IDs	Sequence component of genes	NCBI nr IDs	TFs	KOG	KEGG
				Transcript	SSR repeat-motifs	Chromosomes	SSR repeat-motifs	Start physical positions (bp)	End physical positions (bp)											
TR37743|c0_g2_i1	No	(GTC)5	15	--	--	--	--	--	--	AAAATTACCCGAAGCGACG	60	CTTGGGTGATGCATGATACG	60	166	--	--	--	--	--	--
TR37749|c0_g1_i1	XLOC_013078	(ATC)7	21	--	--	--	--	--	--	AACAAGAAGAAAATGGGTATCATTA	57	ATTTGCGCGCTTTGTAATCT	60	129	--	--	XP_004500347.1	--	--	--
TR37749|c0_g1_i1	XLOC_013078	(T)10cctcaatcaatcccttctacgaagaatgtgattgagaaatgtgaaagaatgtggtggctcagttagaacaagg(A)10	93	--	--	--	--	--	--	TTCTGAGGACGAAGAAACCG	60	TGGTCATCATCCACTCTCCA	60	266	--	--	XP_004500347.1	--	--	--
TR37749|c1_g1_i1	XLOC_013079	(CAT)7	21	--	--	--	--	--	--	TCCTCATTTCCACTTACATCCA	59	TCATGGTGATGATGCTGATG	59	237	--	--	XP_005188380.1	--	--	--
TR37753|c0_g1_i1	No	(CTT)6	18	--	--	--	--	--	--	TGGTTATGCACTGACAACGAA	60	TTTGTTTCGGTTTTGCCAAT	60	158	--	--	--	--	--	--
TR37753|c0_g1_i2	No	(CTT)6	18	--	--	--	--	--	--	TGGTTATGCACTGACAACGAA	60	TTTGTTTCGGTTTTGCCAAT	60	158	--	--	--	--	--	--
TR37753|c0_g1_i3	No	(CTT)6	18	--	--	--	--	--	--	TGGTTATGCACTGACAACGAA	60	TTTGTTTCGGTTTTGCCAAT	60	158	--	--	--	--	--	--
TR37753|c0_g1_i4	No	(CTT)6	18	--	--	--	--	--	--	TGGTTATGCACTGACAACGAA	60	TTTGTTTCGGTTTTGCCAAT	60	158	--	--	--	--	--	--
TR37773|c0_g1_i3	No	(AG)9acggtggg(ATCT)5	46	--	--	--	--	--	--	AAGTGATGGTGTCGGAGAGG	60	AGCAGCTTTGGTTTGGTCAC	60	226	--	--	--	--	--	--
TR37788|c0_g1_i1	XLOC_013104	(TCA)5	15	--	--	--	--	--	--	TGAAGCCCATCAATGACAGT	59	GGCATAACAGTCAAAGGGGA	60	280	--	--	--	--	--	--
TR37790|c0_g2_i1	No	(CTG)5	15	--	--	--	--	--	--	GCGATACTACGATGGGGTGT	60	TGCAGCAAATGCTTCAATGT	60	162	--	--	--	--	--	--
TR37806|c0_g1_i1	No	(TTCA)6	24	--	--	--	--	--	--	TTGTTTTCCAACCCATTCGT	60	TCTGTCTTGGGTTGTTGCTG	60	276	--	--	--	--	--	--
TR37806|c0_g1_i2	No	(TTCA)6	24	--	--	--	--	--	--	TTGTTTTCCAACCCATTCGT	60	TCTGTCTTGGGTTGTTGCTG	60	276	--	--	--	--	--	--
TR37811|c0_g1_i1	XLOC_013122	(AT)6	12	--	--	--	--	--	--	TCCTTCCCACACATCAACAA	60	CTGCACTGAGTCATTTCCTCAC	60	251	--	--	--	--	--	--

Transcript IDs	Differentially expressed gene (DEG) IDs	SSR repeat-motifs	SSR repeat-length (bp)	*In silico* polymorphic SSRs						Forward primers (5'-3')	Annealing tempearture(0C)	Reverse primers (5'-3')	Annealing tempearture(0C)	Amplified product size (bp)	Structural annotation		Functional Annotation			
				C. microphyllum		ICC4958									Gene IDs	Sequence component of genes	NCBI nr IDs	TFs	KOG	KEGG
				Transcript	SSR repeat-motifs	Chromosomes	SSR repeat-motifs	Start physical positions (bp)	End physical positions (bp)											
TR37821|c0_g1_i1	No	(ATTA)5	20	--	--	--	--	--	--	CATCCCGCAGATATTCCTGT	60	GAGAAGAAGGGAACGTGCAG	60	206	--	--	--	--	--	--
TR37821|c0_g2_i1	No	(ATTA)5	20	--	--	--	--	--	--	CATCCCGCAGATATTCCTGT	60	GAGAAGAAGGGAACGTGCAG	60	206	--	--	--	--	--	--
TR37821|c0_g3_i1	No	(ATTA)5	20	--	--	--	--	--	--	CATCCCGCAGATATTCCTGT	60	GAGAAGAAGGGAACGTGCAG	60	206	--	--	--	--	--	--
TR37821|c0_g5_i1	No	(ATTA)5	20	--	--	--	--	--	--	CATCCCGCAGATATTCCTGT	60	GAGAAGAAGGGAACGTGCAG	60	206	--	--	--	--	--	--
TR37827|c0_g1_i1	XLOC_013131	(CAA)5aaacacttcactccttccactctcaccaaacaaatatttac(TAT)7	77	--	--	--	--	--	--	TACGGTAGCTCCTCGTTGGT	60	TTCAGAGTGCAGGGTGAGTG	60	195	--	--	--	--	--	--
TR37839|c0_g1_i1	XLOC_013135	(CAT)6	18	--	--	--	--	--	--	TCATTTTCGTGCAGTCCATC	60	GTACGCGAAGCACAAGTCAA	60	244	--	--	--	--	--	--
TR37845|c0_g1_i1	XLOC_013139	(AAG)5	15	--	--	--	--	--	--	TAAATTCCTCACCACGAGCC	60	GTGCGCTTAACCATGAGGAC	61	133	--	--	EUC53601.1	--	--	--
TR37879|c0_g1_i1	XLOC_013153	(GT)6	12	--	--	--	--	--	--	TGGAACGTGCAAAGCTAGAA	60	TGGTTTTTCTCTTCCTCTCCA	59	219	--	--	--	--	--	--
TR37915|c0_g1_i1	XLOC_013170	(CT)9	18	--	--	--	--	--	--	CGTCCCTTCCTTCTCACAAC	60	TAGGCGCGAAAATTATACGG	60	151	--	--	--	--	--	--
TR37915|c0_g2_i1	No	(CT)9	18	--	--	--	--	--	--	CGTCCCTTCCTTCTCACAAC	60	TAGGCGCGAAAATTATACGG	60	151	--	--	--	--	--	--
TR37917|c0_g1_i1	XLOC_013171	(CA)8	16	--	--	--	--	--	--	GCTGTTTATGCAAACAGACCTTT	60	TGGCAGCTATAGCATAGCAAAA	60	182	--	--	--	--	--	--
TR37942|c0_g1_i1	No	(AGA)6	18	--	--	--	--	--	--	ACCCTCTTGCTTTGCTTCAT	59	GCCACGTGACGTTGTCATAA	61	275	--	--	--	--	--	--
TR37942|c0_g1_i1	No	(GCT)7	21	--	--	--	--	--	--	TGTCCATTGTCTTGAGGTGG	60	CCTGCAGAGTTTTCTCTCTCA	57	148	--	--	--	--	--	--
TR37942|c0_g2_i1	No	(AGA)6	18	--	--	--	--	--	--	ATCACCACCCTCTTGCTTTG	60	GGTTGCAGATCGAGGTCATT	60	257	--	--	--	--	--	--

Transcript IDs	Differentially expressed gene (DEG) IDs	SSR repeat-motifs	SSR repeat-length (bp)	*In silico* polymorphic SSRs						Forward primers (5'-3')	Annealing tempearture(0C)	Reverse primers (5'-3')	Annealing tempearture(0C)	Amplified product size (bp)	Structural annotation		Functional Annotation			
				C. microphyllum		ICC4958									Gene IDs	Sequence component of genes	NCBI nr IDs	TFs	KOG	KEGG
				Transcript	SSR repeat-motifs	Chromosomes	SSR repeat-motifs	Start physical positions (bp)	End physical positions (bp)											
TR37942|c0_g2_i1	No	(GCT)7	21	--	--	--	--	--	--	TGTCCATTGTCTTGAGGTGG	60	CCTGCAGAGTTTTCTCTCTCA	57	148	--	--	--	--	--	--
TR37956|c0_g1_i1	No	(TC)9attcaatactttctttcatccattcattccatttcatcatc acaatcta(CT)7	81	--	--	--	--	--	--	CGCTTCCAACGCAATCTTAT	60	GCGAATGTTCTGTTGAAGCA	60	259	--	--	--	--	--	--
TR3797|c0_g1_i1	XLOC_013184	(TC)8	16	--	--	--	--	--	--	TACTTTCCTTTTCCCCTGCC	60	TTGCATGTGTCGGAGTTTGT	60	253	--	--	--	--	--	--
TR38001|c0_g1_i1	No	(TAT)5	15	--	--	--	--	--	--	GTCCCACAATTCCATCCTTG	60	CCCATAACCACTTTGATTTTCC	60	221	--	--	--	--	--	--
TR38033|c0_g1_i1	XLOC_013202	(CCAGT)5	25	--	--	--	--	--	--	CAGGATTGTTTGCTTCACCA	60	CGCGAAGTGTGTTTTCTTCA	60	216	--	--	--	--	--	--
TR38039|c0_g2_i1	No	(CAA)5	15	--	--	--	--	--	--	TGCTGGGTTTATTGTTGCTTC	60	CATTTTAATCAAATCCAACAGCC	60	188	--	--	--	--	--	--
TR38043|c0_g1_i2	XLOC_013217	(GAA)6	18	TR38043|c0_g1_i2	(GAA)6	Ca_LG_6	(GAA)5	8913949	8913963	CAGTCCACCTTGTTTCGGAT	60	AACGACGACGTATCAGGTCC	60	166	Ca_13461	DRR	--	--	--	--
TR38043|c0_g2_i1	No	(GAA)6	18	--	--	--	--	--	--	CAGTCCACCTTGTTTCGGAT	60	AACGACGACGTATCAGGTCC	60	166	--	--	--	--	--	--
TR38049|c0_g1_i5	No	(CAA)5	15	--	--	--	--	--	--	AGGATGCAAACCAAGCTTTC	59	CCGTGTCTTCGGGTAACAAT	60	226	--	--	--	--	--	--
TR38051|c1_g1_i1	No	(TTC)5atatcaaattactataatttttatatatattgtttggtgt aatgtttctga(T)10	76	--	--	--	--	--	--	TCCGCAGAGTCTCTCTCACA	60	CCTCTGGCTTCTTTTCTTCC	58	168	--	--	--	--	--	--
TR38053|c1_g1_i1	XLOC_013231	(GAA)6	18	TR38053|c1_g1_i1	(GAA)6	Ca_LG_3	(GAA)5	26591101	26591115	TCAGTCCAGCATAGGCATCA	60	GGCGTAAAGAAGCTGAGGTG	60	262		URR	XM_004492613.1	G2-like	[OR]	--
TR38053|c2_g1_i1	No	(CT)6	12	--	--	--	--	--	--	TGTCCTGTTTTGGTTATCCATGT	61	ATTTGAGGGGTGACTTGGTG	60	228	--	--	--	--	--	--
TR38062|c0_g1_i1	XLOC_013240	(TC)6	12	--	--	--	--	--	--	CAAATCTGTTCTGTACTTCCATTG	58	TGATCCACCCCTTCTCACTC	60	279	--	--	ETI33379.1	--	[Q]	--
TR38063|c0_g2_i1	XLOC_013242	(AAC)5	15	--	--	--	--	--	--	CGCTTTTTGACAAGCATTTG	59	AATTTGCATCAATGCTGCTG	60	275	--	--	--	--	--	--

Transcript IDs	Differentially expressed gene (DEG) IDs	SSR repeat-motifs	SSR repeat-length (bp)	*In silico* polymorphic SSRs						Forward primers (5'-3')	Annealing tempearture(0C)	Reverse primers (5'-3')	Annealing tempearture(0C)	Amplified product size (bp)	Structural annotation		Functional Annotation			
				C. microphyllum		ICC4958									Gene IDs	Sequence component of genes	NCBI nr IDs	TFs	KOG	KEGG
				Transcript	SSR repeat-motifs	Chromosomes	SSR repeat-motifs	Start physical positions (bp)	End physical positions (bp)											
TR38070|c1_g1_i2	No	(CT)8	16	--	--	--	--	--	--	CCTCGTTCACAAAGACAATCC	60	CCGTTGGATGATTGCATCT	59	277	--	--	--	--	--	--
TR38070|c1_g1_i5	No	(CT)8	16	--	--	--	--	--	--	CCTCGTTCACAAAGACAATCC	60	CCGTTGGATGATTGCATCT	59	277	--	--	--	--	--	--
TR38091|c0_g1_i1	XLOC_013277	(ACA)6	18	--	--	--	--	--	--	ACCACTTCTTCCCGTCTCCT	60	AATCGAGTTTCCAATCACGG	60	265	--	--	--	--	--	--
TR3810|c0_g1_i1	XLOC_013307	(ATA)6	18	--	--	--	--	--	--	TTCCCAATGTCTCATGCAAA	60	AAAATCCACCACCATGGAAA	60	183	--	--	--	--	--	--
TR38106|c2_g1_i1	XLOC_013303	(AAG)6	18	--	--	--	--	--	--	GCAACAAAAAGGAAGGTGGA	60	TGGAACTAAATTGGGAATTGATG	60	280	--	--	XP_004516934.1	ERF	[G]	--
TR38112|c5_g1_i1	XLOC_013315	(CAC)8	24	--	--	--	--	--	--	CAGGTTGACACCTTCTCCGT	60	AGCCTCTCCTGCAAAGACAA	60	262	--	--	--	--	--	--
TR38113|c0_g1_i2	No	(AGGT)5	20	--	--	--	--	--	--	TTCCCATCCAAACACTACTCG	60	AATGGGGAGAAAATGAAGGG	60	197	--	--	--	--	--	--
TR38120|c0_g1_i1	XLOC_013323	(T)10cttatatttcattag(A)10	35	--	--	--	--	--	--	CGTTTGGAAATCGACCAAGT	60	CTCCACCTCCCCATTCATAA	60	256	--	--	--	--	--	--
TR38120|c0_g1_i1	XLOC_013323	(GAT)6	18	--	--	--	--	--	--	CGTTTGGAAATCGACCAAGT	60	CTCCACCTCCCCATTCATAA	60	256	--	--	--	--	--	--
TR38128|c0_g1_i1	No	(GCT)5	15	--	--	--	--	--	--	GCTCGTCAATGCGTACAAGA	60	CAGAGCCCGTTTAATCGAAG	60	242	--	--	--	--	--	--
TR38128|c0_g1_i2	XLOC_013332	(GCT)5	15	--	--	--	--	--	--	TCAACTACCCCACTATCGCC	60	CAGAGCCCGTTTAATCGAAG	60	276	--	--	CCO30802.1	--	[J]	--
TR38128|c0_g1_i3	No	(GCT)5	15	--	--	--	--	--	--	GCTCGTCAATGCGTACAAGA	60	CAGAGCCCGTTTAATCGAAG	60	242	--	--	--	--	--	--
TR38138|c0_g1_i1	No	(CT)6	12	--	--	--	--	--	--	AAAACGCCTCAAAAGCTTCA	60	CCCCAGCCAAACTCATAAAA	60	275	--	--	--	--	--	--
TR38138|c0_g2_i1	No	(CT)6	12	--	--	--	--	--	--	AAAACGCCTCAAAAGCTTCA	60	ATACTTATCGACGGCGGAGA	60	162	--	--	--	--	--	--

Transcript IDs	Differentially expressed gene (DEG) IDs	SSR repeat-motifs	SSR repeat-length (bp)	*In silico* polymorphic SSRs						Forward primers (5'-3')	Annealing tempearture(0C)	Reverse primers (5'-3')	Annealing tempearture(0C)	Amplified product size (bp)	Structural annotation		Functional Annotation			
				C. microphyllum		ICC4958									Gene IDs	Sequence component of genes	NCBI nr IDs	TFs	KOG	KEGG
				Transcript	SSR repeat-motifs	Chromosomes	SSR repeat-motifs	Start physical positions (bp)	End physical positions (bp)											
TR38140|c0_g1_i1	No	(TTC)5	15	--	--	--	--	--	--	CGAGTTACCCGGAAGTTTGA	60	GAGCGTTCTTTTATGGCAGC	60	183	--	--	--	--	--	--
TR38140|c0_g2_i1	No	(TTC)5	15	--	--	--	--	--	--	CGAGTTACCCGGAAGTTTGA	60	GAGCGTTCTTTTATGGCAGC	60	183	--	--	--	--	--	--
TR38140|c0_g2_i2	No	(TTC)5	15	--	--	--	--	--	--	CGAGTTACCCGGAAGTTTGA	60	GAGCGTTCTTTTATGGCAGC	60	183	--	--	--	--	--	--
TR38140|c0_g2_i5	No	(TTC)5	15	--	--	--	--	--	--	CGAGTTACCCGGAAGTTTGA	60	GAGCGTTCTTTTATGGCAGC	60	183	--	--	--	--	--	--
TR38143|c0_g1_i1	No	(TA)7	14	--	--	--	--	--	--	TGCATTTATTTCGGATCCTTC	59	GAACACATAGGAATTGGGGG	59	241	--	--	--	--	--	--
TR38143|c0_g1_i2	No	(TA)7	14	--	--	--	--	--	--	TGCATTTATTTCGGATCCTTC	59	GCACCCATTGTGAAGTAGCA	60	264	--	--	--	--	--	--
TR38143|c0_g1_i3	No	(TC)11	22	--	--	--	--	--	--	ACAGACTCCAAACCCAAACG	60	GCACCCATTGTGAAGTAGCA	60	222	--	--	--	--	--	--
TR38143|c0_g1_i4	No	(TC)11	22	--	--	--	--	--	--	ACAGACTCCAAACCCAAACG	60	GCACCCATTGTGAAGTAGCA	60	222	--	--	--	--	--	--
TR38143|c0_g1_i5	No	(TA)7	14	--	--	--	--	--	--	TGCATTTATTTCGGATCCTTC	59	GCACCCATTGTGAAGTAGCA	60	264	--	--	--	--	--	--
TR38144|c0_g1_i1	No	(GAA)5	15	--	--	--	--	--	--	TACCCTCCAAATGGCTTCTG	60	TACGCACTGTAGCTTCTGCC	59	268	--	--	--	--	--	--
TR38148|c0_g1_i1	No	(CTT)6	18	--	--	--	--	--	--	ACGTGATCCTGGTCTTCCAC	60	AACCGGCATTTCTGTGAATC	60	248	--	--	--	--	--	--
TR38148|c0_g2_i1	XLOC_013342	(CTT)6	18	--	--	--	--	--	--	ACGTGATCCTGGTCTTCCAC	60	AACCGGCATTTCTGTGAATC	60	248	--	--	--	--	--	--
TR38150|c0_g1_i1	No	(TC)6	12	--	--	--	--	--	--	AAGCAGGAGGCGTTCACTAA	60	GTCGTTGTAGAGAGCGAGGG	60	224	--	--	--	--	--	--
TR38150|c0_g1_i2	No	(TC)6	12	--	--	--	--	--	--	AAGCAGGAGGCGTTCACTAA	60	GTCGTTGTAGAGAGCGAGGG	60	224	--	--	--	--	--	--

Transcript IDs	Differentially expressed gene (DEG) IDs	SSR repeat-motifs	SSR repeat-length (bp)	*In silico* polymorphic SSRs						Forward primers (5'-3')	Annealing tempearture(0C)	Reverse primers (5'-3')	Annealing tempearture(0C)	Amplified product size (bp)	Structural annotation		Functional Annotation			
				C. microphyllum		ICC4958									Gene IDs	Sequence component of genes	NCBI nr IDs	TFs	KOG	KEGG
				Transcript	SSR repeat-motifs	Chromosomes	SSR repeat-motifs	Start physical positions (bp)	End physical positions (bp)											
TR38151|c0_g1_i1	XLOC_013344	(ATA)5	15	--	--	--	--	--	--	GCAGGATCCCCTTCTACCAT	60	AACAGGCGATGATGATCTTTTT	60	201	--	--	XM_004500873.1	--	--	--
TR38156|c0_g1_i1	XLOC_013352	(CT)7	14	--	--	--	--	--	--	ATCAGCACGAACAAACAACG	60	CTGCCGTACTCGCTCTTGAT	61	274	--	--	--	--	--	--
TR38156|c0_g1_i2	No	(CT)7	14	--	--	--	--	--	--	ATCAGCACGAACAAACAACG	60	CTGCCGTACTCGCTCTTGAT	61	274	--	--	--	--	--	--
TR38162|c0_g2_i1	XLOC_013360	(AAG)5	15	--	--	--	--	--	--	TAGATGCCATCCACAAGCAG	60	GGCGAGTCGTTCATTTAGGA	60	214	--	--	--	--	--	--
TR38163|c0_g1_i2	No	(TAA)5	15	--	--	--	--	--	--	ACACTTCGCGGTTCAAATTC	60	AGCATGCTGAAGTTGCTTCC	61	181	--	--	--	--	--	--
TR38163|c0_g1_i3	No	(TAA)5	15	--	--	--	--	--	--	ACACTTCGCGGTTCAAATTC	60	AGCATGCTGAAGTTGCTTCC	61	181	--	--	--	--	--	--
TR38169|c0_g1_i1	No	(TTG)5	15	--	--	--	--	--	--	AGGCAGAGAGACATGAACCC	59	TGTTGGGTGTTGAAGATGGA	60	242	--	--	--	--	--	--
TR38169|c0_g1_i1	No	(GAA)6	18	--	--	--	--	--	--	CCCACTTCAATTTTTGCTCC	60	TCTTCAGAAACTTCCAATACCTCA	59	262	--	--	--	--	--	--
TR38169|c0_g1_i2	No	(TTG)5	15	--	--	--	--	--	--	AGGCAGAGAGACATGAACCC	59	TCAAAAACAGGTGAAGGCTTG	60	257	--	--	--	--	--	--
TR38169|c0_g1_i2	No	(GAA)6	18	--	--	--	--	--	--	TCCAAAGAGGAAGCTTTAGCA	59	TCAGCTCCAACATTCACATCA	60	278	--	--	--	--	--	--
TR38169|c1_g1_i1	No	(TCT)8	24	--	--	--	--	--	--	TGTAACCGTATTTCTCCCCG	60	CCCTGGGTTCAATTTCAAGA	60	222	--	--	--	--	--	--
TR38169|c1_g1_i2	No	(TCT)8	24	--	--	--	--	--	--	TGTAACCGTATTTCTCCCCG	60	CCCTGGGTTCAATTTCAAGA	60	222	--	--	--	--	--	--
TR38169|c1_g1_i3	No	(TCT)8	24	--	--	--	--	--	--	TGTAACCGTATTTCTCCCCG	60	CCCTGGGTTCAATTTCAAGA	60	222	--	--	--	--	--	--
TR38169|c1_g1_i4	No	(TCT)8	24	--	--	--	--	--	--	TGTAACCGTATTTCTCCCCG	60	CCCTGGGTTCAATTTCAAGA	60	222	--	--	--	--	--	--

Transcript IDs	Differentially expressed gene (DEG) IDs	SSR repeat-motifs	SSR repeat-length (bp)	*In silico* polymorphic SSRs						Forward primers (5'-3')	Annealing tempearture(0C)	Reverse primers (5'-3')	Annealing tempearture(0C)	Amplified product size (bp)	Structural annotation		Functional Annotation			
				C. microphyllum		ICC4958									Gene IDs	Sequence component of genes	NCBI nr IDs	TFs	KOG	KEGG
				Transcript	SSR repeat-motifs	Chromosomes	SSR repeat-motifs	Start physical positions (bp)	End physical positions (bp)											
TR38191|c0_g1_i1	XLOC_013384	(CAT)5	15	TR38191|c0_g1_i1	(CAT)5	Ca_LG_2	(CAT)6	13242821	13242838	CCTCTCTATACCTCTCAATCCCA	59	TGAAGGTGAAAAGGGTTTGG	60	272		URR	--	--	--	--
TR38197|c0_g1_i1	XLOC_013389	(CT)7	14	--	--	--	--	--	--	AGCAAACCAACACACTTCCC	60	GGTCGGCTTGTACGAATGTT	60	233	--	--	--	--	--	--
TR38197|c0_g1_i1	XLOC_013389	(ACA)7ta(ATG)5	38	--	--	--	--	--	--	CCAACCGAAACAATAATGGG	60	ATGGAGAAACAGGTGAAGCC	59	202	--	--	--	--	--	--
TR38246|c0_g1_i1	No	(ATG)6	18	--	--	--	--	--	--	TTTGTGGCCTTCAATGAACA	60	GCTCTTTTTGTCCTCGTGCT	60	116	--	--	--	--	--	--
TR38246|c0_g2_i1	No	(ATG)6	18	--	--	--	--	--	--	TTTGTGGCCTTCAATGAACA	60	GCTCTTTTTGTCCTCGTGCT	60	116	--	--	--	--	--	--
TR3835|c3_g1_i1	No	(AT)6	12	--	--	--	--	--	--	TGGACTTTGAGCACCAACAT	59	TTTGATTTTTATGGAGCGCA	59	277	--	--	--	--	--	--
TR3835|c3_g1_i2	No	(AT)6	12	--	--	--	--	--	--	TGGACTTTGAGCACCAACAT	59	TTTGATTTTTATGGAGCGCA	59	277	--	--	--	--	--	--
TR3835|c3_g1_i3	No	(AT)6	12	--	--	--	--	--	--	TGGACTTTGAGCACCAACAT	59	TTTGATTTTTATGGAGCGCA	59	277	--	--	--	--	--	--
TR3837|c0_g1_i1	XLOC_013485	(GAAC)5	20	--	--	--	--	--	--	CCAAAACAACACAGCGTCAG	60	GAAAAACAAAAACGACGGGA	60	246	--	--	--	--	--	--
TR3837|c0_g1_i1	XLOC_013485	(TC)8(TA)8	32	--	--	--	--	--	--	TCCCGTCGTTTTTGTTTTTC	60	TCTGGAACGACGAGGACTCT	60	254	--	--	--	--	--	--
TR3837|c3_g1_i1	No	(AT)14	28	--	--	--	--	--	--	CAGAAGCTCCAAGAACTGCC	60	TGGAATGTGGAAAGCACAAA	60	125	--	--	--	--	--	--
TR3837|c3_g2_i1	No	(AT)12	24	--	--	--	--	--	--	CAGAAGCTCCAAGAACTGCC	60	TGGAATGTGGAAAGCACAAA	60	121	--	--	--	--	--	--
TR38406|c0_g1_i1	No	(T)10aatgcctataattaatactaactttctcgattagctggacttgtcttgtttctttattacatttcataaagc(T)16	98	--	--	--	--	--	--	GAGAAGGGAAGGTTTGGTAGAAA	60	GGATCCAATGTATACCCCCA	59	244	--	--	--	--	--	--
TR38406|c0_g2_i1	No	(T)10aatgcctataattaatactaactttctcgattagctggacttgtcttgtttctttattacatttcataaagc(T)16	98	--	--	--	--	--	--	GAGAAGGGAAGGTTTGGTAGAAA	60	GGATCCAATGTATACCCCCA	59	244	--	--	--	--	--	--

Transcript IDs	Differentially expressed gene (DEG) IDs	SSR repeat-motifs	SSR repeat-length (bp)	*In silico* polymorphic SSRs						Forward primers (5'-3')	Annealing tempearture(0C)	Reverse primers (5'-3')	Annealing tempearture(0C)	Amplified product size (bp)	Structural annotation		Functional Annotation			
				C. microphyllum		ICC4958									Gene IDs	Sequence component of genes	NCBI nr IDs	TFs	KOG	KEGG
				Transcript	SSR repeat-motifs	Chromosomes	SSR repeat-motifs	Start physical positions (bp)	End physical positions (bp)											
TR3841|c1_g1_i1	XLOC_013515	(TC)9	18	--	--	--	--	--	--	TCCATTTGGTCTCTTTAGGAGTG	60	TCCAGCCTTAACCATTCCAG	60	210	--	--	XP_002530852.1	Dof	[Z]	--
TR3845|c3_g1_i1	No	(AG)7	14	--	--	--	--	--	--	TTACCTTGGAGTGCAGCAGA	60	ATATTCGAGGCAAGGCTTCA	60	176	--	--	--	--	--	--
TR3845|c3_g1_i2	No	(A)10ggaatactcatcgccttactacaaatttcatgaaacaaagatcaacaacaattgaccaatatcacactcctttaacc(A)13	100	--	--	--	--	--	--	CATGACAATTCATTGCACCA	59	CGAAGCGGTCTTCCTTACTG	60	231	--	--	--	--	--	--
TR3845|c3_g2_i1	No	(A)10ggaatactcatcgccttactacaaatttcatgaaacaaagatcaacaacaattgaccaatatcacactcctttaacc(A)13	100	--	--	--	--	--	--	CATGACAATTCATTGCACCA	59	CGAAGCGGTCTTCCTTACTG	60	231	--	--	--	--	--	--
TR3845|c3_g2_i2	No	(A)10ggaatactcatcgccttactacaaatttcatgaaacaaagatcaacaacaattgaccaatatcacactcctttaacc(A)13	100	--	--	--	--	--	--	CATGACAATTCATTGCACCA	59	CGAAGCGGTCTTCCTTACTG	60	231	--	--	--	--	--	--
TR3845|c3_g2_i3	No	(A)10ggaatactcatcgccttactacaaatttcatgaaacaaagatcaacaacaattgaccaatatcacactcctttaacc(A)13	100	--	--	--	--	--	--	CATGACAATTCATTGCACCA	59	CGAAGCGGTCTTCCTTACTG	60	231	--	--	--	--	--	--
TR3845|c3_g2_i4	No	(A)10ggaatactcatcgccttactacaaatttcatgaaacaaagatcaacaacaattgaccaatatcacactcctttaacc(A)13	100	--	--	--	--	--	--	CATGACAATTCATTGCACCA	59	CGAAGCGGTCTTCCTTACTG	60	231	--	--	--	--	--	--
TR3845|c3_g2_i5	No	(A)10ggaatactcatcgccttactacaaatttcatgaaacaaagatcaacaacaattgaccaatatcacactcctttaacc(A)13	100	--	--	--	--	--	--	CATGACAATTCATTGCACCA	59	CGAAGCGGTCTTCCTTACTG	60	231	--	--	--	--	--	--
TR3845|c3_g2_i6	No	(A)10ggaatactcatcgccttactacaaatttcatgaaacaaagatcaacaacaattgaccaatatcacactcctttaacc(A)13	100	--	--	--	--	--	--	CATGACAATTCATTGCACCA	59	CGAAGCGGTCTTCCTTACTG	60	231	--	--	--	--	--	--
TR3845|c3_g4_i1	No	(A)10ggaatactcatcgccttactacaaatttcatgaaacaaagatcaacaacaattgaccaatatcacactcctttaacc(A)13	100	--	--	--	--	--	--	CATGACAATTCATTGCACCA	59	CGAAGCGGTCTTCCTTACTG	60	231	--	--	--	--	--	--
TR38450|c0_g2_i1	XLOC_013534	(CT)8	16	--	--	--	--	--	--	TTTCTCCTATCGCTCTCCCC	61	ACCTCCAATCCAATGCCATA	60	269	--	--	--	--	--	--
TR38462|c0_g1_i2	No	(GAA)5	15	--	--	--	--	--	--	CAGTGAGCGAACTGAACAACA	60	GTTCACATTCGCGTTTTCCT	60	173	--	--	--	--	--	--
TR38468|c0_g2_i1	XLOC_013559	(ATT)5	15	--	--	--	--	--	--	ACTCTCACTGGGTTTGGTGG	60	AGAGAGGGAGGGTGTGGTCT	60	193	--	--	--	--	--	--
TR3847|c1_g1_i1	No	(T)10atctttccttttcggttaa(TC)6	41	--	--	--	--	--	--	AATTCCACAAAACCACTCGC	60	GAATTGGGAACGAAACGATG	60	197	--	--	--	--	--	--

Transcript IDs	Differentially expressed gene (DEG) IDs	SSR repeat-motifs	SSR repeat-length (bp)	*In silico* polymorphic SSRs						Forward primers (5'-3')	Annealing tempearture(0C)	Reverse primers (5'-3')	Annealing tempearture(0C)	Amplified product size (bp)	Structural annotation		Functional Annotation			
				C. microphyllum		ICC4958									Gene IDs	Sequence component of genes	NCBI nr IDs	TFs	KOG	KEGG
				Transcript	SSR repeat-motifs	Chromosomes	SSR repeat-motifs	Start physical positions (bp)	End physical positions (bp)											
TR3847|c1_g1_i3	No	(T)10atctttccttttcggttaa(TC)6	41	--	--	--	--	--	--	AATTCCACAAAACCACTCGC	60	GAATTGGGAACGAAACGATG	60	197	--	--	--	--	--	--
TR3847|c1_g1_i4	No	(T)10atctttccttttcggttaa(TC)6	41	--	--	--	--	--	--	AATTCCACAAAACCACTCGC	60	GAATTGGGAACGAAACGATG	60	197	--	--	--	--	--	--
TR3847|c1_g1_i6	No	(T)10atctttccttttcggttaa(TC)6	41	--	--	--	--	--	--	AATTCCACAAAACCACTCGC	60	GAATTGGGAACGAAACGATG	60	197	--	--	--	--	--	--
TR3847|c1_g3_i1	No	(T)10atctttccttttcggttaa(TC)6	41	--	--	--	--	--	--	AATTCCACAAAACCACTCGC	60	GAATTGGGAACGAAACGATG	60	197	--	--	--	--	--	--
TR38488|c1_g1_i1	No	(AT)6	12	--	--	--	--	--	--	TGCTTATGCAGAGAAATATGAAAG	58	GATGAAGCTTTTGCAGCCAT	60	158	--	--	--	--	--	--
TR38497|c0_g1_i1	No	(ATG)5	15	--	--	--	--	--	--	GCATCCACACATGCCATAAG	60	GTAAGCCAAACCACCCAAAA	60	143	--	--	--	--	--	--
TR38497|c0_g2_i1	No	(ATG)5	15	--	--	--	--	--	--	GCATCCACACATGCCATAAG	60	GTAAGCCAAACCACCCAAAA	60	143	--	--	--	--	--	--
TR38511|c0_g1_i1	No	(CAT)5cagcttacccgaaccctttctctctcctccgtcgat cacgattacgtcattcctctactcttctttct(ATTCA)5	108	--	--	--	--	--	--	GGAAAGCAGCAGAAGATTCG	60	GGGTCTGTTGAGAAGGGACA	60	278	--	--	--	--	--	--
TR38511|c0_g1_i2	No	(CAT)5	15	--	--	--	--	--	--	TAGAGAGTGGAGCGGAGGAA	60	GTGATCGACGGAGGAGAGAG	60	165	--	--	--	--	--	--
TR38511|c0_g1_i3	No	(CAT)5cagcttacccgaaccctttctctctcctccgtcgat cacgattacgtcattcctctactcttctttct(ATTCA)5	108	--	--	--	--	--	--	GGAAAGCAGCAGAAGATTCG	60	GGGTCTGTTGAGAAGGGACA	60	278	--	--	--	--	--	--
TR38511|c0_g1_i4	No	(CAT)5	15	--	--	--	--	--	--	TAGAGAGTGGAGCGGAGGAA	60	GTGATCGACGGAGGAGAGAG	60	165	--	--	--	--	--	--
TR38518|c0_g1_i1	XLOC_013617	(TGT)5	15	--	--	--	--	--	--	TGTCGCTCTTCTCGACCTTT	60	ATTTCTGCAACCTTTTGCGT	60	218	--	--	XP_003024736.1	--	[Z]	K08341
TR38519|c0_g1_i1	No	(TA)8	16	--	--	--	--	--	--	TTCACCCACCACTCAACAAA	60	AGCTGCATAGTGCAACAACAG	59	182	--	--	--	--	--	--
TR38519|c0_g1_i2	XLOC_013619	(TA)8gagccaaa(AT)9	42	--	--	--	--	--	--	ACCACTCATCACCCACCACT	60	ACGGAGGATTTGGATTCCTT	60	266	--	--	--	--	--	--

Transcript IDs	Differentially expressed gene (DEG) IDs	SSR repeat-motifs	SSR repeat-length (bp)	*In silico* polymorphic SSRs						Forward primers (5'-3')	Annealing tempearture(0C)	Reverse primers (5'-3')	Annealing tempearture(0C)	Amplified product size (bp)	Structural annotation		Functional Annotation			
				C. microphyllum		ICC4958									Gene IDs	Sequence component of genes	NCBI nr IDs	TFs	KOG	KEGG
				Transcript	SSR repeat-motifs	Chromosomes	SSR repeat-motifs	Start physical positions (bp)	End physical positions (bp)											
TR3852|c0_g1_i1	No	(AT)9	18	--	--	--	--	--	--	TCAAATTGTTTTTAGAGTTGCTGC	60	TGAATTCCAGCCTCTCTCCT	59	109	--	--	--	--	--	--
TR3852|c0_g2_i1	No	(AT)9	18	--	--	--	--	--	--	TCAAATTGTTTTTAGAGTTGCTGC	60	TGAATTCCAGCCTCTCTCCT	59	109	--	--	--	--	--	--
TR3852|c3_g1_i1	No	(AG)9	18	TR3852|c3_g1_i1	(AG)9	Ca_LG_6	(AG)7	14801951	14801964	GAGAAAGGGGCAGACAAATG	60	TCAGAGTGTTAACCCCGTCC	60	163	Ca_10652	URR	--	--	--	--
TR3852|c3_g1_i1	No	(AG)8	16	TR3852|c3_g1_i1	(AG)9	Ca_LG_6	(AG)7	14801951	14801964	GGACGGGGTTAACACTCTGA	60	GCCGATTTCCTCTCCTCTCT	60	105	Ca_10652	URR	--	--	--	--
TR38531|c0_g1_i1	XLOC_013635	(A)12gaacaattcattattgtgtttcaacaattcaaaatttctat(A)15	68	--	--	--	--	--	--	CAAATTTATAATTCTCTGCAATCAAAA	59	ATTGCAATGGAAGAGGAACA	58	228	--	--	--	--	--	--
TR38537|c0_g1_i1	No	(TGC)5tccaggtaat(AAC)6	43	--	--	--	--	--	--	CCATCACCACTCCTCCAACT	60	CATGCACATGTGGTGGTACA	60	176	--	--	--	--	--	--
TR38537|c0_g1_i2	No	(CTG)5	15	--	--	--	--	--	--	CCATCACCACTCCTCCAACT	60	CATGCACATGTGGTGGTACA	60	158	--	--	--	--	--	--
TR38537|c0_g1_i4	No	(CTG)5	15	--	--	--	--	--	--	CCATCACCACTCCTCCAACT	60	GCACATGTGGTGGTACAACTCT	60	155	--	--	--	--	--	--
TR38584|c0_g1_i1	XLOC_013669	(AAT)6	18	--	--	--	--	--	--	GCGACATTTCAAAACCAAAAA	60	CGTTCATGCTTCTCCCTCTC	60	139	--	--	XP_004495887.1	B3	[QR]	K04125
TR38673|c0_g1_i1	No	(CTC)5	15	--	--	--	--	--	--	TTCGCTCTGATCCTCCTCAT	60	TAAAACCCTCTCGTTCCCCT	60	218	--	--	--	--	--	--
TR38675|c0_g1_i1	XLOC_013712	(TAC)5	15	--	--	--	--	--	--	GAATTCATCAACTCCATAACTCCA	59	TGCAGCTTTTCCTTTTCGAT	60	152	--	--	--	--	--	--
TR3870|c4_g1_i2	XLOC_013733	(CTT)5	15	--	--	--	--	--	--	CGCCACTCACTTCCACACT	60	TTGTGTGGTAACGGAACGAA	60	273	--	--	--	--	--	--
TR38708|c0_g1_i1	XLOC_013728	(CCT)5	15	--	--	--	--	--	--	GTTCGTTCAACGGCTGCTAT	60	CAGACCGTATGAAGCACGAA	60	245	--	--	EMT69059.1	--	--	--
TR38720|c0_g1_i1	XLOC_013742	(AAC)5	15	--	--	--	--	--	--	GTGCGAGCGTTTCTTTCTTC	60	ATCGTGAAATCCTCACCGTC	60	272	--	--	--	--	--	--

Transcript IDs	Differentially expressed gene (DEG) IDs	SSR repeat-motifs	SSR repeat-length (bp)	*In silico* polymorphic SSRs						Forward primers (5'-3')	Annealing tempearture(0C)	Reverse primers (5'-3')	Annealing tempearture(0C)	Amplified product size (bp)	Structural annotation		Functional Annotation			
				C. microphyllum		ICC4958									Gene IDs	Sequence component of genes	NCBI nr IDs	TFs	KOG	KEGG
				Transcript	SSR repeat-motifs	Chromosomes	SSR repeat-motifs	Start physical positions (bp)	End physical positions (bp)											
TR38726|c1_g1_i1	No	(TAA)5	15	--	--	--	--	--	--	AAGCCTTCTAACCAAAGCCC	60	TTAAGTGTTCCAAAAACATATCTGA	57	214	--	--	--	--	--	--
TR3873|c9_g1_i3	No	(ATT)5ataccgtttgctcctttttactcttcacgtttttctctctttctttggattcagttctcttgttaggtaacgtagctttggttttctttt(TTA)6	124	--	--	--	--	--	--	ATCGCCGATTTAATCACGTC	60	AAGACTTCACCTGCACCACC	60	210	--	--	--	--	--	--
TR3873|c9_g1_i4	No	(ATT)5ataccgtttgctcctttttactcttcacgtttttctctctttctttggattcagttctcttgttaggtaacgtagctttggttttctttt(TTA)6	124	--	--	--	--	--	--	ATCGCCGATTTAATCACGTC	60	AAGACTTCACCTGCACCACC	60	210	--	--	--	--	--	--
TR3873|c9_g1_i5	No	(ATT)5ataccgtttgctcctttttactcttcacgtttttctctctttctttggattcagttctcttgttaggtaacgtagctttggttttctttt(TTA)6	124	--	--	--	--	--	--	ATCGCCGATTTAATCACGTC	60	AAGACTTCACCTGCACCACC	60	210	--	--	--	--	--	--
TR38730|c0_g1_i2	No	(GA)7	14	--	--	--	--	--	--	TCAGAGTCGTGTAAGCGTGC	60	CTTCCGCCCACCTTCTCTAC	61	107	--	--	--	--	--	--
TR38733|c0_g1_i1	XLOC_013758	(AGA)6	18	--	--	--	--	--	--	CGATGTGAAGGGACATGTTG	60	ACTTGAAACCGCCATACGTC	60	263	--	--	--	--	--	--
TR38748|c0_g2_i1	XLOC_013779	(ATT)7	21	--	--	--	--	--	--	ATCCCAAAACCCTTTCCAAC	60	AGCCGTAGAAGCTGCAAGAG	60	241	--	--	NP_001296583.1	ERF	[K]	--
TR38770|c1_g1_i2	XLOC_013806	(TCT)5	15	--	--	--	--	--	--	TCATCTTCATCCCACGATCA	60	GTCCAGTTAGTCAGGAGGCG	60	150	--	--	--	--	--	--
TR38770|c1_g2_i1	No	(TCT)5	15	--	--	--	--	--	--	TCATCTTCATCCCACGATCA	60	GTCCAGTTAGTCAGGAGGCG	60	150	--	--	--	--	--	--
TR38777|c0_g1_i1	XLOC_013815	(CGA)5	15	--	--	--	--	--	--	CCGTACTCCGACTTCGTCTC	60	GTCATCAACGACAAGGAGCA	60	192	--	--	--	--	--	--
TR38777|c0_g1_i2	XLOC_013816	(CGA)5	15	--	--	--	--	--	--	CCGTACTCCGACTTCGTCTC	60	GTCATCAACGACAAGGAGCA	60	192	--	--	XP_008904756.1	--	[O]	--
TR38788|c0_g1_i1	No	(CT)8	16	--	--	--	--	--	--	GCTTAAGCTGCGAAATCACC	60	CACAGAACAAGAGAGAGAAAGAAAGA	60	206	--	--	--	--	--	--
TR38790|c0_g1_i1	XLOC_013838	(AAT)7	21	--	--	--	--	--	--	GCGTCCACTCACAACACAAT	60	CCTTTTCTCTGGTGGGTCAA	60	229	--	--	--	--	--	--
TR38797|c0_g1_i1	XLOC_013843	(TTGAA)5	25	--	--	--	--	--	--	CATAGCAAATGAGTGAGGGACA	60	ATAAGGACCACCCACATGGA	60	133	--	--	--	--	--	--

Transcript IDs	Differentially expressed gene (DEG) IDs	SSR repeat-motifs	SSR repeat-length (bp)	*In silico* polymorphic SSRs						Forward primers (5'-3')	Annealing tempearture(0C)	Reverse primers (5'-3')	Annealing tempearture(0C)	Amplified product size (bp)	Structural annotation		Functional Annotation			
				C. microphyllum		ICC4958									Gene IDs	Sequence component of genes	NCBI nr IDs	TFs	KOG	KEGG
				Transcript	SSR repeat-motifs	Chromosomes	SSR repeat-motifs	Start physical positions (bp)	End physical positions (bp)											
TR38798|c2_g1_i1	No	(TTC)11	33	--	--	--	--	--	--	CCCAAAAATTCTCCCCTGTT	60	CTGTTGCGCCTTTTAACACA	60	274	--	--	--	--	--	--
TR38798|c2_g3_i1	No	(TTC)10	30	--	--	--	--	--	--	CCCAAAAATTCTCCCCTGTT	60	CTGTTGCGCCTTTTAACACA	60	271	--	--	--	--	--	--
TR3881|c2_g1_i1	No	(A)13gattttttgtgtgtaaaactctactcaaactaaaaaatt gtaatcgagcaaac(A)13	79	--	--	--	--	--	--	TGTACAAGCAACGGGCATTA	60	GCAACCAGCAGCAGTTGATA	60	261	--	--	--	--	--	--
TR3881|c2_g1_i2	No	(A)13gattttttgtgtgtaaaactctactcaaactaaaaaatt gtaatcgagcaaac(A)13	79	--	--	--	--	--	--	TGTACAAGCAACGGGCATTA	60	GCAACCAGCAGCAGTTGATA	60	261	--	--	--	--	--	--
TR38811|c0_g1_i1	No	(GT)6	12	--	--	--	--	--	--	TGGAGCAGAGAAATGAAGTCC	59	CGCCAAACAAAGTAGTAACGC	60	140	--	--	--	--	--	--
TR38813|c1_g1_i1	XLOC_013856	(AAT)7	21	--	--	--	--	--	--	GCTTCATGTTGAAGGAAATGTATG	60	GCCAAACATGACCAAATTCA	59	141	--	--	--	--	--	--
TR38813|c1_g2_i1	XLOC_013857	(AAT)7	21	--	--	--	--	--	--	GCTTCATGTTGAAGGAAATGTATG	60	TGGACCTTTTTAGCAAACCG	60	194	--	--	--	--	--	--
TR3882|c0_g1_i1	No	(TGA)5	15	--	--	--	--	--	--	AAGAAGAAGGCTCCGGTCAT	60	CGATTTCCCCTTTTTCTTCC	60	219	--	--	--	--	--	--
TR3882|c0_g1_i1	No	(TTC)6	18	--	--	--	--	--	--	CAACCCTAGATTCTTTTGTTGTG	58	AAAGGCGAGATTCGTGTTTG	60	197	--	--	--	--	--	--
TR3882|c0_g1_i5	No	(TGA)5	15	--	--	--	--	--	--	AAGAAGAAGGCTCCGGTCAT	60	CGATTTCCCCTTTTTCTTCC	60	219	--	--	--	--	--	--
TR3882|c0_g1_i5	No	(TTC)6	18	--	--	--	--	--	--	CAACCCTAGATTCTTTTGTTGTG	58	AAAGGCGAGATTCGTGTTTG	60	197	--	--	--	--	--	--
TR3883|c6_g1_i1	XLOC_013880	(TCA)7	21	--	--	--	--	--	--	CTTAATTCTCCCACCGACCA	60	GAGATGGGGGAGGTATGGAT	60	133	--	--	--	--	--	--
TR3886|c8_g1_i1	No	(CTT)5	15	--	--	--	--	--	--	TGCTAATTGCCCTGAAAACTG	60	CGAGGCTGAGTCAAACATGA	60	279	--	--	--	--	--	--
TR38907|c0_g1_i1	No	(GTG)5	15	--	--	--	--	--	--	CCATGAGGGGATAAAAGGGT	60	TGGTTTGGTTCCATTTCCAT	60	243	--	--	--	--	--	--

Transcript IDs	Differentially expressed gene (DEG) IDs	SSR repeat-motifs	SSR repeat-length (bp)	*In silico* polymorphic SSRs						Forward primers (5'-3')	Annealing tempearture(0C)	Reverse primers (5'-3')	Annealing tempearture(0C)	Amplified product size (bp)	Structural annotation		Functional Annotation			
				C. microphyllum		**ICC4958**									**Gene IDs**	**Sequence component of genes**	**NCBI nr IDs**	**TFs**	**KOG**	**KEGG**
				**Transcript**	**SSR repeat-motifs**	**Chromosomes**	**SSR repeat-motifs**	**Start physical positions (bp)**	**End physical positions (bp)**											
TR38916|c0_g1_i1	XLOC_013947	(TC)7	14	--	--	--	--	--	--	TGAATTGGGTCGGTGAATTT	60	CCCAATCCATTGAACCTTGT	60	250	--	--	--	--	--	--
TR38925|c0_g1_i2	No	(GA)10	20	--	--	--	--	--	--	AACGAACCCTAACACGACCA	60	GAGCATGAGCATGTTTTCCA	60	207	--	--	--	--	--	--
TR38937|c0_g1_i1	XLOC_013966	(AGC)5	15	--	--	--	--	--	--	GTGTGGAGTCCTCTGGCATT	60	CGATCTGAGGTGGAGCTAGG	60	265	--	--	--	--	--	--
TR38946|c0_g1_i1	No	(GA)8	16	--	--	--	--	--	--	CTTGTGTTCGTGTTGGGTTG	60	ATACCGTGATCTGCCACCAC	61	114	--	--	--	--	--	--
TR3897|c1_g1_i1	No	(TTC)5	15	--	--	--	--	--	--	AGGGATTGGGAGCTAACCTG	60	CCACAACATTTCAGGCAGTG	60	182	--	--	--	--	--	--
TR38971|c1_g1_i1	XLOC_013999	(ATT)5	15	--	--	--	--	--	--	TCATGCGTCCAATCATCATC	60	GGAGCTTCACTGCCAAACAT	60	110	--	--	--	--	--	--
TR38990|c0_g1_i1	XLOC_014022	(A)12(TA)7	26	--	--	--	--	--	--	AGTACAATCAGATGCGCGTG	60	CGTTTGACAGAGCTTGAGCA	60	189	--	--	--	--	--	--
TR39005|c0_g1_i2	No	(TC)7	14	--	--	--	--	--	--	CATCGTTGATTCAAACACGC	60	ATCACGAAACAAAACACCGA	59	257	--	--	--	--	--	--
TR39005|c1_g1_i1	No	(AAC)6agtacaagggtaatccttaaccaaaccaaatcc gaacatagaagggacttttcatca(TCT)5	90	--	--	--	--	--	--	TCTCTCTCCTTCGAACAACCTT	60	CAGAAAATGGAGGGCATGTT	60	212	--	--	--	--	--	--
TR39005|c1_g1_i4	No	(AAC)6agtacaagggtaatccttaaccaaaccaaatcc gaacatagaagggacttttcatca(TCT)5	90	--	--	--	--	--	--	TCTCTCTCCTTCGAACAACCTT	60	CAGAAAATGGAGGGCATGTT	60	212	--	--	--	--	--	--
TR3901|c0_g1_i1	No	(CT)8	16	--	--	--	--	--	--	ACCAAACAGTTTTGGGCG	60	TGTGTTTGGGTCTCGTTTCA	60	123	--	--	--	--	--	--
TR3901|c0_g1_i10	No	(CT)8	16	--	--	--	--	--	--	ACCAAACAGTTTTGGGCG	60	TGTGTTTGGGTCTCGTTTCA	60	123	--	--	--	--	--	--
TR3901|c0_g1_i11	No	(CT)8	16	--	--	--	--	--	--	ACCAAACAGTTTTGGGCG	60	TGTGTTTGGGTCTCGTTTCA	60	123	--	--	--	--	--	--
TR3901|c0_g1_i12	No	(CT)8	16	--	--	--	--	--	--	ACCAAACAGTTTTGGGCG	60	TGTGTTTGGGTCTCGTTTCA	60	123	--	--	--	--	--	--

Transcript IDs	Differentially expressed gene (DEG) IDs	SSR repeat-motifs	SSR repeat-length (bp)	*In silico* polymorphic SSRs						Forward primers (5'-3')	Annealing tempearture(0C)	Reverse primers (5'-3')	Annealing tempearture(0C)	Amplified product size (bp)	Structural annotation		Functional Annotation			
				C. microphyllum		ICC4958									Gene IDs	Sequence component of genes	NCBI nr IDs	TFs	KOG	KEGG
				Transcript	SSR repeat-motifs	Chromosomes	SSR repeat-motifs	Start physical positions (bp)	End physical positions (bp)											
TR3901|c0_g1_i15	No	(CT)8	16	--	--	--	--	--	--	ACCAAACAGTTTTGGGCG	60	TGTGTTTGGGTCTCGTTTCA	60	123	--	--	--	--	--	--
TR3901|c0_g1_i2	No	(CT)8	16	--	--	--	--	--	--	TCTCAATAGAACCGACCAAACA	60	GAACAGTGATCCTTCGGCAT	60	157	--	--	--	--	--	--
TR3901|c0_g1_i3	No	(CT)8	16	--	--	--	--	--	--	ACCAAACAGTTTTGGGCG	60	TGTGTTTGGGTCTCGTTTCA	60	123	--	--	--	--	--	--
TR3901|c0_g1_i4	No	(CT)8	16	--	--	--	--	--	--	TCTCAATAGAACCGACCAAACA	60	GAACAGTGATCCTTCGGCAT	60	157	--	--	--	--	--	--
TR3901|c0_g1_i5	No	(CT)8	16	--	--	--	--	--	--	ACCAAACAGTTTTGGGCG	60	TGTGTTTGGGTCTCGTTTCA	60	123	--	--	--	--	--	--
TR3901|c0_g1_i6	No	(CT)8	16	--	--	--	--	--	--	ACCAAACAGTTTTGGGCG	60	TGTGTTTGGGTCTCGTTTCA	60	123	--	--	--	--	--	--
TR3901|c0_g1_i7	No	(CT)8	16	--	--	--	--	--	--	ACCAAACAGTTTTGGGCG	60	TGTGTTTGGGTCTCGTTTCA	60	123	--	--	--	--	--	--
TR3901|c0_g1_i8	No	(CT)8	16	--	--	--	--	--	--	ACCAAACAGTTTTGGGCG	60	TGTGTTTGGGTCTCGTTTCA	60	123	--	--	--	--	--	--
TR3901|c0_g1_i9	No	(CT)8	16	--	--	--	--	--	--	TCTCAATAGAACCGACCAAACA	60	GAACAGTGATCCTTCGGCAT	60	157	--	--	--	--	--	--
TR39013|c0_g1_i3	No	(T)10ggcgttaaagaaacgcacagaggaggagtaaaag(A)10	54	--	--	--	--	--	--	CCAATTTTTCAAATTTACTCCTTTG	59	ACGTCACTCTTCACCAACCC	60	217	--	--	--	--	--	--
TR39013|c0_g1_i4	No	(T)10ggcgttaaagaaacgcacagaggaggagtaaaag(A)10	54	--	--	--	--	--	--	CCAATTTTTCAAATTTACTCCTTTG	59	ACGTCACTCTTCACCAACCC	60	217	--	--	--	--	--	--
TR39013|c0_g1_i7	No	(T)10ggcgttaaagaaacgcacagaggaggagtaaaag(A)10	54	--	--	--	--	--	--	CCAATTTTTCAAATTTACTCCTTTG	59	ACGTCACTCTTCACCAACCC	60	217	--	--	--	--	--	--
TR39021|c1_g1_i1	No	(TC)10	20	--	--	--	--	--	--	GGAAGCCATTAGCGAAATGA	60	TCTCTGCACGTAGTGGATGC	60	151	--	--	--	--	--	--
TR39021|c1_g2_i1	No	(TC)10	20	--	--	--	--	--	--	GGAAGCCATTAGCGAAATGA	60	TCTCTGCACGTAGTGGATGC	60	151	--	--	--	--	--	--

Transcript IDs	Differentially expressed gene (DEG) IDs	SSR repeat-motifs	SSR repeat-length (bp)	*In silico* polymorphic SSRs						Forward primers (5'-3')	Annealing tempearture(0C)	Reverse primers (5'-3')	Annealing tempearture(0C)	Amplified product size (bp)	Structural annotation		Functional Annotation			
				C. microphyllum		ICC4958														
				Transcript	SSR repeat-motifs	Chromosomes	SSR repeat-motifs	Start physical positions (bp)	End physical positions (bp)						Gene IDs	Sequence component of genes	NCBI nr IDs	TFs	KOG	KEGG
TR39023|c1_g1_i2	No	(ATT)8	24	--	--	--	--	--	--	ATGATACCGCGAACCCAAC	61	CTTCTTCTAAGTTGCGGCGA	61	221	--	--	--	--	--	--
TR39023|c3_g1_i1	XLOC_014089	(TC)9	18	--	--	--	--	--	--	GGAAGATAAACGTTTGGGCA	60	AAAAGAATGGTGAGTGCGCTA	60	164	--	--	XM_004495960.1	bHLH	[KT]	--
TR39023|c3_g1_i1	XLOC_014089	(AAT)8	24	--	--	--	--	--	--	TAACCGATCCAAACACACGA	60	TGGACCCCAATTGTAACACA	60	254	--	--	XM_004495960.1	bHLH	[KT]	--
TR39023|c4_g1_i1	XLOC_014090	(TA)8	16	--	--	--	--	--	--	TACGGACGGAAGTGTCCAAT	60	CCCACACTTTATTTTTGCCA	59	222	--	--	--	--	--	--
TR39031|c0_g1_i1	XLOC_014099	(CT)6	12	--	--	--	--	--	--	TTCAATCTTGGAACGCAAAA	59	GTTACCAGCTGGCACAATGA	60	248	--	--	--	--	--	--
TR39032|c1_g1_i1	No	(GT)8	16	--	--	--	--	--	--	AACGAGCGGCTCTTCAACT	60	TGTTGAAATGGACTTTGGCA	60	112	--	--	--	--	--	--
TR39037|c0_g1_i1	No	(CTT)6	18	--	--	--	--	--	--	CATCAATTGGATGCATCAGG	60	TTGAACTCCCAGCATTGAAA	59	268	--	--	--	--	--	--
TR39037|c0_g1_i10	No	(CTT)6	18	--	--	--	--	--	--	ATGGATATGCATCCTCAGGC	60	CTTTCTCTTGTGAGACCCCC	59	245	--	--	--	--	--	--
TR3904|c11_g1_i1	XLOC_014130	(GTT)5	15	--	--	--	--	--	--	TGATCCATGTAGCCAACCCT	60	AAGGAGCAAACTCACTTGCC	59	141	--	--	--	--	--	--
TR3904|c8_g6_i2	No	(AT)8	16	--	--	--	--	--	--	AGCGAGAGCGATCCTTATCA	60	TCTCTGCCGGTCATAGTTGA	59	269	--	--	--	--	--	--
TR3904|c8_g6_i3	No	(AT)8	16	--	--	--	--	--	--	AGCGAGAGCGATCCTTATCA	60	TCTCTGCCGGTCATAGTTGA	59	269	--	--	--	--	--	--
TR39045|c1_g1_i1	XLOC_014123	(CAG)5	15	--	--	--	--	--	--	ATTTTCGTTGTTCTCCACGC	60	TAGCTGCTGCTGCTGTTGTT	60	232	--	--	--	--	--	--
TR39046|c1_g2_i1	No	(CACAA)5	25	--	--	--	--	--	--	TCCCCACTATACTCATACTCCCTC	60	AACGGTTCAAAGCTAACCGA	60	237	--	--	--	--	--	--
TR39048|c0_g1_i4	No	(AGT)6	18	--	--	--	--	--	--	GTTGTCGCAAATGATGATGC	60	AAGCAACACCTCAAGCTCGT	60	267	--	--	--	--	--	--

Transcript IDs	Differentially expressed gene (DEG) IDs	SSR repeat-motifs	SSR repeat-length (bp)	*In silico* polymorphic SSRs						Forward primers (5'-3')	Annealing tempearture(0C)	Reverse primers (5'-3')	Annealing tempearture(0C)	Amplified product size (bp)	Structural annotation		Functional Annotation			
				C. microphyllum		ICC4958									Gene IDs	Sequence component of genes	NCBI nr IDs	TFs	KOG	KEGG
				Transcript	SSR repeat-motifs	Chromosomes	SSR repeat-motifs	Start physical positions (bp)	End physical positions (bp)											
TR39048|c0_g1_i7	No	(AGT)6	18	--	--	--	--	--	--	GAAGGAATTGGAAACGACGA	60	CTTCCACCAGACCGATCTTC	60	263	--	--	--	--	--	--
TR39049|c0_g1_i1	No	(TC)9	18	TR39049|c0_g1_i1	(TC)9	scaffold22297	(TC)25	12503	12552	GGATTGTCATTGATCGCTGTT	60	AGGCACGTGTTAAGGAACCA	61	265	Ca_25526	DRR	--	--	--	--
TR39055|c0_g1_i1	XLOC_014142	(TGA)6	18	--	--	--	--	--	--	TGAAATTGCATGTCTCGGAA	60	CGCTGCAAAAGACTCATCAA	60	266	--	--	--	--	--	--
TR3907|c5_g3_i1	No	(CT)7	14	--	--	--	--	--	--	TCCAATTTCCCTTTCTTCTTCA	60	TGCAAATCCTTGAGCTCCTT	60	119	--	--	--	--	--	--
TR3907|c5_g3_i2	XLOC_014192	(CT)7	14	--	--	--	--	--	--	TCCAATTTCCCTTTCTTCTTCA	60	TGCAAATCCTTGAGCTCCTT	60	119	--	--	--	--	--	--
TR3908|c1_g1_i2	No	(TC)10	20	--	--	--	--	--	--	TCCACACAATTCAAATCCGA	60	CCAAGCAGATGCAACAGAGA	60	147	--	--	--	--	--	--
TR3908|c2_g1_i4	No	(CTT)5catctctcttgaattctctgtttctcaccgtgcttctgcatttttaacaagaaggttaaaaacccattaacaaaaattgcatattttgttcaacg(TC)7	124	--	--	--	--	--	--	TTTCGCGTCTCATTGAAGC	60	TTCAGCTTTGGACAAAAGGG	60	274	--	--	--	--	--	--
TR3911|c6_g1_i1	XLOC_014237	(TC)9	18	--	--	--	--	--	--	AATGCAAACTCCAAACCCAG	60	GAATCGGAGGAGATTGGTGA	60	204	--	--	--	--	--	--
TR3912|c5_g1_i1	No	(TTC)5	15	--	--	--	--	--	--	ACCGATGAAATGAAGGCAAC	60	GGAAAGAGCGGTGTGTAGGA	60	224	--	--	--	--	--	--
TR3912|c5_g1_i2	No	(TTC)5	15	--	--	--	--	--	--	ACCGATGAAATGAAGGCAAC	60	GGAAAGAGCGGTGTGTAGGA	60	224	--	--	--	--	--	--
TR3912|c5_g1_i4	No	(TTC)5	15	--	--	--	--	--	--	ACCGATGAAATGAAGGCAAC	60	GGAAAGAGCGGTGTGTAGGA	60	224	--	--	--	--	--	--
TR39200|c0_g1_i1	No	(GAG)5	15	--	--	--	--	--	--	ACGGTGCCGTATTGAGAAAC	60	AGCTGCTTTAGAGCCTGGTT	58	174	--	--	--	--	--	--
TR39209|c0_g1_i1	No	(GAT)5	15	--	--	--	--	--	--	TTTTCCGACAACCCAAGAAG	60	TGTGAAGCCATTCGTTTGAG	60	200	--	--	--	--	--	--
TR3923|c0_g1_i1	No	(AG)9	18	--	--	--	--	--	--	TCTGCGTTGTGAGTGTGATG	59	GCATTTCTCTGCCTCCTGTC	60	109	--	--	--	--	--	--

Transcript IDs	Differentially expressed gene (DEG) IDs	SSR repeat-motifs	SSR repeat-length (bp)	*In silico* polymorphic SSRs						Forward primers (5'-3')	Annealing tempearture(0C)	Reverse primers (5'-3')	Annealing tempearture(0C)	Amplified product size (bp)	Structural annotation		Functional Annotation			
				C. microphyllum		ICC4958									Gene IDs	Sequence component of genes	NCBI nr IDs	TFs	KOG	KEGG
				Transcript	SSR repeat-motifs	Chromosomes	SSR repeat-motifs	Start physical positions (bp)	End physical positions (bp)											
TR3923|c0_g2_i1	XLOC_014309	(AG)9	18	--	--	--	--	--	--	GTCGGAGAAGAGGAGAAGGG	60	GCATTTCTCTGCCTCCTGTC	60	185	--	--	--	--	--	--
TR3923|c1_g1_i1	No	(CAA)5	15	--	--	--	--	--	--	CAGACTCCACCGCATCTGTA	60	GAGATTTGCGTTGATGCTGA	60	124	--	--	--	--	--	--
TR3923|c1_g2_i1	No	(CAA)5	15	--	--	--	--	--	--	CAGACTCCACCGCATCTGTA	60	GAGATTTGCGTTGATGCTGA	60	124	--	--	--	--	--	--
TR3923|c3_g1_i1	No	(CTT)10	30	TR3923|c3_g1_i1	(CTT)10	Ca_LG_3	(CTT)6	36334364	36334381	TCAGGAACACAATCCACTCG	60	ATTGGACCATTATCGTTGGC	60	189		URR	--	--	--	--
TR3923|c3_g1_i6	No	(CTT)10	30	--	--	--	--	--	--	TCAGGAACACAATCCACTCG	60	ATTGGACCATTATCGTTGGC	60	189	--	--	--	--	--	--
TR3924|c0_g1_i1	XLOC_014315	(CT)7	14	--	--	--	--	--	--	ACACATGAACAAAAACGGCA	60	GGGTAACTGGGGGAAAGAGA	60	226	--	--	--	--	--	--
TR3924|c0_g1_i1	XLOC_014315	(CAC)7	21	--	--	--	--	--	--	TCTTTCCCCCAGTTACCCTC	60	TGGGGGTTTAGCAGGTACAG	60	178	--	--	--	--	--	--
TR3924|c1_g1_i1	XLOC_014316	(CAC)7	21	--	--	--	--	--	--	TACCCTCCAGTCTCACCACC	60	TTGTGGCGTTTGATGATGTT	60	217	--	--	--	--	--	--
TR3924|c3_g1_i1	XLOC_014320	(GA)9	18	TR3924|c3_g1_i1	(GGT)8	Ca_LG_6	(GGT)6	46746540	46746557	TTTTCCCTTTTGTTTCGCAC	60	GAGCATCTAGATCTTACAGCCGA	60	128	Ca_15546	URR	--	--	--	--
TR39260|c1_g1_i1	No	(GT)10	20	--	--	--	--	--	--	GCGGTGTGTGTCGTTACAAG	60	ATAATAATGCCGCGTGGAAA	60	193	--	--	--	--	--	--
TR39291|c0_g1_i1	No	(TCT)5	15	--	--	--	--	--	--	CAGCCTCCAGCTTACTTGGT	59	TTGCCCACTTCAAGGCTACT	60	190	--	--	--	--	--	--
TR39291|c0_g2_i1	No	(TCT)5	15	--	--	--	--	--	--	CAGCCTCCAGCTTACTTGGT	59	TTGCCCACTTCAAGGCTACT	60	190	--	--	--	--	--	--
TR39306|c0_g1_i1	No	(CT)7	14	--	--	--	--	--	--	CTGTTTGAACCACGAGCTGA	60	TGTCCTGTTGTTGCTTTGACA	60	191	--	--	--	--	--	--
TR39306|c0_g2_i1	No	(CT)7	14	--	--	--	--	--	--	CTGTTTGAACCACGAGCTGA	60	TGTCCTGTTGTTGCTTTGACA	60	191	--	--	--	--	--	--

Transcript IDs	Differentially expressed gene (DEG) IDs	SSR repeat-motifs	SSR repeat-length (bp)	*In silico* polymorphic SSRs						Forward primers (5'-3')	Annealing tempearture(0C)	Reverse primers (5'-3')	Annealing tempearture(0C)	Amplified product size (bp)	Structural annotation		Functional Annotation			
				C. microphyllum		ICC4958									Gene IDs	Sequence component of genes	NCBI nr IDs	TFs	KOG	KEGG
				Transcript	SSR repeat-motifs	Chromosomes	SSR repeat-motifs	Start physical positions (bp)	End physical positions (bp)											
TR3932|c3_g1_i1	No	(TC)6	12	--	--	--	--	--	--	GGCTCCTAGGTGTCATGGAA	60	TTTTCATTGCCACCCATTTC	61	147	--	--	--	--	--	--
TR3932|c3_g2_i1	No	(TC)6	12	--	--	--	--	--	--	GGCTCCTAGGTGTCATGGAA	60	TTTTCATTGCCACCCATTTC	61	147	--	--	--	--	--	--
TR3932|c4_g1_i1	XLOC_014381	(ATA)7	21	--	--	--	--	--	--	CCACCGTAAACACATAACTCAAA	59	TTTGCCACATCAAGCAAGTT	59	260	--	--	--	--	--	--
TR3932|c4_g1_i2	XLOC_014382	(ATA)7	21	--	--	--	--	--	--	CCACCGTAAACACATAACTCAAA	59	TTTGCCACATCAAGCAAGTT	59	260	--	--	--	--	--	--
TR3932|c6_g1_i1	No	(ATG)6	18	--	--	--	--	--	--	TCGTGTGGGAAACACAACTT	59	TGTCTGTAATCCTTCTGCCG	59	279	--	--	--	--	--	--
TR3932|c6_g1_i4	No	(ATG)6	18	--	--	--	--	--	--	TCGTGTGGGAAACACAACTT	59	TGTCTGTAATCCTTCTGCCG	59	279	--	--	--	--	--	--
TR3932|c6_g2_i1	No	(AT)6	12	--	--	--	--	--	--	CTTCATGTTGAGCAGAGCCA	60	TTGAGTTGCCTTACCTACAAGG	58	205	--	--	--	--	--	--
TR3932|c8_g1_i1	XLOC_014384	(TAA)7	21	--	--	--	--	--	--	GCAGAAGGAAAAACCGAACA	60	GCAAAGGGAAAATCATTGGA	60	203	--	--	XP_004495320.1	ERF	[P]	--
TR39332|c0_g1_i1	No	(TGT)5	15	--	--	--	--	--	--	GCGAGCTACCTTCATTCGTC	60	TGCAATCTGATCTCAGCACA	59	183	--	--	--	--	--	--
TR39332|c0_g2_i1	No	(TGT)5	15	--	--	--	--	--	--	GCGAGCTACCTTCATTCGTC	60	TGCAATCTGATCTCAGCACA	59	183	--	--	--	--	--	--
TR39334|c0_g1_i2	No	(AT)6	12	--	--	--	--	--	--	TTGCTTTCCTAATCCCTGGTT	60	TCCTTCGTATTTGCTTTCACA	58	262	--	--	--	--	--	--
TR39334|c0_g1_i4	No	(AT)6	12	--	--	--	--	--	--	TTGCTTTCCTAATCCCTGGTT	60	TCCTTCGTATTTGCTTTCACA	58	262	--	--	--	--	--	--
TR39363|c0_g1_i1	XLOC_014399	(TAG)6	18	--	--	--	--	--	--	GTATGCAGGGGTGCATTCTT	60	GCTGACTCTCCCCTACCCTT	60	227	--	--	--	--	--	--
TR39455|c0_g2_i1	No	(AGA)8	24	--	--	--	--	--	--	CGGCGTACTTTCGAGACACT	60	CAGCTCCAAAGCAAAAACTG	59	221	--	--	--	--	--	--

<table>
<tr><th rowspan="3">Transcript IDs</th><th rowspan="3">Differentially expressed gene (DEG) IDs</th><th rowspan="3">SSR repeat-motifs</th><th rowspan="3">SSR repeat-length (bp)</th><th colspan="6">In silico polymorphic SSRs</th><th rowspan="3">Forward primers (5'-3')</th><th rowspan="3">Annealing tempearture(0C)</th><th rowspan="3">Reverse primers (5'-3')</th><th rowspan="3">Annealing tempearture(0C)</th><th rowspan="3">Amplified product size (bp)</th><th colspan="2">Structural annotation</th><th colspan="4">Functional Annotation</th></tr>
<tr><th colspan="2">C. microphyllum</th><th colspan="4">ICC4958</th><th rowspan="2">Gene IDs</th><th rowspan="2">Sequence component of genes</th><th rowspan="2">NCBI nr IDs</th><th rowspan="2">TFs</th><th rowspan="2">KOG</th><th rowspan="2">KEGG</th></tr>
<tr><th>Transcript</th><th>SSR repeat-motifs</th><th>Chromosomes</th><th>SSR repeat-motifs</th><th>Start physical positions (bp)</th><th>End physical positions (bp)</th></tr>
<tr><td>TR39461|c0_g1_i1</td><td>No</td><td>(TTC)6</td><td>18</td><td>--</td><td>--</td><td>--</td><td>--</td><td>--</td><td>--</td><td>CCCACTCCTTCCTCCTCTTC</td><td>60</td><td>AAGCGGTTGTTGAGAGGTTG</td><td>60</td><td>263</td><td>--</td><td>--</td><td>--</td><td>--</td><td>--</td><td>--</td></tr>
<tr><td>TR39461|c0_g2_i1</td><td>XLOC_014444</td><td>(TTC)6</td><td>18</td><td>--</td><td>--</td><td>--</td><td>--</td><td>--</td><td>--</td><td>CCCACTCCTTCCTCCTCTTC</td><td>60</td><td>AAGCGGTTGTTGAGAGGTTG</td><td>60</td><td>263</td><td>--</td><td>--</td><td>--</td><td>--</td><td>--</td><td>--</td></tr>
<tr><td>TR39461|c0_g3_i1</td><td>No</td><td>(CAT)5</td><td>15</td><td>--</td><td>--</td><td>--</td><td>--</td><td>--</td><td>--</td><td>CAAATCATTACATTGCCCCC</td><td>60</td><td>CGAGCTGGATTTTCTTCCTG</td><td>60</td><td>106</td><td>--</td><td>--</td><td>--</td><td>--</td><td>--</td><td>--</td></tr>
<tr><td>TR39483|c1_g1_i1</td><td>XLOC_014460</td><td>(CCT)6</td><td>18</td><td>--</td><td>--</td><td>--</td><td>--</td><td>--</td><td>--</td><td>CAGGACTCTCCTGCCTTTGT</td><td>59</td><td>GTGTCGTGAGAGAGTGGCAA</td><td>60</td><td>280</td><td>--</td><td>--</td><td>--</td><td>--</td><td>--</td><td>--</td></tr>
<tr><td>TR39497|c0_g2_i1</td><td>No</td><td>(AG)8</td><td>16</td><td>--</td><td>--</td><td>--</td><td>--</td><td>--</td><td>--</td><td>TTCCCTTAGGGTTGATGGTG</td><td>60</td><td>TGTGCTCTTCCGATCTAGCA</td><td>60</td><td>246</td><td>--</td><td>--</td><td>--</td><td>--</td><td>--</td><td>--</td></tr>
<tr><td>TR39500|c0_g1_i1</td><td>XLOC_014471</td><td>(AG)8(AC)7</td><td>30</td><td>--</td><td>--</td><td>--</td><td>--</td><td>--</td><td>--</td><td>TCATTCAGAGTAACCAACCCTATT</td><td>58</td><td>GGACATAGCCCACCACTGTT</td><td>60</td><td>184</td><td>--</td><td>--</td><td>--</td><td>--</td><td>--</td><td>--</td></tr>
<tr><td>TR39500|c0_g1_i1</td><td>XLOC_014471</td><td>(ACA)6ctcctggtttaggttactatttcatggacaataacaa caacaatggaagctcttcttcc(TCT)5</td><td>92</td><td>--</td><td>--</td><td>--</td><td>--</td><td>--</td><td>--</td><td>TCTCTTCCTTCCTATACCTTCCA</td><td>58</td><td>AAGCTTCCAAGAGACGGTGA</td><td>60</td><td>242</td><td>--</td><td>--</td><td>--</td><td>--</td><td>--</td><td>--</td></tr>
<tr><td>TR39500|c0_g1_i1</td><td>XLOC_014471</td><td>(AAT)6</td><td>18</td><td>--</td><td>--</td><td>--</td><td>--</td><td>--</td><td>--</td><td>TGGGCTATGTCCAATGATGA</td><td>60</td><td>CCAGGAGTGTTGTTGTTGTTG</td><td>59</td><td>255</td><td>--</td><td>--</td><td>--</td><td>--</td><td>--</td><td>--</td></tr>
<tr><td>TR39506|c0_g2_i2</td><td>No</td><td>(TG)6</td><td>12</td><td>--</td><td>--</td><td>--</td><td>--</td><td>--</td><td>--</td><td>TCATGTCCATGCAGCAATCT</td><td>60</td><td>CACAAACTTCAGCCGCAATA</td><td>60</td><td>198</td><td>--</td><td>--</td><td>--</td><td>--</td><td>--</td><td>--</td></tr>
<tr><td>TR39512|c0_g1_i1</td><td>XLOC_014482</td><td>(TTC)5</td><td>15</td><td>--</td><td>--</td><td>--</td><td>--</td><td>--</td><td>--</td><td>TCCCAACCAATGCCATAAAT</td><td>60</td><td>TCAGCAGCCATAGCAACAAC</td><td>60</td><td>177</td><td>--</td><td>--</td><td>XM_004492306.1</td><td>--</td><td>[G]</td><td>--</td></tr>
<tr><td>TR39521|c0_g2_i2</td><td>No</td><td>(T)10atcatcgaattcttgcaatttaatatcatgatttcttgtga atttttagctgctgatttattatgaattatgtgcttcttgcattttga gaaag(A)11</td><td>115</td><td>--</td><td>--</td><td>--</td><td>--</td><td>--</td><td>--</td><td>AGAGCCAGAGATCGGATCAA</td><td>60</td><td>TTCCTTTTACTCCAACATGAGC</td><td>58</td><td>249</td><td>--</td><td>--</td><td>--</td><td>--</td><td>--</td><td>--</td></tr>
<tr><td>TR39538|c0_g1_i1</td><td>XLOC_014504</td><td>(TC)8</td><td>16</td><td>--</td><td>--</td><td>--</td><td>--</td><td>--</td><td>--</td><td>CACCCACCACCAACTTTCTC</td><td>60</td><td>CTGTAAAGCTCAGGTTGCCC</td><td>60</td><td>259</td><td>--</td><td>--</td><td>--</td><td>--</td><td>--</td><td>--</td></tr>
<tr><td>TR39551|c0_g1_i1</td><td>XLOC_014510</td><td>(AGA)6</td><td>18</td><td>--</td><td>--</td><td>--</td><td>--</td><td>--</td><td>--</td><td>TTGAGTTTCAGCACACCTCG</td><td>60</td><td>TCTTTTTGCAAACACCATGC</td><td>60</td><td>191</td><td>--</td><td>--</td><td>--</td><td>--</td><td>--</td><td>--</td></tr>
<tr><td>TR39620|c0_g1_i1</td><td>XLOC_014534</td><td>(GA)7</td><td>14</td><td>--</td><td>--</td><td>--</td><td>--</td><td>--</td><td>--</td><td>TACCATCCACGCAAATCTCA</td><td>60</td><td>TTCCAAACATCAAACAACGC</td><td>60</td><td>279</td><td>--</td><td>--</td><td>--</td><td>--</td><td>--</td><td>--</td></tr>
</table>

Transcript IDs	Differentially expressed gene (DEG) IDs	SSR repeat-motifs	SSR repeat-length (bp)	*In silico* polymorphic SSRs						Forward primers (5'-3')	Annealing tempearture(0C)	Reverse primers (5'-3')	Annealing tempearture(0C)	Amplified product size (bp)	Structural annotation		Functional Annotation			
				C. microphyllum		ICC4958									Gene IDs	Sequence component of genes	NCBI nr IDs	TFs	KOG	KEGG
				Transcript	SSR repeat-motifs	Chromosomes	SSR repeat-motifs	Start physical positions (bp)	End physical positions (bp)											
TR39637|c0_g1_i1	XLOC_014552	(ACG)6	18	--	--	--	--	--	--	TTCAATGCCTTTTCCAAACC	60	TGTTGTCCTGTTCTTGCAGC	60	164	--	--	--	--	--	--
TR39660|c1_g1_i2	No	(A)12ggttgtggaacttttgtaagtgtattggaacactgtcacgagaagcttgccgatgcttaattctcccc(TATT)5	100	--	--	--	--	--	--	TGACTTGGTACTGGAAAGTGGA	60	TCGTATACCATCATGCTCCTTG	60	264	--	--	--	--	--	--
TR39672|c0_g1_i1	No	(ATA)6gattgaatggaaaaagaaaaaaggtaaagggccagcttaatattccttgcttgaaatcattttg(T)10	92	--	--	--	--	--	--	ATCAAATTGGTTGGATGGGA	60	CGATTTGGCAAAAGAGTGGT	60	262	--	--	--	--	--	--
TR39672|c0_g2_i1	No	(ATA)6gattgaatggaaaaagaaaaaaggtaaagggccagcttaatattccttgcttgaaatcattttg(T)10	92	--	--	--	--	--	--	ATCAAATTGGTTGGATGGGA	60	CGATTTGGCAAAAGAGTGGT	60	262	--	--	--	--	--	--
TR39712|c5_g1_i1	XLOC_014616	(TC)10tgtattgtatctccttctttcttacgcgtcttcatttctactctcacacacta(CT)9	91	--	--	--	--	--	--	CTCAACACTTTCATTCATTGCC	60	TTTAGAAGCCAACCACCACC	60	176	--	--	--	--	--	--
TR39712|c8_g6_i1	XLOC_014623	(CA)11	22	--	--	--	--	--	--	ACACCAAAGCCGTTTGATTC	60	GCTTTTGTGTGTTGTGTTTGTG	59	186	--	--	--	--	--	--
TR39712|c8_g6_i3	No	(CA)11	22	--	--	--	--	--	--	ACACCAAAGCCGTTTGATTC	60	GCTTTTGTGTGTTGTGTTTGTG	59	186	--	--	--	--	--	--
TR39714|c2_g2_i1	XLOC_014633	(AG)9	18	--	--	--	--	--	--	TTGCAATGTTCCAACACCAT	60	GGTATTCCAACGTCGAAAGC	60	158	--	--	--	--	--	--
TR39716|c0_g5_i1	XLOC_014637	(AC)6	12	--	--	--	--	--	--	AGGAAGAAGCAAAAACCCGT	60	GTTCATCAAGAACGATCCCG	60	137	--	--	--	--	--	--
TR39717|c0_g3_i1	No	(TA)10	20	--	--	--	--	--	--	CATTGGCAGATGTTGATGAAG	59	ATTCAGTTCCAAGCAGTCCG	60	230	--	--	--	--	--	--
TR39717|c0_g3_i2	No	(TA)10	20	--	--	--	--	--	--	CATTGGCAGATGTTGATGAAG	59	CTGGCACAGAACTGCTTGAA	60	280	--	--	--	--	--	--
TR39717|c0_g3_i3	No	(TA)10	20	--	--	--	--	--	--	CATTGGCAGATGTTGATGAAG	59	ATTCAGTTCCAAGCAGTCCG	60	230	--	--	--	--	--	--
TR39722|c2_g2_i10	No	(CT)10	20	--	--	--	--	--	--	TTCCGTTTCACAACCGTTTA	59	CGCAGAGCTTTCACACTCAG	60	106	--	--	--	--	--	--
TR39722|c2_g2_i12	No	(CT)10	20	--	--	--	--	--	--	TTCCGTTTCACAACCGTTTA	59	CGCAGAGCTTTCACACTCAG	60	106	--	--	--	--	--	--

Transcript IDs	Differentially expressed gene (DEG) IDs	SSR repeat-motifs	SSR repeat-length (bp)	*In silico* polymorphic SSRs						Forward primers (5'-3')	Annealing tempearture(0C)	Reverse primers (5'-3')	Annealing tempearture(0C)	Amplified product size (bp)	Structural annotation		Functional Annotation			
				C. microphyllum		ICC4958														
				Transcript	SSR repeat-motifs	Chromosomes	SSR repeat-motifs	Start physical positions (bp)	End physical positions (bp)						Gene IDs	Sequence component of genes	NCBI nr IDs	TFs	KOG	KEGG
TR39722|c2_g2_i2	No	(CT)10	20	--	--	--	--	--	--	TTCCGTTTCACAACCGTTTA	59	CGCAGAGCTTTCACACTCAG	60	106	--	--	--	--	--	--
TR39722|c2_g2_i3	No	(CT)10	20	--	--	--	--	--	--	TTCCGTTTCACAACCGTTTA	59	CGCAGAGCTTTCACACTCAG	60	106	--	--	--	--	--	--
TR39722|c2_g2_i4	No	(CT)10	20	--	--	--	--	--	--	TTCCGTTTCACAACCGTTTA	59	CGCAGAGCTTTCACACTCAG	60	106	--	--	--	--	--	--
TR39722|c2_g2_i7	No	(CT)10	20	--	--	--	--	--	--	TTCCGTTTCACAACCGTTTA	59	CGCAGAGCTTTCACACTCAG	60	106	--	--	--	--	--	--
TR39722|c2_g2_i9	No	(CT)10	20	--	--	--	--	--	--	TTCCGTTTCACAACCGTTTA	59	CGCAGAGCTTTCACACTCAG	60	106	--	--	--	--	--	--
TR39724|c1_g1_i1	No	(AAAT)5	20	--	--	--	--	--	--	TGCATCAGACCACTTGTGTTT	59	TTTGGAAGGAAAGGTGTTGC	60	265	--	--	--	--	--	--
TR39724|c1_g2_i1	No	(AAAT)5	20	--	--	--	--	--	--	TGCATCAGACCACTTGTGTTT	59	TTTGGAAGGAAAGGTGTTGC	60	265	--	--	--	--	--	--
TR39724|c3_g1_i1	XLOC_014663	(TC)11	22	--	--	--	--	--	--	AAACTCTCCCTACCACGCCT	60	TTCATCATATTCGACGCCAA	60	225	--	--	--	--	--	--
TR39724|c3_g1_i2	No	(TC)11	22	--	--	--	--	--	--	AAACTCTCCCTACCACGCCT	60	TTCATCATATTCGACGCCAA	60	225	--	--	--	--	--	--
TR39727|c0_g1_i2	No	(AG)7	14	--	--	--	--	--	--	TTTGTTCAAATTTTGCGCTG	60	CGCACCACAAAACCCTCTAT	60	123	--	--	--	--	--	--
TR39737|c3_g1_i1	No	(GA)6	12	--	--	--	--	--	--	AAGCAATGATTCAGATGGGC	60	GAATGTAGCAGGAAACCGGA	60	263	--	--	--	--	--	--
TR39737|c3_g1_i10	No	(TC)6	12	--	--	--	--	--	--	GCGTGTCTCATTCATCTCCA	60	GAAACGGCGGTTGAGAGTTA	60	250	--	--	--	--	--	--
TR39737|c3_g1_i11	No	(GA)6	12	--	--	--	--	--	--	AAGCAATGATTCAGATGGGC	60	GAATGTAGCAGGAAACCGGA	60	263	--	--	--	--	--	--
TR39737|c3_g1_i13	No	(GA)6	12	--	--	--	--	--	--	AAGCAATGATTCAGATGGGC	60	GAATGTAGCAGGAAACCGGA	60	263	--	--	--	--	--	--

Transcript IDs	Differentially expressed gene (DEG) IDs	SSR repeat-motifs	SSR repeat-length (bp)	*In silico* polymorphic SSRs						Forward primers (5'-3')	Annealing tempearture(0C)	Reverse primers (5'-3')	Annealing tempearture(0C)	Amplified product size (bp)	Structural annotation		Functional Annotation			
				C. microphyllum		ICC4958									Gene IDs	Sequence component of genes	NCBI nr IDs	TFs	KOG	KEGG
				Transcript	SSR repeat-motifs	Chromosomes	SSR repeat-motifs	Start physical positions (bp)	End physical positions (bp)											
TR39737|c3_g1_i4	No	(TC)6	12	--	--	--	--	--	--	GCGTGTCTCATTCATCTCCA	60	GAAACGGCGGTTGAGAGTTA	60	250	--	--	--	--	--	--
TR39737|c3_g1_i5	XLOC_014706	(TC)6	12	--	--	--	--	--	--	GCGTGTCTCATTCATCTCCA	60	GAAACGGCGGTTGAGAGTTA	60	250	--	--	--	--	--	--
TR39737|c3_g1_i6	No	(GA)6	12	--	--	--	--	--	--	AAGCAATGATTCAGATGGGC	60	ACCAATAGCAGCCAGACGTT	60	209	--	--	--	--	--	--
TR39737|c7_g1_i1	XLOC_014710	(AGT)5	15	--	--	--	--	--	--	AATGCCAACAAACCAAAAGC	60	TGACAAATGCAAATTCCAATG	59	236	--	--	--	--	--	--
TR39738|c3_g1_i1	XLOC_014715	(AGA)6	18	--	--	--	--	--	--	TAATCCTCCTCCTCCGTGTG	60	CTTCCGTTTCCGGTACACAT	60	276	--	--	--	--	--	--
TR39740|c0_g4_i1	No	(GA)9	18	--	--	--	--	--	--	AAAAAGAAAACCGGGGAGAA	60	TCTATTCTTCATCCCACGCC	60	115	--	--	--	--	--	--
TR39746|c1_g1_i10	No	(TC)6	12	--	--	--	--	--	--	TGCAGCATTGAATGTGAATTT	59	CCGGTTTGTCTTCCTCTTCA	60	143	--	--	--	--	--	--
TR39746|c1_g1_i2	No	(TC)6	12	--	--	--	--	--	--	TGCAGCATTGAATGTGAATTT	59	CCGGTTTGTCTTCCTCTTCA	60	143	--	--	--	--	--	--
TR39746|c1_g1_i3	No	(TC)6	12	--	--	--	--	--	--	TGCAGCATTGAATGTGAATTT	59	CCGGTTTGTCTTCCTCTTCA	60	143	--	--	--	--	--	--
TR39746|c1_g1_i4	No	(TC)6	12	--	--	--	--	--	--	TGCAGCATTGAATGTGAATTT	59	CCGGTTTGTCTTCCTCTTCA	60	143	--	--	--	--	--	--
TR39746|c1_g1_i6	No	(TC)6	12	--	--	--	--	--	--	TGCAGCATTGAATGTGAATTT	59	CCGGTTTGTCTTCCTCTTCA	60	143	--	--	--	--	--	--
TR39746|c1_g1_i7	No	(TC)6	12	--	--	--	--	--	--	TGCAGCATTGAATGTGAATTT	59	CCGGTTTGTCTTCCTCTTCA	60	143	--	--	--	--	--	--
TR39746|c1_g1_i8	No	(TC)6	12	--	--	--	--	--	--	TGCAGCATTGAATGTGAATTT	59	CCGGTTTGTCTTCCTCTTCA	60	143	--	--	--	--	--	--
TR39746|c1_g1_i9	No	(TC)6	12	--	--	--	--	--	--	TGCAGCATTGAATGTGAATTT	59	CCGGTTTGTCTTCCTCTTCA	60	143	--	--	--	--	--	--

Transcript IDs	Differentially expressed gene (DEG) IDs	SSR repeat-motifs	SSR repeat-length (bp)	*In silico* polymorphic SSRs						Forward primers (5'-3')	Annealing tempearture(0C)	Reverse primers (5'-3')	Annealing tempearture(0C)	Amplified product size (bp)	Structural annotation		Functional Annotation			
				C. microphyllum		ICC4958									Gene IDs	Sequence component of genes	NCBI nr IDs	TFs	KOG	KEGG
				Transcript	SSR repeat-motifs	Chromosomes	SSR repeat-motifs	Start physical positions (bp)	End physical positions (bp)											
TR39747|c2_g1_i1	No	(A)12tttaatcaaagag(A)10	35	--	--	--	--	--	--	GCGCCTGAGATTTTAGTTCG	60	GCGGTTAGATGGAGCTTGAG	60	224	--	--	--	--	--	--
TR39747|c2_g1_i4	No	(A)12tttaatcaaagag(A)10	35	--	--	--	--	--	--	GCGCCTGAGATTTTAGTTCG	60	GCGGTTAGATGGAGCTTGAG	60	224	--	--	--	--	--	--
TR39747|c2_g1_i6	No	(A)12tttaatcaaagag(A)10	35	--	--	--	--	--	--	GCGCCTGAGATTTTAGTTCG	60	GCGGTTAGATGGAGCTTGAG	60	224	--	--	--	--	--	--
TR39747|c2_g1_i8	No	(A)12tttaatcaaagag(A)10	35	--	--	--	--	--	--	GCGCCTGAGATTTTAGTTCG	60	GCGGTTAGATGGAGCTTGAG	60	224	--	--	--	--	--	--
TR39747|c2_g2_i1	No	(A)12tttaatcaaagag(A)10	35	--	--	--	--	--	--	GCGCCTGAGATTTTAGTTCG	60	GCGGTTAGATGGAGCTTGAG	60	224	--	--	--	--	--	--
TR39747|c3_g3_i1	No	(TC)7	14	--	--	--	--	--	--	AAAAGTCAGTGAGCATCGCA	60	ATCCGTAGCCATAGCGAGAA	60	270	--	--	--	--	--	--
TR39747|c3_g3_i10	No	(TC)7	14	--	--	--	--	--	--	AAAAGTCAGTGAGCATCGCA	60	TTCGAGGTGGAGTTTGAGGT	60	147	--	--	--	--	--	--
TR39747|c3_g3_i13	No	(TC)7	14	--	--	--	--	--	--	AAAAGTCAGTGAGCATCGCA	60	TTCGAGGTGGAGTTTGAGGT	60	147	--	--	--	--	--	--
TR39748|c1_g1_i4	XLOC_014744	(AAG)5	15	--	--	--	--	--	--	AGCAGCAATGAAAACAAACA	57	CCTTCATTCAGCCTCACACA	60	207	--	--	--	--	--	--
TR39748|c2_g1_i1	No	(GAT)6ttagatgatgatattggtgata(ATG)6	58	--	--	--	--	--	--	GCTAACGATTCTTCGACATCAG	59	AGAATTCAATGGCTCAGCGT	60	141	--	--	--	--	--	--
TR39760|c4_g1_i2	No	(TAA)7	21	--	--	--	--	--	--	ACCGAATGGTGGTAGTGGAG	60	ACTTGAATTTTGAGGCGGTG	60	265	--	--	--	--	--	--
TR39760|c5_g2_i1	XLOC_014781	(AG)11	22	--	--	--	--	--	--	AGCCTTCAAATCCCCCTTTA	60	TCTCCCTTCTCCTACCTCCC	60	250	--	--	--	--	--	--
TR39761|c0_g1_i2	No	(TC)7ttcagtaactgtgcaatggcgata(GAG)5	53	--	--	--	--	--	--	CACACCCAAAATACAACGCA	60	AGCTCCCAAAAACGATGATG	60	198	--	--	--	--	--	--
TR39761|c0_g1_i3	No	(TC)7ttcagtaactgtgcaatggcgata(GAG)5	53	--	--	--	--	--	--	CACACCCAAAATACAACGCA	60	AGCTCCCAAAAACGATGATG	60	198	--	--	--	--	--	--

Transcript IDs	Differentially expressed gene (DEG) IDs	SSR repeat-motifs	SSR repeat-length (bp)	*In silico* polymorphic SSRs						Forward primers (5'-3')	Annealing tempearture(0C)	Reverse primers (5'-3')	Annealing tempearture(0C)	Amplified product size (bp)	Structural annotation		Functional Annotation			
				C. microphyllum		ICC4958									Gene IDs	Sequence component of genes	NCBI nr IDs	TFs	KOG	KEGG
				Transcript	SSR repeat-motifs	Chromosomes	SSR repeat-motifs	Start physical positions (bp)	End physical positions (bp)											
TR39763|c1_g5_i1	XLOC_014798	(TTA)5	15	--	--	--	--	--	--	CCAATTCAAACACACAAAGCA	60	GGAACCCTTCTCTTTCCCTG	60	193	--	--	--	--	--	--
TR39766|c0_g1_i8	XLOC_014806	(AAAC)5	20	--	--	--	--	--	--	AGGATGGAGCATTGATCCTG	60	TACTATGTTTTGGGCCGAGG	60	273	--	--	--	--	--	--
TR39769|c0_g1_i1	No	(GA)7	14	--	--	--	--	--	--	CTGATGATGGGGCAGAGATT	60	AGAGGCGTTGATGAAACCAC	60	206	--	--	--	--	--	--
TR39775|c2_g1_i1	XLOC_014832	(AG)8	16	--	--	--	--	--	--	CCACCAAGGATTAGCCAGAA	60	TGATTCCCATCCACTAATTGC	60	269	--	--	XP_004495454.1	--	--	--
TR39775|c3_g2_i1	XLOC_014833	(ACA)5	15	--	--	--	--	--	--	TGCGCTGTGTTTGTAATGCT	60	GTTTGTCTCCCTGGTAAGCG	60	178	--	--	--	--	--	--
TR39775|c4_g1_i2	No	(TC)6	12	--	--	--	--	--	--	CAATCTTCGGCTCACACTCA	60	CGGAGAAGACGGTGTCAAAT	60	271	--	--	--	--	--	--
TR39775|c5_g1_i3	No	(AGA)7	21	--	--	--	--	--	--	TATGGTGTGGAAGATGAGCG	60	ATTGTTCGAGTGGTTTTCGC	60	229	--	--	--	--	--	--
TR39775|c5_g2_i1	XLOC_014837	(TCA)6	18	--	--	--	--	--	--	CGAACCTTCAATTCCTCCAA	60	TAGGAGGGTGTGGAGTGAGG	60	174	--	--	XP_004517199.1	--	--	--
TR39775|c6_g2_i1	No	(CTT)7	21	--	--	--	--	--	--	CCTTAAACAGCCTTCACCCA	60	AACCTCCATCTCATCGCATC	60	252	--	--	--	--	--	--
TR39775|c6_g4_i1	XLOC_014841	(ATA)5	15	--	--	--	--	--	--	CGGGTTGGATACAAAAGATTG	59	GATGGCTTACTTGGCAAACA	59	280	--	--	--	--	--	--
TR39781|c3_g1_i1	XLOC_014855	(CTT)6	18	--	--	--	--	--	--	TTCCACAATTCTCATTTTCATCA	59	GATCTCACAATTCGCAGCAA	60	176	--	--	--	--	--	--
TR39782|c0_g1_i1	No	(ATT)5	15	--	--	--	--	--	--	CAACAGCACCTCCTCTCCTC	60	GTGAAGGTTTGGAACGGAAA	60	250	--	--	--	--	--	--
TR39782|c0_g1_i10	No	(ATT)5	15	--	--	--	--	--	--	CAACAGCACCTCCTCTCCTC	60	GTGAAGGTTTGGAACGGAAA	60	250	--	--	--	--	--	--
TR39782|c0_g1_i11	No	(ATT)5	15	--	--	--	--	--	--	CAACAGCACCTCCTCTCCTC	60	GTGAAGGTTTGGAACGGAAA	60	250	--	--	--	--	--	--

Transcript IDs	Differentially expressed gene (DEG) IDs	SSR repeat-motifs	SSR repeat-length (bp)	*In silico* polymorphic SSRs						Forward primers (5'-3')	Annealing tempearture(0C)	Reverse primers (5'-3')	Annealing tempearture(0C)	Amplified product size (bp)	Structural annotation		Functional Annotation			
				C. microphyllum		ICC4958														
				Transcript	SSR repeat-motifs	Chromosomes	SSR repeat-motifs	Start physical positions (bp)	End physical positions (bp)						Gene IDs	Sequence component of genes	NCBI nr IDs	TFs	KOG	KEGG
TR39782|c0_g1_i12	No	(ATT)5	15	--	--	--	--	--	--	CAACAGCACCTCCTCTCCTC	60	GTGAAGGTTTGGAACGGAAA	60	250	--	--	--	--	--	--
TR39782|c0_g1_i13	No	(ATT)5	15	--	--	--	--	--	--	CAACAGCACCTCCTCTCCTC	60	GTGAAGGTTTGGAACGGAAA	60	250	--	--	--	--	--	--
TR39782|c0_g1_i14	No	(ATT)5	15	--	--	--	--	--	--	CAACAGCACCTCCTCTCCTC	60	GTGAAGGTTTGGAACGGAAA	60	250	--	--	--	--	--	--
TR39782|c0_g1_i16	No	(ATT)5	15	--	--	--	--	--	--	CAACAGCACCTCCTCTCCTC	60	GTGAAGGTTTGGAACGGAAA	60	250	--	--	--	--	--	--
TR39782|c0_g1_i3	No	(ATT)5	15	--	--	--	--	--	--	CAACAGCACCTCCTCTCCTC	60	GTGAAGGTTTGGAACGGAAA	60	250	--	--	--	--	--	--
TR39782|c0_g1_i5	No	(ATT)5	15	--	--	--	--	--	--	CAACAGCACCTCCTCTCCTC	60	GTGAAGGTTTGGAACGGAAA	60	250	--	--	--	--	--	--
TR39782|c0_g1_i6	No	(ATT)5	15	--	--	--	--	--	--	CAACAGCACCTCCTCTCCTC	60	GTGAAGGTTTGGAACGGAAA	60	250	--	--	--	--	--	--
TR39782|c0_g1_i7	No	(ATT)5	15	--	--	--	--	--	--	CAACAGCACCTCCTCTCCTC	60	GTGAAGGTTTGGAACGGAAA	60	250	--	--	--	--	--	--
TR39782|c0_g1_i8	No	(ATT)5	15	--	--	--	--	--	--	CAACAGCACCTCCTCTCCTC	60	GTGAAGGTTTGGAACGGAAA	60	250	--	--	--	--	--	--
TR39782|c0_g1_i9	No	(ATT)5	15	--	--	--	--	--	--	CAACAGCACCTCCTCTCCTC	60	GTGAAGGTTTGGAACGGAAA	60	250	--	--	--	--	--	--
TR39786|c1_g1_i1	No	(TC)9	18	--	--	--	--	--	--	CCAAAGGGTCTTCTTTGGTATC	59	GTCACCGTTGCTTTCCAGAT	60	272	--	--	--	--	--	--
TR39786|c1_g1_i2	No	(CT)8	16	--	--	--	--	--	--	TGTGAGATCATCCCAATCCA	60	TCAGAACATCAAAGCCTCCC	60	249	--	--	--	--	--	--
TR39786|c1_g1_i3	No	(CT)8	16	--	--	--	--	--	--	TGTGAGATCATCCCAATCCA	60	TCAGAACATCAAAGCCTCCC	60	249	--	--	--	--	--	--
TR39786|c1_g1_i4	No	(TC)9	18	--	--	--	--	--	--	CCAAAGGGTCTTCTTTGGTATC	59	GTCACCGTTGCTTTCCAGAT	60	272	--	--	--	--	--	--

Transcript IDs	Differentially expressed gene (DEG) IDs	SSR repeat-motifs	SSR repeat-length (bp)	*In silico* polymorphic SSRs						Forward primers (5'-3')	Annealing tempearture(0C)	Reverse primers (5'-3')	Annealing tempearture(0C)	Amplified product size (bp)	Structural annotation		Functional Annotation			
				C. microphyllum		ICC4958									Gene IDs	Sequence component of genes	NCBI nr IDs	TFs	KOG	KEGG
				Transcript	SSR repeat-motifs	Chromosomes	SSR repeat-motifs	Start physical positions (bp)	End physical positions (bp)											
TR39787|c1_g1_i10	XLOC_014875	(TC)6	12	--	--	--	--	--	--	TATAACTCTATCCCTCGCAGGT	57	TTTCTGCATAAGTCAGTCAAGTGTT	59	275	--	--	--	--	--	--
TR39787|c1_g1_i2	No	(TC)6	12	--	--	--	--	--	--	TATAACTCTATCCCTCGCAGGT	57	TTTCTGCATAAGTCAGTCAAGTGTT	59	275	--	--	--	--	--	--
TR39787|c1_g1_i5	No	(TC)6	12	--	--	--	--	--	--	TATAACTCTATCCCTCGCAGGT	57	TTTCTGCATAAGTCAGTCAAGTGTT	59	275	--	--	--	--	--	--
TR39788|c0_g1_i1	No	(TA)8	16	--	--	--	--	--	--	TCACCACTAACGACAGCACA	59	GTCAGCAAGGCCATGAAGTT	60	156	--	--	--	--	--	--
TR39788|c0_g1_i2	No	(TA)8	16	--	--	--	--	--	--	CCCAACACTAACGACAGCAC	59	GTCAGCAAGGCCATGAAGTT	60	157	--	--	--	--	--	--
TR39788|c0_g1_i3	No	(TA)8	16	--	--	--	--	--	--	TCACCACTAACGACAGCACA	59	GTCAGCAAGGCCATGAAGTT	60	156	--	--	--	--	--	--
TR39788|c0_g1_i4	No	(TA)8	16	--	--	--	--	--	--	CCCAACACTAACGACAGCAC	59	GTCAGCAAGGCCATGAAGTT	60	157	--	--	--	--	--	--
TR39791|c1_g1_i1	XLOC_014883	(TAT)8	24	TR39791|c1_g1_i1	(TAT)8	Ca_LG_6	(TAT)9	18616860	18616886	AATCAGCTTCGCCAATCCTA	60	CCCCTTGTTGACGAATCACT	60	178	Ca_14305	URR	XP_004505214.1	ERF	[K]	--
TR39791|c5_g1_i1	XLOC_014888	(ACA)7	21	--	--	--	--	--	--	TCCAATGATGCCAAAGTTGA	60	CAAGCGATGAACATTGAAGG	59	274	--	--	--	--	--	--
TR39793|c3_g1_i1	No	(AG)7	14	--	--	--	--	--	--	GTTGGGTTCAGTCTTGCACA	60	TCAACAGGAAACCCTAACGG	60	129	--	--	--	--	--	--
TR39793|c3_g1_i6	No	(AG)7	14	--	--	--	--	--	--	GTTGGGTTCAGTCTTGCACA	60	TCAACAGGAAACCCTAACGG	60	129	--	--	--	--	--	--
TR39793|c4_g1_i1	No	(GA)7tgggaaaggcttctatggttgtt(AG)6	49	--	--	--	--	--	--	GCAAAAAGAAGCAAGAACGC	60	CGTGAGCGAATCCTCATCCT	63	255	--	--	--	--	--	--
TR39793|c4_g1_i2	No	(GA)7tgggaaaggcttctatggttgtt(AG)6	49	--	--	--	--	--	--	TGGTTGCCAAGTGAGTGTGT	60	AACATTCGTTCTTTTTGCCG	60	117	--	--	--	--	--	--
TR39793|c4_g3_i1	No	(GA)7tgggaaaggcttctatggttgtt(AG)6	49	--	--	--	--	--	--	GTGTTATAGGCGCTGCTGGT	60	AACATTCGTTCTTTTTGCCG	60	199	--	--	--	--	--	--

Transcript IDs	Differentially expressed gene (DEG) IDs	SSR repeat-motifs	SSR repeat-length (bp)	*In silico* polymorphic SSRs						Forward primers (5'-3')	Annealing tempearture(0C)	Reverse primers (5'-3')	Annealing tempearture(0C)	Amplified product size (bp)	Structural annotation		Functional Annotation			
				C. microphyllum		ICC4958									Gene IDs	Sequence component of genes	NCBI nr IDs	TFs	KOG	KEGG
				Transcript	SSR repeat-motifs	Chromosomes	SSR repeat-motifs	Start physical positions (bp)	End physical positions (bp)											
TR39794|c0_g2_i1	XLOC_014901	(AGA)6	18	--	--	--	--	--	--	CTCCTCAGTCGTGCAACAAA	60	CTTCGCCATGTTCCTCTTTC	60	217	--	--	--	--	--	--
TR39799|c4_g1_i1	No	(AAG)5	15	--	--	--	--	--	--	AACGACGGCTTTTTCACAAC	60	CAGATTCCTCTTCCATTTTTCA	58	207	--	--	--	--	--	--
TR39799|c4_g1_i14	No	(AAG)5	15	--	--	--	--	--	--	AACGACGGCTTTTTCACAAC	60	CAGATTCCTCTTCCATTTTTCA	58	207	--	--	--	--	--	--
TR39799|c4_g1_i15	No	(AAG)5	15	--	--	--	--	--	--	AACGACGGCTTTTTCACAAC	60	CAGATTCCTCTTCCATTTTTCA	58	207	--	--	--	--	--	--
TR39799|c4_g1_i17	No	(AAG)5	15	--	--	--	--	--	--	AACGACGGCTTTTTCACAAC	60	CAGATTCCTCTTCCATTTTTCA	58	207	--	--	--	--	--	--
TR39799|c4_g1_i20	No	(AAG)5	15	--	--	--	--	--	--	AACGACGGCTTTTTCACAAC	60	CAGATTCCTCTTCCATTTTTCA	58	207	--	--	--	--	--	--
TR39799|c4_g1_i21	No	(AAG)5	15	--	--	--	--	--	--	AACGACGGCTTTTTCACAAC	60	CAGATTCCTCTTCCATTTTTCA	58	207	--	--	--	--	--	--
TR39799|c4_g1_i22	No	(AAG)5	15	--	--	--	--	--	--	AACGACGGCTTTTTCACAAC	60	CAGATTCCTCTTCCATTTTTCA	58	207	--	--	--	--	--	--
TR3980|c0_g1_i1	No	(A)10tgggttgagaagggggaatgcgggtttcgtgcttctcggaattgcggaacggttactgtcgcacggaacgaaataatt(AG)8	104	--	--	--	--	--	--	TTGGGAGAGAGTTCGCGTAG	61	GCAACCGCAAAGTTTCATTT	60	187	--	--	--	--	--	--
TR39859|c0_g1_i1	No	(AAG)5	15	--	--	--	--	--	--	CCCAAGAACGACAAGGAGAA	60	GCAACTGCTCACAACTCGTC	60	161	--	--	--	--	--	--
TR39890|c0_g1_i1	No	(GAT)6	18	--	--	--	--	--	--	CAATGATGACAGCCATGGAG	60	GCAGCTGATGGAAACCTCAT	60	103	--	--	--	--	--	--
TR39920|c0_g1_i1	XLOC_015009	(CAA)5	15	--	--	--	--	--	--	ACAGCCAACAACACCAACAA	60	ATTCCATCTCCATTCCGTCA	60	206	--	--	--	--	--	--
TR39980|c1_g3_i1	XLOC_015033	(GTT)6	18	--	--	--	--	--	--	TTGGTCTTAGGGTGAGGGTG	60	CAATGCTTCTCTCCACCACA	60	111	--	--	--	--	--	--
TR39980|c2_g1_i1	XLOC_015034	(CAAAT)5	25	--	--	--	--	--	--	CCCAAACAATGAATCCTGCT	60	AAATGTCCCTTGTTCTACGCA	60	183	--	--	--	--	--	--

<table>
<tr><th rowspan="3">Transcript IDs</th><th rowspan="3">Differentially expressed gene (DEG) IDs</th><th rowspan="3">SSR repeat-motifs</th><th rowspan="3">SSR repeat-length (bp)</th><th colspan="6">In silico polymorphic SSRs</th><th rowspan="3">Forward primers (5'-3')</th><th rowspan="3">Annealing tempearture(0C)</th><th rowspan="3">Reverse primers (5'-3')</th><th rowspan="3">Annealing tempearture(0C)</th><th rowspan="3">Amplified product size (bp)</th><th colspan="2">Structural annotation</th><th colspan="4">Functional Annotation</th></tr>
<tr><th colspan="2">C. microphyllum</th><th colspan="4">ICC4958</th><th rowspan="2">Gene IDs</th><th rowspan="2">Sequence component of genes</th><th rowspan="2">NCBI nr IDs</th><th rowspan="2">TFs</th><th rowspan="2">KOG</th><th rowspan="2">KEGG</th></tr>
<tr><th>Transcript</th><th>SSR repeat-motifs</th><th>Chromosomes</th><th>SSR repeat-motifs</th><th>Start physical positions (bp)</th><th>End physical positions (bp)</th></tr>
<tr><td>TR39987|c2_g1_i1</td><td>XLOC_015061</td><td>(CTT)7</td><td>21</td><td>--</td><td>--</td><td>--</td><td>--</td><td>--</td><td>--</td><td>TCACACTCTGCTTTGGTTGG</td><td>60</td><td>ACAAGGTGCTTGAAACCGTC</td><td>60</td><td>213</td><td>--</td><td>--</td><td>--</td><td>--</td><td>--</td><td>--</td></tr>
<tr><td>TR39989|c1_g2_i1</td><td>No</td><td>(TTC)6</td><td>18</td><td>--</td><td>--</td><td>--</td><td>--</td><td>--</td><td>--</td><td>GGAAAAGTTGAGCCCAAAAA</td><td>59</td><td>TCATTGTAAAGGTAGATGGCTCA</td><td>59</td><td>271</td><td>--</td><td>--</td><td>--</td><td>--</td><td>--</td><td>--</td></tr>
<tr><td>TR39989|c1_g2_i2</td><td>No</td><td>(TTC)6</td><td>18</td><td>--</td><td>--</td><td>--</td><td>--</td><td>--</td><td>--</td><td>GGAAAAGTTGAGCCCAAAAA</td><td>59</td><td>TCATTGTAAAGGTAGATGGCTCA</td><td>59</td><td>271</td><td>--</td><td>--</td><td>--</td><td>--</td><td>--</td><td>--</td></tr>
<tr><td>TR39989|c3_g2_i1</td><td>No</td><td>(ATC)10</td><td>30</td><td>--</td><td>--</td><td>--</td><td>--</td><td>--</td><td>--</td><td>CGTGGAACTCAGACTACCCCT</td><td>61</td><td>GAGCATCATAAGCGACGTGA</td><td>60</td><td>271</td><td>--</td><td>--</td><td>--</td><td>--</td><td>--</td><td>--</td></tr>
<tr><td>TR39989|c3_g2_i4</td><td>No</td><td>(ATC)10</td><td>30</td><td>--</td><td>--</td><td>--</td><td>--</td><td>--</td><td>--</td><td>CGTGGAACTCAGACTACCCCT</td><td>61</td><td>GAGCATCATAAGCGACGTGA</td><td>60</td><td>271</td><td>--</td><td>--</td><td>--</td><td>--</td><td>--</td><td>--</td></tr>
<tr><td>TR39993|c0_g2_i1</td><td>No</td><td>(CT)9</td><td>18</td><td>--</td><td>--</td><td>--</td><td>--</td><td>--</td><td>--</td><td>ACCTTCCTCCGTTCATTCCT</td><td>60</td><td>TGCTAATCATCCCAAGTCCC</td><td>60</td><td>250</td><td>--</td><td>--</td><td>--</td><td>--</td><td>--</td><td>--</td></tr>
<tr><td>TR39993|c0_g2_i2</td><td>No</td><td>(CT)9</td><td>18</td><td>--</td><td>--</td><td>--</td><td>--</td><td>--</td><td>--</td><td>ACCTTCCTCCGTTCATTCCT</td><td>60</td><td>TGCTAATCATCCCAAGTCCC</td><td>60</td><td>250</td><td>--</td><td>--</td><td>--</td><td>--</td><td>--</td><td>--</td></tr>
<tr><td>TR39993|c0_g2_i5</td><td>No</td><td>(CT)9</td><td>18</td><td>--</td><td>--</td><td>--</td><td>--</td><td>--</td><td>--</td><td>ACCTTCCTCCGTTCATTCCT</td><td>60</td><td>CTTGAGATTGACCTTTCGGC</td><td>60</td><td>277</td><td>--</td><td>--</td><td>--</td><td>--</td><td>--</td><td>--</td></tr>
<tr><td>TR39993|c0_g2_i6</td><td>No</td><td>(CT)9</td><td>18</td><td>--</td><td>--</td><td>--</td><td>--</td><td>--</td><td>--</td><td>ACCTTCCTCCGTTCATTCCT</td><td>60</td><td>CTTGAGATTGACCTTTCGGC</td><td>60</td><td>277</td><td>--</td><td>--</td><td>--</td><td>--</td><td>--</td><td>--</td></tr>
<tr><td>TR39993|c1_g1_i11</td><td>No</td><td>(CT)8caactccctcaaatcaaaaagcccatccatcgct(TTC)10catctatccaaaccccaacaaatcaaattc(CT)10</td><td>130</td><td>--</td><td>--</td><td>--</td><td>--</td><td>--</td><td>--</td><td>CAGATCAGATCCCTCTCCCA</td><td>60</td><td>GAGTTCGCGTCCAAATGAAT</td><td>60</td><td>266</td><td>--</td><td>--</td><td>--</td><td>--</td><td>--</td><td>--</td></tr>
<tr><td>TR39993|c1_g1_i2</td><td>No</td><td>(CT)8caactccctcaaatcaaaaagcccatccatcgct(TTC)10catctatccaaaccccaacaaatcaaattc(CT)10</td><td>130</td><td>--</td><td>--</td><td>--</td><td>--</td><td>--</td><td>--</td><td>CAGATCAGATCCCTCTCCCA</td><td>60</td><td>GAGTTCGCGTCCAAATGAAT</td><td>60</td><td>266</td><td>--</td><td>--</td><td>--</td><td>--</td><td>--</td><td>--</td></tr>
<tr><td>TR39993|c1_g1_i4</td><td>No</td><td>(CT)8caactccctcaaatcaaaaagcccatccatcgct(TTC)10catctatccaaaccccaacaaatcaaattc(CT)10</td><td>130</td><td>--</td><td>--</td><td>--</td><td>--</td><td>--</td><td>--</td><td>CAGATCAGATCCCTCTCCCA</td><td>60</td><td>GAGTTCGCGTCCAAATGAAT</td><td>60</td><td>266</td><td>--</td><td>--</td><td>--</td><td>--</td><td>--</td><td>--</td></tr>
<tr><td>TR39993|c1_g1_i5</td><td>No</td><td>(CT)8caactccctcaaatcaaaaagcccatccatcgct(TTC)10catctatccaaaccccaacaaatcaaattc(CT)10</td><td>130</td><td>--</td><td>--</td><td>--</td><td>--</td><td>--</td><td>--</td><td>CAGATCAGATCCCTCTCCCA</td><td>60</td><td>GAGTTCGCGTCCAAATGAAT</td><td>60</td><td>266</td><td>--</td><td>--</td><td>--</td><td>--</td><td>--</td><td>--</td></tr>
<tr><td>TR39993|c1_g1_i7</td><td>No</td><td>(CT)8caactccctcaaatcaaaaagcccatccatcgct(TTC)10catctatccaaaccccaacaaatcaaattc(CT)10</td><td>130</td><td>--</td><td>--</td><td>--</td><td>--</td><td>--</td><td>--</td><td>CAGATCAGATCCCTCTCCCA</td><td>60</td><td>GAGTTCGCGTCCAAATGAAT</td><td>60</td><td>266</td><td>--</td><td>--</td><td>--</td><td>--</td><td>--</td><td>--</td></tr>
</table>

Transcript IDs	Differentially expressed gene (DEG) IDs	SSR repeat-motifs	SSR repeat-length (bp)	*In silico* polymorphic SSRs						Forward primers (5'-3')	Annealing tempearture(0C)	Reverse primers (5'-3')	Annealing tempearture(0C)	Amplified product size (bp)	Structural annotation		Functional Annotation			
				C. microphyllum		ICC4958									Gene IDs	Sequence component of genes	NCBI nr IDs	TFs	KOG	KEGG
				Transcript	SSR repeat-motifs	Chromosomes	SSR repeat-motifs	Start physical positions (bp)	End physical positions (bp)											
TR39993|c1_g1_i9	No	(CT)8caactccctcaaatcaaaaagcccatccatcgct(TTC)10catctatccaaaccccaacaaatcaaattc(CT)10	130	--	--	--	--	--	--	CAGATCAGATCCCTCTCCCA	60	GAGTTCGCGTCCAAATGAAT	60	266	--	--	--	--	--	--
TR39998|c0_g3_i1	No	(CTT)5	15	--	--	--	--	--	--	CGTTCCTTCGGTCTCTTCAC	60	GGATGGTTCGAGGATTGAGA	60	205	--	--	--	--	--	--
TR39998|c0_g3_i2	No	(CTT)5	15	--	--	--	--	--	--	CGTTCCTTCGGTCTCTTCAC	60	GGATGGTTCGAGGATTGAGA	60	205	--	--	--	--	--	--
TR39998|c0_g3_i3	No	(CTT)5	15	--	--	--	--	--	--	CGTTCCTTCGGTCTCTTCAC	60	GGATGGTTCGAGGATTGAGA	60	205	--	--	--	--	--	--
TR39999|c0_g1_i2	No	(AACCA)5	25	--	--	--	--	--	--	ACGCGACGCAAACAGGTAG	62	GGAATGTCGGTGGAAGGATA	60	150	--	--	--	--	--	--
TR39999|c0_g1_i2	No	(CT)8	16	--	--	--	--	--	--	CCGCGTTTCTTCTTCTCTCTT	60	CATCATTCATTTCAACGCCT	59	260	--	--	--	--	--	--
TR39999|c0_g1_i3	No	(AACCA)5	25	--	--	--	--	--	--	ACGCGACGCAAACAGGTAG	62	CACGGTGTGGCTTATCAGTG	60	241	--	--	--	--	--	--
TR40000|c2_g2_i5	XLOC_015078	(TC)6	12	TR40000|c2_g2_i5	(TC)6	Ca_LG_1	(TC)10	2940723	2940742	AGCGTAAGGGGAGGAGAGAG	60	CGATCGGATCAAAACCCTAA	60	249	Ca_03764	URR	--	--	--	--
TR40015|c0_g1_i1	No	(AG)6	12	--	--	--	--	--	--	TGAACCTTGTTAGTTCTCATTTTCA	59	TAGCTCTCAGCCCAGCCTTA	60	213	--	--	--	--	--	--
TR40015|c0_g1_i2	No	(AG)6aagcaactctcacgattccaactgcatcgcaatcattcatattctatttccatcgatgctgcttcacgttttcaatcaactcaaggtttgaccaat(AAC)6	126	--	--	--	--	--	--	TGAACCTTGTTAGTTCTCATTTTCA	59	TAAGCCCTAGCGATCTCGAA	60	271	--	--	--	--	--	--
TR40015|c0_g1_i3	No	(AG)6	12	--	--	--	--	--	--	TGAACCTTGTTAGTTCTCATTTTCA	59	TAGCTCTCAGCCCAGCCTTA	60	213	--	--	--	--	--	--
TR40015|c0_g1_i4	No	(AG)6aagcaactctcacgattccaactgcatcgcaatcattcatattctatttccatcgatgctgcttcacgttttcaatcaactcaaggtttgaccaat(AAC)6	126	--	--	--	--	--	--	TGAACCTTGTTAGTTCTCATTTTCA	59	TAAGCCCTAGCGATCTCGAA	60	271	--	--	--	--	--	--
TR40024|c0_g1_i1	No	(TGG)6	18	--	--	--	--	--	--	AACCGTGGACCTACACAACC	60	TCCTGATCTGCAAAGGGATT	60	206	--	--	--	--	--	--
TR40024|c0_g1_i2	No	(TGG)6	18	--	--	--	--	--	--	TTTCTGACAACGTTGGATCG	60	TCCTGATCTGCAAAGGGATT	60	139	--	--	--	--	--	--

Transcript IDs	Differentially expressed gene (DEG) IDs	SSR repeat-motifs	SSR repeat-length (bp)	*In silico* polymorphic SSRs						Forward primers (5'-3')	Annealing tempearture(0C)	Reverse primers (5'-3')	Annealing tempearture(0C)	Amplified product size (bp)	Structural annotation		Functional Annotation			
				C. microphyllum		ICC4958									Gene IDs	Sequence component of genes	NCBI nr IDs	TFs	KOG	KEGG
				Transcript	SSR repeat-motifs	Chromosomes	SSR repeat-motifs	Start physical positions (bp)	End physical positions (bp)											
TR40024|c0_g1_i4	No	(TGG)6	18	--	--	--	--	--	--	AACCGTGGACCTACACAACC	60	TCCTGATCTGCAAAGGGATT	60	206	--	--	--	--	--	--
TR40024|c0_g1_i9	No	(TGG)6	18	--	--	--	--	--	--	TTTCTGACAACGTTGGATCG	60	TCCTGATCTGCAAAGGGATT	60	139	--	--	--	--	--	--
TR40026|c0_g1_i1	No	(TGA)8	24	--	--	--	--	--	--	CTCGTGACCCAATTGAAGGT	60	TTCGATTTCAGGAGGATTGG	60	212	--	--	--	--	--	--
TR40026|c0_g1_i2	No	(TGA)8	24	--	--	--	--	--	--	CTCGTGACCCAATTGAAGGT	60	TTCGATTTCAGGAGGATTGG	60	212	--	--	--	--	--	--
TR40026|c1_g3_i1	XLOC_015137	(ATC)5	15	--	--	--	--	--	--	TCATCCGCTGTCTCCCTAAC	60	GGTTTCCTCAACTGCACCAT	60	105	--	--	--	--	--	--
TR40031|c2_g1_i1	No	(ATA)6	18	--	--	--	--	--	--	TACTGACGACAACGACGCTC	60	ATCATTATGGGGATGGGGAT	60	137	--	--	--	--	--	--
TR40031|c2_g1_i2	No	(ATA)6	18	--	--	--	--	--	--	TACTGACGACAACGACGCTC	60	ATCATTATGGGGATGGGGAT	60	137	--	--	--	--	--	--
TR40040|c0_g5_i14	No	(AGA)5	15	--	--	--	--	--	--	CCGAAAAGGAAGCTTCAACA	60	AAAGGCTCTCACAAGACGGA	60	250	--	--	--	--	--	--
TR40040|c0_g5_i4	No	(AGA)5	15	--	--	--	--	--	--	CCGAAAAGGAAGCTTCAACA	60	AAAGGCTCTCACAAGACGGA	60	250	--	--	--	--	--	--
TR40041|c3_g1_i1	No	(AC)7	14	--	--	--	--	--	--	AGACCAAATGCATGGTGCTT	61	TGGACTTGAAGGAGGATGAGA	60	205	--	--	--	--	--	--
TR40041|c3_g1_i2	No	(AC)7	14	--	--	--	--	--	--	AGACCAAATGCATGGTGCTT	61	TGGACTTGAAGGAGGATGAGA	60	205	--	--	--	--	--	--
TR40046|c10_g2_i1	No	(ATA)7	21	--	--	--	--	--	--	TATCAGTGCTTCCTCGCCTT	60	GCTTTCCTTTGATCACGAGC	60	259	--	--	--	--	--	--
TR40046|c10_g2_i11	No	(ATA)7	21	--	--	--	--	--	--	TATCAGTGCTTCCTCGCCTT	60	GCTTTCCTTTGATCACGAGC	60	259	--	--	--	--	--	--
TR40046|c10_g2_i12	No	(ATA)7	21	--	--	--	--	--	--	TATCAGTGCTTCCTCGCCTT	60	GCTTTCCTTTGATCACGAGC	60	240	--	--	--	--	--	--

Transcript IDs	Differentially expressed gene (DEG) IDs	SSR repeat-motifs	SSR repeat-length (bp)	*In silico* polymorphic SSRs						Forward primers (5'-3')	Annealing tempearture(0C)	Reverse primers (5'-3')	Annealing tempearture(0C)	Amplified product size (bp)	Structural annotation		Functional Annotation			
				C. microphyllum		ICC4958									Gene IDs	Sequence component of genes	NCBI nr IDs	TFs	KOG	KEGG
				Transcript	SSR repeat-motifs	Chromosomes	SSR repeat-motifs	Start physical positions (bp)	End physical positions (bp)											
TR40046|c10_g2_i2	No	(ATA)7	21	--	--	--	--	--	--	TATCAGTGCTTCCTCGCCTT	60	GCTTTCCTTTGATCACGAGC	60	259	--	--	--	--	--	--
TR40046|c10_g2_i6	No	(ATA)7	21	--	--	--	--	--	--	TATCAGTGCTTCCTCGCCTT	60	GCTTTCCTTTGATCACGAGC	60	259	--	--	--	--	--	--
TR40046|c10_g2_i8	No	(ATA)7	21	--	--	--	--	--	--	TATCAGTGCTTCCTCGCCTT	60	GCTTTCCTTTGATCACGAGC	60	259	--	--	--	--	--	--
TR40046|c2_g1_i1	No	(AG)6aatgaaattac(TAT)7	44	--	--	--	--	--	--	CACAAAAGGGGTAAGTGGTCA	60	CGCATTCAACTCATTCGTGT	60	232	--	--	--	--	--	--
TR40046|c2_g2_i1	No	(AG)6aatgaaattac(TAT)7	44	--	--	--	--	--	--	CACAAAAGGGGTAAGTGGTCA	60	CGCATTCAACTCATTCGTGT	60	232	--	--	--	--	--	--
TR40046|c3_g1_i1	No	(ATA)5	15	--	--	--	--	--	--	AGGTACAAATTGCCTGTGGC	60	GAATGTGATTCTGACGCGAT	59	262	--	--	--	--	--	--
TR40046|c3_g2_i1	No	(ATA)5	15	--	--	--	--	--	--	AGGTACAAATTGCCTGTGGC	60	GAATGTGATTCTGACGCGAT	59	262	--	--	--	--	--	--
TR40046|c8_g2_i1	No	(TTC)7	21	--	--	--	--	--	--	GACCCCTCTCCTCATTCCTC	60	ATGAGAAGGTTGGTGGATCG	60	227	--	--	--	--	--	--
TR40046|c8_g2_i2	No	(TTC)7	21	--	--	--	--	--	--	GACCCCTCTCCTCATTCCTC	60	ATGAGAAGGTTGGTGGATCG	60	227	--	--	--	--	--	--
TR40049|c3_g3_i6	XLOC_015229	(TTAT)5	20	--	--	--	--	--	--	GCAGGGTTCCTTGTTTGACT	59	AAAACCTGCAAACGAGATGC	60	179	--	--	--	--	--	--
TR40053|c1_g1_i3	XLOC_015239	(GGT)5	15	--	--	--	--	--	--	CGAGGACCTTGCTAACCTTG	60	TCACCGGATTCTTTCGATTC	60	239	--	--	--	--	--	--
TR40053|c1_g1_i4	No	(GGT)5	15	--	--	--	--	--	--	CGAGGACCTTGCTAACCTTG	60	CCAAGGGCGATAAAAACTCA	60	229	--	--	--	--	--	--
TR40062|c2_g2_i1	XLOC_015258	(GA)6	12	--	--	--	--	--	--	GCTTAATCCATTCCCCCATT	60	ACAGATTTGCCAGCTTCCAT	60	194	--	--	--	--	--	--
TR40062|c2_g2_i2	No	(GA)6	12	--	--	--	--	--	--	GCTTAATCCATTCCCCCATT	60	ACAGATTTGCCAGCTTCCAT	60	194	--	--	--	--	--	--

Transcript IDs	Differentially expressed gene (DEG) IDs	SSR repeat-motifs	SSR repeat-length (bp)	*In silico* polymorphic SSRs						Forward primers (5'-3')	Annealing tempearture(0C)	Reverse primers (5'-3')	Annealing tempearture(0C)	Amplified product size (bp)	Structural annotation		Functional Annotation			
				C. microphyllum		ICC4958									Gene IDs	Sequence component of genes	NCBI nr IDs	TFs	KOG	KEGG
				Transcript	SSR repeat-motifs	Chromosomes	SSR repeat-motifs	Start physical positions (bp)	End physical positions (bp)											
TR40063|c1_g2_i1	No	(AC)8	16	--	--	--	--	--	--	CTGTTGCAATTCTTCTGGCA	60	TGGGACACGTCAGATTTCAA	60	237	--	--	--	--	--	--
TR40068|c2_g1_i2	XLOC_015286	(TTC)5	15	--	--	--	--	--	--	ACCGAGCTTACCCAACTCCT	60	ATGTTTCAGGTTGATTCGCC	60	161	--	--	--	--	--	--
TR40072|c1_g4_i1	No	(TC)7	14	--	--	--	--	--	--	CACACGGGACAGTATTGCAC	60	TGGTTTGAGATTGAAAACGAAA	60	249	--	--	--	--	--	--
TR40072|c1_g4_i2	No	(TC)7	14	--	--	--	--	--	--	CACACGGGACAGTATTGCAC	60	TGGTTTGAGATTGAAAACGAAA	60	249	--	--	--	--	--	--
TR40078|c0_g2_i1	XLOC_015304	(AAC)5	15	--	--	--	--	--	--	TTTCACATGGCTTCCAACAA	60	TGAGAGGGGTGTGATTGTGA	60	188	--	--	--	--	--	--
TR40099|c0_g1_i1	XLOC_015312	(TGG)5(TGA)6	33	--	--	--	--	--	--	ATGGAGAAAGAAGAAAATGATGA	57	AAAGCGCAATTAATCCGTGT	60	200	--	--	--	--	--	--
TR4012|c0_g1_i1	XLOC_015327	(ACA)5	15	--	--	--	--	--	--	CACCCATATGATCCTGTCCA	59	GCTTCCATTTGGGTGAGCTA	60	143	--	--	--	--	--	--
TR40142|c0_g1_i1	XLOC_015336	(GT)6	12	--	--	--	--	--	--	CTTGCTGGAAACAATCCACA	60	TCAAACTTCACTTCACTTCACTCC	60	271	--	--	--	--	--	--
TR40177|c0_g1_i1	XLOC_015347	(AAG)6	18	--	--	--	--	--	--	TTCGGGAAAGATCATGTTGA	59	CTGGGACACACCCGTTTTAC	60	270	--	--	XP_003630173.1	--	--	--
TR40210|c0_g1_i1	XLOC_015363	(ATC)5	15	--	--	--	--	--	--	TCCTTCCCAAGGACTGTTTC	59	CGTCTTTGCTCCCTTCTTTG	60	268	--	--	--	--	--	--
TR4042|c0_g1_i1	XLOC_015416	(AC)8(AT)8	32	--	--	--	--	--	--	GAGATTGGAACAATACAAAAATAT GA	58	TGAAAGCCATACAACCTCCTC	59	198	--	--	--	--	--	--
TR40487|c0_g1_i1	No	(GA)7	14	--	--	--	--	--	--	TCTTCCAAAGATTGCTTGAATG	59	CAAAGAACAGTGACACACTGCAT	60	122	--	--	--	--	--	--
TR40542|c0_g1_i1	No	(CTT)5gggctttc(TCT)5	38	--	--	--	--	--	--	CTTGTTAGGGAACAGCTGGC	60	GATTCTGAAGACGAAGCCGA	60	159	--	--	--	--	--	--
TR40580|c0_g1_i1	XLOC_015475	(AGT)5	15	--	--	--	--	--	--	CAGAGACATGCACACGACAA	59	TCCGACAAGGTTTCAGCTTC	60	228	--	--	--	--	--	--

<table>
<tr><th rowspan="3">Transcript IDs</th><th rowspan="3">Differentially expressed gene (DEG) IDs</th><th rowspan="3">SSR repeat-motifs</th><th rowspan="3">SSR repeat-length (bp)</th><th colspan="6">In silico polymorphic SSRs</th><th rowspan="3">Forward primers (5'-3')</th><th rowspan="3">Annealing tempearture(0C)</th><th rowspan="3">Reverse primers (5'-3')</th><th rowspan="3">Annealing tempearture(0C)</th><th rowspan="3">Amplified product size (bp)</th><th colspan="2">Structural annotation</th><th colspan="4">Functional Annotation</th></tr>
<tr><th colspan="2">C. microphyllum</th><th colspan="4">ICC4958</th><th rowspan="2">Gene IDs</th><th rowspan="2">Sequence component of genes</th><th rowspan="2">NCBI nr IDs</th><th rowspan="2">TFs</th><th rowspan="2">KOG</th><th rowspan="2">KEGG</th></tr>
<tr><th>Transcript</th><th>SSR repeat-motifs</th><th>Chromosomes</th><th>SSR repeat-motifs</th><th>Start physical positions (bp)</th><th>End physical positions (bp)</th></tr>
<tr><td>TR40624|c0_g1_i1</td><td>No</td><td>(GA)6</td><td>12</td><td>--</td><td>--</td><td>--</td><td>--</td><td>--</td><td>--</td><td>GAATTGGGCTAAAGAAATCGG</td><td>60</td><td>AATTCATGCTTGTGAACTCCA</td><td>58</td><td>262</td><td>--</td><td>--</td><td>--</td><td>--</td><td>--</td><td>--</td></tr>
<tr><td>TR40624|c0_g2_i1</td><td>No</td><td>(GA)6</td><td>12</td><td>--</td><td>--</td><td>--</td><td>--</td><td>--</td><td>--</td><td>GAATTGGGCTAAAGAAATCGG</td><td>60</td><td>AATTCATGCTTGTGAACTCCA</td><td>58</td><td>262</td><td>--</td><td>--</td><td>--</td><td>--</td><td>--</td><td>--</td></tr>
<tr><td>TR40871|c0_g1_i1</td><td>No</td><td>(AT)9</td><td>18</td><td>--</td><td>--</td><td>--</td><td>--</td><td>--</td><td>--</td><td>CGATCACTTTCCGGCATAAT</td><td>60</td><td>TGTTCAATGAAAATTAAACACACA A</td><td>59</td><td>197</td><td>--</td><td>--</td><td>--</td><td>--</td><td>--</td><td>--</td></tr>
<tr><td>TR40901|c0_g1_i1</td><td>XLOC_015588</td><td>(TC)6</td><td>12</td><td>--</td><td>--</td><td>--</td><td>--</td><td>--</td><td>--</td><td>GCGTACCAAAGGCTTCCATA</td><td>60</td><td>CACATCATGCACTTCAGGCT</td><td>60</td><td>237</td><td>--</td><td>--</td><td>--</td><td>--</td><td>--</td><td>--</td></tr>
<tr><td>TR41080|c0_g1_i1</td><td>XLOC_015613</td><td>(ATAC)5</td><td>20</td><td>--</td><td>--</td><td>--</td><td>--</td><td>--</td><td>--</td><td>CCGTTGAAGTGAAGCAATCA</td><td>60</td><td>TCACTGGTGTCTGAAAGTGAGC</td><td>60</td><td>229</td><td>--</td><td>--</td><td>XP_004503031.1</td><td>--</td><td>[X]</td><td>--</td></tr>
<tr><td>TR41114|c0_g1_i1</td><td>No</td><td>(GT)9</td><td>18</td><td>--</td><td>--</td><td>--</td><td>--</td><td>--</td><td>--</td><td>TCTGAGCTTTACCCCAGAAGA</td><td>59</td><td>TCATGACGACAGGACACTTCA</td><td>60</td><td>280</td><td>--</td><td>--</td><td>--</td><td>--</td><td>--</td><td>--</td></tr>
<tr><td>TR41116|c0_g1_i1</td><td>XLOC_015630</td><td>(ACAGAC)5</td><td>30</td><td>--</td><td>--</td><td>--</td><td>--</td><td>--</td><td>--</td><td>AGCGTGCATACGATGAGTTG</td><td>60</td><td>AATACGATGGCGACAAGACC</td><td>60</td><td>206</td><td>--</td><td>--</td><td>--</td><td>--</td><td>--</td><td>--</td></tr>
<tr><td>TR41315|c0_g1_i1</td><td>XLOC_015665</td><td>(GT)11</td><td>22</td><td>--</td><td>--</td><td>--</td><td>--</td><td>--</td><td>--</td><td>TGCTTCCTTGTTTGTATGTAGACAG</td><td>60</td><td>TCATTAAGGGACGAGGGAAA</td><td>60</td><td>116</td><td>--</td><td>--</td><td>--</td><td>--</td><td>--</td><td>--</td></tr>
<tr><td>TR41322|c0_g1_i1</td><td>No</td><td>(A)10taggaaacctatcatgcctgctcgaaaaaaag(A) 12</td><td>54</td><td>--</td><td>--</td><td>--</td><td>--</td><td>--</td><td>--</td><td>CCCCAAGGGTAGAGCACATA</td><td>60</td><td>TTCAACTTCGAGAAGCCGTT</td><td>60</td><td>269</td><td>--</td><td>--</td><td>--</td><td>--</td><td>--</td><td>--</td></tr>
<tr><td>TR41322|c0_g2_i1</td><td>No</td><td>(A)10taggaaacctatcatgcctgctcgaaaaaaag(A) 12</td><td>54</td><td>--</td><td>--</td><td>--</td><td>--</td><td>--</td><td>--</td><td>CCCCAAGGGTAGAGCACATA</td><td>60</td><td>TTCAACTTCGAGAAGCCGTT</td><td>60</td><td>269</td><td>--</td><td>--</td><td>--</td><td>--</td><td>--</td><td>--</td></tr>
<tr><td>TR4170|c0_g1_i1</td><td>No</td><td>(AT)6</td><td>12</td><td>--</td><td>--</td><td>--</td><td>--</td><td>--</td><td>--</td><td>TCCTTATACCAGCTTCAATTCACA</td><td>60</td><td>GACCTGCTCCCTTGTCTCTG</td><td>60</td><td>260</td><td>--</td><td>--</td><td>--</td><td>--</td><td>--</td><td>--</td></tr>
<tr><td>TR4170|c0_g2_i1</td><td>No</td><td>(AT)6</td><td>12</td><td>--</td><td>--</td><td>--</td><td>--</td><td>--</td><td>--</td><td>TCCTTATACCAGCTTCAATTCACA</td><td>60</td><td>GACCTGCTCCCTTGTCTCTG</td><td>60</td><td>260</td><td>--</td><td>--</td><td>--</td><td>--</td><td>--</td><td>--</td></tr>
<tr><td>TR41755|c0_g1_i1</td><td>No</td><td>(ATG)6</td><td>18</td><td>--</td><td>--</td><td>--</td><td>--</td><td>--</td><td>--</td><td>CTCAAGCACAGCCAAATGAA</td><td>60</td><td>GGCTGTCGAATTTTCGCTAC</td><td>60</td><td>131</td><td>--</td><td>--</td><td>--</td><td>--</td><td>--</td><td>--</td></tr>
<tr><td>TR41797|c0_g1_i1</td><td>No</td><td>(GAC)5</td><td>15</td><td>--</td><td>--</td><td>--</td><td>--</td><td>--</td><td>--</td><td>TCTACCAGCAGTGGTTCCGT</td><td>61</td><td>CGGCAAATCCCAAAGACTAA</td><td>60</td><td>189</td><td>--</td><td>--</td><td>--</td><td>--</td><td>--</td><td>--</td></tr>
</table>

<table>
<thead>
<tr><th rowspan="3">Transcript IDs</th><th rowspan="3">Differentially expressed gene (DEG) IDs</th><th rowspan="3">SSR repeat-motifs</th><th rowspan="3">SSR repeat-length (bp)</th><th colspan="6">In silico polymorphic SSRs</th><th rowspan="3">Forward primers (5'-3')</th><th rowspan="3">Annealing tempearture(0C)</th><th rowspan="3">Reverse primers (5'-3')</th><th rowspan="3">Annealing tempearture(0C)</th><th rowspan="3">Amplified product size (bp)</th><th colspan="2">Structural annotation</th><th colspan="4">Functional Annotation</th></tr>
<tr><th colspan="2">C. microphyllum</th><th colspan="4">ICC4958</th><th rowspan="2">Gene IDs</th><th rowspan="2">Sequence component of genes</th><th rowspan="2">NCBI nr IDs</th><th rowspan="2">TFs</th><th rowspan="2">KOG</th><th rowspan="2">KEGG</th></tr>
<tr><th>Transcript</th><th>SSR repeat-motifs</th><th>Chromosomes</th><th>SSR repeat-motifs</th><th>Start physical positions (bp)</th><th>End physical positions (bp)</th></tr>
</thead>
<tbody>
<tr><td>TR41862|c0_g1_i1</td><td>XLOC_015794</td><td>(AT)6acatacatagggttaggg(T)11</td><td>41</td><td>--</td><td>--</td><td>--</td><td>--</td><td>--</td><td>--</td><td>CACTTTCACGCTATTCTCCCA</td><td>60</td><td>TAAATTGGCAAGGCAAGAAA</td><td>58</td><td>100</td><td>--</td><td>--</td><td>--</td><td>--</td><td>--</td><td>--</td></tr>
<tr><td>TR4199|c0_g1_i1</td><td>XLOC_015827</td><td>(CA)6</td><td>12</td><td>--</td><td>--</td><td>--</td><td>--</td><td>--</td><td>--</td><td>TGGAAAACAAACAACCACAGA</td><td>59</td><td>TGCTAGGCGAAGAAACAGGT</td><td>60</td><td>255</td><td>--</td><td>--</td><td>--</td><td>--</td><td>--</td><td>--</td></tr>
<tr><td>TR42001|c0_g1_i1</td><td>XLOC_015830</td><td>(CAC)5</td><td>15</td><td>--</td><td>--</td><td>--</td><td>--</td><td>--</td><td>--</td><td>ATGGATACCCTCCCAAATCC</td><td>60</td><td>AATGAAGGATGAGGTGGCAG</td><td>60</td><td>209</td><td>--</td><td>--</td><td>XP_004506774.2</td><td>--</td><td>[C]</td><td>K08915</td></tr>
<tr><td>TR42006|c0_g1_i10</td><td>No</td><td>(AG)6</td><td>12</td><td>--</td><td>--</td><td>--</td><td>--</td><td>--</td><td>--</td><td>GAGCCTGAAGTTCCTCATTCA</td><td>59</td><td>CTCCACTGGCTGGGTTATGT</td><td>60</td><td>209</td><td>--</td><td>--</td><td>--</td><td>--</td><td>--</td><td>--</td></tr>
<tr><td>TR42006|c0_g1_i2</td><td>No</td><td>(AG)6</td><td>12</td><td>--</td><td>--</td><td>--</td><td>--</td><td>--</td><td>--</td><td>GAGCCTGAAGTTCCTCATTCA</td><td>59</td><td>CTCCACTGGCTGGGTTATGT</td><td>60</td><td>209</td><td>--</td><td>--</td><td>--</td><td>--</td><td>--</td><td>--</td></tr>
<tr><td>TR42006|c0_g1_i3</td><td>No</td><td>(AG)6</td><td>12</td><td>--</td><td>--</td><td>--</td><td>--</td><td>--</td><td>--</td><td>GAGCCTGAAGTTCCTCATTCA</td><td>59</td><td>CTCCACTGGCTGGGTTATGT</td><td>60</td><td>209</td><td>--</td><td>--</td><td>--</td><td>--</td><td>--</td><td>--</td></tr>
<tr><td>TR42006|c0_g1_i5</td><td>No</td><td>(AG)6</td><td>12</td><td>--</td><td>--</td><td>--</td><td>--</td><td>--</td><td>--</td><td>GAGCCTGAAGTTCCTCATTCA</td><td>59</td><td>CTCCACTGGCTGGGTTATGT</td><td>60</td><td>209</td><td>--</td><td>--</td><td>--</td><td>--</td><td>--</td><td>--</td></tr>
<tr><td>TR42019|c0_g2_i1</td><td>No</td><td>(TGTAA)5</td><td>25</td><td>--</td><td>--</td><td>--</td><td>--</td><td>--</td><td>--</td><td>CAAAAACCAAAAGGCAAGGA</td><td>60</td><td>CCTCTTCAACGCAAACCAGT</td><td>60</td><td>248</td><td>--</td><td>--</td><td>--</td><td>--</td><td>--</td><td>--</td></tr>
<tr><td>TR42019|c0_g2_i4</td><td>No</td><td>(TGTAA)5</td><td>25</td><td>--</td><td>--</td><td>--</td><td>--</td><td>--</td><td>--</td><td>CAAAAACCAAAAGGCAAGGA</td><td>60</td><td>CCTCTTCAACGCAAACCAGT</td><td>60</td><td>248</td><td>--</td><td>--</td><td>--</td><td>--</td><td>--</td><td>--</td></tr>
<tr><td>TR4202|c0_g2_i1</td><td>XLOC_015862</td><td>(CTC)7</td><td>21</td><td>--</td><td>--</td><td>--</td><td>--</td><td>--</td><td>--</td><td>CCTTCCTCCCTTCTCACACA</td><td>60</td><td>TTGAACGGAACCACCTCTTC</td><td>60</td><td>205</td><td>--</td><td>--</td><td>--</td><td>--</td><td>--</td><td>--</td></tr>
<tr><td>TR42032|c0_g1_i1</td><td>XLOC_015864</td><td>(CTT)5</td><td>15</td><td>--</td><td>--</td><td>--</td><td>--</td><td>--</td><td>--</td><td>TGAACCAACCAACGATTCCT</td><td>60</td><td>AAAAACCTCCTCCCAATGCT</td><td>60</td><td>131</td><td>--</td><td>--</td><td>--</td><td>--</td><td>--</td><td>--</td></tr>
<tr><td>TR42042|c1_g1_i1</td><td>No</td><td>(GT)6</td><td>12</td><td>--</td><td>--</td><td>--</td><td>--</td><td>--</td><td>--</td><td>GCTCGAACGTTTAGTTTCGC</td><td>60</td><td>CCAGACCAGACCACACACAC</td><td>60</td><td>214</td><td>--</td><td>--</td><td>--</td><td>--</td><td>--</td><td>--</td></tr>
<tr><td>TR42050|c3_g1_i1</td><td>No</td><td>(AAC)7</td><td>21</td><td>--</td><td>--</td><td>--</td><td>--</td><td>--</td><td>--</td><td>ACCAACAAGAACAAGCACCC</td><td>60</td><td>GGTGATGGAACAAGTGGCTT</td><td>60</td><td>209</td><td>--</td><td>--</td><td>--</td><td>--</td><td>--</td><td>--</td></tr>
<tr><td>TR42050|c3_g2_i1</td><td>XLOC_015888</td><td>(AAC)7</td><td>21</td><td>--</td><td>--</td><td>--</td><td>--</td><td>--</td><td>--</td><td>ACCAACAAGAACAAGCACCC</td><td>60</td><td>GGTGATGGAACAAGTGGCTT</td><td>60</td><td>209</td><td>--</td><td>--</td><td>XP_004508201.1</td><td>bHLH</td><td>[Z]</td><td>K10418</td></tr>
</tbody>
</table>

Transcript IDs	Differentially expressed gene (DEG) IDs	SSR repeat-motifs	SSR repeat-length (bp)	*In silico* polymorphic SSRs						Forward primers (5'-3')	Annealing tempearture(0C)	Reverse primers (5'-3')	Annealing tempearture(0C)	Amplified product size (bp)	Structural annotation		Functional Annotation			
				C. microphyllum		ICC4958									Gene IDs	Sequence component of genes	NCBI nr IDs	TFs	KOG	KEGG
				Transcript	SSR repeat-motifs	Chromosomes	SSR repeat-motifs	Start physical positions (bp)	End physical positions (bp)											
TR42050|c3_g2_i2	XLOC_015889	(AAC)7	21	--	--	--	--	--	--	ACCAACAAGAACAAGCACCC	60	GGTGATGGAACAAGTGGCTT	60	209	--	--	--	--	--	--
TR42050|c4_g1_i1	XLOC_015890	(GTT)7	21	--	--	--	--	--	--	ATGTGTCGTCCCACAGCATA	60	TCTCCCTCAAAACCGTTAGG	59	139	--	--	XM_004489123.1	--	[J]	K02890
TR42055|c0_g1_i2	No	(GAA)7	21	--	--	--	--	--	--	ATAAAGCGGCAGTGACCAAG	60	GCAGTGACGATTTATCTGCG	59	259	--	--	--	--	--	--
TR42055|c0_g1_i3	No	(GAA)7	21	--	--	--	--	--	--	ATAAAGCGGCAGTGACCAAG	60	GCAGTGACGATTTATCTGCG	59	259	--	--	--	--	--	--
TR42055|c0_g1_i4	No	(GAA)8	24	--	--	--	--	--	--	ATAAAGCGGCAGTGACCAAG	60	GCAGTGACGATTTATCTGCG	59	262	--	--	--	--	--	--
TR42055|c0_g1_i5	No	(GAA)8	24	--	--	--	--	--	--	ATAAAGCGGCAGTGACCAAG	60	GCAGTGACGATTTATCTGCG	59	262	--	--	--	--	--	--
TR42067|c0_g1_i1	XLOC_015912	(TTC)5	15	TR42067|c0_g1_i1	(TTC)5	Ca_LG_3	(TTC)6	33540852	33540869	ACCTGCTCCTGCACCTACAT	60	TGATGGTTGTGTACGCGTTT	60	265		URR	--	--	--	--
TR42072|c1_g1_i1	XLOC_015929	(TTC)7	21	TR42072|c1_g1_i1	(TTC)7	Ca_LG_6	(TTC)6	517338	517355	CCATCGTTGTGACTCTTCCTC	60	CGTTCAGAGGTCTAGGGTGC	60	148	Ca_12991	URR	--	--	--	--
TR42072|c1_g2_i1	XLOC_015930	(TTC)7	21	--	--	--	--	--	--	CCATCGTTGTGACTCTTCCTC	60	CGTTCAGAGGTCTAGGGTGC	60	148	--	--	--	--	--	--
TR42074|c0_g1_i1	No	(AT)6	12	--	--	--	--	--	--	CCCGGACATTTAGCATTGAC	60	AATTTTGGGTCCTTCCCATC	60	200	--	--	--	--	--	--
TR42074|c0_g1_i2	No	(AT)6	12	--	--	--	--	--	--	CCCGGACATTTAGCATTGAC	60	AATTTTGGGTCCTTCCCATC	60	200	--	--	--	--	--	--
TR42074|c0_g1_i3	No	(AT)6	12	--	--	--	--	--	--	CCCGGACATTTAGCATTGAC	60	AATTTTGGGTCCTTCCCATC	60	200	--	--	--	--	--	--
TR42074|c0_g1_i4	No	(AT)6	12	--	--	--	--	--	--	CCCGGACATTTAGCATTGAC	60	AATTTTGGGTCCTTCCCATC	60	200	--	--	--	--	--	--
TR42074|c0_g1_i5	No	(AT)6	12	--	--	--	--	--	--	CCCGGACATTTAGCATTGAC	60	AATTTTGGGTCCTTCCCATC	60	200	--	--	--	--	--	--

Transcript IDs	Differentially expressed gene (DEG) IDs	SSR repeat-motifs	SSR repeat-length (bp)	*In silico* polymorphic SSRs						Forward primers (5'-3')	Annealing tempearture(0C)	Reverse primers (5'-3')	Annealing tempearture(0C)	Amplified product size (bp)	Structural annotation		Functional Annotation			
				C. microphyllum		ICC4958									Gene IDs	Sequence component of genes	NCBI nr IDs	TFs	KOG	KEGG
				Transcript	SSR repeat-motifs	Chromosomes	SSR repeat-motifs	Start physical positions (bp)	End physical positions (bp)											
TR42074|c0_g1_i6	No	(AT)6	12	--	--	--	--	--	--	CCCGGACATTTAGCATTGAC	60	AATTTTGGGTCCTTCCCATC	60	200	--	--	--	--	--	--
TR42074|c0_g2_i1	No	(AT)6	12	--	--	--	--	--	--	CCCGGACATTTAGCATTGAC	60	AATTTTGGGTCCTTCCCATC	60	200	--	--	--	--	--	--
TR42085|c2_g1_i7	No	(TC)10	20	--	--	--	--	--	--	TCCAATGACTAGTGGGCCAT	60	GCCCTGAGTTTGTAAGCTGC	60	210	--	--	--	--	--	--
TR42086|c1_g1_i1	XLOC_015953	(TA)6gtttttttaactattttaccagccttttccataaatcatgtctggctgtgtttgt(A)10	78	--	--	--	--	--	--	TGATGTCATTGATCTTCGGTG	60	AATTGTGGTGCATGAAGTGC	60	245	--	--	--	--	--	--
TR42111|c0_g3_i1	XLOC_015969	(AT)9	18	--	--	--	--	--	--	AAAATCATGGCTCATCCTGC	60	GCCTCTTTCACATTTTGGCT	59	210	--	--	--	--	--	--
TR4212|c0_g1_i1	XLOC_015980	(TGC)5	15	TR4212|c0_g1_i1	(TGC)5	Ca_LG_7	(TGC)6	3844871	3844888	AGAGCCAAAAACAAGGCTCA	60	GTCGACACACCGTGCAGTAT	60	275	Ca_16796	URR	--	--	--	--
TR4212|c0_g2_i1	No	(TGC)5	15	--	--	--	--	--	--	AGAGCCAAAAACAAGGCTCA	60	GTCGACACACCGTGCAGTAT	60	275	--	--	--	--	--	--
TR42122|c0_g1_i1	XLOC_015973	(CGG)5	15	--	--	--	--	--	--	TGCTCTTTTCCTCACCGACT	60	TCGCCGTTAAACCAGTTACC	60	132	--	--	--	--	--	--
TR42128|c0_g2_i1	XLOC_015978	(TGA)5	15	--	--	--	--	--	--	TTTCCGACCCTCGTATTCTG	60	AGGGTTTTTCATGCCAGTTG	60	227	--	--	--	--	--	--
TR42128|c1_g1_i1	XLOC_015979	(T)10attttaaaaaaaaagagtagcgtggatatggatagtaatagcaattttgtaatattaattattaattgtggccgatg(A)12	99	--	--	--	--	--	--	CAACTTTGCAGGTTCGTTTG	59	TTCAGATCTCTTGATCAGATAACAA	57	280	--	--	--	--	--	--
TR42137|c0_g2_i1	No	(TA)9	18	--	--	--	--	--	--	TAATCCTCGTCCCCAACAAG	60	CAATGATACCCTTCACAAGCC	59	250	--	--	--	--	--	--
TR42145|c0_g2_i1	XLOC_015990	(ATC)6	18	--	--	--	--	--	--	TCAAGCCTCTGCAATTTCATT	60	CTGCAAGGATTGTGAAAGCA	60	217	--	--	--	--	--	--
TR42163|c0_g1_i1	No	(TC)6	12	TR42163|c0_g1_i1	(TC)6	Ca_LG_6	(TC)8	48446920	48446935	TCCCTTAAATTGACGCAACC	60	GAGAAGGGACTGAGACGGTG	60	268	Ca_12488	URR	--	--	--	--
TR42163|c0_g2_i1	No	(TC)6	12	--	--	--	--	--	--	TCCCTTAAATTGACGCAACC	60	GAGAAGGGACTGAGACGGTG	60	268	--	--	--	--	--	--

Transcript IDs	Differentially expressed gene (DEG) IDs	SSR repeat-motifs	SSR repeat-length (bp)	*In silico* polymorphic SSRs						Forward primers (5'-3')	Annealing tempearture(0C)	Reverse primers (5'-3')	Annealing tempearture(0C)	Amplified product size (bp)	Structural annotation		Functional Annotation			
				C. microphyllum		ICC4958									Gene IDs	Sequence component of genes	NCBI nr IDs	TFs	KOG	KEGG
				Transcript	SSR repeat-motifs	Chromosomes	SSR repeat-motifs	Start physical positions (bp)	End physical positions (bp)											
TR42170|c1_g1_i1	XLOC_016014	(TAA)5	15	--	--	--	--	--	--	TACGCCCAATCTGAGGTTTC	60	TCTCCCGAATAATTGGTCCT	58	195	--	--	--	--	--	--
TR4220|c0_g1_i1	No	(AG)10	20	--	--	--	--	--	--	TGAGGGTTTTACGAAGAAACAGA	60	CCCACTACGTCCTGCAAAAT	60	154	--	--	--	--	--	--
TR4220|c0_g2_i1	No	(AG)10	20	--	--	--	--	--	--	TGAGGGTTTTACGAAGAAACAGA	60	CCCACTACGTCCTGCAAAAT	60	154	--	--	--	--	--	--
TR42207|c0_g1_i1	No	(TG)8	16	--	--	--	--	--	--	CACCCTTGGTTTGACCTCAT	60	CCGAGCAGACTGTCAATCAA	60	191	--	--	--	--	--	--
TR42224|c0_g1_i1	No	(GTT)5	15	--	--	--	--	--	--	GAGCAGAAGTTATGACCGGC	60	TGCGTGAGACTCCAAAGATG	60	213	--	--	--	--	--	--
TR42269|c0_g1_i1	No	(TTA)5	15	--	--	--	--	--	--	GCTTCCCTTTCAAATTTGTTG	59	GGAAATGTTTGCTGAACACG	59	209	--	--	--	--	--	--
TR42285|c0_g1_i1	XLOC_016049	(AG)7	14	--	--	--	--	--	--	GCTTTTCATTCCATCGTGGT	60	GCATCAACCTGCACTCTCAA	60	128	--	--	--	--	--	--
TR42291|c1_g2_i1	XLOC_016057	(CT)8	16	TR42291|c1_g2_i1	(CT)8	Ca_LG_4	(CT)9	20222359	20222376	GGACTCAACGGTCCCTTTTC	61	TTTGGTAGTGTGGTGACCGA	60	146	Ca_03548	URR	--	--	--	--
TR42298|c0_g2_i2	No	(GT)6	12	--	--	--	--	--	--	TTGCAACGCATCTATCAAGC	60	TATTCCTGCAAATTTCGCCT	60	221	--	--	--	--	--	--
TR42301|c0_g2_i1	No	(GTG)5	15	--	--	--	--	--	--	ACCCTGCCCTTTTGCTATTT	60	GCCTGTAAACCCAGTTACACC	58	190	--	--	--	--	--	--
TR42301|c0_g2_i2	No	(GTG)5	15	--	--	--	--	--	--	ACCCTGCCCTTTTGCTATTT	60	GCCTGTAAACCCAGTTACACC	58	190	--	--	--	--	--	--
TR42309|c0_g2_i1	No	(CAT)5	15	--	--	--	--	--	--	TTGGCCTTTAATCCGAAACA	60	TTTAGGGACCATTATGAGCAAA	59	132	--	--	--	--	--	--
TR42337|c0_g1_i1	No	(CA)6	12	--	--	--	--	--	--	AGCTTAATAGAATTCAGAAACAATCTC	57	TACACGTGAGTTGGGGAACA	60	185	--	--	--	--	--	--
TR42337|c0_g1_i2	XLOC_016103	(CA)6	12	--	--	--	--	--	--	AGCTTAATAGAATTCAGAAACAATCTC	57	TACACGTGAGTTGGGGAACA	60	185	--	--	--	--	--	--

Transcript IDs	Differentially expressed gene (DEG) IDs	SSR repeat-motifs	SSR repeat-length (bp)	*In silico* polymorphic SSRs						Forward primers (5'-3')	Annealing tempearture(0C)	Reverse primers (5'-3')	Annealing tempearture(0C)	Amplified product size (bp)	Structural annotation		Functional Annotation			
				C. microphyllum		ICC4958									Gene IDs	Sequence component of genes	NCBI nr IDs	TFs	KOG	KEGG
				Transcript	SSR repeat-motifs	Chromosomes	SSR repeat-motifs	Start physical positions (bp)	End physical positions (bp)											
TR42338|c0_g1_i1	No	(CACAA)5	25	--	--	--	--	--	--	TGCTCATAATTCATTGTTCATCA	58	CTGGGAGAGCGATTGAAGAG	60	176	--	--	--	--	--	--
TR42338|c0_g1_i2	No	(TA)10	20	--	--	--	--	--	--	ATATCGTGTTGGGGACTTGG	60	CTTCGGGTATGTCACGCTTT	60	221	--	--	--	--	--	--
TR42338|c0_g1_i2	No	(CACAA)5	25	--	--	--	--	--	--	TGCTCATAATTCATTGTTCATCA	58	CTGGGAGAGCGATTGAAGAG	60	176	--	--	--	--	--	--
TR42343|c0_g1_i1	XLOC_016107	(AG)10	20	--	--	--	--	--	--	CTTGTTGGTTGCATCCATTG	60	CGATCATAATCTCGCAGCAA	60	110	--	--	--	--	--	--
TR42360|c0_g1_i1	XLOC_016119	(GAA)5	15	--	--	--	--	--	--	AACAATGTCACCCAGCTTCC	60	TCTACGGAGGTTGTGCTCCT	60	267	--	--	--	--	--	--
TR42360|c0_g2_i1	No	(GAA)5	15	--	--	--	--	--	--	AACAATGTCACCCAGCTTCC	60	CTTCTGATTCCGATTCGGGT	61	125	--	--	--	--	--	--
TR42401|c0_g1_i1	No	(TC)7	14	--	--	--	--	--	--	TTCACACTTCAGCCTCATCG	60	CACATGCCTTGTATGCCTTG	60	218	--	--	--	--	--	--
TR42401|c0_g2_i1	No	(TC)7	14	--	--	--	--	--	--	TTCACACTTCAGCCTCATCG	60	CACATGCCTTGTATGCCTTG	60	218	--	--	--	--	--	--
TR42404|c0_g1_i1	No	(TAA)6	18	--	--	--	--	--	--	CCCAAAAGACTGAAGCAACA	59	TGTGTCATTCCACCCATTGT	60	233	--	--	--	--	--	--
TR42404|c0_g2_i1	No	(TAA)6	18	--	--	--	--	--	--	CCCAAAAGACTGAAGCAACA	59	TGTGTCATTCCACCCATTGT	60	233	--	--	--	--	--	--
TR4243|c0_g1_i1	No	(GTGTCA)5	30	--	--	--	--	--	--	AACCACCTCTCCTCCAACCT	60	ATTGACACACGCTCAGTCCA	60	172	--	--	--	--	--	--
TR42444|c0_g1_i1	XLOC_016180	(TTA)5	15	--	--	--	--	--	--	TCCCTGGGTGATGGTGTAGT	60	GTTCAACAAGGGAGGAACCA	60	265	--	--	XP_011493026.1	Trihelix	[G]	--
TR42444|c0_g1_i1	XLOC_016180	(GT)8	16	--	--	--	--	--	--	TCCTCCCTTGTTGAACGTGT	61	ACGACAACGAATTCGGCTAC	60	247	--	--	XP_011493026.1	Trihelix	[G]	--
TR42451|c0_g1_i1	XLOC_016187	(CCA)6	18	--	--	--	--	--	--	ACCATGAGCCCAAGGAGAAT	61	TTAATGGCGGCTACGTTTTC	60	261	--	--	XM_004495506.1	--	[S]	--

Transcript IDs	Differentially expressed gene (DEG) IDs	SSR repeat-motifs	SSR repeat-length (bp)	*In silico* polymorphic SSRs						Forward primers (5'-3')	Annealing tempearture(0C)	Reverse primers (5'-3')	Annealing tempearture(0C)	Amplified product size (bp)	Structural annotation		Functional Annotation			
				C. microphyllum		ICC4958									Gene IDs	Sequence component of genes	NCBI nr IDs	TFs	KOG	KEGG
				Transcript	SSR repeat-motifs	Chromosomes	SSR repeat-motifs	Start physical positions (bp)	End physical positions (bp)											
TR42469|c0_g1_i2	No	(CT)6	12	--	--	--	--	--	--	ATTCACAAAACGCAGTGCTC	59	ATTTCGTCGCACCAAAATTC	60	167	--	--	--	--	--	--
TR42478|c0_g1_i1	XLOC_016212	(CAG)5	15	--	--	--	--	--	--	TGCTGCTTCCTCCTATCAAAA	60	GGCAAATTGAGCCTGACACT	60	280	--	--	--	--	--	--
TR42479|c0_g1_i1	No	(CAT)5	15	--	--	--	--	--	--	CCACGTTACGCGTCCTAAAT	60	CGACCAACGTTACGGTTCTT	60	251	--	--	--	--	--	--
TR42479|c0_g1_i10	No	(CAT)5	15	--	--	--	--	--	--	CCACGTTACGCGTCCTAAAT	60	CGACCAACGTTACGGTTCTT	60	251	--	--	--	--	--	--
TR42479|c0_g1_i11	No	(CAT)5	15	--	--	--	--	--	--	CCACGTTACGCGTCCTAAAT	60	CGACCAACGTTACGGTTCTT	60	251	--	--	--	--	--	--
TR42479|c0_g1_i12	No	(CAT)5	15	--	--	--	--	--	--	CCACGTTACGCGTCCTAAAT	60	CGACCAACGTTACGGTTCTT	60	251	--	--	--	--	--	--
TR42479|c0_g1_i2	No	(CAT)5	15	--	--	--	--	--	--	CCACGTTACGCGTCCTAAAT	60	CGACCAACGTTACGGTTCTT	60	251	--	--	--	--	--	--
TR42479|c0_g1_i3	No	(CAT)5	15	--	--	--	--	--	--	CCACGTTACGCGTCCTAAAT	60	CGACCAACGTTACGGTTCTT	60	251	--	--	--	--	--	--
TR42479|c0_g1_i4	No	(CAT)5	15	--	--	--	--	--	--	CCACGTTACGCGTCCTAAAT	60	CGACCAACGTTACGGTTCTT	60	251	--	--	--	--	--	--
TR42479|c0_g1_i5	No	(CAT)5	15	--	--	--	--	--	--	CCACGTTACGCGTCCTAAAT	60	CGACCAACGTTACGGTTCTT	60	251	--	--	--	--	--	--
TR42479|c0_g1_i6	No	(CAT)5	15	--	--	--	--	--	--	CCACGTTACGCGTCCTAAAT	60	CGACCAACGTTACGGTTCTT	60	251	--	--	--	--	--	--
TR42479|c0_g1_i7	No	(CAT)5	15	--	--	--	--	--	--	CCACGTTACGCGTCCTAAAT	60	CGACCAACGTTACGGTTCTT	60	251	--	--	--	--	--	--
TR42479|c0_g1_i8	No	(CAT)5	15	--	--	--	--	--	--	CCACGTTACGCGTCCTAAAT	60	CGACCAACGTTACGGTTCTT	60	251	--	--	--	--	--	--
TR42479|c0_g1_i9	No	(CAT)5	15	--	--	--	--	--	--	CCACGTTACGCGTCCTAAAT	60	CGACCAACGTTACGGTTCTT	60	251	--	--	--	--	--	--

Transcript IDs	Differentially expressed gene (DEG) IDs	SSR repeat-motifs	SSR repeat-length (bp)	*In silico* polymorphic SSRs						Forward primers (5'-3')	Annealing tempearture(0C)	Reverse primers (5'-3')	Annealing tempearture(0C)	Amplified product size (bp)	Structural annotation		Functional Annotation			
				C. microphyllum		ICC4958									Gene IDs	Sequence component of genes	NCBI nr IDs	TFs	KOG	KEGG
				Transcript	SSR repeat-motifs	Chromosomes	SSR repeat-motifs	Start physical positions (bp)	End physical positions (bp)											
TR42484|c6_g1_i1	No	(AAAT)5	20	--	--	--	--	--	--	CCCGAAACATACACGCAAAT	61	TAAGCTCTCTGACCCTCCCA	60	236	--	--	--	--	--	--
TR42493|c2_g2_i1	XLOC_016234	(GA)6	12	--	--	--	--	--	--	CCAGCACCCATGATTCTTCT	60	AACGCTATCTACCGCTTCCA	60	243	--	--	--	--	--	--
TR42493|c2_g2_i2	XLOC_016235	(GA)7gtttagaagaaaatgaggcggtgaaaggagggaatggggttcttgtagaacttcaaggcggtagtaatgtg(T)10	95	--	--	--	--	--	--	TGATTCTCCTGTTTTTGGGC	60	TCTGGTTCGGAGTCAGGTTT	60	280	--	--	--	--	--	--
TR42497|c0_g1_i1	XLOC_016240	(TGT)7	21	--	--	--	--	--	--	CATTCAACAAACACCTTGCG	60	CTGCCGCTATTCTTATTGGG	60	267	--	--	--	--	--	--
TR42497|c2_g1_i1	XLOC_016241	(AAC)6	18	--	--	--	--	--	--	ATTGGAGGAACAACCACAGC	60	CGTTCGGCAGTTTTCTTAGC	60	221	--	--	--	--	--	--
TR42497|c4_g1_i1	No	(AAC)6	18	--	--	--	--	--	--	CTTTGATCCATGGGAGAGGA	60	GGTTTTCAATCTGCCTCCTG	60	246	--	--	--	--	--	--
TR42497|c4_g1_i2	XLOC_016242	(AAC)6	18	--	--	--	--	--	--	CTTTGATCCATGGGAGAGGA	60	GGTTTTCAATCTGCCTCCTG	60	246	--	--	--	--	--	--
TR42504|c3_g1_i1	XLOC_016254	(AC)6	12	--	--	--	--	--	--	TTGGAACTTCTCCACAAAATCA	60	CACGAGGTTTTGTCATCCAC	59	137	--	--	--	--	--	--
TR42521|c0_g1_i1	No	(CT)6	12	--	--	--	--	--	--	AGCGAGAAAACAGAGCGGTA	60	ATTTGAGAATTGGCGGTGAG	60	239	--	--	--	--	--	--
TR42521|c0_g2_i1	No	(CT)6	12	--	--	--	--	--	--	AGCGAGAAAACAGAGCGGTA	60	ATTTGAGAATTGGCGGTGAG	60	239	--	--	--	--	--	--
TR42521|c0_g2_i2	No	(CT)6	12	--	--	--	--	--	--	AGCGAGAAAACAGAGCGGTA	60	ATTTGAGAATTGGCGGTGAG	60	239	--	--	--	--	--	--
TR42521|c0_g2_i3	No	(CT)6	12	--	--	--	--	--	--	AGCGAGAAAACAGAGCGGTA	60	ATTTGAGAATTGGCGGTGAG	60	239	--	--	--	--	--	--
TR42521|c0_g2_i4	No	(CT)6	12	--	--	--	--	--	--	AGCGAGAAAACAGAGCGGTA	60	ATTTGAGAATTGGCGGTGAG	60	239	--	--	--	--	--	--
TR42521|c0_g2_i5	No	(CT)6	12	--	--	--	--	--	--	AGCGAGAAAACAGAGCGGTA	60	ATTTGAGAATTGGCGGTGAG	60	239	--	--	--	--	--	--

Transcript IDs	Differentially expressed gene (DEG) IDs	SSR repeat-motifs	SSR repeat-length (bp)	*In silico* polymorphic SSRs						Forward primers (5'-3')	Annealing tempearture(0C)	Reverse primers (5'-3')	Annealing tempearture(0C)	Amplified product size (bp)	Structural annotation		Functional Annotation			
				C. microphyllum		ICC4958									Gene IDs	Sequence component of genes	NCBI nr IDs	TFs	KOG	KEGG
				Transcript	SSR repeat-motifs	Chromosomes	SSR repeat-motifs	Start physical positions (bp)	End physical positions (bp)											
TR42521|c0_g2_i6	No	(CT)6	12	--	--	--	--	--	--	AGCGAGAAAACAGAGCGGTA	60	ATTTGAGAATTGGCGGTGAG	60	239	--	--	--	--	--	--
TR42538|c2_g1_i1	XLOC_016296	(AT)6	12	--	--	--	--	--	--	GCCCCCACTAAGATTTCGAT	60	TTTTTGCCCCGTTTGTTTAG	60	208	--	--	--	--	--	--
TR4256|c0_g1_i1	No	(CT)6	12	--	--	--	--	--	--	GCGCACCTCGTTTACTCACT	60	GAAAGTGGGGCAAAACGTAA	60	176	--	--	--	--	--	--
TR42560|c0_g1_i1	XLOC_016321	(TTA)5	15	--	--	--	--	--	--	AATGCCATCCTCGATCAGTC	60	CCCTGCCAAATAAACTTCCA	60	218	--	--	--	--	--	--
TR42564|c0_g1_i1	No	(TAA)6	18	--	--	--	--	--	--	TCGAGCTTCGGAAGTAAACAA	60	GCTCGCCATTTTTCTTTTCA	60	122	--	--	--	--	--	--
TR42564|c0_g1_i2	No	(TAA)6	18	--	--	--	--	--	--	TCGAGCTTCGGAAGTAAACAA	60	GCTCGCCATTTTTCTTTTCA	60	122	--	--	--	--	--	--
TR42582|c0_g1_i1	No	(TCC)6	18	--	--	--	--	--	--	CGCATCAATGGACAACTCAA	61	TGTCTGTTGTGGCTGTGGTT	60	218	--	--	--	--	--	--
TR42592|c0_g1_i1	No	(ATG)5	15	--	--	--	--	--	--	GAATGGAAGCACGGAGGATA	60	CAGCAGACAAGCCAGACAGA	60	196	--	--	--	--	--	--
TR42727|c1_g1_i1	No	(AT)6	12	--	--	--	--	--	--	GCCCTTAGAGAACACGATGC	60	TCCTCGGTGTTAAATGACCTG	60	229	--	--	--	--	--	--
TR42746|c0_g1_i1	No	(AAC)6	18	--	--	--	--	--	--	AATCCATGGCCAACATCAAT	60	TGTTCCCTTGTGACTGAGCA	60	162	--	--	--	--	--	--
TR42746|c0_g2_i1	XLOC_016370	(AAC)6	18	--	--	--	--	--	--	AATCCATGGCCAACATCAAT	60	CAGCTGTGCACGAGAAGAAG	60	227	--	--	--	--	--	--
TR42755|c0_g1_i1	XLOC_016375	(CAA)5	15	--	--	--	--	--	--	AAACCTTCACCAAACCCCTT	60	TGTTGGTGAGATTGGAAGGA	59	239	--	--	XP_004489677.1	--	[M]	--
TR42755|c0_g1_i1	XLOC_016375	(T)11gttgtgaaacatcaacaacaaaaaaaccaataca aatcttaaacc(A)10	66	--	--	--	--	--	--	TTTTCATGCCTTTTCCCTATG	59	TCCTTGACATTTTTGGCCTT	60	220	--	--	XP_004489677.1	--	[M]	--
TR42767|c0_g2_i1	No	(TC)6	12	--	--	--	--	--	--	TTCTACCTCTCTCTCTTTCAACCA	59	GCGGAGTACAAGGAAGCAAG	60	276	--	--	--	--	--	--

<table>
<tr><th rowspan="3">Transcript IDs</th><th rowspan="3">Differentially expressed gene (DEG) IDs</th><th rowspan="3">SSR repeat-motifs</th><th rowspan="3">SSR repeat-length (bp)</th><th colspan="6">In silico polymorphic SSRs</th><th rowspan="3">Forward primers (5'-3')</th><th rowspan="3">Annealing tempearture(0C)</th><th rowspan="3">Reverse primers (5'-3')</th><th rowspan="3">Annealing tempearture(0C)</th><th rowspan="3">Amplified product size (bp)</th><th colspan="2">Structural annotation</th><th colspan="4">Functional Annotation</th></tr>
<tr><th colspan="2">C. microphyllum</th><th colspan="4">ICC4958</th><th rowspan="2">Gene IDs</th><th rowspan="2">Sequence component of genes</th><th rowspan="2">NCBI nr IDs</th><th rowspan="2">TFs</th><th rowspan="2">KOG</th><th rowspan="2">KEGG</th></tr>
<tr><th>Transcript</th><th>SSR repeat-motifs</th><th>Chromosomes</th><th>SSR repeat-motifs</th><th>Start physical positions (bp)</th><th>End physical positions (bp)</th></tr>
<tr><td>TR42767|c0_g2_i2</td><td>No</td><td>(TC)6</td><td>12</td><td>--</td><td>--</td><td>--</td><td>--</td><td>--</td><td>--</td><td>TTCTACCTCTCTCTCTTTCAACCA</td><td>59</td><td>GCGGAGTACAAGGAAGCAAG</td><td>60</td><td>219</td><td>--</td><td>--</td><td>--</td><td>--</td><td>--</td><td>--</td></tr>
<tr><td>TR42802|c0_g1_i1</td><td>No</td><td>(ACA)6</td><td>18</td><td>--</td><td>--</td><td>--</td><td>--</td><td>--</td><td>--</td><td>GAGTGCTGTTTTCATGGGGT</td><td>60</td><td>ATGGGGTGAAGATGTTCCTG</td><td>60</td><td>256</td><td>--</td><td>--</td><td>--</td><td>--</td><td>--</td><td>--</td></tr>
<tr><td>TR42802|c0_g1_i2</td><td>No</td><td>(ACA)6</td><td>18</td><td>--</td><td>--</td><td>--</td><td>--</td><td>--</td><td>--</td><td>GAGTGCTGTTTTCATGGGGT</td><td>60</td><td>ATGGGGTGAAGATGTTCCTG</td><td>60</td><td>256</td><td>--</td><td>--</td><td>--</td><td>--</td><td>--</td><td>--</td></tr>
<tr><td>TR4281|c0_g1_i1</td><td>No</td><td>(TA)6</td><td>12</td><td>--</td><td>--</td><td>--</td><td>--</td><td>--</td><td>--</td><td>CAATGCGTACCATTACTGCAA</td><td>60</td><td>TTGAGGAGGACAAGGAAGGA</td><td>60</td><td>193</td><td>--</td><td>--</td><td>--</td><td>--</td><td>--</td><td>--</td></tr>
<tr><td>TR42921|c0_g1_i1</td><td>No</td><td>(AT)9</td><td>18</td><td>--</td><td>--</td><td>--</td><td>--</td><td>--</td><td>--</td><td>GCTGTTCATCAGCCCATCTT</td><td>60</td><td>TTAGCTTTCTCCACACACACG</td><td>59</td><td>266</td><td>--</td><td>--</td><td>--</td><td>--</td><td>--</td><td>--</td></tr>
<tr><td>TR42921|c0_g2_i1</td><td>XLOC_016441</td><td>(AT)9</td><td>18</td><td>--</td><td>--</td><td>--</td><td>--</td><td>--</td><td>--</td><td>GCTGTTCATCAGCCCATCTT</td><td>60</td><td>TTAGCTTTCTCCACACACACG</td><td>59</td><td>266</td><td>--</td><td>--</td><td>--</td><td>--</td><td>--</td><td>--</td></tr>
<tr><td>TR42923|c0_g1_i1</td><td>XLOC_016443</td><td>(A)11ctttatcgtgaaagcgaaagaaag(A)10</td><td>45</td><td>--</td><td>--</td><td>--</td><td>--</td><td>--</td><td>--</td><td>ACCGCTACTGCATCTGGTTT</td><td>60</td><td>GCTTCCCATTTCTTCTTCCC</td><td>60</td><td>218</td><td>--</td><td>--</td><td>--</td><td>--</td><td>--</td><td>--</td></tr>
<tr><td>TR42957|c0_g1_i1</td><td>No</td><td>(TC)7</td><td>14</td><td>TR42957|c0_g1_i1</td><td>(TC)7</td><td>Ca_LG_3</td><td>(TC)6</td><td>38974121</td><td>38974132</td><td>ACACAGCCCACAGGGATAAC</td><td>60</td><td>ATATCGGCGGAACCCTAATC</td><td>60</td><td>179</td><td></td><td>URR</td><td>--</td><td>--</td><td>--</td><td>--</td></tr>
<tr><td>TR42959|c1_g1_i1</td><td>XLOC_016457</td><td>(ACA)5agggttcataggaatcaaacctcg(TCA)5</td><td>54</td><td>--</td><td>--</td><td>--</td><td>--</td><td>--</td><td>--</td><td>CGATATCGATGAGGATGCTG</td><td>59</td><td>GAAACCCCTCTTCTTGTCCTT</td><td>59</td><td>144</td><td>--</td><td>--</td><td>--</td><td>--</td><td>--</td><td>--</td></tr>
<tr><td>TR42990|c0_g1_i1</td><td>No</td><td>(ATT)6</td><td>18</td><td>--</td><td>--</td><td>--</td><td>--</td><td>--</td><td>--</td><td>GCTTTTGAGGAAAACTGTTTGTT</td><td>59</td><td>TGATTTCTCGTGATGCCTTG</td><td>60</td><td>148</td><td>--</td><td>--</td><td>--</td><td>--</td><td>--</td><td>--</td></tr>
<tr><td>TR43046|c0_g1_i1</td><td>XLOC_016472</td><td>(AAC)5</td><td>15</td><td>--</td><td>--</td><td>--</td><td>--</td><td>--</td><td>--</td><td>GCATTCAACCCTAACCCAAA</td><td>60</td><td>GTTGTTGTACCCATTTCCGC</td><td>60</td><td>240</td><td>--</td><td>--</td><td>--</td><td>--</td><td>--</td><td>--</td></tr>
<tr><td>TR43100|c0_g1_i1</td><td>XLOC_016497</td><td>(AGA)5</td><td>15</td><td>--</td><td>--</td><td>--</td><td>--</td><td>--</td><td>--</td><td>TTTCCTCAGGGTTGCTTTTG</td><td>60</td><td>TCTCCTCTTTCTCGGCTTCA</td><td>60</td><td>213</td><td>--</td><td>--</td><td>--</td><td>--</td><td>--</td><td>--</td></tr>
<tr><td>TR43154|c0_g1_i1</td><td>XLOC_016514</td><td>(CTG)6</td><td>18</td><td>--</td><td>--</td><td>--</td><td>--</td><td>--</td><td>--</td><td>TCTTTTCCTCAGGTGCTGGT</td><td>60</td><td>ATTCTGCCGTTTTTCAGGTG</td><td>60</td><td>135</td><td>--</td><td>--</td><td>XP_006432413.1</td><td>--</td><td>[X]</td><td>K02942</td></tr>
<tr><td>TR43253|c0_g1_i1</td><td>XLOC_016541</td><td>(TCT)6</td><td>18</td><td>--</td><td>--</td><td>--</td><td>--</td><td>--</td><td>--</td><td>TGCAACAATGGAGAAAATTGA</td><td>59</td><td>TGGGCGGGTTATAGGTCATA</td><td>60</td><td>200</td><td>--</td><td>--</td><td>--</td><td>--</td><td>--</td><td>--</td></tr>
</table>

Transcript IDs	Differentially expressed gene (DEG) IDs	SSR repeat-motifs	SSR repeat-length (bp)	*In silico* polymorphic SSRs						Forward primers (5'-3')	Annealing tempearture(0C)	Reverse primers (5'-3')	Annealing tempearture(0C)	Amplified product size (bp)	Structural annotation		Functional Annotation			
				C. microphyllum		ICC4958									Gene IDs	Sequence component of genes	NCBI nr IDs	TFs	KOG	KEGG
				Transcript	SSR repeat-motifs	Chromosomes	SSR repeat-motifs	Start physical positions (bp)	End physical positions (bp)											
TR43279|c0_g1_i1	XLOC_016556	(TG)6	12	--	--	--	--	--	--	TGAAAAGCGAGATCTTTGGC	60	CCCTCCATCTTTGACTGATTG	60	158	--	--	--	--	--	--
TR43300|c0_g1_i1	XLOC_016568	(T)14ctttccatttcgattttcctctttatgtacactaccaaaattattattgcttctgtatgaagaaaatatt(A)11gcaactt(A)11cgaaggaccatgtaa(T)10	138	--	--	--	--	--	--	TGATTCAGAAAATGAAGGTAGGTT	58	AGCCCAATCATGGACAGATG	61	204	--	--	--	--	--	--
TR43338|c0_g1_i1	No	(TCT)5	15	--	--	--	--	--	--	TGACTGCTGGTTCTGGTTCA	60	TCATGATCAAATGATCGGAAA	59	208	--	--	--	--	--	--
TR43338|c0_g2_i1	No	(TCT)5	15	--	--	--	--	--	--	TGACTGCTGGTTCTGGTTCA	60	TCATGATCAAATGATCGGAAA	59	209	--	--	--	--	--	--
TR43359|c0_g1_i1	No	(TG)6	12	--	--	--	--	--	--	AGACGTACTCGGCCATGACT	60	CCTCCTTCTTCCTCCTCACA	59	148	--	--	--	--	--	--
TR43359|c0_g2_i1	No	(TG)7	14	--	--	--	--	--	--	AGACGTACTCGGCCATGACT	60	AATTGCTCCTCCTTCTTCCC	60	156	--	--	--	--	--	--
TR43373|c0_g1_i1	XLOC_016592	(TC)6	12	--	--	--	--	--	--	AACACAAAGAAGGGCCAAGA	60	TGACCCAGAAATGGAAACAA	59	143	--	--	--	--	--	--
TR43373|c0_g1_i1	XLOC_016592	(A)11gagagattctcttcaatttctcgaattttccctc(T)11	56	--	--	--	--	--	--	TCTCGTTTTTCTTAAACTGGGT	57	TCAGCAACAACAACCGTAGC	60	259	--	--	--	--	--	--
TR43377|c0_g1_i1	No	(GA)6	12	--	--	--	--	--	--	AAGAGCACTCTCGTTTTGCC	60	AATCGCCGATGACAGAAAAC	60	172	--	--	--	--	--	--
TR43377|c0_g1_i1	No	(GA)6	12	--	--	--	--	--	--	TGGAACTGTGGAAGGGAATC	60	AGAAAACCCCACGACAACAC	60	273	--	--	--	--	--	--
TR43377|c0_g2_i1	No	(GA)6	12	--	--	--	--	--	--	AAGAGCACTCTCGTTTTGCC	60	AATCGCCGATGACAGAAAAC	60	172	--	--	--	--	--	--
TR43377|c0_g2_i1	No	(GA)6	12	--	--	--	--	--	--	TGGAACTGTGGAAGGGAATC	60	AGAAAACCCCACGACAACAC	60	274	--	--	--	--	--	--
TR43385|c0_g1_i1	XLOC_016603	(ACT)6	18	--	--	--	--	--	--	TGCCATCAATGAACAAGCAT	60	AACCAAGTTGGGGAAGGTTT	60	257	--	--	--	--	--	--
TR43397|c0_g1_i1	XLOC_016613	(GAT)5	15	--	--	--	--	--	--	ATCCCCTGTTCGAGGAAGTT	60	GAACCCTTCTGTCGATGCTC	60	277	--	--	--	--	--	--

Transcript IDs	Differentially expressed gene (DEG) IDs	SSR repeat-motifs	SSR repeat-length (bp)	*In silico* polymorphic SSRs						Forward primers (5'-3')	Annealing tempearture(0C)	Reverse primers (5'-3')	Annealing tempearture(0C)	Amplified product size (bp)	Structural annotation		Functional Annotation			
				C. microphyllum		**ICC4958**									**Gene IDs**	**Sequence component of genes**	**NCBI nr IDs**	**TFs**	**KOG**	**KEGG**
				**Transcript**	**SSR repeat-motifs**	**Chromosomes**	**SSR repeat-motifs**	**Start physical positions (bp)**	**End physical positions (bp)**											
TR43397|c0_g1_i2	No	(GAT)5	15	--	--	--	--	--	--	ATCCCCTGTTCGAGGAAGTT	60	GAACCCTTCTGTCGATGCTC	60	264	--	--	--	--	--	--
TR43409|c0_g2_i1	No	(CT)7	14	--	--	--	--	--	--	ACCCCAACAACAACTCAAGC	60	GAGTGAAGGAAATGGCCAAA	60	259	--	--	--	--	--	--
TR43421|c0_g1_i1	No	(TC)9aaacctctctgaagcttcctaaaaccc(T)10	55	--	--	--	--	--	--	CTTACAGGCCACAGCCACTT	60	TCGATTTTCCAGCCATTTTC	60	192	--	--	--	--	--	--
TR43421|c0_g2_i1	No	(TC)9aaacctctctgaagcttcctaaaaccc(T)10	55	--	--	--	--	--	--	CTTACAGGCCACAGCCACTT	60	TCGATTTTCCAGCCATTTTC	60	192	--	--	--	--	--	--
TR43421|c0_g3_i1	No	(TC)9aaacctctctgaagcttcctaaaaccc(T)10	55	--	--	--	--	--	--	CTTACAGGCCACAGCCACTT	60	TCGATTTTCCAGCCATTTTC	60	192	--	--	--	--	--	--
TR43426|c0_g1_i1	XLOC_016644	(TAA)6	18	--	--	--	--	--	--	AGCGAGAATTCAGGTCCAAC	59	GAGCAAGGCCTAATTGCTTG	60	244	--	--	--	--	--	--
TR43438|c0_g1_i1	XLOC_016653	(ACA)5	15	--	--	--	--	--	--	GGAGGGAAGTGATAATGGAGAA	59	CCTGAACGTTGTGCTTCAAA	60	202	--	--	--	--	--	--
TR43438|c0_g1_i2	XLOC_016654	(ACA)5	15	--	--	--	--	--	--	GGAGGGAAGTGATAATGGAGAA	59	CCTGAACGTTGTGCTTCAAA	60	202	--	--	--	--	--	--
TR43445|c0_g1_i1	XLOC_016659	(A)10gttgaaaaaaaagataaaaacaaaaaaaatcatggccctctgggcatattatgcctacttaagtatttcatcacaaaaccaaag(T)11	105	--	--	--	--	--	--	AAAGCCATTTTTGTATGCCG	60	TTGATTCTTGCGGGAAAAAC	60	196	--	--	--	--	--	--
TR43457|c0_g1_i1	XLOC_016669	(TC)8	16	--	--	--	--	--	--	CTCACTGGCAGCGATCATTA	60	GTTGCAACGATCTGCACAAG	60	173	--	--	--	--	--	--
TR43478|c0_g1_i1	No	(TTC)5	15	--	--	--	--	--	--	AATACGAATTGGTGCAAGGC	60	TTGACACTGGATGAGTGGGA	60	272	--	--	--	--	--	--
TR43480|c0_g1_i1	XLOC_016686	(TA)6ttttttatttctctagaatattgccat(TTA)5	54	--	--	--	--	--	--	AACGCTACCAACTTGATCCG	60	GGACCATGCAACATACTTGCT	60	191	--	--	--	--	--	--
TR43500|c0_g1_i1	No	(ACA)6	18	--	--	--	--	--	--	CATCGTGCACTACACGCTCT	60	GCAAGCCTACCCATCTTTGA	60	178	--	--	--	--	--	--
TR43500|c0_g2_i1	XLOC_016697	(ACA)6	18	--	--	--	--	--	--	CATCGTGCACTACACGCTCT	60	GCAAGCCTACCCATCTTTGA	60	178	--	--	--	--	--	--

Transcript IDs	Differentially expressed gene (DEG) IDs	SSR repeat-motifs	SSR repeat-length (bp)	*In silico* polymorphic SSRs						Forward primers (5'-3')	Annealing tempearture(0C)	Reverse primers (5'-3')	Annealing tempearture(0C)	Amplified product size (bp)	Structural annotation		Functional Annotation			
				C. microphyllum		ICC4958									Gene IDs	Sequence component of genes	NCBI nr IDs	TFs	KOG	KEGG
				Transcript	SSR repeat-motifs	Chromosomes	SSR repeat-motifs	Start physical positions (bp)	End physical positions (bp)											
TR43504|c0_g1_i1	No	(GA)7	14	--	--	--	--	--	--	TAGGCACCGTTTTACATCCC	60	GCTGATCATATGGATGCGTG	60	274	--	--	--	--	--	--
TR43534|c0_g1_i1	XLOC_016721	(GAA)5	15	--	--	--	--	--	--	AAGCTTGCCCTGAGTTTGAA	60	AAGGAACCTGAAAAGCCCAT	60	270	--	--	--	--	--	--
TR43659|c0_g1_i1	XLOC_016782	(TTC)5	15	--	--	--	--	--	--	AGGGATCAAAGCAAACACACT	59	TTCGTTTTCTTCGAAGGGTT	59	258	--	--	--	--	--	--
TR43706|c0_g1_i1	No	(GTG)6	18	--	--	--	--	--	--	GATGGTGGTGGGTGATAAGG	60	CCACCTCCTCCAGTTCACTC	60	179	--	--	--	--	--	--
TR43706|c0_g2_i1	XLOC_016804	(GTG)6	18	--	--	--	--	--	--	GATGGTGGTGGGTGATAAGG	60	CCACCTCCTCCAGTTCACTC	60	179	--	--	--	--	--	--
TR43722|c0_g1_i4	No	(GTT)8	24	--	--	--	--	--	--	TCCACCGCCATTAATGTTAT	58	CCTTTCAAATAGCGCCAAAC	60	206	--	--	--	--	--	--
TR43722|c0_g1_i5	No	(GTT)8	24	--	--	--	--	--	--	TCCACCGCCATTAATGTTAT	58	CCTTTCAAATAGCGCCAAAC	60	206	--	--	--	--	--	--
TR43733|c1_g1_i1	XLOC_016833	(ATT)5	15	--	--	--	--	--	--	TGGACTGAATTGACAAAATCCT	59	TGGTTTTCTGCAAAATCTCG	59	271	--	--	XM_004493241.1	--	--	--
TR43758|c0_g1_i1	XLOC_016855	(GAT)5	15	--	--	--	--	--	--	ACTCTTTGCGCTCACATTCA	60	TTCCATCACCAAAACCACAA	60	267	--	--	--	--	--	--
TR43758|c3_g1_i1	XLOC_016857	(AG)8	16	--	--	--	--	--	--	GTGCTGTTGTTCATCTCCGA	60	AACCACCATCCACTCTGTCA	59	261	--	--	--	--	--	--
TR43760|c0_g3_i1	No	(AAC)7aagagatcattcaaaatggaggagggtttggatt aagacactt(CAA)5	79	--	--	--	--	--	--	TAGTTGCCTAACATCGTCCG	59	TGACTTTCAACTCCAATGCTCA	61	267	--	--	--	--	--	--
TR43777|c0_g1_i1	XLOC_016870	(GAA)11	33	--	--	--	--	--	--	ACAGCGAGGAGCAACCTAAA	60	TTGTTTTTCGAATGATGCCA	60	177	--	--	--	--	--	--
TR43777|c2_g1_i1	No	(GAA)5	15	--	--	--	--	--	--	CAATGGCAAAAACAAAAACAGA	60	CGATTTCGTTCTGCTTCCTC	60	153	--	--	--	--	--	--
TR43788|c1_g1_i1	XLOC_016881	(TCT)5	15	--	--	--	--	--	--	TGGGTCCTTCTTCCTCCTTT	60	CCAATCCAGTTGGGCTAATG	60	238	--	--	--	--	--	--

Transcript IDs	Differentially expressed gene (DEG) IDs	SSR repeat-motifs	SSR repeat-length (bp)	*In silico* polymorphic SSRs						Forward primers (5'-3')	Annealing tempearture(0C)	Reverse primers (5'-3')	Annealing tempearture(0C)	Amplified product size (bp)	Structural annotation		Functional Annotation			
				C. microphyllum		ICC4958									Gene IDs	Sequence component of genes	NCBI nr IDs	TFs	KOG	KEGG
				Transcript	SSR repeat-motifs	Chromosomes	SSR repeat-motifs	Start physical positions (bp)	End physical positions (bp)											
TR43803|c0_g2_i1	No	(CCA)5	15	--	--	--	--	--	--	ACTTCACGTTCAACCCCATC	60	AGGCTTTGCACCTTGAAGAA	60	192	--	--	--	--	--	--
TR43803|c0_g2_i1	No	(TCA)8	24	--	--	--	--	--	--	CCAACCAAATCCTCACAACA	59	GATGGGGTTGAACGTGAAGT	60	192	--	--	--	--	--	--
TR43803|c0_g2_i2	No	(CCA)5	15	--	--	--	--	--	--	ACTTCACGTTCAACCCCATC	60	AGGCTTTGCACCTTGAAGAA	60	192	--	--	--	--	--	--
TR43803|c0_g2_i2	No	(TCA)8	24	--	--	--	--	--	--	CCAACCAAATCCTCACAACA	59	GATGGGGTTGAACGTGAAGT	60	192	--	--	--	--	--	--
TR43803|c0_g3_i1	XLOC_016901	(TCA)7	21	--	--	--	--	--	--	AGCAACCCTCCATCATCAAC	60	ATTCCAAGATGGGTTCCACA	60	169	--	--	XP_004509356.1	--	[X]	--
TR43803|c0_g3_i2	No	(TCA)7	21	--	--	--	--	--	--	AGCAACCCTCCATCATCAAC	60	ATTCCAAGATGGGTTCCACA	60	169	--	--	--	--	--	--
TR43813|c0_g1_i1	No	(TC)7	14	--	--	--	--	--	--	TCATCTCCCCACCCAAATTA	60	CGTTGAGACGTTGAAAGCAA	60	262	--	--	--	--	--	--
TR43813|c0_g1_i2	No	(TC)7	14	--	--	--	--	--	--	TCATCTCCCCACCCAAATTA	60	CGTTGAGACGTTGAAAGCAA	60	262	--	--	--	--	--	--
TR43813|c0_g3_i1	No	(TC)7	14	--	--	--	--	--	--	TCATCTCCCCACCCAAATTA	60	CGTTGAGACGTTGAAAGCAA	60	262	--	--	--	--	--	--
TR43817|c0_g1_i2	No	(AG)6	12	TR43817|c0_g1_i2	(AG)6	Ca_LG_5	(AG)9	36674513	36674530	AGAGCATTGTTGGGTTTTGG	60	GAAAGGCGTAGTTGGTGCTC	60	146	Ca_06730	CDS	--	--	--	--
TR43817|c0_g1_i4	No	(AG)6	12	--	--	--	--	--	--	AGAGCATTGTTGGGTTTTGG	60	GAAAGGCGTAGTTGGTGCTC	60	146	--	--	--	--	--	--
TR43817|c0_g1_i5	No	(AG)6	12	--	--	--	--	--	--	AGAGCATTGTTGGGTTTTGG	60	GAAAGGCGTAGTTGGTGCTC	60	146	--	--	--	--	--	--
TR43817|c0_g1_i6	No	(AG)6	12	--	--	--	--	--	--	AGAGCATTGTTGGGTTTTGG	60	GAAAGGCGTAGTTGGTGCTC	60	146	--	--	--	--	--	--
TR43817|c0_g1_i7	No	(AG)6	12	--	--	--	--	--	--	AGAGCATTGTTGGGTTTTGG	60	GAAAGGCGTAGTTGGTGCTC	60	146	--	--	--	--	--	--

Transcript IDs	Differentially expressed gene (DEG) IDs	SSR repeat-motifs	SSR repeat-length (bp)	*In silico* polymorphic SSRs						Forward primers (5'-3')	Annealing tempearture(0C)	Reverse primers (5'-3')	Annealing tempearture(0C)	Amplified product size (bp)	Structural annotation		Functional Annotation			
				C. microphyllum		ICC4958									Gene IDs	Sequence component of genes	NCBI nr IDs	TFs	KOG	KEGG
				Transcript	SSR repeat-motifs	Chromosomes	SSR repeat-motifs	Start physical positions (bp)	End physical positions (bp)											
TR43817|c0_g1_i8	No	(AG)6	12	--	--	--	--	--	--	AGAGCATTGTTGGGTTTTGG	60	GAAAGGCGTAGTTGGTGCTC	60	146	--	--	--	--	--	--
TR43828|c0_g1_i1	XLOC_016919	(CAA)6	18	--	--	--	--	--	--	GAGCTACCAAAACCTCATCACA	59	AATGGCGAGGTTGAGATTTG	60	148	--	--	--	--	--	--
TR43828|c0_g1_i2	XLOC_016920	(CAA)6	18	--	--	--	--	--	--	GAGCTACCAAAACCTCATCACA	59	CACCACCAACAGGAACATTG	60	214	--	--	--	--	--	--
TR43837|c0_g1_i1	XLOC_016932	(TCT)5	15	--	--	--	--	--	--	ATCAACCGATCATGGCTTTC	60	GTGGTGGGAGGAGAAGATGA	60	214	--	--	--	--	--	--
TR43839|c0_g1_i1	No	(CT)7cactcactttcgcactctttttcctcgaactttcct(TTC)7	71	--	--	--	--	--	--	CAACCAATAACCAAAACCCG	60	TGCAGCCTAGTCGAAAAACA	60	249	--	--	--	--	--	--
TR43839|c0_g2_i1	XLOC_016933	(CT)7cactcactttcgcactctttttcctcgaactttcct(TTC)7	71	--	--	--	--	--	--	CAACCAATAACCAAAACCCG	60	TGAATAAGAGAAAATGGCATCG	59	278	--	--	--	--	--	--
TR43849|c0_g2_i1	XLOC_016949	(TTC)6	18	--	--	--	--	--	--	AATGGGGTCATTGGATGAAA	60	AAGAGGAGCACCCAACACAC	60	143	--	--	--	--	--	--
TR43850|c1_g1_i1	XLOC_016952	(AGA)6	18	--	--	--	--	--	--	TTTGCAGCTTTGTCTGCATC	60	TGAAAGGGTTAAGCAATCGG	60	214	--	--	--	--	--	--
TR43850|c1_g1_i2	No	(AGA)6	18	--	--	--	--	--	--	TTTGCAGCTTTGTCTGCATC	60	TGAAAGGGTTAAGCAATCGG	60	214	--	--	--	--	--	--
TR43860|c0_g1_i1	XLOC_016964	(TA)6	12	--	--	--	--	--	--	AAAGCCAACTTGCGATGATG	61	GTTTCTTCGGGTGGTTTGAA	60	255	--	--	--	--	--	--
TR43864|c0_g1_i1	XLOC_016971	(TC)8	16	TR43864|c0_g1_i1	(TC)8	Ca_LG_2	(TC)7	26018331	26018344	TGGGGTGCTAGTAGGAGCTG	60	CACAGCTTGACAATTTCCCA	60	163		URR	--	--	--	--
TR43868|c0_g1_i1	No	(CT)9	18	--	--	--	--	--	--	CTCCATCATCCCCACTTTTC	59	AACTGTTTTCGGCTTCGAGA	60	260	--	--	--	--	--	--
TR43868|c0_g2_i1	XLOC_016975	(CT)9	18	--	--	--	--	--	--	CTCCATCATCCCCACTTTTC	59	AACTGTTTTCGGCTTCGAGA	60	260	--	--	--	--	--	--
TR43896|c0_g1_i1	No	(AAC)5	15	--	--	--	--	--	--	TTCATCAAACACAAGCCCAA	60	AAGAAGGTAGCGCGTGATTG	60	229	--	--	--	--	--	--

Transcript IDs	Differentially expressed gene (DEG) IDs	SSR repeat-motifs	SSR repeat-length (bp)	*In silico* polymorphic SSRs						Forward primers (5'-3')	Annealing tempearture(0C)	Reverse primers (5'-3')	Annealing tempearture(0C)	Amplified product size (bp)	Structural annotation		Functional Annotation			
				C. microphyllum		ICC4958														
				Transcript	SSR repeat-motifs	Chromosomes	SSR repeat-motifs	Start physical positions (bp)	End physical positions (bp)						Gene IDs	Sequence component of genes	NCBI nr IDs	TFs	KOG	KEGG
TR43896|c0_g1_i2	No	(AAC)5	15	--	--	--	--	--	--	TTCATCAAACACAAGCCCAA	60	AAGAAGGTAGCGCGTGATTG	60	229	--	--	--	--	--	--
TR44102|c0_g1_i1	XLOC_017081	(AT)7	14	--	--	--	--	--	--	CTGGGCGTCTTACTTGGAAA	60	AGAATGGAATCAATGCTCCG	60	178	--	--	--	--	--	--
TR44113|c0_g1_i1	No	(A)10tactac(A)10	26	--	--	--	--	--	--	TGGTGTGGCTTACAGAAACG	60	TGATGAGTTATGCTGGACGG	60	238	--	--	--	--	--	--
TR44113|c0_g2_i1	No	(A)10tactac(A)10	26	--	--	--	--	--	--	CTCAAGATCTCGTGGTGCAA	60	TGATGAGTTATGCTGGACGG	60	264	--	--	--	--	--	--
TR44129|c0_g1_i1	XLOC_017095	(TA)7	14	--	--	--	--	--	--	CACACACACCACTTGCCTTC	60	GCTCAGAAGGATTCATGGCT	59	192	--	--	--	--	--	--
TR44139|c0_g3_i1	No	(TGGA)5	20	--	--	--	--	--	--	CAGCTGGATCGATGAATGG	60	CACTCTCCTGCACTGCACAT	60	211	--	--	--	--	--	--
TR4419|c0_g1_i1	XLOC_017116	(AG)6	12	--	--	--	--	--	--	AATAATGGGGTTCCAGGGAG	60	GTACCTTGTCCCCCTTGGTC	61	200	--	--	XP_003053669.1	--	--	--
TR44283|c0_g1_i1	XLOC_017131	(AG)7	14	--	--	--	--	--	--	TGGCATGAGATGAGCGATAG	60	TGACATCGCTTAACCTGAACA	59	244	--	--	--	--	--	--
TR44312|c0_g1_i1	XLOC_017137	(AG)6aagagaagaga(AG)6	35	--	--	--	--	--	--	CCGAATTCAGATTATCGCGT	60	AAAGGGCACGTTACATTTGG	60	208	--	--	--	--	--	--
TR44334|c0_g1_i1	XLOC_017147	(AAC)8	24	--	--	--	--	--	--	CTCGATCTCAAACCAAAGCC	60	GAGGGGATCTGATGAACGAA	60	236	--	--	--	--	--	--
TR44338|c0_g1_i1	No	(TGA)5	15	--	--	--	--	--	--	GCTACTGCGACTGACGATGA	60	TCGTCAAGAGGAAGGTCGAT	60	227	--	--	--	--	--	--
TR44343|c0_g1_i1	XLOC_017151	(AAT)5	15	--	--	--	--	--	--	GGCTATGCCAACATTCCTGT	60	TGAAGTAGGCCAATGCAACA	60	247	--	--	--	--	--	--
TR44440|c0_g1_i1	No	(CATT)6(TC)6*	35	--	--	--	--	--	--	AGGTCATGCCCTTCATCATAG	59	CCATTCCTTTCCTTCCTTCC	60	112	--	--	--	--	--	--
TR44456|c0_g1_i1	XLOC_017180	(AGA)5	15	--	--	--	--	--	--	TTGATCAGCCAATGTTGGAG	60	TCCTCTTCCTCTTCCTCTTCC	59	271	--	--	XP_004491502.1	--	--	--

<table>
<tr><th rowspan="3">Transcript IDs</th><th rowspan="3">Differentially expressed gene (DEG) IDs</th><th rowspan="3">SSR repeat-motifs</th><th rowspan="3">SSR repeat-length (bp)</th><th colspan="6">In silico polymorphic SSRs</th><th rowspan="3">Forward primers (5'-3')</th><th rowspan="3">Annealing tempearture(0C)</th><th rowspan="3">Reverse primers (5'-3')</th><th rowspan="3">Annealing tempearture(0C)</th><th rowspan="3">Amplified product size (bp)</th><th colspan="2">Structural annotation</th><th colspan="4">Functional Annotation</th></tr>
<tr><th colspan="2">C. microphyllum</th><th colspan="4">ICC4958</th><th rowspan="2">Gene IDs</th><th rowspan="2">Sequence component of genes</th><th rowspan="2">NCBI nr IDs</th><th rowspan="2">TFs</th><th rowspan="2">KOG</th><th rowspan="2">KEGG</th></tr>
<tr><th>Transcript</th><th>SSR repeat-motifs</th><th>Chromosomes</th><th>SSR repeat-motifs</th><th>Start physical positions (bp)</th><th>End physical positions (bp)</th></tr>
<tr><td>TR44549|c0_g1_i1</td><td>XLOC_017193</td><td>(TC)8</td><td>16</td><td>--</td><td>--</td><td>--</td><td>--</td><td>--</td><td>--</td><td>CGTTGCATTCCAGAGTGAGA</td><td>60</td><td>GCACGAGAGGATGGAAACTC</td><td>60</td><td>142</td><td>--</td><td>--</td><td>--</td><td>--</td><td>--</td><td>--</td></tr>
<tr><td>TR44553|c0_g2_i1</td><td>XLOC_017195</td><td>(ATC)7</td><td>21</td><td>--</td><td>--</td><td>--</td><td>--</td><td>--</td><td>--</td><td>GCGTCGTTGTTGGAGAATTT</td><td>60</td><td>AATTGGAACACCGCTCTCAG</td><td>60</td><td>105</td><td>--</td><td>--</td><td>--</td><td>--</td><td>--</td><td>--</td></tr>
<tr><td>TR44559|c0_g1_i1</td><td>No</td><td>(A)17tagattcattctatgtctgtgtt(AG)7</td><td>54</td><td>--</td><td>--</td><td>--</td><td>--</td><td>--</td><td>--</td><td>CTAGCATCAGCCTTCCCAAG</td><td>60</td><td>TTCCAAAGCATTCAACCAAA</td><td>59</td><td>265</td><td>--</td><td>--</td><td>--</td><td>--</td><td>--</td><td>--</td></tr>
<tr><td>TR44559|c0_g1_i3</td><td>No</td><td>(A)17tagattcattctatgtctgtgtt(AG)7</td><td>54</td><td>--</td><td>--</td><td>--</td><td>--</td><td>--</td><td>--</td><td>CTAGCATCAGCCTTCCCAAG</td><td>60</td><td>TTCCAAAGCATTCAACCAAA</td><td>59</td><td>265</td><td>--</td><td>--</td><td>--</td><td>--</td><td>--</td><td>--</td></tr>
<tr><td>TR44565|c0_g1_i1</td><td>No</td><td>(CAA)5</td><td>15</td><td>--</td><td>--</td><td>--</td><td>--</td><td>--</td><td>--</td><td>TTTTCCGATGAGATTCCGAG</td><td>60</td><td>TGATTGTAGCGGTGGTGGTA</td><td>60</td><td>229</td><td>--</td><td>--</td><td>--</td><td>--</td><td>--</td><td>--</td></tr>
<tr><td>TR44565|c0_g1_i2</td><td>No</td><td>(CAA)5</td><td>15</td><td>--</td><td>--</td><td>--</td><td>--</td><td>--</td><td>--</td><td>TTTTCCGATGAGATTCCGAG</td><td>60</td><td>TGATTGTAGCGGTGGTGGTA</td><td>60</td><td>229</td><td>--</td><td>--</td><td>--</td><td>--</td><td>--</td><td>--</td></tr>
<tr><td>TR44566|c0_g1_i1</td><td>XLOC_017204</td><td>(TA)6cacacacatgcagctaataaaaaattaag(T)10</td><td>51</td><td>--</td><td>--</td><td>--</td><td>--</td><td>--</td><td>--</td><td>GCGTCTCTTCCAAGCAACAT</td><td>60</td><td>CCCATTTTTCAATTCTTGGTTT</td><td>59</td><td>214</td><td>--</td><td>--</td><td>--</td><td>--</td><td>--</td><td>--</td></tr>
<tr><td>TR44566|c0_g2_i1</td><td>No</td><td>(TA)6cacacacatgcagctaataaaaaattaag(T)10</td><td>51</td><td>--</td><td>--</td><td>--</td><td>--</td><td>--</td><td>--</td><td>GCGTCTCTTCCAAGCAACAT</td><td>60</td><td>CCCATTTTTCAATTCTTGGTTT</td><td>59</td><td>214</td><td>--</td><td>--</td><td>--</td><td>--</td><td>--</td><td>--</td></tr>
<tr><td>TR44570|c0_g1_i1</td><td>No</td><td>(TGG)6</td><td>18</td><td>--</td><td>--</td><td>--</td><td>--</td><td>--</td><td>--</td><td>ATTATGGATCGCGAAACTGG</td><td>60</td><td>CCTTGATATCCACCACCACC</td><td>60</td><td>224</td><td>--</td><td>--</td><td>--</td><td>--</td><td>--</td><td>--</td></tr>
<tr><td>TR44570|c0_g1_i2</td><td>No</td><td>(TGG)6</td><td>18</td><td>--</td><td>--</td><td>--</td><td>--</td><td>--</td><td>--</td><td>ATTATGGATCGCGAAACTGG</td><td>60</td><td>CCTTGATATCCACCACCACC</td><td>60</td><td>224</td><td>--</td><td>--</td><td>--</td><td>--</td><td>--</td><td>--</td></tr>
<tr><td>TR44571|c0_g1_i1</td><td>No</td><td>(ATC)8</td><td>24</td><td>--</td><td>--</td><td>--</td><td>--</td><td>--</td><td>--</td><td>GCGAGGATTAGGGTTTGTGA</td><td>60</td><td>TTTGACTTCACGTCTCGCAC</td><td>60</td><td>106</td><td>--</td><td>--</td><td>--</td><td>--</td><td>--</td><td>--</td></tr>
<tr><td>TR44571|c0_g1_i2</td><td>No</td><td>(ATC)8</td><td>24</td><td>--</td><td>--</td><td>--</td><td>--</td><td>--</td><td>--</td><td>GCGAGGATTAGGGTTTGTGA</td><td>60</td><td>TTTGACTTCACGTCTCGCAC</td><td>60</td><td>106</td><td>--</td><td>--</td><td>--</td><td>--</td><td>--</td><td>--</td></tr>
<tr><td>TR44591|c0_g1_i1</td><td>No</td><td>(TA)6</td><td>12</td><td>--</td><td>--</td><td>--</td><td>--</td><td>--</td><td>--</td><td>GCATGCTGCTCTCTCTGCTA</td><td>60</td><td>TACCGACCGTATCGACCAGT</td><td>60</td><td>276</td><td>--</td><td>--</td><td>--</td><td>--</td><td>--</td><td>--</td></tr>
<tr><td>TR44591|c0_g3_i1</td><td>No</td><td>(TA)6</td><td>12</td><td>--</td><td>--</td><td>--</td><td>--</td><td>--</td><td>--</td><td>GCATGCTGCTCTCTCTGCTA</td><td>60</td><td>TACCGACCGTATCGACCAGT</td><td>60</td><td>276</td><td>--</td><td>--</td><td>--</td><td>--</td><td>--</td><td>--</td></tr>
</table>

Transcript IDs	Differentially expressed gene (DEG) IDs	SSR repeat-motifs	SSR repeat-length (bp)	*In silico* polymorphic SSRs						Forward primers (5'-3')	Annealing tempearture(0C)	Reverse primers (5'-3')	Annealing tempearture(0C)	Amplified product size (bp)	Structural annotation		Functional Annotation			
				C. microphyllum		ICC4958									Gene IDs	Sequence component of genes	NCBI nr IDs	TFs	KOG	KEGG
				Transcript	SSR repeat-motifs	Chromosomes	SSR repeat-motifs	Start physical positions (bp)	End physical positions (bp)											
TR44591|c0_g4_i1	No	(TA)6	12	--	--	--	--	--	--	GCATGCTGCTCTCTCTGCTA	60	TACCGACCGTATCGACCAGT	60	276	--	--	--	--	--	--
TR44635|c1_g1_i1	No	(CT)6	12	--	--	--	--	--	--	TTGCAGAAGCCAATTCCTCT	60	CAAGCAAAGAGGCTCAAGAAA	60	120	--	--	--	--	--	--
TR44635|c1_g1_i2	No	(CT)6	12	--	--	--	--	--	--	TTGCAGAAGCCAATTCCTCT	60	CAAGCAAAGAGGCTCAAGAAA	60	120	--	--	--	--	--	--
TR44769|c0_g1_i1	XLOC_017289	(CAA)6	18	TR44769|c0_g1_i1	(CAA)6	Ca_LG_1	(CAA)5	8656881	8656895	ATGGCAACACCAAAAAGGTC	60	CCAATCGCTTTGTTTCGTTT	60	244	Ca_20023	CDS	--	--	--	--
TR4485|c0_g1_i1	XLOC_017327	(GA)7	14	--	--	--	--	--	--	CAGCAAGCGGTAAAAAGACA	59	ATCTACCCCAGTGCCAACAC	60	254	--	--	--	--	--	--
TR44867|c0_g1_i1	No	(TAC)5	15	--	--	--	--	--	--	TTGAATTGCTTACAAAAGGCTG	59	TTATTGCATAATGCCCTGGA	59	162	--	--	--	--	--	--
TR44870|c0_g1_i1	XLOC_017332	(ATT)6	18	--	--	--	--	--	--	CGCGCAAATGAATTAAAACC	60	GGGCAGGAAACTGAATACGA	60	213	--	--	XP_001946617.2	--	[I]	--
TR44931|c0_g1_i1	XLOC_017360	(AT)7	14	--	--	--	--	--	--	CAAACCTAACAGTCCAAACCAA	59	GACGAGACCATCTTTGCCAT	60	261	--	--	XM_004512956.1	--	--	--
TR45056|c0_g1_i1	XLOC_017418	(AT)6	12	--	--	--	--	--	--	TTTTGTTTTGCCCTTTGTGA	59	CTCAAAGAGCTACGGCCAAC	60	246	--	--	XP_011254725.1	--	[K]	--
TR45077|c0_g1_i1	No	(AGC)5	15	--	--	--	--	--	--	TGCTACTGCCTCATCGTGTC	60	GATGATGTTGCTGTTGCTGG	60	196	--	--	--	--	--	--
TR45122|c0_g1_i1	No	(AGG)5	15	--	--	--	--	--	--	GCATGGCTTCTTAATATAATCAAACA	60	TGTTCTGACCCAAACCACAA	60	135	--	--	--	--	--	--
TR45146|c0_g1_i1	XLOC_017445	(CT)9aaatatatatttttattgggttaataactttgttactctctcgcactta(AT)6	79	--	--	--	--	--	--	ATAGAGCATGGCGTTTCACC	60	GGCCTTTAAATTGGGCTTTC	60	239	--	--	--	--	--	--
TR45192|c0_g1_i1	XLOC_017468	(CT)6	12	--	--	--	--	--	--	TTGCTCACGACCTGTTTACG	60	AGTCCCAAGGGCAAGAAAGT	60	130	--	--	KEP46081.1	--	--	--
TR45194|c0_g1_i1	XLOC_017469	(TA)6	12	--	--	--	--	--	--	CACCACATTTTCCTTGCCTT	60	GTTCACACAAACCCTTCAATAAT	57	134	--	--	--	--	--	--

Transcript IDs	Differentially expressed gene (DEG) IDs	SSR repeat-motifs	SSR repeat-length (bp)	*In silico* polymorphic SSRs						Forward primers (5'-3')	Annealing tempearture(0C)	Reverse primers (5'-3')	Annealing tempearture(0C)	Amplified product size (bp)	Structural annotation		Functional Annotation			
				C. microphyllum		ICC4958									Gene IDs	Sequence component of genes	NCBI nr IDs	TFs	KOG	KEGG
				Transcript	SSR repeat-motifs	Chromosomes	SSR repeat-motifs	Start physical positions (bp)	End physical positions (bp)											
TR45279|c0_g1_i1	No	(CT)7	14	--	--	--	--	--	--	GATGCTGTTGCTGTGCTGTT	60	AAACATCGTCATTTGGCTCC	60	139	--	--	--	--	--	--
TR4531|c0_g1_i1	XLOC_017522	(CTT)5	15	--	--	--	--	--	--	CGGCAACCTCCAATATCATC	60	GGCATGAGAGCCGTAGAATTT	61	161	--	--	XP_001949588.1	FAR1	[O]	--
TR45318|c0_g1_i1	XLOC_017521	(TCA)5	15	--	--	--	--	--	--	TTTTCTCTGTCTTCGGTGGC	60	TGGTTGGAGGAGGATTTCAG	60	196	--	--	--	--	--	--
TR4532|c0_g1_i1	XLOC_017526	(CAA)5	15	--	--	--	--	--	--	CCATGTTCCAATTCCAAACC	60	GGGTGTCGAAAAAGAGACCA	60	234	--	--	--	--	--	--
TR45380|c0_g2_i1	XLOC_017552	(ATG)5	15	--	--	--	--	--	--	ATTGATGGCAAAAGCAAGTG	59	GTGTTCTGTTGTGTGTGCCC	60	146	--	--	--	--	--	--
TR45380|c0_g2_i1	XLOC_017552	(TCT)5	15	--	--	--	--	--	--	CACCCCTAATCCATTACCCC	60	ATGACCAATTCATGGCCAAC	61	265	--	--	--	--	--	--
TR45380|c0_g3_i1	No	(ATG)5	15	--	--	--	--	--	--	ATTGATGGCAAAAGCAAGTG	59	GTGTTCTGTTGTGTGTGCCC	60	146	--	--	--	--	--	--
TR45380|c0_g3_i1	No	(TCT)5	15	--	--	--	--	--	--	GGGCACACACAACAGAACAC	60	GGGTTTAATTGGAATTGCAGAA	60	115	--	--	--	--	--	--
TR45381|c2_g1_i1	No	(CTT)6	18	--	--	--	--	--	--	AGCATGACCAGATCCCAATC	60	TTGAACATCAATGGCTTCCA	60	244	--	--	--	--	--	--
TR45387|c0_g1_i1	No	(TC)7	14	--	--	--	--	--	--	AACAAACGGCACTGCAATTA	59	GATCAGGTGCCAGTGAGGAT	60	110	--	--	--	--	--	--
TR45387|c0_g1_i10	No	(TC)7	14	--	--	--	--	--	--	AACAAACGGCACTGCAATTA	59	GATCAGGTGCCAGTGAGGAT	60	110	--	--	--	--	--	--
TR45387|c0_g1_i11	No	(TC)7	14	--	--	--	--	--	--	AACAAACGGCACTGCAATTA	59	GATCAGGTGCCAGTGAGGAT	60	110	--	--	--	--	--	--
TR45387|c0_g1_i12	No	(TC)7	14	--	--	--	--	--	--	AACAAACGGCACTGCAATTA	59	GATCAGGTGCCAGTGAGGAT	60	110	--	--	--	--	--	--
TR45387|c0_g1_i13	No	(TC)7	14	--	--	--	--	--	--	AACAAACGGCACTGCAATTA	59	GATCAGGTGCCAGTGAGGAT	60	110	--	--	--	--	--	--

Transcript IDs	Differentially expressed gene (DEG) IDs	SSR repeat-motifs	SSR repeat-length (bp)	*In silico* polymorphic SSRs						Forward primers (5'-3')	Annealing tempearture(0C)	Reverse primers (5'-3')	Annealing tempearture(0C)	Amplified product size (bp)	Structural annotation		Functional Annotation			
				C. microphyllum		ICC4958									Gene IDs	Sequence component of genes	NCBI nr IDs	TFs	KOG	KEGG
				Transcript	SSR repeat-motifs	Chromosomes	SSR repeat-motifs	Start physical positions (bp)	End physical positions (bp)											
TR45387|c0_g1_i14	No	(TC)7	14	--	--	--	--	--	--	AACAAACGGCACTGCAATTA	59	GATCAGGTGCCAGTGAGGAT	60	110	--	--	--	--	--	--
TR45387|c0_g1_i15	No	(TC)7	14	--	--	--	--	--	--	AACAAACGGCACTGCAATTA	59	GATCAGGTGCCAGTGAGGAT	60	110	--	--	--	--	--	--
TR45387|c0_g1_i16	No	(TC)7	14	--	--	--	--	--	--	AACAAACGGCACTGCAATTA	59	GATCAGGTGCCAGTGAGGAT	60	110	--	--	--	--	--	--
TR45387|c0_g1_i17	No	(TC)7	14	--	--	--	--	--	--	AACAAACGGCACTGCAATTA	59	GATCAGGTGCCAGTGAGGAT	60	110	--	--	--	--	--	--
TR45387|c0_g1_i2	No	(TC)7	14	--	--	--	--	--	--	AACAAACGGCACTGCAATTA	59	GATCAGGTGCCAGTGAGGAT	60	110	--	--	--	--	--	--
TR45387|c0_g1_i3	No	(TC)7	14	--	--	--	--	--	--	AACAAACGGCACTGCAATTA	59	GATCAGGTGCCAGTGAGGAT	60	110	--	--	--	--	--	--
TR45387|c0_g1_i4	No	(TC)7	14	--	--	--	--	--	--	AACAAACGGCACTGCAATTA	59	GATCAGGTGCCAGTGAGGAT	60	110	--	--	--	--	--	--
TR45387|c0_g1_i5	No	(TC)7	14	--	--	--	--	--	--	AACAAACGGCACTGCAATTA	59	GATCAGGTGCCAGTGAGGAT	60	110	--	--	--	--	--	--
TR45387|c0_g1_i6	No	(TC)7	14	--	--	--	--	--	--	AACAAACGGCACTGCAATTA	59	GATCAGGTGCCAGTGAGGAT	60	110	--	--	--	--	--	--
TR45387|c0_g1_i7	No	(TC)7	14	--	--	--	--	--	--	AACAAACGGCACTGCAATTA	59	GATCAGGTGCCAGTGAGGAT	60	110	--	--	--	--	--	--
TR45387|c0_g1_i8	No	(TC)7	14	--	--	--	--	--	--	AACAAACGGCACTGCAATTA	59	GATCAGGTGCCAGTGAGGAT	60	110	--	--	--	--	--	--
TR45387|c0_g1_i9	No	(TC)7	14	--	--	--	--	--	--	AACAAACGGCACTGCAATTA	59	GATCAGGTGCCAGTGAGGAT	60	110	--	--	--	--	--	--
TR45392|c0_g2_i1	XLOC_017570	(GA)7ataaagaaagagaaaacccacttgtacaaatttaattgttttcttatttcttttgttgcaaaagctgtacaaattcaatttaag(T)11	109	--	--	--	--	--	--	GGACAAGAAAAGGTGCACAAA	60	TAAGGGCCAAGTAGAGACGC	59	219	--	--	--	--	--	--
TR45392|c1_g1_i1	XLOC_017571	(CTT)6	18	--	--	--	--	--	--	CCAGCCAAAGAGAAACGAAG	60	ATGGGATTGATGACGTGGAT	60	247	--	--	--	--	--	--

Transcript IDs	Differentially expressed gene (DEG) IDs	SSR repeat-motifs	SSR repeat-length (bp)	*In silico* polymorphic SSRs						Forward primers (5'-3')	Annealing tempearture(0C)	Reverse primers (5'-3')	Annealing tempearture(0C)	Amplified product size (bp)	Structural annotation		Functional Annotation			
				C. microphyllum		ICC4958														
				Transcript	SSR repeat-motifs	Chromosomes	SSR repeat-motifs	Start physical positions (bp)	End physical positions (bp)						Gene IDs	Sequence component of genes	NCBI nr IDs	TFs	KOG	KEGG
TR45392|c4_g1_i1	XLOC_017573	(CTT)7	21	--	--	--	--	--	--	GGCTTCCCATGATCTTGTTT	59	ACCTCAAGCGAAATGTGGAT	60	232	--	--	--	--	--	--
TR45393|c5_g1_i1	XLOC_017578	(TCT)7	21	TR45393|c5_g1_i1	(TCT)7	Ca_LG_5	(TCT)5	25471309	25471323	CTCTCAACCACTCCACCACC	61	TGCATCTCCTGGATTATTAGCA	60	247	Ca_08299	CDS	--	--	--	--
TR45393|c5_g1_i1	XLOC_017578	(CAA)7	21	TR45393|c5_g1_i1	(TCT)7	Ca_LG_5	(TCT)5	25471309	25471323	TCCAACAATCTTCAAACCCC	60	TGGTGGTGGAGTGGTTGAG	61	220	Ca_08299	CDS	--	--	--	--
TR45394|c0_g1_i1	No	(CT)8ataattatattcacattctca(TC)6	49	--	--	--	--	--	--	TATCTTCCACCTGTACCCGC	60	CAATACGGCATCGTTGTGAG	60	159	--	--	--	--	--	--
TR45394|c0_g1_i10	No	(CT)8ataattatattcacattctca(TC)6	49	--	--	--	--	--	--	TATCTTCCACCTGTACCCGC	60	CAATACGGCATCGTTGTGAG	60	159	--	--	--	--	--	--
TR45394|c0_g1_i11	No	(CT)8ataattatattcacattctca(TC)6	49	--	--	--	--	--	--	TATCTTCCACCTGTACCCGC	60	CAATACGGCATCGTTGTGAG	60	159	--	--	--	--	--	--
TR45394|c0_g1_i12	No	(CT)8ataattatattcacattctca(TC)6	49	--	--	--	--	--	--	TATCTTCCACCTGTACCCGC	60	CAATACGGCATCGTTGTGAG	60	159	--	--	--	--	--	--
TR45394|c0_g1_i14	No	(CT)8ataattatattcacattctca(TC)6	49	--	--	--	--	--	--	TATCTTCCACCTGTACCCGC	60	CAATACGGCATCGTTGTGAG	60	159	--	--	--	--	--	--
TR45394|c0_g1_i15	No	(CT)8ataattatattcacattctca(TC)6	49	--	--	--	--	--	--	TATCTTCCACCTGTACCCGC	60	CAATACGGCATCGTTGTGAG	60	159	--	--	--	--	--	--
TR45394|c0_g1_i16	No	(CT)8ataattatattcacattctca(TC)6	49	--	--	--	--	--	--	TATCTTCCACCTGTACCCGC	60	CAATACGGCATCGTTGTGAG	60	159	--	--	--	--	--	--
TR45394|c0_g1_i17	No	(CT)8ataattatattcacattctca(TC)6	49	--	--	--	--	--	--	TATCTTCCACCTGTACCCGC	60	CAATACGGCATCGTTGTGAG	60	159	--	--	--	--	--	--
TR45394|c0_g1_i18	No	(CT)8ataattatattcacattctca(TC)6	49	--	--	--	--	--	--	TATCTTCCACCTGTACCCGC	60	CAATACGGCATCGTTGTGAG	60	159	--	--	--	--	--	--
TR45394|c0_g1_i19	No	(CT)8ataattatattcacattctca(TC)6	49	--	--	--	--	--	--	TATCTTCCACCTGTACCCGC	60	CAATACGGCATCGTTGTGAG	60	159	--	--	--	--	--	--
TR45394|c0_g1_i2	No	(CT)8ataattatattcacattctca(TC)6	49	--	--	--	--	--	--	TATCTTCCACCTGTACCCGC	60	CAATACGGCATCGTTGTGAG	60	159	--	--	--	--	--	--

Transcript IDs	Differentially expressed gene (DEG) IDs	SSR repeat-motifs	SSR repeat-length (bp)	*In silico* polymorphic SSRs						Forward primers (5'-3')	Annealing tempearture(0C)	Reverse primers (5'-3')	Annealing tempearture(0C)	Amplified product size (bp)	Structural annotation		Functional Annotation			
				C. microphyllum		ICC4958									Gene IDs	Sequence component of genes	NCBI nr IDs	TFs	KOG	KEGG
				Transcript	SSR repeat-motifs	Chromosomes	SSR repeat-motifs	Start physical positions (bp)	End physical positions (bp)											
TR45394|c0_g1_i20	No	(CT)8ataattatattcacattctca(TC)6	49	--	--	--	--	--	--	TATCTTCCACCTGTACCCGC	60	CAATACGGCATCGTTGTGAG	60	159	--	--	--	--	--	--
TR45394|c0_g1_i21	No	(CT)8ataattatattcacattctca(TC)6	49	--	--	--	--	--	--	TATCTTCCACCTGTACCCGC	60	CAATACGGCATCGTTGTGAG	60	159	--	--	--	--	--	--
TR45394|c0_g1_i22	No	(CT)8ataattatattcacattctca(TC)6	49	--	--	--	--	--	--	TATCTTCCACCTGTACCCGC	60	CAATACGGCATCGTTGTGAG	60	159	--	--	--	--	--	--
TR45394|c0_g1_i24	No	(CT)8ataattatattcacattctca(TC)6	49	--	--	--	--	--	--	TATCTTCCACCTGTACCCGC	60	CAATACGGCATCGTTGTGAG	60	159	--	--	--	--	--	--
TR45394|c0_g1_i3	No	(CT)8ataattatattcacattctca(TC)6	49	--	--	--	--	--	--	TATCTTCCACCTGTACCCGC	60	CAATACGGCATCGTTGTGAG	60	159	--	--	--	--	--	--
TR45394|c0_g1_i4	No	(CT)8ataattatattcacattctca(TC)6	49	--	--	--	--	--	--	TATCTTCCACCTGTACCCGC	60	CAATACGGCATCGTTGTGAG	60	159	--	--	--	--	--	--
TR45394|c0_g1_i5	No	(CT)8ataattatattcacattctca(TC)6	49	--	--	--	--	--	--	TATCTTCCACCTGTACCCGC	60	CAATACGGCATCGTTGTGAG	60	159	--	--	--	--	--	--
TR45394|c0_g1_i6	No	(CT)8ataattatattcacattctca(TC)6	49	--	--	--	--	--	--	TATCTTCCACCTGTACCCGC	60	CAATACGGCATCGTTGTGAG	60	159	--	--	--	--	--	--
TR45394|c0_g1_i7	No	(CT)8ataattatattcacattctca(TC)6	49	--	--	--	--	--	--	TATCTTCCACCTGTACCCGC	60	CAATACGGCATCGTTGTGAG	60	159	--	--	--	--	--	--
TR45394|c0_g1_i9	No	(CT)8ataattatattcacattctca(TC)6	49	--	--	--	--	--	--	TATCTTCCACCTGTACCCGC	60	CAATACGGCATCGTTGTGAG	60	159	--	--	--	--	--	--
TR45400|c0_g1_i1	XLOC_017583	(TCA)5	15	--	--	--	--	--	--	TGCTTCCCGTCTTCACTTCT	60	TCCTCGATGGTTGTGTGGTA	60	214	--	--	--	--	--	--
TR45401|c1_g6_i1	XLOC_017589	(ATA)7	21	--	--	--	--	--	--	TCAGCTGCAAATTGAGTGAGAT	60	AACCGGCAAGTCACAAAAAG	60	227	--	--	--	--	--	--
TR45403|c3_g1_i1	No	(AAG)5	15	--	--	--	--	--	--	CACGAACATGGAGACACACC	60	CGCTGGAAGAAGGAATTTTG	60	228	--	--	--	--	--	--
TR45403|c3_g2_i1	No	(AAG)5	15	--	--	--	--	--	--	CACGAACATGGAGACACACC	60	CGCTGGAAGAAGGAATTTTG	60	228	--	--	--	--	--	--

Transcript IDs	Differentially expressed gene (DEG) IDs	SSR repeat-motifs	SSR repeat-length (bp)	*In silico* polymorphic SSRs						Forward primers (5'-3')	Annealing tempearture(0C)	Reverse primers (5'-3')	Annealing tempearture(0C)	Amplified product size (bp)	Structural annotation		Functional Annotation			
				C. microphyllum		ICC4958									Gene IDs	Sequence component of genes	NCBI nr IDs	TFs	KOG	KEGG
				Transcript	SSR repeat-motifs	Chromosomes	SSR repeat-motifs	Start physical positions (bp)	End physical positions (bp)											
TR45406|c0_g1_i1	No	(TC)12	24	--	--	--	--	--	--	TACTCTTCCGACGCGAAACT	60	AGGCTCGGAAGGATTGTCTT	60	260	--	--	--	--	--	--
TR45406|c0_g2_i1	No	(TC)12	24	--	--	--	--	--	--	TACTCTTCCGACGCGAAACT	60	AGGCTCGGAAGGATTGTCTT	60	260	--	--	--	--	--	--
TR45406|c3_g1_i1	No	(GCA)5	15	--	--	--	--	--	--	CCAAAAACTCGAAACGGAAA	60	CCACAAAACGACATCGTCAC	60	241	--	--	--	--	--	--
TR45406|c3_g1_i1	No	(CT)9	18	--	--	--	--	--	--	GTCATGACCACACCCAATCA	60	TGGGAGGTGAATTGAGGAAG	60	232	--	--	--	--	--	--
TR45406|c3_g1_i2	No	(GCA)5	15	--	--	--	--	--	--	CCAAAAACTCGAAACGGAAA	60	CCACAAAACGACATCGTCAC	60	241	--	--	--	--	--	--
TR45406|c4_g1_i1	XLOC_017602	(TG)6	12	--	--	--	--	--	--	TTCAAAGCAGGGTCCTCACT	60	ATACCACCCTCCCACTTTCC	60	193	--	--	--	--	--	--
TR45406|c4_g1_i2	No	(TG)6	12	--	--	--	--	--	--	TTCAAAGCAGGGTCCTCACT	60	ATACCACCCTCCCACTTTCC	60	193	--	--	--	--	--	--
TR45406|c4_g1_i3	No	(TG)6	12	--	--	--	--	--	--	TTCAAAGCAGGGTCCTCACT	60	ATACCACCCTCCCACTTTCC	60	193	--	--	--	--	--	--
TR45406|c4_g1_i4	No	(TG)6	12	--	--	--	--	--	--	TTCAAAGCAGGGTCCTCACT	60	ATACCACCCTCCCACTTTCC	60	193	--	--	--	--	--	--
TR45406|c4_g1_i5	No	(TG)6	12	--	--	--	--	--	--	TTCAAAGCAGGGTCCTCACT	60	ATACCACCCTCCCACTTTCC	60	193	--	--	--	--	--	--
TR45406|c4_g1_i6	No	(TG)6	12	--	--	--	--	--	--	TTCAAAGCAGGGTCCTCACT	60	ATACCACCCTCCCACTTTCC	60	193	--	--	--	--	--	--
TR45406|c7_g1_i1	XLOC_017605	(AG)11	22	--	--	--	--	--	--	GGAATGGTTTGATTTGGTCC	59	TCTTTGAAATCCTTTTGTTTTTGA	59	158	--	--	--	--	--	--
TR45414|c2_g1_i2	No	(TA)7	14	--	--	--	--	--	--	ATCCCCTCTGCTACCCTGTT	60	TATGTTGTGTTGGTCCACCG	60	252	--	--	--	--	--	--
TR45414|c2_g1_i8	No	(TA)7	14	--	--	--	--	--	--	ATCCCCTCTGCTACCCTGTT	60	TATGTTGTGTTGGTCCACCG	60	252	--	--	--	--	--	--

Transcript IDs	Differentially expressed gene (DEG) IDs	SSR repeat-motifs	SSR repeat-length (bp)	*In silico* polymorphic SSRs						Forward primers (5'-3')	Annealing tempearture(0C)	Reverse primers (5'-3')	Annealing tempearture(0C)	Amplified product size (bp)	Structural annotation		Functional Annotation			
				C. microphyllum		ICC4958														
				Transcript	SSR repeat-motifs	Chromosomes	SSR repeat-motifs	Start physical positions (bp)	End physical positions (bp)						Gene IDs	Sequence component of genes	NCBI nr IDs	TFs	KOG	KEGG
TR45415|c2_g1_i1	No	(AT)7	14	--	--	--	--	--	--	ACACAGAGATGGGAATTGGC	60	CGAGGATCTCTGGGAAATGA	60	264	--	--	--	--	--	--
TR45415|c2_g2_i1	No	(AT)7	14	--	--	--	--	--	--	ACACAGAGATGGGAATTGGC	60	CGAGGATCTCTGGGAAATGA	60	264	--	--	--	--	--	--
TR45418|c2_g3_i1	No	(AGA)6	18	--	--	--	--	--	--	CGAAACAGAAGGCGTTTAGG	60	TTCTTGTGCAAGTCATTGGC	60	192	--	--	--	--	--	--
TR45423|c0_g1_i2	No	(TTC)5	15	TR45423|c0_g1_i2	(TTC)5	Ca_LG_6	(TTC)10	15327746	15327775	ATTCTGCATCAACCACACCA	60	GTGGGCCAGTACGAATGAGT	60	278	Ca_14134	CDS	--	--	--	--
TR45423|c0_g1_i5	No	(TTC)5	15	--	--	--	--	--	--	ATTCTGCATCAACCACACCA	60	GTGGGCCAGTACGAATGAGT	60	278	--	--	--	--	--	--
TR45423|c0_g1_i6	No	(TTC)5	15	--	--	--	--	--	--	ATTCTGCATCAACCACACCA	60	GTGGGCCAGTACGAATGAGT	60	278	--	--	--	--	--	--
TR45423|c0_g1_i8	No	(TTC)5	15	--	--	--	--	--	--	ATTCTGCATCAACCACACCA	60	GTGGGCCAGTACGAATGAGT	60	278	--	--	--	--	--	--
TR45431|c2_g1_i1	No	(TTC)6	18	--	--	--	--	--	--	GCGACTGTGTGTATGTTGAGC	59	TGTCACCCTGATTGATGATTG	59	262	--	--	--	--	--	--
TR45431|c2_g1_i2	No	(TTC)6	18	--	--	--	--	--	--	GCGACTGTGTGTATGTTGAGC	59	TGTCACCCTGATTGATGATTG	59	262	--	--	--	--	--	--
TR45431|c2_g1_i3	No	(TTC)6	18	--	--	--	--	--	--	GCGACTGTGTGTATGTTGAGC	59	TGTCACCCTGATTGATGATTG	59	262	--	--	--	--	--	--
TR45432|c1_g2_i1	No	(TAT)5	15	--	--	--	--	--	--	CTTCTTTGCAGGGGAGAGTG	60	TCTCACGCTATATGATGAGCAC	58	100	--	--	--	--	--	--
TR45436|c2_g2_i1	No	(AAT)8	24	--	--	--	--	--	--	GCAAGGTTCCTTCCTTCCTT	60	TGTCTAAAGAGGCAACAGCG	59	258	--	--	--	--	--	--
TR45436|c2_g2_i10	No	(TC)7	14	--	--	--	--	--	--	ATGGTTGGGCGATGAAATAA	60	CGATCTCCCCTGTTGAAAAA	60	216	--	--	--	--	--	--
TR45436|c2_g2_i11	No	(TC)7	14	--	--	--	--	--	--	ATGGTTGGGCGATGAAATAA	60	CGATCTCCCCTGTTGAAAAA	60	216	--	--	--	--	--	--

Transcript IDs	Differentially expressed gene (DEG) IDs	SSR repeat-motifs	SSR repeat-length (bp)	*In silico* polymorphic SSRs						Forward primers (5'-3')	Annealing tempearture(0C)	Reverse primers (5'-3')	Annealing tempearture(0C)	Amplified product size (bp)	Structural annotation		Functional Annotation			
				C. microphyllum		ICC4958														
				Transcript	SSR repeat-motifs	Chromosomes	SSR repeat-motifs	Start physical positions (bp)	End physical positions (bp)						Gene IDs	Sequence component of genes	NCBI nr IDs	TFs	KOG	KEGG
TR45436|c2_g2_i12	No	(AAT)8	24	--	--	--	--	--	--	GCAAGGTTCCTTCCTTCCTT	60	TGTCTAAAGAGGCAACAGCG	59	258	--	--	--	--	--	--
TR45436|c2_g2_i13	No	(TC)7	14	--	--	--	--	--	--	ATGGTTGGGCGATGAAATAA	60	CGATCTCCCCTGTTGAAAAA	60	216	--	--	--	--	--	--
TR45436|c2_g2_i2	No	(TC)7	14	--	--	--	--	--	--	ATGGTTGGGCGATGAAATAA	60	CGATCTCCCCTGTTGAAAAA	60	216	--	--	--	--	--	--
TR45436|c2_g2_i3	XLOC_017674	(AAT)8	24	--	--	--	--	--	--	GCAAGGTTCCTTCCTTCCTT	60	TGTCTAAAGAGGCAACAGCG	59	258	--	--	--	--	--	--
TR45436|c2_g2_i4	No	(TC)7	14	--	--	--	--	--	--	ATGGTTGGGCGATGAAATAA	60	CGATCTCCCCTGTTGAAAAA	60	216	--	--	--	--	--	--
TR45436|c2_g2_i5	No	(TC)7	14	--	--	--	--	--	--	ATGGTTGGGCGATGAAATAA	60	CGATCTCCCCTGTTGAAAAA	60	216	--	--	--	--	--	--
TR45436|c2_g2_i6	No	(AAT)8	24	--	--	--	--	--	--	GCAAGGTTCCTTCCTTCCTT	60	TGTCTAAAGAGGCAACAGCG	59	258	--	--	--	--	--	--
TR45436|c2_g2_i7	No	(TC)7	14	--	--	--	--	--	--	ATGGTTGGGCGATGAAATAA	60	CGATCTCCCCTGTTGAAAAA	60	216	--	--	--	--	--	--
TR45436|c2_g2_i8	No	(AAT)8	24	--	--	--	--	--	--	GCAAGGTTCCTTCCTTCCTT	60	TGTCTAAAGAGGCAACAGCG	59	258	--	--	--	--	--	--
TR45436|c2_g2_i9	No	(TC)7	14	--	--	--	--	--	--	ATGGTTGGGCGATGAAATAA	60	CGATCTCCCCTGTTGAAAAA	60	216	--	--	--	--	--	--
TR45436|c2_g3_i1	No	(TC)7	14	--	--	--	--	--	--	ATGGTTGGGCGATGAAATAA	60	CCGGGGAACTTGATGATAGA	60	169	--	--	--	--	--	--
TR45438|c0_g1_i1	No	(GAA)5	15	--	--	--	--	--	--	GTTAACAATGGTTGGGGGTG	60	ACCGATTTGGAATTGGATGA	60	150	--	--	--	--	--	--
TR45438|c0_g1_i2	No	(GAA)5	15	--	--	--	--	--	--	GTTAACAATGGTTGGGGGTG	60	ACCGATTTGGAATTGGATGA	60	150	--	--	--	--	--	--
TR45438|c0_g1_i3	No	(GAA)5	15	--	--	--	--	--	--	GTTAACAATGGTTGGGGGTG	60	ACCGATTTGGAATTGGATGA	60	150	--	--	--	--	--	--

Transcript IDs	Differentially expressed gene (DEG) IDs	SSR repeat-motifs	SSR repeat-length (bp)	*In silico* polymorphic SSRs						Forward primers (5'-3')	Annealing tempearture(0C)	Reverse primers (5'-3')	Annealing tempearture(0C)	Amplified product size (bp)	Structural annotation		Functional Annotation			
				C. microphyllum		ICC4958									Gene IDs	Sequence component of genes	NCBI nr IDs	TFs	KOG	KEGG
				Transcript	SSR repeat-motifs	Chromosomes	SSR repeat-motifs	Start physical positions (bp)	End physical positions (bp)											
TR45438|c0_g1_i4	No	(GAA)5	15	--	--	--	--	--	--	GTTAACAATGGTTGGGGGTG	60	ACCGATTTGGAATTGGATGA	60	150	--	--	--	--	--	--
TR45442|c0_g1_i1	No	(AG)7	14	--	--	--	--	--	--	ACCTCATCCGTCTCCTGTTG	60	CGCCTTCTTCTCTGGTTTTG	60	225	--	--	--	--	--	--
TR45442|c0_g1_i2	No	(AG)7	14	--	--	--	--	--	--	ACCTCATCCGTCTCCTGTTG	60	CGCCTTCTTCTCTGGTTTTG	60	225	--	--	--	--	--	--
TR45442|c0_g1_i4	No	(AG)7	14	--	--	--	--	--	--	ACCTCATCCGTCTCCTGTTG	60	CGCCTTCTTCTCTGGTTTTG	60	225	--	--	--	--	--	--
TR45444|c1_g2_i3	XLOC_017696	(TCT)6	18	--	--	--	--	--	--	CTCTCTTCTCTTTGCATCACCA	60	CAATTCCATTTGCGACACTG	60	227	--	--	--	--	--	--
TR45444|c1_g2_i6	No	(TCT)6	18	--	--	--	--	--	--	CTCTCTTCTCTTTGCATCACCA	60	CAATTCCATTTGCGACACTG	60	227	--	--	--	--	--	--
TR45444|c1_g2_i8	No	(TCT)6	18	--	--	--	--	--	--	CTCTCTTCTCTTTGCATCACCA	60	CAATTCCATTTGCGACACTG	60	227	--	--	--	--	--	--
TR45446|c0_g1_i1	No	(AT)6	12	--	--	--	--	--	--	ACTGGTTATGCCTATTGCGG	60	AACGTTTCATTGGCAACCAT	60	260	--	--	--	--	--	--
TR45446|c0_g2_i1	XLOC_017699	(AT)6	12	--	--	--	--	--	--	ACTGGTTATGCCTATTGCGG	60	AACGTTTCATTGGCAACCAT	60	260	--	--	--	--	--	--
TR45460|c0_g1_i1	No	(CT)8	16	--	--	--	--	--	--	TCTGTCACAACGCCATCATC	61	GTTCCATCCCCTAACCGAAT	60	253	--	--	--	--	--	--
TR45460|c0_g1_i2	No	(CT)8	16	--	--	--	--	--	--	GATCTGTCACAACGCCATCA	61	ACGCAACTTGGGAACAAAAG	60	159	--	--	--	--	--	--
TR45460|c0_g1_i4	No	(CT)8	16	--	--	--	--	--	--	TCTGTCACAACGCCATCATC	61	GTTCCATCCCCTAACCGAAT	60	253	--	--	--	--	--	--
TR45467|c1_g1_i1	XLOC_017753	(CT)7	14	--	--	--	--	--	--	TGAAAGAGCCCTGAAAATGG	60	TGGTATTGCAAACATCGCTT	59	270	--	--	--	--	--	--
TR45467|c1_g1_i2	No	(CT)7	14	--	--	--	--	--	--	GCGAACCCTAAATCAAACGA	60	CGCTTCTGCCTCTTTCTGTT	60	103	--	--	--	--	--	--

<table>
<tr><th rowspan="3">Transcript IDs</th><th rowspan="3">Differentially expressed gene (DEG) IDs</th><th rowspan="3">SSR repeat-motifs</th><th rowspan="3">SSR repeat-length (bp)</th><th colspan="6">In silico polymorphic SSRs</th><th rowspan="3">Forward primers (5'-3')</th><th rowspan="3">Annealing tempearture(0C)</th><th rowspan="3">Reverse primers (5'-3')</th><th rowspan="3">Annealing tempearture(0C)</th><th rowspan="3">Amplified product size (bp)</th><th colspan="2">Structural annotation</th><th colspan="4">Functional Annotation</th></tr>
<tr><th colspan="2">C. microphyllum</th><th colspan="4">ICC4958</th><th rowspan="2">Gene IDs</th><th rowspan="2">Sequence component of genes</th><th rowspan="2">NCBI nr IDs</th><th rowspan="2">TFs</th><th rowspan="2">KOG</th><th rowspan="2">KEGG</th></tr>
<tr><th>Transcript</th><th>SSR repeat-motifs</th><th>Chromosomes</th><th>SSR repeat-motifs</th><th>Start physical positions (bp)</th><th>End physical positions (bp)</th></tr>
<tr><td>TR45472|c0_g1_i3</td><td>No</td><td>(TCA)5</td><td>15</td><td>--</td><td>--</td><td>--</td><td>--</td><td>--</td><td>--</td><td>TGCTCTTCGTGTTTGAAAGC</td><td>59</td><td>TCACTCATCGATATGCCCAA</td><td>60</td><td>182</td><td>--</td><td>--</td><td>--</td><td>--</td><td>--</td><td>--</td></tr>
<tr><td>TR45472|c0_g1_i5</td><td>No</td><td>(TCA)5</td><td>15</td><td>--</td><td>--</td><td>--</td><td>--</td><td>--</td><td>--</td><td>TGCTCTTCGTGTTTGAAAGC</td><td>59</td><td>TCACTCATCGATATGCCCAA</td><td>60</td><td>182</td><td>--</td><td>--</td><td>--</td><td>--</td><td>--</td><td>--</td></tr>
<tr><td>TR45476|c1_g1_i1</td><td>XLOC_017766</td><td>(ATC)8</td><td>24</td><td>--</td><td>--</td><td>--</td><td>--</td><td>--</td><td>--</td><td>TGCATTGCAGAGGAAAAATG</td><td>60</td><td>GATGAAGGGAGACAAGCCAA</td><td>60</td><td>280</td><td>--</td><td>--</td><td>--</td><td>--</td><td>--</td><td>--</td></tr>
<tr><td>TR45476|c1_g4_i1</td><td>No</td><td>(ATC)9</td><td>27</td><td>--</td><td>--</td><td>--</td><td>--</td><td>--</td><td>--</td><td>TTCACAAACCAACAACACAACA</td><td>60</td><td>CGGGGACGACTTTATTGAGA</td><td>60</td><td>269</td><td>--</td><td>--</td><td>--</td><td>--</td><td>--</td><td>--</td></tr>
<tr><td>TR45476|c1_g4_i2</td><td>No</td><td>(ATC)9</td><td>27</td><td>--</td><td>--</td><td>--</td><td>--</td><td>--</td><td>--</td><td>TTCACAAACCAACAACACAACA</td><td>60</td><td>CGGGGACGACTTTATTGAGA</td><td>60</td><td>269</td><td>--</td><td>--</td><td>--</td><td>--</td><td>--</td><td>--</td></tr>
<tr><td>TR45476|c1_g4_i5</td><td>No</td><td>(ATC)9</td><td>27</td><td>--</td><td>--</td><td>--</td><td>--</td><td>--</td><td>--</td><td>TTCACAAACCAACAACACAACA</td><td>60</td><td>CGGGGACGACTTTATTGAGA</td><td>60</td><td>269</td><td>--</td><td>--</td><td>--</td><td>--</td><td>--</td><td>--</td></tr>
<tr><td>TR45476|c2_g1_i1</td><td>XLOC_017768</td><td>(TGA)7</td><td>21</td><td>--</td><td>--</td><td>--</td><td>--</td><td>--</td><td>--</td><td>GCAGCTATGGTTTTGGGAAA</td><td>60</td><td>GTACCCAAATCGCGAATGTC</td><td>60</td><td>153</td><td>--</td><td>--</td><td>--</td><td>--</td><td>--</td><td>--</td></tr>
<tr><td>TR4548|c0_g1_i1</td><td>XLOC_017775</td><td>(TAA)6</td><td>18</td><td>--</td><td>--</td><td>--</td><td>--</td><td>--</td><td>--</td><td>TTGCTGAAAGAAAATATACAAGTACAA</td><td>58</td><td>CGTTTGATGCAGGTTTGTGA</td><td>61</td><td>278</td><td>--</td><td>--</td><td>XM_004503324.1</td><td>NAC</td><td>[M]</td><td>K01051</td></tr>
<tr><td>TR45501|c0_g1_i1</td><td>XLOC_017780</td><td>(TGC)5</td><td>15</td><td>--</td><td>--</td><td>--</td><td>--</td><td>--</td><td>--</td><td>AGTCCGCGGAAGTTTTGTT</td><td>60</td><td>ACACCCACAGGTCAAAGAGC</td><td>60</td><td>178</td><td>--</td><td>--</td><td>CDH56319.1</td><td>--</td><td>[R]</td><td>--</td></tr>
<tr><td>TR45749|c0_g1_i1</td><td>XLOC_017853</td><td>(A)15ccaaagttctccattgtatcatcaatcgatttacc(A)11</td><td>61</td><td>--</td><td>--</td><td>--</td><td>--</td><td>--</td><td>--</td><td>TCTCTCACTGGAGATGCTTTCA</td><td>60</td><td>CGCCTTGTCTTCAGAGAACC</td><td>60</td><td>273</td><td>--</td><td>--</td><td>--</td><td>--</td><td>--</td><td>--</td></tr>
<tr><td>TR4578|c0_g1_i1</td><td>No</td><td>(TG)7</td><td>14</td><td>--</td><td>--</td><td>--</td><td>--</td><td>--</td><td>--</td><td>TGTACGAGTCAAGCGACGAG</td><td>60</td><td>CCTCCACTCGACGAACACTT</td><td>60</td><td>156</td><td>--</td><td>--</td><td>--</td><td>--</td><td>--</td><td>--</td></tr>
<tr><td>TR45855|c0_g1_i1</td><td>XLOC_017893</td><td>(GCG)5</td><td>15</td><td>--</td><td>--</td><td>--</td><td>--</td><td>--</td><td>--</td><td>TGAGAGTGTTGTTGGCCTTG</td><td>60</td><td>TCAAAGCATCTCAACCCACA</td><td>60</td><td>186</td><td>--</td><td>--</td><td>EWZ31996.1</td><td>--</td><td>[B]</td><td>--</td></tr>
<tr><td>TR45923|c0_g1_i1</td><td>No</td><td>(ACA)5</td><td>15</td><td>--</td><td>--</td><td>--</td><td>--</td><td>--</td><td>--</td><td>TCACAGGATGATGTGTGCTTC</td><td>60</td><td>TGCATGTTGTTGTGGTGATG</td><td>60</td><td>129</td><td>--</td><td>--</td><td>--</td><td>--</td><td>--</td><td>--</td></tr>
<tr><td>TR45976|c0_g1_i1</td><td>XLOC_017917</td><td>(CAC)6</td><td>18</td><td>--</td><td>--</td><td>--</td><td>--</td><td>--</td><td>--</td><td>TTCCCTTCCTTGCAAATCAC</td><td>60</td><td>TTGTTGGAAGTGGGAATGAA</td><td>59</td><td>119</td><td>--</td><td>--</td><td>--</td><td>--</td><td>--</td><td>--</td></tr>
</table>

<table>
<tr><th rowspan="3">Transcript IDs</th><th rowspan="3">Differentially expressed gene (DEG) IDs</th><th rowspan="3">SSR repeat-motifs</th><th rowspan="3">SSR repeat-length (bp)</th><th colspan="6">In silico polymorphic SSRs</th><th rowspan="3">Forward primers (5'-3')</th><th rowspan="3">Annealing tempearture(0C)</th><th rowspan="3">Reverse primers (5'-3')</th><th rowspan="3">Annealing tempearture(0C)</th><th rowspan="3">Amplified product size (bp)</th><th colspan="2">Structural annotation</th><th colspan="4">Functional Annotation</th></tr>
<tr><th colspan="2">C. microphyllum</th><th colspan="4">ICC4958</th><th rowspan="2">Gene IDs</th><th rowspan="2">Sequence component of genes</th><th rowspan="2">NCBI nr IDs</th><th rowspan="2">TFs</th><th rowspan="2">KOG</th><th rowspan="2">KEGG</th></tr>
<tr><th>Transcript</th><th>SSR repeat-motifs</th><th>Chromosomes</th><th>SSR repeat-motifs</th><th>Start physical positions (bp)</th><th>End physical positions (bp)</th></tr>
<tr><td>TR45988|c0_g1_i1</td><td>No</td><td>(CT)8</td><td>16</td><td>TR45988|c0_g1_i1</td><td>(CT)8</td><td>Ca_LG_5</td><td>(CT)6</td><td>44819957</td><td>44819968</td><td>GCAAAGGCTGACCTTGAAGA</td><td>61</td><td>ACAAAACTTCCTGTGGCGTC</td><td>60</td><td>193</td><td>Ca_07716</td><td>URR</td><td>--</td><td>--</td><td>--</td><td>--</td></tr>
<tr><td>TR46005|c0_g1_i1</td><td>XLOC_017925</td><td>(ACA)5</td><td>15</td><td>--</td><td>--</td><td>--</td><td>--</td><td>--</td><td>--</td><td>GCAGCTTCAACAACAACAGTCT</td><td>60</td><td>CGCTGCCTCAATTTCTCTCT</td><td>60</td><td>183</td><td>--</td><td>--</td><td>--</td><td>--</td><td>--</td><td>--</td></tr>
<tr><td>TR46030|c0_g1_i1</td><td>No</td><td>(GT)8</td><td>16</td><td>--</td><td>--</td><td>--</td><td>--</td><td>--</td><td>--</td><td>GGGAAGAGGAGATTTCGGTC</td><td>60</td><td>TGGAATTCACAATGCCTTCA</td><td>60</td><td>167</td><td>--</td><td>--</td><td>--</td><td>--</td><td>--</td><td>--</td></tr>
<tr><td>TR46069|c0_g1_i1</td><td>No</td><td>(AT)10</td><td>20</td><td>--</td><td>--</td><td>--</td><td>--</td><td>--</td><td>--</td><td>TGAGGTTTGATTTCATTATATTTCTTT</td><td>58</td><td>TCCATTTGGCACGTCAACTA</td><td>60</td><td>200</td><td>--</td><td>--</td><td>--</td><td>--</td><td>--</td><td>--</td></tr>
<tr><td>TR46116|c0_g1_i1</td><td>XLOC_017945</td><td>(A)12gtaagaactttctttcagcgctcaagacaaaagc(A)10</td><td>56</td><td>--</td><td>--</td><td>--</td><td>--</td><td>--</td><td>--</td><td>TGTTGTGCTTTGACGAGACC</td><td>60</td><td>CTTGCAATCGGGAAACATCT</td><td>60</td><td>190</td><td>--</td><td>--</td><td>--</td><td>--</td><td>--</td><td>--</td></tr>
<tr><td>TR46135|c0_g2_i1</td><td>XLOC_017959</td><td>(AG)6</td><td>12</td><td>--</td><td>--</td><td>--</td><td>--</td><td>--</td><td>--</td><td>AAACATTGCTCACACAAAAACA</td><td>58</td><td>ACTCTTGCATGCCATCATTG</td><td>60</td><td>136</td><td>--</td><td>--</td><td>--</td><td>--</td><td>--</td><td>--</td></tr>
<tr><td>TR46135|c0_g2_i2</td><td>No</td><td>(AG)6</td><td>12</td><td>--</td><td>--</td><td>--</td><td>--</td><td>--</td><td>--</td><td>AAACATTGCTCACACAAAAACA</td><td>58</td><td>ACTCTTGCATGCCATCATTG</td><td>60</td><td>240</td><td>--</td><td>--</td><td>--</td><td>--</td><td>--</td><td>--</td></tr>
<tr><td>TR46137|c0_g2_i1</td><td>XLOC_017960</td><td>(TAT)6</td><td>18</td><td>--</td><td>--</td><td>--</td><td>--</td><td>--</td><td>--</td><td>CTCTCTCCCACCCCCTTTAC</td><td>60</td><td>TGGTCATTGTGAATCTGGGA</td><td>60</td><td>249</td><td>--</td><td>--</td><td>XM_004502512.1</td><td>--</td><td>[S]</td><td>--</td></tr>
<tr><td>TR46162|c0_g1_i1</td><td>No</td><td>(AG)7aaagaggtgaagtctcaactcaagtgtgtgaagttagt(GA)10</td><td>72</td><td>--</td><td>--</td><td>--</td><td>--</td><td>--</td><td>--</td><td>TGGCTATTTGTTTTGTGAGAGTG</td><td>59</td><td>AATCAGCCCAAAAGAAGCAA</td><td>60</td><td>246</td><td>--</td><td>--</td><td>--</td><td>--</td><td>--</td><td>--</td></tr>
<tr><td>TR46162|c0_g2_i1</td><td>No</td><td>(AG)7aaagaggtgaagtctcaactcaagtgtgtgaagttagt(GA)10</td><td>72</td><td>--</td><td>--</td><td>--</td><td>--</td><td>--</td><td>--</td><td>TGGCTATTTGTTTTGTGAGAGTG</td><td>59</td><td>AATCAGCCCAAAAGAAGCAA</td><td>60</td><td>246</td><td>--</td><td>--</td><td>--</td><td>--</td><td>--</td><td>--</td></tr>
<tr><td>TR46185|c0_g1_i1</td><td>No</td><td>(CAA)7</td><td>21</td><td>--</td><td>--</td><td>--</td><td>--</td><td>--</td><td>--</td><td>GAAAACCCACCCACAACACT</td><td>60</td><td>CATCAGGTGCTTCGATGAGA</td><td>60</td><td>226</td><td>--</td><td>--</td><td>--</td><td>--</td><td>--</td><td>--</td></tr>
<tr><td>TR46185|c0_g2_i1</td><td>No</td><td>(CAA)7</td><td>21</td><td>--</td><td>--</td><td>--</td><td>--</td><td>--</td><td>--</td><td>GAAAACCCACCCACAACACT</td><td>60</td><td>CATCAGGTGCTTCGATGAGA</td><td>60</td><td>226</td><td>--</td><td>--</td><td>--</td><td>--</td><td>--</td><td>--</td></tr>
<tr><td>TR46219|c0_g1_i1</td><td>No</td><td>(CT)9</td><td>18</td><td>--</td><td>--</td><td>--</td><td>--</td><td>--</td><td>--</td><td>TTGCCTTAAAGCCCATTATCA</td><td>60</td><td>AGGTGCAGGTTGCTGAGTCT</td><td>60</td><td>261</td><td>--</td><td>--</td><td>--</td><td>--</td><td>--</td><td>--</td></tr>
<tr><td>TR46219|c0_g1_i2</td><td>No</td><td>(CT)9</td><td>18</td><td>--</td><td>--</td><td>--</td><td>--</td><td>--</td><td>--</td><td>TTGCCTTAAAGCCCATTATCA</td><td>60</td><td>AGGTGCAGGTTGCTGAGTCT</td><td>60</td><td>261</td><td>--</td><td>--</td><td>--</td><td>--</td><td>--</td><td>--</td></tr>
</table>

Transcript IDs	Differentially expressed gene (DEG) IDs	SSR repeat-motifs	SSR repeat-length (bp)	*In silico* polymorphic SSRs						Forward primers (5'-3')	Annealing tempearture(0C)	Reverse primers (5'-3')	Annealing tempearture(0C)	Amplified product size (bp)	Structural annotation		Functional Annotation			
				C. microphyllum		ICC4958														
				Transcript	SSR repeat-motifs	Chromosomes	SSR repeat-motifs	Start physical positions (bp)	End physical positions (bp)						Gene IDs	Sequence component of genes	NCBI nr IDs	TFs	KOG	KEGG
TR46264|c0_g1_i1	XLOC_018057	(TTGTA)5	25	--	--	--	--	--	--	CGGAGTTCGACCGAAAGATA	60	GCGGTTGAAAAGGAAATTGA	60	241	--	--	--	--	--	--
TR46290|c0_g1_i1	XLOC_018082	(TCG)5	15	--	--	--	--	--	--	CCGATCCACTTCCTTTTCAA	60	ACCTGGACGGACTTTGTGAG	60	132	--	--	--	--	--	--
TR46298|c0_g1_i1	XLOC_018089	(GAA)5	15	--	--	--	--	--	--	CTGATCCCGATGAAGAGAGC	60	CCTACCTCACCATGCTCCAT	60	106	--	--	--	--	--	--
TR46308|c0_g1_i1	No	(TC)8	16	--	--	--	--	--	--	AGAAGGTGGAGGGAAATTCG	60	GCATAAGACTGAGAAGGGCG	60	208	--	--	--	--	--	--
TR46308|c0_g1_i2	No	(TC)8	16	--	--	--	--	--	--	AGAAGGTGGAGGGAAATTCG	60	GCATAAGACTGAGAAGGGCG	60	208	--	--	--	--	--	--
TR46335|c0_g1_i1	No	(TC)9	18	--	--	--	--	--	--	CCTACCCACCCATCTACCCT	60	AAGCGTCCATGACACAAACA	60	237	--	--	--	--	--	--
TR46335|c0_g2_i1	No	(TC)9	18	--	--	--	--	--	--	CCTACCCACCCATCTACCCT	60	AAGCGTCCATGACACAAACA	60	237	--	--	--	--	--	--
TR46384|c0_g1_i1	No	(ATT)5	15	--	--	--	--	--	--	CATCGCATCCATGTTCAAAG	60	GGAAAGGCACAAATTGATGG	60	219	--	--	--	--	--	--
TR46384|c0_g2_i1	No	(ATT)5	15	--	--	--	--	--	--	CATCGCATCCATGTTCAAAG	60	GGAAAGGCACAAATTGATGG	60	219	--	--	--	--	--	--
TR46389|c0_g1_i1	XLOC_018158	(TTG)5	15	--	--	--	--	--	--	ACATGTGCATTTGCAACACA	60	CATCGAACGGTGTGAATTTG	60	210	--	--	--	--	--	--
TR46391|c1_g1_i1	XLOC_018161	(CTT)7	21	--	--	--	--	--	--	TTGCGCTTAGTCACTTGTGC	60	ACCCGGCATCTGACACTTAC	60	259	--	--	NP_001156301.1	--	[K]	--
TR46474|c0_g1_i1	No	(CTG)5	15	--	--	--	--	--	--	TTTGCTAAGAAATCCCACCG	60	TCCATCAGTGGCAAGAACAA	60	191	--	--	--	--	--	--
TR46533|c0_g1_i1	No	(AC)6	12	--	--	--	--	--	--	TCGCTGTTGAAAACCAAAAA	59	AAGCAAAAAGCGAGCGTTAG	60	117	--	--	--	--	--	--
TR4655|c0_g1_i1	No	(AGC)5	15	--	--	--	--	--	--	ACACAGTCAAGGTCGTGCAG	60	CTTGGACTTCAGTCCCGTTT	59	110	--	--	--	--	--	--

Transcript IDs	Differentially expressed gene (DEG) IDs	SSR repeat-motifs	SSR repeat-length (bp)	*In silico* polymorphic SSRs						Forward primers (5'-3')	Annealing tempearture(0C)	Reverse primers (5'-3')	Annealing tempearture(0C)	Amplified product size (bp)	Structural annotation		Functional Annotation			
				C. microphyllum		ICC4958									Gene IDs	Sequence component of genes	NCBI nr IDs	TFs	KOG	KEGG
				Transcript	SSR repeat-motifs	Chromosomes	SSR repeat-motifs	Start physical positions (bp)	End physical positions (bp)											
TR46559|c0_g1_i1	XLOC_018192	(A)12caaaacaagaaattccattttctgttccaaactctatctctctg(TC)6	68	--	--	--	--	--	--	GAAATCCCCAAACTCTGCAA	60	TGTTTGCTTTCATGGGTTTTC	60	180	--	--	--	--	--	--
TR46658|c0_g1_i1	No	(AAG)5	15	--	--	--	--	--	--	TTGAGCAGAAAGGGGAAAGA	60	CTTTGCATCATCACGTCCAC	60	113	--	--	--	--	--	--
TR4667|c0_g1_i1	No	(TCC)7	21	--	--	--	--	--	--	GGTGGAAGCTGATTGTGTTG	59	GCGTTTGCAGTGCAGAGATA	60	107	--	--	--	--	--	--
TR46709|c0_g1_i1	XLOC_018231	(TC)11	22	--	--	--	--	--	--	TCCACTCTCTCCCTATCCTTCTT	60	CTGGCGATGTGCTTAGATGA	60	269	--	--	--	--	--	--
TR46767|c0_g1_i1	No	(AT)6	12	--	--	--	--	--	--	TAGGAGGGATGGTAGGGAGG	60	GCGTTGCATGGGTGTTAAT	59	274	--	--	--	--	--	--
TR46843|c0_g1_i1	No	(AC)9	18	--	--	--	--	--	--	GCATGTACCAAAAAGGAGGAG	59	GCAAAAAGCAAACCAACCTC	60	129	--	--	--	--	--	--
TR46917|c0_g1_i1	XLOC_018274	(CCA)5	15	--	--	--	--	--	--	TCACCAAATCCTCTTCCACC	60	CAGCAGCAATAATCATGAGACC	60	130	--	--	--	--	--	--
TR46935|c0_g1_i1	No	(AAC)5	15	--	--	--	--	--	--	GAATTTGAATGGCGAGGAAA	60	GGGTTCTTGAAGGTTGGTGA	60	122	--	--	--	--	--	--
TR46935|c0_g2_i1	No	(AAC)5	15	--	--	--	--	--	--	GAATTTGAATGGCGAGGAAA	60	GGGTTCTTGAAGGTTGGTGA	60	122	--	--	--	--	--	--
TR46958|c0_g1_i1	XLOC_018285	(CTG)7	21	--	--	--	--	--	--	GATTAGTTTTGAGGCAGCCG	60	GCAGGTGCGTCATTAATCCT	60	270	--	--	--	--	--	--
TR46958|c0_g1_i1	XLOC_018285	(TCC)7	21	--	--	--	--	--	--	TAATGACGCACCTGCAAGAC	60	CTTGAGTGGGTAGGGGTTGA	60	216	--	--	--	--	--	--
TR46990|c0_g1_i1	XLOC_018294	(AC)6	12	--	--	--	--	--	--	CCCTTTACTTTCCCACCCTC	60	TTTTAACGTACAGAACATGATTAACAG	58	132	--	--	--	--	--	--
TR46990|c0_g1_i1	XLOC_018294	(TC)8	16	--	--	--	--	--	--	TTCCGCTCTCTTCTTGCTTC	60	TGTTGAAGACGCCAAAACAA	60	207	--	--	--	--	--	--
TR47030|c0_g1_i1	XLOC_018306	(CAA)6	18	--	--	--	--	--	--	ATATTGCTTCGCCTTCATCG	60	CGTTCTTCTCTTACACGTGCC	60	142	--	--	--	--	--	--

Transcript IDs	Differentially expressed gene (DEG) IDs	SSR repeat-motifs	SSR repeat-length (bp)	*In silico* polymorphic SSRs						Forward primers (5'-3')	Annealing tempearture(0C)	Reverse primers (5'-3')	Annealing tempearture(0C)	Amplified product size (bp)	Structural annotation		Functional Annotation			
				C. microphyllum		ICC4958														
				Transcript	SSR repeat-motifs	Chromosomes	SSR repeat-motifs	Start physical positions (bp)	End physical positions (bp)						Gene IDs	Sequence component of genes	NCBI nr IDs	TFs	KOG	KEGG
TR47054|c0_g1_i1	XLOC_018310	(TC)7	14	TR47054|c0_g1_i1	(TC)7	Ca_LG_1	(TC)9	29652198	29652215	CGATCATGATGAACCACAGC	60	GATCCTGAAATGCCGAGGTA	60	279		URR	--	--	--	--
TR47076|c0_g1_i1	No	(CTG)6	18	--	--	--	--	--	--	TAACTGCACCTGCGTTCTTG	60	TGCAGAAACTGCATTGAAGG	60	221	--	--	--	--	--	--
TR47076|c0_g2_i1	No	(CTG)6	18	--	--	--	--	--	--	TAACTGCACCTGCGTTCTTG	60	TGCAGAAACTGCATTGAAGG	60	221	--	--	--	--	--	--
TR47121|c0_g1_i1	No	(AG)7	14	--	--	--	--	--	--	GACGACGACGAGTGAGGAG	60	AGAGAAGTCGCATCCCTCAA	60	227	--	--	--	--	--	--
TR47123|c0_g1_i1	No	(AC)6	12	--	--	--	--	--	--	AAATCCCAGAGAATCATTTGGT	59	TCTTCGGTAAAACGTGAAAAA	58	197	--	--	--	--	--	--
TR47190|c0_g1_i1	XLOC_018354	(GA)8	16	--	--	--	--	--	--	TGAGCGACAAATTCAACAGG	60	ATGGACCATGACCACCATCT	60	137	--	--	AAL40718.1	--	[Z]	--
TR47211|c0_g1_i1	XLOC_018370	(TCT)6	18	TR47211|c0_g1_i1	(TCT)6	scaffold04340	(TCT)5	2762	2776	GCTTATGCGATGAACTCTTGC	60	AGAAGGGTTGTTGTTGGGTG	60	271	Ca_22546	CDS	--	--	--	--
TR47255|c0_g1_i1	XLOC_018398	(ACA)6	18	--	--	--	--	--	--	AGGGTAGCAACAAGGCAATG	60	GTTCGCAAATTGTTTCGGAT	60	106	--	--	--	--	--	--
TR47302|c0_g1_i1	No	(GCA)5	15	--	--	--	--	--	--	TAGCTCCAAGGCTTTTCCAA	60	GAAAACTTGGCCCAGTACGA	60	161	--	--	--	--	--	--
TR47365|c1_g4_i1	No	(AG)6	12	--	--	--	--	--	--	CAAAATTGAAATAAAATGGGGAA	59	TCTTTCTTGCATCTGCTCCC	60	240	--	--	--	--	--	--
TR47365|c1_g4_i4	No	(AG)6	12	--	--	--	--	--	--	CAAAATTGAAATAAAATGGGGAA	59	TCTTTCTTGCATCTGCTCCC	60	240	--	--	--	--	--	--
TR47368|c0_g3_i9	XLOC_018447	(CAA)5	15	--	--	--	--	--	--	CAAGTTTCAACACACTTGACTACTCT	58	TGAGAAAATAAACCGGGTCG	60	273	--	--	XM_004490235.1	bHLH	[KT]	--
TR47371|c0_g2_i1	No	(TCTT)5tcataatttcacatttaataataataaacaaataaataaaaggttacacttattttcacactattccgttccgtaacagag(A)13	114	--	--	--	--	--	--	CTCTTCCTTCTTCACTAACACTAGG	57	CGAGTGCCGCCATAGTAAGT	60	199	--	--	--	--	--	--
TR47371|c0_g2_i1	No	(GAT)5	15	--	--	--	--	--	--	CTGAACGGACCGGTTACTGT	60	CAGCATTTCCATTCGTGAGA	60	223	--	--	--	--	--	--

Transcript IDs	Differentially expressed gene (DEG) IDs	SSR repeat-motifs	SSR repeat-length (bp)	*In silico* polymorphic SSRs						Forward primers (5'-3')	Annealing tempearture(0C)	Reverse primers (5'-3')	Annealing tempearture(0C)	Amplified product size (bp)	Structural annotation		Functional Annotation			
				C. microphyllum		ICC4958									Gene IDs	Sequence component of genes	NCBI nr IDs	TFs	KOG	KEGG
				Transcript	SSR repeat-motifs	Chromosomes	SSR repeat-motifs	Start physical positions (bp)	End physical positions (bp)											
TR47371|c0_g2_i2	No	(TCTT)5tcataatttcacatttaataataataaacaaataaataaaaggttacacttattttcacactattccgttccgtaacagag(A)13	114	--	--	--	--	--	--	CTCTTCCTTCTTCACTAACACTAGG	57	CGAGTGCCGCCATAGTAAGT	60	199	--	--	--	--	--	--
TR47371|c0_g2_i2	No	(GAT)5	15	--	--	--	--	--	--	CTGAACGGACCGGTTACTGT	60	CAGCATTTCCATTCGTGAGA	60	223	--	--	--	--	--	--
TR47371|c0_g2_i3	No	(TCTT)5tcataatttcacatttaataataataaacaaataaataaaaggttacacttattttcacactattccgttccgtaacagag(A)13	114	--	--	--	--	--	--	CTCTTCCTTCTTCACTAACACTAGG	57	CGAGTGCCGCCATAGTAAGT	60	199	--	--	--	--	--	--
TR47371|c0_g2_i3	No	(GAT)5	15	--	--	--	--	--	--	CTGAACGGACCGGTTACTGT	60	CAGCATTTCCATTCGTGAGA	60	241	--	--	--	--	--	--
TR47373|c0_g1_i1	XLOC_018458	(AT)6	12	--	--	--	--	--	--	GTTGCTTCCATTCGGAAGAG	60	GCTTCTTTGTACTAGTTGATGATTCG	60	278	--	--	--	--	--	--
TR47373|c2_g1_i1	No	(GGT)5	15	--	--	--	--	--	--	GATGAAGGAAACCAAAGCGA	60	TGTCTCACCTCCGGCTTTAC	60	200	--	--	--	--	--	--
TR47373|c2_g1_i2	No	(GGT)5	15	--	--	--	--	--	--	GATGAAGGAAACCAAAGCGA	60	TGTCTCACCTCCGGCTTTAC	60	200	--	--	--	--	--	--
TR47379|c2_g1_i1	No	(TGA)5	15	--	--	--	--	--	--	CTATGCCCAGGAGAGAACCA	60	CCTTCTTCGACGACCACTTC	60	185	--	--	--	--	--	--
TR47379|c2_g2_i1	No	(TC)9	18	--	--	--	--	--	--	CCAAATTTCAGGTAGTGGTACTGTC	60	AAAGGGTCCAAATCCAAACC	60	182	--	--	--	--	--	--
TR47379|c2_g2_i2	No	(TC)9	18	--	--	--	--	--	--	CCAAATTTCAGGTAGTGGTACTGTC	60	AAAGGGTCCAAATCCAAACC	60	182	--	--	--	--	--	--
TR47379|c3_g1_i1	XLOC_018475	(CTT)5	15	--	--	--	--	--	--	CCATCATGTTACAACGCCTG	60	ACCTTTACGGAGCTGGGAAT	60	259	--	--	--	--	--	--
TR47379|c3_g1_i2	XLOC_018476	(CTT)5	15	--	--	--	--	--	--	TTCACAAACCACCAAACCCT	60	ACCTTTACGGAGCTGGGAAT	60	147	--	--	--	--	--	--
TR47385|c0_g2_i1	XLOC_018481	(AAGA)5a(AAG)12	57	--	--	--	--	--	--	TTCGGATTTGAGCAAGAAGAA	60	CACCACCTCCGCTCACAC	61	129	--	--	--	--	--	--
TR47385|c1_g1_i1	XLOC_018482	(AGA)5	15	--	--	--	--	--	--	AACGACAGCATTGAGTGAAGAA	60	AGTGATGAACATCAGCGTGG	60	260	--	--	XP_004500739.1	--	--	--

Transcript IDs	Differentially expressed gene (DEG) IDs	SSR repeat-motifs	SSR repeat-length (bp)	*In silico* polymorphic SSRs						Forward primers (5'-3')	Annealing tempearture(0C)	Reverse primers (5'-3')	Annealing tempearture(0C)	Amplified product size (bp)	Structural annotation		Functional Annotation			
				C. microphyllum		ICC4958														
				Transcript	SSR repeat-motifs	Chromosomes	SSR repeat-motifs	Start physical positions (bp)	End physical positions (bp)						Gene IDs	Sequence component of genes	NCBI nr IDs	TFs	KOG	KEGG
TR47390|c0_g5_i1	No	(AC)8	16	--	--	--	--	--	--	ATGGCAGCCGCATATAAAAC	60	TTGATGTTGTTGAGGCGAAG	60	270	--	--	--	--	--	--
TR47390|c0_g5_i2	No	(AC)8	16	--	--	--	--	--	--	ATGGCAGCCGCATATAAAAC	60	TTGATGTTGTTGAGGCGAAG	60	270	--	--	--	--	--	--
TR47390|c0_g5_i4	No	(AC)8	16	--	--	--	--	--	--	ATGGCAGCCGCATATAAAAC	60	TTGATGTTGTTGAGGCGAAG	60	270	--	--	--	--	--	--
TR47390|c0_g5_i5	No	(AC)8	16	--	--	--	--	--	--	ATGGCAGCCGCATATAAAAC	60	TTGATGTTGTTGAGGCGAAG	60	270	--	--	--	--	--	--
TR47390|c0_g5_i6	No	(AC)8	16	--	--	--	--	--	--	ATGGCAGCCGCATATAAAAC	60	TTGATGTTGTTGAGGCGAAG	60	270	--	--	--	--	--	--
TR47391|c0_g1_i1	No	(AAC)6(A)10	28	--	--	--	--	--	--	CTTGCAATACGGAGAGACCA	59	AGTCAGCTCCTTGTGGGAGA	60	195	--	--	--	--	--	--
TR47391|c0_g1_i2	XLOC_018497	(AAC)6(A)10	28	--	--	--	--	--	--	CTTGCAATACGGAGAGACCA	59	AGTCAGCTCCTTGTGGGAGA	60	195	--	--	CCO33740.1	--	[OPR]	--
TR47398|c0_g2_i1	XLOC_018509	(AT)7	14	--	--	--	--	--	--	CCTGGTGCTTGAACCAAACT	60	CAACCATTAGCATTACGAACACC	61	116	--	--	--	--	--	--
TR47403|c0_g1_i2	No	(TAG)5	15	--	--	--	--	--	--	CTCCCAAGTTCCCACTCGTA	60	GATTCCACCGTTAGGACGAA	60	235	--	--	--	--	--	--
TR47405|c7_g1_i1	XLOC_018522	(GA)6	12	--	--	--	--	--	--	TTGTGGTTGAGGATTGTGGA	60	CGCTCCTTTCCTTAGCTTCG	61	139	--	--	--	--	--	--
TR47410|c0_g2_i1	No	(AAT)5	15	--	--	--	--	--	--	GGATGATCCGGCTGTTAGTATC	60	GCATCAAATAGATCACACGCA	60	234	--	--	--	--	--	--
TR47420|c2_g8_i8	No	(AT)6	12	--	--	--	--	--	--	AGCACACTGCACACCACTTC	60	GGAAACTCCAATAGCCACCA	60	147	--	--	--	--	--	--
TR47423|c2_g2_i4	No	(ACA)5	15	--	--	--	--	--	--	CAGTGAACGTTCTCGCTCAG	60	TGGAAGAATTTGGTTTGGGT	59	278	--	--	--	--	--	--
TR47423|c2_g2_i7	No	(ACA)5	15	--	--	--	--	--	--	CAGTGAACGTTCTCGCTCAG	60	TGTATTTGCGAGATTTTGGC	59	199	--	--	--	--	--	--

Transcript IDs	Differentially expressed gene (DEG) IDs	SSR repeat-motifs	SSR repeat-length (bp)	*In silico* polymorphic SSRs						Forward primers (5'-3')	Annealing tempearture(0C)	Reverse primers (5'-3')	Annealing tempearture(0C)	Amplified product size (bp)	Structural annotation		Functional Annotation			
				C. microphyllum		ICC4958									Gene IDs	Sequence component of genes	NCBI nr IDs	TFs	KOG	KEGG
				Transcript	SSR repeat-motifs	Chromosomes	SSR repeat-motifs	Start physical positions (bp)	End physical positions (bp)											
TR47435|c0_g1_i1	No	(CT)6	12	--	--	--	--	--	--	TCACTCCTTTTCGCGTTTTC	60	GAAGGAACATAGCGGTTGGA	60	107	--	--	--	--	--	--
TR47435|c0_g1_i2	No	(CCT)5	15	--	--	--	--	--	--	CATTTGGGTTATGGTCAGGG	60	GCAAGAGCCTCAGTCACCTC	60	236	--	--	--	--	--	--
TR47435|c0_g1_i2	No	(CT)6	12	--	--	--	--	--	--	TCACTCCTTTTCGCGTTTTC	60	GCAGATGAACTTGAACGCAA	60	202	--	--	--	--	--	--
TR47440|c0_g1_i1	No	(CT)6	12	--	--	--	--	--	--	CTCCGGTTCCAAACATAGGA	60	TGGTGTTTGTATTCGCAGGA	60	106	--	--	--	--	--	--
TR47440|c0_g1_i2	No	(CT)6	12	--	--	--	--	--	--	CTCCGGTTCCAAACATAGGA	60	TGGTGTTTGTATTCGCAGGA	60	106	--	--	--	--	--	--
TR47440|c0_g1_i3	No	(CT)9	18	--	--	--	--	--	--	GCCCAGCTTCACAGAAACC	61	TGTGGCAATGACAACTCCAT	60	208	--	--	--	--	--	--
TR47440|c0_g1_i4	No	(CT)6	12	--	--	--	--	--	--	CTCCGGTTCCAAACATAGGA	60	TGGTGTTTGTATTCGCAGGA	60	106	--	--	--	--	--	--
TR47440|c0_g1_i5	No	(CT)9	18	--	--	--	--	--	--	GCCCAGCTTCACAGAAACC	61	TGTGGCAATGACAACTCCAT	60	208	--	--	--	--	--	--
TR47440|c0_g1_i6	No	(CT)6	12	--	--	--	--	--	--	CTCCGGTTCCAAACATAGGA	60	TGGTGTTTGTATTCGCAGGA	60	106	--	--	--	--	--	--
TR47443|c1_g2_i1	XLOC_018626	(AAC)5aagaagcgagtttgacttggaatcgtcaaattagtttcttagtttttgattttttgcgaattggagtggatcagcgaggtttctttta(AG)7	117	--	--	--	--	--	--	CGGTCCAATTCAGTTTCACA	60	GAGAGGTTCACCGAAACGAC	60	269	--	--	--	--	--	--
TR47443|c1_g3_i1	XLOC_018627	(AAC)5aagaagcgagtttgacttggaatcgtcaaattagtttcttagtttttgattttttgcgaattggagtggatcagcgaggtttctttta(AG)7	117	--	--	--	--	--	--	CGGTCCAATTCAGTTTCACA	60	GAGAGGTTCACCGAAACGAC	60	269	--	--	--	--	--	--
TR47445|c2_g1_i1	No	(CA)9	18	--	--	--	--	--	--	TCATTCACACAAATCTCACAACC	60	CCGCCATCTATGTGTTGTTG	60	246	--	--	--	--	--	--
TR47445|c2_g1_i10	No	(CA)9	18	--	--	--	--	--	--	TCATTCACACAAATCTCACAACC	60	CCGCCATCTATGTGTTGTTG	60	246	--	--	--	--	--	--
TR47445|c2_g1_i11	No	(CA)9	18	--	--	--	--	--	--	TCATTCACACAAATCTCACAACC	60	CCGCCATCTATGTGTTGTTG	60	246	--	--	--	--	--	--

Transcript IDs	Differentially expressed gene (DEG) IDs	SSR repeat-motifs	SSR repeat-length (bp)	*In silico* polymorphic SSRs						Forward primers (5'-3')	Annealing tempearture(0C)	Reverse primers (5'-3')	Annealing tempearture(0C)	Amplified product size (bp)	Structural annotation		Functional Annotation			
				C. microphyllum		ICC4958									Gene IDs	Sequence component of genes	NCBI nr IDs	TFs	KOG	KEGG
				Transcript	SSR repeat-motifs	Chromosomes	SSR repeat-motifs	Start physical positions (bp)	End physical positions (bp)											
TR47445|c2_g1_i12	No	(CA)9	18	--	--	--	--	--	--	TCATTCACACAAATCTCACAACC	60	CCGCCATCTATGTGTTGTTG	60	246	--	--	--	--	--	--
TR47445|c2_g1_i2	No	(CA)9	18	--	--	--	--	--	--	TCATTCACACAAATCTCACAACC	60	CCGCCATCTATGTGTTGTTG	60	246	--	--	--	--	--	--
TR47445|c2_g1_i3	No	(CA)9	18	--	--	--	--	--	--	TCATTCACACAAATCTCACAACC	60	CCGCCATCTATGTGTTGTTG	60	246	--	--	--	--	--	--
TR47445|c2_g1_i4	No	(CA)9	18	--	--	--	--	--	--	TCATTCACACAAATCTCACAACC	60	CCGCCATCTATGTGTTGTTG	60	246	--	--	--	--	--	--
TR47445|c2_g1_i5	No	(CA)9	18	--	--	--	--	--	--	TCATTCACACAAATCTCACAACC	60	CCGCCATCTATGTGTTGTTG	60	246	--	--	--	--	--	--
TR47445|c2_g1_i6	No	(CA)9	18	--	--	--	--	--	--	TCATTCACACAAATCTCACAACC	60	CCGCCATCTATGTGTTGTTG	60	246	--	--	--	--	--	--
TR47445|c2_g1_i7	No	(CA)9	18	--	--	--	--	--	--	TCATTCACACAAATCTCACAACC	60	CCGCCATCTATGTGTTGTTG	60	246	--	--	--	--	--	--
TR47445|c2_g1_i8	No	(CA)9	18	--	--	--	--	--	--	TCATTCACACAAATCTCACAACC	60	CCGCCATCTATGTGTTGTTG	60	246	--	--	--	--	--	--
TR47445|c2_g1_i9	No	(CA)9	18	--	--	--	--	--	--	TCATTCACACAAATCTCACAACC	60	CCGCCATCTATGTGTTGTTG	60	246	--	--	--	--	--	--
TR4745|c0_g1_i1	XLOC_018648	(GAA)11	33	--	--	--	--	--	--	AGGGTACAGCGAGAGCGATA	60	GTCATAGCGAGGGAGGTTGA	60	110	--	--	--	--	--	--
TR47451|c0_g2_i10	No	(TCA)5	15	--	--	--	--	--	--	ATCGAGAACCGATGAAATGG	60	CAAAATCTGATGCAGTTCCTGA	60	228	--	--	--	--	--	--
TR47451|c0_g2_i2	No	(TCA)5	15	--	--	--	--	--	--	ATCGAGAACCGATGAAATGG	60	CAAAATCTGATGCAGTTCCTGA	60	228	--	--	--	--	--	--
TR47451|c0_g2_i3	No	(TCA)5	15	--	--	--	--	--	--	ATCGAGAACCGATGAAATGG	60	CAAAATCTGATGCAGTTCCTGA	60	228	--	--	--	--	--	--
TR47451|c0_g2_i4	No	(TCA)5	15	--	--	--	--	--	--	ATCGAGAACCGATGAAATGG	60	CAAAATCTGATGCAGTTCCTGA	60	228	--	--	--	--	--	--

Transcript IDs	Differentially expressed gene (DEG) IDs	SSR repeat-motifs	SSR repeat-length (bp)	*In silico* polymorphic SSRs						Forward primers (5'-3')	Annealing tempearture(0C)	Reverse primers (5'-3')	Annealing tempearture(0C)	Amplified product size (bp)	Structural annotation		Functional Annotation			
				C. microphyllum		ICC4958									Gene IDs	Sequence component of genes	NCBI nr IDs	TFs	KOG	KEGG
				Transcript	SSR repeat-motifs	Chromosomes	SSR repeat-motifs	Start physical positions (bp)	End physical positions (bp)											
TR47451|c0_g2_i5	No	(TCA)5	15	--	--	--	--	--	--	ATCGAGAACCGATGAAATGG	60	CAAAATCTGATGCAGTTCCTGA	60	228	--	--	--	--	--	--
TR47451|c0_g2_i7	No	(TCA)5	15	--	--	--	--	--	--	ATCGAGAACCGATGAAATGG	60	CAAAATCTGATGCAGTTCCTGA	60	228	--	--	--	--	--	--
TR47451|c0_g2_i8	No	(TCA)5	15	--	--	--	--	--	--	ATCGAGAACCGATGAAATGG	60	CAAAATCTGATGCAGTTCCTGA	60	228	--	--	--	--	--	--
TR47451|c0_g2_i9	No	(TCA)5	15	--	--	--	--	--	--	ATCGAGAACCGATGAAATGG	60	CAAAATCTGATGCAGTTCCTGA	60	228	--	--	--	--	--	--
TR47453|c2_g1_i1	XLOC_018643	(AC)6	12	--	--	--	--	--	--	CACTTTCTCACTTCATTCCATAAAA	59	TGTGAGAGGAAACAAGGAACG	60	257	--	--	--	--	--	--
TR47455|c0_g1_i1	No	(TTC)5	15	--	--	--	--	--	--	TTCCTACACCACCATCACCA	60	GGCTGCAGTTAGTTGCATGA	60	130	--	--	--	--	--	--
TR47455|c0_g1_i5	No	(TTC)5	15	--	--	--	--	--	--	TTCCTACACCACCATCACCA	60	GGCTGCAGTTAGTTGCATGA	60	130	--	--	--	--	--	--
TR47563|c0_g1_i1	No	(AG)6	12	--	--	--	--	--	--	GGGAAAAAGTGGAGGGGTAA	60	CTGTGGAGACCTCGCAGTTT	60	149	--	--	--	--	--	--
TR47581|c0_g1_i1	XLOC_018670	(TATT)5	20	--	--	--	--	--	--	CATTGAAACGTCAACATGGG	60	TAACGCTGGTAATTGGCACA	60	166	--	--	--	--	--	--
TR47636|c0_g1_i1	No	(CT)15	30	--	--	--	--	--	--	GGACTTCGTGGTCGAGAGAC	60	CGCCATTGTTGCTTCTGTTA	60	197	--	--	--	--	--	--
TR47636|c0_g1_i2	No	(CT)23	46	--	--	--	--	--	--	GGACTTCGTGGTCGAGAGAC	60	CGCCATTGTTGCTTCTGTTA	60	213	--	--	--	--	--	--
TR47641|c0_g1_i1	No	(CT)7	14	--	--	--	--	--	--	AAGAGTGTTGAAATTCCTCCC	57	AGCTTTGGAAACGAGCAAAC	59	135	--	--	--	--	--	--
TR47641|c0_g2_i1	No	(CT)7	14	--	--	--	--	--	--	AAGAGTGTTGAAATTCCTCCC	57	AGCTTTGGAAACGAGCAAAC	59	135	--	--	--	--	--	--
TR47642|c0_g2_i1	No	(AAC)5	15	--	--	--	--	--	--	GCAATTTCCTCAATCAACAAAA	59	GCTGGTGACATGGTGAAGAA	60	186	--	--	--	--	--	--

Transcript IDs	Differentially expressed gene (DEG) IDs	SSR repeat-motifs	SSR repeat-length (bp)	*In silico* polymorphic SSRs						Forward primers (5'-3')	Annealing tempearture(0C)	Reverse primers (5'-3')	Annealing tempearture(0C)	Amplified product size (bp)	Structural annotation		Functional Annotation			
				C. microphyllum		ICC4958									Gene IDs	Sequence component of genes	NCBI nr IDs	TFs	KOG	KEGG
				Transcript	SSR repeat-motifs	Chromosomes	SSR repeat-motifs	Start physical positions (bp)	End physical positions (bp)											
TR47667|c0_g1_i1	XLOC_018702	(AAC)6	18	--	--	--	--	--	--	TGCAATAGCAATCATGATCAAA	59	TTTTCGCCGCTTAGTTGTCT	60	120	--	--	--	--	--	--
TR47720|c0_g1_i1	No	(GAA)5	15	--	--	--	--	--	--	AAATTGTCCCTTGGGGGTAG	60	TCGTCAAATTTGCCAGTACC	59	183	--	--	--	--	--	--
TR4776|c0_g1_i1	XLOC_018758	(CTT)6	18	--	--	--	--	--	--	CAACGTGAAGGTAAATCAAAAGC	60	TCCACCACAACGTCGTAGAA	60	120	--	--	--	--	--	--
TR4776|c0_g2_i1	No	(CTT)6	18	--	--	--	--	--	--	CAACGTGAAGGTAAATCAAAAGC	60	TCCACCACAACGTCGTAGAA	60	120	--	--	--	--	--	--
TR478|c0_g1_i1	XLOC_018796	(TCC)6	18	--	--	--	--	--	--	ATGAAACCAGCTGAGATCCG	60	GTACCTCATCCAGCCACGAT	60	181	--	--	--	--	--	--
TR47840|c0_g1_i1	No	(T)11actaatttattctttctcgaagaaaactgtggaaggat gatacctgaaaaacatcgcatagctttcc(T)10	88	--	--	--	--	--	--	TCAGAGCTCATTCATCAGTGG	59	TCTCAAATACCATCCGCTCC	60	273	--	--	--	--	--	--
TR47845|c1_g1_i1	No	(ACG)5	15	--	--	--	--	--	--	CTTAGGGGCGTTCGTGTAGA	60	CGAGCGCACTGCAATAAATA	60	106	--	--	--	--	--	--
TR47890|c0_g1_i1	No	(TGA)5	15	--	--	--	--	--	--	TTGGGAAATCCGTTTACGAG	60	CAACGCATTCCTCTTCTTCTG	60	235	--	--	--	--	--	--
TR47964|c0_g1_i1	XLOC_018807	(ATC)5	15	--	--	--	--	--	--	ACCAAACCACTCTCTCCCCT	60	GAAGACCGGTTTGTAGTGAGAAA	60	120	--	--	--	--	--	--
TR48003|c0_g1_i1	XLOC_018826	(ATC)5	15	--	--	--	--	--	--	CAAAGGCTTCCAAAAAGCAC	60	CCAACATCTCCAATTCCTTCA	60	262	--	--	--	--	--	--
TR48060|c0_g1_i1	XLOC_018852	(GAG)5	15	--	--	--	--	--	--	ACTCTAAATCGCTTTGCCGA	60	ACATTTTCCTCCTTCGCCTT	60	254	--	--	XM_004507708.1	--	--	K02916
TR48062|c1_g1_i1	XLOC_018855	(ATC)6	18	--	--	--	--	--	--	TACGACTCACACGAGTTCGC	60	TAGCTCTGGACGATGTGTCG	60	265	--	--	--	--	--	--
TR48100|c0_g1_i1	XLOC_018889	(AAT)7	21	--	--	--	--	--	--	ACTCTCGAAATCCCCTTCGT	60	TTTGAAGCTCGTTCCAAACC	60	220	--	--	--	--	--	--
TR48219|c0_g1_i1	No	(GA)10	20	--	--	--	--	--	--	CCTTCCTCACACCATCACAA	60	GTGGTGTGAAGCGTCATGTT	60	212	--	--	--	--	--	--

Transcript IDs	Differentially expressed gene (DEG) IDs	SSR repeat-motifs	SSR repeat-length (bp)	*In silico* polymorphic SSRs						Forward primers (5'-3')	Annealing tempearture(0C)	Reverse primers (5'-3')	Annealing tempearture(0C)	Amplified product size (bp)	Structural annotation		Functional Annotation			
				C. microphyllum		ICC4958									Gene IDs	Sequence component of genes	NCBI nr IDs	TFs	KOG	KEGG
				Transcript	SSR repeat-motifs	Chromosomes	SSR repeat-motifs	Start physical positions (bp)	End physical positions (bp)											
TR48219|c0_g2_i1	No	(GA)10	20	--	--	--	--	--	--	CCTTCCTCACACCATCACAA	60	GTGGTGTGAAGCGTCATGTT	60	212	--	--	--	--	--	--
TR48223|c0_g1_i1	No	(T)11atagtttcgtgggttgttccagaatatataagttgattgggg(T)19	72	--	--	--	--	--	--	CGGTGTTTTGGGATTATTGA	58	CCCTAATTCCCCACTCACAA	60	213	--	--	--	--	--	--
TR48223|c0_g2_i1	No	(T)11atagtttcgtgggttgttccagaatatataagttgattgggg(T)19	72	--	--	--	--	--	--	CGGTGTTTTGGGATTATTGA	58	CTTCCCTGTGCTTCATCCAT	60	242	--	--	--	--	--	--
TR48224|c0_g2_i1	No	(AT)8	16	--	--	--	--	--	--	CGCCTTCAATAGTCATTCCA	59	AAAGCTGCAAAATTGGAGGA	60	258	--	--	--	--	--	--
TR48224|c0_g3_i1	No	(AT)8	16	--	--	--	--	--	--	CGCCTTCAATAGTCATTCCA	59	AAAGCTGCAAAATTGGAGGA	60	258	--	--	--	--	--	--
TR48226|c0_g1_i1	No	(CCA)5	15	--	--	--	--	--	--	TGACGATATTGCTCACCACG	61	TAGTGGCGTTGGTTAGGGAC	60	210	--	--	--	--	--	--
TR48226|c0_g1_i2	No	(CCA)5	15	--	--	--	--	--	--	TGACGATATTGCTCACCACG	61	TAGTGGCGTTGGTTAGGGAC	60	210	--	--	--	--	--	--
TR48228|c0_g2_i1	XLOC_018929	(AT)6	12	--	--	--	--	--	--	CTCCTAACTGACCATTGGCAT	59	TGGTCGTGTTATGCCAGAAA	60	237	--	--	--	--	--	--
TR48232|c0_g1_i1	No	(TCT)7	21	--	--	--	--	--	--	AAAACATCGGCATCTTTTCG	60	CCACAATTTCCAACCTTTGATT	60	260	--	--	--	--	--	--
TR48232|c0_g2_i1	No	(TCT)7	21	--	--	--	--	--	--	AAAACATCGGCATCTTTTCG	60	CCACAATTTCCAACCTTTGATT	60	260	--	--	--	--	--	--
TR48239|c0_g1_i1	XLOC_018937	(AAC)5	15	--	--	--	--	--	--	TTCATTTCATCCCCTCTTGC	60	ATTCGGAGGCTTTGAGGTTT	60	149	--	--	XP_004513863.1	NF-YB	[S]	--
TR48248|c0_g1_i1	No	(CT)6	12	--	--	--	--	--	--	ACAGCTGTGTCCATTCCTGA	59	GCGAGGAGGAGAAAGAGATACA	60	262	--	--	--	--	--	--
TR48248|c0_g2_i1	No	(CT)6	12	--	--	--	--	--	--	ACAGCTGTGTCCATTCCTGA	59	GCGAGGAGGAGAAAGAGATACA	60	261	--	--	--	--	--	--
TR48276|c0_g1_i1	XLOC_018967	(CT)7	14	--	--	--	--	--	--	TGCATTCTACAAGCCTCCCT	60	TTCTCCAATTCCTCTGGCAC	60	209	--	--	--	--	--	--

Transcript IDs	Differentially expressed gene (DEG) IDs	SSR repeat-motifs	SSR repeat-length (bp)	*In silico* polymorphic SSRs						Forward primers (5'-3')	Annealing tempearture(0C)	Reverse primers (5'-3')	Annealing tempearture(0C)	Amplified product size (bp)	Structural annotation		Functional Annotation			
				C. microphyllum		ICC4958														
				Transcript	SSR repeat-motifs	Chromosomes	SSR repeat-motifs	Start physical positions (bp)	End physical positions (bp)						Gene IDs	Sequence component of genes	NCBI nr IDs	TFs	KOG	KEGG
TR48291|c0_g1_i1	No	(TC)8	16	--	--	--	--	--	--	AACTTTGGGACAGTGATGACG	60	AATGTACCAGCGATCCAAGC	60	262	--	--	--	--	--	--
TR48291|c0_g2_i1	XLOC_018978	(TC)8	16	--	--	--	--	--	--	AACTTTGGGACAGTGATGACG	60	AATGTACCAGCGATCCAAGC	60	262	--	--	--	--	--	--
TR48355|c0_g1_i1	No	(TG)6	12	--	--	--	--	--	--	TTTTGTGTTCAACGCTGCTC	60	AAGGTATCAAGGATGCCCAA	59	102	--	--	--	--	--	--
TR4836|c0_g1_i1	XLOC_019023	(TA)8gtggaagggaaatcatatactcattaaatcaaggaagg(T)10	64	--	--	--	--	--	--	GGCACATCACTGAAGTCCAAA	61	CACCTCCACCACTTCCACTT	60	249	--	--	XP_004497441.1	--	[T]	--
TR48388|c0_g1_i1	No	(CA)7	14	TR48388|c0_g1_i1	(CA)7	Ca_LG_6	(CA)6	5782038	5782049	TGGTTACCTGAAGTAATGTGGG	59	AGGAAAGCAAGTGCAGCCTA	60	247	Ca_09749	URR	--	--	--	--
TR484|c0_g1_i1	No	(TC)10gatctttccctctcccttgtct(TC)8	58	--	--	--	--	--	--	GTATATGCCGCTGGGTTCAT	60	AAGGGAAGAGTTTTTGGGGA	60	195	--	--	--	--	--	--
TR48426|c1_g1_i1	XLOC_019053	(TCT)7	21	--	--	--	--	--	--	ACAAAACCCTTCCTTGGCTT	60	ACAAGAAACATGGCATCGTG	60	151	--	--	--	--	--	--
TR48426|c1_g1_i1	XLOC_019053	(TCT)6	18	--	--	--	--	--	--	ATCACCATCGCCGTCTAATC	60	AGCCAAGGAAGGGTTTTGTT	60	171	--	--	--	--	--	--
TR48458|c0_g1_i1	XLOC_019080	(A)12tcttagtactgtgtttcataa(T)10cttgccttaaaaaaccaatgcaacataaccataaaaaaccctcgttcactgttcagtcactgtcccaattgaattccctttttccc(A)11	139	--	--	--	--	--	--	AATCGGAAAATTGCATGAGC	60	CGCTGAGACAGTACCGTGAG	60	278	--	--	--	--	--	--
TR48471|c0_g2_i1	No	(TC)7	14	--	--	--	--	--	--	TTTGCAGGTGAATGAATGGA	60	TTGCTGCAACTTTACTTGCCT	60	205	--	--	--	--	--	--
TR48474|c0_g1_i1	No	(GAT)5	15	--	--	--	--	--	--	AGCCTGCAAGACCACTCATT	60	CCAGTTCCCTCTTGAAGCAC	60	103	--	--	--	--	--	--
TR48474|c0_g1_i2	No	(GAT)5	15	--	--	--	--	--	--	AGCCTGCAAGACCACTCATT	60	CCAGTTCCCTCTTGAAGCAC	60	103	--	--	--	--	--	--
TR48477|c0_g1_i1	XLOC_019097	(AAT)6	18	--	--	--	--	--	--	CACAATGAAAGTTCACAGTTTTTC	58	GCTTGATGCTTCTTCCTTGG	60	264	--	--	XM_004515751.1	B3	[H]	--
TR48483|c0_g1_i1	XLOC_019102	(CAC)6	18	--	--	--	--	--	--	GCAATTCAACTCATCCCGAC	60	TAGCCATTTGTCCGAAGAGG	60	265	--	--	--	--	--	--

Transcript IDs	Differentially expressed gene (DEG) IDs	SSR repeat-motifs	SSR repeat-length (bp)	*In silico* polymorphic SSRs						Forward primers (5'-3')	Annealing tempearture(0C)	Reverse primers (5'-3')	Annealing tempearture(0C)	Amplified product size (bp)	Structural annotation		Functional Annotation			
				C. microphyllum		ICC4958									Gene IDs	Sequence component of genes	NCBI nr IDs	TFs	KOG	KEGG
				Transcript	SSR repeat-motifs	Chromosomes	SSR repeat-motifs	Start physical positions (bp)	End physical positions (bp)											
TR48484|c0_g1_i1	XLOC_019103	(CAT)5	15	--	--	--	--	--	--	TCTCAAAACCTCACCAAATCAA	60	TGCATGTTGGTGTTAATGGG	60	106	--	--	--	--	--	--
TR48501|c1_g1_i1	XLOC_019121	(ATA)6	18	--	--	--	--	--	--	TTCCAAGGCTAGCAGCAGAT	60	TGCAACTGCAACTCTCATCC	60	151	--	--	--	--	--	--
TR48502|c0_g1_i1	No	(GGA)6	18	--	--	--	--	--	--	AAGCACACAACACGAATCCA	60	ATCACCATGCTCCCTTTCAG	60	237	--	--	--	--	--	--
TR48502|c0_g2_i1	No	(GGA)6	18	--	--	--	--	--	--	TGAAGATGACGAGGGTTTGA	59	TCATGCACTTCAATACCGGA	60	234	--	--	--	--	--	--
TR48502|c0_g3_i1	No	(GGA)6	18	--	--	--	--	--	--	AAGCACACAACACGAATCCA	60	ATCACCATGCTCCCTTTCAG	60	263	--	--	--	--	--	--
TR48518|c0_g1_i1	No	(TCT)5	15	--	--	--	--	--	--	GGGTTGAACCTTTTCACAGC	60	CCTGGAATCAGCCGATTATG	60	256	--	--	--	--	--	--
TR48599|c0_g1_i1	No	(ATG)5	15	--	--	--	--	--	--	GCTGAACTTCCATTTCACTTCC	60	TCGCATTCACTCTTTCATCG	60	157	--	--	--	--	--	--
TR48626|c0_g1_i1	No	(CAA)6	18	--	--	--	--	--	--	ATCAGCCTCAACAAATGGGA	60	GAGGCTGCTGATTTAGTGGC	60	204	--	--	--	--	--	--
TR48665|c0_g1_i1	No	(TC)8	16	--	--	--	--	--	--	GAACTCAAAGATAACCTCATCATCTTC	60	TGTTGCTTCCTCTGTCATCG	60	240	--	--	--	--	--	--
TR48756|c0_g1_i1	No	(AACT)5	20	--	--	--	--	--	--	CAAGCACCAGTTATAAAAATAAGGTG	59	ACCATCTTGGTGCTAGCTGA	58	220	--	--	--	--	--	--
TR48779|c0_g1_i1	XLOC_019156	(GTT)5	15	--	--	--	--	--	--	GACGTTTGATCTATGTTGTTGACC	60	GGACATGCTATTTCCATCCC	59	100	--	--	--	--	--	--
TR48829|c1_g1_i1	No	(GA)6	12	--	--	--	--	--	--	CCCCAATCGTTAATTTTCCC	60	TTCTTTCGCTTCTTTGCCTC	60	165	--	--	--	--	--	--
TR48838|c0_g1_i1	XLOC_019169	(AAG)6	18	--	--	--	--	--	--	GTGAGCGATGAACTTGACGA	60	TTCACGTCTTTCCTTCTCGG	60	222	--	--	--	--	--	--
TR48848|c0_g1_i1	No	(CTG)5	15	--	--	--	--	--	--	GGATTGCACTTGATGGCTTT	60	AAGCAACAGCACAGATGGC	60	166	--	--	--	--	--	--

Transcript IDs	Differentially expressed gene (DEG) IDs	SSR repeat-motifs	SSR repeat-length (bp)	*In silico* polymorphic SSRs						Forward primers (5'-3')	Annealing tempearture(0C)	Reverse primers (5'-3')	Annealing tempearture(0C)	Amplified product size (bp)	Structural annotation		Functional Annotation			
				C. microphyllum		ICC4958									Gene IDs	Sequence component of genes	NCBI nr IDs	TFs	KOG	KEGG
				Transcript	SSR repeat-motifs	Chromosomes	SSR repeat-motifs	Start physical positions (bp)	End physical positions (bp)											
TR48885|c0_g2_i1	XLOC_019187	(TGA)6	18	--	--	--	--	--	--	ACTGCCTCTGTCCTGGCTTA	60	TGCTACTGCTCCTTCTGCAA	60	218	--	--	XM_004501628.1	--	[S]	--
TR48885|c0_g2_i1	XLOC_019187	(ATA)5	15	--	--	--	--	--	--	CCCAACAAAACACAGAAAAACA	60	TAAGCCAGGACAGAGGCAGT	60	190	--	--	XM_004501628.1	--	[S]	--
TR48924|c0_g2_i1	No	(TA)6	12	--	--	--	--	--	--	TGCACATTTGGATAGTTCGG	60	TTGTTATGTTTGACTTCCAATCTCA	60	170	--	--	--	--	--	--
TR48964|c0_g1_i1	XLOC_019232	(TA)6	12	--	--	--	--	--	--	TCTTAAAGCAAAGCATAAGAACAC	57	TCCTCCACCCAAATTTCATC	60	278	--	--	XP_012572301.1	--	[X]	--
TR48965|c0_g1_i1	No	(GCA)5	15	--	--	--	--	--	--	ATCCCCAGTTACAGCAGCAG	60	CGGCATGTTAGTTGGTCCTT	60	278	--	--	--	--	--	--
TR48992|c0_g2_i1	No	(CT)6	12	--	--	--	--	--	--	GGTGCCCCACTTACACTCAT	60	TTGGAAAACACAAGAAGGGG	60	149	--	--	--	--	--	--
TR48992|c0_g2_i1	No	(CT)6	12	--	--	--	--	--	--	GGTGCCCCACTTACACTCAT	60	TTGGAAAACACAAGAAGGGG	60	149	--	--	--	--	--	--
TR49087|c0_g1_i1	No	(TAG)5	15	--	--	--	--	--	--	GTCCTCTTCGTCCTGCTGTC	60	GTGTGGAGGAAGGGTTTGTG	60	251	--	--	--	--	--	--
TR4910|c0_g1_i1	No	(TCA)5	15	--	--	--	--	--	--	AGCCTGGAACCTGGATACCT	60	TGATGCTACAGATTATTCGGAGG	60	202	--	--	--	--	--	--
TR4910|c0_g1_i1	No	(TTG)6	18	--	--	--	--	--	--	TGTTTGGCAAGGATGATGAA	60	GAAGGTGAAATTGAGCCAGC	60	253	--	--	--	--	--	--
TR49103|c0_g1_i1	No	(CA)6	12	--	--	--	--	--	--	GGCAAAGAAACAAAACATGGA	60	TTCTTTATTGCCAGAAGCCC	59	235	--	--	--	--	--	--
TR49103|c0_g2_i1	No	(CA)6	12	--	--	--	--	--	--	GGCAAAGAAACAAAACATGGA	60	TTCTTTATTGCCAGAAGCCC	59	235	--	--	--	--	--	--
TR49117|c0_g1_i1	XLOC_019272	(CAT)8	24	--	--	--	--	--	--	ATTTGCACCAAGCAAGGATT	60	GCGTCCATAGTCAGGGTGAT	60	268	--	--	--	--	--	--
TR49134|c0_g1_i1	No	(AAC)5	15	--	--	--	--	--	--	ACACACGTCACACGAACCAT	60	CTCAAGATCAACGGAGGAGC	60	167	--	--	--	--	--	--

Transcript IDs	Differentially expressed gene (DEG) IDs	SSR repeat-motifs	SSR repeat-length (bp)	*In silico* polymorphic SSRs						Forward primers (5'-3')	Annealing tempearture(0C)	Reverse primers (5'-3')	Annealing tempearture(0C)	Amplified product size (bp)	Structural annotation		Functional Annotation			
				C. microphyllum		ICC4958									Gene IDs	Sequence component of genes	NCBI nr IDs	TFs	KOG	KEGG
				Transcript	SSR repeat-motifs	Chromosomes	SSR repeat-motifs	Start physical positions (bp)	End physical positions (bp)											
TR49134|c0_g2_i1	No	(TA)6	12	--	--	--	--	--	--	AGATGTACGGGCTCCAGTGA	61	AAGCATCAAGCACCAAGGAT	60	153	--	--	--	--	--	--
TR49134|c0_g3_i1	XLOC_019288	(AAC)5	15	--	--	--	--	--	--	ACACACGTCACACGAACCAT	60	CTCAAGATCAACGGAGGAGC	60	167	--	--	--	--	--	--
TR49161|c0_g2_i1	XLOC_019308	(A)11gaatatt(A)11	29	--	--	--	--	--	--	CACGAGTAGGTAATCAATCTTCAAA	59	CGGATGAACACTTGTACCCA	59	163	--	--	--	--	--	--
TR49161|c0_g3_i1	XLOC_019309	(A)11gaatatt(A)11	29	--	--	--	--	--	--	CACGAGTAGGTAATCAATCTTCAAA	59	CGGATGAACACTTGTACCCA	59	163	--	--	--	--	--	--
TR49190|c0_g1_i1	No	(TTC)5	15	--	--	--	--	--	--	TCAAATCCTCTGCCATTTCTC	59	GGGTGTTGAACCTTGCATTT	60	226	--	--	--	--	--	--
TR49190|c0_g2_i1	No	(TTC)5	15	--	--	--	--	--	--	TCAAATCCTCTGCCATTTCTC	59	GGGTGTTGAACCTTGCATTT	60	226	--	--	--	--	--	--
TR49384|c0_g2_i1	XLOC_019403	(CT)17	34	--	--	--	--	--	--	TTGAACGTAAAATAAGATCGCAA	59	GAGTCGTCCGAGAGGTTGAG	60	234	--	--	--	--	--	--
TR49386|c0_g1_i1	XLOC_019404	(AG)6	12	--	--	--	--	--	--	TCGAACATGCTGCTACAAAGA	60	CCCCTCTCTATCTCTCCCCTT	60	189	--	--	--	--	--	--
TR49389|c0_g1_i1	No	(TA)6	12	--	--	--	--	--	--	CCCTTCCGAACTAAACCCTC	60	AGCACCTTGAAACCTTCGAC	59	174	--	--	--	--	--	--
TR49403|c0_g1_i1	XLOC_019422	(CCA)5	15	--	--	--	--	--	--	TGAAGTAGCACCACCAGCAG	60	ATGGAGGATGCCACAAACTC	60	280	--	--	--	--	--	--
TR49411|c0_g1_i1	XLOC_019428	(ATC)5	15	--	--	--	--	--	--	ACGAATTGCGCACTAACTCA	59	GTGGAATTACAGGTGGCGTT	60	161	--	--	--	--	--	--
TR49411|c0_g2_i1	No	(ATC)5	15	--	--	--	--	--	--	ACGAATTGCGCACTAACTCA	59	GTGGAATTACAGGTGGCGTT	60	161	--	--	--	--	--	--
TR49432|c0_g1_i1	XLOC_019447	(AG)7	14	--	--	--	--	--	--	AGGAGCGGGTATGTTGTTTG	60	TTCAATTCCACGAAAACGAAG	60	264	--	--	KFM58711.1	--	[A]	K12614
TR49496|c0_g1_i1	XLOC_019489	(GA)7	14	--	--	--	--	--	--	CAGAACCGAAACAGGGAAAA	60	TTGAAAAAGCAGAGAGAAAGGG	60	238	--	--	--	--	--	--

Transcript IDs	Differentially expressed gene (DEG) IDs	SSR repeat-motifs	SSR repeat-length (bp)	In silico polymorphic SSRs						Forward primers (5'-3')	Annealing tempearture(0C)	Reverse primers (5'-3')	Annealing tempearture(0C)	Amplified product size (bp)	Structural annotation		Functional Annotation			
				C. microphyllum		ICC4958														
				Transcript	SSR repeat-motifs	Chromosomes	SSR repeat-motifs	Start physical positions (bp)	End physical positions (bp)						Gene IDs	Sequence component of genes	NCBI nr IDs	TFs	KOG	KEGG
TR49556|c0_g1_i1	XLOC_019509	(CAC)6	18	--	--	--	--	--	--	AATTTCATCCACCCACCCTC	61	GCTTTACTTGCATCAACACCC	60	277	--	--	XP_004488332.1	--	[KR]	--
TR49684|c0_g2_i1	No	(GTT)5aggaattggatagagagaggaagattaaaaact aggagagaaagtgatgaaattttggaatttggat(AGG)5	97	--	--	--	--	--	--	GGGTTGGTTTCAGTGATAAAGA	58	AAAAAGGAGTGAGAGTGAGAGA GT	57	176	--	--	--	--	--	--
TR49734|c0_g1_i1	XLOC_019575	(GAGT)5	20	--	--	--	--	--	--	GCACTTTGGAGAAAGCATCC	60	ATTGATGTTCCATGGGGAAA	60	225	--	--	--	--	--	--
TR4975|c0_g1_i1	No	(T)10agttaagaaatttttttattgg(T)10	42	--	--	--	--	--	--	CAAGAGATTCAATTTGATGTTTGAA	60	TGTGCTCTTCCGATCTTCCT	60	162	--	--	--	--	--	--
TR49765|c0_g1_i1	XLOC_019601	(ATA)6	18	--	--	--	--	--	--	GGATTTGGCTGTACCCTGTG	60	ACCATGAAGGAGATTGGTGC	60	203	--	--	XP_002520136.1	--	[C]	--
TR49767|c0_g2_i1	No	(AGA)5	15	--	--	--	--	--	--	GAGATTTGCATTGGCGAGAT	60	TTGTTGGGGTATGCCCTTTA	60	225	--	--	--	--	--	--
TR49774|c1_g1_i1	XLOC_019609	(CAA)5	15	--	--	--	--	--	--	CGGTTGTTTCTGTCGTTGAA	60	ACAACTCCAACCGTTTTTCG	60	148	--	--	--	--	--	--
TR49783|c0_g2_i1	No	(CT)10	20	--	--	--	--	--	--	TCGCGTAGCTTAGCAGAACA	60	ATGCAAAGCCAAATCACTCC	60	277	--	--	--	--	--	--
TR49783|c0_g2_i2	No	(CT)10	20	--	--	--	--	--	--	TCGCGTAGCTTAGCAGAACA	60	ATGCAAAGCCAAATCACTCC	60	277	--	--	--	--	--	--
TR49786|c0_g1_i1	No	(AGAAG)5	25	--	--	--	--	--	--	ATTAAGAAGCGGTGGTGTGG	60	CGTGTCTCTCCACTCTGTTCG	61	118	--	--	--	--	--	--
TR49786|c0_g2_i1	XLOC_019616	(AGAAG)5	25	--	--	--	--	--	--	ATTAAGAAGCGGTGGTGTGG	60	CGTGTCTCTCCACTCTGTTCG	61	139	--	--	--	--	--	--
TR49786|c1_g1_i1	XLOC_019617	(CA)7	14	--	--	--	--	--	--	ATTTGATTAGCATCAGGCGG	60	CCAAACTTGGGCAATGGTAT	60	181	--	--	--	--	--	--
TR49787|c0_g1_i1	No	(TA)6	12	--	--	--	--	--	--	GGAGAAAACCACACTCCAGG	60	CAGGTGTAGGACCAGTGGCT	60	172	--	--	--	--	--	--
TR49787|c0_g1_i2	No	(TA)6	12	--	--	--	--	--	--	GGAGAAAACCACACTCCAGG	60	CAGGTGTAGGACCAGTGGCT	60	172	--	--	--	--	--	--

Transcript IDs	Differentially expressed gene (DEG) IDs	SSR repeat-motifs	SSR repeat-length (bp)	*In silico* polymorphic SSRs						Forward primers (5'-3')	Annealing tempearture(0C)	Reverse primers (5'-3')	Annealing tempearture(0C)	Amplified product size (bp)	Structural annotation		Functional Annotation			
				C. microphyllum		ICC4958									Gene IDs	Sequence component of genes	NCBI nr IDs	TFs	KOG	KEGG
				Transcript	SSR repeat-motifs	Chromosomes	SSR repeat-motifs	Start physical positions (bp)	End physical positions (bp)											
TR49787|c0_g1_i3	No	(TA)6	12	--	--	--	--	--	--	GGAGAAAACCACACTCCAGG	60	CAGGTGTAGGACCAGTGGCT	60	172	--	--	--	--	--	--
TR49788|c0_g1_i1	XLOC_019618	(AAG)7	21	--	--	--	--	--	--	GGAAGTGCAAGTGTTGAGAGTT	59	GCACTGTTTGTTGTGAACCG	60	161	--	--	--	--	--	--
TR49796|c0_g2_i1	XLOC_019624	(GAA)5	15	--	--	--	--	--	--	GAGCTTGTTGGGTTTCGTGT	60	TCAAACACCAAAATAGCCCA	59	188	--	--	--	--	--	--
TR49813|c0_g1_i1	No	(AC)7	14	--	--	--	--	--	--	CAGTGCAGCCCACTAAACAA	60	CATTCGAGAAGGTAGCGGAG	60	231	--	--	--	--	--	--
TR49813|c0_g2_i1	No	(AC)7	14	--	--	--	--	--	--	CAGTGCAGCCCACTAAACAA	60	CATTCGAGAAGGTAGCGGAG	60	231	--	--	--	--	--	--
TR49828|c0_g3_i3	XLOC_019688	(GTT)5	15	--	--	--	--	--	--	TGACGGAAGGGTTATGGAAC	60	ACCATCACCGACGATCAAAT	60	190	--	--	--	--	--	--
TR49829|c1_g1_i2	XLOC_019695	(CA)8	16	--	--	--	--	--	--	AAAGCTCTTGGCACACAGGT	60	AACTTGGCCTCATAACCCTG	59	107	--	--	--	--	--	--
TR49829|c3_g1_i1	No	(TAA)5	15	--	--	--	--	--	--	AACACGAAAAATCTACCGCTG	59	AACTGACACCACCAGCAACA	60	168	--	--	--	--	--	--
TR49829|c3_g2_i1	XLOC_019696	(TAA)5	15	--	--	--	--	--	--	AACACGAAAAATCTACCGCTG	59	AACTGACACCACCAGCAACA	60	168	--	--	--	--	--	--
TR49829|c4_g4_i1	No	(TG)6	12	--	--	--	--	--	--	TAGCAGGAAATATGGGTCGG	60	CTGCAGGTCAGATTCGAACA	60	229	--	--	--	--	--	--
TR49829|c4_g4_i21	No	(TG)6	12	--	--	--	--	--	--	TAGCAGGAAATATGGGTCGG	60	CTGCAGGTCAGATTCGAACA	60	229	--	--	--	--	--	--
TR49829|c4_g4_i3	No	(TG)6	12	--	--	--	--	--	--	TAGCAGGAAATATGGGTCGG	60	CTGCAGGTCAGATTCGAACA	60	229	--	--	--	--	--	--
TR49829|c4_g5_i1	No	(TC)6	12	--	--	--	--	--	--	TGCAAAATACATGTACCATTTAAGG	59	GGAAGCTTCAACGAACAGAGA	60	233	--	--	--	--	--	--
TR49829|c4_g5_i2	No	(TC)6	12	--	--	--	--	--	--	TGCAAAATACATGTACCATTTAAGG	59	GGAAGCTTCAACGAACAGAGA	60	233	--	--	--	--	--	--

Transcript IDs	Differentially expressed gene (DEG) IDs	SSR repeat-motifs	SSR repeat-length (bp)	*In silico* polymorphic SSRs						Forward primers (5'-3')	Annealing tempearture(0C)	Reverse primers (5'-3')	Annealing tempearture(0C)	Amplified product size (bp)	Structural annotation		Functional Annotation			
				C. microphyllum		ICC4958									Gene IDs	Sequence component of genes	NCBI nr IDs	TFs	KOG	KEGG
				Transcript	SSR repeat-motifs	Chromosomes	SSR repeat-motifs	Start physical positions (bp)	End physical positions (bp)											
TR49829|c4_g5_i5	No	(TC)6	12	--	--	--	--	--	--	TGCAAAATACATGTACCATTTAAGG	59	GGAAGCTTCAACGAACAGAGA	60	233	--	--	--	--	--	--
TR49837|c0_g1_i1	No	(TCTCAT)6	36	--	--	--	--	--	--	CGGTTGGGAAGATTGGATTA	60	TGGCTTGCACTTCCTCTTTT	60	195	--	--	--	--	--	--
TR49837|c0_g1_i10	No	(TCTCAT)6	36	--	--	--	--	--	--	CGGTTGGGAAGATTGGATTA	60	TGGCTTGCACTTCCTCTTTT	60	195	--	--	--	--	--	--
TR49837|c0_g1_i3	No	(TCTCAT)6	36	--	--	--	--	--	--	CGGTTGGGAAGATTGGATTA	60	TGGCTTGCACTTCCTCTTTT	60	195	--	--	--	--	--	--
TR49837|c0_g1_i4	No	(TCTCAT)6	36	--	--	--	--	--	--	CGGTTGGGAAGATTGGATTA	60	TGGCTTGCACTTCCTCTTTT	60	195	--	--	--	--	--	--
TR49837|c0_g1_i5	No	(TCTCAT)6	36	--	--	--	--	--	--	CGGTTGGGAAGATTGGATTA	60	TGGCTTGCACTTCCTCTTTT	60	195	--	--	--	--	--	--
TR49840|c5_g7_i10	No	(CTC)7	21	--	--	--	--	--	--	TTCTTCCTCCTCCTCTTCCC	60	CCAACCGGTGATAATGTTGA	59	200	--	--	--	--	--	--
TR49840|c5_g7_i5	XLOC_019755	(CTC)7	21	--	--	--	--	--	--	CCTCTTCCCCATCAACTTCA	60	GTGCAAACAGCCATGACAAC	60	261	--	--	--	--	--	--
TR49842|c0_g1_i1	No	(AC)6	12	--	--	--	--	--	--	TGATTTCAGGAATGCTCACAA	59	ACCGCTATCCGGGATTAACT	60	265	--	--	--	--	--	--
TR49842|c4_g1_i2	XLOC_019761	(ATA)6	18	--	--	--	--	--	--	TGACAAGCTCGAATCCCTTT	60	CGACTTACAGATGGCGTCAA	60	280	--	--	--	--	--	--
TR49842|c4_g1_i3	XLOC_019762	(AAG)6aaaagcggtttaggtgaaaaggaggaggaagagaaagagaatgagaatgagaaagaggttaatttgggtgatgaagaggg(GAA)5	113	--	--	--	--	--	--	CTCGTCAACAACAAACCGTG	60	CCGATAATGTCCTCGTCGTT	60	229	--	--	--	--	--	--
TR49842|c4_g1_i4	XLOC_019763	(ATA)6	18	--	--	--	--	--	--	TGACAAGCTCGAATCCCTTT	60	CGACTTACAGATGGCGTCAA	60	280	--	--	--	--	--	--
TR49844|c1_g1_i5	XLOC_019766	(CT)8	16	--	--	--	--	--	--	TCCTTCCGAACCCTACACAC	60	GTCTTCACCAAGTCGGCAAT	60	192	--	--	--	--	--	--
TR49845|c1_g1_i1	XLOC_019772	(GTG)6	18	--	--	--	--	--	--	AGCCAGGTTCTGAGTGGAGA	60	CAGCAAATGTTCATGGCATC	60	272	--	--	--	--	--	--

Transcript IDs	Differentially expressed gene (DEG) IDs	SSR repeat-motifs	SSR repeat-length (bp)	*In silico* polymorphic SSRs						Forward primers (5'-3')	Annealing tempearture(0C)	Reverse primers (5'-3')	Annealing tempearture(0C)	Amplified product size (bp)	Structural annotation		Functional Annotation			
				C. microphyllum		ICC4958									Gene IDs	Sequence component of genes	NCBI nr IDs	TFs	KOG	KEGG
				Transcript	SSR repeat-motifs	Chromosomes	SSR repeat-motifs	Start physical positions (bp)	End physical positions (bp)											
TR49845|c4_g1_i1	No	(T)12cttctaaaatagggatttcaaatccttttatttttcttcata tgggcctagccctaaatgttcc(T)11	87	--	--	--	--	--	--	CTGTTACCACCACCGGAAAT	60	GGGTGGACTCACTCTCCAAG	60	269	--	--	--	--	--	--
TR49845|c6_g2_i1	No	(TTC)15	45	--	--	--	--	--	--	CGTTTCTGAGATAAACACTACCAC C	60	AGAGCCACCACTTTCCTTCA	60	117	--	--	--	--	--	--
TR49845|c6_g2_i2	No	(TTC)14	42	--	--	--	--	--	--	CGTTTCTGAGATAAACACTACCAC C	60	AGAGCCACCACTTTCCTTCA	60	114	--	--	--	--	--	--
TR49845|c6_g2_i3	No	(TTC)16	48	--	--	--	--	--	--	CGTTTCTGAGATAAACACTACCAC C	60	AGAGCCACCACTTTCCTTCA	60	120	--	--	--	--	--	--
TR49845|c8_g1_i1	XLOC_019776	(CT)6tccctcactttattcccactgtttcagctcagatcactg tctcatttctcaaatcaaaac(CT)7	86	--	--	--	--	--	--	GCGTGCATTACAAACACACA	59	CTTCAAACGGATTTCAGGGA	60	138	--	--	--	--	--	--
TR49848|c2_g1_i1	No	(TC)7	14	--	--	--	--	--	--	TTTATTTTGACGTCCGCACC	61	GTGTGAAAGTGGTGTCGTGG	60	251	--	--	--	--	--	--
TR49860|c5_g2_i1	XLOC_019843	(GA)6	12	--	--	--	--	--	--	TCTGCGAAAAGAAAACGAGG	60	CAACCATGGCACAAATTGAA	60	253	--	--	--	--	--	--
TR49862|c3_g4_i1	XLOC_019857	(GA)6	12	--	--	--	--	--	--	CTTGACCATTGACGGTTGTG	60	TAGAGTCACCATGACCCGCT	61	158	--	--	XP_013327603.1	--	--	--
TR49862|c3_g4_i4	XLOC_019859	(GA)6	12	--	--	--	--	--	--	CTTGACCATTGACGGTTGTG	60	TAGAGTCACCATGACCCGCT	61	158	--	--	EUC55051.1	--	--	--
TR49867|c0_g2_i1	No	(CAGAG)5	25	--	--	--	--	--	--	AGAGAGAGTGAGACGCGAGC	60	TAGGATCGTTTCCAAGCAGG	60	194	--	--	--	--	--	--
TR49868|c0_g1_i1	No	(TC)6	12	--	--	--	--	--	--	TAAGATCATTGCGGTTGGGT	60	TTCTGGAAAAAGGAGAGCGA	60	178	--	--	--	--	--	--
TR49868|c0_g1_i10	No	(TC)6	12	--	--	--	--	--	--	TAAGATCATTGCGGTTGGGT	60	TTCTGGAAAAAGGAGAGCGA	60	178	--	--	--	--	--	--
TR49868|c0_g1_i2	No	(TC)6	12	--	--	--	--	--	--	TAAGATCATTGCGGTTGGGT	60	TTCTGGAAAAAGGAGAGCGA	60	178	--	--	--	--	--	--
TR49868|c0_g1_i3	No	(TC)6	12	--	--	--	--	--	--	TAAGATCATTGCGGTTGGGT	60	TTCTGGAAAAAGGAGAGCGA	60	178	--	--	--	--	--	--

Transcript IDs	Differentially expressed gene (DEG) IDs	SSR repeat-motifs	SSR repeat-length (bp)	*In silico* polymorphic SSRs						Forward primers (5'-3')	Annealing tempearture(0C)	Reverse primers (5'-3')	Annealing tempearture(0C)	Amplified product size (bp)	Structural annotation		Functional Annotation			
				C. microphyllum		ICC4958									Gene IDs	Sequence component of genes	NCBI nr IDs	TFs	KOG	KEGG
				Transcript	SSR repeat-motifs	Chromosomes	SSR repeat-motifs	Start physical positions (bp)	End physical positions (bp)											
TR49868|c0_g1_i4	No	(TC)6	12	--	--	--	--	--	--	TAAGATCATTGCGGTTGGGT	60	TTCTGGAAAAAGGAGAGCGA	60	178	--	--	--	--	--	--
TR49868|c0_g1_i5	No	(TC)6	12	--	--	--	--	--	--	TAAGATCATTGCGGTTGGGT	60	TTCTGGAAAAAGGAGAGCGA	60	178	--	--	--	--	--	--
TR49868|c0_g1_i7	No	(TC)6	12	--	--	--	--	--	--	TAAGATCATTGCGGTTGGGT	60	TTCTGGAAAAAGGAGAGCGA	60	178	--	--	--	--	--	--
TR49868|c0_g1_i8	No	(TC)6	12	--	--	--	--	--	--	TAAGATCATTGCGGTTGGGT	60	TTCTGGAAAAAGGAGAGCGA	60	178	--	--	--	--	--	--
TR49868|c0_g1_i9	No	(TC)6	12	--	--	--	--	--	--	TAAGATCATTGCGGTTGGGT	60	TTCTGGAAAAAGGAGAGCGA	60	178	--	--	--	--	--	--
TR49868|c0_g2_i1	No	(TC)6	12	--	--	--	--	--	--	TAAGATCATTGCGGTTGGGT	60	TTCTGGAAAAAGGAGAGCGA	60	178	--	--	--	--	--	--
TR49868|c0_g4_i1	No	(TC)6	12	--	--	--	--	--	--	TAAGATCATTGCGGTTGGGT	60	TTCTGGAAAAAGGAGAGCGA	60	178	--	--	--	--	--	--
TR49870|c3_g2_i10	No	(GTTGT)5	25	--	--	--	--	--	--	CGGACCCTCACTTTCTTTCA	60	TGTCTTTGTGAGTGGTCCTGA	59	152	--	--	--	--	--	--
TR49870|c3_g2_i11	No	(GTTGT)5	25	--	--	--	--	--	--	CGGACCCTCACTTTCTTTCA	60	TCTCACCAAACCCACTTCAA	59	278	--	--	--	--	--	--
TR49870|c3_g2_i12	No	(GTTGT)5	25	--	--	--	--	--	--	CGGACCCTCACTTTCTTTCA	60	TGTCTTTGTGAGTGGTCCTGA	59	152	--	--	--	--	--	--
TR49870|c3_g2_i13	No	(GTTGT)5	25	--	--	--	--	--	--	CGGACCCTCACTTTCTTTCA	60	TCTCACCAAACCCACTTCAA	59	278	--	--	--	--	--	--
TR49870|c3_g2_i14	No	(GTTGT)5	25	--	--	--	--	--	--	CGGACCCTCACTTTCTTTCA	60	TCTCACCAAACCCACTTCAA	59	278	--	--	--	--	--	--
TR49870|c3_g2_i16	No	(GTTGT)5	25	--	--	--	--	--	--	CGGACCCTCACTTTCTTTCA	60	TCTCACCAAACCCACTTCAA	59	278	--	--	--	--	--	--
TR49870|c3_g2_i2	No	(GTTGT)5	25	--	--	--	--	--	--	CGGACCCTCACTTTCTTTCA	60	TCTCACCAAACCCACTTCAA	59	278	--	--	--	--	--	--

Transcript IDs	Differentially expressed gene (DEG) IDs	SSR repeat-motifs	SSR repeat-length (bp)	*In silico* polymorphic SSRs						Forward primers (5'-3')	Annealing tempearture(0C)	Reverse primers (5'-3')	Annealing tempearture(0C)	Amplified product size (bp)	Structural annotation		Functional Annotation			
				C. microphyllum		ICC4958									Gene IDs	Sequence component of genes	NCBI nr IDs	TFs	KOG	KEGG
				Transcript	SSR repeat-motifs	Chromosomes	SSR repeat-motifs	Start physical positions (bp)	End physical positions (bp)											
TR49870|c3_g2_i3	No	(GTTGT)5	25	--	--	--	--	--	--	CGGACCCTCACTTTCTTTCA	60	TGTCTTTGTGAGTGGTCCTGA	59	152	--	--	--	--	--	--
TR49870|c3_g2_i4	No	(GTTGT)5	25	--	--	--	--	--	--	CGGACCCTCACTTTCTTTCA	60	TCTCACCAAACCCACTTCAA	59	278	--	--	--	--	--	--
TR49870|c3_g2_i6	No	(GTTGT)5	25	--	--	--	--	--	--	CGGACCCTCACTTTCTTTCA	60	TGTCTTTGTGAGTGGTCCTGA	59	152	--	--	--	--	--	--
TR49870|c3_g2_i7	No	(GTTGT)5	25	--	--	--	--	--	--	CGGACCCTCACTTTCTTTCA	60	TCTCACCAAACCCACTTCAA	59	278	--	--	--	--	--	--
TR49870|c3_g2_i9	No	(GTTGT)5	25	--	--	--	--	--	--	CGGACCCTCACTTTCTTTCA	60	TGTCTTTGTGAGTGGTCCTGA	59	152	--	--	--	--	--	--
TR49874|c0_g2_i13	No	(AGG)5	15	--	--	--	--	--	--	TCAAAGAGATGGAAACCGCT	60	TTAGAGCGAAACGAGGCAAT	60	208	--	--	--	--	--	--
TR49874|c0_g2_i20	No	(TC)6	12	--	--	--	--	--	--	GCTCCTTTCATCTTCCCTCA	59	CTCTTCCTCCTCTGCCATTG	60	225	--	--	--	--	--	--
TR49874|c0_g2_i23	No	(AGG)5	15	--	--	--	--	--	--	TCAAAGAGATGGAAACCGCT	60	TTAGAGCGAAACGAGGCAAT	60	208	--	--	--	--	--	--
TR49874|c0_g2_i25	No	(TC)6	12	--	--	--	--	--	--	GCTCCTTTCATCTTCCCTCA	59	CTCTTCCTCCTCTGCCATTG	60	225	--	--	--	--	--	--
TR49874|c0_g2_i29	No	(AGG)5	15	--	--	--	--	--	--	TCAAAGAGATGGAAACCGCT	60	TTAGAGCGAAACGAGGCAAT	60	208	--	--	--	--	--	--
TR49874|c0_g2_i4	No	(TC)6	12	--	--	--	--	--	--	GCTCCTTTCATCTTCCCTCA	59	CTCTTCCTCCTCTGCCATTG	60	225	--	--	--	--	--	--
TR49880|c2_g3_i10	No	(AT)7	14	--	--	--	--	--	--	TGAAATGGAATGGAAAGGGA	60	GTGGATTGAGACGTGTGGTG	60	197	--	--	--	--	--	--
TR49880|c2_g3_i11	No	(AT)7	14	--	--	--	--	--	--	TGAAATGGAATGGAAAGGGA	60	GTGGATTGAGACGGGAAATG	60	180	--	--	--	--	--	--
TR49880|c2_g3_i14	No	(AT)7	14	--	--	--	--	--	--	TGAAATGGAATGGAAAGGGA	60	GTGGATTGAGACGTGTGGTG	60	197	--	--	--	--	--	--

Transcript IDs	Differentially expressed gene (DEG) IDs	SSR repeat-motifs	SSR repeat-length (bp)	*In silico* polymorphic SSRs						Forward primers (5'-3')	Annealing tempearture(0C)	Reverse primers (5'-3')	Annealing tempearture(0C)	Amplified product size (bp)	Structural annotation		Functional Annotation			
				C. microphyllum		ICC4958									Gene IDs	Sequence component of genes	NCBI nr IDs	TFs	KOG	KEGG
				Transcript	SSR repeat-motifs	Chromosomes	SSR repeat-motifs	Start physical positions (bp)	End physical positions (bp)											
TR49880|c2_g3_i6	No	(AT)7	14	--	--	--	--	--	--	TGAAATGGAATGGAAAGGGA	60	GTGGATTGAGACGGGAAATG	60	180	--	--	--	--	--	--
TR49884|c0_g6_i1	XLOC_019970	(GT)7	14	--	--	--	--	--	--	GGTCCAAGGAGCAGGGTAAT	60	CATCGTTGTTGCTTGTCGAT	60	269	--	--	--	--	--	--
TR49889|c1_g1_i1	XLOC_020003	(CA)8	16	--	--	--	--	--	--	GTCGTTCGCTCATTCCTCTC	60	AAACGACGAAAATCCAAACG	60	206	--	--	NP_001155531.1	--	[C]	K02155
TR49893|c0_g2_i1	No	(A)10gccaataattctctctatttccataactacactttcattgaatcattcctccccatcaagtc(A)16	88	--	--	--	--	--	--	TTTCCACGTAGTTTCCTTTGAA	59	GAATTGTGATGGTGCTTGGA	60	140	--	--	--	--	--	--
TR49893|c0_g2_i11	No	(A)10gccaataattctctctatttccataactacactttcattgaatcattcctccccatcaagtc(A)16	88	--	--	--	--	--	--	TTTCCACGTAGTTTCCTTTGAA	59	GAATTGTGATGGTGCTTGGA	60	140	--	--	--	--	--	--
TR49893|c0_g2_i12	No	(A)10gccaataattctctctatttccataactacactttcattgaatcattcctccccatcaagtc(A)16	88	--	--	--	--	--	--	TTTCCACGTAGTTTCCTTTGAA	59	GAATTGTGATGGTGCTTGGA	60	140	--	--	--	--	--	--
TR49893|c0_g2_i13	No	(A)10gccaataattctctctatttccataactacactttcattgaatcattcctccccatcaagtc(A)16	88	--	--	--	--	--	--	TTTCCACGTAGTTTCCTTTGAA	59	GAATTGTGATGGTGCTTGGA	60	140	--	--	--	--	--	--
TR49893|c0_g2_i14	No	(A)10gccaataattctctctatttccataactacactttcattgaatcattcctccccatcaagtc(A)16	88	--	--	--	--	--	--	TTTCCACGTAGTTTCCTTTGAA	59	GAATTGTGATGGTGCTTGGA	60	140	--	--	--	--	--	--
TR49893|c0_g2_i15	No	(A)10gccaataattctctctatttccataactacactttcattgaatcattcctccccatcaagtc(A)16	88	--	--	--	--	--	--	TTTCCACGTAGTTTCCTTTGAA	59	GAATTGTGATGGTGCTTGGA	60	140	--	--	--	--	--	--
TR49893|c0_g2_i3	No	(A)10gccaataattctctctatttccataactacactttcattgaatcattcctccccatcaagtc(A)16	88	--	--	--	--	--	--	TTTCCACGTAGTTTCCTTTGAA	59	GAATTGTGATGGTGCTTGGA	60	140	--	--	--	--	--	--
TR49893|c0_g2_i5	No	(A)10gccaataattctctctatttccataactacactttcattgaatcattcctccccatcaagtc(A)16	88	--	--	--	--	--	--	TTTCCACGTAGTTTCCTTTGAA	59	GAATTGTGATGGTGCTTGGA	60	140	--	--	--	--	--	--
TR49893|c0_g2_i6	No	(A)10gccaataattctctctatttccataactacactttcattgaatcattcctccccatcaagtc(A)16	88	--	--	--	--	--	--	TTTCCACGTAGTTTCCTTTGAA	59	GAATTGTGATGGTGCTTGGA	60	140	--	--	--	--	--	--
TR49893|c0_g2_i8	No	(A)10gccaataattctctctatttccataactacactttcattgaatcattcctccccatcaagtc(A)16	88	--	--	--	--	--	--	TTTCCACGTAGTTTCCTTTGAA	59	GAATTGTGATGGTGCTTGGA	60	140	--	--	--	--	--	--
TR49894|c0_g2_i1	No	(TCT)5	15	--	--	--	--	--	--	CGGCCATTCTTGACTCTCAT	60	TGAGCGAGCTCATCAAACTG	60	278	--	--	--	--	--	--

Transcript IDs	Differentially expressed gene (DEG) IDs	SSR repeat-motifs	SSR repeat-length (bp)	In silico polymorphic SSRs						Forward primers (5'-3')	Annealing tempearture(0C)	Reverse primers (5'-3')	Annealing tempearture(0C)	Amplified product size (bp)	Structural annotation		Functional Annotation			
				C. microphyllum		ICC4958									Gene IDs	Sequence component of genes	NCBI nr IDs	TFs	KOG	KEGG
				Transcript	SSR repeat-motifs	Chromosomes	SSR repeat-motifs	Start physical positions (bp)	End physical positions (bp)											
TR49894|c0_g2_i11	No	(TCT)5	15	--	--	--	--	--	--	CGGCCATTCTTGACTCTCAT	60	TGAGCGAGCTCATCAAACTG	60	278	--	--	--	--	--	--
TR49894|c0_g2_i13	XLOC_020034	(TCT)5	15	--	--	--	--	--	--	CGGCCATTCTTGACTCTCAT	60	TGAGCGAGCTCATCAAACTG	60	278	--	--	--	--	--	--
TR49894|c0_g2_i14	No	(TCT)5	15	--	--	--	--	--	--	CGGCCATTCTTGACTCTCAT	60	TGAGCGAGCTCATCAAACTG	60	278	--	--	--	--	--	--
TR49894|c0_g2_i2	No	(TCT)5	15	--	--	--	--	--	--	CGGCCATTCTTGACTCTCAT	60	TGAGCGAGCTCATCAAACTG	60	278	--	--	--	--	--	--
TR49894|c0_g2_i3	XLOC_020035	(TCT)6	18	--	--	--	--	--	--	CGTTCCTTTGATTGAGATTGC	60	ACTGTCCGAGGGACAAAATG	60	152	--	--	--	--	--	--
TR49894|c0_g2_i4	No	(TCT)6	18	--	--	--	--	--	--	CGTTCCTTTGATTGAGATTGC	60	ACTGTCCGAGGGACAAAATG	60	152	--	--	--	--	--	--
TR49894|c0_g2_i5	No	(TCT)5	15	--	--	--	--	--	--	CGGCCATTCTTGACTCTCAT	60	TGAGCGAGCTCATCAAACTG	60	278	--	--	--	--	--	--
TR49894|c0_g2_i6	No	(TCT)5	15	--	--	--	--	--	--	CGGCCATTCTTGACTCTCAT	60	TGAGCGAGCTCATCAAACTG	60	278	--	--	--	--	--	--
TR49894|c0_g2_i7	No	(TCT)6	18	--	--	--	--	--	--	CGTTCCTTTGATTGAGATTGC	60	ACTGTCCGAGGGACAAAATG	60	152	--	--	--	--	--	--
TR49894|c0_g2_i8	No	(TCT)5	15	--	--	--	--	--	--	CGGCCATTCTTGACTCTCAT	60	TGAGCGAGCTCATCAAACTG	60	278	--	--	--	--	--	--
TR49894|c0_g2_i9	No	(TCT)5	15	--	--	--	--	--	--	CGGCCATTCTTGACTCTCAT	60	TGAGCGAGCTCATCAAACTG	60	278	--	--	--	--	--	--
TR49895|c0_g1_i1	No	(TC)6	12	--	--	--	--	--	--	GCATCGTGTCATCTTCCCTT	60	CCGGAGAGCCGTTCATAATA	60	194	--	--	--	--	--	--
TR49895|c0_g1_i2	No	(TC)6	12	--	--	--	--	--	--	GCATCGTGTCATCTTCCCTT	60	CCGGAGAGCCGTTCATAATA	60	194	--	--	--	--	--	--
TR49895|c0_g1_i4	No	(TC)6	12	--	--	--	--	--	--	GCATCGTGTCATCTTCCCTT	60	CCGGAGAGCCGTTCATAATA	60	194	--	--	--	--	--	--

Transcript IDs	Differentially expressed gene (DEG) IDs	SSR repeat-motifs	SSR repeat-length (bp)	*In silico* polymorphic SSRs						Forward primers (5'-3')	Annealing tempearture(0C)	Reverse primers (5'-3')	Annealing tempearture(0C)	Amplified product size (bp)	Structural annotation		Functional Annotation			
				C. microphyllum		ICC4958									Gene IDs	Sequence component of genes	NCBI nr IDs	TFs	KOG	KEGG
				Transcript	SSR repeat-motifs	Chromosomes	SSR repeat-motifs	Start physical positions (bp)	End physical positions (bp)											
TR49895|c0_g1_i5	No	(TC)6	12	--	--	--	--	--	--	GCATCGTGTCATCTTCCCTT	60	CCGGAGAGCCGTTCATAATA	60	194	--	--	--	--	--	--
TR49895|c0_g1_i7	No	(TC)6	12	--	--	--	--	--	--	GCATCGTGTCATCTTCCCTT	60	CCGGAGAGCCGTTCATAATA	60	194	--	--	--	--	--	--
TR49895|c0_g1_i8	No	(TC)6	12	--	--	--	--	--	--	GCATCGTGTCATCTTCCCTT	60	CCGGAGAGCCGTTCATAATA	60	194	--	--	--	--	--	--
TR49897|c3_g1_i1	No	(AAT)5	15	--	--	--	--	--	--	GTGCGATTCACGAGTACACA	58	GGCGGCAGTGTGAAATAAGT	60	179	--	--	--	--	--	--
TR49897|c3_g1_i2	No	(AAT)5	15	--	--	--	--	--	--	GTGCGATTCACGAGTACACA	58	GGCGGCAGTGTGAAATAAGT	60	179	--	--	--	--	--	--
TR49897|c3_g2_i3	No	(AAT)5	15	--	--	--	--	--	--	TGCGATTCACGAGTACACACT	59	TTGCAACTGTGGTTGTGGTT	60	255	--	--	--	--	--	--
TR49898|c3_g3_i1	XLOC_020058	(TTA)5	15	--	--	--	--	--	--	TAAGCGTTGGTGATGTGGAA	60	TGCGATTCACGAGTACACACT	59	271	--	--	XP_004502101.1	--	--	--
TR49898|c3_g3_i2	No	(TA)6	12	--	--	--	--	--	--	GGGGATGGAAATTAGGGAAA	60	TGCAACTATGACCACGAGTACTT A	59	255	--	--	--	--	--	--
TR49898|c4_g1_i1	No	(TCA)7ccaccatcatcatcatcaccatcatt(ATC)5	62	--	--	--	--	--	--	TGTGCTAAAGATGGAAACTTTGA	59	TGGTTGAACGTAGAATGCCA	60	254	--	--	--	--	--	--
TR49898|c4_g1_i2	No	(TCT)5	15	--	--	--	--	--	--	GCTTTCACACGCTCAAACAA	60	TGAAAAGGGGGTGCAAATAG	60	265	--	--	--	--	--	--
TR49898|c4_g1_i2	No	(TCA)7ccaccatcatcatcatcaccatcatt(ATC)5	62	--	--	--	--	--	--	TGTGCTAAAGATGGAAACTTTGA	59	GAAAGTGGTGAACGTAAAGGG	59	216	--	--	--	--	--	--
TR49904|c5_g1_i8	No	(A)10cacaatctagaagtccacccataacaacctcattgg tcataactatttctaataataaaaactactacaccttgaaaag gttcctttctctatgcaac(T)10	118	--	--	--	--	--	--	TGGTGTATTCGAGGATCAAACA	60	TGGTCAGTTGGTTTAGTTTCCA	60	223	--	--	--	--	--	--
TR49909|c0_g3_i5	XLOC_020115	(GA)9	18	--	--	--	--	--	--	CGAGATTGGGAACGAGAGAG	60	TTCACTACCTCACCTCGCCT	60	277	--	--	--	--	--	--
TR49909|c0_g5_i6	No	(A)10ttctaaaagacccaaatcaagggttggcaaaaaaa atgcagcaaaaacaaacaaacaaaaaaaaactccaagg aaagcaaaattagaaaagtac(A)11	114	--	--	--	--	--	--	TTGCTGCAACACAAATCAAC	58	GAATTAACACCAATGAAAAATGA GC	60	166	--	--	--	--	--	--

<table>
<tr><th rowspan="3">Transcript IDs</th><th rowspan="3">Differentially expressed gene (DEG) IDs</th><th rowspan="3">SSR repeat-motifs</th><th rowspan="3">SSR repeat-length (bp)</th><th colspan="6">In silico polymorphic SSRs</th><th rowspan="3">Forward primers (5'-3')</th><th rowspan="3">Annealing tempearture(0C)</th><th rowspan="3">Reverse primers (5'-3')</th><th rowspan="3">Annealing tempearture(0C)</th><th rowspan="3">Amplified product size (bp)</th><th colspan="2">Structural annotation</th><th colspan="4">Functional Annotation</th></tr>
<tr><th colspan="2">C. microphyllum</th><th colspan="4">ICC4958</th><th rowspan="2">Gene IDs</th><th rowspan="2">Sequence component of genes</th><th rowspan="2">NCBI nr IDs</th><th rowspan="2">TFs</th><th rowspan="2">KOG</th><th rowspan="2">KEGG</th></tr>
<tr><th>Transcript</th><th>SSR repeat-motifs</th><th>Chromosomes</th><th>SSR repeat-motifs</th><th>Start physical positions (bp)</th><th>End physical positions (bp)</th></tr>
<tr><td>TR49909|c0_g5_i9</td><td>No</td><td>(A)10ttctaaaagacccaaatcaagggttggcaaaaaaaatgcagcaaaaacaaacaaacaaaaaaaaactccaaggaaagcaaaattagaaaagtac(A)11</td><td>114</td><td>--</td><td>--</td><td>--</td><td>--</td><td>--</td><td>--</td><td>TTGCTGCAACACAAATCAAC</td><td>58</td><td>GAATTAACACCAATGAAAAATGAGC</td><td>60</td><td>166</td><td>--</td><td>--</td><td>--</td><td>--</td><td>--</td><td>--</td></tr>
<tr><td>TR49910|c1_g2_i1</td><td>XLOC_020126</td><td>(A)10ttaataaaaagaaaagacaacatt(TC)8</td><td>50</td><td>--</td><td>--</td><td>--</td><td>--</td><td>--</td><td>--</td><td>TTTGCTCTTTTTGCACAATTC</td><td>58</td><td>TGCAAGAGATGAGCAAGATCC</td><td>60</td><td>231</td><td>--</td><td>--</td><td>XP_004516867.1</td><td>--</td><td>[P]</td><td>--</td></tr>
<tr><td>TR49910|c1_g4_i1</td><td>No</td><td>(A)10ttaataaaaagaaaagacaacatt(TC)8</td><td>50</td><td>--</td><td>--</td><td>--</td><td>--</td><td>--</td><td>--</td><td>TTTGCTCTTTTTGCACAATTC</td><td>58</td><td>TGCAAGAGATGAGCAAGATCC</td><td>60</td><td>231</td><td>--</td><td>--</td><td>--</td><td>--</td><td>--</td><td>--</td></tr>
<tr><td>TR49910|c1_g4_i2</td><td>No</td><td>(A)10ttaataaaaagaaaagacaacatt(TC)8</td><td>50</td><td>--</td><td>--</td><td>--</td><td>--</td><td>--</td><td>--</td><td>TTTGCTCTTTTTGCACAATTC</td><td>58</td><td>TGCAAGAGATGAGCAAGATCC</td><td>60</td><td>231</td><td>--</td><td>--</td><td>--</td><td>--</td><td>--</td><td>--</td></tr>
<tr><td>TR49910|c4_g1_i1</td><td>No</td><td>(CT)7</td><td>14</td><td>--</td><td>--</td><td>--</td><td>--</td><td>--</td><td>--</td><td>CACAATCTCTCTTTCTTTTCCCTT</td><td>59</td><td>AAATGCAGTGTGTTGCATGG</td><td>61</td><td>113</td><td>--</td><td>--</td><td>--</td><td>--</td><td>--</td><td>--</td></tr>
<tr><td>TR49912|c1_g2_i1</td><td>XLOC_020150</td><td>(CAG)5</td><td>15</td><td>--</td><td>--</td><td>--</td><td>--</td><td>--</td><td>--</td><td>GCAGCAACAGCTACAGCAAC</td><td>60</td><td>TGTTGGAAGAATCATGGCAA</td><td>60</td><td>199</td><td>--</td><td>--</td><td>--</td><td>--</td><td>--</td><td>--</td></tr>
<tr><td>TR49912|c2_g1_i10</td><td>XLOC_020151</td><td>(CAG)5</td><td>15</td><td>--</td><td>--</td><td>--</td><td>--</td><td>--</td><td>--</td><td>GGTATGCTTCAACACCAGCA</td><td>60</td><td>CCAATTCCATGCCTCATCTT</td><td>60</td><td>236</td><td>--</td><td>--</td><td>--</td><td>--</td><td>--</td><td>--</td></tr>
<tr><td>TR49912|c2_g1_i2</td><td>No</td><td>(AAC)6</td><td>18</td><td>--</td><td>--</td><td>--</td><td>--</td><td>--</td><td>--</td><td>CTTGCAACTTCCCCACTCTC</td><td>60</td><td>TGCTGCCTTATAACTTGGGG</td><td>60</td><td>257</td><td>--</td><td>--</td><td>--</td><td>--</td><td>--</td><td>--</td></tr>
<tr><td>TR49912|c2_g1_i3</td><td>No</td><td>(AAC)6</td><td>18</td><td>--</td><td>--</td><td>--</td><td>--</td><td>--</td><td>--</td><td>CTTGCAACTTCCCCACTCTC</td><td>60</td><td>TGCTGCCTTATAACTTGGGG</td><td>60</td><td>257</td><td>--</td><td>--</td><td>--</td><td>--</td><td>--</td><td>--</td></tr>
<tr><td>TR49912|c2_g1_i4</td><td>No</td><td>(AAC)6</td><td>18</td><td>--</td><td>--</td><td>--</td><td>--</td><td>--</td><td>--</td><td>CTTGCAACTTCCCCACTCTC</td><td>60</td><td>TGCTGCCTTATAACTTGGGG</td><td>60</td><td>257</td><td>--</td><td>--</td><td>--</td><td>--</td><td>--</td><td>--</td></tr>
<tr><td>TR49912|c2_g1_i6</td><td>No</td><td>(AAC)6</td><td>18</td><td>--</td><td>--</td><td>--</td><td>--</td><td>--</td><td>--</td><td>CTTGCAACTTCCCCACTCTC</td><td>60</td><td>TGCTGCCTTATAACTTGGGG</td><td>60</td><td>257</td><td>--</td><td>--</td><td>--</td><td>--</td><td>--</td><td>--</td></tr>
<tr><td>TR49912|c2_g1_i8</td><td>No</td><td>(AAC)6</td><td>18</td><td>--</td><td>--</td><td>--</td><td>--</td><td>--</td><td>--</td><td>CTTGCAACTTCCCCACTCTC</td><td>60</td><td>TGCTGCCTTATAACTTGGGG</td><td>60</td><td>257</td><td>--</td><td>--</td><td>--</td><td>--</td><td>--</td><td>--</td></tr>
<tr><td>TR49912|c2_g1_i9</td><td>No</td><td>(AAC)6</td><td>18</td><td>--</td><td>--</td><td>--</td><td>--</td><td>--</td><td>--</td><td>CTTGCAACTTCCCCACTCTC</td><td>60</td><td>TGCTGCCTTATAACTTGGGG</td><td>60</td><td>257</td><td>--</td><td>--</td><td>--</td><td>--</td><td>--</td><td>--</td></tr>
<tr><td>TR49913|c0_g1_i1</td><td>No</td><td>(CCA)5aagccttactactaccactcaccgcccccaccggtgtactctcctccacctccttaccattacattt(CAC)5</td><td>97</td><td>--</td><td>--</td><td>--</td><td>--</td><td>--</td><td>--</td><td>CCAAAACCACTTTGGGAAAA</td><td>60</td><td>GGTGGAGGAGGAGAAGGGTA</td><td>60</td><td>257</td><td>--</td><td>--</td><td>--</td><td>--</td><td>--</td><td>--</td></tr>
</table>

Transcript IDs	Differentially expressed gene (DEG) IDs	SSR repeat-motifs	SSR repeat-length (bp)	*In silico* polymorphic SSRs						Forward primers (5'-3')	Annealing tempearture(0C)	Reverse primers (5'-3')	Annealing tempearture(0C)	Amplified product size (bp)	Structural annotation		Functional Annotation			
				C. microphyllum		ICC4958														
				Transcript	SSR repeat-motifs	Chromosomes	SSR repeat-motifs	Start physical positions (bp)	End physical positions (bp)						Gene IDs	Sequence component of genes	NCBI nr IDs	TFs	KOG	KEGG
TR49913|c0_g2_i1	No	(CCA)5aagccttactactaccactcaccgcccccaccg gtgtactctcctccacctccttaccattacattt(CAC)5	97	--	--	--	--	--	--	CCAAAACCACTTTGGGAAAA	60	GGTGGAGGAGGAGAAGGGTA	60	257	--	--	--	--	--	--
TR49913|c1_g1_i1	XLOC_020154	(GGT)5	15	--	--	--	--	--	--	TGTTGGGATCCCTCTGTTGT	60	TCACTGCCCTCTTCTCATCA	59	178	--	--	--	--	--	--
TR49916|c0_g1_i1	XLOC_020178	(TTC)8	24	--	--	--	--	--	--	AATGAAATTGCTCCGTGAGG	60	CTCGAACCGGTTAGGGATTT	60	260	--	--	--	--	--	--
TR49916|c2_g5_i1	No	(TA)8	16	--	--	--	--	--	--	AGAAACAGGATTTTGACAGCC	58	TTTGTCCACACATCCTCTCAA	59	186	--	--	--	--	--	--
TR49916|c2_g5_i2	No	(TA)8	16	--	--	--	--	--	--	AGAAACAGGATTTTGACAGCC	58	TTTGTCCACACATCCTCTCAA	59	186	--	--	--	--	--	--
TR49916|c2_g5_i3	No	(TA)8	16	--	--	--	--	--	--	AGAAACAGGATTTTGACAGCC	58	TTTGTCCACACATCCTCTCAA	59	186	--	--	--	--	--	--
TR49916|c2_g5_i4	No	(TA)8	16	--	--	--	--	--	--	AGAAACAGGATTTTGACAGCC	58	TTTGTCCACACATCCTCTCAA	59	186	--	--	--	--	--	--
TR49916|c2_g5_i5	No	(TA)8	16	--	--	--	--	--	--	AGAAACAGGATTTTGACAGCC	58	TTTGTCCACACATCCTCTCAA	59	186	--	--	--	--	--	--
TR49916|c2_g5_i6	No	(TA)8	16	--	--	--	--	--	--	AGAAACAGGATTTTGACAGCC	58	TTTGTCCACACATCCTCTCAA	59	186	--	--	--	--	--	--
TR49916|c2_g8_i1	No	(CT)6	12	--	--	--	--	--	--	ATCCTCGTCCTCAGCACACT	60	TGCATAAACTTCTCGCGTTG	60	164	--	--	--	--	--	--
TR49916|c2_g8_i2	No	(CT)6	12	--	--	--	--	--	--	ATCCTCGTCCTCAGCACACT	60	GGGATCTCGCGTTGTAGAAG	60	158	--	--	--	--	--	--
TR49918|c4_g3_i1	No	(CT)8	16	--	--	--	--	--	--	TACCTTAGCCGCTTCATTGC	60	ATGCGGGATGTAATAGTGCC	60	263	--	--	--	--	--	--
TR49918|c4_g3_i1	No	(AG)8acatatagataataagagagagaatttcacaattgt gtttgttgtgtat(CCA)5	80	--	--	--	--	--	--	TGCTATGCAAAAATTGAGCG	60	TGAAGTGTGTCCACCCAAGA	60	197	--	--	--	--	--	--
TR49918|c4_g3_i4	No	(CT)8	16	--	--	--	--	--	--	TACCTTAGCCGCTTCATTGC	60	ATGCGGGATGTAATAGTGCC	60	263	--	--	--	--	--	--

<table>
<tr><th rowspan="3">Transcript IDs</th><th rowspan="3">Differentially expressed gene (DEG) IDs</th><th rowspan="3">SSR repeat-motifs</th><th rowspan="3">SSR repeat-length (bp)</th><th colspan="6">In silico polymorphic SSRs</th><th rowspan="3">Forward primers (5'-3')</th><th rowspan="3">Annealing tempearture(0C)</th><th rowspan="3">Reverse primers (5'-3')</th><th rowspan="3">Annealing tempearture(0C)</th><th rowspan="3">Amplified product size (bp)</th><th colspan="2">Structural annotation</th><th colspan="4">Functional Annotation</th></tr>
<tr><th colspan="2">C. microphyllum</th><th colspan="4">ICC4958</th><th rowspan="2">Gene IDs</th><th rowspan="2">Sequence component of genes</th><th rowspan="2">NCBI nr IDs</th><th rowspan="2">TFs</th><th rowspan="2">KOG</th><th rowspan="2">KEGG</th></tr>
<tr><th>Transcript</th><th>SSR repeat-motifs</th><th>Chromosomes</th><th>SSR repeat-motifs</th><th>Start physical positions (bp)</th><th>End physical positions (bp)</th></tr>
<tr><td>TR49918|c4_g3_i4</td><td>No</td><td>(AG)8acatatagataataagagagagaatttcacaattgtgtttgttgtgtat(CCA)5</td><td>80</td><td>--</td><td>--</td><td>--</td><td>--</td><td>--</td><td>--</td><td>TGCTATGCAAAAATTGAGCG</td><td>60</td><td>TGAAGTGTGTCCACCCAAGA</td><td>60</td><td>197</td><td>--</td><td>--</td><td>--</td><td>--</td><td>--</td><td>--</td></tr>
<tr><td>TR49918|c4_g3_i6</td><td>No</td><td>(CT)8</td><td>16</td><td>--</td><td>--</td><td>--</td><td>--</td><td>--</td><td>--</td><td>TTCATTACCGGCATCCTTCT</td><td>60</td><td>GCGGGATGTAATAGTGCCAT</td><td>60</td><td>249</td><td>--</td><td>--</td><td>--</td><td>--</td><td>--</td><td>--</td></tr>
<tr><td>TR49918|c4_g3_i8</td><td>No</td><td>(CT)8</td><td>16</td><td>--</td><td>--</td><td>--</td><td>--</td><td>--</td><td>--</td><td>TACCTTAGCCGCTTCATTGC</td><td>60</td><td>ATGCGGGATGTAATAGTGCC</td><td>60</td><td>263</td><td>--</td><td>--</td><td>--</td><td>--</td><td>--</td><td>--</td></tr>
<tr><td>TR49918|c4_g3_i8</td><td>No</td><td>(AG)8acatatagataataagagagagaatttcacaattgtgtttgttgtgtat(CCA)5</td><td>80</td><td>--</td><td>--</td><td>--</td><td>--</td><td>--</td><td>--</td><td>TGCTATGCAAAAATTGAGCG</td><td>60</td><td>TGAAGTGTGTCCACCCAAGA</td><td>60</td><td>197</td><td>--</td><td>--</td><td>--</td><td>--</td><td>--</td><td>--</td></tr>
<tr><td>TR50039|c0_g1_i1</td><td>No</td><td>(ACA)5</td><td>15</td><td>--</td><td>--</td><td>--</td><td>--</td><td>--</td><td>--</td><td>AGCAACCTCAGTCGCCTTTA</td><td>60</td><td>CTTGCGGTAGTAATTGCCGT</td><td>60</td><td>195</td><td>--</td><td>--</td><td>--</td><td>--</td><td>--</td><td>--</td></tr>
<tr><td>TR50060|c0_g1_i1</td><td>XLOC_020244</td><td>(TC)8</td><td>16</td><td>--</td><td>--</td><td>--</td><td>--</td><td>--</td><td>--</td><td>CTGTGGGAAAAAGCTCCAAG</td><td>60</td><td>TCGGAAGACCATTCATCCTC</td><td>60</td><td>278</td><td>--</td><td>--</td><td>--</td><td>--</td><td>--</td><td>--</td></tr>
<tr><td>TR50078|c0_g1_i1</td><td>No</td><td>(CTC)5</td><td>15</td><td>--</td><td>--</td><td>--</td><td>--</td><td>--</td><td>--</td><td>AGCTCTCAGCCCTGACTCAA</td><td>60</td><td>TCTGTTTGAACAGCAGGAACA</td><td>59</td><td>256</td><td>--</td><td>--</td><td>--</td><td>--</td><td>--</td><td>--</td></tr>
<tr><td>TR50078|c0_g1_i2</td><td>XLOC_020257</td><td>(CTC)5</td><td>15</td><td>--</td><td>--</td><td>--</td><td>--</td><td>--</td><td>--</td><td>AGCTCTCAGCCCTGACTCAA</td><td>60</td><td>TCTGTTTGAACAGCAGGAACA</td><td>59</td><td>256</td><td>--</td><td>--</td><td>--</td><td>--</td><td>--</td><td>--</td></tr>
<tr><td>TR50086|c1_g1_i1</td><td>XLOC_020266</td><td>(TTG)5</td><td>15</td><td>--</td><td>--</td><td>--</td><td>--</td><td>--</td><td>--</td><td>TGGTTGCCTTTCAGCTTCTT</td><td>60</td><td>TTGTGTTTGCCTTCCATTCA</td><td>60</td><td>240</td><td>--</td><td>--</td><td>KFM78010.1</td><td>--</td><td>[X]</td><td>--</td></tr>
<tr><td>TR50095|c0_g1_i1</td><td>XLOC_020274</td><td>(AAC)5</td><td>15</td><td>--</td><td>--</td><td>--</td><td>--</td><td>--</td><td>--</td><td>CAGAAAATGGGACACTGCCT</td><td>60</td><td>CAGCATTGGTGTTGATGGAG</td><td>60</td><td>268</td><td>--</td><td>--</td><td>--</td><td>--</td><td>--</td><td>--</td></tr>
<tr><td>TR50106|c0_g1_i1</td><td>No</td><td>(TC)8</td><td>16</td><td>--</td><td>--</td><td>--</td><td>--</td><td>--</td><td>--</td><td>GAATAATGAGGAGGGGAGGG</td><td>60</td><td>TGTCCAATTATCCATTCCACAA</td><td>60</td><td>160</td><td>--</td><td>--</td><td>--</td><td>--</td><td>--</td><td>--</td></tr>
<tr><td>TR50115|c0_g1_i1</td><td>No</td><td>(AG)6</td><td>12</td><td>--</td><td>--</td><td>--</td><td>--</td><td>--</td><td>--</td><td>GCAACATAAATTAAGTGCTGACTTG</td><td>59</td><td>GGGTGAATGTATGCCAACAA</td><td>59</td><td>133</td><td>--</td><td>--</td><td>--</td><td>--</td><td>--</td><td>--</td></tr>
<tr><td>TR50115|c0_g2_i1</td><td>XLOC_020298</td><td>(AG)6</td><td>12</td><td>--</td><td>--</td><td>--</td><td>--</td><td>--</td><td>--</td><td>GCAACATAAATTAAGTGCTGACTTG</td><td>59</td><td>GGGTGAATGTATGCCAACAA</td><td>59</td><td>133</td><td>--</td><td>--</td><td>--</td><td>--</td><td>--</td><td>--</td></tr>
<tr><td>TR5012|c0_g1_i1</td><td>No</td><td>(TA)6</td><td>12</td><td>--</td><td>--</td><td>--</td><td>--</td><td>--</td><td>--</td><td>TGGACTGCCATATTCAGGAAG</td><td>60</td><td>CCTGCACATCACATGCATAA</td><td>59</td><td>275</td><td>--</td><td>--</td><td>--</td><td>--</td><td>--</td><td>--</td></tr>
</table>

Transcript IDs	Differentially expressed gene (DEG) IDs	SSR repeat-motifs	SSR repeat-length (bp)	*In silico* polymorphic SSRs						Forward primers (5'-3')	Annealing tempearture(0C)	Reverse primers (5'-3')	Annealing tempearture(0C)	Amplified product size (bp)	Structural annotation		Functional Annotation			
				C. microphyllum		ICC4958									Gene IDs	Sequence component of genes	NCBI nr IDs	TFs	KOG	KEGG
				Transcript	SSR repeat-motifs	Chromosomes	SSR repeat-motifs	Start physical positions (bp)	End physical positions (bp)											
TR50133|c0_g1_i1	No	(TG)6	12	--	--	--	--	--	--	GGTCGACGCAGATTCAACTT	60	TGAGATTGAGCACCTTCGTG	60	268	--	--	--	--	--	--
TR50133|c0_g2_i1	No	(TG)6	12	--	--	--	--	--	--	GGTCGACGCAGATTCAACTT	60	TGAGATTGAGCACCTTCGTG	60	268	--	--	--	--	--	--
TR50141|c1_g1_i1	XLOC_020319	(TG)6	12	--	--	--	--	--	--	CCCCACAACTCCACATACCT	60	TTGTTTGGTTCATTTGAGAAGTTT	59	151	--	--	--	--	--	--
TR50154|c0_g1_i1	XLOC_020328	(CAC)8actccctcttacaaacaactactcctaacacacc caacatatatatataagaaaaaaggaacaattcctc(CTA)5	109	--	--	--	--	--	--	CACAACCAGATCAATGACTTCTC	59	GGTTTTGCGTGTGGATTTTC	60	182	--	--	--	--	--	--
TR50157|c0_g1_i1	XLOC_020329	(CA)7	14	--	--	--	--	--	--	CAAGCACATATCTTGCACTTCC	60	GTTGTCGGAAGAACCGTCTC	60	104	--	--	EGU82657.1	--	--	--
TR50164|c0_g1_i1	XLOC_020335	(TC)7	14	--	--	--	--	--	--	CAGGCATGGGTCAACATAAA	59	AAGACCAAGCATCACAACCC	60	190	--	--	--	--	--	--
TR5021|c0_g1_i1	No	(TCA)5	15	--	--	--	--	--	--	TTACATCAAGTGGGTCAAGGC	60	GCATACACGGATTTTGGCTT	60	101	--	--	--	--	--	--
TR50210|c0_g1_i1	XLOC_020355	(TCA)6	18	--	--	--	--	--	--	CCAATCTTCTTCCTTTCCCC	60	TGGGTTTGGGTTTGATTGTT	60	202	--	--	--	--	--	--
TR50210|c0_g1_i1	XLOC_020355	(TCA)5	15	--	--	--	--	--	--	TGCCATCATTTCACGAAGAA	60	GGGGAAAGGAAGAAGATTGG	60	146	--	--	--	--	--	--
TR50232|c0_g1_i1	No	(AAG)5	15	--	--	--	--	--	--	CGGTAAGGTCAAGGGTCAAA	60	ACCGTTTTACGTCGAGTTGG	60	163	--	--	--	--	--	--
TR50232|c0_g2_i1	XLOC_020363	(AAG)5	15	--	--	--	--	--	--	CGGTAAGGTCAAGGGTCAAA	60	ACCGTTTTACGTCGAGTTGG	60	163	--	--	XP_001653914.1	--	[JO]	--
TR50236|c0_g1_i1	XLOC_020364	(ACA)5	15	--	--	--	--	--	--	TTGTCCCTGATCCCTGAGTC	60	TGCTCTGATATGAGCCGTTG	60	215	--	--	XP_004498874.1	--	[O]	--
TR50365|c0_g1_i1	No	(CAA)5	15	--	--	--	--	--	--	TCTCCCTCTCTCAATCTCCG	59	ACGAAGAACCCACCATTGAG	60	217	--	--	--	--	--	--
TR50365|c0_g2_i1	No	(CAA)5	15	--	--	--	--	--	--	TCTCCCTCTCTCAATCTCCG	59	ACGAAGAACCCACCATTGAG	60	217	--	--	--	--	--	--

Transcript IDs	Differentially expressed gene (DEG) IDs	SSR repeat-motifs	SSR repeat-length (bp)	*In silico* polymorphic SSRs						Forward primers (5'-3')	Annealing tempearture(0C)	Reverse primers (5'-3')	Annealing tempearture(0C)	Amplified product size (bp)	Structural annotation		Functional Annotation			
				C. microphyllum		ICC4958									Gene IDs	Sequence component of genes	NCBI nr IDs	TFs	KOG	KEGG
				Transcript	SSR repeat-motifs	Chromosomes	SSR repeat-motifs	Start physical positions (bp)	End physical positions (bp)											
TR50422|c5_g1_i1	XLOC_020442	(A)10ttctgaaaaatataaaagttgtttttaagcctaatttgcggcgtatagtaatagtgggtattgaaggcttaattctgcagcaaacccag(A)12	111	--	--	--	--	--	--	GCCCTTGTTTTCCCATCTCT	60	AAACAGAAACTTCTTCTGGGTTTA	58	267	--	--	--	--	--	--
TR50424|c0_g1_i1	XLOC_020443	(TATT)6	24	--	--	--	--	--	--	TCACTCCCTCGATCCTCAAC	60	TTTCAAAGGAACAAAAACTTCACA	60	123	--	--	--	--	--	--
TR50426|c0_g1_i1	XLOC_020445	(TC)8	16	--	--	--	--	--	--	TAACACTCTCACGCTCACGG	60	AAATTGGTGAACCAGTTCGG	60	229	--	--	--	--	--	--
TR50428|c0_g1_i1	XLOC_020447	(AG)6	12	--	--	--	--	--	--	TGTGTCAGCTTTGGAACAACTC	60	CAGGTGCAATCTTGGAGACA	60	206	--	--	--	--	--	--
TR50431|c0_g1_i1	XLOC_020450	(TCA)5	15	--	--	--	--	--	--	GCTCCCATTCACTTTCACTTTC	60	GGTGGTAGGGTTTGGATGTG	60	248	--	--	--	--	--	--
TR50438|c0_g1_i1	No	(TTC)6	18	--	--	--	--	--	--	CGTTTGAGTGGGATGAGGTT	60	TCCCCAAAGTTCACTCTCGT	60	132	--	--	--	--	--	--
TR50442|c0_g1_i1	XLOC_020460	(CCA)6	18	--	--	--	--	--	--	TGAACTGTGGTCACGTCCTC	60	ATGCCTACCGGAAAGGTTCT	60	170	--	--	XP_004490280.1	--	--	--
TR50442|c0_g1_i2	XLOC_020461	(CCA)6	18	--	--	--	--	--	--	TGAACTGTGGTCACGTCCTC	60	ATGCCTACCGGAAAGGTTCT	60	170	--	--	XP_004490280.1	--	--	--
TR50442|c0_g2_i1	No	(CCA)6	18	--	--	--	--	--	--	TGAACTGTGGTCACGTCCTC	60	ATGCCTACCGGAAAGGTTCT	60	170	--	--	--	--	--	--
TR50448|c0_g1_i1	XLOC_020470	(TC)6	12	--	--	--	--	--	--	AGTCAAAGGGATGTCCGCTA	60	CACTGATGTGTTTCGGTTGG	60	277	--	--	--	--	--	--
TR50463|c0_g1_i1	No	(CT)8	16	--	--	--	--	--	--	ACCGGTCATCTAGTTGTGGC	60	GTCAATACAAAACTCGCGCA	60	205	--	--	--	--	--	--
TR50463|c0_g1_i4	No	(CT)8	16	--	--	--	--	--	--	ACCGGTCATCTAGTTGTGGC	60	GTCAATACAAAACTCGCGCA	60	205	--	--	--	--	--	--
TR50478|c0_g1_i1	XLOC_020499	(CTT)5	15	--	--	--	--	--	--	TCGGATTTCCATTTCCCATA	60	GTATCCACGCTTTGCCATTT	60	225	--	--	--	--	--	--
TR50489|c0_g1_i1	XLOC_020509	(TC)8	16	--	--	--	--	--	--	CGACAGCACAACACAACACA	60	TTGGTCGGTGTTTTGAATGA	60	269	--	--	--	--	--	--

Transcript IDs	Differentially expressed gene (DEG) IDs	SSR repeat-motifs	SSR repeat-length (bp)	*In silico* polymorphic SSRs						Forward primers (5'-3')	Annealing tempearture(0C)	Reverse primers (5'-3')	Annealing tempearture(0C)	Amplified product size (bp)	Structural annotation		Functional Annotation			
				C. microphyllum		**ICC4958**									**Gene IDs**	**Sequence component of genes**	**NCBI nr IDs**	**TFs**	**KOG**	**KEGG**
				**Transcript**	**SSR repeat-motifs**	**Chromosomes**	**SSR repeat-motifs**	**Start physical positions (bp)**	**End physical positions (bp)**											
TR50507|c0_g1_i1	XLOC_020523	(TCA)5	15	--	--	--	--	--	--	AAGGGCACTTTCTGCTTCCT	60	AACCATTTGTCTCTGGGACG	60	102	--	--	--	--	--	--
TR50507|c0_g1_i1	XLOC_020523	(GTT)5	15	--	--	--	--	--	--	ATTCCCCAAACCCTTTTGTT	60	AAATTGCGAGAGGAAGCAGA	60	278	--	--	--	--	--	--
TR50532|c0_g2_i1	XLOC_020542	(CT)6	12	--	--	--	--	--	--	GCTTCATTACAGCTTTGAAGCAC	60	GCCTAACGGTTCCATCGTAA	60	217	--	--	--	--	--	--
TR50533|c0_g1_i1	XLOC_020544	(CTC)5	15	--	--	--	--	--	--	CTCAAGAGAAAGTCAGCGCC	60	CTTTCCTGTTCCTGCTGCAT	60	118	--	--	XP_008870622.1	--	--	--
TR50538|c0_g2_i1	XLOC_020550	(AT)6acataaatacatactgtaataaacaattatataagtaagtgcggcgagttgttca(T)10gagaaat(A)11tagaataataaaaataatacttaaaaagctacggaattgttgattaactactagtatttgtattccgtgtcctttatattttttttctt(GAA)5	198	--	--	--	--	--	--	CTCACTGAATCATGTGGGACT	58	GCGTTGAGTTCCTTTGTAGG	57	280	--	--	--	--	--	--
TR50560|c0_g1_i1	XLOC_020567	(ATA)7	21	--	--	--	--	--	--	GGGTCATGGTCATAGCAACA	59	TGTTGGAGCTTCAGAAATGTAGTT	59	247	--	--	--	--	--	--
TR50561|c0_g1_i1	No	(AG)6	12	--	--	--	--	--	--	CTGCATCTCTCTCCTCTCCG	60	GTGTAACGGTGACGGTGTTG	60	267	--	--	--	--	--	--
TR50561|c0_g2_i1	No	(AG)6	12	--	--	--	--	--	--	CTGCATCTCTCTCCTCTCCG	60	GTGTAACGGTGACGGTGTTG	60	267	--	--	--	--	--	--
TR50561|c0_g3_i1	No	(AG)6	12	--	--	--	--	--	--	CTGCATCTCTCTCCTCTCCG	60	GTGTAACGGTGACGGTGTTG	60	267	--	--	--	--	--	--
TR50565|c1_g1_i1	XLOC_020570	(AC)6	12	--	--	--	--	--	--	TTGCATTGCATTTTGAGGAA	60	TTGAAATCCTTGTTTTGGCA	59	248	--	--	--	--	--	--
TR50576|c0_g1_i1	No	(CT)9	18	--	--	--	--	--	--	ATGGAAGAAGATCAAAGCGG	59	GAATTTTCTTGTTGGAGCGG	60	194	--	--	--	--	--	--
TR50576|c0_g2_i1	No	(CT)9	18	--	--	--	--	--	--	ATGGAAGAAGATCAAAGCGG	59	GAATTTTCTTGTTGGAGCGG	60	194	--	--	--	--	--	--
TR50586|c0_g1_i1	XLOC_020587	(TCC)6	18	TR50586|c0_g1_i1	(TCC)6	Ca_LG_6	(TCC)7	18572772	18572792	TGTGGAATACCCTTCTCCAA	58	GCCCTTGAATTGGTTGTGAT	60	242	Ca_14178	URR	XM_004505152.1	--	--	--
TR50600|c1_g1_i1	XLOC_020597	(CT)9	18	--	--	--	--	--	--	CAAACGGCGAATAAATGCTT	60	ACAAGAACCAAAAAGGGGCT	60	246	--	--	--	--	--	--

Transcript IDs	Differentially expressed gene (DEG) IDs	SSR repeat-motifs	SSR repeat-length (bp)	*In silico* polymorphic SSRs						Forward primers (5'-3')	Annealing tempearture(0C)	Reverse primers (5'-3')	Annealing tempearture(0C)	Amplified product size (bp)	Structural annotation		Functional Annotation			
				C. microphyllum		ICC4958									Gene IDs	Sequence component of genes	NCBI nr IDs	TFs	KOG	KEGG
				Transcript	SSR repeat-motifs	Chromosomes	SSR repeat-motifs	Start physical positions (bp)	End physical positions (bp)											
TR50601|c1_g1_i1	XLOC_020600	(ATC)5	15	--	--	--	--	--	--	CTTGTCGTGCTCTTCCTTCC	60	AGGGTAAGGGTGGGATTTTG	60	216	--	--	--	--	--	--
TR50622|c0_g1_i1	No	(TGC)6	18	--	--	--	--	--	--	GTGGTGGGTTGAGAGGTGAT	60	ATCACCAGAACCAGAGCCAG	60	159	--	--	--	--	--	--
TR50694|c0_g1_i1	XLOC_020623	(TAC)5	15	--	--	--	--	--	--	GACGACATGGAATTGGAACA	59	TGAGAAATTGGTGATGGACG	59	267	--	--	XP_004510853.1	--	[X]	--
TR50709|c0_g1_i1	No	(GAA)7	21	--	--	--	--	--	--	TAAATTCCGATTACGCCGAG	60	ACTCCAATCCATGCCAAGAG	60	175	--	--	--	--	--	--
TR50709|c0_g1_i2	No	(GAA)7	21	--	--	--	--	--	--	TAAATTCCGATTACGCCGAG	60	ACTCCAATCCATGCCAAGAG	60	175	--	--	--	--	--	--
TR50709|c0_g1_i3	No	(GAA)7	21	--	--	--	--	--	--	TAAATTCCGATTACGCCGAG	60	ACTCCAATCCATGCCAAGAG	60	175	--	--	--	--	--	--
TR50709|c0_g1_i4	No	(GAA)7	21	--	--	--	--	--	--	TAAATTCCGATTACGCCGAG	60	ACTCCAATCCATGCCAAGAG	60	175	--	--	--	--	--	--
TR50709|c0_g1_i5	No	(GAA)7	21	--	--	--	--	--	--	TAAATTCCGATTACGCCGAG	60	ACTCCAATCCATGCCAAGAG	60	175	--	--	--	--	--	--
TR50710|c0_g1_i1	XLOC_020644	(ACA)5	15	--	--	--	--	--	--	TCTCACATCCAGGTTCTCCA	59	GCCACAGAGTTGGATTTGGT	60	167	--	--	--	--	--	--
TR50711|c1_g2_i1	XLOC_020647	(TAC)6	18	TR50711|c1_g2_i1	(TAC)6	Ca_LG_4	(TAC)7	12461504	12461524	CAAGTGTGGGCATCTCAGAA	60	ATTTGGGCCTGAGAAGGTTT	60	267	Ca_00514	CDS	--	--	--	--
TR50722|c0_g1_i3	No	(TAT)5	15	--	--	--	--	--	--	CACGCATGACTGGAATTTTG	60	GGGAACATAAAGAGGTGGCTC	60	260	--	--	--	--	--	--
TR50729|c0_g1_i1	No	(CAT)5	15	--	--	--	--	--	--	ACACATCATGCCTCCTTTCC	60	AGTTACCACGGGGAACTCCT	60	200	--	--	--	--	--	--
TR50729|c0_g1_i2	No	(CAT)5	15	--	--	--	--	--	--	ACACATCATGCCTCCTTTCC	60	AGTTACCACGGGGAACTCCT	60	200	--	--	--	--	--	--
TR50731|c0_g1_i2	No	(GT)9	18	--	--	--	--	--	--	GCCTTTCAGTTGTTTGGCAT	60	CCCTCCCAGAGAGGCTTAAC	60	280	--	--	--	--	--	--

Transcript IDs	Differentially expressed gene (DEG) IDs	SSR repeat-motifs	SSR repeat-length (bp)	*In silico* polymorphic SSRs						Forward primers (5'-3')	Annealing tempearture(0C)	Reverse primers (5'-3')	Annealing tempearture(0C)	Amplified product size (bp)	Structural annotation		Functional Annotation			
				C. microphyllum		ICC4958														
				Transcript	SSR repeat-motifs	Chromosomes	SSR repeat-motifs	Start physical positions (bp)	End physical positions (bp)						Gene IDs	Sequence component of genes	NCBI nr IDs	TFs	KOG	KEGG
TR50731|c0_g1_i7	No	(GT)9	18	--	--	--	--	--	--	GCCTTTCAGTTGTTTGGCAT	60	CCCTCCCAGAGAGGCTTAAC	60	280	--	--	--	--	--	--
TR50731|c0_g1_i9	No	(GT)9	18	--	--	--	--	--	--	GCCTTTCAGTTGTTTGGCAT	60	CCCTCCCAGAGAGGCTTAAC	60	280	--	--	--	--	--	--
TR50733|c0_g1_i1	No	(ATC)7	21	--	--	--	--	--	--	CTGACGTGAGTGGCATGATT	60	GGCCGAGATTCATTGTTTTC	60	276	--	--	--	--	--	--
TR50733|c0_g1_i2	No	(ATC)7	21	--	--	--	--	--	--	CTGACGTGAGTGGCATGATT	60	GGCCGAGATTCATTGTTTTC	60	276	--	--	--	--	--	--
TR50733|c0_g1_i3	No	(ATC)7	21	--	--	--	--	--	--	CTGACGTGAGTGGCATGATT	60	GGCCGAGATTCATTGTTTTC	60	276	--	--	--	--	--	--
TR50733|c0_g1_i4	No	(ATC)7	21	--	--	--	--	--	--	CTGACGTGAGTGGCATGATT	60	GGCCGAGATTCATTGTTTTC	60	276	--	--	--	--	--	--
TR50733|c0_g1_i5	No	(ATC)7	21	--	--	--	--	--	--	CTGACGTGAGTGGCATGATT	60	GGCCGAGATTCATTGTTTTC	60	276	--	--	--	--	--	--
TR50736|c0_g1_i1	No	(TA)6	12	--	--	--	--	--	--	AAAAAGTCGGTCCCCAATCT	60	ATGAGCATGTCACTGGTTGC	60	269	--	--	--	--	--	--
TR50736|c0_g1_i2	No	(TA)6	12	--	--	--	--	--	--	AAAAAGTCGGTCCCCAATCT	60	ATGAGCATGTCACTGGTTGC	60	269	--	--	--	--	--	--
TR50736|c0_g1_i3	No	(TA)6	12	--	--	--	--	--	--	AAAAAGTCGGTCCCCAATCT	60	ATGAGCATGTCACTGGTTGC	60	269	--	--	--	--	--	--
TR50736|c0_g1_i4	No	(TA)6	12	--	--	--	--	--	--	AAAAAGTCGGTCCCCAATCT	60	ATGAGCATGTCACTGGTTGC	60	269	--	--	--	--	--	--
TR50737|c0_g1_i1	No	(TA)8	16	--	--	--	--	--	--	GGAAAATTTGCAATTTGAAGAGA	60	TGACTTCCATTAACCACCCA	59	280	--	--	--	--	--	--
TR50737|c0_g1_i2	No	(TA)8	16	--	--	--	--	--	--	GGAAAATTTGCAATTTGAAGAGA	60	TGACTTCCATTAACCACCCA	59	280	--	--	--	--	--	--
TR50737|c0_g1_i4	No	(TA)8	16	--	--	--	--	--	--	GGAAAATTTGCAATTTGAAGAGA	60	TGACTTCCATTAACCACCCA	59	280	--	--	--	--	--	--

Transcript IDs	Differentially expressed gene (DEG) IDs	SSR repeat-motifs	SSR repeat-length (bp)	*In silico* polymorphic SSRs						Forward primers (5'-3')	Annealing tempearture(0C)	Reverse primers (5'-3')	Annealing tempearture(0C)	Amplified product size (bp)	Structural annotation		Functional Annotation			
				C. microphyllum		ICC4958														
				Transcript	SSR repeat-motifs	Chromosomes	SSR repeat-motifs	Start physical positions (bp)	End physical positions (bp)						Gene IDs	Sequence component of genes	NCBI nr IDs	TFs	KOG	KEGG
TR50737|c0_g1_i5	No	(CT)6	12	--	--	--	--	--	--	CGCTCCAAGGTCTTCTTCAT	59	AAGCACAGACCGATTCCAAC	60	194	--	--	--	--	--	--
TR50737|c0_g1_i6	No	(CT)6	12	--	--	--	--	--	--	CGCTCCAAGGTCTTCTTCAT	59	AGGCTGGATCATTGACATCC	60	229	--	--	--	--	--	--
TR50745|c1_g1_i1	XLOC_020678	(TAA)5	15	--	--	--	--	--	--	CACGGCTGTCATTTGAGTTG	60	AATGGGAGTTGCTTTGCTTG	60	148	--	--	--	--	--	--
TR50747|c1_g1_i1	XLOC_020681	(TC)6	12	--	--	--	--	--	--	CATGACAAGGAGGAAGCTCAG	60	CCTCTTCCGTTTTTCATCCA	60	227	--	--	--	--	--	--
TR50748|c0_g1_i10	No	(TGA)5	15	--	--	--	--	--	--	TTGATGCACGGACAAGAGAG	60	TTGGAGAATTTTGATGCCAA	59	273	--	--	--	--	--	--
TR50755|c1_g1_i1	XLOC_020693	(ATT)6(GTT)12	54	--	--	--	--	--	--	GCTATCCTGGTACGTGGAGG	60	GCCGGGATTGACAACATACT	60	241	--	--	--	--	--	--
TR50764|c0_g1_i1	XLOC_020704	(AAC)6	18	--	--	--	--	--	--	TCCTCCCTCTCAAAATCCCT	60	GGATTATTTTGGAGCGGGTT	60	217	--	--	--	--	--	--
TR50764|c0_g1_i2	XLOC_020705	(AAC)6	18	--	--	--	--	--	--	TCCTCCCTCTCAAAATCCCT	60	GGATTATTTTGGAGCGGGTT	60	217	--	--	--	--	--	--
TR50765|c0_g1_i1	XLOC_020708	(AGA)5	15	--	--	--	--	--	--	GCAAGGTAGATCGGATTGGA	60	ACCAAGGTCATCACTCCAGC	60	196	--	--	--	--	--	--
TR50767|c0_g2_i1	XLOC_020710	(GAG)6	18	--	--	--	--	--	--	AAGAAGGTTCTCGCTGTTGC	60	GTCAAAGAGACCACCGAAGC	60	204	--	--	XP_003051714.1	--	[J]	--
TR50772|c0_g1_i1	No	(TA)6	12	--	--	--	--	--	--	AAGGGTTGTTGATTTGGCTG	60	TTGCAGAATGAGGAGCAAGA	60	211	--	--	--	--	--	--
TR50775|c0_g1_i1	XLOC_020719	(ATA)5	15	TR50775|c0_g1_i1	(ATA)5	Ca_LG_3	(ATA)7	33924936	33924956	GCAGAGCCAAAAATCCAAAA	60	ACAAACAAAATGCGAAAGGG	60	182		URR	--	--	--	--
TR50775|c0_g1_i1	XLOC_020719	(TTA)5	15	TR50775|c0_g1_i1	(ATA)5	Ca_LG_3	(ATA)7	33924936	33924956	GTCACGGCAAAATACACCCT	60	GAATGCTGCTACTGCTGCTG	60	172		URR	--	--	--	--
TR50783|c0_g1_i1	No	(TCA)5	15	--	--	--	--	--	--	AGGATGGGTCACGAACACTC	60	GGTGAAGAGAGCGATTCCAG	60	180	--	--	--	--	--	--

Transcript IDs	Differentially expressed gene (DEG) IDs	SSR repeat-motifs	SSR repeat-length (bp)	*In silico* polymorphic SSRs						Forward primers (5'-3')	Annealing tempearture(0C)	Reverse primers (5'-3')	Annealing tempearture(0C)	Amplified product size (bp)	Structural annotation		Functional Annotation			
				C. microphyllum		ICC4958														
				Transcript	SSR repeat-motifs	Chromosomes	SSR repeat-motifs	Start physical positions (bp)	End physical positions (bp)						Gene IDs	Sequence component of genes	NCBI nr IDs	TFs	KOG	KEGG
TR50783|c0_g1_i2	No	(TCA)5	15	--	--	--	--	--	--	AGGATGGGTCACGAACACTC	60	GGTGAAGAGAGCGATTCCAG	60	180	--	--	--	--	--	--
TR50783|c0_g1_i3	No	(TCA)5	15	--	--	--	--	--	--	AGGATGGGTCACGAACACTC	60	GGTGAAGAGAGCGATTCCAG	60	180	--	--	--	--	--	--
TR50783|c0_g1_i4	No	(TCA)5	15	--	--	--	--	--	--	AGGATGGGTCACGAACACTC	60	GGTGAAGAGAGCGATTCCAG	60	180	--	--	--	--	--	--
TR50783|c0_g1_i5	No	(TCA)5	15	--	--	--	--	--	--	AGGATGGGTCACGAACACTC	60	GGTGAAGAGAGCGATTCCAG	60	180	--	--	--	--	--	--
TR50783|c0_g1_i6	No	(TCA)5	15	--	--	--	--	--	--	AGGATGGGTCACGAACACTC	60	GGTGAAGAGAGCGATTCCAG	60	180	--	--	--	--	--	--
TR50783|c0_g1_i7	No	(TCA)5	15	--	--	--	--	--	--	AGGATGGGTCACGAACACTC	60	GGTGAAGAGAGCGATTCCAG	60	180	--	--	--	--	--	--
TR50783|c0_g1_i8	No	(TCA)5	15	--	--	--	--	--	--	AGGATGGGTCACGAACACTC	60	GGTGAAGAGAGCGATTCCAG	60	180	--	--	--	--	--	--
TR50783|c0_g1_i9	No	(TCA)5	15	--	--	--	--	--	--	AGGATGGGTCACGAACACTC	60	GGTGAAGAGAGCGATTCCAG	60	180	--	--	--	--	--	--
TR50859|c0_g1_i1	XLOC_020739	(AGA)5	15	--	--	--	--	--	--	AGCAGGATCATTCACATCCC	60	CTAGCTTGCATTGATTTGCG	60	275	--	--	--	--	--	--
TR50861|c0_g1_i1	XLOC_020740	(TTA)5	15	--	--	--	--	--	--	CCCGAGTGCATGAATTTGTT	61	GCAGCTGATAGAGCCTGTCC	60	143	--	--	NP_001155701.1	--	--	--
TR50901|c0_g1_i1	No	(AT)6	12	TR50901|c0_g1_i1	(AT)6	Ca_LG_6	(AT)8	1909792	1909807	TAGGGGACGGAAAACACTTG	60	TCAACGCTGCTTTCAGACAT	60	277	Ca_09473	URR	--	--	--	--
TR50902|c0_g1_i1	XLOC_020770	(AC)9	18	--	--	--	--	--	--	CATCTCCATCCTCCTCCCC	62	CTCCCCCGTAACACCATCTA	60	252	--	--	XP_003246532.1	--	[IR]	--
TR50925|c0_g2_i1	No	(TG)6	12	--	--	--	--	--	--	TTGGATGGAAGGGTAACGAC	60	TATCCTCAGGTGCACATCCA	60	276	--	--	--	--	--	--
TR50925|c0_g2_i2	No	(TG)6	12	--	--	--	--	--	--	TTGGATGGAAGGGTAACGAC	60	TATCCTCAGGTGCACATCCA	60	276	--	--	--	--	--	--

Transcript IDs	Differentially expressed gene (DEG) IDs	SSR repeat-motifs	SSR repeat-length (bp)	*In silico* polymorphic SSRs						Forward primers (5'-3')	Annealing tempearture(0C)	Reverse primers (5'-3')	Annealing tempearture(0C)	Amplified product size (bp)	Structural annotation		Functional Annotation			
				C. microphyllum		ICC4958														
				Transcript	SSR repeat-motifs	Chromosomes	SSR repeat-motifs	Start physical positions (bp)	End physical positions (bp)						Gene IDs	Sequence component of genes	NCBI nr IDs	TFs	KOG	KEGG
TR50934|c1_g1_i1	XLOC_020794	(TC)7	14	--	--	--	--	--	--	ACACACACTTCAGCGTCGTT	59	CTTCCTCCTCCCATTCACAA	60	137	--	--	--	--	--	--
TR50944|c1_g1_i1	XLOC_020804	(AAC)7	21	--	--	--	--	--	--	GCATGAACCCCAGTTTGAGT	60	TGCCTTGTGGTGTTAACGAG	60	247	--	--	--	--	--	--
TR50976|c1_g2_i1	XLOC_020838	(TA)7	14	--	--	--	--	--	--	GGGACAAGCCACCAATAAAA	60	GCTGGCCAAGAGAAAGTAGC	59	204	--	--	--	--	--	--
TR50985|c0_g1_i2	No	(A)10gtttgacaaaacgcgaagaacaacgctttttgtttgaacttgttcctatagcc(AG)6	75	--	--	--	--	--	--	GGAGACGCTGAACTAAACCG	60	CCTTCTCTTCCCATTGCATC	60	224	--	--	--	--	--	--
TR50985|c0_g1_i3	No	(A)10gtttgacaaaacgcgaagaacaacgctttttgtttgaacttgttcctatagcc(AG)6	75	--	--	--	--	--	--	GGAGACGCTGAACTAAACCG	60	CCTTCTCTTCCCATTGCATC	60	224	--	--	--	--	--	--
TR50986|c0_g1_i1	XLOC_020848	(ACC)5	15	--	--	--	--	--	--	ATCACCTGTCCTTGATTGGC	60	CAGCACCAGCTCCTTCTTCT	60	280	--	--	--	--	--	--
TR50986|c0_g1_i1	XLOC_020848	(TGC)5	15	--	--	--	--	--	--	CCATGGAAACGAAGGTGAAT	60	CACCCTTCTCGTGTTGGTTT	60	177	--	--	--	--	--	--
TR51002|c1_g1_i1	XLOC_020863	(GGT)7	21	--	--	--	--	--	--	TGGTTTTGATTTTGAGGTTGTG	60	AAAAGCTGTCAATCCTCTGC	57	176	--	--	--	--	--	--
TR51002|c2_g1_i1	XLOC_020864	(GA)9	18	TR51002|c2_g1_i1	(GA)9	Ca_LG_3	(GA)8	39084938	39084953	TGCAACAAGTTGTGTTGGTTT	59	TCCATGTTCACTTGCCTTCA	60	244		URR	--	--	--	--
TR51004|c0_g1_i1	No	(GAA)5	15	--	--	--	--	--	--	GAGGTTCATTTGGTTCCACAG	59	AGCAGATGCATTTCTCACCA	59	164	--	--	--	--	--	--
TR51004|c0_g2_i1	No	(GAA)5	15	--	--	--	--	--	--	GAGGTTCATTTGGTTCCACAG	59	AGCAGATGCATTTCTCACCA	59	164	--	--	--	--	--	--
TR51005|c0_g1_i1	No	(TA)6	12	--	--	--	--	--	--	AAATGCAGGAGTGGAAATGG	60	CCATTATAGAATAAGAGTTGCAGATTT	58	226	--	--	--	--	--	--
TR51005|c0_g2_i1	No	(AAT)5	15	--	--	--	--	--	--	GCTCGCAAACTCAACAACAA	60	AAACCACCATAGACGACGAC	58	121	--	--	--	--	--	--
TR51005|c0_g2_i2	No	(AAT)5	15	--	--	--	--	--	--	GCTCGCAAACTCAACAACAA	60	AAACCACCATAGACGACGAC	58	121	--	--	--	--	--	--

Transcript IDs	Differentially expressed gene (DEG) IDs	SSR repeat-motifs	SSR repeat-length (bp)	*In silico* polymorphic SSRs						Forward primers (5'-3')	Annealing tempearture(0C)	Reverse primers (5'-3')	Annealing tempearture(0C)	Amplified product size (bp)	Structural annotation		Functional Annotation			
				C. microphyllum		ICC4958									Gene IDs	Sequence component of genes	NCBI nr IDs	TFs	KOG	KEGG
				Transcript	SSR repeat-motifs	Chromosomes	SSR repeat-motifs	Start physical positions (bp)	End physical positions (bp)											
TR51005|c0_g3_i1	No	(TA)6	12	--	--	--	--	--	--	AAATGCAGGAGTGGAAATGG	60	CCATTATAGAATAAGAGTTGCAG ATTT	58	226	--	--	--	--	--	--
TR51007|c0_g1_i1	No	(CAT)5	15	--	--	--	--	--	--	TGATGTACCTGCTGGCAGAC	60	AGCTGGTTTTTGGGGTTCTT	60	272	--	--	--	--	--	--
TR51007|c0_g1_i3	No	(CAT)5	15	--	--	--	--	--	--	TGATGTACCTGCTGGCAGAC	60	AGCTGGTTTTTGGGGTTCTT	60	272	--	--	--	--	--	--
TR51019|c0_g1_i1	XLOC_020875	(AAC)5	15	--	--	--	--	--	--	ATCATGCTTCAACTCCCACC	60	ATTTGCACAACCGTGTTTGA	60	267	--	--	--	--	--	--
TR51021|c0_g1_i1	No	(ATT)7	21	--	--	--	--	--	--	ATAACCATAATTCTGCGGCG	60	GCATCGGGCTCTTATTATGC	60	153	--	--	--	--	--	--
TR51021|c0_g2_i1	No	(ATT)7	21	--	--	--	--	--	--	ATAACCATAATTCTGCGGCG	60	GCATCGGGCTCTTATTATGC	60	153	--	--	--	--	--	--
TR51028|c0_g1_i1	No	(CAT)6	18	--	--	--	--	--	--	AAACCGAAAGTCCAAACAGTG	59	GTGGCAACATGTCGTGAATC	60	129	--	--	--	--	--	--
TR51028|c0_g2_i1	No	(CAT)6	18	--	--	--	--	--	--	AAACCGAAAGTCCAAACAGTG	59	GTGGCAACATGTCGTGAATC	60	129	--	--	--	--	--	--
TR51028|c0_g3_i1	No	(CAT)6	18	--	--	--	--	--	--	AAACCGAAAGTCCAAACAGTG	59	GTGGCAACATGTCGTGAATC	60	129	--	--	--	--	--	--
TR51037|c0_g2_i1	XLOC_020891	(AAC)5	15	--	--	--	--	--	--	GCAAACATTTTGAAGAGCAGC	60	AACCCAGATAGGGTTTTGGC	60	161	--	--	--	--	--	--
TR51082|c0_g1_i1	XLOC_020907	(AAC)5	15	--	--	--	--	--	--	TGCACCTTAATCAAAAGGGC	60	CTTGGACATTTCACAGCTTCTG	60	240	--	--	XM_004485711.1	Dof	[R]	--
TR51099|c0_g1_i1	XLOC_020912	(CAGT)9	36	--	--	--	--	--	--	AACAGCATCAACCACAACCA	60	TGTGTCCACCAAAGGATTGA	60	138	--	--	XP_008614900.1	GRAS	[J]	--
TR51138|c0_g1_i1	No	(TA)6	12	--	--	--	--	--	--	TGTATGCCCATTAATCTCTCTCTC	59	TCATGGTCCTCTCTTACGCC	60	226	--	--	--	--	--	--
TR51178|c0_g1_i1	XLOC_020938	(AT)6	12	--	--	--	--	--	--	CAATTACAAGGAGGCTGTTGG	60	GAGGTTTCCAAAAGAAAATCCA	59	220	--	--	KHN21274.1	--	--	--

Transcript IDs	Differentially expressed gene (DEG) IDs	SSR repeat-motifs	SSR repeat-length (bp)	*In silico* polymorphic SSRs						Forward primers (5'-3')	Annealing tempearture(0C)	Reverse primers (5'-3')	Annealing tempearture(0C)	Amplified product size (bp)	Structural annotation		Functional Annotation			
				C. microphyllum		ICC4958									Gene IDs	Sequence component of genes	NCBI nr IDs	TFs	KOG	KEGG
				Transcript	SSR repeat-motifs	Chromosomes	SSR repeat-motifs	Start physical positions (bp)	End physical positions (bp)											
TR51340|c0_g1_i1	No	(CCT)7	21	--	--	--	--	--	--	AACTGCAACAGCCTCCATCT	60	GGTCGACGTCAGGAAACTGT	60	201	--	--	--	--	--	--
TR51353|c0_g1_i1	No	(GAAC)5	20	--	--	--	--	--	--	ACGGAGCAAACAAACGATTC	60	ATTCTCCCCTCGCTGTCTCT	60	141	--	--	--	--	--	--
TR51534|c0_g1_i1	No	(ATA)5	15	--	--	--	--	--	--	TGCAATTAAGCCAAAGCAAA	59	AGCAGAAAAGGGCAAAGTGA	60	233	--	--	--	--	--	--
TR51637|c0_g1_i2	No	(AT)6	12	--	--	--	--	--	--	ATTGATTGGTTCCTACCCCC	60	GGGCAGGCTCTATCATTTCC	61	275	--	--	--	--	--	--
TR51637|c0_g1_i3	No	(AT)6	12	--	--	--	--	--	--	ATTGATTGGTTCCTACCCCC	60	GGGCAGGCTCTATCATTTCC	61	275	--	--	--	--	--	--
TR51637|c0_g1_i4	No	(AT)6	12	--	--	--	--	--	--	ATTGATTGGTTCCTACCCCC	60	GGGCAGGCTCTATCATTTCC	61	275	--	--	--	--	--	--
TR51637|c0_g2_i1	No	(AT)6	12	--	--	--	--	--	--	ATTGATTGGTTCCTACCCCC	60	GGGCAGGCTCTATCATTTCC	61	275	--	--	--	--	--	--
TR51645|c0_g1_i2	XLOC_021077	(GTT)5	15	--	--	--	--	--	--	CGGTTACCACGATCGAAACT	60	CGAAAGCGAAACAGAGGACT	60	199	--	--	--	--	--	--
TR51647|c2_g1_i1	XLOC_021080	(AT)6	12	--	--	--	--	--	--	CCCTGCAATTAGCAAGAGGT	59	ACCTTAAATCTGAAATTGTATTAAGGA	57	164	--	--	--	--	--	--
TR51649|c0_g1_i1	No	(AAT)7	21	--	--	--	--	--	--	CAGAAAATATAATGAAAATTAGGGTGT	57	TCTCCACCAAAAGTGTGTGC	60	136	--	--	--	--	--	--
TR51649|c0_g2_i1	No	(AAT)7	21	--	--	--	--	--	--	CAGAAAATATAATGAAAATTAGGGTGT	57	TCTCCACCAAAAGTGTGTGC	60	136	--	--	--	--	--	--
TR51649|c4_g1_i1	No	(T)10g(A)10	21	--	--	--	--	--	--	CGAACATGTTTGGTTTTCCTT	59	TTTCAGCAGGCAAGTACAAAG	58	199	--	--	--	--	--	--
TR51650|c0_g1_i1	No	(GTG)5	15	--	--	--	--	--	--	ACACCCGTATCAAGCCACTC	60	ACTGGAGTCTATGGCTGCGT	60	209	--	--	--	--	--	--
TR51650|c0_g1_i1	No	(GTG)5	15	--	--	--	--	--	--	GCATGACATCTTTTGGTGCT	59	GAGTGGCTTGATACGGGTGT	60	273	--	--	--	--	--	--

<table>
<tr><th rowspan="3">Transcript IDs</th><th rowspan="3">Differentially expressed gene (DEG) IDs</th><th rowspan="3">SSR repeat-motifs</th><th rowspan="3">SSR repeat-length (bp)</th><th colspan="6">In silico polymorphic SSRs</th><th rowspan="3">Forward primers (5'-3')</th><th rowspan="3">Annealing tempearture(0C)</th><th rowspan="3">Reverse primers (5'-3')</th><th rowspan="3">Annealing tempearture(0C)</th><th rowspan="3">Amplified product size (bp)</th><th colspan="2">Structural annotation</th><th colspan="4">Functional Annotation</th></tr>
<tr><th colspan="2">C. microphyllum</th><th colspan="4">ICC4958</th><th rowspan="2">Gene IDs</th><th rowspan="2">Sequence component of genes</th><th rowspan="2">NCBI nr IDs</th><th rowspan="2">TFs</th><th rowspan="2">KOG</th><th rowspan="2">KEGG</th></tr>
<tr><th>Transcript</th><th>SSR repeat-motifs</th><th>Chromosomes</th><th>SSR repeat-motifs</th><th>Start physical positions (bp)</th><th>End physical positions (bp)</th></tr>
<tr><td>TR51650|c0_g1_i2</td><td>XLOC_021085</td><td>(GTG)5</td><td>15</td><td>--</td><td>--</td><td>--</td><td>--</td><td>--</td><td>--</td><td>ACACCCGTATCAAGCCACTC</td><td>60</td><td>ACTGGAGTCTATGGCTGCGT</td><td>60</td><td>209</td><td>--</td><td>--</td><td>ETK81791.1</td><td>--</td><td>--</td><td>--</td></tr>
<tr><td>TR51650|c1_g1_i1</td><td>XLOC_021086</td><td>(CAC)6ggacaccatgg(CCA)6</td><td>47</td><td>--</td><td>--</td><td>--</td><td>--</td><td>--</td><td>--</td><td>CATCACCACGGTCATCACTC</td><td>60</td><td>CAATTCCTCACGCTCCAACT</td><td>60</td><td>255</td><td>--</td><td>--</td><td>--</td><td>--</td><td>--</td><td>--</td></tr>
<tr><td>TR51651|c0_g1_i1</td><td>XLOC_021087</td><td>(AT)6</td><td>12</td><td>--</td><td>--</td><td>--</td><td>--</td><td>--</td><td>--</td><td>GGGTGTGTCTGGGTTTGATT</td><td>60</td><td>TTTTGTAAGGGCTTTGCCAT</td><td>60</td><td>170</td><td>--</td><td>--</td><td>--</td><td>--</td><td>--</td><td>--</td></tr>
<tr><td>TR51652|c0_g1_i1</td><td>No</td><td>(AATT)6</td><td>24</td><td>--</td><td>--</td><td>--</td><td>--</td><td>--</td><td>--</td><td>TCTTTTGACTCCACCAACCC</td><td>60</td><td>CAGGAAAGCCTCTTCCAAAA</td><td>59</td><td>254</td><td>--</td><td>--</td><td>--</td><td>--</td><td>--</td><td>--</td></tr>
<tr><td>TR51652|c0_g1_i2</td><td>No</td><td>(AGA)5</td><td>15</td><td>--</td><td>--</td><td>--</td><td>--</td><td>--</td><td>--</td><td>CAAGTGTGAGACATTTTCCTTCC</td><td>60</td><td>GTTGACCAGGGAGCTCAAAA</td><td>60</td><td>247</td><td>--</td><td>--</td><td>--</td><td>--</td><td>--</td><td>--</td></tr>
<tr><td>TR51669|c0_g2_i1</td><td>No</td><td>(AC)8</td><td>16</td><td>--</td><td>--</td><td>--</td><td>--</td><td>--</td><td>--</td><td>TGGAAATCGCAGAAAGTTGC</td><td>61</td><td>TCAGAAGGTAGCGAACCCTG</td><td>60</td><td>267</td><td>--</td><td>--</td><td>--</td><td>--</td><td>--</td><td>--</td></tr>
<tr><td>TR51669|c0_g4_i1</td><td>No</td><td>(AC)8</td><td>16</td><td>--</td><td>--</td><td>--</td><td>--</td><td>--</td><td>--</td><td>TGGAAATCGCAGAAAGTTGC</td><td>61</td><td>TCAGAAGGTAGCGAACCCTG</td><td>60</td><td>266</td><td>--</td><td>--</td><td>--</td><td>--</td><td>--</td><td>--</td></tr>
<tr><td>TR51676|c2_g1_i1</td><td>XLOC_021125</td><td>(A)13gaaacccatcaaacccatcaactcg(ATC)7</td><td>59</td><td>--</td><td>--</td><td>--</td><td>--</td><td>--</td><td>--</td><td>CTTGCTTTCTTGCTTCGCTT</td><td>60</td><td>CCTCTTTGCTCGCTGGTACT</td><td>60</td><td>124</td><td>--</td><td>--</td><td>--</td><td>--</td><td>--</td><td>--</td></tr>
<tr><td>TR51677|c0_g1_i3</td><td>No</td><td>(GT)7</td><td>14</td><td>--</td><td>--</td><td>--</td><td>--</td><td>--</td><td>--</td><td>ACAAATCAACAACCGCTTCC</td><td>60</td><td>TTGGAATGGAAAATGTGCCT</td><td>60</td><td>208</td><td>--</td><td>--</td><td>--</td><td>--</td><td>--</td><td>--</td></tr>
<tr><td>TR51677|c0_g1_i4</td><td>No</td><td>(GT)7</td><td>14</td><td>--</td><td>--</td><td>--</td><td>--</td><td>--</td><td>--</td><td>ACAAATCAACAACCGCTTCC</td><td>60</td><td>TTGGAATGGAAAATGTGCCT</td><td>60</td><td>208</td><td>--</td><td>--</td><td>--</td><td>--</td><td>--</td><td>--</td></tr>
<tr><td>TR51687|c0_g1_i10</td><td>No</td><td>(A)12gttattcaatttttctataaaatagtggtattgaa(T)10</td><td>57</td><td>--</td><td>--</td><td>--</td><td>--</td><td>--</td><td>--</td><td>TCTTTTTCACCACCAAATGC</td><td>59</td><td>AAGCGTCATTTATGTTGCAGT</td><td>57</td><td>277</td><td>--</td><td>--</td><td>--</td><td>--</td><td>--</td><td>--</td></tr>
<tr><td>TR51687|c0_g1_i5</td><td>No</td><td>(A)12gttattcaatttttctataaaatagtggtattgaa(T)10</td><td>57</td><td>--</td><td>--</td><td>--</td><td>--</td><td>--</td><td>--</td><td>TCTTTTTCACCACCAAATGC</td><td>59</td><td>AAGCGTCATTTATGTTGCAGT</td><td>57</td><td>277</td><td>--</td><td>--</td><td>--</td><td>--</td><td>--</td><td>--</td></tr>
<tr><td>TR51697|c1_g1_i1</td><td>No</td><td>(TC)6</td><td>12</td><td>--</td><td>--</td><td>--</td><td>--</td><td>--</td><td>--</td><td>CACGGCAAGTTAAACTGCAA</td><td>60</td><td>CTTCGCTGATTTTGAGGCAT</td><td>60</td><td>197</td><td>--</td><td>--</td><td>--</td><td>--</td><td>--</td><td>--</td></tr>
<tr><td>TR51718|c0_g2_i1</td><td>No</td><td>(CAC)5</td><td>15</td><td>--</td><td>--</td><td>--</td><td>--</td><td>--</td><td>--</td><td>GGTCACCACCACCACCAT</td><td>60</td><td>TCATCGATGTTCACGACGAC</td><td>61</td><td>101</td><td>--</td><td>--</td><td>--</td><td>--</td><td>--</td><td>--</td></tr>
</table>

Transcript IDs	Differentially expressed gene (DEG) IDs	SSR repeat-motifs	SSR repeat-length (bp)	*In silico* polymorphic SSRs						Forward primers (5'-3')	Annealing tempearture(0C)	Reverse primers (5'-3')	Annealing tempearture(0C)	Amplified product size (bp)	Structural annotation		Functional Annotation			
				C. microphyllum		ICC4958									Gene IDs	Sequence component of genes	NCBI nr IDs	TFs	KOG	KEGG
				Transcript	SSR repeat-motifs	Chromosomes	SSR repeat-motifs	Start physical positions (bp)	End physical positions (bp)											
TR51726|c0_g1_i1	No	(ACC)5	15	--	--	--	--	--	--	ATTACCCGTCCACGATGAAG	60	CACCTTCACCTCGAACCAAT	60	236	--	--	--	--	--	--
TR51726|c0_g2_i1	XLOC_021170	(ACC)5	15	--	--	--	--	--	--	GATCAATGCTGTTGCCAATG	60	GATTGGGTTGAATGATTGGG	60	197	--	--	--	--	--	--
TR51862|c0_g1_i1	No	(AAC)5	15	--	--	--	--	--	--	GCTAGCCGCAGAGAGAGAAA	60	TCTTCGACACACCATTTGGA	60	154	--	--	--	--	--	--
TR51862|c0_g1_i2	XLOC_021259	(AAC)5	15	--	--	--	--	--	--	GCTAGCCGCAGAGAGAGAAA	60	TCTTCGACACACCATTTGGA	60	154	--	--	--	--	--	--
TR51869|c11_g1_i1	XLOC_021266	(TTG)5	15	--	--	--	--	--	--	GGGTTTCCATCTTCAAAGCA	60	AGCACACCAGCTACCCAAAC	60	244	--	--	--	--	--	--
TR51869|c4_g1_i3	XLOC_021270	(TC)8	16	--	--	--	--	--	--	GCAAAATGACGGTGAGTGAA	60	CGCCGTTGTTTTTAAGGAAG	60	146	--	--	--	--	--	--
TR51871|c0_g1_i2	No	(TC)9	18	--	--	--	--	--	--	AGCCCAACAAGAAGGAACAC	59	AACGAAAGGGGTGAACAGTG	60	104	--	--	--	--	--	--
TR51871|c0_g1_i4	No	(TC)9	18	--	--	--	--	--	--	AGCCCAACAAGAAGGAACAC	59	AACGAAAGGGGTGAACAGTG	60	104	--	--	--	--	--	--
TR51872|c0_g2_i1	XLOC_021276	(GTTA)5	20	--	--	--	--	--	--	CGTTTTTGCCATGATCCAAT	61	GACACTGCTGGTCAAGAACG	59	277	--	--	--	--	--	--
TR51888|c1_g1_i1	No	(AT)7	14	TR51888|c1_g1_i1	(AT)7	Ca_LG_4	(AT)6	30369246	30369257	ATGCGCTAGGATTGGAATTG	60	TTCATACCCCAAGCAGATCC	60	280		URR	--	--	--	--
TR51888|c1_g1_i2	No	(AT)7	14	--	--	--	--	--	--	ATGCGCTAGGATTGGAATTG	60	TTCATACCCCAAGCAGATCC	60	280	--	--	--	--	--	--
TR51888|c1_g1_i3	No	(AT)7	14	--	--	--	--	--	--	ATGCGCTAGGATTGGAATTG	60	TTCATACCCCAAGCAGATCC	60	280	--	--	--	--	--	--
TR51890|c0_g2_i1	XLOC_021317	(TTC)12	36	--	--	--	--	--	--	CGTCAGCGTATGACAGAAACA	60	GAGAGCGTCTTTGGAAGTGG	60	263	--	--	--	--	--	--
TR51901|c0_g1_i1	XLOC_021328	(AG)9	18	--	--	--	--	--	--	GGAACCAACCCAAGAGACAA	60	TTGGACAAGCTCAGGGATTC	60	269	--	--	--	--	--	--

Transcript IDs	Differentially expressed gene (DEG) IDs	SSR repeat-motifs	SSR repeat-length (bp)	*In silico* polymorphic SSRs						Forward primers (5'-3')	Annealing tempearture(0C)	Reverse primers (5'-3')	Annealing tempearture(0C)	Amplified product size (bp)	Structural annotation		Functional Annotation			
				C. microphyllum		ICC4958														
				Transcript	SSR repeat-motifs	Chromosomes	SSR repeat-motifs	Start physical positions (bp)	End physical positions (bp)						Gene IDs	Sequence component of genes	NCBI nr IDs	TFs	KOG	KEGG
TR51901|c0_g2_i1	No	(AG)9	18	--	--	--	--	--	--	GGAACCAACCCAAGAGACAA	60	TTGGACAAGCTCAGGGATTC	60	270	--	--	--	--	--	--
TR51901|c2_g1_i1	XLOC_021330	(TA)7	14	--	--	--	--	--	--	CCTTACTCTTTCCCATCCCA	59	TGAAGAAGATGATGCAGCCA	61	266	--	--	--	--	--	--
TR51901|c4_g4_i1	XLOC_021334	(TTG)5	15	--	--	--	--	--	--	TGAGTTTGTGGTGGAACCAA	60	CATAAGATGGTGAAGGGTTTGA	59	170	--	--	--	--	--	--
TR51901|c6_g3_i2	XLOC_021337	(TCA)5	15	--	--	--	--	--	--	GTGGCACTCTCTTCTCCAGG	60	ACTCATGTGGTTTGTGGCAA	60	152	--	--	--	--	--	--
TR51901|c7_g1_i1	XLOC_021339	(TA)7	14	--	--	--	--	--	--	ATCAACCTGGAACACCAAGC	60	ATTGCATGCACTCCAATCAT	59	224	--	--	--	--	--	--
TR51909|c0_g2_i2	No	(GAT)6	18	--	--	--	--	--	--	TGTTGCTGAAGAGGCTGTTG	60	ATCATCCCATGGCTTCACAT	60	214	--	--	--	--	--	--
TR51909|c0_g2_i4	No	(GAT)6	18	--	--	--	--	--	--	TGTTGCTGAAGAGGCTGTTG	60	ATCATCCCATGGCTTCACAT	60	214	--	--	--	--	--	--
TR51913|c5_g3_i1	No	(TTC)6	18	TR51913|c5_g3_i1	(TTC)6	scaffold05908	(TTC)5	12519	12533	GTCACACTTCTCTCGCCTCC	60	TGAAGGAACAACGGGAAATC	60	157	Ca_22840	URR	--	--	--	--
TR51913|c5_g3_i3	No	(TTC)6	18	--	--	--	--	--	--	GTCACACTTCTCTCGCCTCC	60	TGAAGGAACAACGGGAAATC	60	157	--	--	--	--	--	--
TR51914|c1_g1_i1	XLOC_021356	(TC)8	16	--	--	--	--	--	--	TGAGCTTTTCACTCTCCGGT	60	ATGGAAGCCATAACGGTGTC	60	137	--	--	--	--	--	--
TR51918|c1_g1_i2	No	(T)10ctcattaattagcagcccttttttcat(A)11	47	--	--	--	--	--	--	TGAACAACCTATGGCCCAAT	60	TGGGTGGTGTAAAAGACATGG	61	206	--	--	--	--	--	--
TR51918|c1_g1_i6	No	(T)10ctcattaattagcagcccttttttcat(A)11	47	--	--	--	--	--	--	TGAACAACCTATGGCCCAAT	60	TGGGTGGTGTAAAAGACATGG	61	206	--	--	--	--	--	--
TR51920|c0_g2_i1	XLOC_021367	(GA)9	18	--	--	--	--	--	--	GGAAACAAAAAGAGAGCGTGA	59	ACGAATTTTCTTAAGCGCGA	60	273	--	--	--	--	--	--
TR51922|c2_g1_i1	No	(TC)6	12	--	--	--	--	--	--	GGTTCCTCACAAGATGTCCC	59	GAAGGAGGTTTCGTATGGCA	60	146	--	--	--	--	--	--

Transcript IDs	Differentially expressed gene (DEG) IDs	SSR repeat-motifs	SSR repeat-length (bp)	*In silico* polymorphic SSRs						Forward primers (5'-3')	Annealing tempearture(0C)	Reverse primers (5'-3')	Annealing tempearture(0C)	Amplified product size (bp)	Structural annotation		Functional Annotation			
				C. microphyllum		ICC4958									Gene IDs	Sequence component of genes	NCBI nr IDs	TFs	KOG	KEGG
				Transcript	SSR repeat-motifs	Chromosomes	SSR repeat-motifs	Start physical positions (bp)	End physical positions (bp)											
TR51922|c2_g1_i3	No	(TC)6	12	--	--	--	--	--	--	GCTCAACCGGTAACCCTTCT	60	CTGGTCGTTGCCATAACAGA	60	231	--	--	--	--	--	--
TR51922|c2_g1_i3	No	(TC)6	12	--	--	--	--	--	--	GGTTCCTCACAAGATGTCCC	59	GAAGGAGGTTTCGTATGGCA	60	146	--	--	--	--	--	--
TR51922|c2_g1_i4	No	(TC)6	12	--	--	--	--	--	--	GGTTCCTCACAAGATGTCCC	59	GAAGGAGGTTTCGTATGGCA	60	146	--	--	--	--	--	--
TR51936|c2_g2_i1	XLOC_021398	(TTA)5	15	--	--	--	--	--	--	TTGCAACGTTTTCTTGTTGC	60	ACAGTTGGGACAGAAATGGC	60	227	--	--	--	--	--	--
TR51940|c1_g1_i1	XLOC_021410	(GTA)5	15	--	--	--	--	--	--	ATGGTCACACTCACACCCAA	60	CTCTCTTGTCTCCTCCCACG	60	237	--	--	--	--	--	--
TR51943|c0_g1_i2	No	(CTT)6	18	--	--	--	--	--	--	CCCGAAAACCAAAGACAAAA	60	AAAGCTCCGAGTCCTTCTCC	60	210	--	--	--	--	--	--
TR51943|c0_g1_i3	No	(CTT)6	18	--	--	--	--	--	--	CCCGAAAACCAAAGACAAAA	60	AAAGCTCCGAGTCCTTCTCC	60	210	--	--	--	--	--	--
TR51944|c0_g1_i2	No	(GCA)5	15	--	--	--	--	--	--	TTCATTGCCAGCAAGACATC	60	ACCAAGGAACTCTCCACCCT	60	268	--	--	--	--	--	--
TR51944|c0_g1_i3	No	(GCA)5	15	--	--	--	--	--	--	TTCATTGCCAGCAAGACATC	60	ACCAAGGAACTCTCCACCCT	60	268	--	--	--	--	--	--
TR51944|c0_g1_i5	XLOC_021417	(GCA)5	15	--	--	--	--	--	--	TTCATTGCCAGCAAGACATC	60	ACCAAGGAACTCTCCACCCT	60	268	--	--	KEP54532.1	--	[J]	K02936
TR51945|c1_g1_i10	No	(CCT)6	18	--	--	--	--	--	--	GTACCAGTCTCCTCCCCCTC	60	TCTTCTTCATTCGTCTGGGC	60	258	--	--	--	--	--	--
TR51945|c1_g1_i11	No	(CCT)6	18	--	--	--	--	--	--	GTACCAGTCTCCTCCCCCTC	60	TCTTCTTCATTCGTCTGGGC	60	258	--	--	--	--	--	--
TR51945|c1_g1_i2	No	(CCT)6	18	--	--	--	--	--	--	GTACCAGTCTCCTCCCCCTC	60	TCTTCTTCATTCGTCTGGGC	60	258	--	--	--	--	--	--
TR51945|c1_g1_i3	No	(CCT)6	18	--	--	--	--	--	--	GTACCAGTCTCCTCCCCCTC	60	TCTTCTTCATTCGTCTGGGC	60	258	--	--	--	--	--	--

Transcript IDs	Differentially expressed gene (DEG) IDs	SSR repeat-motifs	SSR repeat-length (bp)	*In silico* polymorphic SSRs						Forward primers (5'-3')	Annealing tempearture(0C)	Reverse primers (5'-3')	Annealing tempearture(0C)	Amplified product size (bp)	Structural annotation		Functional Annotation			
				C. microphyllum		ICC4958									Gene IDs	Sequence component of genes	NCBI nr IDs	TFs	KOG	KEGG
				Transcript	SSR repeat-motifs	Chromosomes	SSR repeat-motifs	Start physical positions (bp)	End physical positions (bp)											
TR51945|c1_g1_i4	No	(CCT)6	18	--	--	--	--	--	--	GTACCAGTCTCCTCCCCCTC	60	TCTTCTTCATTCGTCTGGGC	60	258	--	--	--	--	--	--
TR51945|c1_g1_i5	No	(CCT)6	18	--	--	--	--	--	--	GTACCAGTCTCCTCCCCCTC	60	TCTTCTTCATTCGTCTGGGC	60	258	--	--	--	--	--	--
TR51945|c1_g1_i6	No	(CCT)6	18	--	--	--	--	--	--	GTACCAGTCTCCTCCCCCTC	60	TCTTCTTCATTCGTCTGGGC	60	258	--	--	--	--	--	--
TR51945|c1_g1_i7	No	(CCT)6	18	--	--	--	--	--	--	GTACCAGTCTCCTCCCCCTC	60	TCTTCTTCATTCGTCTGGGC	60	258	--	--	--	--	--	--
TR51945|c1_g1_i9	No	(CCT)6	18	--	--	--	--	--	--	GTACCAGTCTCCTCCCCCTC	60	TCTTCTTCATTCGTCTGGGC	60	258	--	--	--	--	--	--
TR51946|c0_g1_i1	XLOC_021420	(CCA)5	15	TR51946|c0_g1_i1	(CCA)5	Ca_LG_2	(CCA)7	11228079	11228099	CCACCCACACTCACTGTCAC	60	TCGGAGTCCATCATTGTTGA	60	210		URR	--	--	--	--
TR51964|c0_g2_i1	No	(ATT)5	15	--	--	--	--	--	--	GCTTGTTCAGTGCAAAGTGG	59	CACATTTCAACAACCAAATCACA	61	222	--	--	--	--	--	--
TR51986|c0_g1_i1	XLOC_021460	(CTT)5	15	--	--	--	--	--	--	GCATTTGCACCATCCTCTCT	60	ACGGTGAGGAACACGAAGAT	60	159	--	--	--	--	--	--
TR520|c0_g1_i1	XLOC_021499	(GAT)5	15	--	--	--	--	--	--	ACGATGCTTCAGACGAGGAA	61	TGGACCTCGTTTCCTACCAC	60	266	--	--	--	--	--	--
TR520|c0_g1_i1	XLOC_021499	(TGG)6	18	--	--	--	--	--	--	GTGGTAGGAAACGAGGTCCA	60	CAGGCCCAATCTATCCTCAA	60	192	--	--	--	--	--	--
TR52105|c0_g1_i1	XLOC_021506	(AG)6tttttgttgctgatggcaagtggtctgcttcgcgccttcaatatctcactgta(T)11	76	--	--	--	--	--	--	GGGTTTTGTTGCATCTCACC	60	AGCTCACACTCGCGTCTTTT	60	251	--	--	--	--	--	--
TR52112|c0_g1_i1	XLOC_021513	(ACA)6	18	TR52112|c0_g1_i1	(ACA)6	Ca_LG_2	(ACA)7	22982361	22982381	GGGAAAGAGTTCAAAACCCA	59	CAGGAACCAAGATTGCAAGA	59	189		URR	--	--	--	--
TR52114|c0_g3_i1	XLOC_021515	(TGC)5	15	--	--	--	--	--	--	CTGCTGCATCTGTTGCTGTT	60	ATGAACCAGCTACTCCCCCT	60	236	--	--	--	--	--	--
TR52135|c0_g1_i1	XLOC_021532	(GAT)5	15	--	--	--	--	--	--	CATGGCAGAAGCAGAAAACA	60	TAACCAGGCCGCTTATATGG	60	190	--	--	CCO30545.1	--	[E]	--

Transcript IDs	Differentially expressed gene (DEG) IDs	SSR repeat-motifs	SSR repeat-length (bp)	In silico polymorphic SSRs: *C. microphyllum* Transcript	In silico polymorphic SSRs: *C. microphyllum* SSR repeat-motifs	In silico polymorphic SSRs: ICC4958 Chromosomes	In silico polymorphic SSRs: ICC4958 SSR repeat-motifs	In silico polymorphic SSRs: ICC4958 Start physical positions (bp)	In silico polymorphic SSRs: ICC4958 End physical positions (bp)	Forward primers (5'-3')	Annealing tempearture(0C)	Reverse primers (5'-3')	Annealing tempearture(0C)	Amplified product size (bp)	Structural annotation: Gene IDs	Structural annotation: Sequence component of genes	Functional Annotation: NCBI nr IDs	Functional Annotation: TFs	Functional Annotation: KOG	Functional Annotation: KEGG
TR52155|c0_g1_i1	No	(TGA)5	15	--	--	--	--	--	--	TTTGCAGATTGGTGATTCCA	60	TTTCTTTGGAGGGAGAGGGT	60	143	--	--	--	--	--	--
TR52155|c0_g2_i1	No	(TGA)5	15	--	--	--	--	--	--	TTTGCAGATTGGTGATTCCA	60	TTTCTTTGGAGGGAGAGGGT	60	143	--	--	--	--	--	--
TR52188|c3_g1_i1	XLOC_021572	(ATT)7	21	--	--	--	--	--	--	ACAAACAGAACCCAGATGCC	60	CTCTGCAACTGCCAAACAAA	60	234	--	--	--	--	--	--
TR52212|c0_g2_i1	No	(AGG)5	15	TR52212|c0_g2_i1	(AGG)5	Ca_LG_3	(AGG)6	24363271	24363288	GGACCTCATCAAACCCAGAA	60	GCTTTGACAAATCCTTCCCA	60	228		URR	--	--	--	--
TR52212|c0_g2_i2	No	(AGG)5	15	--	--	--	--	--	--	GGACCTCATCAAACCCAGAA	60	GCTTTGACAAATCCTTCCCA	60	228	--	--	--	--	--	--
TR52212|c0_g2_i3	No	(AGG)5	15	--	--	--	--	--	--	GGACCTCATCAAACCCAGAA	60	GCTTTGACAAATCCTTCCCA	60	228	--	--	--	--	--	--
TR52221|c0_g1_i1	XLOC_021606	(TCT)5	15	--	--	--	--	--	--	AATCTTTCTCCACCGACACG	60	CCAGATGCGATCTGATTGAG	59	188	--	--	--	--	--	--
TR52235|c0_g1_i1	No	(CTC)7	21	--	--	--	--	--	--	TAGTTGGCAGCTGCATCAAG	60	GAACAGCACTACAGCAACGC	60	271	--	--	--	--	--	--
TR52235|c0_g1_i2	No	(CTC)7	21	--	--	--	--	--	--	TAGTTGGCAGCTGCATCAAG	60	GAACAGCACTACAGCAACGC	60	271	--	--	--	--	--	--
TR52235|c0_g1_i3	No	(CTC)7	21	--	--	--	--	--	--	TAGTTGGCAGCTGCATCAAG	60	GAACAGCACTACAGCAACGC	60	271	--	--	--	--	--	--
TR52235|c0_g1_i4	No	(CTC)7	21	--	--	--	--	--	--	TAGTTGGCAGCTGCATCAAG	60	GAACAGCACTACAGCAACGC	60	271	--	--	--	--	--	--
TR52235|c0_g1_i5	No	(CTC)7	21	--	--	--	--	--	--	TAGTTGGCAGCTGCATCAAG	60	GAACAGCACTACAGCAACGC	60	271	--	--	--	--	--	--
TR52246|c0_g1_i1	No	(TTA)5	15	--	--	--	--	--	--	GTGGGCCCATCTCATGTACT	60	GAGATTGAAGGTGGTGAGGG	60	119	--	--	--	--	--	--
TR52246|c0_g1_i2	No	(TTA)5	15	--	--	--	--	--	--	CCCCACCAACAAGACTGTTT	60	CCAAACAAACACCCCACTTC	60	211	--	--	--	--	--	--

Transcript IDs	Differentially expressed gene (DEG) IDs	SSR repeat-motifs	SSR repeat-length (bp)	*In silico* polymorphic SSRs						Forward primers (5'-3')	Annealing tempearture(0C)	Reverse primers (5'-3')	Annealing tempearture(0C)	Amplified product size (bp)	Structural annotation		Functional Annotation			
				C. microphyllum		ICC4958														
				Transcript	SSR repeat-motifs	Chromosomes	SSR repeat-motifs	Start physical positions (bp)	End physical positions (bp)						Gene IDs	Sequence component of genes	NCBI nr IDs	TFs	KOG	KEGG
TR52246|c0_g1_i3	No	(TTA)5	15	--	--	--	--	--	--	GTGGGCCCATCTCATGTACT	60	GAGATTGAAGGTGGTGAGGG	60	119	--	--	--	--	--	--
TR52247|c0_g1_i1	XLOC_021636	(TTG)5	15	--	--	--	--	--	--	TGATTGTTGTGGTGATGGAA	58	ATGGAGATGGAAATGAAGCG	60	162	--	--	--	--	--	--
TR5225|c0_g1_i1	XLOC_021653	(AG)6aatagtgagagagataggggaggggataagagggatagagaaatttgtccact(A)15	80	--	--	--	--	--	--	CAGCGGTGACAGAGTAAGACA	59	CGGACATGTCTATGTTCCGA	60	276	--	--	XP_001943200.2	--	[I]	K10249
TR52251|c1_g2_i1	XLOC_021642	(AAG)5	15	--	--	--	--	--	--	AAATAGGTACTAGAAGAAGAAACAGCA	58	GTTTTTGTTTCGGAAAACGC	60	181	--	--	--	--	--	--
TR52254|c1_g1_i1	No	(TC)8	16	--	--	--	--	--	--	TAGGGAACCGTCACTCCAAC	60	TTTGGGGTTGTTCTTTGAGG	60	273	--	--	--	--	--	--
TR52254|c1_g2_i1	No	(TC)8	16	--	--	--	--	--	--	TAGGGAACCGTCACTCCAAC	60	TTTGGGGTTGTTCTTTGAGG	60	272	--	--	--	--	--	--
TR52259|c1_g2_i1	XLOC_021651	(CT)10	20	--	--	--	--	--	--	TGCAAATCGAAACCAAATGA	60	TTTTGCAAGTCTGCGAAATG	60	160	--	--	--	--	--	--
TR52259|c2_g1_i1	XLOC_021652	(TC)10	20	--	--	--	--	--	--	CATATGGACAACCCAAAGGG	60	GAAAACAAAAGGCTGTTCGC	60	195	--	--	CCO27020.1	--	--	--
TR52265|c0_g1_i1	XLOC_021663	(TC)10tattgggagtttgtgacacaacaaaacacctgatccatccgtgaaccactttactaaaaaccattcattctactct(TC)8	112	TR52265|c0_g1_i1	(AG)8	Ca_LG_3	(AG)6	35864358	35864369	CACTCTCACTCTCAGTCCCTCA	60	TTTTGCTTCTGCTTCCATCA	60	271		URR	--	--	--	--
TR52265|c0_g1_i1	XLOC_021663	(AG)8	16	TR52265|c0_g1_i1	(AG)8	Ca_LG_3	(AG)6	35864358	35864369	TTGGGAGAAGAACAGGGTTG	60	CAACAAGCACCTAATTTCCATC	59	112		URR	--	--	--	--
TR52267|c0_g1_i2	No	(T)11a(T)11	23	--	--	--	--	--	--	CAAATATATCAAACCTTTGTTGACC	58	GATGCTGTGCACTTGGAAGA	60	258	--	--	--	--	--	--
TR52289|c0_g1_i1	XLOC_021686	(A)17gaaaaatcaattttaatttttcaccttattatcacttgttcaaattcatcaattcca(T)10	85	--	--	--	--	--	--	TGCTTCTTCCTTCTAAAAACCC	59	AACAAGCTCTGATGCGCTTT	60	149	--	--	--	--	--	--
TR52366|c0_g1_i1	No	(ACA)5	15	--	--	--	--	--	--	CCTCCATTCTCCAATTCCAA	60	CCAGTCTCGCTCTCTGCTTT	60	280	--	--	--	--	--	--
TR52366|c0_g1_i2	No	(ACA)5	15	--	--	--	--	--	--	CCTCCATTCTCCAATTCCAA	60	CCAGTCTCGCTCTCTGCTTT	60	280	--	--	--	--	--	--

Transcript IDs	Differentially expressed gene (DEG) IDs	SSR repeat-motifs	SSR repeat-length (bp)	*In silico* polymorphic SSRs						Forward primers (5'-3')	Annealing tempearture(0C)	Reverse primers (5'-3')	Annealing tempearture(0C)	Amplified product size (bp)	Structural annotation		Functional Annotation			
				C. microphyllum		ICC4958									Gene IDs	Sequence component of genes	NCBI nr IDs	TFs	KOG	KEGG
				Transcript	SSR repeat-motifs	Chromosomes	SSR repeat-motifs	Start physical positions (bp)	End physical positions (bp)											
TR52366|c0_g1_i3	No	(ACA)5	15	--	--	--	--	--	--	CCTCCATTCTCCAATTCCAA	60	CCAGTCTCGCTCTCTGCTTT	60	280	--	--	--	--	--	--
TR52366|c0_g1_i4	No	(ACA)5	15	--	--	--	--	--	--	CCTCCATTCTCCAATTCCAA	60	CCAGTCTCGCTCTCTGCTTT	60	280	--	--	--	--	--	--
TR52378|c0_g1_i1	No	(TC)8	16	--	--	--	--	--	--	TAAATCAACGTGCATCGGAA	60	GGATTAATGTCGCGAAAAGC	60	258	--	--	--	--	--	--
TR52383|c0_g2_i1	XLOC_021744	(TG)6	12	--	--	--	--	--	--	CCTCAACAAACCAAAGTTTTGAA	60	CAAGAGGCCTTGATGGAACT	59	198	--	--	--	--	--	--
TR52394|c1_g2_i1	XLOC_021757	(CTT)5	15	--	--	--	--	--	--	GGGTAGTATGTGCCAGCCTC	60	GGTGAAACTTGTTGCACATGA	60	163	--	--	--	--	--	--
TR52426|c1_g1_i1	XLOC_021792	(GAG)8	24	--	--	--	--	--	--	TACCAGTGAACCTGCTGCTG	60	GGTGCTCGACGACCAATTAT	60	104	--	--	XP_004494888.1	G2-like	[R]	--
TR52428|c0_g1_i2	XLOC_021794	(TA)6	12	--	--	--	--	--	--	GCATAATGTCTCGCTCTAAACC	58	CTTAGGTAGCTCGCGTTTGG	60	270	--	--	--	--	--	--
TR52440|c0_g1_i2	No	(AT)6	12	--	--	--	--	--	--	GAAACAAAGCTCCTAAACAGTGA	58	CACAGGATGGTATGCCAATTT	60	253	--	--	--	--	--	--
TR52443|c0_g1_i1	No	(TTG)5	15	--	--	--	--	--	--	TCATTCCCCCTCAATCAAAA	60	AAATCGTGATGGTGGTGGTT	60	159	--	--	--	--	--	--
TR52443|c0_g2_i1	XLOC_021811	(TTG)5	15	--	--	--	--	--	--	TCATTCCCCCTCAATCAAAA	60	AAATCGTGATGGTGGTGGTT	60	159	--	--	GAO36092.1	--	[X]	--
TR52443|c0_g3_i1	No	(TTG)5	15	--	--	--	--	--	--	TCATTCCCCCTCAATCAAAA	60	AAATCGTGATGGTGGTGGTT	60	159	--	--	--	--	--	--
TR52453|c0_g1_i1	XLOC_021822	(CGC)5	15	--	--	--	--	--	--	TCAATTTGTGGCAAGAGCTG	60	CCTCCAAACCAAATCCTTGA	60	176	--	--	--	--	--	--
TR52453|c0_g2_i1	XLOC_021823	(AT)10	20	--	--	--	--	--	--	AGCTCCTTGCCATGATTGTT	60	CAAAACCGCAATATTAAGTATATCACA	60	112	--	--	--	--	--	--
TR52453|c0_g2_i1	XLOC_021823	(CGC)5	15	--	--	--	--	--	--	TCAATTTGTGGCAAGAGCTG	60	CCTCCAAACCAAATCCTTGA	60	176	--	--	--	--	--	--

Transcript IDs	Differentially expressed gene (DEG) IDs	SSR repeat-motifs	SSR repeat-length (bp)	*In silico* polymorphic SSRs						Forward primers (5'-3')	Annealing tempearture(0C)	Reverse primers (5'-3')	Annealing tempearture(0C)	Amplified product size (bp)	Structural annotation		Functional Annotation			
				C. microphyllum		ICC4958									Gene IDs	Sequence component of genes	NCBI nr IDs	TFs	KOG	KEGG
				Transcript	SSR repeat-motifs	Chromosomes	SSR repeat-motifs	Start physical positions (bp)	End physical positions (bp)											
TR52497|c0_g2_i1	No	(TC)8	16	TR52497|c0_g2_i1	(TC)8	Ca_LG_6	(TC)10	47134043	47134062	GCTCAAACTTTCCAACCCTC	59	TGCCACAAAACTCTGTCCCT	61	231	Ca_12375	CDS	--	--	--	--
TR5250|c1_g1_i1	No	(GTG)5	15	--	--	--	--	--	--	CGCAGGTAATGGAGGAGAAG	60	AAACGATTCAACTAACGCCG	60	172	--	--	--	--	--	--
TR52528|c0_g1_i1	XLOC_021872	(A)10taaacattaattaaaagatctcgccgccgtgttgcctccaccgtggtcggagaaataaacacattacaaactctccgatcctaaaatcatttcagaa(TC)7	121	--	--	--	--	--	--	AAAGTTTAAAGATCTCGTTGTCAGC	59	GAAAATCGGTCAACGGAAAA	60	274	--	--	--	--	--	--
TR52546|c0_g1_i1	No	(CAG)6caccagcaccagcaccaacagtac(CAG)6	60	--	--	--	--	--	--	TCGTTGTGCCTATCATCCAA	60	TGGTACTGTTGGTGCTGGTG	61	146	--	--	--	--	--	--
TR52577|c0_g1_i1	XLOC_021889	(TCA)5	15	--	--	--	--	--	--	GCTTTCAGTGGGAACGAGAG	60	ATGGAGGCAGTAATGGCTTG	60	195	--	--	KDP30176.1	TALE	[K]	--
TR52612|c0_g1_i1	XLOC_021901	(CCG)5	15	--	--	--	--	--	--	TGCTTCAACAGTGCTTGGAC	60	GCCTCTGAGTCCTGGTGGTA	60	229	--	--	--	--	--	--
TR52651|c0_g1_i1	No	(ATC)5	15	--	--	--	--	--	--	GTGGTCAAGACTCTGGGGAA	60	TGCGTGATTACGGAATTTGT	59	131	--	--	--	--	--	--
TR52691|c0_g1_i1	No	(AAC)6	18	--	--	--	--	--	--	TCATCACCATCTGATTCTCCT	57	TCCACTTCCTCTTTCAGTCCA	60	115	--	--	--	--	--	--
TR52737|c0_g1_i1	XLOC_021933	(TC)7	14	--	--	--	--	--	--	GTGTATCTCTCTCCGCCACC	60	TTGCCAAAAACATGGCAGTA	60	128	--	--	--	--	--	--
TR52737|c0_g2_i1	XLOC_021934	(TC)7	14	--	--	--	--	--	--	GTGTATCTCTCTCCGCCACC	60	CCAAAAACATGGCTGCAGTA	60	128	--	--	--	--	--	--
TR52765|c0_g1_i1	XLOC_021941	(CT)8	16	--	--	--	--	--	--	AGTGGCTCCCTTCCTTTCAT	60	GTGCGTGTTGTGTTGGATTC	60	135	--	--	--	--	--	--
TR52787|c0_g1_i1	XLOC_021947	(CT)8	16	--	--	--	--	--	--	CCTGTAAAACCCCGTATCCC	60	GGTGTTTTCAGATTCGGCAT	60	126	--	--	--	--	--	--
TR528|c0_g1_i1	No	(AGT)5	15	--	--	--	--	--	--	TGCGTATCCAGCAGTTGAAG	60	CGGATATGCGTCTACACCCT	60	208	--	--	--	--	--	--
TR52825|c0_g1_i1	XLOC_021956	(GAA)7	21	--	--	--	--	--	--	GCTCAAATCAAAAGAGAAAGAGAA	58	ATGGCATAACAGCACCAACA	60	145	--	--	--	--	--	--

Transcript IDs	Differentially expressed gene (DEG) IDs	SSR repeat-motifs	SSR repeat-length (bp)	*In silico* polymorphic SSRs						Forward primers (5'-3')	Annealing tempearture(0C)	Reverse primers (5'-3')	Annealing tempearture(0C)	Amplified product size (bp)	Structural annotation		Functional Annotation			
				C. microphyllum		ICC4958									Gene IDs	Sequence component of genes	NCBI nr IDs	TFs	KOG	KEGG
				Transcript	SSR repeat-motifs	Chromosomes	SSR repeat-motifs	Start physical positions (bp)	End physical positions (bp)											
TR529|c1_g1_i1	XLOC_022000	(T)15ccgagatcaaggttttgttttttaattttatatcatccttttgtagattatgctcctgtggct(TA)6	90	--	--	--	--	--	--	CATGAAATGGCTCGGTTTTT	60	TGGCAGAAACAGAACACCAA	60	242	--	--	XP_004508985.1	--	[S]	--
TR529|c1_g1_i1	XLOC_022000	(A)10ttaaaaattgcgacaaaaaaaaagaaag(A)11	48	--	--	--	--	--	--	TTGCTGCATAGTTGGTTTGG	60	CAAAAACCGAGCCATTTCAT	60	213	--	--	XP_004508985.1	--	[S]	--
TR53046|c0_g1_i1	No	(GGT)5	15	--	--	--	--	--	--	GGTGGTGGAGGTGCTACACT	60	CTTTGATGTCTAAGGCCCCA	60	229	--	--	--	--	--	--
TR53046|c0_g1_i2	No	(GGT)5	15	--	--	--	--	--	--	ATGGAGGAAGAGGAGGTGGT	60	CTTTGATGTCTAAGGCCCCA	60	171	--	--	--	--	--	--
TR53052|c0_g1_i1	XLOC_022020	(AAG)5	15	--	--	--	--	--	--	GGAGTTGCTCAAAGCCTACG	60	GCACTTTCTGCTCGATTTCC	60	128	--	--	KKA76157.1	--	[K]	--
TR53060|c0_g1_i1	No	(CAT)6	18	--	--	--	--	--	--	CTGGTGTCATACATGCAGGC	60	TTCCATCCATTCTTTGCCTC	60	260	--	--	--	--	--	--
TR53060|c0_g2_i1	No	(CAT)6	18	--	--	--	--	--	--	CTGGTGTCATACATGCAGGC	60	TTCCATCCATTCTTTGCCTC	60	260	--	--	--	--	--	--
TR53060|c0_g3_i1	No	(CAT)6	18	--	--	--	--	--	--	CTGGTGTCATACATGCAGGC	60	TTCCATCCATTCTTTGCCTC	60	260	--	--	--	--	--	--
TR53060|c0_g4_i1	No	(CAT)6	18	--	--	--	--	--	--	CTGGTGTCATACATGCAGGC	60	TTCCATCCATTCTTTGCCTC	60	260	--	--	--	--	--	--
TR53063|c0_g1_i1	XLOC_022028	(ACG)5	15	--	--	--	--	--	--	GGAGAAGAATACCGCTGCTG	60	ACAGCGGTAATGGTACCGAG	60	189	--	--	XP_009519584.1	--	[P]	--
TR53074|c0_g1_i1	XLOC_022038	(CT)7	14	--	--	--	--	--	--	TAACGTATGAATCCGCCACA	60	TCCTGATTCGGGTCAAGTTC	60	136	--	--	--	--	--	--
TR53076|c0_g1_i1	No	(TGC)6	18	--	--	--	--	--	--	TCGTCATCCCAAGGCTTAAC	60	GGGAATGCTCAAGGTGTGAA	61	239	--	--	--	--	--	--
TR53107|c0_g2_i1	XLOC_022069	(CT)19	38	--	--	--	--	--	--	CTGCGTGCATTCACAGACTC	61	TTGTTGTAGTTGTCGCTGCC	60	129	--	--	--	--	--	--
TR53265|c0_g1_i1	No	(AC)6	12	--	--	--	--	--	--	CCATTCTGCATGGAACACTG	60	CCATTGACTCATGGTCATATACTAA	57	226	--	--	--	--	--	--

Transcript IDs	Differentially expressed gene (DEG) IDs	SSR repeat-motifs	SSR repeat-length (bp)	*In silico* polymorphic SSRs						Forward primers (5'-3')	Annealing tempearture(0C)	Reverse primers (5'-3')	Annealing tempearture(0C)	Amplified product size (bp)	Structural annotation		Functional Annotation			
				C. microphyllum		ICC4958									Gene IDs	Sequence component of genes	NCBI nr IDs	TFs	KOG	KEGG
				Transcript	SSR repeat-motifs	Chromosomes	SSR repeat-motifs	Start physical positions (bp)	End physical positions (bp)											
TR533|c0_g1_i1	No	(AAT)6	18	--	--	--	--	--	--	AAACTGGCCAAACCCAATTA	59	CAATCATTCCCCAAAGTTGC	60	275	--	--	--	--	--	--
TR533|c0_g1_i2	No	(AAT)6	18	--	--	--	--	--	--	AAACTGGCCAAACCCAATTA	59	CAATCATTCCCCAAAGTTGC	60	275	--	--	--	--	--	--
TR533|c0_g1_i3	No	(AAT)6	18	--	--	--	--	--	--	AAACTGGCCAAACCCAATTA	59	CAATCATTCCCCAAAGTTGC	60	275	--	--	--	--	--	--
TR533|c0_g1_i4	No	(AAT)6	18	--	--	--	--	--	--	AAACTGGCCAAACCCAATTA	59	CAATCATTCCCCAAAGTTGC	60	275	--	--	--	--	--	--
TR53319|c0_g1_i1	XLOC_022135	(TG)6	12	--	--	--	--	--	--	TTGTGGCTTTCTTTTTGGGT	60	TCAGCACAACATGAACCTCC	60	144	--	--	--	--	--	--
TR53349|c0_g1_i1	XLOC_022149	(ACA)5	15	--	--	--	--	--	--	TGATGTGGATGATGAAAAGGA	59	TGGCCATATACATCCACCAA	60	161	--	--	XM_004503107.1	C2H2	[R]	--
TR53370|c0_g1_i1	XLOC_022159	(AG)6	12	TR53370|c0_g1_i1	(AG)6	Ca_LG_4	(AG)7	34284926	34284939	TGAAGAACATGCGAAACCTG	60	CCTTCTCAACCTCCACTTCG	60	268		URR	--	--	--	--
TR53495|c0_g1_i1	No	(T)12ctctcggaagaatgtatctttcttttcagactgagatcg gt(A)11	64	--	--	--	--	--	--	ACTTCACCTTCAGCAAGCGT	60	ACAGAGATGGTGCGATTTGA	59	124	--	--	--	--	--	--
TR53637|c0_g1_i1	XLOC_022233	(TC)7	14	--	--	--	--	--	--	TTTCTTGTTATGCCAGCACG	60	AGCATACAGTTTCCGTCCGT	60	220	--	--	CCO27844.1	--	--	--
TR53643|c0_g1_i1	No	(CCA)5	15	--	--	--	--	--	--	CTCCAAATTGGCATTGGTCT	60	CCTCGCAGGTCAAGTATTCC	60	245	--	--	--	--	--	--
TR53670|c0_g1_i1	No	(GTT)6	18	--	--	--	--	--	--	AATCAGATTCATCTTGGCCG	60	GCCAACAAACAAATGCTGAA	60	123	--	--	--	--	--	--
TR53713|c0_g1_i1	XLOC_022264	(CCA)5	15	--	--	--	--	--	--	CAAAAGAACCAACATGGCCT	60	AGAAAATGGATGAGATGGCG	60	217	--	--	--	--	--	--
TR53792|c0_g1_i1	XLOC_022311	(TCG)5	15	--	--	--	--	--	--	ATCAACTAGTCGCCACGCTT	60	GCTCATGCAGTGGAAACAGA	60	199	--	--	XP_009515050.1	--	[C]	--
TR53807|c1_g1_i1	No	(TTC)6	18	--	--	--	--	--	--	TTCTTCGGTTGAGGAATTGG	60	CACCAACGATGGAATCACAG	60	118	--	--	--	--	--	--

Transcript IDs	Differentially expressed gene (DEG) IDs	SSR repeat-motifs	SSR repeat-length (bp)	*In silico* polymorphic SSRs						Forward primers (5'-3')	Annealing tempearture(0C)	Reverse primers (5'-3')	Annealing tempearture(0C)	Amplified product size (bp)	Structural annotation		Functional Annotation			
				C. microphyllum		ICC4958									Gene IDs	Sequence component of genes	NCBI nr IDs	TFs	KOG	KEGG
				Transcript	SSR repeat-motifs	Chromosomes	SSR repeat-motifs	Start physical positions (bp)	End physical positions (bp)											
TR53827|c0_g1_i1	XLOC_022329	(TA)8	16	--	--	--	--	--	--	GCAAAAGTGGTAGCAGAGCC	60	GCAGCTGGACATGCAGATTA	60	205	--	--	--	--	--	--
TR53841|c0_g1_i1	No	(ACA)5	15	TR53841|c0_g1_i1	(ACA)5	Ca_LG_6	(ACA)6	12553554	12553571	ACCGAAGATGACGACGAATC	60	ACGGTCCAACAAATAACGGA	60	243	Ca_14118	URR	--	--	--	--
TR53841|c0_g1_i2	No	(ACA)5	15	--	--	--	--	--	--	ACCGAAGATGACGACGAATC	60	ACGGTCCAACAAATAACGGA	60	243	--	--	--	--	--	--
TR53878|c0_g1_i1	No	(AT)6	12	--	--	--	--	--	--	CTTCGCATTTTCTGGTAAAGA	57	TCCCAGCATAATCAACAATGA	59	172	--	--	--	--	--	--
TR5388|c0_g1_i1	No	(TTG)5	15	--	--	--	--	--	--	GTGGCTGCTGCATCAAAATA	60	ACATCAATATGGAGGCCGAG	60	272	--	--	--	--	--	--
TR5388|c0_g2_i1	XLOC_022364	(TTG)5	15	--	--	--	--	--	--	GTGGCTGCTGCATCAAAATA	60	ACATCAATATGGAGGCCGAG	60	272	--	--	--	--	--	--
TR539|c3_g2_i1	XLOC_022417	(GT)8	16	--	--	--	--	--	--	TGGGCTCTTGTGGTTCTTCT	60	CCATGCGATTCTGGAGGTAT	60	184	--	--	--	--	--	--
TR53923|c0_g1_i1	XLOC_022386	(AGG)5	15	--	--	--	--	--	--	CCACAGCAGCATAAAAACCA	60	CATGGATCCACCACCACT	58	117	--	--	--	--	--	--
TR53951|c0_g1_i1	XLOC_022396	(GAA)5	15	--	--	--	--	--	--	TCCTAAACCCTAAACCCTACGA	59	GGTTTGGAAGAAGAGGGGAG	60	147	--	--	--	--	--	--
TR5406|c0_g1_i1	No	(TA)7	14	--	--	--	--	--	--	CAGATGGAGGTACAAAGTAAGCTACA	60	CATACATGCCCAAACCACAT	59	232	--	--	--	--	--	--
TR54145|c0_g1_i1	No	(GAT)6	18	--	--	--	--	--	--	AGAAACACATGATCCCTCCG	60	CCAAGATGAGCAAAACAGCA	60	170	--	--	--	--	--	--
TR54167|c0_g1_i1	No	(TGA)5	15	--	--	--	--	--	--	CGCTTTGTAGTAGGTGGGGT	59	AAGTTTGAGGTGCCTGGTTG	60	247	--	--	--	--	--	--
TR54454|c0_g1_i1	No	(CA)9	18	--	--	--	--	--	--	CCGCTCAAAATTGATTGTCAT	60	TGAACTGTTTCGACATTACTTGTG	59	156	--	--	--	--	--	--
TR54484|c0_g1_i1	No	(TC)6	12	--	--	--	--	--	--	TGTGATGATGGTCAATTGTTAGC	60	TGGGAAAGAGGGAAAAAGGT	60	189	--	--	--	--	--	--

Transcript IDs	Differentially expressed gene (DEG) IDs	SSR repeat-motifs	SSR repeat-length (bp)	*In silico* polymorphic SSRs						Forward primers (5'-3')	Annealing tempearture(0C)	Reverse primers (5'-3')	Annealing tempearture(0C)	Amplified product size (bp)	Structural annotation		Functional Annotation			
				C. microphyllum		ICC4958									Gene IDs	Sequence component of genes	NCBI nr IDs	TFs	KOG	KEGG
				Transcript	SSR repeat-motifs	Chromosomes	SSR repeat-motifs	Start physical positions (bp)	End physical positions (bp)											
TR54486|c0_g1_i1	No	(GAA)7	21	--	--	--	--	--	--	TGATTGTTCACCAATGCCA	59	TTTCTCTTCCTCCGTCGAGA	60	197	--	--	--	--	--	--
TR54504|c0_g1_i1	No	(TAA)5	15	--	--	--	--	--	--	AGGTGATGATTACATGGGCTG	60	AATGAGTGCCAAGTCCATCA	59	230	--	--	--	--	--	--
TR54548|c0_g1_i1	XLOC_022587	(CCA)5	15	--	--	--	--	--	--	AAGCAAAAGCAAGAAATGGC	59	CGATTCATCTTGCCCTTCAT	60	202	--	--	--	--	--	--
TR54553|c0_g1_i1	XLOC_022593	(A)10tatgtggattacagaaatgtaacttctagcacttaattt gatcaaacagtgatattagtagccacacg(T)10	88	--	--	--	--	--	--	ATTAGTTTCCGCAGGGGAGT	60	ATCATTCTGCCTGGTTGGTC	60	259	--	--	--	--	--	--
TR54587|c0_g1_i1	No	(ATAA)5	20	--	--	--	--	--	--	CACCACCAACATGGAAATAAG	58	TGCCACCACTTATGCTATCCT	60	191	--	--	--	--	--	--
TR54587|c0_g2_i1	No	(ATAA)5	20	--	--	--	--	--	--	CACCACCAACATGGAAATAAG	58	TGCCACCACTTATGCTATCCT	60	191	--	--	--	--	--	--
TR546|c0_g2_i1	XLOC_022653	(A)14t(A)12	27	--	--	--	--	--	--	ACTCGCTCCCCAATCTGTTA	60	ACCCATGAAAAACGGTTTGA	60	100	--	--	--	--	--	--
TR54611|c0_g1_i1	No	(TCT)7	21	--	--	--	--	--	--	TACAGCTGGTTAGGGCAGGT	60	ACGCAAGGAGAAGAAGGACA	60	167	--	--	--	--	--	--
TR54660|c0_g1_i1	No	(GT)9	18	TR54660|c0_g1_i1	(GT)9	Ca_LG_8	(GT)10	3589458	3589477	GAAGAAAGCCAATGACTCCG	60	TAGCCCTGCAAGTAGCAACA	60	257	Ca_19205	URR	--	--	--	--
TR54688|c0_g1_i1	No	(TCG)5	15	--	--	--	--	--	--	CTGCTTCTTGGTTGGAGGAG	60	GAGGACGATGAGAACACGGT	60	216	--	--	--	--	--	--
TR54688|c0_g1_i2	XLOC_022639	(TCG)5	15	--	--	--	--	--	--	CTGCTTCTTGGTTGGAGGAG	60	GAGGACGATGAGAACACGGT	60	180	--	--	--	--	--	--
TR54696|c0_g1_i1	XLOC_022647	(AC)9	18	--	--	--	--	--	--	AGCACACTCGCTACCAACACT	60	TGCTCCGAGTGTGTTCTGTC	60	216	--	--	--	--	--	--
TR54696|c0_g2_i1	No	(AC)9	18	--	--	--	--	--	--	AGCACACTCGCTACCAACACT	60	TGCTCCGAGTGTGTTCTGTC	60	216	--	--	--	--	--	--
TR54730|c0_g1_i1	No	(TTC)5	15	--	--	--	--	--	--	TCATACATGAACCTTCCCGC	61	AACATCACCACCGGAAGAAG	60	126	--	--	--	--	--	--

Transcript IDs	Differentially expressed gene (DEG) IDs	SSR repeat-motifs	SSR repeat-length (bp)	*In silico* polymorphic SSRs						Forward primers (5'-3')	Annealing tempearture(0C)	Reverse primers (5'-3')	Annealing tempearture(0C)	Amplified product size (bp)	Structural annotation		Functional Annotation			
				C. microphyllum		ICC4958									Gene IDs	Sequence component of genes	NCBI nr IDs	TFs	KOG	KEGG
				Transcript	SSR repeat-motifs	Chromosomes	SSR repeat-motifs	Start physical positions (bp)	End physical positions (bp)											
TR54730|c0_g2_i1	XLOC_022667	(TTC)5	15	--	--	--	--	--	--	CATACATGAAACTTCCCGCC	60	AACATCACCACCGGAAGAAG	60	125	--	--	--	--	--	--
TR54737|c0_g1_i1	XLOC_022672	(CCA)6	18	--	--	--	--	--	--	GAATCAGCTCCACCATCGTT	60	GGCGTGGATTGAAGTTTTGT	60	159	--	--	--	--	--	--
TR54770|c0_g1_i1	XLOC_022686	(TTG)5	15	--	--	--	--	--	--	TGCAAAACCTCTTGGACCTC	60	GGAATGGCATCACAGACTGA	60	261	--	--	--	--	--	--
TR548|c0_g1_i1	XLOC_022730	(TC)11	22	--	--	--	--	--	--	TCTCTCTTTCTCCTCCGCCT	61	TGAACTCGTCACGAACCTTG	60	264	--	--	--	--	--	--
TR548|c1_g3_i1	XLOC_022731	(CT)11	22	--	--	--	--	--	--	TCTTCCAAGTTCCCCCTCTT	60	ATGTTCACCGAGAAAATGGC	60	202	--	--	--	--	--	--
TR54815|c0_g1_i1	XLOC_022703	(GTT)6	18	--	--	--	--	--	--	TGATCAATCGTTGAGGGACA	60	CAAGCAGCAGTGAAAACCAA	60	187	--	--	--	--	--	--
TR5484|c0_g1_i1	XLOC_022708	(TTC)7	21	--	--	--	--	--	--	TCTCTCCTTGCACCACACAA	60	CGAAGAAGAGTTGGAGAAGGAA	60	161	--	--	--	--	--	--
TR54875|c0_g1_i1	XLOC_022714	(AC)7	14	--	--	--	--	--	--	TCTCACCATTTCCATCACTCA	59	GGTGGGTTGTGGTGTTAAGG	60	257	--	--	XP_004493513.1	--	[X]	--
TR54880|c0_g1_i2	No	(CT)8	16	--	--	--	--	--	--	ATGGGAAACGAAGACGATTG	60	CCGAAGCATGACAAACAAAA	60	250	--	--	--	--	--	--
TR54880|c0_g1_i5	No	(CT)8	16	--	--	--	--	--	--	ATGGGAAACGAAGACGATTG	60	CCGAAGCATGACAAACAAAA	60	250	--	--	--	--	--	--
TR54888|c0_g1_i1	No	(CT)6	12	--	--	--	--	--	--	CACTCACTGTCCTCCGTTCA	60	TCTGTGAATGCAACAACGGT	60	231	--	--	--	--	--	--
TR54888|c0_g1_i2	No	(CT)6	12	--	--	--	--	--	--	CACTCACTGTCCTCCGTTCA	60	TCTGTGAATGCAACAACGGT	60	231	--	--	--	--	--	--
TR54891|c0_g1_i1	XLOC_022723	(AG)9	18	--	--	--	--	--	--	AACCGACTTTGGCTGAGATG	60	GGTTCACCGATCCTGACACT	60	269	--	--	--	--	--	--
TR54900|c0_g1_i1	No	(GTG)6	18	--	--	--	--	--	--	CGTTCTTGCACTTGAAACCA	60	GTGTGACCCTGGCTTTGTCT	60	251	--	--	--	--	--	--

Transcript IDs	Differentially expressed gene (DEG) IDs	SSR repeat-motifs	SSR repeat-length (bp)	*In silico* polymorphic SSRs						Forward primers (5'-3')	Annealing tempearture(0C)	Reverse primers (5'-3')	Annealing tempearture(0C)	Amplified product size (bp)	Structural annotation		Functional Annotation			
				C. microphyllum		ICC4958									Gene IDs	Sequence component of genes	NCBI nr IDs	TFs	KOG	KEGG
				Transcript	SSR repeat-motifs	Chromosomes	SSR repeat-motifs	Start physical positions (bp)	End physical positions (bp)											
TR54900|c0_g1_i2	No	(GTG)5	15	--	--	--	--	--	--	TAGCAGATCTCATCGCCCTT	60	ACCACCACCACAAGAAGGAG	60	277	--	--	--	--	--	--
TR54900|c0_g1_i2	No	(GTG)5	15	--	--	--	--	--	--	TAGCAGATCTCATCGCCCTT	60	TGGTTTCAAGTGCAAGAACG	60	102	--	--	--	--	--	--
TR54900|c0_g1_i3	No	(GTG)6	18	--	--	--	--	--	--	CGTTCTTGCACTTGAAACCA	60	GTGTGACCCTGGCTTTGTCT	60	251	--	--	--	--	--	--
TR54900|c0_g1_i3	No	(GTG)5	15	--	--	--	--	--	--	TAGCAGATCTCATCGCCCTT	60	ACAAGGAGGCTGAAGTCGAA	60	265	--	--	--	--	--	--
TR54922|c0_g1_i1	XLOC_022754	(GAC)6	18	--	--	--	--	--	--	CAGAAACACAACACCGACGA	61	CGGTGTATGCGATTGAGAAA	60	213	--	--	--	--	--	--
TR54922|c0_g2_i1	No	(GAC)6	18	--	--	--	--	--	--	CAGAAACACAACACCGACGA	61	CGGTGTATGCGATTGAGAAA	60	213	--	--	--	--	--	--
TR54922|c0_g3_i1	No	(GAC)6	18	--	--	--	--	--	--	CAGAAACACAACACCGACGA	61	CGGTGTATGCGATTGAGAAA	60	213	--	--	--	--	--	--
TR54924|c0_g1_i1	XLOC_022755	(CAA)5	15	TR54924|c0_g1_i1	(CAA)5	Ca_LG_1	(CAA)11	16621778	16621810	CCCAAACCAAAAACCACATC	60	AAATTCATACCGCGAACACC	60	219	Ca_22545	URR	--	--	--	--
TR54933|c0_g2_i1	XLOC_022765	(TCA)6	18	--	--	--	--	--	--	AGGGTGAAAATGGTAGCGTG	60	GTTGAGGTGTAAGGGGCGTA	60	208	--	--	--	--	--	--
TR54934|c0_g1_i1	No	(TTA)5	15	--	--	--	--	--	--	GCTCGTCCAACCTGTCCTAC	60	TTCTTTGGCGCCATTAATTC	60	230	--	--	--	--	--	--
TR54934|c0_g1_i2	No	(TTA)5	15	--	--	--	--	--	--	GCTCGTCCAACCTGTCCTAC	60	TTCTTTGGCGCCATTAATTC	60	230	--	--	--	--	--	--
TR54936|c0_g1_i1	XLOC_022767	(TGA)5	15	--	--	--	--	--	--	CCGCAACAATGCTGAAACTA	60	TGGGTGTGGGAGGATATGAT	60	168	--	--	--	--	--	--
TR54939|c0_g1_i1	XLOC_022768	(TC)6	12	--	--	--	--	--	--	TTCCCAAACAGGAGTGGTTC	60	GATGGTTCAGATCAAAGGGC	59	256	--	--	--	--	--	--
TR5494|c0_g1_i1	No	(TC)8	16	--	--	--	--	--	--	TCGGTTCACTTCCTTCTCGT	60	CAGCTCCTTTCCTGGTTCAG	60	173	--	--	--	--	--	--

Transcript IDs	Differentially expressed gene (DEG) IDs	SSR repeat-motifs	SSR repeat-length (bp)	*In silico* polymorphic SSRs						Forward primers (5'-3')	Annealing tempearture(0C)	Reverse primers (5'-3')	Annealing tempearture(0C)	Amplified product size (bp)	Structural annotation		Functional Annotation			
				C. microphyllum		ICC4958									Gene IDs	Sequence component of genes	NCBI nr IDs	TFs	KOG	KEGG
				Transcript	SSR repeat-motifs	Chromosomes	SSR repeat-motifs	Start physical positions (bp)	End physical positions (bp)											
TR5494|c0_g1_i2	No	(TC)8	16	--	--	--	--	--	--	TCGGTTCACTTCCTTCTCGT	60	CAGCTCCTTTCCTGGTTCAG	60	173	--	--	--	--	--	--
TR54950|c0_g1_i1	No	(AT)6	12	--	--	--	--	--	--	GGTGATATAGACAGGGCCGA	60	CGGTTAAACCACAACCGACT	60	280	--	--	--	--	--	--
TR54958|c0_g1_i1	XLOC_022791	(TCA)5	15	--	--	--	--	--	--	GAACCAGAAAAAGGCCAACA	60	TCTTAACGCTGCTGTCGATG	60	197	--	--	--	--	--	--
TR54962|c0_g1_i1	XLOC_022796	(TC)8	16	--	--	--	--	--	--	TTTGTCACATGTTCTCTCCCTT	59	TTTTGGTGTTGTGTTGGAGG	59	224	--	--	--	--	--	--
TR54966|c0_g2_i1	XLOC_022802	(AG)7	14	--	--	--	--	--	--	AAAGCGGAGGGAGAGAGATT	59	ACGATGTTGGAGGAACCTTG	60	189	--	--	--	--	--	--
TR55007|c0_g1_i1	No	(ATTA)5	20	--	--	--	--	--	--	GCTTGAAGATGGGAGAAAGACA	61	AGGGGGAAATTACTTTTTCG	57	131	--	--	--	--	--	--
TR55007|c0_g1_i2	No	(ATTA)5	20	--	--	--	--	--	--	GCTTGAAGATGGGAGAAAGACA	61	AGGGGGAAATTACTTTTTCG	57	131	--	--	--	--	--	--
TR55014|c0_g2_i1	XLOC_022847	(AGA)5	15	--	--	--	--	--	--	TCTTTGGAGAACGGAACACC	60	AGCAGCCAAATTCTCCAAGA	60	276	--	--	--	--	--	--
TR55015|c1_g1_i1	XLOC_022849	(TCT)5	15	--	--	--	--	--	--	CATCAGTAACCGCTCAACGA	60	TTGGAGAGAGTGGAGAGGGA	60	234	--	--	XM_004505016.1	--	[J]	K02988
TR55041|c0_g1_i1	XLOC_022873	(AAG)6	18	--	--	--	--	--	--	CACTGAATCGCAGTGGAAGA	60	GATCGGTGGTTTTGGCTCTA	60	196	--	--	--	--	--	--
TR55047|c0_g2_i1	XLOC_022880	(GA)17	34	--	--	--	--	--	--	AGACTCACAAGCAAAACCAACA	60	CCTCAATCCCACAATTCCAC	60	228	--	--	--	--	--	--
TR5505|c0_g1_i1	XLOC_022889	(GAT)6gaggatgaggatgaagttgaggttgaggttgagg ataaagcaagagcagcagccgcg(GCA)5	90	--	--	--	--	--	--	CAATTTTCCATGCTGAGGTTG	60	TTGCTTAATGGGACACGAGA	59	221	--	--	KFM67492.1	--	[S]	--
TR55053|c0_g1_i1	XLOC_022885	(ATC)5	15	--	--	--	--	--	--	CCAAGAAACAAACCTCGGAA	60	CCCCTTGTTGTTTTCTCCAA	60	256	--	--	--	--	--	--
TR55053|c0_g1_i1	XLOC_022885	(TCA)5	15	--	--	--	--	--	--	TTGGAGAAAACAACAAGGGG	60	CGTATGCCTCTTTGACAGCA	60	245	--	--	--	--	--	--

Transcript IDs	Differentially expressed gene (DEG) IDs	SSR repeat-motifs	SSR repeat-length (bp)	*In silico* polymorphic SSRs						Forward primers (5'-3')	Annealing tempearture(0C)	Reverse primers (5'-3')	Annealing tempearture(0C)	Amplified product size (bp)	Structural annotation		Functional Annotation			
				C. microphyllum		ICC4958									Gene IDs	Sequence component of genes	NCBI nr IDs	TFs	KOG	KEGG
				Transcript	SSR repeat-motifs	Chromosomes	SSR repeat-motifs	Start physical positions (bp)	End physical positions (bp)											
TR55054|c0_g1_i1	XLOC_022886	(AG)7	14	--	--	--	--	--	--	TCCCCAAGTCTCATTAACGC	60	GATCGGTGAGATCGAATGTG	59	213	--	--	--	--	--	--
TR55070|c0_g2_i1	No	(CT)6	12	--	--	--	--	--	--	TCCATTCCAACACGTACAGC	60	AGGAAGCGTCCACGTCTAAA	60	112	--	--	--	--	--	--
TR5523|c0_g1_i1	XLOC_022960	(AT)6acaacaaaaccatgtgtagctgctagctaataagcagaatgtacagaaaaaattgaagcatgaggactgaggagacaaagaaag(A)10	106	--	--	--	--	--	--	CGTGATGCATGATGCTTTCT	60	CCGTGCTAAGCTGCAAAGAT	61	260	--	--	--	--	--	--
TR55234|c0_g1_i1	XLOC_022958	(GGT)5	15	--	--	--	--	--	--	TGCCACACACCAAATAGCAT	60	AAAACATGTTCCAAGCCCAC	60	124	--	--	--	--	--	--
TR55269|c0_g1_i1	XLOC_022969	(GAA)5	15	TR55269|c0_g1_i1	(GAA)5	Ca_LG_2	(GAA)6	26542292	26542309	TTGCTGAACAAGCCTTCCTT	60	CACCAACCACAAAATCACCA	60	280		URR	--	--	--	--
TR55269|c0_g1_i2	No	(GAA)5	15	--	--	--	--	--	--	TTGCTGAACAAGCCTTCCTT	60	CACCAACCACAAAATCACCA	60	280	--	--	--	--	--	--
TR55309|c0_g1_i1	XLOC_023002	(AAG)5	15	--	--	--	--	--	--	AGTTGATGATGCAAACGCAA	60	ACGAAGGGGTCAGAGGAGAT	60	218	--	--	--	--	--	--
TR55314|c0_g1_i1	XLOC_023007	(TCC)5	15	--	--	--	--	--	--	CCTGAGATTTCCGTGGTTGT	60	GGCCATCTTGAATCAAAGGA	60	181	--	--	XP_004491771.1	--	[T]	K01611
TR55350|c0_g1_i1	XLOC_023035	(AG)8	16	TR55350|c0_g1_i1	(AG)8	Ca_LG_3	(AG)9	34358378	34358395	TCCAACCTTTGCATTTGTCA	60	TGCCCACAAAAATATCAGCA	60	243		URR	--	--	--	--
TR55350|c0_g1_i1	XLOC_023035	(TCT)6	18	TR55350|c0_g1_i1	(AG)8	Ca_LG_3	(AG)9	34358378	34358395	TGCTGATATTTTTGTGGGCA	60	GCAATGAAAAAGACACAAAAGAAA	60	194		URR	--	--	--	--
TR55378|c0_g1_i1	No	(AAG)5	15	--	--	--	--	--	--	TTTCTTTTGAGAACCGAAAGATT	58	CATTTTCATGAATACAAAGACGC	59	218	--	--	--	--	--	--
TR554|c0_g2_i1	XLOC_023117	(CCG)5	15	--	--	--	--	--	--	AAGTGGCTTGTTCTTCTCCG	59	CCCTTGCCTCCAACAACTTA	60	165	--	--	--	--	--	--
TR55442|c1_g1_i2	No	(TA)6	12	--	--	--	--	--	--	TCAGTGTCCACACCAACACA	60	GTTGGTCAAAATGTGGCTTG	59	258	--	--	--	--	--	--
TR55450|c0_g1_i1	XLOC_023063	(GCA)6	18	--	--	--	--	--	--	ACCTACCGAAATTCTCTCGC	58	TGCAATCGTAATCATCTCCG	60	247	--	--	CEJ93411.1	WRKY	[T]	K14512

Transcript IDs	Differentially expressed gene (DEG) IDs	SSR repeat-motifs	SSR repeat-length (bp)	*In silico* polymorphic SSRs						Forward primers (5'-3')	Annealing tempearture(0C)	Reverse primers (5'-3')	Annealing tempearture(0C)	Amplified product size (bp)	Structural annotation		Functional Annotation			
				C. microphyllum		ICC4958														
				Transcript	SSR repeat-motifs	Chromosomes	SSR repeat-motifs	Start physical positions (bp)	End physical positions (bp)						Gene IDs	Sequence component of genes	NCBI nr IDs	TFs	KOG	KEGG
TR55454|c0_g3_i1	XLOC_023069	(CAA)6	18	--	--	--	--	--	--	TTTGGAGAATGAAGGAGAAGG	58	ATCCACCAGAGTAGCGATGG	60	202	--	--	--	--	--	--
TR55464|c0_g1_i2	No	(TG)6	12	--	--	--	--	--	--	CCTTGGACTGTTTGTAGCCC	60	CAAGCTGGCATTTGAACAGA	60	275	--	--	--	--	--	--
TR55475|c0_g1_i2	No	(A)10tcaaatattaaaaattgtgggcgacaactataaaaa tacttgagcttcctc(A)12	73	--	--	--	--	--	--	CCTTATGTGTTTCAGATATTTCCC	58	GGTTGGTGGTCCCATTCTAA	60	178	--	--	--	--	--	--
TR55475|c1_g1_i1	XLOC_023094	(AAT)6	18	--	--	--	--	--	--	TTTTGTGTGACTGAGATAGGGG	59	AATTGGTTGGGGAAGGAAAC	60	204	--	--	--	--	--	--
TR55479|c0_g1_i1	XLOC_023097	(A)10tatatattcccaaggtagttgtgtgttgtactagcatttt gttggtcactaaaccataacacgaaaccgaaag(A)10	93	--	--	--	--	--	--	AGGGAAGTCACAATCAAAGCA	60	CACGTTCACATGGATCATCA	59	229	--	--	--	--	--	--
TR55488|c0_g1_i1	No	(TC)7	14	--	--	--	--	--	--	TGCTCAAAGTTCTTTCGACATT	59	TGATGCTGAATTCAAAAAGGG	60	195	--	--	--	--	--	--
TR55488|c0_g2_i1	No	(TC)7	14	--	--	--	--	--	--	TGCTCAAAGTTCTTTCGACATT	59	TGATGCTGAATTCAAAAAGGG	60	195	--	--	--	--	--	--
TR55498|c0_g1_i1	No	(AACTCT)5	30	--	--	--	--	--	--	CAAGGGCAAGACACAAAACA	60	GTGGGCCCAACACTAGAAAA	60	238	--	--	--	--	--	--
TR55498|c0_g3_i1	No	(AACTCT)6	36	--	--	--	--	--	--	CAAGGGCAAGACACAAAACA	60	GTGGGCCCAACACTAGAAAA	60	244	--	--	--	--	--	--
TR55498|c1_g1_i1	No	(TA)6	12	--	--	--	--	--	--	GCTTCCTACTCTGTGGAAGGTT	59	TCCAAAATGTGGGATGTGAT	59	141	--	--	--	--	--	--
TR55498|c1_g1_i1	No	(TA)9	18	--	--	--	--	--	--	TGCCAGTGATGTGATATTGTTG	59	GGGATAAATCTATCTCATAGACA TGAA	58	193	--	--	--	--	--	--
TR55505|c0_g1_i1	XLOC_023124	(AT)8	16	--	--	--	--	--	--	AAACCACACATGCTAACTCGC	60	AAGCCCTTCTTCTCCTTTCG	60	172	--	--	NP_001287926.1	--	[X]	--
TR55515|c0_g1_i1	No	(T)12ccgcgcacgccctatatatatatcgtgagagtggga agagggggatgttctttcttgtttttca(T)11	87	--	--	--	--	--	--	GCGGGGCACAGTTTTATTTA	60	GGCAGAGGCTAGAACTCCCT	60	139	--	--	--	--	--	--
TR55534|c1_g1_i1	XLOC_023159	(CA)6	12	--	--	--	--	--	--	CATCACAGTCCAGAAATTGGAT	59	TGTGGTAACGAGGCATTTCA	60	265	--	--	--	--	--	--

Transcript IDs	Differentially expressed gene (DEG) IDs	SSR repeat-motifs	SSR repeat-length (bp)	*In silico* polymorphic SSRs						Forward primers (5'-3')	Annealing tempearture(0C)	Reverse primers (5'-3')	Annealing tempearture(0C)	Amplified product size (bp)	Structural annotation		Functional Annotation			
				C. microphyllum		ICC4958														
				Transcript	SSR repeat-motifs	Chromosomes	SSR repeat-motifs	Start physical positions (bp)	End physical positions (bp)						Gene IDs	Sequence component of genes	NCBI nr IDs	TFs	KOG	KEGG
TR55534|c1_g1_i3	No	(CA)6	12	--	--	--	--	--	--	CATCACAGTCCAGAAATTGGAT	59	TGTGGTAACGAGGCATTTCA	60	265	--	--	--	--	--	--
TR55541|c2_g1_i1	No	(CT)8	16	--	--	--	--	--	--	CTTCATTTTCCTCAACCCCA	60	TTCTGCCATCGACTCTGTTG	60	272	--	--	--	--	--	--
TR55541|c2_g1_i2	No	(CT)8	16	--	--	--	--	--	--	CTTCATTTTCCTCAACCCCA	60	TAGACGACGCCGTATTGTTG	60	211	--	--	--	--	--	--
TR55571|c0_g1_i1	XLOC_023190	(CT)7	14	--	--	--	--	--	--	TAGCATGTGCTTGGGTTCAG	60	GTGGGAGGAAGAGGAACACA	60	149	--	--	--	--	--	--
TR55571|c0_g2_i1	No	(CT)7	14	--	--	--	--	--	--	TAGCATGTGCTTGGGTTCAG	60	GTGGGAGGAAGAGGAACACA	60	149	--	--	--	--	--	--
TR55615|c0_g1_i1	XLOC_023217	(CTC)6	18	--	--	--	--	--	--	CCTCCATTGTTCCAAACCAC	60	TCTCAATGATTTTGGAGGAGG	59	148	--	--	--	--	--	--
TR55623|c0_g1_i1	No	(TGTT)6	24	--	--	--	--	--	--	AGCCAATGTCGTTCTTTCAA	58	TGCTTGCAGAATTTATGGTTTG	60	236	--	--	--	--	--	--
TR55646|c0_g1_i1	XLOC_023228	(TC)6	12	TR55646|c0_g1_i1	(TC)6	Ca_LG_5	(TC)15	24314121	24314150	TCAATGGACCTGTGTTTTGC	60	TGTACAACAAAGCTGCCCAA	60	127	Ca_04694	URR	--	--	--	--
TR55663|c0_g1_i1	No	(CAT)5	15	--	--	--	--	--	--	TCGTCAACAATGTGGCCTAA	60	TTGATGATTCAGTTGGAACCC	60	145	--	--	--	--	--	--
TR55781|c0_g1_i1	XLOC_023278	(TCT)5	15	--	--	--	--	--	--	GCACCAACGGATCTTCACTT	60	GCTCGCGTCCATTATCTTGT	60	220	--	--	--	--	--	--
TR558|c0_g1_i3	No	(TC)7	14	--	--	--	--	--	--	GCACCAAACAGAAACAACCA	60	AGAACTTGACCGCATTGACC	60	280	--	--	--	--	--	--
TR558|c0_g1_i3	No	(TTG)5	15	--	--	--	--	--	--	GCACCAAACAGAAACAACCA	60	AGAACTTGACCGCATTGACC	60	280	--	--	--	--	--	--
TR558|c0_g1_i5	XLOC_023314	(TC)7	14	--	--	--	--	--	--	GCACCAAACAGAAACAACCA	60	AGAACTTGACCGCATTGACC	60	280	--	--	--	--	--	--
TR558|c0_g1_i5	XLOC_023314	(TTG)5	15	--	--	--	--	--	--	GCACCAAACAGAAACAACCA	60	AGAACTTGACCGCATTGACC	60	280	--	--	--	--	--	--

Transcript IDs	Differentially expressed gene (DEG) IDs	SSR repeat-motifs	SSR repeat-length (bp)	*In silico* polymorphic SSRs						Forward primers (5'-3')	Annealing tempearture(0C)	Reverse primers (5'-3')	Annealing tempearture(0C)	Amplified product size (bp)	Structural annotation		Functional Annotation			
				C. microphyllum		ICC4958									Gene IDs	Sequence component of genes	NCBI nr IDs	TFs	KOG	KEGG
				Transcript	SSR repeat-motifs	Chromosomes	SSR repeat-motifs	Start physical positions (bp)	End physical positions (bp)											
TR55864|c0_g1_i1	No	(CAG)5	15	--	--	--	--	--	--	CACCGCACAGTGCTTCATAG	60	CTGTCGCCACTTCTGCTGT	60	214	--	--	--	--	--	--
TR55913|c0_g1_i1	No	(CCA)5	15	--	--	--	--	--	--	CAATCCCACCATCATTTTCC	60	CAGGTCGAGGATTTTTGCAT	60	202	--	--	--	--	--	--
TR55913|c0_g2_i1	No	(CCA)5	15	--	--	--	--	--	--	CAATCCCACCATCATTTTCC	60	CAGGTCGAGGATTTTTGCAT	60	201	--	--	--	--	--	--
TR55913|c0_g3_i1	No	(CCA)5	15	--	--	--	--	--	--	CCAATCCTAATAATTAAAAACAACA A	57	CAGGTCGAGGATTTTTGCAT	60	131	--	--	--	--	--	--
TR55933|c0_g1_i1	No	(AAG)5	15	--	--	--	--	--	--	CAAAATGTCCGAGAAAGCAA	59	AAAACGGTGAAGTTGATGGC	60	264	--	--	--	--	--	--
TR55933|c0_g1_i2	No	(AAG)5	15	--	--	--	--	--	--	CAAAATGTCCGAGAAAGCAA	59	TCAAGTCCTGCTTGTCGATG	60	170	--	--	--	--	--	--
TR55933|c0_g1_i3	No	(AAG)5	15	--	--	--	--	--	--	CAAAATGTCCGAGAAAGCAA	59	TCAAGTCCTGCTTGTCGATG	60	170	--	--	--	--	--	--
TR55934|c0_g2_i1	No	(TTG)5	15	--	--	--	--	--	--	GAACTTGGAGGGTCACTCAGA	59	TCAGTTGCCCTCATAGCTTGT	60	268	--	--	--	--	--	--
TR55966|c0_g1_i1	XLOC_023368	(A)18(CA)6	30	--	--	--	--	--	--	TTTCCGTGTGTTGACTTTATCG	60	TGGGGCAGAGGAAATGAGTA	61	274	--	--	--	--	--	--
TR55967|c0_g1_i1	XLOC_023369	(AC)8tctcgttgttgtttatcaccatggagcctctctcttccac caaacac(AAT)5	78	--	--	--	--	--	--	ACCCAAACTCAAACGCAAAA	61	CTTCCTTCAAACCAAGCCAA	60	189	--	--	--	--	--	--
TR55977|c0_g1_i1	No	(TGA)5tccgctgaaataaaatctcaatcgtaactctcaaa taaaaccatctacctccaaaaaatagatt(A)12	91	--	--	--	--	--	--	CCGAAGTCCAATCCGTTACA	61	AAAAGTGCTGCCAGGTTTGT	60	186	--	--	--	--	--	--
TR55977|c0_g1_i2	No	(TTTAT)5	25	--	--	--	--	--	--	TCACTCCACCTTAGATCCACC	59	AGCTCCCAACAATCTCTGACA	60	249	--	--	--	--	--	--
TR55977|c0_g1_i3	No	(TTTAT)5	25	--	--	--	--	--	--	TCACTCCACCTTAGATCCACC	59	AGCTCCCAACAATCTCTGACA	60	249	--	--	--	--	--	--
TR55994|c0_g1_i1	XLOC_023387	(CCA)5	15	--	--	--	--	--	--	ACCCAAAACCCACATAACCA	60	GGAACTTGGTGAGGTTTGGA	60	173	--	--	--	--	--	--

Transcript IDs	Differentially expressed gene (DEG) IDs	SSR repeat-motifs	SSR repeat-length (bp)	*In silico* polymorphic SSRs						Forward primers (5'-3')	Annealing tempearture(0C)	Reverse primers (5'-3')	Annealing tempearture(0C)	Amplified product size (bp)	Structural annotation		Functional Annotation			
				C. microphyllum		ICC4958									Gene IDs	Sequence component of genes	NCBI nr IDs	TFs	KOG	KEGG
				Transcript	SSR repeat-motifs	Chromosomes	SSR repeat-motifs	Start physical positions (bp)	End physical positions (bp)											
TR56021|c0_g1_i1	XLOC_023397	(AAC)6	18	--	--	--	--	--	--	TTCCATGGCTTCTTCAATCC	60	AATCCAACCAGCAGCAGAAG	60	274	--	--	--	--	--	--
TR56152|c0_g1_i1	No	(GATA)6	24	--	--	--	--	--	--	GGACTCTTTAGGCCAATTCCA	60	TCTGAAGTGCAAAGTTCTCAGG	60	106	--	--	--	--	--	--
TR56171|c0_g1_i1	XLOC_023428	(TCT)5	15	--	--	--	--	--	--	GCATCAGTACTAGACAATCACAATCA	60	CAAGTTCAGCCGGAGAAGAA	61	250	--	--	--	--	--	--
TR56200|c0_g1_i1	XLOC_023434	(CA)7	14	--	--	--	--	--	--	CCATTGTTTGTTTGCGTTTG	60	CGAAAAGCAAGGATAGCGAG	60	127	--	--	--	--	--	--
TR56297|c0_g1_i1	XLOC_023453	(ACA)5	15	--	--	--	--	--	--	TTGCAATAACAATGCACATACTACA	60	GGTTTTGGGATTTGGGATTT	60	271	--	--	--	--	--	--
TR56402|c0_g1_i1	No	(CCT)5	15	--	--	--	--	--	--	CTACTTCCCATCGGCAAAAA	60	ATCCTCCAGAAGAAGCAGCA	60	256	--	--	--	--	--	--
TR56482|c0_g1_i1	No	(GGC)5	15	--	--	--	--	--	--	ATACGTCCCAGGACTTGTGC	60	GACCCGAGCCTCCATGAC	62	239	--	--	--	--	--	--
TR56485|c0_g1_i1	XLOC_023501	(AG)6	12	--	--	--	--	--	--	AGTGCCTCCAATTTCACCTC	59	CCATATGTATCCAATCAAAGCTCA	60	231	--	--	--	--	--	--
TR565|c0_g1_i1	No	(AT)7	14	--	--	--	--	--	--	AACAGAAGAAGCATGGCACA	59	GGTAGCATTTTACCGGAGCA	60	177	--	--	--	--	--	--
TR565|c0_g1_i2	No	(AT)7	14	--	--	--	--	--	--	AACAGAAGAAGCATGGCACA	59	GGTAGCATTTTACCGGAGCA	60	177	--	--	--	--	--	--
TR565|c0_g1_i5	No	(AT)7	14	--	--	--	--	--	--	AACAGAAGAAGCATGGCACA	59	GGTAGCATTTTACCGGAGCA	60	177	--	--	--	--	--	--
TR56565|c0_g1_i1	No	(TGA)6	18	--	--	--	--	--	--	ATGAAATCACCGGGAGACTG	60	TCATCAATCGCTGAAACTGC	60	279	--	--	--	--	--	--
TR56575|c0_g1_i1	No	(GA)6	12	--	--	--	--	--	--	AATATTAAGGGTCCCCGACG	60	AAGAGGTGCATCGAGGTGTT	60	231	--	--	--	--	--	--
TR56575|c0_g2_i1	No	(GA)7a(AG)7	29	--	--	--	--	--	--	AATATTAAGGGTCCCCGACG	60	AAAGAGGTGCATCGAGGTGT	60	248	--	--	--	--	--	--

Transcript IDs	Differentially expressed gene (DEG) IDs	SSR repeat-motifs	SSR repeat-length (bp)	*In silico* polymorphic SSRs						Forward primers (5'-3')	Annealing tempearture(0C)	Reverse primers (5'-3')	Annealing tempearture(0C)	Amplified product size (bp)	Structural annotation		Functional Annotation			
				C. microphyllum		ICC4958									Gene IDs	Sequence component of genes	NCBI nr IDs	TFs	KOG	KEGG
				Transcript	SSR repeat-motifs	Chromosomes	SSR repeat-motifs	Start physical positions (bp)	End physical positions (bp)											
TR56576|c0_g1_i2	No	(ATA)5	15	--	--	--	--	--	--	AACTCCCTGCCAGTACGAGA	60	TGTTGCTTGCTTGCTTCTTG	60	204	--	--	--	--	--	--
TR56576|c0_g1_i4	No	(ATA)5	15	--	--	--	--	--	--	AACTCCCTGCCAGTACGAGA	60	TGTTGCTTGCTTGCTTCTTG	60	204	--	--	--	--	--	--
TR56576|c0_g1_i5	No	(ATA)5	15	--	--	--	--	--	--	AACTCCCTGCCAGTACGAGA	60	TGTTGCTTGCTTGCTTCTTG	60	204	--	--	--	--	--	--
TR56576|c0_g1_i6	No	(ATA)5	15	--	--	--	--	--	--	AACTCCCTGCCAGTACGAGA	60	TGTTGCTTGCTTGCTTCTTG	60	204	--	--	--	--	--	--
TR56581|c0_g1_i1	XLOC_023528	(AAC)7	21	--	--	--	--	--	--	CTCTCAACTCCAACACCACC	58	GCTTGATCCAAACCAGAATCA	60	280	--	--	--	--	--	--
TR56581|c3_g1_i1	XLOC_023532	(GAA)5	15	--	--	--	--	--	--	TGGGTATTTGACCATTTGGG	60	GCCCAACCGAATAAGTTTCA	60	143	--	--	--	--	--	--
TR56581|c5_g1_i1	XLOC_023533	(CAA)7	21	--	--	--	--	--	--	CAATCTTAACTTAAACCTTCCCAA	58	GCTGACCCCATTCCTGTAAC	59	140	--	--	--	--	--	--
TR56582|c0_g1_i1	No	(CT)9	18	--	--	--	--	--	--	CCCTAGCAGCCACATTCATT	60	CCCTTTTCCAACCTTCCAAT	60	231	--	--	--	--	--	--
TR56582|c0_g1_i2	No	(CT)9	18	--	--	--	--	--	--	CCCTAGCAGCCACATTCATT	60	CCCTTTTCCAACCTTCCAAT	60	231	--	--	--	--	--	--
TR56584|c0_g1_i1	XLOC_023534	(TCT)6	18	--	--	--	--	--	--	CATTGAGTGGCTCAAACCCT	60	GCAATCCAATGTTCAAAGCC	60	236	--	--	--	--	--	--
TR56584|c0_g2_i1	XLOC_023535	(GAA)5	15	TR56584|c0_g2_i1	(GAA)5	Ca_LG_1	(GAA)6	8719947	8719964	TTGCACATTGGATTGCCTAA	60	TCCTAAATTCGGATCCATGC	60	259	Ca_21318	DRR	--	--	--	--
TR56600|c0_g1_i1	No	(GAA)8ggctcaacatcatcacaagcagcttcttcatctgaacaagt(AGA)7	86	--	--	--	--	--	--	GCCTCCAAGGATGATCAGAA	60	GATTCAGCGTCGGTTTCTTC	60	260	--	--	--	--	--	--
TR56600|c0_g1_i2	No	(GAA)8ggctcaacatcatcacaagcagcttcttcatctgaacaagt(AGA)7	86	--	--	--	--	--	--	GCCTCCAAGGATGATCAGAA	60	GAGGATTTTGAAGAAGGGGG	60	218	--	--	--	--	--	--
TR56601|c1_g1_i1	No	(TC)8taa(AAG)5	34	--	--	--	--	--	--	ACCTTGTCACAGTTCGGACC	60	GGAGAGGGAGGTTGTTGTGA	60	228	--	--	--	--	--	--

Transcript IDs	Differentially expressed gene (DEG) IDs	SSR repeat-motifs	SSR repeat-length (bp)	*In silico* polymorphic SSRs						Forward primers (5'-3')	Annealing tempearture(0C)	Reverse primers (5'-3')	Annealing tempearture(0C)	Amplified product size (bp)	Structural annotation		Functional Annotation			
				C. microphyllum		ICC4958									Gene IDs	Sequence component of genes	NCBI nr IDs	TFs	KOG	KEGG
				Transcript	SSR repeat-motifs	Chromosomes	SSR repeat-motifs	Start physical positions (bp)	End physical positions (bp)											
TR56601|c1_g1_i2	No	(TC)8taa(AAG)5	34	--	--	--	--	--	--	ACCTTGTCACAGTTCGGACC	60	GGAGAGGGAGGTTGTTGTGA	60	228	--	--	--	--	--	--
TR56615|c0_g2_i1	No	(TC)8	16	--	--	--	--	--	--	AAGCTCTCAGAACCACCGAA	60	AGAAAGAGGCATAGCCACCA	60	231	--	--	--	--	--	--
TR56625|c0_g1_i2	No	(AG)7	14	--	--	--	--	--	--	ATTCGTCATTTCAGTCCCCA	60	CCACCATCTCTTCGTATCGC	61	221	--	--	--	--	--	--
TR56625|c0_g1_i3	No	(AG)7	14	--	--	--	--	--	--	ATTCGTCATTTCAGTCCCCA	60	CCACCATCTCTTCGTATCGC	61	221	--	--	--	--	--	--
TR56631|c0_g1_i2	No	(TCG)5	15	--	--	--	--	--	--	TCCTTTCAATTTGGTTGGTTG	60	GGTGAAAGTGTTGCGTGTGT	60	206	--	--	--	--	--	--
TR56631|c0_g1_i3	No	(TCG)5	15	--	--	--	--	--	--	TCCTTTCAATTTGGTTGGTTG	60	GGTGAAAGTGTTGCGTGTGT	60	206	--	--	--	--	--	--
TR56641|c4_g1_i1	XLOC_023597	(A)13cattctacataaagaatgataaaaccaatctataca agaacatacaaaaaaagcac(AAG)6	87	--	--	--	--	--	--	CAGCACCAAAAGAATCGAAA	59	GCTTGTTGGGCAAAGAAGAG	60	275	--	--	--	--	--	--
TR56642|c0_g1_i1	No	(TAT)5	15	--	--	--	--	--	--	GTTCGATGTTTCCTTCCCCT	60	GCATCACCTTCTTTCTCTGGTT	60	222	--	--	--	--	--	--
TR56642|c0_g2_i1	No	(TAT)5	15	--	--	--	--	--	--	GTTCGATGTTTCCTTCCCCT	60	GCATCACCTTCTTTCTCTGGTT	60	221	--	--	--	--	--	--
TR56656|c0_g2_i1	No	(GA)7a(AG)11	37	--	--	--	--	--	--	AGGAATCTGTGTGCGGAATC	60	CATCCCAAACCCACAAAAAC	60	235	--	--	--	--	--	--
TR56656|c0_g2_i2	No	(GA)7a(AG)11	37	--	--	--	--	--	--	AGGAATCTGTGTGCGGAATC	60	CATCCCAAACCCACAAAAAC	60	235	--	--	--	--	--	--
TR56660|c0_g1_i1	XLOC_023619	(GA)6	12	--	--	--	--	--	--	ACCATCACCATCATCGACCT	60	GGCGGTACTTTCATGCTGTT	60	165	--	--	--	--	--	--
TR56695|c0_g1_i1	No	(TCT)6	18	--	--	--	--	--	--	GGCAGCTTTTCGAGTGGATA	60	ACCCGATGAAGAGGAGGAAG	61	241	--	--	--	--	--	--
TR5670|c0_g1_i1	XLOC_023641	(TTG)5	15	--	--	--	--	--	--	TATGGCGTATGGAGGAGGAG	60	GTAGTAGCAGCAGCGGCAGT	61	124	--	--	NP_001119609.1	GeBP	[R]	--

Transcript IDs	Differentially expressed gene (DEG) IDs	SSR repeat-motifs	SSR repeat-length (bp)	*In silico* polymorphic SSRs						Forward primers (5'-3')	Annealing tempearture(0C)	Reverse primers (5'-3')	Annealing tempearture(0C)	Amplified product size (bp)	Structural annotation		Functional Annotation			
				C. microphyllum		ICC4958														
				Transcript	SSR repeat-motifs	Chromosomes	SSR repeat-motifs	Start physical positions (bp)	End physical positions (bp)						Gene IDs	Sequence component of genes	NCBI nr IDs	TFs	KOG	KEGG
TR56715|c0_g1_i1	No	(AC)8	16	TR56715|c0_g1_i1	(AC)8	Ca_LG_2	(AC)6	31614783	31614794	AAAAAGGTTGACAGGTTGGC	59	GGTGCGTAAGAAAAGGGACA	60	183		URR	--	--	--	--
TR56834|c0_g1_i1	No	(TG)7	14	--	--	--	--	--	--	TTCGCAGCACCTGCTTATAC	59	TAAGTGGTGCAACGCAAATG	61	193	--	--	--	--	--	--
TR56843|c0_g1_i1	XLOC_023677	(CAA)5	15	--	--	--	--	--	--	CCCTTCTCCACCCAATTTTT	60	CACGGTAAGTAACATGGGGG	60	259	--	--	--	--	--	--
TR56843|c0_g1_i1	XLOC_023677	(CA)6	12	--	--	--	--	--	--	GCTCGTCACTTTGTCTTCCC	60	AAAAATTGGGTGGAGAAGGG	60	206	--	--	--	--	--	--
TR56850|c0_g1_i1	XLOC_023680	(TC)9	18	--	--	--	--	--	--	TTGTGAAGCGAAGCGTATTG	60	TCAAAAGGGTGATTCCCATC	60	278	--	--	--	--	--	--
TR56980|c0_g1_i1	XLOC_023735	(ATA)5	15	--	--	--	--	--	--	TTGTTAATGGTATTCGTTCATTAGA	57	GATTTCCCCCACAAAACAGA	60	157	--	--	XM_004511728.1	--	[RT]	--
TR57095|c0_g1_i1	XLOC_023751	(TCA)5	15	--	--	--	--	--	--	TTTTCGAAAGTCATGGTCCAC	60	CCATGATGGTGGTGGTCATA	60	185	--	--	--	--	--	--
TR571|c0_g1_i1	No	(AT)9	18	--	--	--	--	--	--	AAGTTGGACGTGATTTTGCC	60	TTCCACTTATTTTCAGGCGG	60	206	--	--	--	--	--	--
TR571|c0_g1_i10	No	(AT)9	18	--	--	--	--	--	--	AAGTTGGACGTGATTTTGCC	60	TTCCACTTATTTTCAGGCGG	60	206	--	--	--	--	--	--
TR571|c0_g1_i2	No	(AT)9	18	--	--	--	--	--	--	AAGTTGGACGTGATTTTGCC	60	TTCCACTTATTTTCAGGCGG	60	206	--	--	--	--	--	--
TR571|c0_g1_i3	No	(AT)9	18	--	--	--	--	--	--	AAGTTGGACGTGATTTTGCC	60	TTCCACTTATTTTCAGGCGG	60	206	--	--	--	--	--	--
TR571|c0_g1_i4	No	(AT)9	18	--	--	--	--	--	--	AAGTTGGACGTGATTTTGCC	60	TTCCACTTATTTTCAGGCGG	60	206	--	--	--	--	--	--
TR571|c0_g1_i5	No	(AT)9	18	--	--	--	--	--	--	AAGTTGGACGTGATTTTGCC	60	TTCCACTTATTTTCAGGCGG	60	206	--	--	--	--	--	--
TR571|c0_g1_i6	No	(AT)9	18	--	--	--	--	--	--	AAGTTGGACGTGATTTTGCC	60	TTCCACTTATTTTCAGGCGG	60	206	--	--	--	--	--	--

Transcript IDs	Differentially expressed gene (DEG) IDs	SSR repeat-motifs	SSR repeat-length (bp)	*In silico* polymorphic SSRs						Forward primers (5'-3')	Annealing tempearture(0C)	Reverse primers (5'-3')	Annealing tempearture(0C)	Amplified product size (bp)	Structural annotation		Functional Annotation			
				C. microphyllum		ICC4958									Gene IDs	Sequence component of genes	NCBI nr IDs	TFs	KOG	KEGG
				Transcript	SSR repeat-motifs	Chromosomes	SSR repeat-motifs	Start physical positions (bp)	End physical positions (bp)											
TR571|c0_g1_i7	XLOC_023776	(AT)8	16	--	--	--	--	--	--	AAGTTGGACGTGATTTTGCC	60	TTCCACTTATTTTCAGGCGG	60	202	--	--	--	--	--	--
TR571|c0_g1_i8	No	(AT)9	18	--	--	--	--	--	--	AAGTTGGACGTGATTTTGCC	60	TTCCACTTATTTTCAGGCGG	60	206	--	--	--	--	--	--
TR571|c0_g1_i9	XLOC_023777	(AT)9	18	--	--	--	--	--	--	AAGTTGGACGTGATTTTGCC	60	TTCCACTTATTTTCAGGCGG	60	206	--	--	--	--	--	--
TR57118|c0_g1_i1	No	(GAC)5	15	--	--	--	--	--	--	AGTGGTGGTTGAAGAGGTGG	60	TAGGCCTTCTTCATGATGCC	60	153	--	--	--	--	--	--
TR57161|c0_g1_i1	XLOC_023775	(T)10atatgaaccttaataagaa(T)10	39	--	--	--	--	--	--	TGCTAAAATTAGGAGGGTATGGA	59	TGAATAATTGATGAGTAGGCGAAA	60	157	--	--	AIH15714.1	--	--	--
TR57164|c0_g1_i1	No	(AGA)5	15	--	--	--	--	--	--	AGAAGAGGAGGAAGTTAGAAGAAGA	58	CCTGCTCCCTCTCTGCTG	60	197	--	--	--	--	--	--
TR57257|c0_g1_i1	No	(AG)6	12	--	--	--	--	--	--	ATGCTCTGTGCACTTCATGC	60	TTGCCAACCACTACACTGCT	59	265	--	--	--	--	--	--
TR57286|c0_g1_i1	XLOC_023803	(GAC)5	15	--	--	--	--	--	--	GAATCGTATGCAGGACAATTTTT	59	TGTACGAGGCTACCCCTCTG	60	104	--	--	--	--	--	--
TR57296|c0_g1_i1	No	(TGA)6	18	--	--	--	--	--	--	CGGAAAATGATTGTGGTGGT	61	ATCAGTCAAGGCCTCAGCAT	60	230	--	--	--	--	--	--
TR57346|c0_g1_i1	No	(AG)6	12	--	--	--	--	--	--	TTCTGCGTTAACCACATCCA	60	CTTCGTCTCCACCGCTTAAC	60	200	--	--	--	--	--	--
TR57363|c0_g1_i1	No	(CAA)5	15	--	--	--	--	--	--	GCGGCTTCATCTGCAAGTA	60	CATGCAAGTTCGGCTGTAGA	60	190	--	--	--	--	--	--
TR57379|c0_g1_i1	No	(TCT)5	15	--	--	--	--	--	--	TGCCTATGCCAACTTGTTCA	60	AGTTTGGCGAAGACGAAGAA	60	197	--	--	--	--	--	--
TR5741|c0_g1_i1	XLOC_023849	(ACT)6	18	--	--	--	--	--	--	TGGAAAAATTGACAGCTCAAAA	60	TGAGAAACCAGACAGAACACAAA	60	221	--	--	--	--	--	--
TR57459|c0_g1_i1	XLOC_023862	(CCG)5	15	--	--	--	--	--	--	CTGTTCCTCTTGTCGCAGGT	60	GAAAGGCTCGGACTGAGAGA	60	249	--	--	--	--	--	--

Transcript IDs	Differentially expressed gene (DEG) IDs	SSR repeat-motifs	SSR repeat-length (bp)	*In silico* polymorphic SSRs						Forward primers (5'-3')	Annealing tempearture(0C)	Reverse primers (5'-3')	Annealing tempearture(0C)	Amplified product size (bp)	Structural annotation		Functional Annotation			
				C. microphyllum		ICC4958									Gene IDs	Sequence component of genes	NCBI nr IDs	TFs	KOG	KEGG
				Transcript	SSR repeat-motifs	Chromosomes	SSR repeat-motifs	Start physical positions (bp)	End physical positions (bp)											
TR57518|c0_g1_i1	No	(AGA)5	15	--	--	--	--	--	--	CAAGATCGTGCTGATGCTGT	60	GCTGGTTGACTCGATTTGGT	60	153	--	--	--	--	--	--
TR5754|c0_g1_i1	XLOC_023880	(GA)6	12	--	--	--	--	--	--	CAGGCAACTTCGTTCTCCTC	60	CGGAATTAAGCTCCTTGCTG	60	233	--	--	--	--	--	--
TR576|c2_g1_i1	XLOC_023923	(TTA)5	15	--	--	--	--	--	--	TTAACACTTAAACAAGACAGACAATCT	57	CTCCTCATTCACCAATGCCT	60	130	--	--	XP_004492797.1	--	[T]	--
TR57631|c0_g1_i1	No	(GAG)5	15	--	--	--	--	--	--	CTTAAATCCCTCCTCTGCCC	60	TCCTCCTCCTCACCATCATC	60	184	--	--	--	--	--	--
TR57725|c0_g1_i1	XLOC_023934	(T)12caaatggattttgatttttcagcgcc(T)10	48	--	--	--	--	--	--	GCGTACACTGCAGAGACGAAT	60	AGAAGAATCATCCGCCATTG	60	168	--	--	--	--	--	--
TR57797|c0_g1_i1	XLOC_023950	(CT)6	12	--	--	--	--	--	--	AACCTTAATCCTAGGGGCGA	60	TTCATCGACTCCATCTTCACA	59	193	--	--	--	--	--	--
TR57833|c0_g1_i1	No	(CAA)6	18	--	--	--	--	--	--	ATTCCACTCCACAATCAGCC	60	AGGAGGTTTCTCCATTGCCT	60	203	--	--	--	--	--	--
TR57834|c0_g1_i1	XLOC_023960	(CAC)6	18	--	--	--	--	--	--	GAAGAGCATTGGGTGGACAT	60	TTTTTGAGGACTCGGTTTCG	60	261	--	--	--	--	--	--
TR5784|c0_g1_i1	No	(TC)7	14	TR5784|c0_g1_i1	(TC)7	Ca_LG_4	(TC)8	39604105	39604120	TTTGTAGTTGGTACTTTACTACAGCC	57	GGAGACATTGGCATCCTTGT	60	276		URR	--	--	--	--
TR5784|c0_g2_i1	No	(TC)7	14	--	--	--	--	--	--	TTTGTAGTTGGTACTTTACTACAGCC	57	GGAGACATTGGCATCCTTGT	60	276	--	--	--	--	--	--
TR57874|c0_g1_i1	No	(A)10(AC)8*	25	--	--	--	--	--	--	TGACCCAGAAAACTTTATTTGTAAAAG	60	TTTTCATTTCTCTCTGGGGTTT	59	176	--	--	--	--	--	--
TR57901|c0_g1_i1	XLOC_023987	(TGTT)5	20	--	--	--	--	--	--	GCGGGAGAGGGTAAGTCTTT	60	CACAACAATAAGAACCAACCAAA	59	250	--	--	--	--	--	--
TR58017|c0_g1_i1	No	(TG)7	14	--	--	--	--	--	--	AGACAAACATGGAGGTGGGA	60	CTTGTCTACTTGCACACACGC	60	127	--	--	--	--	--	--
TR58052|c0_g1_i1	No	(AAC)5	15	--	--	--	--	--	--	GAGGATCTCCGACGACTCAT	59	GGGGGTAGAAAACATCACCA	60	169	--	--	--	--	--	--

Transcript IDs	Differentially expressed gene (DEG) IDs	SSR repeat-motifs	SSR repeat-length (bp)	*In silico* polymorphic SSRs						Forward primers (5'-3')	Annealing tempearture(0C)	Reverse primers (5'-3')	Annealing tempearture(0C)	Amplified product size (bp)	Structural annotation		Functional Annotation			
				C. microphyllum		ICC4958									Gene IDs	Sequence component of genes	NCBI nr IDs	TFs	KOG	KEGG
				Transcript	SSR repeat-motifs	Chromosomes	SSR repeat-motifs	Start physical positions (bp)	End physical positions (bp)											
TR58078|c0_g1_i1	XLOC_024035	(CT)6	12	TR58078|c0_g1_i1	(CT)6	Ca_LG_2	(CT)7	9629293	9629306	CCATCAACAACACCATCCAA	60	TGTGGGAAGAGAGGAAATGG	60	267		URR	--	--	--	--
TR58112|c1_g1_i1	No	(CT)10	20	--	--	--	--	--	--	CTCGTGGGGTCTCGTAAAAG	60	GCCAGTTGGATGAGAGAGGA	60	153	--	--	--	--	--	--
TR5814|c0_g1_i1	No	(TTC)5	15	--	--	--	--	--	--	AACCATAATCCCCATCTCCC	60	TGGAGCTTCAAAGGAGCACT	60	279	--	--	--	--	--	--
TR5814|c0_g2_i1	No	(TTC)5	15	--	--	--	--	--	--	AACCATAATCCCCATCTCCC	60	TGGAGCTTCAAAGGAGCACT	60	279	--	--	--	--	--	--
TR58171|c0_g1_i1	No	(TGC)5tgttggtga(TGT)5	39	--	--	--	--	--	--	TCTGCAATTGATGTTGATGTTG	60	TTACCTCCACCTCGTCCATC	60	178	--	--	--	--	--	--
TR58272|c0_g1_i1	No	(TCT)5	15	--	--	--	--	--	--	TACTTCCTCACCCGAAACGA	61	CTGGAAGTGTTGTCAACGGA	60	223	--	--	--	--	--	--
TR5833|c0_g2_i1	No	(GTA)5	15	--	--	--	--	--	--	GGAATTGGGTGATTTAGCGA	60	CCACCTCTTCGTGGTTTGTT	60	140	--	--	--	--	--	--
TR58397|c0_g1_i1	No	(ATT)7	21	--	--	--	--	--	--	CCCACAATCCAAACCAAAAC	60	TGCATGTTCATGTTCCTTTGA	60	260	--	--	--	--	--	--
TR58404|c0_g1_i1	No	(TC)8	16	--	--	--	--	--	--	GCATCTGCTTACTGCCATCA	60	GGGGCTTCTTGTTTAGGGTT	59	209	--	--	--	--	--	--
TR58404|c0_g1_i2	No	(TC)8	16	--	--	--	--	--	--	GCATCTGCTTACTGCCATCA	60	GGGGCTTCTTGTTTAGGGTT	59	209	--	--	--	--	--	--
TR58404|c0_g2_i1	No	(TC)17	34	--	--	--	--	--	--	ATGGGGAGAATTGGTTTGAA	59	TGAAACCTCTCCAACTCGCT	60	165	--	--	--	--	--	--
TR58419|c0_g2_i1	No	(CAA)5	15	--	--	--	--	--	--	CAACACCCCTCTCACTTTCC	60	AAGCCCAAGGTTATTTGCCT	60	263	--	--	--	--	--	--
TR58419|c0_g2_i1	No	(AT)9	18	--	--	--	--	--	--	CCATGACCCGTGATCTTCTT	60	AATGGTTGAGATTTGAGAGTGC	58	270	--	--	--	--	--	--
TR58428|c0_g1_i1	No	(ACT)5	15	--	--	--	--	--	--	ATGCAAGGTGAGCTCAGACA	60	CAAAAGACCAAGCAATGGGT	60	251	--	--	--	--	--	--

<table>
<tr><th rowspan="3">Transcript IDs</th><th rowspan="3">Differentially expressed gene (DEG) IDs</th><th rowspan="3">SSR repeat-motifs</th><th rowspan="3">SSR repeat-length (bp)</th><th colspan="6">In silico polymorphic SSRs</th><th rowspan="3">Forward primers (5'-3')</th><th rowspan="3">Annealing tempearture(0C)</th><th rowspan="3">Reverse primers (5'-3')</th><th rowspan="3">Annealing tempearture(0C)</th><th rowspan="3">Amplified product size (bp)</th><th colspan="2">Structural annotation</th><th colspan="4">Functional Annotation</th></tr>
<tr><th colspan="2">C. microphyllum</th><th colspan="4">ICC4958</th><th rowspan="2">Gene IDs</th><th rowspan="2">Sequence component of genes</th><th rowspan="2">NCBI nr IDs</th><th rowspan="2">TFs</th><th rowspan="2">KOG</th><th rowspan="2">KEGG</th></tr>
<tr><th>Transcript</th><th>SSR repeat-motifs</th><th>Chromosomes</th><th>SSR repeat-motifs</th><th>Start physical positions (bp)</th><th>End physical positions (bp)</th></tr>
<tr><td>TR58428|c0_g1_i2</td><td>No</td><td>(ACT)5</td><td>15</td><td>--</td><td>--</td><td>--</td><td>--</td><td>--</td><td>--</td><td>ATGCAAGGTGAGCTCAGACA</td><td>60</td><td>CAAAAGACCAAGCAATGGGT</td><td>60</td><td>251</td><td>--</td><td>--</td><td>--</td><td>--</td><td>--</td><td>--</td></tr>
<tr><td>TR58429|c0_g1_i1</td><td>No</td><td>(TCT)5</td><td>15</td><td>--</td><td>--</td><td>--</td><td>--</td><td>--</td><td>--</td><td>TGAATCTTCTTCCGCTGCTT</td><td>60</td><td>CCCTTGAGAATGACTCCTGC</td><td>60</td><td>260</td><td>--</td><td>--</td><td>--</td><td>--</td><td>--</td><td>--</td></tr>
<tr><td>TR58429|c0_g1_i2</td><td>No</td><td>(TCT)5</td><td>15</td><td>--</td><td>--</td><td>--</td><td>--</td><td>--</td><td>--</td><td>TGAATCTTCTTCCGCTGCTT</td><td>60</td><td>CCCTTGAGAATGACTCCTGC</td><td>60</td><td>260</td><td>--</td><td>--</td><td>--</td><td>--</td><td>--</td><td>--</td></tr>
<tr><td>TR58432|c1_g1_i1</td><td>No</td><td>(A)10caccattttcgtgacgcaacagattcattcttttatttcaacc(A)11</td><td>64</td><td>--</td><td>--</td><td>--</td><td>--</td><td>--</td><td>--</td><td>TCCATAGGAGGAAATGCTGG</td><td>60</td><td>AGGTAATCGTAATCGGCACG</td><td>60</td><td>247</td><td>--</td><td>--</td><td>--</td><td>--</td><td>--</td><td>--</td></tr>
<tr><td>TR58442|c0_g1_i1</td><td>XLOC_024151</td><td>(GTT)5</td><td>15</td><td>--</td><td>--</td><td>--</td><td>--</td><td>--</td><td>--</td><td>TGCAAAGAGATGAGAAGGGTG</td><td>60</td><td>TGTGGCCATTGCAATATCAT</td><td>60</td><td>200</td><td>--</td><td>--</td><td>XM_004490251.1</td><td>--</td><td>[I]</td><td>K13356</td></tr>
<tr><td>TR58458|c0_g1_i1</td><td>XLOC_024171</td><td>(AT)9</td><td>18</td><td>--</td><td>--</td><td>--</td><td>--</td><td>--</td><td>--</td><td>AAGGAGTTGTCTCCAATTCACA</td><td>59</td><td>AGCGGGAGGAGGAGATAGAG</td><td>60</td><td>226</td><td>--</td><td>--</td><td>--</td><td>--</td><td>--</td><td>--</td></tr>
<tr><td>TR58479|c1_g1_i1</td><td>XLOC_024195</td><td>(TA)8</td><td>16</td><td>--</td><td>--</td><td>--</td><td>--</td><td>--</td><td>--</td><td>TGGGGTTGAAAAAGTGGAAG</td><td>60</td><td>TGATGGAAGAAATAGAATAAGTTAGCA</td><td>59</td><td>225</td><td>--</td><td>--</td><td>--</td><td>--</td><td>--</td><td>--</td></tr>
<tr><td>TR5854|c0_g1_i1</td><td>XLOC_024228</td><td>(TCA)5</td><td>15</td><td>--</td><td>--</td><td>--</td><td>--</td><td>--</td><td>--</td><td>AACGAGACGCTTATCGGAGA</td><td>60</td><td>GATGCTTCCGTTACTGAGGG</td><td>60</td><td>253</td><td>--</td><td>--</td><td>XP_003048236.1</td><td>--</td><td>--</td><td>--</td></tr>
<tr><td>TR58603|c0_g1_i1</td><td>No</td><td>(ATA)6</td><td>18</td><td>--</td><td>--</td><td>--</td><td>--</td><td>--</td><td>--</td><td>GAGTTGTTAGAATCTTGTGCATGT</td><td>58</td><td>TGACTAATTCAACATAAACCAAACA</td><td>58</td><td>214</td><td>--</td><td>--</td><td>--</td><td>--</td><td>--</td><td>--</td></tr>
<tr><td>TR58616|c0_g1_i1</td><td>No</td><td>(TA)7</td><td>14</td><td>--</td><td>--</td><td>--</td><td>--</td><td>--</td><td>--</td><td>TAGGAACACACCACCCAAGG</td><td>61</td><td>GATTGGGGTCAAAGAGCAAA</td><td>60</td><td>262</td><td>--</td><td>--</td><td>--</td><td>--</td><td>--</td><td>--</td></tr>
<tr><td>TR58655|c0_g1_i1</td><td>No</td><td>(GA)6</td><td>12</td><td>--</td><td>--</td><td>--</td><td>--</td><td>--</td><td>--</td><td>AAGCAATTCGCCAAAGTTGT</td><td>60</td><td>AAGAGCACGCTTGATGGAAT</td><td>60</td><td>240</td><td>--</td><td>--</td><td>--</td><td>--</td><td>--</td><td>--</td></tr>
<tr><td>TR58768|c0_g2_i1</td><td>No</td><td>(GAG)5</td><td>15</td><td>--</td><td>--</td><td>--</td><td>--</td><td>--</td><td>--</td><td>GGAGGGAGTAGGAGAAACGG</td><td>60</td><td>ACCATGCTTTCTCGCAAACT</td><td>60</td><td>235</td><td>--</td><td>--</td><td>--</td><td>--</td><td>--</td><td>--</td></tr>
<tr><td>TR58775|c0_g1_i1</td><td>No</td><td>(CTT)7cctccactcgcaggcacagtcactttcacagacacagtatcaatatcatagccgccaaaagcaagaagcagcatgatgcggctcagggttctgaacg(ACC)5</td><td>133</td><td>--</td><td>--</td><td>--</td><td>--</td><td>--</td><td>--</td><td>TTCCATCAACATCCCATTCA</td><td>60</td><td>CCATATTTTGAGGAAGCGGA</td><td>60</td><td>239</td><td>--</td><td>--</td><td>--</td><td>--</td><td>--</td><td>--</td></tr>
<tr><td>TR58775|c0_g1_i3</td><td>No</td><td>(CTT)7cctccactcgcaggcacagtcactttcacagacacagtatcaatatcatagccgccaaaagcaagaagcagcatgatgcggctcagggttctgaacg(ACC)5</td><td>133</td><td>--</td><td>--</td><td>--</td><td>--</td><td>--</td><td>--</td><td>TTCCATCAACATCCCATTCA</td><td>60</td><td>CCATATTTTGAGGAAGCGGA</td><td>60</td><td>239</td><td>--</td><td>--</td><td>--</td><td>--</td><td>--</td><td>--</td></tr>
</table>

Transcript IDs	Differentially expressed gene (DEG) IDs	SSR repeat-motifs	SSR repeat-length (bp)	*In silico* polymorphic SSRs						Forward primers (5'-3')	Annealing tempearture(0C)	Reverse primers (5'-3')	Annealing tempearture(0C)	Amplified product size (bp)	Structural annotation		Functional Annotation			
				C. microphyllum		ICC4958														
				Transcript	SSR repeat-motifs	Chromosomes	SSR repeat-motifs	Start physical positions (bp)	End physical positions (bp)						Gene IDs	Sequence component of genes	NCBI nr IDs	TFs	KOG	KEGG
TR58775|c0_g1_i5	No	(CTT)7cctccactcgcaggcacagtcactttcacagacacagtatcaatatcatagccgccaaaagcaagaagcagcatgatgcggctcagggttctgaacg(ACC)5	133	--	--	--	--	--	--	TTCCATCAACATCCCATTCA	60	CCATATTTTGAGGAAGCGGA	60	239	--	--	--	--	--	--
TR58799|c0_g2_i1	XLOC_024332	(TC)6	12	--	--	--	--	--	--	CGAAAACTTCAATCTCACCAG	57	ATCGGTTCTGGGATCAGATG	60	275	--	--	--	--	--	--
TR58799|c0_g3_i1	No	(TC)6	12	--	--	--	--	--	--	CGAAAACTTCAATCTCACCAG	57	ATCGGTTCTGGGATCAGATG	60	275	--	--	--	--	--	--
TR58811|c0_g1_i1	No	(AAG)6	18	--	--	--	--	--	--	GTAAATGGCGTCATTGGGTC	60	CCAGAGAAATCAGGGAGCTG	60	175	--	--	--	--	--	--
TR58829|c2_g1_i1	No	(CACAA)5	25	--	--	--	--	--	--	CCGTCGCGAACTGTATCTCT	60	TGAGGTTTGGAAATTAGAAGAAAA	58	224	--	--	--	--	--	--
TR58829|c2_g1_i1	No	(CTCA)5	20	--	--	--	--	--	--	CGGTTTTCACTCCAAAGTCC	60	TTGTGTTGTGGAGTGAGGGA	60	202	--	--	--	--	--	--
TR58831|c1_g2_i1	XLOC_024366	(CTT)5	15	--	--	--	--	--	--	CCAATTCATCGATCCAAACC	60	CCCTTTGAAGATGGCAACTC	60	180	--	--	--	--	--	--
TR58831|c1_g2_i1	XLOC_024366	(TC)9	18	--	--	--	--	--	--	TTACTACCAGAGAACACCATCATC	57	CGGAATGGTTTAGGTTTGGA	60	128	--	--	--	--	--	--
TR58831|c2_g1_i1	XLOC_024367	(AGA)7	21	--	--	--	--	--	--	TTGTGGATTTGCTACGCTTG	60	CGTGACCATCACATTCGTTC	60	127	--	--	--	--	--	--
TR58831|c2_g2_i1	No	(AGA)7	21	--	--	--	--	--	--	TTGTGGATTTGCTACGCTTG	60	CGTGACCATCACATTCGTTC	60	127	--	--	--	--	--	--
TR58831|c3_g1_i1	XLOC_024368	(TCT)5	15	--	--	--	--	--	--	CTGGTGGGATCTGTGCTTTT	60	TATGGTTGTTGTGGGTGGTG	60	233	--	--	--	--	--	--
TR58831|c5_g1_i1	No	(TTC)7tgctgttcgggttactcttgttcttttgggtcctccaccacatccaccgcctctggtcaccattaccagaccagtcat(TC)9	117	--	--	--	--	--	--	CGCTACAAGTGAAAGTTGGTTC	59	TTGAATGCAACCCAACAAAA	60	230	--	--	--	--	--	--
TR58831|c6_g1_i1	XLOC_024372	(AAT)6	18	--	--	--	--	--	--	AGGATGATGATGAACGGAGG	60	ACAACAACAAAACACCGCAC	60	277	--	--	--	--	--	--
TR58840|c0_g2_i1	No	(TC)22	44	--	--	--	--	--	--	TTTGCCACCAATCACAAAAA	60	CGGACTAAATCGGAACTGGA	60	201	--	--	--	--	--	--

Transcript IDs	Differentially expressed gene (DEG) IDs	SSR repeat-motifs	SSR repeat-length (bp)	*In silico* polymorphic SSRs						Forward primers (5'-3')	Annealing tempearture(0C)	Reverse primers (5'-3')	Annealing tempearture(0C)	Amplified product size (bp)	Structural annotation		Functional Annotation			
				C. microphyllum		ICC4958									Gene IDs	Sequence component of genes	NCBI nr IDs	TFs	KOG	KEGG
				Transcript	SSR repeat-motifs	Chromosomes	SSR repeat-motifs	Start physical positions (bp)	End physical positions (bp)											
TR58840|c0_g2_i2	No	(TC)22	44	--	--	--	--	--	--	TTTGCCACCAATCACAAAAA	60	CGGACTAAATCGGAACTGGA	60	201	--	--	--	--	--	--
TR58840|c0_g2_i3	No	(TC)22	44	--	--	--	--	--	--	TTTGCCACCAATCACAAAAA	60	CGGACTAAATCGGAACTGGA	60	201	--	--	--	--	--	--
TR58840|c1_g1_i1	XLOC_024383	(TC)6	12	--	--	--	--	--	--	CTCGGTCCCTAATCCCTTTC	60	TGGTAGCCTAGGAGTGGTGG	60	101	--	--	--	--	--	--
TR58912|c0_g1_i1	XLOC_024414	(GT)6	12	--	--	--	--	--	--	CTCATTCCTTGGGATGGAGA	60	GTTTCTGAAATTCGGCTTGC	60	183	--	--	--	--	--	--
TR58913|c0_g1_i1	No	(GAA)5	15	--	--	--	--	--	--	CGACGCATACGAAAAAGACA	60	AACAGCAGCAGGACCAAAAT	60	108	--	--	--	--	--	--
TR59048|c0_g1_i1	XLOC_024450	(TCT)6	18	--	--	--	--	--	--	CCACTCTTCTCCTTAGCTCATCA	60	AAAAGCAAGAAACCAAAACCAA	60	213	--	--	XP_006599642.1	--	--	--
TR59105|c0_g1_i1	XLOC_024476	(AAC)5	15	--	--	--	--	--	--	TCATCATCTAGCTCTCACCAACA	60	GGCAAAACCAAACTTGAAGC	60	111	--	--	--	--	--	--
TR59113|c0_g1_i1	No	(TTC)7	21	--	--	--	--	--	--	ATTTCGAAGCGGAAGAAACA	60	CAAGAATAGAAACATGCCTTATGTG	59	119	--	--	--	--	--	--
TR59171|c0_g1_i1	No	(TC)6	12	--	--	--	--	--	--	CAGCGCTCTTACTCAAACACA	59	ACCGGACGTCGATCTCTGTA	61	199	--	--	--	--	--	--
TR59304|c0_g1_i1	No	(TGG)5	15	--	--	--	--	--	--	CATTTCTCCGGCTCCTACAC	60	CACAGCATTACCCTCCACCT	60	224	--	--	--	--	--	--
TR59316|c0_g1_i1	No	(AAG)5	15	--	--	--	--	--	--	GACCTAGAACCCGAACCACA	60	TTCGGCGTCTTAATTATCGG	60	236	--	--	--	--	--	--
TR59316|c0_g2_i1	No	(AAG)5	15	--	--	--	--	--	--	GACCTAGAACCCGAACCACA	60	TTCGGCGTCTTAATTATCGG	60	236	--	--	--	--	--	--
TR59324|c1_g1_i1	No	(AC)6	12	--	--	--	--	--	--	CGCTTTCCCTGTACCTTCTTT	60	CTTCTTTTGCACCGGTGTGT	61	271	--	--	--	--	--	--
TR59324|c1_g2_i1	XLOC_024516	(AC)6	12	--	--	--	--	--	--	CGCTTTCCCTGTACCTTCTTT	60	CTTCTTTTGCACCGGTGTGT	61	271	--	--	--	--	--	--

Transcript IDs	Differentially expressed gene (DEG) IDs	SSR repeat-motifs	SSR repeat-length (bp)	*In silico* polymorphic SSRs						Forward primers (5'-3')	Annealing tempearture(0C)	Reverse primers (5'-3')	Annealing tempearture(0C)	Amplified product size (bp)	Structural annotation		Functional Annotation			
				C. microphyllum		ICC4958									Gene IDs	Sequence component of genes	NCBI nr IDs	TFs	KOG	KEGG
				Transcript	SSR repeat-motifs	Chromosomes	SSR repeat-motifs	Start physical positions (bp)	End physical positions (bp)											
TR59341|c0_g3_i2	No	(TCA)7	21	--	--	--	--	--	--	AAAAGTGCGAGGGAGAGTGA	60	CACGCAAAAACTCCGTATGA	60	228	--	--	--	--	--	--
TR59377|c0_g1_i1	XLOC_024550	(AAG)7	21	--	--	--	--	--	--	TTCCAAAACCTCATGGCTTC	60	CGGAAGCATCAACAACCTCT	60	232	--	--	--	--	--	--
TR59400|c0_g1_i1	No	(ACC)5	15	--	--	--	--	--	--	GCTAACCCCGAAATTCACAA	60	TGGAGGGATTCTTTGATTGC	60	274	--	--	--	--	--	--
TR59400|c0_g2_i1	No	(ACC)5	15	--	--	--	--	--	--	GCTAACCCCGAAATTCACAA	60	TGGAGGGATTCTTTGATTGC	60	275	--	--	--	--	--	--
TR59401|c0_g1_i1	XLOC_024565	(TC)8	16	--	--	--	--	--	--	TTCAATAGAAATCAACCAAACAGA A	59	GTGCCACTTGCAGTCAGAAA	60	219	--	--	--	--	--	--
TR59404|c1_g1_i1	XLOC_024570	(CT)6	12	TR59404|c1_g1_i1	(CT)6	Ca_LG_5	(CT)7	14634357	14634370	AACCATCAGCAAAGAACAGTGA	60	GCTGCAGCAGATGCTATCAA	60	217	Ca_04320	CDS	--	--	--	--
TR59413|c0_g1_i2	XLOC_024582	(AT)7	14	--	--	--	--	--	--	GTCCAAAGAAAATACCGGGG	61	AAAGAGCGACACTCCGAAAA	60	278	--	--	--	--	--	--
TR59415|c0_g2_i1	XLOC_024588	(CAT)5	15	--	--	--	--	--	--	CCGAACTTTAGCAACTGCAAC	60	TTGGTTTGATGAGGAGAGGG	60	208	--	--	--	--	--	--
TR59415|c0_g3_i1	No	(CAT)5	15	--	--	--	--	--	--	CCGAACTTTAGCAACTGCAAC	60	TTGGTTTGATGAGGAGAGGG	60	208	--	--	--	--	--	--
TR59419|c4_g1_i1	No	(AAAT)5	20	--	--	--	--	--	--	TTGGCCATGACTATAATCCCA	60	TTGGTTGTTCCTCGATTTCC	60	154	--	--	--	--	--	--
TR59419|c4_g1_i2	No	(AAAT)5	20	--	--	--	--	--	--	TTGGCCATGACTATAATCCCA	60	TTGGTTGTTCCTCGATTTCC	60	154	--	--	--	--	--	--
TR59419|c4_g1_i3	No	(AAAT)5	20	--	--	--	--	--	--	TTGGCCATGACTATAATCCCA	60	TTGGTTGTTCCTCGATTTCC	60	154	--	--	--	--	--	--
TR59429|c1_g2_i1	XLOC_024624	(GTC)5	15	--	--	--	--	--	--	GCCGAAGAAAGGAAAATTGA	59	ACTCTCCGATGCATCAGCTT	60	251	--	--	--	--	--	--
TR59435|c3_g1_i1	No	(TTA)6	18	--	--	--	--	--	--	TTCCCAACCTCTTTTCCCTT	60	GGAACCCAGCTTCGGATATT	60	277	--	--	--	--	--	--

Transcript IDs	Differentially expressed gene (DEG) IDs	SSR repeat-motifs	SSR repeat-length (bp)	*In silico* polymorphic SSRs						Forward primers (5'-3')	Annealing tempearture(0C)	Reverse primers (5'-3')	Annealing tempearture(0C)	Amplified product size (bp)	Structural annotation		Functional Annotation			
				C. microphyllum		ICC4958									Gene IDs	Sequence component of genes	NCBI nr IDs	TFs	KOG	KEGG
				Transcript	SSR repeat-motifs	Chromosomes	SSR repeat-motifs	Start physical positions (bp)	End physical positions (bp)											
TR59435|c3_g1_i3	No	(TTA)6	18	--	--	--	--	--	--	TTCCCAACCTCTTTTCCCTT	60	GGAACCCAGCTTCGGATATT	60	277	--	--	--	--	--	--
TR59435|c3_g1_i7	No	(TTA)6	18	--	--	--	--	--	--	TTCCCAACCTCTTTTCCCTT	60	GGAACCCAGCTTCGGATATT	60	277	--	--	--	--	--	--
TR59435|c3_g3_i1	No	(TTA)6	18	--	--	--	--	--	--	TTCCCAACCTCTTTTCCCTT	60	GGAACCCAGCTTCGGATATT	60	277	--	--	--	--	--	--
TR59435|c3_g3_i2	XLOC_024646	(TTA)6	18	--	--	--	--	--	--	TTCCCAACCTCTTTTCCCTT	60	GGAACCCAGCTTCGGATATT	60	277	--	--	--	--	--	--
TR59435|c4_g2_i3	No	(AGC)5	15	--	--	--	--	--	--	ATGGAGCAATGGCAGGTTAC	60	TGAACAATGCTGGTCCTACTG	59	242	--	--	--	--	--	--
TR59437|c0_g1_i1	No	(CT)6	12	--	--	--	--	--	--	TATCATATGGCCCATCACCA	60	GCAACAATTTTGGTGTTGCTT	60	179	--	--	--	--	--	--
TR59437|c0_g1_i10	No	(CT)6	12	--	--	--	--	--	--	TATCATATGGCCCATCACCA	60	GCAACAATTTTGGTGTTGCTT	60	168	--	--	--	--	--	--
TR59437|c0_g1_i2	No	(CT)6	12	--	--	--	--	--	--	TATCATATGGCCCATCACCA	60	GCAACAATTTTGGTGTTGCTT	60	179	--	--	--	--	--	--
TR59437|c0_g1_i3	No	(CT)6	12	--	--	--	--	--	--	TATCATATGGCCCATCACCA	60	GCAACAATTTTGGTGTTGCTT	60	168	--	--	--	--	--	--
TR59437|c0_g1_i4	No	(CT)6	12	--	--	--	--	--	--	TATCATATGGCCCATCACCA	60	GCAACAATTTTGGTGTTGCTT	60	179	--	--	--	--	--	--
TR59437|c0_g1_i5	No	(CT)6	12	--	--	--	--	--	--	TATCATATGGCCCATCACCA	60	GCAACAATTTTGGTGTTGCTT	60	179	--	--	--	--	--	--
TR59437|c0_g1_i6	No	(CT)6	12	--	--	--	--	--	--	TATCATATGGCCCATCACCA	60	GCAACAATTTTGGTGTTGCTT	60	168	--	--	--	--	--	--
TR59437|c0_g1_i7	No	(CT)6	12	--	--	--	--	--	--	TATCATATGGCCCATCACCA	60	GCAACAATTTTGGTGTTGCTT	60	179	--	--	--	--	--	--
TR59437|c0_g1_i8	No	(CT)6	12	--	--	--	--	--	--	TATCATATGGCCCATCACCA	60	GCAACAATTTTGGTGTTGCTT	60	179	--	--	--	--	--	--

Transcript IDs	Differentially expressed gene (DEG) IDs	SSR repeat-motifs	SSR repeat-length (bp)	*In silico* polymorphic SSRs						Forward primers (5'-3')	Annealing tempearture(0C)	Reverse primers (5'-3')	Annealing tempearture(0C)	Amplified product size (bp)	Structural annotation		Functional Annotation			
				C. microphyllum		ICC4958														
				Transcript	SSR repeat-motifs	Chromosomes	SSR repeat-motifs	Start physical positions (bp)	End physical positions (bp)						Gene IDs	Sequence component of genes	NCBI nr IDs	TFs	KOG	KEGG
TR59437|c0_g1_i9	No	(CT)6	12	--	--	--	--	--	--	TATCATATGGCCCATCACCA	60	GCAACAATTTTGGTGTTGCTT	60	179	--	--	--	--	--	--
TR59444|c1_g1_i1	No	(TTC)6	18	--	--	--	--	--	--	CTGCAAATTTGTGAACGTGG	60	CCTCGAGATCCAAATCGAAA	60	258	--	--	--	--	--	--
TR59444|c1_g1_i2	No	(CT)9	18	--	--	--	--	--	--	AAATCAAGGGCTTTCCCCT	60	ACAGTTGAACACGCCAACAA	60	270	--	--	--	--	--	--
TR59444|c1_g1_i3	No	(TTC)6	18	--	--	--	--	--	--	CTGCAAATTTGTGAACGTGG	60	CCTCGAGATCCAAATCGAAA	60	258	--	--	--	--	--	--
TR59444|c1_g1_i4	No	(CT)9	18	--	--	--	--	--	--	AAATCAAGGGCTTTCCCCT	60	ACAGTTGAACACGCCAACAA	60	270	--	--	--	--	--	--
TR59456|c1_g1_i1	No	(TCT)6	18	--	--	--	--	--	--	CCGTTGCAAGAATGAAACCT	60	TTCCTTCGCAAGTGACCTCT	60	265	--	--	--	--	--	--
TR59456|c1_g1_i10	No	(TCT)6	18	--	--	--	--	--	--	CCGTTGCAAGAATGAAACCT	60	TTCCTTCGCAAGTGACCTCT	60	265	--	--	--	--	--	--
TR59456|c1_g1_i13	No	(TCT)6	18	--	--	--	--	--	--	CCGTTGCAAGAATGAAACCT	60	TTCCTTCGCAAGTGACCTCT	60	264	--	--	--	--	--	--
TR59456|c1_g1_i14	No	(TCT)6	18	--	--	--	--	--	--	CCGTTGCAAGAATGAAACCT	60	TTCCTTCGCAAGTGACCTCT	60	264	--	--	--	--	--	--
TR59456|c1_g1_i15	No	(TCT)6	18	--	--	--	--	--	--	CCGTTGCAAGAATGAAACCT	60	TTCCTTCGCAAGTGACCTCT	60	264	--	--	--	--	--	--
TR59456|c1_g1_i5	No	(TCT)6	18	--	--	--	--	--	--	CCGTTGCAAGAATGAAACCT	60	TTCCTTCGCAAGTGACCTCT	60	264	--	--	--	--	--	--
TR59456|c1_g1_i6	No	(TCT)6	18	--	--	--	--	--	--	CCGTTGCAAGAATGAAACCT	60	TTCCTTCGCAAGTGACCTCT	60	265	--	--	--	--	--	--
TR59456|c1_g1_i7	No	(TCT)6	18	--	--	--	--	--	--	CCGTTGCAAGAATGAAACCT	60	TTCCTTCGCAAGTGACCTCT	60	264	--	--	--	--	--	--
TR59456|c1_g1_i8	No	(TCT)6	18	--	--	--	--	--	--	CCGTTGCAAGAATGAAACCT	60	TTCCTTCGCAAGTGACCTCT	60	264	--	--	--	--	--	--

Transcript IDs	Differentially expressed gene (DEG) IDs	SSR repeat-motifs	SSR repeat-length (bp)	*In silico* polymorphic SSRs						Forward primers (5'-3')	Annealing tempearture(0C)	Reverse primers (5'-3')	Annealing tempearture(0C)	Amplified product size (bp)	Structural annotation		Functional Annotation			
				C. microphyllum		ICC4958									Gene IDs	Sequence component of genes	NCBI nr IDs	TFs	KOG	KEGG
				Transcript	SSR repeat-motifs	Chromosomes	SSR repeat-motifs	Start physical positions (bp)	End physical positions (bp)											
TR59461|c2_g2_i6	XLOC_024701	(GA)8	16	--	--	--	--	--	--	TGCAATTCCAATTTTCTCTGC	60	ACTGCAGCATCAACAGCATT	59	238	--	--	AAP34633.1	Dof	[Z]	--
TR59461|c2_g5_i1	No	(GA)8	16	--	--	--	--	--	--	TGCAATTCCAATTTTCTCTGC	60	ACTGCAGCATCAACAGCATT	59	238	--	--	--	--	--	--
TR59465|c0_g1_i1	No	(TC)11	22	--	--	--	--	--	--	ATACTTGCGCCTCTCTCCAA	60	GCAGCTAATGTGTGAACGGA	60	180	--	--	--	--	--	--
TR59465|c0_g2_i1	No	(TC)11	22	--	--	--	--	--	--	ATACTTGCGCCTCTCTCCAA	60	GGTCACCACCACTTCACCTT	60	234	--	--	--	--	--	--
TR59465|c2_g2_i1	No	(A)13tt(A)10	25	--	--	--	--	--	--	GTCCATGATTAAGGGAGGCA	60	TCAACTATTGCAATGCGGTC	60	185	--	--	--	--	--	--
TR59465|c2_g2_i2	No	(A)13tt(A)10	25	--	--	--	--	--	--	GTCCATGATTAAGGGAGGCA	60	TCAACTATTGCAATGCGGTC	60	185	--	--	--	--	--	--
TR59465|c2_g2_i3	No	(A)13tt(A)10	25	--	--	--	--	--	--	GTCCATGATTAAGGGAGGCA	60	TCAACTATTGCAATGCGGTC	60	185	--	--	--	--	--	--
TR59465|c2_g2_i4	No	(A)13tt(A)10	25	--	--	--	--	--	--	GTCCATGATTAAGGGAGGCA	60	TCAACTATTGCAATGCGGTC	60	185	--	--	--	--	--	--
TR59465|c2_g2_i5	No	(A)13tt(A)10	25	--	--	--	--	--	--	GTCCATGATTAAGGGAGGCA	60	TCAACTATTGCAATGCGGTC	60	185	--	--	--	--	--	--
TR59465|c2_g2_i6	No	(A)13tt(A)10	25	--	--	--	--	--	--	GTCCATGATTAAGGGAGGCA	60	TCAACTATTGCAATGCGGTC	60	185	--	--	--	--	--	--
TR59465|c2_g2_i7	No	(A)13tt(A)10	25	--	--	--	--	--	--	GTCCATGATTAAGGGAGGCA	60	TCAACTATTGCAATGCGGTC	60	185	--	--	--	--	--	--
TR59465|c2_g2_i8	No	(A)13tt(A)10	25	--	--	--	--	--	--	GTCCATGATTAAGGGAGGCA	60	TCAACTATTGCAATGCGGTC	60	185	--	--	--	--	--	--
TR59466|c1_g1_i1	No	(TTC)7	21	--	--	--	--	--	--	AACAAACACCATCGCGACTT	61	AAACGACTCGGGGACTTCTT	60	213	--	--	--	--	--	--
TR59466|c2_g1_i1	XLOC_024712	(TTC)7	21	--	--	--	--	--	--	TCAGAGCACAGTTTTGTGTCA	58	CTTTTGGCTGCTCAAGGTTC	60	188	--	--	XP_004511602.1	B3	[R]	--

Transcript IDs	Differentially expressed gene (DEG) IDs	SSR repeat-motifs	SSR repeat-length (bp)	*In silico* polymorphic SSRs						Forward primers (5'-3')	Annealing tempearture(0C)	Reverse primers (5'-3')	Annealing tempearture(0C)	Amplified product size (bp)	Structural annotation		Functional Annotation			
				C. microphyllum		ICC4958									Gene IDs	Sequence component of genes	NCBI nr IDs	TFs	KOG	KEGG
				Transcript	SSR repeat-motifs	Chromosomes	SSR repeat-motifs	Start physical positions (bp)	End physical positions (bp)											
TR59466|c2_g2_i1	XLOC_024713	(CAT)8	24	--	--	--	--	--	--	TGGATCTGTTTCAAGTGAAGATG	59	GCTTGGGTTACTTGCAGCTC	60	268	--	--	--	--	--	--
TR59466|c4_g1_i1	No	(TTC)7	21	--	--	--	--	--	--	ACAACAGCAACAACGCATTT	59	AACAGTGTCCACCACCGAAT	60	106	--	--	--	--	--	--
TR59466|c4_g1_i2	No	(TTC)7	21	--	--	--	--	--	--	ACAACAGCAACAACGCATTT	59	AACAGTGTCCACCACCGAAT	60	106	--	--	--	--	--	--
TR59466|c4_g1_i3	No	(TTC)7	21	--	--	--	--	--	--	ACAACAGCAACAACGCATTT	59	AACAGTGTCCACCACCGAAT	60	106	--	--	--	--	--	--
TR59466|c4_g1_i4	No	(TTC)7	21	--	--	--	--	--	--	ACAACAGCAACAACGCATTT	59	AACAGTGTCCACCACCGAAT	60	106	--	--	--	--	--	--
TR59466|c4_g1_i5	No	(TTC)7	21	--	--	--	--	--	--	ACAACAGCAACAACGCATTT	59	AACAGTGTCCACCACCGAAT	60	106	--	--	--	--	--	--
TR59466|c4_g1_i6	No	(TTC)7	21	--	--	--	--	--	--	ACAACAGCAACAACGCATTT	59	AACAGTGTCCACCACCGAAT	60	106	--	--	--	--	--	--
TR59466|c8_g1_i1	XLOC_024718	(CT)6	12	--	--	--	--	--	--	GAAAGCAATGGAAAAACGGA	60	GGAACAAGCAAAACCAAACC	59	132	--	--	--	--	--	--
TR59468|c1_g1_i1	No	(TC)9	18	--	--	--	--	--	--	CCAAATTTCAGGTAGTGGTACTGTC	60	ACTGGTGGCAAAAGTAACGG	60	190	--	--	--	--	--	--
TR59468|c1_g1_i2	No	(TC)9	18	--	--	--	--	--	--	CCAAATTTCAGGTAGTGGTACTGTC	60	ACTGGTGGCAAAAGTAACGG	60	190	--	--	--	--	--	--
TR59468|c1_g1_i3	No	(TC)9	18	--	--	--	--	--	--	CCAAATTTCAGGTAGTGGTACTGTC	60	ACTGGTGGCAAAAGTAACGG	60	190	--	--	--	--	--	--
TR59468|c1_g1_i4	No	(TC)9	18	--	--	--	--	--	--	CCAAATTTCAGGTAGTGGTACTGTC	60	ACTGGTGGCAAAAGTAACGG	60	190	--	--	--	--	--	--
TR59468|c1_g2_i1	No	(TC)9	18	--	--	--	--	--	--	CCAAATTTCAGGTAGTGGTACTGTC	60	ACTGGTGGCAAAAGTAACGG	60	190	--	--	--	--	--	--
TR59468|c2_g1_i1	XLOC_024720	(TC)10	20	--	--	--	--	--	--	CTTGCACAACACTTTCCCCT	60	TAAAAGGAGCCGGTGAAGAA	60	217	--	--	--	--	--	--

Transcript IDs	Differentially expressed gene (DEG) IDs	SSR repeat-motifs	SSR repeat-length (bp)	*In silico* polymorphic SSRs						Forward primers (5'-3')	Annealing tempearture(0C)	Reverse primers (5'-3')	Annealing tempearture(0C)	Amplified product size (bp)	Structural annotation		Functional Annotation			
				C. microphyllum		ICC4958									Gene IDs	Sequence component of genes	NCBI nr IDs	TFs	KOG	KEGG
				Transcript	SSR repeat-motifs	Chromosomes	SSR repeat-motifs	Start physical positions (bp)	End physical positions (bp)											
TR59474|c0_g1_i1	XLOC_024731	(CT)6	12	--	--	--	--	--	--	CCAAACCAAAACATTGTTCTCT	58	CCACCATTTTGCATTCCTTT	60	229	--	--	--	--	--	--
TR59474|c0_g1_i2	No	(CT)6	12	--	--	--	--	--	--	CCAAACCAAAACATTGTTCTCT	58	CCACCATTTTGCATTCCTTT	60	229	--	--	--	--	--	--
TR59477|c2_g1_i1	XLOC_024743	(CAA)5	15	--	--	--	--	--	--	ATCCGGTATCCTACGTGCAG	60	GGTTAATCCGGGGAAGAAGA	60	184	--	--	--	--	--	--
TR59477|c2_g1_i1	XLOC_024743	(AATTC)5	25	--	--	--	--	--	--	CCGGATTAACCCATTCATTG	60	GCCAGACATGGGAGAGGTAG	60	152	--	--	--	--	--	--
TR59482|c1_g1_i1	XLOC_024751	(TG)6	12	--	--	--	--	--	--	CCAGTTGTCCCTCCTGCTTA	60	AAACCAAAGGCTTTACTTGATAG AAA	60	208	--	--	--	--	--	--
TR59482|c2_g1_i1	XLOC_024753	(CAA)6cattagtgttgttcatcaacagaacatgattccggc atcatcatcggacggagta(AAC)5	88	--	--	--	--	--	--	AATCACGCCCAATGTTGATT	60	AACACATTCCGATCCTTTGC	60	268	--	--	--	--	--	--
TR59482|c2_g2_i1	XLOC_024754	(CT)7	14	--	--	--	--	--	--	ACCACAAACACAACCAACCC	61	TTGTCTTTCGAAGAACCGAA	58	233	--	--	--	--	--	--
TR59482|c2_g2_i1	XLOC_024754	(AAT)6	18	--	--	--	--	--	--	CTCTTCGCTCACGTCTTTCC	60	GGCGCGTGAATCATACTCTT	60	130	--	--	--	--	--	--
TR59482|c3_g1_i5	No	(GT)9ctgcagctgaaaaggaaaacgccatcaagaatct cctcaattcacactcacattcctcctgtttactgttc(CT)6	101	--	--	--	--	--	--	ATTCCTTCGCGTTGAGAAGA	60	GAGAGAAGCGCCCATTGTAG	60	206	--	--	--	--	--	--
TR59482|c4_g1_i1	XLOC_024756	(TC)6gcaaccctatcatattcattctccttctatctctcatctc tctg(CAA)5	71	--	--	--	--	--	--	AACTCACGCACGCTTTTCTT	60	TGCCTTGAGCCCCTTTTTAT	61	257	--	--	--	--	--	--
TR59483|c0_g1_i2	No	(GAT)5	15	--	--	--	--	--	--	TGGCATGGACAAAAAGAGAA	59	AAATGCCACAAACTTCGACC	60	246	--	--	--	--	--	--
TR59483|c0_g1_i3	No	(GAT)5	15	--	--	--	--	--	--	TGGCATGGACAAAAAGAGAA	59	AAATGCCACAAACTTCGACC	60	246	--	--	--	--	--	--
TR59483|c0_g1_i4	No	(GAT)5	15	--	--	--	--	--	--	TGGCATGGACAAAAAGAGAA	59	AAATGCCACAAACTTCGACC	60	246	--	--	--	--	--	--
TR59483|c0_g1_i5	No	(GAT)5	15	--	--	--	--	--	--	TGGCATGGACAAAAAGAGAA	59	AAATGCCACAAACTTCGACC	60	246	--	--	--	--	--	--

Transcript IDs	Differentially expressed gene (DEG) IDs	SSR repeat-motifs	SSR repeat-length (bp)	*In silico* polymorphic SSRs						Forward primers (5'-3')	Annealing tempearture(0C)	Reverse primers (5'-3')	Annealing tempearture(0C)	Amplified product size (bp)	Structural annotation		Functional Annotation			
				C. microphyllum		ICC4958									Gene IDs	Sequence component of genes	NCBI nr IDs	TFs	KOG	KEGG
				Transcript	SSR repeat-motifs	Chromosomes	SSR repeat-motifs	Start physical positions (bp)	End physical positions (bp)											
TR59483|c0_g1_i6	No	(GAT)5	15	--	--	--	--	--	--	TGGCATGGACAAAAAGAGAA	59	AAATGCCACAAACTTCGACC	60	246	--	--	--	--	--	--
TR59483|c0_g1_i7	No	(GAT)5	15	--	--	--	--	--	--	TGGCATGGACAAAAAGAGAA	59	AAATGCCACAAACTTCGACC	60	246	--	--	--	--	--	--
TR59483|c0_g1_i8	No	(GAT)5	15	--	--	--	--	--	--	TGGCATGGACAAAAAGAGAA	59	AAATGCCACAAACTTCGACC	60	246	--	--	--	--	--	--
TR59486|c1_g1_i1	XLOC_024761	(CAA)6	18	--	--	--	--	--	--	CACTTCCCTCACCCACCTAA	60	GGGGAAGAAGGAGAAGGATG	60	250	--	--	--	--	--	--
TR59486|c2_g1_i1	No	(CT)11	22	--	--	--	--	--	--	ACATCGTGTGTGCCACATTT	60	CGACGTTTCGAATGTGAATG	60	129	--	--	--	--	--	--
TR59486|c2_g1_i2	No	(CT)11	22	--	--	--	--	--	--	ACATCGTGTGTGCCACATTT	60	CGACGTTTCGAATGTGAATG	60	129	--	--	--	--	--	--
TR59486|c2_g1_i3	No	(CT)11	22	--	--	--	--	--	--	ACATCGTGTGTGCCACATTT	60	CGACGTTTCGAATGTGAATG	60	129	--	--	--	--	--	--
TR59486|c2_g1_i6	No	(CT)11	22	--	--	--	--	--	--	ACATCGTGTGTGCCACATTT	60	CGACGTTTCGAATGTGAATG	60	129	--	--	--	--	--	--
TR59486|c2_g1_i8	No	(CT)11	22	--	--	--	--	--	--	ACATCGTGTGTGCCACATTT	60	CGACGTTTCGAATGTGAATG	60	129	--	--	--	--	--	--
TR59486|c2_g1_i9	No	(CT)11	22	--	--	--	--	--	--	ACATCGTGTGTGCCACATTT	60	CGACGTTTCGAATGTGAATG	60	129	--	--	--	--	--	--
TR59486|c4_g1_i1	XLOC_024766	(TC)10	20	--	--	--	--	--	--	AAACACCACCTACGCACACA	60	CAACAATGGCACGATGAGAG	60	255	--	--	--	--	--	--
TR59488|c0_g1_i1	No	(CT)6	12	--	--	--	--	--	--	TCGATCGATCCCTCAATTTC	60	CTTCTGTTGACGCCTTGTGA	60	228	--	--	--	--	--	--
TR59488|c0_g1_i2	No	(CT)6	12	--	--	--	--	--	--	TCGATCGATCCCTCAATTTC	60	CTTCTGTTGACGCCTTGTGA	60	228	--	--	--	--	--	--
TR59488|c0_g1_i3	No	(CT)6	12	--	--	--	--	--	--	TCGATCGATCCCTCAATTTC	60	CTTCTGTTGACGCCTTGTGA	60	228	--	--	--	--	--	--

Transcript IDs	Differentially expressed gene (DEG) IDs	SSR repeat-motifs	SSR repeat-length (bp)	*In silico* polymorphic SSRs						Forward primers (5'-3')	Annealing tempearture(0C)	Reverse primers (5'-3')	Annealing tempearture(0C)	Amplified product size (bp)	Structural annotation		Functional Annotation			
				C. microphyllum		ICC4958									Gene IDs	Sequence component of genes	NCBI nr IDs	TFs	KOG	KEGG
				Transcript	SSR repeat-motifs	Chromosomes	SSR repeat-motifs	Start physical positions (bp)	End physical positions (bp)											
TR59492|c0_g1_i1	No	(CCT)5	15	--	--	--	--	--	--	GCAGTTGCTAGTTCCCCAAC	60	TTGTGAAGTTGAAGAGGGGG	60	239	--	--	--	--	--	--
TR59492|c0_g1_i2	No	(CCT)5	15	--	--	--	--	--	--	CTCCTCCACTCCTTCACCAC	60	TGAAGTTGAAGAGGGGGTTG	60	257	--	--	--	--	--	--
TR59492|c0_g1_i3	No	(CCT)5	15	--	--	--	--	--	--	GCAGTTGCTAGTTCCCCAAC	60	TTGTGAAGTTGAAGAGGGGG	60	239	--	--	--	--	--	--
TR59492|c0_g1_i4	No	(CCT)5	15	--	--	--	--	--	--	CTCCTCCACTCCTTCACCAC	60	TGAAGTTGAAGAGGGGGTTG	60	257	--	--	--	--	--	--
TR59497|c1_g1_i12	No	(TGT)5	15	--	--	--	--	--	--	GGGTTGTTGTTGCTGTGTTG	60	TCATTTTCTTCGGCGAGATT	60	206	--	--	--	--	--	--
TR59497|c1_g1_i13	No	(TGT)5	15	--	--	--	--	--	--	GGGTTGTTGTTGCTGTGTTG	60	TCATTTTCTTCGGCGAGATT	60	206	--	--	--	--	--	--
TR59497|c1_g1_i5	No	(TGT)5	15	--	--	--	--	--	--	GGGTTGTTGTTGCTGTGTTG	60	TCATTTTCTTCGGCGAGATT	60	206	--	--	--	--	--	--
TR59502|c0_g1_i3	No	(A)12ggg(A)11ggaaaaacg(A)10	45	--	--	--	--	--	--	ACTCATTGCAGCAACATCCA	60	TTGAGAGCAATGAGAGCTGC	59	228	--	--	--	--	--	--
TR59514|c0_g1_i1	XLOC_024831	(CCG)5	15	--	--	--	--	--	--	TTCCCCAGTCACAGAAAAGC	60	TGGGCTTTTTATTGCTGTCC	60	140	--	--	XP_011493760.1	--	--	--
TR59517|c0_g1_i1	No	(AG)8	16	--	--	--	--	--	--	GCGAAACGTTTTTACCCAAA	60	TCCGAGTGGTATATGTGCGA	60	249	--	--	--	--	--	--
TR59544|c0_g1_i1	XLOC_024839	(AAG)5	15	--	--	--	--	--	--	CAGTATACGCCGACGAGTCA	60	CAACAACAAAGACAGCAGCG	61	251	--	--	--	--	--	--
TR596|c0_g1_i2	No	(AGG)6	18	--	--	--	--	--	--	ATCGACCATTTGAAAGCTGG	60	TGTTCTCTTCGCGTGATGTC	60	270	--	--	--	--	--	--
TR59699|c0_g1_i1	XLOC_024896	(TTCA)6	24	--	--	--	--	--	--	TTTTCGCGTTGAGAGAATCA	60	GCGGTGTTGATCTGGAAAAT	60	148	--	--	--	--	--	--
TR59701|c0_g1_i1	No	(GTT)6	18	--	--	--	--	--	--	TTTGTGGCTAATGGCTTGGT	61	TGAATCAGGAACAAAAACACG	59	222	--	--	--	--	--	--

Transcript IDs	Differentially expressed gene (DEG) IDs	SSR repeat-motifs	SSR repeat-length (bp)	*In silico* polymorphic SSRs						Forward primers (5'-3')	Annealing tempearture(0C)	Reverse primers (5'-3')	Annealing tempearture(0C)	Amplified product size (bp)	Structural annotation		Functional Annotation			
				C. microphyllum		ICC4958									Gene IDs	Sequence component of genes	NCBI nr IDs	TFs	KOG	KEGG
				Transcript	SSR repeat-motifs	Chromosomes	SSR repeat-motifs	Start physical positions (bp)	End physical positions (bp)											
TR59701|c0_g1_i2	No	(GTT)6	18	--	--	--	--	--	--	TTTGTGGCTAATGGCTTGGT	61	TGAATCAGGAACAAAAACACG	59	222	--	--	--	--	--	--
TR59708|c0_g1_i1	XLOC_024905	(AT)10	20	--	--	--	--	--	--	AAATGAAATAAATGCCACCCC	60	TGGAGATTTGGTGCTGTCAA	60	278	--	--	--	--	--	--
TR59708|c1_g1_i1	XLOC_024906	(TTC)6	18	--	--	--	--	--	--	CGTGCTCTTCTGGGTCTCTT	60	TCGCACATTTGTTCCCAATA	60	259	--	--	--	--	--	--
TR59711|c0_g1_i1	No	(TC)6	12	--	--	--	--	--	--	CACCAACACCTTCCACAACA	60	TTGTTTCCCTTCACTTTGGG	60	165	--	--	--	--	--	--
TR59711|c0_g1_i1	No	(TCT)6	18	--	--	--	--	--	--	CCCAAAGTGAAGGGAAACAA	60	GCCCCTACACCTTTCCAAAT	60	278	--	--	--	--	--	--
TR59711|c0_g1_i2	No	(TC)6	12	--	--	--	--	--	--	CACCAACACCTTCCACAACA	60	TTGTTTCCCTTCACTTTGGG	60	165	--	--	--	--	--	--
TR59711|c0_g1_i2	No	(TCT)6	18	--	--	--	--	--	--	CCCAAAGTGAAGGGAAACAA	60	GCCCCTACACCTTTCCAAAT	60	278	--	--	--	--	--	--
TR59714|c0_g1_i1	No	(ATG)5	15	--	--	--	--	--	--	GAAGGAACCAACAACGAGGA	60	CATTAGCATTAGCCGCATCA	60	124	--	--	--	--	--	--
TR59714|c0_g2_i1	No	(ATG)5	15	--	--	--	--	--	--	GAAGGAACCAACAACGAGGA	60	CATTAGCATTAGCCGCATCA	60	124	--	--	--	--	--	--
TR59739|c0_g1_i1	No	(CTT)6	18	TR59739|c0_g1_i1	(CTT)6	Ca_LG_5	(CTT)5	27497544	27497558	TGAGAACCATGGCAATAGCA	60	TGCGTTGTTGTTGAGAGGAG	60	234	Ca_08477	CDS	--	--	--	--
TR59739|c0_g2_i1	No	(CTT)6	18	--	--	--	--	--	--	TGAGAACCATGGCAATAGCA	60	TGCGTTGTTGTTGAGAGGAG	60	234	--	--	--	--	--	--
TR59766|c0_g1_i1	XLOC_024955	(TTC)5	15	--	--	--	--	--	--	AGTCGGGTCAAGGTCCTTCT	60	CGCTCTCCAAACGATACCTC	60	268	--	--	XP_008862576.1	--	[J]	--
TR59779|c0_g1_i1	No	(GA)6tatgtagtacataaataaacaaaacaaaag(A)11	53	TR59779|c0_g1_i1	(AT)6	Ca_LG_3	(AT)7	27976220	27976233	AGATGCAGAGTGAGGATGAAA	57	CCCAACACAAACCTCATCTCT	59	209		URR	--	--	--	--
TR59779|c0_g1_i1	No	(AT)6	12	TR59779|c0_g1_i1	(AT)6	Ca_LG_3	(AT)7	27976220	27976233	GACAAAGGACAATCAAGTTGAGG	60	TTCATCCTCACTCTGCATCTACA	60	203		URR	--	--	--	--

Transcript IDs	Differentially expressed gene (DEG) IDs	SSR repeat-motifs	SSR repeat-length (bp)	*In silico* polymorphic SSRs						Forward primers (5'-3')	Annealing tempearture(0C)	Reverse primers (5'-3')	Annealing tempearture(0C)	Amplified product size (bp)	Structural annotation		Functional Annotation			
				C. microphyllum		ICC4958									Gene IDs	Sequence component of genes	NCBI nr IDs	TFs	KOG	KEGG
				Transcript	SSR repeat-motifs	Chromosomes	SSR repeat-motifs	Start physical positions (bp)	End physical positions (bp)											
TR59779|c0_g1_i1	No	(A)10gg(T)11	23	TR59779|c0_g1_i1	(AT)6	Ca_LG_3	(AT)7	27976220	27976233	TGCAAATTGAAGAAGGAGCA	60	AACCTCAACTTGATTGTCCTTTG	60	257		URR	--	--	--	--
TR59779|c0_g2_i1	XLOC_024967	(TA)7	14	--	--	--	--	--	--	CGTCTTCACAAAAGCAAAACC	60	TTGTCAGAAGCTTGAAGGATCA	60	189	--	--	--	--	--	--
TR59779|c0_g3_i1	XLOC_024968	(TA)7	14	--	--	--	--	--	--	CGTCTTCACAAAAGCAAAACC	60	TTGTCAGAAGCTTGAAGGATCA	60	189	--	--	--	--	--	--
TR59780|c0_g1_i1	No	(TC)6(TA)7	26	--	--	--	--	--	--	GATTTTACCACTTCCATGGTCA	59	GAAAGATGGGGCAATGTTGT	60	142	--	--	--	--	--	--
TR59780|c0_g1_i2	No	(TC)6(TA)7	26	--	--	--	--	--	--	GATTTTACCACTTCCATGGTCA	59	GAAAGATGGGGCAATGTTGT	60	142	--	--	--	--	--	--
TR59841|c0_g1_i1	XLOC_025000	(AG)6	12	--	--	--	--	--	--	AAGTGAAGCAAGGGACGAGA	60	GAACTGGATCGGGTTTTTCA	60	246	--	--	--	--	--	--
TR60075|c0_g1_i2	No	(AAG)5	15	--	--	--	--	--	--	ATATCTCGTTCCTTGGTGCG	60	CTCCCATGGAGACTTACCCA	60	113	--	--	--	--	--	--
TR60075|c0_g1_i3	No	(AAG)5	15	--	--	--	--	--	--	GCGATCCTATGGTTGAGGAA	60	CTCCCATGGAGACTTACCCA	60	102	--	--	--	--	--	--
TR60078|c0_g1_i1	No	(TAA)5	15	TR60078|c0_g1_i1	(TAA)5	scaffold04127	(TAA)9	7811	7837	TTGAGAGTCCTTCATTGCCA	59	TCCAAAGCTTGACAACACTCC	60	239	Ca_22435	URR	--	--	--	--
TR60078|c0_g2_i1	No	(TAA)5	15	--	--	--	--	--	--	TTGAGAGTCCTTCATTGCCA	59	TCCAAAGCTTGACAACACTCC	60	239	--	--	--	--	--	--
TR60104|c0_g1_i1	No	(TG)6	12	--	--	--	--	--	--	TCTCTCGTTTCCCAACTGCT	60	ATTGGGGTCCTCCCTAATTG	60	270	--	--	--	--	--	--
TR602|c0_g1_i1	No	(A)10tcaacaaattctactattcaatttaggaacatagatcaatagtgttcattcattctccaatggccctcgccgttacttcttctt(CAG)7	115	--	--	--	--	--	--	CCGTTACTTCTTCTTCAGCAGC	61	ATCGGAGGAAGATCCGATG	60	190	--	--	--	--	--	--
TR602|c0_g1_i2	No	(A)10tcaacaaattctactattcaatttaggaacatagatcaatagtgttcattcattctccaatggccctcgccgttacttcttctt(CAG)7	115	--	--	--	--	--	--	CCGTTACTTCTTCTTCAGCAGC	61	ATCGGAGGAAGATCCGATG	60	190	--	--	--	--	--	--
TR602|c0_g1_i3	No	(A)10tcaacaaattctactattcaatttaggaacatagatcaatagtgttcattcattctccaatggccctcgccgttacttcttctt(CAG)7	115	--	--	--	--	--	--	CCGTTACTTCTTCTTCAGCAGC	61	ATCGGAGGAAGATCCGATG	60	190	--	--	--	--	--	--

Transcript IDs	Differentially expressed gene (DEG) IDs	SSR repeat-motifs	SSR repeat-length (bp)	*In silico* polymorphic SSRs						Forward primers (5'-3')	Annealing tempearture(0C)	Reverse primers (5'-3')	Annealing tempearture(0C)	Amplified product size (bp)	Structural annotation		Functional Annotation			
				C. microphyllum		ICC4958									Gene IDs	Sequence component of genes	NCBI nr IDs	TFs	KOG	KEGG
				Transcript	SSR repeat-motifs	Chromosomes	SSR repeat-motifs	Start physical positions (bp)	End physical positions (bp)											
TR602|c0_g1_i4	No	(A)10tcaacaaattctactattcaatttaggaacatagatcaatagtgttcattcattctccaatggccctcgccgttacttcttctt(CAG)7	115	--	--	--	--	--	--	CCGTTACTTCTTCTTCAGCAGC	61	ATCGGAGGAAGATCCGATG	60	190	--	--	--	--	--	--
TR602|c0_g1_i5	No	(A)10tcaacaaattctactattcaatttaggaacatagatcaatagtgttcattcattctccaatggccctcgccgttacttcttctt(CAG)7	115	--	--	--	--	--	--	CCGTTACTTCTTCTTCAGCAGC	61	ATCGGAGGAAGATCCGATG	60	190	--	--	--	--	--	--
TR602|c0_g1_i6	No	(A)10tcaacaaattctactattcaatttaggaacatagatcaatagtgttcattcattctccaatggccctcgccgttacttcttctt(CAG)7	115	--	--	--	--	--	--	CCGTTACTTCTTCTTCAGCAGC	61	ATCGGAGGAAGATCCGATG	60	190	--	--	--	--	--	--
TR602|c0_g2_i4	XLOC_025121	(CAG)7	21	--	--	--	--	--	--	GCTCAGGATCAGGACAAGGA	60	AGTGTTTGATGCTGGTGCAG	60	158	--	--	--	--	--	--
TR60348|c0_g1_i1	XLOC_025128	(GAG)7	21	--	--	--	--	--	--	GGTTGCGGTAAAGGAGACG	61	AGCAGGTGTTTGTCTCCCAC	60	105	--	--	--	--	--	--
TR60348|c0_g2_i1	No	(GAG)7	21	--	--	--	--	--	--	GGTTGCGGTAAAGGAGACG	61	AGCAGGTGTTTGTCTCCCAC	60	105	--	--	--	--	--	--
TR60467|c0_g1_i1	No	(AT)6	12	--	--	--	--	--	--	TGCTATGCAACTCATCCACC	60	AGAGAGTGTGTTGGTAATACTTCTCA	58	176	--	--	--	--	--	--
TR60578|c0_g1_i1	No	(GCA)7	21	--	--	--	--	--	--	GCACAAAGTCCTTGGAGGAG	60	TCGACCTCTTCGACAAGTCC	60	124	--	--	--	--	--	--
TR60598|c0_g1_i1	XLOC_025196	(GA)9	18	--	--	--	--	--	--	GTTTCCAGTGTTTAGGGCGA	60	GCTCAGGTAGCACTTCCTCC	59	241	--	--	--	--	--	--
TR60610|c0_g1_i1	XLOC_025205	(TA)8	16	--	--	--	--	--	--	GTTGGAAGCGGTAACGACAT	60	CAGCAAGCAAACTGTCTGTG	58	222	--	--	--	--	--	--
TR60610|c0_g1_i1	XLOC_025205	(GGA)10	30	--	--	--	--	--	--	TCGGAATTACCACCGTTCTC	60	ATGTCGTTACCGCTTCCAAC	60	240	--	--	--	--	--	--
TR60610|c0_g1_i2	No	(GGA)10	30	--	--	--	--	--	--	TCGGAATTACCACCGTTCTC	60	CAAACTTTTTCGCTGCAACA	60	239	--	--	--	--	--	--
TR60610|c0_g1_i2	No	(TA)8	16	--	--	--	--	--	--	TGTTGCAGCGAAAAAGTTTG	60	CTGAAAACGACAGCAAGCAA	60	211	--	--	--	--	--	--
TR60617|c0_g1_i1	XLOC_025208	(AAT)5	15	--	--	--	--	--	--	TGCACATGCAAGGAATTTGT	60	TGCAACAAAGTGAAAGGTGG	60	156	--	--	XM_004492808.1	AP2	--	--

Transcript IDs	Differentially expressed gene (DEG) IDs	SSR repeat-motifs	SSR repeat-length (bp)	*In silico* polymorphic SSRs						Forward primers (5'-3')	Annealing tempearture(0C)	Reverse primers (5'-3')	Annealing tempearture(0C)	Amplified product size (bp)	Structural annotation		Functional Annotation			
				C. microphyllum		ICC4958														
				Transcript	SSR repeat-motifs	Chromosomes	SSR repeat-motifs	Start physical positions (bp)	End physical positions (bp)						Gene IDs	Sequence component of genes	NCBI nr IDs	TFs	KOG	KEGG
TR60659|c0_g1_i1	No	(CAC)5	15	--	--	--	--	--	--	TCTTTGCACCCATAACACCA	60	CAAAATTTGCTGACCCCATT	60	216	--	--	--	--	--	--
TR607|c1_g1_i1	No	(TG)7	14	--	--	--	--	--	--	CAGCCCAGCCCACTATTTTA	60	CTGCCTCTGTGATTTTGGTG	59	169	--	--	--	--	--	--
TR607|c1_g1_i2	No	(TG)7	14	--	--	--	--	--	--	CAGCCCAGCCCACTATTTTA	60	CTGCCTCTGTGATTTTGGTG	59	169	--	--	--	--	--	--
TR607|c1_g1_i3	No	(TG)7	14	--	--	--	--	--	--	CAGCCCAGCCCACTATTTTA	60	CTGCCTCTGTGATTTTGGTG	59	169	--	--	--	--	--	--
TR607|c1_g1_i4	No	(TG)7	14	--	--	--	--	--	--	CAGCCCAGCCCACTATTTTA	60	CTGCCTCTGTGATTTTGGTG	59	169	--	--	--	--	--	--
TR60704|c0_g1_i1	No	(GGA)5	15	--	--	--	--	--	--	GGACTGGGCTCCATCAGTTA	60	CTGGTAGGCTTGGTCGTCTC	60	165	--	--	--	--	--	--
TR60769|c0_g1_i1	XLOC_025263	(AT)6	12	--	--	--	--	--	--	CCAACAACTTTTGCGACCAT	61	ATTGGCTTAAAACCATTGCG	60	280	--	--	--	--	--	--
TR608|c0_g1_i1	No	(TCT)5	15	--	--	--	--	--	--	TTAAGGGTCCACTTTGGGAA	59	GATTTATAATAGCTTCCACTTGAAAAA	58	185	--	--	--	--	--	--
TR608|c0_g1_i3	No	(TCT)5	15	--	--	--	--	--	--	TTAAGGGTCCACTTTGGGAA	59	GATTTATAATAGCTTCCACTTGAAAAA	58	185	--	--	--	--	--	--
TR608|c0_g2_i1	No	(TAA)7	21	--	--	--	--	--	--	GGAACACGTCTCAAGGAAGC	60	TTTCCTTCACTTGGCTTTTCA	60	210	--	--	--	--	--	--
TR608|c0_g2_i2	No	(TAA)7	21	--	--	--	--	--	--	GGAACACGTCTCAAGGAAGC	60	TTTCCTTCACTTGGCTTTTCA	60	210	--	--	--	--	--	--
TR60855|c0_g1_i1	XLOC_025288	(AGAT)5	20	--	--	--	--	--	--	AGCGAAAGGAATCACAGTGG	60	CACACTCAATCCACAAACGG	60	275	--	--	--	--	--	--
TR60885|c0_g1_i1	XLOC_025295	(AT)8	16	--	--	--	--	--	--	TGGACTTCAACGCAATCAAA	60	TTCCTCATCCATAGCCCAAC	60	252	--	--	--	--	--	--
TR61067|c0_g1_i1	No	(ATG)5	15	--	--	--	--	--	--	CAGAACGAGGCTTCAGCTTT	60	TTTGTTAACCTACCCTGGTCA	57	238	--	--	--	--	--	--

Transcript IDs	Differentially expressed gene (DEG) IDs	SSR repeat-motifs	SSR repeat-length (bp)	*In silico* polymorphic SSRs						Forward primers (5'-3')	Annealing tempearture(0C)	Reverse primers (5'-3')	Annealing tempearture(0C)	Amplified product size (bp)	Structural annotation		Functional Annotation			
				C. microphyllum		ICC4958									Gene IDs	Sequence component of genes	NCBI nr IDs	TFs	KOG	KEGG
				Transcript	SSR repeat-motifs	Chromosomes	SSR repeat-motifs	Start physical positions (bp)	End physical positions (bp)											
TR61071|c0_g1_i1	No	(GA)8	16	--	--	--	--	--	--	AGTGACCAGTGGTAGTGGGC	60	TAGCTCTCTTCCGGATCGAA	60	100	--	--	--	--	--	--
TR61071|c0_g2_i1	No	(GA)8	16	--	--	--	--	--	--	CCTTGGTGCGGTATGTCTTT	60	TAGCTCTCTTCCGGATCGAA	60	143	--	--	--	--	--	--
TR61108|c0_g1_i1	No	(GAT)5	15	--	--	--	--	--	--	TTTTAATGGCCCTGATGCTC	60	AAAACACTATCCGCAGGCAG	60	156	--	--	--	--	--	--
TR61127|c0_g1_i1	No	(TA)8	16	--	--	--	--	--	--	TTGCCCCTCTCATCTCTCAT	60	TTTTGCTAATGCAGAACGGA	59	105	--	--	--	--	--	--
TR6114|c0_g1_i1	XLOC_025352	(TTG)5	15	--	--	--	--	--	--	TTGGGCAGGTCCAAGTTTAC	60	AAAAAGACCTCAGACGCGAA	60	121	--	--	EUC57052.1	--	--	--
TR61163|c0_g1_i1	No	(TA)8	16	--	--	--	--	--	--	GGGCCTTCTTTCCTTTGTTT	60	TGAGCCAATGAATTTCCACTC	60	119	--	--	--	--	--	--
TR61169|c0_g1_i1	No	(GAT)5	15	--	--	--	--	--	--	AGTGAGGAAGGCGTTGAAGA	60	GCAGAGTGAACTGGAGGACC	60	152	--	--	--	--	--	--
TR61169|c0_g1_i1	No	(GAC)5	15	--	--	--	--	--	--	GGTCCTCCAGTTCACTCTGC	60	AAGCGTCATCGTCATCATCA	60	118	--	--	--	--	--	--
TR61172|c0_g1_i1	No	(GT)6	12	--	--	--	--	--	--	ATTGTTTTGGGCCTCAAGTG	60	GTCATCCTCGGAACATGGAG	60	230	--	--	--	--	--	--
TR61181|c0_g1_i1	XLOC_025358	(AAT)5	15	--	--	--	--	--	--	CAATGCATGCACAATCCAAT	60	AGTGCCTTGCCTCCTCTTTT	60	271	--	--	--	--	--	--
TR612|c2_g1_i1	XLOC_025450	(AC)6ccttgttttctatt(TC)6	38	--	--	--	--	--	--	TTACCCAACCAAACCACACA	60	TTTTTGTCGAATGAAACGCA	60	234	--	--	XM_004490397.1	bHLH	[T]	--
TR61206|c0_g1_i1	XLOC_025368	(TGT)5	15	--	--	--	--	--	--	TAGTGGCTCATGCCAGATTG	60	CACCAGTAAAGACCGTTGGA	59	252	--	--	AHX56895.1	--	[GC]	--
TR61233|c0_g1_i1	No	(CTC)6	18	--	--	--	--	--	--	AAAAGCGCAACTGAAGCAAT	60	AAATATGCTCCGACACCGAG	60	192	--	--	--	--	--	--
TR61233|c0_g2_i1	No	(CTC)6	18	--	--	--	--	--	--	AAAAGCGCAACTGAAGCAAT	60	AAATATGCTCCGACACCGAG	60	192	--	--	--	--	--	--

Transcript IDs	Differentially expressed gene (DEG) IDs	SSR repeat-motifs	SSR repeat-length (bp)	*In silico* polymorphic SSRs						Forward primers (5'-3')	Annealing tempearture(0C)	Reverse primers (5'-3')	Annealing tempearture(0C)	Amplified product size (bp)	Structural annotation		Functional Annotation			
				C. microphyllum		ICC4958									Gene IDs	Sequence component of genes	NCBI nr IDs	TFs	KOG	KEGG
				Transcript	SSR repeat-motifs	Chromosomes	SSR repeat-motifs	Start physical positions (bp)	End physical positions (bp)											
TR61256|c0_g1_i1	XLOC_025394	(TGC)5	15	--	--	--	--	--	--	TCCAATTCAATCAACCATGC	59	CTTTAACCTGTTTCTGCCCG	60	158	--	--	--	--	--	--
TR61257|c0_g1_i1	XLOC_025396	(AAG)5	15	--	--	--	--	--	--	GAGGACCTTTATGTGCCCAA	60	TCCAGCTGCATTGTTAGCAT	59	144	--	--	BAJ87032.1	--	[R]	--
TR61259|c0_g1_i1	No	(TTC)8	24	TR61259|c0_g1_i1	(TTC)8	Ca_LG_4	(TTC)5	32553040	32553054	TTTGTGTCTAACATTCTCTACCGC	60	AAATCACAGCCACCATAGGC	60	216	Ca_01775	DRR	--	--	--	--
TR61259|c0_g1_i3	No	(TTC)8	24	--	--	--	--	--	--	TTTGTGTCTAACATTCTCTACCGC	60	AAATCACAGCCACCATAGGC	60	216	--	--	--	--	--	--
TR61259|c1_g1_i1	XLOC_025398	(CT)6	12	--	--	--	--	--	--	AGGAGAGTCACTGAATCGCAA	60	TAGAAGCCATGGCCAAAGTC	60	186	--	--	--	--	--	--
TR61259|c1_g1_i2	No	(CT)6	12	--	--	--	--	--	--	AGGAGAGTCACTGAATCGCAA	60	GCCTTGGAAATGGTGAAAAA	60	132	--	--	--	--	--	--
TR61259|c2_g1_i1	No	(CT)6	12	TR61259|c2_g1_i1	(CT)6	Ca_LG_2	(CT)7	28351522	28351535	GCCTCACGAGCTTTTCTTCT	59	GCCAAAGTGTTTGGAATTGTG	60	166		URR	--	--	--	--
TR61283|c1_g1_i1	No	(GAT)5	15	--	--	--	--	--	--	TTTGGAGAGCTTGAATGGCT	60	AACAGATTCATGGACCAGGC	60	265	--	--	--	--	--	--
TR61298|c2_g1_i1	XLOC_025447	(GAG)6	18	--	--	--	--	--	--	ATCCAATCCAATCCAATCCA	60	AGGTCGTGATGCTTTGGTTC	60	125	--	--	--	--	--	--
TR61301|c0_g1_i1	No	(ATT)5	15	--	--	--	--	--	--	AAAGGTCCTCTCCATTTGGG	60	AACCAATGCCAACACCATTT	60	216	--	--	--	--	--	--
TR61301|c0_g1_i2	No	(ATT)5	15	--	--	--	--	--	--	AAAGGTCCTCTCCATTTGGG	60	AACCAATGCCAACACCATTT	60	216	--	--	--	--	--	--
TR61301|c0_g1_i3	No	(ATT)5	15	--	--	--	--	--	--	AAAGGTCCTCTCCATTTGGG	60	AACCAATGCCAACACCATTT	60	216	--	--	--	--	--	--
TR61301|c0_g1_i4	No	(ATT)5	15	--	--	--	--	--	--	AAAGGTCCTCTCCATTTGGG	60	AACCAATGCCAACACCATTT	60	216	--	--	--	--	--	--
TR61307|c0_g1_i1	XLOC_025458	(TG)7	14	--	--	--	--	--	--	GAGGGTGAATGATCCCTGAA	60	GGACGAGTGAGACGTTACAACA	60	151	--	--	--	--	--	--

Transcript IDs	Differentially expressed gene (DEG) IDs	SSR repeat-motifs	SSR repeat-length (bp)	*In silico* polymorphic SSRs						Forward primers (5'-3')	Annealing tempearture(0C)	Reverse primers (5'-3')	Annealing tempearture(0C)	Amplified product size (bp)	Structural annotation		Functional Annotation			
				C. microphyllum		ICC4958														
				Transcript	SSR repeat-motifs	Chromosomes	SSR repeat-motifs	Start physical positions (bp)	End physical positions (bp)						Gene IDs	Sequence component of genes	NCBI nr IDs	TFs	KOG	KEGG
TR61307|c0_g1_i2	XLOC_025459	(TG)7	14	--	--	--	--	--	--	GAGGGTGAATGATCCCTGAA	60	GGACGAGTGAGACGTTACAACA	60	151	--	--	--	--	--	--
TR61313|c0_g2_i1	XLOC_025462	(TAT)7	21	--	--	--	--	--	--	CCAATCCCAGCAAACCTTTA	60	GCTTTAATCAGGCTGTCGGA	60	259	--	--	--	--	--	--
TR61326|c0_g1_i2	No	(CAC)6	18	--	--	--	--	--	--	GTCACTTTCAGCCTCCTTCG	60	TGTGAGAGAAGAGGGGCACT	60	219	--	--	--	--	--	--
TR61326|c0_g1_i3	No	(CAC)6	18	--	--	--	--	--	--	GTCACTTTCAGCCTCCTTCG	60	TGTGAGAGAAGAGGGGCACT	60	219	--	--	--	--	--	--
TR61326|c0_g1_i4	No	(CAC)6	18	--	--	--	--	--	--	GTCACTTTCAGCCTCCTTCG	60	TGTGAGAGAAGAGGGGCACT	60	219	--	--	--	--	--	--
TR61338|c0_g1_i1	No	(TTA)5	15	--	--	--	--	--	--	TGAGGTATCTAATCACCTGCAAT	58	GATGGATTGAAGGCCAAAAA	60	221	--	--	--	--	--	--
TR6134|c0_g1_i1	No	(TAA)5	15	--	--	--	--	--	--	ACAGCTTGTGAACCCAATCC	60	TAGCTCACCAATGCTGTTGC	60	104	--	--	--	--	--	--
TR6134|c0_g1_i2	No	(TAA)5	15	--	--	--	--	--	--	ACAGCTTGTGAACCCAATCC	60	TGGTCAACTTGCACACAACA	60	134	--	--	--	--	--	--
TR61343|c0_g1_i1	XLOC_025494	(ATC)5	15	--	--	--	--	--	--	GGGGCTTGCAGATATTGTGT	60	CAGTAAACTGGCCGGACAAA	61	211	--	--	--	--	--	--
TR61343|c1_g1_i3	No	(AGA)7	21	--	--	--	--	--	--	GATAGCAACCCCAAAAACGA	60	TCCATTGCACTCCTACCACA	60	269	--	--	--	--	--	--
TR61352|c0_g2_i1	XLOC_025498	(TC)6	12	--	--	--	--	--	--	CGGCATTGCAAATTACACAC	60	CAGTCCCTACACCACAACCA	59	103	--	--	--	--	--	--
TR614|c2_g1_i1	XLOC_025592	(GA)8	16	--	--	--	--	--	--	AAACGAGGTTGAGCGAGAGA	60	GCGCAAACAGTTTCTCAACA	60	120	--	--	--	--	--	--
TR614|c2_g2_i1	XLOC_025593	(GA)15	30	--	--	--	--	--	--	AAGAACGGTCAAGCGAGAGA	60	TCAAACTTGTTCCTCTCTTCTCA	58	163	--	--	--	--	--	--
TR614|c4_g1_i2	No	(TC)8	16	--	--	--	--	--	--	ACTACTGTGGCGCCATTCTC	60	GCGAGTTACCCTCGTCATTC	60	207	--	--	--	--	--	--

<table>
<tr><th rowspan="3">Transcript IDs</th><th rowspan="3">Differentially expressed gene (DEG) IDs</th><th rowspan="3">SSR repeat-motifs</th><th rowspan="3">SSR repeat-length (bp)</th><th colspan="6">In silico polymorphic SSRs</th><th rowspan="3">Forward primers (5'-3')</th><th rowspan="3">Annealing tempearture(0C)</th><th rowspan="3">Reverse primers (5'-3')</th><th rowspan="3">Annealing tempearture(0C)</th><th rowspan="3">Amplified product size (bp)</th><th colspan="2">Structural annotation</th><th colspan="4">Functional Annotation</th></tr>
<tr><th colspan="2">C. microphyllum</th><th colspan="4">ICC4958</th><th rowspan="2">Gene IDs</th><th rowspan="2">Sequence component of genes</th><th rowspan="2">NCBI nr IDs</th><th rowspan="2">TFs</th><th rowspan="2">KOG</th><th rowspan="2">KEGG</th></tr>
<tr><th>Transcript</th><th>SSR repeat-motifs</th><th>Chromosomes</th><th>SSR repeat-motifs</th><th>Start physical positions (bp)</th><th>End physical positions (bp)</th></tr>
<tr><td>TR614|c4_g1_i3</td><td>No</td><td>(TC)8</td><td>16</td><td>--</td><td>--</td><td>--</td><td>--</td><td>--</td><td>--</td><td>ACTACTGTGGCGCCATTCTC</td><td>60</td><td>GCGAGTTACCCTCGTCATTC</td><td>60</td><td>207</td><td>--</td><td>--</td><td>--</td><td>--</td><td>--</td><td>--</td></tr>
<tr><td>TR614|c4_g2_i2</td><td>No</td><td>(TC)8</td><td>16</td><td>--</td><td>--</td><td>--</td><td>--</td><td>--</td><td>--</td><td>ACTACTGTGGCGCCATTCTC</td><td>60</td><td>GCGAGTTACCCTCGTCATTC</td><td>60</td><td>207</td><td>--</td><td>--</td><td>--</td><td>--</td><td>--</td><td>--</td></tr>
<tr><td>TR614|c5_g1_i3</td><td>No</td><td>(T)10cttttagtgtgagatggactaatgatagtagaagaagaaatatgctgtgggtattttgctaactttagtttggttgagaattttgtgg(T)10</td><td>109</td><td>--</td><td>--</td><td>--</td><td>--</td><td>--</td><td>--</td><td>TCTCCTTCACCTTAATTCGTCC</td><td>60</td><td>TTTTCTCCCTGCAGGATCTC</td><td>59</td><td>267</td><td>--</td><td>--</td><td>--</td><td>--</td><td>--</td><td>--</td></tr>
<tr><td>TR614|c6_g3_i1</td><td>XLOC_025597</td><td>(AT)7</td><td>14</td><td>--</td><td>--</td><td>--</td><td>--</td><td>--</td><td>--</td><td>TGACAATATTTGAATGAAATGACACA</td><td>60</td><td>TTCAGAACATGGAGCGACAG</td><td>60</td><td>233</td><td>--</td><td>--</td><td>--</td><td>--</td><td>--</td><td>--</td></tr>
<tr><td>TR614|c6_g4_i3</td><td>XLOC_025599</td><td>(CTT)5</td><td>15</td><td>--</td><td>--</td><td>--</td><td>--</td><td>--</td><td>--</td><td>ACCACCACCAGCTGTACTCC</td><td>60</td><td>TGCCTGCTGGTCTTCTTTTT</td><td>60</td><td>209</td><td>--</td><td>--</td><td>--</td><td>--</td><td>--</td><td>--</td></tr>
<tr><td>TR614|c8_g1_i1</td><td>XLOC_025603</td><td>(GA)6</td><td>12</td><td>--</td><td>--</td><td>--</td><td>--</td><td>--</td><td>--</td><td>TGGTCAAGCGAGAGAGAGAAG</td><td>60</td><td>CGCAAAAACACTTTCAGCAA</td><td>60</td><td>173</td><td>--</td><td>--</td><td>--</td><td>--</td><td>--</td><td>--</td></tr>
<tr><td>TR61419|c0_g1_i1</td><td>XLOC_025530</td><td>(ACA)7</td><td>21</td><td>TR61419|c0_g1_i1</td><td>(ACA)7</td><td>Ca_LG_4</td><td>(ACA)6</td><td>42901907</td><td>42901924</td><td>CAACAACAATGGTTCCTGCT</td><td>59</td><td>ATCCTTGTGGTGATGTGGGT</td><td>60</td><td>189</td><td>Ca_01904</td><td>URR</td><td>--</td><td>--</td><td>--</td><td>--</td></tr>
<tr><td>TR61419|c0_g1_i1</td><td>XLOC_025530</td><td>(A)10catacactcaaaacaaaacaaatctctgaataaacttcatcatggaagaaactatg(AAC)5</td><td>81</td><td>TR61419|c0_g1_i1</td><td>(ACA)7</td><td>Ca_LG_4</td><td>(ACA)6</td><td>42901907</td><td>42901924</td><td>CACTTTGCAACCTTCCCATT</td><td>60</td><td>ATCCTGGTGGTGGTGTTGTT</td><td>60</td><td>275</td><td>Ca_01904</td><td>URR</td><td>--</td><td>--</td><td>--</td><td>--</td></tr>
<tr><td>TR61423|c1_g1_i1</td><td>XLOC_025533</td><td>(ACA)6</td><td>18</td><td>--</td><td>--</td><td>--</td><td>--</td><td>--</td><td>--</td><td>TTAACCGAAAAGGATGGCTG</td><td>60</td><td>GAAATCGAAAATCCGGAACA</td><td>60</td><td>166</td><td>--</td><td>--</td><td>XM_004489437.1</td><td>ERF</td><td>--</td><td>--</td></tr>
<tr><td>TR6143|c0_g1_i1</td><td>XLOC_025548</td><td>(A)11ccacaactttgtttcattcattcac(CTT)5</td><td>51</td><td>--</td><td>--</td><td>--</td><td>--</td><td>--</td><td>--</td><td>TCAAATCAATAACTCTTCCGATACT</td><td>58</td><td>GCCTGGATCTAAGAAAAGCAA</td><td>59</td><td>149</td><td>--</td><td>--</td><td>--</td><td>--</td><td>--</td><td>--</td></tr>
<tr><td>TR61432|c1_g1_i1</td><td>XLOC_025543</td><td>(CTC)5</td><td>15</td><td>--</td><td>--</td><td>--</td><td>--</td><td>--</td><td>--</td><td>CACCATGTCCAAACCAAACA</td><td>60</td><td>ATGGGTAGGCCATGATGTGT</td><td>60</td><td>201</td><td>--</td><td>--</td><td>--</td><td>--</td><td>--</td><td>--</td></tr>
<tr><td>TR61454|c0_g3_i1</td><td>No</td><td>(CT)7</td><td>14</td><td>TR61454|c0_g3_i1</td><td>(CT)7</td><td>Ca_LG_8</td><td>(CT)9</td><td>2991391</td><td>2991408</td><td>TCGATCCGTCGCTATTCTCT</td><td>60</td><td>GGCAAAGACCCAACTTTGAA</td><td>60</td><td>210</td><td>Ca_18990</td><td>CDS</td><td>--</td><td>--</td><td>--</td><td>--</td></tr>
<tr><td>TR61460|c0_g1_i1</td><td>No</td><td>(CA)8</td><td>16</td><td>--</td><td>--</td><td>--</td><td>--</td><td>--</td><td>--</td><td>TTGCAACAACAGCTTCACAA</td><td>59</td><td>CGAATATGAGGGCATGTTGA</td><td>59</td><td>264</td><td>--</td><td>--</td><td>--</td><td>--</td><td>--</td><td>--</td></tr>
<tr><td>TR61460|c0_g2_i1</td><td>No</td><td>(CA)8</td><td>16</td><td>--</td><td>--</td><td>--</td><td>--</td><td>--</td><td>--</td><td>TTGCAACAACAGCTTCACAA</td><td>59</td><td>CGAATATGAGGGCATGTTGA</td><td>59</td><td>264</td><td>--</td><td>--</td><td>--</td><td>--</td><td>--</td><td>--</td></tr>
</table>

Transcript IDs	Differentially expressed gene (DEG) IDs	SSR repeat-motifs	SSR repeat-length (bp)	*In silico* polymorphic SSRs						Forward primers (5'-3')	Annealing tempearture(0C)	Reverse primers (5'-3')	Annealing tempearture(0C)	Amplified product size (bp)	Structural annotation		Functional Annotation			
				C. microphyllum		ICC4958									Gene IDs	Sequence component of genes	NCBI nr IDs	TFs	KOG	KEGG
				Transcript	SSR repeat-motifs	Chromosomes	SSR repeat-motifs	Start physical positions (bp)	End physical positions (bp)											
TR61461|c0_g1_i1	XLOC_025562	(ATC)7	21	--	--	--	--	--	--	TGCAGTCAAGACACGAAAGG	60	GCTTTTGCAATGCTCAGACA	60	268	--	--	--	--	--	--
TR61470|c0_g1_i1	XLOC_025571	(GA)6	12	--	--	--	--	--	--	AGCTGTGATTGTGAAAACGCT	60	AAGAGAATCCCTCGCATCAA	60	243	--	--	--	--	--	--
TR61491|c0_g1_i1	No	(CAA)5	15	--	--	--	--	--	--	TTCCACACTTCACCATCACAA	60	GCTGCAGAGGAAGGATTGAG	60	107	--	--	--	--	--	--
TR61491|c0_g1_i2	No	(CAA)5	15	--	--	--	--	--	--	TTCCACACTTCACCATCACAA	60	GCTGCAGAGGAAGGATTGAG	60	107	--	--	--	--	--	--
TR61491|c0_g2_i1	No	(CAA)5	15	--	--	--	--	--	--	TTCCACACTTCACCATCACAA	60	GCTGCAGAGGAAGGATTGAG	60	107	--	--	--	--	--	--
TR6155|c0_g2_i1	No	(AAG)8	24	--	--	--	--	--	--	GCATAATCATGATCCGCTCC	60	TCTTCTGTGCACGCTTTTTG	60	132	--	--	--	--	--	--
TR61656|c0_g1_i1	XLOC_025649	(GTT)5	15	--	--	--	--	--	--	GGCGGTTCTTGATGATGTTT	60	TATCCGTCACGATCCTCCTC	60	163	--	--	--	--	--	--
TR61657|c0_g1_i1	No	(GAA)7	21	--	--	--	--	--	--	GGAACTTAGGCGGAAAGGTT	60	AATGAGGAGTCTGGAGCTGG	59	156	--	--	--	--	--	--
TR61680|c0_g1_i1	XLOC_025656	(TA)6	12	--	--	--	--	--	--	CAGAACAAAGCATGTGAAACAA	59	TTTATCCCTGGGGTTCACAG	60	101	--	--	--	--	--	--
TR61708|c0_g1_i1	XLOC_025666	(CGT)5	15	--	--	--	--	--	--	CTGACGTGGCACACAACTTT	60	GGAGTGCCACCGTTGATAAT	60	238	--	--	--	--	--	--
TR61787|c0_g1_i1	No	(TG)6	12	--	--	--	--	--	--	AGGTACCAAACCTCTAACCAAGT	57	TTTCCTATCTCTCCCCTCCC	59	226	--	--	--	--	--	--
TR618|c0_g1_i1	XLOC_025714	(GA)10	20	--	--	--	--	--	--	TCGAGTATGGGGTGCCTAGA	61	CACTGGCTCATCCTCCAACT	60	170	--	--	--	--	--	--
TR61882|c0_g1_i1	No	(AC)6	12	--	--	--	--	--	--	TATCTCTCTCAACACGCGCA	61	ACCTCCTCAGGGAAGCAAAT	60	116	--	--	--	--	--	--
TR61885|c0_g1_i1	XLOC_025698	(CT)10	20	TR61885|c0_g1_i1	(CT)10	Ca_LG_7	(CT)12	71911	71934	CCGCTATTTAAATTTCCTCCAA	59	AGCGACATCGTTTGGATAGG	60	140	Ca_15857	DRR	--	--	--	--

Transcript IDs	Differentially expressed gene (DEG) IDs	SSR repeat-motifs	SSR repeat-length (bp)	In silico polymorphic SSRs						Forward primers (5'-3')	Annealing tempearture(0C)	Reverse primers (5'-3')	Annealing tempearture(0C)	Amplified product size (bp)	Structural annotation		Functional Annotation			
				C. microphyllum		ICC4958														
				Transcript	SSR repeat-motifs	Chromosomes	SSR repeat-motifs	Start physical positions (bp)	End physical positions (bp)						Gene IDs	Sequence component of genes	NCBI nr IDs	TFs	KOG	KEGG
TR61885|c0_g3_i1	No	(CT)10	20	--	--	--	--	--	--	CCGCTATTTAAATTTCCTCCAA	59	AGCGACATCGTTTGGATAGG	60	140	--	--	--	--	--	--
TR61889|c0_g1_i1	XLOC_025700	(TA)6	12	--	--	--	--	--	--	CTCTCTTGGATTGCCGACAT	60	CAGTTCTGGGGTTTTGAGGA	60	242	--	--	XP_004507866.1	bHLH	[T]	K04733
TR61899|c0_g1_i1	No	(TGT)5	15	--	--	--	--	--	--	TGCTTCTCTGAGCCAATGTG	60	CCAACTATTCATTCCAGGAGAA	58	177	--	--	--	--	--	--
TR61899|c0_g1_i2	No	(TGT)5	15	--	--	--	--	--	--	TGCTTCTCTGAGCCAATGTG	60	CCAACTATTCATTCCAGGAGAA	58	177	--	--	--	--	--	--
TR61901|c0_g2_i1	No	(TTC)5	15	--	--	--	--	--	--	TGTGTTTGTCTATTCACACCCG	61	CCAGATGATTGATGGGAACA	59	171	--	--	--	--	--	--
TR61902|c0_g2_i1	No	(TA)6	12	--	--	--	--	--	--	TTGTTGGTTCAGCAGCAAAG	60	CCAAGCCCACTTACTGGAGA	60	249	--	--	--	--	--	--
TR61902|c0_g2_i3	No	(TA)6	12	--	--	--	--	--	--	TTGTTGGTTCAGCAGCAAAG	60	CCAAGCCCACTTACTGGAGA	60	249	--	--	--	--	--	--
TR61902|c0_g2_i4	No	(TA)6	12	--	--	--	--	--	--	TTGTTGGTTCAGCAGCAAAG	60	CCAAGCCCACTTACTGGAGA	60	249	--	--	--	--	--	--
TR61902|c0_g2_i5	No	(TA)6	12	--	--	--	--	--	--	TTGTTGGTTCAGCAGCAAAG	60	CCAAGCCCACTTACTGGAGA	60	249	--	--	--	--	--	--
TR61906|c0_g1_i3	XLOC_025719	(TA)6	12	--	--	--	--	--	--	AAGCGTGACAGCGATTAATGT	60	AAAGCCTTGGGTTCCAAGTT	60	172	--	--	XP_004494693.1	HD-ZIP	[C]	--
TR61911|c0_g1_i1	XLOC_025724	(CAA)6	18	--	--	--	--	--	--	CCGTGACAAACTCACACACC	60	CCATCGGAGATAAGATCGGA	60	235	--	--	--	--	--	--
TR61912|c0_g2_i1	XLOC_025726	(T)10cccaacacatttttttcacacaacttttgcataactccaaaaatcacacaccccttttg(CT)6	81	--	--	--	--	--	--	TATTCTCTCCCAATTCCCCA	59	GGGTTGTTCTTTGATGGGTG	60	275	--	--	--	--	--	--
TR61930|c0_g1_i1	XLOC_025744	(TC)8	16	--	--	--	--	--	--	CACAAATCACAACCTTCTTTCG	60	CTTCGACGCACGTTCAACTA	60	275	--	--	--	--	--	--
TR61940|c1_g1_i1	No	(TGA)5	15	--	--	--	--	--	--	AGGACGAGTTAGGTCGGGTT	60	TCTTCTCCTCCCTGCATGAT	60	204	--	--	--	--	--	--

Transcript IDs	Differentially expressed gene (DEG) IDs	SSR repeat-motifs	SSR repeat-length (bp)	*In silico* polymorphic SSRs						Forward primers (5'-3')	Annealing tempearture(0C)	Reverse primers (5'-3')	Annealing tempearture(0C)	Amplified product size (bp)	Structural annotation		Functional Annotation			
				C. microphyllum		ICC4958									Gene IDs	Sequence component of genes	NCBI nr IDs	TFs	KOG	KEGG
				Transcript	SSR repeat-motifs	Chromosomes	SSR repeat-motifs	Start physical positions (bp)	End physical positions (bp)											
TR61950|c0_g2_i1	No	(AAG)5	15	--	--	--	--	--	--	ACACGGTGGTAGACGGTAGC	60	GCACACAGGAATTCAAACGA	60	143	--	--	--	--	--	--
TR61957|c0_g1_i1	No	(TA)8	16	--	--	--	--	--	--	TGTTTCCAGAACATTTGGGAG	60	CCACACAAATCCATGTCCAA	60	228	--	--	--	--	--	--
TR61957|c0_g1_i2	No	(TA)8	16	--	--	--	--	--	--	GAGTGCATGGCAAAGTGCTA	60	CCCAATTGGTGAATCAATGTT	60	273	--	--	--	--	--	--
TR61957|c0_g1_i3	No	(TA)8	16	--	--	--	--	--	--	TGTTTCCAGAACATTTGGGAG	60	CCACACAAATCCATGTCCAA	60	228	--	--	--	--	--	--
TR61957|c0_g1_i4	No	(TA)8	16	--	--	--	--	--	--	GAGTGCATGGCAAAGTGCTA	60	CCCAATTGGTGAATCAATGTT	60	273	--	--	--	--	--	--
TR61961|c0_g1_i2	No	(TG)6	12	--	--	--	--	--	--	GCATTTTCTTTCCTTCCTGG	59	TCGGCAACACGAGAATGTAG	60	132	--	--	--	--	--	--
TR62037|c0_g1_i1	No	(AAG)5	15	--	--	--	--	--	--	GTCGGACAAAACGGTGAAGT	60	GCCTTCGTAGTCGGTGTAGC	60	163	--	--	--	--	--	--
TR621|c0_g2_i1	No	(AT)6	12	--	--	--	--	--	--	CTTGTGGAGCAAGAACCTCC	60	CCTCCTACATTTTAATTTGGGTACA	60	157	--	--	--	--	--	--
TR6211|c0_g3_i1	No	(A)11taaaaacatgatttgcttacaacatacaaaaagt(AG)8	61	--	--	--	--	--	--	TGAAGTGTCAAAAACCTTAATTGC	60	GGTTGGAATGGTGGAAAAGA	60	277	--	--	--	--	--	--
TR62205|c0_g1_i1	XLOC_025862	(GCT)5	15	--	--	--	--	--	--	TGAATGTCGAACAGGTCAGG	60	AGTACCAGATGCAAGACGCC	60	135	--	--	XP_003052526.1	--	--	--
TR62214|c0_g1_i1	XLOC_025868	(CAC)5	15	TR62214|c0_g1_i1	(CAC)5	Ca_LG_6	(CAC)7	20563370	20563390	ATCCAGGAGCAGGAGCAGTA	60	GATCGTGAGCAGGACAGTGA	60	214	Ca_14594	URR	--	--	--	--
TR62225|c0_g1_i1	No	(CA)6	12	--	--	--	--	--	--	ACACCCACACAAAAATCCGT	60	GCACACGCAAAGTCTCATCA	61	150	--	--	--	--	--	--
TR62355|c0_g1_i1	No	(ACA)5	15	--	--	--	--	--	--	TGTCATTTTCCAGAGAAACAGG	59	GGTGGGAAAAGACTCCCATT	60	148	--	--	--	--	--	--
TR62427|c0_g1_i1	No	(ATT)6	18	TR62427|c0_g1_i1	(ATT)6	scaffold19201	(ATT)5	1942	1956	TTGAAGAACACCCTCAACCA	59	CTTGGAGGAGCATCAACACA	60	250	Ca_24233	CDS	--	--	--	--

<table>
<tr><th rowspan="3">Transcript IDs</th><th rowspan="3">Differentially expressed gene (DEG) IDs</th><th rowspan="3">SSR repeat-motifs</th><th rowspan="3">SSR repeat-length (bp)</th><th colspan="6">In silico polymorphic SSRs</th><th rowspan="3">Forward primers (5'-3')</th><th rowspan="3">Annealing tempearture(0C)</th><th rowspan="3">Reverse primers (5'-3')</th><th rowspan="3">Annealing tempearture(0C)</th><th rowspan="3">Amplified product size (bp)</th><th colspan="2">Structural annotation</th><th colspan="4">Functional Annotation</th></tr>
<tr><th colspan="2">C. microphyllum</th><th colspan="4">ICC4958</th><th rowspan="2">Gene IDs</th><th rowspan="2">Sequence component of genes</th><th rowspan="2">NCBI nr IDs</th><th rowspan="2">TFs</th><th rowspan="2">KOG</th><th rowspan="2">KEGG</th></tr>
<tr><th>Transcript</th><th>SSR repeat-motifs</th><th>Chromosomes</th><th>SSR repeat-motifs</th><th>Start physical positions (bp)</th><th>End physical positions (bp)</th></tr>
<tr><td>TR62427|c0_g1_i2</td><td>No</td><td>(ATT)6</td><td>18</td><td>--</td><td>--</td><td>--</td><td>--</td><td>--</td><td>--</td><td>TTGAAGAACACCCTCAACCA</td><td>59</td><td>CTTGGAGGAGCATCAACACA</td><td>60</td><td>250</td><td>--</td><td>--</td><td>--</td><td>--</td><td>--</td><td>--</td></tr>
<tr><td>TR62431|c0_g1_i1</td><td>XLOC_025934</td><td>(TTG)6</td><td>18</td><td>--</td><td>--</td><td>--</td><td>--</td><td>--</td><td>--</td><td>CAAAACAAAATTGAGCTAGAGGAA</td><td>59</td><td>CAAATCTGGTTGTGCTGGAA</td><td>60</td><td>204</td><td>--</td><td>--</td><td>--</td><td>--</td><td>--</td><td>--</td></tr>
<tr><td>TR62436|c0_g1_i1</td><td>No</td><td>(A)16caagtcaacaaattaacatcctaacaaaaataata acaataatgttgtcaatgtgatgaagagat(A)11</td><td>92</td><td>--</td><td>--</td><td>--</td><td>--</td><td>--</td><td>--</td><td>CATGGTGAAACCCCATCTCT</td><td>60</td><td>ATGTTCGACATTGCAGCTTG</td><td>60</td><td>202</td><td>--</td><td>--</td><td>--</td><td>--</td><td>--</td><td>--</td></tr>
<tr><td>TR62436|c0_g2_i1</td><td>No</td><td>(A)16caagtcaacaaattaacatcctaacaaaaataata acaataatgttgtcaatgtgatgaagagat(A)11</td><td>92</td><td>--</td><td>--</td><td>--</td><td>--</td><td>--</td><td>--</td><td>CATGGTGAAACCCCATCTCT</td><td>60</td><td>TTGATGACATTGCAGCAAGA</td><td>59</td><td>201</td><td>--</td><td>--</td><td>--</td><td>--</td><td>--</td><td>--</td></tr>
<tr><td>TR6245|c0_g1_i1</td><td>XLOC_025950</td><td>(GAA)7</td><td>21</td><td>--</td><td>--</td><td>--</td><td>--</td><td>--</td><td>--</td><td>TGCATGGATCATGACAAAGA</td><td>59</td><td>TTCCAAACATGGGTGGTTTT</td><td>60</td><td>232</td><td>--</td><td>--</td><td>XP_004497573.1</td><td>--</td><td>[X]</td><td>--</td></tr>
<tr><td>TR62456|c0_g2_i1</td><td>XLOC_025947</td><td>(AG)6</td><td>12</td><td>--</td><td>--</td><td>--</td><td>--</td><td>--</td><td>--</td><td>CCAAACACAACAAAACGCAC</td><td>60</td><td>CCTTAACCAAATCCCCGAAT</td><td>60</td><td>142</td><td>--</td><td>--</td><td>--</td><td>--</td><td>--</td><td>--</td></tr>
<tr><td>TR62462|c0_g1_i1</td><td>XLOC_025954</td><td>(CT)7</td><td>14</td><td>TR62462|c0_g1_i1</td><td>(CT)7</td><td>scaffold13737</td><td>(CT)14</td><td>1641</td><td>1668</td><td>AAACAAATTCATTTCAAGTCTCAA</td><td>57</td><td>GCGGTTGTAGAAATCGGTGT</td><td>60</td><td>157</td><td>Ca_23331</td><td>DRR</td><td>--</td><td>--</td><td>--</td><td>--</td></tr>
<tr><td>TR62477|c1_g1_i1</td><td>XLOC_025971</td><td>(CAG)6</td><td>18</td><td>--</td><td>--</td><td>--</td><td>--</td><td>--</td><td>--</td><td>CGCACACAAACACATACAGTTG</td><td>60</td><td>TCGCCTTAGCTCTGCTATCC</td><td>60</td><td>215</td><td>--</td><td>--</td><td>--</td><td>--</td><td>--</td><td>--</td></tr>
<tr><td>TR62484|c0_g1_i1</td><td>XLOC_025978</td><td>(AAG)7</td><td>21</td><td>TR62484|c0_g1_i1</td><td>(AAG)7</td><td>Ca_LG_1</td><td>(AAG)5</td><td>1520938</td><td>1520952</td><td>GAGCACATGAGAAATAGACAGCA</td><td>60</td><td>ATGGGTCGCACTTTGATCTT</td><td>60</td><td>251</td><td>Ca_13420</td><td>URR</td><td>--</td><td>--</td><td>--</td><td>--</td></tr>
<tr><td>TR62513|c0_g1_i1</td><td>No</td><td>(T)10(TTC)5*</td><td>23</td><td>--</td><td>--</td><td>--</td><td>--</td><td>--</td><td>--</td><td>CAAACTAGGTTGCGATCCAAAT</td><td>60</td><td>GTCGGCAAGGAACTGTGATT</td><td>60</td><td>111</td><td>--</td><td>--</td><td>--</td><td>--</td><td>--</td><td>--</td></tr>
<tr><td>TR62528|c0_g1_i1</td><td>No</td><td>(TGA)5</td><td>15</td><td>--</td><td>--</td><td>--</td><td>--</td><td>--</td><td>--</td><td>ATCTCACTCACCTCGCACCT</td><td>60</td><td>TCTTCGTCATACAAATCAGCG</td><td>59</td><td>134</td><td>--</td><td>--</td><td>--</td><td>--</td><td>--</td><td>--</td></tr>
<tr><td>TR62542|c0_g1_i1</td><td>No</td><td>(TA)8</td><td>16</td><td>--</td><td>--</td><td>--</td><td>--</td><td>--</td><td>--</td><td>GGTGCTTCTAACCAAAGATAACAA A</td><td>60</td><td>GTCCTATGGTGCACCGTTTC</td><td>60</td><td>218</td><td>--</td><td>--</td><td>--</td><td>--</td><td>--</td><td>--</td></tr>
<tr><td>TR62660|c0_g1_i1</td><td>No</td><td>(CAA)5</td><td>15</td><td>--</td><td>--</td><td>--</td><td>--</td><td>--</td><td>--</td><td>GATATTCTCGAGGAGGCAGC</td><td>59</td><td>GTCCAGGAGGACCAGACG</td><td>59</td><td>255</td><td>--</td><td>--</td><td>--</td><td>--</td><td>--</td><td>--</td></tr>
<tr><td>TR62699|c0_g1_i1</td><td>No</td><td>(TA)6</td><td>12</td><td>--</td><td>--</td><td>--</td><td>--</td><td>--</td><td>--</td><td>TGAACATATGCAAGTAGGCCC</td><td>60</td><td>CGCAAACAAACACATCCATA</td><td>58</td><td>105</td><td>--</td><td>--</td><td>--</td><td>--</td><td>--</td><td>--</td></tr>
</table>

Transcript IDs	Differentially expressed gene (DEG) IDs	SSR repeat-motifs	SSR repeat-length (bp)	*In silico* polymorphic SSRs						Forward primers (5'-3')	Annealing tempearture(0C)	Reverse primers (5'-3')	Annealing tempearture(0C)	Amplified product size (bp)	Structural annotation		Functional Annotation			
				C. microphyllum		ICC4958														
				Transcript	SSR repeat-motifs	Chromosomes	SSR repeat-motifs	Start physical positions (bp)	End physical positions (bp)						Gene IDs	Sequence component of genes	NCBI nr IDs	TFs	KOG	KEGG
TR62754|c0_g1_i1	No	(GAG)7	21	--	--	--	--	--	--	TCAGCAACAGCATAACCAGG	60	GATCACCACCACCATCATCA	60	206	--	--	--	--	--	--
TR62788|c0_g1_i1	XLOC_026051	(TC)8	16	--	--	--	--	--	--	GAACCCTTTCCCTTCTCGTC	60	TAGAGTTGAGGGATCCTGCG	60	249	--	--	--	--	--	--
TR62788|c1_g1_i1	XLOC_026053	(TA)8	16	--	--	--	--	--	--	TCCTTTGATGTCCAAGACCA	59	GGATGCTCATCCTCTTCCAT	59	239	--	--	--	--	--	--
TR62804|c1_g1_i1	XLOC_026072	(GAT)5	15	--	--	--	--	--	--	GCAGTTGGTTGTACAATTTTGCT	60	GACGCTTGCTCCATTTTCTC	60	109	--	--	--	--	--	--
TR62806|c0_g2_i2	XLOC_026077	(CA)8	16	--	--	--	--	--	--	TGACAAACTCATGTGGGGAA	60	CGGAATCAGAATCGGAGTGT	60	245	--	--	--	--	--	--
TR62813|c0_g1_i1	No	(CT)8	16	--	--	--	--	--	--	TGATAGAAGATCCCGTTGGC	60	GGGAAGAGAGGAGGGAGAGA	60	253	--	--	--	--	--	--
TR62813|c0_g1_i2	No	(CT)8	16	--	--	--	--	--	--	TGATAGAAGATCCCGTTGGC	60	GGGAAGAGAGGAGGGAGAGA	60	253	--	--	--	--	--	--
TR62813|c0_g1_i3	No	(CT)8	16	--	--	--	--	--	--	TGATAGAAGATCCCGTTGGC	60	GGGAAGAGAGGAGGGAGAGA	60	253	--	--	--	--	--	--
TR62813|c0_g1_i4	No	(CT)8	16	--	--	--	--	--	--	TGATAGAAGATCCCGTTGGC	60	GGGAAGAGAGGAGGGAGAGA	60	253	--	--	--	--	--	--
TR62813|c0_g1_i6	No	(TTA)5	15	--	--	--	--	--	--	TGCAGGCAATCTTAATGCAC	60	GGTCAAAATCAATTTCAACTCAA	58	247	--	--	--	--	--	--
TR62816|c0_g1_i1	XLOC_026089	(CT)10	20	--	--	--	--	--	--	AGCTACTTCAACTCTGCCGC	60	TGTTGCTTCCGAAACTCCTT	60	165	--	--	--	--	--	--
TR62819|c0_g1_i1	No	(TCT)5	15	--	--	--	--	--	--	TTCACTCGCTATTCCATCCTTT	60	CGGTTAGTGGTGATGGCTTT	60	134	--	--	--	--	--	--
TR62819|c0_g1_i3	No	(TCT)5	15	--	--	--	--	--	--	TTCACTCGCTATTCCATCCTTT	60	CGGTTAGTGGTGATGGCTTT	60	134	--	--	--	--	--	--
TR62819|c0_g1_i4	No	(TCT)5	15	--	--	--	--	--	--	TTCACTCGCTATTCCATCCTTT	60	CGGTTAGTGGTGATGGCTTT	60	134	--	--	--	--	--	--

Transcript IDs	Differentially expressed gene (DEG) IDs	SSR repeat-motifs	SSR repeat-length (bp)	*In silico* polymorphic SSRs						Forward primers (5'-3')	Annealing tempearture(0C)	Reverse primers (5'-3')	Annealing tempearture(0C)	Amplified product size (bp)	Structural annotation		Functional Annotation			
				C. microphyllum		ICC4958														
				Transcript	SSR repeat-motifs	Chromosomes	SSR repeat-motifs	Start physical positions (bp)	End physical positions (bp)						Gene IDs	Sequence component of genes	NCBI nr IDs	TFs	KOG	KEGG
TR62858|c0_g1_i1	XLOC_026133	(TAC)6taatactacagg(TTC)5	45	--	--	--	--	--	--	GAACCAACAACATCTTTGGTGA	60	CACCAGCAGAAGAAGCAACA	60	208	--	--	XP_004486508.1	WRKY	[KT]	--
TR62858|c0_g1_i1	XLOC_026133	(ACA)6	18	--	--	--	--	--	--	GTGGTGGAGCTGAACCTCTT	59	CCAAAGATGTTGTTGGTTCCT	59	163	--	--	XP_004486508.1	WRKY	[KT]	--
TR62858|c0_g1_i1	XLOC_026133	(ACA)8	24	--	--	--	--	--	--	TTCAACCCCTCAAAAACCTG	60	GGTTCAGCTCCACCACTAGC	60	280	--	--	XP_004486508.1	WRKY	[KT]	--
TR62863|c1_g2_i1	No	(AG)7	14	--	--	--	--	--	--	TTGGGACAACAAGTGATAGGTG	60	CCAACAGCTTCTTTGCTCCT	60	206	--	--	--	--	--	--
TR62863|c1_g2_i2	No	(AG)7	14	--	--	--	--	--	--	TTGGGACAACAAGTGATAGGTG	60	CCAACAGCTTCTTTGCTCCT	60	206	--	--	--	--	--	--
TR62878|c0_g2_i1	No	(AT)10	20	--	--	--	--	--	--	TCACAAATGGTTGAAATGGC	59	AACTGAAAATTCATTGGGTGA	57	271	--	--	--	--	--	--
TR6288|c0_g1_i1	XLOC_026174	(TC)7	14	--	--	--	--	--	--	TTTTGTTCTTTCATTCATTCTCTCC	60	CGAGATTCCGGTAAGGTGAA	60	121	--	--	--	--	--	--
TR62883|c0_g1_i1	XLOC_026169	(AAC)5	15	--	--	--	--	--	--	GCAACAGCGCAAAGTAATGA	60	ACATGCGATGCGACTAAATG	60	249	--	--	--	--	--	--
TR62883|c0_g3_i1	No	(AAC)5	15	--	--	--	--	--	--	GCAACAGCGCAAAGTAATGA	60	ACATGCGATGCGACTAAATG	60	249	--	--	--	--	--	--
TR6300|c0_g1_i1	No	(AAT)7	21	--	--	--	--	--	--	ATTCTTGGTGCGACTCCATC	60	CCAGCACCAGATGGAAGAAT	60	215	--	--	--	--	--	--
TR6300|c0_g2_i1	No	(AAT)7	21	--	--	--	--	--	--	ATTCTTGGTGCGACTCCATC	60	CCAGCACCAGATGGAAGAAT	60	215	--	--	--	--	--	--
TR63027|c0_g1_i1	XLOC_026209	(GGA)7	21	--	--	--	--	--	--	CGAAGAACGCAGAGATTTCC	60	CTCGGAACCTTTCTGTGGAG	60	266	--	--	--	--	--	--
TR63178|c0_g1_i1	No	(AGA)6	18	--	--	--	--	--	--	GGAAAGGTTCACGGAGGACT	60	GAGCATCCTCTTCTTGGCAC	60	114	--	--	--	--	--	--
TR63194|c0_g1_i1	No	(TTA)5	15	--	--	--	--	--	--	TCCAATGGAAGCATCATCAA	60	CCACATGCATAATCCAATGC	60	171	--	--	--	--	--	--

Transcript IDs	Differentially expressed gene (DEG) IDs	SSR repeat-motifs	SSR repeat-length (bp)	*In silico* polymorphic SSRs						Forward primers (5'-3')	Annealing tempearture(0C)	Reverse primers (5'-3')	Annealing tempearture(0C)	Amplified product size (bp)	Structural annotation		Functional Annotation			
				C. microphyllum		ICC4958														
				Transcript	SSR repeat-motifs	Chromosomes	SSR repeat-motifs	Start physical positions (bp)	End physical positions (bp)						Gene IDs	Sequence component of genes	NCBI nr IDs	TFs	KOG	KEGG
TR63194|c0_g2_i1	No	(TTA)5	15	--	--	--	--	--	--	TCCAATGGAAGCATCATCAA	60	CCACATGCATAATCCAATGC	60	171	--	--	--	--	--	--
TR63219|c0_g1_i1	No	(AC)7	14	--	--	--	--	--	--	ACTTGTATCTCCCCTGGCCT	60	TCGTTAGGTCGTTCCAAACC	60	245	--	--	--	--	--	--
TR63264|c0_g1_i1	XLOC_026297	(CAT)7	21	--	--	--	--	--	--	TTCACTCTTTCCCTTCAATTCC	60	CCAATCAAAATCTTCCATTTCC	60	225	--	--	--	--	--	--
TR63266|c0_g1_i1	No	(AAG)5	15	--	--	--	--	--	--	CAATGCCATCACCAGAAGAA	60	CCAACCTCATTTTCCCTTCA	60	264	--	--	--	--	--	--
TR63306|c0_g1_i1	XLOC_026311	(CT)6	12	--	--	--	--	--	--	TTAATGGCTTCTTGACCAGG	58	AGACCGATTGATGAACGACC	60	280	--	--	XM_004494275.1	--	--	--
TR63343|c0_g1_i1	No	(A)11ggaataaataaatgaattctacaataagtatttattctatttatatatctgtgaaataaacaaaataggaaaaacc(A)13	100	--	--	--	--	--	--	GCCAAAACTGATAACAGCAAAA	59	CGTATTAAACGCCTTTGTTGG	60	209	--	--	--	--	--	--
TR63343|c0_g2_i1	No	(A)11ggaataaataaatgaattctacaataagtatttattctatttatatatctgtgaaataaacaaaataggaaaaacc(A)13	100	--	--	--	--	--	--	GCCAAAACTGATAACAGCAAAA	59	CGTATTAAACGCCTTTGTTGG	60	209	--	--	--	--	--	--
TR63343|c0_g3_i1	No	(A)11ggaataaataaatgaattctacaataagtatttattctatttatatatctgtgaaataaacaaaataggaaaaacc(A)13	100	--	--	--	--	--	--	GCCAAAACTGATAACAGCAAAA	59	CGTATTAAACGCCTTTGTTGG	60	209	--	--	--	--	--	--
TR63348|c0_g1_i1	No	(TG)8	16	--	--	--	--	--	--	AGGGTGAAAAGCAGTCATGG	60	TGTTCCCATAGTCCGATTCC	60	207	--	--	--	--	--	--
TR63348|c0_g2_i1	No	(TG)8	16	--	--	--	--	--	--	AGGGTGAAAAGCAGTCATGG	60	TGTTCCCATAGTCCGATTCC	60	207	--	--	--	--	--	--
TR63351|c0_g1_i1	XLOC_026330	(CCA)5	15	--	--	--	--	--	--	ATGACCCTGGATGCTTTGAC	60	GGGTGCAAATAGTGGTGGAC	60	135	--	--	--	--	--	--
TR63392|c0_g1_i1	XLOC_026357	(TG)8	16	--	--	--	--	--	--	CAGACAGGCGAGGAAGACTC	60	TCTTCACATGCAAAGCCAAG	60	130	--	--	XP_003048793.1	--	--	--
TR63417|c0_g2_i1	No	(T)10a(T)10	21	--	--	--	--	--	--	AGTGGCGAAGAGAACGAGAA	60	TGAAACTCTCACCCACCCTT	60	231	--	--	--	--	--	--
TR63579|c0_g1_i1	No	(GGT)5	15	--	--	--	--	--	--	AAAGAAATGCGCTCGTCAAT	60	CTTTTCTTCATCAGCACCAGT	57	229	--	--	--	--	--	--

Transcript IDs	Differentially expressed gene (DEG) IDs	SSR repeat-motifs	SSR repeat-length (bp)	*In silico* polymorphic SSRs						Forward primers (5'-3')	Annealing tempearture(0C)	Reverse primers (5'-3')	Annealing tempearture(0C)	Amplified product size (bp)	Structural annotation		Functional Annotation			
				C. microphyllum		ICC4958									Gene IDs	Sequence component of genes	NCBI nr IDs	TFs	KOG	KEGG
				Transcript	SSR repeat-motifs	Chromosomes	SSR repeat-motifs	Start physical positions (bp)	End physical positions (bp)											
TR63601|c0_g1_i1	No	(TCA)6	18	--	--	--	--	--	--	TGGTGGAATGAGCAAAAATG	60	TGAAGGGAGGCCTATGTCTG	60	223	--	--	--	--	--	--
TR63618|c0_g1_i1	No	(A)11tcaacacacgcgctctttacttgtcg(A)10	47	--	--	--	--	--	--	AAACCGAGAACAAGTGGGAA	60	TTACTTGTTGAAAGAGCGCGT	60	159	--	--	--	--	--	--
TR63660|c0_g1_i1	No	(TCA)5	15	--	--	--	--	--	--	TTTGCCAATTCCAATTCCAT	60	ACTCGATCACCACCAACCTC	60	124	--	--	--	--	--	--
TR63850|c0_g2_i1	No	(TCA)6	18	--	--	--	--	--	--	GCAATTGGAATGGAACCCTA	60	ATGGTGATTTACCAACCCCA	60	221	--	--	--	--	--	--
TR63883|c0_g1_i1	XLOC_026508	(TCG)5	15	--	--	--	--	--	--	TCCAAGAGTAGGGGTTGTGC	60	GAGCAGTACAGCTTGAGGGC	60	271	--	--	XP_001940792.1	--	[OE]	--
TR63886|c0_g1_i2	No	(T)11cactccaaaattttcagcctcaaaatcactactatggtactcactctctc(T)10	71	--	--	--	--	--	--	CAACTTTTGCTGCACCTTGA	60	TTTGAAAGCTCTCTCTCTCAATTTT	59	274	--	--	--	--	--	--
TR63991|c0_g1_i1	No	(TGA)5	15	--	--	--	--	--	--	TGGTGAAAGATGATTTTTGTGG	60	AACGGTATGGTGAACGATTTG	60	269	--	--	--	--	--	--
TR64019|c0_g1_i1	XLOC_026570	(CT)7	14	--	--	--	--	--	--	TCCGAATCAAAATGTGTTCAA	59	TACGTTTGACATGCACACCA	60	158	--	--	--	--	--	--
TR64020|c0_g1_i1	XLOC_026571	(A)11cctcttcgggacggataatccggagggtgacacgaaccaggtccatttttttactttgtctttgtcctgtc(T)10	91	--	--	--	--	--	--	CAGAAAACAAGAGGAACACAAGG	60	AGCGAACTTGATTTAGCCCA	60	267	--	--	--	--	--	--
TR64036|c0_g1_i1	No	(TTG)5	15	--	--	--	--	--	--	GGGGAGGTTTCCACTTCTCT	60	CCGCAGACGATCATGAGATA	60	227	--	--	--	--	--	--
TR64063|c0_g1_i1	XLOC_026580	(GTG)6	18	--	--	--	--	--	--	TCGGGATCAGGAGGTAGAAG	59	CCATAACCCTCACCATGACC	60	101	--	--	--	--	--	--
TR64095|c0_g1_i1	No	(TCT)5	15	--	--	--	--	--	--	TCTCCGAGGACGAGAAAGAA	60	TTTCGTGAACGACAAGAACG	60	277	--	--	--	--	--	--
TR64095|c0_g2_i1	No	(TCT)5	15	--	--	--	--	--	--	TCTCCGAGGACGAGAAAGAA	60	TTTCGTGAACGACAAGAACG	60	276	--	--	--	--	--	--
TR64132|c0_g1_i1	XLOC_026612	(CTT)5	15	--	--	--	--	--	--	GCAAATCATTGGAAGTGGCT	60	ATGCCAACAGCCAGGATTAC	60	196	--	--	--	--	--	--

Transcript IDs	Differentially expressed gene (DEG) IDs	SSR repeat-motifs	SSR repeat-length (bp)	*In silico* polymorphic SSRs						Forward primers (5'-3')	Annealing tempearture(0C)	Reverse primers (5'-3')	Annealing tempearture(0C)	Amplified product size (bp)	Structural annotation		Functional Annotation			
				C. microphyllum		**ICC4958**									**Gene IDs**	**Sequence component of genes**	**NCBI nr IDs**	**TFs**	**KOG**	**KEGG**
				**Transcript**	**SSR repeat-motifs**	**Chromosomes**	**SSR repeat-motifs**	**Start physical positions (bp)**	**End physical positions (bp)**											
TR64139|c0_g1_i1	XLOC_026616	(AG)6	12	--	--	--	--	--	--	GATCAAGCAAAGAGGGGACA	60	GTCTGCACCAACCCAAAGTT	60	163	--	--	--	--	--	--
TR64339|c0_g1_i1	XLOC_026676	(CCA)7	21	--	--	--	--	--	--	GATGGTTGCCAAACCTCTTC	60	GGGAATAGAAGTGTTGTTGTTTTG	59	201	--	--	--	--	--	--
TR64371|c0_g1_i1	XLOC_026687	(AT)6	12	--	--	--	--	--	--	GCTTGCTGTTGTGTGTTGGT	60	ATCACCACTGGAAAAGCCAC	60	276	--	--	--	--	--	--
TR64475|c0_g1_i1	XLOC_026715	(AG)7	14	TR64475|c0_g1_i1	(AG)7	scaffold05086	(AG)42	6845	6928	CCTCATGTTGATCTCATTCTTTTCT	60	AAGGCTGCAAAACTAGAGCA	58	159	Ca_22798	DRR	--	--	--	--
TR64692|c0_g1_i1	No	(GAC)5	15	--	--	--	--	--	--	GCGAGCTCGACAACTACCTC	60	CCGATGTGAAAGTGCTGCTA	60	276	--	--	--	--	--	--
TR64774|c0_g2_i1	XLOC_026801	(AATG)5	20	--	--	--	--	--	--	CCGAAAAGACATGCGAAAAT	60	CGTGTGACTCGATTTATTTCCA	60	278	--	--	--	--	--	--
TR64782|c0_g1_i1	No	(CT)6	12	--	--	--	--	--	--	AGTTCTACATTTTGGCGGAA	57	TAGAACAGGCGACTGCATGA	61	278	--	--	--	--	--	--
TR64782|c0_g1_i2	No	(CT)6	12	--	--	--	--	--	--	AGTTCTACATTTTGGCGGAA	57	TAGAACAGGCGACTGCATGA	61	278	--	--	--	--	--	--
TR64809|c0_g1_i2	No	(TC)7	14	--	--	--	--	--	--	ACCATCACCACCTTCTCCTG	60	GGTGGATCAATAGCAAGGGA	60	192	--	--	--	--	--	--
TR64809|c0_g1_i3	No	(TC)7	14	--	--	--	--	--	--	ACCATCACCACCTTCTCCTG	60	GGTGGATCAATAGCAAGGGA	60	192	--	--	--	--	--	--
TR64809|c0_g1_i4	No	(TC)7	14	--	--	--	--	--	--	ACCATCACCACCTTCTCCTG	60	GGTGGATCAATAGCAAGGGA	60	192	--	--	--	--	--	--
TR64809|c0_g1_i5	No	(TC)7	14	--	--	--	--	--	--	ACCATCACCACCTTCTCCTG	60	GGTGGATCAATAGCAAGGGA	60	192	--	--	--	--	--	--
TR64809|c0_g1_i6	No	(TC)7	14	--	--	--	--	--	--	ACCATCACCACCTTCTCCTG	60	GGTGGATCAATAGCAAGGGA	60	192	--	--	--	--	--	--
TR64809|c0_g1_i7	No	(TC)7	14	--	--	--	--	--	--	ACCATCACCACCTTCTCCTG	60	GGTGGATCAATAGCAAGGGA	60	192	--	--	--	--	--	--

Transcript IDs	Differentially expressed gene (DEG) IDs	SSR repeat-motifs	SSR repeat-length (bp)	*In silico* polymorphic SSRs						Forward primers (5'-3')	Annealing tempearture(0C)	Reverse primers (5'-3')	Annealing tempearture(0C)	Amplified product size (bp)	Structural annotation		Functional Annotation			
				C. microphyllum		ICC4958									Gene IDs	Sequence component of genes	NCBI nr IDs	TFs	KOG	KEGG
				Transcript	SSR repeat-motifs	Chromosomes	SSR repeat-motifs	Start physical positions (bp)	End physical positions (bp)											
TR64816|c0_g1_i1	XLOC_026835	(AT)7	14	--	--	--	--	--	--	TAGCTGGCAATGGCTTCTTT	60	CTTTGCAAACACAAACCCG	60	169	--	--	--	--	--	--
TR64818|c0_g1_i1	No	(CT)6	12	TR64818|c0_g1_i1	(CT)6	Ca_LG_4	(CT)10	22259502	22259521	CCTCAAATACCACCCTCACAA	60	TGTTGATGGCATTGAAGCAT	60	278	Ca_03586	CDS	--	--	--	--
TR64818|c0_g2_i1	No	(CT)6	12	--	--	--	--	--	--	CCTCAAATACCACCCTCACAA	60	TGCAAACATTTTCACAGCAGA	60	173	--	--	--	--	--	--
TR64831|c0_g1_i1	XLOC_026850	(CCA)5	15	--	--	--	--	--	--	CTCTCCTCCTGCACCATAGC	60	GGTGGTGGTGGAGAATATGG	60	279	--	--	--	--	--	--
TR64834|c0_g2_i1	No	(GAAA)5	20	--	--	--	--	--	--	TTCCAAAATGTAGGACCCGA	60	CTTTGCACGTCATTACCCCT	60	161	--	--	--	--	--	--
TR64838|c1_g1_i1	XLOC_026861	(AG)11	22	--	--	--	--	--	--	AAGCATCAGAAGAATCAGACAGG	60	CAACTACCACTTCAACACCTTCC	60	216	--	--	--	--	--	--
TR64851|c0_g1_i1	XLOC_026878	(AT)6	12	--	--	--	--	--	--	AGTCAACAACACCATTCTTCCA	59	TGGTTCCATCCTTTTTAGTGG	59	266	--	--	--	--	--	--
TR64861|c0_g1_i1	XLOC_026888	(TA)7	14	--	--	--	--	--	--	GTCTCAAGGACTTTGACCGC	60	GCAAATATAGGCCGTTCCCT	60	248	--	--	--	--	--	--
TR64861|c0_g2_i1	No	(TCA)5	15	--	--	--	--	--	--	TATCCAACCACATGGACCAA	60	GTGGGAAAACAGCCGTAAAA	60	138	--	--	--	--	--	--
TR64861|c0_g3_i1	No	(TCA)5	15	--	--	--	--	--	--	TATCCAACCACATGGACCAA	60	GTGGGAAAACAGCCGTAAAA	60	138	--	--	--	--	--	--
TR64883|c1_g1_i1	No	(TTTC)5	20	--	--	--	--	--	--	CACACACTCTGTCGCTGCTT	60	TCAAAAGGGGTCGGTTACTG	60	116	--	--	--	--	--	--
TR64883|c1_g1_i2	No	(TTTC)5	20	--	--	--	--	--	--	CACACACTCTGTCGCTGCTT	60	TCAAAAGGGGTCGGTTACTG	60	116	--	--	--	--	--	--
TR64883|c1_g1_i3	No	(TTTC)5	20	--	--	--	--	--	--	CACACACTCTGTCGCTGCTT	60	TCAAAAGGGGTCGGTTACTG	60	116	--	--	--	--	--	--
TR64894|c1_g1_i1	No	(TC)8	16	--	--	--	--	--	--	ATTCCCCACCTTCTTTGCTT	60	TTGGAGGGGTATTGAGCTTG	60	133	--	--	--	--	--	--

Transcript IDs	Differentially expressed gene (DEG) IDs	SSR repeat-motifs	SSR repeat-length (bp)	*In silico* polymorphic SSRs						Forward primers (5'-3')	Annealing tempearture(0C)	Reverse primers (5'-3')	Annealing tempearture(0C)	Amplified product size (bp)	Structural annotation		Functional Annotation			
				C. microphyllum		ICC4958									Gene IDs	Sequence component of genes	NCBI nr IDs	TFs	KOG	KEGG
				Transcript	SSR repeat-motifs	Chromosomes	SSR repeat-motifs	Start physical positions (bp)	End physical positions (bp)											
TR64894|c1_g1_i1	No	(T)12ggtaatttggtcctctctgatattgaagagtccgtcttctcttgtggcacattaaagaaagca(T)10(TC)6*	96	--	--	--	--	--	--	GAGAGTAGTGGTGGTGCCGT	60	AAGCAAAGAAGGTGGGGAAT	60	253	--	--	--	--	--	--
TR64894|c1_g2_i2	No	(TC)8	16	--	--	--	--	--	--	ATTCCCCACCTTCTTTGCTT	60	TTCCTTCTTGCCCATTTTTG	60	171	--	--	--	--	--	--
TR64894|c1_g2_i2	No	(T)12ggtaatttggtcctctctgatattgaagagtccgtcttctcttgtggcacattaaagaaagca(T)10(TC)6*	96	--	--	--	--	--	--	GAGAGTAGTGGTGGTGCCGT	60	AAGCAAAGAAGGTGGGGAAT	60	253	--	--	--	--	--	--
TR64894|c1_g2_i3	No	(TC)8	16	--	--	--	--	--	--	ATTCCCCACCTTCTTTGCTT	60	TTCCTTCTTGCCCATTTTTG	60	171	--	--	--	--	--	--
TR64894|c1_g2_i3	No	(T)12ggtaatttggtcctctctgatattgaagagtccgtcttctcttgtggcacattaaagaaagca(T)10(TC)6*	96	--	--	--	--	--	--	GAGAGTAGTGGTGGTGCCGT	60	AAGCAAAGAAGGTGGGGAAT	60	253	--	--	--	--	--	--
TR64896|c0_g1_i1	No	(ATCCCA)7atcttatttcatttcctactcaacagtcaacaccaaa(AC)8	95	--	--	--	--	--	--	TAACGCCGCGTAAAGAACT	59	GGAAGAGGGAGGGAAAGTGA	61	197	--	--	--	--	--	--
TR64896|c0_g1_i2	No	(ATCCCA)5atcttatttcatttcctactcaacagtcaacaccaaa(AC)8	83	--	--	--	--	--	--	TAACGCCGCGTAAAGAACT	59	GGAAGAGGGAGGGAAAGTGA	61	185	--	--	--	--	--	--
TR64896|c0_g1_i3	No	(ATCCCA)7atcttatttcatttcctactcaacagtcaacaccaaa(AC)8	95	--	--	--	--	--	--	TAACGCCGCGTAAAGAACT	59	GGAAGAGGGAGGGAAAGTGA	61	197	--	--	--	--	--	--
TR64896|c0_g1_i4	No	(ATCCCA)5atcttatttcatttcctactcaacagtcaacaccaaa(AC)8	83	--	--	--	--	--	--	TAACGCCGCGTAAAGAACT	59	GGAAGAGGGAGGGAAAGTGA	61	185	--	--	--	--	--	--
TR64912|c2_g1_i1	No	(TC)6	12	--	--	--	--	--	--	TCTTCCCTCTCCTTCCAACA	60	TGGGTTGGAGGAGTGTTTTC	60	196	--	--	--	--	--	--
TR64915|c0_g2_i1	No	(ATAA)5	20	--	--	--	--	--	--	TCGTGTGGACCTTGTTCAAA	60	TCAGCAATATTGTGCTTCTGG	59	240	--	--	--	--	--	--
TR64946|c0_g1_i1	XLOC_026965	(TTC)5	15	--	--	--	--	--	--	CAAATCCATACTCACCCGCT	60	GGTGGAGCTGAGGAAATTCA	60	226	--	--	--	--	--	--
TR64949|c3_g1_i1	No	(TC)7	14	--	--	--	--	--	--	ACAAATCCCTTTCCCCACTT	60	TTGGAAGCTTCACGATGTTG	60	168	--	--	--	--	--	--
TR64949|c3_g1_i1	No	(ACA)7	21	--	--	--	--	--	--	CATGGAAAAGCAACATCGTG	60	TGAAGAACCCCCAAACTCAG	60	271	--	--	--	--	--	--

Transcript IDs	Differentially expressed gene (DEG) IDs	SSR repeat-motifs	SSR repeat-length (bp)	*In silico* polymorphic SSRs						Forward primers (5'-3')	Annealing tempearture(0C)	Reverse primers (5'-3')	Annealing tempearture(0C)	Amplified product size (bp)	Structural annotation		Functional Annotation			
				C. microphyllum		ICC4958														
				Transcript	SSR repeat-motifs	Chromosomes	SSR repeat-motifs	Start physical positions (bp)	End physical positions (bp)						Gene IDs	Sequence component of genes	NCBI nr IDs	TFs	KOG	KEGG
TR64949|c3_g1_i1	No	(GTG)5	15	--	--	--	--	--	--	TCAACTGAGTTTGGGGGTTC	60	TCTCTCTTGGTCCCTCCTGA	60	191	--	--	--	--	--	--
TR64949|c3_g1_i2	No	(TC)7	14	--	--	--	--	--	--	ACAAATCCCTTTCCCCACTT	60	TTGGAAGCTTCACGATGTTG	60	168	--	--	--	--	--	--
TR64949|c3_g1_i2	No	(ACA)7	21	--	--	--	--	--	--	CATGGAAAAGCAACATCGTG	60	TGAAGAACCCCCAAACTCAG	60	271	--	--	--	--	--	--
TR64949|c3_g1_i2	No	(GTG)5	15	--	--	--	--	--	--	TCAACTGAGTTTGGGGGTTC	60	TCTCTCTTGGTCCCTCCTGA	60	191	--	--	--	--	--	--
TR64958|c1_g2_i2	No	(CCGAGT)5	30	--	--	--	--	--	--	TGAATTGAATTTGCGTCGAA	60	CACAAGAGTGTTTGTGCCGT	60	121	--	--	--	--	--	--
TR64958|c1_g2_i3	No	(CCGAGT)5	30	--	--	--	--	--	--	TGAATTGAATTTGCGTCGAA	60	CACAAGAGTGTTTGTGCCGT	60	121	--	--	--	--	--	--
TR64958|c1_g2_i4	No	(CCGAGT)5	30	--	--	--	--	--	--	TGAATTGAATTTGCGTCGAA	60	CACAAGAGTGTTTGTGCCGT	60	121	--	--	--	--	--	--
TR64958|c1_g2_i6	No	(CCGAGT)5	30	--	--	--	--	--	--	TGAATTGAATTTGCGTCGAA	60	CACAAGAGTGTTTGTGCCGT	60	121	--	--	--	--	--	--
TR64960|c0_g1_i1	XLOC_026980	(TCA)5	15	--	--	--	--	--	--	CCACTTTTGCCACCACTTTT	60	TCCTTCTTTGGGACCAGATG	60	267	--	--	--	--	--	--
TR64993|c0_g2_i1	XLOC_027008	(GTG)5	15	--	--	--	--	--	--	GGGGGTGAAACAGAATCAGA	60	CTGTTCAGGGTTCGGTTGAT	60	271	--	--	--	--	--	--
TR65010|c0_g1_i1	XLOC_027017	(CCAT)5	20	--	--	--	--	--	--	CACCCCTCGCTCACTTGTAT	60	GGGACATTGCTATTCGTCGT	60	205	--	--	--	--	--	--
TR65059|c0_g1_i1	XLOC_027054	(CAA)6	18	--	--	--	--	--	--	CCATGACTGCTTCTACTGACCA	60	CATTACCACCCTTTGGAGTCA	60	225	--	--	XP_004487911.1	--	--	--
TR65062|c0_g1_i1	XLOC_027056	(AG)9	18	--	--	--	--	--	--	GGGAAATCAAGACGAGACGA	60	TGGTGGGTAATGGGTATTGG	60	108	--	--	--	--	--	--
TR65137|c0_g1_i1	No	(TTG)5	15	--	--	--	--	--	--	CGTATCCTGGACGACGAGTT	60	TCACTCCAATTACAAGACCAACA	60	174	--	--	--	--	--	--

Transcript IDs	Differentially expressed gene (DEG) IDs	SSR repeat-motifs	SSR repeat-length (bp)	*In silico* polymorphic SSRs						Forward primers (5'-3')	Annealing tempearture(0C)	Reverse primers (5'-3')	Annealing tempearture(0C)	Amplified product size (bp)	Structural annotation		Functional Annotation			
				C. microphyllum		ICC4958									Gene IDs	Sequence component of genes	NCBI nr IDs	TFs	KOG	KEGG
				Transcript	SSR repeat-motifs	Chromosomes	SSR repeat-motifs	Start physical positions (bp)	End physical positions (bp)											
TR65137|c0_g1_i1	No	(GAT)5	15	--	--	--	--	--	--	GGTGTGACTGGAGGAGCAAT	60	AACAACCACAAGCACCACAA	60	234	--	--	--	--	--	--
TR65256|c0_g1_i1	XLOC_027121	(GAA)5	15	--	--	--	--	--	--	CCTCCTCTTCCTCTTCCGAC	60	TCTCTGCGGAGCTTTTGAAT	60	216	--	--	--	--	--	--
TR65256|c0_g2_i1	No	(GAA)5	15	--	--	--	--	--	--	CCTCCTCTTCCTCTTCCGAC	60	TCTCTGCGGAGCTTTTGAAT	60	216	--	--	--	--	--	--
TR65274|c0_g1_i1	XLOC_027132	(CT)6	12	--	--	--	--	--	--	TGAAATTCCATTCCTCACTCG	60	ATCACACACTTCCCGTCACA	60	251	--	--	--	--	--	--
TR65312|c0_g1_i1	XLOC_027159	(AG)6	12	--	--	--	--	--	--	ACTTCTTGCCCACGAGAGAG	60	CCCCTCCTTGTGTTGTGATT	60	151	--	--	XP_008813767.1	--	[O]	--
TR65312|c2_g1_i1	No	(ACA)5aacaatacagcatagaacaacacag(A)11	51	--	--	--	--	--	--	ACATCATGTTTGTGAACCAATC	57	TTTCCTCCCCACTTTCCTCT	60	132	--	--	--	--	--	--
TR65326|c0_g1_i1	XLOC_027172	(TC)9	18	--	--	--	--	--	--	AATCACGCTTGCTCCCTCT	60	CCGCATGCTGTTTTATCCTT	60	152	--	--	--	--	--	--
TR65329|c0_g1_i1	XLOC_027175	(TGA)8	24	--	--	--	--	--	--	TTCCTTCGTTCCCTTTGTTG	60	AAAACCCTTTTGATTTGGGC	60	144	--	--	--	--	--	--
TR65397|c0_g1_i1	XLOC_027227	(TCC)5	15	--	--	--	--	--	--	AAAAACCAAACAAAGTGGCG	60	CTGTTGAGTGGCGGATAGGT	60	249	--	--	--	--	--	--
TR65497|c0_g1_i1	XLOC_027263	(TTC)5	15	--	--	--	--	--	--	AGCCAAAACTTTGGTGAAAGA	59	AAATCACCCGAATTGCAGAC	60	150	--	--	--	--	--	--
TR65497|c0_g2_i1	No	(TTC)5	15	--	--	--	--	--	--	AGCCAAAACTTTGGTGAAAGA	59	AAATCACCCGAATTGCAGAC	60	150	--	--	--	--	--	--
TR65518|c0_g1_i1	XLOC_027284	(AG)7	14	--	--	--	--	--	--	GGTAAACTAACAAACCATCTCATTG	58	ATGATGAGCAAACACGTCCA	60	146	--	--	--	--	--	--
TR65529|c1_g1_i1	No	(AG)6	12	--	--	--	--	--	--	TGTTGTTCTTGTGTTTTGCCA	60	ATGGATCGAACGTTGGAGAG	60	235	--	--	--	--	--	--
TR65529|c1_g1_i3	No	(AG)6	12	--	--	--	--	--	--	GGCAAAACACAAGAACAACAAA	60	ATGGATCGAACGTTGGAGAG	60	241	--	--	--	--	--	--

Transcript IDs	Differentially expressed gene (DEG) IDs	SSR repeat-motifs	SSR repeat-length (bp)	*In silico* polymorphic SSRs						Forward primers (5'-3')	Annealing tempearture(0C)	Reverse primers (5'-3')	Annealing tempearture(0C)	Amplified product size (bp)	Structural annotation		Functional Annotation			
				C. microphyllum		ICC4958									Gene IDs	Sequence component of genes	NCBI nr IDs	TFs	KOG	KEGG
				Transcript	SSR repeat-motifs	Chromosomes	SSR repeat-motifs	Start physical positions (bp)	End physical positions (bp)											
TR65536|c2_g1_i1	XLOC_027300	(AG)9	18	--	--	--	--	--	--	GCAGACAACGAACGAAGACA	60	TCCAATGAACCTTTTCACGA	59	131	--	--	--	--	--	--
TR65536|c2_g1_i1	XLOC_027300	(GT)6	12	--	--	--	--	--	--	GCAGACAACGAACGAAGACA	60	GCATTGCATTGCATTCATAC	58	253	--	--	--	--	--	--
TR65540|c0_g1_i1	XLOC_027304	(AG)6	12	--	--	--	--	--	--	GGTTGATCTGATGGTGAGCA	60	CTCAGGTTGCCTGTCCTCTC	60	138	--	--	--	--	--	--
TR65543|c1_g1_i1	XLOC_027309	(GAA)5	15	--	--	--	--	--	--	CTTCCTTAGAGCCCAAACCC	60	TCTCCCTCTCGGTTACCTCA	60	226	--	--	--	--	--	--
TR65543|c1_g1_i1	XLOC_027309	(TTA)7	21	--	--	--	--	--	--	TGAGGTAACCGAGAGGGAGA	60	GGATATATGACACGCCCCGT	62	128	--	--	--	--	--	--
TR65546|c0_g1_i1	XLOC_027314	(CAT)5	15	--	--	--	--	--	--	TGAAGACAGAACGCACGAAG	60	ATTCCGTGATTTTTGCGAAG	60	249	--	--	--	--	--	--
TR65551|c0_g1_i1	No	(AAC)7	21	--	--	--	--	--	--	TGAACCAAACGATCATCGAA	60	TGTTACGGAACGTGAGCTTG	60	133	--	--	--	--	--	--
TR65551|c0_g2_i1	No	(AAC)7	21	--	--	--	--	--	--	TGAACCAAACGATCATCGAA	60	TGTTACGGAACGTGAGCTTG	60	133	--	--	--	--	--	--
TR65551|c1_g1_i1	XLOC_027317	(TGT)5	15	--	--	--	--	--	--	TCCCAAAAATAGAACAACCACTT	59	ACAAAGGTTGAAATGGGCAA	60	264	--	--	XM_004497533.1	Trihelix	[S]	--
TR65551|c1_g2_i1	No	(TTG)8	24	--	--	--	--	--	--	TCCTCTTCCCCTTCTCCATT	60	GAACAACAACACCACCACCA	60	145	--	--	--	--	--	--
TR65551|c4_g1_i1	No	(AAC)7	21	--	--	--	--	--	--	TACAAACCCAATATTCCCCC	58	CAGACACAGCTGAGACATGAAA	59	241	--	--	--	--	--	--
TR65551|c4_g1_i2	XLOC_027320	(AAC)7	21	--	--	--	--	--	--	TACAAACCCAATATTCCCCC	58	CAGACACAGCTGAGACATGAAA	59	241	--	--	--	--	--	--
TR65551|c4_g1_i3	No	(AAC)7	21	--	--	--	--	--	--	TACAAACCCAATATTCCCCC	58	CAGACACAGCTGAGACATGAAA	59	241	--	--	--	--	--	--
TR65551|c4_g1_i4	No	(AAC)7	21	--	--	--	--	--	--	TACAAACCCAATATTCCCCC	58	CAGACACAGCTGAGACATGAAA	59	241	--	--	--	--	--	--

Transcript IDs	Differentially expressed gene (DEG) IDs	SSR repeat-motifs	SSR repeat-length (bp)	*In silico* polymorphic SSRs						Forward primers (5'-3')	Annealing tempearture(0C)	Reverse primers (5'-3')	Annealing tempearture(0C)	Amplified product size (bp)	Structural annotation		Functional Annotation			
				C. microphyllum		ICC4958									Gene IDs	Sequence component of genes	NCBI nr IDs	TFs	KOG	KEGG
				Transcript	SSR repeat-motifs	Chromosomes	SSR repeat-motifs	Start physical positions (bp)	End physical positions (bp)											
TR65555|c0_g1_i2	No	(CT)6	12	--	--	--	--	--	--	ATCTGAGCTGAGCCATGGAG	61	TGTTCTTCCACACCAATGGA	60	261	--	--	--	--	--	--
TR65555|c0_g1_i3	XLOC_027324	(CT)6	12	--	--	--	--	--	--	ATCTGAGCTGAGCCATGGAG	61	TGTTCTTCCACACCAATGGA	60	261	--	--	--	--	--	--
TR65569|c0_g1_i2	No	(AG)6	12	--	--	--	--	--	--	TGTCTCCTCCAGAGTCAGCTT	59	AACACGCTCTAGATCCTCGG	59	108	--	--	--	--	--	--
TR65569|c0_g1_i3	No	(AG)6	12	--	--	--	--	--	--	TGTCTCCTCCAGAGTCAGCTT	59	TGGTGCTGACCATGATGTTT	60	144	--	--	--	--	--	--
TR65578|c1_g1_i2	No	(ATTT)5	20	--	--	--	--	--	--	CCCACTAAACCTTTGTGATGAA	59	CTCCCCAAACAGCCTTACAA	60	229	--	--	--	--	--	--
TR65578|c1_g7_i2	XLOC_027355	(ATTT)5	20	--	--	--	--	--	--	CCCACTAAACCTTTGTGATGAA	59	CTCCCCAAACAGCCTTACAA	60	229	--	--	--	--	--	--
TR65587|c0_g1_i1	No	(AC)6	12	--	--	--	--	--	--	TTGCTGCTTATCTGCAATGG	60	GCGGTAAGGAGGAAAAGAGG	60	196	--	--	--	--	--	--
TR65587|c0_g1_i2	No	(AC)6	12	--	--	--	--	--	--	TTGCTGCTTATCTGCAATGG	60	GCGGTAAGGAGGAAAAGAGG	60	196	--	--	--	--	--	--
TR65587|c0_g1_i4	No	(TG)7	14	--	--	--	--	--	--	GGTTGCACCATTTTGTCTTG	59	GCACAAGCCATTTCTTTCCT	59	257	--	--	--	--	--	--
TR65644|c0_g1_i1	XLOC_027396	(CTT)5	15	--	--	--	--	--	--	GGGTGCAGACAACGAAGATT	60	GAACCAAAGTATCGGCCAAA	60	275	--	--	--	--	--	--
TR6568|c0_g1_i1	No	(TGA)6	18	--	--	--	--	--	--	CATTGTGATCCATTCCACCA	60	CCTCCATGCTCCTCTTCTTG	60	268	--	--	--	--	--	--
TR65687|c0_g1_i1	XLOC_027409	(AC)7	14	TR65687|c0_g1_i1	(AC)7	Ca_LG_4	(AC)6	30711902	30711913	AATCGAACTGTGGTTCTCGG	60	AGCATGTTTTCGTGAGGACC	60	264		URR	--	--	--	--
TR65911|c0_g1_i1	XLOC_027471	(GT)8	16	TR65911|c0_g1_i1	(GT)8	Ca_LG_5	(GT)7	39446335	39446348	TGGGTGCAGTGTTGGTAAGA	60	AACAAGAGCATAAGCTACCAAGC	59	265	Ca_07177	URR	--	--	--	--
TR66050|c0_g1_i1	XLOC_027509	(ACA)5	15	--	--	--	--	--	--	GACGGATTCACGTTTGGAAT	60	TATATGAGCCGTTTGCGACA	60	274	--	--	--	--	--	--

Transcript IDs	Differentially expressed gene (DEG) IDs	SSR repeat-motifs	SSR repeat-length (bp)	*In silico* polymorphic SSRs						Forward primers (5'-3')	Annealing tempearture(0C)	Reverse primers (5'-3')	Annealing tempearture(0C)	Amplified product size (bp)	Structural annotation		Functional Annotation			
				C. microphyllum		ICC4958														
				Transcript	SSR repeat-motifs	Chromosomes	SSR repeat-motifs	Start physical positions (bp)	End physical positions (bp)						Gene IDs	Sequence component of genes	NCBI nr IDs	TFs	KOG	KEGG
TR66111|c0_g1_i1	No	(TA)6	12	--	--	--	--	--	--	CGGCATCCCATTATCTGCTA	61	TTTCCTTTTTCTCTCGGTCC	58	106	--	--	--	--	--	--
TR66139|c0_g1_i1	No	(GCT)5	15	--	--	--	--	--	--	GCGTCGATTTACCACCACTT	60	TGTACGAGAGCCTCATGGTG	60	144	--	--	--	--	--	--
TR6620|c0_g1_i1	XLOC_027552	(TA)6	12	--	--	--	--	--	--	CACCATCACAAAGACCACTCA	60	CGCTTGATTCCCCAAGAGTA	60	209	--	--	--	--	--	--
TR66244|c0_g2_i1	No	(TC)6	12	--	--	--	--	--	--	CAAACTTTTGCACTCATTGCTT	59	TCCTGCAAAAGCATAACAAGAA	60	245	--	--	--	--	--	--
TR66244|c2_g1_i1	No	(AAT)5	15	--	--	--	--	--	--	CGCATTCACTCTCTTGATTCTTT	60	GGACATGCATTCCCTTTCTT	59	147	--	--	--	--	--	--
TR66244|c2_g1_i3	No	(AAT)5	15	--	--	--	--	--	--	CGCATTCACTCTCTTGATTCTTT	60	GGACATGCATTCCCTTTCTT	59	147	--	--	--	--	--	--
TR66264|c0_g1_i3	No	(AC)7	14	--	--	--	--	--	--	AACTCCACGTGAATCGAAAAA	60	AAAAATGGAACCTACACAGAGAA A	58	276	--	--	--	--	--	--
TR66265|c0_g2_i1	No	(TCT)5	15	--	--	--	--	--	--	GCGTGTGTGAGGAAATGATG	60	AAAGCTGGTTGTTGGAGACG	60	151	--	--	--	--	--	--
TR66271|c0_g1_i1	No	(GT)7	14	--	--	--	--	--	--	GAACCATCCATCATTCACCC	60	CGTAACGTTGTTGGCTCTCTC	60	232	--	--	--	--	--	--
TR66272|c0_g1_i1	XLOC_027587	(CAT)7(CTT)6	39	--	--	--	--	--	--	ACGCCTCAATCCATCAAATC	60	CACGATTCATTCCCGTAGGT	60	269	--	--	--	--	--	--
TR66304|c0_g1_i2	No	(GCA)5	15	--	--	--	--	--	--	TCACAAGCAGGTGCAGATTC	60	CAAGACCTAAAGCGTTCCCA	60	151	--	--	--	--	--	--
TR66304|c0_g1_i3	No	(GCA)5	15	--	--	--	--	--	--	TCACAAGCAGGTGCAGATTC	60	CAAGACCTAAAGCGTTCCCA	60	151	--	--	--	--	--	--
TR66304|c0_g1_i7	No	(GCA)5	15	--	--	--	--	--	--	TCACAAGCAGGTGCAGATTC	60	CAAGACCTAAAGCGTTCCCA	60	151	--	--	--	--	--	--
TR66314|c0_g1_i1	XLOC_027628	(TAA)6	18	--	--	--	--	--	--	GCAAATTGCTTTGCTTTGAA	59	ATTCCCTTCCCCATATTGCT	60	280	--	--	--	--	--	--

Transcript IDs	Differentially expressed gene (DEG) IDs	SSR repeat-motifs	SSR repeat-length (bp)	*In silico* polymorphic SSRs						Forward primers (5'-3')	Annealing tempearture(0C)	Reverse primers (5'-3')	Annealing tempearture(0C)	Amplified product size (bp)	Structural annotation		Functional Annotation			
				C. microphyllum		ICC4958									Gene IDs	Sequence component of genes	NCBI nr IDs	TFs	KOG	KEGG
				Transcript	SSR repeat-motifs	Chromosomes	SSR repeat-motifs	Start physical positions (bp)	End physical positions (bp)											
TR66316|c0_g1_i2	No	(TTC)5	15	--	--	--	--	--	--	CAAAAGGACCCTTCCTGTCA	60	GGCAAGAGACCAAGAGCATC	60	243	--	--	--	--	--	--
TR66316|c0_g1_i3	No	(TTC)5	15	--	--	--	--	--	--	CAAAAGGACCCTTCCTGTCA	60	GGCAAGAGACCAAGAGCATC	60	243	--	--	--	--	--	--
TR66316|c0_g1_i6	No	(TTC)5	15	--	--	--	--	--	--	CAAAAGGACCCTTCCTGTCA	60	GGCAAGAGACCAAGAGCATC	60	243	--	--	--	--	--	--
TR66316|c0_g1_i7	No	(TTC)5	15	--	--	--	--	--	--	CAAAAGGACCCTTCCTGTCA	60	GGCAAGAGACCAAGAGCATC	60	243	--	--	--	--	--	--
TR66317|c2_g1_i1	XLOC_027631	(CT)8	16	--	--	--	--	--	--	CACGCGAGTATTTGACATCG	60	CAACGATTGTTGTCCCATCA	60	184	--	--	--	--	--	--
TR66328|c0_g1_i1	XLOC_027640	(GAA)5	15	--	--	--	--	--	--	CCAAGAACACTCCTCAAACACA	60	TGCGAGACCAGAAGAAAGGT	60	259	--	--	--	--	--	--
TR66335|c1_g1_i1	No	(CT)6	12	--	--	--	--	--	--	TAGATTCCCGGTATGGGTCA	60	ACGATTGCAAAAGAATTCGG	60	232	--	--	--	--	--	--
TR66367|c0_g1_i1	No	(AAG)5	15	--	--	--	--	--	--	CCAGACACTCCCGAGGTAAC	60	AACGTCACCTTCTTGTTGGG	60	117	--	--	--	--	--	--
TR66398|c0_g1_i1	No	(A)12gtacagacacgaagagagcgcgcaagtgc(A)10	51	--	--	--	--	--	--	CACAGGACGACCATTGAACA	61	GCTCCGAGGTCAAGTACGAA	60	197	--	--	--	--	--	--
TR66409|c0_g1_i1	No	(TGA)5	15	--	--	--	--	--	--	ACAGTGACTCCCAATACGGC	60	ACAGCCAGGCTTGCTTTAGA	60	257	--	--	--	--	--	--
TR66433|c0_g1_i1	XLOC_027660	(TG)6	12	--	--	--	--	--	--	AGGTTGCTGGTGAAATGGAG	60	AAGCTGGCTAGCAACAGCTC	60	147	--	--	--	--	--	--
TR6654|c0_g1_i1	No	(T)10cttcac(A)12	28	--	--	--	--	--	--	GGAGTATTCTCCCGAGGGTC	60	AATCGGTTCTTGCGAACG	60	100	--	--	--	--	--	--
TR66554|c0_g1_i1	XLOC_027674	(AAC)7	21	--	--	--	--	--	--	CCTAACCCAACTCCGAACCT	60	TGGTTTTCCTCTGCTGTGAA	59	168	--	--	--	--	--	--
TR66591|c0_g1_i1	XLOC_027677	(AAC)6	18	--	--	--	--	--	--	TGGCTACCAAAGAAATGGAAA	60	GACTATGAGAATGAAACATTTCTTGA	58	266	--	--	--	--	--	--

Transcript IDs	Differentially expressed gene (DEG) IDs	SSR repeat-motifs	SSR repeat-length (bp)	*In silico* polymorphic SSRs						Forward primers (5'-3')	Annealing tempearture(0C)	Reverse primers (5'-3')	Annealing tempearture(0C)	Amplified product size (bp)	Structural annotation		Functional Annotation			
				C. microphyllum		ICC4958														
				Transcript	SSR repeat-motifs	Chromosomes	SSR repeat-motifs	Start physical positions (bp)	End physical positions (bp)						Gene IDs	Sequence component of genes	NCBI nr IDs	TFs	KOG	KEGG
TR66602|c0_g1_i1	XLOC_027680	(AG)9	18	--	--	--	--	--	--	GGGGAAGAGAAGAAGAGGGA	60	AGTCGCTGAAAACCTCCTCA	60	225	--	--	--	--	--	--
TR66605|c0_g1_i1	XLOC_027681	(TA)8	16	--	--	--	--	--	--	CCTTCACAAAAGGAGCCAAA	60	TTAATCCATCCGAGATTCGC	60	136	--	--	--	--	--	--
TR66677|c0_g2_i1	XLOC_027697	(AAT)5	15	--	--	--	--	--	--	GACACTTCGTAGAACCACCGA	60	CGATGAGTGAAGGGTAGGGA	60	228	--	--	XM_004500618.1	--	[E]	K01657
TR66682|c0_g1_i1	XLOC_027700	(GTT)5	15	--	--	--	--	--	--	GCTGATCCAAAATCCAAACC	59	AGGATAATGGTTGCACCAGG	60	236	--	--	--	--	--	--
TR66704|c0_g1_i1	XLOC_027708	(AAC)5	15	--	--	--	--	--	--	ACCGACCACCACTCTGATTC	60	TTTTGTGTGCGTTTTGGTGT	60	187	--	--	--	--	--	--
TR66704|c0_g1_i1	XLOC_027708	(CT)7	14	--	--	--	--	--	--	ACTTTGAGAAGGCAAAGGCA	60	GAAGAGAAAGGGGTGAAGGG	60	181	--	--	--	--	--	--
TR66849|c0_g1_i1	No	(TA)6	12	--	--	--	--	--	--	CGCTGGCAAGATTCTTCTTC	60	CATCACACAAACTTCATACCCC	59	248	--	--	--	--	--	--
TR66890|c0_g1_i1	No	(TGA)6	18	--	--	--	--	--	--	CAGCTCTGGTGATAGCTCCC	60	GAAGACTTGGCACTGCTTCC	60	228	--	--	--	--	--	--
TR66890|c0_g1_i3	No	(TGA)6	18	--	--	--	--	--	--	CAGCTCTGGTGATAGCTCCC	60	GAAGACTTGGCACTGCTTCC	60	228	--	--	--	--	--	--
TR66890|c0_g1_i4	No	(TGA)6	18	--	--	--	--	--	--	CAGCTCTGGTGATAGCTCCC	60	GAAGACTTGGCACTGCTTCC	60	228	--	--	--	--	--	--
TR66893|c0_g1_i11	No	(CA)6	12	--	--	--	--	--	--	TACCACTTAGGCCATCAGCC	60	AATGGGAAGATTGGCAACAG	60	112	--	--	--	--	--	--
TR66901|c1_g1_i1	XLOC_027792	(TCA)5	15	--	--	--	--	--	--	GTCCGGCCCATAAGATAACA	60	ATGCATGACAAGGATCACCA	60	257	--	--	--	--	--	--
TR66902|c0_g1_i1	XLOC_027793	(GAGT)5	20	--	--	--	--	--	--	CAAATCCCGTCATACACAACA	59	GAAGGGTTAGGGAAAATGGG	60	148	--	--	--	--	--	--
TR66902|c0_g1_i1	XLOC_027793	(CTC)6	18	--	--	--	--	--	--	CCCATTTTCCCTAACCCTTC	60	TGACCGAAAATTTGGAGGAG	60	192	--	--	--	--	--	--

Transcript IDs	Differentially expressed gene (DEG) IDs	SSR repeat-motifs	SSR repeat-length (bp)	*In silico* polymorphic SSRs						Forward primers (5'-3')	Annealing tempearture(0C)	Reverse primers (5'-3')	Annealing tempearture(0C)	Amplified product size (bp)	Structural annotation		Functional Annotation			
				C. microphyllum		ICC4958									Gene IDs	Sequence component of genes	NCBI nr IDs	TFs	KOG	KEGG
				Transcript	SSR repeat-motifs	Chromosomes	SSR repeat-motifs	Start physical positions (bp)	End physical positions (bp)											
TR66902|c0_g1_i1	XLOC_027793	(GGT)6	18	--	--	--	--	--	--	CGGCTCTAGTTCCCATTTCA	60	ATGTGGCCTCTGTTGAAACC	60	212	--	--	--	--	--	--
TR66902|c0_g1_i2	XLOC_027794	(GAGT)5	20	--	--	--	--	--	--	CAAATCCCGTCATACACAACA	59	GAAGGGTTAGGGAAAATGGG	60	148	--	--	--	--	--	--
TR66902|c0_g1_i2	XLOC_027794	(CTC)6	18	--	--	--	--	--	--	CCCATTTTCCCTAACCCTTC	60	TGACCGAAAATTTGGAGGAG	60	192	--	--	--	--	--	--
TR66902|c0_g1_i2	XLOC_027794	(GGT)6	18	--	--	--	--	--	--	CGGCTCTAGTTCCCATTTCA	60	ATGTGGCCTCTGTTGAAACC	60	212	--	--	--	--	--	--
TR66906|c1_g1_i1	XLOC_027797	(ATA)6	18	--	--	--	--	--	--	CCTCACAAACCCAACAAACA	59	TCCGGTGGGTATAGTCAGTTG	60	274	--	--	XP_004506906.1	--	[X]	--
TR66906|c1_g1_i1	XLOC_027797	(TTC)5	15	--	--	--	--	--	--	TTTTCCGGTACGGTAGTTGC	60	TCAGGTCTTGTTTGGGATGA	59	186	--	--	XP_004506906.1	--	[X]	--
TR66909|c1_g1_i1	XLOC_027813	(TTA)5attttttttctctatcattcg(A)11	47	--	--	--	--	--	--	TGACACCCTGGATTCCTTCT	60	TCTCAAGCCTCCTACGTTCAA	60	280	--	--	--	--	--	--
TR66912|c0_g1_i1	No	(CT)10	20	TR66912|c0_g1_i1	(CT)10	Ca_LG_3	(CT)11	29341398	29341419	TGCAGTATTTGTTTGCACCC	60	AACGAAATCAGAGGCTTGGA	60	232		URR	--	--	--	--
TR66912|c0_g2_i1	No	(CT)10	20	--	--	--	--	--	--	TGCAGTATTTGTTTGCACCC	60	AACGAAATCAGAGGCTTGGA	60	233	--	--	--	--	--	--
TR66918|c0_g2_i1	XLOC_027826	(TTC)6	18	--	--	--	--	--	--	TGGATCTGCTCCGATCTCTT	60	TCTTCGCTCGAAAAAGAAGC	60	262	--	--	--	--	--	--
TR66918|c2_g1_i1	XLOC_027828	(GCG)5	15	--	--	--	--	--	--	TTCTCTCCTCATTTCTCCCTACC	60	TTGAAACCCGATTGGAGAAG	60	142	--	--	--	--	--	--
TR66919|c0_g1_i1	No	(GTG)5	15	--	--	--	--	--	--	TGGTGAAAAGAGAAAAGGGG	59	TCCCATTACCATTACCATTACCA	60	182	--	--	--	--	--	--
TR66919|c0_g3_i1	No	(GTG)5	15	--	--	--	--	--	--	TGGTGAAAAGAGAAAAGGGG	59	TCCCATTACCATTACCATTACCA	60	182	--	--	--	--	--	--
TR66919|c8_g1_i1	XLOC_027837	(GAC)5	15	--	--	--	--	--	--	TTCACTTCCACTCCGATTCC	60	AGTCGGTTGGTCGTTGGTAG	60	217	--	--	--	--	--	--

Transcript IDs	Differentially expressed gene (DEG) IDs	SSR repeat-motifs	SSR repeat-length (bp)	*In silico* polymorphic SSRs						Forward primers (5'-3')	Annealing tempearture(0C)	Reverse primers (5'-3')	Annealing tempearture(0C)	Amplified product size (bp)	Structural annotation		Functional Annotation			
				C. microphyllum		ICC4958									Gene IDs	Sequence component of genes	NCBI nr IDs	TFs	KOG	KEGG
				Transcript	SSR repeat-motifs	Chromosomes	SSR repeat-motifs	Start physical positions (bp)	End physical positions (bp)											
TR66919|c9_g1_i1	XLOC_027838	(TTGT)6	24	--	--	--	--	--	--	GGTAGGAGGAGAAGAAAGAAAAGG	60	GTCCATGCTGGCAAAAGATT	60	215	--	--	--	--	--	--
TR66920|c0_g1_i1	No	(CT)9	18	--	--	--	--	--	--	TCCATTTTTCTCATTCCCCA	60	GGCATGTTAGCCCAACAACT	60	277	--	--	--	--	--	--
TR66920|c0_g1_i2	No	(CT)9	18	--	--	--	--	--	--	TCCATTTTTCTCATTCCCCA	60	GGCATGTTAGCCCAACAACT	60	277	--	--	--	--	--	--
TR66920|c0_g2_i1	No	(CT)9	18	--	--	--	--	--	--	TAATGATTCCGCAGCTTCCT	60	GAGGTTGAGGTTGAGGACCA	60	267	--	--	--	--	--	--
TR66920|c0_g2_i2	No	(CT)9	18	--	--	--	--	--	--	TAATGATTCCGCAGCTTCCT	60	GAGGTTGAGGTTGAGGACCA	60	267	--	--	--	--	--	--
TR66923|c0_g1_i1	XLOC_027848	(GA)9	18	--	--	--	--	--	--	GGGGTATGTGTGTGGGTTTT	59	CAACGCAGCGCAGAATAATA	60	237	--	--	--	--	--	--
TR66923|c0_g2_i1	No	(GA)9	18	--	--	--	--	--	--	GGGGTATGTGTGTGGGTTTT	59	CAACGCAGCGCAGAATAATA	60	237	--	--	--	--	--	--
TR66925|c0_g1_i1	No	(GA)10	20	--	--	--	--	--	--	ATCAGGAGGGGAAAGAGGAA	60	CCAGAGCGGTAAAACAAACC	60	204	--	--	--	--	--	--
TR66943|c0_g1_i6	XLOC_027867	(AT)6	12	--	--	--	--	--	--	ACCATAAGTGCCTTAGAAGAGGT	58	ACACGACCTTGAGCAGCTTT	60	116	--	--	--	--	--	--
TR66945|c0_g1_i2	No	(TC)6	12	--	--	--	--	--	--	GTAAACGATCTTTCTCCCGC	59	CGGTAGACAATCCAGCCATT	60	231	--	--	--	--	--	--
TR66948|c0_g1_i2	No	(TAC)5	15	--	--	--	--	--	--	GGCATGTCTCCTGGTTCATT	60	GGAGGGAGGATTGGAAGAAG	60	263	--	--	--	--	--	--
TR66948|c1_g1_i3	No	(AG)6	12	--	--	--	--	--	--	ATGGGCCCAATGTAAAATCA	60	ATACGAGAGAGGCGGACAAA	60	191	--	--	--	--	--	--
TR66952|c0_g3_i2	No	(TC)6	12	--	--	--	--	--	--	CGCACCACTCACACAACTTT	60	ATGCCCTCCAACAAATTCAA	60	278	--	--	--	--	--	--
TR66952|c0_g3_i3	No	(TC)6	12	--	--	--	--	--	--	CGCACCACTCACACAACTTT	60	ATGCCCTCCAACAAATTCAA	60	278	--	--	--	--	--	--

Transcript IDs	Differentially expressed gene (DEG) IDs	SSR repeat-motifs	SSR repeat-length (bp)	*In silico* polymorphic SSRs						Forward primers (5'-3')	Annealing tempearture(0C)	Reverse primers (5'-3')	Annealing tempearture(0C)	Amplified product size (bp)	Structural annotation		Functional Annotation			
				C. microphyllum		ICC4958									Gene IDs	Sequence component of genes	NCBI nr IDs	TFs	KOG	KEGG
				Transcript	SSR repeat-motifs	Chromosomes	SSR repeat-motifs	Start physical positions (bp)	End physical positions (bp)											
TR66952|c0_g3_i4	No	(TC)6	12	--	--	--	--	--	--	CGCACCACTCACACAACTTT	60	ATGCCCTCCAACAAATTCAA	60	278	--	--	--	--	--	--
TR66952|c0_g3_i5	No	(TC)6	12	--	--	--	--	--	--	CGCACCACTCACACAACTTT	60	ATGCCCTCCAACAAATTCAA	60	278	--	--	--	--	--	--
TR66952|c0_g3_i6	No	(TC)6	12	--	--	--	--	--	--	CGCACCACTCACACAACTTT	60	ATGCCCTCCAACAAATTCAA	60	278	--	--	--	--	--	--
TR66963|c2_g1_i1	XLOC_027890	(GAT)6	18	--	--	--	--	--	--	TGCAAGATGTAGTACGGAGCA	60	CTCATGCCTTACCCAACACA	60	162	--	--	--	--	--	--
TR66963|c2_g1_i2	XLOC_027891	(GAT)6	18	--	--	--	--	--	--	TGCAAGATGTAGTACGGAGCA	60	CTCATGCCTTACCCAACACA	60	162	--	--	--	--	--	--
TR66965|c0_g1_i1	XLOC_027894	(TA)6	12	--	--	--	--	--	--	CAGCGTGGAAGAAAGTGAGA	59	TGGTCGGAGAACGAAAAGAG	60	180	--	--	--	--	--	--
TR66972|c0_g1_i1	No	(TCT)10	30	--	--	--	--	--	--	CCATGGAGCTCTGTACTACTCG	59	TGGAAGACGGTCCTTTTCAG	60	269	--	--	--	--	--	--
TR66972|c0_g1_i1	No	(CTT)5	15	--	--	--	--	--	--	TTCTTCCTCTTCGTTCCAACA	60	GGATTCCTCTGTTTCGGTGA	60	212	--	--	--	--	--	--
TR66972|c0_g1_i2	No	(TCT)10	30	--	--	--	--	--	--	CCATGGAGCTCTGTACTACTCG	59	TGGAAGACGGTCCTTTTCAG	60	269	--	--	--	--	--	--
TR66972|c0_g1_i2	No	(CTT)5	15	--	--	--	--	--	--	TTCTTCCTCTTCGTTCCAACA	60	GGATTCCTCTGTTTCGGTGA	60	212	--	--	--	--	--	--
TR66972|c1_g1_i1	XLOC_027905	(AGA)7	21	--	--	--	--	--	--	TCAGCAATCAAAATCTAGCAGC	60	CTTGCCAAGCAAATTTCACA	60	126	--	--	--	--	--	--
TR66980|c1_g1_i1	No	(CTA)5	15	--	--	--	--	--	--	CCTTTTAGCGGCGTTAGTGA	60	GAAAGACAAAATTGGCGGAA	60	171	--	--	--	--	--	--
TR66980|c1_g1_i1	No	(CTT)6	18	--	--	--	--	--	--	TGCTTGGTTCCTTGAAGTTG	59	CGATAATCACTAACGCCGCT	60	186	--	--	--	--	--	--
TR66980|c1_g1_i2	No	(CTA)5	15	--	--	--	--	--	--	CCTTTTAGCGGCGTTAGTGA	60	GAAAGACAAAATTGGCGGAA	60	171	--	--	--	--	--	--

Transcript IDs	Differentially expressed gene (DEG) IDs	SSR repeat-motifs	SSR repeat-length (bp)	*In silico* polymorphic SSRs						Forward primers (5'-3')	Annealing tempearture(0C)	Reverse primers (5'-3')	Annealing tempearture(0C)	Amplified product size (bp)	Structural annotation		Functional Annotation			
				C. microphyllum		ICC4958														
				Transcript	SSR repeat-motifs	Chromosomes	SSR repeat-motifs	Start physical positions (bp)	End physical positions (bp)						Gene IDs	Sequence component of genes	NCBI nr IDs	TFs	KOG	KEGG
TR66980|c1_g1_i2	No	(CTT)6	18	--	--	--	--	--	--	TGCTTGGTTCCTTGAAGTTG	59	CGATAATCACTAACGCCGCT	60	186	--	--	--	--	--	--
TR66980|c1_g1_i4	XLOC_027931	(CTA)5	15	--	--	--	--	--	--	CCTTTTAGCGGCGTTAGTGA	60	GAAAGACAAAATTGGCGGAA	60	171	--	--	--	--	--	--
TR66980|c1_g1_i4	XLOC_027931	(CTT)6	18	--	--	--	--	--	--	TGCTTGGTTCCTTGAAGTTG	59	CGATAATCACTAACGCCGCT	60	186	--	--	--	--	--	--
TR66986|c1_g2_i1	XLOC_027944	(CTT)6	18	--	--	--	--	--	--	GAGGAACACTCATTCCCCAA	60	CCAAATGGTGCCTTCTTTGT	60	184	--	--	--	--	--	--
TR66986|c11_g1_i1	No	(CTT)7	21	--	--	--	--	--	--	TCCCACTCACCGATCTTTTC	60	CGTTTTTCGTGGCTGATTTT	60	120	--	--	--	--	--	--
TR66986|c11_g1_i1	No	(GTG)5	15	--	--	--	--	--	--	TCCCACTCACCGATCTTTTC	60	TTCATAGGGAGACTCACCGC	60	262	--	--	--	--	--	--
TR66986|c11_g1_i2	No	(CTT)7	21	--	--	--	--	--	--	TCCCACTCACCGATCTTTTC	60	CGTTTTTCGTGGCTGATTTT	60	120	--	--	--	--	--	--
TR66986|c11_g1_i2	No	(GTG)5	15	--	--	--	--	--	--	TCCCACTCACCGATCTTTTC	60	TTCATAGGGAGACTCACCGC	60	262	--	--	--	--	--	--
TR66986|c11_g1_i3	No	(CTT)7	21	--	--	--	--	--	--	TCCCACTCACCGATCTTTTC	60	CGTTTTTCGTGGCTGATTTT	60	120	--	--	--	--	--	--
TR66986|c11_g1_i3	No	(GTG)5	15	--	--	--	--	--	--	TCCCACTCACCGATCTTTTC	60	TTCATAGGGAGACTCACCGC	60	262	--	--	--	--	--	--
TR66986|c11_g1_i4	No	(CTT)7	21	--	--	--	--	--	--	TCCCACTCACCGATCTTTTC	60	CGTTTTTCGTGGCTGATTTT	60	120	--	--	--	--	--	--
TR66986|c11_g1_i4	No	(GTG)5	15	--	--	--	--	--	--	TCCCACTCACCGATCTTTTC	60	TTCATAGGGAGACTCACCGC	60	262	--	--	--	--	--	--
TR66986|c12_g2_i1	XLOC_027938	(CAA)6	18	TR66986|c12_g2_i1	(CTT)7	Ca_LG_7	(CTT)9	36756808	36756834	AATCCCCGCTAACTTCACCT	60	GCTCCTGTTTAGCCTGCAAC	60	251	Ca_18369	DRR	--	--	--	--
TR66986|c13_g2_i1	No	(A)10(AC)6*	21	--	--	--	--	--	--	CGCGGGAAGGAACTAACATA	60	GGGGGCATTATGGGAATACT	60	225	--	--	--	--	--	--

Transcript IDs	Differentially expressed gene (DEG) IDs	SSR repeat-motifs	SSR repeat-length (bp)	*In silico* polymorphic SSRs						Forward primers (5'-3')	Annealing tempearture(0C)	Reverse primers (5'-3')	Annealing tempearture(0C)	Amplified product size (bp)	Structural annotation		Functional Annotation			
				C. microphyllum		ICC4958									Gene IDs	Sequence component of genes	NCBI nr IDs	TFs	KOG	KEGG
				Transcript	SSR repeat-motifs	Chromosomes	SSR repeat-motifs	Start physical positions (bp)	End physical positions (bp)											
TR66986|c2_g1_i1	XLOC_027945	(CAA)6	18	TR66986|c2_g1_i1	(CAA)6	Ca_LG_6	(CAA)5	42994077	42994091	TTTCTCGGGTTTTGAATTGG	60	CAGTCTTCGCTCCACAAACA	60	248	Ca_15340	DRR	--	--	--	--
TR66986|c8_g1_i1	No	(CTT)7	21	--	--	--	--	--	--	CAAAGAAACAAAGAAGAAAAAGGG	60	TGTTGAGAACCCATTTCCAA	59	208	--	--	--	--	--	--
TR66986|c8_g1_i1	No	(AAC)6	18	--	--	--	--	--	--	TTGGAAATGGGTTCTCAACA	59	GGCCACGAAAAACATCAAGT	60	163	--	--	--	--	--	--
TR66986|c8_g1_i2	XLOC_027949	(CTT)7	21	--	--	--	--	--	--	CAAAGAAACAAAGAAGAAAAAGGG	60	TGTTGAGAACCCATTTCCAA	59	209	--	--	--	--	--	--
TR66986|c8_g1_i2	XLOC_027949	(AAC)6	18	--	--	--	--	--	--	TTGGAAATGGGTTCTCAACA	59	GGCCACGAAAAACATCAAGT	60	163	--	--	--	--	--	--
TR66986|c9_g2_i1	No	(CT)8	16	--	--	--	--	--	--	CACATTTCATTCATTCCCTTCA	60	CGTTTCGACCCAGTTATCGT	60	141	--	--	--	--	--	--
TR66986|c9_g2_i1	No	(TCT)5tattcttctc(CTT)6	43	--	--	--	--	--	--	GTCGACGACGATAACTGGGT	60	CGAACGGTCCAGTGAAGATT	60	161	--	--	--	--	--	--
TR66986|c9_g2_i2	XLOC_027950	(CT)8	16	--	--	--	--	--	--	CACATTTCATTCATTCCCTTCA	60	CGTTTCGACCCAGTTATCGT	60	141	--	--	--	--	--	--
TR66986|c9_g2_i2	XLOC_027950	(TCT)5tattcttctc(CTT)6	43	--	--	--	--	--	--	GTCGACGACGATAACTGGGT	60	CGAACGGTCCAGTGAAGATT	60	161	--	--	--	--	--	--
TR66990|c2_g1_i1	No	(TTTA)5	20	--	--	--	--	--	--	CCTGCATCGCCTTATATCCT	59	GACGGTGTCCATATCCCATC	60	161	--	--	--	--	--	--
TR66993|c1_g1_i1	XLOC_027958	(T)10ctcgagaaaaaccgaaggaaactgaatagagggt tttattgtgtgttgttgaaaaaatttga(T)10	82	--	--	--	--	--	--	GTCCCACTTTGCTTGTGGAT	60	TGTTTCCATGCCAAGATGAA	60	224	--	--	XM_004493084.1	--	[O]	--
TR67002|c2_g3_i1	XLOC_027982	(GTT)5	15	--	--	--	--	--	--	GTCGTCGTTGTCGTCTTCAA	60	TCTTCTCCACCACCAATTCC	60	124	--	--	--	--	--	--
TR67002|c2_g3_i2	XLOC_027983	(GTT)5	15	--	--	--	--	--	--	GTCGTCGTTGTCGTCTTCAA	60	TCTTCTCCACCACCAATTCC	60	124	--	--	--	--	--	--
TR67012|c0_g1_i1	No	(AG)6	12	--	--	--	--	--	--	CTGTGTCAATGTCGGACACC	60	CCCTTCACTTCTCGTAACGC	60	231	--	--	--	--	--	--

Transcript IDs	Differentially expressed gene (DEG) IDs	SSR repeat-motifs	SSR repeat-length (bp)	*In silico* polymorphic SSRs						Forward primers (5'-3')	Annealing tempearture(0C)	Reverse primers (5'-3')	Annealing tempearture(0C)	Amplified product size (bp)	Structural annotation		Functional Annotation			
				C. microphyllum		ICC4958									Gene IDs	Sequence component of genes	NCBI nr IDs	TFs	KOG	KEGG
				Transcript	SSR repeat-motifs	Chromosomes	SSR repeat-motifs	Start physical positions (bp)	End physical positions (bp)											
TR67012|c0_g1_i2	No	(AG)6	12	--	--	--	--	--	--	CTGTGTCAATGTCGGACACC	60	CCCTTCACTTCTCGTAACGC	60	231	--	--	--	--	--	--
TR67012|c0_g2_i1	XLOC_028002	(AG)6	12	--	--	--	--	--	--	GAAATGGATCGGGAATTGAA	60	CTCGTAACGCGTTGTGTTGT	60	108	--	--	--	--	--	--
TR67012|c2_g1_i1	XLOC_028004	(CCA)10	30	--	--	--	--	--	--	ATCCACCACTTCCACCTCAG	60	TGGTAAGGGTGTTTTGGAGG	60	172	--	--	--	--	--	--
TR67013|c2_g1_i1	No	(TCC)6	18	--	--	--	--	--	--	TCTTGAAGTTCCTCTTCCTCCA	60	CCCGTGGTTAATTCTGCTGT	60	143	--	--	--	--	--	--
TR67013|c2_g2_i1	No	(TCC)6	18	--	--	--	--	--	--	TCTTGAAGTTCCTCTTCCTCCA	60	CCCGTGGTTAATTCTGCTGT	60	143	--	--	--	--	--	--
TR67013|c2_g2_i2	No	(TCC)6	18	--	--	--	--	--	--	TCTTGAAGTTCCTCTTCCTCCA	60	CCCGTGGTTAATTCTGCTGT	60	143	--	--	--	--	--	--
TR67013|c2_g2_i3	No	(TCC)6	18	--	--	--	--	--	--	TCTTGAAGTTCCTCTTCCTCCA	60	CCCGTGGTTAATTCTGCTGT	60	143	--	--	--	--	--	--
TR67013|c3_g1_i6	XLOC_028012	(GAG)7gaagtagaagaagttgaa(GAG)5	54	--	--	--	--	--	--	TTCCCCCAGGAGCTAGTTTT	60	GCATCATGGGCCATTTACTT	60	248	--	--	--	--	--	--
TR67016|c0_g2_i1	XLOC_028023	(TG)6	12	--	--	--	--	--	--	CCCTGATGATGTCTTCCCTG	60	AGGCCAAGATTTATGGGGAA	61	218	--	--	--	--	--	--
TR67030|c1_g1_i1	No	(TC)6	12	--	--	--	--	--	--	CGCACTTTCCCATTCTAGGT	59	GGGAGCTCCTTTTTGTGTGA	60	275	--	--	--	--	--	--
TR67030|c1_g1_i2	No	(TC)6	12	--	--	--	--	--	--	AGAAGCACGCAGACAGGATT	60	GCTTTTTCTCCATTCTCCCA	59	249	--	--	--	--	--	--
TR67030|c1_g1_i2	No	(AAGA)5	20	--	--	--	--	--	--	TGCTTGCACTATCAGCCATC	60	TTGTCCGCACCACTATCTGA	60	102	--	--	--	--	--	--
TR67030|c1_g1_i3	No	(TC)6	12	--	--	--	--	--	--	AGAAGCACGCAGACAGGATT	60	GCTTTTTCTCCATTCTCCCA	59	249	--	--	--	--	--	--
TR67030|c1_g1_i3	No	(AAGA)5	20	--	--	--	--	--	--	TGCTTGCACTATCAGCCATC	60	TTGTCCGCACCACTATCTGA	60	102	--	--	--	--	--	--

Transcript IDs	Differentially expressed gene (DEG) IDs	SSR repeat-motifs	SSR repeat-length (bp)	*In silico* polymorphic SSRs						Forward primers (5'-3')	Annealing tempearture(0C)	Reverse primers (5'-3')	Annealing tempearture(0C)	Amplified product size (bp)	Structural annotation		Functional Annotation			
				C. microphyllum		ICC4958									Gene IDs	Sequence component of genes	NCBI nr IDs	TFs	KOG	KEGG
				Transcript	SSR repeat-motifs	Chromosomes	SSR repeat-motifs	Start physical positions (bp)	End physical positions (bp)											
TR67030|c1_g1_i4	No	(TC)6	12	--	--	--	--	--	--	CGCACTTTCCCATTCTAGGT	59	GGGAGCTCCTTTTTGTGTGA	60	259	--	--	--	--	--	--
TR67030|c1_g1_i4	No	(AAGA)5	20	--	--	--	--	--	--	TGCTTGCACTATCAGCCATC	60	TTGTCCGCACCACTATCTGA	60	102	--	--	--	--	--	--
TR67037|c0_g1_i1	No	(TTC)7tcttaaacctaattccct(TTC)6	57	--	--	--	--	--	--	CACCCTTAACCCCACTCTCA	60	GGTGGCAAAGGTGTCTTCAT	60	273	--	--	--	--	--	--
TR67037|c0_g2_i1	No	(TTC)7tcttaaacctaattccct(TTC)6	57	--	--	--	--	--	--	CACCCTTAACCCCACTCTCA	60	GGTGGCAAAGGTGTCTTCAT	60	273	--	--	--	--	--	--
TR67037|c1_g1_i1	XLOC_028066	(CT)7	14	--	--	--	--	--	--	GAAAAGCCAAACCAAACCAA	60	GCTGTTGCTCCAACTCTTCC	60	270	--	--	--	--	--	--
TR67040|c2_g2_i1	No	(CAA)5	15	TR67040|c2_g2_i1	(CAA)5	Ca_LG_6	(CAA)6	12500876	12500893	CCATCACCACCAACCCTAAC	60	AGAAGAGAGGGCGGAGTAGG	60	212	Ca_14101	URR	--	--	--	--
TR67040|c2_g2_i1	No	(TAG)6	18	TR67040|c2_g2_i1	(CAA)5	Ca_LG_6	(CAA)6	12500876	12500893	GGAAGGATGAGACGTTAAGGC	60	TCCGATGGATACAACTGCAA	60	161	Ca_14101	URR	--	--	--	--
TR67040|c2_g2_i2	No	(CAA)5	15	--	--	--	--	--	--	CCATCACCACCAACCCTAAC	60	AGAAGAGAGGGCGGAGTAGG	60	212	--	--	--	--	--	--
TR67040|c2_g2_i2	No	(TAG)6	18	--	--	--	--	--	--	GGAAGGATGAGACGTTAAGGC	60	TCCGATGGATACAACTGCAA	60	161	--	--	--	--	--	--
TR67040|c2_g2_i3	No	(CAA)5	15	--	--	--	--	--	--	CCATCACCACCAACCCTAAC	60	AGAAGAGAGGGCGGAGTAGG	60	212	--	--	--	--	--	--
TR67040|c2_g2_i3	No	(TAG)6	18	--	--	--	--	--	--	GGAAGGATGAGACGTTAAGGC	60	TCCGATGGATACAACTGCAA	60	161	--	--	--	--	--	--
TR67043|c2_g1_i1	No	(AG)7	14	--	--	--	--	--	--	TGATGGATGATTCCCAAGACT	59	TCCATCTGTTCCCCACTTTC	60	277	--	--	--	--	--	--
TR67043|c2_g3_i2	No	(TC)9	18	--	--	--	--	--	--	TGGAACTGACCACCTTCCAT	60	CTGAAAACTGAGGCAACCCT	59	247	--	--	--	--	--	--
TR67043|c3_g1_i1	No	(AG)8	16	--	--	--	--	--	--	TGCATATGTGAGTGTGCCTG	59	CAGGAACGGTGTTTGTGTTG	60	254	--	--	--	--	--	--

Transcript IDs	Differentially expressed gene (DEG) IDs	SSR repeat-motifs	SSR repeat-length (bp)	*In silico* polymorphic SSRs						Forward primers (5'-3')	Annealing tempearture(0C)	Reverse primers (5'-3')	Annealing tempearture(0C)	Amplified product size (bp)	Structural annotation		Functional Annotation			
				C. microphyllum		ICC4958									Gene IDs	Sequence component of genes	NCBI nr IDs	TFs	KOG	KEGG
				Transcript	SSR repeat-motifs	Chromosomes	SSR repeat-motifs	Start physical positions (bp)	End physical positions (bp)											
TR67043|c3_g2_i1	XLOC_028093	(AAG)6	18	--	--	--	--	--	--	TTCACACACGTTAAACCTTGAAA	60	TCCTTGTTCCTTCTTCCTTCC	60	233	--	--	--	--	--	--
TR67049|c0_g1_i2	XLOC_028105	(GA)10aaatacaaaatttgtgtctaagagagtttcagtgg ctctgacttcatctatgatatgtattgtaagaagagttagaagt (GATA)5	119	--	--	--	--	--	--	AAGTTAAAAATGCAGAAGGAACA	57	ATGGCACGTCCTTTTCAAAC	60	198	--	--	--	--	--	--
TR67049|c2_g1_i1	XLOC_028111	(TCA)5	15	--	--	--	--	--	--	TTGCAAATCAGTCCTCCAAA	59	TTGGAAGTGATGATGAATGAGG	60	267	--	--	--	--	--	--
TR67053|c0_g1_i1	XLOC_028122	(ATG)5	15	--	--	--	--	--	--	CGGAAAAGACGCGAAAATTA	60	CTCGTTCTCTCCGGTTCAAT	59	180	--	--	--	--	--	--
TR67065|c0_g1_i1	No	(TC)8	16	--	--	--	--	--	--	CCACACAGCCAAACCCTAAT	60	TTCGATCTTTGGGGATGAAG	60	267	--	--	--	--	--	--
TR67065|c0_g3_i1	No	(TC)8	16	--	--	--	--	--	--	CCACACAGCCAAACCCTAAT	60	TTCGATCTTTGGGGATGAAG	60	267	--	--	--	--	--	--
TR67065|c0_g3_i2	No	(TC)8	16	--	--	--	--	--	--	CCACACAGCCAAACCCTAAT	60	TTCGATCTTTGGGGATGAAG	60	267	--	--	--	--	--	--
TR67065|c0_g3_i3	No	(TC)8	16	--	--	--	--	--	--	CCACACAGCCAAACCCTAAT	60	TTCGATCTTTGGGGATGAAG	60	267	--	--	--	--	--	--
TR67071|c0_g1_i1	No	(CAA)5	15	--	--	--	--	--	--	AAGGGAAGGGGAAAAAGTGA	60	TCAAACCGTGATGGTAATGG	59	249	--	--	--	--	--	--
TR67071|c0_g1_i2	No	(CAA)5	15	--	--	--	--	--	--	CCTGTAGCACCTAACCTCCG	60	TCAAACCGTGATGGTAATGG	59	246	--	--	--	--	--	--
TR67077|c0_g1_i1	XLOC_028166	(TG)9	18	--	--	--	--	--	--	TCGTCCGTGAGAGGAGAAAC	60	TTATTCCAACAAGCCGAAGG	60	194	--	--	--	--	--	--
TR67077|c0_g2_i1	XLOC_028167	(TG)9	18	--	--	--	--	--	--	TCGTCCGTGAGAGGAGAAAC	60	TTATTCCAACAAGCCGAAGG	60	192	--	--	--	--	--	--
TR67077|c1_g1_i10	No	(TG)9	18	--	--	--	--	--	--	CTTGCAGAGAAATGGGAAGC	60	CGAAGGAAAAGGCATTGAAA	60	263	--	--	--	--	--	--
TR67077|c1_g1_i9	No	(TG)9	18	--	--	--	--	--	--	CTTGCAGAGAAATGGGAAGC	60	CGAAGGAAAAGGCATTGAAA	60	263	--	--	--	--	--	--

Transcript IDs	Differentially expressed gene (DEG) IDs	SSR repeat-motifs	SSR repeat-length (bp)	*In silico* polymorphic SSRs						Forward primers (5'-3')	Annealing tempearture(0C)	Reverse primers (5'-3')	Annealing tempearture(0C)	Amplified product size (bp)	Structural annotation		Functional Annotation			
				C. microphyllum		ICC4958									Gene IDs	Sequence component of genes	NCBI nr IDs	TFs	KOG	KEGG
				Transcript	SSR repeat-motifs	Chromosomes	SSR repeat-motifs	Start physical positions (bp)	End physical positions (bp)											
TR67082|c2_g4_i1	No	(ATG)6	18	--	--	--	--	--	--	TTCGATACTCAGCGCTTAATTG	60	TGGCCATTCTGTTTTTCACA	60	222	--	--	--	--	--	--
TR67208|c0_g1_i1	XLOC_028197	(ATG)5	15	--	--	--	--	--	--	AACATAAAAGTTGTAGTAACTGAAGCA	57	GCGCATCTTATGTAGCGTCTC	60	213	--	--	--	--	--	--
TR67240|c0_g1_i1	No	(TCA)5	15	--	--	--	--	--	--	TCTGATAAACCCTCTTCTCCTCA	59	CCGGTCCATCAACTAAATCC	59	133	--	--	--	--	--	--
TR67325|c0_g1_i1	No	(ACA)5	15	--	--	--	--	--	--	ATTCCAAATGGGTAGTGGCA	60	TCAGCACCAAAACTGTGACTTT	60	228	--	--	--	--	--	--
TR67325|c0_g1_i2	No	(ACA)5	15	--	--	--	--	--	--	ATTCCAAATGGGTAGTGGCA	60	TCAGCACCAAAACTGTGACTTT	60	228	--	--	--	--	--	--
TR67325|c0_g1_i3	No	(ACA)5	15	--	--	--	--	--	--	ATTCCAAATGGGTAGTGGCA	60	TCTGCAGCCATCACTTTTCA	61	252	--	--	--	--	--	--
TR67325|c0_g1_i5	No	(ACA)5	15	--	--	--	--	--	--	ATTCCAAATGGGTAGTGGCA	60	TCTGCAGCCATCACTTTTCA	61	252	--	--	--	--	--	--
TR67352|c1_g1_i1	XLOC_028251	(GA)6	12	--	--	--	--	--	--	TCTACAGGGGGAAAGACAGC	59	ATTTTACCCCTCTCAAGGGC	59	173	--	--	--	--	--	--
TR67384|c0_g1_i1	XLOC_028281	(GA)8	16	--	--	--	--	--	--	TGCAGGGTAAAATAGTGCCA	59	TATCTGGGATGCGATGACAA	60	264	--	--	--	--	--	--
TR67490|c1_g1_i1	XLOC_028326	(TG)6	12	--	--	--	--	--	--	TTGTGGTTTTTGTGAAACTTGG	60	AATTGGTAGCCCAAAAACCC	60	279	--	--	--	--	--	--
TR67499|c0_g1_i1	XLOC_028332	(CAA)7	21	--	--	--	--	--	--	TTTCTCTCCCTCCCATTCCT	60	CGGGTCGGATTTTTAGGTCT	60	115	--	--	--	--	--	--
TR67506|c0_g1_i1	XLOC_028336	(TC)7	14	--	--	--	--	--	--	AGAGTTGACCGGAGCTTCAA	60	ATAGTGTCCGGCGAATTGAC	60	254	--	--	--	--	--	--
TR67511|c0_g1_i1	XLOC_028339	(AT)9	18	--	--	--	--	--	--	GGTTTCATGGGGTTTCTTTG	59	CTACTGGCTTTTGGGCTTTG	60	224	--	--	KEH37993.1	C3H	[R]	--
TR67512|c0_g1_i1	No	(A)11tttggctttggtttgctcaatattaaattttggaa(T)11	57	--	--	--	--	--	--	GCCATGCATGTATTTTTGAGG	60	AAGAACTCACATCTATTCTTTCCAA	58	133	--	--	--	--	--	--

Transcript IDs	Differentially expressed gene (DEG) IDs	SSR repeat-motifs	SSR repeat-length (bp)	*In silico* polymorphic SSRs						Forward primers (5'-3')	Annealing tempearture(0C)	Reverse primers (5'-3')	Annealing tempearture(0C)	Amplified product size (bp)	Structural annotation		Functional Annotation			
				C. microphyllum		ICC4958									Gene IDs	Sequence component of genes	NCBI nr IDs	TFs	KOG	KEGG
				Transcript	SSR repeat-motifs	Chromosomes	SSR repeat-motifs	Start physical positions (bp)	End physical positions (bp)											
TR67512|c0_g2_i1	XLOC_028340	(TAG)5	15	--	--	--	--	--	--	TCCACCTCAATCATTATGCAA	59	TAAAACAAACAAGCCCCACC	60	245	--	--	--	--	--	--
TR67560|c0_g1_i1	No	(GA)7	14	--	--	--	--	--	--	AATTGTGCACCCCCATAGAG	60	GGTGTGCAACTTGTGTGGAG	60	197	--	--	--	--	--	--
TR67726|c0_g1_i1	XLOC_028403	(TC)9tgaagcgagcaagacaacagtcaatcacaaccc cacaaactcacactcattttggtccactccattccaacacac cctttcaaattttaaa(TC)9	127	--	--	--	--	--	--	CAGTACTAACTACGGTCCCCAC	58	CGCGGGTTAGAGAGACAGAA	61	204	--	--	--	--	--	--
TR67775|c0_g1_i1	No	(CAG)5	15	--	--	--	--	--	--	GATATCTCCGGAACGAAACG	60	CCGTCGTGTGTGGATAGATG	60	141	--	--	--	--	--	--
TR67798|c0_g1_i1	XLOC_028419	(AT)6	12	--	--	--	--	--	--	CCATTACTCAAGGCCTGCAC	61	GAGCTAAGGGAAGGGGAGAA	60	270	--	--	--	--	--	--
TR67802|c0_g1_i1	XLOC_028424	(ATA)8ttaatgatcttaaagattctgatcctcaacaaaatg gtcacgcaccttctcta(TCT)6	95	--	--	--	--	--	--	CAAGAGCTTCCACATTCAAGG	60	CATAAGCATCAAAGCTCGCA	60	264	--	--	--	--	--	--
TR67802|c1_g1_i1	XLOC_028425	(ATA)6	18	--	--	--	--	--	--	TGTTGTAAGGTGGCAGGTGA	60	GTCGGCAAATGGAAAATGAT	60	148	--	--	XP_004514140.1	--	[X]	K10956
TR67819|c0_g1_i1	No	(CT)9	18	--	--	--	--	--	--	AACCCCCGTCTACGAAAAGT	60	ACAAGAACCATTCTCGCTCG	60	199	--	--	--	--	--	--
TR67819|c0_g1_i2	No	(CT)9	18	--	--	--	--	--	--	AACCCCCGTCTACGAAAAGT	60	ACAAGAACCATTCTCGCTCG	60	199	--	--	--	--	--	--
TR67820|c0_g1_i1	XLOC_028447	(TC)8	16	--	--	--	--	--	--	CAACATTACAACAAACAAAACCA	58	ATCTCCACAACCACTGGTCC	60	261	--	--	--	--	--	--
TR67820|c0_g2_i1	No	(TC)8	16	--	--	--	--	--	--	ACCACCATCTTCTCTCCCCT	60	TGCCCTAAATTGTGATGTTCA	59	272	--	--	--	--	--	--
TR67820|c0_g2_i2	No	(TC)8	16	--	--	--	--	--	--	ACCACCATCTTCTCTCCCCT	60	TGCCCTAAATTGTGATGTTCA	59	272	--	--	--	--	--	--
TR67820|c0_g2_i3	No	(TC)8	16	--	--	--	--	--	--	ACCACCATCTTCTCTCCCCT	60	TGCCCTAAATTGTGATGTTCA	59	272	--	--	--	--	--	--
TR67820|c2_g1_i1	No	(CT)9	18	--	--	--	--	--	--	GTATCCGTAAACGGCGAAGA	60	GGGAGAGGAAGAAGATGAAGAA	59	220	--	--	--	--	--	--

Transcript IDs	Differentially expressed gene (DEG) IDs	SSR repeat-motifs	SSR repeat-length (bp)	*In silico* polymorphic SSRs						Forward primers (5'-3')	Annealing tempearture(0C)	Reverse primers (5'-3')	Annealing tempearture(0C)	Amplified product size (bp)	Structural annotation		Functional Annotation			
				C. microphyllum		ICC4958									Gene IDs	Sequence component of genes	NCBI nr IDs	TFs	KOG	KEGG
				Transcript	SSR repeat-motifs	Chromosomes	SSR repeat-motifs	Start physical positions (bp)	End physical positions (bp)											
TR67834|c0_g1_i1	No	(AGAT)5	20	--	--	--	--	--	--	GATGAAGTGAAATCACCCGC	60	TGCAGAAAGTGGATGAGTCG	60	174	--	--	--	--	--	--
TR67834|c0_g1_i2	No	(AGAT)5	20	--	--	--	--	--	--	GATGAAGTGAAATCACCCGC	60	TGCAGAAAGTGGATGAGTCG	60	174	--	--	--	--	--	--
TR67834|c0_g1_i4	No	(AGAT)5	20	--	--	--	--	--	--	GATGAAGTGAAATCACCCGC	60	TGCAGAAAGTGGATGAGTCG	60	174	--	--	--	--	--	--
TR67835|c0_g1_i1	No	(CCT)5	15	--	--	--	--	--	--	CAATTGCTCGAGCTTGTGTC	60	CTTCGGTCGAAGATCTGGAG	60	202	--	--	--	--	--	--
TR67835|c0_g2_i1	XLOC_028466	(CCT)5	15	--	--	--	--	--	--	CAATTGCTCGAGCTTGTGTC	60	CTTCGGTCGAAGATCTGGAG	60	202	--	--	XP_004487728.1	HD-ZIP	[R]	--
TR67841|c0_g1_i1	No	(CT)9	18	--	--	--	--	--	--	CTCTGTCCCAAACCCCACT	60	GGTGAATAGAGTGTGCAGCG	59	111	--	--	--	--	--	--
TR67841|c0_g1_i2	No	(CT)9	18	--	--	--	--	--	--	CTCTGTCCCAAACCCCACT	60	GGTGAATAGAGTGTGCAGCG	59	111	--	--	--	--	--	--
TR67849|c0_g1_i2	XLOC_028475	(TAC)5	15	--	--	--	--	--	--	GCTTTTGGAGTTCCGTGAAG	60	ATGTGGTTGCCTAGTTGGCT	60	232	--	--	--	--	--	--
TR67851|c0_g1_i1	No	(TC)7	14	--	--	--	--	--	--	AAATGAGGAGAGTGGAGCGA	60	CCTAAAAATCCAAAAGGCCA	59	227	--	--	--	--	--	--
TR67851|c0_g1_i2	No	(TC)7	14	--	--	--	--	--	--	AAATGAGGAGAGTGGAGCGA	60	CCTAAAAATCCAAAAGGCCA	59	227	--	--	--	--	--	--
TR67861|c2_g1_i1	XLOC_028485	(AG)8	16	--	--	--	--	--	--	TTGAGGCATGCTTGTTATGC	60	CCGATCTCTTGGCTTTTCTTT	60	170	--	--	--	--	--	--
TR67868|c0_g1_i1	No	(GAA)6(GAT)6	36	--	--	--	--	--	--	ACGACAACCACCCAGAACAT	60	AGCCCTGCTGCAAGATACAT	60	244	--	--	--	--	--	--
TR67868|c0_g1_i2	No	(GAA)6(GAT)6	36	--	--	--	--	--	--	ACGACAACCACCCAGAACAT	60	AGCCCTGCTGCAAGATACAT	60	244	--	--	--	--	--	--
TR67869|c0_g2_i1	XLOC_028493	(TTA)5	15	--	--	--	--	--	--	GCAGCAAGAAACGAAACCTC	60	TTAGGCAACAGCACTGGATG	60	229	--	--	--	--	--	--

Transcript IDs	Differentially expressed gene (DEG) IDs	SSR repeat-motifs	SSR repeat-length (bp)	*In silico* polymorphic SSRs						Forward primers (5'-3')	Annealing tempearture(0C)	Reverse primers (5'-3')	Annealing tempearture(0C)	Amplified product size (bp)	Structural annotation		Functional Annotation			
				C. microphyllum		ICC4958														
				Transcript	SSR repeat-motifs	Chromosomes	SSR repeat-motifs	Start physical positions (bp)	End physical positions (bp)						Gene IDs	Sequence component of genes	NCBI nr IDs	TFs	KOG	KEGG
TR67871|c1_g2_i1	No	(GA)8	16	--	--	--	--	--	--	CCGAACACGAATTTGGAGAT	60	TCTGCCGTCCTCTCAAGTTT	60	171	--	--	--	--	--	--
TR67871|c1_g2_i2	No	(GA)8	16	--	--	--	--	--	--	CCGAACACGAATTTGGAGAT	60	TCTGCCGTCCTCTCAAGTTT	60	171	--	--	--	--	--	--
TR67872|c0_g1_i1	No	(AGA)5	15	--	--	--	--	--	--	AGAGAAGGCAAAGGGGAAAG	60	TCCCTGTCCTTCTTTTCATCA	60	141	--	--	--	--	--	--
TR67872|c0_g1_i2	No	(AGA)5	15	--	--	--	--	--	--	AGAGAAGGCAAAGGGGAAAG	60	TCCCTGTCCTTCTTTTCATCA	60	141	--	--	--	--	--	--
TR67874|c0_g4_i1	XLOC_028499	(TC)6	12	--	--	--	--	--	--	AAACAAACAAACAGAGCCGC	60	CGAAGCAACGCAAATTATGA	60	133	--	--	--	--	--	--
TR67879|c0_g1_i1	No	(TC)11	22	--	--	--	--	--	--	GGAGGAAGCTCTCTTGACCA	60	ACAAAACCAACACTCACTGAAA	57	271	--	--	--	--	--	--
TR67880|c0_g1_i1	XLOC_028503	(TAT)6	18	--	--	--	--	--	--	TGATGTGTTTAGGTGGCTGG	60	CAAACCCACCGCTCATATCT	60	153	--	--	--	--	--	--
TR67882|c1_g1_i1	No	(ATG)5	15	--	--	--	--	--	--	TGGGAGCCTATAAGGCATTG	60	TGCTCATAATGTTTTCCAGCC	60	221	--	--	--	--	--	--
TR67898|c0_g1_i1	XLOC_028516	(CT)6	12	--	--	--	--	--	--	CACATGGTGCACACTCACAC	60	GAATCGTCTTCACCCCAAGA	60	216	--	--	--	--	--	--
TR67898|c0_g1_i1	XLOC_028516	(AAC)5	15	--	--	--	--	--	--	GTAAGCATCTCACCCCCAAA	60	TCATTGTCTTCAGAAAGTGCTGT	59	152	--	--	--	--	--	--
TR67900|c0_g1_i1	XLOC_028517	(GAA)7	21	--	--	--	--	--	--	AGAAGACTTGCGAAATGCGT	60	TGAAGATTCAACCTTTCCCTT	58	280	--	--	--	--	--	--
TR68027|c0_g2_i1	No	(TC)6	12	--	--	--	--	--	--	CGTCTGATTCTGATATCATCTCTC	57	AACGGAATCGAAAACCTCCT	60	181	--	--	--	--	--	--
TR68027|c0_g2_i2	No	(TC)6	12	--	--	--	--	--	--	CGTCTGATTCTGATATCATCTCTC	57	CAGCCTTGGAAGGAAGAGTG	60	128	--	--	--	--	--	--
TR68027|c0_g2_i4	No	(TC)6	12	--	--	--	--	--	--	CGTCTGATTCTGATATCATCTCTC	57	CAGCCTTGGAAGGAAGAGTG	60	128	--	--	--	--	--	--

Transcript IDs	Differentially expressed gene (DEG) IDs	SSR repeat-motifs	SSR repeat-length (bp)	*In silico* polymorphic SSRs						Forward primers (5'-3')	Annealing tempearture(0C)	Reverse primers (5'-3')	Annealing tempearture(0C)	Amplified product size (bp)	Structural annotation		Functional Annotation			
				C. microphyllum		ICC4958									Gene IDs	Sequence component of genes	NCBI nr IDs	TFs	KOG	KEGG
				Transcript	SSR repeat-motifs	Chromosomes	SSR repeat-motifs	Start physical positions (bp)	End physical positions (bp)											
TR68032|c1_g1_i1	XLOC_028556	(AAC)6	18	--	--	--	--	--	--	TGTTGGGTCCTCTTTTGTTTG	60	TCACCATCGTGTGATGGACT	60	250	--	--	--	--	--	--
TR68041|c0_g1_i1	No	(TA)7	14	--	--	--	--	--	--	CCGGATGTTTTACAATGGGT	60	CTATGATGGTGGGAGTGGGT	60	256	--	--	--	--	--	--
TR68041|c0_g2_i1	XLOC_028563	(TA)7	14	--	--	--	--	--	--	CCGGATGTTTTACAATGGGT	60	CTATGATGGTGGGAGTGGGT	60	256	--	--	XP_004503590.1	--	[S]	--
TR68042|c0_g2_i1	XLOC_028565	(AT)7	14	TR68042|c0_g2_i1	(AT)7	Ca_LG_6	(AT)6	45747524	45747535	TCACAAAGCTTGCAAAATGAA	59	ATCGGAGTGGATTAGGGGTC	60	103	Ca_12220	CDS	--	--	--	--
TR68054|c0_g1_i1	No	(TC)9	18	--	--	--	--	--	--	GGTACGCTGACGCCATCTAT	60	TGAAAGAAGCATGGAAACCC	60	255	--	--	--	--	--	--
TR68054|c0_g2_i1	No	(TC)9	18	--	--	--	--	--	--	GGTACGCTGACGCCATCTAT	60	TGAAAGAAGCATGGAAACCC	60	255	--	--	--	--	--	--
TR68055|c1_g1_i1	XLOC_028574	(AG)8	16	--	--	--	--	--	--	TCACTTGTGATCCCGAAACA	60	ACAAATGGCCATATCCTTCG	60	244	--	--	--	--	--	--
TR68071|c0_g1_i1	XLOC_028587	(TCATTC)5	30	--	--	--	--	--	--	ATCGCAGGGTTAACACCAAG	60	GATCCTCATTGCGTCCTCTC	60	229	--	--	--	--	--	--
TR68072|c0_g1_i1	No	(ATTC)5	20	--	--	--	--	--	--	TGACCCACCATTCATTCTCA	60	CATAGTGCTGCTCCTCCACA	60	123	--	--	--	--	--	--
TR68072|c0_g2_i1	No	(ATTC)5	20	--	--	--	--	--	--	TGACCCACCATTCATTCTCA	60	CATAGTGCTGCTCCTCCACA	60	123	--	--	--	--	--	--
TR68072|c0_g2_i2	No	(ATTC)5	20	--	--	--	--	--	--	TGACCCACCATTCATTCTCA	60	CATAGTGCTGCTCCTCCACA	60	123	--	--	--	--	--	--
TR68093|c0_g1_i1	No	(TAC)5	15	--	--	--	--	--	--	TGGAGTTCAAGACTTCCGAGA	60	TCATGATTTGTTTTCCAAGTTGA	59	227	--	--	--	--	--	--
TR68093|c0_g2_i1	XLOC_028597	(TAC)5	15	--	--	--	--	--	--	TGGAGTTCAAGACTTCCGAGA	60	TCATGATTTGTTTTCCAAGTTGA	59	227	--	--	--	--	--	--
TR68108|c0_g1_i1	No	(A)11taaagaagatgctctagatgacaatctttcaacatag gtaaccaaataaatgtttg(A)11	78	--	--	--	--	--	--	TAAGAACAGCGCAATGCAAC	60	TGACTGGCATCGAATGACTC	60	210	--	--	--	--	--	--

<table>
<tr><th rowspan="3">Transcript IDs</th><th rowspan="3">Differentially expressed gene (DEG) IDs</th><th rowspan="3">SSR repeat-motifs</th><th rowspan="3">SSR repeat-length (bp)</th><th colspan="6">In silico polymorphic SSRs</th><th rowspan="3">Forward primers (5'-3')</th><th rowspan="3">Annealing tempearture(0C)</th><th rowspan="3">Reverse primers (5'-3')</th><th rowspan="3">Annealing tempearture(0C)</th><th rowspan="3">Amplified product size (bp)</th><th colspan="2">Structural annotation</th><th colspan="4">Functional Annotation</th></tr>
<tr><th colspan="2">C. microphyllum</th><th colspan="4">ICC4958</th><th rowspan="2">Gene IDs</th><th rowspan="2">Sequence component of genes</th><th rowspan="2">NCBI nr IDs</th><th rowspan="2">TFs</th><th rowspan="2">KOG</th><th rowspan="2">KEGG</th></tr>
<tr><th>Transcript</th><th>SSR repeat-motifs</th><th>Chromosomes</th><th>SSR repeat-motifs</th><th>Start physical positions (bp)</th><th>End physical positions (bp)</th></tr>
<tr><td>TR68172|c0_g1_i1</td><td>XLOC_028621</td><td>(A)12caaaaatacccgaaaaccttaagaaatgttttacggaacaataa(T)13</td><td>69</td><td>--</td><td>--</td><td>--</td><td>--</td><td>--</td><td>--</td><td>TAGCTCCGGCCTACATTTTT</td><td>59</td><td>TCCTACGAGATTTTAATTGTGTATCAA</td><td>59</td><td>207</td><td>--</td><td>--</td><td>--</td><td>--</td><td>--</td><td>--</td></tr>
<tr><td>TR68231|c0_g1_i1</td><td>No</td><td>(CAA)5</td><td>15</td><td>--</td><td>--</td><td>--</td><td>--</td><td>--</td><td>--</td><td>ATTTGATGAAAACCCCACCA</td><td>60</td><td>TGTTGTGACCCCACCAACTA</td><td>60</td><td>181</td><td>--</td><td>--</td><td>--</td><td>--</td><td>--</td><td>--</td></tr>
<tr><td>TR68231|c0_g2_i1</td><td>No</td><td>(CAA)5</td><td>15</td><td>--</td><td>--</td><td>--</td><td>--</td><td>--</td><td>--</td><td>ATTTGATGAAAACCCCACCA</td><td>60</td><td>TGTTGTGACCCCACCAACTA</td><td>60</td><td>181</td><td>--</td><td>--</td><td>--</td><td>--</td><td>--</td><td>--</td></tr>
<tr><td>TR68237|c0_g1_i1</td><td>No</td><td>(TC)6</td><td>12</td><td>--</td><td>--</td><td>--</td><td>--</td><td>--</td><td>--</td><td>GACAACGTCGTCTCGTGTCT</td><td>59</td><td>CCAAGAAAGTTGTTGGAACTGA</td><td>59</td><td>180</td><td>--</td><td>--</td><td>--</td><td>--</td><td>--</td><td>--</td></tr>
<tr><td>TR68237|c0_g2_i1</td><td>No</td><td>(TC)6</td><td>12</td><td>--</td><td>--</td><td>--</td><td>--</td><td>--</td><td>--</td><td>GACAACGTCGTCTCGTGTCT</td><td>59</td><td>CCAAGAAAGTTGTTGGAACTGA</td><td>59</td><td>180</td><td>--</td><td>--</td><td>--</td><td>--</td><td>--</td><td>--</td></tr>
<tr><td>TR68241|c0_g1_i1</td><td>XLOC_028651</td><td>(CT)7</td><td>14</td><td>--</td><td>--</td><td>--</td><td>--</td><td>--</td><td>--</td><td>CGGAAAACTTTTGAACCTGG</td><td>60</td><td>AACAACATTAGAGCAAAGAGAAAGA</td><td>58</td><td>278</td><td>--</td><td>--</td><td>XP_010015314.1</td><td>--</td><td>[O]</td><td>K10590</td></tr>
<tr><td>TR68284|c0_g1_i1</td><td>XLOC_028681</td><td>(AG)10</td><td>20</td><td>--</td><td>--</td><td>--</td><td>--</td><td>--</td><td>--</td><td>TTCGAGAAGTGAAGCGAGTG</td><td>59</td><td>TTGTTGGAAGGAAGAATCGG</td><td>60</td><td>263</td><td>--</td><td>--</td><td>--</td><td>--</td><td>--</td><td>--</td></tr>
<tr><td>TR68309|c0_g1_i1</td><td>No</td><td>(GA)7</td><td>14</td><td>--</td><td>--</td><td>--</td><td>--</td><td>--</td><td>--</td><td>CCGTAACATCCGCATTTTCT</td><td>60</td><td>TCGATGACTCAGATTCCGGT</td><td>61</td><td>230</td><td>--</td><td>--</td><td>--</td><td>--</td><td>--</td><td>--</td></tr>
<tr><td>TR68390|c0_g1_i1</td><td>No</td><td>(TCA)5</td><td>15</td><td>--</td><td>--</td><td>--</td><td>--</td><td>--</td><td>--</td><td>GCAACATTGATGCTTGTTTG</td><td>58</td><td>TTAAACGTGCGTGGATTTGA</td><td>60</td><td>133</td><td>--</td><td>--</td><td>--</td><td>--</td><td>--</td><td>--</td></tr>
<tr><td>TR68390|c0_g2_i1</td><td>No</td><td>(TCA)5</td><td>15</td><td>--</td><td>--</td><td>--</td><td>--</td><td>--</td><td>--</td><td>GCAACATTGATGCTTGTTTG</td><td>58</td><td>TTAAACGTGCGTGGATTTGA</td><td>60</td><td>133</td><td>--</td><td>--</td><td>--</td><td>--</td><td>--</td><td>--</td></tr>
<tr><td>TR68627|c0_g1_i1</td><td>No</td><td>(TA)6</td><td>12</td><td>--</td><td>--</td><td>--</td><td>--</td><td>--</td><td>--</td><td>AGCTTGGAAATTTGGCTGAA</td><td>60</td><td>GGCTGGCATTGGTTACACTT</td><td>60</td><td>194</td><td>--</td><td>--</td><td>--</td><td>--</td><td>--</td><td>--</td></tr>
<tr><td>TR68655|c0_g1_i1</td><td>No</td><td>(AT)6</td><td>12</td><td>--</td><td>--</td><td>--</td><td>--</td><td>--</td><td>--</td><td>TGCTTTTCTGTATTTCCCGTG</td><td>60</td><td>TGGAATTAGTTGATGTGACAATGA</td><td>59</td><td>221</td><td>--</td><td>--</td><td>--</td><td>--</td><td>--</td><td>--</td></tr>
<tr><td>TR68688|c0_g1_i1</td><td>XLOC_028813</td><td>(CA)6</td><td>12</td><td>TR68688|c0_g1_i1</td><td>(CA)6</td><td>Ca_LG_5</td><td>(CA)8</td><td>14883925</td><td>14883940</td><td>AACCAGAATTCGTTTTCACG</td><td>58</td><td>TGGCTAATCAAAGACCATGC</td><td>59</td><td>244</td><td>Ca_04501</td><td>CDS</td><td>XP_004514830.1</td><td>--</td><td>[T]</td><td>--</td></tr>
<tr><td>TR68740|c0_g1_i1</td><td>XLOC_028830</td><td>(ACT)5</td><td>15</td><td>--</td><td>--</td><td>--</td><td>--</td><td>--</td><td>--</td><td>ACAGCCGGTTTTCAGTCAAG</td><td>60</td><td>CGATCTCAGCAATTTGTCCA</td><td>60</td><td>227</td><td>--</td><td>--</td><td>--</td><td>--</td><td>--</td><td>--</td></tr>
</table>

Transcript IDs	Differentially expressed gene (DEG) IDs	SSR repeat-motifs	SSR repeat-length (bp)	*In silico* polymorphic SSRs						Forward primers (5'-3')	Annealing tempearture(0C)	Reverse primers (5'-3')	Annealing tempearture(0C)	Amplified product size (bp)	Structural annotation		Functional Annotation			
				C. microphyllum		ICC4958														
				Transcript	SSR repeat-motifs	Chromosomes	SSR repeat-motifs	Start physical positions (bp)	End physical positions (bp)						Gene IDs	Sequence component of genes	NCBI nr IDs	TFs	KOG	KEGG
TR6877|c0_g1_i1	No	(TTC)6	18	--	--	--	--	--	--	CGTCGCATAGTGGTGTGGTA	61	TTTTCGAAGAGGATCATGGG	60	217	--	--	--	--	--	--
TR68782|c0_g1_i1	XLOC_028844	(TTG)5tgttaagcgtagcgaggaagaggaagagtt(TG)8	61	--	--	--	--	--	--	CCGGAAATTGGAAAGGAAAG	61	CATCATCGACCTGAAAACCC	60	250	--	--	--	--	--	--
TR68785|c0_g1_i1	No	(AG)6	12	--	--	--	--	--	--	AACCCCAAAACCACCTCAAT	60	TTTCGCCTTTTCCTCTTTCA	60	179	--	--	--	--	--	--
TR68801|c0_g1_i1	No	(CTG)5	15	--	--	--	--	--	--	CGTCTTCACACCAAAACCCT	60	TTGAAGGGGGAAGGAATAGG	60	151	--	--	--	--	--	--
TR68849|c1_g5_i1	No	(TGG)6	18	--	--	--	--	--	--	TGGTGGGTGTGGAATTTCTT	60	CCTCCACCCGTCCATTATTA	60	180	--	--	--	--	--	--
TR68849|c1_g5_i2	XLOC_028871	(TGG)6	18	--	--	--	--	--	--	GATGGTGGAGGTGGTGACTT	60	CCTCCACCCGTCCATTATTA	60	257	--	--	--	--	--	--
TR68849|c1_g5_i3	No	(TGG)6	18	--	--	--	--	--	--	TGGTGGGTGTGGAATTTCTT	60	CCTCCACCCGTCCATTATTA	60	180	--	--	--	--	--	--
TR68893|c0_g1_i1	XLOC_028905	(TC)8	16	--	--	--	--	--	--	GAGAGGAGCCAACTGGAACA	60	TGGTGCTATGAGAAATTGCC	59	211	--	--	--	--	--	--
TR68893|c0_g1_i2	No	(TC)8	16	--	--	--	--	--	--	GAGAGGAGCCAACTGGAACA	60	TGGTGCTATGAGAAATTGCC	59	211	--	--	--	--	--	--
TR68906|c0_g1_i1	XLOC_028915	(AT)6	12	--	--	--	--	--	--	TGCCTATTTTCCCCTTTTCTT	59	AAACTTGAGCATCACCTCCG	60	278	--	--	--	--	--	--
TR68909|c0_g1_i1	XLOC_028919	(TC)11	22	--	--	--	--	--	--	GGGACGGACAAAGAAACAAA	60	CACCTCCTCCATTAACCGAA	60	236	--	--	--	--	--	--
TR68917|c1_g2_i1	No	(TCA)5	15	--	--	--	--	--	--	TCATCACATGGCAGCTGAAC	61	TCTAAACCGGGAACATCTGG	60	276	--	--	--	--	--	--
TR68917|c1_g2_i2	No	(TCA)5	15	--	--	--	--	--	--	TCATCACATGGCAGCTGAAC	61	TCTAAACCGGGAACATCTGG	60	276	--	--	--	--	--	--
TR68917|c1_g2_i3	No	(TCA)5	15	--	--	--	--	--	--	TCATCACATGGCAGCTGAAC	61	TCTAAACCGGGAACATCTGG	60	276	--	--	--	--	--	--

Transcript IDs	Differentially expressed gene (DEG) IDs	SSR repeat-motifs	SSR repeat-length (bp)	*In silico* polymorphic SSRs						Forward primers (5'-3')	Annealing tempearture(0C)	Reverse primers (5'-3')	Annealing tempearture(0C)	Amplified product size (bp)	Structural annotation		Functional Annotation			
				C. microphyllum		ICC4958									Gene IDs	Sequence component of genes	NCBI nr IDs	TFs	KOG	KEGG
				Transcript	SSR repeat-motifs	Chromosomes	SSR repeat-motifs	Start physical positions (bp)	End physical positions (bp)											
TR68917|c1_g2_i4	No	(TCA)5	15	--	--	--	--	--	--	TCATCACATGGCAGCTGAAC	61	TCTAAACCGGGAACATCTGG	60	276	--	--	--	--	--	--
TR68917|c1_g2_i5	No	(TCA)5	15	--	--	--	--	--	--	TCATCACATGGCAGCTGAAC	61	TCTAAACCGGGAACATCTGG	60	276	--	--	--	--	--	--
TR68921|c1_g2_i1	No	(GTT)5	15	--	--	--	--	--	--	TTGTAGTGAAATGGGGCACA	60	TCAACTCAACCAAACAACAACA	59	130	--	--	--	--	--	--
TR68927|c1_g1_i1	XLOC_028938	(CTC)7	21	--	--	--	--	--	--	TGGTTTTGTTTTTCGCCTTC	60	GGGGAGAAGAGACAACAGAGG	60	143	--	--	XM_004516336.1	--	[S]	--
TR6893|c1_g1_i1	XLOC_028949	(AG)8	16	TR6893|c1_g1_i1	(AG)8	Ca_LG_6	(AG)6	43489508	43489519	GGAACCAAAAGATCCATACCAT	59	GGGGGTCTAAAGCAAGTGGT	60	268	Ca_11768	URR	--	--	--	--
TR6894|c0_g1_i1	XLOC_028951	(ATTT)6	24	--	--	--	--	--	--	CACCAAGCACCAAAATAGCA	60	TGGGATTAAGAGAAAATAGGTGC	59	182	--	--	--	--	--	--
TR6894|c0_g1_i1	XLOC_028951	(TTC)7	21	--	--	--	--	--	--	GTTTCTTCATCTTCTTCAGCTTTT	58	TCGAAGAAGTCAAAGTGGGG	60	223	--	--	--	--	--	--
TR68962|c0_g1_i1	No	(CAT)5	15	--	--	--	--	--	--	CATTGGCACAGAGGCACA	60	AATTTTCACCTGGCGTTCAG	60	238	--	--	--	--	--	--
TR68986|c0_g1_i1	No	(TC)7	14	--	--	--	--	--	--	ATGAGCAACCCACAAACATT	58	GGTGGTTTTCACAGCAGCTT	60	122	--	--	--	--	--	--
TR69003|c0_g1_i1	XLOC_028961	(TA)6	12	--	--	--	--	--	--	TTTTGTTTAATTGCTCCCGC	60	GAACGCAGTCCGGGATAAC	60	162	--	--	--	--	--	--
TR6905|c0_g1_i1	XLOC_028973	(TCT)6	18	TR6905|c0_g1_i1	(TCT)6	Ca_LG_4	(TCT)10	21910544	21910573	TCCAACCATTTTCCCTTTTC	59	TCCACACGTTTCTGCTTCTG	60	216	Ca_01106	CDS	--	--	--	--
TR69147|c0_g1_i1	XLOC_029005	(TA)7	14	--	--	--	--	--	--	AAGGAAATCGAAGGTTTCTGC	60	TTCTTTTTCCGGTTTGGGTT	61	100	--	--	--	--	--	--
TR69147|c0_g2_i1	No	(TA)7	14	--	--	--	--	--	--	AAGGAAATCGAAGGTTTCTGC	60	TTCTTTTTCCGGTTTGGGTT	61	100	--	--	--	--	--	--
TR69223|c0_g1_i1	No	(GAA)5	15	--	--	--	--	--	--	GTACATGGGGGACCTTGTGT	60	CATCCTCTGCTCCTTTTTGC	60	107	--	--	--	--	--	--

Transcript IDs	Differentially expressed gene (DEG) IDs	SSR repeat-motifs	SSR repeat-length (bp)	*In silico* polymorphic SSRs						Forward primers (5'-3')	Annealing tempearture(0C)	Reverse primers (5'-3')	Annealing tempearture(0C)	Amplified product size (bp)	Structural annotation		Functional Annotation			
				C. microphyllum		ICC4958														
				Transcript	SSR repeat-motifs	Chromosomes	SSR repeat-motifs	Start physical positions (bp)	End physical positions (bp)						Gene IDs	Sequence component of genes	NCBI nr IDs	TFs	KOG	KEGG
TR69247|c0_g1_i1	XLOC_029035	(TGC)5	15	--	--	--	--	--	--	ACCACCGAAGAACTCTCCCT	60	ATCGTCCTTCTTGTTGTCGG	60	279	--	--	XP_002899876.1	GRAS	[U]	--
TR6926|c0_g1_i1	No	(AGC)5	15	--	--	--	--	--	--	CCGTCTTGTAGCTTCCCTTG	60	AGTTTCAAGGTTGTTGCTGC	58	166	--	--	--	--	--	--
TR6926|c0_g2_i1	No	(AGC)6	18	--	--	--	--	--	--	CCGTCTTGTAGCTTCCCTTG	60	GGATCCTCGAAGTTATTGCG	60	205	--	--	--	--	--	--
TR6938|c0_g1_i1	No	(ACA)5	15	--	--	--	--	--	--	CCCAATTTTCGTTCTCCAAA	60	ATTCCTCGGGTTCTTCGTCT	60	251	--	--	--	--	--	--
TR6938|c0_g1_i2	No	(ACA)5	15	--	--	--	--	--	--	CCCAATTTTCGTTCTCCAAA	60	ATTCCTCGGGTTCTTCGTCT	60	251	--	--	--	--	--	--
TR69382|c0_g1_i1	No	(TTA)5	15	--	--	--	--	--	--	TAACAAAAAGCGCTCGTCAC	59	ACACGAAATGTGTTCACCGA	60	218	--	--	--	--	--	--
TR69397|c0_g1_i1	XLOC_029094	(CT)6	12	TR69397|c0_g1_i1	(CT)6	Ca_LG_4	(CT)8	109882	109897	AAAAGCTTCTCCTCTTTCATTTCA	60	AAGCATCACAGCCATTGAAG	59	203	Ca_02099	URR	XM_004498211.1	ERF	[MVG]	--
TR69399|c0_g1_i1	XLOC_029096	(TTG)6	18	--	--	--	--	--	--	CATGAACAATCCCAACACCA	60	CAACGACGTCATCATTTGGA	61	246	--	--	XP_004498980.1	--	[X]	--
TR69407|c0_g1_i1	XLOC_029103	(CT)7	14	--	--	--	--	--	--	ACCAACTCCGACACGAAAAC	60	AGCTCTTTTCAGCATCCCTG	60	244	--	--	--	--	--	--
TR69411|c0_g1_i1	No	(GCC)5	15	--	--	--	--	--	--	GAGAAAGAGAGAGTCCGCGA	60	AAACAGATCGGTGCGTAACC	60	279	--	--	--	--	--	--
TR69411|c0_g2_i1	No	(GCC)5	15	--	--	--	--	--	--	GAGAAAGAGAGAGTCCGCGA	60	AAACAGATCGGTGCGTAACC	60	279	--	--	--	--	--	--
TR69411|c0_g3_i1	No	(GCC)5	15	--	--	--	--	--	--	GAGAAAGAGAGAGTCCGCGA	60	AAACAGATCGGTGCGTAACC	60	279	--	--	--	--	--	--
TR6943|c0_g1_i1	No	(GAT)8ggagattatgaagaagaa(GAT)5	57	--	--	--	--	--	--	CCACCCACGCTATTTCTCAT	60	CCTCTGCCGTGTACACCTTT	60	182	--	--	--	--	--	--
TR6943|c0_g2_i1	No	(GAT)8ggagattatgaagaagaa(GAT)5	57	--	--	--	--	--	--	CCACCCACGCTATTTCTCAT	60	CCTCTGCCGTGTACACCTTT	60	182	--	--	--	--	--	--

Transcript IDs	Differentially expressed gene (DEG) IDs	SSR repeat-motifs	SSR repeat-length (bp)	*In silico* polymorphic SSRs						Forward primers (5'-3')	Annealing tempearture(0C)	Reverse primers (5'-3')	Annealing tempearture(0C)	Amplified product size (bp)	Structural annotation		Functional Annotation			
				C. microphyllum		ICC4958														
				Transcript	SSR repeat-motifs	Chromosomes	SSR repeat-motifs	Start physical positions (bp)	End physical positions (bp)						Gene IDs	Sequence component of genes	NCBI nr IDs	TFs	KOG	KEGG
TR69464|c0_g1_i1	XLOC_029149	(AT)6	12	--	--	--	--	--	--	TCCGAAAAGTCCAAAATATCAG	58	TCGGTGGATTTGGTAGGAGA	60	243	--	--	KFM75839.1	E2F/DP	[O]	--
TR69476|c0_g1_i1	No	(ATT)5	15	TR69476|c0_g1_i1	(ATT)5	Ca_LG_5	(ATT)9	38694218	38694244	CACTTTGCCATTCCCATTCT	60	AGGATCTTGCTTGCATTTCC	59	257	Ca_08698	DRR	--	--	--	--
TR69476|c0_g1_i2	No	(ATT)5	15	--	--	--	--	--	--	CACTTTGCCATTCCCATTCT	60	AGGATCTTGCTTGCATTTCC	59	257	--	--	--	--	--	--
TR69483|c0_g1_i1	XLOC_029163	(CT)8	16	--	--	--	--	--	--	GCCCACACATTTCTCTCTCC	60	GATTGAATGCGATTGGAAGAA	60	151	--	--	--	--	--	--
TR69513|c0_g1_i1	No	(TG)6	12	--	--	--	--	--	--	GCCAAAATTGATTCTGGAGG	60	GGTTGACGCACTTTCTGGAT	60	248	--	--	--	--	--	--
TR69548|c0_g1_i1	XLOC_029181	(GCA)5	15	--	--	--	--	--	--	TTTGGTCACAACCGTCAAAA	60	AGCCCATCACTACTGGCATC	60	280	--	--	--	--	--	--
TR69558|c0_g1_i1	XLOC_029185	(TTA)5t(A)11catagcacagatcccaacagatttcagaaaatcaaaagaacacagagttttcctgattcctgaacgattttctgacatgaaaacatgatatagaa(AG)7	136	--	--	--	--	--	--	CTCTCTTTCCATATCCCAAAAA	57	TACTGAAAAAGATTCTCACTTTGTTT	57	237	--	--	--	--	--	--
TR69572|c2_g1_i1	No	(AGA)6	18	--	--	--	--	--	--	GCGGAAATCCCAACTTACAA	60	ATCCAAACTCCCTTCTTGCC	60	193	--	--	--	--	--	--
TR69572|c2_g1_i2	No	(AGA)6	18	--	--	--	--	--	--	GGAAATCCCAACGTAAGCAA	60	ATCCAAACTCCCTTCTTGCC	60	210	--	--	--	--	--	--
TR69572|c2_g1_i3	No	(AGA)6	18	--	--	--	--	--	--	GGAAATCCCAACGTAAGCAA	60	ATCCAAACTCCCTTCTTGCC	60	210	--	--	--	--	--	--
TR69580|c0_g1_i1	No	(AC)8	16	--	--	--	--	--	--	TGTGCAAGCTCCAACAATTT	59	CACCCTTCCATAAGCACCAT	60	224	--	--	--	--	--	--
TR69580|c0_g1_i2	No	(AC)8	16	--	--	--	--	--	--	TGTGCAAGCTCCAACAATTT	59	CACCCTTCCATAAGCACCAT	60	224	--	--	--	--	--	--
TR69584|c0_g1_i1	XLOC_029212	(CT)10	20	--	--	--	--	--	--	TCCACTTCACTTCCTCACACA	59	GGTTTGACGGCTGTTGATTT	60	235	--	--	--	--	--	--
TR69588|c0_g2_i1	No	(CCA)5	15	--	--	--	--	--	--	ACCTTCACCTTATCCCCACC	60	TTTTGAATGAATTGGGCGTT	60	250	--	--	--	--	--	--

Transcript IDs	Differentially expressed gene (DEG) IDs	SSR repeat-motifs	SSR repeat-length (bp)	*In silico* polymorphic SSRs						Forward primers (5'-3')	Annealing tempearture(0C)	Reverse primers (5'-3')	Annealing tempearture(0C)	Amplified product size (bp)	Structural annotation		Functional Annotation			
				C. microphyllum		ICC4958														
				Transcript	SSR repeat-motifs	Chromosomes	SSR repeat-motifs	Start physical positions (bp)	End physical positions (bp)						Gene IDs	Sequence component of genes	NCBI nr IDs	TFs	KOG	KEGG
TR69588|c0_g2_i2	No	(CCA)5	15	--	--	--	--	--	--	ACCTTCACCTTATCCCCACC	60	TTTTGAATGAATTGGGCGTT	60	250	--	--	--	--	--	--
TR69589|c0_g2_i1	XLOC_029219	(AGG)5	15	--	--	--	--	--	--	CCGTGATTTTGTCAATCCAA	59	TGGCCCAAATTACAATGGTT	60	275	--	--	--	--	--	--
TR69596|c1_g1_i1	XLOC_029228	(TAA)5	15	--	--	--	--	--	--	GTGGTGGGGACAGAAGAGAA	60	GTTGGTTGCGGATTCAACTT	60	280	--	--	--	--	--	--
TR696|c0_g1_i1	XLOC_029333	(AG)9	18	--	--	--	--	--	--	TCCGTAAAACAACGCTTTCA	59	CAGAATCACCTTGCCACTGA	60	264	--	--	--	--	--	--
TR69604|c1_g2_i1	XLOC_029238	(AG)8	16	--	--	--	--	--	--	AGGATGGCATCTGAAACCTG	60	GCGAGTCATTCGCGTATTATG	61	260	--	--	XP_004494065.1	--	[H]	--
TR69605|c0_g1_i1	No	(GCC)5	15	--	--	--	--	--	--	CCCAAGCCCATTTCATTTTA	60	GGCGGTGTGGTAACTTTGAT	60	189	--	--	--	--	--	--
TR69605|c0_g1_i2	No	(GCC)5	15	--	--	--	--	--	--	ATTTTCCTTCCTTGTTCCGC	60	GGCGGTGTGGTAACTTTGAT	60	129	--	--	--	--	--	--
TR69605|c0_g2_i1	No	(GCC)5	15	--	--	--	--	--	--	CCCAAGCCCATTTCATTTTA	60	TGCACTTTGCTGGTTTTCAG	60	111	--	--	--	--	--	--
TR69605|c0_g2_i2	No	(GCC)5	15	--	--	--	--	--	--	CCCAAGCCCATTTCATTTTA	60	TGCACTTTGCTGGTTTTCAG	60	266	--	--	--	--	--	--
TR69605|c0_g2_i5	No	(GCC)5	15	--	--	--	--	--	--	CCCAAGCCCATTTCATTTTA	60	CTTGTGCACTTTGCCTTGAA	60	126	--	--	--	--	--	--
TR69605|c0_g2_i6	No	(GCC)5	15	--	--	--	--	--	--	CCCAAGCCCATTTCATTTTA	60	TGCACTTTGCTGGTTTTCAG	60	168	--	--	--	--	--	--
TR69621|c1_g1_i1	XLOC_029266	(GAA)6	18	--	--	--	--	--	--	CAGATCCGACCCATGAAACT	60	CAGCCTCAGATCTCTACCGC	60	138	--	--	--	--	--	--
TR69621|c2_g1_i1	XLOC_029267	(AGA)5	15	--	--	--	--	--	--	TTGCGATGCATCAAAATCAT	60	CACATGGAAAAGGAGCCACT	60	273	--	--	--	--	--	--
TR69629|c0_g2_i1	XLOC_029275	(AG)7	14	--	--	--	--	--	--	TCCGTCGAATCAACAAACAC	60	GCCATGGTTTTGGCTTAGAA	60	247	--	--	--	--	--	--

Transcript IDs	Differentially expressed gene (DEG) IDs	SSR repeat-motifs	SSR repeat-length (bp)	*In silico* polymorphic SSRs						Forward primers (5'-3')	Annealing tempearture(0C)	Reverse primers (5'-3')	Annealing tempearture(0C)	Amplified product size (bp)	Structural annotation		Functional Annotation			
				C. microphyllum		ICC4958									Gene IDs	Sequence component of genes	NCBI nr IDs	TFs	KOG	KEGG
				Transcript	SSR repeat-motifs	Chromosomes	SSR repeat-motifs	Start physical positions (bp)	End physical positions (bp)											
TR69629|c0_g3_i1	No	(AG)7	14	--	--	--	--	--	--	TCCGTCGAATCAACAAACAC	60	GCCATGGTTTTGGCTTAGAA	60	247	--	--	--	--	--	--
TR69639|c0_g2_i1	XLOC_029285	(GGC)6	18	--	--	--	--	--	--	TGCTTACTTCTTGGCTGGCT	60	CGCAAGTACCGTGATGCTAA	60	273	--	--	--	--	--	--
TR6964|c0_g1_i1	XLOC_029304	(AGA)6	18	--	--	--	--	--	--	AGAACGTGGTGATGGTAGGC	60	TCATTCTCCTTTTCACTTTCACC	60	132	--	--	XM_004493609.1	--	[X]	--
TR69640|c0_g1_i1	No	(CA)10(A)10*	29	--	--	--	--	--	--	CTCTCAAAATTGGAGTCTTCTTTAC	57	CTTTGGTGGATGTGGTTGTG	60	234	--	--	--	--	--	--
TR69640|c0_g2_i1	No	(CA)10(A)10*	29	--	--	--	--	--	--	CTCTCAAAATTGGAGTCTTCTTTAC	57	GCACCTTTTGAAACAATGTCA	59	169	--	--	--	--	--	--
TR69640|c0_g2_i2	XLOC_029287	(CA)10(A)10*	29	--	--	--	--	--	--	CTCTCAAAATTGGAGTCTTCTTTAC	57	GCACCTTTTGAAACAATGTCA	59	169	--	--	--	--	--	--
TR69646|c0_g1_i1	No	(AT)7	14	--	--	--	--	--	--	AAAAGCAATGCCTCGGTAAA	60	ATGCTGAAGGTTTTTGGCAC	60	199	--	--	--	--	--	--
TR69646|c0_g2_i1	No	(AT)7	14	--	--	--	--	--	--	AAAAGCAATGCCTCGGTAAA	60	ATGCTGAAGGTTTTTGGCAC	60	199	--	--	--	--	--	--
TR69646|c0_g2_i2	No	(AT)7	14	--	--	--	--	--	--	AAAAGCAATGCCTCGGTAAA	60	ATGCTGAAGGTTTTTGGCAC	60	199	--	--	--	--	--	--
TR69651|c1_g1_i2	No	(TAT)5	15	--	--	--	--	--	--	GCAGCATTAGTTCCTTGCTG	59	GGTGGGAGCATTAACTCCAA	60	131	--	--	--	--	--	--
TR69653|c0_g1_i11	No	(CT)6	12	--	--	--	--	--	--	TGAACATTGAAGCAACGAAGA	59	GCATGCTGAACCTTGTGAGA	60	177	--	--	--	--	--	--
TR69653|c0_g1_i13	No	(CT)6	12	--	--	--	--	--	--	TGAACATTGAAGCAACGAAGA	59	CGGTCAATGCCTTACCTTGT	60	181	--	--	--	--	--	--
TR69653|c0_g1_i2	No	(CT)6	12	--	--	--	--	--	--	TGAACATTGAAGCAACGAAGA	59	GCATGCTGAACCTTGTGAGA	60	177	--	--	--	--	--	--
TR69653|c0_g1_i3	No	(CT)6	12	--	--	--	--	--	--	TGAACATTGAAGCAACGAAGA	59	CGGTCAATGCCTTACCTTGT	60	181	--	--	--	--	--	--

Transcript IDs	Differentially expressed gene (DEG) IDs	SSR repeat-motifs	SSR repeat-length (bp)	*In silico* polymorphic SSRs						Forward primers (5'-3')	Annealing tempearture(0C)	Reverse primers (5'-3')	Annealing tempearture(0C)	Amplified product size (bp)	Structural annotation		Functional Annotation			
				C. microphyllum		ICC4958														
				Transcript	SSR repeat-motifs	Chromosomes	SSR repeat-motifs	Start physical positions (bp)	End physical positions (bp)						Gene IDs	Sequence component of genes	NCBI nr IDs	TFs	KOG	KEGG
TR69653|c0_g1_i4	No	(CT)6	12	--	--	--	--	--	--	TGAACATTGAAGCAACGAAGA	59	GCATGCTGAACCTTGTGAGA	60	177	--	--	--	--	--	--
TR69653|c0_g1_i5	No	(CT)6	12	--	--	--	--	--	--	TGAACATTGAAGCAACGAAGA	59	CGGTCAATGCCTTACCTTGT	60	181	--	--	--	--	--	--
TR69653|c0_g1_i6	No	(CT)6	12	--	--	--	--	--	--	TGAACATTGAAGCAACGAAGA	59	CGGTCAATGCCTTACCTTGT	60	181	--	--	--	--	--	--
TR69653|c0_g1_i8	No	(CT)6	12	--	--	--	--	--	--	TGAACATTGAAGCAACGAAGA	59	GCATGCTGAACCTTGTGAGA	60	177	--	--	--	--	--	--
TR69655|c0_g1_i1	No	(GT)6	12	--	--	--	--	--	--	GCAAAAGTAGGGCATGGAGA	60	TTTGGGTCTTCGTTCTTTGG	60	268	--	--	--	--	--	--
TR69655|c0_g2_i1	No	(GT)6	12	--	--	--	--	--	--	GCAAAAGTAGGGCATGGAGA	60	TTTGGGTCTTCGTTCTTTGG	60	268	--	--	--	--	--	--
TR69655|c0_g2_i2	No	(GT)6	12	--	--	--	--	--	--	GCAAAAGTAGGGCATGGAGA	60	TTTGGGTCTTCGTTCTTTGG	60	268	--	--	--	--	--	--
TR69655|c0_g2_i3	No	(GT)6	12	--	--	--	--	--	--	GCAAAAGTAGGGCATGGAGA	60	TTTGGGTCTTCGTTCTTTGG	60	268	--	--	--	--	--	--
TR69655|c0_g2_i4	No	(GT)6	12	--	--	--	--	--	--	GCAAAAGTAGGGCATGGAGA	60	TTTGGGTCTTCGTTCTTTGG	60	268	--	--	--	--	--	--
TR69655|c0_g2_i5	No	(GT)6	12	--	--	--	--	--	--	GCAAAAGTAGGGCATGGAGA	60	TTTGGGTCTTCGTTCTTTGG	60	268	--	--	--	--	--	--
TR69655|c0_g2_i6	No	(GT)6	12	--	--	--	--	--	--	GCAAAAGTAGGGCATGGAGA	60	TTTGGGTCTTCGTTCTTTGG	60	268	--	--	--	--	--	--
TR69657|c1_g1_i1	XLOC_029317	(ATT)5g(TTA)6	34	--	--	--	--	--	--	AGAACCGGAAGGGAAGAGAA	60	GATTTGCTGACTTGCAATGG	59	199	--	--	--	--	--	--
TR69659|c2_g2_i1	No	(TA)7	14	--	--	--	--	--	--	TGGATGTAATGGATTTTAAGTGTACG	60	TGCATTATTTGTCCCCGTTT	60	176	--	--	--	--	--	--
TR69660|c1_g1_i1	No	(A)10ttgttattaaaaagttatatacttcaagatttttctataatgtttacaaatattgatttttgttcttt(A)15	94	--	--	--	--	--	--	TCCGAAAAGGTTAAGATGTCG	59	TGTTCTGGTGAAGGGAGAAAA	60	256	--	--	--	--	--	--

Transcript IDs	Differentially expressed gene (DEG) IDs	SSR repeat-motifs	SSR repeat-length (bp)	*In silico* polymorphic SSRs						Forward primers (5'-3')	Annealing tempearture(0C)	Reverse primers (5'-3')	Annealing tempearture(0C)	Amplified product size (bp)	Structural annotation		Functional Annotation			
				C. microphyllum		ICC4958									Gene IDs	Sequence component of genes	NCBI nr IDs	TFs	KOG	KEGG
				Transcript	SSR repeat-motifs	Chromosomes	SSR repeat-motifs	Start physical positions (bp)	End physical positions (bp)											
TR69660|c1_g1_i3	No	(A)10ttgttattaaaaagttatatacttcaagatttttctataat gtttacaaatattgatttttgttcttt(A)15	94	--	--	--	--	--	--	TCCGAAAAGGTTAAGATGTCG	59	TGTTCTGGTGAAGGGAGAAAA	60	256	--	--	--	--	--	--
TR69660|c1_g2_i1	No	(A)10ttgttattaaaaagttatatacttcaagatttttctataat gtttacaaatattgatttttgttcttt(A)15	94	--	--	--	--	--	--	TCCGAAAAGGTTAAGATGTCG	59	TGTTCTGGTGAAGGGAGAAAA	60	256	--	--	--	--	--	--
TR69661|c1_g2_i1	XLOC_029324	(AT)7	14	--	--	--	--	--	--	TGCAACAAAAACCGAAACTG	60	GCGTTGGAGGATTGCATAGT	60	261	--	--	--	--	--	--
TR69678|c0_g1_i1	No	(TTG)5	15	--	--	--	--	--	--	GGTGTCGAATGTTCCTAGCC	60	GAACAACAAGCGAAACGACA	60	158	--	--	--	--	--	--
TR69692|c0_g1_i1	No	(TG)8	16	--	--	--	--	--	--	GTGATTGAATGACGGTGTGC	60	ACAGCACCCTCTTCTGTCGT	60	202	--	--	--	--	--	--
TR69758|c0_g1_i1	No	(CAT)5caaaagaaaagatcaaagaga(AAG)6	54	--	--	--	--	--	--	AGCCTTGAAGGAAGTTCCAA	59	TGGTCCAAAGATGTTGGAGT	58	170	--	--	--	--	--	--
TR69779|c0_g1_i1	No	(GTG)7	21	--	--	--	--	--	--	TGTCGTCTGCTTTTACGTCG	60	AACCTCTCAGAAATTCCGCC	61	170	--	--	--	--	--	--
TR6981|c0_g1_i1	No	(CA)7	14	--	--	--	--	--	--	TTTCCCACCAAACTTCTCCA	60	GGCCAAAGTTTTCATCTTTGTT	60	229	--	--	--	--	--	--
TR6981|c0_g2_i1	No	(CA)7	14	--	--	--	--	--	--	TTTCCCACCAAACTTCTCCA	60	GGCCAAAGTTTTCATCTTTGTT	60	228	--	--	--	--	--	--
TR6981|c0_g3_i1	No	(CA)7	14	--	--	--	--	--	--	TTTCCCACCAAACTTCTCCA	60	GGCCAAAGTTTTCATCTTTGTT	60	227	--	--	--	--	--	--
TR6981|c0_g4_i1	No	(CA)7	14	--	--	--	--	--	--	TTTCCCACCAAACTTCTCCA	60	GGCCAAAGTTTTCATCTTTGTT	60	228	--	--	--	--	--	--
TR69833|c0_g1_i1	No	(ATA)5	15	--	--	--	--	--	--	TTCACTTTCACCACCACATGA	60	CCAAAACTTCTTGTGCTTTCC	59	212	--	--	--	--	--	--
TR69886|c0_g1_i1	XLOC_029380	(TGG)5	15	--	--	--	--	--	--	TCAATTAAAATTCCTCTTTTGAGGT	59	GGGATGGGATCTTCTCCATT	60	133	--	--	--	--	--	--
TR69922|c0_g1_i1	XLOC_029390	(TAA)5	15	--	--	--	--	--	--	ACTACATTCCCATTTCCCGC	61	TATGTTGGTGCTGCTTCTGC	60	212	--	--	XM_004511274.1	TALE	[T]	--

Transcript IDs	Differentially expressed gene (DEG) IDs	SSR repeat-motifs	SSR repeat-length (bp)	*In silico* polymorphic SSRs						Forward primers (5'-3')	Annealing tempearture(0C)	Reverse primers (5'-3')	Annealing tempearture(0C)	Amplified product size (bp)	Structural annotation		Functional Annotation			
				C. microphyllum		ICC4958														
				Transcript	SSR repeat-motifs	Chromosomes	SSR repeat-motifs	Start physical positions (bp)	End physical positions (bp)						Gene IDs	Sequence component of genes	NCBI nr IDs	TFs	KOG	KEGG
TR69970|c0_g1_i1	XLOC_029405	(CA)6	12	--	--	--	--	--	--	CCTTCCAAAAGAACACCCAA	60	GAAAGACGTTCGAGGATTGC	60	232	--	--	--	--	--	--
TR70027|c0_g1_i1	No	(TA)7	14	--	--	--	--	--	--	CCTTACTTCTCACACCTCCCA	59	TGGTGTGTAAACCAACCCCT	60	268	--	--	--	--	--	--
TR70027|c0_g2_i1	XLOC_029430	(TA)7	14	--	--	--	--	--	--	CCTTACTTCTCACACCTCCCA	59	CCAACACCTTTCTCCTTCCA	60	257	--	--	--	--	--	--
TR70039|c0_g1_i1	XLOC_029433	(TCA)5	15	--	--	--	--	--	--	CGTTTCTGTGACGGTGTTGT	60	CAAACCCTCCAACAACCATT	60	203	--	--	XP_001661570.1	--	[J]	--
TR70044|c0_g1_i1	No	(TG)6	12	TR70044|c0_g1_i1	(TG)6	Ca_LG_7	(TG)7	25367629	25367642	TCGGTAAAGCTTCTGGGTTG	60	CTTTTCCCCTCGTGCTACTG	60	134	Ca_16303	URR	--	--	--	--
TR70097|c0_g1_i1	XLOC_029456	(CT)6	12	--	--	--	--	--	--	TTCTGCATTTACAGCTTGTGC	59	CTTCTCGAGCTTGGAACCAG	60	239	--	--	--	--	--	--
TR70101|c0_g1_i1	XLOC_029461	(T)10catatcaaaaat(A)10	32	--	--	--	--	--	--	CTTCCATTCCCTTCCCTCTC	60	CTCTTCCCCCTCCTCTTGTT	60	194	--	--	--	--	--	--
TR70103|c0_g1_i4	No	(AT)9	18	--	--	--	--	--	--	TGAACCTGCCAAATGTCAAA	60	TTTCATCATCTTACAACACCATCA	59	209	--	--	--	--	--	--
TR70120|c0_g1_i1	XLOC_029477	(AG)6ggttttggtatgaatcccgaggagagagaaaggacaaatgaaagagtgccacgtaataaggagaggttgatatcttcaattatgcgct(GA)9	118	--	--	--	--	--	--	AGAAAATTGGGGGAATTTGG	60	AAAAACCTGCTTAGTGGGGC	60	266	--	--	--	--	--	--
TR70120|c0_g2_i1	No	(AG)6ggttttggtatgaatcccgaggagagagaaaggacaaatgaaagagtgccacgtaataaggagaggttgatatcttcaattatgcgct(GA)9	118	--	--	--	--	--	--	AGAAAATTGGGGGAATTTGG	60	AAAAACCTGCTTAGTGGGGC	60	266	--	--	--	--	--	--
TR70120|c1_g1_i1	XLOC_029478	(TC)10	20	--	--	--	--	--	--	ACCAAGGCGTAATCAAAACG	60	GGAGGGTGGCCTCTTTAATC	60	242	--	--	XP_004499127.1	--	[X]	--
TR70120|c1_g1_i3	No	(TC)10	20	--	--	--	--	--	--	ACCAAGGCGTAATCAAAACG	60	GGAGGGTGGCCTCTTTAATC	60	242	--	--	--	--	--	--
TR70120|c1_g2_i1	XLOC_029479	(TC)10	20	--	--	--	--	--	--	ACCAAGGCGTAATCAAAACG	60	GGAGGGTGGCCTCTTTAATC	60	242	--	--	--	--	--	--
TR70124|c0_g1_i2	No	(GC)6	12	--	--	--	--	--	--	CGAAGAGAAGGTTCCTGTGC	60	GCACTGCACTTTGAGCATGT	60	242	--	--	--	--	--	--

Transcript IDs	Differentially expressed gene (DEG) IDs	SSR repeat-motifs	SSR repeat-length (bp)	*In silico* polymorphic SSRs						Forward primers (5'-3')	Annealing tempearture(0C)	Reverse primers (5'-3')	Annealing tempearture(0C)	Amplified product size (bp)	Structural annotation		Functional Annotation			
				C. microphyllum		ICC4958									Gene IDs	Sequence component of genes	NCBI nr IDs	TFs	KOG	KEGG
				Transcript	SSR repeat-motifs	Chromosomes	SSR repeat-motifs	Start physical positions (bp)	End physical positions (bp)											
TR70124|c0_g1_i3	No	(GC)6	12	--	--	--	--	--	--	CGAAGAGAAGGTTCCTGTGC	60	GCACTGCACTTTGAGCATGT	60	262	--	--	--	--	--	--
TR70124|c0_g1_i4	No	(GC)6	12	--	--	--	--	--	--	CGAAGAGAAGGTTCCTGTGC	60	GCACTGCACTTTGAGCATGT	60	242	--	--	--	--	--	--
TR70133|c0_g2_i1	XLOC_029491	(TAA)6	18	--	--	--	--	--	--	TATGTCGGGTGTCATGTTGG	60	CCATTGGCTCACGTCTTTTT	60	194	--	--	--	--	--	--
TR70133|c0_g4_i1	No	(AT)8	16	--	--	--	--	--	--	CCAGAACAATGAAGCCAAAAA	60	TCACATGATTTGCTTCTGCC	60	177	--	--	--	--	--	--
TR70137|c0_g2_i2	No	(TTG)5	15	--	--	--	--	--	--	TGGAGTTGAACACTTGAACCA	59	CACACAGTTCACCCCTCAGA	60	185	--	--	--	--	--	--
TR70137|c0_g2_i5	No	(TTG)5	15	--	--	--	--	--	--	TGGAGTTGAACACTTGAACCA	59	CACACAGTTCACCCCTCAGA	60	185	--	--	--	--	--	--
TR70144|c1_g1_i1	XLOC_029506	(AGC)6	18	--	--	--	--	--	--	CAATCCAATCCAATCCAAGG	60	ATGATTTGAATTTGCGAGGC	60	160	--	--	XM_004499310.1	ARF	--	--
TR70144|c1_g1_i1	XLOC_029506	(TCT)6	18	--	--	--	--	--	--	GCCTCGCAAATTCAAATCAT	60	AACTTGTCGTAGCCGGTAGC	59	258	--	--	XM_004499310.1	ARF	--	--
TR70146|c0_g1_i1	No	(ATC)5	15	--	--	--	--	--	--	AATCAAACCGCCAAAAACAG	60	CAGTCCGATGGAGGGATAAA	60	135	--	--	--	--	--	--
TR70146|c0_g1_i2	No	(ATC)5	15	--	--	--	--	--	--	AATCAAACCGCCAAAAACAG	60	CAGTCCGATGGAGGGATAAA	60	135	--	--	--	--	--	--
TR70147|c1_g1_i1	XLOC_029509	(T)11atcgtactacttgttttttg(T)13	45	--	--	--	--	--	--	TCAAAACCAACCATGTCCAA	60	CCCAAAACCAAAACAGTAATCA	59	239	--	--	--	--	--	--
TR70172|c0_g1_i1	No	(TCC)6	18	--	--	--	--	--	--	CCTGAAGATGGGTCAGGAAA	60	GGTCAAGTTGTGGGAGGAAA	60	215	--	--	--	--	--	--
TR70172|c0_g1_i2	No	(TCC)6	18	--	--	--	--	--	--	CCTGAAGATGGGTCAGGAAA	60	GGTCAAGTTGTGGGAGGAAA	60	215	--	--	--	--	--	--
TR70172|c0_g1_i3	No	(TCC)6	18	--	--	--	--	--	--	CCTGAAGATGGGTCAGGAAA	60	GGTCAAGTTGTGGGAGGAAA	60	215	--	--	--	--	--	--

Transcript IDs	Differentially expressed gene (DEG) IDs	SSR repeat-motifs	SSR repeat-length (bp)	*In silico* polymorphic SSRs						Forward primers (5'-3')	Annealing tempearture(0C)	Reverse primers (5'-3')	Annealing tempearture(0C)	Amplified product size (bp)	Structural annotation		Functional Annotation			
				C. microphyllum		ICC4958									Gene IDs	Sequence component of genes	NCBI nr IDs	TFs	KOG	KEGG
				Transcript	SSR repeat-motifs	Chromosomes	SSR repeat-motifs	Start physical positions (bp)	End physical positions (bp)											
TR70172|c1_g1_i1	No	(TA)7	14	--	--	--	--	--	--	CCACCATAAACAAGTGGGGA	61	CAAGAGATCAAGTTAGATGCTTCA	58	211	--	--	--	--	--	--
TR70187|c0_g1_i1	No	(AAC)5	15	TR70187|c0_g1_i1	(AAC)6	Ca_LG_7	(AAC)5	4478168	4478182	TGCAAAATGATCAACAAAAATG	59	GGCCTTAATCCTAATGGGTTTT	60	197	Ca_16798	CDS	--	--	--	--
TR70187|c0_g1_i1	No	(AAC)6	18	TR70187|c0_g1_i1	(AAC)6	Ca_LG_7	(AAC)5	4478168	4478182	TGCAAGTCAAGAAAAAGAAGGA	59	GATTCGGCAAAAGAATCGAA	60	258	Ca_16798	CDS	--	--	--	--
TR70187|c0_g2_i1	No	(AAC)5	15	--	--	--	--	--	--	TGCAAAATGATCAACAAAAATG	59	GGCCTTAATCCTAATGGGTTTT	60	197	--	--	--	--	--	--
TR70187|c0_g2_i1	No	(AAC)6	18	--	--	--	--	--	--	TGCAAGTCAAGAAAAAGAAGGA	59	GATTCGGCAAAAGAATCGAA	60	258	--	--	--	--	--	--
TR70327|c0_g1_i1	XLOC_029581	(AT)6	12	--	--	--	--	--	--	CGGACAATAAATTAAGCAAGCC	60	GGGAGCAATATCCTCACTGC	60	280	--	--	--	--	--	--
TR70335|c0_g2_i1	XLOC_029586	(CAC)5	15	--	--	--	--	--	--	CTCCTCCACCACCACCAG	60	GAATGGAGGCGGATATGGTA	60	107	--	--	--	--	--	--
TR70344|c0_g1_i1	No	(TCA)7	21	--	--	--	--	--	--	CCAGGCTGGATCATTTTGTT	60	CTGAAAATGCTGATGAGGACAG	60	188	--	--	--	--	--	--
TR70427|c4_g1_i1	No	(GAG)6	18	--	--	--	--	--	--	CGCCGAAGATTACGAAGAAG	60	CCACCACCATCATGTAGCAG	60	141	--	--	--	--	--	--
TR70427|c4_g1_i2	No	(GAG)6	18	--	--	--	--	--	--	CGCCGAAGATTACGAAGAAG	60	CCACCACCATCATGTAGCAG	60	141	--	--	--	--	--	--
TR70436|c0_g1_i1	No	(AT)7	14	--	--	--	--	--	--	TTACGGTGAAAGTGGCAAAA	59	TCTTGGACATTCGATTTCTCCT	60	239	--	--	--	--	--	--
TR70436|c0_g1_i3	No	(AT)7	14	--	--	--	--	--	--	TTACGGTGAAAGTGGCAAAA	59	TCTTGGACATTCGATTTCTCCT	60	239	--	--	--	--	--	--
TR70436|c0_g1_i4	No	(AT)7	14	--	--	--	--	--	--	TTACGGTGAAAGTGGCAAAA	59	TCTTGGACATTCGATTTCTCCT	60	239	--	--	--	--	--	--
TR70436|c0_g2_i1	No	(AT)7	14	--	--	--	--	--	--	TTACGGTGAAAGTGGCAAAA	59	TCTTGGACATTCGATTTCTCCT	60	239	--	--	--	--	--	--

Transcript IDs	Differentially expressed gene (DEG) IDs	SSR repeat-motifs	SSR repeat-length (bp)	*In silico* polymorphic SSRs						Forward primers (5'-3')	Annealing tempearture(0C)	Reverse primers (5'-3')	Annealing tempearture(0C)	Amplified product size (bp)	Structural annotation		Functional Annotation			
				C. microphyllum		ICC4958														
				Transcript	SSR repeat-motifs	Chromosomes	SSR repeat-motifs	Start physical positions (bp)	End physical positions (bp)						Gene IDs	Sequence component of genes	NCBI nr IDs	TFs	KOG	KEGG
TR70440|c0_g1_i1	XLOC_029608	(TCA)7	21	--	--	--	--	--	--	CCATTAAAAGCAAAGGCTTCA	59	CTATCTTTTCCCCCAAAGGC	60	116	--	--	--	--	--	--
TR70440|c0_g2_i1	No	(TCA)7	21	--	--	--	--	--	--	CCATTAAAAGCAAAGGCTTCA	59	CTATCTTTTCCCCCAAAGGC	60	116	--	--	--	--	--	--
TR70440|c0_g2_i3	No	(TCA)7	21	--	--	--	--	--	--	CCATTAAAAGCAAAGGCTTCA	59	CTATCTTTTCCCCCAAAGGC	60	116	--	--	--	--	--	--
TR70445|c0_g1_i1	XLOC_029614	(TGC)5	15	--	--	--	--	--	--	TTGAAAGCCCCAAGAAAATG	60	TCTCATACCGAATCCTTGGC	60	100	--	--	--	--	--	--
TR70448|c3_g1_i1	XLOC_029619	(TG)9	18	--	--	--	--	--	--	TGACTCAAAACCCAGGAGGA	61	CCAGACCATTCTAATCGAGAGTC	59	237	--	--	XM_004509644.1	--	[R]	--
TR70453|c2_g1_i1	XLOC_029626	(AT)7	14	TR70453|c2_g1_i1	(AT)7	Ca_LG_8	(AT)6	11036123	11036134	CATTCGTTCATCGTTTTCCA	60	ACTTGAATTTTTCGCCGATG	60	205	Ca_20083	CDS	--	--	--	--
TR70456|c3_g1_i2	No	(TAA)8	24	--	--	--	--	--	--	CAAGTTCACAACTCCACCCA	60	AGATTCCAATTGCATCAGGG	60	245	--	--	--	--	--	--
TR70456|c3_g1_i3	No	(TAA)8	24	--	--	--	--	--	--	CAAGTTCACAACTCCACCCA	60	AGATTCCAATTGCATCAGGG	60	245	--	--	--	--	--	--
TR70456|c3_g1_i6	No	(TAA)8	24	--	--	--	--	--	--	CAAGTTCACAACTCCACCCA	60	AGATTCCAATTGCATCAGGG	60	245	--	--	--	--	--	--
TR70466|c1_g1_i1	XLOC_029646	(CTA)6	18	--	--	--	--	--	--	TGCTCAAGGTTCATGGAAAG	59	GCATTTGACCTGTGACCAGA	60	194	--	--	--	--	--	--
TR70467|c0_g1_i1	XLOC_029647	(ATA)7	21	--	--	--	--	--	--	GAGCCATGCATCAAGGAGAT	60	AATTAGGGTTGGATGGAGGG	60	255	--	--	--	--	--	--
TR70482|c1_g1_i1	XLOC_029664	(TGT)5	15	--	--	--	--	--	--	GACGGTGTTCTTTCCCTTGA	60	GGCAACCTCTTCCCTACAAA	59	157	--	--	XP_004490156.1	--	--	--
TR70497|c0_g1_i1	No	(GTT)5	15	--	--	--	--	--	--	CGATCGGCTAAAGAGAATGG	60	CCTGCATTTGGAGCTTCTTC	60	233	--	--	--	--	--	--
TR70503|c0_g1_i1	XLOC_029683	(GCG)5	15	--	--	--	--	--	--	CCCCTTTCCTTCATCAACAA	60	TTCGTTTCAACAAACCACCA	60	192	--	--	--	--	--	--

Transcript IDs	Differentially expressed gene (DEG) IDs	SSR repeat-motifs	SSR repeat-length (bp)	*In silico* polymorphic SSRs						Forward primers (5'-3')	Annealing tempearture(0C)	Reverse primers (5'-3')	Annealing tempearture(0C)	Amplified product size (bp)	Structural annotation		Functional Annotation			
				C. microphyllum		ICC4958									Gene IDs	Sequence component of genes	NCBI nr IDs	TFs	KOG	KEGG
				Transcript	SSR repeat-motifs	Chromosomes	SSR repeat-motifs	Start physical positions (bp)	End physical positions (bp)											
TR70503|c1_g1_i1	XLOC_029684	(GGC)5	15	--	--	--	--	--	--	TGGTAGTAAACTGCCGGAGG	60	TGAAGAATCGGAAACGTGTG	60	228	--	--	XP_004496690.1	S1Fa-like	[M]	--
TR70505|c0_g1_i2	No	(GA)7	14	--	--	--	--	--	--	TGCTTTCATTTGGCATGAGA	60	CATGTTTTGACCTGTGTGCC	60	154	--	--	--	--	--	--
TR70505|c0_g1_i3	No	(GA)7	14	--	--	--	--	--	--	TGCTTTCATTTGGCATGAGA	60	CATGTTTTGACCTGTGTGCC	60	154	--	--	--	--	--	--
TR70511|c0_g1_i1	XLOC_029694	(TC)11	22	--	--	--	--	--	--	ACCAGGCACCAATCAAAGTC	60	AGATGAGGAATAGCATGCGG	60	170	--	--	--	--	--	--
TR70514|c2_g1_i2	XLOC_029697	(AAATT)5	25	--	--	--	--	--	--	CGCAGATTCCGATACTGCTT	60	AAGCTGCTTTCATCGTCGTT	60	278	--	--	--	--	--	--
TR70516|c0_g1_i1	XLOC_029698	(CAT)5	15	--	--	--	--	--	--	ATCAGGTTGAGCTTGCCAGT	60	TGGGAGGAAACAATCAAAGG	60	263	--	--	--	--	--	--
TR70516|c0_g1_i2	XLOC_029699	(CAT)5	15	--	--	--	--	--	--	ATCAGGTTGAGCTTGCCAGT	60	TGGGAGGAAACAATCAAAGG	60	260	--	--	--	--	--	--
TR70517|c0_g2_i1	XLOC_029701	(T)10cttgtctaggataccctattagtttaatttaatgagt(A)11	58	--	--	--	--	--	--	GGGCAAAATTTTTCATGGTC	59	GCAGAAAGTGATGTATGGTGACA	60	236	--	--	--	--	--	--
TR70525|c0_g1_i1	No	(GCG)7	21	--	--	--	--	--	--	GAGTTGCTTCGTTGGGAAGA	60	TAGGACCTTGACCATCAGGC	60	232	--	--	--	--	--	--
TR70525|c0_g1_i3	No	(GCG)7	21	--	--	--	--	--	--	GAGTTGCTTCGTTGGGAAGA	60	TAGGACCTTGACCATCAGGC	60	232	--	--	--	--	--	--
TR70706|c0_g1_i1	No	(TAT)5	15	--	--	--	--	--	--	GCAGAAAGAAACAGAAGTGTGG	59	GTTCCTCCCTCATCTCCTCC	60	133	--	--	--	--	--	--
TR70706|c0_g1_i2	No	(TAT)5	15	--	--	--	--	--	--	GCAGAAAGAAACAGAAGTGTGG	59	GTTCCTCCCTCATCTCCTCC	60	133	--	--	--	--	--	--
TR70706|c0_g1_i3	No	(TAT)5	15	--	--	--	--	--	--	GCAGAAAGAAACAGAAGTGTGG	59	GTTCCTCCCTCATCTCCTCC	60	133	--	--	--	--	--	--
TR70706|c0_g1_i4	No	(TAT)5	15	--	--	--	--	--	--	GCAGAAAGAAACAGAAGTGTGG	59	GTTCCTCCCTCATCTCCTCC	60	133	--	--	--	--	--	--

Transcript IDs	Differentially expressed gene (DEG) IDs	SSR repeat-motifs	SSR repeat-length (bp)	In silico polymorphic SSRs						Forward primers (5'-3')	Annealing tempearture(0C)	Reverse primers (5'-3')	Annealing tempearture(0C)	Amplified product size (bp)	Structural annotation		Functional Annotation			
				C. microphyllum		ICC4958									Gene IDs	Sequence component of genes	NCBI nr IDs	TFs	KOG	KEGG
				Transcript	SSR repeat-motifs	Chromosomes	SSR repeat-motifs	Start physical positions (bp)	End physical positions (bp)											
TR70706|c0_g1_i5	No	(TAT)5	15	--	--	--	--	--	--	GCAGAAAGAAACAGAAGTGTGG	59	GTTCCTCCCTCATCTCCTCC	60	133	--	--	--	--	--	--
TR70706|c0_g1_i6	No	(TAT)5	15	--	--	--	--	--	--	GCAGAAAGAAACAGAAGTGTGG	59	GTTCCTCCCTCATCTCCTCC	60	133	--	--	--	--	--	--
TR70706|c0_g1_i7	No	(TAT)5	15	--	--	--	--	--	--	GCAGAAAGAAACAGAAGTGTGG	59	GTTCCTCCCTCATCTCCTCC	60	133	--	--	--	--	--	--
TR70706|c1_g1_i1	XLOC_029732	(GA)6	12	--	--	--	--	--	--	TTCAAGACCGTCTCTAAAGCTG	59	GGACCATGTCCTTCCAAACT	59	193	--	--	--	--	--	--
TR70723|c0_g1_i1	No	(T)10agcagaaaacaatgcaaaaagggagtggag(T)11	51	--	--	--	--	--	--	GGAGGTGGGAATAGCAACAA	60	TCTTCTCACTCACACACTCAAACA	60	164	--	--	--	--	--	--
TR70723|c0_g2_i1	XLOC_029756	(T)10agcagaaaacaatgcaaaaagggagtggag(T)11	51	--	--	--	--	--	--	GGAGGTGGGAATAGCAACAA	60	TCTTCTCACTCACACACTCAAACA	60	164	--	--	--	--	--	--
TR70723|c1_g1_i1	No	(CAA)5	15	--	--	--	--	--	--	GAAGGAGATCTTCAGCAGCC	59	GATTCGCAACCGCATATTTT	60	279	--	--	--	--	--	--
TR70732|c0_g1_i1	XLOC_029767	(CT)7	14	--	--	--	--	--	--	CCATGCTCCACCTCTTTTGT	60	CCGGCATTTTAAAACGGATA	60	222	--	--	--	--	--	--
TR70739|c0_g1_i1	XLOC_029778	(GA)9	18	--	--	--	--	--	--	ATGGTGAGAGATGGAGTGCC	60	TCCTAAGCGGTTTGTCTGCT	60	207	--	--	--	--	--	--
TR70739|c0_g2_i1	No	(GA)9	18	--	--	--	--	--	--	ATGGTGAGAGATGGAGTGCC	60	TCCTAAGCGGTTTGTCTGCT	60	169	--	--	--	--	--	--
TR70745|c0_g1_i1	No	(GAA)5	15	--	--	--	--	--	--	CAATAATCGCAAGCGAACCT	60	TCGTCCTCTTTGCAACTTCA	60	134	--	--	--	--	--	--
TR70745|c0_g1_i2	No	(GAA)5	15	--	--	--	--	--	--	CAATAATCGCAAGCGAACCT	60	TCGTCCTCTTTGCAACTTCA	60	134	--	--	--	--	--	--
TR70782|c0_g1_i1	No	(CT)11	22	--	--	--	--	--	--	TCCAACACAGGACACACAAA	59	GCCATCAGAGCTTTCGTTTC	60	151	--	--	--	--	--	--
TR70782|c0_g3_i1	No	(CT)11	22	--	--	--	--	--	--	TCCAACACAGGACACACAAA	59	GCCATCAGAGCTTTCGTTTC	60	151	--	--	--	--	--	--

Transcript IDs	Differentially expressed gene (DEG) IDs	SSR repeat-motifs	SSR repeat-length (bp)	*In silico* polymorphic SSRs						Forward primers (5'-3')	Annealing tempearture(0C)	Reverse primers (5'-3')	Annealing tempearture(0C)	Amplified product size (bp)	Structural annotation		Functional Annotation			
				C. microphyllum		ICC4958									Gene IDs	Sequence component of genes	NCBI nr IDs	TFs	KOG	KEGG
				Transcript	SSR repeat-motifs	Chromosomes	SSR repeat-motifs	Start physical positions (bp)	End physical positions (bp)											
TR70782|c0_g3_i2	No	(CT)11	22	--	--	--	--	--	--	TCCAACACAGGACACACAAA	59	GCCATCAGAGCTTTCGTTTC	60	151	--	--	--	--	--	--
TR70782|c0_g3_i3	No	(CT)11	22	--	--	--	--	--	--	TCCAACACAGGACACACAAA	59	GCCATCAGAGCTTTCGTTTC	60	151	--	--	--	--	--	--
TR70791|c4_g1_i1	XLOC_029845	(AAT)6	18	TR70791|c4_g1_i1	(ACT)5	Ca_LG_6	(ACT)6	18582452	18582469	AAAGCACTGCTGGCAAAGAT	60	CTGCTCCACACCAATTCTCA	60	173	Ca_14190	DRR	--	--	--	--
TR70791|c4_g1_i1	XLOC_029845	(ACT)5	15	TR70791|c4_g1_i1	(ACT)5	Ca_LG_6	(ACT)6	18582452	18582469	TGAGAATTGGTGTGGAGCAG	60	TTTTGCCACTGGTTTTGACA	60	233	Ca_14190	DRR	--	--	--	--
TR70793|c0_g2_i2	No	(CT)12	24	--	--	--	--	--	--	TTTCAGTTGCACAAGCAAAGA	60	TTCGAGGAGTCTGAGGCACT	60	276	--	--	--	--	--	--
TR70793|c0_g2_i3	No	(CT)12	24	--	--	--	--	--	--	TTTCAGTTGCACAAGCAAAGA	60	TTCGAGGAGTCTGAGGCACT	60	276	--	--	--	--	--	--
TR70810|c1_g1_i1	No	(A)12tccattccataattcatcaacaaaatctaagaaaca aagaaaaaaaaaatacgttaccgaaattcaaatt(GA)9	99	--	--	--	--	--	--	TCAACACCCCTAACTCGGAC	60	GGGTATTTCAAATTTCCCCC	59	199	--	--	--	--	--	--
TR70813|c0_g1_i1	XLOC_029866	(TCA)8	24	TR70813|c0_g1_i1	(TCA)8	Ca_LG_8	(TCA)5	14298919	14298933	CCATCCAATCAAAGGCATCT	60	TTGCAAATGCAACCAATGTT	60	212	Ca_20821	URR	--	--	--	--
TR70910|c0_g1_i1	XLOC_029912	(TC)6	12	--	--	--	--	--	--	GGTGAAGGTGAATTGAAGGC	60	TGGAAAACCCTTCAAAGGAA	60	275	--	--	--	--	--	--
TR70912|c0_g1_i1	XLOC_029913	(TTG)5gtttt(CAC)5	35	--	--	--	--	--	--	AGATCCATGATCGCCTGAAG	60	CCATATCCAAGCCGTACGTT	60	274	--	--	XM_003609015.1	ZF-HD	[S]	--
TR70913|c1_g1_i1	XLOC_029915	(A)14gatttgtgaaattcgattaaaatgtaaaaaggtcaaa acatgagatagtcttgctgttcagtgtcgtaggtatggagata aaagaaagaaag(A)11	117	--	--	--	--	--	--	CCGAGAAAGAAATATGGGCA	60	TCAAACTCTTCATTCATCCCA	58	235	--	--	--	--	--	--
TR70925|c0_g1_i1	XLOC_029921	(CAA)7	21	TR70925|c0_g1_i1	(CAA)7	Ca_LG_6	(CAA)5	20770769	20770783	ACCATACCCACCACCTTCTG	60	ACAGTGATTGATGATGCCGA	60	207	Ca_14713	DRR	--	--	--	--
TR70925|c0_g1_i1	XLOC_029921	(CA)10	20	TR70925|c0_g1_i1	(CAA)7	Ca_LG_6	(CAA)5	20770769	20770783	TCACTCCTTGGCTTTCTCTCA	60	TTTGGATGGAACTTGGAAGG	60	236	Ca_14713	DRR	--	--	--	--
TR70925|c0_g2_i1	No	(CAA)7	21	--	--	--	--	--	--	ACCATACCCACCACCTTCTG	60	ACAGTGATTGATGATGCCGA	60	207	--	--	--	--	--	--

Transcript IDs	Differentially expressed gene (DEG) IDs	SSR repeat-motifs	SSR repeat-length (bp)	*In silico* polymorphic SSRs						Forward primers (5'-3')	Annealing tempearture(0C)	Reverse primers (5'-3')	Annealing tempearture(0C)	Amplified product size (bp)	Structural annotation		Functional Annotation			
				C. microphyllum		ICC4958									Gene IDs	Sequence component of genes	NCBI nr IDs	TFs	KOG	KEGG
				Transcript	SSR repeat-motifs	Chromosomes	SSR repeat-motifs	Start physical positions (bp)	End physical positions (bp)											
TR70925|c0_g2_i1	No	(CA)10	20	--	--	--	--	--	--	TCACTCCTTGGCTTTCTCTCA	60	TTTGGATGGAACTTGGAAGG	60	236	--	--	--	--	--	--
TR70955|c0_g1_i1	No	(TC)8	16	--	--	--	--	--	--	CGTTGGCAGTTCCGTTCTAT	60	CTCCGCCATCATCATTTCTT	60	255	--	--	--	--	--	--
TR70955|c0_g2_i1	No	(TC)8	16	--	--	--	--	--	--	CGTTGGCAGTTCCGTTCTAT	60	CTCCGCCATCATCATTTCTT	60	255	--	--	--	--	--	--
TR70979|c0_g1_i1	No	(AG)7	14	--	--	--	--	--	--	TAGGGTCGTGAGGATGGAAG	60	AAGACTTCCGACATATAGCTTTG G	60	106	--	--	--	--	--	--
TR70991|c0_g1_i1	No	(TG)6	12	--	--	--	--	--	--	GGGAAACCTGGTGCTACAAA	60	CCACAACTCATGCGTTACAAA	60	155	--	--	--	--	--	--
TR71027|c0_g1_i1	XLOC_029969	(GCC)5	15	--	--	--	--	--	--	CGTCAATTCCAGCTGTCGTA	60	CTCGATGGGGTATCCAAATG	60	267	--	--	--	--	--	--
TR71075|c0_g1_i1	No	(CCT)5	15	--	--	--	--	--	--	CAGAATAATGCGGTGTGTCG	60	ATGATGCGGCAGAAGATACC	60	246	--	--	--	--	--	--
TR71158|c0_g1_i1	No	(CAC)5	15	--	--	--	--	--	--	GAAGGAATTGGTGCTTTTGG	60	GCTTTTGCCTCCAAAACTTG	60	154	--	--	--	--	--	--
TR71158|c0_g1_i2	No	(CAC)5	15	--	--	--	--	--	--	GAAGGAATTGGTGCTTTTGG	60	GCTTTTGCCTCCAAAACTTG	60	154	--	--	--	--	--	--
TR71158|c0_g1_i3	No	(CAC)5	15	--	--	--	--	--	--	GAAGGAATTGGTGCTTTTGG	60	GCTTTTGCCTCCAAAACTTG	60	154	--	--	--	--	--	--
TR71158|c0_g1_i4	No	(CAC)5	15	--	--	--	--	--	--	GAAGGAATTGGTGCTTTTGG	60	GCTTTTGCCTCCAAAACTTG	60	154	--	--	--	--	--	--
TR71158|c0_g1_i5	No	(CAC)5	15	--	--	--	--	--	--	GAAGGAATTGGTGCTTTTGG	60	GCTTTTGCCTCCAAAACTTG	60	154	--	--	--	--	--	--
TR71158|c0_g1_i6	No	(CAC)5	15	--	--	--	--	--	--	GAAGGAATTGGTGCTTTTGG	60	GCTTTTGCCTCCAAAACTTG	60	154	--	--	--	--	--	--
TR71158|c0_g1_i7	No	(CAC)5	15	--	--	--	--	--	--	GAAGGAATTGGTGCTTTTGG	60	GCTTTTGCCTCCAAAACTTG	60	154	--	--	--	--	--	--

Transcript IDs	Differentially expressed gene (DEG) IDs	SSR repeat-motifs	SSR repeat-length (bp)	*In silico* polymorphic SSRs						Forward primers (5'-3')	Annealing tempearture(0C)	Reverse primers (5'-3')	Annealing tempearture(0C)	Amplified product size (bp)	Structural annotation		Functional Annotation			
				C. microphyllum		ICC4958									Gene IDs	Sequence component of genes	NCBI nr IDs	TFs	KOG	KEGG
				Transcript	SSR repeat-motifs	Chromosomes	SSR repeat-motifs	Start physical positions (bp)	End physical positions (bp)											
TR71225|c0_g1_i1	XLOC_030077	(CAT)5	15	--	--	--	--	--	--	GTTCGTTGATATGGGGATGG	60	AACGGAGAGAGCGTAGTGGA	60	208	--	--	--	--	--	--
TR71228|c1_g1_i1	XLOC_030082	(ATC)5	15	--	--	--	--	--	--	GACAAAAACACCTTTAGCCCA	59	TGTAAGTGAGCCTGACGACG	60	252	--	--	--	--	--	--
TR71253|c0_g1_i1	XLOC_030096	(CCA)5	15	--	--	--	--	--	--	GCCAGAAATTGGACACTGGT	60	CGATACTCTCCTGGCCACTG	61	114	--	--	ETK81808.1	--	--	--
TR713|c0_g1_i1	XLOC_030138	(AT)6	12	--	--	--	--	--	--	TGTGGTATCATGCAAAATCTCAA	60	TCATGAGAAGTCCCACATCG	60	222	--	--	XP_004486746.1	--	[S]	--
TR71473|c0_g1_i1	XLOC_030153	(TA)6	12	--	--	--	--	--	--	TGGACTTCCACTCGGATCTC	60	GATCACGGTTCCTAAGTGGC	60	219	--	--	--	--	--	--
TR71482|c0_g1_i1	No	(TC)6	12	--	--	--	--	--	--	CCCTTGGCAGAGAATAACCA	60	TCAGGTGCCAATGTTTTCAA	60	192	--	--	--	--	--	--
TR71533|c0_g1_i1	No	(TGA)5	15	--	--	--	--	--	--	ATTAACTGCGGGTCATGCTC	60	GTCCCAAGTCCCTGCAAATA	60	252	--	--	--	--	--	--
TR71545|c0_g1_i1	No	(GGA)7	21	--	--	--	--	--	--	GAAATGGGTGAGCATGAGGT	60	CCTTCCACCACCTCCGAC	62	206	--	--	--	--	--	--
TR71581|c0_g1_i1	No	(CA)6	12	--	--	--	--	--	--	CAAACAAACAAAGAATGACACACA	60	TGGATCTTTTGAGCCTGACA	59	190	--	--	--	--	--	--
TR71666|c0_g1_i1	XLOC_030194	(TA)7	14	--	--	--	--	--	--	CTTGTAGTACTCCGCCCCCT	61	TGCGTCCAGAGAATACATGG	60	280	--	--	--	--	--	--
TR71778|c0_g1_i2	XLOC_030230	(TTC)8	24	--	--	--	--	--	--	GCATCTGGTGGTAGAATGGG	60	TCATTGTCCTTGTTGCCTTTT	60	149	--	--	--	--	--	--
TR71778|c1_g1_i1	XLOC_030231	(ATC)8	24	--	--	--	--	--	--	GTTGGATGGAGAAGCAGAGG	60	AAGATGAATCGATGAACGGC	60	263	--	--	XM_004494630.1	Trihelix	[K]	--
TR7178|c0_g1_i1	XLOC_030246	(CTT)5	15	--	--	--	--	--	--	CAACCTCATCGGGGATTCTA	60	TCACCACACCATGACAATGA	59	124	--	--	--	--	--	--
TR7178|c0_g2_i1	XLOC_030247	(CTT)5	15	--	--	--	--	--	--	CAACCTCATCGGGGATTCTA	60	TCACCACACCATGACAATGA	59	124	--	--	--	--	--	--

Transcript IDs	Differentially expressed gene (DEG) IDs	SSR repeat-motifs	SSR repeat-length (bp)	*In silico* polymorphic SSRs						Forward primers (5'-3')	Annealing tempearture(0C)	Reverse primers (5'-3')	Annealing tempearture(0C)	Amplified product size (bp)	Structural annotation		Functional Annotation			
				C. microphyllum		ICC4958									Gene IDs	Sequence component of genes	NCBI nr IDs	TFs	KOG	KEGG
				Transcript	SSR repeat-motifs	Chromosomes	SSR repeat-motifs	Start physical positions (bp)	End physical positions (bp)											
TR71822|c0_g2_i1	No	(TA)7	14	--	--	--	--	--	--	TCAGTATGGCAACGGCACTA	60	GATTGCGTAAACACGAGCAA	60	232	--	--	--	--	--	--
TR71822|c0_g3_i2	XLOC_030283	(TA)7	14	--	--	--	--	--	--	TCAGTATGGCAACGGCACTA	60	GATTGCGTAAACACGAGCAA	60	232	--	--	XP_004509083.1	--	--	--
TR71829|c1_g1_i1	XLOC_030290	(GA)6	12	--	--	--	--	--	--	GATCCACAAATCGAGGCAAC	60	GAAATGCAAACCGTGGAAAT	60	235	--	--	--	--	--	--
TR71835|c0_g1_i1	No	(AGC)5	15	--	--	--	--	--	--	GTGGAGAGATGGAAAGCTGC	60	GGCCACTGAAAGAAAAACCA	60	261	--	--	--	--	--	--
TR71835|c0_g1_i2	No	(AGC)5	15	--	--	--	--	--	--	GTGGAGAGATGGAAAGCTGC	60	GGCCACTGAAAGAAAAACCA	60	261	--	--	--	--	--	--
TR71835|c0_g1_i3	No	(AGC)5	15	--	--	--	--	--	--	ATCCACTTTTCCCCTCAACC	60	GCTTTGACGTTTCCACCAAT	60	169	--	--	--	--	--	--
TR71835|c0_g1_i4	No	(AGC)5	15	--	--	--	--	--	--	GTGGAGAGATGGAAAGCTGC	60	GGCCACTGAAAGAAAAACCA	60	261	--	--	--	--	--	--
TR71835|c0_g1_i5	No	(AGC)5	15	--	--	--	--	--	--	ATCCACTTTTCCCCTCAACC	60	GCTTTGACGTTTCCACCAAT	60	169	--	--	--	--	--	--
TR71835|c0_g1_i6	No	(AGC)5	15	--	--	--	--	--	--	ATCCACTTTTCCCCTCAACC	60	GCTTTGACGTTTCCACCAAT	60	169	--	--	--	--	--	--
TR71835|c0_g1_i7	No	(AGC)5	15	--	--	--	--	--	--	GTGGAGAGATGGAAAGCTGC	60	GGCCACTGAAAGAAAAACCA	60	261	--	--	--	--	--	--
TR71835|c1_g1_i1	No	(TG)8	16	--	--	--	--	--	--	TTGGGCCTTCACCTAATGTC	60	TTGCATAAGCAAATCTTGAAAA	58	225	--	--	--	--	--	--
TR71842|c1_g1_i2	No	(TG)7	14	--	--	--	--	--	--	CATTTCTGTGTTTGTCTTTGCAT	59	TGCCGACATAGCTTCCTCTT	60	270	--	--	--	--	--	--
TR71842|c1_g1_i3	XLOC_030305	(TG)7	14	--	--	--	--	--	--	CATTTCTGTGTTTGTCTTTGCAT	59	TGCCGACATAGCTTCCTCTT	60	270	--	--	--	--	--	--
TR71842|c1_g1_i4	No	(TGA)7	21	--	--	--	--	--	--	TTTAATTTGAAGTTGGGGCCT	60	AGACGAGTACCAGCACCACC	60	220	--	--	--	--	--	--

Transcript IDs	Differentially expressed gene (DEG) IDs	SSR repeat-motifs	SSR repeat-length (bp)	*In silico* polymorphic SSRs						Forward primers (5'-3')	Annealing tempearture(0C)	Reverse primers (5'-3')	Annealing tempearture(0C)	Amplified product size (bp)	Structural annotation		Functional Annotation			
				C. microphyllum		ICC4958									Gene IDs	Sequence component of genes	NCBI nr IDs	TFs	KOG	KEGG
				Transcript	SSR repeat-motifs	Chromosomes	SSR repeat-motifs	Start physical positions (bp)	End physical positions (bp)											
TR71843|c0_g1_i1	XLOC_030306	(TC)9	18	--	--	--	--	--	--	AATGACGCGTGTCTTCTCGT	61	GGGGAAGAAGAAGAATGGGA	60	116	--	--	--	--	--	--
TR71843|c0_g1_i1	XLOC_030306	(CT)6	12	--	--	--	--	--	--	CCCCCATTTCACTCCTCTCT	60	TATGTAGTCAGCGAGCCACG	60	267	--	--	--	--	--	--
TR71843|c0_g1_i1	XLOC_030306	(CCA)5	15	--	--	--	--	--	--	CTTCAGAAATCGTTCTCCGC	60	TAACTTGCTGCACCATCTGC	60	274	--	--	--	--	--	--
TR71846|c0_g1_i1	No	(TATT)6tgagagtttt(A)13	47	--	--	--	--	--	--	GCTCTTGTGTTTGGGGTCAT	60	CACTCCAACCACCACTTCCT	60	147	--	--	--	--	--	--
TR71846|c0_g1_i2	No	(TATT)6tgagagtttt(A)13	47	--	--	--	--	--	--	GCTCTTGTGTTTGGGGTCAT	60	CACTCCAACCACCACTTCCT	60	147	--	--	--	--	--	--
TR71846|c0_g1_i3	No	(TATT)6tgagagtttt(A)13	47	--	--	--	--	--	--	GCTCTTGTGTTTGGGGTCAT	60	CACTCCAACCACCACTTCCT	60	147	--	--	--	--	--	--
TR71846|c0_g1_i4	No	(TATT)6tgagagtttt(A)13	47	--	--	--	--	--	--	GCTCTTGTGTTTGGGGTCAT	60	CACTCCAACCACCACTTCCT	60	147	--	--	--	--	--	--
[truncated: 456,113 more chars]
